# p38α in macrophages aggravates arterial endothelium injury by releasing IL-6 through phosphorylating megakaryocytic leukemia 1

**DOI:** 10.1016/j.redox.2020.101775

**Published:** 2020-11-01

**Authors:** Meng Zhang, Jianing Gao, Xuyang Zhao, Mingming Zhao, Dong Ma, Xinhua Zhang, Dongping Tian, Bing Pan, Xiaoxiang Yan, Jianwei Wu, Xia Meng, Huiyong Yin, Lemin Zheng

**Affiliations:** a The Institute of Cardiovascular Sciences and Institute of Systems Biomedicine, School of Basic Medical Sciences, Key Laboratory of Molecular Cardiovascular Sciences of Ministry of Education, NHC Key Laboratory of Cardiovascular Molecular Biology and Regulatory Peptides, Health Science Center, Peking University, Beijing, 100191, China; b Beijing Tiantan Hospital, China National Clinical Research Center for Neurological Diseases, Advanced Innovation Center for Human Brain Protection, The Capital Medical University, Beijing, 100050, China; c School of Public Health, North China University of Science and Technology, 21 Bohai Avenue, Caofeidian New City, Tangshan, 063210, Hebei, China; d Department of Biochemistry and Molecular Biology, The Key Laboratory of Neural and Vascular Biology, Ministry of Education. Hebei Medical University, No. 361 Zhongshan E Rd, Shijiazhuang, 050017, Hebei, China; e Dept. of Pathology, Shantou University Medical College, No.22 Xinling Road, Shantou, 515041, Guangdong, China; f Department of Cardiology, Rui Jin Hospital, Shanghai Jiaotong University School of Medicine, China; g Institute of Cardiovascular Diseases, Shanghai Jiaotong University School of Medicine, China; h CAS Key Laboratory of Nutrition, Metabolism and Food Safety, Shanghai Institute of Nutrition and Health (SINH), Chinese Academy of Sciences (CAS), Shanghai, 200031, China, University of the Chinese Academy of Sciences, CAS, Beijing, China, Key Laboratory of Food Safety Risk Assessment, Ministry of Health, Beijing, China, School of Life Science and Technology, ShanghaiTech University, Shanghai, 201210, China

**Keywords:** p38, Endothelial cell, Re-endothelialization, Megakaryocytic leukemia 1, Interleukin-6, ApoE, Apolipoprotein E, BrdU, 5-bromo-2-deoxyuridine, CAD, Coronary heart disease, EC, Endothelial cell, ERK, Extracellular signal-regulated kinase, HUVEC, Human umbilical vein endothelial cell, IL-6, Interleukin 6, LDL, Low density lipoprotein, LPS, Lipopolysaccharide, MAPK, Mitogen-activated protein kinase, MKL1, megakaryocytic leukemia 1, NF-κB, Nuclear factor-κB, SRF, Serum response factor, TNF-α, Tumor Necrosis Factor-α

## Abstract

**Background:**

Macrophages regulate the inflammatory response and affect re-endothelialization. Inflammation and macrophages play important roles in promoting tissue repair, but p38α mitogen-activated protein kinase's role in re-endothelialization is unknown.

**Methods and results:**

Wire injuries of carotid arteries and Evans blue staining were performed in macrophage-specific p38α-knockout (p38α^fl/fl^LysMCre^+/-^) mice and control mice (p38α^fl/fl^). *Re*-endothelialization of the carotid arteries at 3, 5 and 7 days was significantly promoted in p38α^fl/fl^LysMCre^+/-^ mice. *In vitro* experiments indicated that both the proliferation and migration of endothelial cells were enhanced in conditioned medium from peritoneal macrophages of p38α^fl/fl^LysMCre^+/-^ mice. Interleukin-6 (IL-6) level was decreased significantly in macrophages of p38α^fl/fl^LysMCre^+/-^ mice and an IL-6-neutralizing antibody promoted endothelial cell migration *in vitro* and re-endothelialization in p38α^fl/fl^ mice *in vivo*. Phosphoproteomics revealed that the phosphorylation level of S544/T545/S549 sites in megakaryocytic leukemia 1 (MKL1) was decreased in p38α^fl/fl^LysMCre^+/-^ mice. The mutation of either S544/S549 or T545/S549 sites could reduce the expression of IL-6 and the inhibition of MKL1 reduced the expression of IL-6 *in vitro* and promoted re-endothelialization *in vivo*.

**Conclusion:**

p38α in macrophages aggravates injury of arteries by phosphorylating MKL1, and increasing IL-6 expression after vascular injury.

## Highlights

• Re-endothelialization of the carotid arteries is significantly promoted when p38α is knocked out in macrophages.
• Inhibiting IL-6 activity by a neutralizing antibody promotes re-endothelialization.
• p38α mediates S544/S549 and T545/S549 phosphorylation motif in MKL1 which regulates the expression of IL-6 in mouse.

## Graphical abstract

**Figure:**
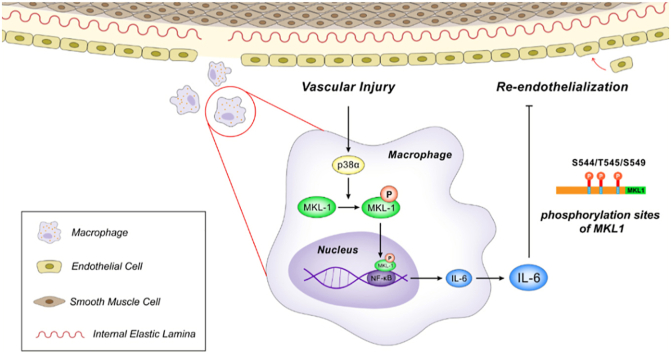

## 1 Introduction

In recent years, the incidence and mortality of cardiovascular disease have increased significantly [1,2]. Percutaneous transluminal coronary angioplasty is the main method for the treatment of coronary heart disease (CAD) and has achieved remarkable curative effects [3]. Several studies have shown that implantation of a stent can cause vascular injury and restenosis [4]. Blood vessels not only act as a transport conduit system, but also participate in organ development, tissue morphogenesis, inflammation, barrier formation, and wound healing [5]. Arteries are usually divided into the intima, media and adventitia [6]. The intima is a mechanical barrier between circulating blood and vascular smooth muscle cells. It is also the largest and most important endocrine organ in the human body [7]. After endothelial cell damage, the endocrine function is broken, leading to vascular endothelial dysfunction [8]. However, re-endothelialization effectively reduces the incidence of restenosis, and the incidence and mortality of CAD [9,10]. Our previous study shows that re-endothelialization plays an important role in atherosclerosis [11].

Macrophages are highly plastic cells and prominent inflammatory cells in wounds [12,13]. They participate not only in pro-inflammatory responses, but also in the resolution of inflammation and tissue repair by secreting anti-inflammatory cytokines to drive angiogenesis [14]. p38 mitogen-activated protein kinase (MAPK) is activated by a variety of extracellular stresses and cytokines [15,16]. p38 MAPK is reported to regulate intracellular signal transduction. There is considerable evidence implicating p38 MAPK as a potentially mediator in the inflammatory response and atherosclerosis [[17], [18], [19], [20]]. Although p38α has been extensively studied in macrophages, its role in macrophages regulating the repair of injured vessel is still unclear. Here, we determined whether deficiency of p38α in macrophages regulates the inflammatory response and re-endothelialization after vascular injury, and then revealed the mechanism of endothelial repair. In this process, p38α mediates the phosphorylation of megakaryocytic leukemia 1 (MKL1), a transcriptional regulator that regulates transcription by interacting with sequence-specific transcription factors [21], and then promotes the expression of interleukin 6 (IL-6). In the current study, we demonstrated that deficiency of p38α in macrophages reduces inflammation, promotes the proliferation and migration of endothelial cells, and promotes re-endothelialization of injured arteries.

## 2 Results

### 2.1 p38α MAPK Expression is Markedly Increased in atherosclerotic plaques of patients and ApoE-/- mice

**Fig. 1:**
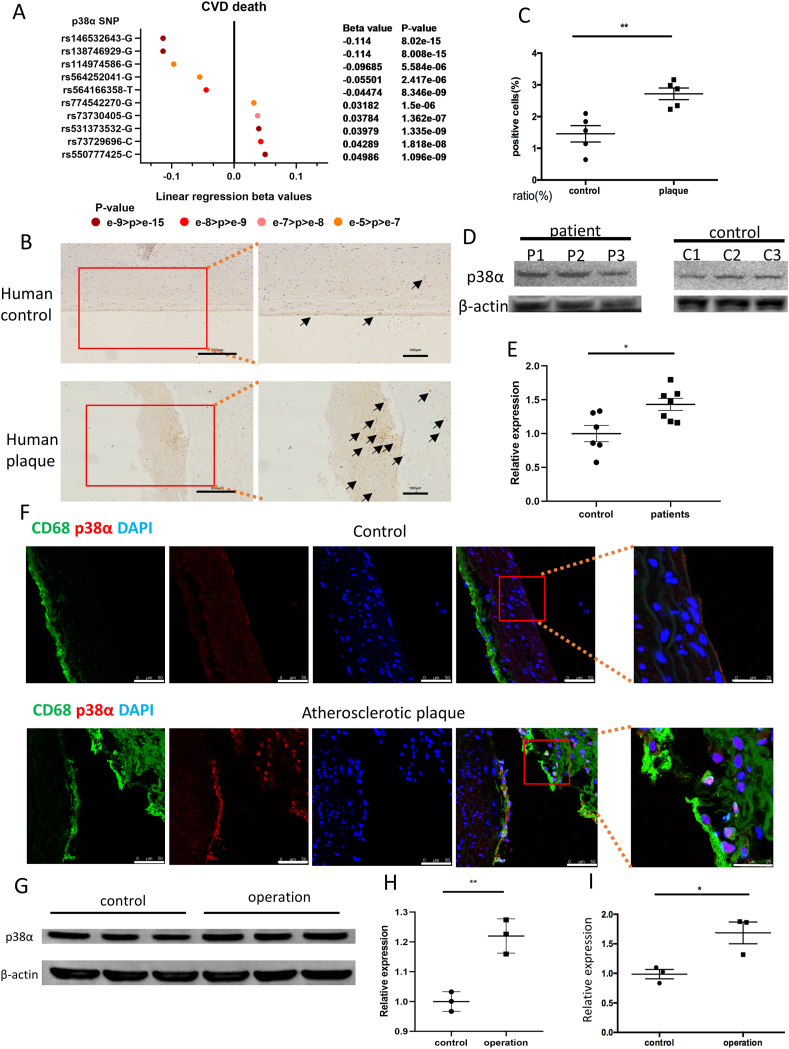
p38α MAPK expression is increased in response to atherosclerosis and vascular injury. (A) 10 SNPs of p38α related to cardiovascular disease identified by PhenoScanner database (n = 7637). (B) Representative images of p38α in atherosclerotic plaques of patients and healthy aortic vessels. (Bar = 250 μm and 100 μm). (C) Statistical results of the percentage of p38α-positive cells. Data are the mean ± SEM. (n = 5, **P < 0.01). (D) Representative western blotting of p38α protein in monocytes of 3 individual atherosclerotic patients and 3 healthy volunteers. (E) Statistical results of p38α protein expression determined by western blotting. Results are presented as the mean ± SEM of three independent experiments and normalized to the β-actin (n = 6 and 7, *P < 0.05). (F) Representative images of p38α on CD68-positive aortic vessels in ApoE-/- mice (Bar = 50 μm and 25 μm). (G) Representative western blotting of p38α protein in peritoneal macrophages of C57BL/6 mice at 3 days after vascular injury and control group. (H) Statistical results of p38α protein expression determined by western blotting. Results are presented as the mean ± SEM of three independent experiments and normalized to the control (n = 3, **P < 0.01). (I) RT-PCR analysis of p38α gene expression in peritoneal macrophages of C57BL/6 mice at 3 days after vascular injury (n = 3, *P < 0.05).

The p38α MAPK pathway plays an important role in regulating macrophages in atherosclerosis [22]. Using GWAS summary statistics in PhenoScanner, we identified 10 single nucleotide polymorphisms (SNPs) in p38α for the cardiovascular disease (CVD) such as hypertensive heart disease, congestive heart failure and atherosclerotic heart disease. The p-value showed a strong correlation between p38α and CVD (n = 7637, Fig. 1A). To investigate whether p38α MAPK levels are increased in patients with atherosclerosis, atherosclerotic plaques of patients and control aortic vessels were collected. We next explored the changes in p38α MAPK of aortic vessels. As shown in Fig. 1B and C, increased p38α MAPK levels were detected in patients with atherosclerosis. And, we analyzed p38α expression levels in monocytes of atherosclerotic patients (atherosclerotic plaque was diagnosed by ultra-sound). In light of the fact that non-atherosclerotic patients over 60 years of age people could not be easily found, we chose some 30–40 years of age people as control (Supplemental Table 1). We found that p38α levels were significantly higher in patients with atherosclerotic plaques as compared with the control group (Fig. 1D and E). p38α MAPK levels in ApoE^-/-^ mice with atherosclerosis was also examined. We found that p38α expression was significantly increased in atherosclerotic plaques (Fig. 1F). Endothelial recovery, also known as re-endothelialization, relates to the inhibition of plaque formation [[23], [24], [25]]. To confirm whether the p38α MAPK pathway in macrophages takes part in endothelial recovery, wire-injury of C57BL/6 mice was performed. We found that p38α MAPK expression of peritoneal macrophages was increased at 3 days after vascular injury in C57BL/6 mice (Fig. 1G–I).

### 2.2 Deficiency of p38α in Macrophages Promotes Re-endothelialization of Injured Arteries

**Fig. 2:**
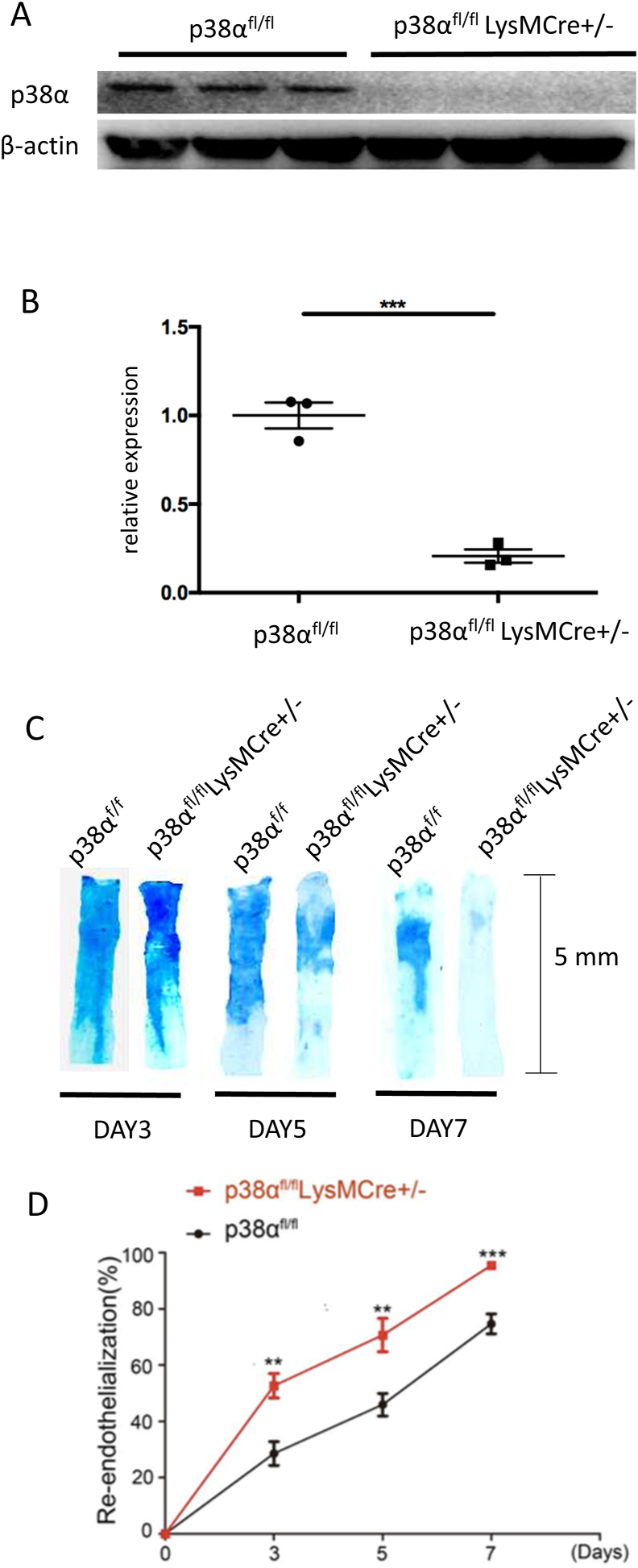
Deficiency of p38α in macrophages promotes re-endothelialization of injured arteries. (A) Western blotting analysis of p38α protein in peritoneal macrophages from p38α^fl/fl^LysMCre^+/-^ and p38α^fl/fl^ mice. (B) Statistical results of p38α protein expression determined by western blotting. Results are presented as the mean ± SEM of three independent experiments and normalized to the control (n = 3, ***P < 0.001). (C) Re-endothelialization quantified in Evans blue-stained carotid arteries at 3, 5 and 7 days after vascular injury (representative images). Blue staining indicates endothelial denudation. Scale bar, 5 mm. (D) Statistical results of Evans blue staining (n = 6 each group, **P < 0.01, ***P < 0.001).

To determine the role of macrophage p38α in endothelial repair, macrophage-specific p38α knockout (p38α^fl/fl^LysMCre^+/-^) mice were established. Peritoneal macrophages were extracted and p38α expression was detected in the mice. The protein expression of p38α was specifically absent in macrophages from p38α^fl/fl^LysMCre^+/-^ mice (Fig. 2A and B, P < 0.001).

To test the hypothesis concerning whether p38α in macrophages is involved in post-injury endothelial recovery, wire injury was performed in the carotid artery of p38α^fl/fl^LysMCre^+/-^ mice and littermate p38α^fl/fl^ mice (Fig. 2C). As shown in Fig. 2D, p38α^fl/fl^ endothelial cells (ECs) were more severely damaged after injury and recovered by approximately 28.55% at day 3, 45.93% at day 5 and 74.67% at day 7. In contrast, re-endothelialization in p38α^fl/fl^LysMCre^+/-^ mice was promoted post-injury and recovered by approximately 52.62% at day 3, 70.67% at day 5 and 95.48% at day 7.

### 2.3 Deficiency of p38α in macrophages promotes ECs proliferation and migration *in vitro*

**Fig. 3:**
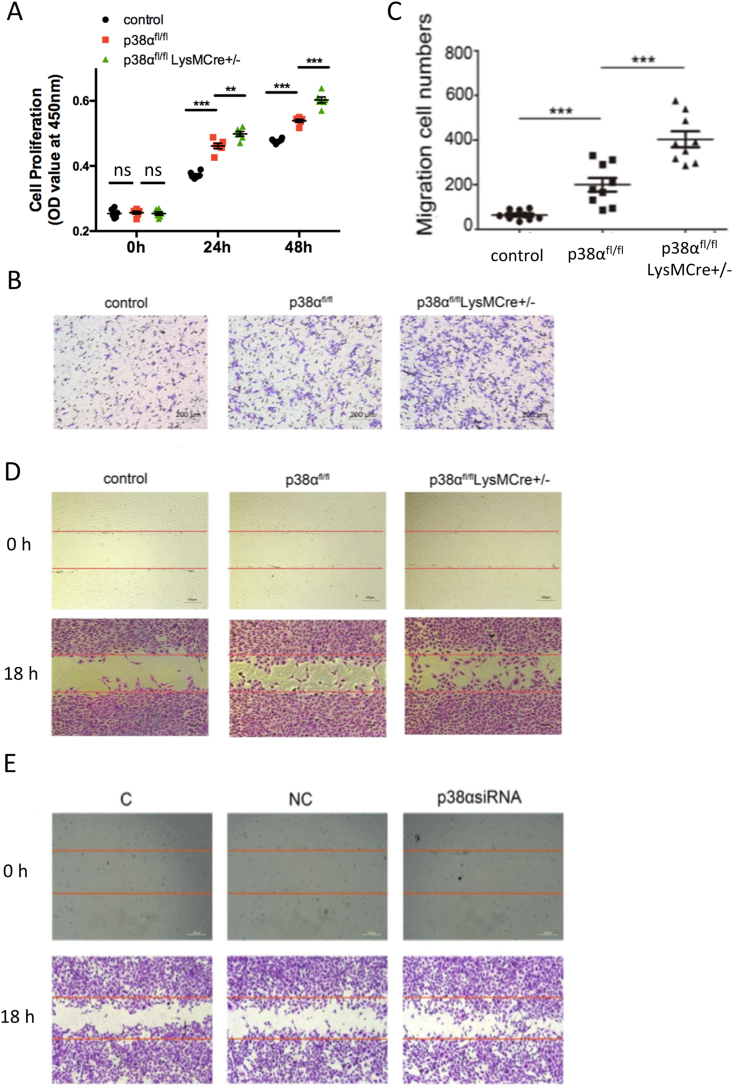
Deficiency of p38α in macrophage promotes ECs proliferation and migration. (A) HUVECs cultured in supernatants from macrophages of p38α^fl/fl^LysMCre^+/-^ mice and p38α^fl/fl^ mice for 24 and 48 h. Proliferation of HUVECs was determined by BrdU assays. Results are means ± SEM from three independent experiments performed in duplicate (n = 5 each group, ***P < 0.001; **P < 0.01; ns: P ≥ 0.05). (B) Transwell chamber migration assays of HUVECs cultured in conditioned medium of peritoneal macrophages from p38α^fl/fl^ and p38α^fl/fl^LysMCre^+/-^ mice for 8 h. Control: endothelial cell medium containing 5% bovine serum; p38α^fl/fl^: culture supernatant from p38α^fl/fl^ mouse peritoneal macrophages; p38α^fl/fl^LysMCre^+/-^: culture supernatant from p38α^fl/fl^LysMCre^+/-^ mouse peritoneal macrophages. (C) Migratory cells were counted and images were obtained in 10 random fields. Results are means ± SEM from three independent experiments performed in triplicate (n = 9, ***P < 0.001). (D) HUVEC monolayers were scratched by manual scraping and treated with ECM or conditioned medium of peritoneal macrophages from p38α^fl/fl^ and p38α^fl/fl^LysMCre^+/-^ mice for 18 h. Migration into the wound was imaged. The red line indicates the scratch edge. (E) HUVEC monolayers were scratched by manual scraping and treated with ECM or conditioned medium of THP1 cells transfected with siRNAs for 18 h. Migration into the wound was imaged. The red line indicates the scratch edge. C: THP-1 cells with no treatment; NC: THP-1 transfected with negative control siRNA; p38α siRNA: THP-1 transfected with 100 nM p38α siRNA. (For interpretation of the references to colour in this figure legend, the reader is referred to the Web version of this article.)

Next, we examined whether deficiency of p38α in macrophages affects ECs proliferation *in vitro*. Conditioned medium was extracted from the medium supernatant of p38α^fl/fl^ and p38α^fl/fl^LysMCre^+/-^ mice peritoneal macrophages, then co-cultured with HUVECs. As shown in Fig. 3A, HUVECs proliferation was significantly enhanced with the conditioned medium of p38α^fl/fl^LysMCre^+/-^ mice.

Endothelial cells migration is an essential process of re-endothelialization [26]. Thus, we assessed whether deficiency of p38α in macrophages affected ECs migration *in vitro*. HUVECs migration was promoted in the p38α^fl/fl^LysMCre^+/-^ group cultured with conditioned medium in Transwell chamber migration and wound healing assays. The migration cell numbers of the p38α^fl/fl^LysMCre^+/-^ group was 2.02 fold of the p38α^fl/fl^ group and was 6.30 fold of the control (Fig. 3B–D). In addition, THP-1 cells were used to confirm that HUVECs migration was promoted when p38α was knocked out. After transfection with 100 nM p38α siRNA, p38α was knocked down successfully in THP-1 cells (Supplemental Figure 2). When HUVECs were treated with conditioned medium from transfected THP-1 cells, the wound healing migration of HUVECs was increased visibly (Fig. 3E).

### 2.4 Deficiency of p38α in macrophages reduces expression of Interleukin-6

**Fig. 4:**
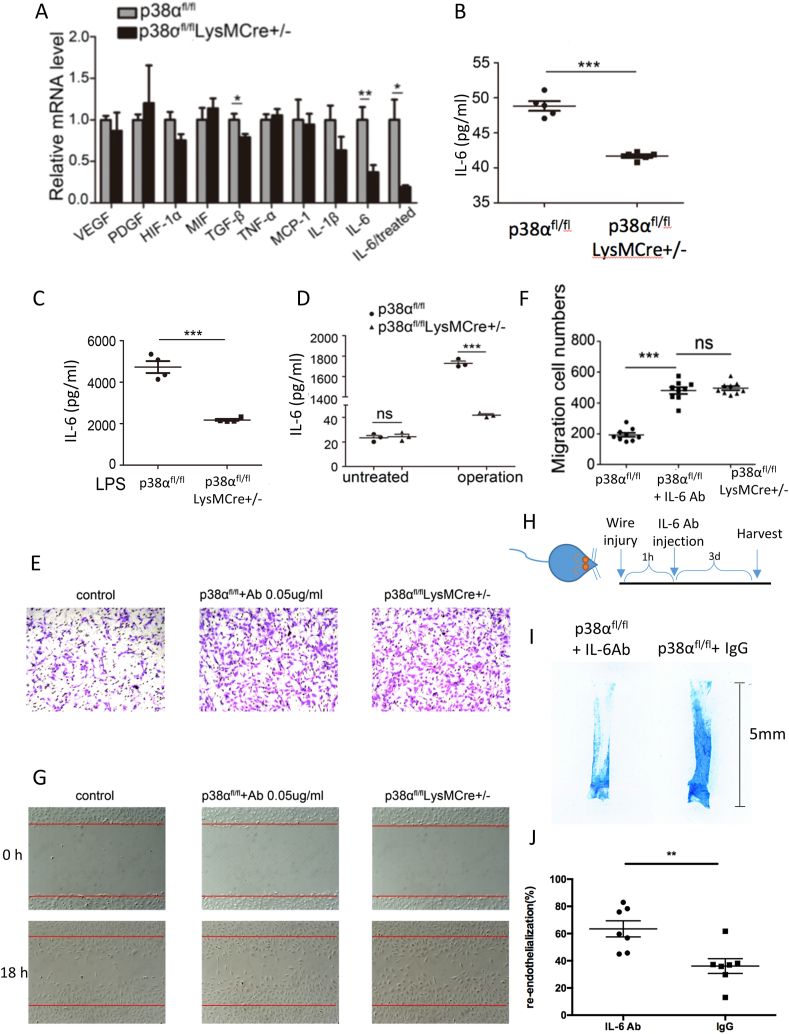
Deficiency of p38α in macrophages reduces secretion of interleukin-6. (A) Detection of cytokines and inflammatory factors by RT-PCR. Peritoneal macrophages were stimulated with 100 ng/ml LPS for 12 h. Results are means ± SEM from three independent experiments (n = 3 each group, **P < 0.01, *P < 0.05, ns: P ≥ 0.05). (B) Detection of secreted IL-6 in culture supernatants of experimental group and control group macrophages by ELISA. Results are means ± SEM from three independent experiments (n = 5, ***P < 0.001). (C) Peritoneal macrophages were stimulated with 100 ng/ml LPS for 12 h and then secretion of IL-6 in culture supernatants was detected by ELISA. Results are means ± SEM from three independent experiments (n = 4, ***P < 0.001). (D) Detection of IL-6 in plasma drawn from the medial canthus at 12 h after wire injury by ELISA. Untreated: plasma of mice without guide wire injury. Operation: plasma of mice with guide wire injury. Results are means ± SEM from three independent experiments (***P < 0.001, ns: P ≥ 0.05, n = 3 each group). (E) Transwell chamber migration assays of HUVECs cultured in conditioned medium of peritoneal macrophages for 8 h. The results showed that ECs migration was enhanced after treating p38α^fl/fl^ mouse peritoneal macrophages with an IL-6 -neutralizing antibody. control: endothelial cell medium containing 5% bovine serum. p38α^fl/fl^ + Ab 0.05 μg/ml: culture supernatant from p38α^fl/fl^ mouse peritoneal macrophages treated with 0.05 μg/ml IL-6-neutralizing antibody. p38α^fl/fl^LysMCre^+/-^: culture supernatant from p38α^fl/fl^LysMCre^+/-^ mouse peritoneal macrophages without treatment. (F) Migratory cells were counted and images were obtained in 10 random fields. Results are means ± SEM from three independent experiments performed in triplicate (n = 9 each group, ***P < 0.001, ns: P ≥ 0.05). (G) HUVEC monolayers were scratched by manual scraping and treated with ECM or conditioned medium of peritoneal macrophages for 18 h. Migration into the wound was imaged. The red line indicates the scratch edge. (H) The schematic diagram of IL-6 antibody or IgG injection. (I) IL-6-neutralizing antibody was applied at 1 h after carotid artery injury, and re-endothelialization was quantified at 3 days after vascular injury. Blue staining indicates endothelial denudation. Scale bar, 5 mm. (J) Statistical results of Evans blue staining (**P < 0.01) compared with control mice (n = 7 each group). (For interpretation of the references to colour in this figure legend, the reader is referred to the Web version of this article.)

Many investigations have demonstrated that macrophages regulate the expression of inflammatory factors [27,28]. After knocking out p38α in macrophages, inflammatory factors secreted by macrophages may be altered, and then affect the inflammatory response and repair of vascular injury. Therefore, we detected the changes in the transcriptional level of angiogenesis-related inflammatory factors (VEGF, PDGF, HIF-α, MIF, TGF-β, TNF-α, MCP-1, IL-1β and IL-6) in the peritoneal macrophages of p38α^fl/fl^ and p38α^fl/fl^LysMCre^+/-^ mice. As shown in Fig. 4A, the mRNA level of IL-6 in macrophages was reduced significantly after knocking out p38α. Thus, it may reduce the inflammatory response at the injury site and promote vascular repair. To test our hypothesis, lipopolysaccharide (LPS) was used to stimulate macrophages and IL-6 was decreased significantly in the p38α^fl/fl^LysMCre^+/-^ mice (Fig. 4A, P < 0.05). In addition, the secretion of IL-6 in macrophage culture supernatants was decreased after knocking out p38α in macrophages with or without stimulation (Fig. 4B and C). Wire injury was performed in the carotid artery of control and p38α^fl/fl^LysMCre^+/-^ mice, and then secretion of IL-6 was detected in plasma at 12 h after injury. As shown in Fig. 4D, the plasma IL-6 level in p38α^fl/fl^LysMCre^+/-^ mice was decreased significantly compared with the control group.

### 2.5 Inhibiting IL-6 Activity in p38α^fl/fl^ Mouse Macrophages by a Neutralizing Antibody Promotes Re-endothelialization

Deficiency of p38α in macrophages decreased the expression and secretion of IL-6. Thus, the inflammatory response was ameliorated at the injury site. To verify the observation, the neutralizing antibody of IL-6 was used to treat control peritoneal macrophages and the supernatant was collected to culture ECs. In Transwell chamber migration assays, the migration of cells was significantly increased after treatment with the neutralizing antibody compared with the p38α^fl/fl^ group by about 2.49 fold (Fig. 4E and F). Similarly, wound healing migration showed that inhibiting IL-6 in p38α^fl/fl^ mouse macrophages by the neutralization antibody also promoted ECs migration (Fig. 4G).

Tocilizumab is the IL-6 receptor alpha inhibitor. HUVEC was treated by recombinant human IL-6 protein with or without Tocilizumab. The migration of HUVEC treated by IL-6 was reduced and Tocilizumab reversed the decreased migration (Supplemental Figure 4). Because p38α^fl/fl^ mice macrophages treated with the IL-6-neutralizing antibody promoted the migration of the endothelial cells, mouse IL-6 antibody or IgG as a negative control was intraperitoneally injected at 1 h after carotid artery injury. The IL-6 neutralizing antibody could block the IL-6 *in vivo* at 24 h after injection (Supplemental Figure 3). Then re-endothelialization was quantified at 3 days after vascular injury (Fig. 4H). As shown in Fig. 4I and J, the ECs of p38α^fl/fl^ injected with IgG recovered by approximately 36.09% at day 3. In contrast, re-endothelialization in p38α^fl/fl^ mice injected with IL-6 antibody was promoted post-injury and recovered by approximately 63.46% at day 3.

### 2.6 MKL1 is the target protein of p38α phosphorylation in regulating IL-6 expression

**Fig. 5:**
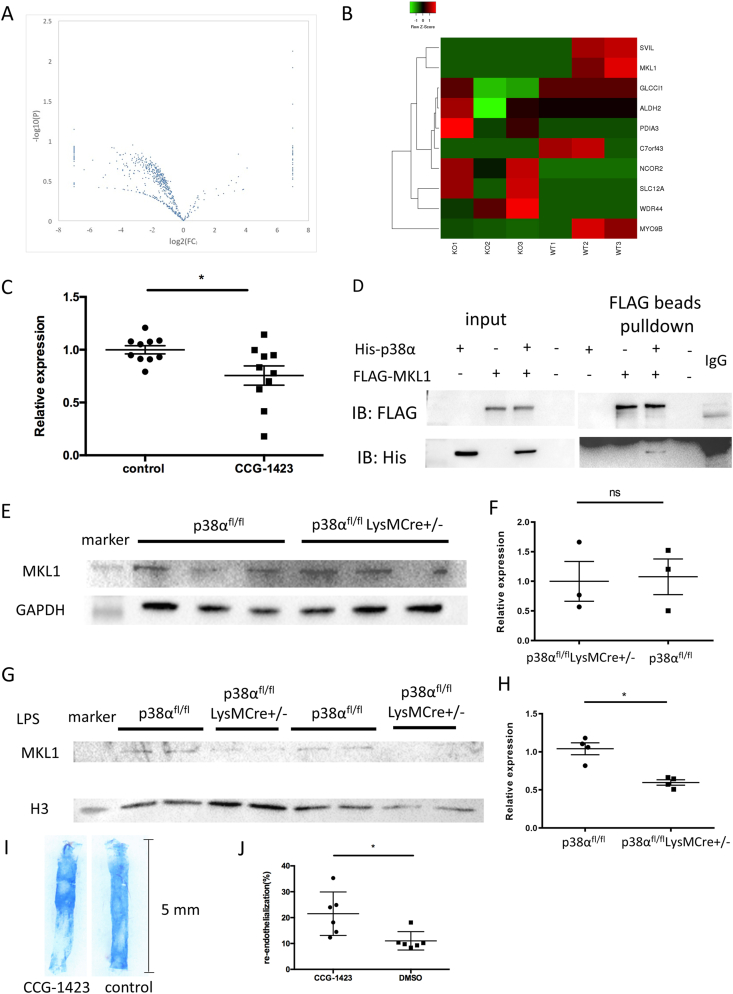
Mutation of the p38α phosphorylation target in MKL1 sites reduces secretion of IL-6. (A) Volcano plot of quantitative phosphoproteomics showing changes in 846 protein phosphorylations. (B) Heat map of the top 10 proteins with the lowest differential *P*-value. (C) RT-PCR analysis of IL-6 mRNA expression of in RAW264.7 cells treated with 10 μmol/L CCG-1423 for 18 h. Results are means ± SEM from three independent experiments (n = 9, *P < 0.05). (D) Validation of the MKL1 interaction with p38α in 293Ts cells. 293Ts cells were transfected with pcDNA3.1-V5/hisB-p38α plasmid (His-p38α) and/or pcDNA3.1-C3Flag-MKL1 (Flag-MKL1) for 48 h before whole cell lysates were prepared for Flag-bead pulldown assays (n = 3 each group). (E) Western blotting analysis of MKL1 in peritoneal macrophages from p38α^fl/fl^LysMCre^+/-^ and p38α^fl/fl^ mice. (F) Statistical results of MKL1 protein expression determined by western blotting. Results are presented as the mean ± SEM of three independent experiments and normalized to the control (n = 3, ns: P ≥ 0.05). (G) Western blotting analysis of MKL1 in peritoneal macrophages nucleoprotein of p38α^fl/fl^LysMCre^+/-^ and p38α^fl/fl^ mice. (H) Statistical results of MKL1 protein expression determined by western blotting. Results are presented as the mean ± SEM of three independent experiments and normalized to the control (n = 4 each group, *P < 0.05). (I) CCG-1423 was injected at 1 mg/kg/d daily for 2 consecutive weeks before carotid artery injury, and re-endothelialization was quantified at 3 days after vascular injury. Blue staining indicates endothelial denudation. Scale bar, 5 mm. (J) Statistical results of Evans blue staining (n = 6 each group, *P < 0.05). (For interpretation of the references to colour in this figure legend, the reader is referred to the Web version of this article.)

Since p38α is a kind of protein kinase, we determined whether p38α deficiency affected the phosphorylation level of proteins and inhibited their corresponding functions in IL-6 expression. We compared the phosphorylation level of proteins in peritoneal macrophages of p38α^fl/fl^ and p38α^fl/fl^LysMCre^+/-^ mice by quantitative phosphoproteomic method. A volcano plot showed changes in 846 phosphorylated proteins. Phosphorylation levels of 660 proteins were downregulated and those of 186 proteins were upregulated (Fig. 5A and Supplemental Data File 1). A Heat map showed the top 10 proteins with the lowest differential *P*-value (Fig. 5B). Megakaryocytic leukemia 1 (MKL1) is a key cofactor of nuclear factor-κB (NF-κB)/p65 participating in tumor necrosis factor-α (TNF-α) induced pro-inflammatory transcription in macrophages [[29], [30], [31]]. The phosphorylation level of MKL1 was significantly downregulated in the p38α^fl/fl^LysMCre^+/-^ group. To investigate whether MKL1 was involved in the regulation of IL-6 expression by p38α, MKL1 inhibitor CCG-1423 was used to treat RAW264.7 cells. We found that the mRNA level of IL-6 was reduced in treated cells, indicating that MKL1 promoted IL-6 expression (Fig. 5C). We further validated the interaction of MKL1 with p38α by pulldown assays, indicating p38α and MKL1 binding in cells (Fig. 5D). And the phosphorylation level of MKL1 in 293T cells with MKL1 and p38α expression plasmids was promoted by 1.65 fold (Supplemental Figure 5 and Supplemental Data File 2). Because MKL1 is a cofactor of transcription, protein expression of MKL1 in the nucleus demonstrates its function. Total protein expression of MKL1 in peritoneal macrophages of p38α^fl/fl^ and p38α^fl/fl^LysMCre^+/-^ groups showed no difference (Fig. 5E and F). However, in the nucleus, we found that MKL1 expression was lower when p38α was knocked out (Fig. 5G and H). Next, the MKL1 inhibitor CCG-1423 was injected into the mice and DMSO was injected as control. Wire injury was performed in the carotid artery, and the level of re-endothelialization was observed after 3 days. The CCG-1423 could block the IL-6 *in vivo* at 24 h after injury (Supplemental Figure 6). As a result, the control group showed more severe damage and recovered by approximately 18.61% at day 3 and the CCG-1423 group recovered by approximately 31.39% at day 3 (Fig. 5I and J).

### 2.7 p38α Mediates S544/S549 and T545/S549 Phosphorylation Motif Which Regulates the Expression of IL-6 in mouse

**Fig. 6:**
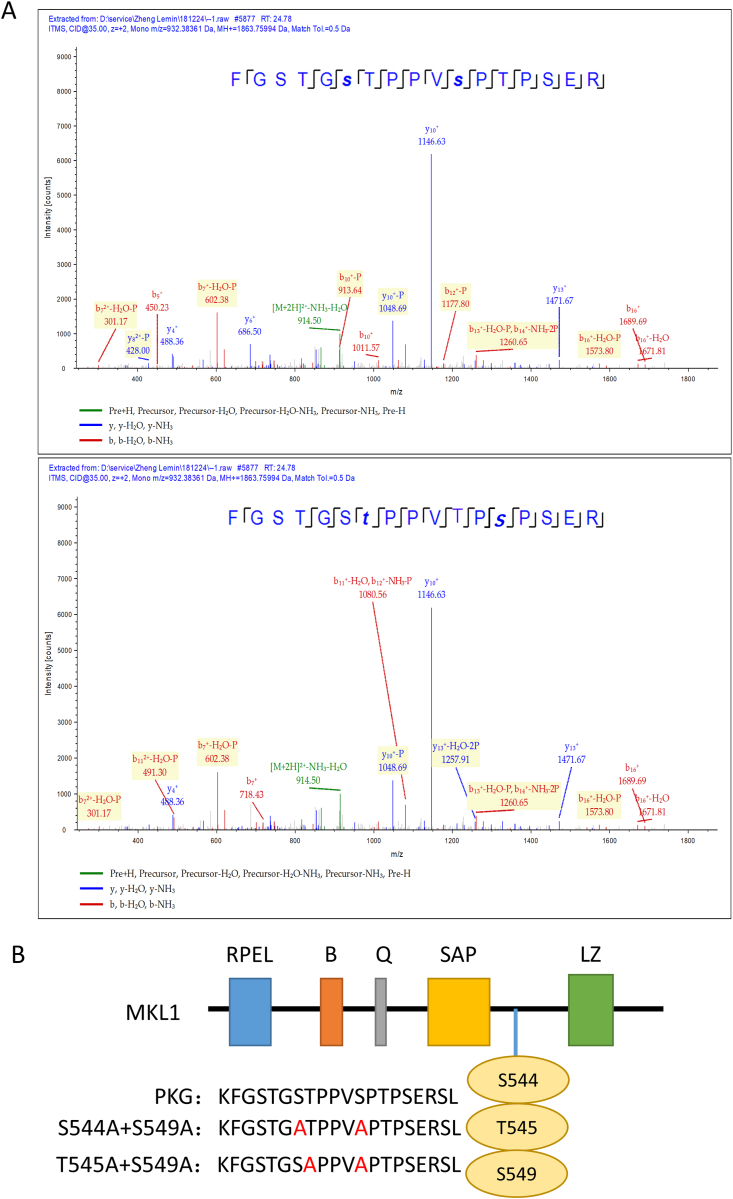
p38α mediates S544/T545/S549 phosphorylation of MKL1. (A) Sequence confirmation of phosphorylation level of MKL1 in p38α^fl/fl^ mice. (B) Mapping of MKL1 amino acid mutation sites that are also phosphorylation sites.

**Fig. 7:**
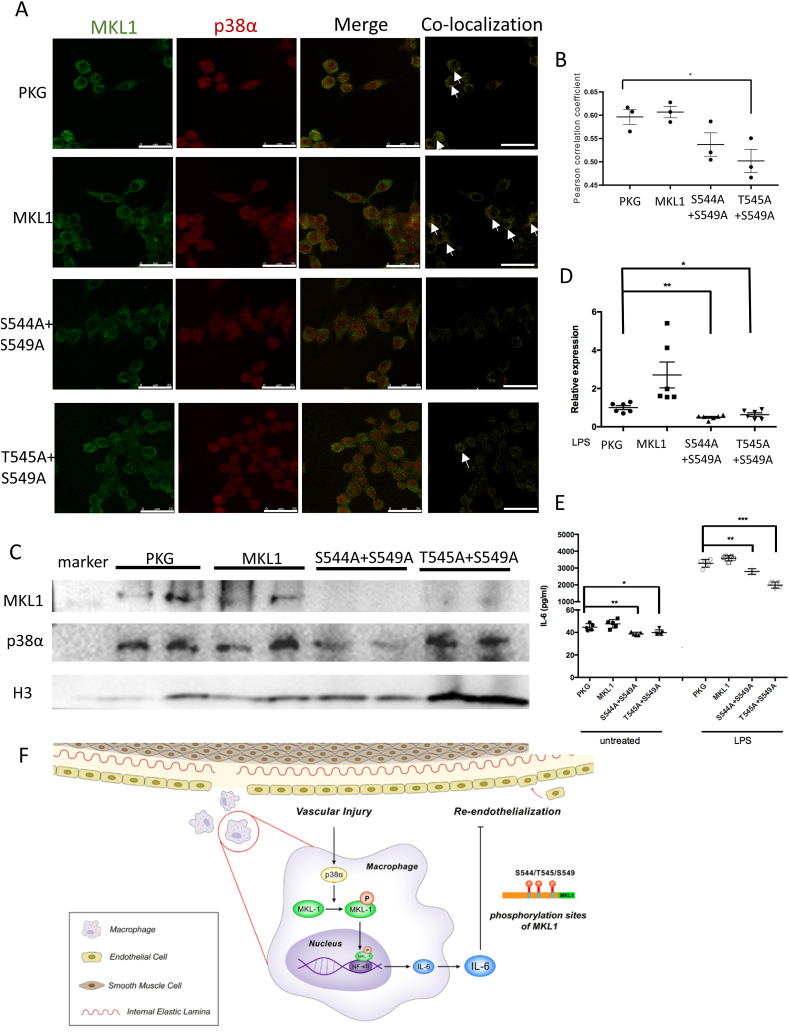
p38α regulates the expression of IL-6 by mediates phosphorylation of MKL1. (A) Representative images of the co-localization of MKL1 and p38α in LV-PKG, LV-MKL1, LV-S544A+S549A, and LV-T545A+S549A-infected RAW264.7 cells (Bar=25 μm). (B) Statistical results of the Pearson correlation coefficient of the co-localization of MKL1 and p38α. The results are means ± SEM of three independent experiments and normalized to the control (n=3, PKG vs T545A+S549A group, *P<0.05). (C) Representative western blotting of MKL1 and p38α proteins in infected RAW264.7 cells. (D) mRNA expression of IL-6 in peritoneal macrophages infected by lentiviruses after 100 ng/ml LPS treatment for 12 hours. Results are the means ± SEM from three independent experiments (n=6, PKG vs S544A+S549A group and PKG vs T545A+S549A group, **P<0.01, *P<0.05). (E) Detection of secreted IL-6 from infected RAW264.7 cells stimulated with or without 100 ng/ml LPS for 12 h by ELISA. Results are means ± SEM from three independent experiments (n=5, PKG vs S544A+S549A group and PKG vs T545A+S549A group, ***P<0.001, ** P<0.01, *P<0.05). (F) A Schematic model for the role of p38α in re-endothelialization.

Because p38α is a phosphorylase that phosphorylates some specific sites on proteins for downstream biological effects, we determined which sites on MKL1 were phosphorylated by p38α. Through quantitative phosphoproteomics, we found that three major sites were phosphorylated in mice macrophages MKL1, S544/T545/S549, and the phosphorylation level of S549 was the highest (Fig. 6A). To testify the S/T-P motif reported by Panayiotou et al. in NIH3T3 cells [32], one lentivirus was constructed in which S544 and S549 were mutated to alanine and the other lentivirus was constructed in which T545 and S549 were mutated to alanine (Fig. 6B). The lentiviruses were used to infect RAW264.7 cells which were treated with LPS. We found that after overexpression of MKL1, the co-localization of MKL1 and p38α was increased compared with the control group, and the co-localization area was decreased significantly after T545 and S549 mutation (Fig. 7A and B). We also observed that the expression of MKL1 in the nucleus was decreased in mutation groups (Fig. 7C). In addition, the expression levels of IL-6 mRNA in mutation groups were decreased after LPS stimulation (Fig. 7D). Moreover, the secreted level of IL-6 in culture media of mutation groups was decreased regardless of LPS treatment (Fig. 7E).

## 3 Discussion

In the present study, we demonstrated the effects of p38α MAPK in macrophages on the progression of re-endothelialization. We confirmed that p38α MAPK expression was increased in macrophages in response to vascular injury. We observed that knocking out p38α in macrophages reduced the expression of IL-6 by downregulating phosphorylation of MKL1 and promoted re-endothelialization of injured arteries. The three sites, S544/T545/S549, in mice macrophages MKL1 were phosphorylated by p38α and S549 was essential. We demonstrated that inhibition of MKL1 by CCG-1423 reduced the expression of IL-6 and promoted re-endothelialization (Fig. 7F).

The pathogenesis of coronary heart disease is due to atherosclerosis caused by lipid deposition and cholesterol accumulation [33]. The main clinical treatment of CAD is interventional therapy, but it is accompanied by vascular damage, causing inflammation of the vascular wall and restenosis [34]. Macrophages secrete a variety of cytokines in a paracrine manner to participate in tissue repair and regeneration. p38α MAPK regulates macrophages to repair injuries. Deficiency of p38α in macrophages ameliorates acute liver injury in mice [35]. Endothelial cells recovery is inversely related to neointima formation during atherosclerosis and post-injury restenosis. p38α deficiency in macrophages leads to enhanced macrophage apoptosis and expression of markers for advanced plaque progression. It is still unknown whether p38α deficiency in macrophages influences vascular repair or re-endothelialization. In this study, we established an injury model of the mouse carotid artery by removing the endothelium with a flexible wire, which allowed us to examine the mechanisms between vascular biology and atherosclerosis.

Recent studies have shown that important events during atherogenesis, such as endothelial apoptosis, expression and secretion of IL-8 by human monocyte–derived macrophages, are induced by LDL via p38 MAPKs and NF-κB [36]. Deficiency of p38α MAPK in macrophages does not affect the pathogenesis of atherosclerosis in ApoE^-^^/-^ mice [37]. In addition, it has been shown that p38α MAPK is required for neointima formation after vascular injury and deletion of p38α in macrophages partially impairs lipopolysaccharide-induced cellular activation [20,38]. In general, deficiency of p38α MAPK in macrophages influences the process of endothelial repair, and the mechanisms are still unclear. Here, we found that the expression level of p38α protein in primary peritoneal macrophages of mice was increased significantly at 3 days after wire injury of the C57BL/6 mouse carotid artery. This result indicates that p38α MAPK in macrophages may be involved in regulating vascular injury and repair. Next, we used a guide wire to injure carotid arteries of macrophage-specific p38α knockout (p38α^fl/fl^LysMCre^+/-^) mice and found that p38α deficiency in macrophages promoted re-endothelialization after injury in the mice. Furthermore, we collected conditioned medium of primary peritoneal macrophages to culture human umbilical vein endothelial cells and found that the migration and proliferation of HUVECs were also promoted. In agreement with previous studies, our results suggest that macrophages play an important role in regulation of the inflammatory response and re-endothelialization after vascular injury [39].

There is the possibility of macrophage-mediated ECs recovery. It has been reported that inflammation plays a major role in the development of restenosis and atherosclerosis [12,40]. Vascular damage can stimulate the body to produce a large number of inflammatory factors and proinflammatory cytokines. Various mediators form a complex network that regulates the formation of atherosclerosis and affects re-endothelialization after vascular injury [41]. Macrophages play a central role in inflammation and phagocytosis through the ability to recognize and phagocytize apoptotic cells. Macrophages arrive at the lesion, extend filopodia or lamellipodia, and mediate the repair of brain vascular rupture through adhesion and traction [42]. After acute myocardial infarction, IL-17A aggravates inflammatory response and ischemic injury by inducing macrophages infiltration, and NLRP3 inflammasome and p38 MAPK are significantly activated [43]. It was also reported that chemokine CCL2 could recruit monocytes and activate p38 pathway *in vitro* [44], while endothelial cell could express CCL2 [[45], [46]]. It might be the similar progress in vessel injury. After tissue injury, macrophages infiltrate into the injury site and secrete a series of inflammatory factors, such as IL-6 and TNF-α, to participate in the regulation of the repair [47]. p38α MAPK plays an important role in inflammatory responses, tissue repair and endothelial activation [48,49]. Recent study demonstrated that p38α in mesenchymal cells restrains a TGF-β-induced tumor angiogenesis program including their ability to transdifferentiate into endothelial cells [50]. This might suggest that there are more other factors involved in the regulation of p38α on endothelial injury. Therefore, we believe that p38α deficiency in macrophages may affect the secretion of related cytokines and influence the proliferation and migration of endothelial cells, and thus affect the process of re-endothelialization.

To further determine whether macrophages affect the inflammatory response through paracrine mechanisms after deficiency of p38α, we examined the secretion of inflammatory factors. After knocking out p38α in macrophages, the expression level of IL-6 in macrophages was decreased significantly. IL-6 is an important cytokine involved in various physiological metabolic processes. A large number of studies have shown that the level of IL-6 is closely related to the occurrence of cardiovascular diseases and plays an important role in endothelium dysfunction. In myocarditis, coronary heart disease and other chronic immune diseases, the level of IL-6 is significantly elevated and related to disease severity [51]. Continuous mechanical stimulation could induce endothelial cells to secrete IL-6, which plays an important role in the regenerative and inflammatory responses [52]. In obesity and type 2 diabetes mice, the interactions between TNF-a and IL-6 aggravate oxidative stress and contribute to coronary endothelial dysfunction [53]. Besides, T cells are both important source and target of IL-6. IL-6 is produced by T cells during chronic inflammation, such as inflammatory-bowel diseases and atopic dermatitis [54,55]. Studies have demonstrated IL-6 plays a prominent role in regulating the balance between Th17 cells and regulatory T cells [56], which affects inflammation. Inhibiting T lymphocyte infiltration and decreasing Th17 cells differentiation could provide neuroprotection of ischemic stroke [57]. It was also reported that IL-6 produced by mature adipocytes and fibroblast supports breast tumor growth and metastasis [58,59]. These studies suggest the effect of IL-6 from other cells. Here, we revealed that deficiency of p38α in macrophages reduced secretion of inflammatory factors such as IL-6 and promoted re-endothelialization after vascular injury. Other cytokines, like TGF-β and HIF-1a also showed difference but not as significant as IL-6. So, p38α didn't regulate IL-6 specifically, but the change of IL-6 was a very important factor in this model. Inhibiting the activity of IL-6 in p38α^fl/fl^ mouse macrophages by a neutralizing antibody promoted re-endothelialization, which confirmed our results. Because p38α MAPK is a kind of protein kinase, we determined whether deficiency of p38α MAPK affected the level of phosphorylation of some proteins to suppress their corresponding functions in IL-6 expression.

Megakaryocytic leukemia 1 (MKL1), also known as myocardin-related transcription factor A (MRTF-A), is a Rho-Rock signaling-responsive coactivator of serum response factor (SRF), which regulates a variety of cellular functions [[29], [30], [31]]. MKL1 regulates cardiac and vascular remodeling, and plays pathological roles in cardiovascular diseases [[60], [61], [62]]. An et al. reported that expression of MKL1 in macrophages is involved in the pathogenesis of atherosclerosis [63]. Recently, some studies have correlated MKL1 as a transcription factor with cardiovascular diseases [64,65]. Xu et al. reported that MKL1 activates proinflammatory transcription and is a coordinator defining the epigenetic landscape for p65 [[21], [66]]. Several inflammatory factors are encoded by NF-κB target genes, of which most notably is IL-6 [67]. Rho- and ERK-dependent pathways induce MKL phosphorylation, and Panayiotou et al. identified multiple sites for MKL phosphorylation that contributes to transcriptional activation [32]. We found that phosphorylation of MKL1 was increased to a lesser extent when p38α was knocked out. We verified that MKL1 was phosphorylated by p38α *in vivo*. The re-endothelialization of mice injected with an MKL1 inhibitor was also promoted. These findings suggest that MKL1 is phosphorylated by p38α and then promotes the combination of MKL1 and NF-κB. Furthermore, we determined the sites of phosphorylation. Quantitative phosphoproteomics revealed that the phosphorylation of S544/T545/S549 in MKL1 in macrophage-specific p38α-knockout mouse was most evidently declined. The phosphorylation levels of S549 was highest. S544/T545/S549 in mouse are the same motifs as S449/T450/S454 phosphorylated in human MKL1 by extracellular signal-regulated kinase 1/2 reported by Muehlich et al. [68]. In addition, these sites contain S/T-P motifs that are reported to be essential for transcriptional activation [32]. After substituting the S544/T545 and S544/S549 sites with alanine, we found attenuation of MKL1 translocation from the cytosol to nucleus, which reduced proinflammatory stimuli by potentiating NF-κB/p65-dependent transcription of MKL1. This has also been reported by Kojonazarov et al. in cardiac fibroblasts [69]. With the decline of p38α binding to MKL1 after point mutation, expression of IL-6 was downregulated. These findings suggest that p38α activates MKL1 by phosphorylation of S544/T545 and S544/S549 sites, promoting its role as a transcriptional cofactor for NF-κB. Although the two pairs of mutations are different, it appeared to be similar in terms of function. Thus, S549 may be a more important site for phosphorylation.

Our findings imply a possible role of macrophages in re-endothelialization. Deficiency of p38α MAPK in macrophages rescued the inflammation by decreasing phosphorylation of MKL1 and reducing the expression of IL-6, and then regulating the inflammatory response. Knocking out p38α in macrophages ameliorated the inflammatory reaction in the damaged area and promoted re-endothelialization after vascular injury. Besides, macrophages can be converted into endothelial-like cells [70], and whether p38α deficiency in macrophages affects endothelial cell transdifferentiation or the ability to recruit endothelial progenitor cells is unclear. In subsequent studies, we can further explore the other aspects of p38α deficiency in macrophages, which influence re-endothelialization.

In conclusion, p38α deficiency in macrophages decrease the secretion of IL-6 and promoting re-endothelialization of injured arteries. Our study provides a new theoretical support for the investigation of vascular injury and re-endothelialization, and new therapeutic targets for clinical treatment of coronary heart disease such as atherosclerosis.

## 4 Materials and methods

### 4.1 Human samples

Human aortic tissues for immunohistochemical staining were obtained from patients with atherosclerotic plaques, and control tissues were obtained from organ donors. Samples for immunohistochemical staining were processed for paraffin embedding and cut 7 μm thick. Written informed consent was obtained from patients and organ donors, and the study protocol was approved by Hebei Medical University Ethics Committee.

Human blood samples were obtained from patients with atherosclerotic plaques, and control blood samples were obtained from healthy volunteers. The monocytes from whole blood were isolated using OptiPrep (07820, STEMCELL Technologies Inc., Vancouver, Canada). The study protocol was approved by Institutional Review Board of Beijing Tiantan Hospital Affiliated to Capital Medical University.

### 4.2 Animals

Macrophage-specific knockout (p38α^fl/fl^LysMCre^+/-^) mice with the C57BL/6 background were obtained from Dr. Hao Ying (Chinese Academy of Sciences) [35]. Briefly, mice with the p38α floxed allele (p38α^fl/fl^) were crossed with LysMCre mice expressing Cre in macrophages to generate macrophage-specific knockout (p38α^fl/fl^LysMCre^+/-^) mice. Littermate p38α^fl/fl^ mice lacking the Cre transgene were used as controls. All mice were maintained with *ad lib* access to pellet food and water. All the experiments were performed in mice aged 8–12 weeks. Experimental protocols were approved by Peking University Institutional Animal Care and Use Committee (LA2018088). Animal husbandry and experimental procedures were carried out strictly in accordance with the ethical regulations enforced and conformed to the Guide for the Care and Use of Laboratory Animals (National Institutes of Health). The identification of Cre gene is in the Supplemental Figure 1.

For the CCG-1423 injection, 3.5 mg CCG-1423 was dissolved by 50ul DMSO, and then dissolved in 6950ul corn oil. The suspension was sub-packaged into 14 injectors and conserved in −80 °C. Every mouse (25g) was injected by 50ul suspension every day (1 mg/kg/d). 50ul DMSO in 6950ul corn oil without CCG-1423 was injected as control.

### 4.3 Cell culture and treatments

Human umbilical vein endothelial cells (HUVECs) were bought from ATCC (PCS-100-010, VA, USA) and cultured in endothelial cell medium (1001, Sciencell, CA, USA) containing 5% bovine serum, 1% endothelial cell growth supplement, and 1% penicillin/streptomycin solution.

RAW264.7 (3111C0001CCC000146) and 293T (3111C0002000000112) cells were obtained from the National Infrastructure of Cell Line Resource (Beijing, China). RAW264.7 and 293T cells were cultured in DMEM (SH30243.01, Hyclone, UT, USA) containing 10% fetal bovine serum (SH30070.03, Hyclone). All cells were cultured in cell culture incubator containing 5% CO_2_ at 37 °C.

For transfection, 293T cells were seeded in 100-mm culture dishes and cultured overnight. Then, the cells were transfected with 10 μg plasmid DNA mixed with 20 μl jetPRIME reagent and 500 μl jetPRIME buffer (n 114–07, Polyplus-transfection, Illkirch, France). The medium was replaced at 4 h after transfection, and cells were collected after 48 h. The synthesis sequence of the plasmid is in the Supplemental Data File 3.

### 4.4 Small interfering RNA transfection

siRNAs were synthesized by Shanghai GenePharma Co. (Shanghai, China). p38α siRNAs were sense 5′-UGAAGACUGUGAGCUGAAG-3′ and antisense 5′-CUUCAGCUCACAGUCUUCA-3’.

THP-1 cells were seeded in 6-well plates and stimulated by 100 ng/mL phorbol 12-myristate 13-acetate. At 30%–50% confluence, the cells were transfected with p38α siRNA in Opti-MEM with siRNA-mate (GenePharma Co.) for 4–6 h and then incubated in DMEM with 10% FBS for 24–48 h. The cells were then used in further experiments.

### 4.5 Wire-injury of the mouse carotid artery

Wire-injury of the mouse carotid artery was performed in male p38α^fl/fl^LysMCre^+/-^ and p38α^fl/fl^ mice aged 10–12 weeks as described by Lindner et al. [71]. Mice were anesthetized by intraperitoneal injection of pentobarbital sodium (50 mg/kg). Then, the mice were subjected to injury of the left carotid artery by a 0.38 mm-diameter flexible angioplasty guide wire. The wire was passed 3 times to induce endothelial damage before it was removed. The Evans blue dye was injected at 3, 5, and 7 days after wire-injury, and the area of remaining denudation was determined. In the Evans blue dye, mice were injected with 50 μl 5% Evans blue dye into the tail vein, and the mice were sacrificed after 5 min of internal circulation. Then the mice were anesthetized by intraperitoneal injection of pentobarbital sodium (50 mg/kg) and immediately infused with PBS buffer, and then harvested the carotid arterials.

### 4.6 Transwell chamber migration assay

Cell migration was measured in a modified Boyden chamber (Minicell, Millipore, MA, USA) with an 8 μm-pore polycarbonate filter inserted in 24-well plates. Endothelial cell medium was used to culture peritoneal macrophages from p38α^fl/fl^ and p38α^fl/fl^LysMCre^+/-^ mice. Then, the supernatant was collected as conditioned medium to culture HUVECs. HUVECs (1 × 10^5^/well) in endothelial cell medium without bovine serum were plated in the upper chamber as a control, and culture supernatant from different peritoneal macrophages was used to culture HUVECs for 8 h. The lower chamber was filled with 500 μl endothelial cell medium containing 5% bovine serum. Migrated cells were fixed and stained with 0.01% (m/v) crystal violet for 30 min. Cells in each chamber were photographed under an inverted microscope (DM3000, Leica, German).

### 4.7 Wound healing assay

Wound healing assays also reflect the cell migratory capacity. In this experiment, HUVECs were seeded in 12-well plates with endothelial cell medium containing 5% bovine serum and cultured until monolayer formation. Then, HUVECs were scratched with a 200-μl micropipette tip. Endothelial cell medium was used to culture peritoneal macrophages from p38α^fl/fl^ and p38α^fl/fl^LysMCre^+/-^ mice. Then, the culture supernatant was collected as conditioned medium to culture HUVECs. HUVECs incubated in endothelial cell medium without bovine serum were used as a control. After 18 h, cells were photographed under the inverted microscope.

### 4.8 5-Bromo-2-deoxyuridine proliferation assay

Cell proliferation was analyzed by a 5-bromo-2-deoxyuridine (BrdU) assay. Briefly, equal numbers (6 × 10^3^ cells/well) of HUVECs were seeded in 96-well plates. Endothelial cell medium was used to culture peritoneal macrophages from p38α^fl/fl^ and p38α^fl/fl^LysMCre^+/-^ mice. Then, the culture supernatant was collected as conditioned medium to culture HUVECs. Endothelial cell medium without bovine serum was used as a control. Cell proliferation was measured using a BrdU assay kit (11674229001, Roche, Basel, Switzerland), according to the manufacturer's protocol.

### 4.9 Western blotting

Cell lysates were subjected to electrophoresis on 12% SDS-polyacrylamide gels and then transferred onto nitrocellulose membranes (Pall Corporation, NY, USA), according to standard procedures. Then, the membranes were blocked for 1 h with 5% non-fat milk. Membranes were incubated with each primary antibody (1:1000–2000 dilutions) overnight at 4 °C, followed by the appropriate secondary antibody (1:1000 dilution). Proteins were detected using a Super Signal West Pico Kit (Thermo Scientific, MA, USA) based on the manufacturer's instructions.

**Table:** 

Target	Cat.	Company
p38α	2371 S	Cell Signaling Technology, MA, USA
CD68	ab955	Abcam, Cambridge, UK
GAPDH	KC-5G4	KangChen, Beijing, China
MRTFA	sc-398675	Santa Cruz Biotechnology, TX, USA
mouse IL-6	Monoclonal Rat antibody, MAB406	R&D system, MN, USA
Rat IgG	MAB005	R&D system, MN, USA
H3	9725	Cell Signaling Technology, MA, USA
His-tag	AH367	Beyotime, Shanghai, China
Flag-tag	AF519	Beyotime, Shanghai, China
Alexa Fluor 555 goat anti-rabbit antibody	ab150078	Thermo Scientific, MA, USA
Alexa Fluor 488 goat anti-mouse antibody	ab150113	Thermo Scientific, MA, USA
anti-IgG, H + L chains, mouse	330	MBL, Nagoya, Japan
anti-IgG, H + L chains, rabbit	458	MBL, Nagoya, Japan

### 4.10 Interleukin-6 ELISA

Culture supernatants collected from peritoneal macrophages and lipopolysaccharide (100 ng/mL)-stimulated peritoneal macrophages were analyzed for interleukin (IL)-6 by a Mouse ELISA Kit (EK0411, Boster Biological Technology, CA, USA), following the manufacturer's instructions.

### 4.11 Isolation of mouse peritoneal macrophages

Peritoneal macrophages from adult male p38α^fl/fl^ mice and p38α^fl/fl^LysMCre^+/-^ mice were harvested at 3 days after i.p. injection of 4% thioglycollate medium (211716, Becton, Dickinson and Company, NJ, USA) into mice by peritoneal lavage. Macrophages were grown in DMEM containing 25 mM glucose and 10% FBS.

### 4.12 Quantitative PCR (qPCR)

RNA was isolated using TRIzol reagent (Invitrogen, MA, USA), according to the manufacturer's instructions. mRNA levels were quantified by quantitative PCR with SYBR Green (Invitrogen, MA, USA). Samples were normalized against 18 S mRNA levels.

The primer sequences of qPCR:

**Table:** 

TNF-α	Forward: CCAGACCCTCACACTCAGATC Reverse: CACTTGGTGGTTTGCTACGAC
IL-6	Forward: ACAACCACGGCCTTCCCTACTT Reverse: CACGATTTCCCAGAGAACATGTG
VEGF	Forward: AGACGGACACACATGGAGGT Reverse: AAAGACTCAATGCATGCCAC
β-actin	Forward: ATCTGGCACCACACCTTC Reverse: AGCCAGGTCCAGACGCA
MIF	Forward: GAGGGGTTTCTGTCGGAGC Reverse: GTTCGTGCCGCTAAAAGTCA
PDGF	Forward: AAGTGTGAGACAATAGTGACCCC Reverse: CATGGGTGTGCTTAAACTTTCG
TGF-β	Forward: CTTCAATACGTCAGACATTCGGG Reverse: GTAACGCCAGGAATTGTTGCTA
HIF1α	Forward: ATAGCTTCGCAGAATGCTCAGA Reverse: CAGTCACCTGGTTGCTGCAA
p38α	Forward: TGACCCTTATGACCAGTCCTTT Reverse: GTCAGGCTCTTCCACTCATCTAT
18s	Forward: AGGGGAGAGCGGGTAAGAGA Reverse: GGACAGGACTAGGCGGAACA

### 4.13 LC‐MS/MS analysis

Peptide mixtures were suspended in 10 μl of 1% formic acid and separated by online reversed‐phase nanoscale capillary liquid chromatography (Easy‐nLC 1000, Thermo Scientific). A 5 μl sample was autosampled directly onto a 100 μm × 10 cm in‐house-prepared fused silica emitter column packed with reverse‐phase ReproSil‐Pur C18‐AQ resin (3 μm, 120 Å, Dr. Maisch GmbH, Germany). The peptide mixtures were separated by a 200-min linear gradient from 5% to 32% acetonitrile. Data‐dependent mass spectra was acquired by a LTQ‐Orbitrap Elite mass spectrometer (Thermo Scientific) equipped with a nanoelectrospray ion source (Thermo Scientific). Full scan MS spectra (*m*/*z* 300–1600) were acquired in the Orbitrap analyzer with a resolution of 240,000 at 400 *m*/*z* after accumulation to a target value of 1,000,000. Then, up to 20 of the most intense ions per scan with charge states ≥2 were selected for collision induced dissociation fragmentation in the linear ion trap with normalized collision energy of 35% after accumulation to a target value of 10,000. Peptides with detected neutral loss of phosphoric acid (147.00, 98.00, 65.33, 49.00, and 32.66 *m*/*z*) were selected for additional multistage activation. The MS^3^ spectrum of neutral loss peptide was combined with the MS^2^ spectrum to construct a pseudo‐MS^3^ spectrum and searched with the same parameters as the MS^2^ spectrum.

### 4.14 Immunohistochemistry and immunofluorescence

Immunohistochemistry was performed on frozen sections of carotid arteries. The frozen sections were incubated with 3% hydrogen peroxide for 12 min, washed with PBS, and blocked in PBS with 10% normal goat serum for 60 min at 37 °C. The sections were incubated with primary antibodies at 4 °C overnight, followed by the respective secondary antibody before staining with a DAB Kit (PV9001, ZSGB-BIO, Beijing, China). The sections were then sealed with gum.

Immunofluorescence staining was performed on frozen sections. The frozen sections were incubated with 3% hydrogen peroxide for 12 min, washed with PBS, and blocked in PBS with 10% normal goat serum for 60 min at 37 °C. The sections were incubated with primary antibodies at 4 °C overnight, followed by a fluorescent secondary antibody. The sections were sealed with glycerinum containing DAPI (ZLI-557, ZSGB-BIO).

### 4.15 Recombinant lentivirus construction

A mouse-derived lentivirus constructed by LiKeLi Company Beijing. rLV-PGK-Puro was used as a negative control. rLV-MKL1-PGK-Puro was used as a positive control. After RAW267.4 cells reached 50%–70% confluence in 6-well plates, rLV-PGK-Puro, rLV-Mmkl1-PGK-Puro, rLV-MKL1-S544A + S549A-Puro, or rLV-Mmkl1-T545A + S549A-Puro was added to the cells for 16–24 h, and then infected cells were selected by puromycin (2.5 μg/ml) in DMEM with 10% FBS for 48–72 h. The cells were then used for further experiments. The synthesis sequence of the lentivirus is in the Supplemental Data File 4.

### 4.16 Immunoprecipitation

The 293T cells were seeded in 100-mm culture dishes before infection with pcDNA3.1-V5/hisB-p38α (His-p38α) and/or pcDNA3.1-C3Flag-MKL1 (Flag-MKL1) plasmids for 48 h. Whole cell lysates were collected for immunoprecipitation using an Immunoprecipitation Kit (Kit-1, Proteintech, USA), following the manufacturer's instructions.

### 4.17 Statistical analysis

Results are presented as means ± standard error of the mean (SEM) and derived from at least three independent experiments unless indicated otherwise. The unpaired Student's t-test was used to compare two groups followed by the demonstration of homogeneity of variance with an F test, and one-way ANOVA was used to calculate significant differences among three groups or more followed by Bonferroni multiple comparison post-test. Signiﬁcant differences between groups are represented by *0.01 < P < 0.05, **0.001 < P < 0.01, and ***P < 0.001; ns, no significance.

## Sources of funding

This work was supported by the 10.13039/501100001809National Natural Science Foundation of China (91639108, 81770272, 81970425).

## Author contributions

Meng Zhang and Jianing Gao contributed equally to this study. MZ and JG designed and performed experiments, analyzed data and wrote the manuscript. XZ performed LC‐MS/MS analysis and analyzed data. MZ assisted with experiments and analyzed data. DM, XZ, JW, XM and DT collected human data and analyzed data. XY and BP helped with the experiments design and edited the manuscript. LZ conceived the experiments, analyzed data and wrote the manuscript. All authors reviewed the manuscript.

## Declaration of competing interest

The authors declare that they have no conflict of interest.
