# Supplementary material for: p38α in macrophages aggravates arterial endothelium injury by releasing IL-6 through phosphorylating megakaryocytic leukemia 1
Source: Redox Biol. 2020 Nov 1;38:101775. doi: 10.1016/j.redox.2020.101775 (PMC7658717; doi:10.1016/j.redox.2020.101775)
Supplement: Multimedia component 1 [file mmc1.docx]

| Patients |  |  |  |  |
| --- | --- | --- | --- | --- |
|  | number | gender | age | case |
|  | 1 | M | 38 | carotid plaque, CI, DM, HP |
|  | 2 | M | 61 | carotid plaque, CI, HP |
|  | 3 | M | 67 | carotid plaque, CI, AF, |
|  | 4 | M | 63 | carotid plaque, CI, |
|  | 5 | M | 57 | carotid plaque, DM, CH |
|  | 6 | F | 62 | carotid plaque, CI, HP |
|  | 7 | F | 51 | carotid plaque, CI, HP |
| Control |  |  |  |  |
|  | 1 | M | 44 | Healthy volunteer (no carotid plaque) |
|  | 2 | M | 52 | Healthy volunteer (no carotid plaque) |
|  | 3 | M | 34 | Healthy volunteer (no carotid plaque) |
|  | 4 | M | 32 | Healthy volunteer (no carotid plaque) |
|  | 5 | F | 32 | Healthy volunteer (no carotid plaque) |
|  | 6 | F | 32 | Healthy volunteer (no carotid plaque) |

**Supplemental table 1**

Characteristics of the study population of p38α expression levels in monocytes. Atherosclerotic plaque was diagnosed by ultra-sound. DM: diabetes mellitus; CI, cerebral infarction; HP, hypertension; AF, atrial fibrillation; CH, cerebral hemorrhage.

**
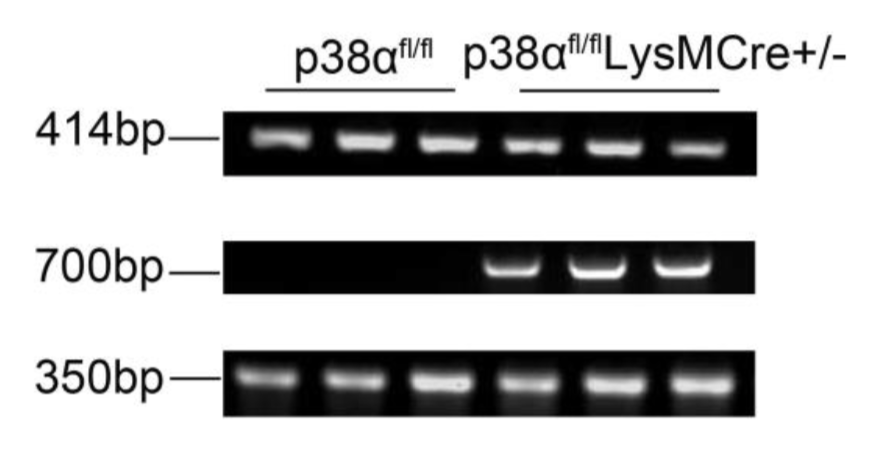
**

**Supplemental Figure 1**

The genotypic result of p38α^fl/fl^ and p38α^fl/fl^LysMCre^+/-^ mice. The p38α floxed allele (p38α^fl/fl^) were crossed with LysMCre mice expressing Cre^+/-^. The genotype of F0 was p38α^fl/+^ Cre^+/-^ and p38α^fl/+^ Cre^-/-^. p38α^fl/+^ Cre^+/-^ were chosen to cross with p38α^fl/fl^. The genotype of F1 were p38α^fl/+^ Cre^+/-^, p38α^fl/+^ Cre^-/-^, p38α^fl/fl^ Cre^+/-^ and p38α^fl/fl^ Cre^-/-^. The p38α^fl/fl^ Cre^+/-^ were crossed with p38α^fl/fl^ and the littermate groups were what we used.


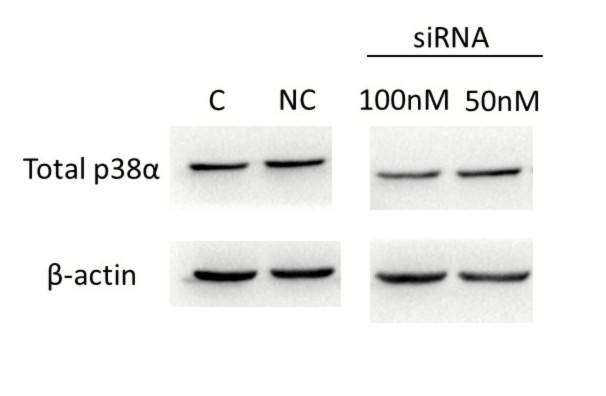


**Supplemental Figure 2**

Western blotting analysis of p38α in THP-1 cells after siRNA transfection.

C: THP-1 cells without treatment; NC: THP-1 cells transfected with negative control siRNA.


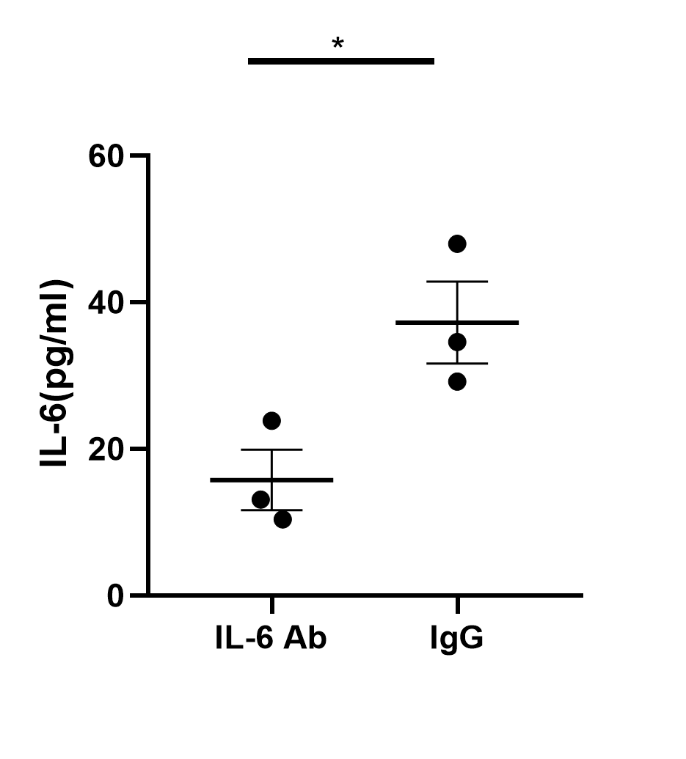


**Supplemental Figure 3**

Quantification of plasma IL-6 at 24 h after injection of IL-6 neutralizing antibody or IgG. n=3 each group, * P<0.05.


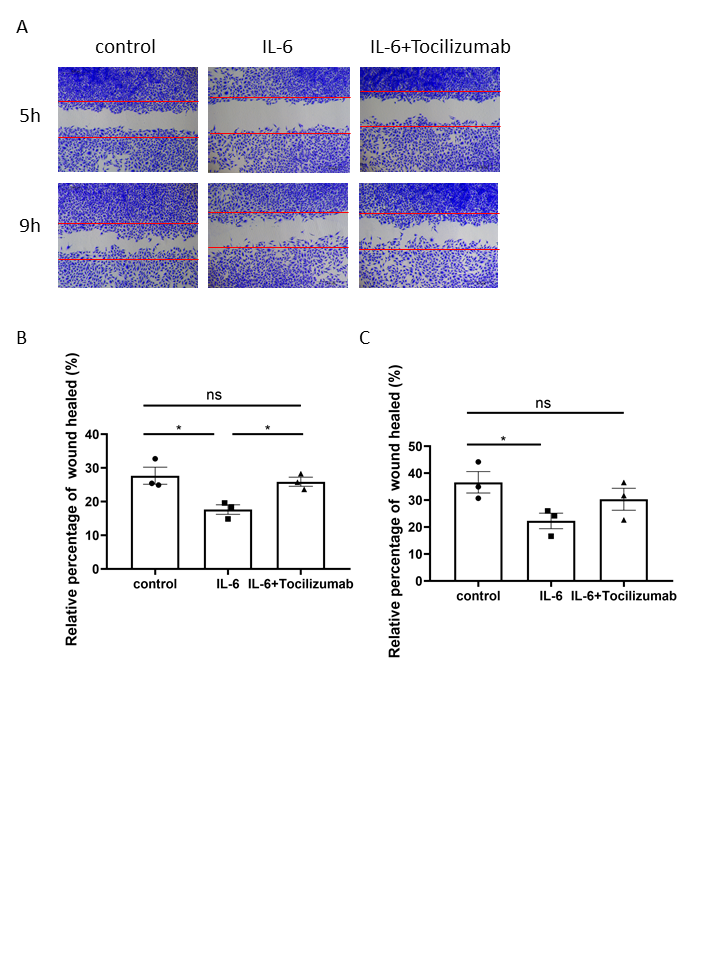


**Supplemental Figure 4**

(A) HUVEC were seeded in 2 well culture- insert and treated with ECM, ECM containing 10ng/ml IL-6 or ECM containing 10ng/ml IL-6 and 1ug/ml Tocilizumab. Migration into the wound was imaged. The red line indicates the scratch edge.

(B) Relative percentage of wound healed was calculated and images were obtained in 5 random fields. Results are means ± SEM from three independent experiments performed in triplicate (n=3 each group, *P<0.05, ns: P≥0.05).

**Supplemental Figure 5**

Sequence confirmation of phosphorylation level of MKL1 in 293T cells which were transfected by pcDNA3.1-V5/hisB-p38α and pcDNA3.1-C3Flag-MKL1.


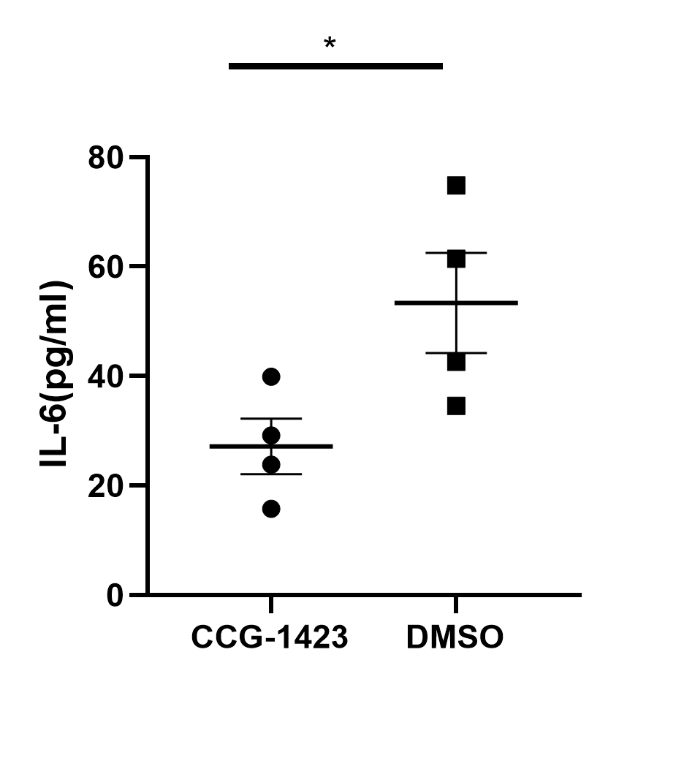


**Supplemental Figure 6**

After injection of CCG-1423 as inhibitor of MKL1 or DMSO as control for 14 days, IL-6 in plasma from carotid artery injured mice 24h. n=4 each group, * P<0.05.
