# Supplementary material for: p38α in macrophages aggravates arterial endothelium injury by releasing IL-6 through phosphorylating megakaryocytic leukemia 1
Source: Redox Biol. 2020 Nov 1;38:101775. doi: 10.1016/j.redox.2020.101775 (PMC7658717; doi:10.1016/j.redox.2020.101775)
Supplement: Multimedia component 2 [file mmc2.pdf]

Supplemental Table 2

MKL1	MPPLKSPAAFHEQRRSLERARTEDYLKRKIRSRPERSELVRMHILEETSAEPSLQAKQLKLKRARLADDLNEKIA<br>QRPGPMELVEKNILPVESSLKEAIIVGQVNYPKVADSSSFDEDSSDALSPEQPASHESQGSVPSPLEARVSEPLL<br>SATSASPTQVVSQLPMGRDSREMLFLAEQPPLPPPPLLPPSLTNGTTIPTAKSTPTLIKQSQPKSASEKSQRSKK<br>AKELKPKVKKLKYHQYIPPDQKQDRGAPPMDSSYAKILQQQLFLQLQILNQQQQQHHNYQAILPAPPKSAGEAL<br>GSSGTPPVRSLSTTNSSSSSGAPGPCGLARQNSTSLTGKPGALPANLDDMKVAELKQELKLRSLPVSGTKTELIE<br>RLRAYQDQISPVPGAPKAPAATSILHKAGEVVVAFPAARLSTGPALVAAGLAPAEVVVATVASSGVVKFGSTGST<br>PPVSPTPSERSLLSTGDENSTPGDTFGEMVTSPLTQLTLQASPLQILVKEEGPRAGSCCLSPGGRAELEGRDKDQ<br>MLQEKDKQIEALTRMLRQKQQLVERLKLQLEQEKRAQQPAPAPAPLGTPVKQENSFSSCQLSQQPLGPAHPFNPS<br>LAAPATNHIDPCAVAPGPPSVVVKQEALQPEPEPVPAPQLLLGPQGPSLIKGVAPPTLITDSTGTHLVLTVTNKN<br>ADSPGLSSGSPQQPSSQPGSPAPAPSAQMDLEHPLQPLFGTPTSLLKKEPPGYEEAMSQQPKQQENGSSSQQMDD<br>LFDILIQSGEISADFKEPPSLPGKEKPSPKTVCGSPLAAQPSPSAELPQAAPPPPGSPSLPGRLEDFLESSTGLP<br>LLTSGHDGPEPLSLIDDLHSQMLSSTAILDHPPSPMDTSELHFVPEPSSTMGLDLADGHLDSMDWLELSSGGPVL<br>SLAPLSTTAPSLFSTDFLDGHDLQLHWDSCL
MKL1-S449A/S454A	MPPLKSPAAFHEQRRSLERARTEDYLKRKIRSRPERSELVRMHILEETSAEPSLQAKQLKLKRARLADDLNEKIA<br>QRPGPMELVEKNILPVESSLKEAIIVGQVNYPKVADSSSFDEDSSDALSPEQPASHESQGSVPSPLEARVSEPLL<br>SATSASPTQVVSQLPMGRDSREMLFLAEQPPLPPPPLLPPSLTNGTTIPTAKSTPTLIKQSQPKSASEKSQRSKK<br>AKELKPKVKKLKYHQYIPPDQKQDRGAPPMDSSYAKILQQQLFLQLQILNQQQQQHHNYQAILPAPPKSAGEAL<br>GSSGTPPVRSLSTTNSSSSSGAPGPCGLARQNSTSLTGKPGALPANLDDMKVAELKQELKLRSLPVSGTKTELIE<br>RLRAYQDQISPVPGAPKAPAATSILHKAGEVVVAFPAARLSTGPALVAAGLAPAEVVVATVASSGVVKFGSTGAT<br>PPVAPTPSERSLLSTGDENSTPGDTFGEMVTSPLTQLTLQASPLQILVKEEGPRAGSCCLSPGGRAELEGRDKDQ<br>MLQEKDKQIEALTRMLRQKQQLVERLKLQLEQEKRAQQPAPAPAPLGTPVKQENSFSSCQLSQQPLGPAHPFNPS<br>LAAPATNHIDPCAVAPGPPSVVVKQEALQPEPEPVPAPQLLLGPQGPSLIKGVAPPTLITDSTGTHLVLTVTNKN<br>ADSPGLSSGSPQQPSSQPGSPAPAPSAQMDLEHPLQPLFGTPTSLLKKEPPGYEEAMSQQPKQQENGSSSQQMDD<br>LFDILIQSGEISADFKEPPSLPGKEKPSPKTVCGSPLAAQPSPSAELPQAAPPPPGSPSLPGRLEDFLESSTGLP<br>LLTSGHDGPEPLSLIDDLHSQMLSSTAILDHPPSPMDTSELHFVPEPSSTMGLDLADGHLDSMDWLELSSGGPVL<br>SLAPLSTTAPSLFSTDFLDGHDLQLHWDSCL
MKL1-T450A/S454A	MPPLKSPAAFHEQRRSLERARTEDYLKRKIRSRPERSELVRMHILEETSAEPSLQAKQLKLKRARLADDLNEKIA<br>QRPGPMELVEKNILPVESSLKEAIIVGQVNYPKVADSSSFDEDSSDALSPEQPASHESQGSVPSPLEARVSEPLL<br>SATSASPTQVVSQLPMGRDSREMLFLAEQPPLPPPPLLPPSLTNGTTIPTAKSTPTLIKQSQPKSASEKSQRSKK<br>AKELKPKVKKLKYHQYIPPDQKQDRGAPPMDSSYAKILQQQLFLQLQILNQQQQQHHNYQAILPAPPKSAGEAL<br>GSSGTPPVRSLSTTNSSSSSGAPGPCGLARQNSTSLTGKPGALPANLDDMKVAELKQELKLRSLPVSGTKTELIE<br>RLRAYQDQISPVPGAPKAPAATSILHKAGEVVVAFPAARLSTGPALVAAGLAPAEVVVATVASSGVVKFGSTGSA<br>PPVAPTPSERSLLSTGDENSTPGDTFGEMVTSPLTQLTLQASPLQILVKEEGPRAGSCCLSPGGRAELEGRDKDQ<br>MLQEKDKQIEALTRMLRQKQQLVERLKLQLEQEKRAQQPAPAPAPLGTPVKQENSFSSCQLSQQPLGPAHPFNPS<br>LAAPATNHIDPCAVAPGPPSVVVKQEALQPEPEPVPAPQLLLGPQGPSLIKGVAPPTLITDSTGTHLVLTVTNKN<br>ADSPGLSSGSPQQPSSQPGSPAPAPSAQMDLEHPLQPLFGTPTSLLKKEPPGYEEAMSQQPKQQENGSSSQQMDD<br>LFDILIQSGEISADFKEPPSLPGKEKPSPKTVCGSPLAAQPSPSAELPQAAPPPPGSPSLPGRLEDFLESSTGLP<br>LLTSGHDGPEPLSLIDDLHSQMLSSTAILDHPPSPMDTSELHFVPEPSSTMGLDLADGHLDSMDWLELSSGGPVL<br>SLAPLSTTAPSLFSTDFLDGHDLQLHWDSCL
