# Supplementary material for: p38α in macrophages aggravates arterial endothelium injury by releasing IL-6 through phosphorylating megakaryocytic leukemia 1
Source: Redox Biol. 2020 Nov 1;38:101775. doi: 10.1016/j.redox.2020.101775 (PMC7658717; doi:10.1016/j.redox.2020.101775)
Supplement: Multimedia component 3 [file mmc3.pdf]

Supplemental Table 3

**Gene Name**	mouse Mrtfa（NM_153049.3）
**Cloning Vector**	pLVX-PGK-Puro
**Cloning Strategy**	XhoI+NotI
**Vector map**	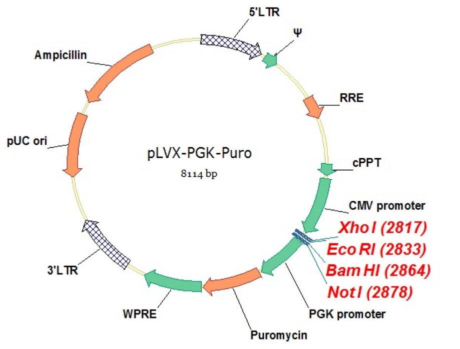
Mrtfa synthesis sequence	2915bp XhoI---NotI in pUC57 Vector
	CTCGAGGCCACCATGACTCTGCTGGAGCCTGAGATGCTAATGATGGCTGTGCAGTCAGTGCTGCAGTTGAAGCTCCAGCAGCGGCGGACCCGGGAGGAACTGGTGAGCCAAGGGATCA<br>TGCCGCCTTTGAAAAGCCCCGCTGCATTTCATGAGCAGAGAAGAAGCCTGGAGCGGGCCAGGACCGAGGACTATTTGAAACGGAAGATCCGTTCCCGGCCCGAGAGAGCAGAGCTGG<br>TCAGGATGCACATTCTGGAAGAGACCTCGGCTGAGCCTTCGCTCCAGGCCAAGCAGCTGAAGCTGAAGAGAGCCAGGCTGGCTGATGACCTCAATGAAAAGATTGCACAGAGGCCTG<br>GCCCCATGGAGCTGGTGGAGAAGAATATCCTGCCTGTGGAGTCCAGCCTGAAGGAGGCTATCATTGTGGGCCAGGTAAATTACCCAAAGGTAGCAGACAGTTCCTCCTTCGACGAGGA<br>CAGCAGCGATGCCCTGTCTCCTGAGCAGCCTGCCAGCCATGAGTCCCAGGGTTCAGTGCCATCACCCTTGGAGTCCCGAGTCAGTGATCCACTGCCCAGTGCCACCTCCATATCACCCA<br>CTCAGGTTCTTTCTCAACTCCCAATGGCTCCGGATCCTGGAGAGACGCTTTTTCTGGCAGAGCAGCCTCCTCTGCCTCCCGCACCTCTGCTGCCCCCAAGCCTAGCCAATGGAAGCATCG<br>TCCCCACTGCCAAGCCTGCTCCCACACTCATCAAGCAAAGCCAACCCAAGTCTGCCAGCGAGAAATCACAGCGCAGCAAGAAGGCCAAGGAGCTGAAGCCAAAGGTGAAGAAGCTCA<br>AGTACCACCAGTACATCCCCCCGGACCAGAAGCAGGACAAGGGGGCGCCCGCCATGGACTCCTCCTATGCCAAGATCCTGCAGCAGCAGCAGCTCTTCCTGCAGCTGCAGATCCTCAA<br>CCAGCAGCAGCAGCAGCAGCAGCAACAGCACTACAACTACCAGGCCATCCTGCCTGCCCCTCCCAAGCCCTCGGCTGAGACTCCTGGAAGCAGTGCCCCTACCCCATCACGCAGCCTC<br>TCCACCAGTAGCAGCCCCAGCTCAGGCACCCCAGGGCCCAGCGGGCTGGCACGCCAGAGCAGCACCGCACTAGCTGCCAAACCAGGAGCCCTGCCAGCCAACCTGGATGACATGAAG<br>GTGGCAGAGCTGAAGCAAGAACTGAAGTTGCGGTCCCTTCCCGTCTCAGGCACCAAGACAGAGCTGATAGAACGCCTGCGTGCCTACCAAGACCAAGTCAGCCCAGCTCCAGGAGCCC<br>CCAAGGCCCCTGCCACCACCTCTGTGCTGTCCAAGGCTGGTGAGGTAGTGGTCGCCTTCCCTGCGGCCCTGCTAAGCACAGGGTCAGCTCTTGTAACAGCAGGCCTTGCACCAGCTGAG<br>ATGGTGGTGGCCACAGTAACCAGCAATGGCATGGTGAAGTTTGGCAGCACAGGCTCCACACCCCCCGTGTCTCCCACCCCTTCAGAGCGCTCACTGCTCAGCACGGGTGATGAGAATT<br>CCACACCTGGGGATGCCTTTGGTGAAATGGTGACATCGCCGCTGACACAGCTCACCCTGCAGGCCTCCCCACTGCAGATCGTGAAGGAGGAGGGTGCCCGTGCTGCGTCCTGCTGTCTA<br>AGCCCTGGTGCTCGGGCTGAGCTGGAGGGACTGGACAAGGACCAGATGCTGCAGGAGAAGGACAAGCAGATTGAGGAGCTGACCCGAATGCTCCAACAGAAGCAGCAGCTGGTTGAG<br>CTGCTGCGGCTACAGCTGGAGCAGCAGAAGCGGGCCCAGCAGCCAGCCCCAGCCAGCAGCCCTGTGAAGAGGGAAAGTGGTTTCTCCAGTTGCCAGCTGAGCTGCCAGCCCCAGGGCT<br>CTGCCCATGCTTTTGGCTCTGGCCTAGTGGTTCCCACTACCAACCATGGAGACACTCAGGCCCCAGCGCCAGAGTCCCCACCTGTGGTGGTGAAGCAGGAAGCTGGGCCACCTGAGCC<br>AGATCTGGCCCCCAGCTCCCAGCTGCTCTTGGGCTCCCAGGGCACCAGCTTCCTCAAGAGGGTCAGCCCTCCTACCCTGGTCACTGACTCTACAGGGACTCACCTCATCCTCACTGTGA<br>CCAATAAGAGTGCTGATGGCCCTGGCTTGCCTGCAGGGAGCCCCCAGCAGCCCTTGTCCCAGCCTGGTTCTCCAGCCCCTGGTCCACCTGCCCAGATGGACCTGGAGCACCCACCTCAG<br>CCTCCGTTTGCAACCCCCACATCTCTGCTGAAGAAGGAGCCCCCTGGTTATGAAGAGACTGTGACCCAGCAGCCTAAGCAGCAGGAAAATGGCTCCTCCAGTCAGCACATGGATGATC<br>TGTTTGATATTCTTATTCAGAGTGGAGAGATTTCAGCAGATTTCAAAGAGCCACCATCCCTACCAGGCAAGGAAAAGTCACCTCCAGCAGCAGCAGCGTATGGGCCTCCATTGACACC<br>ACAACCCTCGCCTTTGAGTGAACTCCCCCAAGCTGCTCCTCCACCAGGTTCCCCCACCCTCCCAGGGCGCCTTGAAGACTTCCTGGAGAGCAGCACAGGGCTGCCCCTGCTGACAAGTG<br>GGCACGAGGGACCAGAACCCCTTTCCCTCATTGATGACCTCCACAGCCAGATGCTGAGCAGCTCCGCCATCCTGGACCACCCCCCATCACCCATGGACACCTCTGAATTGCACTTTGCT<br>CCTGAGCCCAGCAGTGGTATGGGCCTGGACCTGGCTGTTGGCCACCTGGACAGCATGGACTGGCTGGAGCTGTCGTCTGGTGGCCCTGTGCTCAGCCTGGCTCCCCTCAGCACTGCAGC<br>CCCCAGCCTCTTCTCGATGGACTTCCTGGATGGACACGACTTGCAGCTCCACTGGGATTCCTGCTTGTAGGCGGCCGC
Mrtfa-S492+S487 synthesis sequence	2915bp XhoI---NotI in pUC57 Vector
	CTCGAGGCCACCATGACTCTGCTGGAGCCTGAGATGCTAATGATGGCTGTGCAGTCAGTGCTGCAGTTGAAGCTCCAGCAGCGGCGGACCCGGGAGGAACTGGTGAGCCAAGGGATCA<br>TGCCGCCTTTGAAAAGCCCCGCTGCATTTCATGAGCAGAGAAGAAGCCTGGAGCGGGCCAGGACCGAGGACTATTTGAAACGGAAGATCCGTTCCCGGCCCGAGAGAGCAGAGCTGG<br>TCAGGATGCACATTCTGGAAGAGACCTCGGCTGAGCCTTCGCTCCAGGCCAAGCAGCTGAAGCTGAAGAGAGCCAGGCTGGCTGATGACCTCAATGAAAAGATTGCACAGAGGCCTG<br>GCCCCATGGAGCTGGTGGAGAAGAATATCCTGCCTGTGGAGTCCAGCCTGAAGGAGGCTATCATTGTGGGCCAGGTAAATTACCCAAAGGTAGCAGACAGTTCCTCCTTCGACGAGGA<br>CAGCAGCGATGCCCTGTCTCCTGAGCAGCCTGCCAGCCATGAGTCCCAGGGTTCAGTGCCATCACCCTTGGAGTCCCGAGTCAGTGATCCACTGCCCAGTGCCACCTCCATATCACCCA<br>CTCAGGTTCTTTCTCAACTCCCAATGGCTCCGGATCCTGGAGAGACGCTTTTTCTGGCAGAGCAGCCTCCTCTGCCTCCCGCACCTCTGCTGCCCCCAAGCCTAGCCAATGGAAGCATCG<br>TCCCCACTGCCAAGCCTGCTCCCACACTCATCAAGCAAAGCCAACCCAAGTCTGCCAGCGAGAAATCACAGCGCAGCAAGAAGGCCAAGGAGCTGAAGCCAAAGGTGAAGAAGCTCA<br>AGTACCACCAGTACATCCCCCCGGACCAGAAGCAGGACAAGGGGGCGCCCGCCATGGACTCCTCCTATGCCAAGATCCTGCAGCAGCAGCAGCTCTTCCTGCAGCTGCAGATCCTCAA<br>CCAGCAGCAGCAGCAGCAGCAGCAACAGCACTACAACTACCAGGCCATCCTGCCTGCCCCTCCCAAGCCCTCGGCTGAGACTCCTGGAAGCAGTGCCCCTACCCCATCACGCAGCCTC<br>TCCACCAGTAGCAGCCCCAGCTCAGGCACCCCAGGGCCCAGCGGGCTGGCACGCCAGAGCAGCACCGCACTAGCTGCCAAACCAGGAGCCCTGCCAGCCAACCTGGATGACATGAAG<br>GTGGCAGAGCTGAAGCAAGAACTGAAGTTGCGGTCCCTTCCCGTCTCAGGCACCAAGACAGAGCTGATAGAACGCCTGCGTGCCTACCAAGACCAAGTCAGCCCAGCTCCAGGAGCCC<br>CCAAGGCCCCTGCCACCACCTCTGTGCTGTCCAAGGCTGGTGAGGTAGTGGTCGCCTTCCCTGCGGCCCTGCTAAGCACAGGGTCAGCTCTTGTAACAGCAGGCCTTGCACCAGCTGAG<br>ATGGTGGTGGCCACAGTAACCAGCAATGGCATGGTGAAGTTTGGCAGCACAGGCGCCACACCCCCCGTGGCTCCCACCCCTTCAGAGCGCTCACTGCTCAGCACGGGTGATGAGAATT<br>CCACACCTGGGGATGCCTTTGGTGAAATGGTGACATCGCCGCTGACACAGCTCACCCTGCAGGCCTCCCCACTGCAGATCGTGAAGGAGGAGGGTGCCCGTGCTGCGTCCTGCTGTCTA<br>AGCCCTGGTGCTCGGGCTGAGCTGGAGGGACTGGACAAGGACCAGATGCTGCAGGAGAAGGACAAGCAGATTGAGGAGCTGACCCGAATGCTCCAACAGAAGCAGCAGCTGGTTGAG<br>CTGCTGCGGCTACAGCTGGAGCAGCAGAAGCGGGCCCAGCAGCCAGCCCCAGCCAGCAGCCCTGTGAAGAGGGAAAGTGGTTTCTCCAGTTGCCAGCTGAGCTGCCAGCCCCAGGGCT<br>CTGCCCATGCTTTTGGCTCTGGCCTAGTGGTTCCCACTACCAACCATGGAGACACTCAGGCCCCAGCGCCAGAGTCCCCACCTGTGGTGGTGAAGCAGGAAGCTGGGCCACCTGAGCC<br>AGATCTGGCCCCCAGCTCCCAGCTGCTCTTGGGCTCCCAGGGCACCAGCTTCCTCAAGAGGGTCAGCCCTCCTACCCTGGTCACTGACTCTACAGGGACTCACCTCATCCTCACTGTGA<br>CCAATAAGAGTGCTGATGGCCCTGGCTTGCCTGCAGGGAGCCCCCAGCAGCCCTTGTCCCAGCCTGGTTCTCCAGCCCCTGGTCCACCTGCCCAGATGGACCTGGAGCACCCACCTCAG<br>CCTCCGTTTGCAACCCCCACATCTCTGCTGAAGAAGGAGCCCCCTGGTTATGAAGAGACTGTGACCCAGCAGCCTAAGCAGCAGGAAAATGGCTCCTCCAGTCAGCACATGGATGATC<br>TGTTTGATATTCTTATTCAGAGTGGAGAGATTTCAGCAGATTTCAAAGAGCCACCATCCCTACCAGGCAAGGAAAAGTCACCTCCAGCAGCAGCAGCGTATGGGCCTCCATTGACACC<br>ACAACCCTCGCCTTTGAGTGAACTCCCCCAAGCTGCTCCTCCACCAGGTTCCCCCACCCTCCCAGGGCGCCTTGAAGACTTCCTGGAGAGCAGCACAGGGCTGCCCCTGCTGACAAGTG<br>GGCACGAGGGACCAGAACCCCTTTCCCTCATTGATGACCTCCACAGCCAGATGCTGAGCAGCTCCGCCATCCTGGACCACCCCCCATCACCCATGGACACCTCTGAATTGCACTTTGCT<br>CCTGAGCCCAGCAGTGGTATGGGCCTGGACCTGGCTGTTGGCCACCTGGACAGCATGGACTGGCTGGAGCTGTCGTCTGGTGGCCCTGTGCTCAGCCTGGCTCCCCTCAGCACTGCAGC<br>CCCCAGCCTCTTCTCGATGGACTTCCTGGATGGACACGACTTGCAGCTCCACTGGGATTCCTGCTTGTAGGCGGCCGC
Mrtfa-S492+T488 synthesis sequence	2915bp XhoI---NotI in pUC57 Vector
	CTCGAGGCCACCATGACTCTGCTGGAGCCTGAGATGCTAATGATGGCTGTGCAGTCAGTGCTGCAGTTGAAGCTCCAGCAGCGGCGGACCCGGGAGGAACTGGTGAGCCAAGGGATCA<br>TGCCGCCTTTGAAAAGCCCCGCTGCATTTCATGAGCAGAGAAGAAGCCTGGAGCGGGCCAGGACCGAGGACTATTTGAAACGGAAGATCCGTTCCCGGCCCGAGAGAGCAGAGCTGG<br>TCAGGATGCACATTCTGGAAGAGACCTCGGCTGAGCCTTCGCTCCAGGCCAAGCAGCTGAAGCTGAAGAGAGCCAGGCTGGCTGATGACCTCAATGAAAAGATTGCACAGAGGCCTG<br>GCCCCATGGAGCTGGTGGAGAAGAATATCCTGCCTGTGGAGTCCAGCCTGAAGGAGGCTATCATTGTGGGCCAGGTAAATTACCCAAAGGTAGCAGACAGTTCCTCCTTCGACGAGGA<br>CAGCAGCGATGCCCTGTCTCCTGAGCAGCCTGCCAGCCATGAGTCCCAGGGTTCAGTGCCATCACCCTTGGAGTCCCGAGTCAGTGATCCACTGCCCAGTGCCACCTCCATATCACCCA<br>CTCAGGTTCTTTCTCAACTCCCAATGGCTCCGGATCCTGGAGAGACGCTTTTTCTGGCAGAGCAGCCTCCTCTGCCTCCCGCACCTCTGCTGCCCCCAAGCCTAGCCAATGGAAGCATCG<br>TCCCCACTGCCAAGCCTGCTCCCACACTCATCAAGCAAAGCCAACCCAAGTCTGCCAGCGAGAAATCACAGCGCAGCAAGAAGGCCAAGGAGCTGAAGCCAAAGGTGAAGAAGCTCA<br>AGTACCACCAGTACATCCCCCCGGACCAGAAGCAGGACAAGGGGGCGCCCGCCATGGACTCCTCCTATGCCAAGATCCTGCAGCAGCAGCAGCTCTTCCTGCAGCTGCAGATCCTCAA<br>CCAGCAGCAGCAGCAGCAGCAGCAACAGCACTACAACTACCAGGCCATCCTGCCTGCCCCTCCCAAGCCCTCGGCTGAGACTCCTGGAAGCAGTGCCCCTACCCCATCACGCAGCCTC<br>TCCACCAGTAGCAGCCCCAGCTCAGGCACCCCAGGGCCCAGCGGGCTGGCACGCCAGAGCAGCACCGCACTAGCTGCCAAACCAGGAGCCCTGCCAGCCAACCTGGATGACATGAAG<br>GTGGCAGAGCTGAAGCAAGAACTGAAGTTGCGGTCCCTTCCCGTCTCAGGCACCAAGACAGAGCTGATAGAACGCCTGCGTGCCTACCAAGACCAAGTCAGCCCAGCTCCAGGAGCCC<br>CCAAGGCCCCTGCCACCACCTCTGTGCTGTCCAAGGCTGGTGAGGTAGTGGTCGCCTTCCCTGCGGCCCTGCTAAGCACAGGGTCAGCTCTTGTAACAGCAGGCCTTGCACCAGCTGAG<br>ATGGTGGTGGCCACAGTAACCAGCAATGGCATGGTGAAGTTTGGCAGCACAGGCTCCGCACCCCCCGTGGCTCCCACCCCTTCAGAGCGCTCACTGCTCAGCACGGGTGATGAGAATT<br>CCACACCTGGGGATGCCTTTGGTGAAATGGTGACATCGCCGCTGACACAGCTCACCCTGCAGGCCTCCCCACTGCAGATCGTGAAGGAGGAGGGTGCCCGTGCTGCGTCCTGCTGTCTA<br>AGCCCTGGTGCTCGGGCTGAGCTGGAGGGACTGGACAAGGACCAGATGCTGCAGGAGAAGGACAAGCAGATTGAGGAGCTGACCCGAATGCTCCAACAGAAGCAGCAGCTGGTTGAG<br>CTGCTGCGGCTACAGCTGGAGCAGCAGAAGCGGGCCCAGCAGCCAGCCCCAGCCAGCAGCCCTGTGAAGAGGGAAAGTGGTTTCTCCAGTTGCCAGCTGAGCTGCCAGCCCCAGGGCT<br>CTGCCCATGCTTTTGGCTCTGGCCTAGTGGTTCCCACTACCAACCATGGAGACACTCAGGCCCCAGCGCCAGAGTCCCCACCTGTGGTGGTGAAGCAGGAAGCTGGGCCACCTGAGCC<br>AGATCTGGCCCCCAGCTCCCAGCTGCTCTTGGGCTCCCAGGGCACCAGCTTCCTCAAGAGGGTCAGCCCTCCTACCCTGGTCACTGACTCTACAGGGACTCACCTCATCCTCACTGTGA<br>CCAATAAGAGTGCTGATGGCCCTGGCTTGCCTGCAGGGAGCCCCCAGCAGCCCTTGTCCCAGCCTGGTTCTCCAGCCCCTGGTCCACCTGCCCAGATGGACCTGGAGCACCCACCTCAG<br>CCTCCGTTTGCAACCCCCACATCTCTGCTGAAGAAGGAGCCCCCTGGTTATGAAGAGACTGTGACCCAGCAGCCTAAGCAGCAGGAAAATGGCTCCTCCAGTCAGCACATGGATGATC<br>TGTTTGATATTCTTATTCAGAGTGGAGAGATTTCAGCAGATTTCAAAGAGCCACCATCCCTACCAGGCAAGGAAAAGTCACCTCCAGCAGCAGCAGCGTATGGGCCTCCATTGACACC<br>ACAACCCTCGCCTTTGAGTGAACTCCCCCAAGCTGCTCCTCCACCAGGTTCCCCCACCCTCCCAGGGCGCCTTGAAGACTTCCTGGAGAGCAGCACAGGGCTGCCCCTGCTGACAAGTG<br>GGCACGAGGGACCAGAACCCCTTTCCCTCATTGATGACCTCCACAGCCAGATGCTGAGCAGCTCCGCCATCCTGGACCACCCCCCATCACCCATGGACACCTCTGAATTGCACTTTGCT<br>CCTGAGCCCAGCAGTGGTATGGGCCTGGACCTGGCTGTTGGCCACCTGGACAGCATGGACTGGCTGGAGCTGTCGTCTGGTGGCCCTGTGCTCAGCCTGGCTCCCCTCAGCACTGCAGC<br>CCCCAGCCTCTTCTCGATGGACTTCCTGGATGGACACGACTTGCAGCTCCACTGGGATTCCTGCTTGTAGGCGGCCGC
