# Supplementary material for: p38α in macrophages aggravates arterial endothelium injury by releasing IL-6 through phosphorylating megakaryocytic leukemia 1
Source: Redox Biol. 2020 Nov 1;38:101775. doi: 10.1016/j.redox.2020.101775 (PMC7658717; doi:10.1016/j.redox.2020.101775)
Supplement: Multimedia component 4 [file mmc4.pdf]

Supplemental Table 4

supplemental Table 3-phosphorylation sites of p38αfl/flLysMCre+/- mice and p38αfl/fl mice.

	KO	KO	KO	WT	WT	WT	
Q9Y618	19152682.8	10072986	20853477.2	0	0	0	0.007551
P30101	13194543.9	5887394.8	8678906.88	0	0	0	0.012188
P05091	7494967.5	2001485	5937374.59	0	0	0	0.034604
Q5JSH3	10451534.3	32770179	57738897.4	0	0	0	0.069389
Q969V6	0	0	0	5676887.1	29351993.3	41134036.1	0.071587
Q13459	12139657.6	10264914	0	10415992	111819230	87908812.5	0.112286
Q86VQ1	0	3932977.9	4135924.03	0	0	0	0.116368
Q8WVR3	0	0	0	12747016	13829926.8	0	0.116776
O95425	0	0	0	0	56600615	61632601.5	0.116836
Q9UHW9	57749071.8	0	70108023.3	0	0	0	0.11982
Q8N5F7	0	0	0	0	43469998.5	35520271.5	0.12013
Q9Y4E6	0	0	0	0	82529689.5	66657299.7	0.120599
Q9Y5X3	0	3268101.6	0	2437889.9	30061680.9	45919031	0.120916
Q92934	0	2574860.7	3321218.98	0	0	0	0.122451
Q05655	8969133.17	3186859.6	5768111.08	3181567.1	187133203	196479020	0.122522
Q68E01	0	0	0	0	55180944	71811736	0.122893
Q9H2G2	0	0	0	0	48803058.3	63889562.3	0.123196
O00264	0	0	0	0	298426666	392477836	0.123435
O43395	0	0	0	12235058	0	16438610.8	0.124594
O95071	0	0	0	1727195.8	15708482	30251182	0.125769
Q92688	0	0	0	0	34053625.9	47648636.8	0.127011
P26368	0	0	0	0	92811737.3	132521161	0.12832
Q5T5C0	0	0	0	0	35023747.9	50787349.6	0.129363
P35606	0	0	0	0	22575137.9	33081767.2	0.130094
O75382	7244968.69	0	4919167.59	0	0	0	0.13045
Q3KQU3	17607189.8	11875013	0	0	0	0	0.13093
Q9BXI6	0	0	0	0	52448651.4	79831767.9	0.132875
O15144	0	0	0	206722.82	20124225	32257894.7	0.13386
Q9UKG1	0	0	0	0	59660404.1	93968866.5	0.135563
Q9C073	0	1860168.5	2957789.17	0	0	0	0.136337
Q12996	0	0	26065271.8	5083241	198402908	293190580	0.139471
O75676	0	0	0	0	46253527	76531785.5	0.139719
P42684	27459918.4	0	16453472.5	0	0	0	0.140478
Q02410	0	0	0	0	37396580	62615680.3	0.140767
Q8TBZ3	0	0	0	0	65119899.6	38475340	0.141734
P00558	53030773.8	4228736.8	34036488.6	26891624	225571326	326337693	0.142275
Q16827	0	4874365.3	0	0	30715470.5	32984606.1	0.142493
Q99759	0	21124864	36108435.9	0	0	0	0.142631
Q92769	24581072.3	16525793	116057141	8262659.5	1161089068	901920389	0.142943
Q9UPU7	13583852.4	0	23356017.3	0	0	0	0.143173
Q8TEV9	10292220.4	4115963	0	1697347	57000801.1	60532973	0.144223
Q9BWW4	0	1434281	2502892.69	0	0	0	0.144552
P84103	0	0	0	0	220385522	390940731	0.146107
P43243	0	0	0	0	39813549	70633007.5	0.146118
Q04721	0	0	0	0	41200742	73429251	0.146557
Q86X10	12863270.8	0	12134611.2	2195742	0	0	0.147162
O95819	0	8849610	0	6911605.6	81980331.1	37926358.4	0.14809
Q9C0C2	3589209.38	0	6520376.75	0	0	0	0.148419
Q7Z5L9	58273390.7	15921031	47936692.5	0	578210359	698876575	0.149357
Q9H4L5	29379885.4	0	0	5437636.8	97603962.5	114964290	0.150094
Q8TEH3	0	0	24683743.4	0	164353080	237858835	0.150102
P14859	0	5607580.3	0	0	38058794.6	55857886.1	0.150491
Q9Y4B4	5841813.38	3132560.6	0	0	0	0	0.151028
Q9ULV4	0	0	0	0	15428849.9	29500705.5	0.153577
Q5VZ89	0	0	0	0	30561761.5	58783360.9	0.154191
Q9H7N4	23089526.8	13436002	40317803.8	9093460.7	237199454	210451210	0.155273

P16401	0	0	0	0	16317504.6	32287416.1	0.157162
Q14103	0	0	0	0	38786782.1	77146533.1	0.157714
Q13185	0	0	0	0	15879916.8	32027332.9	0.159202
P05771	22635707.2	0	6120541.5	6035457.2	123131100	80190030.4	0.159278
Q9NQG5	77188010.3	7047627.7	0	26984744	152186306	181450592	0.159537
Q13523	19171048.7	10518494	38706765.2	12969935	363182706	219188520	0.160071
Q2LD37	10860020.5	3110861.9	0	2399680.1	35918099.8	49060411.9	0.161355
O60885	0	0	0	0	19903973.6	41455956.3	0.162698
Q16629	0	0	0	0	49445158.1	105767549	0.165647
Q9BTE3	0	0	13263625.3	0	53073237.3	74010961.5	0.166524
P11717	189674807	54976912	99665511.1	32161224	859361215	881909753	0.166857
P48730	0	8394001.9	18281516.4	0	0	0	0.167661
Q13303	0	2072198.7	3995855.22	0	20683127.2	27358588.3	0.167773
Q7L014	18429255.1	0	3160794.04	0	75665556.5	77949598.8	0.168761
P61247	0	2454210.8	5399965.25	0	0	0	0.168812
P49840	218092928	132656640	85580125	55217630	1080766916	1642987877	0.169407
P42356	26943093.1	12151215	0	0	0	0	0.169685
P43307	24347681.5	0	44121606.5	0	369433509	661156152	0.169691
Q96Q42	4818179.43	5111391.7	0	0	30831191.4	36636869.8	0.170366
Q9Y520	40723568.7	21259079	36631900.8	16892627	200188169	308093937	0.17048
Q9GZV4	9074871.64	4056734.5	0	0	0	0	0.170688
P00338	0	3380048.5	7396221.78	0	34455360	44014618.6	0.170845
P00491	0	0	0	0	17032593.6	38158116	0.170856
O15320	0	0	0	1524793.5	23679861.1	60253934.1	0.171523
Q9H792	36637764.1	0	16222749.8	0	0	0	0.171774
Q00610	48177834	37637575	101576910	20477989	417175238	556662908	0.171821
Q9BUQ8	0	0	0	0	12731341	29110529.6	0.17319
P26232	15760501.3	0	6788502.75	0	0	0	0.174944
Q13427	47510402.1	9551413.6	63298637.4	2878982.6	435347051	738109876	0.175251
P17612	0	1598411.9	0	0	27669239.9	61104043.3	0.175484
Q6Y7W6	34136003.1	2903807.4	14043730.1	4617066.1	124627106	177778042	0.176369
Q8N4C8	68452452.9	50044954	48941355	16824988	391107944	609699799	0.177167
A0FGR8	63448718.3	0	25065926.6	13184973	247914441	442792064	0.177656
P85037	33626092.8	0	8427344.23	9934166.5	95005346	172826092	0.177781
P08621	63589352.9	12012937	33014784.9	12884044	215171857	244490899	0.178021
O95104	16512498.5	21629608	0	0	238770617	492713833	0.179928
P26373	0	0	0	0	33838685.5	82443110	0.180545
Q8TBA6	6506861.25	6001589.4	0	0	32995906.5	35378314	0.183874
P06733	28276212	11794407	31072966.2	5626631.9	149392494	144300774	0.184424
P27987	0	0	45859232.3	0	147593346	231462709	0.184577
Q15311	15745577.3	0	31178604.8	0	198015957	131786874	0.184589
Q9P035	29051604.4	39365381	133617221	20337650	597356786	418070345	0.1849
P17931	68853313.4	0	11285614	5197336.5	195647469	217924372	0.186045
Q15742	36518536.5	0	0	0	116025700	109123233	0.186641
P38606	0	6417144.6	8466452.15	0	42065365.6	68527123.2	0.18791
Q6A1A2	0	0	24831644.2	0	143248026	82885518.2	0.188182
O15357	35631627.7	19630464	42952706.5	8744413	178064349	207584771	0.188386
P24928	49957524.8	16592238	34257793.2	8673906.5	193735850	200276101	0.188446
Q9UEE5	9044769.5	0	3445336	0	0	0	0.189328
P30044	0	3816112.8	0	1336704	27567321.5	70798301.1	0.190334
O43399	15914733.4	0	0	4235914.7	57656368	136462453	0.191817
P36915	12126855.8	0	0	0	37763094.4	64966332.6	0.192041
O95218	45210852.2	12957116	23144425.1	11512667	128013820	135135837	0.192691
O00559	128709710	38493613	113906617	12257048	562336303	729935795	0.19357
Q8TB72	15636240.4	3910582.2	878211.607	2240641.5	39328958.3	45736380.4	0.193623
Q15477	77073525.1	18174207	35870835.8	9959445	245036202	314925474	0.194212
Q96I25	0	0	6463860.31	0	28853149	62833530	0.195262
P49761	17815105.2	4432914.5	0	0	54335424.3	76325137.5	0.195952

Q99590	22388542.5	12144971	13785662.3	0	100933053	120199570	0.198425
P30085	34863343.2	7391333	24802312.3	5878217.9	115359177	135047377	0.198527
Q9NYV4	0	8676145.7	13407080.3	4069173.4	58790954.2	34444193	0.198993
Q96ST3	0	7503819.2	21381912.5	0	0	0	0.199029
Q04637	11279900.9	0	4020158.63	0	35828519.2	38818281.9	0.199941
Q86TB9	16816583.3	12768677	6884703.19	4376174	78853666.8	159439653	0.200304
Q8IWW6	168761211	101258864	221971606	44732772	792016612	907221063	0.200537
Q96QR8	54998199.6	24628877	15572450.9	7796761.4	163386392	220011983	0.201132
Q5T8P6	21964694	2838763.7	17482578.2	3429121.8	81248990.9	137846098	0.201883
Q9H0B6	29682103.8	8789209.6	12314643.1	4367902	85134459.9	117069418	0.203027
Q96PK6	0	34945576	50651720.8	10880237	303219191	743632419	0.203657
Q02818	103962993	17680603	39075167.9	16628799	265194378	370419228	0.204014
P31943	115371761	35872528	39763138.5	32371518	260307633	406369391	0.204597
Q14980	58371727	18160334	0	9431936.9	129604214	175550090	0.204795
P19532	0	0	30440222.9	0	122208646	282820353	0.2048
Q9Y463	119520442	47478031	84733347.1	19508633	430692996	681383196	0.205217
Q14204	417625219	37474112	107612106	30709120	1089569437	1596333846	0.205798
P61978	291049562	56100979	0	0	1232629057	797194621	0.205851
Q9NRG9	15790611.6	6458267.3	0	0	61568447.6	124424829	0.206202
Q5JWF2	0	0	0	0	17745168.9	53805463.4	0.206359
Q07955	0	22869423	405058228	4555199.9	972265617	1243093094	0.208002
O43312	169095908	113338382	117276173	49677721	524680874	655886646	0.2089
O00160	22689211.9	0	14090632.6	0	130500084	310070715	0.209619
O60502	53569592.5	0	0	0	163340008	122505238	0.212339
P78344	0	17411847	0	20334151	33433326.3	152727709	0.21236
Q9H4G0	38395051.8	12042030	25797577.5	10387706	109900946	192741149	0.212474
O60341	0	0	16836446.5	0	38857009.5	63000641.3	0.213779
Q5VSL9	135474163	73296939	152824119	26077707	552971415	626722294	0.214013
Q05209	43046305.8	8788232.4	0	5303041.2	90464223.4	143527607	0.214083
P08238	248547487	101507092	366341330	81145948	1002146874	1464332774	0.214115
P40818	88776252.5	19882102	51412511.2	12665116	287965358	246130815	0.216571
Q9BZH6	15407890.4	0	4633262.33	0	0	0	0.217125
Q9H2K8	9920583.97	0	0	2506860.3	15629127.5	32395891.7	0.217589
Q9Y6K9	17316952.2	4811896	12565868.1	2517510.3	54266042.3	57826602.8	0.217962
Q6KC79	11937468.9	12108435	31792756.4	2368329.4	99998818.8	178772396	0.218111
Q16514	81451487.9	13576585	11699610.8	6376172.7	0	0	0.220222
Q96MU7	58028801.2	21903810	30292588.7	6836074.8	167054915	204666406	0.220315
P25788	218908979	82543294	427546038	48850566	1174941535	1256022137	0.220478
P53396	28018131.5	6654371.4	17984698.4	6047423.6	79136703.9	149470146	0.22088
Q93008	10488294.6	1779087.4	21545033.3	1488152.7	113628766	60913095	0.222962
P11021	208134954	72943663	117699359	68418841	429332957	619626237	0.223555
O00429	0	0	17540422.5	0	36729242.4	53199637	0.223729
Q7Z6E9	0	6369404.4	0	0	87361117.3	29876793.4	0.224162
P15056	69849610.4	19371603	51022852.3	9301717.1	203770467	252401029	0.22545
Q96CG3	14079806.2	0	3929015.83	0	0	0	0.225654
P51991	43193095.5	14576712	26435013.6	12532915	95980774.2	141058951	0.226006
Q15208	11901348.9	3280891.7	0	0	0	0	0.227025
Q8IYB3	279913018	66225137	121637566	39205452	673691640	1101177804	0.227465
P62995	247251935	32707985	196039809	2710571.7	820835081	1364605187	0.227803
P26038	20097901.2	3736906.6	8757961.7	2536804.4	47563636.4	76025070	0.228238
Q7Z417	25922309.6	8785309.7	17918569.8	5135592.8	154022183	77919538.4	0.228357
Q13029	17213729.8	4672209	0	0	0	0	0.228765
Q01543	15907839.3	5097428.2	8931636.95	2467113.4	40712084.3	51742829.5	0.228812
O00410	0	1227279.8	0	0	21830450	77625970	0.229592
Q9UI08	57405177.9	48696403	87447679.2	17450326	286418390	574372985	0.22983
Q9UPQ3	8109065.19	4641820.5	4528051.97	0	36585545.1	27582539.8	0.230778
P04406	14093781.6	0	20193927.5	0	127557563	380186199	0.231157
P11831	0	0	16952799.4	0	48651072.1	129599129	0.232215

P50613	28991329.1	10982651	19432764	4128060.5	82960691.3	92139370	0.232533
Q13242	24979380.6	12482435	20767769.1	2683810.4	102036187	226629458	0.233264
Q8NC56	135725148	38834357	116561850	18858137	399689284	528580232	0.233275
Q69YN4	70396690.3	25460058	47536573.8	16731071	169894546	258956589	0.233917
Q14C86	41773924.8	7379906.8	24777999.5	9106498.4	93614339.9	165855249	0.234328
Q9BTL3	23243922.7	5204609.8	14241228.8	2848617.9	102529881	60472548.3	0.234545
P31146	0	0	7318922.25	0	18238081.4	45783920.9	0.234917
Q9H1K1	11144015.5	0	0	0	25093302.6	58233355.1	0.235945
O75376	11742932.9	2973398.5	0	0	0	0	0.236416
O60361	0	0	9205593.77	0	33391884.5	103139749	0.236947
Q9NV70	60136802.4	10523352	29302814.1	17806326	104708125	156684958	0.237183
P19105	0	0	7400344.28	0	20921318.8	58614696.3	0.237508
Q7L9B9	15364495.5	0	3804816.88	0	0	0	0.238819
Q96D71	36672994.5	21680979	47810115.3	19506869	107477039	211259353	0.23882
Q8IWB9	16658052.7	0	0	0	31654396.3	43537876.5	0.239456
Q8TEU7	11114722.7	0	6853319.84	1458560	25530505.8	47586377.3	0.240693
Q15052	422741428	133491473	269443039	47016820	1200868379	1199821100	0.241318
Q17RY0	55883373.8	12633910	29892494.1	3182700.5	143581891	177764096	0.241539
P06576	6684812.5	2419519.5	7405483.4	0	25871217	47610512.6	0.242186
Q96CX2	91858751.5	56517264	89368588.6	13980376	302739849	452439118	0.242191
Q9ULH1	133550041	45117698	75708138.1	23934572	310671228	382636996	0.242438
Q00839	61556217.8	5348992.9	4818213.81	12747473	101664036	240479030	0.24249
P49915	40317506.1	0	0	4659288.8	63162756	87954876.1	0.242845
Q15149	43182814.4	4278326.8	21674114.4	11567596	120521059	353454048	0.24311
Q96RK0	12905559.9	7558158.5	4210607.31	0	52977392.3	36069669	0.246668
Q9P2B4	108928001	28042874	16074163.4	21762960	178208644	287236116	0.247713
Q5T4S7	116808302	36712009	66678671.8	18748424	264240917	431401758	0.248577
Q13573	355978807	121262984	166996304	85031667	683701092	1069538530	0.248965
P08559	173281636	38829948	64021806.5	17901373	375998902	434180962	0.249085
Q9UBC2	39791328	15603202	67122631.1	0	178387483	262056889	0.24935
P05412	22037276.6	3458431.2	9601125.38	2319395	45638760.7	76200982.7	0.250663
Q13425	49730057.5	17958909	127906303	11223627	263362155	489444744	0.252785
P42566	36515678.8	7866459.9	0	0	0	0	0.253293
Q9UKM9	25744335.1	25392478	47351566	3578971.4	143935065	330265564	0.253593
P35579	22240947.5	6449000.9	4708214.81	0	55603046.3	130408892	0.25368
P62258	87060421.5	34883275	71220936.3	37091020	1341247856	376097464	0.253963
O15173	73911972	8294378	6403981.28	0	0	0	0.254039
P26641	20671211.9	4418829.6	0	0	0	0	0.254066
Q14019	0	9360596.8	44546548.6	0	118368273	362224811	0.256362
P19634	51681980	10449918	18931292.7	0	116789026	162964410	0.256685
Q86YS7	25708405.8	6652968.2	15899299.5	0	69919240.1	81623169.1	0.257122
O75494	168833387	48390534	85180413.6	40268538	309870787	420261485	0.257883
Q92540	20109812.6	4124765.5	0	0	0	0	0.258137
P11142	241860413	73590961	116374335	48275643	463161591	659577644	0.258299
P21283	5287184.5	2325649.6	5982376.28	0	18408978.2	32070091.6	0.259237
Q9UNZ2	18812639.5	11169659	13811767	6112273.1	99155743	375101237	0.259379
Q13283	725467840	258735190	259019688	192314566	1579041224	1218772401	0.259529
O15047	38925467.9	0	23032419.1	7556271.3	73178396.4	88169142.1	0.260333
P49585	655662269	255112354	572154692	123642921	1684237764	2018551123	0.260527
P23528	32609652.3	13079249	32471439.6	5900822.9	91931608.4	104081408	0.261457
P49815	39035791.9	8850629.1	17521280.1	6569132	74734855.5	91362087.1	0.262671
O15143	77076086.3	7199365.9	17774234.3	12668432	117262142	185013195	0.263852
Q15831	42562572.6	6665281.2	17997136.4	5403285.1	84273581.3	94423438.4	0.264737
Q6VN20	38535942.6	8196344.1	20119940.9	5166922	76671544.2	109947253	0.264864
Q8WV28	123978927	0	63265406.9	18849410	238181654	260302474	0.265028
Q9H3Z4	163602605	39058976	71293895.9	17661339	352907133	832113355	0.265361
P17096	0	0	12065182.4	41678318	22982147	319721388	0.265849
Q7Z460	11974638.7	3026413.5	4959098.2	0	28753514.5	32005395.4	0.265987

Q9Y2W1	163304736	72208624	113303782	18733419	401658843	731240495	0.266787
Q96AE4	76743429.4	38981997	82380728.5	15455209	233797324	237395285	0.267029
Q7Z422	45480796.4	0	42350809.9	0	124933961	152730053	0.267834
O94885	36183183.7	19534113	39265647.7	0	122112395	164767949	0.267937
Q9UBF8	163183127	50821203	90526599.7	31981036	332543936	375327325	0.268156
Q9UQC2	10070673.9	1855715.2	0	0	0	0	0.26823
P63244	15932611	3979076.3	12659484.4	1992733.5	37026321.9	63662271.6	0.268672
O75379	160020031	59359062	31838875.2	21776825	306282425	333631360	0.270323
P10412	23835853.7	8165953.7	15451348.6	3337679.2	51740094.8	88936479.5	0.270462
Q92882	2656619409	440231666	0	257868288	4586094339	4658009723	0.270484
P58876	18382979.8	4175969.1	7729531.13	2154022.4	78623617.9	332302908	0.270485
Q99880	18382979.8	4175969.1	7729531.13	2154022.4	78623617.9	332302908	0.270485
P39019	14576872.9	3577769.4	0	2214328.7	22625591.8	26944205.9	0.271583
P02765	100098761	7688998.4	137715469	27046932	294916851	291253554	0.27387
Q9HCH0	0	0	11599388.5	0	23310287.6	19355693.5	0.273953
Q15773	32437710.5	7173864.9	16028933.3	5848586.5	56992968	86562532	0.2746
Q3MII6	16877523.6	0	9791045.12	0	35825285.3	49986007.9	0.276496
Q16512	79221433.5	38627498	40897211	7890665.1	176220398	343459286	0.276963
Q14004	21396022.8	7027809.9	17812565.1	2586853.5	57327386.2	56263351.3	0.277891
O95251	4869834.88	0	0	0	10785280.5	43019468.5	0.278657
Q99543	71953992.2	9690280	6334834.63	7627646.6	0	0	0.280584
Q5VT52	35219426.2	0	17710082.3	0	88747414.5	73417178.1	0.280628
Q15019	1817201545	401552332	1280745149	125217660	4103353761	9326895878	0.281669
Q6ZS17	29657423.4	10173940	30430412	2953052	77252825.2	118987564	0.281737
P61160	0	31208919	82695223.7	0	147536850	273026490	0.283086
Q8N3F8	0	6431575.4	19753164.3	0	56530871.6	34165631.3	0.284982
O43586	37116925	21245726	48988231.7	6960009.1	132228907	428016222	0.287628
P61981	660239363	179052604	309762509	137783795	1116432751	1371783494	0.288482
Q9C0B5	0	4391256.1	0	0	22171204.3	132271334	0.288741
O43491	16256464.6	5582776	2019975.94	3153569	27572891.3	26024569.5	0.289094
Q96JH7	36491020.6	12591575	0	3981703.9	57216306.3	76013413.1	0.289491
Q96T37	87014257.4	20633518	61029216	11860826	170044636	258892514	0.291863
Q96PY6	11988357.1	0	2689508.38	2286263.2	14369381.3	31192682	0.292757
P27105	35304750.8	4811809.9	0	0	0	0	0.292988
A7E2V4	54682172.8	12612914	33537623.6	7311420.3	103947273	139303236	0.293168
Q9Y6D5	129407842	26657579	66053978	14768230	232677892	322701198	0.294244
P52565	16186493.4	2610073.9	8046894.69	0	33048429.8	90335533.2	0.294249
P60709	202210107	48080457	184521150	33331126	436555177	1096492683	0.295495
P09525	14207705.9	4799340.4	29345864.2	1795752.6	51953781	90045059.8	0.296737
P25705	0	2326035.6	7022610.93	0	12837118.3	44039034.9	0.297497
Q6P2E9	55542599	80660090	96315659.9	5074773.6	240443292	406132744	0.298266
Q92625	74589409	20349548	51284571.1	8815252.9	169241017	165860251	0.298539
P49841	384096118	132656640	91913619	88497619	557533888	1642987877	0.298642
Q9UPU5	110866698	19288698	50564480.1	26782367	154487505	380454196	0.300291
P21333	10221085.2	3957778.4	21326453.4	0	62501167.7	307322653	0.301039
P27824	1949207414	213008848	758701015	232339510	3067520027	4192118625	0.301277
Q5T200	36865770.6	13385763	27023692.8	0	97830449.2	98001310.6	0.301507
Q494V2	36357655.8	4380259	0	0	0	0	0.301604
Q9Y4G2	16733828	4197274.9	9592339.58	0	33269947.6	65628124.9	0.302855
Q6UN15	77594611.3	16320493	39342619.4	14401777	127521755	159921858	0.303103
O60271	233241123	18869935	92339791.7	19924989	388140615	483781508	0.303724
Q06830	29438020.9	11388288	23160814.1	4480080.4	59178872	132278152	0.304748
P20020	130782976	14929658	44861367.8	25012024	266275165	176159690	0.304835
P10644	949515883	136587854	478705064	75893986	1648662785	2252993593	0.308755
Q9Y2V2	482432428	163119636	335760243	68698455	923291569	1264957131	0.311679
P78536	49306160.9	8265771.2	20593107.7	7176155.2	73642884.9	108981127	0.312477
Q6PJT7	24653040.9	5468475	6260265.03	3621060.3	33023468.4	61933394.1	0.312683
Q96EZ8	14314096.3	2986227.7	0	2044553.5	0	0	0.313335

O75122	98984419.2	9950969.2	34110821.4	11223016	193528085	148922316	0.313637
P31946	35983705.4	11864971	29241529.7	8145236.5	63105918.2	131781914	0.313913
Q9H1B7	63507773.5	24865221	37110596.9	15694680	100394254	152183478	0.314166
Q86VM9	27771063.8	5765932.1	9526942.75	3975738.9	41444331	55746435.8	0.314822
O76080	12497086.5	5392511.7	13011264.3	1482760.1	32833361.9	34279159.2	0.316235
P29692	139417438	34411174	56759551.2	10951414	244166721	294562566	0.316598
Q13177	627062931	175554265	261179779	55792866	1053828795	1461988241	0.317087
Q9H1E3	101027807	26344995	30725247.2	23496692	123237555	270278132	0.317594
Q8NAV1	18145423.2	7916896	7505677.72	0	34120518.8	60500099	0.317991
O14497	47647234.1	30963764	75364778.5	12555077	186790000	137336635	0.317996
P46527	20050211.4	0	9018763.81	0	31299040.3	62151835.9	0.318592
Q8IZP0	18295263.3	0	4816315.22	0	25329944.8	60627149.5	0.31877
Q9UQB8	34572934.3	5243126.9	0	6622492.4	49831341.3	41508280.8	0.319261
Q99683	32209993.6	8421522.8	6647866.38	0	107812968	49339132	0.319295
P63267	151717081	31007089	112000292	17111807	267831663	710355648	0.319774
Q9Y5S2	186511007	101464966	212276990	52799285	423537510	523571892	0.32068
P67809	131481200	76475761	148109526	64008501	320434358	238540732	0.3212
Q9NSK0	11109550.7	0	0	0	13912351.6	23292560.8	0.322506
Q6PKG0	70574530	15060023	33806884.6	14522939	231282014	93596266.4	0.324976
Q9BRQ0	12106685.9	3240577.8	0	0	18625614.9	23874370.3	0.326254
Q99613	326549273	33635231	73580377.4	24487189	478033680	582489451	0.326407
P14618	29678834.8	8626485.6	25120730.5	3506416.6	54993889.4	138539446	0.326436
O60333	12479368.8	0	0	0	17520204	20523443.1	0.326774
Q12802	97169509.8	13132384	52638813.9	18130242	142452841	197352520	0.327418
Q9H299	22044831.3	6780629.4	26013619.5	3808802.7	48350360.9	73535483.5	0.3279
Q8NBN3	120191908	44514901	52595144.1	13492914	188192377	346165891	0.328207
Q9H0D6	12914953.6	5623035.6	24326287.2	4097241.8	54478063.3	36769676.3	0.328359
Q92538	214444416	66036308	162203176	33048482	448336621	440068538	0.329299
Q96E39	68459696.6	12158450	0	7341873.6	73803087	210319750	0.32951
Q15366	0	0	38706520.6	0	55334255.1	60728283.3	0.330581
P07900	25181583.7	5395099	13917681.5	0	54814566.6	51216624.4	0.332149
O15400	37831468.5	4309534.1	43050140.3	5887088.8	83077363.1	99712579.3	0.332948
Q15365	78734268.1	42891153	64618947.6	0	353834539	171239277	0.333149
Q9BWU0	23211678.7	3310655.4	0	2542393.3	0	0	0.334925
Q14671	18760592.5	3178695.1	0	0	26827270.8	34197640.1	0.335038
Q9P2C4	0	0	0	3802904.6	62210441	0	0.335822
Q06210	152101813	23199188	48155429.4	18240025	200442570	318569738	0.336509
P51116	173617605	50965953	125937332	24984753	425315283	302493033	0.339232
O95155	67606873.9	32912416	76103141.2	0	175177975	233564694	0.33953
Q9ULS5	0	0	0	3442053.7	0	62937201.7	0.339705
P68104	43986867.6	12586604	38662648.5	6514224.7	75222416.4	189441000	0.340287
Q96PU5	87604387	33427672	80262600.8	12692361	193114848	202941275	0.341581
H0YGG7	15726776.5	4787775.6	19915385.8	0	37344345.5	70874263.6	0.341687
P18433	24045939.1	6453822.8	11715021.2	0	49546436.3	47494427	0.34297
P62269	0	0	0	1166762.7	0	24770253.9	0.34432
P62805	15669767	3480002.7	7683828.62	0	24856682.9	124095799	0.345257
P98175	36898489.5	7749090.7	12136344.8	3713210.6	47178399.8	95832306	0.346302
Q0JRZ9	191521121	22240164	59351316.6	17450825	247291647	391753313	0.348639
Q92930	10076232.8	0	12210414.9	0	20365706.1	44993473.7	0.348814
Q09666	744940577	157795470	265734253	91231891	1008587769	1491076015	0.350003
P51532	65577420.4	21277437	30400261.9	9539491.7	94739895.7	147083732	0.350158
P62750	0	0	0	12104919	335141392	0	0.351079
P63104	14363893.1	3235019.9	23086620.6	4305265.2	28885711.3	92050775.7	0.351596
Q12979	177120826	7233199.4	14199174	1774680.4	243963545	277615811	0.352917
P37837	0	0	0	1814508.9	0	58570189	0.35427
P31749	24155629.8	0	0	4943098.1	15404086.2	109501360	0.361251
Q14839	39490657.5	10940324	31842376.5	0	78027649	104903364	0.361496
Q04917	20154837.3	6465045.5	27901761.2	0	45187115.7	103886908	0.362901

Q8TEQ0	45942946	896451.97	4886367	6900850.8	0	0	0.363362
P07737	26245036.5	0	41670691.1	5483920.9	50214056.5	127280840	0.36529
P10398	70640480.1	0	24939238.2	9970812.4	86210759	113186433	0.365437
Q3KP44	40495108.5	8428372.4	19595558.1	3779582.6	61345397	77258124	0.366783
P35125	0	6223870.7	0	5160904	0	69390153.8	0.367064
P08758	24349240.2	5989436.4	16578615.2	3156153	32483367.9	95477023.4	0.369032
P15153	10007869.5	3241457.2	8696412.94	0	17279999.2	43311396.8	0.369721
Q9Y4H4	192300516	20098219	117002636	10304027	350236380	334210351	0.372675
P04083	52563884.8	15931468	20242919.1	5899674.4	80077609.7	89186264	0.373736
P62328	0	0	0	0	0	57263163.1	0.373901
Q96KK5	0	0	0	0	0	75477052.5	0.373901
Q8N3U1	0	0	0	0	0	226614664	0.373901
Q9P013	0	0	0	0	56635490	0	0.373901
P60903	0	0	7536698.65	0	0	0	0.373901
P06703	25580042.1	0	0	0	0	0	0.373901
P60866	0	0	0	0	0	44962052.8	0.373901
P62857	0	0	45262899.6	0	0	0	0.373901
P52566	0	0	0	1970635.1	0	0	0.373901
O75995	0	0	0	0	0	92135175.6	0.373901
Q9H7T3	0	10177776	0	0	0	0	0.373901
P62081	0	0	0	0	0	24420515.8	0.373901
P20800	0	0	5048576.44	0	0	0	0.373901
P27361	0	0	0	0	0	61715193.3	0.373901
Q9Y5S9	0	0	0	0	11494983.5	0	0.373901
Q13636	0	0	8811773.25	0	0	0	0.373901
Q9NP95	0	4376964.1	0	0	0	0	0.373901
Q9H063	0	0	25716441.3	0	0	0	0.373901
Q9BWG4	56101239.9	0	0	0	0	0	0.373901
Q8NGP0	31215259.7	0	0	0	0	0	0.373901
Q9NXW9	21175763.5	0	0	0	0	0	0.373901
Q96KP6	0	0	7355047.5	0	0	0	0.373901
P24534	31401964.4	0	0	0	0	0	0.373901
P35268	0	0	0	0	0	27208580.3	0.373901
P10599	0	0	24176194.5	0	0	0	0.373901
Q96E09	0	0	0	0	0	45988678.2	0.373901
P09488	0	0	0	0	0	53092943	0.373901
Q86WR0	16422680.5	0	0	0	0	0	0.373901
Q13404	0	0	0	0	0	302170989	0.373901
P46779	0	0	0	0	23236889.5	0	0.373901
P62899	40907629.7	0	0	0	0	0	0.373901
O15511	0	0	0	0	0	28486835.4	0.373901
A0A1B0GT	0	0	0	0	0	133722325	0.373901
P49207	0	4495480	0	0	0	0	0.373901
P16219	0	0	0	0	0	15569907.8	0.373901
P49069	0	2277440.3	0	0	0	0	0.373901
P62318	0	0	0	0	0	28499815.1	0.373901
P53999	0	0	0	0	0	36729260.6	0.373901
Q99623	0	0	0	0	35925177	0	0.373901
Q01469	0	0	0	0	0	13380317.3	0.373901
Q8N831	0	573952891	0	0	0	0	0.373901
Q9H4G4	0	0	0	0	0	26292515.4	0.373901
P05141	0	0	0	0	0	55965683.9	0.373901
Q01130	0	0	0	0	27297052	0	0.373901
P50213	0	0	0	0	0	41483611	0.373901
P62241	0	7107758.7	0	0	0	0	0.373901
P14174	0	0	0	0	0	8085409.17	0.373901
Q7Z309	0	0	0	0	20494943	0	0.373901
P12277	0	7128046.9	0	0	0	0	0.373901

P62277	0	0	0	0	0	17495380	0.373901
P09382	0	0	0	0	0	37996197.5	0.373901
P46108	0	0	0	0	0	37127082.5	0.373901
P07948	0	1782798.7	0	0	0	0	0.373901
B8ZZF3	0	0	0	3816408.6	0	0	0.373901
O15371	171265322	0	0	0	0	0	0.373901
Q9UBT2	0	0	0	0	17736816.3	0	0.373901
P28482	0	0	0	0	0	129337370	0.373901
P18124	12324685.3	0	0	0	0	0	0.373901
P60953	0	0	0	0	0	33394659.4	0.373901
Q8NHS2	0	0	12991888	0	0	0	0.373901
Q9UHB4	0	1948780	0	0	0	0	0.373901
P05496	0	0	0	0	16310354.9	0	0.373901
Q86U70	0	0	4559011.54	0	0	0	0.373901
P19338	0	0	0	0	86588569.3	0	0.373901
Q96K49	7935989.06	0	0	0	0	0	0.373901
P27797	0	0	5461452.56	0	0	0	0.373901
Q14938	0	0	0	0	0	37869903.3	0.373901
P04792	22618941.8	0	0	0	0	0	0.373901
Q5T0N5	0	0	0	0	0	13888856.3	0.373901
Q9NWB6	0	0	0	0	0	147587802	0.373901
P59998	0	0	0	0	0	43294785.6	0.373901
P60174	0	0	0	0	0	44053685.5	0.373901
P61313	0	0	0	0	0	34527661.8	0.373901
Q8NBS3	117121712	0	0	0	0	0	0.373901
P50395	0	0	0	0	0	32105940	0.373901
P32969	0	0	0	0	0	65628359.4	0.373901
P28161	5135069.02	0	0	0	0	0	0.373901
A0A0J9YW	0	0	11598124.4	0	0	0	0.373901
P45880	0	0	0	0	0	27858554.1	0.373901
Q8TE23	0	0	0	0	88838057	0	0.373901
Q9UI15	0	0	0	0	0	39128005.9	0.373901
Q9UH62	0	0	0	0	0	18716947.5	0.373901
Q12857	23481999.3	0	0	0	0	0	0.373901
Q15435	0	0	0	0	0	17619959.6	0.373901
Q13098	0	0	0	3688773.4	0	0	0.373901
P02794	0	0	0	0	0	14803041.9	0.373901
Q3V5L5	194526299	0	0	0	0	0	0.373901
P15880	0	0	0	0	0	25702364	0.373901
P30050	0	0	0	0	0	22982246.3	0.373901
Q9H8W4	0	0	0	0	0	22845647.5	0.373901
Q86VQ6	0	0	0	0	0	80856948	0.373901
Q4G0F5	6060631.47	0	0	0	0	0	0.373901
P17600	0	0	45237347.5	0	0	0	0.373901
O43521	0	0	0	0	22563839.7	0	0.373901
P12814	0	0	0	0	0	38554609.3	0.373901
P57682	0	0	0	0	0	27207941.5	0.373901
P0DN76	0	0	0	0	0	26833779.8	0.373901
Q53ET0	0	3073884.5	0	0	0	0	0.373901
Q9UBR2	0	0	0	0	0	17508326.5	0.373901
P0DMP2	0	0	0	0	28230262.9	0	0.373901
Q01664	0	2538845.5	0	0	0	0	0.373901
Q15022	0	0	48256252.9	0	0	0	0.373901
P05165	0	0	0	0	0	76904158	0.373901
P04899	7607673.95	0	0	0	0	0	0.373901
P43246	0	3752535.3	0	0	0	0	0.373901
Q9BQK8	56960198.5	0	0	0	0	0	0.373901
P40925	0	0	0	0	55067926.9	0	0.373901

P35237	0	0	0	0	0	28104123.3	0.373901
Q92543	624951.922	0	0	0	0	0	0.373901
P00367	10231615	0	0	0	0	0	0.373901
Q01196	0	0	8150702	0	0	0	0.373901
A6ND91	0	0	8852493.66	0	0	0	0.373901
Q8IUR7	0	0	0	0	213649012	0	0.373901
Q8WWY3	10778952.3	0	0	0	0	0	0.373901
Q8WYQ5	0	0	0	0	30077535.4	0	0.373901
Q8TB45	0	3553150.9	0	0	0	0	0.373901
Q12797	0	0	0	0	0	15747486.9	0.373901
P11279	0	0	0	0	0	41637643.5	0.373901
Q9UGH3	0	0	0	0	0	38569319.6	0.373901
P59045	0	56762792	0	0	0	0	0.373901
Q9UQL6	0	0	7156200.44	0	0	0	0.373901
Q96JD6	0	0	0	0	0	64618334.2	0.373901
P16615	0	0	0	0	0	18544657.8	0.373901
P60842	0	0	0	0	0	27882473.5	0.373901
Q9Y2K6	14178697	0	0	0	0	0	0.373901
Q8N142	0	0	2913178.33	0	0	0	0.373901
P55212	0	0	0	0	0	27523007.5	0.373901
Q9H361	0	0	0	0	22899456.8	0	0.373901
Q9Y2W2	0	0	0	0	36232272	0	0.373901
Q7L1W4	28337493.5	0	0	0	0	0	0.373901
Q9NP66	7181573.75	0	0	0	0	0	0.373901
Q15814	0	0	0	0	0	10093243	0.373901
Q9H598	0	0	0	0	0	37326054.3	0.373901
Q09161	30732154.1	0	0	0	0	0	0.373901
Q9H1Z4	0	0	0	0	15571027.1	0	0.373901
P0C629	1754347.84	0	0	0	0	0	0.373901
O94776	0	1532984.8	0	0	0	0	0.373901
Q9NQL2	0	0	0	0	0	417061983	0.373901
Q96PN6	0	0	0	0	340971117	0	0.373901
P42680	0	0	0	0	0	35118564	0.373901
Q5T7W0	0	0	0	0	0	353115742	0.373901
Q9UPT5	0	0	0	0	0	48124301.8	0.373901
Q9NQG6	15760557	0	0	0	0	0	0.373901
Q9UBW5	0	0	0	0	0	256853538	0.373901
Q8N5Y2	12908168	0	0	0	0	0	0.373901
Q14814	0	0	0	0	0	23249156.6	0.373901
Q96S55	0	0	0	0	0	32968533.8	0.373901
P13804	0	0	0	0	0	17106790.4	0.373901
O75400	0	0	0	0	0	30396029.5	0.373901
P52209	0	0	7269539.31	0	0	0	0.373901
Q6P2S7	211434203	0	0	0	0	0	0.373901
Q9BSQ5	0	0	0	0	0	30376869.9	0.373901
Q9UPQ9	16479975.9	0	0	0	0	0	0.373901
Q9Y561	0	0	0	0	33649958.3	0	0.373901
Q6Q0C0	0	2845728.9	0	0	0	0	0.373901
Q15057	0	0	0	0	0	33237649.5	0.373901
Q8N370	0	0	0	0	0	67824997.5	0.373901
O60256	7827468.88	0	0	0	0	0	0.373901
O00148	0	1657764.4	0	0	0	0	0.373901
Q92945	0	0	16960134.4	0	0	0	0.373901
Q7KZ85	0	0	0	0	0	24257800	0.373901
Q96F86	0	0	0	0	0	16164263.8	0.373901
Q9UMZ2	0	0	0	0	0	62702500.4	0.373901
P25098	0	0	0	0	24341001.5	0	0.373901
P04198	0	0	8690599.88	0	0	0	0.373901

Q96RR4	0	3181462.6	0	0	0	0	0.373901
O00186	0	0	0	0	11345513.3	0	0.373901
Q9H7D7	8813515.1	0	0	0	0	0	0.373901
Q8N766	0	0	20207965.4	0	0	0	0.373901
Q9UPT8	0	0	0	0	0	162865255	0.373901
Q15569	0	4505841.4	0	0	0	0	0.373901
A3KMH1	0	0	713473.625	0	0	0	0.373901
P29375	10348192.5	0	0	0	0	0	0.373901
Q9NXD2	0	0	0	0	0	49289939.8	0.373901
P07602	0	6869520.3	0	0	0	0	0.373901
Q13003	0	0	0	0	0	403291705	0.373901
Q9BZ67	0	3156710.8	0	0	0	0	0.373901
H3BQZ7	0	0	0	0	0	22574036.6	0.373901
Q9Y3Z3	0	0	0	0	0	48445217.3	0.373901
Q9UDT6	0	0	0	0	0	27102182	0.373901
P41182	15695854.8	0	0	0	0	0	0.373901
P26927	0	0	0	0	0	39946120.5	0.373901
Q9NSY1	21503919	0	0	0	0	0	0.373901
Q71RC2	17620009.9	0	0	0	0	0	0.373901
Q9BXF6	0	0	0	0	0	97372573.1	0.373901
O75116	0	0	0	0	0	76487543.5	0.373901
Q01804	0	0	12826408	0	0	0	0.373901
Q12851	0	4023423.6	0	0	0	0	0.373901
Q8N2M8	0	0	0	0	0	136756920	0.373901
Q14644	43554118.1	0	0	0	0	0	0.373901
Q9Y6K1	0	0	0	0	0	63963969.9	0.373901
P21918	0	0	0	0	35953527.1	0	0.373901
P18054	31540118.8	0	0	0	0	0	0.373901
O15523	0	0	0	0	0	25852297.4	0.373901
O43524	0	0	0	0	0	87600513	0.373901
P48553	0	0	0	0	0	30864235.8	0.373901
Q8IVT5	0	0	0	0	0	13604723	0.373901
P17812	0	0	0	0	43001118.4	0	0.373901
Q9UJ41	0	0	0	0	10340494.9	0	0.373901
O75161	0	0	0	0	0	344333481	0.373901
P28290	0	0	0	0	0	30444399	0.373901
Q9H2G4	0	1908151.8	0	0	0	0	0.373901
Q4ZHG4	14905886.9	0	0	0	0	0	0.373901
Q7Z7A1	27878806.9	0	0	0	0	0	0.373901
Q6WCQ1	0	0	0	0	14466041.6	0	0.373901
Q14164	0	0	0	0	0	21163614.9	0.373901
P18206	5300391.31	0	0	0	0	0	0.373901
Q96TA1	0	0	4747191.39	0	0	0	0.373901
Q8N264	0	0	0	0	0	30189175.3	0.373901
Q92598	0	0	0	0	0	41098491.5	0.373901
A0AVI2	0	0	0	0	0	88218164	0.373901
Q86VP3	0	0	0	0	13448379.5	0	0.373901
P51798	0	0	0	0	28482737.5	0	0.373901
Q13469	0	0	0	0	41623534	0	0.373901
Q2PPJ7	13817996	0	0	0	0	0	0.373901
Q92545	0	0	3366939.81	0	0	0	0.373901
O00512	0	3542338.3	0	0	0	0	0.373901
Q6P3S1	0	0	0	0	0	22402919.4	0.373901
Q8WXX0	0	0	0	0	1901328.13	0	0.373901
P11274	13399057.6	0	0	0	0	0	0.373901
U3KPZ7	0	0	0	0	36737338.8	0	0.373901
O15042	0	0	0	0	0	9853636.25	0.373901
Q8N122	0	0	0	5183106.8	0	0	0.373901

P42338	0	0	0	0	0	24050301.8	0.373901
Q6IQ26	0	0	0	0	24876065.8	0	0.373901
Q9UKJ3	0	0	0	0	0	13488643.9	0.373901
B2RTY4	0	7238171.4	0	0	0	0	0.373901
Q8N3D4	0	0	0	0	23036820	0	0.373901
Q9ULI4	0	0	0	0	55763773.4	0	0.373901
Q13315	0	6699670.3	0	0	0	0	0.373901
Q8NI35	0	1503362.4	0	0	0	0	0.373901
Q4G0P3	0	0	22394064.8	0	0	0	0.373901
Q99698	0	0	0	0	0	39807554.8	0.373901
P49756	55537128.5	4472992.3	9705349	12364504	46095471.9	108292312	0.375169
P22059	428419368	75246247	109939849	40616384	480056878	915813457	0.377369
Q13233	0	0	137346889	90674068	0	1002227656	0.38029
Q9UQ35	373012608	84796973	157724991	36381675	528720600	675333671	0.3809
P07437	68509564.5	7934855.1	21867742	7158781.1	76655232.1	139581183	0.381134
P52594	436685838	46178219	137346889	0	539770438	1002227656	0.381435
Q6VY07	166522899	51720694	110628320	22625703	281998854	311773446	0.381801
Q07002	0	3453114	0	0	0	146188378	0.384282
Q9NZN8	58160575.3	24365325	37738211.3	11419143	75060440.8	181557137	0.385398
P62937	42364230.5	11491104	20862500.5	5116203.8	55382442.1	92046459.1	0.387906
Q15811	142463446	59637692	143822564	18237931	262987346	395780205	0.387914
P0DP23	0	0	5598816.56	0	0	163809938	0.389077
P51812	35697237.1	6677061.7	12575526	4463333	50054875	51774523.3	0.391823
Q92608	79248513.8	0	0	9743392.3	61187664.4	144281806	0.391924
P08670	303509591	104981057	208566350	32030800	439893365	817944653	0.39223
O00178	249186270	60800590	109001672	45356372	393867028	326987365	0.392297
O00193	1201810788	228312407	274079762	104820650	1345719544	2253888947	0.394143
P10809	869414.797	0	0	0	0	18063957	0.39556
P02545	187120564	33340192	84981125.6	37194005	283555736	234924409	0.396127
P16070	617707756	97196781	0	10737727	59086376	97261925.3	0.398522
O15258	130921738	25522513	45585308.6	11887707	181243338	202101338	0.399429
P62263	0	2035185.8	0	0	0	29263784.8	0.405837
P14866	23317308.5	0	0	1628275.6	0	0	0.405977
P35221	33808784.2	0	0	2459459.8	0	0	0.407407
Q7Z3J3	19143500.3	3330007.9	0	0	20976897.8	32605316.4	0.407686
Q9UEY8	12927353.1	0	0	0	0	174956172	0.407969
Q5H9R7	123115292	12473419	37528412.9	10031469	170366832	165470513	0.408326
Q96FW1	0	0	14035608.8	4773639	0	102540573	0.408905
Q3KR37	63075474	10459042	25189018.6	4367054.9	72890782.3	132642244	0.409155
P55196	183071526	22733800	48896531.3	40390860	158696898	313626124	0.409295
P98082	70010545.5	12955672	36213493	6757412.8	92704603.9	130808799	0.409666
Q9NUQ9	0	5379910.7	0	0	0	63200366.4	0.413559
Q02078	81122444.1	14603107	31678044.1	6912955.3	105091810	132884414	0.41497
P07355	142830103	24979371	60641376.3	28375880	144340929	263452989	0.415531
P25490	15083185.5	4469865.1	13062604.4	0	53367077.8	22080861.5	0.417833
Q13501	324960247	93151099	220717643	36631977	617956247	493095929	0.420141
O60381	0	3472465.6	0	0	34776174.2	0	0.421046
Q9Y6D6	16107044.2	0	0	1685955.3	0	0	0.423536
Q14160	44109635.4	8948390	12127182.8	0	26847627.8	0	0.423747
Q01518	0	2622725.2	4730167.99	2235841.7	0	39021007.9	0.424643
Q9UGJ0	21253849.6	14251355	29264385.3	8331860.9	29210283.7	114386820	0.424817
P18583	10461942.3	3696710.6	33673180.6	1791711.9	113472336	25746051.3	0.426676
Q96C19	174823576	8783885.9	12454954.5	42703059	91960290.5	361750889	0.426695
Q8TCU6	71856887.8	21227768	48911092.1	6324790.9	217664250	85901022.5	0.426839
Q96B97	662256934	166346743	318209529	72654955	1110989650	883008177	0.427479
O43182	34989314.7	6305445.4	17620126.3	6028031.6	45256653.1	50811141	0.429088
Q9H7L9	389311443	153625865	282386665	69139300	573577440	746902557	0.429847
P51149	0	4273359.9	0	0	0	34747649	0.433171

P27816	51090705.3	16162964	41808610.1	7610386.8	81171844.5	95265788.4	0.439001
Q14157	188304050	40948682	84547503.2	29321158	249631395	256123472	0.441431
Q8TBN0	0	8739696.2	0	0	0	61515815.5	0.443508
Q9Y4I1	20909547.1	6320543.7	0	3012980.8	0	158276360	0.443666
Q7Z589	0	2378359	0	0	16441269.1	0	0.44493
Q9BXP5	57164370.4	6243500.1	0	0	36862864.2	149325818	0.445586
Q6P4R8	0	0	5558562.88	0	0	37626387.1	0.446628
Q7L7X3	53855628.4	10700495	21874954.9	0	74712722.8	87331272	0.450403
O00505	57515280.6	0	0	0	57883875.6	73020485.8	0.451742
P04075	39035511.2	14396932	17576762.9	4876480.3	44762306.8	76459831.2	0.452888
Q15637	231410768	46835129	0	26820311	222062220	295158056	0.453386
Q8TAQ2	41691340.8	13900668	26497360	4303288.8	48292555.9	103962502	0.453567
P46379	105086474	12886383	17529335.3	5195618.5	47454556.3	0	0.45658
Q16836	14815385.8	0	0	2470172.3	0	0	0.457289
Q15036	56438602.4	57868496	165215451	0	213298919	306908799	0.457665
Q8N4L2	0	0	9854419.5	0	0	58339489.4	0.458533
O60315	38918492.9	11528332	0	3844800.7	29646029.4	74870910	0.461926
Q96JC9	70763762.2	30883828	21616170.6	22981009	41675444.5	199859776	0.463131
P0CG48	66954311.4	1737381.9	36741844.8	0	63296985.5	173704212	0.463352
Q9HCN4	0	0	12507661.6	0	0	70529754	0.463363
O75179	44256981	20889336	68423588.8	15524475	68108370	152122063	0.463648
P02679	0	3623593	0	0	0	20047232.9	0.465336
Q9NS56	13405184.9	3187708.3	0	1896967.5	14604668.8	14000694.3	0.467843
P13639	0	0	9628486.98	0	0	51528428.4	0.468886
Q9NZM1	0	2141036.1	0	0	0	10679808.9	0.476903
Q68EM7	0	0	7448725.06	0	0	37098371.5	0.477086
P09651	31614915.1	0	27351091.9	7912981.9	0	218651284	0.482727
P10451	18639040	0	0	3967937.3	0	0	0.484324
Q96BY7	0	2938510	5913550.31	0	0	39385833.6	0.484903
P60660	19382309.1	5150123.5	12511568.2	0	0	19400870.5	0.485555
P52756	49023035.5	0	15297194.8	0	41325953.1	93823233.3	0.485679
Q9BZF1	1142113867	236469829	509758672	157221174	1630321792	1291090750	0.487906
Q96N67	78518223.6	18430128	40425360	13362977	93610764.2	108007194	0.492438
Q2M2I8	107001637	13957323	0	0	145253033	98809465.1	0.492833
P02689	11362678.3	0	0	0	0	49553122.1	0.494311
A0A087X0	12446801.7	0	0	0	0	53354750.4	0.49676
P55327	61064848.8	0	9848122.48	15395171	9266069.63	169744372	0.501691
Q14669	98116447.3	31852494	87221081.2	15724481	128570033	199458121	0.502397
Q96T58	11228317.8	0	0	1541131.7	0	39067452	0.502685
P25440	72030550.4	59851105	144235769	14449669	154599868	292665085	0.504774
P02768	63595556.4	11969816	55967477.6	8526285.3	55156371.9	197470282	0.504985
Q8ND30	45892387.3	9092591.2	16236643.1	2882242.9	34152890.4	0	0.508158
Q6GYQ0	44040936.9	13995474	0	5929593.2	0	192007124	0.508842
Q9UQN3	0	0	6709724.94	0	26533775.5	0	0.508964
P68431	18072773.9	5248574.4	0	0	0	87825933.8	0.510017
Q12929	20507536.4	2372472.3	5919044.72	3543565.4	18094648.9	25543920.8	0.511619
Q4V328	29895474.3	3752639.1	59718082.3	9013952.8	294644765	0	0.514327
P18669	203231738	41004565	41418261.8	8450686.4	356261602	163539000	0.517893
Q86U42	8346433	7398340.7	19227403.8	0	120752301	0	0.518442
P08651	31681861	6842777.4	10584549.1	3137600.2	34425051.9	41326739.4	0.519273
Q92835	146047437	4788752.7	97467081.5	25431535	171275588	192241920	0.521766
Q68CP4	48740944.9	12784582	24466049.9	0	46503236	0	0.521853
Q8NFD5	27601218	9484951.1	18828248.1	8971101.3	0	148979515	0.52192
P61081	39544407.8	11496677	13939117.8	5296441	38815681.3	58561261.7	0.522492
Q14202	14598845.1	0	0	0	52995916	0	0.523338
P05023	8111935.19	0	0	0	0	29205163	0.524808
Q9BVC5	0	0	11469105.1	0	0	41247117	0.525003
Q8IW50	484367423	121517278	296597798	81289557	997672510	418932041	0.52775

P04049	73695927.5	15566950	23226739.3	12568234	137216492	48224820.1	0.528075
Q9Y4F3	0	6417962.3	0	0	22678580	0	0.528192
P40763	17255557.9	0	12189315.6	0	19585381	31631082.3	0.529121
Q5VWJ9	17886344.5	0	34687753.5	0	40141062	49853333	0.531886
Q8WZA1	7136566.75	6454064.6	0	0	0	43734891	0.533244
P84098	0	2880271.2	7119190.56	0	32221007.7	0	0.535408
Q96SB4	96884302.1	13508248	29424371	0	70048562.4	0	0.538504
Q9ULH0	8833855	0	0	0	29005564.8	0	0.542273
P05388	268857710	31100029	0	18931665	100782980	0	0.542519
Q9Y4E8	67868489.1	17469068	26743491.9	6272560.2	78739978.4	85247876.1	0.549055
P22626	6682632.13	0	0	0	0	20761735	0.553757
P62820	0	0	15970892.4	0	0	49492903.4	0.554304
P07339	106939302	36401713	108952566	8885468.6	158553625	208630899	0.557599
Q06413	0	0	11686906.3	0	0	35012614.4	0.561746
Q96A49	294136782	51523193	67777316.5	30690784	256690741	362904572	0.563161
Q13243	8365411.44	2831212.3	0	1484840	26731798.7	0	0.563287
Q9Y2I7	19008197.3	0	0	4223151.9	0	43162619.1	0.565707
Q8IZQ1	9230507.25	0	0	2024972.3	0	20811921.5	0.568324
Q6DN90	56737732.2	6175186.5	18552564.7	5888845.2	67002884.6	52976755.8	0.569884
P21281	0	3920274.2	8409922.58	0	0	33317873.2	0.571598
Q53GL0	41010744.5	10482968	13540598	4448158.5	0	157677241	0.572592
Q13153	8287508.06	1710836	0	0	27457613.4	0	0.573049
Q15185	2490144630	308731027	485832547	97150100	750497193	1069301362	0.578866
Q9H920	13039358.9	0	0	0	0	35655059.5	0.583442
Q13085	12339363.6	0	0	0	0	33730624.3	0.583519
P68363	9952234.09	2320985.1	18930236.6	0	13205759.4	41433007.1	0.583863
P55081	189811851	40800112	58580598.7	20386817	299769981	135654162	0.585225
O75643	22917158.5	7209338.1	22337671.8	0	34709784.1	42769081	0.585891
Q9NTI5	54744108.4	15615778	21115016.5	5159855.9	56601325.6	69098397.5	0.599795
Q9Y608	54203148.3	7781146.8	18696495.6	3131862.2	20734329.4	125951762	0.602565
P46940	13955403.5	4975213.8	7316131.15	0	0	60788565.8	0.603316
Q9NYF3	0	4205234.8	9803238.3	0	33374710	0	0.603965
Q9BQE3	9952234.09	2320985.1	18930236.6	0	13205759.4	38746431.9	0.605173
O00299	0	10907665	22606109.1	0	78581181.8	0	0.607534
O60861	21569696.1	27367850	67760739.8	6537780.5	56259816	107992548	0.610654
Q99442	41346786.4	10792945	8790209.94	4053012.8	32578001.3	0	0.610933
Q12846	59377129.3	9044423.9	10817349.3	0	71009351.6	52012910.8	0.615971
P23588	74974419.3	23263054	0	9627649.8	199590321	0	0.618608
Q9UHY1	5748447.38	0	0	0	13769298.6	0	0.619398
P06396	0	0	7002990.97	0	16669514.3	0	0.621241
P05386	3956184662	647128445	698274895	339584263	3410779373	4031440385	0.628737
O60307	34388790	20112716	32632575.5	6996028.6	34424629.9	79215463.9	0.631235
Q5SW79	27167004.8	0	15183264.8	1884347.8	86424562.5	0	0.631668
P27708	11145661.6	2917948.8	7463395.69	0	43429191.3	0	0.644851
O15379	83113070.9	22851576	31823352.6	0	57453126	42663789.6	0.647916
Q13769	162704882	46718849	102341579	23137270	236272717	153774582	0.656649
Q16204	64109049.3	8198230.3	12133782.1	6529882	45059373.3	0	0.656829
O15075	26753340.9	5703780.8	11763052.5	0	28188551.7	0	0.66061
Q96BY6	26943347.1	12504230	0	0	0	77940294.6	0.660808
Q9UGV2	12832956.1	0	0	2687319.5	21420697	0	0.662215
Q9BQE9	77284528.1	18455069	32624566.9	0	77863241.4	99983427.6	0.663086
Q86TI0	9231445.32	3577204.9	11656175.2	0	46526128.3	0	0.663738
Q86UP2	10138729.8	0	0	0	0	21021833.6	0.66526
P08134	8271293.13	2397768	0	0	0	21051679.5	0.665796
Q13148	5084181	0	0	0	0	10519864	0.66597
P24723	0	5928333	22902695	0	0	56691663.8	0.668143
Q8TDY2	31208175.5	10753009	27629297.8	2322740.6	0	125048005	0.668756
Q8WW12	13579438.5	0	0	6632392.5	15617199	0	0.675007

Q6V9R5	0	257080782	0	105026871	0	28702295	0.675606
Q92508	40103430.3	6361863.1	13772665.2	2099185.8	37318361.5	0	0.684162
O75083	0	3926838.1	8603553.66	0	0	22899655.3	0.688986
Q9UPP1	23699395.7	0	13245278	0	66627566	0	0.692248
Q07954	0	0	7854654.88	4108673.6	0	0	0.694322
O75592	14431860.1	6828068.4	8909917.83	2163845.8	0	47840540.9	0.696444
P14625	33401168	5900011.6	12460771.7	2953197.2	23156778.1	43783287.3	0.696493
Q7KZI7	16954947.8	2392390.8	0	0	0	35686495	0.697247
P08865	0	0	7140506.56	0	13501572.8	0	0.698438
Q9NRY4	13390249.7	0	0	0	0	25278772.6	0.699035
Q15648	20152771.3	11101104	23274691.8	3697253.3	86082201.4	0	0.699636
Q86VR2	161258412	29137366	51344555.7	14219268	151405797	151542781	0.702852
P61158	9670784.5	5202572.4	12735259.8	0	0	47016208.2	0.70361
Q96B36	16234322	0	0	2476775.5	25568681.3	0	0.707743
Q16637	91513416.3	10917021	14991408.3	3759910.8	75749527.4	80520211.5	0.714119
P16885	10722937.5	0	5267828.31	0	27150979	0	0.71715
P40121	17735701.6	10612935	39085397.9	6152307.8	43519853.2	0	0.731142
P15144	10329713.1	2831755.5	9992037.5	0	0	36985754.3	0.732318
Q9Y485	13384119	4220693.3	0	0	0	28984972	0.734686
Q9NYF8	1294186960	218087216	440191357	174279544	857821301	1451788811	0.737918
Q9UHB7	17881935.3	0	0	0	10658594.5	0	0.74609
O60292	9648207.88	0	0	1627875.9	13213775.8	0	0.758497
Q8NCC3	0	0	8115900.81	0	13213887	0	0.758837
Q9NRA8	16996595.2	2830999.8	6034425.33	2149606.3	0	35812046.4	0.760611
Q8WX93	0	0	6758834.41	0	10629114.3	0	0.773977
Q15836	35594471	4627337.3	11497947.2	4710679.9	34051242.4	0	0.776157
Q04727	29699071	11408565	25535427.7	7380473.4	84259989	0	0.777024
P62070	50149731.3	8569918	7540264.67	9034230.5	0	84390048.6	0.779312
Q9Y6G9	73544787.1	5173686.2	4556641.88	2007607.5	21544313.6	91466652.4	0.780608
P21796	50503105.6	12512741	11204544.3	2859743.8	35609847.3	53016495	0.783123
Q9UPT6	13196685.1	10250940	1014206.98	1483328.4	0	32771064.9	0.786994
Q8IU81	16604857.2	2553722.8	0	0	0	13286010.1	0.787793
P51610	0	9220931.6	34262748.7	0	63215681.7	0	0.792815
Q9ULR3	77283496.8	17930159	30688355.6	8719954.8	49655381.7	92977310.6	0.793268
P37802	47329071.1	22482091	49958924.8	5866675.8	50962868	82883132.3	0.795475
P20645	139902018	0	0	0	208701240	0	0.797779
O94913	15605580.8	0	0	3694826.8	18081559.1	0	0.799581
Q13191	49584314.1	15554120	29828792.3	5431154.5	57362818.4	46974274.5	0.804768
Q5VTR2	101462235	26889838	26329348.6	5169072.2	58354079.4	66424010	0.806405
O95391	48683922.3	20433817	4407554.16	4097830	25915165.9	59281947.3	0.811311
O94804	67512172.6	0	0	0	0	97604188.9	0.812335
Q92556	84043384.8	15725626	27572260.3	5694353.3	85140863.3	59643301.8	0.818671
Q9Y5B6	0	12505864	0	17814689	0	0	0.819304
Q9NYB9	9906841.53	0	0	0	0	13957839.1	0.824535
Q13435	43785565.3	22499539	50140023	7881902	87842290.1	0	0.825254
Q14847	9117564.59	3493825.7	6155948.97	0	0	24616768.1	0.827128
Q9H6U6	31503409.1	18990577	50105595.6	0	0	80864655.8	0.82837
Q9H4A3	12507723.9	3940926.2	7628251.27	2090332.8	0	28565413.3	0.829157
O60711	24675218	7617160.4	13044716	3685210.1	52831373.6	0	0.844035
O15085	40561456.7	10234039	0	0	0	65029584.5	0.857953
Q9UQ88	85275465	13509466	21004285.1	3902632.5	58756080.6	75016890.8	0.858339
Q7Z6Z7	39538189.8	0	10457494.5	0	39852294.9	0	0.858495
P61224	42555375.3	3527191	8327584.28	0	0	43772155.5	0.861576
Q9HBL0	51225126.8	10502323	0	4152548.5	36512069.4	30803654.9	0.869437
Q641Q2	0	17022441	0	21866566	0	0	0.869722
P29401	14921580.9	3607895.8	0	2372762.8	0	20051005.8	0.875263
P13796	330199426	47438677	51856060.5	8012892	228012360	254440715	0.875628
P23396	101956476	0	0	0	80453662.6	0	0.876531

O94876	129542647	30456763	46471423.9	17922281	78620404.3	91313619.8	0.878707
Q15819	89345157.4	26637590	46629076	0	194776155	0	0.881509
P31949	10717015.4	0	0	0	13427368.5	0	0.882287
P42858	31513605.3	9176608.5	0	0	48991228.9	0	0.890234
Q9Y4H2	16783766	2861225.9	5251204.28	2185242.8	26864705.9	0	0.892592
Q09472	0	0	8758344.06	7214158.4	0	0	0.898326
Q68DQ2	279020809	0	0	0	0	230283796	0.899344
P36543	10035509	2990436.7	6966196.39	0	0	23096071.5	0.902907
Q9Y519	26859795.2	0	0	2241916.1	0	20476630.9	0.90665
O75976	485160253	63698138	112316061	50626830	330977100	229402931	0.919962
Q8N163	408737569	79339128	128302933	0	697329601	0	0.920539
Q13043	53877426.5	4650226.1	34855018.7	4209523.9	100438302	0	0.921502
O95817	73666352	18574711	23797682.1	21427842	78603706.5	23943426	0.922722
P05089	14764461.5	3390900.8	14094073.1	0	0	36087569.5	0.923894
Q9H307	202611818	34237096	145034598	0	90866454.6	263841573	0.926152
Q8WWM7	246718482	65760236	75970178	15054620	235225071	158114402	0.942892
P06753	26481416.2	5209239.5	10357980.9	0	0	39124142.9	0.94973
Q04726	49865485.8	10021377	0	0	55696696.5	0	0.956406
O75569	49490146.3	7698647.7	0	0	61530683.4	0	0.957682
Q92974	0	10923898	12291691.4	3193967.9	21071531.9	0	0.965585
Q6ICG6	46808235.7	10218269	13434787.9	3722448.6	30470624	34501709.2	0.970896
P13637	8111935.19	0	0	0	8537635.25	0	0.972897
Q9Y490	24098135.6	3345553.3	8536282	0	34631835.1	0	0.974318
Q9Y4G8	28563100.8	13960803	12333623.4	9094773.9	44756805.5	0	0.982777
Q8ND56	143695766	21835025	24454362.6	8389655.1	75280896	109365050	0.984733
Q9BTC0	38107997.1	15393636	0	0	21827522.1	30802298.8	0.984822
O43815	174908556	10278284	69418105.1	7125654.4	176407572	67177422	0.98591
E9PAV3	17411870.1	12336438	22107006.8	30480167	13180370	7798619.25	0.986619
Q13586	23505221	0	0	0	22950677.7	0	0.987341
Q9UPR0	74754840	5755729.1	13037991	3303833.3	44128533	44964604.1	0.988862
Q6PJF5	20881650	2827823.3	0	3319746.7	20719652.4	0	0.991004
Q58A45	13464589.5	0	9430295.69	0	0	23180988.6	0.991775
Q14498	1065629017	248287566	228835308	144018816	579023479	830568810	0.992038
Q9UKV3	323107665	76216928	87130928.7	26402047	124721465	338508988	0.99351
O43318	64105917.9	24800585	48461522.6	4692899.4	0	133647094	0.994631
Q9Y2X7	32777788.9	0	10233059.5	0	43348221	0	0.995151
Q9NRP4	0	0	0	0	0	0	#DIV/0!
A0A087W!	0	0	0	0	0	0	#DIV/0!
Q9HD90	0	0	0	0	0	0	#DIV/0!
Q13188	0	0	0	0	0	0	#DIV/0!
Q00169	0	0	0	0	0	0	#DIV/0!
P13682	0	0	0	0	0	0	#DIV/0!
Q8N108	0	0	0	0	0	0	#DIV/0!
P26599	0	0	0	0	0	0	#DIV/0!
Q9NPB6	0	0	0	0	0	0	#DIV/0!
O75342	0	0	0	0	0	0	#DIV/0!
Q53EZ4	0	0	0	0	0	0	#DIV/0!
Q9C0H2	0	0	0	0	0	0	#DIV/0!
Q9NQ32	0	0	0	0	0	0	#DIV/0!
P78364	0	0	0	0	0	0	#DIV/0!
Q9NYV6	0	0	0	0	0	0	#DIV/0!
O60524	0	0	0	0	0	0	#DIV/0!
Q9Y233	0	0	0	0	0	0	#DIV/0!
Q9NZQ8	0	0	0	0	0	0	#DIV/0!
Q8IZF3	0	0	0	0	0	0	#DIV/0!
P14868	0	0	0	0	0	0	#DIV/0!
Q2M218	0	0	0	0	0	0	#DIV/0!
Q8IWA5	0	0	0	0	0	0	#DIV/0!

Q9NTJ3	0	0	0	0	0	0	#DIV/0!
P35251	0	0	0	0	0	0	#DIV/0!
O14617	0	0	0	0	0	0	#DIV/0!
P78314	0	0	0	0	0	0	#DIV/0!
Q9C093	0	0	0	0	0	0	#DIV/0!
Q86W92	0	0	0	0	0	0	#DIV/0!
P31629	0	0	0	0	0	0	#DIV/0!
