# Supplementary material for: p38α in macrophages aggravates arterial endothelium injury by releasing IL-6 through phosphorylating megakaryocytic leukemia 1
Source: Redox Biol. 2020 Nov 1;38:101775. doi: 10.1016/j.redox.2020.101775 (PMC7658717; doi:10.1016/j.redox.2020.101775)
Supplement: Multimedia component 5 [file mmc5.pdf]

Supplemental Table 5-phosphorylation sites of 293T cells which were transfected by pcDNA3.1-V5/hisB-p38α and pcDNA3.1-C3Flag-MKL1.

Proteins	Positions	Protein names	Gene names	Localization prob	PEP	Score	Amino acid	Sequence window	Intensity M	Intensity P+M	id	Fold change
O76080	48	AN1-type zinc finger protein 5	ZFAND5	1	8.99E-241	413.47	S	CYKEHLQRQQNSGRMSPMGTASGSNSPTSDS	3102900	2612000	676	
Q9ULF5	591	Zinc transporter ZIP10	SLC39A10	1	3.87E-235	412.78	S	RLNETELTDLEGQQESPPKNYLCIEEEKIID	1779800	708370	6467	
Q9UPU5	2561	Ubiquitin carboxyl-terminal hydrolase 24	USP24	0.999102	9.61E-182	378.15	S	VSNETSTGKTFQRTISAQDTLAYATALLNEK	2948900	1064400	6588	
Q9UPU5	2559	Ubiquitin carboxyl-terminal hydrolase 24	USP24	0.751508	9.61E-182	378.15	T	SNVSNETSTGKTFQRTISAQDTLAYATALLN	0	1064400	8251	
Q9Y2W1	939	Thyroid hormone receptor-associated protein 3	THRAP3	0.999643	2.45E-182	377.88	S	HDKFSGEEGEIEDDESGTENREEKDNIQPTT	2432000	4918100	6820	
P24534	95	Elongation factor 1-beta	EEF1B2	0.999422	4.99E-182	370.35	S	GPADVEDTTGSGATDSKDDDDIDLFGSDDEE	4144400	5305600	1235	
Q9NTI5	1162	Sister chromatid cohesion protein PDS5 homolog B	PDS5B	0.954488	4.31E-182	369.77	S	TKSSRMETVSNASSSSNPSSPGRIKGRLDSS	1128500	1212100	6010	
Q9NTI5	1166	Sister chromatid cohesion protein PDS5 homolog B	PDS5B	0.999685	4.31E-182	369.77	S	RMETVSNASSSSNPSSPGRIKGRLDSSEMDH	9158700	5301600	6012	
Q16637	28	Survival motor neuron protein	SMN1	1	2.78E-159	367.05	S	PEQEDSVLFRRGTGQSDDSDIWDDTALIKAY	65612000	57722000	3024	
Q16637	31	Survival motor neuron protein	SMN1	1	2.78E-159	367.05	S	EDSVLFRRGTGQSDDSDIWDDTALIKAYDKA	45398000	39918000	3025	
Q8WVC0	14	RNA polymerase-associated protein LEO1	LEO1	1	9.95E-159	363.97	S	__MADMEDLFGSDADSEAERKDSDSGSDSDS	4661900	1822400	4537	
Q8N6T3	346	ADP-ribosylation factor GTPase-activating protein 1	ARFGAP1	0.495432	3.10E-155	361.52	S	TFGSAEPTKTRKSPSSDSWTCADTSTERRSS	0	0	4294	
Q2KHR3	1211	Glutamine and serine-rich protein 1	QSER1	0.997478	1.70E-162	359.08	S	SSTTPTPLVSETGGNSPSDKVDNELKNLEHL	3390600	2739900	3039	
Q9NU22	4538	Midasin	MDN1	1	1.32E-158	355.45	S	EERKNEKAEENTDQASPQEDYAGFERLQSGH	2463700	1728500	6018	
P22059	193	Oxysterol-binding protein 1	OSBP	1	1.48E-158	353.96	S	LAKAKAVKMLAESDESGDEESVSQTDKTELQ	806680	772860	1204	
Q92625	626	Ankyrin repeat and SAM domain-containing protein 1A	ANKS1A	0.8202	1.60E-158	352.77	S	SKPKAELKLSRSLSKSDSDLLTCSPTEDATM	4119600	4865300	4681	
Q9ULX3	201	RNA-binding protein NOB1	NOB1	1	2.18E-157	352.05	S	EEEEEENGFEDRKDDSDDDGGGWITPSNIKQ	8950600	7518900	6514	
Q9NYF8	385	Bcl-2-associated transcription factor 1	BCLAF1	1	9.65E-158	351.16	S	RAEGEWEDQEALDYFSDKESGKQKFNDSEGD	3283900	3571400	6101	
Q12778	287	Forkhead box protein O1	FOXO1	1	6.35E-141	350.58	S	KKKASLQSGQEGAGDSPGSQFSKWPASPGSH	1960500	1757000	2295	
Q13283	230	Ras GTPase-activating protein-binding protein 1	G3BP1	0.645668	5.77E-137	350.42	S	PEPVLEETAPEDAQKSSSPAPADIAQTVQED	683360	479430	2463	
Q13283	231	Ras GTPase-activating protein-binding protein 1	G3BP1	0.744041	5.77E-137	350.42	S	EPVLEETAPEDAQKSSSPAPADIAQTVQEDL	683360	479430	2464	
Q5JSZ5	225	Protein PRRC2B	PRRC2B	0.766443	7.25E-136	349.2	T	REGGGRHIISATSLSTSPTELGSRNSSTGDG	1161700	0	7705	
Q6IN85	117	Serine/threonine-protein phosphatase 4 regulatory subunit 3/	SMEK1	1	2.65E-136	348.33	S	GKDPSVDITQDLVDESEEERFDDMSSPGLEL	5903300	4632800	3474	
P40855	147	Peroxisomal biogenesis factor 19	PEX19	0.880587	4.68E-136	348.26	S	TLSGLAKNATDLQNSSMSEEELTKAMEGLGM	0	9669200	1479	
Q70Z53	278	Protein FRA10AC1	FRA10AC1	1	4.73E-133	346.63	S	GHSSSKKSEDSLLRNSDEEESASESELWKGP	2424800	1207800	3690	
Q9UQ35	416	Serine/arginine repetitive matrix protein 2	SRRM2	0.992226	4.33E-136	346.63	S	RDRSPPKSPEKLPQSSSSESSPPSPQPTKVS	0	0	6648	
Q13451	13	Peptidyl-prolyl cis-trans isomerase FKBP5;Peptidyl-prolyl ci	FKBP5	0.999977	1.22E-139	343.05	S	___MTTDEGAKNNEESPTATVAEQGEDITSK	516180	1040300	2536	
Q9UPP1	804	Histone lysine demethylase PHF8	PHF8	0.999793	1.54E-119	332.36	S	RSSGSSSSGLGTVSNSPASQRTPGKRPIKRP	756180	508670	6567	
P54727	160	UV excision repair protein RAD23 homolog B	RAD23B	0.999903	3.98E-120	332.29	S	QEKPAEKPAETPVATSPTATDSTSGDSSRSN	9326600	11915000	1855	
Q13263	19	Transcription intermediary factor 1-beta	TRIM28	0.996216	2.08E-131	331.47	S	SAAAASAAAASAASGSPGPGEGSAGGEKRST	4576800	5568600	2448	
Q9BPX3	674	Condensin complex subunit 3	NCAPG	1	1.35E-115	329.78	S	TKKIKTLHCEGTEINSDDEQESKEVEETATA	23881000	20446000	5293	
Q15424	24	Scaffold attachment factor B1	SAFB	0.957337	1.41E-130	329.21	S	GDSGAAGAAALSSASSETGTRRLSDLRVIDL	0	734950	2919	
Q9NTI5	1358	Sister chromatid cohesion protein PDS5 homolog B	PDS5B	0.999989	1.70E-115	328.73	S	SKQHRVSRRAQQRAESPESSAIESTQSTPQK	5200000	1536900	6003	
P38432	122	Coilin	COIL	1	2.28E-112	328.34	T	RKAKKRAFQLEEGEETEPDCKYSKKHWKSRE	2112800	1612300	7373	
P11388	1247	DNA topoisomerase 2-alpha	TOP2A	1	6.11E-116	328	S	EKKNKKKIKNENTEGSPQEDGVELEGLKQRL	3035200	4508100	1019	
Q9NY27	226	Serine/threonine-protein phosphatase 4 regulatory subunit 2	PPP4R2	0.999973	6.11E-116	328	S	NHSDSSTSESEVSSVSPLKNKHPDEDAVEAE	2615700	1831500	6088	
Q9Y3S2	291	Zinc finger protein 330	ZNF330	1	9.25E-115	327.02	S	EAEDDEEEDEGRKDSDTESSDLFTNLNLGR	1553600	1291200	6871	
Q12830	2465	Nucleosome-remodeling factor subunit BPTF	BPTF	1	8.13E-119	325.6	S	QVAAQSQPQSNVQGQSPVRVQSPSQTRIRPS	6872900	7143000	2313	
Q96N67	900	Dedicator of cytokinesis protein 7	DOCK7	1	5.53E-119	324.71	S	RPASLNLNRSRSLSNSNPDISGTPTSPDDEV	17699000	7479700	5034	
Q9UQ35	417	Serine/arginine repetitive matrix protein 2	SRRM2	0.999176	3.09E-115	324.45	S	DRSPPKSPEKLPQSSSSESSPPSPQPTKVSR	1860900	927770	6649	
O00566	163	U3 small nucleolar ribonucleoprotein protein MPP10	MPHOSPH10	1	4.50E-115	324.29	S	MGERAENSSKSDLRKSPVFSDEDSDLDFDIS	5508200	2215200	131	
O00566	171	U3 small nucleolar ribonucleoprotein protein MPP10	MPHOSPH10	1	4.50E-115	324.29	S	SKSDLRKSPVFSDEDSDLDFDISKLEQQSKV	3455900	1472800	133	
O75643	225	U5 small nuclear ribonucleoprotein 200 kDa helicase	SNRNP200	1	4.40E-113	323.16	S	EEGDEDVYGEVREEASDDDMEGDEAVVRCTL	3690100	4014100	649	
P67809	165	Nuclease-sensitive element-binding protein 1	YBX1	0.999977	3.00E-112	323.16	S	RRRGPPRNYQQNYQNSESGEKNEGSESAPEG	2169200	2020500	1937	
Q9NTI5	1283	Sister chromatid cohesion protein PDS5 homolog B	PDS5B	1	3.10E-114	321.67	S	KRLKEDILENEDEQNSPPKKGKRGRPPKPLG	4254200	2648900	6009	
Q9ULH0	1526	Kinase D-interacting substrate of 220 kDa	KIDINS220	0.998429	1.20E-118	321.67	S	GSGLRYQKLPSDEDESGTEESDNTPLLKDDK	1618500	1754800	6469	
P15336	69	Cyclic AMP-dependent transcription factor ATF-2	ATF2	0.999926	3.29E-112	320.5	T	KFGPARNDSVIVADQTPTPTRFLKNCEEVGL	0	0	7286	
P15336	71	Cyclic AMP-dependent transcription factor ATF-2	ATF2	0.980655	3.29E-112	320.5	T	GPARNDSVIVADQTPTPTRFLKNCEEVGLFN	352110	367220	7287	
Q15022	546	Polycomb protein SUZ12	SUZ12	1	4.52E-115	320.06	S	CRPKRTKASMSEFLESEDGEVEQQRTYSSGH	2962200	3473200	2857	
Q12926	221	ELAV-like protein 2	ELAVL2	1	1.39E-112	320	S	PSQKTNQAILSQLYQSPNRRYPGPLAQQAQR	1159400	930390	2379	
Q9UK76	87	Hematological and neurological expressed 1 protein;Hematolog	HN1	0.999679	3.88E-112	320	S	GGREDLESSGLQRRNSSEASSGDFLDLKGEG	52367000	40206000	6431	
Q9H3N1	247	Thioredoxin-related transmembrane protein 1	TMX1	1	1.62E-118	317.34	S	LKKVEEEQEADEEDVSEEEAESKEGTNKDFP	1214000	839250	5636	
P08238;Q5	255;177	Heat shock protein HSP 90-beta;Putative heat shock protein H	HSP90AB1;HS	1	3.11E-112	317.03	S	EDKDDEEKPKIEDVGSDEEDDSGKDKKKKTK	27489000	22399000	949	
P08708	113	40S ribosomal protein S17	RPS17	0.999959	4.12E-119	316.88	S	VDPDTKEMLKLLDFGSLSNLQVTQPTVGMNF	9243400	6105200	966	
P07900	263	Heat shock protein HSP 90-alpha	HSP90AA1	1	1.06E-98	316.84	S	EEKESEDKPEIEDVGSDEEEEKKDGDKKKKK	38585000	35208000	941	
P51991	355	Heterogeneous nuclear ribonucleoprotein A3	HNRNPA3	0.396328	1.27E-102	316.84	S	QSNYGPMKGGSFGGRSSGSPYGGGYGSGGGS	0	0	1808	
P51991	356	Heterogeneous nuclear ribonucleoprotein A3	HNRNPA3	0.396328	1.27E-102	316.84	S	SNYGPMKGGSFGGRSSGSPYGGGYGSGGGSG	0	0	1809	
Q9UBT2	207	SUMO-activating enzyme subunit 2	UBA2	1	3.32E-101	316.6	S	LFNQLFGEEDADQEVSPDRADPEAAWEPTEA	2922100	1619300	6273	
Q15276	407	Rab GTPase-binding effector protein 1	RABEP1	1	3.72E-96	316.58	S	KSDNDMFKDGLRRAQSTDSLGTSGSLQSKAL	10810000	6980500	2893	
Q9Y5T5	552	Ubiquitin carboxyl-terminal hydrolase 16	USP16	0.978025	4.93E-97	315.21	S	MKNINMDNDLEVLTSSPTRNLNGAYLTEGSN	1091200	1338700	6968	
Q16539	182	Mitogen-activated protein kinase 14	MAPK14	0.999794	3.34E-94	315.2	Y	LDFGLARHTDDEMTGYVATRWYRAPEIMLNW	0	435960	8383	
P62070	186	Ras-related protein R-Ras2	RRAS2	0.99626	1.32E-94	314.61	S	LVRVIRKFQEQECPPSPEPTRKEKDKKGCHC	15433000	13851000	1917	
Q96N67	896	Dedicator of cytokinesis protein 7	DOCK7	0.946045	2.08E-100	314.02	S	RSAVRPASLNLNRSRSLSNSNPDISGTPTSP	3803600	130390	5032	
Q96N67	898	Dedicator of cytokinesis protein 7	DOCK7	0.70485	2.08E-100	314.02	S	AVRPASLNLNRSRSLSNSNPDISGTPTSPDD	2086500	738200	5033	
P18858	141	DNA ligase 1	LIG1	1	9.53E-95	313.76	S	KQLPKRTIQEVLEEQSEDEDREAKRKKEEEE	3776800	2183100	1153	
Q86U70	302	LIM domain-binding protein 1	LDB1	0.999006	3.89E-100	313.59	S	EPTRQQPSKRRKRKMSGGSTMSSGGGNTNNS	2198700	1813500	3918	
Q5UIP0	1554	Telomere-associated protein RIF1	RIF1	0.938691	4.17E-100	313.36	S	RRASQGLLSSIENSESDSSEAKEEGSRKKRS	1246200	0	3312	
O60231	106	Putative pre-mRNA-splicing factor ATP-dependent RNA helicase	DHX16	1	1.09E-94	313.32	S	LEKNRSYRLLEDSEESSEETVSRAGSSLQKK	3315500	4274200	438	
P35613	362	Basigin	BSG	1	6.48E-96	311.54	S	KRRKPEDVLDDDDAGSAPLKSSGQHQNDKGK	3283700	1994000	1415	
Q86YP4	100	Transcriptional repressor p66-alpha	GATAD2A	1	3.07E-99	311.46	S	MRTSHSDMKSERRPPSPDVIVLSDNEQPSSP	46743000	39234000	4022	
Q86YP4	114	Transcriptional repressor p66-alpha	GATAD2A	0.975693	3.07E-99	311.46	S	PSPDVIVLSDNEQPSSPRVNGLTTVALKETS	46743000	39234000	4025	
Q9UQ88;P2	271;283	Cyclin-dependent kinase 11A;Cyclin-dependent kinase 11B	CDK11A;CDK1	0.999928	3.91E-94	310.17	S	EKMEERDLLSDLQDISDSERKTSSAESSSAE	18433000	21458000	1192	
Q5JSZ5	226	Protein PRRC2B	PRRC2B	0.886593	1.39E-96	308.78	S	EGGGRHIISATSLSTSPTELGSRNSSTGDGA	1361700	1044900	3191	
P55795;P3	104;104	Heterogeneous nuclear ribonucleoprotein H2;Heterogeneous nuc	HNRNPH2;HNI	1	1.06E-94	308.42	S	NSVEMDWVLKHTGPNSPDTANDGFVRLRGLP	37665000	44974000	1360	
Q9P2B4	481	CTTNBP2 N-terminal-like protein	CTTNBP2NL	1	1.14E-99	307.25	S	FQSQADQDQQASGLQSPPSRDLSPTLIDNSA	9856600	5717200	6228	
P51991	358	Heterogeneous nuclear ribonucleoprotein A3	HNRNPA3	0.649955	3.13E-100	306.2	S	YGPMKGGSFGGRSSGSPYGGGYGSGGGSGGY	5102300	0	1810	
Q8WUM0	45	Nuclear pore complex protein Nup133	NUP133	0.996324	4.92E-97	305.98	S	RTASRKGLPLGSAVSSPVLFSPVGRRSSLSS	1649800	1374900	4519	
Q8WUM0	50	Nuclear pore complex protein Nup133	NUP133	1	4.92E-97	305.98	S	KGLPLGSAVSSPVLFSPVGRRSSLSSRGTPT	1443200	1194600	4520	
P25788	250	Proteasome subunit alpha type-3	PSMA3	1	3.09E-93	305.14	S	EAEKYAKESLKEEDESDDDNM__________	11232000	5947100	1272	
O60841	182	Eukaryotic translation initiation factor 5B	EIF5B	1	2.06E-96	304.91	S	DEDNSKKIKERSRINSSGESGDESDEFLQSR	3242000	138560	529	
Q8WWM7	594	Ataxin-2-like protein	ATXN2L	1	2.31E-96	303.45	S	KEKEVDGLLTSEPMGSPVSSKTESVSDKEDK	5811900	3173800	4561	
Q12888	294	Tumor suppressor p53-binding protein 1	TP53BP1	1	7.36E-97	303.34	S	LSAQELMESGLQIQKSPEPEVLSTQEDLFDQ	7334900	7368100	2367	
Q7Z460	646	CLIP-associating protein 1	CLASP1	0.999972	1.64E-95	303.34	S	PGSYASLGRIRTRRQSSGSATNVASTPDNRG	1701300	999370	3809	
Q16643	337	Drebrin	DBN1	0.999718	2.37E-96	303.07	S	LDSHRRMAPTPIPTRSPSDSSTASTPVAEQI	30904000	911230	3027	
Q16643	339	Drebrin	DBN1	0.999845	2.37E-96	303.07	S	SHRRMAPTPIPTRSPSDSSTASTPVAEQIER	0	13875000	3028	
O14974	299	Protein phosphatase 1 regulatory subunit 12A	PPP1R12A	1	4.13E-96	302.9	S	QKKQNLLHSEKRDKKSPLIESTANMDNNQSQ	1843600	1592900	220	
O15015	930	Zinc finger protein 646	ZNF646	1	4.13E-96	302.9	S	EEGEEEGVAEAAPARSPPLQLSEAELLNQLQ	1029800	598350	236	
Q8NHW5;P0	304;304	60S acidic ribosomal protein P0-like;60S acidic ribosomal pr	RPLP0P6;RPI	1	1.02E-50	302.85	S	PAAAAAPAKVEAKEESEESDEDMGFGLFD__	0	0	900	
Q8NHW5;P0	307;307	60S acidic ribosomal protein P0-like;60S acidic ribosomal pr	RPLP0P6;RPI	1	1.02E-50	302.85	S	AAAPAKVEAKEESEESDEDMGFGLFD_____	0	0	901	
Q96N67	907	Dedicator of cytokinesis protein 7	DOCK7	0.997922	8.08E-99	302.61	T	NRSRSLSNSNPDISGTPTSPDDEVRSIIGSK	4634400	0	7994	
P49736	13	DNA replication licensing factor MCM2	MCM2	0.999958	1.04E-94	302.35	S	___MAESSESFTMASSPAQRRRGNDPLTSSP	2177900	1795400	1676	
Q12888	552	Tumor suppressor p53-binding protein 1	TP53BP1	1	3.70E-104	301.93	S	DEDGENTQIEDTEPMSPVLNSKFVPAENDSI	938320	331730	2344	
O60231	103	Putative pre-mRNA-splicing factor ATP-dependent RNA helicase	DHX16	1	4.03E-94	301.04	S	RALLEKNRSYRLLEDSEESSEETVSRAGSSL	2602200	4274200	437	
P46821	1501	Microtubule-associated protein 1B;MAP1B heavy chain;MAP1 lig	MAP1B	0.999985	2.81E-96	300.5	S	SQPALALDERKLGDVSPTQIDVSQFGSFKED	4467800	5291500	1586	
Q6UN15	492	Pre-mRNA 3'-end-processing factor FIP1	FIP1L1	0.999948	4.16E-94	300.5	S	RERERTRERERERDHSPTPSVFNSDEERYRY	20306000	16058000	3586	
Q6UN15	500	Pre-mRNA 3'-end-processing factor FIP1	FIP1L1	1	4.16E-94	300.5	S	RERERDHSPTPSVFNSDEERYRYREYAERGY	13238000	9238100	3588	
P13861	78	cAMP-dependent protein kinase type II-alpha regulatory subur	PRKAR2A	1	2.37E-96	300.29	S	PPEPGPDRVADAKGDSESEEDEDLEVPVPSR	54948000	43023000	1056	
P13861	80	cAMP-dependent protein kinase type II-alpha regulatory subur	PRKAR2A	1	2.37E-96	300.29	S	EPGPDRVADAKGDSESEEDEDLEVPVPSRFN	58699000	42555000	1057	
P18858	76	DNA ligase 1	LIG1	1	4.91E-96	300.29	S	ARVLGSEGEEEDEALSPAKGQKPALDCSQVS	1511700	3261800	1155	
Q96CX2	187	BTB/POZ domain-containing protein KCTD12	KCTD12	0.900313	1.03E-99	299.9	S	AGAPSPTLELASRSPSGGAAGPLLTPSQSLD	24674000	33808000	4834	
Q96CX2	198	BTB/POZ domain-containing protein KCTD12	KCTD12	0.938389	1.03E-99	299.9	S	SRSPSGGAAGPLLTPSQSLDGSRRSGYITIG	318250	1219700	4835	
Q13439	40	Golgin subfamily A member 4	GOLGA4	0.446823	1.36E-78	299.64	S	ASSNSSTPTRMRSRTSSFTEQLDEGTPNRES	0	0	2531	
Q13439	39	Golgin subfamily A member 4	GOLGA4	0.446823	1.36E-78	299.64	T	QASSNSSTPTRMRSRTSSFTEQLDEGTPNRE	0	0	7543	
Q9H8G2	89	Caspase activity and apoptosis inhibitor 1	CAAP1	0.499998	3.92E-79	299.31	S	WGGSSVERSERRKRRSTDSSSVSGSLQQETK	2984300	0	5785	
Q9H8G2	90	Caspase activity and apoptosis inhibitor 1	CAAP1	0.499998	3.92E-79	299.31	T	GGSSVERSERRKRRSTDSSSVSGSLQQETKY	2984300	0	8122	
O60841	190	Eukaryotic translation initiation factor 5B	EIF5B	1	1.49E-80	298.96	S	KERSRINSSGESGDESDEFLQSRKGQKKNQK	4031700	308440	532	
P10644	83	cAMP-dependent protein kinase type I-alpha regulatory subuni	PRKAR1A	1	3.02E-78	298.78	S	LQKAGTRTDSREDEISPPPPNPVVKGRRRRG	14265000	7245100	1001	
P46013	1375	Antigen KI-67	MKI67	0.521647	3.56E-80	298.1	S	EEAVAAGKTTKMPCESSPPESADTPTSTRRQ	0	0	1532	
Q96GM8	5	Target of EGR1 protein 1	TOE1	1	2.65E-83	298	S	___________MAADSDDGAVSAPAASDGGV	1267900	1375700	4917	
Q01804	1023	OTU domain-containing protein 4	OTUD4	1	3.62E-78	297.45	S	GANSVDSRVQRPKEESSEDENEVSNILRSGR	3924100	4589800	2074	
Q01804	1024	OTU domain-containing protein 4	OTUD4	1	3.62E-78	297.45	S	ANSVDSRVQRPKEESSEDENEVSNILRSGRS	5030600	5311700	2075	
Q96K21	354	Abscission/NoCut checkpoint regulator	ZFYVE19	1	2.20E-78	296.95	S	QDPERVTLQDYRLPDSDDDEDEETAIQRVLQ	318000	367870	5002	
Q9H8G2	203	Caspase activity and apoptosis inhibitor 1	CAAP1	1	1.16E-54	295.86	S	EKKILKILEGDNGMDSDMEEEADDGSKMGSD	0	0	5784	
Q00341	31	Vigilin	HDLBP	0.999999	3.06E-78	295.82	S	RSGLVPQQIKVATLNSEEESDPPTYKDAFPP	2299000	1194200	2021	
Q9UIG0	1468	Tyrosine-protein kinase BAZ1B	BAZ1B	1	1.74E-79	294.93	S	KRKKFPDRLAEDEGDSEPEAVGQSRGRRQKK	921050	1514300	6384	
P35221;P2	655;654	Catenin alpha-1;Catenin alpha-2	CTNNA1;CTNI	0.982379	7.23E-79	294.85	S	DSDFETEDFDVRSRTSVQTEDDQLIAGQSAR	6164600	1869000	1376	
P35221;P2	654;653	Catenin alpha-1;Catenin alpha-2	CTNNA1;CTNI	0.85803	7.23E-79	294.85	T	DDSDFETEDFDVRSRTSVQTEDDQLIAGQSA	14328000	7600200	7356	
P18583	1556	Protein SON	SON	0.999564	3.24E-83	294.28	S	IAKEMEHNTVCAAGTSPVGEIGEEKILPTSE	15912000	10310000	1133	
Q5JTV8	156	Torsin-1A-interacting protein 1	TOR1AIP1	0.999948	1.81E-53	293.63	S	QPSPVMTRRGLRDSHSSEEDEASSQTDLSQT	0	0	3199	
Q5JTV8	157	Torsin-1A-interacting protein 1	TOR1AIP1	0.999922	1.81E-53	293.63	S	PSPVMTRRGLRDSHSSEEDEASSQTDLSQTI	0	0	3200	
Q9NTI5	1370	Sister chromatid cohesion protein PDS5 homolog B	PDS5B	0.98749	9.98E-79	293.63	T	RAESPESSAIESTQSTPQKGRGRPSKTPSPS	0	1536900	8161	
Q9UQ35	1539	Serine/arginine repetitive matrix protein 2	SRRM2	1	6.69E-79	292.65	S	DQKTVARTPLGQRSRSGSSQELDVKPSASPQ	3832800	2544900	6687	
Q9UQ35	1541	Serine/arginine repetitive matrix protein 2	SRRM2	1	6.69E-79	292.65	S	KTVARTPLGQRSRSGSSQELDVKPSASPQER	6722200	12799000	6688	
Q9UQ35	1542	Serine/arginine repetitive matrix protein 2	SRRM2	1	6.69E-79	292.65	S	TVARTPLGQRSRSGSSQELDVKPSASPQERS	2877000	2600800	6689	
P27824	564	Calnexin	CANX	0.780488	2.66E-79	292.35	S	EEKQKSDAEEDGGTVSQEEEDRKPKAEEDEI	614550	287660	1301	
Q9BUL5	74	PHD finger protein 23	PHF23	1	1.58E-79	292.2	S	ASGSSSPLRGESAADSDGWDSAPSDLRTIQT	1435000	1819400	5383	
Q9NTI5	1257	Sister chromatid cohesion protein PDS5 homolog B	PDS5B	0.999908	5.84E-78	292.19	S	KPKGSQRSRKRGHTASESDEQQWPEEKRLKE	0	633000	6006	
Q9C0E2	521	Exportin-4	XPO4	0.999937	2.78E-79	292.02	S	RLHGQLQRHQQQLLASPGSSTVDNKMLDDLY	3265900	1098500	5541	
Q9UHD8	238	Septin-9	SEPT9	0.999995	1.85E-82	291.99	S	LEPKPQPPVAEATPRSQEATEAAPSCVGDMA	93566	426910	6350	
O60502	364	Protein O-GlcNAcase	MGEA5	1	9.30E-87	291.78	S	EDSTVSIQIKLENEGSDEDIETDVLYSPQMA	3880400	2597000	502	
Q9Y608	328	Leucine-rich repeat flightless-interacting protein 2	LRRFIP2	0.999688	5.34E-80	291.68	S	ASATTPLSGNSSRRGSGDTSSLIDPDTSLSE	5317600	5029400	6972	
A0MZ66	494	Shootin-1	KIAA1598	0.955877	1.96E-82	291.51	S	RILRRRKVTAEADSSSPTGILATSESKSMPV	1266300	731290	10	
Q02952	598	A-kinase anchor protein 12	AKAP12	0.998857	1.21E-82	291.49	S	LAEVQQDGEAEEGATSDGEKKREGVTPWASF	808160	2196900	2117	
Q8N6T3	343	ADP-ribosylation factor GTPase-activating protein 1	ARFGAP1	0.999709	6.66E-78	291.13	S	FWETFGSAEPTKTRKSPSSDSWTCADTSTER	1586700	0	4293	
Q01082	2341	Spectrin beta chain, non-erythrocytic 1	SPTBN1	0.998468	6.00E-80	290.67	S	AQTLPTSVVTITSESSPGKREKDKEKDKEKR	11402000	8420300	2041	
P08238;Q5	226;205	Heat shock protein HSP 90-beta;Putative heat shock protein H	HSP90AB1;HS	1	1.65E-76	290.38	S	YPITLYLEKEREKEISDDEAEEEKGEKEEED	0	1136500	948	

P18858	66	DNA ligase 1	LIG1	1	2.89E-79	290.09	S	SPVKRPGRKAARVLGSEGEEEDEALSPAKGQ	869640	2527000	1154
P24534	106	Elongation factor 1-beta	EEF1B2	1	2.22E-42	289.68	S	GATDSKDDDDIDLFGSDDEEESEEAKRLREE	0	0	1233
Q7Z6E9	1328	E3 ubiquitin-protein ligase RBBP6	RBBP6	1	1.34E-76	289.59	S	MIQVPQSKWDKDDFESEEEDVKSTQPISSVG	1435600	1477000	3859
Q01082	2128	Spectrin beta chain, non-erythrocytic 1	SPTBN1	1	3.65E-79	289.58	S	AESQQQWDTSKGEQVSQNGLPAEQGSPRMAE	1517800	1035200	2045
Q9UQ80	2	Proliferation-associated protein 2G4	PA2G4	1	3.04E-80	288.96	S	______________MSGEDEQQEQTIAEDLV	58104000	55371000	6752
Q96AC1	351	Fermitin family homolog 2	FERMT2	1	2.65E-82	288.57	S	LNNSDKEVDEVDAALSDLEITLEGGKTSTIL	375070	461340	4771
P46821	1378	Microtubule-associated protein 1B;MAP1B heavy chain;MAP1 li	MAP1B	0.996393	7.49E-80	288.41	S	FEFSDAKDENERASVSPMDEPVPDSESPIEK	2438900	1863600	1571
Q9UQ35	421	Serine/arginine repetitive matrix protein 2	SRRM2	0.965745	2.39E-79	288.3	S	PKSPEKLPQSSSSESSPPSPQPTKVSRHASS	0	0	6651
Q96N67	910	Dedicator of cytokinesis protein 7	DOCK7	0.860408	1.42E-82	288.02	S	RSLSNSNPDISGTPTSPDDEVRSIIGSKGLD	0	0	5035
Q9Y6G9	207	Cytoplasmic dynein 1 light intermediate chain 1	DYNC1LI1	1	2.50E-79	287.75	S	RDFQEYVEPGEDFPASPQRRNTASQEDKDDS	18404000	16399000	7004
Q9NYL9	25	Tropomodulin-3	TMOD3	0.978302	4.40E-79	287.48	S	EKYKDLDEDELLGNLSETELKQLETVLDDLD	0	0	6121
Q9Y3T9	672	Nucleolar complex protein 2 homolog	NOC2L	1	8.10E-80	287.48	S	KDEDRKQFKDLFDLNSSEEDDTEGFSERGIL	9997500	4268000	6873
Q13428	983	Treacle protein	TCOF1	0.996208	3.35E-79	287.31	T	PAGPAATPAQAQAASTPRKARASESTARSSS	1661600	765870	7538
Q15276	410	Rab GTPase-binding effector protein 1	RABEP1	0.999944	8.16E-78	287	S	NDMFKDGLRRAQSTDSLGTSGSLQSKALGYN	5827700	3867000	2894
Q96F86	131	Enhancer of mRNA-decapping protein 3	EDC3	1	2.35E-77	287	S	NIPKRTDVKSQDVAVSPQQQQCSKSYVDRHM	1682500	1067100	4903
Q15007	306	Pre-mRNA-splicing regulator WTAP	WTAP	0.999955	2.69E-79	286.85	S	GFHREGNTTEDDFPSSPGNGNKSSNSSEERT	2742900	3967900	2852
Q8NHQ9	544	ATP-dependent RNA helicase DDX55	DDX55	1	8.51E-80	286.85	S	EKKKKMNEKRKREEGSDIEDEDMEELLNDTR	1256700	1249300	4418
Q01082	2138	Spectrin beta chain, non-erythrocytic 1	SPTBN1	1	4.94E-79	285.96	S	KGEQVSQNGLPAEQGSPRMAETVDTSEMVNG	9009000	6270600	2046
O95999	134	B-cell lymphoma/leukemia 10	BCL10	1	5.26E-79	285.06	S	CSSCEPFPDGATNNLSRSNSDESNFSEKLRA	1468300	505300	842
P52292	62	Importin subunit alpha-1	KPNA2	0.997956	5.26E-79	285.06	S	MLKRRNVSSFPDDATSPLQENRNNQGTVNWS	22588000	21975000	1811
P98175	738	RNA-binding protein 10	RBM10	1	3.10E-79	284.84	S	SPPRGLVAAYSGESDSEEEQERGGPEREEKL	12865000	2028400	2016
O75448	873	Mediator of RNA polymerase II transcription subunit 24	MED24	0.998726	3.17E-79	284.51	S	MRLLSSNEDDANILSSPTDRSMSSSLSASQL	1147900	2937000	631
Q99700	889	Ataxin-2	ATXN2	0.999281	5.56E-79	284.24	S	HKRGPEVTSQGVQTSSPACKQEKDDKEEKKD	7566100	4157300	5257
Q9UQ35	420	Serine/arginine repetitive matrix protein 2	SRRM2	0.920491	3.35E-79	283.64	S	PPKSPEKLPQSSSSESSPPSPQPTKVSRHAS	1361900	689210	6650
Q8NDC0	15	MAPK-interacting and spindle-stabilizing protein-like	MAPK1IP1L	1	2.48E-79	283.47	S	_MSDEFSLADALPEHSPAKTSAVSNTKPGQP	9920000	8129800	4346
Q96EZ8	282	Microspherule protein 1	MCRS1	1	3.44E-79	283.19	S	QTVQPLPKGDQVLNFSDAEDLIDDSKLKDMR	2107900	1124600	4895
Q8WVM8	303	Sec1 family domain-containing protein 1	SCFD1	1	6.01E-79	282.97	S	HLNRVNLEESSGVENSPAGARPKRKNKKSYD	5709400	3684900	4543
Q9Y2L5	273	Trafficking protein particle complex subunit 8	TRAPPC8	1	6.01E-79	282.97	S	ESYEDGPCTITSNKNSDNNLLSLDGLDNEVK	1333800	743140	6795
Q6A1A2;O	214;241	Putative 3-phosphoinositide-dependent protein kinase 2;3-pho	PDPK2P;PDPK	1	5.05E-66	282.41	S	TAKVLSPESKQARANSFVGTAQYVSPELLTE	2641500	2259700	324
Q01082	2320	Spectrin beta chain, non-erythrocytic 1	SPTBN1	0.978344	5.32E-63	281.99	T	AISSDKHEVSASTQSTPASSRAQTLPTSVVT	856110	499890	7473
O00193	15	Small acidic protein	SMAP	1	1.19E-56	281.95	S	_MSAARESHPHGVKRSASPDDDLGSSNWEAA	68460000	28016000	104
O00193	17	Small acidic protein	SMAP	1	1.19E-56	281.95	S	SAARESHPHGVKRSASPDDDLGSSNWEAADL	68460000	28016000	105
O60832	494	H/ACA ribonucleoprotein complex subunit 4	DKC1	0.996709	7.50E-65	281.09	S	AKAGLESGAEPGDGDSDTTKKKKKKKKAKEV	1127000	2675800	525
Q9NYL9	27	Tropomodulin-3	TMOD3	0.964267	5.26E-64	281.09	T	YKDLDEDELLGNLSETELKQLETVLDDLDPE	1809300	2139300	8178
Q8N3U4	1061	Cohesin subunit SA-2	STAG2	0.999995	1.55E-67	280.2	S	NSLLAGGDDDTMSVISGISSRGSTVRSKKSK	1043800	794830	4258
Q8N122	863	Regulatory-associated protein of mTOR	RPTOR	0.999946	1.23E-64	280.01	S	QRVLDTSSLTQSAPASPTNKGVHIHQAGGSP	22346000	18996000	4211
Q9BWW4	347	Single-stranded DNA-binding protein 3	SSBP3	1	2.12E-64	280.01	S	NGSLGSGDIDGLPKNSPNNISGISNPPGTPR	10073000	5911200	5440
P49006	22	MARCKS-related protein	MARCKSL1	1	4.81E-63	279.91	S	KAPRGDVTAEEAAGASPAKANGQENGHVKSN	2675300	5483900	1638
O75400	933	Pre-mRNA-processing factor 40 homolog A	PRPF40A	0.614506	3.43E-65	279.86	S	SPKKKTGKDSGNWDTSGSELSEGELEKRRRT	0	2253900	623
O75400	938	Pre-mRNA-processing factor 40 homolog A	PRPF40A	0.999992	3.43E-65	279.86	S	TGKDSGNWDTSGSELSEGELEKRRRTLLEQL	6481900	4018200	624
Q9H1B7	657	Interferon regulatory factor 2-binding protein-like	IRF2BPL	0.999958	3.19E-63	279.5	S	DGSSVHSTTASARRNSSSPVSPASVPGQRRL	11776000	2994100	5595
Q9H1B7	659	Interferon regulatory factor 2-binding protein-like	IRF2BPL	0.996793	3.19E-63	279.5	S	SSVHSTTASARRNSSSPVSPASVPGQRRLAS	5354400	2994100	5597
Q9H1B7	662	Interferon regulatory factor 2-binding protein-like	IRF2BPL	0.999957	3.19E-63	279.5	S	HSTTASARRNSSSPVSPASVPGQRRLASRNG	1675900	3290200	5598
P55209	62	Nucleosome assembly protein 1-like 1	NAP1L1	1	1.04E-63	279.44	T	QILAALQERLDGLVETPTGYIESLPRVVKRR	2110900	2164400	7451
P05412	62	Transcription factor AP-1	JUN	0.993318	3.48E-62	279.19	T	GSLKPHLRAKNSDLLTSPDVGLLKLASPELE	6170400	4549600	7262
O14745	288	Na(+)/H(+) exchange regulatory cofactor NHE-RF1	SLC9A3R1	0.47771	1.63E-64	279.12	S	LAEAALESPRPALVRSASSDTSEELNSQDSP	0	0	206
O14745	290	Na(+)/H(+) exchange regulatory cofactor NHE-RF1	SLC9A3R1	0.47771	1.63E-64	279.12	S	EAALESPRPALVRSASSDTSEELNSQDSPPK	0	0	207
Q99543	60	DnaJ homolog subfamily C member 2;DnaJ homolog subfamily C m	DNAJC2	1	2.99E-67	279.12	S	NASASFQELEDKKELSEESEDEELQLEEFPM	8585400	9128000	5200
Q99543	63	DnaJ homolog subfamily C member 2;DnaJ homolog subfamily C m	DNAJC2	1	2.99E-67	279.12	S	ASFQELEDKKELSEESEDEELQLEEFPMLKT	8137700	9238200	5201
Q9BTK6	237	PAXIP1-associated glutamate-rich protein 1	PAGR1	0.999981	1.63E-64	279.12	S	GRDLFSLDSEDPSPASPPLRSSGSSLFPRQR	3283300	2072200	5365
Q7Z6Z7	3662	E3 ubiquitin-protein ligase HUWE1	HUWE1	0.999999	3.61E-67	279.1	S	EYNLEQQRRAQCETLSPDGLPEEQPQTTKLK	667190	656770	3868
C9JLW8	21	Protein FAM195B	FAM195B	0.99957	1.73E-64	278.89	S	VSRVVYNGKRTSSPRSPPSSSEIFTPAHEEN	41459000	11459000	74
P52948	623	Nuclear pore complex protein Nup98-Nup96;Nuclear pore comple	NUP98	0.999986	2.99E-64	278.88	S	SNLFSPVNRDSENLASPSEYPENGERFSFLS	2378300	1921100	1824
Q92625	628	Ankyrin repeat and SAM domain-containing protein 1A	ANKS1A	0.5	5.64E-65	278.79	S	PKAELKLSRSLSKSDSDLLTCSPTEDATMGS	0	4865300	4682
P49736	139	DNA replication licensing factor MCM2	MCM2	1	1.37E-62	278.62	S	AGRGLGRMRRGLLYDSDEEDEERPARKRRQV	1269500	1295100	1679
P08621	410	U1 small nuclear ribonucleoprotein 70 kDa	SNRNP70	1	1.99E-64	278.32	S	GGGGQDNGLEGLGNDSRDMYMESEGGDGYLA	19263000	13248000	958
Q8NDI1	171	EH domain-binding protein 1	EHBP1	1	2.86E-63	278.07	S	CIFLREGKATDEDMQSLASLMSMKQADIGNL	3036700	1633100	4349
Q8NDI1	174	EH domain-binding protein 1	EHBP1	1	2.86E-63	278.07	S	LREGKATDEDMQSLASLMSMKQADIGNLDDF	3036700	1633100	4350
Q99549	136	M-phase phosphoprotein 8	MPHOSPH8	1	2.78E-67	277.25	S	DIQRLSLNNDIFEANSDSDQQSETKEDTSPK	2769800	2218000	5206
Q99549	138	M-phase phosphoprotein 8	MPHOSPH8	1	2.78E-67	277.25	S	QRLSLNNDIFEANSDSDQQSETKEDTSPKKK	2769800	2218000	5207
Q96CX2	185	BTB/POZ domain-containing protein KCTD12	KCTD12	0.815023	3.45E-67	277.07	S	ASAGAPSPTLELASRSPSGGAAGPLLTPSQS	16620000	22618000	4833
P46937	61	Transcriptional coactivator YAP1	YAP1	0.995127	2.96E-64	276.14	S	QAPPAGHQIVHVRGDSETDLEALFNAVMNPK	309820	186190	1613
Q9UQ35	876	Serine/arginine repetitive matrix protein 2	SRRM2	1	2.02E-62	274.8	S	NEQSVTPQRRSCFESSPDPELKSRTPSRHSC	34544000	28142000	6683
Q9NVF7	344	F-box only protein 28	FBXO28	1	4.90E-67	274.68	S	ESAVGNSSGSGQNEESPRKRKKATEAIDSLR	2728700	1690700	6039
P07900	231	Heat shock protein HSP 90-alpha	HSP90AA1	1	1.02E-61	273.84	S	YPITLFVEKERDKEVSDDEAEEKEDKEEEKE	680760	761400	940
O43823	328	A-kinase anchor protein 8	AKAP8	1	4.01E-64	273.8	S	EPDTKLARVDSEGDFSENDDAAGDFRSGDEE	1121100	1933700	431
P35221	641	Catenin alpha-1	CTNNA1	1	4.02E-64	273.77	S	RKAVLMIRTPEELDDSDFETEDFDVRSRTSV	7824600	5590100	1377
Q13428	679	Treacle protein	TCOF1	0.998588	8.87E-67	273.77	S	QAPRKAGTATSPAGSSPAVAGGTQRPAEDSS	1717000	2049400	2506
Q9Y6G9	516	Cytoplasmic dynein 1 light intermediate chain 1	DYNC1LI1	0.999803	5.38E-64	273.54	S	RITRKPVTVSPTTPTSPTEGEAS________	44993000	52060000	7006
Q15435	12	Protein phosphatase 1 regulatory subunit 7	PPP1R7	1	1.56E-62	273.52	S	____MAAERGAGQQQSQEMMEVDRRVESEES	1599000	6220300	2933
Q5H9R7	617	Serine/threonine-protein phosphatase 6 regulatory subunit 3	PPP6R3	1	4.13E-64	273.52	S	EACCKERIQQFDDGGSDEEDIWEEKHIAFTP	36674000	21041000	3166
Q6QNY0	27	Biogenesis of lysosome-related organelles complex 1 subunit	BLOC1S3	1	6.31E-63	273.01	S	RRPETVVPGEATETDSERSASSSEEEELYLG	9811600	4607900	3573
Q9HCK8	550	Chromodomain-helicase-DNA-binding protein 8	CHD8	0.914395	3.63E-64	273.01	S	TITPVVGKKRKRNTSSDNSDVEVMPAQSPRE	2400900	1304300	5866
Q9HCK8	553	Chromodomain-helicase-DNA-binding protein 8	CHD8	0.881752	3.63E-64	273.01	S	PVVGKKRKRNTSSDNSDVEVMPAQSPREDEE	2400900	0	5867
Q6QNY0	25	Biogenesis of lysosome-related organelles complex 1 subunit	BLOC1S3	0.99995	6.31E-63	273.01	T	PLRRPETVVPGEATETDSERSASSSEEEELY	8775600	4286400	7768
Q8NC51	25	Plasminogen activator inhibitor 1 RNA-binding protein	SERBP1	1	8.08E-63	271.73	S	GCVVTNRFDQLFDDESDPFEVLKAAENKKKE	3028500	2396800	4320
Q5VT52	374	Regulation of nuclear pre-mRNA domain-containing protein 2	RPRD2	1	8.28E-63	271.45	S	PEPVTDNRDVEDMELSDVEDDGSKIIVEDRK	547030	1060800	3329
Q9UJK0	228	Ribosome biogenesis protein TSR3 homolog	TSR3	1	8.84E-64	271.27	S	EVLQAEQEFLANAKESPQEEEIDPFDVDSGR	508880	888940	6402
Q9UQ35	1404	Serine/arginine repetitive matrix protein 2	SRRM2	0.99936	1.63E-64	271.27	S	DAVEKAGMSSNQSISSPVLDAVPRTPSRERS	6188500	6092500	6600
Q9Y5A9	39	YTH domain-containing family protein 2	YTHDF2	1	8.84E-64	271.27	S	HQKDGLNDDDFEPYLSPQARPNNAYTAMSDS	764340	1311200	6955
Q9NRF2	125	SH2B adapter protein 1	SH2B1	0.5	2.82E-67	271.2	S	SCRVGGPLAVLGPSRSSEDLAGPLPSSVSSS	1470600	525100	5958
Q9NRF2	126	SH2B adapter protein 1	SH2B1	0.5	2.82E-67	271.2	S	CRVGGPLAVLGPSRSSEDLAGPLPSSVSSSS	1470600	525100	5959
P40855	146	Peroxisomal biogenesis factor 19	PEX19	0.740625	9.09E-64	270.95	S	ETLSGLAKNATDLQNSSMSEEELTKAMEGLG	3043600	1147900	1478
P52948	888	Nuclear pore complex protein Nup98-Nup96;Nuclear pore comple	NUP98	1	3.69E-75	270.95	S	WVFKVSHFSKYGLQDSDEEEEEHPSKTSTKK	2170800	3506400	1830
Q9NR30	121	Nucleolar RNA helicase 2	DDX21	1	8.96E-62	270.95	S	VVSSKTKKVTKNEEPSEEEIDAPKPKKMKKE	2918900	6812300	5946
Q9UQ35	142	Serine/arginine repetitive matrix protein 2	SRRM2	0.999994	1.68E-64	270.95	S	LNEKKNERLRAAFGISDSYVDGSSFDPQRRA	664690	0	6594
Q8WWQ0	1783	PH-interacting protein	PHIP	1	2.86E-62	270.79	S	TRNQGRRTAFYNEDDSEEEQRQLLFEDTSLT	831220	0	4571
Q9C0B0	385	RING finger protein unkempt homolog	UNK	0.999998	1.71E-64	270.73	S	LCRNSSLGSPSNLCGSPPGSIRKPPNLEGIV	8240600	7247700	5509
O00264	57	Membrane-associated progesterone receptor component 1	PGRMC1	0.999987	1.22E-66	270.37	S	LYKIVRGDQPAASGDSDDDEPPPLPRLKRRD	35154000	22998000	113
Q6F5E8	1315	Leucine-rich repeat-containing protein 16C	RLTPR	1	7.52E-67	270.37	S	LRSRGWGQQDGPGPPSPGQSPSPCRTSPSPD	3868000	2774500	3454
Q92882	213	Osteoclast-stimulating factor 1	OSTF1	1	2.17E-62	269.87	S	VRTLSNAEDYLDDEDSD______________	5065300	6122600	4724
P63104	207	14-3-3 protein zeta/delta	YWHAZ	0.999986	1.91E-64	269.34	S	AKTAFDEAIAELDTLSEESYKDSTLIMQLLR	660280	407570	1935
Q6Y7W6	382	PERQ amino acid-rich with GYF domain-containing protein 2	GIGYF2	0.999987	4.50E-63	268.42	T	SEETPQTSSSSARPGTPSDHQSQEASQFERK	32650000	22394000	7778
Q9H8Y8	449	Golgi reassembly-stacking protein 2	GORASP2	0.695431	7.92E-70	268.31	S	PVSEKPVSAAVDANASESP____________	915490	0	5795
Q9H8Y8	433	Golgi reassembly-stacking protein 2	GORASP2	0.798095	7.92E-70	268.31	T	TAKAPTTVEDRVGDSTPVSEKPVSAAVDANA	915490	0	8125
O60841	135	Eukaryotic translation initiation factor 5B	EIF5B	0.999468	4.91E-49	268.18	S	DSKSKKTAKPKVEMYSGSDDDDDFNKLPKKA	15542000	9958000	535
Q9NYF8	196	Bcl-2-associated transcription factor 1	BCLAF1	0.997957	1.07E-62	268.18	S	SKSQEEPKDTFEHDPSESIDEFNKSSATSGD	0	0	6104
Q96RS0	89	Trimethylguanosine synthase	TGS1	1	1.12E-63	268.17	S	SHDSKGIGLDESELDSEAELMRSMGLPLQFG	1898300	686580	5100
O95218	188	Zinc finger Ran-binding domain-containing protein 2	ZRANB2	1	1.43E-61	268.13	S	EDEDDADLSKYNLDASEEEDSNKKKSNRRSR	14593000	12582000	741
Q99733	5	Nucleosome assembly protein 1-like 4	NAP1L4	0.999999	2.80E-64	268.08	S	___________MADHSFSDGVPSDSVEAAKN	3783700	2662600	5268
Q9Y2D5	393	A-kinase anchor protein 2	AKAP2	1	3.62E-67	267.93	S	GPPEDSGASAAKGQKSPGALETPSAAGSQGN	2483900	1101000	6778
Q86YP4	113	Transcriptional repressor p66-alpha	GATAD2A	0.759356	7.09E-66	267.7	S	PPSPDVIVLSDNEQPSSPRVNGLTTVALKET	7563800	0	4024
P46821	1387	Microtubule-associated protein 1B;MAP1B heavy chain;MAP1 li	MAP1B	0.985276	1.78E-64	267.69	S	NERASVSPMDEPVPDSESPIEKVLSPLRSPP	2787200	1991600	1572
P62258	210	14-3-3 protein epsilon	YWHAE	0.999712	2.14E-64	267.69	S	AKAAFDDAIAELDTLSEESYKDSTLIMQLLR	2412900	841330	1918
O75781	141	Paralemmin-1	PALM	0.994691	2.70E-62	267.69	T	AKEERKTEVVMNSQQTPVGTPKDKRVSNTPL	992190	968250	7217
O75781	145	Paralemmin-1	PALM	1	2.70E-62	267.69	T	RKTEVVMNSQQTPVGTPKDKRVSNTPLRTVD	992190	968250	7218
Q8WWM7	111	Ataxin-2-like protein	ATXN2L	1	1.16E-62	266.99	S	IGSARGQSTGKGPPQSPVFEGVYNNSRMLHF	2108500	2569900	4563
Q96B36	203	Proline-rich AKT1 substrate 1	AKT1S1	0.629761	9.60E-63	266.99	S	VPVWGFKEKRTEARSSDEENGPPSSPDLDRI	606460	1096600	4799
Q96B36	211	Proline-rich AKT1 substrate 1	AKT1S1	0.98902	9.60E-63	266.99	S	KRTEARSSDEENGPPSSPDLDRIAASMRALV	396610	1745700	4800
Q9BY44	506	Eukaryotic translation initiation factor 2A;Eukaryotic trans	EIF2A	0.999602	3.30E-63	266.39	S	HEAKKAAKQEARSDKSPDLAPTPAPQSTPRN	1912500	778710	5465
Q9H6Z4	219	Ran-binding protein 3	RANBP3	0.662045	2.81E-62	266.07	S	TGAVPAASPDTAAWRSPSEAADEVCALEEKE	407290	1510200	5744
Q9C073	213	Protein FAM117A	FAM117A	1	4.07E-51	265.56	S	PSGSPVLRLSPCLHRSLEGLNQELEEVFVKE	1383800	1036200	5503
Q9H1A4	688	Anaphase-promoting complex subunit 1	ANAPC1	0.999987	5.01E-51	265.53	S	DRLAWTRNFDFEGSLSPVIAPKKARPSETGS	1061400	647180	5591
P41229	1359	Lysine-specific demethylase 5C	KDM5C	0.975867	3.71E-51	265.43	S	MPKVQGLLENGDSVTSPEKVAPEEGSGKRDL	2315500	4214600	1487
O60841	214	Eukaryotic translation initiation factor 5B	EIF5B	1	1.26E-51	265.09	S	GQKKNQKNKPGPNIESGNEDDDASFKIKTVA	48096000	72710000	533
O00559	36	Receptor-binding cancer antigen expressed on SiSo cells	EBAG9	1	3.90E-53	264.1	S	FLKRLICRSGRGRKLSGDQITLPTTVDYSSV	2891600	1183200	130
Q96T37	670	Putative RNA-binding protein 15	RBM15	1	8.73E-50	264.01	S	RSPESDRPRKRHCAPSPDRSPELSSSRDRYN	65363000	57245000	5156
Q96T37	679	Putative RNA-binding protein 15	RBM15	0.570595	8.73E-50	264.01	S	KRHCAPSPDRSPELSSSRDRYNSDNDRSSRL	0	0	5159
P60174	58	Triosephosphate isomerase	TPI1	1	2.66E-49	263.47	S	KFFVGGNWKMNGRKQSLGELIGTLNAAKVPA	3954800	3250800	1903
Q9UQ35	1378	Serine/arginine repetitive matrix protein 2	SRRM2	0.49915	3.42E-50	263.15	S	LETDPSLDMKEQSTRSSGHSSSELSPDAVEK	0	0	6733
Q9UQ35	1379	Serine/arginine repetitive matrix protein 2	SRRM2	0.593255	3.42E-50	263.15	S	ETDPSLDMKEQSTRSSGHSSSELSPDAVEKA	0	0	6734
Q04726	286	Transducin-like enhancer protein 3	TLE3	0.998243	1.20E-51	263.07	S	NGLDKARSLKKDAPTSPASVASSSSTPSSKT	18973000	13744000	2171
Q15361	487	Transcription termination factor 1	TTF1	1	9.90E-54	262.98	S	SEIRYLSADSGDADDSDADLGSAVKQLQEFI	1554300	713830	2904
P46087	732	Probable 28S rRNA (cytosine(4447)-C(5))-methyltransferase	NOP2	0.99998	1.08E-49	262.82	S	NAPPKGTDTQTPAVLSPSKTQATLKPKDHHQ	15050000	10469000	1546
Q92625	663	Ankyrin repeat and SAM domain-containing protein 1A	ANKS1A	0.992968	9.66E-50	262.48	S	LSNCSIGKKRLEKSPSFASEWDEIEKIMSSI	1435100	833390	4685
P18615	51	Negative elongation factor E	NELFE	0.772803	4.74E-51	262.37	S	SSSSTTSQGGVKRSLSEQPVMDTATATEQAK	1775600	0	1142
Q12888	500	Tumor suppressor p53-binding protein 1	TP53BP1	1	4.46E-50	262.37	S	LTVECSKTSEIEPKNSPEDLGLSLTGDSCKL	7246700	5230100	2354
Q8TDD1	75	ATP-dependent RNA helicase DDX54	DDX54	0.983569	2.11E-51	261.06	S	GPGRPLPTFPTSECTSDVEPDTREMVRAQNK	22191000	9065700	4467
P24534	90	Elongation factor 1-beta	EEF1B2	0.997551	6.72E-51	260.97	S	ALGKYGPADVEDTTGSGATDSKDDDDIDLFG	1184200	1499300	1234
Q9NS69	15	Mitochondrial import receptor subunit TOM22 homolog	TOMM22	1	7.32E-54	260.97	S	_MAAAVAAAGAGEPQSPDELLPKGDAEKPEE	22615000	34697000	5996
Q92878	690	DNA repair protein RAD50	RAD50	1	2.74E-50	260.97	T	DENQSCCPVCQRVFQTEAELQEVISDLQSKL	235790	709050	7948
Q8WZ73	240	E3 ubiquitin-protein ligase rififylin	RFFL	0.814613	6.75E-51	260.96	S	QSIDSEDSFVPGRRASLSDLTDLEDIEGLTV	453920	448340	4615
Q9UHR4	261	Brain-specific angiogenesis inhibitor 1-associated protein 2	BAIAP2L1	1	1.70E-50	260.89	S	IKTPASTPVSGTPQASPMIERSNVVRKDYDT	7911400	6309100	6363
Q9UHR4	257	Brain-specific angiogenesis inhibitor 1-associated protein 2	BAIAP2L1	0.991137	1.70E-50	260.89	T	MIEEIKTPASTPVSGTPQASPMIERSNVVRK	6091400	4440400	8215
Q6Y7W6	26	PERQ amino acid-rich with GYF domain-containing protein 2	GIGYF2	0.995232	2.33E-51	260.56	S	GPEWLRALSSGGSITSPPLSPALPKYKLADY	6206000	4604300	3642
Q9UQ35	1271	Serine/arginine repetitive matrix protein 2	SRRM2	0.999994	6.88E-50	260.56	S	LSSELKEMSTSNFESSPEVEERPAVSLTLDQ	5573600	880350	6623
O43719	713	HIV Tat-specific factor 1	HTATSF1	1	1.98E-49	260.5	S	EDDDSNEKLFDEEEDSSEKLFDDSDERGTLG	675580	1675000	410
Q6WKZ4	357	Rab11 family-interacting protein 1	RAB11FIP1	0.992682	1.59E-49	260.25	S	SSPSPKGFRKKHLFSSTENLAAGSWKEPAEG	2929700	1651200	3637
Q9Y2J2	460	Band 4.1-like protein 3;Band 4.1-like protein 3, N-terminal	EPB41L3	0.999998	1.59E-49	260.25	S	DGEVGTGQYATTKGISQTNLITTVTPEKKAE	6693900	3510600	6781

O75448	862	Mediator of RNA polymerase II transcription subunit 24	MED24	0.997806	7.77E-51	260.24	S	FPLDDVQPSKLMRLLSSNEDDANILSSPTDR	1776700	958640	630
Q9UKJ3	1107	G patch domain-containing protein 8	GPATCH8	0.999994	8.66E-49	260.07	S	LLLEKIQSRKVERKPSVSEEVQATPNKAGPK	907130	639240	6444
Q15424;Q!247;246		Scaffold attachment factor B1;Scaffold attachment factor B2	SAFB;SAFB2	0.987188	2.14E-53	259.81	S	EESSELEQPFAQDTSSVGPDRKLAEEEDLFD	1317500	1389000	2923
P10155	19	60 kDa SS-A/Ro ribonucleoprotein	TROVE2	1	9.05E-51	259.34	S	SVNQMQPLNEKQIANSQDGYVWQVTDMNRLH	1691400	1902400	983
P26358	714	DNA (cytosine-5)-methyltransferase 1	DNMT1	1	2.89E-51	259.34	S	ADDDEEVDDNIPEMPSPKKMHQGKKKKQNKN	7974200	10746000	1275
P35659	13	Protein DEK	DEK	0.996869	1.48E-53	259.34	T	____MSASAPAAEGEGTPTQPASEKEPEMPGP	727020	1247700	7365
P78559	1654	Microtubule-associated protein 1A;MAP1A heavy chain;MAP1 li	MAP1A	0.976649	1.88E-49	259.22	S	KYWRGQDVVQEWQETSPTREEPAGEQKELAP	3693300	1718900	1978
O15446	285	DNA-directed RNA polymerase I subunit RPA34	CD3EAP	0.969336	9.39E-51	259.1	S	KQEQINTEPLEDTVLSPTKKRKRQKGTEGME	2336100	1514300	323
Q9Y2X3	502	Nucleolar protein 58	NOP58	1	3.37E-53	258.83	S	KKKRGKKKHIKEEPLSEEEPCTSTAIASPEK	6556600	7990900	6834
Q9Y2X3	514	Nucleolar protein 58	NOP58	1	3.37E-53	258.83	S	EPLSEEEPCTSTAIASPEKKKKKKKKRENED	3596300	6260900	6835
O43818	53	U3 small nucleolar RNA-interacting protein 2	RRP9	0.997946	1.72E-50	258.68	S	KSKGGGKMNEEISSDSESESLAPRKPEEEEE	695030	783750	428
Q641Q2	539	WASH complex subunit FAM21A	FAM21A	1	4.15E-53	258.68	S	NLKPSSETKTQKGLFSDEEDSEDLFSSQSAS	1502400	1194500	3388
P35221;P2652;651		Catenin alpha-1;Catenin alpha-2	CTNNA1;CTNN	0.998299	2.68E-50	258.47	S	ELDDSDFETEDFDVRSRTSVQTEDDQLIAGQ	4184900	2629800	1375
P35221;P2658;657		Catenin alpha-1;Catenin alpha-2	CTNNA1;CTNN	1	2.68E-50	258.47	T	FETEDFDVRSRTSVQTEDDQLIAGQSARAIM	10437000	5439800	7357
O14545	415	TRAF-type zinc finger domain-containing protein 1	TRAFD1	0.994659	2.73E-49	258.41	S	VTEGIPRLDSQPQETSPELPRRRVRHQGDLS	24848000	13255000	152
P50479	112	PDZ and LIM domain protein 4	PDLIM4	0.999405	1.01E-49	258.17	S	AQAHRIHIDPEIQDGSPTTSRRPSGTGTGPE	8670300	5464800	1747
P35251	69	Replication factor C subunit 1	RFC1	0.999872	8.43E-50	258.13	S	FKQKQPSKKKRIIYSDSDSEEETLQVKNAKK	18384000	16815000	1385
P35251	71	Replication factor C subunit 1	RFC1	0.99837	8.43E-50	258.13	S	QKQPSKKKRIIYSDSDSEEETLQVKNAKKPP	19602000	17411000	1386
Q13033	229	Striatin-3	STRN3	1	2.31E-49	258.01	S	SVETKNLEQILNGGESPKQKGQEIKRSSGDV	6200700	2998800	2391
A1L390	1037	Pleckstrin homology domain-containing family G member 3	PLEKHG3	1	1.91E-49	257.81	S	VSQRTTSPGGRPSARSPLSPTETFSWPDVRE	1599700	1373600	19
A1L390	1040	Pleckstrin homology domain-containing family G member 3	PLEKHG3	0.999999	1.91E-49	257.81	S	RTTSPGGRPSARSPLSPTETFSWPDVRELCS	1599700	1373600	20
P35611	358	Alpha-adducin	ADD1	1	2.30E-49	257.81	S	LLNPEKYKAKSRSPGSPVGEGTGSPPKWQIG	17647000	8415800	1401
O75376	1977	Nuclear receptor corepressor 1	NCOR1	0.999936	6.14E-57	257.72	S	SSHRYETPSDAIEVISPASSPAPPQEKLQTY	4843500	2524100	606
O75376	1981	Nuclear receptor corepressor 1	NCOR1	0.921913	6.14E-57	257.72	S	YETPSDAIEVISPASSPAPPQEKLQTYQPEV	987040	620060	608
O94804	952	Serine/threonine-protein kinase 10	STK10	0.999494	9.68E-49	257.54	T	MFFKLSEEAECPNPSTPSKAAKFFPYSSADA	757950	1326700	7228
Q8N3F8	612	MICAL-like protein 1	MICALL1	0.999991	9.27E-50	257.37	S	CSGATPTPLLLVGDRSPVPSPGSSSPQLQVK	1641700	1403100	4253
Q8N3F8	619	MICAL-like protein 1	MICALL1	0.683945	9.27E-50	257.37	S	PLLLVGDRSPVPSPGSSSPQLQVKSSCKENP	1641700	0	4254
Q9Y383	18	Putative RNA-binding protein Luc7-like 2	LUC7L2	0.999969	3.23E-49	257.2	S	AQAQMRAMLDQLMGTSRDGDTTRQRIKFSDD	6143200	4078600	6843
Q8N122	859	Regulatory-associated protein of mTOR	RPTOR	0.999922	1.02E-50	257.06	S	NARPQRVLDTSSLTQSAPASPTNKGVHIHQA	4946600	4293000	4210
Q9C0B0	378	RING finger protein unkempt homolog	UNK	0.999984	3.36E-51	256.8	S	APDLSALLCRNSSLGSPSNLCGSPPGSIRKP	2936500	2154700	5508
P37275	646	Zinc finger E-box-binding homeobox 1	ZEB1	0.997103	5.26E-53	256.79	S	KMQAGQISVQSSEPSSPEPGKVNIPAKNNDQ	0	0	1439
Q8NHV4	516	Protein NEDD1	NEDD1	0.998777	3.24E-53	256.79	S	AKLVTSGAESGNLNTSPSSNQTRNSEKFEKP	2387100	1676200	4420
Q02880	1466	DNA topoisomerase 2-beta	TOP2B	1	4.11E-51	256.66	S	PSYSQKSEDDSAKFDSNEEDSASVFSPSFGL	4595600	2486100	2100
Q99733	12	Nucleosome assembly protein 1-like 4	NAP1L4	0.99999	5.68E-51	256.33	S	____MADHSFSDGVPSDSVEAAKNASNTEKL	5363000	3034400	5270
Q01167	398	Forkhead box protein K2	FOXK2	1	2.44E-50	255.72	S	HSSGAQTPESLSREGSPAPLEPEPGAAQPKL	17562000	11979000	2060
P18206	288	Vinculin	VCL	0.999999	3.00E-49	255.39	S	IDSKLNQAKGWLRDPSASPGDAGEQAIRQIL	3854400	0	1128
Q4G0J3	261	La-related protein 7	LARP7	0.999382	1.20E-49	254.8	S	TSISKMKRSRPTSEGSDIESTEPQKQCSKKK	1371700	1167500	3092
Q9NQS7	306	Inner centromere protein	INCENP	1	9.74E-50	254.8	S	STPTGSRTDSQSVRHSPIAPSSPSPQVLAQK	3036700	3243800	5919
Q9NQS7	312	Inner centromere protein	INCENP	0.920188	9.74E-50	254.8	S	RTDSQSVRHSPIAPSSPSPQVLAQKYSLVAK	1626500	1033000	5921
Q9NQS7	314	Inner centromere protein	INCENP	0.999997	9.74E-50	254.8	S	DSQSVRHSPIAPSSPSPQVLAQKYSLVAKQE	3036700	389880	5922
O15047	1103	Histone-lysine N-methyltransferase SETD1A	SETD1A	1	1.29E-50	254.79	S	TPAPVEVPVPERVAGSPVTPLPEQEASPARP	3987600	2927800	270
O15047	1114	Histone-lysine N-methyltransferase SETD1A	SETD1A	1	1.29E-50	254.79	S	RVAGSPVTPLPEQEASPARPAGPTEESPPSA	1855800	1472100	271
Q14141	416	Septin-6	SEPT6	0.989919	6.59E-53	254.54	S	TAAELLQSQSQAGGSQTLKRDKEKKNNPWL	1037200	865900	2651
Q9C0E2	524	Exportin-4	XPO4	0.732871	2.86E-50	254.02	S	GQLQRHQQLLASPGSSTVDNKMLDDLYEDI	1502400	0	5542
O00566	167	U3 small nucleolar ribonucleoprotein protein MPP10	MPHOSPH10	0.999998	2.43E-50	253.73	S	AENSSKSDLRKSPVFSDEDSDLDFDISKLEQ	898370	808290	132
P22059	190	Oxysterol-binding protein 1	OSBP	1	3.15E-21	253.52	S	ALELAKAKAVKMLAESDESGDEESVSQTDKT	0	0	1203
Q14160	504	Protein scribble homolog	SCRIB	0.99855	7.19E-53	253.52	S	EGRRSEACPCQPDSGSPLPAEEEKRLSAESG	1612900	1413300	2687
Q5VSL9	335	Striatin-interacting protein 1	STRIP1	1	3.02E-50	253.36	S	LPEDSIKVIRNMRAASPPASASDLIEQQQKR	5844000	2208000	3319
Q9HAN9	117	Nicotinamide/nicotinic acid mononucleotide adenylyltransfera	NMNAT1	0.999978	3.02E-50	253.36	S	HQEKLEASDCDHQQNSPTLERPGRKRKWTET	3095300	4333000	5816
Q9HB90;Q95;96		Ras-related GTP-binding protein C;Ras-related GTP-binding pr	RRAGC;RRAGI	0.997788	4.62E-49	253.36	S	VVFHKMSPNETLFLESTNKIYKDDISNSSFV	937240	813490	5834
Q9HB90;Q96;97		Ras-related GTP-binding protein C;Ras-related GTP-binding pr	RRAGC;RRAGI	0.997788	4.62E-49	253.36	T	VFHKMSPNETLFLESTNKIYKDDISNSSFVN	0	16761000	8128
P49959	688	Double-strand break repair protein MRE11A	MRE11A	0.821138	2.51E-23	253.09	S	KIMSQSQVSKGVDFESSEDDDDDPFMNTSSL	0	0	1743
Q96ST3	832	Paired amphipathic helix protein Sin3a	SIN3A	1	8.11E-57	253.09	S	HHFIPDLLFAQRGDLSDVEEEEEEEMDVDEA	435960	0	5139
Q9H0G5	33	Nuclear speckle splicing regulatory protein 1	NSRP1	0.999999	1.89E-53	252.8	S	QLHPVLQKPSVFGNDSDDDDETSVSESLQRE	1125100	1581300	5582
Q52LR7	538	Enhancer of polycomb homolog 2	EPC2	0.998069	3.75E-49	252.79	S	CRLQCFQPRLLNLQDSDSEECTSRKPGQTVN	1659200	1589700	3123
P42858	419	Huntingtin	HTT	0.988242	3.97E-53	252.55	S	LTAAKEESGGRSRSGSIVELIAGGGSSCSPV	2489300	1242300	1520
P42858	432	Huntingtin	HTT	0.99981	3.97E-53	252.55	S	SGSIVELIAGGGSSCSPVLSRKQKGKVLLGE	2489300	1242300	1521
P55196	1779	Afadin	MLLT4	1	5.97E-51	252.55	S	CRDAKEKRSKSQDADSPGSSGAPENLTFKER	4734100	2039800	1869
O94804	438	Serine/threonine-protein kinase 10	STK10	1	3.84E-49	252.47	S	QVAQEKQVAEQGGDLSPAANRSQKASQSRPN	9056900	6141100	690
Q14247	418	Src substrate cortactin	CTTN	0.999936	4.96E-49	252.46	S	PVSPAPQPTEERLPSSPVYEDAASFKAELSY	18749000	14843000	2731
Q15637	80	Splicing factor 1	SF1	1	3.30E-49	252.46	S	LRTGDLGIPPNPEDRSPSPEPIYNSEGKRLN	19722000	23317000	2954
Q15637	82	Splicing factor 1	SF1	1	3.30E-49	252.46	S	TGDLGIPPNPEDRSPSPEPIYNSEGKRLNTR	19722000	23317000	2955
Q53F19	25	Uncharacterized protein C17orf85	C17orf85	1	1.94E-53	252.41	S	SVKAEAPAGPALGLPSPEAESGVDRGEPEPM	4645400	3949900	3136
Q9Y6G9	510	Cytoplasmic dynein 1 light intermediate chain 1	DYNC1LI1	0.999841	6.73E-51	252.38	S	VHAELDRITRKPVTVSPTTPTSPTEGEAS__	14434000	13330000	7005
Q9Y6G9	515	Cytoplasmic dynein 1 light intermediate chain 1	DYNC1LI1	0.932507	6.73E-51	252.38	T	DRITRKPVTVSPTTPTSPTEGEAS_______	9212000	2246800	8321
Q12830	656	Nucleosome-remodeling factor subunit BPTF	BPTF	1	1.90E-50	252.37	S	TTNATSEETSPSEGRSPVGCLSETPDSSNMA	671070	455520	2315
Q7Z460	1091	CLIP-associating protein 1	CLASP1	0.921634	1.78E-49	252.37	S	LLHNHLKNSSNTSVGSPSNTIGRTPSRHTSS	24477000	17865000	3808
Q14C86	746	GTPase-activating protein and VPS9 domain-containing protei	GAPVD1	0.987963	8.02E-53	252.11	S	QEERLQELESCSGLGSTSDDTDVREVSSRPS	1210600	899540	2846
P08670	459	Vimentin	VIM	0.992715	1.83E-49	252.01	S	IKTVETRDGQVINETSQHHDDLE________	555000	707730	964
O43379	1226	WD repeat-containing protein 62	WDR62	0.5	8.24E-53	251.74	S	SYMEATASSRARISRSISLGDSEGPIVATLA	1129600	403310	373
O43379	1228	WD repeat-containing protein 62	WDR62	0.5	8.24E-53	251.74	S	MEATASSRARISRSISLGDSEGPIVATLAQP	1129600	403310	374
Q5VZL5	122	Zinc finger MYM-type protein 4	ZMYM4	0.999987	3.46E-50	251.56	S	TDDSLEVERRVTQHESDNENEIQIQNKLKKD	4376900	4847700	3372
Q9H1E3	19	Nuclear ubiquitous casein and cyclin-dependent kinase substr	NUCKS1	1	6.51E-51	251.38	S	PVRNRKVVDYSQFQESDDADEDYGRDSGPPT	67465000	59364000	5607
Q9BQG0	11	Myb-binding protein 1A	MYBBP1A	1	3.59E-50	251	S	_____MESRDPAQPMSPGEATQSGARPADRY	15055000	21190000	5308
Q9NZT2	537	Opioid growth factor receptor	OGFR	0.999989	8.68E-53	251	S	PGPSPAGPAGDEPAESPSETPGPRPAGPAGD	4445500	3402300	6170
P52948	612	Nuclear pore complex protein Nup98-Nup96;Nuclear pore compl	NUP98	1	4.57E-49	250.46	S	IKKLVLKNLNNSNLFSPVNRDSENLASPSEY	1472000	1363200	1826
Q9Y388	188	RNA-binding motif protein, X-linked 2	RBMX2	0.994851	4.63E-49	250.26	S	EKADREVQAEQPSSSSPRRKTVKEKDDTGPK	0	1598400	6846
Q96RS0	85	Trimethylguanosine synthase	TGS1	1	3.18E-50	250.08	S	GTAESHDSKGIGLDESELDSEAELMRSMGLP	945650	686580	5099
Q13428	1378	Treacle protein	TCOF1	0.999996	4.60E-49	249.89	S	KKKKLGAGEGGEASVSPEKTSTTSKGKAKRD	6022000	7120500	2510
Q8NCF5	204	NFATC2-interacting protein	NFATC2IP	1	3.87E-50	249.89	S	KTEFLDLDNSPLSPPSPRTKSRTHTRALKKL	5108900	3064200	4330
Q8NE71	109	ATP-binding cassette sub-family F member 1	ABCF1	1	1.01E-49	249.89	S	EKELMERLKKLSVPTSDEEDEVPAPKPRGGK	66519000	60367000	4367
Q9NZL4	351	Hsp70-binding protein 1	HSPBP1	0.999002	2.16E-49	249.54	S	EELEFCEKLLQTCFSSPADDSMDR_______	1236200	4110000	6158
O60343	754	TBC1 domain family member 4	TBC1D4	0.996122	7.44E-52	249.49	S	ESSDGEGRKRTSSTCSNESLSVGGTSVTPRR	714710	3148500	498
O60343	752	TBC1 domain family member 4	TBC1D4	0.579352	7.44E-52	249.49	T	ASESSDGEGRKRTSSTCSNESLSVGGTSVTP	0	2817400	7183
O43823	323	A-kinase anchor protein 8	AKAP8	1	2.32E-50	249.41	S	FQLYEEPDTKLARVDSEGDFSENDDAAGDFR	455100	571040	430
O43399	12	Tumor protein D54	TPD52L2	1	2.02E-49	249.36	S	____MDSAGQDINLNSPNKGLLSDSMTDVPV	2125700	2143800	379
Q8TC07	675	TBC1 domain family member 15	TBC1D15	0.999989	4.93E-49	248.75	S	SNALPTLSASGARNDSPTQIPVSSDVCRLTP	14729000	9560700	4458
Q96T37	678	Putative RNA-binding protein 15	RBM15	0.970292	8.76E-49	248.7	S	RKRHCAPSPDRSPELSSSRDRYNSDNDRSSR	13457000	6687900	5158
Q9UHD8	30	Septin-9	SEPT9	1	2.28E-49	248.67	S	RLRRLGDSSGPALKRSFEVEEVETPNSTPPR	2333000	1261500	6349
P49792;Q2251;127		E3 SUMO-protein ligase RanBP2;RanBP2-like and GRIP domain-co	RANBP2;RGPI	0.997223	2.37E-46	248.46	S	DSVSSSSVHASPLASSPVRKNLFRFGESTTG	4099800	3775300	1708
Q9C0C9	838	E2/E3 hybrid ubiquitin-protein ligase UBE2O	UBE2O	0.71398	7.44E-44	248.33	T	SLKNMTVEQLLTGSPTSPTVEPEKPTREKKF	295990	560890	8078
P05387	17	60S acidic ribosomal protein P2	RPLP2	0.990143	1.20E-40	248.23	S	RYVASYLLAALGGNSSPSAKDIKKILDSVGI	971640	887700	899
Q4VCS5	714	Angiomotin	AMOT	0.998931	2.81E-42	248.23	S	AATVAAQRDTTVISHSPNTSYDTALEARIQK	8333800	2687400	3113
O75400	932	Pre-mRNA-processing factor 40 homolog A	PRPF40A	0.964685	2.38E-39	247.58	T	KSPKKKTGKDSGNWDTSGSELSEGELEKRRR	2746900	0	7202
P05387;P(105;104		60S acidic ribosomal protein P2;60S acidic ribosomal protei	RPLP2;RPLP:	1	7.55E-39	247.44	S	AAEEKKDEKKEESEESDDDMGFGLFD_____	2050300	0	895
O43719	676	HIV Tat-specific factor 1	HTATSF1	1	2.95E-38	247.3	S	KKAEEGDADEKLFEESDDKEDEDADGKEVED	3166000	5003400	413
Q96HR8	315	H/ACA ribonucleoprotein complex non-core subunit NAF1	NAF1	1	2.06E-39	247.3	S	SWKNDQEPPPEALDFSDDEKEKEAKQRKKSQ	2365000	2761000	4946
O15047	1106	Histone-lysine N-methyltransferase SETD1A	SETD1A	1	9.32E-39	247.3	T	PVEVPVPERVAGSPVTPLPEQEASPARPAGP	881590	539650	7148
Q8IWQ3	382	Serine/threonine-protein kinase BRSK2	BRSK2	1	1.74E-39	247.28	S	SPMLNRHGKRRPERKSMEVLSVTDGGSPVPA	3343900	2507100	4069
Q8IWQ3	393	Serine/threonine-protein kinase BRSK2	BRSK2	0.999445	1.74E-39	247.28	S	PERKSMEVLSVTDGGSPVPARRAIEMAQHGQ	3427700	1990700	4070
Q96C19	74	EF-hand domain-containing protein D2	EFHD2	0.999976	1.54E-38	247.02	S	LLRRADLNQGIGEPQSPSRRVFNPYTEFKEF	43478000	24528000	4816
P05387;P(102;101		60S acidic ribosomal protein P2;60S acidic ribosomal protei	RPLP2;RPLP:	1	7.09E-38	246.9	S	APAAAEEKKDEKKEESEESDDDMGFGLFD__	0	679410	894
Q86WB0	395	Nuclear-interacting partner of ALK	ZC3HC1	0.99088	1.69E-39	246.75	S	RSMGTGDTPGLEVPSSPLRKAKRARLCSSSS	993910	506800	3981
P51608	80	Methyl-CpG-binding protein 2	MECP2	0.999525	8.14E-42	246.67	S	SEGSGSAPAVPEASASPKQRRSIIRDRGPMY	6080400	6000600	1785
P20042	67	Eukaryotic translation initiation factor 2 subunit 2	EIF2S2	1	1.89E-38	246.66	S	KDLEADEEDTRKKDASDDLDDLNFFNQKKKK	371460	615610	1178
Q13112	429	Chromatin assembly factor 1 subunit B	CHAF1B	0.974778	1.89E-38	246.66	S	RPVEGTPASRTQDPSSPGTTPPQARQAPAPT	7851300	5711000	2411
Q9UBQ5	217	Eukaryotic translation initiation factor 3 subunit K	EIF3K	0.981085	2.25E-38	246.63	S	VEKIDFDSVSSIMASSQ______________	1783300	3486800	6271
Q6PKG0	548	La-related protein 1	LARP1	0.984591	1.92E-39	246.49	S	EEVSNLKTLPKGLSASLPDLDSENWIEVKKR	7198000	6245900	3559
P46379	973	Large proline-rich protein BAG6	BAG6	1	3.40E-39	246.42	S	QGAERASPEPQRENASPAPGTTAEEAMSRGP	24737000	14416000	1565
P49815	1420	Tuberin	TSC2	0.766876	9.61E-42	246.32	S	ADVGRLSPEVKARSQSGTLDGESAAWSASGE	830860	516890	1727
Q9UNZ2	114	NSFL1 cofactor p47	NSFL1C	1	3.05E-38	246.31	S	SERSGQQIVGPPRKKSPNELVDDLFKGAKEH	5068100	6548700	6554
Q9UK76	54	Hematological and neurological expressed 1 protein;Hematolo	HN1	1	8.48E-39	246.31	T	EQPVRKNKMASNIFGTPEENQASWAKSAGAK	954520	1045300	8229
Q5T200	993	Zinc finger CCCH domain-containing protein 13	ZC3H13	1	1.16E-38	246.25	S	ERGNIETTSEDGQVFSPKKGQKKKSIEKKRK	3360900	3344100	3256
Q71RC2	594	La-related protein 4	LARP4	0.820655	5.53E-45	245.63	S	TIPVSPPSTTKPSRASTASPCNNNINAATAV	0	11677000	3694
Q71RC2	597	La-related protein 4	LARP4	0.386508	5.53E-45	245.63	S	VSPPSTTKPSRASTASPCNNNINAATAVALQ	0	0	3695
Q71RC2	595	La-related protein 4	LARP4	0.49095	5.53E-45	245.63	T	IPVSPPSTTKPSRASTASPCNNNINAATAVA	0	0	7785
P20042	2	Eukaryotic translation initiation factor 2 subunit 2	EIF2S2	1	8.82E-39	245.5	S	_______________MSGDEMIFDPTMSKKKK	9835200	8154800	1180
Q13177	2	Serine/threonine-protein kinase PAK 2;PAK-2p27;PAK-2p34	PAK2	1	1.30E-38	245.39	S	_______________MSDNGELEDKPPAPPVR	6271300	4680300	2429
Q9UPN6	617	Protein SCAF8	SCAF8	0.995645	3.24E-38	245.39	S	VKSSEPVKETVQTTQSPTPVEKETVVTTQAE	3485000	5610900	6562
Q96KC8	479	DnaJ homolog subfamily C member 1	DNAJC1	1	4.65E-29	245.18	S	RAKRQKDFDIAEQNESSDEESLRKERARSAE	1151400	1694600	5004
Q96KC8	480	DnaJ homolog subfamily C member 1	DNAJC1	1	4.65E-29	245.18	S	AKRQKDFDIAEQNESSDEESLRKERARSAEE	1151400	1694600	5005
P05455	366	Lupus La protein	SSB	1	2.38E-41	245.1	S	GKGKVQFQGKKTKFASDDEHDEHDENGATGP	70888000	53032000	905
Q9NZN5	1288	Rho guanine nucleotide exchange factor 12	ARHGEF12	0.794191	1.02E-39	245.1	S	SVQEDWQHFPRYRTASQGPQTDSVIQNSENI	598790	570860	6165
Q16539	180	Mitogen-activated protein kinase 14	MAPK14	0.546564	9.57E-38	245.08	T	KILDFGLARHTDDEMTGYVATRWYRAPEIML	0	4251000	7658
O43150	701	Arf-GAP with SH3 domain, ANK repeat and PH domain-containin	ASAP2	1	1.75E-38	245.04	S	HVEYEWRLLHEDLDESDDDMDEKLQPSPNRR	1234300	857150	338
Q9Y6G9	513	Cytoplasmic dynein 1 light intermediate chain 1	DYNC1LI1	0.985524	8.69E-39	245.04	T	ELDRITRKPVTVSPTTPTSPTEGEAS_____	5442500	9342200	8320
Q96T23	1359	Remodeling and spacing factor 1	RSF1	1	5.48E-42	244.68	S	ESDEEEDFENVGKVGSPLDYSLVDLPSTNGQ	1582100	1298700	5152
Q96T23	1375	Remodeling and spacing factor 1	RSF1	0.999811	5.48E-42	244.68	S	PLDYSLVDLPSTNGQSPGKAIENLIGKPTEK	3835000	2266800	5154
P49790	334	Nuclear pore complex protein Nup153	NUP153	0.982672	3.24E-38	244.54	S	SSPLADAKRIPSIVSSPLNSPLDRSGIDITD	1001100	3872400	1696
P49790	338	Nuclear pore complex protein Nup153	NUP153	1	3.24E-38	244.54	S	ADAKRIPSIVSSPLNSPLDRSGIDITDFQAK	1001100	2859900	1697
Q13439	41	Golgin subfamily A member 4	GOLGA4	0.949084	4.18E-38	244.23	S	SSNSSTPTRMRSRTSSFTEQLDEGTPNRESG	4134000	0	2532
Q01082	2340	Spectrin beta chain, non-erythrocytic 1	SPTBN1	0.902541	1.23E-40	244.18	S	RAQTLPTSVVTITSESSPGKREKDKEKDKEK	1078700	0	2040
P09874	782	Poly [ADP-ribose] polymerase 1	PARP1	0.986203	3.13E-38	244.1	S	NLLDIEVAYSLLRGGSDDSSKDPIDVNYEKL	0	0	973
Q6AI08	643	HEAT repeat-containing protein 6	HEATR6	0.992895	2.25E-38	244	S	KKAPAGPSLEETSVSSPKGSSEPCWLIRLCI	5476200	2297300	3440
Q7Z460	797	CLIP-associating protein 1	CLASP1	0.833161	7.11E-39	243.99	S	GRIPGSVNAMRVLSTSTDLEAAVADALKKPV	971780	1656600	3813
Q6PD62	925	RNA polymerase-associated protein CTR9 homolog	CTR9	1	1.27E-40	243.99	T	RSKKGGEFDEFVNDDTDDDLPISKKKKRRKG	11034000	11735000	7761
Q7Z6Z7	1907	E3 ubiquitin-protein ligase HUWE1	HUWE1	0.999942	5.27E-38	243.92	S	CIRIALPAPRGSGTASDDEFENLRIKGPNAV	13783000	11487000	3878
P18583	1555	Protein SON	SON	0.770074	3.20E-41	243.92	T	LIAKEMEHNTVCAAGTSPVGEIGEEKILPTS	0	0	7300
Q15154	65	Pericentriolar material 1 protein	PCM1	1	3.79E-38	243.83	S	KKFGVESDKRVTNDISPESSPGVGRRRTKTP	13454000	9553000	2887

Q15154	69	Pericentriolar material 1 protein	PCM1	1	3.79E-38	243.83	S		VESDKRVTNDISPESSPGVGRRRTKTPHTFP	5414800	4648100	2889
P46013	1253	Antigen KI-67	MKI67	0.999999	4.48E-39	243.61	S		EELVAAGKTTKIPCDSPQSDPVDTPTSTKQR	1234900	912210	1531
P20810	243	Calpastatin	CAST	0.983019	4.02E-45	243.21	S		PDDAIDALSSDFTCGSPTAAGKKTEKEESTE	7590600	12132000	1190
Q9Y2J2	469	Band 4.1-like protein 3;Band 4.1-like protein 3, N-terminal	EPB41L3	1	2.24E-37	243.16	T		ATTKGISQTNLITTVTPEKKAEEERDEEEDK	3909900	1714400	8282
Q14C86	566	GTPase-activating protein and VPS9 domain-containing protei	GAPVD1	1	4.22E-39	242.94	S		DENKLHGKPDKTLRFSLCSDNLEGISEGPSN	931550	1139600	2843
Q14C86	569	GTPase-activating protein and VPS9 domain-containing protei	GAPVD1	1	4.22E-39	242.94	S		KLHGKPDKTLRFSLCSDNLEGISEGPSNRSN	1514400	1614700	2844
Q15398	662	Disks large-associated protein 5	DLGAP5	0.998696	2.35E-41	242.94	S		VSQSRNEMGIPQQTTSPENAGPQNTKSEHVK	18488000	14081000	2911
Q6F5E8	1246	Leucine-rich repeat-containing protein 16C	RLTPR	1	4.16E-41	242.45	S		GKRKQSKDGEIKKAGSDGDIMDSSTEAPPIS	2791000	2076800	3451
P23396	221	40S ribosomal protein S3	RPS3	0.998379	1.69E-37	242.35	T		DHVSIVEPKDEILPTTPISEQKGGKPEPPAM	0	20352000	7317
Q13263	473	Transcription intermediary factor 1-beta	TRIM28	1	9.46E-38	241.39	S		YSSAEPHVSGVKRSRSGEGEVSGLMRKVPRV	28676000	30449000	2460
Q13153	230	Serine/threonine-protein kinase PAK 1	PAK1	0.999738	4.92E-41	241.39	T		RDVATSPISPTENNTTPPDALTRNTEKQKKK	2887400	0	7528
O75379	30	Vesicle-associated membrane protein 4	VAMP4	1	3.65E-38	241.13	S		TGSVKSERRNLLEDDSDEEEDFFLRGPSGPR	6600100	7943300	610
Q9BY89	600	Uncharacterized protein KIAA1671	KIAA1671	0.999994	8.78E-39	241.13	T		CRGGSSVEAPCPSDVTPEDDRSFQTVWATVF	1013400	767770	8066
O00116	65	Alkyldihydroxyacetonephosphate synthase, peroxisomal	AGPS	0.999614	1.39E-44	241.08	S		REALSTNECKARRAASAATAAPTATPAAQES	801730	394820	89
Q9UQ35	424	Serine/arginine repetitive matrix protein 2	SRRM2	0.999631	6.97E-39	240.82	S		PEKLPQSSSSESSPPSPQPTKVSRHASSSPE	9297100	2613100	6652
P50502;Q8	76;72	Hsc70-interacting protein;Putative protein FAM10A4	ST13;ST13P	0.96268	6.41E-38	240.5	S		DSKKVEEDLKADEPSSEESDLEIDKEGVIEP	808670	1028300	1749
P50502;Q8	79;75	Hsc70-interacting protein;Putative protein FAM10A4	ST13;ST13P	1	6.41E-38	240.5	S		KVEEDLKADEPSSEESDLEIDKEGVIEPDTD	2727600	1554200	1750
O15550	769	Lysine-specific demethylase 6A	KDM6A	0.998831	2.36E-39	240.42	S		MTADAVCSPSHGDSKSPGLLSSDNPQLSALL	743300	835740	334
Q9UQ35	1552	Serine/arginine repetitive matrix protein 2	SRRM2	0.999965	2.82E-38	240.42	S		SRSGSSQELDVKPSASPQERSESDSSPDSKA	2769000	2478700	6691
Q9UEY8	681	Gamma-adducin	ADD3	0.999998	5.71E-38	240.34	S		EKIEEVLSPEGSPSKSPSKKKKKFRTPSFLK	24153000	18297000	6302
Q08J23	456	tRNA (cytosine(34)-C(5))-methyltransferase	NSUN2	0.973954	2.68E-40	239.8	S		KLQGKSAETRESTQLSPADLTEGKPTDPSKL	1200300	571330	2268
P46013	1376	Antigen KI-67	MKI67	0.989062	7.96E-39	239.7	S		EAVAAGKTTKMPCESSPPESADTPTSTRRQP	3188500	2203000	1533
P18583	1697	Protein SON	SON	1	6.01E-38	239.51	S		PLPVKESDQTLAALLSPKESSGGEKEVPPPP	2516400	2003600	1134
O95218	120	Zinc finger Ran-binding domain-containing protein 2	ZRANB2	1	4.97E-38	239.48	S		GFNERENVEYIEREESDGEYDEFGRKKKKYR	7491700	9405200	740
O76074	86	cGMP-specific 3',5'-cyclic phosphodiesterase	PDE5A	1	8.96E-38	239.19	S		GIRGHTESCSCPLQQSPRADNSAPGTPTRKI	1092400	501710	675
Q7Z417	212	Nuclear fragile X mental retardation-interacting protein 2	NUFIP2	0.999999	1.45E-38	239.13	S		YITNGYMGKGADNDGSGSESGYTTPKKRKAR	675720	1134200	3794
Q86YP4	107	Transcriptional repressor p66-alpha	GATAD2A	1	8.51E-41	239.13	S		MKSERRPPSPDVIVLSDNEQPSSPRVNGLTT	35586000	28181000	4023
Q9NYV4	251	Cyclin-dependent kinase 12	CDK12	0.969922	6.41E-41	239.1	S		SPSGASYGQDYDLSPSRSHTSSNYDSYKKSP	1317300	0	6130
Q96B36	212	Proline-rich AKT1 substrate 1	AKT1S1	0.99541	3.94E-38	238.88	S		RTEARSSDEENGPPSSPDLDRIAASMRALVL	606460	0	4801
Q12888	1067	Tumor suppressor p53-binding protein 1	TP53BP1	0.759284	9.30E-38	238.83	S		DPPTTPIRGNLLHFPSSQGEEEKEKLEGDHT	2762700	1593400	2342
Q53EZ4	428	Centrosomal protein of 55 kDa	CEP55	0.99551	9.30E-38	238.83	S		QGETENREKVAASPKSPTAALNESLVECPKC	1405500	3840100	3134
Q8N9T8	171	Protein KRI1 homolog	KRI1	1	1.64E-41	238.79	S		EQKQLKESFRAFVEDSEDEDGAGEGGSSLLQ	12630000	9193200	4305
O60231	107	Putative pre-mRNA-splicing factor ATP-dependent RNA helicase	DHX16	1	7.88E-38	238.64	S		EKNRSYRLLEDSEESSEETVSRAGSSLQKKR	0	1354300	439
O43719	721	HIV Tat-specific factor 1	HTATSF1	1	1.05E-14	238.49	S		LFDEEEDSSEKLFDDSDERGTLGGFGSVEEG	7929500	3342000	409
Q9BU76	217	Multiple myeloma tumor-associated protein 2	MMTAG2	0.981167	4.88E-37	238.45	S		KKKDKEHRRPAEATSSPTSPERPRHHHHDSD	0	0	5372
Q9HCK8	2008	Chromodomain-helicase-DNA-binding protein 8	CHD8	1	9.08E-39	238.45	S		TASPLPLRPDAPVEKSPEETATQVPSLESLT	1129900	706030	5868
Q9BU76	219	Multiple myeloma tumor-associated protein 2	MMTAG2	0.858899	1.84E-36	238.45	T		KDKEHRRPAEATSSPTSPERPRHHHHDSDSN	35072000	31171000	8049
Q8NFC6	2907	Biorientation of chromosomes in cell division protein 1-like	BOD1L1	0.691579	5.07E-38	238.2	S		DSKTDTGIVTVEQSPSSSKLKVMQTDESNKE	1198000	563160	4394
Q9BU76	220	Multiple myeloma tumor-associated protein 2	MMTAG2	0.992665	4.88E-37	238.05	S		DKEHRRPAEATSSPTSPERPRHHHHDSDSNS	0	8596300	5373
O43318	439	Mitogen-activated protein kinase kinase kinase 7	MAP3K7	0.999994	9.64E-39	237.81	S		VPEIVISGNGQPRRRSIQDLTVTGTEPGQVS	6731400	7239700	361
O60832	451	H/ACA ribonucleoprotein complex subunit 4	DKC1	1	1.49E-38	237.76	S		QVVAEAAKTAKRKRESESESDETPPAAPQLI	21836000	10343000	526
O60832	453	H/ACA ribonucleoprotein complex subunit 4	DKC1	0.999982	1.49E-38	237.76	S		VAEAAKTAKRKRESESESDETPPAAPQLIKK	19962000	9434300	527
Q53T59	194	HCLS1-binding protein 3	HS1BP3	1	4.50E-41	237.73	S		EGPPVQSLKGEDAEESLEEEEALDPLGIMRS	1779800	1530100	3145
Q9UQ35	1401	Serine/arginine repetitive matrix protein 2	SRRM2	0.999927	2.62E-39	237.66	S		LSPDAVEKAGMSSNQSISSPVLDAVPRTPSR	3816900	5095500	6598
O00161	110	Synaptosomal-associated protein 23	SNAP23	1	1.72E-38	237.39	S		SGKAYKTTWGDGGENSPCNVVSKQPGPVTNG	4760800	0	97
Q8IU81	453	Interferon regulatory factor 2-binding protein 1	IRF2BP1	1	3.24E-39	237.34	S		KDPGGGGGPVRAGGASPAASSTAQPPTQHRL	9011300	6019600	4036
Q06210	261	Glutamine--fructose-6-phosphate aminotransferase [isomerizir	GFPT1	0.992077	7.61E-38	237.07	S		RGKDKKGSCNLSRVDSTTCLFPVEEKAVEYY	15349000	16781000	2198
P46109	41	Crk-like protein	CRKL	0.720789	1.06E-38	236.75	S		TRLQGQRHGMFLVRDSSTCPGDYVLSVSENS	1151800	639310	1562
P46109	42	Crk-like protein	CRKL	0.436289	1.06E-38	236.75	S		RLQGQRHGMFLVRDSSTCPGDYVLSVSENSR	0	0	1563
Q8N3F8	620	MICAL-like protein 1	MICALL1	0.617239	4.80E-38	236.75	S		LLLVGDRSPVPSPGSSSPQLQVKSSCKENPF	0	3785000	4255
O14654	775	Insulin receptor substrate 4	IRS4	0.997601	8.05E-41	236.66	S		KAPDTNKEDDSKDNDSESDYMFMAPGAGAIP	1387900	947470	171
Q5UIP0	2161	Telomere-associated protein RIF1	RIF1	0.997717	3.44E-39	236.66	S		QKLRELDPSLVSANDSPSGMQTRCVWSPLAS	5275100	3981900	3314
Q02952	483	A-kinase anchor protein 12	AKAP12	1	8.09E-41	236.6	S		ETCVSGEDPTQGADLSPDEKVLSKPPEGVVS	503540	692860	2112
Q15054	307	DNA polymerase delta subunit 3	POLD3	1	5.27E-37	236.31	S		KVLQKEKKRGKRVALSDDETKETENMRKKRR	902140	1006900	2869
Q9UHY1	2	Nuclear receptor-binding protein	NRBP1	0.999991	2.56E-38	236.27	S		______________MSEGESQTVLSSGSDPK	7748000	5354000	6371
P17706	304	Tyrosine-protein phosphatase non-receptor type 2	PTPN2	0.999999	8.28E-38	236.05	S		WKELSKEDLSPAFDHSPNKIMTEKYNGNRIG	5324600	5439400	1119
P49756	677	RNA-binding protein 25	RBM25	1	1.42E-37	235.93	S		HRPKIGLSLKLGASNSPGQPNSVKRKKLPVD	44199000	20733000	1684
O14757	296	Serine/threonine-protein kinase Chk1	CHEK1	1	5.28E-41	235.85	S		GGVSESPSGFSKHIQSNLDFSPVNSASSEEN	3679100	2787300	208
Q12789	1865	General transcription factor 3C polypeptide 1	GTF3C1	1	5.28E-41	235.85	S		QAPPSHSPRGTKRRASWASENGETDAEGTQM	3590500	1280800	2297
P35658	433	Nuclear pore complex protein Nup214	NUP214	0.992449	1.53E-37	235.78	S		ERLSLEGERQPKSPGSTPTTPTSSQAPQKLD	7749400	909290	1418
Q9UKX7	221	Nuclear pore complex protein Nup50	NUP50	0.993853	1.40E-37	235.78	S		RNSESESNKVAAETQSPSLFGSTKLQQESTF	4507300	3808400	6462
P35658	437	Nuclear pore complex protein Nup214	NUP214	0.972123	1.53E-37	235.78	T		LEGERQPKSPGSTPTTPTSSQAPQKLDASAA	5729000	2103200	7364
Q9H6F5	91	Coiled-coil domain-containing protein 86	CCDC86	1	2.36E-44	235.76	S		AGLESPQGQPEPGAASPQRQQDLHLESPQRQ	152360000	104190000	5731
Q9H307	381	Pinin	PNN	1	4.30E-41	235.75	S		MEEETEVRESEKQQDSQPEEVMDVLEMVENV	1956100	5338100	5634
Q7Z417	629	Nuclear fragile X mental retardation-interacting protein 2	NUFIP2	0.996934	2.17E-41	235.61	S		FLSKDYEIESQNPLASPTNTLLGSAKEQRYQ	2622100	2631500	3793
Q8N163	675	Cell cycle and apoptosis regulator protein 2	CCAR2	1	3.76E-39	235.54	S		EEEFAGAKLEDSEVRSVASNQSEMEFSSLQD	7455000	5473400	4213
Q8N163	678	Cell cycle and apoptosis regulator protein 2	CCAR2	1	3.76E-39	235.54	S		FAGAKLEDSEVRSVASNQSEMEFSSLQDMPK	5903500	5473400	4214
Q96S94	330	Cyclin-L2	CCNL2	0.999867	5.55E-41	235.21	S		LLPGGTQVLDGTSGFSPAPKLVESPKEGKGS	1605000	1150800	5118
Q86WB0	335	Nuclear-interacting partner of ALK	ZC3HC1	0.994608	1.29E-37	235.02	S		ESPRRMMTRSQDATFSPGSEQAEKSPGPIVS	1230800	1701600	3984
Q9Y6X4	350	Soluble lamin-associated protein of 75 kDa	FAM169A	0.792225	1.31E-37	234.82	S		RPKIGKRFQDSEFSSSQGEDEKTSQTSLTAS	231920	0	7029
Q13895	98	Bystin	BYSL	1	1.25E-38	234.62	S		RERTTRLGPRMPQDGSDDEDEEWPTLEKAAT	12755000	11808000	2628
Q96F63	337	Coiled-coil domain-containing protein 97	CCDC97	1	5.61E-14	234.61	S		EEERYFDEEEPEDAPSPELDGD_________	1768300	2073900	4901
O15173	90	Membrane-associated progesterone receptor component 2	PGRMC2	0.649368	4.05E-39	234.51	S		WGRRGLGAGAGAGEESPATSLPRMKKRDFSL	0	0	299
Q14247	405	Src substrate cortactin	CTTN	1	5.34E-37	234.51	S		KLEEQARAKTQTPPVSPAPQPTEERLPSSPV	23408000	18613000	2732
Q9BTU6	47	Phosphatidylinositol 4-kinase type 2-alpha	PI4K2A	0.999995	3.37E-39	234.51	S		PGGAVRVAAAAGSGPSPPGSPGHDRERQPLL	26500000	21495000	5368
Q9BTU6	51	Phosphatidylinositol 4-kinase type 2-alpha	PI4K2A	1	3.37E-39	234.51	S		VRVAAAAGSGPSPPGSPGHDRERQPLLDRAR	25812000	20900000	5369
Q9UEY8	677	Gamma-adducin	ADD3	0.99757	8.31E-38	234.51	S		IKSPEKIEEVLSPEGSPSKSPSKKKKKFRTP	21383000	18961000	6300
Q14247	401	Src substrate cortactin	CTTN	0.996119	3.15E-37	234.51	T		EARRKLEEQARAKTQTPPVSPAPQPTEERLP	25164000	22260000	7595
Q9BUR4	90	Telomerase Cajal body protein 1	WRAP53	0.999175	3.07E-59	234.37	S		LSTPLETEFGSPSELSPRIEEQELSENTSLP	12408000	14754000	5385
Q9NZT2	577	Opioid growth factor receptor	OGFR	0.999613	2.69E-45	234.37	S		PGPRPAGPAGDEPAESPSETPGPSPAGPTRD	6847700	6805800	6175
Q9NZT2	585	Opioid growth factor receptor	OGFR	0.99999	2.69E-45	234.37	S		AGDEPAESPSETPGPSPAGPTRDEPAESPSE	14468000	10765000	6176
Q5JTD0	545	Tight junction-associated protein 1	TJAP1	0.999991	5.56E-37	234.36	S		RSPKRMGVHHLHRKDSLTQAQEQGNLLN___	4909900	3214700	3196
Q8NB49	445	Phospholipid-transporting ATPase IG	ATP11C	0.995225	5.90E-41	234.36	S		DGHKYKGVTQEVDGLSQTDGTLTYFDKVDKN	1508100	720790	4312
Q04726	285	Transducin-like enhancer protein 3	TLE3	0.711865	1.67E-38	234.36	T		ENGLDKARSLKKDAPTSPASVASSSSTPSSK	0	1950100	7493
O43598	169	2'-deoxynucleoside 5'-phosphate N-hydrolase 1	DNPH1	1	1.29E-38	234.12	S		LDRYFEADPPGQVAASPDPTT__________	1024500	566190	403
Q8IWQ3	423	Serine/threonine-protein kinase BRSK2	BRSK2	0.908574	1.07E-38	234.12	S		QRSRSISGASSGLSTSPLSSPRVTPHPSPRG	4578900	2265300	4067
Q8IWQ3	427	Serine/threonine-protein kinase BRSK2	BRSK2	0.990748	1.07E-38	234.12	S		SISGASSGLSTSPLSSPRVTPHPSPRGSPLP	3215100	1911400	4068
Q14676	376	Mediator of DNA damage checkpoint protein 1	MDC1	0.999977	1.09E-44	233.87	S		PGAPGLAHLQESQAGSDTDVEEGKAPQAVPL	45561000	38743000	2768
Q9UJ14	72	Gamma-glutamyltransferase 7;Gamma-glutamyltransferase 7 heav	GGT7	0.909164	2.26E-38	233.84	S		DPDSFLKSARLQRLPSSSSEMGSQDGSPLRE	1255400	373350	6391
Q9BX95	112	Sphingosine-1-phosphate phosphatase 1	SGPP1	1	4.06E-37	233.62	S		LGPASPRRAGALRRNSLTGEEGQLARVSNWP	12037000	0	5446
Q15019	218	Septin-2	SEPT2	1	4.56E-40	233.57	S		IEEHNIKIYHLPDAESDEDEDFKEQTRLLKA	46888000	26183000	2853
P46821	1915	Microtubule-associated protein 1B;MAP1B heavy chain;MAP1 lig	MAP1B	0.957319	1.69E-37	233.52	S		DVGGYYYEKIERTTKSPSDSGYSYETIGKTT	0	3338900	1595
Q8IXQ3	69	Uncharacterized protein C9orf40	C9orf40	0.997987	1.44E-37	233.48	S		RKRKIDAGTMAEPSASPSKRRDSGDNSAPSG	13657000	10825000	4108
P06748	70	Nucleophosmin	NPM1	1	4.36E-39	233.46	S		DELHIVEAEAMNYEGSPIKVTLATLKMSVQP	2105500	1810900	920
O15173	211	Membrane-associated progesterone receptor component 2	PGRMC2	0.998375	2.53E-36	233.38	T		YVGRLLKPGEEPSEYTDEEDTKDHNKQD___	2348100	2575300	7157
Q9UK58	335	Cyclin-L1	CCNL1	0.999994	6.32E-41	233.34	S		LNPDGTPALSTLGGFSPASKPSSPREVKAEE	16084000	2032000	6419
Q6DN90	512	IQ motif and SEC7 domain-containing protein 1	IQSEC1	0.999998	1.73E-37	233.14	S		EQTLSKQTYHKEARNSWDSPAFSNDVIRKRH	1488200	1378300	3447
Q8N1G4	518	Leucine-rich repeat-containing protein 47	LRRC47	0.997011	1.75E-49	232.87	S		MAEMKKYTLENKEEGSLSDTEADAVSGQLPD	82349000	54758000	4236
Q9UHR4	255	Brain-specific angiogenesis inhibitor 1-associated protein 2	BAIAP2L1	0.882756	3.76E-39	232.87	S		MNMIEEIKTPASTPVSGTPQASPMIERSNVV	0	0	6362
Q7Z6E9	1274	E3 ubiquitin-protein ligase RBBP6	RBBP6	0.513805	1.46E-44	232.87	T		EGSSSTLVDYTSTSSTGGSPVRKSEEKTDTK	506530	639590	7814
Q9UPW0	223	Forkhead box protein J3	FOXJ3	0.789547	1.51E-37	232.73	S		TNKVTLYNTDQDGSDSPRSSLNNSLSDQSLA	3080700	1446200	6590
P81877	321	Single-stranded DNA-binding protein 2	SSBP2	1	7.78E-38	232.62	S		NGSLGSGDMDSISKNSPNNMSLSNQPGTPRD	2396500	870660	1985
P06454	2	Prothymosin alpha;Prothymosin alpha, N-terminally processed;	PTMA	1	7.74E-38	232.37	S		______________MSDAAVDTSSEITTKDL	6105000	5252200	915
Q13428	967	Treacle protein	TCOF1	1	4.67E-39	232.37	S		AQVIKPPLIFVDPNRSPAGPAATPAQAQAAS	5000200	2901300	2519
Q9NUL3	486	Double-stranded RNA-binding protein Staufen homolog 2	STAU2	0.813841	1.54E-37	232.37	S		NGTSSTAEAIGLKGSSPTPPCSPVQPSKQLE	4846600	1053900	6023
Q5HYJ3	193	Protein FAM76B	FAM76B	1	1.83E-37	232.26	S		HHHRHSSSHHKISNLSPEEEQGLWKQSHKSS	5600200	3890400	3167
Q9Y5Q9	43	General transcription factor 3C polypeptide 3	GTF3C3	1	9.50E-44	232.11	S		RKTREKKSLQEKGKLSAEENPDDSEVPSSSG	582670	409200	6962
P10412	4	Histone H1.4	HIST1H1E	0.958067	1.45E-39	232.11	T		____________MSETAPAAPAAPAPAEKTP	888700	379810	7273
P10412	18	Histone H1.4	HIST1H1E	1	1.45E-39	232.11	T		ETAPAAPAAPAPAEKTPVKKKARKSAGAAKR	8627100	4690700	7274
Q5T1M5	956	FK506-binding protein 15	FKBP15	1	5.89E-32	231.42	S		SEEEEEKAEERPRRPSQEQSASASSGQPQAP	1029900	846550	3251
O75533	350	Splicing factor 3B subunit 1	SF3B1	0.995252	6.50E-32	231.42	T		KSRWDETPASQMGGSTPVLTPGKTPIGTPAM	5891900	3261800	7214
Q9BVG9	24	Phosphatidylserine synthase 2	PTDSS2	1	6.11E-33	231.41	S		AGGPRPESPVPAGRASLEEPPDGPSAGQATG	406450	1120200	5401
Q9H3Z4	10	DnaJ homolog subfamily C member 5	DNAJC5	0.955427	3.01E-30	231.41	S		______MADQRQRSLSTSGESLYHVLGLDKN	4293100	1010400	5650
Q9UKN8	611	General transcription factor 3C polypeptide 4	GTF3C4	1	7.96E-36	231.41	S		LWKPTHEDSKILLVDSPGMGNADDEQQEEGT	15184000	13882000	6449
O75781	138	Paralemmin-1	PALM	0.676662	4.64E-29	231.4	S		PSPAKEERKTEVVMNSQQTPVGTPKDKRVSN	0	0	663
O75533	354	Splicing factor 3B subunit 1	SF3B1	1	6.50E-32	231.4	T		DETPASQMGGSTPVLTPGKTPIGTPAMNMAT	4131700	3261800	7215
Q13615	613	Myotubularin-related protein 3	MTMR3	0.999979	2.06E-30	231.38	S		TSPDDPPLSRLPKTRSYDNLTTACDNTVPLA	4968200	3450400	2606
O14654	817	Insulin receptor substrate 4	IRS4	0.81147	7.00E-31	231.37	S		SKSWSSYFSLPNPFRSSPLGQNDNSEYVPML	1589500	1066300	180
O14654	818	Insulin receptor substrate 4	IRS4	0.5	7.00E-31	231.37	S		KSWSSYFSLPNPFRSSPLGQNDNSEYVPMLP	1589500	1066300	181
P36507	295	Dual specificity mitogen-activated protein kinase kinase 2	MAP2K2	0.994872	6.81E-30	231.37	S		GRPVVDGEEGEPHSISPRPRPPGRPVSGHGM	2848800	4216100	1428
Q9Y2W1	248	Thyroid hormone receptor-associated protein 3	THRAP3	1	6.46E-29	231.37	S		ASRASAVSELSPRERSPALKSPLQSVVVRRR	5481100	3426100	6817
Q9Y2W1	253	Thyroid hormone receptor-associated protein 3	THRAP3	1	6.46E-29	231.37	S		AVSELSPRERSPALKSPLQSVVVRRRSPRPS	16076000	9287400	6818
Q9HCK8	549	Chromodomain-helicase-DNA-binding protein 8	CHD8	0.906083	2.86E-29	231.21	S		NTITPVVGKKRKRNTSSDNSDVEVMPAQSPR	134690	1304300	5865
Q9HCK8	548	Chromodomain-helicase-DNA-binding protein 8	CHD8	0.604381	2.86E-29	231.21	T		LNTITPVVGKKRKRNTSSDNSDVEVMPAQSP	134690	1304300	8133
Q8N1G4	520	Leucine-rich repeat-containing protein 47	LRRC47	0.99817	3.16E-40	231.04	S		EMKKYTLENKEEGSLSDTEADAVSGQLPDPT	12630000	12797000	4237
Q9UFC0	245	Leucine-rich repeat and WD repeat-containing protein 1	LRWD1	0.944908	6.93E-29	230.96	S		AALKRPDDVPLSLSPSKRACASPSAQVEGSP	0	1755500	6312
Q9H814	349	Phosphorylated adapter RNA export protein	PHAX	0.922348	9.14E-29	230.69	T		MKQAIKSLNFQEDDDTSRETFASDTNEALAS	2933400	0	8121
O95817	171	BAG family molecular chaperone regulator 3	BAG3	0.963821	6.02E-35	230.66	S		AAAAAQPPASHGPERSQSPAASDCSSSSSSA	1812000	825520	824
O95817	173	BAG family molecular chaperone regulator 3	BAG3	0.499998	6.02E-35	230.66	S		AAAQPPASHGPERSQSPAASDCSSSSSSASL	1812000	825520	825
Q13435	307	Splicing factor 3B subunit 2	SF3B2	0.999626	2.61E-30	230.62	S		EEEEMETDARSSLGQSASETEEDTVSVSKKE	971350	2411400	2527
Q13435	309	Splicing factor 3B subunit 2	SF3B2	0.998552	2.61E-30	230.62	S		EEMETDARSSLGQSASETEEDTVSVSKKEKN	971350	2898500	2528
O75376	158	Nuclear receptor corepressor 1	NCOR1	0.896583	5.24E-35	230.61	S		AKKDPAFGGKHEAPSSPISGQPCGDDQNASP	934960	395690	596
O75376	172	Nuclear receptor corepressor 1	NCOR1	0.908157	5.24E-35	230.61	S		SSPISGQPCGDDQNASPSKLSKEELIQSMDR	8954300	5391200	597
Q07021	213	Complement component 1 Q subcomponent-binding protein, mitoc	C1QBP	0.791197	2.72E-29	230.57	S		EAESDIFSIREVSFQSTGESEWKDTNYTLNT	0	1257600	2214
P35251	190	Replication factor C subunit 1	RFC1	0.999995	1.09E-29	230.48	S		QRSNKKMVASKRKELSQNTDESGLNDEAIAK	285780	1302100	1383
Q7Z5L9	455	Interferon regulatory factor 2-binding protein 2	IRF2BP2	1	5.05E-29	230.45	S		ASKDANQVHSTTRRNSNSPPSPSSMNQRRLG	5485600	3313300	3851
Q7Z5L9	460	Interferon regulatory factor 2-binding protein 2	IRF2BP2	0.999782	5.05E-29	230.45	S		NQVHSTTRRNSNSPPSPSSMNQRRLGPREVG	5485600	11812000	3853
Q14692	639	Ribosome biogenesis protein BMS1 homolog	BMS1	0.988417	1.30E-29	230.27	S		SSGQKLGPQNFIDETSDIENLLKEEEDYKEE	474270	175690	2799
O00203	276	AP-3 complex subunit beta-1	AP3B1	1	1.53E-28	230.23	S		KEGDELEDNGKNFYESDDDQKEKTDKKKKPY	1915700	852460	106

Q14566	762	DNA replication licensing factor MCM6	MCM6	0.999998	6.72E-29	230.23	S	ELVNWYLKEIESEIDSEEELINKKRIIEKVI	106570	928370	2745
Q92797	1169	Symplekin	SYMPK	0.351902	5.21E-31	230	S	EEEQKLKPGGVGAPSSSSPSPSPSARPGPPP	0	0	4709
Q92797	1171	Symplekin	SYMPK	0.898862	5.21E-31	230	S	EQKLKPGGVGAPSSSSPSPSPSARPGPPPSE	2418000	20741000	4711
Q92797	1175	Symplekin	SYMPK	0.999023	5.21E-31	230	S	KPGGVGAPSSSSPSPSPSARPGPPPSEEAMD	23664000	17475000	4713
Q96A47	279	Insulin gene enhancer protein ISL-2	ISL2	1	1.60E-29	229.96	S	KTSLQGLTGTPLVAGSPIRHENAVQGSAVEV	664030	378980	4767
Q8NE71	108	ATP-binding cassette sub-family F member 1	ABCF1	1	3.05E-27	229.92	T	EEKELMERLKKLSVPTSDEEDEVPAPKPRGG	10031000	2623600	7893
Q14676	168	Mediator of DNA damage checkpoint protein 1	MDC1	1	3.19E-29	229.69	S	VQGETQPQRLLLAEDSEEEVDFLSERRMVKK	837700	773740	2770
Q14677	299	Clathrin interactor 1	CLINT1	1	3.19E-29	229.69	S	TIDLGAAAHYTGDKASPDQNASTHTPQSSVK	2709300	1306900	2772
Q70Z53	283	Protein FRA10AC1	FRA10AC1	0.999371	7.10E-12	229.62	S	KKSEDSLLRNSDEEESASESELWKGPLPETD	0	0	3691
Q9Y3X0	386	Coiled-coil domain-containing protein 9	CCDC9	0.970309	1.40E-31	229.62	S	KEGAASPAPETPQPTSPETSPKETPMQPPEI	4141000	3365700	6882
Q9Y3X0	390	Coiled-coil domain-containing protein 9	CCDC9	0.994962	1.40E-31	229.62	S	ASPAPETPQPTSPETSPKETPMQPPEIPAPA	172710	1663400	6883
O43719	642	HIV Tat-specific factor 1	HTATSF1	1	9.32E-29	229.48	S	KEEEEDTYEKVFDDESDEKEDEEYADEKGLE	979350	1112500	414
O60343	757	TBC1 domain family member 4	TBC1D4	0.980703	5.31E-30	229.46	S	DGEGRKRTSSTCSNESLSVGGTSVTPRRISW	714710	1166900	499
Q9UN36	332	Protein NDRG2	NDRG2	0.999844	6.51E-28	229.22	S	MASSCMTRLSRSRTASLTSAASVDGNRSRSR	7108400	4152100	6527
Q9UN36	330	Protein NDRG2	NDRG2	0.999999	6.51E-28	229.22	T	GYMASSCMTRLSRSRTASLTSAASVDGNRSR	1892000	988930	8242
P12270	1185	Nucleoprotein TPR	TPR	0.999886	8.05E-29	229.02	S	VVASVKEGVQGPLNVSLSEEGKSQEQILEIL	166920	1080800	1038
Q99956;Q	328;331;	Dual specificity protein phosphatase 9;Dual specificity prot	DUSP9;DUSP	0.955567	2.69E-29	228.85	S	LNDAYDLVKRKKSNISPNFNFMGQLLDFERS	3080800	1054800	5285
Q13595	2	Transformer-2 protein homolog alpha	TRA2A	1	1.07E-28	228.7	S	______________MSDVEENNFEGRESRSQ	3234600	6278900	2588
Q8IYB3	220	Serine/arginine repetitive matrix protein 1	SRRM1	1	1.35E-26	228.7	T	TKSRSPSPAPEKKEKTPELPEPSVKVKEPSV	6416500	4874500	7859
Q96GX5	453	Serine/threonine-protein kinase greatwall	MASTL	0.983291	2.56E-28	228.62	S	LGKRSLKRNFELVDSSPCKKIIQNKKTCVEY	1877500	1029600	4927
P08651	323	Nuclear factor 1 C-type	NFIC	0.972527	1.54E-47	228.49	S	SSSRNWTEDMEGGISSPVKKTEMDKSPFNSP	2012300	1788900	960
Q9Y6D5	276	Brefeldin A-inhibited guanine nucleotide-exchange protein 2	ARFGEF2	0.499997	9.70E-29	228.49	S	GKVSTENGDAPRERGSSLSGTDDGAQEVVKD	0	1962200	6995
Q9Y6D5	277	Brefeldin A-inhibited guanine nucleotide-exchange protein 2	ARFGEF2	0.499997	9.70E-29	228.49	S	KVSTENGDAPRERGSSLSGTDDGAQEVVKDI	0	1962200	6996
Q13017	1218	Rho GTPase-activating protein 5	ARHGAP5	0.944378	5.50E-29	228.32	S	PVETWKGGIDNPAITSDQELDDKKMKKKTHK	1826200	1298300	2388
Q9H8G2	312	Caspase activity and apoptosis inhibitor 1	CAAP1	0.998965	5.50E-29	228.32	S	DIEKSVNEILGLAESSPNEPKAATLAVPPPE	1124500	1768500	5786
Q13428	674	Treacle protein	TCOF1	0.99243	3.84E-31	228.26	S	VRVGTQAPRKAGTATSPAGSSPAVAGGTQRP	4103600	4303600	2504
P29966	29	Myristoylated alanine-rich C-kinase substrate	MARCKS	0.944839	4.93E-29	228.19	S	AAAERPGEAAVASSPSKANGQENGHVKVNGD	9772000	0	1337
O43166	1549	Signal-induced proliferation-associated 1-like protein 1	SIPA1L1	0.999127	9.15E-29	228.17	S	KSTIEEDLKKLIDLESPTPESQKSFKFHALS	1892000	923060	343
Q8N7R7	344	Cyclin-Y-like protein 1	CCNYL1	0.999995	8.57E-29	228.17	S	CEDKDLCRAAMRRSFSADNFIGIQRSKAILS	1886500	2121800	4297
P52756	624	RNA-binding protein 5	RBM5	0.999824	1.12E-29	228.11	S	ENPLKRGLVAAYSGDSDNEEELVERLESEEE	4281100	4215100	1823
P23508	294	Colorectal mutant cancer protein	MCC	0.992551	1.96E-28	227.91	S	ELQTRLQSVQATGPSSPGRLTSTNRPINPST	8085300	6436400	1224
Q8N3X1	432	Formin-binding protein 4	FNBP4	0.99255	1.82E-28	227.91	S	ELRALEEGDGSVSGSSPRSDISQPASQDGMR	3972000	1560000	4261
Q14498	136	RNA-binding protein 39	RBM39	1	8.61E-28	227.74	S	SRRRSRSKSPFRKDKSPVREPIDNLTPEERD	57783000	35294000	2737
Q9NZJ0	511	Denticleless protein homolog	DTL	0.733103	9.64E-28	227.63	S	SFKMSIRNWVTRTPSSSPPITPPASETKIMS	0	0	6156
Q9NZJ0	516	Denticleless protein homolog	DTL	0.999999	9.64E-28	227.63	T	IRNWVTRTPSSSPPITPPASETKIMSPRKAL	3477400	2091900	8181
P22626	259	Heterogeneous nuclear ribonucleoproteins A2/B1	HNRNPA2B1	1	1.18E-39	227.43	S	YNGYGGGPGGGNFGGSPGYGGGRGGYGGGGP	23645000	12637000	1213
Q99733	7	Nucleosome assembly protein 1-like 4	NAP1L4	0.999999	1.75E-29	227.41	S	_________MADHSFSDGVPSDSVEAAKNAS	5410300	2874500	5269
Q13286	12	Battenin	CLN3	1	1.07E-27	227.09	S	____MGGCAGSRRRFSDSEGEETVPEPRLPL	831350	802760	2467
Q13286	14	Battenin	CLN3	1	1.07E-27	227.09	S	__MGGCAGSRRRFSDSEGEETVPEPRLPLLD	1369400	1561100	2468
Q9BQG0	1267	Myb-binding protein 1A	MYBBP1A	0.999982	6.12E-52	227.09	S	QKKNQKPSQVNGAPGSPTEPAGQKQHQKALP	4468600	1922800	5311
O43524	284	Forkhead box protein O3	FOXO3	0.946466	1.50E-29	226.93	S	KKKAALQTAPESADDSPSQLSKWPGSPTSRS	1181600	687150	396
Q92797	1243	Symplekin	SYMPK	0.999968	2.49E-28	226.93	S	PKETAAGGLTLKEERSPQTLAPVGEDAMKTP	20300000	10881000	4715
Q06587	229	E3 ubiquitin-protein ligase RING1	RING1	0.999985	1.20E-45	226.82	S	GTGGGGTGGVGGGAGSEDSGDRGGTLGGGTL	2870300	1611000	2204
Q12873	1601	Chromodomain-helicase-DNA-binding protein 3	CHD3	1	8.15E-29	226.74	S	NSRIGEKMETEADAPSPAPSLGERLEPRKIP	3582700	2225600	2337
Q5W0B1	438	RING finger protein 219	RNF219	1	1.73E-47	226.65	S	LRKLVFDDFCDSSNVSNKDSSEDDISRSENE	0	0	3376
Q9UBF8	428	Phosphatidylinositol 4-kinase beta	PI4KB	1	2.58E-28	226.39	S	SVPARIPENRIRSTRSVENLPECGITHEQRA	13490000	12860000	6265
Q8WXF1	477	Paraspeckle component 1	PSPC1	0.999943	1.68E-28	226.22	S	VHNDRFPQGPPSQMGSPMGSRTGSETPQAPM	1303400	856010	4592
Q9UHB7	1043	AF4/FMR2 family member 4	AFF4	0.999992	1.68E-28	226.22	S	LTEHLKNSYNNSQAPSPGLGSKAVGMPSPVS	3833300	2272000	6345
Q14692	638	Ribosome biogenesis protein BMS1 homolog	BMS1	0.90677	2.15E-28	226.22	T	VSSGQKLGPQNFIDETSDIENLLKEEEDYKE	1520500	0	7617
P61978	216	Heterogeneous nuclear ribonucleoprotein K	HNRNPK	1	1.38E-28	226.09	S	RVVECIKIILDLISESPIKGRAQPYDPNFYD	2189900	1856500	1915
Q9UNS2	410	COP9 signalosome complex subunit 3	COPS3	0.949292	4.17E-28	226.08	S	QEITVNPQFVQKSMGSQEDDSGNKPSSYS__	0	238030	6550
P17987	551	T-complex protein 1 subunit alpha	TCP1	1	2.74E-28	226.06	S	ESKDDKHGSYEDAVHSGALND__________	1461000	950200	1126
Q14137	126	Ribosome biogenesis protein BOP1	BOP1	1	7.26E-12	226.06	S	TEMASARIGDEYAEDSSDEEDIRNTVGNVPL	0	0	2649
Q14137	127	Ribosome biogenesis protein BOP1	BOP1	1	7.26E-12	226.06	S	EMASARIGDEYAEDSSDEEDIRNTVGNVPLE	0	0	2650
Q9UFC0	243	Leucine-rich repeat and WD repeat-containing protein 1	LRWD1	0.991814	6.07E-28	226.06	S	RLAALKRPDDVPLSLSPSKRACASPSAQVEG	3489200	0	6311
Q9UKL0	260	REST corepressor 1	RCOR1	1	2.89E-35	225.88	S	VMDRHARKQKREREESEDELEEANGNNPIDI	1216000	963670	6447
P46821	1389	Microtubule-associated protein 1B;MAP1B heavy chain;MAP1 lig	MAP1B	0.997704	1.87E-29	225.79	S	RASVSPMDEPVPDSESPIEKVLSPLRSPPLI	2265000	2508900	1573
Q14980	1757	Nuclear mitotic apparatus protein 1	NUMA1	0.992857	1.93E-29	225.6	S	PRTQPDGTSVPGEPASPISQRLPPKVESLES	8393300	6005700	2829
Q5JSH3	96	WD repeat-containing protein 44	WDR44	0.999628	3.20E-28	225.6	S	SLDSKGKELSDQATASPIVARTDLSNIPGLL	5805600	3770800	3181
Q92785	142	Zinc finger protein ubi-d4	DPF2	1	2.97E-28	225.6	S	PLEKRGAPDPRVDDDSLGEFPVTNSRARKRI	4692100	2869000	4701
Q04726	203	Transducin-like enhancer protein 3	TLE3	0.967161	3.08E-28	225.37	S	ELDHRERESSANNSVSPSESLRASEKHRGSA	21738000	20183000	2173
Q9NYF8	198	Bcl-2-associated transcription factor 1	BCLAF1	0.999788	1.95E-28	225.37	S	SQEEPKDTFEHDPSESIDEFNKSSATSGDIW	8155200	2648800	6105
Q14684	731	Ribosomal RNA processing protein 1 homolog B	RRP1B	0.985505	2.78E-28	225.33	S	DPEQKPLHGVLKTPTSSPASSPLVAKKPLTT	0	6203800	2784
Q14684	735	Ribosomal RNA processing protein 1 homolog B	RRP1B	0.720934	2.78E-28	225.33	S	KPLHGVLKTPTSSPASSPLVAKKPLTTTPRR	7052500	0	2786
P40818	718	Ubiquitin carboxyl-terminal hydrolase 8	USP8	0.951146	2.01E-28	225.17	S	PAERDREPSKLKRSYSSPDITQAIQEEEKRK	1772500	0	1477
Q13459	1290	Unconventional myosin-IXb	MYO9B	0.999939	9.06E-28	225.17	S	KSKPCGSPRVQEKPDSPGGSTQIQRYLDAER	1418900	1476300	2540
Q99956	193	Dual specificity protein phosphatase 9	DUSP9	1	3.74E-30	224.99	T	ADRDSMSCGLDSEGATPPPVGLRASFPVQIL	1868100	1166800	8034
Q9C0C9	839	E2/E3 hybrid ubiquitin-protein ligase UBE2O	UBE2O	0.966193	1.37E-33	224.8	S	LKNMTVEQLLTGSPTSPTVEPEKPTREKKFL	18814000	23909000	5536
P29375	1111	Lysine-specific demethylase 5A	KDM5A	1	6.83E-29	224.64	S	LIEKEKEKDLDLEPLSDLEEGLEETRDTAMV	659100	1112700	1317
Q9P035	114	Very-long-chain (3R)-3-hydroxyacyl-CoA dehydratase 3	HACD3	1	1.79E-28	224.43	S	RPLFLAPDFDRWLDESDAEMELRAKEEERLN	6048800	5027100	6179
P18615	49	Negative elongation factor E	NELFE	0.5	7.06E-29	224.4	S	KQSSSSTTSQGGVKRSLSEQPVMDTATATEQ	1775600	0	1141
P13051	64	Uracil-DNA glycosylase	UNG	0.958517	3.63E-33	224.38	S	KKAPAGQEEPGTPPSSPLSAEQLDRIQRNKA	6271600	3592600	1047
P13051	60	Uracil-DNA glycosylase	UNG	0.999998	3.63E-33	224.38	T	AIPAKKAPAGQEEPGTPPSSPLSAEQLDRIQ	9650700	5441300	7283
O43237	194	Cytoplasmic dynein 1 light intermediate chain 2	DYNC1LI2	1	7.34E-29	224.12	S	KDFQDYMEPEEGCQGSPQRRGPLTSGSDEEN	15192000	8541500	346
Q96T60	118	Bifunctional polynucleotide phosphatase/kinase;Polynucleotic	PNKP	0.999804	4.23E-30	224.12	T	TLRWEETRTPESQPDTPPGTPLVSQDEKRDA	581410	335150	8017
Q13045	856	Protein flightless-1 homolog	FLII	1	4.02E-28	224.06	S	VLTVDYTRNAEAVLQSPGLSGKVKRDAEKKD	4658800	3090200	2393
Q53HL2	219	Borealin	CDCA8	0.999989	3.73E-28	224.06	S	TPAAGERIYNISGNGSPLADSKEIFLTVPVG	2456300	1355100	3142
Q09666	216	Neuroblast differentiation-associated protein AHNAK	AHNAK	0.994412	7.53E-29	223.93	S	SGKTVIRLPSGSGAASPTGSAVDIRAGAISA	7678900	4739600	2280
Q7Z5L9	457	Interferon regulatory factor 2-binding protein 2	IRF2BP2	1	4.36E-28	223.93	S	KDANQVHSTTRRNSNSPPSPSSMNQRRLGPR	13219000	1881800	3852
Q03164	3511	Histone-lysine N-methyltransferase 2A;MLL cleavage product N	KMT2A	0.996468	1.67E-34	223.91	S	EQNKALSSAVQASPTSPGGSPSSPSSGQRSA	10597000	7914700	2131
Q03164	3517	Histone-lysine N-methyltransferase 2A;MLL cleavage product N	KMT2A	0.738722	1.67E-34	223.91	S	SSAVQASPTSPGGSPSSPSSGQRSASPSVPG	312810	3171600	2133
O75122	596	CLIP-associating protein 2	CLASP2	0.999703	1.31E-28	223.82	S	RLSSSVSAMRVLNTGSDVEEAVADALKKPAR	1001700	1187600	565
Q14653	173	Interferon regulatory factor 3	IRF3	0.825253	6.64E-31	223.82	S	PSLAVAPEPCPQPLRSPSLDNPTPFPNLGPS	1883800	1296200	2747
Q14653	175	Interferon regulatory factor 3	IRF3	0.5	6.64E-31	223.82	S	LAVAPEPCPQPLRSPSLDNPTPFPNLGPSEN	1883800	1296200	2748
Q96CX2	196	BTB/POZ domain-containing protein KCTD12	KCTD12	0.995926	5.25E-30	223.7	T	LASRSPSGGAAGPLLTPSQSLDGSRRSGYIT	678980	940030	7973
P54105	102	Methylosome subunit pICln	CLNS1A	1	2.74E-31	223.65	S	EEESKEPVADEEEEDSDDDVEPITEFRFVPS	19885000	16250000	1840
Q8NEF9	203	Serum response factor-binding protein 1	SRFBP1	0.999617	2.00E-27	223.29	S	IAKMEHGPKAVTIANSPSKPSEKDSVVSLES	1815000	1156700	4369
Q9BVV8	120	Uncharacterized membrane protein C19orf24	C19orf24	0.999463	6.65E-39	223.15	S	LLANTEDPTEMASLDSDEETVFESRNLR___	866490	11008000	5410
P61978	379	Heterogeneous nuclear ribonucleoprotein K	HNRNPK	1	2.73E-29	223.11	S	QGGSGYDYSYAGGRGSYGDLGGPIITTQVTI	2406100	1535400	1914
Q99717	463	Mothers against decapentaplegic homolog 5	SMAD5	0.998682	2.25E-28	222.96	S	DKVLTQMGSPLNPISSVS_____________	5988200	4923400	5262
Q99717	465	Mothers against decapentaplegic homolog 5	SMAD5	1	2.25E-28	222.96	S	VLTQMGSPLNPISSVS_______________	5988200	4923400	5263
Q99504	269	Eyes absent homolog 3	EYA3	0.943406	1.13E-28	222.96	T	LSSGDPSTSPSLSQTTPSKDTDDQSRKNMTS	1151500	1568300	8025
Q9BVW5	222	TIMELESS-interacting protein	TIPIN	0.818894	8.51E-29	222.93	S	KQLALERRQAKLLSNSQTLGNDMLMNTPRAH	455320	930380	5411
Q9UQ35	866	Serine/arginine repetitive matrix protein 2	SRRM2	1	3.47E-28	222.93	T	RQGSITSPQANEQSVTPQRRSCFESSPDPEL	5744600	5238100	8265
O43719	714	HIV Tat-specific factor 1	HTATSF1	1	7.66E-28	222.73	S	DDDSNEKLFDEEEDSSEKLFDDSDERGTLGG	3514400	5425800	411
O14979	241	Heterogeneous nuclear ribonucleoprotein D-like	HNRNPDL	0.999977	2.80E-28	222.64	S	LKGKEPPKKVFVGGLSPDTSEEQIKEYFGAF	1345600	968430	222
Q1KMD3	161	Heterogeneous nuclear ribonucleoprotein U-like protein 2	HNRNPUL2	1	4.83E-28	222.56	S	EQGLGKREEDEPEERSGDETPGSEVPGDKAA	1169500	2389800	3032
Q58WW2	336	DDB1- and CUL4-associated factor 6	DCAF6	1	2.42E-28	222.55	S	PRARPESERERDGEQSPNVSLMQRMSDMLSR	3576600	4497200	3150
Q8NEM2	42	SHC SH2 domain-binding protein 1	SHCBP1	0.992012	3.47E-09	222.51	S	QELASLEKGLFQDEDSCSDCSYRDKPGSSLQ	0	0	4373
Q9NW82	638	WD repeat-containing protein 70	WDR70	1	4.90E-28	222.42	S	AYSKTQPKTMFAQVESDDEEAKNEPEWKKRK	8012700	3881300	6055
O43524	286	Forkhead box protein O3	FOXO3	0.855106	3.05E-29	222.13	S	KAALQTAPESADDSPSQLSKWPGSPTSRSSD	0	0	397
Q86XP3	109	ATP-dependent RNA helicase DDX42	DDX42	0.964338	4.25E-34	222.13	S	ENSPTRQQFHSKPVDSDSDDDPLEAFMAEVE	648040	247430	4016
Q14680	505	Maternal embryonic leucine zipper kinase	MELK	1	2.97E-28	222.09	S	DKLMTGVISPERRCRSVELDLNQAHMEETPK	1003000	0	2775
Q14684	245	Ribosomal RNA processing protein 1 homolog B	RRP1B	1	3.12E-29	221.9	S	ETMEEQKTKVGDGDLSAEEIPENEVSLRRAV	4286800	3682400	2792
Q8IWI9	2088	MAX gene-associated protein	MGA	0.911147	1.63E-28	221.9	S	VKVEQQKGFDNPEENSSEFPVTFKEESKFEL	574320	741030	4057
Q9Y485	1970	DmX-like protein 1	DMXL1	1	1.64E-28	221.81	S	PSVVFQDDSLELKWDSDNDEENEDVPISMKE	2736500	2723100	6900
O15173	93	Membrane-associated progesterone receptor component 2	PGRMC2	0.887455	1.28E-28	221.81	T	RGLGAGAGAGEESPATSLPRMKKRDFSLEQL	588070	529420	7156
Q6UUV7	370	CREB-regulated transcription coactivator 3	CRTC3	0.876152	3.25E-29	221.51	S	PNASALHPSLRLFSLSNPSLSTTNLSGPSRR	639280	633570	3590
O43318	454	Mitogen-activated protein kinase kinase kinase 7	MAP3K7	0.810026	8.29E-29	221.44	S	SIQDLTVTGTEPGQVSSRSSSPSVRMITTSG	977950	0	362
P30086	54	Phosphatidylethanolamine-binding protein 1;Hippocampal choli	PEBP1	0.964073	2.29E-27	221.34	S	KVLTPTQVKNRPTSISWDGLDSGKLYTLVLT	0	0	1342
Q9UIF9	496	Bromodomain adjacent to zinc finger domain protein 2A	BAZ2A	0.999929	2.82E-29	221.05	S	SLEVPLTASVTSPKASPVTSPAAAFPTASPA	6325900	3994100	6380
Q9UIF9	509	Bromodomain adjacent to zinc finger domain protein 2A	BAZ2A	0.999275	2.82E-29	221.05	S	KASPVTSPAAAFPTASPANKDVSSFLETTAD	4133400	2232800	6382
P55081	267	Microfibrillar-associated protein 1	MFAP1	1	2.78E-33	221.05	T	LEENKRSLAALDALNTDDENDEEEYEAWKVR	25730000	13244000	7450
Q96NB3	351	Zinc finger protein 830	ZNF830	1	3.68E-31	220.91	S	LTIKELQKKEEENADSDDEGELQDLLSQDWR	4806400	2426200	5041
Q9BU76	216	Multiple myeloma tumor-associated protein 2	MMTAG2	0.711164	3.18E-27	220.89	S	KKKKDKEHRRPAEATSSPTSPERPRHHHHDS	0	31171000	5371
Q03164	3518	Histone-lysine N-methyltransferase 2A;MLL cleavage product N	KMT2A	0.904929	2.32E-34	220.88	S	SAVQASPTSPGGSPSSPSSGQRSASPSVPGP	0	0	2134
O43823	339	A-kinase anchor protein 8	AKAP8	1	8.19E-28	220.78	S	EGDFSENDDAAGDFRSGDEEFKGEDELCDSG	0	423450	429
P20265	341	POU domain, class 3, transcription factor 2	POU3F2	0.901932	1.82E-28	220.78	S	PLLNKWLEEADSSSGSPTSIDKIAAQGRKRK	1020400	1110200	1181
Q8WVB6	871	Chromosome transmission fidelity protein 18 homolog	CHTF18	1	4.99E-34	220.5	S	AEASARVENSPQVDGSPPGLEGLLGGIGEKG	5835800	4357600	4536
Q9UQ35	377	Serine/arginine repetitive matrix protein 2	SRRM2	0.999998	3.40E-39	220.46	S	EPPAPTPLLAERHGGSPQPLATTPLSQEPVN	111830000	111110000	6640
Q9UQ35	398	Serine/arginine repetitive matrix protein 2	SRRM2	0.961757	3.40E-39	220.46	S	TTPLSQEPVNPPSEASPTRDRSPPKSPEKLP	128160000	111110000	6643
P22234	27	Multifunctional protein ADE2;Phosphoribosylaminoimidazole-su	PAICS	1	9.75E-28	220.31	S	KLYEGKTKEVYELLDSPGKVLLQSKDQITAG	8564200	5776700	1208
P35568	348	Insulin receptor substrate 1	IRS1	0.999143	5.62E-28	220.27	S	EGTMSRPASVDGSPVSPSTNRTHAHRHRGSA	1234400	888360	1392
Q15678	314	Tyrosine-protein phosphatase non-receptor type 14	PTPN14	0.998859	3.04E-28	220.25	S	HKFYKQNKICTEQSNSPPPIRRQPTWSRSSL	3083300	1590500	2974
Q5THK1	1029	Protein PRR14L	PRR14L	1	1.14E-28	219.96	S	DLLVSSGSNNSLPCGSPKKCNLKGAFVKMSG	2792400	2486100	3309
P04183	13	Thymidine kinase, cytosolic	TK1	0.950706	2.47E-28	219.89	S	___MSCINLPTVLPGSPSKTRGQIQVILGPM	18137000	21768000	880
Q641Q2	1054	WASH complex subunit FAM21A	FAM21A	0.967682	3.67E-28	219.85	S	RPQTRAARRLAAQESSETEDMSVPRGPIAQW	1490400	0	3389
Q3YEC7	596	Rab-like protein 6	RABL6	1	7.90E-30	219.72	S	DTQRRADDFPVRDDPSDVTDEDEGPAEPPPP	9157200	12688000	3080
Q3YEC7	599	Rab-like protein 6	RABL6	1	5.38E-30	219.72	T	RRADDFPVRDDPSDVTDEDEGPAEPPPPPKL	8955000	12938000	7672
P27816	507	Microtubule-associated protein 4	MAP4	1	6.55E-28	219.35	S	VAPSTVKEVGLLKDMSPLSETEMALGKDVTP	4171500	4019600	1290
Q9UPN4	105	Centrosomal protein of 131 kDa	CEP131	0.997443	1.22E-28	219.23	S	PRPTEPTDFLMLFEGSPSGKKRPASLSTAPS	2657000	1371800	6561
Q9P2N6	536	KAT8 regulatory NSL complex subunit 3	KANSL3	0.992217	6.65E-34	219.21	S	PASPSGSEDLSSVSSSPTSSPKTKVTTVTSA	1802000	541230	6248
Q9P2N6	540	KAT8 regulatory NSL complex subunit 3	KANSL3	0.858879	6.65E-34	219.21	S	SGSEDLSSVSSSPTSSPKTKVTTVTSAQKSS	0	809460	6250
Q9H4L4	169	Sentrin-specific protease 3	SENP3	1	1.44E-30	219.16	S	HRGRRRGLAHPKNHLSPQQGGATPQVPSPCC	12246000	8804000	5675
Q9H4L4	181	Sentrin-specific protease 3	SENP3	1	1.44E-30	219.16	S	NHLSPQQGGATPQVPSPCCRFDSPRGPPPPR	14012000	10427000	5676
Q9UJ14	83	Gamma-glutamyltransferase 7;Gamma-glutamyltransferase 7 hea	GGT7	1	2.11E-28	219.06	S	QRLPSSSSEMGSQDGSPLRETRKDPFSAAAA	584530	700450	6392
Q7Z401	1098	C-myc promoter-binding protein	DENND4A	0.5	1.23E-28	219.03	S	GKPPLRSKRDSLEKESSDDDTPFDGSNYLAD	1004600	853550	3787
Q7Z401	1099	C-myc promoter-binding protein	DENND4A	0.971838	1.23E-28	219.03	S	KPPLRSKRDSLEKESSDDDTPFDGSNYLADK	1004600	853550	3788
Q99549	51	M-phase phosphoprotein 8	MPHOSPH8	1	1.77E-30	218.88	S	EDNDAAARGAEAFGDSEEDGEDVFEVEKILD	3098200	3193000	5205

Q7L2J0	217	7SK snRNA methylphosphate capping enzyme	MEPCE	0.995602	5.56E-27	218.83	S	DEEVSRTLNAETPKSSPLPAKGRDPVEILIP	0	852940	3727
Q7L2J0	213	7SK snRNA methylphosphate capping enzyme	MEPCE	0.999989	5.56E-27	218.83	T	NSLLDEEVSRTLNAETPKSSPLPAKGRDPVE	0	852940	7791
Q69YN4	1579	Protein virilizer homolog	KIAA1429	0.998579	1.11E-27	218.73	S	DLHSELERSFLSEPSSPGRTKTTKGFKLGKH	10978000	6915700	3436
Q9NQ55	359	Suppressor of SWI4 1 homolog	PPAN	1	6.91E-28	218.68	S	RKKSLEGMKKARVGGSDEEASGIPSRTASLE	33777000	35633000	5901
Q8TDD1	74	ATP-dependent RNA helicase DDX54	DDX54	0.499988	1.70E-28	218.64	T	LGPGRPLPTFPTSECTSDVEPDTREMVRAQN	0	0	7906
Q9Y2W1	320	Thyroid hormone receptor-associated protein 3	THRAP3	0.999993	6.95E-28	218.6	S	GSGSLSPSKKSPVGKSPPSTGSTYGSSQKEE	23123000	10286000	6825
Q9Y6E2	412	Basic leucine zipper and W2 domain-containing protein 2	BZW2	1	5.70E-08	218.42	S	QMKKFVEWLQNAEEESESEGEEN________	0	0	7002
Q9Y6E2	414	Basic leucine zipper and W2 domain-containing protein 2	BZW2	1	5.70E-08	218.42	S	KKFVEWLQNAEEESESEGEEN__________	0	0	7003
Q56P03	109	E2F-associated phosphoprotein	EAPP	0.999999	2.28E-07	218.36	S	VATAPTRYYDDIYFDSDSEDEDRAVQVTKKK	0	0	3147
Q56P03	111	E2F-associated phosphoprotein	EAPP	1	2.28E-07	218.36	S	TAPTRYYDDIYFDSDSEDEDRAVQVTKKKKK	0	0	3148
Q6Y7W6	30	PERQ amino acid-rich with GYF domain-containing protein 2	GIGYF2	1	3.54E-29	218.36	S	LRALSSGGSITSPPLSPALPKYKLADYRYGR	1913700	1478700	3643
Q9H4L5	304	Oxysterol-binding protein-related protein 3	OSBPL3	0.70905	2.22E-28	218.36	S	LQVPKPFSGPVRLHSSNPNLSTLDFGEEKNY	0	2467400	5680
Q96D46	470	60S ribosomal export protein NMD3	NMD3	1	9.12E-28	218.28	T	NVNIYRDSAIPVESDTDDEGAPRISLAEMLE	1371500	5316600	7975
Q9P2N6	539	KAT8 regulatory NSL complex subunit 3	KANSL3	0.847479	7.18E-34	218.22	S	PSGSEDLSSVSSSPTSSPKTKVTTVTSAQKS	1802000	541230	6249
Q9NRL2	1363	Bromodomain adjacent to zinc finger domain protein 1A	BAZ1A	0.999958	4.22E-28	218.12	S	FVELLSPRRKRRGRKSANNTPENSPNFPNFR	650020	591900	5974
Q9BWW4	360	Single-stranded DNA-binding protein 3	SSBP3	1	9.24E-28	218.11	T	KNSPNNISGISNPPGTPRDDGELGGNFLHSF	993120	868060	8059
P52701	254	DNA mismatch repair protein Msh6	MSH6	1	1.13E-29	218.05	S	RRSSRQIKKRRVISDSESDIGGSDVEFKPDT	6527000	3527400	1820
P52701	256	DNA mismatch repair protein Msh6	MSH6	1	1.13E-29	218.05	S	SSRQIKKRRVISDSESDIGGSDVEFKPDTKE	4587400	3835900	1821
P52701	261	DNA mismatch repair protein Msh6	MSH6	1	1.13E-29	218.05	S	KKRRVISDSESDIGGSDVEFKPDTKEEGSSD	10574000	6612800	1822
Q32MZ4	768	Leucine-rich repeat flightless-interacting protein 1	LRRFIP1	0.995066	2.30E-28	217.91	S	SEHPSQTVRKALDSNSLENDDLSAPGREPGH	4318000	4422400	3052
Q9H6F5	80	Coiled-coil domain-containing protein 86	CCDC86	1	7.47E-34	217.69	S	TSPGSPRLQQGAGLESPQGQPEPGAASPQRQ	35277000	23020000	5730
P17509	214	Homeobox protein Hox-B6	HOXB6	1	6.91E-28	217.65	S	MKWKKESKLLSASQLSAEEEEEKQAE_____	1766200	2055200	1110
Q96ST2	287	Protein IWS1 homolog	IWS1	1	2.66E-27	217.65	S	RISDSESEDPPRNQASDSENEELPKPRVSDS	10544000	8550700	5136
Q96ST2	289	Protein IWS1 homolog	IWS1	1	2.66E-27	217.65	S	SDSESEDPPRNQASDSENEELPKPRVSDSES	10544000	8856700	5137
P10809	70	60 kDa heat shock protein, mitochondrial	HSPD1	1	3.56E-28	217.39	S	MGPKGRTVIIEQSWGSPKVTKDGVTVAKSID	1862400	1471900	1003
Q9NRY4	1179	Rho GTPase-activating protein 35	ARHGAP35	0.999997	7.04E-28	217.39	S	RLGRFASYRTSFSVGSDDELGPIRKKEEDQA	2271900	1267300	5988
Q9NSK0	590	Kinesin light chain 4	KLC4	1	4.57E-29	217.39	S	GTEPRPSSSNMKRAASLNYLNQPSAAPLQVS	1494000	906310	5998
Q9UQ35	1326	Serine/arginine repetitive matrix protein 2	SRRM2	1	1.04E-27	217.19	S	SPEHKELSNSPLRENSFGSPLEFRNSGPLGT	13193000	3792900	6618
Q9UQ35	1329	Serine/arginine repetitive matrix protein 2	SRRM2	1	1.04E-27	217.19	S	HKELSNSPLRENSFGSPLEFRNSGPLGTEMN	3410300	16781000	6619
Q9UFC0	251	Leucine-rich repeat and WD repeat-containing protein 1	LRWD1	0.98999	3.78E-34	217.1	S	DDVPLSLSPSKRACASPSAQVEGSPVAGSDG	2538900	1663600	6307
P05455	92	Lupus La protein	SSB	0.869045	3.51E-27	217.01	S	AELMEISEDKTKIRRSPSKPLPEVTDEYKND	8977200	3491000	906
Q9Y314	138	Nitric oxide synthase-interacting protein	NOSIP	0.834051	1.26E-30	216.9	S	SRPLNPFTAKALSGTSPDDVQPGPSVGPPSK	881310	919180	6840
Q14680	356	Maternal embryonic leucine zipper kinase	MELK	1	7.93E-28	216.77	S	QASATPFTDIKSNNWSLEDVTASDKNYVAGL	1115100	2394000	2774
Q9BVJ6	31	U3 small nucleolar RNA-associated protein 14 homolog A	UTP14A	1	2.49E-28	216.77	S	QEELADLPKDYLLSESEDEGDNDGERKHQKL	557100	589540	5404
Q5VT52	593	Regulation of nuclear pre-mRNA domain-containing protein 2	RPRD2	0.992275	2.08E-30	216.61	S	LPSSAQPFIPKSFNYSPNSSTSEVSSTSASK	1958700	0	3335
Q14678	325	KN motif and ankyrin repeat domain-containing protein 1	KANK1	0.971292	7.57E-28	216.34	S	AASQINVCGVRKRSYSAGNASQLEQLSRARR	1282800	432960	2773
Q9Y3T9	673	Nucleolar complex protein 2 homolog	NOC2L	1	7.48E-08	216.32	S	DEDRKQFKDLFDLNSSEEDDTEGFSERGILR	0	0	6874
Q9UQ88;P2	265;277	Cyclin-dependent kinase 11A;Cyclin-dependent kinase 11B	CDK11A;CDK1	1	8.20E-28	216.26	S	PAQLKEEKMEERDLLSDLQDISDSERKTSSA	2125700	1382000	1191
Q9NYA4	610	Myotubularin-related protein 4	MTMR4	1	2.62E-28	216.01	S	QEFSGRSLDRLPKTRSMDDLLSACDTSSPLT	963930	751200	6092
Q5R372	119	Rab GTPase-activating protein 1-like	RABGAP1L	0.921033	2.62E-28	216	S	ILDPSNTEISTPRPSSPGGLPEEDSVLFNKL	16494000	7600200	3210
Q9BVC5	189	Ashwin	C2orf49	1	3.48E-28	215.96	S	NHDLTHRKSPSGPVKSPPLSPVGTTPVKLKR	9778700	7539900	5398
Q9BVC5	193	Ashwin	C2orf49	0.999998	3.48E-28	215.96	S	THRKSPSGPVKSPPLSPVGTTPVKLKRAAPK	4758200	4864900	5399
P20290	161	Transcription factor BTF3	BTF3	0.648284	5.42E-31	215.88	S	MLPSILNQLGADSLTSLRRLAEALPKQSVDG	851720	234080	1183
Q86UP2	1313	Kinectin	KTN1	0.998872	5.52E-31	215.59	S	VKAAGDTTVIENSDVSPETESSEKETMSVSL	115940	488660	3931
Q92922	328	SWI/SNF complex subunit SMARCC1	SMARCC1	1	6.71E-27	215.49	S	RRDRKASANARKRKHSPSPPPPTPTESRKKS	63240000	36397000	4728
Q9ULM3	536	YEATS domain-containing protein 2	YEATS2	0.999956	2.71E-28	215.49	S	TNKISTASQVSQGTGSPVPKIHGSSFVTSTV	4543000	4295600	6493
Q92922	335	SWI/SNF complex subunit SMARCC1	SMARCC1	0.998428	6.71E-27	215.49	T	ANARKRKHSPSPPPPTPTESRKKSGKKGQAS	8316400	0	7952
Q14683	957	Structural maintenance of chromosomes protein 1A	SMC1A	0.92727	7.26E-34	215.43	S	LSKGTMDDISQEEGSSQGEDSVSGSQRISSI	0	1066800	2778
O76080	58	AN1-type zinc finger protein 5	ZFAND5	0.969872	4.65E-31	215.39	S	NSGRMSPMGTASGSNSPTSDSASVQRADTSL	234640	749260	678
P46013	357	Antigen KI-67	MKI67	1	8.67E-28	215.37	S	PAKMKTPVQYSQQQNSPQKHKNKDLYTTGRR	2989200	1446200	1540
Q9UKA8;P5	148;163	Calcipressin-3;Calcipressin-1	RCAN3;RCAN1	1	6.78E-28	215.15	S	KSYLLPPQPVKQFLISPPASPPVGWKQSEDA	2181100	2545600	1837
Q9UKA8;P5	152;167	Calcipressin-3;Calcipressin-1	RCAN3;RCAN1	1	6.78E-28	215.15	S	LPPQPVKQFLISPPASPPVGWKQSEDAMPVI	2181100	2545600	1838
Q9C0C2	437	182 kDa tankyrase-1-binding protein	TNKS1BP1	0.989301	8.15E-28	215.15	S	SLVQRRFSEGVLQSPSQDQEKLGGSLAALPQ	1426600	995900	5518
P21796	104	Voltage-dependent anion-selective channel protein 1	VDAC1	0.999834	4.47E-28	214.89	S	DQLARGLKLTFDSSFSPNTGKKNAKIKTGYK	11618000	6495200	1201
Q969V6	385	MKL/myocardin-like protein 1	MKL1	1	9.06E-28	214.65	S	KTELIERLRAYQDQISPVPGAPKAPAATSIL	520370	2714300	4751
O60343	569	TBC1 domain family member 4	TBC1D4	0.860965	4.26E-28	214.61	S	FKLDILKNKAKRSLTSSLENIFSRGANRMRG	3425900	0	492
Q9Y281	3	Cofilin-2	CFL2	1	1.81E-28	214.61	S	_____________MASGVTVNDEVIKVFNDM	9516400	9129000	6769
O60343	566	TBC1 domain family member 4	TBC1D4	0.998719	3.55E-28	214.58	S	SGRFKLDILKNKAKRSLTSSLENIFSRGANR	947340	512660	491
O60343	570	TBC1 domain family member 4	TBC1D4	0.98651	3.55E-28	214.58	S	KLDILKNKAKRSLTSSLENIFSRGANRMRGR	947340	2582900	493
Q12888	1028	Tumor suppressor p53-binding protein 1	TP53BP1	1	8.45E-28	214.54	S	GERKNGSTAVAESVASPQKTMSVLSCICEAR	1765900	1198100	2353
Q7Z6Z7	2889	E3 ubiquitin-protein ligase HUWE1	HUWE1	0.67843	2.24E-28	214.54	T	TSAAGSSEQPRAGSSTPGDAPPAVAEVQGRS	2271500	1517600	7815
Q9BW71	289	HIRA-interacting protein 3	HIRIP3	1	1.38E-22	214.48	S	RSCKQKSQAKRLLGDSDSEEEQKEAASSGDD	0	1408800	5417
Q9BW71	291	HIRA-interacting protein 3	HIRIP3	1	1.38E-22	214.48	S	CKQKSQAKRLLGDSDSEEEQKEAASSGDDSG	141520	1408800	5418
Q13263	26	Transcription intermediary factor 1-beta	TRIM28	0.968996	4.43E-33	214.44	S	AAASAASGSPGPGEGSAGGEKRSTAPSAAAS	185510000	780960	2449
P11717	2484	Cation-independent mannose-6-phosphate receptor	IGF2R	1	5.72E-22	214.42	S	QKTVSSTKLVSFHDDSDEDLLHI________	3741400	3109600	1029
Q96CB8	128	Integrator complex subunit 12	INTS12	0.788583	2.56E-21	214.34	S	DIPKKPRLEKPETQSSPITVQSSKDLPMADL	0	0	4826
Q7KZF4	426	Staphylococcal nuclease domain-containing protein 1	SND1	1	1.82E-21	214.18	S	IGKKVNVTVDYIRPASPATETVPAFSERTCA	1825800	5265800	3710
Q15084	428	Protein disulfide-isomerase A6	PDIA6	1	5.57E-24	214.05	S	DGRDGELPVEDDIDLSDVELDDLGKDEL___	1545500	1375800	2872
Q13242	204	Serine/arginine-rich splicing factor 9	SRSF9	1	1.18E-18	214.04	S	SYGYSRSRSGSRGRDSPYQSRGSPHYFSPFR	3323000	0	2434
O43741	39	5'-AMP-activated protein kinase subunit beta-2	PRKAB2	0.846287	9.41E-22	213.99	S	AGGHAPGKEHKIMVGSTDDPSVFSLPDSKLP	1050800	873340	416
Q12789	1868	General transcription factor 3C polypeptide 1	GTF3C1	1	1.70E-23	213.82	S	PSHSPRGTKRRASWASENGETDAEGTQMTPA	2551000	748300	2298
P63165	2	Small ubiquitin-related modifier 1	SUMO1	1	3.50E-21	213.71	S	______________MSDQEAKPSTEDLGDKK	7836600	12817000	1936
Q93008	1600	Probable ubiquitin carboxyl-terminal hydrolase FAF-X	USP9X	1	9.54E-24	213.71	S	ILAIEGTGSDVDDDMSGDEKQDNESNVDPRD	1885000	1400000	4742
Q9UQ35	1099	Serine/arginine repetitive matrix protein 2	SRRM2	1	1.95E-20	213.71	S	PSLQSKSQTSPKGGRSRSSSPVTELASRSPI	11772000	10410000	6719
Q9UQ35	1101	Serine/arginine repetitive matrix protein 2	SRRM2	0.999998	1.95E-20	213.71	S	LQSKSQTSPKGGRSRSSSPVTELASRSPIRQ	11772000	10673000	6720
Q9UQ35	1103	Serine/arginine repetitive matrix protein 2	SRRM2	0.999804	1.95E-20	213.71	S	SKSQTSPKGGRSRSSSPVTELASRSPIRQDR	27060000	24921000	6722
Q92945	181	Far upstream element-binding protein 2	KHSRP	1	2.03E-21	213.69	S	QINKIQQDSGCKVQISPDSGGLPERSVSLTG	14612000	22553000	4737
Q9NUQ3	105	Gamma-taxilin	TXLNG	0.998793	2.03E-21	213.69	S	TENRNLVSPAYCTQESREEIPGGEARTDPPD	4686900	1798600	6025
Q8TEU7	230	Rap guanine nucleotide exchange factor 6	RAPGEF6	1	4.15E-23	213.64	S	VGDVDLTRLPEGPVDSEDDEEEDEEIDRTDP	627210	903350	4488
P0C1Z6	252	TCF3 fusion partner	TFPT	1	2.18E-21	213.63	S	GPDKLLPYPTLASPASD______________	6845700	5207700	975
Q9BXB4	181	Oxysterol-binding protein-related protein 11	OSBPL11	0.91805	4.63E-21	213.63	S	PPLKSRSFSLASSSNSPISQRRPSQNAISFF	618660	317680	5448
P78559	526	Microtubule-associated protein 1A;MAP1A heavy chain;MAP1 lig	MAP1A	1	2.34E-21	212.92	S	TVPLPTISGHRELVLSSPEDLTQDFEEMKRE	2143800	1308500	1976
P78559	527	Microtubule-associated protein 1A;MAP1A heavy chain;MAP1 lig	MAP1A	1	2.34E-21	212.92	S	VPLPTISGHRELVLSSPEDLTQDFEEMKREE	2143800	1308500	1977
Q01167	428	Forkhead box protein K2	FOXK2	0.999592	5.15E-23	212.74	S	LAVIQEARFAQSAPGSPLSSQPVLITVQRQL	1915400	2355800	2061
Q9H0W5	261	Coiled-coil domain-containing protein 8	CCDC8	1	9.32E-21	212.72	S	GDRLGNAGDVCVPQASPRRWRPKINWASFRR	4741700	5095700	5584
Q9UBC2	255	Epidermal growth factor receptor substrate 15-like 1	EPS15L1	0.999027	9.23E-23	212.55	S	PSHGSVSSLNSTGSLSPKHSLKQTQPTVNWV	2971400	1294300	6261
Q8N3F8	621	MICAL-like protein 1	MICALL1	0.767685	8.54E-21	212.43	S	LLVGDRSPVPSPGSSSPQLQVKSSCKENPFN	0	0	4256
Q92614	2041	Unconventional myosin-XVIIIa	MYO18A	0.999999	9.16E-21	212.35	S	PLDNTSRPRYSHSYLSDSDTEAKLTETNA__	3427600	1842700	4667
Q92614	2043	Unconventional myosin-XVIIIa	MYO18A	0.999977	9.16E-21	212.35	S	DNTSRPRYSHSYLSDSDTEAKLTETNA____	3427600	1842700	4668
P49736	27	DNA replication licensing factor MCM2	MCM2	0.999934	3.09E-20	212.1	S	SSPAQRRRGNDPLTSSPGRSSRRTDALTSSP	45281000	26769000	1681
P43699	254	Homeobox protein Nkx-2.1	NKX2-1	1	2.26E-37	212.09	S	GGGTGCPQQQQAQQQSPRRVAVPVLVKDGKP	5044800	4508000	1528
P55196	1782	Afadin	MLLT4	0.939666	1.21E-21	212.06	S	AKEKRSKSQDADSPGSSGAPENLTFKERQRL	1120500	784720	1870
O95639	212	Cleavage and polyadenylation specificity factor subunit 4	CPSF4	0.991757	4.75E-21	211.82	S	LQRSSSLIQLTSQNSSPNQQRTPQVIGVMQS	1311600	623390	786
Q9UQ35	1102	Serine/arginine repetitive matrix protein 2	SRRM2	0.999728	3.68E-20	211.63	S	QSKSQTSPKGGRSRSSSPVTELASRSPIRQD	32048000	19402000	6721
P46108	40	Adapter molecule crk	CRK	0.474563	5.39E-21	211.45	S	ALLQGQRHGVFLVRDSSTSPGDYVLSVSENS	0	0	1560
P46108	41	Adapter molecule crk	CRK	0.787304	5.39E-21	211.45	S	LLQGQRHGVFLVRDSSTSPGDYVLSVSENSR	0	4729100	1561
Q9UQ35	311	Serine/arginine repetitive matrix protein 2	SRRM2	0.990357	9.17E-21	211.45	S	PSPASGRRGEGDAPFSEPGTTSTQRPSSPET	554650	63679000	6628
Q00587	121	Cdc42 effector protein 1	CDC42EP1	1	1.45E-22	211.43	S	SPAPPAISPIIKNAISLPQLNQAAYDSLVVG	551450	613280	2029
O14654	751	Insulin receptor substrate 4	IRS4	1	3.75E-20	211.26	S	PFEDSRGYMMMFPRVSPPPAPSPPKAPDTNK	11526000	7387800	183
O14654	757	Insulin receptor substrate 4	IRS4	1	3.75E-20	211.26	S	GYMMMFPRVSPPPAPSPPKAPDTNKEDDSKD	4267900	2749300	184
Q96B54	99	Zinc finger protein 428	ZNF428	1	1.67E-20	211.22	S	RAAQPPAQPCQLCGRSPLGEAPPGTPPCRLC	2435900	3453500	4803
Q9H307	347	Pinin	PNN	1	4.21E-20	211.22	S	EEAGEEEEKEIAIVHSDAEKEQEEEEQKQEM	6877100	8713000	5631
O60343	787	TBC1 domain family member 4	TBC1D4	0.993413	8.24E-21	211.13	S	WRQRIFLRVASPMNKSPSAMQQQDGLDRNEL	13282000	9393900	494
P52701	252	DNA mismatch repair protein Msh6	MSH6	1	1.07E-21	211.11	S	GSRRSSRQIKKRRVISDSESDIGGSDVEFKP	9286800	5746700	1819
Q12873	1605	Chromodomain-helicase-DNA-binding protein 3	CHD3	1	8.50E-21	211.09	S	GEKMETEADAPSPAPSLGERLEPRKIPLEDE	1645000	975180	2338
O00264	178	Membrane-associated progesterone receptor component 1	PGRMC1	0.999569	3.02E-20	211.06	T	KYHHVGKLLKEGEEPTVYSDEEEPKDESARK	0	0	7120
Q14157	467	Ubiquitin-associated protein 2-like	UBAP2L	0.999637	4.81E-26	211.03	S	PSSPLPSKSTSAPQMSPGSSDNQSSSPQPAQ	1994800	1160500	2669
Q6NZY4	598	Zinc finger CCHC domain-containing protein 8	ZCCHC8	0.972418	2.14E-21	210.91	S	VPEIFTKKSEAGHASSPDSEVTSLCQKEKAE	0	656780	3499
Q9UQ35	857	Serine/arginine repetitive matrix protein 2	SRRM2	0.999199	5.28E-20	210.66	S	PKVKSGTPPRQGSITSPQANEQSVTPQRRSC	2170200	2686800	6668
Q9C0C9	836	E2/E3 hybrid ubiquitin-protein ligase UBE2O	UBE2O	0.999951	3.71E-25	210.46	S	LESLKNMTVEQLLTGSPTSPTVEPEKPTREK	5781900	5752800	5535
Q9UH62	110	Armadillo repeat-containing X-linked protein 3	ARMCX3	0.999661	1.22E-20	210.43	S	RARATRARRAVQKRASPNSDDTVLSPQELQK	1182200	775940	6327
Q5SXM2	1398	snRNA-activating protein complex subunit 4	SNAPC4	1	2.04E-21	210.39	S	PQGVRTTLSVPSRVGSESEDEDLLSELELAD	978570	1028500	3227
Q5SXM2	1400	snRNA-activating protein complex subunit 4	SNAPC4	1	2.04E-21	210.39	S	GVRTTLSVPSRVGSESEDEDLLSELELADRD	978570	1028500	3228
Q9Y3X0	389	Coiled-coil domain-containing protein 9	CCDC9	0.786938	9.25E-22	210.39	T	AASPAPETPQPTSPETSPKETPMQPPEIPAP	1467400	860770	8296
Q13425	393	Beta-2-syntrophin	SNTB2	0.999984	1.01E-20	210.38	S	PLVATRLVHSGSGCRSPSLGSDLTFATRTGS	2361200	2158300	2490
P51531	1568	Probable global transcription activator SNF2L2	SMARCA2	1	4.80E-20	210.34	S	KGKKRPNRGKAKPVVSDFDSDEEQDEREQSE	799380	1321300	1779
P51531	1572	Probable global transcription activator SNF2L2	SMARCA2	1	4.80E-20	210.34	S	RPNRGKAKPVVSDFDSDEEQDEREQSEGSGT	1748600	1967400	1780
Q14CS0	235	UBX domain-containing protein 2B	UBXN2B	0.931242	2.03E-22	210.2	S	QKLGSLTPEIVSTPSSPEEEDKSILNAVVLI	1570400	1491100	2849
P12270	379	Nucleoprotein TPR	TPR	0.999278	1.27E-22	210.12	S	KRKGAILSEEELAAMSPTAAAVAKIVKPGMK	1022600	1096600	1039
Q02952	1390	A-kinase anchor protein 12	AKAP12	0.488723	5.12E-23	210.12	S	LQTVNVPIIDGAKEVSSLEGSPPPCLGQEEA	0	0	2113
Q02952	1391	A-kinase anchor protein 12	AKAP12	0.488723	5.12E-23	210.12	S	QTVNVPIIDGAKEVSSLEGSPPPCLGQEEAV	0	0	2114
Q96PU5	448	E3 ubiquitin-protein ligase NEDD4-like	NEDD4L	0.773304	1.33E-20	210.08	S	NNHLIEPQIRRPRSLSSPTVTLSAPLEGAKD	0	1218900	5061
Q9Y6X4	635	Soluble lamin-associated protein of 75 kDa	FAM169A	1	6.75E-21	209.89	S	EQLDQFTQSAEKAVDSSSEEIEVEVPVVDRR	2924100	2485400	7025
Q9Y6X4	636	Soluble lamin-associated protein of 75 kDa	FAM169A	1	6.75E-21	209.89	S	QLDQFTQSAEKAVDSSSEEIEVEVPVVDRRN	2924100	2485400	7026
Q13501	272	Sequestosome-1	SQSTM1	0.999519	1.72E-19	209.73	S	IDVEHGGKRSRLTPVSPESSSTEEKSSSQPS	2223000	1754200	2553
Q96TC7	46	Regulator of microtubule dynamics protein 3	RMDN3	0.962584	8.41E-21	209.73	S	YSQRWKRTQRHGRSQSLPNSLDYTQTSDPGR	3617000	1729000	5191
Q13501	269	Sequestosome-1	SQSTM1	0.982525	1.72E-19	209.73	T	EVDIDVEHGGKRSRLTPVSPESSSTEEKSSS	0	0	7548
P85037	445	Forkhead box protein K1	FOXK1	1	2.45E-20	209.67	S	RSGGLQTPECLSREGSPIPHDPEFGSKLASV	4142600	3093900	1995
Q53SF7	294	Cordon-bleu protein-like 1	COBLL1	0.999964	2.49E-20	209.6	S	FSFFQRSKKKRDQTASAPATPLVNKHRPTFT	1041300	583810	3144
P38919	12	Eukaryotic initiation factor 4A-III;Eukaryotic initiation fa	EIF4A3	0.999783	5.16E-21	209.53	S	____MATTATMATSGSARKRLLKEEDMTKVE	11210000	6751200	1459
O95297	210	Myelin protein zero-like protein 1	MPZL1	0.999171	2.68E-20	209.33	S	NSKRDYTGCSTSESLSPVKQAPRKSPSDTEG	2498300	1644100	747
A4D1S0	204	Killer cell lectin-like receptor subfamily G member 2	KLRG2	0.999984	3.49E-38	209.23	S	AAARTESGCDAEGRASPAEGSAGSPGSPTCC	0	535630	32
P60468	13	Protein transport protein Sec61 subunit beta	SEC61B	0.936909	2.73E-20	209.23	S	___MPGPTPSGTNVGSSGRSPSKAVAARAAG	3044500	914830	1904
Q96MG7	64	Melanoma-associated antigen G1	NDNL2	0.998986	1.58E-20	209.23	S	STSRGPGGSQGSQGPSPQGARRAQAAPAVGP	1412700	1118900	5021
Q99575	730	Ribonucleases P/MRP protein subunit POP1	POP1	0.998434	2.16E-20	209.23	S	WESRVQAYEEPSVASSPNGKESDLRRSEVPC	6241800	3179300	5215
Q96ST2	157	Protein IWS1 homolog	IWS1	1	1.07E-20	209.22	S	HASDSENEDVGKHPASDSEIEELQKSPASDS	513110	898290	5127
Q96ST2	159	Protein IWS1 homolog	IWS1	1	1.07E-20	209.22	S	SDSENEDVGKHPASDSEIEELQKSPASDSET	513110	898290	5128
P22059	351	Oxysterol-binding protein 1	OSBP	1	1.15E-30	209.16	S	VGSGKDQCCSGKGDMSDEDDENEFFDAPEII	6233900	5334800	1202
Q9BW71	223	HIRA-interacting protein 3	HIRIP3	0.999986	6.48E-20	208.87	S	AKKVEGNKGTKSLKESEQESEEEILAQKKEQ	411250	535500	5420

Q9BW71	227	HIRA-interacting protein 3	HIRIP3	1	6.48E-20	208.87	S	EGNKGTKSLKESEQESEEEILAQKKEQREEE	411250	535500	5421	
P68402	2	Platelet-activating factor acetylhydrolase IB subunit beta	PAFAH1B2	0.999812	2.92E-23	208.63	S	______________MSQGDSNPAAIPHAAED	5809100	12441000	1947	
O95628	324	CCR4-NOT transcription complex subunit 4	CNOT4	1	3.50E-21	208.62	S	SNPVIPISSSNHSARSPFEGAVTESQSLFSD	631390	451790	783	
P11717	2409	Cation-independent mannose-6-phosphate receptor	IGF2R	1	2.01E-25	208.61	S	KSVKALSSLHGDDQDSEDEVLTIPEVKVHSG	15847000	16694000	1028	
Q641Q2;Q9	498;498	WASH complex subunit FAM21A;WASH complex subunit FAM21C	FAM21A;FAM2	1	2.42E-20	208.59	S	FGDEEGDLFKEKAVASPEATVSQTDENKARA	427020	760230	3386	
Q9UN81	25	LINE-1 retrotransposable element ORF1 protein	L1RE1	0.333188	3.52E-21	208.59	S	GNSKTQSASPPPKERSSSPATEQSWMENDFD	0	0	6537	
Q9UN81	26	LINE-1 retrotransposable element ORF1 protein	L1RE1	0.455247	3.52E-21	208.59	S	NSKTQSASPPPKERSSSPATEQSWMENDFDE	0	0	6538	
Q9UN81	27	LINE-1 retrotransposable element ORF1 protein	L1RE1	0.455247	3.52E-21	208.59	S	SKTQSASPPPKERSSSPATEQSWMENDFDEL	0	0	6539	
Q8TAP6	83	Centrosomal protein of 76 kDa	CEP76	0.932705	1.05E-20	208.52	S	ELNFVTDSVEQELPSSPKQPICFDRQSTLKK	646660	648850	4436	
Q9BRS2	22	Serine/threonine-protein kinase RIO1	RIOK1	0.998361	1.79E-20	208.52	S	LMSRVVPGQFDDADSSDSENRDLKTVKEKDD	23140000	18790000	5327	
O95155	31	Ubiquitin conjugation factor E4 B	UBE4B	0.999599	1.06E-20	208.49	S	RLAGGQTSQPTTPLTSPQRENPPGPPIAASA	1455600	966920	732	
Q3KQU3	813	MAP7 domain-containing protein 1	MAP7D1	0.99999	1.02E-19	208.39	T	GFSTNGPSGDKSLSRTPETLLPFAEAEAFLK	421980	585400	7668	
O75717	407	WD repeat and HMG-box DNA-binding protein 1	WDHD1	1	1.77E-22	208.37	S	GSSLLKEEEEDGQEGSIHNLPLVTSQRPFYD	16245000	9542100	662	
Q9BX63	1032	Fanconi anemia group J protein	BRIP1	0.998767	1.92E-20	208.07	S	PKATPELGSSENSASSPPRFKTEKMESKTVL	1972400	1095200	5442	
O00567	520	Nucleolar protein 56	NOP56	1	3.88E-21	207.97	S	KPKKKKSFSKEELMSSDLEETAGSTSIPKRK	6640600	6848500	135	
Q8WXI9	135	Transcriptional repressor p66-beta	GATAD2B	0.99936	3.98E-21	207.81	S	TPSPDIIVLSDNEASSPRSSSRMEERLKAAN	13391000	10320000	4599	
O75179	2067	Ankyrin repeat domain-containing protein 17	ANKRD17	0.999997	1.67E-20	207.66	S	PGGVSRNSPLDCGSASPNKVASSSEQEAGSP	25944000	16566000	588	
P18858	195	DNA ligase 1	LIG1	0.999966	1.18E-19	207.4	T	QPTTPPKPLKTSKAETPTESVSEPEVATKQE	266560	604050	7301	
O95071	2486	E3 ubiquitin-protein ligase UBR5	UBR5	1	9.83E-26	207.38	S	EKVQQENRKRHGSSRSVVDMDLDDTDDGDDN	3078000	2140000	728	
Q659C4	60	La-related protein 1B	LARP1B	1	4.23E-21	207.38	S	KENRETKLNGPGENVSEDEAQSSNQRKRANK	820570	891770	3398	
Q96Q42	483	Alsin	ALS2	1	1.72E-19	207.38	S	VDIREEETEGGSRRLSLPGLLSQVSPRLLRK	1202200	847890	5066	
Q96Q42	492	Alsin	ALS2	1	1.72E-19	207.38	S	GGSRRLSLPGLLSQVSPRLLRKAARVKTRTV	1756300	1445000	5067	
P67809	2	Nuclease-sensitive element-binding protein 1	YBX1	0.997689	4.27E-26	207.24	S	______________MSSEAETQQPPAAPPAA	0	4442300	1943	
P67809	3	Nuclease-sensitive element-binding protein 1	YBX1	0.997913	4.27E-26	207.24	S	_____________MSSEAETQQPPAAPPAAP	0	4442300	1944	
O15234	363	Protein CASC3	CASC3	1	2.98E-20	207.21	S	SYRSRRLEQTSVRDPSPEADAPVLGSPEKEE	13212000	10916000	304	
P04626	1054	Receptor tyrosine-protein kinase erbB-2	ERBB2	0.999996	4.33E-21	207.21	S	AGGMVHHRHRSSSTRSGGGDLTLGLEPSEEE	1125600	1107600	881	
Q01804	1006	OTU domain-containing protein 4	OTUD4	0.998527	3.00E-20	207.16	S	KTEKVKDPKTAADVVSPGANSVDSRVQRPKE	878240	0	2076	
Q6ZSZ5	921	Rho guanine nucleotide exchange factor 18	ARHGEF18	0.944753	3.86E-20	207.05	S	PPRRAETFAGYDCTNSPTKNGSFKKKVSSTD	2214300	1804900	3673	
Q9NXC5	766	WD repeat-containing protein mio	MIOS	0.999098	1.81E-20	207.05	S	VPHQGRGFSQYGVSGSPTKSKVTSCPGCRKP	3041900	1425900	6077	
Q4KMP7	148	TBC1 domain family member 10B	TBC1D10B	0.99773	7.70E-20	206.81	T	PKTEEARPSPAPGPGTPTGTPTRTPSRTAPG	23244000	11638000	7677	
Q4KMP7	152	TBC1 domain family member 10B	TBC1D10B	0.998925	7.70E-20	206.81	T	EARPSPAPGPGTPTGTPTRTPSRTAPGALTA	17521000	10594000	7679	
Q5JSH3	50	WD repeat-containing protein 44	WDR44	1	9.89E-20	206.74	S	TFKETENTAYKVGNESPVQELKQDVSKKIIE	0	8570100	3185	
O14654	921	Insulin receptor substrate 4	IRS4	0.992671	7.63E-20	206.74	Y	QKPTHEQREADSSSDYVNMDFTKRESNTPAP	1860700	729730	8343	
O60504	530	Vinexin	SORBS3	0.999218	1.91E-20	206.67	S	EPRLRLCDDGPQLPTSPRLTAAARSARHPSS	5188700	3648800	504	
Q5JSH3	162	WD repeat-containing protein 44	WDR44	0.908978	9.12E-23	206.67	S	CEKPVDETTKLTQTSSTEQLNVLETETEVLN	0	0	3184	
Q9UKG1	401	DCC-interacting protein 13-alpha	APPL1	0.960994	1.38E-20	206.67	S	TAARVNQSALEAVTPSPSFQQRHESLRPAAG	2039800	1990300	6440	
Q96JB3	197	Hypermethylated in cancer 2 protein	HIC2	1	2.27E-22	206.64	S	RKGAHAPQELPQAKGSDDELFLGGSNQDSVQ	1696700	1098100	4957	
P37802	163	Transgelin-2	TAGLN2	1	1.03E-19	206.45	S	PNWFPKKSKENPRNFSDNQLQEGKNVIGLQM	854330	736550	1445	
O60832	455	H/ACA ribonucleoprotein complex subunit 4	DKC1	0.999989	1.18E-20	206.43	S	EAAKTAKRKRESESESDETPPAAPQLIKKEK	9705600	4485900	528	
P27816	1151	Microtubule-associated protein 4	MAP4	0.994207	4.12E-20	206.36	S	DQREAQTLDSQIQETSI______________	1596800	1068800	1292	
Q641Q2;Q9	333;333	WASH complex subunit FAM21A;WASH complex subunit FAM21C	FAM21A;FAM2	1	3.37E-19	206.13	S	KPRKTLKEKKERRTPSDDEEDNLFAPPKLTD	4155400	2287800	3391	
Q9UNS1	1173	Protein timeless homolog	TIMELESS	1	4.33E-20	206.13	S	KEPLKAAPKKRQLLDSDEEQEEDEGRNRAPE	421680	0	6549	
Q641Q2;Q9	331;331	WASH complex subunit FAM21A;WASH complex subunit FAM21C	FAM21A;FAM2	1	3.37E-19	206.13	T	EAKPRKTLKEKKERRTPSDDEEDNLFAPPKL	1714900	851670	7737	
Q9UGV2	331	Protein NDRG3	NDRG3	0.999944	2.11E-20	205.98	S	IPSASMTRLARSRTHSTSSSLGSGESPFSRS	2132200	968220	6323	
Q9UGV2	335	Protein NDRG3	NDRG3	0.999601	2.11E-20	205.98	S	SMTRLARSRTHSTSSSLGSGESPFSRSVTSN	2132200	0	6326	
Q9ULT8	632	E3 ubiquitin-protein ligase HECTD1	HECTD1	0.992032	1.12E-21	205.92	S	RLGVISKVSTLAGPSSDDENEEESKPEKEDE	0	0	6502	
Q8IX01	773	SURP and G-patch domain-containing protein 2	SUGP2	0.967624	2.06E-25	205.84	S	PKPAGVDISEAPQTSSPCPSADIDMKTMETA	7018500	3620400	4090	
P40222	515	Alpha-taxilin	TXLNA	0.98993	1.84E-19	205.66	S	GPERRPEGPGAQAPSSPRVTEAPCYPGAPST	34878000	20957000	1469	
P51610	666	Host cell factor 1;HCF N-terminal chain 1;HCF N-terminal cha	HCFC1	0.999999	2.64E-20	205.66	S	TTVVGGVTKTITLVKSPISVPGGSALISNLG	4692600	4964600	1792	
Q14157	439	Ubiquitin-associated protein 2-like	UBAP2L	0.999999	2.13E-22	205.63	S	FTPSSTMMEVFLQEKSPAVATSTAAPPPPSS	131640	3170700	2664	
Q14157	454	Ubiquitin-associated protein 2-like	UBAP2L	0.944933	2.13E-22	205.63	S	SPAVATSTAAPPPPSSPLPSKSTSAPQMSPG	15271000	1058900	2666	
O00505	60	Importin subunit alpha-4	KPNA3	0.999999	1.59E-20	205.43	S	LLKKRNVPQEESLEDSDVDADFKAQNVTLEA	8594800	14992000	125	
Q02952	1395	A-kinase anchor protein 12	AKAP12	0.999992	1.07E-22	205.33	S	VPIIDGAKEVSSLEGSPPPCLGQEEAVCTKI	4031300	2705600	2115	
Q13112	428	Chromatin assembly factor 1 subunit B	CHAF1B	0.905922	4.74E-20	205.33	S	PRPVEGTPASRTQDPSSPGTTPPQARQAPAP	0	0	2410	
Q5SRE5	1709	Nucleoporin NUP188 homolog	NUP188	0.99457	2.74E-20	205.33	S	LLSSLSRYFRRGAPSSPATGVLPSPQGKSTS	6716100	5156900	3211	
Q9NX63	50	MICOS complex subunit MIC19	CHCHD3	1	4.74E-20	205.33	S	RMKESSPSGSKSQRYSGAYGASVSDEELKRR	2563400	926620	6076	
O94887	427	FERM, RhoGEF and pleckstrin domain-containing protein 2	FARP2	0.678865	4.76E-20	205.3	S	STLVPSGLPEFKDSSSSLTDPQVSYVKSPAA	2091700	0	715	
Q9C0C2	601	182 kDa tankyrase-1-binding protein	TNKS1BP1	1	5.46E-21	205.3	S	AEERYESQEPLAGQESPLPLATREAALPILE	2113900	1900100	5529	
P17029	13	Zinc finger protein with KRAB and SCAN domains 1	ZKSCAN1	0.999922	2.25E-20	205.26	S	___MMTAESREATGLSPQAAQEKDGIVIVKV	0	562510	1105	
Q86WB0	62	Nuclear-interacting partner of ALK	ZC3HC1	0.999981	1.28E-25	205.25	S	VDAKDTSATSQSVNGSPQAEQPSLESTSKEA	1786500	2177100	3978	
Q8NBN3	540	Transmembrane protein 87A	TMEM87A	1	1.28E-25	205.24	S	VPSSVTDVALPALLDSDEERMITHFERSKME	8861800	5481400	4314	
O95365	526	Zinc finger and BTB domain-containing protein 7A	ZBTB7A	0.992738	3.92E-26	205.23	S	SPGATATPGAPAQPSSPDARRNGQEKHFKDE	23241000	9198400	757	
Q9H8Y8	451	Golgi reassembly-stacking protein 2	GORASP2	0.999486	1.74E-24	205.2	S	SEKPVSAAVDANASESP______________	10910000	9924600	5796	
Q9Y5K6	458	CD2-associated protein	CD2AP	1	4.61E-19	205.07	S	SKLKLDSEQLPLRPKSVDFDSLTVRTSKETD	2060900	1024300	6961	
Q969V6	454	MKL/myocardin-like protein 1	MKL1	0.999944	2.23E-19	204.9	S	SGVVKFGSTGSTPPVSPTPSERSLLSTGDEN	26898000	44321000	4753	1.647743327
Q9H6F5	18	Coiled-coil domain-containing protein 86	CCDC86	0.999998	1.29E-19	204.9	S	TPLRRSRRLGGLRPESPESLTSVSRTRRALV	6802400	8194900	5728	
P17544	51	Cyclic AMP-dependent transcription factor ATF-7	ATF7	0.999943	1.22E-19	204.9	T	KFGPARTDSVIIADQTPTPTRFLKNCEEVGL	0	727060	7296	
P17544	53	Cyclic AMP-dependent transcription factor ATF-7	ATF7	0.967405	1.22E-19	204.9	T	GPARTDSVIIADQTPTPTRFLKNCEEVGLFN	0	727060	7297	
Q969V6	450	MKL/myocardin-like protein 1	MKL1	0.99596	2.23E-19	204.9	T	TVASSGVVKFGSTGSTPPVSPTPSERSLLST	5445500	13793000	7961	2.532917088
Q9C0C9	87	E2/E3 hybrid ubiquitin-protein ligase UBE2O	UBE2O	1	3.29E-20	204.81	S	GSVHFGLVRLIHGEDSDSEGEEEGRGSSGCS	1078400	863590	5533	
Q9C0C9	89	E2/E3 hybrid ubiquitin-protein ligase UBE2O	UBE2O	1	3.29E-20	204.81	S	VHFGLVRLIHGEDSDSEGEEEGRGSSGCSEA	1078400	863590	5534	
O95376	353	E3 ubiquitin-protein ligase ARIH2	ARIH2	1	5.08E-20	204.67	S	YECSRYKENPDIVNQSQQAQAREALKKYLFY	0	577040	758	
Q96JC9;Q9	266;258	ELL-associated factor 1;ELL-associated factor 2	EAF1;EAF2	0.998026	4.97E-20	204.67	S	NTLRNDLQLSESGSDSDD_____________	0	611640	4959	
Q9NW75	117	G patch domain-containing protein 2	GPATCH2	1	2.39E-20	204.67	S	DYRENHNNNKKDHSDSDDQMLVAKRRPSSNL	603450	716570	6054	
O94842	178	TOX high mobility group box family member 4	TOX4	0.999976	2.30E-21	204.51	S	LPPAQSPEDRLSTTPSPTSSLHEDGVEDFRR	15748000	17404000	704	
O94842	182	TOX high mobility group box family member 4	TOX4	0.919501	2.30E-21	204.51	S	QSPEDRLSTTPSPTSSLHEDGVEDFRRQLPS	13824000	17404000	706	
O94842	175	TOX high mobility group box family member 4	TOX4	0.513801	2.30E-21	204.51	T	GTILPPAQSPEDRLSTTPSPTSSLHEDGVED	1726300	0	7229	
Q5JTV8	143	Torsin-1A-interacting protein 1	TOR1AIP1	1	6.20E-21	204.05	S	TRLQQQHSEQPPLQPSPVMTRRGLRDSHSSE	2640900	2020600	3201	
Q9UGV2	334	Protein NDRG3	NDRG3	0.795308	7.22E-06	203.93	S	ASMTRLARSRTHSTSSSLGSGESPFSRSVTS	0	0	6325	
Q9UQ35	2100	Serine/arginine repetitive matrix protein 2	SRRM2	1	3.53E-19	203.93	S	SDRSRSATPPATRNHSGSRTPPVALNSSRMS	14513000	22263000	6660	
Q9UQ35	2102	Serine/arginine repetitive matrix protein 2	SRRM2	1	3.53E-19	203.93	S	RSRSATPPATRNHSGSRTPPVALNSSRMSCF	44288000	22263000	6661	
Q9UQ35	2104	Serine/arginine repetitive matrix protein 2	SRRM2	1	3.53E-19	203.93	T	RSATPPATRNHSGSRTPPVALNSSRMSCFSR	33324000	16595000	8263	
Q05519	207	Serine/arginine-rich splicing factor 11	SRSF11	0.999344	5.35E-20	203.92	S	STVDPKLNHVAAGLVSPSLKSDTSSKEIEEA	1168500	1836000	2189	
Q9Y6G9	512	Cytoplasmic dynein 1 light intermediate chain 1	DYNC1LI1	0.695999	3.36E-20	203.92	T	AELDRITRKPVTVSPTTPTSPTEGEAS____	0	0	8319	
Q9Y6X4	349	Soluble lamin-associated protein of 75 kDa	FAM169A	0.436552	5.49E-20	203.88	S	KRPKIGKRFQDSEFSSSQGEDEKTSQTSLTA	0	0	7028	
Q8NI27	1516	THO complex subunit 2	THOC2	0.952451	2.59E-20	203.87	S	KEENGTMGVSKHKSESPCESPYPNEKDKEKN	1696200	1088700	4429	
Q05655	295	Protein kinase C delta type;Protein kinase C delta type regu	PRKCD	1	1.42E-19	203.85	T	CGINQKLLAEALNQVTQRASRRSDSASSEPV	1907600	502440	7498	
Q9NPF5	448	DNA methyltransferase 1-associated protein 1	DMAP1	0.914467	4.45E-20	203.61	S	KDTIIDVVGAPLTPNSRKRRESASSSSSVKK	0	0	5888	
P35658	430	Nuclear pore complex protein Nup214	NUP214	0.999802	3.28E-20	203.52	S	KTPERLSLEGERQPKSPGSTPTTPTSSQAPQ	3034000	1774000	1417	
Q9BTX1	406	Nucleoporin NDC1	NDC1	1	2.67E-20	203.52	S	GRVSSSYPVEPKKLNSPEETAFQTPKSSQMP	1687300	929080	5370	
Q9NPF5	445	DNA methyltransferase 1-associated protein 1	DMAP1	0.999317	1.83E-19	203.52	T	PDPKDTIIDVVGAPLTPNSRKRRESASSSSS	1271600	740460	8136	
P78316	96	Nucleolar protein 14	NOP14	0.999986	2.73E-20	203.27	S	NVFRDKRFGEYNSNMSPEEKMMKRFALEQQR	4284700	2597500	1953	
P07910	260	Heterogeneous nuclear ribonucleoproteins C1/C2	HNRNPC	0.999993	5.67E-10	203.26	S	DETNVKMESEGGADDSAEEGDLLDDDDNEDR	0	0	944	
Q8IYB3	874	Serine/arginine repetitive matrix protein 1	SRRM1	1	3.87E-19	203.23	S	QEEPVAAPEPKKETESEAEDNLDDLEKHLRE	2272200	2971700	4140	
Q8IYB3	872	Serine/arginine repetitive matrix protein 1	SRRM1	1	3.87E-19	203.23	T	LAQEEPVAAPEPKKETESEAEDNLDDLEKHL	465360	1839000	7861	
Q9NYB0	203	Telomeric repeat-binding factor 2-interacting protein 1	TERF2IP	0.999359	5.72E-20	203.2	S	LRGQEHKYLLGDAPVSPSSQKLKRKAEEDPE	13223000	9968900	6095	
Q9Y467	802	Sal-like protein 2	SALL2	1	2.23E-25	203.2	S	GSESGGEKAISVRGDSEEASGAEEEVGTVAA	1678900	1567600	6895	
Q9Y467	806	Sal-like protein 2	SALL2	1	2.23E-25	203.2	S	GGEKAISVRGDSEEASGAEEEVGTVAAAATA	699500	939140	6896	
Q9NTJ3	39	Structural maintenance of chromosomes protein 4	SMC4	0.715755	6.08E-27	203.06	T	DGASSDAEPEPPSGRTESPATAAETASEELD	4253300	0	8163	
P49023	302	Paxillin	PXN	0.5	4.67E-20	203.05	S	GERCWAAGWPRDGGRSSPGGQDEGGFMAQGK	2176200	943980	1645	
P49023	303	Paxillin	PXN	0.5	4.67E-20	203.05	S	ERCWAAGWPRDGGRSSPGGQDEGGFMAQGKT	2176200	943980	1646	
Q13620	85	Cullin-4B	CUL4B	0.997973	8.19E-20	203.05	T	SNEREDFDSTSSSSSTPPLQPRDSASPSTSS	1487000	1100900	7569	
O75122	370	CLIP-associating protein 2	CLASP2	0.999675	8.32E-06	202.96	S	SSKASSLPGSLQRSRSDIDVNAAAGAKAHHA	451260	536320	564	
O15014	467	Zinc finger protein 609	ZNF609	0.999216	6.03E-20	202.84	S	SSSEDSKGSKRVRTNSMGSATGPLPGTKVEP	3303600	1931700	234	
Q96T58	1268	Msx2-interacting protein	SPEN	1	5.03E-20	202.81	S	EDSERTGGSPSVRHGSFHEDEDPIGSPRLLS	0	0	5171	
Q96T58	1278	Msx2-interacting protein	SPEN	1	5.03E-20	202.81	S	SVRHGSFHEDEDPIGSPRLLSVKGSPKVDEK	0	758460	5172	
P24723	317	Protein kinase C eta type	PRKCH	0.966594	3.49E-20	202.79	S	LAKTLAGMGLQPGNISPTSKLVSRSTLRRQG	705960	669420	1238	
Q9UQ35	1657	Serine/arginine repetitive matrix protein 2	SRRM2	0.955984	6.95E-21	202.79	S	SKGRGPSPEGSSSTESSPEHPPKSRTARRGS	2694500	884480	6633	
Q6P2E9	729	Enhancer of mRNA-decapping protein 4	EDC4	0.997407	5.00E-26	202.66	S	VEPLGLPQASPSRTRSPDVISSASTALSQDI	11935000	7972500	3523	
Q9Y520	779	Protein PRRC2C	PRRC2C	0.999988	2.09E-20	202.61	S	MIPPKPLMRRDQMEGSPNSSESFEHIARSAR	2663700	1731600	6944	
Q8WUI4	486	Histone deacetylase 7	HDAC7	0.781909	3.45E-22	202.52	S	LPLAQGGHRPLSRAQSSPAAPASLSAPEPAS	0	648170	4515	
P27816	280	Microtubule-associated protein 4	MAP4	0.9993	2.78E-19	202.32	S	ALATKTEVALAKDMESPTKLDVTLAKDMQPS	6943100	3874100	1289	
Q13085	29	Acetyl-CoA carboxylase 1;Biotin carboxylase	ACACA	0.999983	5.76E-22	202.21	S	NQHSRFIIGSVSEDNSEDEISNLVKLDLLEE	1623700	1132300	2396	
Q5JTV8	154	Torsin-1A-interacting protein 1	TOR1AIP1	0.999948	7.66E-23	202.16	S	PLQPSPVMTRRGLRDSHSSEEDEASSQTDLS	23206000	15243000	3198	
P29692	162	Elongation factor 1-delta	EEF1D	1	2.14E-25	202.15	S	TPAEDDEDDDIDLFGSDNEEEDKEAAQLREE	45731000	80406000	1325	
Q9Y6M1	162	Insulin-like growth factor 2 mRNA-binding protein 2	IGF2BP2	0.989771	3.73E-20	202.01	S	NYSFKISYIPDEEVSSPSPPQRAQRGDHSSR	3927300	3106200	7014	
Q9BWU0	466	Kanadaptin	SLC4A1AP	0.999986	8.30E-06	201.96	S	RKRKAKNWEDEDFYDSDDDTFLDRTGLIEKK	0	0	5435	
Q9Y2U8	187	Inner nuclear membrane protein Man1	LEMD3	0.995906	7.44E-21	201.96	S	APPAPLAASEVTNSNSAERRKPHSWWGARRP	829070	304720	6807	
Q9H7D7	121	WD repeat-containing protein 26	WDR26	1	6.66E-19	201.76	S	ATPELGSSLKKKKRLSQSDEDVIRLIGQHLN	0	0	5757	
Q15154	68	Pericentriolar material 1 protein	PCM1	1	5.48E-20	201.75	S	GVESDKRVTNDISPESSPGVGRRRTKTPHTF	577400	909810	2888	
Q93009	18	Ubiquitin carboxyl-terminal hydrolase 7	USP7	1	3.06E-25	201.61	S	HQQQQQQQKAGEQQLSEPEDMEMEAGDTDDP	1406400	2231300	4743	
A6NKD9	178	Coiled-coil domain-containing protein 85C	CCDC85C	0.497876	4.43E-25	201.52	S	AASGGGGGGGGAGSRSSIDSQASLSGPLSGG	0	0	40	
A6NKD9	179	Coiled-coil domain-containing protein 85C	CCDC85C	0.497876	4.43E-25	201.52	S	ASGGGGGGGGAGSRSSIDSQASLSGPLSGGA	0	0	41	
Q86YS7	659	C2 domain-containing protein 5	C2CD5	0.988303	1.89E-20	201.52	S	PIPEPRQRSRLLRSQSESSDEVTELDLSHGK	1352100	693320	4032	
Q86YS7	662	C2 domain-containing protein 5	C2CD5	0.945299	1.89E-20	201.52	S	EPRQRSRLLRSQSESSDEVTELDLSHGKKDA	959650	431130	4034	
Q86XP3	111	ATP-dependent RNA helicase DDX42	DDX42	0.893384	3.09E-25	201.48	S	SPTRQQFHSKPVDSDSDDDPLEAFMAEVEDQ	1850000	675850	4017	
P17987	544	T-complex protein 1 subunit alpha	TCP1	1	6.90E-20	201.16	S	DLIKLHPESKDDKHGSYEDAVHSGALND___	2106700	688250	1125	
Q96AC1	666	Fermitin family homolog 2	FERMT2	1	3.24E-20	201.16	S	GGYIFLSTRAKDQNESLDEEMFYKLTSGWV_	591270	590830	4770	
O14686	1671	Histone-lysine N-methyltransferase 2D	KMT2D	1	2.82E-21	201.14	S	GVEHMECEIKLEGPVSPDVEPGKEETEESKK	1806400	1962200	191	
Q9BW85	211	Coiled-coil domain-containing protein 94	CCDC94	1	3.20E-22	201.14	S	AALLEEARKRRLLEDSDSEDEAAPSPLQPAL	18256000	16116000	5425	
Q9BW85	213	Coiled-coil domain-containing protein 94	CCDC94	1	3.20E-22	201.14	S	LLEEARKRRLLEDSDSEDEAAPSPLQPALRP	17332000	16384000	5426	
P23193	97	Transcription elongation factor A protein 1	TCEA1	0.946946	3.26E-20	201.08	S	TEKDLDEKKKEPAITSQNSPEAREESTSSGN	0	0	1217	
Q5W0B1	719	RING finger protein 219	RNF219	0.98972	4.39E-20	201.08	S	NQSTKRKIQSSLSSASPSKATKS________	7107400	5571800	3373	
Q32MZ4	714	Leucine-rich repeat flightless-interacting protein 1	LRRFIP1	0.954396	3.27E-20	201.06	S	GVAKDNAKIDGATQSSPAEPKSEDADRCTLP	0	1937500	3055	
O14654	779	Insulin receptor substrate 4	IRS4	0.990901	1.20E-21	200.97	Y	TNKEDDSKDNDSESDYMFMAPGAGAIPKNPR	1413600	318750	8342	
P98175	736	RNA-binding protein 10	RBM10	0.999984	2.38E-20	200.94	S	RPSPPRGLVAAYSGESDSEEEQERGGPEREE	160310	2544800	2015	
Q92597	330	Protein NDRG1	NDRG1	0.99999	5.76E-20	200.81	S	MPSASMTRLMRSRTASGSSVTSLDGTRSRSH	15544000	6495400	4649	
Q92597	336	Protein NDRG1	NDRG1	0.874019	5.76E-20	200.81	S	TRLMRSRTASGSSVTSLDGTRSRSHTSEGTR	1271300	1904800	4652	
Q8ND56	192	Protein LSM14 homolog A	LSM14A	0.992515	4.70E-25	200.73	S	LSQGRSSPQLDPLRKSPTMEQAVQTASAHLP	2674200	1361400	4339	

Q8ND56	194	Protein LSM14 homolog A	LSM14A	0.53989	4.70E-25	200.73	T	QGRSSPQLDPLRKSPTMEQAVQTASAHLPAP	4386000	2917800	7888	
O60583	480	Cyclin-T2	CCNT2	0.985421	4.11E-20	200.71	S	HKQGQSQAASSSSVTSPIKMKIPIANTEKYM	2078200	1796400	510	
Q15717	202	ELAV-like protein 1	ELAVL1	1	6.97E-20	200.71	S	PNQNKNVALLSQLYHSPARRFGGPVHHQAQR	1756300	675420	2979	
Q9H6K1	215	Uncharacterized protein C6orf106	C6orf106	1	3.37E-20	200.63	S	TQPHRKVEGNFNPFASPQKNRQSDENNLKDP	1461600	882980	5735	
Q86WB0	354	Nuclear-interacting partner of ALK	ZC3HC1	0.999989	1.54E-21	200.57	S	EQAEKSPGPIVSRTRSWDSSSPVDRPEPEAA	47050000	31387000	3985	
Q86WB0	370	Nuclear-interacting partner of ALK	ZC3HC1	0.999755	1.54E-21	200.57	S	WDSSSPVDRPEPEAASPTTRTRPVTRSMGTG	33119000	23414000	3989	
Q9UNE7	19	E3 ubiquitin-protein ligase CHIP	STUB1	1	9.49E-20	200.45	S	KEEKEGGARLGAGGGSPEKSPSAQELKEQGN	13948000	24201000	6541	
Q9UNE7	23	E3 ubiquitin-protein ligase CHIP	STUB1	0.999967	9.49E-20	200.45	S	EGGARLGAGGGSPEKSPSAQELKEQGNRLFV	10693000	14429000	6542	
P46100	677	Transcriptional regulator ATRX	ATRX	0.999855	1.69E-21	200.2	S	TPLRRPTETNPVTSNSDEECNETVKEKQKLS	11883000	10826000	1558	
Q13620	146	Cullin-4B	CUL4B	0.980083	2.00E-25	200	S	DAKMAEESSSSSSSSPTAATSQQQQLKNKS	10120000	3911000	2616	
P17812	574	CTP synthase 1	CTPS1	0.928128	7.35E-20	199.96	S	CRLSPRDTYSDRSGSSSPDSEITELKFPSIN	0	6516400	1122	
O60293	1303	Zinc finger C3H1 domain-containing protein	ZFC3H1	0.996083	3.31E-21	199.7	S	WKPKFWRKPISDNSFSSDEEQSTGPIKYAFQ	3689100	0	472	
O60293	1304	Zinc finger C3H1 domain-containing protein	ZFC3H1	0.998616	3.31E-21	199.7	S	KPKFWRKPISDNSFSSDEEQSTGPIKYAFQP	3689100	0	473	
O14497	696	AT-rich interactive domain-containing protein 1A	ARID1A	0.999669	4.44E-20	199.61	S	FSPHTSPHLPGIRGPSPSPVGSPASVAQSRS	5648700	4486600	140	
P55196	1182	Afadin	MLLT4	1	7.70E-20	199.61	S	RADHRSSPNVANQPPSPGGKSAYASGTTAKI	4191900	2378500	1863	
Q5JTD0	300	Tight junction-associated protein 1	TJAP1	1	7.65E-20	199.38	S	PQPNGECHSLGTARGSPEEELPLPAFEKLNP	2480400	2156700	3195	
O75822	11	Eukaryotic translation initiation factor 3 subunit J	EIF3J	0.999999	8.88E-26	199.37	S	_____MAAAAAAAGDSDSWDADAFSVEDPVR	4873600	5512600	665	
Q02880	1522	DNA topoisomerase 2-beta	TOP2B	1	3.76E-20	199.36	S	PKRAPKQKKVVEAVNSDSDSEFGIPKKTTTP	15120000	13870000	2108	
Q02880	1524	DNA topoisomerase 2-beta	TOP2B	1	3.76E-20	199.36	S	RAPKQKKVVEAVNSDSDSEFGIPKKTTTPKG	18188000	13870000	2109	
Q96EV2	41	RNA-binding protein 33	RBM33	1	5.16E-25	199.36	S	AERSWRRRAADEDWDSELEDDLLGEDLLSGK	2259400	1371600	4891	
P27816	510	Microtubule-associated protein 4	MAP4	0.998917	6.38E-20	199.33	S	STVKEVGLLKDMSPLSETEMALGKDVTPPPE	2186300	1645900	1291	
P45880	115	Voltage-dependent anion-selective channel protein 2	VDAC2	0.99035	3.69E-20	199.33	S	DQICQGLKLTFDTTFSPNTGKKSGKIKSSYK	3190300	0	1529	
Q13098	474	COP9 signalosome complex subunit 1	GPS1	1	7.84E-20	199.33	S	VLRNQIHVKSPPREGSQGELTPANSQSRMST	3175000	4997400	2398	
Q6ZS17	351	Protein FAM65A	FAM65A	0.999957	7.84E-20	199.33	S	NKASTVTKRFSTYSQSPPDTPSLREQAFYNM	3086200	1514800	3669	
Q9NZJ0	512	Denticleless protein homolog	DTL	0.986639	4.82E-19	199.33	S	FKMSIRNWVTRTPSSSPPITPPASETKIMSP	3477400	2091900	6157	
Q13428	1257	Treacle protein	TCOF1	1	1.96E-19	199.13	S	DGKQEAKPQQAAGMLSPKTGGKEAASGTTPQ	3042800	3000400	2511	
P49736	41	DNA replication licensing factor MCM2	MCM2	0.983786	7.51E-19	199.12	S	SSPGRSSRRTDALTSSPGRDLPPFEDESEGL	11588000	5964500	1683	
P27448;Q7211;208;2		MAP/microtubule affinity-regulating kinase 3;Serine/threonir	MARK3;MARK2	0.999977	3.74E-20	199.05	T	DFGFSNEFTFGNKLDTFCGSPPYAAPELFQG;DFGI	1021100	301940	7330	
Q9UPP1	857	Histone lysine demethylase PHF8	PHF8	0.999997	9.18E-21	199.01	S	GACFKDAEYIYPSLESDDDDPALKSRPKKKK	4385300	2332500	6565	
O43818	51	U3 small nucleolar RNA-interacting protein 2	RRP9	0.996921	3.85E-20	198.98	S	RGKSKGGGKMNEEISSDSESESLAPRKPEEE	0	537150	427	
Q96B97	230	SH3 domain-containing kinase-binding protein 1	SH3KBP1	1	3.81E-20	198.81	S	FGDIFKDKPIKLRPRSIEVENDFLPVEKTIG	1338100	918530	4804	
Q5VTR2	138	E3 ubiquitin-protein ligase BRE1A	RNF20	1	4.70E-20	198.74	S	ERKALVVPEPEPDSDSNQERKDDRERGEGQE	24997000	14786000	3339	
P10075	86	Zinc finger protein GLI4	GLI4	0.999824	6.48E-20	198.61	S	TFWPDSEPKPEQAPRSPGSQAPDEGAGGALR	1891300	1640600	982	
Q13129	632	Zinc finger protein Rlf	RLF	0.97211	4.74E-20	198.61	S	KKLLLKGSQKGICPKSPSAIPEQNHSLNDQA	4181700	1886500	2412	
Q13129	634	Zinc finger protein Rlf	RLF	0.5	4.74E-20	198.61	S	LLLKGSQKGICPKSPSAIPEQNHSLNDQAKG	1128000	715840	2413	
A6NKF1	402	SAC3 domain-containing protein 1	SAC3D1	1	8.33E-22	198.6	S	VVMAEEEDEGTDRPGSPA_____________	2952700	3006400	43	
P78332	360	RNA-binding protein 6	RBM6	1	2.14E-30	198.54	S	QGVAFEHESPADFQNSQSPVQDQDKSQLSGR	16637000	7615900	1956	
P78332	362	RNA-binding protein 6	RBM6	1	2.14E-30	198.54	S	VAFEHESPADFQNSQSPVQDQDKSQLSGREE	3968200	17514000	1957	
O00567	519	Nucleolar protein 56	NOP56	1	7.88E-21	198.51	S	SKPKKKKSFSKEELMSSDLEETAGSTSIPKR	6742500	6742800	134	
O15014	358	Zinc finger protein 609	ZNF609	0.999997	4.35E-20	198.48	S	LDCTRHDWAPPRFCDSPTSDLEMRNGRGRGK	2380700	2352600	224	
O15014	361	Zinc finger protein 609	ZNF609	0.867051	4.35E-20	198.48	S	TRHDWAPPRFCDSPTSDLEMRNGRGRGKRMR	1318500	1515900	225	
Q9NQP4	125	Prefoldin subunit 4	PFDN4	1	5.23E-20	198.48	S	RVLADLKVQLYAKFGSNINLEADES______	2756000	2579300	5917	
P35269	385	General transcription factor IIF subunit 1	GTF2F1	0.999952	2.89E-21	198.47	S	PKRERKPSGGSSRGNSRPGTPSAEGGSTSST	13737000	9374600	1389	
P35269	391	General transcription factor IIF subunit 1	GTF2F1	0.99775	2.89E-21	198.47	S	PSGGSSRGNSRPGTPSAEGGSTSSTLRAAAS	11604000	8479600	1390	
Q92793	121	CREB-binding protein	CREBBP	0.999999	8.33E-20	198.38	S	QPNSANMASLSAMGKSPLSQGDSSAPSLPKQ	1145200	666820	4705	
P48634	380	Protein PRRC2A	PRRC2A	0.999979	1.62E-19	198.29	S	SGEERPPEADGKKGNSPNSEPPTPKTAWAET	1465700	1014900	1625	
P48634	761	Protein PRRC2A	PRRC2A	0.990422	3.94E-20	198.29	S	PGVHPSGLVPRERSDSGGSSSEPFDRHAPAM	960180	732860	1630	
Q15021	1333	Condensin complex subunit 1	NCAPD2	0.990149	9.76E-21	198.05	S	KPSTGSRYQPLASTASDNDFVTPEPRRTTRR	2081000	1375800	2855	
Q96GN5	21	Cell division cycle-associated 7-like protein	CDCA7L	1	9.82E-21	197.94	S	RYQIPKEVADIFNAPSDDEEFVGFRDDVPME	1268300	1436900	4920	
Q08999	690	Retinoblastoma-like protein 2	RBL2	0.947749	6.84E-20	197.75	S	ASTTRRRLFVENDSPSDGGTPGRMPPQPLVN	827440	1019000	2253	
P55196	1172	Afadin	MLLT4	1	8.67E-20	197.73	S	LPGDDRLMKNRADHRSSPNVANQPPSPGGKS	2833800	0	1861	
P55196	1173	Afadin	MLLT4	1	8.67E-20	197.73	S	PGDDRLMKNRADHRSSPNVANQPPSPGGKSA	2696400	400540	1862	
Q7Z309	115	Protein FAM122B	FAM122B	0.999999	7.05E-20	197.73	S	PEKLYSPKRIDFTPVSPAPSPTRGFGKMFVS	31617000	36411000	3771	
Q7Z309	119	Protein FAM122B	FAM122B	0.999421	7.05E-20	197.73	S	YSPKRIDFTPVSPAPSPTRGFGKMFVSSSGL	34666000	38458000	3772	
Q7Z6Z7	2362	E3 ubiquitin-protein ligase HUWE1	HUWE1	1	3.39E-20	197.73	S	ELEDLIDELLERDGGSGNSTIIVSRSGEDES	6263900	4450200	3870	
Q7Z6Z7	2365	E3 ubiquitin-protein ligase HUWE1	HUWE1	0.950814	3.39E-20	197.73	S	DLIDELLERDGGSGNSTIIVSRSGEDESQED	2373700	2191400	3871	
Q92576	1614	PHD finger protein 3	PHF3	0.969535	9.75E-23	197.6	S	EDQENNLQDNQTSNSSPCRSNVGKGNIDGNV	9842900	9461400	4643	
Q8N9T8	136	Protein KRI1 homolog	KRI1	0.999997	8.78E-20	197.51	S	KVILEKAGKYVDEENSDGETSNHRLQETSSQ	0	0	4308	
P36915	50	Guanine nucleotide-binding protein-like 1	GNL1	0.890242	2.07E-19	197.51	T	NSRSGSRERREEQTDTSDGESVTHHIRRLNQ	297420	4694100	7372	
Q5T200	986	Zinc finger CCCH domain-containing protein 13	ZC3H13	0.998741	5.22E-16	197.41	S	KEDDVGIERGNIETTSEDGQVFSPKKGQKKK	0	637190	3255	
Q6UUV7	368	CREB-regulated transcription coactivator 3	CRTC3	0.520917	1.21E-16	197.41	S	SVPNASALHPSLRLFSLSNPSLSTTNLSGPS	0	0	3589	
Q9NYF8	222	Bcl-2-associated transcription factor 1	BCLAF1	0.999846	1.63E-17	197.41	S	ATSGDIWPGLSAYDNSPRSPHSPSPIATPPS	13418000	13774000	6114	
Q96EB6	14	NAD-dependent protein deacetylase sirtuin-1;SirtT1 75 kDa fr	SIRT1	0.999329	3.08E-17	197.05	S	__MADEAALALQPGGSPSAAGADREAASSPA	9323100	9107500	4882	
P18887	241	DNA repair protein XRCC1	XRCC1	1	9.94E-15	196.97	S	SPVSRAIGSTSKPQESPKGKRKLDLNQEEKK	2276900	2672600	1156	
P29966	150	Myristoylated alanine-rich C-kinase substrate	MARCKS	0.99983	2.10E-14	196.97	T	SPKAEDGATPSPSNETPKKKKKRFSFKKSFK	0	1016700	7344	
Q9NYF8	512	Bcl-2-associated transcription factor 1	BCLAF1	1	2.16E-14	196.67	S	PEQVKSEKLKDLFDYSPPLHKNLDAREKSTF	22449000	15585000	6103	
Q969V6	458	MKL/myocardin-like protein 1	MKL1	0.99975	3.95E-14	196.65	S	KFGSTGSTPPVSPTPSERSLLSTGDENSTPG	2914700	7346100	4754	
Q9Y3C6	149	Peptidyl-prolyl cis-trans isomerase-like 1	PPIL1	0.998998	3.62E-14	196.65	S	QGIGMVNRVGMVETNSQDRPVDDVKIIKAYP	0	1573300	6854	
P43250;P3484;484		G protein-coupled receptor kinase 6;G protein-coupled recept	GRK6;GRK5	1	1.10E-16	196.37	S	PQAIYCKDVLDIEQFSTVKGVELEPTDQDFY	630550	611110	1373	
Q9NQC3	13	Reticulon-4	RTN4	0.917649	2.17E-19	196.36	S	___MEDLDQSPLVSSSDSPPRPQPAFKYQFV	0	12921000	5911	
P43250;P3485;485		G protein-coupled receptor kinase 6;G protein-coupled recept	GRK6;GRK5	1	1.10E-16	196.18	T	QAIYCKDVLDIEQFSTVKGVELEPTDQDFYQ	0	0	7354	
Q9Y520	1248	Protein PRRC2C	PRRC2C	0.996679	3.52E-14	196.16	S	SGPLRQREESETRSESSDFEVVPKRRRQRGS	8946800	3677700	6947	
O15258	95	Protein RER1	RER1	1	8.56E-15	196.01	S	IAFLSPKVDPSLMEDSDDGPSLPTKQNEEFR	4468000	5658100	310	
Q08J23	452	tRNA (cytosine(34)-C(5))-methyltransferase	NSUN2	0.482574	1.84E-15	196.01	S	KRQPKLQGKSAETRESTQLSPADLTEGKPTD	0	0	2267	
Q08J23	453	tRNA (cytosine(34)-C(5))-methyltransferase	NSUN2	0.783523	1.84E-15	196.01	T	RQPKLQGKSAETRESTQLSPADLTEGKPTDP	6395100	2631900	7506	
Q12873	713	Chromodomain-helicase-DNA-binding protein 3	CHD3	0.965967	8.97E-15	195.94	S	YKKKKKELQGDGPPSSPTNDPTVKYETQPRF	2124000	1702600	2336	
Q9NW75	115	G patch domain-containing protein 2	GPATCH2	1	6.79E-15	195.94	S	SKDYRENHNNNKKDHSDSDDQMLVAKRRPSS	603450	1110500	6053	
Q15149	4386	Plectin	PLEC	0.976211	9.08E-15	195.92	S	DMLSGNAGGFRSRSSSVGSSSSYPISPAVSR	3971000	0	2877	
Q9Y608	320	Leucine-rich repeat flightless-interacting protein 2	LRRFIP2	0.999101	1.81E-14	195.72	S	TRPSSRNSASATTPLSGNSSRRGSGDTSSLI	1240100	504210	6975	
Q9NYV4	1083	Cyclin-dependent kinase 12	CDK12	0.999291	7.80E-15	195.71	S	KETTSGTSTEPVKNSSPAPPQPAPGKVESGA	27141000	20427000	6127	
P11171	555	Protein 4.1	EPB41	0.999998	9.05E-14	195.6	S	SRSLDGAAAVDSADRSPRPTSAPAITQGQVA	3632300	1060800	1008	
Q15788	22	Nuclear receptor coactivator 1	NCOA1	0.999999	6.66E-14	195.6	S	SSSDPANPDSHKRKGSPCDTLASSTEKRRRE	577480	443990	2987	
Q7Z417	112	Nuclear fragile X mental retardation-interacting protein 2	NUFIP2	0.999202	8.29E-15	195.6	S	LNGNAGEREISLKNLSSDEATNPISRVLNGN	4582300	2499800	3797	
Q9Y3S2	293	Zinc finger protein 330	ZNF330	0.344412	8.44E-15	195.6	T	EDDEEEEDEGRKDSDTESSDLFTNLNLGRTY	0	0	8293	
Q9Y2W1	928	Thyroid hormone receptor-associated protein 3	THRAP3	1	1.80E-11	195.59	S	FRKSSTSPKWAHDKFSGEEGEIEDDESGTEN	4614900	4873200	6819	
Q96Q45	47	Transmembrane protein 237	TMEM237	0.970294	1.89E-14	195.55	S	SRPKKKKPRTKNTPASASLEGLAQTAGRRPS	0	0	5072	
Q92597	328	Protein NDRG1	NDRG1	0.999868	1.87E-13	195.48	T	GYMPSASMTRLMRSRTASGSSVTSLDGTRSR	2181900	1343000	7934	
Q9BUR4	85	Telomerase Cajal body protein 1	WRAP53	0.999835	4.05E-19	195.43	S	GDPVSLSTPLETEFGSPSELSPRIEEQELSE	11155000	12605000	5384	
Q7Z4V5	137	Hepatoma-derived growth factor-related protein 2	HDGFRP2	0.999849	2.36E-14	195.28	S	DRGVMAVTAVTATAASDRMESDSDSDKSSDN	277630	294830	3831	
P49840	20	Glycogen synthase kinase-3 alpha	GSK3A	0.333333	4.37E-29	195.25	S	GPSGGGPGGSGRARTSSFAEPGGGGGGGGGG	0	0	1733	
P49840	21	Glycogen synthase kinase-3 alpha	GSK3A	0.333333	4.37E-29	195.25	S	PSGGGPGGSGRARTSSFAEPGGGGGGGGGGP	0	0	1734	
P49840	19	Glycogen synthase kinase-3 alpha	GSK3A	0.333333	4.37E-29	195.25	T	GGPSGGGPGGSGRARTSSFAEPGGGGGGGGG	0	0	7427	
Q96N21	356	AP-4 complex accessory subunit tepsin	ENTHD2	1	9.93E-15	195.23	S	ATKILRHFEASCGQLSPARGTSAEPGPTAAL	1581300	1051500	5024	
O75164	523	Lysine-specific demethylase 4A	KDM4A	0.999716	2.44E-14	195.2	S	SSSSLGSGSSRDSISSDSETSEPLSCRAQGQ	1254100	896950	579	
Q01105	63	Protein SET	SET	1	6.99E-14	194.86	S	IDEVQNEIDRLNEQASEEILKVEQKYNKLRQ	1487500	2227000	2051	
Q5UIP0	2144	Telomere-associated protein RIF1	RIF1	1	8.30E-24	194.77	S	SGTAISELIIEDNNASPQKLRELDPSLVSAN	3467600	3409900	3317	
Q15836;P(63;80		Vesicle-associated membrane protein 3;Vesicle-associated mem	VAMP3;VAMP2	0.917496	2.75E-14	194.67	S	DRADALQAGASQFETSAAKLKRKYWWKNCKM	1151300	969860	1934	
Q09666	5731	Neuroblast differentiation-associated protein AHNAK	AHNAK	0.999914	2.90E-14	194.67	S	KFNFSKPKGKGGVTGSPEASISGSKGDLKSS	10147000	5595100	2276	
Q14966	128	Zinc finger protein 638	ZNF638	1	1.24E-14	194.67	S	SVAVKQSSVTQVTEQSPKVQSRYTKESASSI	924540	567720	2824	
Q9UQ35	437	Serine/arginine repetitive matrix protein 2	SRRM2	0.973727	2.24E-14	194.67	S	PPSPQPTKVSRHASSSPESPKPAPAPGSHRE	15063000	5122500	6637	
Q9UQ35	440	Serine/arginine repetitive matrix protein 2	SRRM2	0.999969	2.24E-14	194.67	S	PQPTKVSRHASSSPESPKPAPAPGSHREISS	43607000	19478000	6638	
O60841	137	Eukaryotic translation initiation factor 5B	EIF5B	0.999997	2.45E-09	194.58	S	KSKKTAKPKVEMYSGSDDDDDFNKLPKKAKG	5711700	3786600	536	
O60841	134	Eukaryotic translation initiation factor 5B	EIF5B	0.943999	1.89E-05	194.58	Y	KDSKSKKTAKPKVEMYSGSDDDDDFNKLPKK	0	0	8348	
Q9Y520	1249	Protein PRRC2C	PRRC2C	0.999792	8.01E-14	194.48	S	GPLRQREESETRSESSDFEVVPKRRRQRGSE	1398200	1172100	6948	
Q96CW6	308	Probable RNA polymerase II nuclear localization protein SLC7	SLC7A6OS	1	1.36E-14	194.39	S	DVQKEFGYDSPHDLDSD______________	4920800	2438300	4828	
Q969V6	449	MKL/myocardin-like protein 1	MKL1	0.8494	2.57E-14	194.32	S	ATVASSGVVKFGSTGSTPPVSPTPSERSLLS	26898	109140	4752	4.057550747
Q9NZN5	1286	Rho guanine nucleotide exchange factor 12	ARHGEF12	0.5	2.82E-14	194.15	T	EKSVQEDWQHFPRYRTASQGPQTDSVIQNSE	0	0	8182	
Q9UPN3	4521	Microtubule-actin cross-linking factor 1, isoforms 1/2/3/5	MACF1	1	2.38E-14	194.12	S	QAESNKAFLAELEQNSPKIQKVKEALAGLLV	1235100	915340	6556	
O15027	1939	Protein transport protein Sec16A	SEC16A	0.996582	1.02E-29	194.11	S	GALGIANPLLAVPAPSPEHSSPSVRLLPSAP	923060	669490	244	
Q96AP0	425	Adrenocortical dysplasia protein homolog	ACD	0.961974	3.01E-14	194.1	S	ATLTPRSPHASRTPSSPLQSCTPSLSPRSHV	840390	558410	4774	
Q96AP0	435	Adrenocortical dysplasia protein homolog	ACD	0.997349	3.01E-14	194.1	S	SRTPSSPLQSCTPSLSPRSHVPSPHQALVTR	840390	558410	4775	
Q9UDY2	130	Tight junction protein ZO-2	TJP2	1	3.32E-14	194.1	S	VVKRPRKVQVAALQASPPLDQDDRAFEVMDE	2179300	2196100	6283	
Q8N3U4	1064	Cohesin subunit SA-2	STAG2	0.816473	4.90E-16	194.04	S	LAGGDDDTMSVISGISSRGSTVRSKKSKPST	484440	366540	4259	
Q2VPK5	419	Cytoplasmic tRNA 2-thiolation protein 2	CTU2	1	3.00E-14	194.01	S	TAFGAQTSSRLSQMQSPIPLTETRTPPGPCC	1018700	1211600	3050	
P08670	56	Vimentin	VIM	0.991453	3.03E-14	193.98	S	GSALRPSTSRSLYASSPGGVYATRSSAVRLR	6263100	2434600	965	
P23193	100	Transcription elongation factor A protein 1	TCEA1	0.999999	1.55E-14	193.98	S	DLDEKKKEPAITSQNSPEAREESTSSGNVSN	1584500	2776300	1218	
Q9UER7	495	Death domain-associated protein 6	DAXX	1	2.48E-14	193.98	S	EDEEEEAAAGKDGDKSPMSSLQISNEKNLEP	1304000	817340	6291	
P46100	1992	Transcriptional regulator ATRX	ATRX	0.728375	3.28E-14	193.8	S	PSVSLKLEESKATSSSNPSSPAPDWYKDFVT	0	859460	1548	
P46100	1996	Transcriptional regulator ATRX	ATRX	0.987988	3.28E-14	193.8	S	LKLEESKATSSSNPSSPAPDWYKDFVTDADA	2617800	859460	1549	
A1L390	827	Pleckstrin homology domain-containing family G member 3	PLEKHG3	0.999938	3.21E-14	193.77	S	SEIVKIWEGMESSGGSPGKGPGQGQANGFDL	2316100	1254400	18	
Q9P2K5	17	Myelin expression factor 2	MYEF2	0.999827	8.84E-19	193.76	S	ADANKAEVPGATGGDSPHLQPAEPPGEPRRE	20491000	11547000	6243	
P10636	519	Microtubule-associated protein tau	MAPT	0.999967	3.98E-14	193.62	S	EPPKSGDRSGYSSPGSPGTPGSRSRTPSLPT	1766200	1247200	997	
Q8N3X1	116	Formin-binding protein 4	FNBP4	0.999977	9.26E-19	193.59	S	VKATGGLCLLGAYADSDDDDNDVSEKLAQSK	3556500	3906700	4264	
O43491	550	Band 4.1-like protein 2	EPB41L2	1	4.23E-14	193.54	S	PAPHFERTSSKRVSRSLDGAPIGVMDQSLMK	1404200	642610	394	
Q8TEK3	374	Histone-lysine N-methyltransferase, H3 lysine-79 specific	DOT1L	1	3.87E-14	193.54	S	GPEGKVAGPADAPMDSGAEEEKAGAATVKKP	1500300	1869200	4482	
Q2NKX8	1028	DNA excision repair protein ERCC-6-like	ERCC6L	1	1.76E-14	193.51	S	VVVKAKIRSKARRIVSDGEDEDDSFKDTSSI	399330	579550	3045	
O43719	702	HIV Tat-specific factor 1	HTATSF1	1	4.79E-13	193.49	S	KEVEDADEKLFEDDDSNEKLFDEEEDSSEKL	11165000	11970000	412	
P31749	122	RAC-alpha serine/threonine-protein kinase	AKT1	0.497748	8.33E-15	193.47	S	ADGLKKQEEEEMDFRSGSPSDNSGAEEMEVS	0	0	1358	
P31749	124	RAC-alpha serine/threonine-protein kinase	AKT1	0.497748	8.33E-15	193.47	S	GLKKQEEEEMDFRSGSPSDNSGAEEMEVSLA	0	0	1359	
Q96ST3	1112	Paired amphipathic helix protein Sin3a	SIN3A	0.998976	2.85E-14	193.45	S	RWSDYVERYMNSDTTSPELREHLAQKPVFLP	0	4459900	5142	
O00178	580	GTP-binding protein 1	GTPBP1	0.999975	6.54E-14	193.42	S	KAVGTITKLLQTTNNSPMNSKPQQIKMQSTK	11967000	6596000	99	
Q9Y6X9	730	MORC family CW-type zinc finger protein 2	MORC2	0.99799	1.56E-12	193.42	S	IKTPVVKKTESPIKLSPATPSRKRSVAVSDE	2630400	0	7033	
Q9Y6X9	723	MORC family CW-type zinc finger protein 2	MORC2	0.990169	1.56E-12	193.42	T	EVPSPKVIKTPVVKKTESPIKLSPATPSRKR	830380	0	8325	
P49790	632	Nuclear pore complex protein Nup153	NUP153	0.890084	9.69E-14	193.41	T	FASPKIDSVAAQPTATSPVVYTRPAISSFSS	650570	0	7418	
P06493;P215;15;15		Cyclin-dependent kinase 1;Cyclin-dependent kinase 2;Cyclin-c	CDK1;CDK2;(	1	2.08E-13	193.38	Y	_MEDYTKIEKIGEGTYGVVYKGRHKTTGQVV	61715000	55505000	8352	
Q9UQ35	435	Serine/arginine repetitive matrix protein 2	SRRM2	0.991622	3.29E-14	193.36	S	SSPPSPQPTKVSRHASSSPESPKPAPAPGSH	47611000	19478000	6635	
Q9NZN8	165	CCR4-NOT transcription complex subunit 2	CNOT2	0.999999	3.60E-14	193.32	S	GIPSRTNSMSSSGLGSPNRSSPSIICMPKQQ	12878000	6360300	6166	
Q02952	286	A-kinase anchor protein 12	AKAP12	0.986229	2.44E-14	193.31	S	EKQEKEPSKSAESPTSPSVTSETGSTFKKFFT	3136900	660470	2119	
Q9UQ35	1179	Serine/arginine repetitive matrix protein 2	SRRM2	1	4.31E-14	193.31	S	SKEKMALPPQEDATASPPRQKDKFSPFPVQD	3431300	2006500	6654	

Q04724	285	Transducin-like enhancer protein 1	TLE1	0.809749	8.67E-15	193.3	S	ENGIDKNRLLKKDASSSPASTASSASSTSLK	0	79122	2169
Q13428	1228	Treacle protein	TCOF1	0.998962	3.63E-14	193.28	S	PANSQASKATPKLDSSPSVSSTLAAKDDPDG	18972000	20023000	2509
O15541	253	RING finger protein 113A	RNF113A	1	7.56E-16	193.06	S	EGRYGVYEDENYEVGSDDEEIPFKCFICRQS	832650	1389500	333
Q9NVU7	585	Protein SDA1 homolog	SDAD1	1	1.20E-13	193	S	AAPGKSQKRKYIEIDSDEEPRGELLSLRDIE	15740000	12182000	6050
Q9UHI6	677	Probable ATP-dependent RNA helicase DDX20	DDX20	1	2.64E-14	193	S	RTSSQSKGNKSYLEGSSDNQLKDSESTPVDD	2366600	1293100	6356
Q9UHI6	678	Probable ATP-dependent RNA helicase DDX20	DDX20	1	2.64E-14	193	S	TSSQSKGNKSYLEGSSDNQLKDSESTPVDDR	4039000	2679700	6357
P60981	3	Destrin	DSTN	1	1.49E-14	192.54	S	_____________MASGVQVADEVCRIFYDM	16110000	19180000	1905
Q8TD19	332	Serine/threonine-protein kinase Nek9	NEK9	0.810124	5.19E-14	192.46	S	EKVTLLNAPTKRPRSSTVTEAPIAVVTSRTS	775540	678590	4465
O14654	777	Insulin receptor substrate 4	IRS4	0.907463	1.41E-15	192.4	S	PDTNKEDDSKDNDSESDYMFMAPGAGAIPKN	3938900	4310200	172
P49916	242	DNA ligase 3	LIG3	0.969372	4.40E-14	192.4	S	SGFSAKPNNSGEAPSSPTPKRSLSSSKCDPR	1941200	1919800	1742
Q9P035	135	Very-long-chain (3R)-3-hydroxyacyl-CoA dehydratase 3	HACD3	0.999922	4.40E-14	192.4	S	LRAKEEERLNKLRLESEGSPETLTNLRKGYL	659130	570600	6178
Q6ZRP7	578	Sulfhydryl oxidase 2	QSOX2	0.656618	3.60E-16	192.27	S	GRDNLLDTYSADQGDSSEGGTLARGEEEEKR	1140400	0	3653
Q96D46	468	60S ribosomal export protein NMD3	NMD3	1	1.35E-13	192.24	S	RKNVNIYRDSAIPVESDTDDEGAPRISLAEM	1378100	5784400	4839
Q9Y6D5	227	Brefeldin A-inhibited guanine nucleotide-exchange protein 2	ARFGEF2	1	4.54E-14	192.24	S	IQSKPQSPVIQAAAVSPKFVRLKHSQAQSKP	4947500	2979100	6993
Q15149	4384	Plectin	PLEC	0.418218	3.06E-14	192.23	S	FADMLSGNAGGFRSRSSSVGSSSSYPISPAV	0	0	2875
Q15149	4385	Plectin	PLEC	0.418218	3.06E-14	192.23	S	ADMLSGNAGGFRSRSSSVGSSSSYPISPAVS	0	0	2876
Q9H0B6	589	Kinesin light chain 2	KLC2	0.999999	5.15E-14	192.23	S	NPRMKRASSLNFLNKSVEEPTQPGGTGLSDS	0	1196900	5562
Q9NYF8	285	Bcl-2-associated transcription factor 1	BCLAF1	0.984508	4.51E-14	192.22	S	ERSGSGSVGNGSSRYSPSQNSPIHHIPSRRS	318810000	10931000	6115
Q9NYF8	297	Bcl-2-associated transcription factor 1	BCLAF1	0.999985	4.51E-14	192.22	S	SRYSPSQNSPIHHIPSRRSPAKTIAPQNAPR	0	39278000	6118
O75822	13	Eukaryotic translation initiation factor 3 subunit J	EIF3J	0.999999	3.31E-19	191.95	S	___MAAAAAAAGDSDSWDADAFSVEDPVRKV	1354100	39581000	666
Q15649	80	Zinc finger HIT domain-containing protein 3	ZNHIT3	1	2.19E-05	191.89	S	VENKDDDDSIADFLNSDEEEDRVSLQNLKNL	0	0	2971
Q9NTJ3	27	Structural maintenance of chromosomes protein 4	SMC4	0.925882	1.33E-18	191.89	S	RRREEGPPPPSPDGASSDAEPEPPSGRTESP	9533300	10731000	6015
Q9NTJ3	28	Structural maintenance of chromosomes protein 4	SMC4	0.963932	1.33E-18	191.89	S	RREEGPPPPSPDGASSDAEPEPPSGRTESPA	23925000	17190000	6016
Q7Z5K2	221	Wings apart-like protein homolog	WAPAL	0.999975	1.98E-13	191.63	S	ETNDTWNSQFGKRPESPSEISPIKGSVRTGL	6902800	5724600	3841
Q7Z5K2	226	Wings apart-like protein homolog	WAPAL	1	1.98E-13	191.63	S	WNSQFGKRPESPSEISPIKGSVRTGLFEWDN	3308400	3616300	3843
P55201	1076	Peregrin	BRPF1	1	4.06E-16	191.61	S	HSMVRKSLGRGAGWLSEDEDSPLDALDLVWA	446640	231130	1880
P78559	1675	Microtubule-associated protein 1A;MAP1A heavy chain;MAP1 lig	MAP1A	0.998425	6.38E-14	191.53	S	PAGEQKELAPAWEDTSPEQDNRYWRGREDVA	4155300	2121200	1975
Q641Q2;Q	1180;147	WASH complex subunit FAM21A;Putative WASH complex subunit F	FAM21A;FAM	0.999751	1.03E-15	191.47	S	GKAKSPMFPALGEASSDDDLFQSAKPKPAKK	1792800	1635100	3393
O60583	601	Cyclin-T2	CCNT2	0.999999	3.55E-14	191.41	S	GSKHSADGIPPTVLRSPVGLSSDGISSSSSS	2797300	1399700	511
Q03468	1348	DNA excision repair protein ERCC-6	ERCC6	0.998131	5.97E-14	191.41	S	KKRNSNFSVQHPSSTSPTEKCQDGIMKKEGK	0	712670	2151
Q14258	100	E3 ubiquitin/ISG15 ligase TRIM25	TRIM25	0.999561	5.97E-14	191.41	S	PPADVWTPPARASAPSPNAQVACDHCLKEAA	0	372580	2733
Q8WWM7	559	Ataxin-2-like protein	ATXN2L	0.996658	5.97E-14	191.41	S	ELRKFGAQFKLQPSSSPENSLDPFPPRILKE	3841400	3686900	4564
Q9H410	81	Kinetochore-associated protein DSN1 homolog	DSN1	0.997813	6.51E-14	191.41	S	DLSHQERLQSKSLHLSPQEQSASYQDRRQSW	0	1825000	5654
Q9UGP4	316	LIM domain-containing protein 1	LIMD1	0.917804	6.27E-14	191.41	S	LALSCPRQGGLPRSNSGLGGEVSGVMSKPNV	0	0	6314
Q86UE8	770	Serine/threonine-protein kinase tousled-like 2	TLK2	0.492543	8.46E-19	191.36	S	AGAAIASTSGASNNSSSN_____________	0	0	3929
Q86UE8	771	Serine/threonine-protein kinase tousled-like 2	TLK2	0.715633	8.46E-19	191.36	S	GAAIASTSGASNNSSSN______________	500430	0	3930
P98175	89	RNA-binding protein 10	RBM10	0.999988	3.76E-07	191.33	S	PGSETQRRRRRRHRHSPTGPPGFPRDGDYRD	1804800	2228200	2017
O96018	9	Amyloid beta A4 precursor protein-binding family A member 3	APBA3	0.985542	5.45E-14	191.19	S	_______MDFPTISRSPSGPPAMDLEGPRDI	1475000	817420	849
Q92945	274	Far upstream element-binding protein 2	KHSRP	0.93532	6.54E-14	191.15	S	LQERAGVKMILIQDGSQNTNVDKPLRIIGDP	0	234030	4736
Q6KC79	350	Nipped-B-like protein	NIPBL	0.999928	4.69E-14	190.85	S	EKEQSEKAAMYDIISSPSKDSTKLTLRLSRV	3428400	1997400	3484
P48681	768	Nestin	NES	1	3.10E-13	190.82	S	ETLRTLEKETQQRRRSLGEQDQMTLRPPEKV	1279700	434910	1632
Q5R372	118	Rab GTPase-activating protein 1-like	RABGAP1L	0.613802	6.54E-14	190.82	S	LILDPSNTEISTPRPSSPGGLPEEDSVLFNK	16494000	0	3209
O60343	750	TBC1 domain family member 4	TBC1D4	0.557972	1.45E-14	190.8	S	DTASESSDGEGRKRTSSTCSNESLSVGGTSV	1403100	550340	496
O60343	751	TBC1 domain family member 4	TBC1D4	0.547394	1.45E-14	190.8	S	TASESSDGEGRKRTSSTCSNESLSVGGTSVT	0	550340	497
O60343	749	TBC1 domain family member 4	TBC1D4	0.557972	1.45E-14	190.8	T	QDTASESSDGEGRKRTSSTCSNESLSVGGTS	1403100	0	7182
O00178	25	GTP-binding protein 1	GTPBP1	0.964281	2.29E-18	190.79	S	MDSPVPASMFAPEPSSPGAARAAAAAARLHG	8201000	7051800	103
Q9H2P0	953	Activity-dependent neuroprotector homeobox protein	ADNP	1	1.32E-14	190.64	S	TIHLTEEPTKLMHNASDSEVDQDDVVEWKDG	5508500	4440100	5619
Q3KQU3	544	MAP7 domain-containing protein 1	MAP7D1	0.999996	1.95E-18	190.63	S	SKRRASNEKESAAPASPAPSPAPSPTPAPPQ	33668000	22235000	3059
Q3KQU3	552	MAP7 domain-containing protein 1	MAP7D1	0.993089	1.95E-18	190.63	S	KESAAPASPAPSPAPSPTPAPPQKEQPPAET	1775100	7322200	3061
Q71RC2	583	La-related protein 4	LARP4	0.989742	6.79E-14	190.57	S	SSVQKDGLNQTTIPVSPPSTTKPSRASTASP	1782000	0	3696
Q9BPX3	1015	Condensin complex subunit 3	NCAPG	1	4.89E-14	190.57	S	EKSKLNLAQFLNEDLS_______________	1489600	1613900	5292
Q9C0C9	401	E2/E3 hybrid ubiquitin-protein ligase UBE2O	UBE2O	0.99999	4.89E-14	190.57	S	VKRLLKKQVVRIMSCSPDTQCSRDHSMEDPD	8079200	5226700	5532
Q99590	771	Protein SCAF11	SCAF11	0.999336	2.62E-13	190.55	S	NMPSSDLADEKVETVSQPSESPKDTIDKTKK	689490	1512200	5224
Q99590	776	Protein SCAF11	SCAF11	0.994547	2.62E-13	190.55	S	DLADEKVETVSQPSESPKDTIDKTKKPRTRR	370480	786040	5226
Q15797;O	463;465	Mothers against decapentaplegic homolog 1;Mothers against de	SMAD1;SMAD	0.988044	6.26E-14	190.46	S	DKVLTQMGSPHNPISSVS_____________	4781900	2794200	301
Q15797;O	465;467	Mothers against decapentaplegic homolog 1;Mothers against de	SMAD1;SMAD	0.999996	6.26E-14	190.46	S	VLTQMGSPHNPISSVS_______________	5893200	3340600	302
Q6PJG6	742	BRCA1-associated ATM activator 1	BRAT1	0.999708	6.10E-14	190.44	S	RDKIASYSSLREARGSPNTASAEATLPRWRA	1245900	1162000	3550
P29966	27	Myristoylated alanine-rich C-kinase substrate	MARCKS	0.991156	5.06E-14	190.34	S	GEAAAERPGEAAVASSPSKANGQENGHVKVN	2280000	2578100	1336
Q8IZT6	425	Abnormal spindle-like microcephaly-associated protein	ASPM	0.999999	4.22E-14	190.25	S	TCKVPLSNENSQVPQSPEDWRKSEVSPRIPE	1055600	1121800	4192
Q5T8P6	616	RNA-binding protein 26	RBM26	0.999544	3.21E-14	190.24	S	VYWHREGSTQQLQTTSPKVMQPLVQQPILPV	0	1731600	3295
O14733	55	Dual specificity mitogen-activated protein kinase kinase 7	MAP2K7	0.974898	6.51E-14	190.19	S	RPTLQLPLANDGGSRSPSSESSPQHPTPPAR	1888700	1810200	200
O14733	60	Dual specificity mitogen-activated protein kinase kinase 7	MAP2K7	0.970497	6.51E-14	190.19	S	LPLANDGGSRSPSSESSPQHPTPPARPRHML	2537100	0	203
Q5SYE7	1190	NHS-like protein 1	NHSL1	0.716338	7.54E-14	190.18	S	EAEARPSPSTTPLPDSSPSRKPPPISKKPKL	0	1220600	3237
Q8IX01	757	SURP and G-patch domain-containing protein 2	SUGP2	0.999694	1.74E-18	190.18	S	VGPSPQDPSLEASGPSPKPAGVDISEAPQTS	1913700	676950	4088
P17544	55	Cyclic AMP-dependent transcription factor ATF-7	ATF7	0.501481	1.85E-13	190.18	T	ARTDSVIIADQTPTPTRFLKNCEEVGLFNEL	0	727060	7298
P17812	575	CTP synthase 1	CTPS1	0.994373	6.49E-14	189.99	S	RLSPRDTYSDRSGSSSPDSEITELKFPSINH	5867500	2376200	1123
Q9NQC3	15	Reticulon-4	RTN4	0.994113	1.47E-18	189.98	S	_MEDLDQSPLVSSSDSPPRPQPAFKYQFVRE	7451300	691370	5912
Q13111	775	Chromatin assembly factor 1 subunit A	CHAF1A	0.990348	1.31E-15	189.84	S	HCRRGLLSNHTGSPRSPSTTYLHTPTPSEDA	19457000	12123000	2404
Q13111	777	Chromatin assembly factor 1 subunit A	CHAF1A	0.515891	1.31E-15	189.84	S	RRGLLSNHTGSPRSPSTTYLHTPTPSEDAAI	0	0	2405
Q641Q2;Q	1179;146	WASH complex subunit FAM21A;Putative WASH complex subunit F	FAM21A;FAM	0.999886	1.31E-15	189.84	S	GGKAKSPMFPALGEASSDDDLFQSAKPKPAK	1792800	362970	3392
Q96GP6	687	Scavenger receptor class F member 2	SCARF2	1	6.24E-14	189.84	S	WIHGKHSAAAAGRAPSPPPPGSEAAPSPSKR	1133400	877620	4921
Q96GP6	698	Scavenger receptor class F member 2	SCARF2	0.996762	6.24E-14	189.84	S	GRAPSPPPPGSEAAPSPSKRKRTPSDKSAHT	1133400	877620	4922
Q9H2P0	955	Activity-dependent neuroprotector homeobox protein	ADNP	1	1.32E-14	189.84	S	HLTEEPTKLMHNASDSEVDQDDVVEWKDGAS	5508500	4440100	5620
Q9UQ35	383	Serine/arginine repetitive matrix protein 2	SRRM2	0.904865	3.40E-23	189.84	T	PLLAERHGGSPQPLATTPLSQEPVNPPSEAS	8392200	19617000	8260
P29692	133	Elongation factor 1-delta	EEF1D	0.999987	3.40E-14	189.8	S	SSPGHRATAPQTQHVSPMRQVEPPAKKPATP	20087000	16277000	1324
Q9NRL2	1413	Bromodomain adjacent to zinc finger domain protein 1A	BAZ1A	1	1.62E-13	189.8	S	SLQESESKRRCRKRQSPEPSPVTLGRRSSGR	8186600	4873700	5972
Q9NRL2	1417	Bromodomain adjacent to zinc finger domain protein 1A	BAZ1A	0.99977	1.62E-13	189.8	S	SESKRRCRKRQSPEPSPVTLGRRSSGRQGGV	1287200	671580	5973
Q9H3Z4	8	DnaJ homolog subfamily C member 5	DNAJC5	0.692256	7.75E-14	189.59	S	________MADQRQRSLSTSGESLYHVLGLD	0	0	5649
Q99590	614	Protein SCAF11	SCAF11	0.990975	7.06E-14	189.33	S	TLKTEELIESPKLESSEGEIIQTVDRQSVKS	672990	887360	5220
Q9UI36	491	Dachshund homolog 1	DACH1	1	8.42E-14	189.33	S	PSRVETSVIKERVPDSPSPAPSLEEGRRPGS	1462700	1313500	6373
Q9H0B6	582	Kinesin light chain 2	KLC2	0.990344	1.16E-12	189.17	S	GTPQEPPNPRMKRASSLNFLNKSVEEPTQPG	8760200	6593200	5559
Q9NPI6	522	mRNA-decapping enzyme 1A	DCP1A	0.499979	9.14E-14	188.96	S	FQQTVTRSSDLERKASSPSPLTIGTPESQRK	0	0	5890
Q9NPI6	523	mRNA-decapping enzyme 1A	DCP1A	0.583013	9.14E-14	188.96	S	QQTVTRSSDLERKASSPSPLTIGTPESQRKP	887430	363410	5891
Q9H1E3	58	Nuclear ubiquitous casein and cyclin-dependent kinase subst	NUCKS1	0.999965	0.000104177	188.9	S	EAKNKRRSGKNSQEDSEDSEDKDVKTKKDDS	0	0	5605
Q9H1E3	61	Nuclear ubiquitous casein and cyclin-dependent kinase subst	NUCKS1	1	0.000104177	188.9	S	NKRRSGKNSQEDSEDSEDKDVKTKKDDSHSA	0	0	5606
Q9Y6G9	419	Cytoplasmic dynein 1 light intermediate chain 1	DYNC1LI1	0.994613	8.43E-14	188.9	S	GSPRTPNRSVSSNVASVSPIPAGSKKIDPNM	1179400	1026400	7007
Q12888	1113	Tumor suppressor p53-binding protein 1	TP53BP1	0.681487	1.60E-18	188.86	S	DPVSPASQKMVIQGPSSPQGEAMVTDVLEDQ	1270800	853680	2351
Q96JM3	308	Chromosome alignment-maintaining phosphoprotein 1	CHAMP1	0.999969	2.57E-13	188.63	S	FPAVSPEPRRPAPAVSPGSWKPGPPGSPRPW	26770000	10874000	4984
Q9NUL3	492	Double-stranded RNA-binding protein Staufen homolog 2	STAU2	1	1.33E-13	188.59	S	AEAIGLKGSSPTPPCSPVQPSKQLEYLARIQ	3752400	2081700	6024
Q9NUL3	488	Double-stranded RNA-binding protein Staufen homolog 2	STAU2	0.998907	7.15E-13	188.59	T	TSSTAEAIGLKGSSPTPPCSPVQPSKQLEYL	1500900	1066200	8167
O75533	223	Splicing factor 3B subunit 1	SF3B1	0.999984	2.15E-13	188.54	T	PGATPKKLSSWDQAETPGHTPSLRWDETPGR	2516000	2042000	7212
O75533	227	Splicing factor 3B subunit 1	SF3B1	0.999979	2.15E-13	188.54	T	PKKLSSWDQAETPGHTPSLRWDETPGRAKGS	0	2042000	7213
Q15036	334	Sorting nexin-17	SNX17	0.489004	3.62E-13	188.44	T	RCWRVTSSVPLPSGSTSSPGRGRGEVRLELA	0	0	7632
P27816	571	Microtubule-associated protein 4	MAP4	1	3.25E-13	188.27	T	APLAKDGVLTLANNVTPAKDVPPLSETEATP	3135800	2380300	7334
P85037	441	Forkhead box protein K1	FOXK1	1	6.62E-14	188.13	S	LMSPRSGGLQTPECLSREGSPIPHDPEFGSK	13005000	9395500	2000
Q9P2E9	959	Ribosome-binding protein 1	RRBP1	1	6.62E-14	188.13	S	QEARAENSQLTERIRSIEALLEAGQARDAQD	0	0	6239
Q08AD1	599	Calmodulin-regulated spectrin-associated protein 2	CAMSAP2	0.984746	4.14E-14	188.12	S	QEMSILNSNIKLNQSSPDNVTDTKGALSPIT	1140800	1382300	2256
O43166	1585	Signal-induced proliferation-associated 1-like protein 1	SIPA1L1	0.999851	6.65E-14	188.09	S	FPSTPTSRRALHRTLSDESIYNSQREHFFTS	1146800	707180	345
Q7L2J0	60	7SK snRNA methylphosphate capping enzyme	MEPCE	0.999941	8.14E-14	188.09	S	GGTERGPGRCAPSAGSPAAAVGRESPGAAAT	2925600	2714900	3722
Q5SYE7	1191	NHS-like protein 1	NHSL1	0.931071	9.80E-14	188	S	AEARPSPSTTPLPDSSPSRKPPPISKKPKLF	1274100	0	3238
A6NFI3	10	Zinc finger protein 316	ZNF316	0.99992	4.44E-14	187.78	S	______MAALHTTPDSPAAQLERAEDGSECD	3225900	2144800	37
Q8TAP6	82	Centrosomal protein of 76 kDa	CEP76	0.5	5.69E-14	187.75	S	KELNFVTDSVEQELPSSPKQPICFDRQSTLK	0	648850	4435
Q9BTC0	1040	Death-inducer obliterator 1	DIDO1	1	4.31E-14	187.74	S	LSVPPSPNISTSESRSPPEGDTTLFLSRLST	2293700	1998200	5359
Q9BZL4	560	Protein phosphatase 1 regulatory subunit 12C	PPP1R12C	0.775075	1.06E-12	187.74	T	QRKARSRLMRQSRRSTQGVTLTDLKEAEKAA	962730	377680	8071
Q9Y3L3;Q	550;558	SH3 domain-binding protein 1	SH3BP1	0.999998	8.54E-14	187.63	S	ESEVPPRPASPKVTRSPPETAAPVEDMARRT	6828000	3875100	6859
Q9NYV4	681	Cyclin-dependent kinase 12	CDK12	1	2.08E-19	187.59	S	LTDLPLPPELPGGDLSPPDSPEPKAITPPQQ	15798000	12066000	6123
P37275	645	Zinc finger E-box-binding homeobox 1	ZEB1	0.702476	2.76E-15	187.57	S	EKMQAGQISVQSSEPSSPEPGKVNIPAKNND	1316100	1140100	1438
Q15424	23	Scaffold attachment factor B1	SAFB	0.77589	2.61E-23	187.56	S	LGDSGAAGAAALSSASSETGTRRLSDLRVID	1317700	0	2918
Q9H9J4	856	Ubiquitin carboxyl-terminal hydrolase 42	USP42	1	5.80E-14	187.56	S	EGPRDSALAEAPEGLSPAPPARSEEPCEQPL	3355200	1958500	5802
Q9NYV4	238	Cyclin-dependent kinase 12	CDK12	0.793685	0.00166764	187.56	S	PHRKWSDSSKQDDSPSGASYGQDYDLSPSRS	0	0	6128
P26045	425	Tyrosine-protein phosphatase non-receptor type 3	PTPN3	0.974851	1.07E-13	187.5	S	EDVFYTYKGSLAPQDSDSEVSQNRSPHQESL	1467600	897650	1274
Q9UQ35	780	Serine/arginine repetitive matrix protein 2	SRRM2	0.999998	4.06E-13	187.31	S	PRSKAKSRLSLRRSLSGSSPCPKQKSQTPPR	10105000	8488800	6693
Q9UQ35	783	Serine/arginine repetitive matrix protein 2	SRRM2	0.998868	4.06E-13	187.31	S	KAKSRLSLRRSLSGSSPCPKQKSQTPPRRSR	6817000	7745300	6694
Q7Z4H7	552	HAUS augmin-like complex subunit 6	HAUS6	0.999109	4.61E-14	187.08	S	QDHLVEEVARAVLSDSPQLSEGKEIKLEELI	2405700	1697600	3815
Q9C0C2	836	182 kDa tankyrase-1-binding protein	TNKS1BP1	1	7.37E-14	187.08	S	QDRVVGKPAQLGTQRSQEADVQDWEFRKRDS	3861300	1400800	5524
Q04726	295	Transducin-like enhancer protein 3	TLE3	0.603004	1.80E-14	187.04	S	KKDAPTSPASVASSSSTPSSKTKDLGHNDKS	0	0	2172
O94804	454	Serine/threonine-protein kinase 10	STK10	0.999793	4.64E-14	187	S	PAANRSQKASQSRPNSSALETLGGEKLANGS	1471100	3067100	688
Q99590	774	Protein SCAF11	SCAF11	0.997167	4.65E-13	186.98	S	SSDLADEKVETVSQPSESPKDTIDKTKKPRT	689490	1512200	5225
Q9NY27	159	Serine/threonine-protein phosphatase 4 regulatory subunit 2	PPP4R2	0.998567	1.13E-13	186.96	S	SNSLNRMNGVMFPGNSPSYTERSNINGPGTP	1334300	900120	6087
Q96JC9	165	ELL-associated factor 1	EAF1	1	2.42E-13	186.74	S	KPPVGPKTSPLKDNPSPEPQLDDIKRELRAE	7449100	3864900	4958
Q96JC9	158	ELL-associated factor 1	EAF1	0.836778	2.42E-13	186.74	S	MPFRAPTKPPVGPKTSPLKDNPSPEPQLDDI	5370900	2051200	4960
Q15477	245	Helicase SKI2W	SKIV2L	0.99998	1.11E-13	186.69	S	AVGQPGGPRGDTVSASPCSAPLARASSLEDL	1091400	768400	2941
Q14566	13	DNA replication licensing factor MCM6	MCM6	1	9.65E-15	186.58	S	___MDLAAAAEPGAGSQHLEVRDEVAEKCQK	1089100	2556000	2746
P53621	173	Coatomer subunit alpha;Xenin;Proxenin	COPA	1	7.93E-14	186.29	S	TVRVWDISGLRKKNLSPGAVESDVRGITGVD	2334800	3609100	1835
Q14684	736	Ribosomal RNA processing protein 1 homolog B	RRP1B	0.921138	8.06E-14	186.29	S	PLHGVLKTPTSSPASSPLVAKKPLTTTPRRR	975160	6864500	2787
Q7Z6E9	1179	E3 ubiquitin-protein ligase RBBP6	RBBP6	1	3.57E-13	186.29	S	SMKISKLEVTEIVKPSPKRKMEPDTEKMDRT	1612200	757820	3858
O43318	455	Mitogen-activated protein kinase kinase kinase 7	MAP3K7	0.712119	5.55E-14	186.06	S	IQDLTVTGTEPGQVSSRSSSPSVRMITTSGP	0	1273600	363
Q8WWQ0	1281	PH-interacting protein	PHIP	1	1.22E-14	186.06	S	YNIIPLYNSMKKKVLSDSEDEEKDADVPGTS	8181600	10510000	4572
Q8WWQ0	1283	PH-interacting protein	PHIP	1	1.22E-14	186.06	S	IIPLYNSMKKKVLSDSEDEEKDADVPGTSTR	8181600	10510000	4573
Q9NYF8	287	Bcl-2-associated transcription factor 1	BCLAF1	0.998941	9.90E-14	185.96	S	SGSGSVGNGSSRYSPSQNSPIHHIPSRRSPA	1724100	39278000	6116
Q9Y314	137	Nitric oxide synthase-interacting protein	NOSIP	0.500893	8.08E-15	185.92	T	VSRPLNPFTAKALSGTSPDDVQPGPSVGPPS	0	2241900	8289
Q9ULJ3	1003	Zinc finger and BTB domain-containing protein 21	ZBTB21	0.989997	8.05E-16	185.86	S	PPPPPLPKIQPLEPDSPTGLSENPTPATEKL	1324600	1254900	6478
Q9Y4P1	383	Cysteine protease ATG4B	ATG4B	1	1.01E-13	185.81	S	SLDSSDVERLERFFDSEDEDFEILSL_____	6053300	5599600	6932
O14974	695	Protein phosphatase 1 regulatory subunit 12A	PPP1R12A	0.5	1.21E-13	185.74	S	SQRKARSRQARQSRRSTQGVTLTDLQEAEKT	8582400	2604300	221
O14974	696	Protein phosphatase 1 regulatory subunit 12A	PPP1R12A	0.5	1.21E-13	185.74	T	QRKARSRQARQSRRSTQGVTLTDLQEAEKTI	8582400	2604300	7136
P49790	633	Nuclear pore complex protein Nup153	NUP153	0.787206	6.88E-14	185.71	S	ASPKIDSVAAQPTATSPVVYTRPAISSFSSS	0	402870	1694
P29966	101	Myristoylated alanine-rich C-kinase substrate	MARCKS	1	6.91E-14	185.66	S	EKGEPAAAAAPEAGASPVEKEAPAEGEAAEP	1208800	1563000	1338
P27816	99	Microtubule-associated protein 4	MAP4	0.950018	5.01E-23	185.64	S	LANGGHGVEGSDTTGSPTEFLEEKMAYQEYP	3796300	1771800	1296
Q8IWC1	441	MAP7 domain-containing protein 3	MAP7D3	0.999992	8.43E-14	185.57	S	PKESVKGSPKESMEASPEAMVKASPKTSLEA	726690	628150	4055

O15439	646	Multidrug resistance-associated protein 4	ABCC4	0.945631	4.87E-13	185.32	T	LKKDNEESEQPPVPGTPTLRNRTFSESSVWS	2329500	62790	7159
O14686	4359	Histone-lysine N-methyltransferase 2D	KMT2D	1	1.26E-13	185.28	S	PKPQGPTLEPPPGRVSPAAAQLADTLFSKGL	1777500	1605700	199
P55081	118	Microfibrillar-associated protein 1	MFAP1	1	3.41E-15	185.23	S	RHRKIVEPEVVGESDSEVEGDAWRMEREDSS	20007000	26531000	1858
Q29RF7	1305	Sister chromatid cohesion protein PDS5 homolog A	PDS5A	1	1.21E-13	185.2	S	KPINKGRKRAAVGQESPGGLEAGNAKAPKLQ	8533000	8843400	3036
P17029	10	Zinc finger protein with KRAB and SCAN domains 1	ZKSCAN1	0.727128	2.22E-13	185.2	T	______MMTAESREATGLSPQAAQEKDGIVI	0	0	7293
Q9UQ35	1041	Serine/arginine repetitive matrix protein 2	SRRM2	0.333333	7.21E-14	185.14	S	VQSCPGSLSLCAGVKSSTPPGESYFGVSSLQ	0	0	6740
Q9UQ35	1042	Serine/arginine repetitive matrix protein 2	SRRM2	0.429393	7.21E-14	185.14	S	QSCPGSLSLCAGVKSSTPPGESYFGVSSLQL	0	0	6741
Q9UQ35	1043	Serine/arginine repetitive matrix protein 2	SRRM2	0.674783	7.21E-14	185.14	T	SCPGSLSLCAGVKSSTPPGESYFGVSSLQLK	0	0	8275
Q6BDS2	1103	UHRF1-binding protein 1	UHRF1BP1	0.675373	2.88E-15	185.06	S	GGAARLRFFSMKRTVSQQSFDGVSLDSSGPE	1477000	797980	3443
Q6BDS2	1106	UHRF1-binding protein 1	UHRF1BP1	1	2.88E-15	185.06	S	ARLRFFSMKRTVSQQSFDGVSLDSSGPEDRI	1477000	797980	3444
P46109	43	Crk-like protein	CRKL	0.333333	7.28E-14	185.02	T	LQGQRHGMFLVRDSSTCPGDYVLSVSENSRV	0	0	7385
Q14160	502	Protein scribble homolog	SCRIB	0.558672	3.49E-15	184.97	S	SIEGRRSEACPCQPDSGSPLPAEEEKRLSAE	0	0	2686
Q9BRS2	21	Serine/threonine-protein kinase RIO1	RIOK1	0.998361	1.23E-13	184.97	S	LLMSRVVPGQFDDADSSDSENRDLKTVKEKD	4901300	2784100	5326
Q9P2M7	131	Cingulin	CGN	0.986846	7.32E-14	184.97	S	EEPGAYWNGKLLRSHSQASLAGPGPVDPSNR	785870	539590	6245
Q9NYV4	249	Cyclin-dependent kinase 12	CDK12	0.671784	3.49E-15	184.95	S	DDSPSGASYGQDYDLSPSRSHTSSNYDSYKK	0	597450	6129
P85037	420	Forkhead box protein K1	FOXK1	0.998994	1.08E-13	184.92	S	FRTPFGPLSSRSAPASPTHPGLMSPRSGGLQ	4255600	2213100	1998
P85037	428	Forkhead box protein K1	FOXK1	1	1.08E-13	184.92	S	SSRSAPASPTHPGLMSPRSGGLQTPECLSRE	2773800	2169000	1999
Q12888	1068	Tumor suppressor p53-binding protein 1	TP53BP1	0.928988	1.30E-13	184.92	S	PPTTPIRGNLLHFPSSQGEEEKEKLEGDHTI	2762700	1593400	2343
O60841	186	Eukaryotic translation initiation factor 5B	EIF5B	1	0.000322352	184.91	S	SKKIKERSRINSSGESGDESDEFLQSRKGQK	432510	276380	531
Q14157	477	Ubiquitin-associated protein 2-like	UBAP2L	0.99765	1.74E-18	184.91	S	SAPQMSPGSSDNQSSSPQPAQQKLKQQKKKA	56824000	30634000	2672
Q32MZ4	735	Leucine-rich repeat flightless-interacting protein 1	LRRFIP1	0.776035	1.49E-18	184.91	S	SEDADRCTLPEHESPSQDISDACEAESTERC	7142000	0	3054
Q8TAQ2	347	SWI/SNF complex subunit SMARCC2	SMARCC2	1	7.40E-14	184.83	S	REEEQEDLTKDMDEPSPVPNVEEVTLPKTVN	1803600	729990	4442
Q9UQE7	1081	Structural maintenance of chromosomes protein 3	SMC3	0.498184	3.52E-15	184.83	S	QSQDEGEGSGESERGSGSQSSVPSVDQFTGV	0	0	6758
Q9UQE7	1083	Structural maintenance of chromosomes protein 3	SMC3	0.923404	3.52E-15	184.83	S	QDEGEGSGESERGSGSQSSVPSVDQFTGVGI	1263300	0	6759
O95817	377	BAG family molecular chaperone regulator 3	BAG3	1	3.64E-18	184.68	S	VEVKVPPAPVPCPPPSPGPSAVPSSPKSVAT	20601000	12185000	829
O95817	386	BAG family molecular chaperone regulator 3	BAG3	0.738778	3.64E-18	184.68	S	VPCPPPSPGPSAVPSSPKSVATEERAAPSTA	1545800	1123900	832
Q8WXI9	122	Transcriptional repressor p66-beta	GATAD2B	1	3.41E-15	184.6	S	MSARRSEPERGRLTPSPDIIVLSDNEASSPR	13293000	10390000	4596
Q8WXI9	129	Transcriptional repressor p66-beta	GATAD2B	0.999999	3.41E-15	184.6	S	PERGRLTPSPDIIVLSDNEASSPRSSSRMEE	6714200	4539400	4597
Q8WXI9	120	Transcriptional repressor p66-beta	GATAD2B	1	3.41E-15	184.6	T	VDMSARRSEPERGRLTPSPDIIVLSDNEASS	8262800	6780000	7928
Q96G46	276	tRNA-dihydrouridine(47) synthase [NAD(P)(+)]-like	DUS3L	0.779952	2.22E-18	184.56	S	GAQQVPAGPGTSTPPSSPVRTCGPLTDEDVV	1802000	0	4910
Q96G46	273	tRNA-dihydrouridine(47) synthase [NAD(P)(+)]-like	DUS3L	0.947242	2.22E-18	184.56	T	ENCGAQQVPAGPGTSTPPSSPVRTCGPLTDE	24219000	20078000	7982
Q15366	189	Poly(rC)-binding protein 2	PCBP2	0.995906	1.12E-13	184.51	S	PPKGVTIPYRPKPSSSPVIFAGGQDRYSTGS	46263000	30142000	2909
Q96GP6	651	Scavenger receptor class F member 2	SCARF2	0.997963	9.37E-14	184.48	S	ARPARARGEIGGLSLSPSPERRKPPPPDPAT	3034000	2677000	4923
Q96GP6	653	Scavenger receptor class F member 2	SCARF2	0.999999	9.37E-14	184.48	S	PARARGEIGGLSLSPSPERRKPPPPDPATKP	3121300	2677000	4924
P55196	1083	Afadin	MLLT4	0.995203	3.49E-13	184.44	S	GLSQERAAELMTRTSSVVTLEVAKQGAIYHG	2182800	1674800	1873
Q9UIF9	1783	Bromodomain adjacent to zinc finger domain protein 2A	BAZ2A	0.992837	6.26E-13	184.44	S	RESPAAGPRYSEEGLSPSKRRRLSMRNHHSD	935760	1102200	6383
Q9H7N4	548	Splicing factor, arginine/serine-rich 19	SCAF1	0.981251	4.50E-18	184.34	S	ASSSSGTQPAPPAPSPWDSKKHRSRDRKPG	646700	671690	5764
P12694	347	2-oxoisovalerate dehydrogenase subunit alpha, mitochondrial	BCKDHA	1	6.32E-13	184.31	S	RIGHHSTSDDSSAYRSVDEVNYWDKQDHPIS	2523900	1163700	1044
Q9UQ35	1658	Serine/arginine repetitive matrix protein 2	SRRM2	0.980598	2.75E-14	184.22	S	KGRGPSPEGSSSTESSPEHPPKSRTARRGSR	1124000	1220600	6634
P42566	814	Epidermal growth factor receptor substrate 15	EPS15	1	1.37E-13	184.21	S	PFKLNDPFQPFPGNDSPKEKDPEIFCDPFTS	6423300	5659100	1505
Q5UIP0	1688	Telomere-associated protein RIF1	RIF1	0.999996	4.72E-13	184.21	S	NLRTRNAIKRLHKRDSFDNCSLGESSKIGIS	1131800	0	3315
P50402	8	Emerin	EMD	0.92607	5.56E-14	184.2	S	________MDNYADLSDTELTTLLRRYNIPH	639210	0	1746
O75376	2184	Nuclear receptor corepressor 1	NCOR1	0.999977	1.16E-13	184.11	S	SQRGAEPAEQRNDARSPGSISYLPSFFTKLE	1162000	0	604
Q8IYB3	696	Serine/arginine repetitive matrix protein 1	SRRM1	0.986486	9.47E-14	184.11	S	RHSPSPRPRAPQTSSSPPPVRRGASSSPQRR	32262000	23792000	4135
O00264	181	Membrane-associated progesterone receptor component 1	PGRMC1	0.930149	1.39E-13	184	S	HVGKLLKEGEPTVYSDEEEPKDESARKND_	0	14384000	111
Q14684	732	Ribosomal RNA processing protein 1 homolog B	RRP1B	0.990373	9.72E-14	184	S	PEQKPLHGVLKTPTSSPASSPLVAKKPLTTT	8508100	4962200	2785
Q8WYP5	1541	Protein ELYS	AHCTF1	1	1.17E-13	184	S	EKLEAQDSGEEARNLSFNELYPSGTLKLQYN	1520700	1904800	4606
P46821	936	Microtubule-associated protein 1B;MAP1B heavy chain;MAP1 li	MAP1B	0.843398	0.000113994	183.96	S	DDIEKFEDEGAGFEESETGDYEEKAETEEA	0	0	1583
P29966	147	Myristoylated alanine-rich C-kinase substrate	MARCKS	0.495798	1.41E-13	183.85	S	STSSPKAEDGATPSPSNETPKKKKKRFSFKK	0	0	1327
O15075	330	Serine/threonine-protein kinase DCLK1	DCLK1	0.999356	1.44E-12	183.76	S	NGTPGSQLSTPRSGKSPSPSPTSPGSLRKQR	0	194000	278
O15075	332	Serine/threonine-protein kinase DCLK1	DCLK1	0.992079	1.44E-12	183.76	S	TPGSQLSTPRSGKSPSPSPTSPGSLRKQRSS	0	194000	279
O15075	334	Serine/threonine-protein kinase DCLK1	DCLK1	0.875694	1.44E-12	183.76	S	GSQLSTPRSGKSPSPSPTSPGSLRKQRSSQH	0	194000	280
P55196	1512	Afadin	MLLT4	0.992282	1.42E-13	183.75	S	YITVSKEELSSGDSLSPDPWKRDAKEKLEKQ	1123900	911930	1865
Q8TBB5	413	Kelch domain-containing protein 4	KLHDC4	1	3.83E-15	183.73	S	IKQVLTAPGSAGQPRSEDEDSLEEAGSPAPG	895250	666250	4450
Q9Y388	148	RNA-binding motif protein, X-linked 2	RBMX2	0.999899	5.20E-13	183.73	S	EKGCGARTPSPSLSESSEDEKPTKKHKKDKK	940850	342950	6848
Q9Y388	149	RNA-binding motif protein, X-linked 2	RBMX2	0.999962	5.20E-13	183.73	S	KGCGARTPSPSLSESSEDEKPTKKHKKDKKE	940850	342950	6849
Q09666	210	Neuroblast differentiation-associated protein AHNAK	AHNAK	0.999868	6.70E-14	183.71	S	VDVETQSGKTVIRLPSGSGAASPTGSAVDIR	0	215710	2278
Q9P258	50	Protein RCC2	RCC2	0.715104	5.95E-19	183.46	S	KRERPERCSSSSGGGSSGDEDGLELDGAPGG	680240	384100	6216
P46379	1117	Large proline-rich protein BAG6	BAG6	0.999998	3.80E-13	183.28	S	LRSDIQRKLQEDPNYSPQRFPNAQRAFADDP	4808700	5316600	1566
Q9NYF8	208	Bcl-2-associated transcription factor 1	BCLAF1	0.397984	3.08E-14	183.25	T	HDPSESIDEFNKSSATSGDIWPGLSAYDNSP	0	0	8176
Q00839	66	Heterogeneous nuclear ribonucleoprotein U	HNRNPU	1	2.96E-14	183.21	S	PAMEPGNGSLDLGGDSAGRSGAGLEQEAAAG	1368400	0	2038
P49815	1385	Tuberin	TSC2	0.597702	3.98E-15	183.18	S	PSVDLSFQPSQPLSKSSSSPELQTLQDILGD	266960	183260	1728
P49815	1386	Tuberin	TSC2	0.499977	3.98E-15	183.18	S	SVDLSFQPSQPLSKSSSSPELQTLQDILGDP	266960	183260	1729
P49815	1387	Tuberin	TSC2	0.499977	3.98E-15	183.18	S	VDLSFQPSQPLSKSSSSPELQTLQDILGDPG	266960	183260	1730
P46776	68	60S ribosomal protein L27a	RPL27A	1	1.02E-13	183.06	S	FGKVGMKHYHLKRNQSFCPTVNLDKLWTLVS	6027200	1919200	1568
Q9NZI8	181	Insulin-like growth factor 2 mRNA-binding protein 1	IGF2BP1	1	1.02E-13	183.06	S	GRRGGFGSRGQPRQGSPVAAGAPAKQQQVDI	51096000	33700000	6151
Q9UNF1	265	Melanoma-associated antigen D2	MAGED2	0.999415	6.39E-14	183.06	S	ARRGKARRRAAKLQSSQEPEAPPPRDVALLQ	1056500	1949000	6547
Q9H0D6	499	5'-3' exoribonuclease 2	XRN2	1	0.00183477	183.01	S	GSPSPLGGIKRKAEDSDSEPEPEDNVRLWEA	0	0	5567
Q9H0D6	501	5'-3' exoribonuclease 2	XRN2	1	0.00183477	183.01	S	PSPLGGIKRKAEDSDSEPEPEDNVRLWEAGW	0	0	5568
Q7Z422	105	SUZ domain-containing protein 1	SZRD1	0.711486	3.84E-13	182.91	S	QREAEYAEARKRILGSAPSEEEQEKPILDRP	0	0	3799
Q96ST2	196	Protein IWS1 homolog	IWS1	1	5.80E-13	182.91	S	QISDSESEEPPRHQASDSENEEPPKPRMSDS	2422600	2569000	5131
Q96ST2	198	Protein IWS1 homolog	IWS1	1	5.80E-13	182.91	S	SDSESEEPPRHQASDSENEEPPKPRMSDSES	2422600	2569000	5132
O95684	156	FGFR1 oncogene partner	FGFR1OP	0.999994	1.35E-17	182.77	S	GPTTGEGALDLSDVHSPPKSPEGKTSAQTTP	4892200	3459200	795
O95684	160	FGFR1 oncogene partner	FGFR1OP	1	1.35E-17	182.77	S	GEGALDLSDVHSPPKSPEGKTSAQTTPSKIP	4892200	3459200	796
Q9BXB4	179	Oxysterol-binding protein-related protein 11	OSBPL11	0.807239	1.53E-13	182.7	S	NNPPLKSRSFSLASSSNSPISQRRPSQNAIS	0	5179200	5447
Q9Y4H2	391	Insulin receptor substrate 2	IRS2	1	1.53E-13	182.7	S	AAAGARPVSVAGSPLSPGPVRAPLSRSHTLS	2360400	1391000	6926
O75044	990	SLIT-ROBO Rho GTPase-activating protein 2	SRGAP2	0.886837	2.14E-15	182.47	S	DTLEPLKTSPVVAPTSEPSSPLHTQLLKDPE	754610	588730	549
O75044	993	SLIT-ROBO Rho GTPase-activating protein 2	SRGAP2	0.500386	2.14E-15	182.47	S	EPLKTSPVVAPTSEPSSPLHTQLLKDPEPAF	754610	588730	550
O75044	994	SLIT-ROBO Rho GTPase-activating protein 2	SRGAP2	0.500386	2.14E-15	182.47	S	PLKTSPVVAPTSEPSSPLHTQLLKDPEPAFQ	754610	588730	551
Q9H307	66	Pinin	PNN	0.999984	1.07E-13	182.41	S	GGGRGRGSLLLRRGFSDSGGGPPAKQRDLEG	4981400	4184700	5632
Q6SPF0	161	Atherin	SAMD1	1	4.21E-15	182.37	S	ARAPRAAAAAATAPPSPGPAQPGPRAQRAAP	0	2443800	3580
Q86UP2	75	Kinectin	KTN1	0.999982	1.49E-13	182.28	S	KKNKKKEIQNGNLHESDSESVPRDFKLSDAL	3730900	1889300	3932
P30041	44	Peroxiredoxin-6	PRDX6	1	4.44E-13	182.1	T	LGDSWGILFSHPRDFTPVCTTELGRAAKLAP	2777900	1040500	7347
P48382	185	DNA-binding protein RFX5	RFX5	0.934944	1.66E-13	182.02	S	VSMPPLPGLDLKGSESPEMGPEVTPAPRDEL	1063200	926500	1622
Q9HCD6	168	Protein TANC2	TANC2	0.923572	6.22E-13	181.97	T	ISTNATAKDCSYGAVTSPTSTLESRDSGIIA	1187400	0	8132
O43719	481	HIV Tat-specific factor 1	HTATSF1	0.999553	3.52E-13	181.86	S	ESEEGCPKRGFEGSCSQKESEEGNPVRGSEE	2020700	2901000	407
O43719	485	HIV Tat-specific factor 1	HTATSF1	1	3.52E-13	181.86	S	GCPKRGFEGSCSQKESEEGNPVRGSEEDSPK	409300	896910	408
Q5VT52	723	Regulation of nuclear pre-mRNA domain-containing protein 2	RPRD2	1	6.84E-13	181.86	T	FQRGPTSTSIDNIDGTPVRDERSGTPTQDEM	1099400	985590	7730
Q96NE9	522	FERM domain-containing protein 6	FRMD6	0.999947	5.77E-14	181.81	S	STSSSSETVVKLRGQSTDSLPQTICRKPKTS	4159900	2486900	5042
Q96NE9	525	FERM domain-containing protein 6	FRMD6	0.999431	5.77E-14	181.81	S	SSSETVVKLRGQSTDSLPQTICRKPKTSTDR	2580300	1735600	5043
Q9UQ35	1501	Serine/arginine repetitive matrix protein 2	SRRM2	0.995537	4.29E-13	181.75	S	PKALPQTPRPRSRSPSSPELNNKCLTPQRER	1928500	2390500	6700
Q9UQ35	1497	Serine/arginine repetitive matrix protein 2	SRRM2	0.998918	4.29E-13	181.75	S	SSPEPKALPQTPRPRSRSPSSPELNNKCLTP	10482000	1948000	6711
P24928	1910	DNA-directed RNA polymerase II subunit RPB1	POLR2A	0.978311	1.14E-13	181.69	S	YSPTSPYYTPTSPKYSPTSPTYSPTSPKYSP	1145200	0	1242
P24928	1920	DNA-directed RNA polymerase II subunit RPB1	POLR2A	0.883549	1.14E-13	181.69	S	TSPKYSPTSPTYSPTSPKYSPTSPTYSPTSP	9118500	7470900	1245
Q9H3U1	15	Protein unc-45 homolog A	UNC45A	1	2.86E-13	181.67	T	_MTVSGPGTPEPRPATPGASSVEQLRKEGNE	4535400	2767200	8099
P22626	344	Heterogeneous nuclear ribonucleoproteins A2/B1	HNRNPA2B1	0.999987	2.19E-18	181.65	S	GPYGGGNYGPGSGGSGGYGGRSRY______	4364600	2258900	1214
Q66K74	763	Microtubule-associated protein 1S;MAP1S heavy chain;MAP1S li	MAP1S	0.440173	4.41E-15	181.64	S	RKAVPMAPAPASPGSSNDSSARSQERAGGLG	0	0	3405
Q8IXQ4	140	GPALPP motifs-containing protein 1	GPALPP1	0.930557	5.42E-18	181.64	S	KSDKGRDDPGQQETDSSEDEDIIGPMPAKGP	2450000	449120	4109
Q9NQW6	54	Actin-binding protein anillin	ANLN	1	9.29E-14	181.6	S	RARQPLSEASNQQPLSGGEEKSCTKPSPSKK	1230400	1193800	5929
Q9UQ35	1398	Serine/arginine repetitive matrix protein 2	SRRM2	0.999471	2.74E-14	181.6	S	SSELSPDAVEKAGMSSNQSISSPVLDAVPRT	1517000	1752900	6597
Q9UQ35	1403	Serine/arginine repetitive matrix protein 2	SRRM2	0.927666	2.74E-14	181.6	S	PDAVEKAGMSSNQSISSPVLDAVPRTPSRER	1414400	1490800	6599
Q14839	1535	Chromodomain-helicase-DNA-binding protein 4	CHD4	0.999986	7.72E-13	181.41	S	ELAEVEENKKMSQPGSPSPKTPTPSTPGDTQ	25048000	16970000	2816
P49792	21	E3 SUMO-protein ligase RanBP2	RANBP2	1	7.13E-14	181.4	S	ADVERYIASVQGSTPSPRQKSMKGFYFAKLY	4000100	2030300	1720
Q9ULM3	519	YEATS domain-containing protein 2	YEATS2	0.995652	3.35E-14	181.34	S	KQPGQVIGATTPSTGSPTNKISTASQVSQGT	6706400	3399800	6499
Q9NV70	501	Exocyst complex component 1	EXOC1	0.997476	1.13E-15	181.13	S	RSQSSSLLDMGNMSASDLDVADRTKFDKIFE	858070	847760	6032
P25054	2674	Adenomatous polyposis coli protein	APC	0.992987	1.78E-13	180.93	S	VRIEDCPINNPRSGRSPTGNTPPVIDSVSEK	2959700	1827400	1262
Q92729	853	Receptor-type tyrosine-protein phosphatase U	PTPRU	1	1.78E-13	180.93	S	QRSGGVTEASSLLGGSPRRPCGRKGSPYHTG	1502800	1191300	4691
Q09666	5749	Neuroblast differentiation-associated protein AHNAK	AHNAK	1	8.13E-14	180.76	S	ASISGSKGDLKSSKASLGSLEGEAEAEASSP	772880	1665600	2273
Q09666	5752	Neuroblast differentiation-associated protein AHNAK	AHNAK	1	8.13E-14	180.76	S	SGSKGDLKSSKASLGSLEGEAEAEASSPKGK	772880	1665600	2274
P61006	185	Ras-related protein Rab-8A	RAB8A	0.99999	1.46E-13	180.7	S	KAKMDKKLEGNSPQGSNQGVKITPDQQKRSS	2086000	1104900	1907
Q9H1E3	214	Nuclear ubiquitous casein and cyclin-dependent kinase substr	NUCKS1	1	1.19E-13	180.67	S	KEKTPSPKEEDEEPESPPEKKTSTSPPPEKS	1004500	1840400	5603
Q9BXP5	544	Serrate RNA effector molecule homolog	SRRT	0.999983	9.50E-18	180.64	T	HTLDDRTQLWASEPGTPPLPTSLPSQNPILK	21588000	17351000	8065
Q13523	578	Serine/threonine-protein kinase PRP4 homolog	PRPF4B	1	4.61E-13	180.56	S	VPSEPSSPQSSTRTRSPSPDDILERVAADVK	3474700	3171400	2560
Q13523	580	Serine/threonine-protein kinase PRP4 homolog	PRPF4B	1	4.61E-13	180.56	S	SEPSSPQSSTRTRSPSPDDILERVAADVKEY	3474700	3171400	2561
O95817	385	BAG family molecular chaperone regulator 3	BAG3	0.573128	4.85E-18	180.45	S	PVPCPPPSPGPSAVPSSPKSVATEERAAPST	0	0	831
P42684	631	Abelson tyrosine-protein kinase 2	ABL2	0.999896	1.56E-10	180.3	S	ASSGPSALPRKQRDKSPSSLLEDAKETCFTR	2770100	2133300	1509
Q3KQU3	460	MAP7 domain-containing protein 1	MAP7D1	0.999959	3.46E-11	180.3	S	ASPRARLSASTASELSPKSARPSSPSTSWH	7187900	6378600	3063
Q5JRA6	1741	Melanoma inhibitory activity protein 3	MIA3	0.61357	4.42E-11	180.08	S	PSDPGSGTATMMNSSSRGSSPTRVLDEGKVN	5809900	0	3178
P35580	1956	Myosin-10	MYH10	0.999956	5.02E-11	180.03	S	SGRRQLHLEGASLELSDDDTESKTSDVNETQ	5435900	3532400	1396
Q9NYF8	209	Bcl-2-associated transcription factor 1	BCLAF1	0.999854	8.54E-12	180.03	S	DPSESIDEFNKSSATSGDIWPGLSAYDNSPR	0	1021000	6113
P30086	52	Phosphatidylethanolamine-binding protein 1;Hippocampal choli	PEBP1	0.82313	7.53E-10	180.02	S	LGKVLTPTQVKNRPTSISWDGLDSGKLYTLV	2253400	2240000	1341
Q86WR0	204	Coiled-coil domain-containing protein 25	CCDC25	1	0.000962288	180.02	S	LMKVENMSSNQDGNDSDEFM___________	0	0	3992
Q9NQW6	792	Actin-binding protein anillin	ANLN	1	3.66E-10	179.97	S	LNKLKNEGPQRKNKASPQSEFMPSKGSVTLS	610850	1232000	5928
Q9Y520	1246	Protein PRRC2C	PRRC2C	0.869577	4.83E-10	179.94	S	IPSGPLRQREESETRSESSDFEVVPKRRRQR	0	0	6946
Q09666	5739	Neuroblast differentiation-associated protein AHNAK	AHNAK	0.999241	2.92E-10	179.84	S	GKGGVTGSPEASISGSKGDLKSSKASLGSLE	2012100	1520100	2277
Q86VQ1	105	Glucocorticoid-induced transcript 1 protein	GLCCI1	0.978189	8.01E-12	179.84	S	ASLGSLPGPGAARGPSPSSPTPPAAAAPAEQ	1130000	536070	3960
Q86VQ1	108	Glucocorticoid-induced transcript 1 protein	GLCCI1	0.920292	8.01E-12	179.84	S	GSLPGPGAARGPSPSSPTPPAAAAPAEQAPR	3715600	1856000	3962
Q8NE71	22	ATP-binding cassette sub-family F member 1	ABCF1	0.975775	1.57E-11	179.84	S	QQPPEPEWIGDGESTSPSDKVVKKGKKDKKI	11361000	6206700	4368
Q92625	647	Ankyrin repeat and SAM domain-containing protein 1A	ANKS1A	0.999195	5.85E-10	179.83	S	TCSPTEDATMGSRSESLSNCSIGKKRLEKSP	0	2520100	4683
Q9UQ35	1383	Serine/arginine repetitive matrix protein 2	SRRM2	0.646641	2.80E-10	179.81	S	SLDMKEQSTRSSGHSSSELSPDAVEKAGMSS	768310	0	6736
P78317	94	E3 ubiquitin-protein ligase RNF4	RNF4	0.999945	1.42E-11	179.78	S	NARRLPQDHADSCVVSSDDEELSRDRDVYVT	3456100	1645700	1954
P78317	95	E3 ubiquitin-protein ligase RNF4	RNF4	0.999986	1.42E-11	179.78	S	ARRLPQDHADSCVVSSDDEELSRDRDVYVTT	1256600	859160	1955
Q04726	296	Transducin-like enhancer protein 3	TLE3	0.974981	6.47E-10	179.78	T	KDAPTSPASVASSSSTPSSKTKDLGHNDKSS	1356700	0	7494
O00139	586	Kinesin-like protein KIF2A	KIF2A	0.991153	7.02E-15	179.76	S	PNQIDDLETQWGVGSSPQRDDLKLLCEQNEE	3800000	2555100	92
Q96PM9	153	Zinc finger protein 385A	ZNF385A	1	4.17E-10	179.69	S	PRPVSMENGLGPAPGSPEKQPGSPSPPSIPE	859810	733410	5055
Q9UQ35	871	Serine/arginine repetitive matrix protein 2	SRRM2	1	2.26E-10	179.67	S	TSPQANEQSVTPQRRSCFESSPDPELKSRTP	6119000	3783100	6681
Q96BY7	1576	Autophagy-related protein 2 homolog B	ATG2B	0.988652	3.08E-10	179.55	S	GGKDFGIVPPTSPAKSYISPHSSPSHTPTRH	2185700	749420	4811
Q96BY7	1583	Autophagy-related protein 2 homolog B	ATG2B	0.797372	3.08E-10	179.55	S	VPPTSPAKSYISPHSSPSHTPTRHGRNTVCG	2185700	0	4814
Q9NYF8	177	Bcl-2-associated transcription factor 1	BCLAF1	1	6.52E-10	179.55	S	QEKQTKKAEGEPQEESPLKSKSQEEPKDTFE	688620	745900	6100

Q9UQ35	1382	Serine/arginine repetitive matrix protein 2	SRRM2	0.450698	4.78E-10	179.55	S	PSLDMKEQSTRSSGHSSSELSPDAVEKAGMS	0	0	6735
Q6KC79	2658	Nipped-B-like protein	NIPBL	1	2.89E-10	179.46	S	TNARNKAITSLLGGGSPKNNTAAETEDDESD	9340200	6607500	3485
P04183	231	Thymidine kinase, cytosolic	TK1	1	3.71E-10	179.19	S	VAARKLFAPQQILQCSPAN____________	2759400	4296100	879
Q99459	385	Cell division cycle 5-like protein	CDC5L	1	4.03E-10	179.17	T	MALTNVDTPLKGGLNTPLHESDFSGVTPQRQ	1030500	1529600	8019
Q99459	396	Cell division cycle 5-like protein	CDC5L	1	4.03E-10	179.17	T	GGLNTPLHESDFSGVTPQRQVVQTPNTVLST	1568000	2142500	8020
Q9NR30	71	Nucleolar RNA helicase 2	DDX21	1	9.64E-10	179.14	S	KQVKKKAEPSEVDMNSPKSKKAKKKEEPSQN	3392700	3565800	5944
Q5SRE5	1717	Nucleoporin NUP188 homolog	NUP188	1	6.27E-10	179.1	S	FRRGAPSSPATGVLPSPQGKSTSLSKASPES	3227700	2393400	3212
P16989	370	Y-box-binding protein 3	YBX3	0.999061	1.74E-10	179.09	S	GEAPTENPAPPTQQSSAE_____________	0	633810	1099
Q9Y2J2	88	Band 4.1-like protein 3;Band 4.1-like protein 3, N-terminal	EPB41L3	1	2.77E-09	178.99	S	AAKQLEYQQLEDDKLSQKSSSSKLSRSPLKI	2931700	2882700	6782
Q9UQR1	784	Zinc finger protein 148	ZNF148	0.965396	8.10E-10	178.95	S	FPLVNVNDNRAGMTSSPDATTGQTFG_____	2940400	2406500	6765
P08708	115	40S ribosomal protein S17	RPS17	0.567582	5.74E-12	178.71	S	PDTKEMLKLLDFGSLSNLQVTQPTVGMNFKT	9243400	6105200	967
Q07157	297	Tight junction protein ZO-1	TJP1	0.999458	7.88E-10	178.71	S	NASERDDISEIQSLASDHSGRSHDRPPRRSR	2155400	1405400	2221
Q07157	300	Tight junction protein ZO-1	TJP1	0.999999	7.88E-10	178.71	S	ERDDISEIQSLASDHSGRSHDRPPRRSRSRS	2155400	2114500	2222
Q9H2J7	687	Sodium-dependent neutral amino acid transporter B(0)AT2	SLC6A15	1	5.25E-10	178.68	S	DDTSLIHGKIPSEMPSPNFGKNIYRKQSGSP	10418000	8842900	5617
Q96G74	508	OTU domain-containing protein 5	OTUD5	0.5	3.07E-09	178.67	S	PGTSSQFSAGADRATSPLVSLYPALECRALI	921810	0	4913
Q96G74	507	OTU domain-containing protein 5	OTUD5	0.808927	3.07E-09	178.67	T	APGTSSQFSAGADRATSPLVSLYPALECRAL	921810	838070	7983
Q69YQ0	220	Cytospin-A	SPECC1L	1	7.92E-10	178.57	S	ELRDMRAQLGINEDHSEGDEKSEKETIMAHQ	0	807530	3438
P98175	733	RNA-binding protein 10	RBM10	0.999779	5.35E-10	178.56	S	SDDRPSPPRGLVAAYSGESDSEEEQERGGPE	2505200	2028400	2014
Q9NQC3	181	Reticulon-4	RTN4	0.763153	4.76E-13	178.55	S	PAAPPSTPAAPKRRGSSGSVDETLFALPAAS	872200	664400	5905
Q9NQC3	184	Reticulon-4	RTN4	0.99909	4.76E-13	178.55	S	PPSTPAAPKRRGSSGSVDETLFALPAASEPV	872200	664400	5907
Q9NS91	471	E3 ubiquitin-protein ligase RAD18	RAD18	0.999999	1.89E-09	178.54	S	AWEASHKNDLQDTEISPRQNRRTRAAESAEI	2040200	8290500	5997
P28715	384	DNA repair protein complementing XP-G cells	ERCC5	0.999963	5.91E-10	178.47	S	ARGRNAPAAVDEGSISPRTLSAIKRALDDDE	6799600	5528600	1312
Q9NYF8	290	Bcl-2-associated transcription factor 1	BCLAF1	0.999764	6.94E-10	178.47	S	GSVGNGSSRYSPSQNSPIHHIPSRRSPAKTI	306620000	202040000	6117
Q9H1E3	130	Nuclear ubiquitous casein and cyclin-dependent kinase subst	NUCKS1	0.999099	0.00178587	178.23	S	EEQEEEDEAPFQEKDSGSDEDFLMEDDDDSD	0	0	5601
Q9H1E3	132	Nuclear ubiquitous casein and cyclin-dependent kinase subst	NUCKS1	0.999527	0.00178587	178.23	S	QEEEDEAPFQEKDSGSDEDFLMEDDDDSDYG	0	0	5602
P24928	1878	DNA-directed RNA polymerase II subunit RPB1	POLR2A	0.967177	5.56E-10	178.22	S	TSPKYSPTSPKYSPTSPTYSPTTPKYSPTSP	1522800	1610300	1251
P24928	1882	DNA-directed RNA polymerase II subunit RPB1	POLR2A	0.986429	5.56E-10	178.22	S	YSPTSPKYSPTSPTYSPTTPKYSPTSPTYSP	2749100	1610300	1252
Q7Z417	652	Nuclear fragile X mental retardation-interacting protein 2	NUFIP2	1	2.56E-09	178.22	S	SAKEQRYQRGLERNDSWGSFDLRAAIVYHTK	6898500	7829300	3796
Q8N1G0	253	Zinc finger protein 687	ZNF687	0.999954	5.97E-11	178.22	S	GSGSSPKATDIPASASPPPVAGVPFFKQSPG	449770	388730	4221
O60825	466	6-phosphofructo-2-kinase/fructose-2,6-bisphosphatase 2;6-ph	PFKFB2	0.999858	4.19E-10	178.14	S	NNFPKNQTPVRMRRNSFTPLSSSNTIRRPRN	2661800	1330900	523
Q9NQC3	182	Reticulon-4	RTN4	0.706171	5.87E-13	178.11	S	AAPPSTPAAPKRRGSSGSVDETLFALPAASE	1157100	0	5906
P20042	105	Eukaryotic translation initiation factor 2 subunit 2	EIF2S2	1	1.59E-11	178.07	S	DIDEAEEGVKDLKIESDVQEPTEPEDDLDIM	842810	894230	1179
P55081	116	Microfibrillar-associated protein 1	MFAP1	1	5.29E-11	178.07	S	LARHRKIVEPEVVGESDSEVEGDAWRMERED	16982000	29442000	1857
Q9UQR0	499	Sex comb on midleg-like protein 2	SCML2	0.999996	1.03E-09	178.02	S	QSAKEDVTERQSTKRSPQQTVPYVVPLSPKL	2793600	1220400	6761
Q9BRJ6	175	Uncharacterized protein C7orf50	C7orf50	1	7.57E-10	177.93	S	VQKAEALMRELDEEGSDPPLPGRAQRIRQVL	3597600	2989400	5322
Q9Y2W2	237	WW domain-binding protein 11	WBP11	0.999999	4.58E-10	177.93	S	ALDLPPRRRDEDMLYSPELAQRGHDDDVSST	1324600	745520	6833
Q5T200	372	Zinc finger CCCH domain-containing protein 13	ZC3H13	0.999696	9.85E-10	177.66	S	PSYQRTLTPPLRRSASPYPSHSLSSPQRKQS	6536300	1705600	3266
Q5T200	380	Zinc finger CCCH domain-containing protein 13	ZC3H13	0.980832	9.85E-10	177.66	S	PPLRRSASPYPSHSLSSPQRKQSPPRHRSPM	3589000	1705600	3267
Q8NDV7	991	Trinucleotide repeat-containing gene 6A protein	TNRC6A	0.999938	1.53E-09	177.66	S	PAPAKEEEPTGWEEPSPESIRRKMEIDDGTS	1730100	1118500	4360
P78347	146	General transcription factor II-I	GTF2I	1	1.62E-09	177.6	S	STVVPVPYEKMLRDQSAVVVQGLPEGVAFKH	898430	0	1960
P16949	38	Stathmin	STMN1	1	9.07E-10	177.42	S	ILSPRSKESVPEFPLSPPKKKDLSLEEIQKK	12549000	16131000	1096
Q14676	329	Mediator of DNA damage checkpoint protein 1	MDC1	1	1.34E-08	177.41	S	AEVHLERAQPFGFIDSDTDAEEERIPATPVV	5396200	3137300	2762
Q14676	331	Mediator of DNA damage checkpoint protein 1	MDC1	1	1.34E-08	177.41	T	VHLERAQPFGFIDSDTDAEEERIPATPVVIP	2044200	1257100	7601
O60244	617	Mediator of RNA polymerase II transcription subunit 14	MED14	1	3.76E-09	177.39	S	TKTGKQTRTNAKRKLSDDPCPVESKKTKRAG	0	36469	441
Q05209	673	Tyrosine-protein phosphatase non-receptor type 12	PTPN12	0.999999	1.30E-09	177.39	S	TTHSGAEKDVDVSEDSPPPLPERTPESFVLA	1462200	825300	2184
Q04724	286	Transducin-like enhancer protein 1	TLE1	0.97529	4.01E-10	177.38	S	NGIDKNRLLKKDASSSPASTASSASSTSLKS	2859000	0	2170
O95251	57	Histone acetyltransferase KAT7	KAT7	0.969705	9.26E-10	177.36	S	VTRSSARLSQSSQDSSPVRNLQSFGTEEPAY	0	1345600	742
Q99590	796	Protein SCAF11	SCAF11	1	8.07E-10	177.21	S	IDKTKKPRTRRSRFHSPSTTWSPNKDTPQEK	8427600	7506300	5217
Q99590	802	Protein SCAF11	SCAF11	0.999935	8.07E-10	177.21	S	PRTRRSRFHSPSTTWSPNKDTPQEKKRPQSP	7490400	7506300	5219
Q6AI08	642	HEAT repeat-containing protein 6	HEATR6	0.940936	1.81E-09	177.16	S	WKKAPAGPSLEETSVSSPKGSSEPCWLIRLC	0	0	3439
Q12888	1114	Tumor suppressor p53-binding protein 1	TP53BP1	0.995788	9.60E-14	177.09	S	PVSPASQKMVIQGPSSPQGEAMVTDVLEDQK	1734800	1211800	2352
Q14839	1544	Chromodomain-helicase-DNA-binding protein 4	CHD4	0.874336	1.09E-14	177.09	S	KMSQPGSPSPKTPTPSTPGDTQPNTPAPVPP	2179100	1039900	2819
Q14839	1545	Chromodomain-helicase-DNA-binding protein 4	CHD4	0.874336	1.09E-14	177.09	T	MSQPGSPSPKTPTPSTPGDTQPNTPAPVPPA	813230	0	7622
O95429	179	BAG family molecular chaperone regulator 4	BAG4	0.995124	3.45E-09	176.99	S	SYSTEVPSTYRSSGNSPTPVSRWIYPQQDCQ	1371000	1241800	769
Q8WWI1	1424	LIM domain only protein 7	LMO7	0.688721	4.22E-09	176.99	T	ASSGFLPGDRNKSRSTTELDDYSTNKNGNNK	563030	309960	7922
P49840;P	279;216	Glycogen synthase kinase-3 alpha;Glycogen synthase kinase-3	GSK3A;GSK3	0.703877	6.65E-09	176.95	Y	FGSAKQLVRGEPNVSYICSRYYRAPELIFGA	0	0	8366
Q9UKS6	319	Protein kinase C and casein kinase substrate in neurons pro	PACSIN3	1	1.07E-09	176.89	S	LDTQRTISRKEKGGRSPDEVTLTSIVPTRDG	961660	640920	6451
Q86X02	303	Cerebellar degeneration-related protein 2-like	CDR2L	0.835251	1.99E-11	176.88	S	PQPGRGDDLGAQDGVSSPAASPGHVVRKSCS	567240	520540	3995
Q86X02	308	Cerebellar degeneration-related protein 2-like	CDR2L	0.999999	1.99E-11	176.88	S	GDDLGAQDGVSSPAASPGHVVRKSCSDTALN	3571800	3107700	3997
Q4G0J3	298	La-related protein 7	LARP7	0.463612	1.07E-09	176.87	S	EASSLPEVRTGKRKRSSSEDAESLAPRSKVK	0	0	3093
Q4G0J3	299	La-related protein 7	LARP7	0.634604	1.07E-09	176.87	S	ASSLPEVRTGKRKRSSSEDAESLAPRSKVKK	0	0	3094
P49915	330	GMP synthase [glutamine-hydrolyzing]	GMPS	0.633851	1.50E-08	176.78	T	EDRTPRKRISKTLNMTTSPEEKRKIIGDTFV	0	269740	7428
P49915	331	GMP synthase [glutamine-hydrolyzing]	GMPS	0.956849	1.50E-08	176.78	T	DRTPRKRISKTLNMTTSPEEKRKIIGDTFVK	0	269740	7429
O60271	217	C-Jun-amino-terminal kinase-interacting protein 4	SPAG9	1	1.21E-13	176.61	T	ISLGIFPLPAGDGLLTPDAQKGGETPGSEQW	7826400	6301400	7178
Q6Y7W6	388	PERQ amino acid-rich with GYF domain-containing protein 2	GIGYF2	0.931049	1.76E-08	176.46	S	TSSSSARPGTPSDHQSQEASQFERKDEPKTE	239410	0	3644
Q9Y3X0	385	Coiled-coil domain-containing protein 9	CCDC9	0.96605	5.14E-10	176.46	T	TKEGAASPAPETPQPTSPETSPKETPMQPPE	4942300	3365700	8295
P07197	837	Neurofilament medium polypeptide	NEFM	1	1.71E-09	176.44	S	GREEEKGVVTNGLDLSPADEKKGGDKSEEKV	6507700	2760800	931
P19634	785	Sodium/hydrogen exchanger 1	SLC9A1	0.995972	2.13E-13	176.43	S	SSPGTDDVFTPAPSDSPSSQRIQRCLSDPGP	1191500	942850	1172
Q96N67	1438	Dedicator of cytokinesis protein 7	DOCK7	0.999999	1.22E-09	176.42	S	IASPPERSPSGSAFGSQENLRWRKDMTHWRQ	2878200	1830800	5040
Q9HAW4	225	Claspin	CLSPN	1	8.62E-14	176.42	S	VDKDLFETGLEDENNSPLEDEESLESIRAAV	783300	791590	5823
Q9BXP5	74	Serrate RNA effector molecule homolog	SRRT	1	4.74E-08	176.33	S	DRNRRERFSPPRHELSPPQKRMRRDWDEHSS	6511000	4810500	5458
Q9Y4F5	853	Centrosomal protein of 170 kDa protein B	CEP170B	1	7.54E-10	176.32	S	VSANGRMVIQLRPGRSPEPDGPAPAFLRQES	2891000	1738900	6919
Q9NRF8	568	CTP synthase 2	CTPS2	0.999646	0.000427594	176.27	S	AYLQQGCKLSSSDRYSDASDDSFSEPRIAEL	0	0	5960
Q9NRF8	571	CTP synthase 2	CTPS2	1	0.000427594	176.27	S	QQGCKLSSSDRYSDASDDSFSEPRIAELEIS	0	0	5961
Q9NRF8	574	CTP synthase 2	CTPS2	0.999251	0.000427594	176.27	S	CKLSSSDRYSDASDDSFSEPRIAELEIS___	0	0	5962
P18858	51	DNA ligase 1	LIG1	0.996452	1.27E-09	176.25	S	KAALKEWNGVVSESDSPVKRPGRKAARVLGS	4151200	4088700	1147
O94885	721	SAM and SH3 domain-containing protein 1	SASH1	0.999771	1.29E-09	176.17	S	SQEKLLVDSQGLSGCSPRDSGCYESSENLEN	1307000	1412900	712
Q9UQ35	1652	Serine/arginine repetitive matrix protein 2	SRRM2	0.737903	5.60E-10	176.17	S	RSGSSSKGRGPSPEGSSSTESSPEHPPKSRT	0	0	6632
O60841	113	Eukaryotic translation initiation factor 5B	EIF5B	1	1.33E-09	176.05	S	KKGQKGKKQSFDDNDSEELEDKDSKSKKTAK	569250	683480	534
Q13247	301	Serine/arginine-rich splicing factor 6	SRSF6	1	4.58E-09	175.88	S	KGDIKSKSRSRSQSRSNSPLPVPPSKARSVS	37338000	14732000	2441
Q96G46	277	tRNA-dihydrouridine(47) synthase [NAD(P)(+)]-like	DUS3L	0.77784	1.24E-13	175.77	S	AQQVPAGPGTSTPPSSPVRTCGPLTDEDVVR	0	20078000	4911
O95295	133	SNARE-associated protein Snapin	SNAPIN	1	2.01E-09	175.74	S	ARRRAMLDSGIYPPGSPGK____________	0	0	746
P18858	183	DNA ligase 1	LIG1	0.977993	4.93E-07	175.66	T	EVATEKEGEDGDQPTTPPKPLKTSKAETPTE	1594500	1471200	7302
Q9UEY8	673	Gamma-adducin	ADD3	1	2.67E-08	175.65	S	IEITIKSPEKIEEVLSPEGSPSKSPSKKKKK	1632100	3257500	6299
P46821	1917	Microtubule-associated protein 1B;MAP1B heavy chain;MAP1 li	MAP1B	0.627724	1.46E-09	175.6	S	GGYYYEKIERTTKSPSDSGYSYETIGKTTKT	6307200	0	1596
Q8NFC6	484	Biorientation of chromosomes in cell division protein 1-lik	BOD1L1	0.975653	1.46E-09	175.6	S	AYVHKPYLYSKYYSDSDDELTVEQRRQSIAK	878320	130210	4396
Q99956;Q	325;328;	Dual specificity protein phosphatase 9;Dual specificity pro	DUSP9;DUSP	0.5	1.68E-09	175.56	S	HLSLNDAYDLVKRKKSNISPNFNFMGQLLDF	0	0	5284
Q9HAW4	950	Claspin	CLSPN	0.997837	2.24E-09	175.56	S	ENMEELLNLCSGKFTSQDASTPASSELNKQE	1462400	1809800	5824
Q9HAW4	954	Claspin	CLSPN	0.851163	2.24E-09	175.56	S	ELLNLCSGKFTSQDASTPASSELNKQEKESS	0	586830	5825
Q00613	363	Heat shock factor protein 1	HSF1	0.999437	1.17E-09	175.52	S	LTDARGHTDTEGRPPSPPPTSTPEKCLSVAC	9455000	5160600	2032
Q00613	367	Heat shock factor protein 1	HSF1	0.751257	1.17E-09	175.52	T	RGHTDTEGRPPSPPPTSTPEKCLSVACLDKN	429020	232480	7470
Q14181	141	DNA polymerase alpha subunit B	POLA2	1	1.15E-10	175.34	S	STPETPLTKRSVSTRSPHQLLSPSSFSPSAT	3774800	4995600	2694
Q14181	152	DNA polymerase alpha subunit B	POLA2	0.994273	1.15E-10	175.34	S	VSTRSPHQLLSPSSFSPSATPSQKYNSRSNR	8145300	3969800	2696
Q7L014	804	Probable ATP-dependent RNA helicase DDX46	DDX46	1	3.04E-18	175.34	S	NERKKLQKAALGLQDSDDEDAAVDIDEQIES	1410800	2076500	3717
Q7Z5K2	77	Wings apart-like protein homolog	WAPAL	0.999935	1.89E-13	175.33	S	PKVEEESTGDPFGFDSDDESLPVSSKNLAQV	15290000	13643000	3844
O75821	41	Eukaryotic translation initiation factor 3 subunit G	EIF3G	0.987094	8.56E-14	175.33	T	VTSELLKGIPLATGDTSPEPELLPGAPLPPP	44484000	47434000	7219
O14733	57	Dual specificity mitogen-activated protein kinase kinase 7	MAP2K7	0.881916	2.37E-09	175.27	S	TLQLPLANDGGSRSPSESSSPQHPTPPARPR	2537100	0	201
Q6WKZ4;Q	156;150	Rab11 family-interacting protein 1;Rab11 family-interacting	RAB11FIP1;	0.999979	9.47E-10	175.27	S	EIEVDIQFMRNNMTASMFDLSMKDKSRNPFG	3233400	2511000	3639
Q12792	142	Twinfilin-1	TWF1	0.498926	3.38E-10	175.26	S	EDVSLHGYKKYLLSQSSPAPLTAAEEELRQI	0	0	2304
Q12792	143	Twinfilin-1	TWF1	0.498926	3.38E-10	175.26	S	DVSLHGYKKYLLSQSSPAPLTAAEEELRQIK	0	0	2305
Q6ZU35	975	Uncharacterized protein KIAA1211	KIAA1211	0.999979	7.39E-09	175.26	S	KPALAPKPTSQTPPASPLSKLSRPYLVELLS	993380	575290	3675
Q6ZU35	971	Uncharacterized protein KIAA1211	KIAA1211	0.999944	7.39E-09	175.26	T	PLAQKPALAPKPTSQTPPASPLSKLSRPYLV	993380	575290	7784
Q9Y2W1	315	Thyroid hormone receptor-associated protein 3	THRAP3	1	2.67E-09	175.16	S	GQFDHGSGSLSPSKKSPVGKSPPSTGSTYGS	4493500	1718700	6824
Q16512	562	Serine/threonine-protein kinase N1	PKN1	0.580598	9.96E-10	175.01	S	DISVEKLNLGTDSDSSPQKSSRDPPSSPSSL	8842400	6098100	3008
Q8IX01	754	SURP and G-patch domain-containing protein 2	SUGP2	0.982025	1.02E-13	174.91	S	PDPVGPSPQDPSLEASGPSPKPAGVDISEAP	6450800	3091800	4087
Q9BUR4	491	Telomerase Cajal body protein 1	WRAP53	0.988601	2.94E-13	174.91	S	LATASGQRVFPEPTESGDEGEELGLPLLSTR	2691400	2369900	5392
Q9H0B6	610	Kinesin light chain 2	KLC2	0.837742	5.57E-09	174.9	S	QPGGTGLSDSRTLSSSSMDLSRRSSLVG___	0	1033400	5564
P21359	2515	Neurofibromin;Neurofibromin truncated	NF1	0.994025	1.63E-15	174.87	S	SEGYLAATYPTVGQTSPRARKSMSLDMGQPS	2097200	2517400	1196
Q7Z422	107	SUZ domain-containing protein 1	SZRD1	0.997739	8.66E-09	174.87	S	EAEYAEARKRILGSASPEEEQEKPILDRPTR	5021400	3952600	3800
Q9Y6X4	526	Soluble lamin-associated protein of 75 kDa	FAM169A	0.972418	3.18E-09	174.87	S	EEKLSLLPRKKAHLGSSDNVATMSNEERSDG	538430	0	7024
O96013	181	Serine/threonine-protein kinase PAK 4	PAK4	1	3.85E-10	174.81	S	GSGGPQESSRDKRPLSGPDVGTPQPAGLASG	2059600	538610	843
Q53H80	21	Akirin-2	AKIRIN2	0.999944	1.72E-09	174.77	S	TLKRTLDFDPLLSPASPKRRRCAPLSAPTSA	476960	881200	3141
O43818	50	U3 small nucleolar RNA-interacting protein 2	RRP9	0.99999	0.00119933	174.72	S	DRGKSKGGGKMNEEISSDSESESLAPRKPEE	0	0	426
O95400	195	CD2 antigen cytoplasmic tail-binding protein 2	CD2BP2	0.958861	2.36E-08	174.72	S	ARGGGKGRKGPGQPSSPQRLDRLSGLADQMV	8886800	9411900	761
Q66K74	762	Microtubule-associated protein 1S;MAP1S heavy chain;MAP1S li	MAP1S	0.905208	1.55E-10	174.72	S	HRKAVPMAPAPASPGSSNDSSARSQERAGGL	13142000	0	3404
O15234	45	Protein CASC3	CASC3	0.977752	9.56E-11	174.7	S	LRGGGSCSGSAGGGGSGSLPSQRGGRTGALH	780060	0	307
Q96D71	170	RalBP1-associated Eps domain-containing protein 1	REPS1	0.999901	6.85E-18	174.7	S	ADAQEPASPVVSPQQSPPTSPHTWRKHSRHP	5609400	8041000	4859
Q96D71	174	RalBP1-associated Eps domain-containing protein 1	REPS1	0.938902	6.85E-18	174.7	S	EPASPVVSPQQSPPTSPHTWRKHSRHPSGGN	8417300	5294500	4860
Q96D71	173	RalBP1-associated Eps domain-containing protein 1	REPS1	0.938902	6.85E-18	174.7	T	QEPASPVVSPQQSPPTSPHTWRKHSRHPSGG	13891000	2746500	7977
Q15366	188	Poly(rC)-binding protein 2	PCBP2	0.687077	1.43E-08	174.68	S	SPPKGVTIPYRPKPSSSPVIFAGGQDRYSTG	3059500	3028700	2908
P20338	2	Ras-related protein Rab-4A	RAB4A	0.985891	7.52E-10	174.62	S	_______________MSQTAMSETYDFLFKFL	364250	691590	1184
O94887	425	FERM, RhoGEF and pleckstrin domain-containing protein 2	FARP2	0.907111	2.51E-09	174.59	S	SPSTLVPSGLPEFKDSSSSLTDPQVSYVKSP	0	0	713
O94887	426	FERM, RhoGEF and pleckstrin domain-containing protein 2	FARP2	0.426405	2.51E-09	174.59	S	PSTLVPSGLPEFKDSSSSLTDPQVSYVKSPA	0	0	714
Q7Z6Z7	2887	E3 ubiquitin-protein ligase HUWE1	HUWE1	0.873074	6.39E-10	174.59	S	GDTSAAGSSEQPRAGSSTPGDAPPAVAEVQG	1290400	1831600	3866
Q7Z6Z7	2888	E3 ubiquitin-protein ligase HUWE1	HUWE1	0.873074	6.39E-10	174.59	S	DTSAAGSSEQPRAGSSTPGDAPPAVAEVQGR	1290400	693160	3867
Q13595	202	Transformer-2 protein homolog alpha	TRA2A	0.999822	7.40E-09	174.42	T	GRRIRVDYSITKRAHTPTPGIYMGRPTHSGG	6238000	0	7564
P84157	128	Matrix-remodeling-associated protein 7	MXRA7	0.964767	2.08E-09	174.4	S	RQEEEQDLDGEKGPSSEGPEEEDGEGFSFKY	1479900	986650	1987
Q96S55	153	ATPase WRNIP1	WRNIP1	1	7.52E-09	174.4	S	GSGKRPAAAAAAGSASPRSWDEAEAQEEEEA	7989800	2843400	5110
Q99700	772	Ataxin-2	ATXN2	0.89852	8.47E-09	174.24	S	NSPAGNKENIKPNETSPSFSKAENKGISPVV	2998300	3387100	5254
Q9P1Y5	431	Calmodulin-regulated spectrin-associated protein 3	CAMSAP3	0.99927	6.25E-09	174.23	S	DVDVVMGDPVLLRSVSSDSLGPPRPAPARTP	1686600	970930	6198
P50402	10	Emerin	EMD	0.880297	1.48E-09	173.87	T	______MDNYADLSDTELTTLLRRYNIPHGP	482530	0	7430
P48506	5	Glutamate--cysteine ligase catalytic subunit	GCLC	0.999929	2.86E-09	173.77	S	___________MGLLSQGSPLSWEETKRHAD	195580	336700	1623
Q86TN4	240	tRNA 2'-phosphotransferase 1	TRPT1	0.979985	1.78E-09	173.77	S	KPLSLAGDEETECQSSPKHSSRERRRIQQ__	0	892990	3905
Q8IXM2	96	Chromatin complexes subunit BAP18	BAP18	1	2.88E-09	173.72	S	KRKVYEDSGIPLPAESPKKGPKKVASGVLSP	15814000	13227000	4107
Q9BVS4	332	Serine/threonine-protein kinase RIO2	RIOK2	0.990571	2.94E-09	173.59	S	HPLGPDDKNIETKEGSEFSFSDGEVAEKAEV	2287500	606900	5405
P11831	224	Serum response factor	SRF	0.997634	2.97E-09	173.51	S	ETGKALIQTCLNSPDSPPRSDPTTDQRMSAT	732440	345470	1030
P31350	20	Ribonucleoside-diphosphate reductase subunit M2	RRM2	1	2.40E-09	173.49	S	RVPLAPITDPQQLQLSPLKGLSLVDKENTPP	21244000	20840000	1356
O15541	84	RING finger protein 113A	RNF113A	0.997691	2.60E-13	173.43	S	KTRDSGKQKAAYGDLSSEEEEENEPESLGVV	24661000	21824000	326
Q9UQ35	384	Serine/arginine repetitive matrix protein 2	SRRM2	0.83585	1.18E-17	173.39	T	LLAERHGGSPQPLATTPLSQEPVNPPSEASP	11926000	0	8261

Q99613;B	181;181	Eukaryotic translation initiation factor 3 subunit C;Eukaryo	EIF3C;EIF3C	1	1.72E-06	173.37	S	SADEDAEKNEEDSEGSSDEDEDEDGVSAATF	0	0	71
Q99613;B	182;182	Eukaryotic translation initiation factor 3 subunit C;Eukaryo	EIF3C;EIF3C	1	1.72E-06	173.37	S	ADEDAEKNEEDSEGSSDEDEDEDGVSAATFL	0	0	72
O60271	730	C-Jun-amino-terminal kinase-interacting protein 4	SPAG9	0.917009	1.17E-08	173.34	S	DVAGLDTEGSKQRSASQSSLDKLDQELKEQQ	12262000	10099000	461
O60271	733	C-Jun-amino-terminal kinase-interacting protein 4	SPAG9	0.999175	1.17E-08	173.34	S	GLDTEGSKQRSASQSSLDKLDQELKEQQKEL	12262000	10099000	463
P04075	36	Fructose-bisphosphate aldolase A	ALDOA	0.773441	2.16E-09	173.33	S	HRIVAPGKGILAADESTGSIAKRLQSIGTEN	0	687470	873
Q9UFC0	259	Leucine-rich repeat and WD repeat-containing protein 1	LRWD1	0.999999	1.29E-13	173.24	S	PSKRACASPSAQVEGSPVAGSDGSQPAVKLE	3106700	2642100	6309
Q14202	213	Zinc finger MYM-type protein 3	ZMYM3	0.717409	1.96E-10	173.19	S	TLGDGINSSQTKPGGSSPPAHPSLPGDGLTA	1801500	1188800	2719
Q63HN8	2273	E3 ubiquitin-protein ligase RNF213	RNF213	0.999996	4.01E-09	173.19	S	MARDFATPSLHTSDQSPGKHMVTMDGVREED	635040	497590	3380
Q9P2D0	1045	Inhibitor of Bruton tyrosine kinase	IBTK	0.999999	3.11E-09	173.19	S	SEGSYAGVGSPRDLQSPDFTTGFHSDKIEAK	2140500	956660	6232
P19105;P	19;20;20	Myosin regulatory light chain 12A;Myosin regulatory light po	MYL12A;MYL9	0.5	1.04E-08	173.09	S	KRTKTKTKKRPQRATSNVFAMFDQSQIQEFK	1311300	440460	219
Q9Y4H2	306	Insulin receptor substrate 2	IRS2	0.448509	2.54E-09	173.09	S	MKALKELFEFRPRSKSQSSGSSATHPISVPG	0	0	6928
Q9Y4H2	308	Insulin receptor substrate 2	IRS2	0.394669	2.54E-09	173.09	S	ALKELFEFRPRSKSQSSGSSATHPISVPGAR	0	0	6929
P19105;P	18;19;19	Myosin regulatory light chain 12A;Myosin regulatory light po	MYL12A;MYL9	0.5	1.04E-08	173.09	T	SKRTKTKTKKRPQRATSNVFAMFDQSQIQEF	1311300	440460	7135
O95490	1430	Latrophilin-2	LPHN2	1	2.23E-09	173.06	S	AGHQLQMCYQISRGNSDGYIIPINKEGCIPE	2714400	1483900	780
Q6R327	21	Rapamycin-insensitive companion of mTOR	RICTOR	1	0.000405197	173.03	S	RGRSLKNLRVRGRNDSGEENVPLDLTREPSD	0	0	3578
Q96T58	309	Msx2-interacting protein	SPEN	0.998207	2.01E-10	173.03	S	SSDSSSSSSDDSPARSVQSAAVPAPTSQLLS	3009400	2990700	5183
Q9UQ35	1397	Serine/arginine repetitive matrix protein 2	SRRM2	0.999836	8.10E-10	173.03	S	SSSELSPDAVEKAGMSSNQSISSPVLDAVPR	2914900	1508200	6596
P46821	1503	Microtubule-associated protein 1B;MAP1B heavy chain;MAP1 lig	MAP1B	0.5	1.27E-09	173.03	T	PALALDERKLGDVSPTQIDVSQFGSFKEDTK	716610	0	7390
P40818	716	Ubiquitin carboxyl-terminal hydrolase 8	USP8	0.684061	4.14E-09	172.95	S	QIPAERDREPSKLKRSYSSPDITQAIQEEEK	0	1264600	1476
Q9NR19	265	Acetyl-coenzyme A synthetase, cytoplasmic	ACSS2	0.517676	4.17E-09	172.91	S	VKHLGRAELGMGDSTSQSPPIKRSCPDVQIS	0	428320	5941
Q3KQU3	548	MAP7 domain-containing protein 1	MAP7D1	1	1.49E-12	172.77	S	ASNEKESAAPASPAPSPAPSPTPAPPQKEQP	28802000	24138000	3060
P08559	232	Pyruvate dehydrogenase E1 component subunit alpha, somatic f	PDHA1	0.938597	1.15E-08	172.61	S	PCIFICENNRYGMGTSVERAAASTDYYKRGD	8923400	5348700	954
Q15554	365	Telomeric repeat-binding factor 2	TERF2	1	4.34E-09	172.6	S	LDQKDLVLPTQALPASPALKNKRPRKDENES	3651900	3331100	2944
P24928	1906	DNA-directed RNA polymerase II subunit RPB1	POLR2A	0.853071	1.31E-10	172.58	S	TSPTYSPTSPVYTPTSPKYSPTSPTYSPTSP	0	512850	1249
O14745	280	Na(+)/H(+) exchange regulatory cofactor NHE-RF1	SLC9A3R1	1	2.38E-09	172.57	S	QKENSREALAEAALESPRPALVRSASSDTSE	468820	417890	204
Q07157	617	Tight junction protein ZO-1	TJP1	1	6.51E-09	172.57	S	WRFRGLRSSKRNLRKSREDLSAQPVQTKFPA	5905700	2057100	2225
Q13200	16	26S proteasome non-ATPase regulatory subunit 2	PSMD2	1	1.89E-12	172.57	S	MEEGGRDKAPVQPQQSPAAAPGGTDEKPSGK	41751000	22468000	2433
O75376	2438	Nuclear receptor corepressor 1	NCOR1	0.999561	2.72E-09	172.56	S	EPAPLLSAQYETLSDSDD_____________	2466900	0	594
Q13459	1267	Unconventional myosin-IXb	MYO9B	1	3.40E-08	172.56	S	TSLALDSRVSPPAPGSAPETPEDKSKPCGSP	2418200	1507500	2541
Q99684	56	Zinc finger protein Gfi-1	GFI1	0.999788	1.52E-08	172.56	S	TSNAGGAKAEPRDRLSPESQLTEAPDRASAS	0	0	5243
Q13459	1271	Unconventional myosin-IXb	MYO9B	1	3.40E-08	172.56	T	LDSRVSPPAPGSAPETPEDKSKPCGSPRVQE	2418200	1507500	7545
P53396	481	ATP-citrate synthase	ACLY	0.999998	2.42E-09	172.43	S	PAKKAKPAMPQDSVPSPRSLQGKSTTLFSRH	6058000	5135700	1833
Q05655	506	Protein kinase C delta type;Protein kinase C delta type regu	PRKCD	0.945412	2.17E-10	172.42	S	DFGMCKENIFGESRASTFCGTPDYIAPEILQ	991810	336850	2194
Q05655	507	Protein kinase C delta type;Protein kinase C delta type regu	PRKCD	0.5	2.17E-10	172.42	T	FGMCKENIFGESRASTFCGTPDYIAPEILQG	991810	336850	7497
Q8ND76	326	Cyclin-Y	CCNY	0.996359	8.16E-09	172.33	S	DKYKDLRRSARKRSASADNLTLPRWSPAIIS	3012100	1756700	4343
Q96RU3	296	Formin-binding protein 1	FNBP1	0.999994	8.16E-09	172.33	S	DIEFEDYTQPMKRTVSDNSLSNSRGEGKPDL	2417100	1935900	5105
Q8NDT2	552	Putative RNA-binding protein 15B	RBM15B	0.965516	2.47E-09	172.27	S	LYSDRDRTFLEGDWTSPSKSSDRRNSLEGYS	3087700	1394500	4359
O75925	488	E3 SUMO-protein ligase PIAS1	PIAS1	0.499876	1.02E-08	172.21	S	EEPSAKRTCPSLSPTSPLNNKGILSLPHQAS	0	0	670
O75925	487	E3 SUMO-protein ligase PIAS1	PIAS1	0.499876	1.02E-08	172.21	T	EEEPSAKRTCPSLSPTSPLNNKGILSLPHQA	0	0	7220
P04075	39	Fructose-bisphosphate aldolase A	ALDOA	0.99996	2.53E-09	172.09	S	VAPGKGILAADESTGSIAKRLQSIGTENTEE	2009500	1570200	874
P05455	94	Lupus La protein	SSB	0.830168	1.14E-08	172.09	S	LMEISEDKTKIRRSPSKPLPEVTDEYKNDVK	2273600	736040	907
Q14839	1531	Chromodomain-helicase-DNA-binding protein 4	CHD4	1	4.02E-08	172.09	S	WSMPELAEVEENKKMSQPGSPSPKTPTPSTP	4319300	2511000	2815
P30419	47	Glycylpeptide N-tetradecanoyltransferase 1	NMT1	1	2.55E-09	171.99	S	DCENEEDNSYNRGGLSPANDTGAKKKKKKQK	8804200	7418000	1345
Q14004	437	Cyclin-dependent kinase 13	CDK13	0.99994	2.12E-09	171.99	S	SRSRHRLSRSRSRHSSISPSTLTLKSSLAAE	6067900	3095200	2637
Q14004	439	Cyclin-dependent kinase 13	CDK13	0.998082	2.12E-09	171.99	S	SRHRLSRSRSRHSSISPSTLTLKSSLAAELN	6067900	3095200	2638
Q9Y2W1	379	Thyroid hormone receptor-associated protein 3	THRAP3	0.995814	2.55E-09	171.99	S	QTNTDKEKIKEKGSFSDTGLGDGKMKSDSFA	6306600	6343000	6821
P06748	125	Nucleophosmin	NPM1	1	7.93E-24	171.94	S	HISGQHLVAVEEDAESEDEEEEDVKLLSISG	4002000	4279600	919
Q13523	565	Serine/threonine-protein kinase PRP4 homolog	PRPF4B	0.486222	4.79E-11	171.9	S	QKYKYLAEDSNMSVPSEPSSPQSSTRTRSPS	0	0	2565
Q13523	572	Serine/threonine-protein kinase PRP4 homolog	PRPF4B	0.792703	4.79E-11	171.9	S	EDSNMSVPSEPSSPQSSTRTRSPSPDDILER	3272300	614830	2568
Q9NUE0	19	Palmitoyltransferase ZDHHC18	ZDHHC18	1	1.42E-10	171.9	S	CEYQQISPGAAPLPASPGARRPGPAASPTPG	6917200	4571300	6020
Q9NZT2	581	Opioid growth factor receptor	OGFR	0.999983	4.05E-14	171.9	T	PAGPAGDEPAESPSETPGPSPAGPTRDEPAE	10763000	8242200	8186
Q9UQ35	400	Serine/arginine repetitive matrix protein 2	SRRM2	0.679863	5.13E-18	171.87	T	PLSQEPVNPPSEASPTRDRSPPKSPEKLPQS	8829900	15670000	8262
Q9UQR0	511	Sex comb on midleg-like protein 2	SCML2	0.999998	3.07E-09	171.84	S	TKRSPQQTVPYVVPLSPKLPKTKEYASEGEP	1695900	887240	6762
P51532	695	Transcription activator BRG1	SMARCA4	1	3.71E-09	171.78	S	PTLPVEEKKKIPDPDSDDVSEVDARHIIENA	955060	894510	1784
Q8TDY2	237	RB1-inducible coiled-coil protein 1	RB1CC1	0.5	2.65E-09	171.67	S	SLPEHEDSEKAEMKRSTELVLSPDMPRTTNE	1337900	941410	4472
Q8TDY2	238	RB1-inducible coiled-coil protein 1	RB1CC1	0.752455	2.65E-09	171.67	T	LPEHEDSEKAEMKRSTELVLSPDMPRTTNES	1337900	941410	7907
Q14181	127	DNA polymerase alpha subunit B	POLA2	0.998924	2.18E-08	171.62	T	SYTTPSKGSQKRAISTPETPLTKRSVSTRSP	2044400	1539300	7587
Q14181	130	DNA polymerase alpha subunit B	POLA2	0.999998	2.18E-08	171.62	T	TPSKGSQKRAISTPETPLTKRSVSTRSPHQL	2044400	1539300	7588
Q13185	176	Chromobox protein homolog 3	CBX3	0.99994	2.70E-09	171.49	S	PQIVIAFYEERLTWHSCPEDEAQ________	5076200	16642000	2431
Q15036	333	Sorting nexin-17	SNX17	0.406828	5.13E-09	171.46	S	MRCWRVTSSVPLPSGSTSSPGRGRGEVRLEL	0	0	2860
Q02447	73	Transcription factor Sp3	SP3	1	1.06E-31	171.36	S	PLALLAATCSKIGPPSPGDDEEEAAAAAGAP	1491000	1696900	2095
Q8TAD8	52	Smad nuclear-interacting protein 1	SNIP1	0.991092	1.50E-08	171.33	S	EVAPPAHRRPDHSGGSPSPPTSEPARSGHRG	10316000	6341700	4433
Q8TAD8	54	Smad nuclear-interacting protein 1	SNIP1	0.999986	1.50E-08	171.33	S	APPAHRRPDHSGGSPSPPTSEPARSGHRGNR	10221000	4920200	4434
Q13425	228	Beta-2-syntrophin	SNTB2	0.452299	2.41E-18	171.3	S	LPWEGAAPQSPSFSGSEDSGSPKHQNSTKDR	0	0	2487
Q8WVB6	64	Chromosome transmission fidelity protein 18 homolog	CHTF18	0.820186	1.53E-10	171.3	S	PRTFEEALARGDAASSPAPAASVGSSQGGAR	0	0	4535
P33981	281	Dual specificity protein kinase TTK	TTK	1	2.78E-09	171.29	S	KQSCPFGRVPVNLLNSPDCDVKTDDSVVPCF	1166000	1151000	1365
P10636	721	Microtubule-associated protein tau	MAPT	0.973102	1.69E-08	171.26	S	GAEIVYKSPVVSGDTSPRHLSNVSSTGSIDM	6417200	8562900	1000
P31689	335	DnaJ homolog subfamily A member 1	DNAJA1	1	1.69E-09	171.26	S	LIIEFKVNFPENGFLSPDKLSLLEKLLPERK	1388800	1347200	1357
P10636	720	Microtubule-associated protein tau	MAPT	0.698105	3.77E-08	171.26	T	HGAEIVYKSPVVSGDTSPRHLSNVSSTGSID	4422500	0	7276
O00161	5	Synaptosomal-associated protein 23	SNAP23	0.847987	1.19E-09	171.21	S	___________MDNLSSEEIQQRAHQITDES	2209000	1666800	95
Q9BVJ6	453	U3 small nucleolar RNA-associated protein 14 homolog A	UTP14A	0.98955	2.79E-09	171.21	S	QDAEPAGSQETKDSGSQEVLSELRVLSQKLK	376690	982240	5403
Q96JM3	459	Chromosome alignment-maintaining phosphoprotein 1	CHAMP1	0.999392	1.16E-08	171.15	S	GSPDLWKLSPDQRKTSPASLDFPESQKSSRG	0	0	4980
Q9HC35	144	Echinoderm microtubule-associated protein-like 4	EML4	0.999966	5.14E-09	171.15	S	KEESHSNDQSPQIRASPSPQPSSQPLQIHRQ	17528000	14732000	5838
P24928	1917	DNA-directed RNA polymerase II subunit RPB1	POLR2A	0.931883	2.83E-09	171.11	S	YTPTSPKYSPTSPTYSPTSPKYSPTSPTYSP	1498400	780210	1244
P61006	181	Ras-related protein Rab-8A	RAB8A	0.610933	2.83E-09	171.11	S	ARDIKAKMDKKLEGNSPQGSNQGVKITPDQQ	0	0	1906
Q8WUA4	893	General transcription factor 3C polypeptide 2	GTF3C2	0.986523	2.83E-09	171.11	S	QLESRAHFNAMFQPSSPTRRPGFSPTSHRLL	0	0	4503
O00139	585	Kinesin-like protein KIF2A	KIF2A	0.783341	1.01E-13	171	S	PPNQIDDLETQWGVGSSPQRDDLKLLCEQNE	1966800	0	91
Q6P1L5	106	Protein FAM117B	FAM117B	0.494886	2.21E-08	170.95	S	STSPTRGGGNAAARTSPTVATQTGASATSTR	0	0	3510
Q6P1L5	105	Protein FAM117B	FAM117B	0.494886	2.21E-08	170.95	T	RSTSPTRGGGNAAARTSPTVATQTGASATST	0	0	7754
Q6IQ49	278	Protein SDE2 homolog	SDE2	0.997885	1.59E-10	170.92	S	PSGSQRARVVNTDHGSPEQLQIPVTDSGRHI	23785000	14059000	3483
Q8NEL9	723	Phospholipase DDHD1	DDHD1	0.999995	1.59E-12	170.92	S	EPTSVSENEGISTIPSPVTSPVLSRRHYGES	1738900	984190	4370
Q8NEL9	726	Phospholipase DDHD1	DDHD1	0.980239	1.59E-12	170.92	T	SVSENEGISTIPSPVTSPVLSRRHYGESITN	1738900	0	7894
P49840;P4	278;215	Glycogen synthase kinase-3 alpha;Glycogen synthase kinase-3	GSK3A;GSK3B	0.648955	9.62E-09	170.89	S	DFGSAKQLVRGEPNVSYICSRYYRAPELIFG	9870400	8140600	1732
Q15477	256	Helicase SKI2W	SKIV2L	0.953425	1.11E-08	170.89	S	TVSASPCSAPLARASSLEDLVLKEASTAVST	3514700	2967900	2939
Q96KR1	1054	Zinc finger RNA-binding protein	ZFR	1	1.19E-08	170.89	S	SQRFNIHNNRKRRRDSDGVDGFEAEGKKDKK	0	1654300	5008
Q14157	460	Ubiquitin-associated protein 2-like	UBAP2L	0.269397	2.07E-13	170.8	S	STAAPPPPSSPLPSKSTSAPQMSPGSSDNQS	0	0	2667
Q14157	462	Ubiquitin-associated protein 2-like	UBAP2L	0.269397	2.07E-13	170.8	S	AAPPPPSSPLPSKSTSAPQMSPGSSDNQSSS	0	0	2668
Q99856	77	AT-rich interactive domain-containing protein 3A	ARID3A	0.999991	3.67E-18	170.8	S	LAAMRAAAAGLGHPASPGGSEDGPPGSEEED	20203000	8512100	5277
Q14157	461	Ubiquitin-associated protein 2-like	UBAP2L	0.269397	2.07E-13	170.8	T	TAAPPPPSSPLPSKSTSAPQMSPGSSDNQSS	0	0	7579
Q9NUL3	485	Double-stranded RNA-binding protein Staufen homolog 2	STAU2	0.499067	4.16E-09	170.74	S	MNGTSSTAEAIGLKGSSPTPPCSPVQPSKQL	0	0	6022
Q04637	314	Eukaryotic translation initiation factor 4 gamma 1	EIF4G1	0.999959	2.90E-14	170.5	S	ESTPISRETGEPYRLSPEPTPLAEPILEVEV	3393500	1854100	2157
Q96PC5	1202	Melanoma inhibitory activity protein 2	MIA2	0.994656	4.58E-09	170.5	S	CDRLTDPHRAPSDTGSLSPPWDQDRRMMFPP	659920	604300	5050
Q96PC5	1204	Melanoma inhibitory activity protein 2	MIA2	1	4.58E-09	170.5	S	RLTDPHRAPSDTGSLSPPWDQDRRMMFPPPG	1365600	604300	5051
Q8N5F7	149	NF-kappa-B-activating protein	NKAP	1	4.27E-09	170.48	S	RERIGELGAPEVWGLSPKNPEPDSDEHTPVE	8372900	9159300	4283
Q9Y6D6	394	Brefeldin A-inhibited guanine nucleotide-exchange protein 1	ARFGEF1	0.624709	6.74E-11	170.47	S	PISVAYTPSLPDDRLSVSSNDTQESGNSSGP	0	462470	6997
Q9UQ35	1265	Serine/arginine repetitive matrix protein 2	SRRM2	0.905818	2.26E-08	170.42	T	GQILSHLSSELKEMSTSNFESSPEVEERPAV	0	5329900	8255
Q4G0J3	300	La-related protein 7	LARP7	0.968553	3.03E-09	170.4	S	SSLPEVRTGKRKRSSSEDAESLAPRSKVKKI	0	0	3095
Q13627;Q	321;273	Dual specificity tyrosine-phosphorylation-regulated kinase 1	DYRK1A;DYRK	1	2.24E-07	170.24	Y	VDFGSSCQLGQRIYQYIQSRFYRSPEVLLGM	10523000	6990500	8378
P29966	26	Myristoylated alanine-rich C-kinase substrate	MARCKS	0.672657	6.06E-09	170.22	S	KGEAAAERPGEAAVASSPSKANGQENGHVKV	14311000	7488800	1335
Q5SW79	928	Centrosomal protein of 170 kDa	CEP170	0.991952	2.24E-13	170.02	S	KTDEGPDTPSYNRDNSISPESDVDTASTISL	1778400	1002800	3216
Q96B01	120	RAD51-associated protein 1	RAD51AP1	1	4.48E-09	170	S	LSVKELPTVTTNVQNSQDKSIEKHGSSKIET	2475400	3590900	4787
P23396	220	40S ribosomal protein S3	RPS3	0.627981	7.82E-09	170	T	PDHVSIVEPKDEILPTTPISEQKGGKPEPPA	27009000	0	7316
P46821	1779	Microtubule-associated protein 1B;MAP1B heavy chain;MAP1 lig	MAP1B	1	1.42E-08	169.98	S	SLTSEKVQSLEGEKLSPKSDISPLTPRESSP	5560600	3036800	1608
Q86TV6	160	Tetratricopeptide repeat protein 7B	TTC7B	0.868116	3.18E-09	169.98	S	YATKGLCLEKLPISSSTSNLHVDREQDVITC	5255700	3443500	3907
Q9H7N4	738	Splicing factor, arginine/serine-rich 19	SCAF1	0.999991	3.18E-09	169.98	S	ASSPKREVLYDSEGLSGEERGGKSSQKDRRR	0	0	5767
Q9NU22	4898	Midasin	MDN1	1	3.27E-10	169.77	T	DDLNLDSEDKNGGEDTDNEEGEEENPLEIKE	787320	1001300	8165
Q6KC79	306	Nipped-B-like protein	NIPBL	0.990764	8.97E-10	169.67	S	SRPPLILQSQSLPCSSPRDVPPDILLDSPER	2898500	4334700	3487
Q12789	739	General transcription factor 3C polypeptide 1	GTF3C1	1	3.74E-09	169.62	S	KTSQPPVPQGEAEEDSQGKEGPSGSGDSQLS	0	553450	2303
P18669	14	Phosphoglycerate mutase 1	PGAM1	1	1.09E-08	169.58	S	__MAAYKLVLIRHGESAWNLENRFSGWYDAD	3909300	2278800	1143
P43487	60	Ran-specific GTPase-activating protein	RANBP1	1	1.09E-08	169.58	S	EEELFKMRAKLFRFASENDLPEWKERGTGDV	1177500	1070500	1526
Q14157	453	Ubiquitin-associated protein 2-like	UBAP2L	0.840086	1.81E-10	169.58	S	KSPAVATSTAAPPPPSSPLPSKSTSAPQMSP	0	9200700	2665
Q9H2Y7	1279	Zinc finger protein 106	ZNF106	0.999838	6.02E-09	169.58	S	LKFSVEQRNTRNRENSPSSQSAGLSSINKEG	6598200	3806500	5627
Q9Y606	426	tRNA pseudouridine synthase A, mitochondrial	PUS1	1	1.90E-08	169.58	T	AKVPSPLEGSEGDGDTD______________	1570200	4961100	8312
Q08170;Q	113;117;1	Serine/arginine-rich splicing factor 4;Serine/arginine-rich	SRSF4;SRSF	0.903011	1.60E-08	169.52	S	PPTRTEYRLIVENLSSRCSWQDLKDYMRQAG;PPVI	6595600	2556600	2243
P01891	356	HLA class I histocompatibility antigen, A-68 alpha chain	HLA-A	0.999965	3.39E-13	169.44	S	GGSYSQAASSDSAQGSDVSLTACKV______	6773000	4440600	857
P01891	359	HLA class I histocompatibility antigen, A-68 alpha chain	HLA-A	0.921779	3.39E-13	169.44	S	YSQAASSDSAQGSDVSLTACKV_________	3553200	2726400	858
P04920	132	Anion exchange protein 2	SLC4A2	1	2.93E-18	169.3	S	ETPTIEEGEEDEDEASEAEGARALTQPSPVS	2597300	1693100	892
Q9BRR8	6	G patch domain-containing protein 1	GPATCH1	1	1.49E-13	169.3	S	__________MAARDSDSEEDLVSYGTGLEP	199410	0	5324
Q9BRR8	8	G patch domain-containing protein 1	GPATCH1	1	1.49E-13	169.3	S	________MAARDSDSEEDLVSYGTGLEPLE	199410	0	5325
Q6WCQ1	218	Myosin phosphatase Rho-interacting protein	MPRIP	0.546803	6.12E-14	169.28	S	LWQEEMRTKDQPDGSSLSPAQSPSQSQPPAA	0	627180	3629
Q6WCQ1	226	Myosin phosphatase Rho-interacting protein	MPRIP	0.833708	6.12E-14	169.28	S	KDQPDGSSLSPAQSPSQSQPPAASSLREPGL	1000900	627180	3632
Q86VM9	534	Zinc finger CCCH domain-containing protein 18	ZC3H18	0.999664	1.65E-08	169.21	S	WRRSKSPKKKLGVSVSPSRARRRRKTSASSA	27908000	22566000	3951
P07814	886	Bifunctional glutamate/proline--tRNA ligase;Glutamate--tRNA	EPRS	0.995549	1.25E-09	169.17	S	EYIPGQPPLSQSSDSSPTRNSEPAGLETPEA	66860000	43498000	939
Q14004	436	Cyclin-dependent kinase 13	CDK13	0.999983	2.83E-09	169.17	S	RSRSRHRLSRSRSRHSSISPSTLTLKSSLAA	1625600	927800	2636
Q92625	661	Ankyrin repeat and SAM domain-containing protein 1A	ANKS1A	0.941705	2.07E-09	169.17	S	ESLSNCSIGKKRLEKSPSFASEWDEIEKIMS	0	0	4684
Q96ST2	720	Protein IWS1 homolog	IWS1	0.999911	1.32E-08	169.09	S	EQRDLEQMPQRRRMNSTGGQTPRRDLEKVLT	1293700	409170	5135
Q96ST2	235	Protein IWS1 homolog	IWS1	1	1.88E-08	169.06	S	QVSDSESEEPPRHQASDSENEELPKPRISDS	25470000	22939000	5129
Q96ST2	237	Protein IWS1 homolog	IWS1	1	1.88E-08	169.06	S	SDSESEEPPRHQASDSENEELPKPRISDSES	25470000	22939000	5130
Q9UER7	688	Death domain-associated protein 6	DAXX	0.983476	1.65E-10	169.06	S	LASLAPVADSSTRVDSPSHGLVTSSLCIPSP	5861600	2613500	6294
Q9UER7	702	Death domain-associated protein 6	DAXX	1	1.65E-10	169.06	S	DSPSHGLVTSSLCIPSPARLSQTPHSQPPRP	3751000	2613500	6296
P07814	882	Bifunctional glutamate/proline--tRNA ligase;Glutamate--tRNA	EPRS	0.936268	1.25E-09	168.99	S	KTGKEYIPGQPPLSQSSDSSPTRNSEPAGLE	0	5354200	936
Q8TEW0	852	Partitioning defective 3 homolog	PARD3	1	1.15E-08	168.98	S	SDASQLDFVKTRKSKSMDLGIADETKLNTVD	1873600	1305200	4494
Q3V6T2	1702	Girdin	CCDC88A	0.737693	2.12E-09	168.93	S	LEESNKLTSVQIKSSSQENLLDEVMKSLSVS	1143200	0	3078
Q76FK4	378	Nucleolar protein 8	NOL8	0.99495	2.72E-08	168.93	S	HDSDDDIMRNDREYDSGDTDEIIAMKKNVAK	1092600	1236800	3699
Q92560	595	Ubiquitin carboxyl-terminal hydrolase BAP1	BAP1	0.783141	3.50E-09	168.93	S	KGSSPSIRPIQGSQGSSSPVEKEVVEATDSR	0	1323100	4636
Q76FK4	381	Nucleolar protein 8	NOL8	0.999116	2.72E-08	168.93	T	DDDIMRNDREYDSGDTDEIIAMKKNVAKVKN	1092600	1236800	7788
P09543	170	2',3'-cyclic-nucleotide 3'-phosphodiesterase	CNP	1	1.16E-08	168.89	S	RLDCAQLKEKNQWQLSADDLKKLKPGLEKDF	3199200	1628000	969
P0C1Z6	249	TCF3 fusion partner	TFPT	1	1.77E-09	168.83	S	VSRGPDKLLPYPTLASPASD___________	2620300	2000400	974
Q9UQ35	2123	Serine/arginine repetitive matrix protein 2	SRRM2	0.999975	1.35E-08	168.83	S	ALNSSRMSCFSRPSMSPTPLDRCRSPGMLEP	758370	824850	6659

Q4KWH8	1307	1-phosphatidylinositol 4,5-bisphosphate phosphodiesterase et	PLCH1	0.939678	3.37E-11	168.82	S	SSKAKTAALESNLPGSPNTSRGWLPKSPTKG	1819600	868280	3099
Q9NWH9	553	SAFB-like transcription modulator	SLTM	1	3.53E-09	168.76	S	ESIKKSEEKKRISSKSPGHMVILDQTKGDHC	15106000	8695900	6058
P51946	315	Cyclin-H	CCNH	1	2.05E-08	168.74	T	DDYVSKKSKHEEEEWTDDDLVESL_______	0	872300	7442
Q9Y6D6	396	Brefeldin A-inhibited guanine nucleotide-exchange protein 1	ARFGEF1	0.491159	7.95E-11	168.68	S	SVAYTPSLPDDRLSVSSNDTQESGNSSGPSP	0	0	6998
Q9Y6D6	397	Brefeldin A-inhibited guanine nucleotide-exchange protein 1	ARFGEF1	0.438367	7.95E-11	168.68	S	VAYTPSLPDDRLSVSSNDTQESGNSSGPSPG	0	0	6999
Q9UK58	352	Cyclin-L1	CCNL1	0.999983	1.60E-08	168.67	S	ASKPSSPREVKAEEKSPISINVKTVKKEPED	2826500	2551700	6417
Q5H9R7	579	Serine/threonine-protein phosphatase 6 regulatory subunit 3	PPP6R3	1	3.58E-09	168.64	S	FNDEKFADQDDIGNVSFDRVSDINFTLNTNE	2068500	2087000	3164
Q96Q45	49	Transmembrane protein 237	TMEM237	0.899124	6.76E-09	168.62	S	PKKKKPRTKNTPASASLEGLAQTAGRRPSEG	1309400	1667800	5073
Q9H7L9	234	Sin3 histone deacetylase corepressor complex component SDS3	SUDS3	0.99998	3.82E-10	168.62	S	DLRTLNKLKSPKRPASPSSPEHLPATPAESP	82900000	51163000	5758
Q9H7L9	237	Sin3 histone deacetylase corepressor complex component SDS3	SUDS3	0.87295	3.82E-10	168.62	S	TLNKLKSPKRPASPSSPEHLPATPAESPAQR	1481800	1272100	5760
O60684	6	Importin subunit alpha-7	KPNA6	0.999984	1.81E-09	168.5	S	__________METMASPGKDNYRMKSYKNNA	2723300	2338200	513
Q13112	432	Chromatin assembly factor 1 subunit B	CHAF1B	0.974807	4.00E-08	168.47	T	EGTPASRTQDPSSPGTTPPQARQAPAPTVIR	1793100	1507300	7524
P16403	4	Histone H1.2	HIST1H1C	0.676421	2.62E-09	168.45	T	____________MSETAPAAPAAAPPAEKAP	41932000	18045000	7292
Q9ULL5	1381	Proline-rich protein 12	PRR12	0.996491	3.46E-09	168.42	S	LDEELKRNLETLPSFSSDEEDSVAKNRDLQE	4556100	2670800	6487
Q9ULL5	1382	Proline-rich protein 12	PRR12	0.991592	3.46E-09	168.42	S	DEELKRNLETLPSFSSDEEDSVAKNRDLQES	4556100	2670800	6488
Q4VCS5	712	Angiomotin	AMOT	0.903457	1.65E-10	168.16	S	DAAATVAAQRDTTVISHSPNTSYDTALEARI	5451600	7789700	3112
Q99856	81	AT-rich interactive domain-containing protein 3A	ARID3A	0.999998	4.69E-18	168.12	S	RAAAAGLGHPASPGGSEDGPPGSEEEDAARE	0	2867000	5278
Q684P5	609	Rap1 GTPase-activating protein 2	RAP1GAP2	0.868499	5.71E-09	168.05	S	PDGGHSSQEIKSETSSNPSSPEICPNKEKPF	0	0	3416
Q684P5	613	Rap1 GTPase-activating protein 2	RAP1GAP2	0.969956	5.71E-09	168.05	S	HSSQEIKSETSSNPSSPEICPNKEKPFMKLK	0	0	3417
Q9UQ35	1233	Serine/arginine repetitive matrix protein 2	SRRM2	0.99532	2.07E-10	168.05	S	SSPETKEQNSALPTSSQDEELMEVVEKSEEP	395890	3635300	6626
Q9HC35	146	Echinoderm microtubule-associated protein-like 4	EML4	0.999819	6.94E-09	167.92	S	ESHSNDQSPQIRASPSPQPSSQPLQIHRQTP	13182000	10199000	5839
Q9HCH0	489	Nck-associated protein 5-like	NCKAP5L	0.999982	3.80E-09	167.92	S	PQLPRNSRIPCRNSGSDGSPSPLLARRGLGG	1019900	892460	5859
Q9Y4F1	427	FERM, RhoGEF and pleckstrin domain-containing protein 1	FARP1	0.999998	5.38E-09	167.92	S	GKEPKVSAGEPGSHPSPAPRRSPAGNKQADG	1013000	596460	6912
Q13136	1159	Liprin-alpha-1	PPFIA1	0.999946	2.19E-08	167.92	T	FRPKDIRGLAAGSAETLPANFRVTSSMSSPS	890790	617860	7525
Q99460	270	26S proteasome non-ATPase regulatory subunit 1	PSMD1	0.791778	2.73E-12	167.9	T	SASQQFLSSVIQNLRTVGTPIASVPGSTNTG	1764300	1703200	8022
Q99460	273	26S proteasome non-ATPase regulatory subunit 1	PSMD1	0.5	2.73E-12	167.9	T	QQFLSSVIQNLRTVGTPIASVPGSTNTGTVP	1764300	1703200	8023
Q9H2U2	316	Inorganic pyrophosphatase 2, mitochondrial	PPA2	0.782169	3.81E-09	167.84	S	RCTQEEARSLVESVSSSPNKESNEEEQVWHF	0	0	5621
Q8NFC6	1531	Biorientation of chromosomes in cell division protein 1-like	BOD1L1	0.999279	6.67E-08	167.73	S	SVATSTEGKDKDVTLSPVKAGPATTTSSETR	1471900	1321800	4392
P49790	516	Nuclear pore complex protein Nup153	NUP153	0.969511	5.48E-09	167.69	S	PINSSQALTNKVQMTSPSSTGSPMFKFSSPI	1682400	2905000	1698
Q02880	1581	DNA topoisomerase 2-beta	TOP2B	0.89661	2.77E-13	167.59	S	KTTSKKPKKTSFDQDSDVDIFPSDFPTEPPS	1302000	934240	2107
Q8IWX8	817	Calcium homeostasis endoplasmic reticulum protein	CHERP	0.999942	3.96E-12	167.36	S	SKSYSPGRRRRSRSRSPTPPSSAGLGSNSAP	58199000	27564000	4078
Q8IWX8	815	Calcium homeostasis endoplasmic reticulum protein	CHERP	0.999897	3.96E-12	167.36	S	SRSKSYSPGRRRRSRSRSPTPPSSAGLGSNS	54676000	27518000	4081
Q8IWX8	819	Calcium homeostasis endoplasmic reticulum protein	CHERP	0.832573	3.96E-12	167.36	T	SYSPGRRRRSRSRSPTPPSSAGLGSNSAPPI	0	1178000	7847
P27816	687	Microtubule-associated protein 4	MAP4	0.993478	2.97E-08	167.29	T	EEGRPVVSGTGNDITTPPNKELPPSPEKKTK	9512300	8870400	7337
Q9UPU5	2047	Ubiquitin carboxyl-terminal hydrolase 24	USP24	1	1.33E-08	167.22	S	WNAYMLFYQRVSDQNSPVLPKKSRVSVVRQE	19054000	9341700	6589
Q9H0B6	609	Kinesin light chain 2	KLC2	0.70437	1.34E-08	167.2	S	TQPGGTGLSDSRTLSSSSMDLSRRSSLVG__	0	0	5563
Q63ZY6;Q	143;327	Putative methyltransferase NSUN5C;Probable 28S rRNA (cytosir	NSUN5P2;NSU	0.999961	4.05E-09	167.11	S	GMPSRQLEEPGAGTPSPVRLHALAGFQQRAL	619230	323430	3383
P18583	1769	Protein SON	SON	0.999963	2.47E-09	167.03	S	GPLLASDVGRDRSAASPVVSSMPERASESSS	12010000	7144800	1132
Q9P260	180	LisH domain and HEAT repeat-containing protein KIAA1468	KIAA1468	0.999894	4.08E-09	167.03	S	EPSTASGGGQLNRAGSISTLDSLDFARYSDD	863820	2178900	6218
Q9ULM3	465	YEATS domain-containing protein 2	YEATS2	0.956484	7.75E-09	166.9	S	KSFQPITMSCKIVSGSPISTPSPSPLPRTPT	2976300	2000000	6496
P23921	780	Ribonucleoside-diphosphate reductase large subunit	RRM1	1	4.13E-09	166.79	S	KEEEEKERNTAAMVCSLENRDECLMCGS___	980530	450500	1230
Q9HCS5	448	Band 4.1-like protein 4A	EPB41L4A	0.626377	5.87E-09	166.77	S	SPKFPYTRRRNPSCGSDNDSVQPVRRRKAHN	0	271400	5875
Q9UQ35	875	Serine/arginine repetitive matrix protein 2	SRRM2	1	4.15E-09	166.7	S	ANEQSVTPQRRSCFESSPDPELKSRTPSRHS	4525200	6118600	6682
Q01518	308	Adenylyl cyclase-associated protein 1	CAP1	0.982439	1.59E-07	166.69	S	VRSGPKPFSAPKPQTSPSPKRATKKEPAVLE	11021000	26075000	2069
Q6ZN18	206	Zinc finger protein AEBP2	AEBP2	1	2.43E-08	166.67	S	TGGGGSSATSGGRRGSLEMSSDGEPLSRMDS	804740	518990	3651
Q86X29	493	Lipolysis-stimulated lipoprotein receptor	LSR	1	1.81E-09	166.67	S	EQAGGGWRARRPRARSVDALDDLTPPSTAES	2679400	2186200	4009
P78536	791	Disintegrin and metalloproteinase domain-containing protein	ADAM17	1	4.18E-09	166.62	S	GFEKDPFPNSSTAAKSFEDLTDHPVTRSEKA	3266700	0	1971
Q71RC2	586	La-related protein 4	LARP4	0.418761	7.91E-09	166.62	S	QKDGLNQTTIPVSPPSTTKPSRASTASPCNN	0	0	3697
Q9UQ35	2449	Serine/arginine repetitive matrix protein 2	SRRM2	0.999993	4.18E-09	166.62	S	APSQSLLPPAQDQPRSPVPSAFSDQSRCLIA	56268000	42605000	6704
Q71RC2	588	La-related protein 4	LARP4	0.581248	7.91E-09	166.62	T	DGLNQTTIPVSPPSTTKPSRASTASPCNNNI	3851100	4345900	7787
Q2NKX8	946	DNA excision repair protein ERCC-6-like	ERCC6L	0.942345	1.14E-17	166.6	S	AQASEAKLEEEPSASSPQYACDFNLFLEDSA	1785000	2958400	3046
P35612	697	Beta-adducin	ADD2	0.998932	1.98E-12	166.57	S	DKTESVTSGPMSPEGSPSKSPSKKKKKFRTP	4527800	3108000	1405
P35612	701	Beta-adducin	ADD2	0.993834	1.98E-12	166.57	S	SVTSGPMSPEGSPSKSPSKKKKKFRTPSFLK	7105300	1306000	1407
P52948	934	Nuclear pore complex protein Nup98-Nup96;Nuclear pore comple	NUP98	1	3.96E-09	166.52	S	QMALNGKPAPPPQSQSPEVEQLGRVVELDSD	8263700	5219600	1828
Q7Z5L9	360	Interferon regulatory factor 2-binding protein 2	IRF2BP2	1	1.74E-08	166.52	S	NGSKAVARTARKRKPSPEPEGEVGPPKINGE	6701300	4320800	3850
P85037	436	Forkhead box protein K1	FOXK1	1	3.27E-08	166.52	T	PTHPGLMSPRSGGLQTPECLSREGSPIPHDP	4715300	4750600	7466
P46100	1991	Transcriptional regulator ATRX	ATRX	0.462899	6.44E-09	166.48	S	NPSVSLKLEESKATSSSNPSSPAPDWYKDFV	0	0	1547
O94842	176	TOX high mobility group box family member 4	TOX4	0.797947	1.86E-09	166.47	T	TILPPAQSPEDRLSTTPSPTSSLHEDGVEDF	0	9152200	7230
P14618	37	Pyruvate kinase PKM	PKM	1	1.41E-08	166.45	S	MADTFLEHMCRLDIDSPPITARNTGIICTIG	4612900	5161700	1061
Q6KC79	280	Nipped-B-like protein	NIPBL	0.986405	7.76E-09	166.45	S	RNAASFPLRSPQPVCSPAGSEGTPKGSRPPL	755190	566910	3489
Q9Y6M1	164	Insulin-like growth factor 2 mRNA-binding protein 2	IGF2BP2	0.999769	8.01E-09	166.45	S	SFKISYIPDEEVSSPSPPQRAQRGDHSSREQ	2205800	2280500	7015
Q96RY5	1201	Protein cramped-like	CRAMP1L	0.999975	1.50E-13	166.38	S	NSGTSLLGPSLLDGNSRDSFVSRSLADVAEV	1016500	857810	5107
Q9H2Y7	1370	Zinc finger protein 106	ZNF106	0.998007	4.86E-09	166.38	S	LRKKKSLRAAHVPENSDTEQDVLTVKPVRKV	1518200	981630	5623
Q76L83	137	Putative Polycomb group protein ASXL2	ASXL2	0.671354	1.86E-09	166.36	S	SNKEGKKSRWKRKVSSSSPQSGCPSPTIPAG	0	939530	3706
P35226	251	Polycomb complex protein BMI-1	BMI1	1	0.00180847	166.34	S	ISHQRDGLTNAGELESDSGSDKANSPAGGIP	0	0	1380
P35226	253	Polycomb complex protein BMI-1	BMI1	1	0.00180847	166.34	S	HQRDGLTNAGELESDSGSDKANSPAGGIPST	0	0	1381
P35226	255	Polycomb complex protein BMI-1	BMI1	1	0.00180847	166.34	S	RDGLTNAGELESDSGSDKANSPAGGIPSTSS	0	0	1382
Q15642	296	Cdc42-interacting protein 4	TRIP10	0.999763	2.21E-08	166.3	S	DVEFEDFSQPMNRAPSDSSLGTPSDGRPELR	15293000	13216000	2956
Q9NV70	470	Exocyst complex component 1	EXOC1	0.971918	1.37E-08	166.24	S	QETESLHGSSGKLTGSTSSLNKLSVQSSGNR	6340000	6462100	6028
Q9NV70	473	Exocyst complex component 1	EXOC1	0.974486	1.37E-08	166.24	S	ESLHGSSGKLTGSTSSLNKLSVQSSGNRRSQ	0	4322900	6030
O15042	485	U2 snRNP-associated SURP motif-containing protein	U2SURP	0.970012	4.32E-09	166.14	S	HVYYRWKLYSILQGDSPTKWRTEDFRMFKNG	1514700	1374100	263
Q4KWH8	1310	1-phosphatidylinositol 4,5-bisphosphate phosphodiesterase et	PLCH1	0.499974	7.93E-09	166.14	T	AKTAALESNLPGSPNTSRGWLPKSPTKGEDW	0	0	7682
Q0JRZ9	403	F-BAR domain only protein 2	FCHO2	1	2.11E-08	166.11	S	PAISRHSPVQMNRNLSNEELTKSKPSAPPNE	711400	683840	2286
Q12888	580	Tumor suppressor p53-binding protein 1	TP53BP1	1	2.41E-17	165.96	S	DSILMNPAQDGEVQLSQNDDKTKGDDTDTRD	19590000	17737000	2341
Q14839	1540	Chromodomain-helicase-DNA-binding protein 4	CHD4	0.798729	2.35E-13	165.9	T	EENKKMSQPGSPSPKTPTPSTPGDTQPNTPA	813230	8596100	7620
Q14839	1542	Chromodomain-helicase-DNA-binding protein 4	CHD4	0.677417	2.35E-13	165.9	T	NKKMSQPGSPSPKTPTPSTPGDTQPNTPAPV	2179100	2345100	7621
P07197	628	Neurofilament medium polypeptide	NEFM	1	2.54E-08	165.89	S	AKSPVPKSPVEEKGKSPVPKSPVEEKGKSPV	335150	1047800	929
P07197	633	Neurofilament medium polypeptide	NEFM	1	2.54E-08	165.89	S	PKSPVEEKGKSPVPKSPVEEKGKSPVPKSPV	335150	216270	930
P49368	252	T-complex protein 1 subunit gamma	CCT3	0.999982	1.81E-08	165.89	S	RIVLLDSSLEYKKGESQTDIEITREEDFTRI	0	0	1657
P35659	306	Protein DEK	DEK	0.89808	5.21E-09	165.86	S	KKNQNSSKKESESEDSSDDEPLIKKLKKPPT	0	147700	1422
P35659	307	Protein DEK	DEK	0.94271	5.21E-09	165.86	S	KNQNSSKKESESEDSSDDEPLIKKLKKPPTD	0	147700	1423
Q99700	771	Ataxin-2	ATXN2	0.499898	8.26E-07	165.86	T	QNSPAGNKENIKPNETSPSFSKAENKGISPV	0	0	8030
O43847	94	Nardilysin	NRD1	0.998882	2.70E-09	165.78	S	ARLGADESEEEGRRGSLSNAGDPEIVKSPSD	4026200	2625300	432
Q9HAW4	762	Claspin	CLSPN	0.999977	4.43E-09	165.78	S	DENSGKQPSKLDEDDSCSLLTKESSHNSSFE	0	909280	5826
O95425	261	Supervillin	SVIL	0.95522	3.73E-09	165.66	S	PKHAHSSSLQQAASRSPSFGDPQLSPEARPS	1571300	587120	767
O95425	270	Supervillin	SVIL	1	3.73E-09	165.66	S	QQAASRSPSFGDPQLSPEARPSTGKPKHEWF	1571300	587120	768
Q02880	1526	DNA topoisomerase 2-beta	TOP2B	1	8.46E-09	165.66	S	PKQKKVVEAVNSDSDSEFGIPKKTTTPKGKG	0	1400500	2110
Q66K14	435	TBC1 domain family member 9B	TBC1D9B	0.984532	1.38E-23	165.66	S	SPAPQEGSEQPASPASPLSSRQSFCAQEAPT	9835600	5717400	3401
Q8N1G0	1191	Zinc finger protein 687	ZNF687	0.999673	4.01E-10	165.66	S	EEEAPPSRSDPDGGDSPLPASGGPLTCKVCG	619550	302250	4233
Q7L2J0	69	7SK snRNA methylphosphate capping enzyme	MEPCE	1	1.96E-09	165.58	S	CAPSAGSPAAAVGRESPGAAATSSSGPQAQQ	12407000	4085000	3725
Q8WX92	557	Negative elongation factor B	NELFB	0.999994	4.04E-13	165.53	S	SQLGEKLEQLDHRKPSPAQAAETPALELPLP	8982300	5544500	4574
P28715	563	DNA repair protein complementing XP-G cells	ERCC5	0.999838	2.75E-09	165.51	S	ELCPYESKFDSSLLSSDDETKCKPNSASEVI	1995600	1145800	1311
Q99613;B	39;39	Eukaryotic translation initiation factor 3 subunit C;Eukaryo	EIF3C;EIF3C	1	2.75E-09	165.49	S	TKPVGGNYGKQPLLLSEDEEDTKRVVRSAKD	7736200	3789600	73
Q14157	470	Ubiquitin-associated protein 2-like	UBAP2L	0.847351	1.60E-13	165.49	S	PLPSKSTSAPQMSPGSSDNQSSSPQPAQQKL	2035800	0	2670
Q86VR2	433	Protein FAM134C	FAM134C	0.333254	1.10E-23	165.47	S	PSGAPAQRATRGFLRSPSSDLDTDAEGDDFE	0	1748400	3967
Q86VR2	435	Protein FAM134C	FAM134C	0.620463	1.10E-23	165.47	S	GAPAQRATRGFLRSPSSDLDTDAEGDDFELL	0	1748400	3968
Q86VR2	436	Protein FAM134C	FAM134C	0.606016	1.10E-23	165.47	S	APAQRATRGFLRSPSSDLDTDAEGDDFELLD	0	1748400	3969
Q9UPU5	2565	Ubiquitin carboxyl-terminal hydrolase 24	USP24	0.974458	4.10E-09	165.24	T	TSTGKTFQRTISAQDTLAYATALLNEKEQSG	0	665160	8252
O60784	462	Target of Myb protein 1	TOM1	0.927411	1.04E-10	165.03	S	LEERAKAADRLPNLSSPSAEGPPGPPSGPAP	957480	1124100	522
O75533	259	Splicing factor 3B subunit 1	SF3B1	0.814161	4.15E-08	165.03	S	TPGATPGSKIWDPTPSHTPAGAATPGRGDTP	5669600	0	639
O75533	267	Splicing factor 3B subunit 1	SF3B1	0.999999	1.40E-08	165.03	T	KIWDPTPSHTPAGAATPGRGDTPGHATPGHG	1226100	816880	7211
P20290	158	Transcription factor BTF3	BTF3	0.892982	5.18E-11	165.01	S	LTEMLPSILNQLGADSLTSLRRLAEALPKQS	3372600	1456300	1182
P16402	2	Histone H1.3	HIST1H1D	0.5	3.66E-09	164.93	S	______________MSETAPLAPTIPAPAEK	2939900	0	1090
Q5JSL3	440	Dedicator of cytokinesis protein 11	DOCK11	0.999771	8.88E-09	164.93	S	REMLWGSSTQLASDGSPKGSSPESYIHGIAE	85128	584280	3187
Q5T200	370	Zinc finger CCCH domain-containing protein 13	ZC3H13	0.99944	2.31E-05	164.93	S	PSPSYQRTLTPPLRRSASPYPSHSLSSPQRK	2947300	0	3265
Q5T200	381	Zinc finger CCCH domain-containing protein 13	ZC3H13	0.937815	1.29E-05	164.93	S	PLRRSASPYPSHSLSSPQRKQSPPRHRSPMR	2947300	1060400	3268
Q6ICG6	324	Uncharacterized protein KIAA0930	KIAA0930	0.999973	4.69E-09	164.93	S	RKVPRNRIAEMKKSHSANDSEEFFREDDGGA	1687400	595810	3471
Q99590	533	Protein SCAF11	SCAF11	0.902083	2.85E-09	164.93	S	KGGDPLEKQDQISGLSQSEVKTDVCTVHLPN	182610	0	5221
P16402	4	Histone H1.3	HIST1H1D	0.801372	3.66E-09	164.93	T	____________MSETAPLAPTIPAPAEKTP	2939900	2339700	7291
O95817	291	BAG family molecular chaperone regulator 3	BAG3	0.999999	3.65E-08	164.92	S	REGSPARSSTPLHSPSPIRVHTVVDRPQQPM	1338200	1215500	828
Q07666	20	KH domain-containing, RNA-binding, signal transduction-assoc	KHDRBS1	0.957161	4.72E-09	164.92	S	DDPAARMSRSSGRSGSMDPSGAHPSVRQTPS	10297000	3834200	2230
Q9NQQ7	335	Solute carrier family 35 member C2	SLC35C2	0.846723	2.86E-09	164.92	S	HSRGDGGPKALKGLGSSPDLELLLRSSQREE	429320	335740	5918
O95817	285	BAG family molecular chaperone regulator 3	BAG3	0.924179	3.65E-08	164.92	T	VQGASSREGSPARSSTPLHSPSPIRVHTVVD	0	3453200	7247
P85037	213	Forkhead box protein K1	FOXK1	1	7.88E-09	164.89	S	KIQFTSLYHKEEAPASPLRPLYPQISPLKIH	26592000	18661000	1993
P85037	223	Forkhead box protein K1	FOXK1	1	7.88E-09	164.89	S	EEAPASPLRPLYPQISPLKIHIPEPDLRSMV	25081000	17475000	1994
Q9Y4F1	872	FERM, RhoGEF and pleckstrin domain-containing protein 1	FARP1	0.981648	8.95E-09	164.82	S	LAEKSSSPAPEFLASSPPDNKSPDEATAADQ	1720700	739350	6911
P17812	573	CTP synthase 1	CTPS1	0.918688	3.95E-09	164.81	S	GCRLSPRDTYSDRSGSSSPDSEITELKFPSI	1154100	3171200	1121
Q96G61;Q	148;148	Diphosphoinositol polyphosphate phosphohydrolase 3-beta;Dipl	NUDT11;NUD	0.979716	4.48E-15	164.8	S	KPVHAEYLEKLKLGGSPTNGNSMAPSSPDSD	1931700	1156900	4399
Q96J02	240	E3 ubiquitin-protein ligase Itchy homolog	ITCH	0.999618	6.77E-09	164.7	S	EDAGAGENRRVSGNNSPSLSNGGFKPSRPPR	3241300	2006700	4954
Q96QC0	382	Serine/threonine-protein phosphatase 1 regulatory subunit 1(	PPP1R10	1	2.90E-09	164.7	S	TASLEPGALDAKPVESPGDPNQLTRKGRKRK	8327200	10323000	5077
Q9Y4H2	1176	Insulin receptor substrate 2	IRS2	0.99988	4.76E-09	164.7	S	VSPSFAHNPKRHNSASVENVSLRKSSEGGVG	0	517600	6924
O95714;Q	1577;304	E3 ubiquitin-protein ligase HERC2;Putative HERC2-like protei	HERC2;HERC2	1	4.48E-13	164.69	S	KPESTDDEEKIGNEESDLEEACILPHSPINV	2843400	1712000	800
O95714;Q	1588;315	E3 ubiquitin-protein ligase HERC2;Putative HERC2-like protei	HERC2;HERC2	1	4.48E-13	164.69	S	GNEESDLEEACILPHSPINVDKRPIAIKSPK	3759100	2443900	801
P12270	2037	Nucleoprotein TPR	TPR	0.961639	1.68E-13	164.69	S	MGGGEGNHRAADSQNSGEGNTGAAESSFSQE	0	0	1035
P12270	2042	Nucleoprotein TPR	TPR	0.50253	1.68E-13	164.69	T	GNHRAADSQNSGEGNTGAAESSFSQEVSREQ	0	0	7280
Q99618	68	Cell division cycle-associated protein 3	CDCA3	0.999401	1.82E-07	164.68	S	EQLEGLKHAQDSDPRSPTLGIARTPMKTSSG	2546700	1828800	5233
Q6P5R6	118	60S ribosomal protein L22-like 1	RPL22L1	0.99313	0.000134565	164.66	S	TYELRYFQISQDEDESESED__________	1565900	1630800	3531
Q99543	47	DnaJ homolog subfamily C member 2;DnaJ homolog subfamily C m	DNAJC2	1	3.98E-09	164.63	S	GRWFEAFVKRRNRNASASFQELEDKKELSEE	11558000	7288600	5202
Q99543	49	DnaJ homolog subfamily C member 2;DnaJ homolog subfamily C m	DNAJC2	1	3.98E-09	164.63	S	WFEAFVKRRNRNASASFQELEDKKELSEESE	3887500	4164700	5203
O94888	288	UBX domain-containing protein 7	UBXN7	0.971866	5.49E-09	164.58	S	KKCARSESLIDASEDSQLEAAIRASLQETHF	405760	378000	716
P54760	769	Ephrin type-B receptor 4	EPHB4	0.909749	5.50E-09	164.56	S	CKVSDFGLSRFLEENSSDPTYTSSLGGKIPI	1292700	785320	1856
P46100	1527	Transcriptional regulator ATRX	ATRX	0.814201	1.02E-10	164.53	S	REREKLREVIEIEDASPTKCPITTKLVLDED	888290	835040	1551
Q15678	312	Tyrosine-protein phosphatase non-receptor type 14	PTPN14	0.807251	2.93E-09	164.53	S	TRHKFYKQNKICTEQSNSPPPIRRQPTWSRS	0	0	2973
Q96Q15	3570	Serine/threonine-protein kinase SMG1	SMG1	0.973741	4.29E-08	164.5	S	MSQNARKLIQKNLATSADTPPSTVPGTGKSV	4298600	2906700	5065
Q9Y6N7	1442	Roundabout homolog 1	ROBO1	0.595659	4.59E-09	164.5	S	AGRRHFHASQCPRPTSPVSTDSNMSAAVMQK	2730300	697800	7016
Q9Y6N7	1445	Roundabout homolog 1	ROBO1	0.825241	4.59E-09	164.5	S	RHFHASQCPRPTSPVSTDSNMSAAVMQKTRP	1482700	0	7017
Q96Q15	3573	Serine/threonine-protein kinase SMG1	SMG1	0.96945	4.29E-08	164.5	T	NARKLIQKNLATSADTPPSTVPGTGKSVACS	3410200	2401900	7997
P23528	3	Cofilin-1	CFL1	1	2.06E-09	164.48	S	_____________MASGVAVSDGVIKVFNDM	1473300	1261700	1225
Q9NTJ3	22	Structural maintenance of chromosomes protein 4	SMC4	1	4.93E-13	164.46	S	QPSTARRREEGPPPPSPDGASSDAEPEPPSG	9533300	8388800	6014

Q8IYB3	597	Serine/arginine repetitive matrix protein 1	SRRM1	0.999997	7.65E-07	164.33	S	PSPPPRRRSPSPRRYSPPIQRRYSPSPPPKR	3179700	1579200	4165
Q9BXW6	499	Oxysterol-binding protein-related protein 1	OSBPL1A	1	4.91E-09	164.22	S	ESERSLSRLEAVTARSFEEEGEHLGSRKHRM	771000	1013000	5462
Q96AY4	2398	Tetratricopeptide repeat protein 28	TTC28	1	1.64E-08	164.2	S	GTMTSKRDVLSLLNLSPRHNKKEEGVDKLEL	827110	342930	4781
Q69YN4	138	Protein virilizer homolog	KIAA1429	0.999999	6.79E-14	164.19	S	AIYGSVDRVISHDRDSPPPPPPPPPPPQPQP	381850	221240	3433
Q01433	190	AMP deaminase 2	AMPD2	0.998087	1.65E-08	164.13	S	PDILLRAKQDFLKTDSDSDLQLYKEQGEGQG	751640	348790	2063
Q6PKG0	90	La-related protein 1	LARP1	1	6.34E-31	164.03	S	SPRPLQLPGAEGPAISDGEEGGGEPGAGGGA	29670000	17842000	3557
Q5T1M5	960	FK506-binding protein 15	FKBP15	0.673554	2.12E-10	164.01	S	EEKAEERPRRPSQEQSASASSGQPQAPLNRE	14861000	0	3252
Q96H79	257	Zinc finger CCCH-type antiviral protein 1-like	ZC3HAV1L	0.830224	4.46E-10	164.01	S	KHMKLHKMLENTDNSSPSTEHSQGLEKQGVH	900660	917220	4936
Q9C0D5	1668	Protein TANC1	TANC1	0.977886	2.17E-09	164.01	S	DVRHPASLTSSGSSGSPSSSIKMSSSTSSLT	0	628590	5540
Q9Y2U8	140	Inner nuclear membrane protein Man1	LEMD3	0.98939	7.82E-09	164.01	S	ASAAPAAGSKVLLGFSSDESDVEASPRDQAG	890210	552670	6808
Q9Y2U8	141	Inner nuclear membrane protein Man1	LEMD3	0.995288	7.82E-09	164.01	S	SAAPAAGSKVLLGFSSDESDVEASPRDQAGG	890210	552670	6809
Q9UGU5	502	HMG domain-containing protein 4	HMGXB4	0.999943	5.00E-09	163.99	S	KVKASSVGVLSPQKKSPPTTMLLPASPAKAP	1368400	851180	6319
Q9BX40	165	Protein LSM14 homolog B	LSM14B	0.977184	2.17E-09	163.96	S	PVGKSPMVEQAVQTGSADNLNAKKLLPGKGT	510930	406130	5441
O75533	261	Splicing factor 3B subunit 1	SF3B1	0.981676	1.40E-08	163.96	T	GATPGSKIWDPTPSHTPAGAATPGRGDTPGH	0	3397100	7210
Q86X27	308	Ras-specific guanine nucleotide-releasing factor RalGPS2	RALGPS2	0.986355	9.17E-09	163.91	S	SAASREDLVGPEVGASPQSGRKSVAAEGALL	7481100	5196000	4001
Q8N5G2	305	Macoilin	TMEM57	0.80529	5.03E-09	163.91	S	ENHINSKRLNNDLVGSTENLLKEDSCTASSK	870390	1125100	4284
Q8ND30	387	Liprin-beta-2	PPFIBP2	1	2.43E-08	163.9	S	PQKSLETRAQKKLSCSLEDLRSESVDKCMDG	755310	350160	4338
P53985	466	Monocarboxylate transporter 1	SLC16A1	0.610629	1.77E-06	163.9	T	EQKANEQKKESKEEETSIDVAGKPNEVTKAA	2452800	0	7446
O15541	85	RING finger protein 113A	RNF113A	0.997691	5.12E-13	163.85	S	TRDSGKQKAAYGDLSSEEEEENEPESLGVVY	26432000	21493000	327
Q09666	177	Neuroblast differentiation-associated protein AHNAK	AHNAK	0.993661	1.93E-08	163.84	S	VDVTGREGAKDIDISSPEFKIKIPRHELTEI	1895400	1172700	2275
Q9UQR1	306	Zinc finger protein 148	ZNF148	0.772931	2.27E-12	163.8	S	KGGLLTSEEDSGFSTSPKDNSLPKKKRQKTE	1158000	1076300	6766
Q13330	522	Metastasis-associated protein MTA1	MTA1	0.999137	5.09E-09	163.62	S	AIKAECTARLPEASQSPLVLKQAVRKPLEAV	1505200	1247000	2474
Q15642	299	Cdc42-interacting protein 4	TRIP10	0.96585	2.25E-08	163.56	S	FEDFSQPMNRAPSDSSLGTPSDGRPELRGPG	2754400	2851500	2957
P55081	53	Microfibrillar-associated protein 1	MFAP1	0.996949	2.18E-10	163.53	S	RYVSGKRPDYAPMESSDEEDEEFQFIKKAKE	0	1421800	1860
Q8N201	1326	Integrator complex subunit 1	INTS1	0.5	1.71E-08	163.45	S	SLPPRRDSTEAPKPKSSPEQPIGQGRIRVGT	1415100	1403600	4239
Q8N201	1327	Integrator complex subunit 1	INTS1	0.5	1.71E-08	163.45	S	LPPRRDSTEAPKPKSSPEQPIGQGRIRVGTQ	1415100	1403600	4240
Q96E09	62	Protein FAM122A	FAM122A	0.999189	1.03E-07	163.45	S	TSPVFQAEAPSARRNSTTFPSRHGLLLPASP	3635800	1527900	4870
P27816	14	Microtubule-associated protein 4	MAP4	0.999998	1.23E-10	163.44	S	__MADLSLADALTEPSPDIEGEIKRDFIATL	219720	444170	1288
O43432	1409	Eukaryotic translation initiation factor 4 gamma 3	EIF4G3	0.959594	2.10E-12	163.34	S	HNFLLEQKLDFIESDSPCSSEALSKKELSAE	1144400	987970	384
Q6ZN17	203	Protein lin-28 homolog B	LIN28B	0.943604	9.81E-09	163.34	S	TSTLPREVGGGHGCTSPPFPQEARAEISERS	5671100	3877500	3648
Q9Y3S1	1150	Serine/threonine-protein kinase WNK2	WNK2	0.961442	1.42E-07	162.93	S	CESYGGSDVTSGKELSDSCEGAFGGGRLEGR	1432900	1000800	6868
Q9Y520	2105	Protein PRRC2C	PRRC2C	1	3.30E-07	162.93	S	QPRAGPIKAQKLPDLSPVENKEHKPGPIGKE	6614000	3118500	6945
O75821	42	Eukaryotic translation initiation factor 3 subunit G	EIF3G	0.994842	2.44E-11	162.92	S	TSELLKGIPLATGDTSPEPELLPGAPLPPPK	15918000	16606000	664
Q6F5E8	1321	Leucine-rich repeat-containing protein 16C	RLTPR	0.991201	8.58E-09	162.82	S	GQQDGPGPPSPGQSPSPCRTSPSPDSLGLPE	3655500	2401700	3455
Q9C0B5	694	Palmitoyltransferase ZDHHC5	ZDHHC5	0.965998	8.87E-08	162.81	S	SLGSASPGPGQPPLSSPTRGGVKKVSGVGGT	0	733060	5515
Q8WWI1	988	LIM domain only protein 7	LMO7	0.997524	2.21E-07	162.77	S	QKEVAATEEDVTRLPSPTSPFSSLSQDQAAT	1223200	961170	4553
Q8WWI1	990	LIM domain only protein 7	LMO7	0.652433	2.21E-07	162.77	T	EVAATEEDVTRLPSPTSPFSSLSQDQAATSK	1223200	961170	7921
P04075	46	Fructose-bisphosphate aldolase A	ALDOA	1	1.33E-07	162.76	S	LAADESTGSIAKRLQSIGTENTEENRRFYRQ	1488500	2397800	875
Q4VCS5	309	Angiomotin	AMOT	0.679058	4.09E-15	162.73	S	QDISLPLSARNSQPHSPTSSLTSGGSLPLLQ	0	0	3115
Q76FK4	365	Nucleolar protein 8	NOL8	1	1.86E-07	162.72	S	SLIGLGIKNRVSCHDSDDDIMRNDREYDSGD	979850	1091200	3701
P85037	257	Forkhead box protein K1	FOXK1	0.999794	6.50E-11	162.71	S	PSPTGTISVPNSCPASPRGAGSSSYRFVQNV	6985600	4036000	2005
P85037	247	Forkhead box protein K1	FOXK1	0.957562	4.86E-10	162.71	T	PDLRSMVSPVPSPTGTISVPNSCPASPRGAG	5355800	2378500	7468
P09651	4	Heterogeneous nuclear ribonucleoprotein A1;Heterogeneous nuc	HNRNPA1	1	1.10E-07	162.7	S	____________MSKSESPKEPEQLRKLFIG	0	0	970
P09651	6	Heterogeneous nuclear ribonucleoprotein A1;Heterogeneous nuc	HNRNPA1	1	1.10E-07	162.7	S	__________MSKSESPKEPEQLRKLFIGGL	6647900	8916700	971
Q14186	23	Transcription factor Dp-1	TFDP1	1	5.40E-07	162.67	S	IEANGELKVFIDQNLSPGKGVVSLVAVHPST	6972900	2939400	2697
Q9Y4E1	539	WASH complex subunit FAM21C	FAM21C	1	8.48E-09	162.65	S	NLKPSSETKTQKGLFSDEEDSEDLFSSQSAS	670620	650880	6908
P35222	552	Catenin beta-1	CTNNB1	0.499976	1.32E-06	162.64	S	VQLLVRAHQDTQRRTSMGGTQQQFVEGVRME	6147500	0	1379
Q9H910	97	Hematological and neurological expressed 1-like protein	HN1L	0.99966	2.33E-07	162.64	S	QHLNPPGGKTSDIFGSPVTATSRLAHPNKPK	1688400	1200000	5798
P35222	551	Catenin beta-1	CTNNB1	0.682098	1.32E-06	162.64	T	LVQLLVRAHQDTQRRTSMGGTQQQFVEGVRM	6147500	3343000	7359
Q7Z2W4	284	Zinc finger CCCH-type antiviral protein 1	ZC3HAV1	1	1.95E-07	162.48	S	ERSCTPSPDQISHRASLEDAPVDDLTRKFTY	2891400	3026500	3750
Q9BZI7	169	Regulator of nonsense transcripts 3B	UPF3B	0.999998	2.77E-06	162.48	T	IDDDPEYRKFLESYATDNEKMTSTPETLLEE	1778200	543410	8070
Q92538	1298	Golgi-specific brefeldin A-resistance guanine nucleotide exc	GBF1	0.999961	1.58E-11	162.43	S	LQATARADAPDAGAQSDSELPSYHQNDVSLD	3393900	1735000	4625
Q12789	1854	General transcription factor 3C polypeptide 1	GTF3C1	0.641935	6.42E-11	162.42	S	GSSSEDSPPEGQAPPSHSPRGTKRRASWASE	848800	6207300	2301
Q12789	1856	General transcription factor 3C polypeptide 1	GTF3C1	0.993779	6.42E-11	162.42	S	SSEDSPPEGQAPPSHSPRGTKRRASWASENG	9983700	5281900	2302
O60832	458	H/ACA ribonucleoprotein complex subunit 4	DKC1	0.968209	1.66E-07	162.42	T	KTAKRKRESESESDETPPAAPQLIKKEKKKS	0	165870	7191
Q5TCQ9	832	Membrane-associated guanylate kinase, WW and PDZ domain-cont	MAGI3	0.999985	1.65E-07	162.32	S	PEDDSSQAFISTQNGSPRLNRAEVPARPAPQ	578630	414470	3297
O15258	2	Protein RER1	RER1	1	9.96E-08	162.31	S	______________MSEGDSVGESVHGKPSV	0	309020	309
Q9H2Y7	861	Zinc finger protein 106	ZNF106	0.999749	8.77E-07	162.22	S	VSISSSPGLARKRSLSESSVIMDRAPSVYSF	1319500	756940	5629
P12931	17	Proto-oncogene tyrosine-protein kinase Src	SRC	1	1.11E-10	162.2	S	GSNKSKPKDASQRRRSLEPAENVHGAGGGAF	1474300	715760	1045
P85037	243	Forkhead box protein K1	FOXK1	0.997897	6.50E-11	162.09	S	HIPEPDLRSMVSPVPSPTGTISVPNSCPASP	3151200	906050	2003
Q14671	229	Pumilio homolog 1	PUM1	0.998953	3.51E-11	162.09	S	SGGLGVSMVEYVLSSSPGDSCLRKGGFGPRD	1655700	614080	2757
Q9UIF9	1397	Bromodomain adjacent to zinc finger domain protein 2A	BAZ2A	0.970088	5.17E-07	161.99	S	LAPKRRAGDPGEMPQSPTGLGQPKRRGRPPS	1644100	1340200	6379
Q96E09	143	Protein FAM122A	FAM122A	0.999999	3.80E-07	161.94	S	VEKSASPKRIDFIPVSPAPSPTRGIGKQCFS	11824000	13071000	4868
Q96E09	147	Protein FAM122A	FAM122A	0.981068	3.80E-07	161.94	S	ASPKRIDFIPVSPAPSPTRGIGKQCFSPSLQ	11824000	13071000	4869
Q96B36	183	Proline-rich AKT1 substrate 1	AKT1S1	1	4.12E-07	161.93	S	SVPPASALPTQQYAKSLPVSVPVWGFKEKRT	16065000	11827000	4798
Q16799	352	Reticulon-1	RTN1	0.991222	3.30E-07	161.87	S	GTEPSAAESQGKGSISEDELITAIKEAKGLS	406720	325540	3030
Q9BZ23	189	Pantothenate kinase 2, mitochondrial	PANK2	0.999992	4.29E-07	161.87	S	AVGASAEGTRRDRLGSYSGPTSVSRQRVESL	3300800	2120200	5482
Q9UQ35	2132	Serine/arginine repetitive matrix protein 2	SRRM2	1	4.29E-07	161.87	S	FSRPSMSPTPLDRCRSPGMLEPLGSSRTPMS	25751000	28137000	6605
P24928	1905	DNA-directed RNA polymerase II subunit RPB1	POLR2A	0.56903	1.78E-07	161.87	T	PTSPTYSPTSPVYTPTSPKYSPTSPTYSPTS	2256300	736760	7323
Q04727	292	Transducin-like enhancer protein 4	TLE4	0.999999	2.55E-07	161.75	S	NGLDKTRLLKKDAPISPASIASSSSTPSSKS	2767400	1517400	2178
Q92922	330	SWI/SNF complex subunit SMARCC1	SMARCC1	1	3.36E-07	161.75	S	DRKASANARKRKHSPSPPPPTPTESRKKSGK	63240000	36397000	4729
Q96T58	2481	Msx2-interacting protein	SPEN	0.999999	5.55E-07	161.59	S	SIPIPTLPSVTAAKLSPPVASGGIPHQSPPT	7927000	4185300	5174
Q96T58	2493	Msx2-interacting protein	SPEN	0.999998	5.55E-07	161.59	S	AKLSPPVASGGIPHQSPPTKVTEWITRQEEP	2809300	1568100	5175
Q9Y3Z3	592	Deoxynucleoside triphosphate triphosphohydrolase SAMHD1	SAMHD1	1	5.23E-05	161.59	T	NFTKPQDGDVIAPLITPQKKEWNDSTSVQNP	1625800	1099300	8297
Q9NYZ3	592	G2 and S phase-expressed protein 1	GTSE1	0.999812	6.82E-07	161.55	S	DRGSPPSRVPQALNFSPEESDSTFSKSTATE	841060	982660	6146
Q9UFW8	164	CGG triplet repeat-binding protein 1	CGGBP1	1	2.86E-07	161.55	S	AYLPDGYENENQLLNSQDC____________	1096100	709190	6313
Q12802	2728	A-kinase anchor protein 13	AKAP13	0.995738	4.13E-07	161.49	S	PSIAKSGSLDSELSVSPKRNSISRTHKDKGP	0	0	2311
P49321	464	Nuclear autoantigenic sperm protein	NASP	1	2.19E-06	161.49	T	EKSPEDKVQIAANEETQEREEQMKEGEETEG	0	0	7410
Q8NFC6	2905	Biorientation of chromosomes in cell division protein 1-like	BOD1L1	0.920714	1.10E-06	161.48	S	KDDSKTDTGIVTVEQSPSSSKLKVMQTDESN	0	0	4393
Q9Y6D5	1528	Brefeldin A-inhibited guanine nucleotide-exchange protein 2	ARFGEF2	0.999934	6.07E-07	161.48	S	SSIDKNPSERGQSQLSNPTDDSWKGRPYANQ	1842300	1349300	6994
Q3V6T2	1561	Girdin	CCDC88A	0.695353	2.44E-08	161.31	S	AGFRSKQLVNNKDTTSFEDISPQGVSDDSST	915670	710840	3077
Q9H6S0	1090	Probable ATP-dependent RNA helicase YTHDC2	YTHDC2	0.869301	1.23E-06	161.26	S	QEPSSFRVDGIPNDSSDSEMEDKTTANLAAL	844250	2467700	5739
P51858	165	Hepatoma-derived growth factor	HDGF	1	1.54E-06	161.25	S	EKGALKRRAGDLLEDSPKRPKEAENPEGEEK	11577000	20903000	1800
Q9UQ35	1731	Serine/arginine repetitive matrix protein 2	SRRM2	0.985102	6.04E-07	161.25	S	TRSRTPPRHRRSPSVSSPEPAEKSRSSRRRR	0	1105000	6702
O76021	361	Ribosomal L1 domain-containing protein 1	RSL1D1	0.95648	1.29E-06	161.24	S	KKRGRGKAQVKATNESEDEIPQLVPIGKKTP	11666000	5879700	674
P39687	27	Acidic leucine-rich nuclear phosphoprotein 32 family member	ANP32A	1	1.83E-06	161.21	S	RNRTPSDVKELVLDNSRSNEGKLEGLTDEFE	0	0	1460
P39687	29	Acidic leucine-rich nuclear phosphoprotein 32 family member	ANP32A	1	1.83E-06	161.21	S	RTPSDVKELVLDNSRSNEGKLEGLTDEFEEL	0	0	1461
Q6W2J9	423	BCL-6 corepressor	BCOR	0.999129	6.78E-06	161.21	S	GHARKTAVQDRKDGSSPPLLEKQTVTKDVTD	2256400	2108300	3611
Q13057	178	Bifunctional coenzyme A synthase;Phosphopantetheine adenylyl	COASY	1	1.59E-06	161.19	S	VEPLDVPLPSTIRPASPVAGSPKQPVRGYYR	2931400	2871400	2394
Q6IQ22	21	Ras-related protein Rab-12	RAB12	1	1.08E-07	161.1	S	ALQRRAGGGGGLGAGSPALSGGQGRRRKQPP	453840	666390	3482
Q13283	232	Ras GTPase-activating protein-binding protein 1	G3BP1	0.703943	4.27E-07	161.08	S	PVLEETAPEDAQKSSSPAPADIAQTVQEDLR	683360	479430	2465
P85037	239	Forkhead box protein K1	FOXK1	0.957344	8.11E-10	161.04	S	PLKIHIPEPDLRSMVSPVPSPTGTISVPNSC	3151200	906050	2002
Q8IY81	471	pre-rRNA processing protein FTSJ3	FTSJ3	0.99991	1.53E-15	161.04	S	YVSDVEDDGDDTSLDSDLDPEELAGVRGHQG	2365700	1662300	4122
P10636	437	Microtubule-associated protein tau	MAPT	0.790268	7.65E-15	160.98	S	PLIQPSSPAVCPEPPSSPKYVSSVTSRTGSS	6649100	3220400	994
P10636	438	Microtubule-associated protein tau	MAPT	0.500027	7.65E-15	160.98	S	LIQPSSPAVCPEPPSSPKYVSSVTSRTGSSG	6649100	3220400	995
Q8NDI1	428	EH domain-binding protein 1	EHBP1	0.87147	8.88E-06	160.95	S	VLNENTVSAGKDLSTSPKPSPIPSPVLGRKP	6581300	1912700	4352
Q8NDI1	432	EH domain-binding protein 1	EHBP1	0.999952	8.88E-06	160.95	S	NTVSAGKDLSTSPKPSPIPSPVLGRKPNASQ	6581300	6103100	4353
Q8NDI1	436	EH domain-binding protein 1	EHBP1	1	8.88E-06	160.95	S	AGKDLSTSPKPSPIPSPVLGRKPNASQSLLV	10295000	8015800	4354
O95785	1012	Protein Wiz	WIZ	0.999348	1.08E-06	160.86	S	PTPKNPEDKSPQLSLSPRPASPKAQWPQSED	2327100	960200	816
Q6UN15	494	Pre-mRNA 3'-end-processing factor FIP1	FIP1L1	0.930949	8.80E-07	160.84	T	RERTRERERERDHSPTPSVFNSDEERYRYRE	25618000	18313000	7771
P27816	825	Microtubule-associated protein 4	MAP4	0.999225	7.92E-06	160.82	S	TTTAAAVASTGPSSRSPSTLLPKKPTAIKTE	4326700	2120500	1297
O14639	450	Actin-binding LIM protein 1	ABLIM1	0.457521	8.26E-07	160.79	S	SAEGYQDVRDRMIHRSTSQGSINSPVYSRHS	0	0	161
O14639	451	Actin-binding LIM protein 1	ABLIM1	0.457521	8.26E-07	160.79	T	AEGYQDVRDRMIHRSTSQGSINSPVYSRHSY	0	0	7124
Q01664	124	Transcription factor AP-4	TFAP4	0.981389	1.98E-06	160.77	S	QNTQLKRFIQELSGSSPKRRRAEDKDEGIGS	6592300	4477500	2073
Q9H7N4	498	Splicing factor, arginine/serine-rich 19	SCAF1	1	3.42E-07	160.76	S	RRKILTQRRERYRQRSPSPAPAPAPAAAAGP	44299000	33642000	5771
Q9H7N4	500	Splicing factor, arginine/serine-rich 19	SCAF1	1	3.42E-07	160.76	S	KILTQRRERYRQRSPSPAPAPAPAAAAGPPT	44299000	33642000	5772
Q9H2U2	317	Inorganic pyrophosphatase 2, mitochondrial	PPA2	0.973279	7.48E-07	160.75	S	CTQEEARSLVESVSSSPNKESNEEEQVWHFL	22250000	10298000	5622
Q96JN0	37	Ligand-dependent corepressor	LCOR	0.957813	1.07E-07	160.67	S	PSQPNSTKNQSLPKASPVTTSPTAATTQNPV	0	0	4997
Q96JN0	42	Ligand-dependent corepressor	LCOR	0.969617	1.07E-07	160.67	S	STKNQSLPKASPVTTSPTAATTQNPVLSKLL	696440	452120	4998
Q92538	1789	Golgi-specific brefeldin A-resistance guanine nucleotide exc	GBF1	0.941482	1.70E-06	160.56	S	PRAASSSSPGSPVASSPSRLSPTPDGPPPLA	1548700	629610	4624
Q9BSJ6	129	Protein FAM64A	FAM64A	0.76646	8.03E-07	160.56	S	TQVKARRRKRGAQKGSGSPTHSLSQKSTRLS	0	0	5340
P28370	116	Probable global transcription activator SNF2L1	SMARCA1	0.996834	1.22E-06	160.53	S	QTELFAHFIQPSAQKSPTSPLNMKLGRPRIK	11456000	6685100	1308
Q5JSZ5	1395	Protein PRRC2B	PRRC2B	0.999999	3.91E-10	160.52	S	SSDFSERRERREGPGSEPDSQVDGGLSGASL	703880	624730	3189
Q9UQ35	1876	Serine/arginine repetitive matrix protein 2	SRRM2	1	5.93E-06	160.48	S	WKRSRSRASPATHRRSRSRTPLISRRRSRSR	20684000	16088000	6713
Q9UQ35	1878	Serine/arginine repetitive matrix protein 2	SRRM2	0.999992	5.93E-06	160.48	S	RSRSRASPATHRRSRSRTPLISRRRSRSRTS	36496000	26712000	6714
Q9UQ35	1884	Serine/arginine repetitive matrix protein 2	SRRM2	0.998779	5.93E-06	160.48	S	SPATHRRSRSRTPLISRRRSRSRTSPVSRRR	15811000	0	6715
Q7Z417	633	Nuclear fragile X mental retardation-interacting protein 2	NUFIP2	0.714334	2.69E-08	160.47	T	DYEIESQNPLASPTNTLLGSAKEQRYQRGLE	0	0	7804
Q7Z589	209	Protein EMSY	EMSY	0.998703	8.30E-07	160.46	S	CSDEDEKPRKRRRTNSSSSSPVVLKEVPKAV	1807900	1323600	3836
Q96P16	156	Regulation of nuclear pre-mRNA domain-containing protein 1A	RPRD1A	0.990628	3.03E-10	160.2	S	EQIKVDENENCSSLGSPSEPPQTLDLVRALQ	1852600	891700	5044
Q86X27	311	Ras-specific guanine nucleotide-releasing factor RalGPS2	RALGPS2	0.946651	1.94E-06	160.03	S	SREDLVGPEVGASPQSGRKSVAAEGALLPQT	0	0	4002
A0FGR8	755	Extended synaptotagmin-2	ESYT2	0.930905	5.82E-07	160.02	S	LLASPGHISVKEPTPSIASDISLPIATQELR	11611000	1634600	1
A0FGR8	758	Extended synaptotagmin-2	ESYT2	1	5.82E-07	160.02	S	SPGHISVKEPTPSIASDISLPIATQELRQRL	13099000	9054600	2
A0FGR8	761	Extended synaptotagmin-2	ESYT2	1	5.82E-07	160.02	S	HISVKEPTPSIASDISLPIATQELRQRLRQL	13951000	9807300	3
Q5VT52	665	Regulation of nuclear pre-mRNA domain-containing protein 2	RPRD2	0.569382	7.79E-07	159.83	S	QSSEVSKPKLESESTSPSLEMKIHNFLKGNP	0	0	3331
Q9NV70	499	Exocyst complex component 1	EXOC1	0.639658	2.12E-08	159.77	S	NRRSQSSSLLDMGNMSASDLDVADRTKFDKI	0	0	6031
Q6QNY0	65	Biogenesis of lysosome-related organelles complex 1 subunit	BLOC1S3	1	3.90E-09	159.72	S	RPTGLRVAGEAAETDSEPEPEPEPTAAPRDL	2662200	5075100	3577
Q9UGV2	361	Protein NDRG3	NDRG3	0.999997	2.15E-08	159.72	S	SVTSNQSDGTQESCESPDVLDRHQTMEVSC_	1767900	1841900	6322
Q6QNY0	63	Biogenesis of lysosome-related organelles complex 1 subunit	BLOC1S3	1	3.90E-09	159.72	T	RGRPTGLRVAGEAAETDSEPEPEPEPTAAPR	1312300	3260800	7769
O95453	557	Poly(A)-specific ribonuclease PARN	PARN	1	1.06E-06	159.66	S	QCIPYTLQNHYYRNNSFTAPSTVGKRNLSPS	1180300	954020	778
Q92538	1318	Golgi-specific brefeldin A-resistance guanine nucleotide exc	GBF1	0.978842	1.22E-06	159.58	S	PSYHQNDVSLDRGYTSDSEVYTDHGRPGKIH	10023000	3544200	4628
P35251	67	Replication factor C subunit 1	RFC1	0.930805	2.10E-06	159.54	Y	DDFKQKQPSKKKRIIYDSDSESEETLQVKNA	0	0	8362
Q86X95	202	Corepressor interacting with RBPJ 1	CIR1	0.999999	3.24E-11	159.53	S	GRNLTANDPSQEYVASEGEEDPEVEFLKSLT	6129800	3343500	4010
O94804	450	Serine/threonine-protein kinase 10	STK10	0.810232	5.59E-06	159.5	S	GDLSPAANRSQKASQSRPNSSALETLGGEKL	2121000	1412300	687
O94804	455	Serine/threonine-protein kinase 10	STK10	0.92725	5.59E-06	159.5	S	AANRSQKASQSRPNSSALETLGGEKLANGSL	2121000	1412300	689
Q3KQU3	113	MAP7 domain-containing protein 1	MAP7D1	0.601384	1.21E-06	159.5	S	PPAMGPRDARPPRRSSQPSPTAVPASDSPPT	0	581720	3071
Q3KQU3	116	MAP7 domain-containing protein 1	MAP7D1	0.953075	1.21E-06	159.5	S	MGPRDARPPRRSSQPSPTAVPASDSPPTKQE	1839800	857020	3072
Q9UK61	1552	Protein FAM208A	FAM208A	0.877	9.26E-08	159.44	S	RGTDQKKNTQIELQSSPDVQNSLLEDKTYLD	1168300	820220	6426
Q9UPT8	1269	Zinc finger CCCH domain-containing protein 4	ZC3H4	0.992403	1.05E-06	159.44	S	TLFGTVKQTPKTGSGSPFAGNSPAREGEQDA	6001100	5634900	6579
Q9UPT8	1275	Zinc finger CCCH domain-containing protein 4	ZC3H4	1	1.05E-06	159.44	S	KQTPKTGSGSPFAGNSPAREGEQDAASLKDV	8524200	7175700	6580

Q5VZL5	118	Zinc finger MYM-type protein 4	ZMYM4	0.895379	1.87E-06	159.41	T	SSIHTDDSLEVERRVTQHESDNENEIQIQNK	35187000	30405000	7735
Q6PCT2	408	F-box/LRR-repeat protein 19	FBXL19	0.998773	6.27E-07	159.4	S	RPPQLERHVVRPPPRSPEPDTLPLAAGSDHP	3660800	2332500	3536
Q9BXJ9	856	N-alpha-acetyltransferase 15, NatA auxiliary subunit	NAA15	0.956187	1.49E-06	159.4	S	PGYEEDMKITVNGDSSAEAEELANEI_____	922180	1435400	5452
Q9UIS9	297	Methyl-CpG-binding domain protein 1	MBD1	0.985928	2.94E-10	159.38	S	RRPGAQPLPPPPPSQSPEPTEPHPRALAPSP	2179000	599970	6387
Q9BQE9	114	B-cell CLL/lymphoma 7 protein family member B	BCL7B	0.993967	3.73E-11	159.26	S	DVYQLKVDSSTNSSPSPQQSESLSPAHTSDF	9283900	6843900	5304
Q9BQE9	122	B-cell CLL/lymphoma 7 protein family member B	BCL7B	0.979011	3.73E-11	159.26	S	SSTNSSPSPQQSESLSPAHTSDFRTDDSQPP	11267000	6843900	5305
P98174	48	FYVE, RhoGEF and PH domain-containing protein 1	FGD1	0.5	6.09E-15	159.25	S	SDPGASEPGLLARRGSGSALGGPLDPQFVGP	3266900	2676800	2010
P98174	50	FYVE, RhoGEF and PH domain-containing protein 1	FGD1	0.5	6.09E-15	159.25	S	PGASEPGLLARRGSGSALGGPLDPQFVGPSD	3266900	2676800	2011
O94782	67	Ubiquitin carboxyl-terminal hydrolase 1	USP1	0.815685	1.99E-07	159.18	S	YRASEIDQVVPAAQSSPINCEKRENLLPFVG	3409000	2129600	685
P12270	2155	Nucleoprotein TPR	TPR	1	7.81E-07	159.18	S	LVVPHRTDGFAEAIHSPQVAGVPRFRFGPPE	1949800	2453500	1040
Q4VCS5	718	Angiomotin	AMOT	0.935217	1.65E-07	159.18	S	AAQRDTTVISHSPNTSYDTALEARIQKEEEE	1318800	825570	3114
Q641Q2;Q(	728;728	WASH complex subunit FAM21A;WASH complex subunit FAM21C	FAM21A;FAM2	1	2.56E-06	159.14	S	TKKKETVSEAPPLLFSDEEEKEAQLGVKSVD	636430	799770	3387
P42166	351	Lamina-associated polypeptide 2, isoform alpha;Thymopoietin;	TMPO	0.999236	1.21E-06	159.12	S	GIQPLCPERSHISDQSPLSSKRKALEESESS	1468200	941770	1500
Q14188	24	Transcription factor Dp-2	TFDP2	0.999961	3.23E-06	159.12	S	TSTNAEVRGFIDQNLSPTKGNISFVAFPVSN	1100600	705070	2698
Q5SW79	930	Centrosomal protein of 170 kDa	CEP170	0.793703	1.97E-10	159.1	S	DEGPDTPSYNRDNSISPESDVDTASTISLVT	1778400	665070	3217
Q5SW79	933	Centrosomal protein of 170 kDa	CEP170	0.995475	1.97E-10	159.1	S	PDTPSYNRDNSISPESDVDTASTISLVTGET	1778400	1132700	3218
Q9UER7	690	Death domain-associated protein 6	DAXX	0.722339	1.68E-07	159.1	S	SLAPVADSSTRVDSPSHGLVTSSLCIPSPAR	1143300	1516400	6295
Q12888	525	Tumor suppressor p53-binding protein 1	TP53BP1	0.999704	9.43E-07	159.09	S	GDSCKLMLSTSEYSQSPKMESLSSHRIDEDG	6519700	2800600	2348
Q6VN20	365	Ran-binding protein 10	RANBP10	0.965296	1.15E-06	159.04	S	SLSSRSPKSQDSYPGSPSLSPRHGPSSSHMH	1555700	757850	3604
Q6VN20	369	Ran-binding protein 10	RANBP10	0.999533	1.15E-06	159.04	S	RSPKSQDSYPGSPSLSPRHGPSSSHMHNTGA	1555700	757850	3605
Q9UKE5	769	TRAF2 and NCK-interacting protein kinase	TNIK	0.992364	9.61E-07	159	S	SSERTRVRANSKSEGSPVLPHEPAKVKPEES	2318600	1181800	6437
Q96SI1	35	BTB/POZ domain-containing protein KCTD15	KCTD15	0.994093	5.01E-08	158.92	S	GTAEGGNMSRLSLTRSPVSPLAAQGIPLPAQ	1418600	673020	5121
Q13111	778	Chromatin assembly factor 1 subunit A	CHAF1A	0.25	1.30E-07	158.92	T	RGLLSNHTGSPRSPSTTYLHTPTPSEDAAIP	0	0	7521
Q13111	779	Chromatin assembly factor 1 subunit A	CHAF1A	0.25	1.30E-07	158.92	T	GLLSNHTGSPRSPSTTYLHTPTPSEDAAIPS	0	0	7522
P16403	2	Histone H1.2	HIST1H1C	0.849977	1.10E-06	158.91	S	_______________MSETAPAAPAAAPPAEK	21260000	0	1091
P46821	831	Microtubule-associated protein 1B;MAP1B heavy chain;MAP1 li	MAP1B	0.999998	2.86E-06	158.86	S	AIGPAKELEAERSLMSSPEDLTKDFEELKAE	933230	468630	1593
P46821	832	Microtubule-associated protein 1B;MAP1B heavy chain;MAP1 li	MAP1B	1	2.86E-06	158.86	S	IGPAKELEAERSLMSSPEDLTKDFEELKAEE	933230	468630	1594
Q8WUA2	178	Peptidyl-prolyl cis-trans isomerase-like 4	PPIL4	0.981313	1.35E-14	158.8	S	DDPFDDPPDLLIPDRSPEPTREQLDSGRIGA	4237000	2753300	4502
Q9NXG2	86	THUMP domain-containing protein 1	THUMPD1	1	2.44E-09	158.75	S	DMYGPEKFTDKDQQPSGSEGEDDDAEAALKK	13962000	21871000	6079
Q9NXG2	88	THUMP domain-containing protein 1	THUMPD1	1	2.44E-09	158.75	S	YGPEKFTDKDQQPSGSEGEDDDAEAALKKEV	12195000	20835000	6080
Q13330	576	Metastasis-associated protein MTA1	MTA1	0.98398	2.11E-06	158.65	S	SSLTPAKVAPVINNGSPTILGKRSYEQHNGV	10899000	6460800	2476
Q8TAQ2	302	SWI/SNF complex subunit SMARCC2	SMARCC2	0.999552	9.85E-06	158.65	S	DRRDKKGGNYKKRKRSPSPSPTPEAKKKNAK	3030900	1940200	4443
Q8TAQ2	304	SWI/SNF complex subunit SMARCC2	SMARCC2	0.989014	9.85E-06	158.65	S	RDKKGGNYKKRKRSPSPSPTPEAKKKNAKKG	5203600	2448200	4444
Q8TAQ2	306	SWI/SNF complex subunit SMARCC2	SMARCC2	0.568046	9.85E-06	158.65	S	KKGGNYKKRKRSPSPSPTPEAKKKNAKKGPS	0	0	4445
Q92538	1779	Golgi-specific brefeldin A-resistance guanine nucleotide ex	GBF1	0.749536	2.86E-06	158.65	S	GACDFEKPESPRAASSSSPGSPVASSPSRLS	803380	637540	4622
Q6PKG0	627	La-related protein 1	LARP1	0.997938	0.00486022	158.53	S	EMEQMDGRKNTFTAWSDEESDYEIDDRDVNK	0	0	3561
P51114	409	Fragile X mental retardation syndrome-related protein 1	FXR1	0.8052	2.30E-10	158.52	S	NYTSGYGTNSELSNPSETESERKDELSDWSL	920840	0	1774
Q76L83	440	Putative Polycomb group protein ASXL2	ASXL2	0.999474	3.73E-06	158.47	S	VVSQSECKEEALQMSSPGRKEECESQGEVQP	894470	660850	3703
Q14157	609	Ubiquitin-associated protein 2-like	UBAP2L	0.983378	3.83E-06	158.34	S	QRSTQTRRYPSSISSSPQKDLTQAKNGFSSV	6177300	576020	2663
Q9P2F8	1488	Signal-induced proliferation-associated 1-like protein 2	SIPA1L2	0.99995	3.86E-06	158.3	S	FYGNLSPRRSLYRTLSDESICSNRRGSSFGS	1006300	741860	6241
O95155	105	Ubiquitin conjugation factor E4 B	UBE4B	0.999865	1.14E-06	158.17	S	SNSLETQSQSLSRSQSMDIDGVSCEKSMSQV	893900	842730	733
Q9NWQ8	169	Phosphoprotein associated with glycosphingolipid-enriched mi	PAG1	0.822033	9.69E-07	158.17	S	DQGLGMEGPYEVLKDSSSQENMVEDCLYETV	0	556920	6062
O75376	157	Nuclear receptor corepressor 1	NCOR1	0.817395	3.82E-10	158.15	S	DAKKDPAFGGKHEAPSSPISGQPCGDDQNAS	8954300	0	595
P42566	324	Epidermal growth factor receptor substrate 15	EPS15	0.997423	2.34E-06	158.14	S	PPSDRASLQKNIIGSSPVADFSAIKELDTLN	855910	751170	1506
Q13425	233	Beta-2-syntrophin	SNTB2	0.973016	5.35E-15	158.14	S	AAPQSPSFSGSEDSGSPKHQNSTKDRKIIPL	0	15588000	2489
Q9H165	625	B-cell lymphoma/leukemia 11A	BCL11A	0.99701	1.39E-06	158.14	S	GESASGGLSKKLLLGSPSSLSPFSKRIKLEK	788120	1234100	5588
Q9H165	630	B-cell lymphoma/leukemia 11A	BCL11A	0.999187	1.39E-06	158.14	S	GGLSKKLLLGSPSSLSPFSKRIKLEKEFDLP	788120	1234100	5589
P51957	563	Serine/threonine-protein kinase Nek4	NEK4	1	4.00E-06	158.11	S	GEKRQVRRDLFAFQESPPRFLPSHPIVGKVD	891480	539080	1807
O95405	668	Zinc finger FYVE domain-containing protein 9	ZFYVE9	1	2.36E-06	158.08	S	EAEISTRPCLALAPDSPDNDLRAGQFGISAR	1813200	1863000	766
Q7Z6Z7	3373	E3 ubiquitin-protein ligase HUWE1	HUWE1	0.994754	2.54E-10	158.02	S	ETNCESDRERGNKACSPCSSQSSSSGICTDF	2162900	1518100	3865
Q8IWQ3	513	Serine/threonine-protein kinase BRSK2	BRSK2	0.999564	4.96E-08	158.02	S	QVPTPEEMSNLTPESSPELAKKSWFGNFISL	1942500	1546600	4063
Q8IWQ3	509	Serine/threonine-protein kinase BRSK2	BRSK2	0.998093	4.96E-08	158.02	T	RRKLQVPTPEEMSNLTPESSPELAKKSWFGN	5620100	3006300	7843
P02545	458	Prelamin-A/C;Lamin-A/C	LMNA	1	1.11E-05	157.84	S	EEVDEEGKFVRLRNKSNEDQSMGNWQIKRQN	2067300	1551300	864
Q96B18	520	Dapper homolog 3	DACT3	0.99712	3.22E-06	157.84	S	SPSAPQRRLLYGCAGSDSECSAGRLGPLGRR	1656200	828350	4791
Q9H426	257	Regulating synaptic membrane exocytosis protein 4	RIMS4	0.998448	3.35E-06	157.84	S	VDPATGPLLRQASQLSLESTVGPCGERS___	815940	495220	5657
Q14687	857	Genetic suppressor element 1	GSE1	1	2.47E-06	157.83	S	RLALSTRYSPDEMNNSPNFEEKKKFLTIFNL	941640	534600	2797
Q2KHR2	1081	DNA-binding protein RFX7	RFX7	0.999714	2.51E-06	157.74	S	RCRSVSPAVHRQRNLSGSTLYPVSNIPRSNV	905480	653530	3037
Q96DT7	210	Zinc finger and BTB domain-containing protein 10	ZBTB10	1	8.47E-15	157.64	S	DGSGAEGGSCSSSRRSGGDGGDEVEGSGVGA	5461200	4234300	4865
Q96FW1	16	Ubiquitin thioesterase OTUB1	OTUB1	0.615042	4.85E-11	157.63	S	MAAEEPQQQKQEPLGSDSEGVNCLAYDEAIM	7679000	7417500	4907
Q96FW1	18	Ubiquitin thioesterase OTUB1	OTUB1	0.5	4.85E-11	157.63	S	AEEPQQQKQEPLGSDSEGVNCLAYDEAIMAQ	0	7417500	4908
Q86V48	659	Leucine zipper protein 1	LUZP1	1	9.74E-06	157.62	S	LRCRVIKSSGREKPDSDDDLDIASLVTAKLV	0	785630	3945
P27816	521	Microtubule-associated protein 4	MAP4	1	7.67E-06	157.5	T	MSPLSETEMALGKDVTPPPETEVVLIKNVCL	7322300	5024200	7336
O00267	666	Transcription elongation factor SPT5	SUPT5H	1	3.46E-06	157.42	S	PRDVTNFTVGGFAPMSPRISSPMHPSAGGQR	880850	1337800	114
Q7Z3K3	1360	Pogo transposable element with ZNF domain	POGZ	0.998607	1.69E-05	157.37	S	MQEELIASLEEQLKLSGEHSESSTPRPRSSP	0	0	3780
Q9BW71	87	HIRA-interacting protein 3	HIRIP3	1	3.84E-05	157.37	S	KLDLTKKGKRPPTPCSDPERKRFRFNSESES	1596800	2844700	5419
Q7Z3K3	1368	Pogo transposable element with ZNF domain	POGZ	0.78015	1.69E-05	157.37	T	LEEQLKLSGEHSESSTPRPRSSPEETIEPES	2338200	1402000	7801
Q9BW71	84	HIRA-interacting protein 3	HIRIP3	1	3.84E-05	157.37	T	REDKLDLTKKGKRPPTPCSDPERKRFRFNSE	0	1307500	8057
Q3MHD2	75	Protein LSM12 homolog	LSM12	0.999618	7.86E-06	157.35	T	LQYVSEVEIINDRTETPPPLASLNVSKLASK	1856300	1169100	7670
Q12888	1678	Tumor suppressor p53-binding protein 1	TP53BP1	1	7.77E-06	157.33	S	VLSGKRKLITSEEERSPAKRGRKSATVKPGA	907310	2469200	2346
Q14687	826	Genetic suppressor element 1	GSE1	1	6.63E-06	157.33	S	VAQKRRKRRRMLRERSPSPPTIQSKRQTPSP	2394200	1876500	2795
Q14687	828	Genetic suppressor element 1	GSE1	0.999184	6.63E-06	157.33	S	QKRRKRRRMLRERSPSPPTIQSKRQTPSPRL	2394200	1876500	2796
Q9UQ35	395	Serine/arginine repetitive matrix protein 2	SRRM2	0.995416	2.12E-14	157.3	S	PLATTPLSQEPVNPPSEASPTRDRSPPKSPE	8829900	7982300	6642
A6NDB9	303	Paralemmin-3	PALM3	0.924063	4.70E-06	157.2	S	AMGEIGRVPEVVQTSSPRLQERLEAAASIEG	0	0	36
Q9P1Y6	917	PHD and RING finger domain-containing protein 1	PHRF1	0.912236	2.96E-10	157.14	T	MSKLRGAVAAEGASDTEREEPTESQGLAARL	0	4932300	8189
Q9UK76	88	Hematological and neurological expressed 1 protein;Hematolo	HN1	0.791222	2.16E-06	157.09	S	GREDLESSGLQRRNSSEASSGDFLDLKGEGD	13072000	610190	6432
Q8IYB3	234	Serine/arginine repetitive matrix protein 1	SRRM1	0.991617	2.81E-06	157.07	S	KTPELPEPSVKVEPSVQEATSTSDILKVPK	686090	414690	4136
Q8TDR0	476	TRAF3-interacting protein 1	TRAF3IP1	1	7.39E-06	157.07	S	PGSARPAPPRVKRQDSMEALQMDRSGSGKTV	1722700	0	4470
Q9BYW2	1084	Histone-lysine N-methyltransferase SETD2	SETD2	0.611962	2.81E-06	157.07	S	VVVVPKNSTLPMEETSPCSSRSSQSYRHYSD	876870	896530	5475
Q9BSJ6	131	Protein FAM64A	FAM64A	0.744653	1.81E-06	157.03	S	VKARRRKRGAQKGSGSPTHSLSQKSTRLSGA	5812200	2403100	5341
Q9ULT0	647	Tetratricopeptide repeat protein 7A	TTC7A	1	4.83E-06	157.03	S	TLYSFSQLGGLEKDGSFGEGLTMKKQSGMHL	891530	597690	6500
Q9Y2W1	243	Thyroid hormone receptor-associated protein 3	THRAP3	1	4.60E-06	157.03	S	YGTGSASRASAVSELSPRERSPALKSPLQSV	35571000	40376000	6815
Q9Y3Q8	279	TSC22 domain family protein 4	TSC22D4	1	1.81E-06	157.03	S	SPALYFTHDASLVHKSPDPFGAVAAQKFSLA	1166000	860970	6863
Q5VV41	230	Rho guanine nucleotide exchange factor 16	ARHGEF16	0.994253	3.01E-10	157.02	S	LYQEIQERGLNTSQESDDDILDESSSPEGTQ	1714500	0	3353
P35269	224	General transcription factor IIF subunit 1	GTF2F1	0.999941	3.02E-10	157	S	DLEDDLEMSSDASDASGEEGGRVPKAKKKAP	2479100	387560	1391
Q9Y606	420	tRNA pseudouridine synthase A, mitochondrial	PUS1	1	2.23E-05	156.96	S	TAGGTGAKVPSPLEGSEGDGDTD________	1570200	1399200	6971
Q8NEL9	727	Phospholipase DDHD1	DDHD1	0.620787	2.98E-10	156.92	S	VSENEGISTIPSPVTSPVLSRRHYGESITNI	0	984190	4371
O75179	2045	Ankyrin repeat domain-containing protein 17	ANKRD17	0.993869	3.86E-08	156.83	S	PMPTAKEHYPVSSPSSPSPPAQPGGVSRNSP	28066000	4304500	584
Q92560	596	Ubiquitin carboxyl-terminal hydrolase BAP1	BAP1	0.365615	2.13E-06	156.72	S	GSSPSIRPIQGSQGSSSPVEKEVVEATDSRE	0	0	4637
Q92560	597	Ubiquitin carboxyl-terminal hydrolase BAP1	BAP1	0.365615	2.13E-06	156.72	S	SSPSIRPIQGSQGSSSPVEKEVVEATDSREK	0	0	4638
Q9H1B7	658	Interferon regulatory factor 2-binding protein-like	IRF2BPL	0.837305	2.97E-06	156.72	S	GSSVHSTTASARRNSSSPVSPASVPGQRRLA	7177300	3482400	5596
Q9H5H4	160	Zinc finger protein 768	ZNF768	0.999859	1.90E-06	156.72	S	ESSRYESQNTELKTQSPEFEAQSSKFQEGAE	0	516620	5718
Q99504	438	Eyes absent homolog 3	EYA3	1	5.15E-06	156.6	S	REIYDKHKSNVGGLLSPQRKEALQRLRAEIE	652950	394950	5199
Q13153	225	Serine/threonine-protein kinase PAK 1	PAK1	0.760446	5.75E-10	156.57	T	PVTPTRDVATSPISPTENNTTPPDALTRNTE	0	3017100	7526
Q8IY81	335	pre-rRNA processing protein FTSJ3	FTSJ3	0.999134	2.03E-10	156.51	S	AKKLKEQAKALDISLSSGEEDEGDEEDSTAG	9444800	9376500	4120
Q8IY81	336	pre-rRNA processing protein FTSJ3	FTSJ3	0.992645	2.03E-10	156.51	S	KKLKEQAKALDISLSSGEEDEGDEEDSTAGT	9444800	9376500	4121
Q8NDC0	2	MAPK-interacting and spindle-stabilizing protein-like	MAPK1IP1L	0.998331	1.71E-06	156.51	S	_______________MSDEFSLADALPEHSPA	603600	561700	4345
Q9UQ35	2272	Serine/arginine repetitive matrix protein 2	SRRM2	1	2.24E-06	156.36	S	ADSRTPAAAAAMNLASPRTAVAPSAVNLADP	699840	869390	6745
Q00587	101	Cdc42 effector protein 1	CDC42EP1	0.999832	9.41E-07	156.2	S	LQLVRRVGAPPRRMASPPAPSPAPPAISPII	755100	713040	2027
Q00587	113	Cdc42 effector protein 1	CDC42EP1	1	9.41E-07	156.2	S	RMASPPAPSPAPPAISPIIKNAISLPQLNQA	755100	713040	2028
Q7Z2W4	275	Zinc finger CCCH-type antiviral protein 1	ZC3HAV1	1	1.83E-06	156.2	S	FLASASASAERSCTPSPDQISHRASLEDAPV	5926600	4081600	3754
Q8N108	369	Mesoderm induction early response protein 1	MIER1	0.996878	1.70E-06	156.2	S	PGVTDYMDRLLDESESAASSRAPSPPPTASN	0	408020	4205
Q8N108	372	Mesoderm induction early response protein 1	MIER1	0.991	1.70E-06	156.2	S	TDYMDRLLDESESAASSRAPSPPPTASNSSN	0	408020	4206
Q7Z2W4	273	Zinc finger CCCH-type antiviral protein 1	ZC3HAV1	1	1.83E-06	156.2	T	QEFLASASASAERSCTPSPDQISHRASLEDA	5599500	3612300	7798
O00512	907	B-cell CLL/lymphoma 9 protein	BCL9	1	3.48E-06	156.17	S	LAGMLAGPAAAASIKSPPVLGSAAASPVHLK	840100	905150	128
O00512	917	B-cell CLL/lymphoma 9 protein	BCL9	0.99982	3.48E-06	156.17	S	AASIKSPPVLGSAAASPVHLKSPSLPAPSPG	840100	905150	129
P06400	807	Retinoblastoma-associated protein	RB1	0.999997	1.61E-05	156.17	S	FPSSPLRIPGGNIYISPLKSPYKISEGLPTP	1561400	1093000	911
P06400	811	Retinoblastoma-associated protein	RB1	1	1.61E-05	156.17	S	PLRIPGGNIYISPLKSPYKISEGLPTPTKMT	1561400	1093000	912
Q00587	192	Cdc42 effector protein 1	CDC42EP1	0.999991	2.21E-05	156.16	S	RDGSFPSEPGLRRSDSLLSFRLDLDLGPSLL	716180	592910	2030
Q9BUH6	148	Protein PAXX	C9orf142	0.999999	2.08E-06	156.08	S	WSLERRLAAAEETAVSPRKSPRPAGPQLFLP	5520200	4942300	5378
O94885	1028	SAM and SH3 domain-containing protein 1	SASH1	0.962353	9.59E-07	156.06	S	SPDAPCLPVKRGSPASPTSPSDCPPALAPRP	1036800	811550	710
O94885	1031	SAM and SH3 domain-containing protein 1	SASH1	0.600203	9.59E-07	156.06	S	APCLPVKRGSPASPTSPSDCPPALAPRPLSG	1036800	0	711
Q9NZ63	261	Uncharacterized protein C9orf78	C9orf78	0.999998	0.000167978	156.06	S	RPLRVGDTEKPEPERSPPNRKRPANEKATDD	16127000	17012000	6150
Q9BU76	215	Multiple myeloma tumor-associated protein 2	MMTAG2	0.749076	1.48E-05	156.06	T	EKKKKDKEHRRPAEATSSPTSPERPRHHHHD	0	0	8048
Q5T481	1120	RNA-binding protein 20	RBM20	1	5.33E-06	156.01	S	PENSRYVEMKSLEVRSPEYTEVELKQPLSLP	1250800	635460	3283
P53985	467	Monocarboxylate transporter 1	SLC16A1	0.99356	2.01E-05	156	S	QKANEQKKESKEEETSIDVAGKPNEVTKAAE	0	2625300	1839
Q7Z4H7	584	HAUS augmin-like complex subunit 6	HAUS6	1	2.18E-05	156	T	SLGSNPFLTRNQIPRTPENLITEIRSSWRKA	1798900	2361800	7808
O60343	591	TBC1 domain family member 4	TBC1D4	1	3.14E-06	155.97	S	SRGANRMRGRLGSVDSFERSNSLASEKDYSP	2372000	1488500	490
Q13425	231	Beta-2-syntrophin	SNTB2	0.636102	7.59E-15	155.95	S	EGAAPQSPSFSGSEDSGSPKHQNSTKDRKII	0	0	2488
Q9UQ35	1880	Serine/arginine repetitive matrix protein 2	SRRM2	0.999871	4.61E-05	155.88	T	RSRASPATHRRSRSRTPLISRRRSRSRTSPV	24943000	17776000	8269
Q92733	267	Proline-rich protein PRCC	PRCC	0.99972	7.33E-10	155.71	S	AALQVTKQITQEEDDSDEEVAPENFFSLPEK	7725300	3430200	4694
P08559;P1	293;291	Pyruvate dehydrogenase E1 component subunit alpha, somatic f	PDHA1;PDHA2	0.991656	2.06E-06	155.63	S	GPILMELQTYRYHGHSMSDPGVSYRTREEIQ	7176000	0	955
P08559;P1	300;298	Pyruvate dehydrogenase E1 component subunit alpha, somatic f	PDHA1;PDHA2	0.999927	2.06E-06	155.63	S	QTYRYHGHSMSDPGVSYRTREEIQEVRSKSD	3222700	0	957
P19338	67	Nucleolin	NCL	0.99969	0.000405897	155.58	S	KGKKAAATSAKKVVVSPTKKVAVATPAKKAA	50763000	61348000	1161
P29375	1331	Lysine-specific demethylase 5A	KDM5A	0.829983	5.72E-06	155.47	S	FQQSAFNRVVSSVSSSPRQTMYDDEETDSD	1276100	883100	1318
Q8IYB3	738	Serine/arginine repetitive matrix protein 1	SRRM1	1	5.72E-06	155.47	S	RVSRTPEPKKIKKAASPSPQSVRRVSSSRSV	22785000	22239000	4130
Q9Y2W1	575	Thyroid hormone receptor-associated protein 3	THRAP3	1	5.72E-06	155.47	S	SFSITREAQVNVRMDSFDEDLARPSGLLAQE	864530	944780	6827
P11388	1377	DNA topoisomerase 2-alpha	TOP2A	0.999985	2.27E-06	155.43	S	PKLSNKELKPQKSVVSDLEADDVKGSVPLSS	441960	284260	1022
O15014	576	Zinc finger protein 609	ZNF609	0.999934	1.90E-06	155.39	S	PARSATPKVRLVEPHSPSPSSKFSTKGLCKK	7456300	4304800	227
O15014	580	Zinc finger protein 609	ZNF609	0.701982	1.90E-06	155.39	S	ATPKVRLVEPHSPSPSSKFSTKGLCKKKLSG	183380	0	229
Q15311	463	RalA-binding protein 1	RALBP1	0.998001	5.78E-06	155.39	S	RQECETKIAQEIASLSKEDVSKEEMNENEEV	3498700	2258000	2902
Q6PJT7	515	Zinc finger CCCH domain-containing protein 14	ZC3H14	1	1.03E-05	155.39	S	HLMQTRDLVQPDKPASPKFIVTLDGVPSPPG	8298500	4535000	3552
Q7Z7L8	399	Uncharacterized protein C11orf96	C11orf96	1	5.78E-06	155.39	S	VSPMEEEKAKKSFLQSLECLRRSTQSLSLQR	453650	535780	3889
Q15036	335	Sorting nexin-17	SNX17	0.433804	4.84E-06	155.37	S	CWRVTSSVPLPSGSTSSPGRGRGEVRLELAF	0	0	2861
Q15036	336	Sorting nexin-17	SNX17	0.433804	4.84E-06	155.37	S	WRVTSSVPLPSGSTSSPGRGRGEVRLELAFE	0	0	2862
Q5T200	265	Zinc finger CCCH domain-containing protein 13	ZC3H13	1	3.62E-05	155.37	S	NSKTNQSKKKGPRTPSPPPPIPEDIALGKKY	6807400	5903000	3275
Q5T200	263	Zinc finger CCCH domain-containing protein 13	ZC3H13	1	3.62E-05	155.37	T	QRNSKTNQSKKKGPRTPSPPPPIPEDIALGK	6807400	5903000	7722
Q9BW71	555	HIRA-interacting protein 3	HIRIP3	0.997925	5.82E-06	155.33	S	PDWSHMRGIISSDGESN______________	0	378280	5416
Q0JRZ9	387	F-BAR domain only protein 2	FCHO2	0.995045	2.60E-06	155.26	S	ASLDELKVSIGNITLSPAISRHSPVQMNRNL	757490	478050	2287
P49792;Q(	2242;126	E3 SUMO-protein ligase RanBP2;RanBP2-like and GRIP domain-c	RANBP2;RGPI	0.845141	6.46E-10	155.25	S	YDLREDALDDSVSSSSVHASPLASSPVRKNL	1891100	913210	1705

Q6IN85	127	Serine/threonine-protein phosphatase 4 regulatory subunit 3/	SMEK1	0.939374	3.56E-08	155.2	S	DLVDESEEERFDDMSSPGLELPSCELSRLEE	6883000	4960600	3476
Q9NWQ8	171	Phosphoprotein associated with glycosphingolipid-enriched mi	PAG1	0.699656	1.52E-06	155.2	S	GLGMEGPYEVLKDSSSQENMVEDCLYETVKE	0	0	6064
Q9UQ35	1014	Serine/arginine repetitive matrix protein 2	SRRM2	0.892426	3.42E-06	155.17	S	VKAQTPPGPSLSGSKSPCPQEKSKDSLVQSC	20770000	14229000	6604
Q9UQ35	1003	Serine/arginine repetitive matrix protein 2	SRRM2	1	3.42E-06	155.17	T	SHSGSISPYPKVKAQTPPGPSLSGSKSPCPQ	67678000	44124000	8253
P46821	1438	Microtubule-associated protein 1B;MAP1B heavy chain;MAP1 li	MAP1B	1	8.11E-08	155.16	S	RGAESPPEEKSGKQGSPDQVSPVSEMTSTSL	1474600	1021200	1587
P46821	1443	Microtubule-associated protein 1B;MAP1B heavy chain;MAP1 li	MAP1B	0.999964	8.11E-08	155.16	S	PFEEKSGKQGSPDQVSPVSEMTSTSLYQDKQ	7450000	6235600	1588
O15173	104	Membrane-associated progesterone receptor component 2	PGRMC2	1	2.52E-05	155.15	S	ESPATSLPRMKKRDFSLEQLRQYDGSRNPRI	1403000	729160	298
Q9NX40	108	OCIA domain-containing protein 1	OCIAD1	1	6.26E-06	155.14	S	SYVKTCQEKFKKLENSPLGEALRSGQARRSS	2637200	1271800	6075
Q9P1Y6	1360	PHD and RING finger domain-containing protein 1	PHRF1	0.988559	2.63E-06	155.14	S	HLLRPDAAEKAEAPSSPDVAPAGKEDSPSAS	0	2276700	6199
Q9P260	186	LisH domain and HEAT repeat-containing protein KIAA1468	KIAA1468	1	2.20E-06	155.1	S	GGGQLNRAGSISTLDSLDFARYSDDGNRETD	479940	1639300	6219
P46821	1260	Microtubule-associated protein 1B;MAP1B heavy chain;MAP1 li	MAP1B	0.987288	2.65E-06	155.08	S	AVSSEKVSPSKSPSLSPSPPSPLEKTPLGER	140820	430760	1599
Q13523	569	Serine/threonine-protein kinase PRP4 homolog	PRPF4B	0.62886	8.19E-08	155.08	S	YLAEDSNMSVPSEPSSPQSSTRTRSPSPDDI	0	0	2567
Q5JTH9	1080	RRP12-like protein	RRP12	1	9.85E-08	155.08	S	PAQGKGDSIEEILADSEDEEDNEEEERSRGK	507890	1424600	3197
O60508	20	Pre-mRNA-processing factor 17	CDC40	0.549127	2.38E-14	154.84	S	IAALAASYGSGSGSESDSDSESSRCPLPAAD	0	406860	506
P05412;P[illegible]73;100		Transcription factor AP-1;Transcription factor jun-D	JUN;JUND	1	2.62E-05	154.84	S	SDLLTSPDVGLLKLASPELERLIIQSSNGHI	12519000	8371000	904
Q15942	281	Zyxin	ZYX	1	4.98E-06	154.81	S	FSPVTPKFTPVASKFSPGAPGGSGSQPNQKL	2859100	2381100	2994
Q9NYV4	893	Cyclin-dependent kinase 12	CDK12	0.999978	0.00035128	154.81	T	FGLARLYNSEESRPYTNKVITLWYRPPELLL	0	900480	8179
P49792;Q[illegible]2246;127[illegible]		E3 SUMO-protein ligase RanBP2;RanBP2-like and GRIP domain-co	RANBP2;RGPI	0.980316	6.83E-10	154.79	S	EDALDDSVSSSSVHASPLASSPVRKNLFRFG	5216700	1988200	1706
O43482	47	Protein Mis18-beta	OIP5	0.5	1.28E-14	154.7	S	SFTTSMEWDTQVVKGSSPLGPAGLGAEEPAA	1311500	633800	390
O43482	48	Protein Mis18-beta	OIP5	0.5	1.28E-14	154.7	S	FTTSMEWDTQVVKGSSPLGPAGLGAEEPAAG	1311500	633800	391
Q14160	1561	Protein scribble homolog	SCRIB	0.762906	1.28E-14	154.69	S	PSPTPVEDLGPQTSTSPGRLSPDFAEELRSL	777040	3301700	2676
P78310	332	Coxsackievirus and adenovirus receptor	CXADR	0.999188	3.57E-06	154.67	S	QYNQVPSEDFERTPQSPTLPPAKVAAPNLSR	2607900	993330	1949
Q5SW79	501	Centrosomal protein of 170 kDa	CEP170	0.866239	4.82E-14	154.67	T	ATSEKDNDDDQSDKGTYTIELENPNSEEVEA	0	0	7712
Q8WVC0	658	RNA polymerase-associated protein LEO1	LEO1	0.999999	2.49E-06	154.66	S	EDDDKANKKHKKYVISDEEEEDDD_______	1005000	1647100	4539
O75151	882	Lysine-specific demethylase PHF2	PHF2	0.999906	1.40E-06	154.52	S	DACFKDSDYVYPSLESDEDNPIFKSRSKKRK	1308900	796130	570
Q9UQ35	436	Serine/arginine repetitive matrix protein 2	SRRM2	0.825707	5.40E-06	154.48	S	SPPSPQPTKVSRHASSSPESPKPAPAPGSHR	4004300	3932700	6636
Q6W2J9	1410	BCL-6 corepressor	BCOR	1	1.17E-05	154.47	S	RRFRKRPEPSSDYDLSPAKQEPKPFDRLQQL	1809600	890420	3621
Q7Z3K3	425	Pogo transposable element with ZNF domain	POGZ	0.997868	6.34E-06	154.46	S	SLDSEPSVPSAAKPPSEKTAPVASTPSSTP	1392500	535670	3783
Q04323	200	UBX domain-containing protein 1	UBXN1	0.77425	5.87E-15	154.45	S	SQPPPVAPEPGPVPSSPSQEPPTKREYDQCR	6838800	9231300	2154
P07814	885	Bifunctional glutamate/proline--tRNA ligase;Glutamate--tRNA	EPRS	0.937344	1.42E-06	154.39	S	KEYIPGQPPLSQSSDSSPTRNSEPAGLETPE	10144000	0	938
Q9NZT2	517	Opioid growth factor receptor	OGFR	0.95748	1.74E-20	154.34	S	EDTEGRTGPKEGTPGSPSETPGPSPAGPAGD	2414900	2718200	6172
O75179	2059	Ankyrin repeat domain-containing protein 17	ANKRD17	1	1.66E-06	154.31	S	SSPSPPAQPGGVSRNSPLDCGSASPNKVASS	0	506770	587
Q86WB0	329	Nuclear-interacting partner of ALK	ZC3HC1	0.99979	4.04E-06	154.31	S	RLPLVPESPRRMMTRSQDATFSPGSEQAEKS	0	0	3983
Q15648	805	Mediator of RNA polymerase II transcription subunit 1	MED1	1	2.15E-05	154.31	T	ASKLPSTSDDCPAIGTPLRDSSSSGHSQSTL	782170	558760	7645
P49006	71	MARCKS-related protein	MARCKSL1	1	1.76E-20	154.25	S	EAAGATGDAIEPAPPSQGAEAKGEVPPKETP	1436400	1417700	1639
O75362	407	Zinc finger protein 217	ZNF217	0.99987	2.01E-06	154.23	S	LHSRVHKKDRRAGAESPTMSVDGRQPGTCSP	0	1445500	591
Q13247	303	Serine/arginine-rich splicing factor 6	SRSF6	1	6.95E-06	154.23	S	DIKSKSRSRSQSRSNSPLPVPPSKARSVSPP	32649000	16211000	2442
Q8IYB3	754	Serine/arginine repetitive matrix protein 1	SRRM1	0.99987	5.78E-06	154.23	S	PSPQSVRRVSSSRSVSGSPEPAAKKPPAPPS	0	2335300	4171
Q8IYB3	756	Serine/arginine repetitive matrix protein 1	SRRM1	1	5.78E-06	154.23	S	PQSVRRVSSSRSVSGSPEPAAKKPPAPPSPV	0	2624800	4172
Q92845	60	Kinesin-associated protein 3	KIFAP3	1	0.000199808	154.13	S	MLGERKECQKIIRLKSLNANTDITSLARKVV	0	536340	4722
Q13409	101	Cytoplasmic dynein 1 intermediate chain 2	DYNC1I2	0.728136	2.72E-07	154.08	S	MSPSSKSVSTPSEAGSQDSGDGAVGSRTLHW	0	274160	2481
P49848	653	Transcription initiation factor TFIID subunit 6	TAF6	1	4.21E-06	153.95	S	LSGSALCGGKQEAGDSPPPAPGTPKANGSQP	453460	416910	1740
Q8ND56	216	Protein LSM14 homolog A	LSM14A	0.999991	1.36E-05	153.94	S	TASAHLPAPAAVGRRSPVSTRPLPSASQKAG	42206000	17375000	4340
Q09472	1038	Histone acetyltransferase p300	EP300	0.958813	3.34E-10	153.88	S	IKEEEDQPSTSATQSSPAPGQSKKKIFKPEE	1498600	1449200	2272
Q9NW68	92	BSD domain-containing protein 1	BSDC1	0.997874	1.50E-06	153.86	S	GLSDFLGVISDTFAPSPDKTIDCDVITLMGT	271630	190640	6052
P23193	96	Transcription elongation factor A protein 1	TCEA1	0.600483	8.58E-06	153.81	T	STEKDLDEKKKEPAITSQNSPEAREESTSSG	0	0	7315
P51858	132	Hepatoma-derived growth factor	HDGF	1	1.76E-06	153.76	S	EAAEGDGDKKGNAEGSSDEEGKLVIDEPAKE	15101000	9397300	1801
P51858	133	Hepatoma-derived growth factor	HDGF	1	1.76E-06	153.76	S	AAEGDGDKKGNAEGSSDEEGKLVIDEPAKEK	15101000	9397300	1802
Q9H8Y8	432	Golgi reassembly-stacking protein 2	GORASP2	0.499983	4.14E-09	153.76	S	PTAKAPTTVEDRVGDSTPVSEKPVSAAVDAN	0	0	5793
O95400	194	CD2 antigen cytoplasmic tail-binding protein 2	CD2BP2	0.791909	3.00E-05	153.75	S	GARGGGKGRKGPGQPSSPQRLDRLSGLADQM	2206600	1724400	760
Q9Y2X7	361	ARF GTPase-activating protein GIT1	GIT1	0.688041	3.08E-06	153.74	S	DILSEAKRRQQGKSLSSPTDNLELSLRSQSD	948900	1130700	6838
Q8N6T3	135	ADP-ribosylation factor GTPase-activating protein 1	ARFGAP1	1	1.48E-05	153.72	T	EGREWSLESSPAQNWTPPQPRTLPSMVHRVS	1294100	970070	7880
Q9H5H4	125	Zinc finger protein 768	ZNF768	0.999999	7.36E-06	153.7	S	QSPEFESQSPRYEPQSPGYEPRSPGYEPRSP	6704500	4186900	5719
Q8WVB6	63	Chromosome transmission fidelity protein 18 homolog	CHTF18	0.793163	2.84E-07	153.68	S	PPRTFEEALARGDAASSPAPAASVGSSQGGA	689520	487730	4534
Q9Y6G9	421	Cytoplasmic dynein 1 light intermediate chain 1	DYNC1LI1	0.497142	5.87E-06	153.68	S	PRTPNRSVSSNVASVSPIPAGSKKIDPNMKA	0	0	7008
P78527	3205	DNA-dependent protein kinase catalytic subunit	PRKDC	1	2.87E-07	153.58	S	LSKIEEKLTPLPEDNSMNVDQDGDPSDRMEV	4465400	3312100	1968
Q69YN4	184	Protein virilizer homolog	KIAA1429	1	7.35E-10	153.52	T	QFNGSPPRPQPRGPRTPPGPPPPDDDEDDPV	0	1114000	7747
Q6PL24	21	Protein TMED8	TMED8	0.999998	1.46E-14	153.49	S	AAEGPGSWSPTARPGSAGGVGDCQGVEGSQA	0	4113200	3571
Q8NEN9	538	PDZ domain-containing protein 8	PDZD8	1	7.51E-06	153.49	S	KRVPTTLSIKPLGAISPVLNRKLAVGSHPLP	1345900	1460100	4376
Q9UBF8	266	Phosphatidylinositol 4-kinase beta	PI4KB	0.897575	5.98E-06	153.49	S	KRELPSLSPAPDTGLSPSKRTHQRSKSDATA	2843200	2051000	6264
Q9NR30	89	Nucleolar RNA helicase 2	DDX21	0.999999	1.17E-05	153.41	S	SKKAKKKEEPSQNDISPKTKSLRKKKEPIEK	0	0	5945
Q9ULW0	738	Targeting protein for Xklp2	TPX2	0.999896	2.19E-06	153.41	S	GLEIKSSDQPLTVPVSPKFSTRFHC______	3050700	1671500	6513
P38398	425	Breast cancer type 1 susceptibility protein	BRCA1	0.685459	4.76E-07	153.36	S	ADVLDVLNEVDEYSGSSEKIDLLASDPHEAL	614170	431310	1456
Q15648	1207	Mediator of RNA polymerase II transcription subunit 1	MED1	1	2.28E-05	153.17	S	SPSHSRPPGGSDKLASPMKPVPGTPPSSKAK	10205000	6871900	2962
Q15648	1218	Mediator of RNA polymerase II transcription subunit 1	MED1	0.916534	2.28E-05	153.17	S	DKLASPMKPVPGTPPSSKAKSPISSGSGGSH	0	6871900	2963
O75925	485	E3 SUMO-protein ligase PIAS1	PIAS1	0.989739	3.28E-06	153.14	S	EEEEEPSAKRTCPSLSPTSPLNNKGILSLPH	987870	507280	669
Q8WWI1	1593	LIM domain only protein 7	LMO7	0.997185	3.28E-06	153.14	S	TTGVATTQSPTPRSHSPSASQSGSQLRNRSV	792720	393840	4555
Q9H4L7	211	SWI/SNF-related matrix-associated actin-dependent regulator	SMARCAD1	0.684077	1.43E-06	153.14	S	LMFGDAGGGPRKRKLSSSSEPYEEDEFNDDQ	526940	0	5694
Q9H4L7	214	SWI/SNF-related matrix-associated actin-dependent regulator	SMARCAD1	0.985545	1.43E-06	153.14	S	GDAGGGPRKRKLSSSSEPYEEDEFNDDQSIK	526940	588910	5697
Q7Z4Q2	15	HEAT repeat-containing protein 3	HEATR3	0.953986	9.46E-27	153.13	S	_MGKSRTKRFKRPQFSPTGDCQAEAAAAANG	0	0	3816
Q9Y4B4	1169	Helicase ARIP4	RAD54L2	1	7.53E-10	153.11	S	SSPKAPDPEGLARPVSPDSPEIISELQQYAD	5474400	3738100	6901
Q9Y4B4	1172	Helicase ARIP4	RAD54L2	1	7.53E-10	153.11	S	KAPDPEGLARPVSPDSPEIISELQQYADVAA	5474400	3738100	6902
P08559	231	Pyruvate dehydrogenase E1 component subunit alpha, somatic f	PDHA1	0.523532	1.77E-05	153.1	T	LPCIFICENNRYGMGTSVERAAASTDYYKRG	0	0	7268
O14497	702	AT-rich interactive domain-containing protein 1A	ARID1A	0.989658	5.19E-06	153.06	S	PHLPGIRGPSPSPVGSPASVAQSRSGPLSPA	1056500	0	141
Q15276	374	Rab GTPase-binding effector protein 1	RABEP1	1	4.07E-07	153.06	S	AQLSNEEEHLDSTRGSVHSLDAGLLLPSGDP	6070100	2478900	2895
Q15276	377	Rab GTPase-binding effector protein 1	RABEP1	1	4.07E-07	153.06	S	SNEEEHLDSTRGSVHSLDAGLLLPSGDPFSK	6070100	2478900	2896
Q9H2Z4	271	Homeobox protein Nkx-2.4	NKX2-4	1	6.09E-08	153.04	S	LQQEGGLGPPPPPPPSPRRVAVPVLVKDGKP	2523500	1195000	5630
Q9P2B4	488	CTTNBP2 N-terminal-like protein	CTTNBP2NL	0.999999	2.28E-06	153	S	DQQASGLQSPPSRDLSPTLIDNSAAKQLARN	4126200	3059500	6227
Q9ULJ3	714	Zinc finger and BTB domain-containing protein 21	ZBTB21	1	2.97E-06	152.97	S	GVNKVAKPKEHAPLASPVENKEVYQCRLCNA	0	628410	6475
O94776	435	Metastasis-associated protein MTA2	MTA2	0.999487	4.66E-06	152.94	S	PHSRGHLSRPEAQSLSPYTTSANRAKLLAKN	0	518350	683
P27816	1073	Microtubule-associated protein 4	MAP4	1	6.32E-06	152.94	S	SQKLNFKEKAQAKVGSLDNVGHLPAGGAVKT	13610000	5848200	1298
P46821	1154	Microtubule-associated protein 1B;MAP1B heavy chain;MAP1 li	MAP1B	0.948683	9.98E-10	152.94	S	PRDVMSDETNNEETESPSQEFVNITKYESSL	993520	511230	1578
P50502;Q[illegible]75;71		Hsc70-interacting protein;Putative protein FAM10A4	ST13;ST13P[illegible]	0.928119	3.87E-06	152.94	S	PDSKKVEEDLKADEPSSEESDLEIDKEGVIE	808670	1028300	1748
Q6KC79	274	Nipped-B-like protein	NIPBL	1	6.07E-06	152.94	S	GDSSTMRNAASFPLRSPQPVCSPAGSEGTPK	541060	371480	3488
Q99638	387	Cell cycle checkpoint control protein RAD9A	RAD9A	1	6.31E-06	152.94	S	PVRSPQGPSPVLAEDSEGEG___________	3647700	2550700	5240
Q9NS56	98	E3 ubiquitin-protein ligase Topors	TOPORS	0.999587	3.34E-06	152.94	S	KAGTSKLQQTVPADASPDSKCPICLDRFDNV	0	1918900	5994
Q9P2D1	2272	Chromodomain-helicase-DNA-binding protein 7	CHD7	0.996209	1.23E-06	152.94	S	PEAGAVSRGKNFDEESNASMSTARDETRDGF	845120	368080	6233
P0C1Z6	180	TCF3 fusion partner	TFPT	1	1.65E-06	152.9	S	TLSPPRRTPAPPEPGSPAPGEGPSGRKRRRV	3551500	2529900	976
Q76L83	136	Putative Polycomb group protein ASXL2	ASXL2	0.573112	1.65E-06	152.9	S	GSNKEGKKSRWKRKVSSSSPQSGCPSPTIPA	1380700	0	3705
Q9UFC0	253	Leucine-rich repeat and WD repeat-containing protein 1	LRWD1	0.572877	4.85E-10	152.88	S	VPLSLSPSKRACASPSAQVEGSPVAGSDGSQ	6404600	3093900	6308
Q8IY95	229	Transmembrane protein 192	TMEM192	0.873256	8.00E-06	152.85	S	AYPSNITSETGFRTISSLEEIVEKQGDTIEY	209280	203750	4129
P04637	315	Cellular tumor antigen p53	TP53	0.995983	2.35E-06	152.7	S	PPGSTKRALPNNTSSSPQPKKKPLDGEYFTL	57265000	48124000	882
Q86WR7	43	Proline and serine-rich protein 2	PROSER2	0.998771	7.73E-06	152.69	S	RGGSLESRSSSSRSRSFTLDDESLKYLTHEE	880390	642560	3994
Q08AD1	462	Calmodulin-regulated spectrin-associated protein 2	CAMSAP2	0.5	6.22E-06	152.68	S	EDSVQRSTPNRGITRSISNEGLTLNNSHVSK	2403200	1274100	2258
Q08AD1	464	Calmodulin-regulated spectrin-associated protein 2	CAMSAP2	0.991751	6.22E-06	152.68	S	SVQRSTPNRGITRSISNEGLTLNNSHVSKHI	1349000	633060	2259
O75376	1195	Nuclear receptor corepressor 1	NCOR1	0.644536	7.75E-06	152.67	S	EALVKGSISRMPIEDSSPEKGREEAASKGHV	797890	576760	602
Q4G0J3	258	La-related protein 7	LARP7	0.986614	2.50E-05	152.66	S	TSNTSISKMKRSRPTSEGSDIESTEPQKQCS	504040	375520	3091
Q96S99	227	Pleckstrin homology domain-containing family F member 1	PLEKHF1	1	5.09E-07	152.66	S	AAQQRQEEAEEQGAGSPGQPAHLARPICGAS	8777300	6193800	5119
P62987;P[illegible]57;57;57		Ubiquitin-60S ribosomal protein L40;Ubiquitin;60S ribosomal	UBA52;RPS2[illegible]	0.999604	4.54E-06	152.64	S	RLIFAGKQLEDGRTLSDYNIQKESTLHLVLR	727740	916070	978
P10071	664	Transcriptional activator GLI3;Transcriptional repressor GL	GLI3	0.976882	8.08E-08	152.64	S	RPPPPRDSGSHSQSRSPGRPTQGALGEQQDL	7283300	0	981
Q9H792	1036	Pseudopodium-enriched atypical kinase 1	PEAK1	0.99292	7.77E-06	152.64	S	ENALLQDSEKKRSHSSPSQIPKKILSHMTHE	1195800	75185	5755
Q9P289	4	Serine/threonine-protein kinase 26	STK26	1	2.56E-10	152.57	S	____________MAHSPVAVQVPGMQNNIAD	8582300	6913600	6226
Q9NZT2	519	Opioid growth factor receptor	OGFR	0.826869	2.10E-20	152.52	S	TEGRTGPKEGTPGSPSETPGPSPAGPAGDEP	2834900	3174200	6173
Q8IX90	119	Spindle and kinetochore-associated protein 3	SKA3	0.999782	7.41E-10	152.51	S	PRVKKNSVHEQEAINSDPELSNCENFQKTDV	4147900	4591200	4096
Q15831	31	Serine/threonine-protein kinase STK11	STK11	0.975619	8.35E-06	152.4	S	ELMSVGMDTFIHRIDSTEVIYQPRRKRAKLI	3057000	0	2989
Q6WCQ1	220	Myosin phosphatase Rho-interacting protein	MPRIP	0.99902	9.99E-11	152.38	S	QEEMRTKDQPDGSSLSPAQSPSQSQPPAASS	16227000	12045000	3630
Q6WCQ1	224	Myosin phosphatase Rho-interacting protein	MPRIP	0.993969	9.99E-11	152.38	S	RTKDQPDGSSLSPAQSPSQSQPPAASSLREP	11598000	9426900	3631
P49790	333	Nuclear pore complex protein Nup153	NUP153	0.744232	4.93E-06	152.34	S	MSSPLADAKRIPSIVSSPLNSPLDRSGIDIT	923390	0	1695
O75179	2042	Ankyrin repeat domain-containing protein 17	ANKRD17	0.839704	7.04E-08	152.28	S	ATYPMPTAKEHYPVSSPSSPSPPAQPGGVSR	3572100	8492900	582
O75179	2047	Ankyrin repeat domain-containing protein 17	ANKRD17	0.99995	7.04E-08	152.28	S	PTAKEHYPVSSPSSPSPPAQPGGVSRNSPLD	7108400	5371300	585
O94979	527	Protein transport protein Sec31A	SEC31A	1	9.38E-11	152.28	S	DGANVALKDSDQVAQSDGEESPAAEEQLLGE	10658000	15118000	723
Q6WKZ4	202	Rab11 family-interacting protein 1	RAB11FIP1	0.84926	7.18E-14	152.27	S	DTASAIIPSTTPSVDSDDESVVKDKKKKSKI	2331900	1168900	3635
Q6P2E9	879	Enhancer of mRNA-decapping protein 4	EDC4	0.922306	1.66E-14	152.17	S	HLLQQRDSQDASAEQSDHDDEVASLASASGG	9164200	0	3515
P35251	73	Replication factor C subunit 1	RFC1	0.788433	3.48E-06	152.16	S	QPSKKKRIIYDSDSESEETLQVKNAKKPPEK	2611500	0	1387
P19532	554	Transcription factor E3	TFE3	0.593771	5.43E-06	152.13	S	GALSPLRAASDPLLSSVSPAVSKASSRRSSF	0	835610	1167
P19532	560	Transcription factor E3	TFE3	1	5.43E-06	152.13	S	RAASDPLLSSVSPAVSKASSRRSSFSMEEES	864320	835610	1169
Q9UBB9	210	Tuftelin-interacting protein 11	TFIP11	1	1.55E-09	152.09	S	SERTTQSMQDFPVVDSEEEAEEEFQKELSQW	6780300	4334200	6258
Q5VUJ6	518	Leucine-rich repeat and calponin homology domain-containing	LRCH2	0.721765	7.74E-10	152.04	S	QTVECEKSVSADEVNSPLSPLTWQPLENQKD	1032300	0	3350
Q13428	171	Treacle protein	TCOF1	0.94659	5.32E-14	152.03	S	SPRKSAEPSANTTLVSETEEEGSVPAFGAAA	3118800	782410	2518
P06748	4	Nucleophosmin	NPM1	1	1.07E-06	152	S	____________MEDSMDMDMSPLRPQNYLF	996600	1300800	921
Q14BN4	452	Sarcolemmal membrane-associated protein	SLMAP	0.662909	3.25E-06	152	S	ENQTRAKESDFSDTLSPSKEKSSDDTTDAQM	560910	348380	2831
Q14814	192	Myocyte-specific enhancer factor 2D	MEF2D	1	8.25E-06	151.98	S	RLLSPQQPALQRNSVSPGLPQRPASAGAMLG	2204100	1442700	2813
Q96N67	1383	Dedicator of cytokinesis protein 7	DOCK7	0.998627	0.000599386	151.83	S	SCFEYKGKKVFERMNSLTFKKSKDMRAKLEE	0	1056200	5030
Q12906	504	Interleukin enhancer-binding factor 3	ILF3	0.962768	1.03E-19	151.75	T	KPAVVAPAPVVEAVSTPSAAFPSDATAEQGP	359740	1370000	7518
Q6P0N0	1008	Mis18-binding protein 1	MIS18BP1	0.999992	3.41E-06	151.73	S	TPLQHQRILLPSFQDSEDDDDILPNMDKNPT	1251000	1293200	3500
Q9Y4F5	969	Centrosomal protein of 170 kDa protein B	CEP170B	0.999912	2.77E-06	151.7	S	SDVDTASTVSLRSGKSGPSPTTPQPLRAQKE	0	603930	6917
Q9Y4F5	972	Centrosomal protein of 170 kDa protein B	CEP170B	0.999441	2.77E-06	151.7	S	DTASTVSLRSGKSGPSPTTPQPLRAQKEMSP	0	603930	6918
O43303	366	Centriolar coiled-coil protein of 110 kDa	CCP110	1	7.40E-06	151.66	S	NNVIKSLTGSYAKLPSPEPSMSPKMHRRRSR	1809100	1177100	355
O43303	370	Centriolar coiled-coil protein of 110 kDa	CCP110	0.990531	7.40E-06	151.66	S	KSLTGSYAKLPSPEPSMSPKMHRRRSRTSSA	3435000	732950	356
Q5THJ4	2435	Vacuolar protein sorting-associated protein 13D	VPS13D	0.991065	8.90E-06	151.66	S	IKKQNHVTPSRHRNSSSESAIVPKTVKSGVV	685140	0	3308
Q9UHB6	374	LIM domain and actin-binding protein 1	LIMA1	0.981174	8.90E-06	151.66	S	KPLSPDSRASSLESSSPPKAMKKFQAPARET	1461800	1424900	6328
Q9UQ35	2702	Serine/arginine repetitive matrix protein 2	SRRM2	1	2.37E-05	151.66	S	DSRSLSYSPVERRRPSPQPSPRDQQSSSSER	2316900	4023100	6677
Q9UQ35	2706	Serine/arginine repetitive matrix protein 2	SRRM2	1	2.37E-05	151.66	S	LSYSPVERRRPSPQPSPRDQQSSSSERGSRR	5880800	6983100	6678
Q14676	780	Mediator of DNA damage checkpoint protein 1	MDC1	0.999999	3.24E-10	151.64	S	TQPFDTHLEAYGPCLSPPRAIPGDQHPESPV	4255500	2967200	2765
P04920	113	Anion exchange protein 2	SLC4A2	0.935589	1.21E-14	151.6	S	TPQGPGRKPRRRPGASPTGETPTIEEGEEDE	0	962400	891
Q6P2E9	723	Enhancer of mRNA-decapping protein 4	EDC4	0.958081	8.89E-20	151.6	S	SLELQEVEPLGLPQASPSRTRSPDVISSAST	17407000	9398900	3518
P04920	115	Anion exchange protein 2	SLC4A2	0.469096	1.21E-14	151.6	T	QGPGRKPRRRPGASPTGETPTIEEGEEDEDE	0	0	7260
Q06413	222	Myocyte-specific enhancer factor 2C	MEF2C	1	3.77E-06	151.59	S	GAGTSAGNGYGNPRNSPGLLVSPGNLNKNMQ	1915100	1152700	2202
Q8NHQ9	594	ATP-dependent RNA helicase DDX55	DDX55	0.999998	2.60E-06	151.55	S	LTTGKRTIKTVDLGISDLEDDC_________	1097100	569590	4419

Q9NXE8	218	Pre-mRNA-splicing factor CWC25 homolog	CWC25	0.877504	8.56E-06	151.55	S	DEHSAGRSQKKMANSSPVLSKVPGYGLQVRN	1188800	1104500	6078
P85037	416	Forkhead box protein K1	FOXK1	0.999994	4.41E-06	151.49	S	GVSCFRTPFGPLSSRSAPASPTHPGLMSPRS	590820	6076600	1997
Q13595	14	Transformer-2 protein homolog alpha	TRA2A	1	3.14E-06	151.49	S	__MSDVEENNFEGRESRSQSKSPTGTPARVK	2757200	3953100	2589
Q9Y618	2258	Nuclear receptor corepressor 2	NCOR2	0.999801	3.80E-06	151.49	S	YRDGEQTEPSRMGSKSPGNTSQPPAFFSKLT	2098800	1389400	6984
Q5VTL8	5	Pre-mRNA-splicing factor 38B	PRPF38B	0.999976	9.52E-21	151.44	S	___________MANNSPALTGNSQPQHQAAA	4474500	4861600	3337
P67809	209	Nuclease-sensitive element-binding protein 1	YBX1	0.853325	5.96E-14	151.42	S	PPYYMRRPYGRRPQYSNPPVQGEVMEGADNQ	0	1574600	1942
Q9C0C2	691	182 kDa tankyrase-1-binding protein	TNKS1BP1	0.939318	7.25E-06	151.39	S	PGPESSSRWLDDLLASPPPSGGGARRGAGAE	825150	548250	5527
Q9H0C8	13	Integrin-linked kinase-associated serine/threonine phosphata	ILKAP	1	4.41E-06	151.39	S	___MDLFGDLPEPERSPRPAAGKEAQKGPLL	2247500	1759500	5566
Q9BVG9	16	Phosphatidylserine synthase 2	PTDSS2	1	2.41E-05	151.38	S	MRRGERRDAGGPRPESPVPAGRASLEEPPDG	0	2835600	5402
P55196	1799	Afadin	MLLT4	0.999999	9.24E-06	151.22	S	GAPENLTFKERQRLFSQGQDVSNKVKASRKL	5389300	6558000	1867
Q6GYQ0	773	Ral GTPase-activating protein subunit alpha-1	RALGAPA1	0.436241	5.77E-10	151.22	S	DDAQILPRSTRVRHFSQSEETGNEVFGALNE	0	0	3462
Q6GYQ0	775	Ral GTPase-activating protein subunit alpha-1	RALGAPA1	0.436241	5.77E-10	151.22	S	AQILPRSTRVRHFSQSEETGNEVFGALNEEQ	0	0	3463
Q96HB5	360	Coiled-coil domain-containing protein 120	CCDC120	0.996073	9.24E-06	151.22	S	SDRASLFVARTRRSNSSEALLVDRAAGGGAG	455220	322810	4942
Q9H4L7	124	SWI/SNF-related matrix-associated actin-dependent regulator	SMARCAD1	0.999994	1.39E-09	151.21	S	TVQEKTFNKDTVIIVSEPSEDEESQGLPTMA	10977000	12796000	5683
Q9H4L7	127	SWI/SNF-related matrix-associated actin-dependent regulator	SMARCAD1	0.999997	1.39E-09	151.21	S	EKTFNKDTVIIVSEPSEDEESQGLPTMARRN	4922000	5147700	5684
Q9Y294	165	Histone chaperone ASF1A	ASF1A	0.782355	3.35E-10	151.21	S	HINWEDNTEKLEDAESSNPNLQSLLSTDALP	2373700	0	6771
Q9Y294	166	Histone chaperone ASF1A	ASF1A	0.764673	3.35E-10	151.21	S	INWEDNTEKLEDAESSNPNLQSLLSTDALPS	792100	913940	6772
Q6IBW4	284	Condensin-2 complex subunit H2	NCAPH2	1	8.84E-06	151.16	S	EASAPKAALEPKESRSPQQSAALPRRYMLRE	9577000	6066500	3468
Q9NXR1	282	Nuclear distribution protein nudE homolog 1	NDE1	1	8.87E-06	151.13	S	LESKLASCRNLVYDQSPNRTGGPASGRSSKN	6249100	3919300	6081
Q13428	375	Treacle protein	TCOF1	0.99478	3.21E-05	151.11	S	LQAKASGKTSQVGAASAPAKESPRKGAAPAP	0	0	2502
P41208	20	Centrin-2	CETN2	1	1.58E-05	151.07	S	FKKANMASSSQRKRMSPKPELTEEQKQEIRE	3351400	3295900	1483
P42694	1763	Probable helicase with zinc finger domain	HELZ	0.46281	5.85E-10	151.05	S	YEPRGPGRPLYQRRISSSSVQPCSEEVSTPQ	0	0	1512
P42694	1764	Probable helicase with zinc finger domain	HELZ	0.46281	5.85E-10	151.05	S	EPRGPGRPLYQRRISSSSVQPCSEEVSTPQD	0	0	1513
P42694	1765	Probable helicase with zinc finger domain	HELZ	0.493139	5.85E-10	151.05	S	PRGPGRPLYQRRISSSSVQPCSEEVSTPQDS	0	0	1514
A4D1S0	143	Killer cell lectin-like receptor subfamily G member 2	KLRG2	0.999944	6.30E-05	151.03	S	VRVKPVGAAGGSSTPSPRPSTRFLKVPVPES	892150	0	33
A4D1S0	141	Killer cell lectin-like receptor subfamily G member 2	KLRG2	0.922586	6.30E-05	151.03	T	VDVRVKPVGAAGGSSTPSPRPSTRFLKVPVP	892150	0	7110
Q5VV41	227	Rho guanine nucleotide exchange factor 16	ARHGEF16	0.581995	5.92E-10	150.91	S	DPQLYQEIQERGLNTSQESDDDILDESSSPE	0	1172300	3352
O43491	386	Band 4.1-like protein 2	EPB41L2	1	7.25E-06	150.9	S	LEEKVAELHKTHRGLSPAQADSQFLENAKRL	1129700	777450	392
P24928	1849	DNA-directed RNA polymerase II subunit RPB1	POLR2A	0.71898	3.65E-07	150.9	S	PASPKYTPTSPSYSPSSPEYTPTSPKYSPTS	1469800	380650	1256
Q13573	224	SNW domain-containing protein 1	SNW1	1	4.63E-06	150.9	S	PPRFKINKKIPRGPPSPPAPVMHSPSRKMTV	85842000	41596000	2581
Q13573	232	SNW domain-containing protein 1	SNW1	0.991049	4.63E-06	150.9	S	KIPRGPPSPPAPVMHSPSRKMTVKEQQEWKI	81496000	37921000	2582
Q14160	1220	Protein scribble homolog	SCRIB	1	9.48E-06	150.9	S	GVIANPFAAGIGHRNSLESISSIDRELSPEG	9276100	5187000	2684
Q9UHF7	1081	Zinc finger transcription factor Trps1	TRPS1	0.997733	2.28E-06	150.9	S	SPIEKYMRPAKHPNYSPPGSPIEKYQYPLFG	1538700	1024300	6352
Q9UHF7	1085	Zinc finger transcription factor Trps1	TRPS1	0.99998	2.28E-06	150.9	S	KYMRPAKHPNYSPPGSPIEKYQYPLFGLPFV	1538700	1024300	6353
Q9ULM3	473	YEATS domain-containing protein 2	YEATS2	0.999862	6.27E-06	150.9	S	SCKIVSGSPISTPSPSPLPRTPTSTPVHVKQ	0	971660	6498
Q9BUR4	489	Telomerase Cajal body protein 1	WRAP53	0.5	1.36E-09	150.69	T	PLLATASGQRVFPEPTESGDEGEELGLPLLS	2691400	2369900	8053
O60264	66	SWI/SNF-related matrix-associated actin-dependent regulator	SMARCA5	1	1.17E-20	150.62	S	AGPADAEMEEIFDDASPGKQKEIQEPDPTYE	2301500	985730	451
Q9HC35	978	Echinoderm microtubule-associated protein-like 4	EML4	0.727791	4.10E-06	150.57	S	KEQAKATLLEDQQDPSPSS____________	0	422250	5841
Q96T37	294	Putative RNA-binding protein 15	RBM15	1	3.67E-06	150.51	S	GHRHPPGGGGGQRSLSPGGAALGYRDYRLQQ	25710000	8806400	5165
O94876	382	Transmembrane and coiled-coil domains protein 1	TMCC1	1	9.80E-06	150.49	S	SKPREIASLIRNKFGSADNIPNLKDSLEEGQ	1467300	902950	709
Q9UID3	18	Vacuolar protein sorting-associated protein 51 homolog	VPS51	0.99864	2.64E-14	150.36	S	AAAAAGPSPGSGPGDSPEGPEGEAPERRRKA	7726300	0	6378
P58004	249	Sestrin-2	SESN2	0.999995	2.88E-06	150.26	S	PSEQSSPPSRDPLNNSGGFESARDVEALMER	1681400	1824500	1899
Q9NRY4	975	Rho GTPase-activating protein 35	ARHGAP35	0.997272	7.35E-10	150.14	S	ACSTTEEVFNSPRAGSPLCNSNLQDSEEDIE	736330	2482100	5985
Q8TE77	9	Protein phosphatase Slingshot homolog 3	SSH3	0.984234	3.12E-07	150.13	S	_______MALVTVSRSPPGSGASTPVGPWDQ	1259700	795900	4474
Q99733	125	Nucleosome assembly protein 1-like 4	NAP1L4	1	4.58E-10	150.13	S	ITGDVEPTDAESEWHSENEEEEKLAGDMKSK	100440000	116310000	5271
Q70CQ2	3406	Ubiquitin carboxyl-terminal hydrolase 34	USP34	0.993199	1.57E-07	150.12	S	RDSSIIDPGTEQDLPSPENSSVKEYRMEVPS	817130	484630	3679
Q8TD19	331	Serine/threonine-protein kinase Nek9	NEK9	0.456265	5.92E-06	150.12	S	EEKVTLLNAPTKRPRSSTVTEAPIAVVTSRT	0	0	4464
Q8TEV9	639	Smith-Magenis syndrome chromosomal region candidate gene 8 p	SMCR8	0.95529	2.92E-06	150.12	S	QGFRVDFSVENANPSSRDNSCEGFPAYELDP	1225800	1019600	4491
P17812	571	CTP synthase 1	CTPS1	0.734792	1.55E-05	150.11	S	QKGCRLSPRDTYSDRSGSSSPDSEITELKFP	0	795050	1120
O14686	4849	Histone-lysine N-methyltransferase 2D	KMT2D	0.999999	9.61E-06	150.09	S	EAEGPGGKEKGLEGKSPDTGPDWLKQFDAVL	686030	890930	198
O60716	920	Catenin delta-1	CTNND1	1	1.01E-05	150.09	S	STPNERGDHNRTLDRSGDLGDMEPLKGTTPL	2748200	1935400	516
Q5JSZ5	613	Protein PRRC2B	PRRC2B	1	9.61E-06	150.09	S	VAQSNSSEEEAREAGSPAQEFKYQKSLPPRF	0	0	3188
Q9Y520	2686	Protein PRRC2C	PRRC2C	0.918547	7.48E-05	150.09	S	PGTPPIAGRSTTPTSSPFRATSTSPNSQSSK	1691800	1311200	6950
Q9Y520	2682	Protein PRRC2C	PRRC2C	0.999982	7.48E-05	150.09	T	IDIKPGTPPIAGRSTTPTSSPFRATSTSPNS	4576000	3099200	8307
Q9NZT2	541	Opioid growth factor receptor	OGFR	0.950137	3.73E-20	150.06	T	PAGPAGDEPAESPSETPGPRPAGPAGDEPAE	2448700	0	8183
Q86YR5	492	G-protein-signaling modulator 1	GPSM1	0.916248	5.95E-06	150.05	S	DVRVHVPRTSIPRAPSSDEECFFDLLTKFQS	620190	564820	4026
Q08AD1	862	Calmodulin-regulated spectrin-associated protein 2	CAMSAP2	0.999135	9.65E-06	150.04	S	DIKESMENPQAKWLKSPTTPIDPEKQWNLAS	435790	350090	2260
Q9BQG0	1163	Myb-binding protein 1A	MYBBP1A	0.999921	3.81E-06	150.04	S	KLEKKDAKEIPSATQSPISKKRKKKGFLPET	18627000	11471000	5309
Q96BY7	1585	Autophagy-related protein 2 homolog B	ATG2B	0.41365	4.96E-06	150.01	S	PTSPAKSYISPHSSPSHTPTRHGRNTVCGGK	0	0	4815
P68363;P(	340;340	Tubulin alpha-1B chain;Tubulin alpha-4A chain	TUBA1B;TUB/	1	2.95E-06	149.97	S	PKDVNAAIATIKTKRSIQFVDWCPTGFKVGI	1535300	1041100	1945
P49327	2204	Fatty acid synthase;[Acyl-carrier-protein] S-acetyltransfera	FASN	1	1.20E-05	149.96	T	ELSSKADEASELACPTPKEDGLAQQQTQLNL	0	1068700	7411
Q9H2H9	54	Sodium-coupled neutral amino acid transporter 1	SLC38A1	0.726318	2.32E-05	149.94	T	INSKFISDRESRRSLTNSHLEKKKCDEYIPG	14013000	70634	8092
O15173	208	Membrane-associated progesterone receptor component 2	PGRMC2	0.97118	4.43E-05	149.93	S	KYDYVGRLLKPGEEPSEYTDEEDTKDHNKQD	1839000	2479300	300
Q96B23	66	Uncharacterized protein C18orf25	C18orf25	0.993335	7.05E-07	149.93	S	VISSMPCLLMELRRDSSESQLASTESDKPTT	4414000	3501600	4792
Q9BUA3	251	Uncharacterized protein C11orf84	C11orf84	0.91377	5.38E-10	149.93	S	MTKNLDPDPEPPSPDSPTETFAAPAEVRHFT	17962000	11383000	5377
Q9BUA3	253	Uncharacterized protein C11orf84	C11orf84	0.889996	5.38E-10	149.93	T	KNLDPDPEPPSPDSPTETFAAPAEVRHFTDG	2792400	11383000	8050
P46821	1792	Microtubule-associated protein 1B;MAP1B heavy chain;MAP1 lig	MAP1B	0.821589	4.22E-06	149.86	S	KLSPKSDISPLTPRESSPLYSPTFSDSTSAV	6368200	0	1580
P46821	1797	Microtubule-associated protein 1B;MAP1B heavy chain;MAP1 lig	MAP1B	0.995542	4.22E-06	149.86	S	SDISPLTPRESSPLYSPTFSDSTSAVKEKTA	3529500	3787400	1582
Q14839	1549	Chromodomain-helicase-DNA-binding protein 4	CHD4	0.934298	1.49E-10	149.85	T	GSPSPKTPTPSTPGDTQPNTPAPVPPAEDGI	7637100	12979000	7623
Q96RL1	653	BRCA1-A complex subunit RAP80	UIMC1	1	3.52E-06	149.82	S	DADIKSSETGAFRVPSPGMEEAGCSREMQSS	0	0	5097
P12270	2034	Nucleoprotein TPR	TPR	0.615977	3.75E-10	149.8	S	EESMGGGEGNHRAADSQNSGEGNTGAAESSF	0	2172900	1034
Q9UMZ2	935	Synergin gamma	SYNRG	0.970765	8.26E-06	149.73	S	PSATSILQKKETSFGSSENITMTSLSKVTTF	1562600	2095000	6523
O15173	210	Membrane-associated progesterone receptor component 2	PGRMC2	0.925265	1.25E-05	149.72	Y	DYVGRLLKPGEEPSEYTDEEDTKDHNKQD__	35449000	32284000	8345
O14686	609	Histone-lysine N-methyltransferase 2D	KMT2D	0.998573	4.73E-14	149.67	S	PEASRLFPPFEESPLSPPPEESPLSPPPEAS	2153900	1018200	192
O14686	618	Histone-lysine N-methyltransferase 2D	KMT2D	0.997173	4.73E-14	149.67	S	FEESPLSPPPEESPLSPPPEASRLSPPPEDS	0	1018200	193
O95365	525	Zinc finger and BTB domain-containing protein 7A	ZBTB7A	0.5	1.16E-10	149.67	S	PSPGATATPGAPAQPSSPDARRNGQEKHFKD	23241000	9198400	756
Q14160	1306	Protein scribble homolog	SCRIB	1	4.73E-14	149.67	S	KMAESPCSPSGQQPPSPPSPDELPANVKQAY	10677000	6043700	2682
Q14160	1309	Protein scribble homolog	SCRIB	1	4.73E-14	149.67	S	ESPCSPSGQQPPSPPSPDELPANVKQAYRAF	16084000	8738900	2683
Q8NCP5	161	Zinc finger and BTB domain-containing protein 44	ZBTB44	0.981978	8.00E-06	149.6	S	ENNSNCNFTSRDGSISPVSSECSVVERTIPV	1510800	0	4331
Q9HCH0	494	Nck-associated protein 5-like	NCKAP5L	0.998217	4.41E-06	149.6	S	NSRIPCRNSGSDGSPSPLLARRGLGGGELSP	793760	759510	5860
O95644	233	Nuclear factor of activated T-cells, cytoplasmic 1	NFATC1	1	3.94E-06	149.57	S	EEGFPRGLGACTLLGSPRHSPSTSPRASVTE	1245200	582310	787
Q13428	1350	Treacle protein	TCOF1	1	4.10E-05	149.57	S	KESSRKGWESRKRKLSGDQPAARTPRSKKKK	32418000	15270000	2507
Q6P597	497	Kinesin light chain 3	KLC3	0.792227	3.94E-06	149.57	S	FPSWHLDKAPRTLSASTQDLSPH________	854550	497540	3530
Q96JM3	282	Chromosome alignment-maintaining phosphoprotein 1	CHAMP1	0.996648	2.85E-07	149.52	S	SRKSARTTSPEPRKPSPSESPEPWKPFPAVS	12320000	8652800	4976
Q96JM3	286	Chromosome alignment-maintaining phosphoprotein 1	CHAMP1	0.996648	2.85E-07	149.52	S	ARTTSPEPRKPSPSESPEPWKPFPAVSPEPR	11413000	7274700	4978
Q96JM3	297	Chromosome alignment-maintaining phosphoprotein 1	CHAMP1	1	2.85E-07	149.52	S	SPSESPEPWKPFPAVSPEPRRPAPAVSPGSW	11413000	7274700	4979
O95391	215	Pre-mRNA-splicing factor SLU7	SLU7	1	5.87E-06	149.49	S	LQEELASGKLVEQANSPKHQWGEEEPNSQME	4203000	3538200	759
Q8N6H7	146	ADP-ribosylation factor GTPase-activating protein 2	ARFGAP2	1	2.03E-07	149.42	S	TDLWIDNMSSAVPNHSPEKKDSDFFTEHTQP	0	0	4291
P46108	42	Adapter molecule crk	CRK	0.480864	2.60E-06	149.42	T	LQGQRHGVFLVRDSSTSPGDYVLSVSENSRV	0	0	7384
Q15831	32	Serine/threonine-protein kinase STK11	STK11	0.771427	4.34E-05	149.38	T	LMSVGMDTFIHRIDSTEVIYQPRRKRAKLIG	0	1618500	7651
Q7Z3C6	828	Autophagy-related protein 9A	ATG9A	1	3.31E-07	149.36	S	GQSASRHPEPVPEEGSEDELPPQVHKV____	4019100	2001800	3778
Q13425	110	Beta-2-syntrophin	SNTB2	1	3.09E-06	149.35	S	SPPAPPRGPAGEAGASPPVRRVRVVKQEAGG	5670400	3499100	2485
Q9H7N4	239	Splicing factor, arginine/serine-rich 19	SCAF1	0.978044	9.58E-10	149.34	S	RFDIYDPFHPTDEAYSPPPAPEQKYDPFEPT	0	5592100	5768
Q13111	951	Chromatin assembly factor 1 subunit A	CHAF1A	0.999896	1.07E-05	149.33	S	GGGVGVDTGKATLTASPLGAS__________	2600600	1917200	2399
Q86TV6	677	Tetratricopeptide repeat protein 7B	TTC7B	0.99991	7.87E-08	149.33	S	SVAASRVEQALSEVASSLQSSAPKQGPLHPW	376020	335060	3908
Q86TV6	678	Tetratricopeptide repeat protein 7B	TTC7B	0.9999	7.87E-08	149.33	S	VAASRVEQALSEVASSLQSSAPKQGPLHPWM	376020	335060	3909
Q8IV63	59	Inactive serine/threonine-protein kinase VRK3	VRK3	0.994949	1.07E-05	149.33	S	SFQGSKRGLNSSFETSPKKVKWSSTVTSPRL	1320800	1049700	4041
Q14157	416	Ubiquitin-associated protein 2-like	UBAP2L	0.998719	3.10E-06	149.31	S	LVQYDLKNPSDSAVHSPFTKRQAFTPSSTMM	3654100	4116100	2659
Q5T0N5	295	Formin-binding protein 1-like	FNBP1L	0.999994	1.08E-05	149.23	S	DFPFEDYSQHIYRTISDGTISASKQESGKMD	1834900	706140	3240
Q7Z2Z1	441	Treslin	TICRR	1	4.04E-06	149.22	S	EAEFQRHVLQTAVADSPRDTASLFSDVVDSI	0	0	3764
P16949	16	Stathmin	STMN1	1	2.80E-06	149.18	S	MASSDIQVKELEKRASGQAFELILSPRSKES	1428400	1086900	1093
P16949	25	Stathmin	STMN1	1	2.80E-06	149.18	S	ELEKRASGQAFELILSPRSKESVPEFPLSPP	1428400	1086900	1094
Q13426	233	DNA repair protein XRCC4	XRCC4	0.940031	6.80E-14	149.18	T	CSEMTADRDPVYDESTDEESENQTDLSGLAS	5276300	6404200	7532
Q13426	229	DNA repair protein XRCC4	XRCC4	0.578462	6.80E-14	149.18	Y	ETAICSEMTADRDPVYDESTDEESENQTDLS	5276300	0	8376
Q8IW50	115	Protein FAM219A	FAM219A	0.954417	2.68E-14	149.16	S	DQSPDEKPLVALDTDSDDDFDMSRYSSSGYS	5772200	4806600	4053
O14639	431	Actin-binding LIM protein 1	ABLIM1	0.968649	6.38E-06	149.09	S	DKQERQSLGESPRTLSPTPSAEGYQDVRDRM	1181000	1279900	164
O60333	1454	Kinesin-like protein KIF1B	KIF1B	0.999813	1.10E-05	148.89	S	TGIYELSLCKMSDTGSPGMQRRRRKILDTSV	1283400	702290	482
P35612	530	Beta-adducin	ADD2	0.99232	1.10E-05	148.89	S	PQSQLLASVIAEKSRSPSTESQLMSKGDEDT	636890	500260	1413
Q8IYB3	713	Serine/arginine repetitive matrix protein 1	SRRM1	0.997727	3.92E-05	148.89	S	PPVRRGASSSPQRRQSPSPSTRPIRRVSRTP	26932000	0	4149
Q8IYB3	715	Serine/arginine repetitive matrix protein 1	SRRM1	0.951043	3.92E-05	148.89	S	VRRGASSSPQRRQSPSPSTRPIRRVSRTPEP	800160	19027000	4150
Q8TAP9	133	M-phase-specific PLK1-interacting protein	MPLKIP	1	1.05E-05	148.89	S	TSTPFGSGRVREKRMSNELENYFKPSMLEDP	2256100	1677800	4441
Q8IYB3	402	Serine/arginine repetitive matrix protein 1	SRRM1	1	3.64E-05	148.85	S	RLSPSASPPRRRHRPSPPATPPPKTRHSPTP	53681000	20630000	4137
Q8IYB3	406	Serine/arginine repetitive matrix protein 1	SRRM1	1	3.64E-05	148.85	T	SASPPRRRHRPSPPATPPPKTRHSPTPQQSN	53681000	20630000	7860
Q9UHD8	42	Septin-9	SEPT9	0.814933	3.48E-05	148.7	T	LKRSFEVEEVETPNSTPPRRVQTPLLRATVA	798540	489110	8214
Q9UHB6	490	LIM domain and actin-binding protein 1	LIMA1	0.999926	6.63E-06	148.52	S	LERPAQLANARETPHSPGVEDAPIAKVGVLA	7008200	5806300	6330
O75179	2044	Ankyrin repeat domain-containing protein 17	ANKRD17	0.88557	1.48E-07	148.44	S	YPMPTAKEHYPVSSPSSPSPPAQPGGVSRNS	3572100	1215700	583
Q86WB0	359	Nuclear-interacting partner of ALK	ZC3HC1	0.980059	2.96E-07	148.36	S	SPGPIVSRTRSWDSSSPVDRPEPEAASPTTR	13007000	7266400	3988
Q02952	283	A-kinase anchor protein 12	AKAP12	0.79943	4.70E-06	148.33	S	EGEEKQEKEPSKSAESPTSPVTSETGSTFKK	778770	660470	2118
Q9HB90	2	Ras-related GTP-binding protein C	RRAGC	0.999912	8.56E-10	148.3	S	______________MSLQYGAEETPLAGSYG	4420600	1331900	5835
Q9HB90	15	Ras-related GTP-binding protein C	RRAGC	0.999442	8.56E-10	148.3	S	_MSLQYGAEETPLAGSYGAADSFPKDFGYGV	1807100	1994900	5836
Q9H1E3	181	Nuclear ubiquitous casein and cyclin-dependent kinase subst	NUCKS1	1	7.04E-06	148.28	S	EKKMPKPRLKATVTPSPVKGKGKVGRPTASK	97934000	55124000	5600
Q9H1E3	179	Nuclear ubiquitous casein and cyclin-dependent kinase subst	NUCKS1	1	4.96E-05	148.28	T	RKEKKMPKPRLKATVTPSPVKGKGKVGRPTA	4903100	7295000	8087
Q13620	145	Cullin-4B	CUL4B	0.81585	4.57E-10	148.2	S	FDAKMAEESSSSSSSSSPTAATSQQQQLKNK	0	0	2615
Q5T1M5	1114	FK506-binding protein 15	FKBP15	1	2.24E-14	148.2	S	TSDPEEGDPLALGPESPGEPQPPQLKKDDVT	8782300	9555000	3247
Q9NVP2	179	Histone chaperone ASF1B	ASF1B	1	1.63E-09	148.2	T	ETQDPSLGCGLPLNCTPIKGLGLPGCIPGLL	1920400	1557700	8171
P13861	99	cAMP-dependent protein kinase type II-alpha regulatory subu	PRKAR2A	0.999777	7.22E-07	148.19	S	EDLEVPVPSRFNRRVSVCAETYNPDEEEEDT	5595900	5384800	1058
P48634	1089	Protein PRRC2A	PRRC2A	0.999475	1.16E-05	148.18	S	NHPPAPRGRTASETRSEGSEYEEIPKRRRQR	579840	880230	1631
Q9Y2J2	960	Band 4.1-like protein 3;Band 4.1-like protein 3, N-terminal	EPB41L3	0.988636	1.43E-06	148.18	S	HFESSTVKTETISFGSVSPGGVKLEISTKEV	0	0	6784
Q9UQ35	387	Serine/arginine repetitive matrix protein 2	SRRM2	0.997168	3.81E-14	148.14	S	ERHGGSPQPLATTPLSQEPVNPPSEASPTRD	15522000	19302000	6641
Q01813	386	ATP-dependent 6-phosphofructokinase, platelet type	PFKP	1	1.10E-05	148.13	S	MDERRFQDAVRLRGRSFAGNLNTYKRLAIKL	2258800	645390	2080
P46821	1076	Microtubule-associated protein 1B;MAP1B heavy chain;MAP1 lig	MAP1B	1	6.48E-06	148.04	S	QYGFLTTPTKQLGAQSPGREPASSIHDETLP	904920	873960	1590
P06454	9	Prothymosin alpha;Prothymosin alpha, N-terminally processed;	PTMA	0.454908	2.11E-06	147.91	S	_______MSDAAVDTSSEITTKDLKEKKEVV	0	0	916
P06454	10	Prothymosin alpha;Prothymosin alpha, N-terminally processed;	PTMA	0.454908	2.11E-06	147.91	S	______MSDAAVDTSSEITTKDLKEKKEVVE	0	0	917
Q13177	141	Serine/threonine-protein kinase PAK 2;PAK-2p27;PAK-2p34	PAK2	0.999797	6.54E-06	147.91	S	VLKFYDSNTVKQKYLSFTPPEKDGFPSGTPA	4986300	3314000	2430
Q9H9J4	754	Ubiquitin carboxyl-terminal hydrolase 42	USP42	1	1.00E-13	147.87	S	ERGPPEDRDAEPQPGSPAAESLEEPDAAAGL	654920	430750	5801
Q5JSH3	161	WD repeat-containing protein 44	WDR44	0.735558	1.85E-07	147.84	S	TCEKPVDETTKLTQTSSTEQLNVLETETEVL	3250500	0	3183
P36871	115	Phosphoglucomutase-1	PGM1	0.967831	1.67E-09	147.84	T	SCIIRKIKAIGGIILTASHNPGGPNGDFGIK	7486900	5105000	7369
Q9Y4H2	388	Insulin receptor substrate 2	IRS2	1	5.77E-06	147.83	S	AGAAAAGARPVSVAGSPLSPGPVRAPLSRSH	1236100	1046100	6925
P24928	1875	DNA-directed RNA polymerase II subunit RPB1	POLR2A	0.813894	4.16E-06	147.75	S	YSPTSPKYSPTSPKYSPTSPTYSPTTPKYSP	2749100	972690	1250

Q00839	4	Heterogeneous nuclear ribonucleoprotein U	HNRNPU	0.988721	2.04E-05	147.75	S	____________MSSSPVNVKKLKVSELKEE	73781000	66706000	2039
Q8NCD3	473	Holliday junction recognition protein	HJURP	1	6.97E-06	147.75	S	LPDSWAMNMYRGGPASPGGLQGLETRRLSLP	2606400	2860600	4325
Q92547	888	DNA topoisomerase 2-binding protein 1	TOPBP1	0.996386	4.46E-06	147.75	S	LALANSSRNAVALSASPQLKEAQSEKEEAPK	2182600	3598100	4632
P52701	137	DNA mismatch repair protein Msh6	MSH6	0.987827	4.48E-06	147.7	S	EKGKSVRVHVQFFDDSPTRGWVSKRLLKPYT	1042800	1027700	1818
Q01850	309	Cerebellar degeneration-related protein 2	CDR2	0.329764	9.49E-06	147.7	S	MFLTVPESHRKPLKRSSSETILSSLAGSDIV	0	0	2085
Q01850	310	Cerebellar degeneration-related protein 2	CDR2	0.329764	9.49E-06	147.7	S	FLTVPESHRKPLKRSSSETILSSLAGSDIVK	0	0	2086
Q01850	311	Cerebellar degeneration-related protein 2	CDR2	0.329764	9.49E-06	147.7	S	LTVPESHRKPLKRSSSETILSSLAGSDIVKG	0	0	2087
P42166	66	Lamina-associated polypeptide 2, isoform alpha;Thymopoietin;	TMPO	1	1.69E-10	147.67	S	PPLPAGTNSKGPPDFSSDEEREPTPVLGSGA	54654000	15403000	1498
P42166	67	Lamina-associated polypeptide 2, isoform alpha;Thymopoietin;	TMPO	1	1.69E-10	147.67	S	PLPAGTNSKGPPDFSSDEEREPTPVLGSGAA	46132000	15403000	1499
Q92797	1168	Symplekin	SYMPK	0.525756	2.08E-06	147.64	S	LEEEQKLKPGGVGAPSSSSPSPSPSARPGPP	0	0	4708
Q9H040	268	SprT-like domain-containing protein Spartan	SPRTN	1	7.03E-06	147.62	S	FSGKGYVLGETSNLPSPGKLITSHAINKTQD	636360	586360	5556
O94842	181	TOX high mobility group box family member 4	TOX4	0.746116	5.83E-06	147.6	S	AQSPEDRLSTTPSPTSSLHEDGVEDFRRQLP	0	0	705
Q86TN4	239	tRNA 2'-phosphotransferase 1	TRPT1	0.676101	2.43E-05	147.6	S	RKPLSLAGDEETECQSSPKHSSRERRRIQQ_	0	0	3904
Q14157	607	Ubiquitin-associated protein 2-like	UBAP2L	0.497027	1.20E-05	147.58	S	YEQRSTQTRRYPSSISSSPQKDLTQAKNGFS	0	0	2661
Q14157	608	Ubiquitin-associated protein 2-like	UBAP2L	0.582648	1.20E-05	147.58	S	EQRSTQTRRYPSSISSSPQKDLTQAKNGFSS	0	0	2662
Q96F86	161	Enhancer of mRNA-decapping protein 3	EDC3	0.999976	6.68E-06	147.58	S	MESLSQSKSFRRRHNSWSSSSRHPNQATPKK	1269900	729500	4902
Q8NDT2	109	Putative RNA-binding protein 15B	RBM15B	0.99993	3.48E-06	147.56	S	GRGGKASGDPGASGMSPRASPLPPPPPPPGA	1333800	977530	4356
Q13247	297	Serine/arginine-rich splicing factor 6	SRSF6	1	0.000125326	147.53	S	KENGKGDIKSKSRSRSQSRSNSPLPVPPSKA	2242400	1032300	2443
Q13247	299	Serine/arginine-rich splicing factor 6	SRSF6	1	0.000125326	147.53	S	NGKGDIKSKSRSRSQSRSNSPLPVPPSKARS	2242400	2398600	2444
P35659	303	Protein DEK	DEK	0.794796	5.88E-06	147.52	S	STTKKNQNSSKKESESEDSSDDEPLIKKLKK	0	0	1421
Q01167	30	Forkhead box protein K2	FOXK2	1	5.24E-27	147.52	S	PAGGGAGGGGAGGGGSPPGGWAVARLEGREF	0	4105500	2059
Q13136	666	Liprin-alpha-1	PPFIA1	0.999957	1.21E-05	147.52	S	ENTEQRAEEIESRVGSGSLDNLGRFRSMSSI	1087100	812390	2419
Q5T200	242	Zinc finger CCCH domain-containing protein 13	ZC3H13	0.916942	3.49E-06	147.52	S	SSSASKKDRKTSAVSSPLLDQQRNSKTNQSK	0	0	3277
Q6WKZ4	545	Rab11 family-interacting protein 1	RAB11FIP1	1	4.53E-06	147.52	S	PRAPQTRAVKPRLEVSPEAQPTARLPSPTDS	1109200	607710	3638
Q92793	2063	CREB-binding protein	CREBBP	0.999559	4.53E-06	147.52	S	VAGPRMPSVQPPRSISPSALQDLLRTLKSPS	1636600	855740	4704
Q9Y519	403	Transmembrane protein 184B	TMEM184B	1	1.15E-05	147.52	S	SLSGARDNEKTLLLSSDDEF___________	1982000	2077800	6943
Q01167	13	Forkhead box protein K2	FOXK2	0.999985	5.24E-27	147.52	T	___MAAAAAALSGAGTPPAGGGAGGGGAGGG	0	2971600	7474
P08621	226	U1 small nuclear ribonucleoprotein 70 kDa	SNRNP70	1	2.21E-06	147.51	S	SGRDDTSRYDERPGPSPLPHRDRDRDRERER	597600	485110	959
O15014	470	Zinc finger protein 609	ZNF609	0.956382	5.89E-06	147.49	S	EDSKGSKRVRTNSMGSATGPLPGTKVEPTVL	806630	383610	235
Q5JRA6	1906	Melanoma inhibitory activity protein 3	MIA3	1	1.20E-07	147.49	S	ASQSTSQDCSQALKQSP______________	6168600	2946300	3173
Q6PD62	1020	RNA polymerase-associated protein CTR9 homolog	CTR9	0.999908	3.55E-05	147.49	S	KGKIKSKAIISSSDDSSDEDKLKIADEGHPR	593730	206410	3537
Q6PD62	1021	RNA polymerase-associated protein CTR9 homolog	CTR9	0.99976	3.55E-05	147.49	S	GKIKSKAIISSSDDSSDEDKLKIADEGHPRN	593730	206410	3538
Q8N108	377	Mesoderm induction early response protein 1	MIER1	0.999969	5.96E-06	147.49	S	RLLDESESAASSRAPSPPPTASNSSNSQSEK	826710	561130	4198
Q9UQ35	1270	Serine/arginine repetitive matrix protein 2	SRRM2	0.999958	9.23E-06	147.49	S	HLSSELKEMSTSNFESSPEVEERPAVSLTLD	1540700	0	6622
Q14676	964	Mediator of DNA damage checkpoint protein 1	MDC1	0.845062	3.08E-20	147.42	S	GEPEGGSQDQKGQASSPTPEPGVGAGDLPGP	0	8851900	2769
Q86UE4	311	Protein LYRIC	MTDH	0.863801	1.21E-05	147.4	S	QISAGEEKWNSVSPASAGKRKTEPSAWSQDT	2210500	0	3928
Q86UW7	56	Calcium-dependent secretion activator 2	CADPS2	0.999996	2.92E-06	147.39	S	RRDAPGRAGGGGAARSVSPSPSVLSEGRDEP	3016300	1010400	3941
Q86UW7	58	Calcium-dependent secretion activator 2	CADPS2	0.999661	2.92E-06	147.39	S	DAPGRAGGGGAARSVSPSPSVLSEGRDEPQR	4607200	1799100	3942
Q96N64	81	PWWP domain-containing protein 2A	PWWP2A	1	5.12E-06	147.39	S	PPLPPPPPPPGELARSPEAVGPELEAEEKLS	318180	510020	5025
Q9Y2D5	152	A-kinase anchor protein 2	AKAP2	0.934643	7.60E-07	147.39	S	SQPRDALGDSLQVPVSPSSTTSSRCSSRDGE	1766500	2222400	6775
O00512	687	B-cell CLL/lymphoma 9 protein	BCL9	0.99455	1.22E-05	147.33	S	GNNPIFPRIPVEGPLSPSRGDFPKGIPPQMG	3710300	2412400	126
Q5TCZ1	421	SH3 and PX domain-containing protein 2A	SH3PXD2A	0.991966	6.79E-06	147.3	S	SPAVARIAPQRAQISSPNLRTRPPPRRESSL	2720200	1342600	3299
Q9BYW2	624	Histone-lysine N-methyltransferase SETD2	SETD2	0.999939	4.92E-05	147.3	S	REKAGSPAPSNRLNDSPTLKKLDELPIFKSE	1183200	1018800	5474
Q99856	88	AT-rich interactive domain-containing protein 3A	ARID3A	1	2.56E-14	147.26	S	GHPASPGGSEDGPPGSEEEDAAREGTPGSPG	14488000	8512100	5279
Q8WUZ0	126	B-cell CLL/lymphoma 7 protein family member C	BCL7C	0.999137	6.15E-20	147.17	S	TEPSPGGTPQPSRPVSPAGPPEGVPEEAQPP	11501000	6691800	4525
P28715	562	DNA repair protein complementing XP-G cells	ERCC5	0.999887	2.98E-06	147.1	S	NELCPYESKFDSSLLSSDDETKCKPNSASEV	917020	701470	1310
P11388	4	DNA topoisomerase 2-alpha	TOP2A	1	4.20E-06	147.09	S	____________MEVSPLQPVNENMQVNKIK	1032000	1178000	1020
Q9NYF8	205	Bcl-2-associated transcription factor 1	BCLAF1	0.817438	6.46E-07	147.03	S	TFEHDPSESIDEFNKSSATSGDIWPGLSAYD	0	463400	6111
Q9Y6X4	618	Soluble lamin-associated protein of 75 kDa	FAM169A	0.499996	1.12E-09	146.97	S	QNAGQKNQSEEQSEASSEQLDQFTQSAEKAV	1714500	0	7030
Q9Y6X4	619	Soluble lamin-associated protein of 75 kDa	FAM169A	0.877143	1.12E-09	146.97	S	NAGQKNQSEEQSEASSEQLDQFTQSAEKAVD	1714500	0	7031
Q66K74	767	Microtubule-associated protein 1S;MAP1S heavy chain;MAP1S li	MAP1S	0.398305	7.84E-07	146.88	S	PMAPAPASPGSSNDSSARSQERAGGLGAEET	0	0	3406
Q9H1B7	547	Interferon regulatory factor 2-binding protein-like	IRF2BPL	1	5.69E-06	146.86	S	PSGRGAAASLRKRKASPEPPDSAEGALKLGE	697370	0	5594
O15344	96	E3 ubiquitin-protein ligase Midline-1	MID1	0.882787	3.64E-06	146.84	S	NIIDRFQKASVSGPNSPSETRRERAFDANTM	2887500	0	311
O15357	132	Phosphatidylinositol 3,4,5-trisphosphate 5-phosphatase 2	INPPL1	1	3.33E-05	146.84	S	VEGEREPDPPDDRDASDGEDEKPPLPPRSGS	2217300	2036200	313
P49792	18	E3 SUMO-protein ligase RanBP2	RANBP2	0.679585	3.02E-06	146.84	S	RSKADVERYIASVQGSTPSPRQKSMKGFYFA	2431900	0	1719
Q9H6R7	690	WD repeat and coiled-coil-containing protein C2orf44	C2orf44	0.998764	5.29E-06	146.84	S	GSLSRSDVFRDSFSHSPGAVSSLKVFTGLAA	0	718420	5737
Q9UQ35	1266	Serine/arginine repetitive matrix protein 2	SRRM2	0.643028	9.64E-06	146.79	S	QILSHLSSELKEMSTSNFESSPEVEERPAVS	1289400	916410	6621
Q9Y2V2	52	Calcium-regulated heat stable protein 1	CARHSP1	0.999984	0.000858862	146.79	S	NVVPSPLPTRRTRTFSATVRASQGPVYKGVC	18968000	11720000	6813
Q9BX95	114	Sphingosine-1-phosphate phosphatase 1	SGPP1	0.833875	3.05E-06	146.79	T	PASPRRAGALRRNSLTGEEGQLARVSNWPLY	0	0	8061
Q02952	644	A-kinase anchor protein 12	AKAP12	0.991797	2.18E-06	146.78	S	SDKEDELDKVKSATLSSTESTASEMQEEMKG	1244400	960500	2120
Q02952	645	A-kinase anchor protein 12	AKAP12	0.85383	2.18E-06	146.78	S	DKEDELDKVKSATLSSTESTASEMQEEMKGS	1244400	960500	2121
Q14C86	902	GTPase-activating protein and VPS9 domain-containing protei	GAPVD1	0.796688	7.04E-06	146.71	S	FKQRHSYPERLVRSRSSDIVSSVRRPMSDPS	8490500	3046600	2847
Q9UQ35	1975	Serine/arginine repetitive matrix protein 2	SRRM2	0.999874	0.000447426	146.71	S	SRTPPVTRRRSRSRTSPITRRRSRSRTSPVT	7207500	6211000	6728
Q9UQ35	1974	Serine/arginine repetitive matrix protein 2	SRRM2	0.722052	0.000447426	146.71	T	RSRTPPVTRRRSRSRTSPITRRRSRSRTSPV	7207500	6211000	8274
O95292	159	Vesicle-associated membrane protein-associated protein B/C	VAPB	0.947208	4.77E-06	146.67	S	TASKTETPIVSKSLSSSLDDTEVKKVMEECK	0	220340	744
Q14684	706	Ribosomal RNA processing protein 1 homolog B	RRP1B	0.999466	1.27E-05	146.67	S	NMTAEFKKTDKSILVSPTGPSRVAFDPEQKP	1227900	633060	2783
Q69YN4	222	Protein virilizer homolog	KIAA1429	1	1.27E-05	146.67	S	KEEDAPHREDYFEPISPDRNSVPQEGQYSDE	1775200	1738100	3435
Q9Y5S2	1690	Serine/threonine-protein kinase MRCK beta	CDC42BPB	0.999998	2.61E-07	146.67	S	KHSTPSNSSNPSGPPSPNSPHRSQLPLEGLE	7641700	5838400	6964
Q9Y5S2	1693	Serine/threonine-protein kinase MRCK beta	CDC42BPB	1	2.61E-07	146.67	S	TPSNSSNPSGPPSPNSPHRSQLPLEGLEQPA	4570100	3505000	6965
Q04726	240	Transducin-like enhancer protein 3	TLE3	0.944073	9.98E-10	146.53	S	KKRKAEEKDSLSRYDSDGDKSDDLVVDVSNE	0	4415400	2176
Q04726	245	Transducin-like enhancer protein 3	TLE3	0.91686	9.98E-10	146.53	S	EEKDSLSRYDSDGDKSDDLVVDVSNEDPATP	5438600	4415400	2177
O14639	452	Actin-binding LIM protein 1	ABLIM1	0.419314	5.40E-06	146.5	S	EGYQDVRDRMIHRSTSQGSINSPVYSRHSYT	0	0	162
Q6IAA8	27	Ragulator complex protein LAMTOR1	LAMTOR1	0.937095	1.28E-05	146.5	S	SDQDREERKLLLDPSSPPTKALNGAEPNYHS	2910500	1736800	3467
P51610	3	Host cell factor 1;HCF N-terminal chain 1;HCF N-terminal cha	HCFC1	0.965209	4.40E-06	146.3	S	_____________MASAVSPANLPAVLLQPR	0	2357700	1787
Q3KQU3	70	MAP7 domain-containing protein 1	MAP7D1	0.981476	9.93E-06	146.3	S	MKNATSSKQLPLEPESPSGQVGPRPAPPQEE	2192000	3305100	3068
Q9UHJ3	765	Scm-like with four MBT domains protein 1	SFMBT1	0.463261	3.03E-05	146.28	S	LPPDRQRRKRELRTFSFSDDENKPPSPKEIR	0	0	6359
Q9UHJ3	767	Scm-like with four MBT domains protein 1	SFMBT1	0.892106	3.03E-05	146.28	S	PDRQRRKRELRTFSFSDDENKPPSPKEIRIE	1258800	0	6360
Q9UHJ3	775	Scm-like with four MBT domains protein 1	SFMBT1	1	3.03E-05	146.28	S	ELRTFSFSDDENKPPSPKEIRIEVAERLHLD	1258800	551450	6361
P09651	2	Heterogeneous nuclear ribonucleoprotein A1;Heterogeneous nuc	HNRNPA1	0.985879	2.98E-06	146.27	S	______________MSKSESPKEPEQLRKLF	2880400	2975400	972
Q5JSH3	561	WD repeat-containing protein 44	WDR44	0.999914	5.80E-06	146.27	S	YFNNMRMKYNTEGRVSPSPSQESLSSSKSDT	3816700	1471000	3186
Q9UMN6	861	Histone-lysine N-methyltransferase 2B	KMT2B	0.999892	3.67E-06	146.27	S	DESVEAKRERPSGPESPVQGPRIKHVCRHAA	1200000	531590	6516
Q01082	2165	Spectrin beta chain, non-erythrocytic 1	SPTBN1	0.934539	0.000113603	146.18	S	MVNGATEQRTSSKESSPIPSPTSDRKAKTAL	1121800	2267300	2043
Q01082	2169	Spectrin beta chain, non-erythrocytic 1	SPTBN1	0.997506	0.000113603	146.18	S	ATEQRTSSKESSPIPSPTSDRKAKTALPAQS	1121800	2267300	2044
Q01082	2161	Spectrin beta chain, non-erythrocytic 1	SPTBN1	0.446067	0.000113603	146.18	S	DTSEMVNGATEQRTSSKESSPIPSPTSDRKA	0	0	2050
Q13426	328	DNA repair protein XRCC4	XRCC4	1	1.47E-05	146.16	S	HISAENMSLETLRNSSPEDLFDEI_______	946290	1393700	2493
Q13426	232	DNA repair protein XRCC4	XRCC4	0.939751	8.91E-14	146.15	S	ICSEMTADRDPVYDESTDEESENQTDLSGLA	0	6404200	2491
Q15648	1215	Mediator of RNA polymerase II transcription subunit 1	MED1	0.937427	0.00010697	146.15	T	GGSDKLASPMKPVPGTPPSSKAKSPISSGSG	10205000	0	7644
O96017	379	Serine/threonine-protein kinase Chk2	CHEK2	0.796929	8.56E-06	146.11	S	IKITDFGHSKILGETSLMRTLCGTPTYLAPE	999560	0	848
Q0JRZ9	488	F-BAR domain only protein 2	FCHO2	0.999995	1.87E-13	146.04	S	TSGKLSGINEIPRPFSPPVTSNTSPPPAAPL	5371400	4282900	2284
Q0JRZ9	495	F-BAR domain only protein 2	FCHO2	0.757686	1.87E-13	146.04	T	INEIPRPFSPPVTSNTSPPPAAPLARAESSS	0	4282900	7507
Q14202	214	Zinc finger MYM-type protein 3	ZMYM3	0.753777	1.29E-06	145.94	S	LGDGINSSQTKPGGSSPPAHPSLPGDGLTAK	1801500	1188800	2720
Q96T58	740	Msx2-interacting protein	SPEN	0.998541	4.37E-05	145.94	S	SQSPVHLRRPQSPGASPSQAERLPSDSERRL	3041200	1913600	5178
O43399	21	Tumor protein D54	TPD52L2	0.843744	4.13E-06	145.91	S	QDINLNSPNKGLLSDSMTDVPVDTGVAARTP	1767400	0	378
Q86WB0	321	Nuclear-interacting partner of ALK	ZC3HC1	1	9.94E-06	145.91	S	PGLEGRPERLPLVPESPRRMMTRSQDATFSP	5667200	3271100	3979
Q9H4L7	213	SWI/SNF-related matrix-associated actin-dependent regulator	SMARCAD1	0.567506	4.58E-07	145.88	S	FGDAGGGPRKRKLSSSSEPYEEDEFNDDQSI	0	0	5696
Q9H4L7	217	SWI/SNF-related matrix-associated actin-dependent regulator	SMARCAD1	0.972046	4.58E-07	145.88	Y	GGGPRKRKLSSSSEPYEEDEFNDDQSIKKTR	2664300	0	8422
O43149	1518	Zinc finger ZZ-type and EF-hand domain-containing protein 1	ZZEF1	0.999535	1.19E-13	145.83	S	LPAADVSPATAEEPLSPSTPTRRPPFTRGRL	4185500	2546400	336
O43149	1520	Zinc finger ZZ-type and EF-hand domain-containing protein 1	ZZEF1	0.543592	1.19E-13	145.83	S	AADVSPATAEEPLSPSTPTRRPPFTRGRLRL	0	3470400	337
Q8IYB3	414	Serine/arginine repetitive matrix protein 1	SRRM1	0.998936	1.81E-05	145.83	S	HRPSPPATPPPKTRHSPTPQQSNRTRKSRVS	5062700	1610300	4138
Q96EB6	172	NAD-dependent protein deacetylase sirtuin-1;SirtT1 75 kDa fr	SIRT1	0.996037	6.81E-06	145.83	S	FHSCESDEEDRASHASSSDWTPRPRIGPYTF	956280	725740	4883
Q96EB6	173	NAD-dependent protein deacetylase sirtuin-1;SirtT1 75 kDa fr	SIRT1	0.976596	6.81E-06	145.83	S	HSCESDEEDRASHASSSDWTPRPRIGPYTFV	956280	725740	4884
O43149	1521	Zinc finger ZZ-type and EF-hand domain-containing protein 1	ZZEF1	0.504412	1.19E-13	145.83	T	ADVSPATAEEPLSPSTPTRRPPFTRGRLRLL	0	2546400	7163
O75376	2436	Nuclear receptor corepressor 1	NCOR1	0.821056	1.05E-05	145.81	S	EREPAPLLSAQYETLSDSDD___________	0	1121400	593
Q9UQ88;P2	740;752	Cyclin-dependent kinase 11A;Cyclin-dependent kinase 11B	CDK11A;CDK1	1	3.26E-07	145.77	S	TWPAKSEQQRVKRGTSPRPPEGGLGYSQLGD	12603000	10029000	1194
Q9UQ88;P2	739;751	Cyclin-dependent kinase 11A;Cyclin-dependent kinase 11B	CDK11A;CDK1	1	3.26E-07	145.77	T	PTWPAKSEQQRVKRGTSPRPPEGGLGYSQLG	12603000	3530900	7312
P43307	246	Translocon-associated protein subunit alpha	SSR1	0.484914	5.50E-10	145.73	S	SRKRKRPIQKVEMGTSSQNDVDMSWIPQETL	0	0	1523
P43307	245	Translocon-associated protein subunit alpha	SSR1	0.736953	5.50E-10	145.73	T	ESRKRKRPIQKVEMGTSSQNDVDMSWIPQET	0	6421400	7380
O94762	815	ATP-dependent DNA helicase Q5	RECQL5	1	2.86E-09	145.71	S	APEKYTGEEDGAGGHSPAPPQTEECLRERPS	6703700	4999500	682
Q5T200	316	Zinc finger CCCH domain-containing protein 13	ZC3H13	0.977142	8.28E-05	145.71	S	GRDFERQREKRDKPRSTSPAGQHHSPISSRH	4272400	1646200	3271
Q5T200	328	Zinc finger CCCH domain-containing protein 13	ZC3H13	0.995435	8.28E-05	145.71	S	KPRSTSPAGQHHSPISSRHHSSSSQSGSSIQ	4272400	1646200	3274
Q8IWQ3	512	Serine/threonine-protein kinase BRSK2	BRSK2	0.999564	2.89E-07	145.71	S	LQVPTPEEMSNLTPESSPELAKKSWFGNFIS	596440	3006300	4062
Q5T200	317	Zinc finger CCCH domain-containing protein 13	ZC3H13	0.986733	8.28E-05	145.71	T	RDFERQREKRDKPRSTSPAGQHHSPISSRHH	1826600	900840	7721
P02545	22	Prelamin-A/C;Lamin-A/C	LMNA	0.926475	1.40E-05	145.7	S	RRATRSGAQASSTPLSPTRITRLQEKEDLQE	1362400	1286500	865
O15164	1028	Transcription intermediary factor 1-alpha	TRIM24	0.999981	2.99E-06	145.61	S	PEFRNESEDNKFSDDSDDDFVQPRKKRLKSI	1746100	463680	292
O75152	132	Zinc finger CCCH domain-containing protein 11A	ZC3H11A	0.999304	9.77E-06	145.61	S	QLSVQQNKLSVQSNPSPQLRSVMKVESSENV	2748000	2351300	571
P29966	81	Myristoylated alanine-rich C-kinase substrate	MARCKS	0.857041	6.07E-18	145.6	S	AADKEEPAAAGSGAASPSAAEKGEPAAAAAP	2814600	1049700	1334
Q08170	458	Serine/arginine-rich splicing factor 4	SRSF4	0.999985	9.92E-05	145.52	S	RSRSNSKSKPNLPSESRSRSKSASKTRSRSK	11865000	5805800	2245
P49792	19	E3 SUMO-protein ligase RanBP2	RANBP2	0.935776	8.48E-05	145.52	T	SKADVERYIASVQGSTPSPRQKSMKGFYFAK	0	1434000	7425
P41252	1047	Isoleucine--tRNA ligase, cytoplasmic	IARS	0.915569	5.03E-05	145.49	S	FIFTTIKAPLKPYPVSPSDKVLIQEKTQLKG	1893400	1962300	1488
P18583	2011	Protein SON	SON	1	5.90E-05	145.46	S	SRRRRSRSVVRRRSFSISPVRLRRSRTPLRR	9507000	4449700	1139
P18583	2013	Protein SON	SON	1	5.90E-05	145.46	S	RRRSRSVVRRRSFSISPVRLRRSRTPLRRRF	9507000	4449700	1140
O75151	458	Lysine-specific demethylase PHF2	PHF2	0.962192	1.33E-06	145.33	S	ENASKAVRPEVNTVASSDEVCDGDREKEEPP	1500200	920250	568
Q13625	480	Apoptosis-stimulating of p53 protein 2	TP53BP2	0.999836	1.49E-05	145.23	S	QSNAPPSFGTLRKNQSSEDILRDAQVANKNV	978640	1423800	2618
Q9BUL5	62	PHD finger protein 23	PHF23	0.70581	2.44E-05	145.23	S	YIPPSKEESDWPASGSSSPLRGESAADSDGW	0	0	5381
Q9BUL5	63	PHD finger protein 23	PHF23	0.477167	2.44E-05	145.23	S	IPPSKEESDWPASGSSSPLRGESAADSDGWD	0	0	5382
O14654	770	Insulin receptor substrate 4	IRS4	0.461704	2.24E-09	145.19	S	APSPPKAPDTNKEDDSKDNDSESDYMFMAPG	0	0	170
O00443	259	Phosphatidylinositol 4-phosphate 3-kinase C2 domain-containi	PIK3C2A	1	1.55E-05	145.14	S	TDLEITDSKVSNLQVSPKSEDISKFDWLDLD	0	359890	120
P52594	181	Arf-GAP domain and FG repeat-containing protein 1	AGFG1	0.999996	2.82E-05	145.14	S	DSAPTLHLNKGTPSQSPVVGRSQGQQQEKKQ	10738000	8506700	1815
Q9H7P9	1261	Pleckstrin homology domain-containing family G member 2	PLEKHG2	0.999996	0.000210623	145.04	S	SHVARLESSDLTPPHSPPPSSRQLLGPNAAA	1137900	691530	5773
Q9Y4R8	836	Telomere length regulation protein TEL2 homolog	TELO2	1	0.00106218	145.04	S	LLLLQRLKNRLLPPASP______________	1388200	902670	6935
Q9H7P9	1257	Pleckstrin homology domain-containing family G member 2	PLEKHG2	0.99986	0.000210623	145.04	T	GSLASHVARLESSDLTPPHSPPPSSRQLLGP	1137900	691530	8116
Q13523	573	Serine/threonine-protein kinase PRP4 homolog	PRPF4B	0.793426	6.55E-07	144.95	S	DSNMSVPSEPSSPQSSTRTRSPSPDDILERV	3272300	614830	2569
Q9NYV4	685	Cyclin-dependent kinase 12	CDK12	1	6.72E-10	144.95	S	PLPPELPGGDLSPPDSPEPKAITPPQQPYKK	17458000	11036000	6124
Q9Y4W2	617	Ribosomal biogenesis protein LAS1L	LAS1L	0.887249	7.75E-06	144.95	S	EEDRMEVGPFSTGQESPTAENARLLAQKRGA	1080800	823930	6941
Q13523	574	Serine/threonine-protein kinase PRP4 homolog	PRPF4B	0.440273	6.55E-07	144.95	T	SNMSVPSEPSSPQSSTRTRSPSPDDILERVA	0	0	7550
P24588	178	A-kinase anchor protein 5	AKAP5	0.806374	3.16E-05	144.88	S	IQTQTPLNDQATKAKSTQDLSEGISRKDGDE	2611700	0	1236
Q16204	351	Coiled-coil domain-containing protein 6	CCDC6	0.478562	3.13E-05	144.88	S	YFNEMSAQGLRPRTVSSPIPYTPSPSSSRPI	0	0	3005
Q16204	352	Coiled-coil domain-containing protein 6	CCDC6	0.493161	3.13E-05	144.88	S	FNEMSAQGLRPRTVSSPIPYTPSPSSSRPIS	0	0	3006
Q7Z2W4	271	Zinc finger CCCH-type antiviral protein 1	ZC3HAV1	1	1.83E-06	144.88	S	GSQEFLASASASAERSCTPSPDQISHRASLE	0	0	3753

P24588	179	A-kinase anchor protein 5	AKAP5	0.64451	3.16E-05	144.88	T	QTQTPLNDQATKAKSTQDLSEGISRKDGDEV	2611700	2007400	7318
Q9UKV3	414	Apoptotic chromatin condensation inducer in the nucleus	ACIN1	0.99993	8.50E-09	144.82	T	ASLVALPEQTASEEETPPPLLTKEASSPPPH	1968600	896300	8233
P46821	1265	Microtubule-associated protein 1B;MAP1B heavy chain;MAP1 li	MAP1B	0.999957	2.69E-05	144.8	S	KVSPSKSPSLSPSPPSPLEKTPLGERSVNFS	5492800	2538700	1601
Q8NEY8	133	Periphilin-1	PPHLN1	0.999998	7.03E-05	144.8	S	RERSPYKRDNTFFRESPVGRKDSPHSRSGSS	1990700	1317200	4379
Q8WW12	119	PEST proteolytic signal-containing nuclear protein	PCNP	1	3.92E-09	144.74	S	PKTLSVAAAFNEDEDSEPEEMPPEAKMRMKN	5737800	5378800	4549
Q7Z2W4	378	Zinc finger CCCH-type antiviral protein 1	ZC3HAV1	0.99949	3.40E-05	144.7	S	TSWTNDQGARRKTVFSPTLPAARSSLGSLQT	958550	593680	3755
O60343	588	TBC1 domain family member 4	TBC1D4	1	1.62E-05	144.57	S	NIFSRGANRMRGRLGSVDSFERSNSLASEKD	13261000	8415700	489
Q9BZF1	14	Oxysterol-binding protein-related protein 8	OSBPL8	0.941385	2.57E-06	144.57	S	__MEGGLADGEPDRTSLLGDSKDVLGPSTVV	689780	350260	5491
P18754	11	Regulator of chromosome condensation	RCC1	1	1.97E-05	144.55	S	_____MSPKRIAKRRSPPADAIPKSKKVKVS	1543200	1248400	1146
Q13439	71	Golgin subfamily A member 4	GOLGA4	1	1.97E-05	144.55	S	GDTQSFAQKLQLRVPSVESLFRSPIKESLFR	1559300	1152500	2533
Q5FWF5	412	N-acetyltransferase ESCO1	ESCO1	0.999996	9.13E-05	144.55	S	SKFNSVQHNKLDSQVSPKLGLLRTSFSPPAL	0	0	3160
Q147X3	55	N-alpha-acetyltransferase 30	NAA30	1	2.02E-05	144.51	S	EDEEDDEEHEGGGSRSPAGGESATVAAKGHP	2063900	3289300	2811
Q9NS37	16	CREB/ATF bZIP transcription factor	CREBZF	0.836826	1.77E-05	144.51	S	MRHSLTKLLAASGSNSPTRSESPEPAATCSL	828020	449880	5993
Q5JSH3	403	WD repeat-containing protein 44	WDR44	0.999986	5.43E-13	144.5	S	IMRRTKEYVSNDAAQSDDEEKLQSQPTDTDG	11361000	6275400	3182
Q5BKZ1	478	DBIRD complex subunit ZNF326	ZNF326	1	2.64E-09	144.43	S	ERFVKGENPFEIQDHSQDQQIEGDEEDEEKI	855340	3106300	3156
Q6P1N0	238	Coiled-coil and C2 domain-containing protein 1A	CC2D1A	0.5	9.79E-06	144.41	S	EPRVTLEGPSATAPASSPGLAKPQMPPGPCS	469230	322150	3512
Q6P1N0	239	Coiled-coil and C2 domain-containing protein 1A	CC2D1A	0.5	9.79E-06	144.41	S	PRVTLEGPSATAPASSPGLAKPQMPPGPCSP	469230	322150	3513
Q7Z2Z1	1750	Treslin	TICRR	1	4.17E-07	144.4	S	LEDFELEGVCQLPDQSPPRNSMPKAEEASSW	1106000	833860	3769
Q96PU5	446	E3 ubiquitin-protein ligase NEDD4-like	NEDD4L	0.493298	3.55E-05	144.4	S	NSNNHLIEPQIRRPRSLSSPTVTLSAPLEGA	0	0	5060
Q01831	94	DNA repair protein complementing XP-C cells	XPC	1	0.000153562	144.3	S	TVKSENLKVIKDEALSDGDDLRDFPSDLKKA	1696900	1284300	2084
Q13769	314	THO complex subunit 5 homolog	THOC5	0.999129	3.57E-09	144.3	S	ALFKPPEDSQDDESDSDAEEEQTTKRRRPTL	4036500	751030	2625
P62263	139	40S ribosomal protein S14	RPS14	0.366247	2.23E-05	144.29	S	MKIGRIEDVTPIPSDSTRRKGGRRGRRL___	0	0	1921
Q7Z478	200	ATP-dependent RNA helicase DHX29	DHX29	0.999987	1.84E-05	144.29	S	KSRPKFQSPQIQATISPPLQPKTKTYEEDPK	1085200	722860	3814
Q01130	191	Serine/arginine-rich splicing factor 2	SRSF2	1	8.31E-05	144.28	S	VSRSRSRSRSRSRSRSRSPPPVSKRESKSRSRS	12743000	14668000	2056
Q01130	189	Serine/arginine-rich splicing factor 2	SRSF2	1	8.31E-05	144.28	S	SSVSRSRSRSRSRSRSRSPPPVSKRESKSRS	6298600	6838400	2057
Q96KQ7	140	Histone-lysine N-methyltransferase EHMT2	EHMT2	0.999971	3.95E-05	144.28	S	SCPSRAKMSMTGAGKSPPSVQSLAMRLLSMP	2804500	1887700	5007
Q15910	367	Histone-lysine N-methyltransferase EZH2	EZH2	0.898389	8.21E-05	144.17	T	GRRRGRLPNNSSRPSTPTINVLESKDTDSDR	1300900	3389000	7653
Q8WWI1	1510	LIM domain only protein 7	LMO7	1	8.33E-05	144.15	S	NKEPVSLPGIMRRGESLDNLDSPRSNSWRQP	0	0	4554
Q96N66	285	Lysophospholipid acyltransferase 7	MBOAT7	0.993402	3.97E-05	144.08	S	ARAGGGPTLQCPPPSSPEKAASLEYDYETIR	764020	960990	5028
Q9NYZ3	575	G2 and S phase-expressed protein 1	GTSE1	1	0.000143196	144.08	S	TRESNRKTDSRLVDVSPDRGSPPSRVPQALN	8756100	6843700	6142
Q9NYZ3	580	G2 and S phase-expressed protein 1	GTSE1	0.976415	0.000143196	144.08	S	RKTDSRLVDVSPDRGSPPSRVPQALNFSPEE	6889400	4859100	6143
Q7Z6Z7	3818	E3 ubiquitin-protein ligase HUWE1	HUWE1	0.962794	3.04E-17	144.04	S	VRREESPMDVDQPSPSAQDTQSIASDGTPQG	406290	563730	3875
Q96MH2	76	Protein HEXIM2	HEXIM2	0.999366	4.37E-05	143.99	S	AVGGLGWNSRSPRTQSPGGCSAEAVLARKKH	1197600	946080	5022
Q9UHI6	703	Probable ATP-dependent RNA helicase DDX20	DDX20	0.989992	4.16E-05	143.94	S	TPVDDRISLEQPPNGSDTPNPEKYQESPGIQ	1962300	1230100	6355
Q9BTE7	9	DCN1-like protein 5	DCUN1D5	1	4.47E-05	143.93	S	_______MPVKKKRKSPGVAAAVAEDGGLKK	1417600	1225900	5364
Q9UQ35	359	Serine/arginine repetitive matrix protein 2	SRRM2	0.496518	0.000173066	143.88	T	EKSATRPSPSPERSSTGPEPPAPTPLLAERH	0	0	8264
Q9H8K7	424	Uncharacterized protein C10orf88	C10orf88	0.999284	1.26E-06	143.83	S	DDKIALLLDLLQNPNSPPTGIPLRHYDSGER	970420	512960	5788
O14639	706	Actin-binding LIM protein 1	ABLIM1	1	4.64E-05	143.76	S	LPDGHMPAMRMDRGVSMPNMLEPKIFPYEML	1537200	1240100	160
Q16637	25	Survival motor neuron protein	SMN1	1	1.37E-05	143.74	T	GGVPEQEDSVLFRRGTGQSDDSDIWDDTALI	6497300	7978400	7661
O15234	373	Protein CASC3	CASC3	1	4.06E-05	143.72	S	SVRDPSPEADAPVLGSPEKEEAASEPPAAAP	0	8867100	305
O15042	67	U2 snRNP-associated SURP motif-containing protein	U2SURP	0.990569	2.83E-05	143.64	S	KHNYRNESARESLCDSPHQNLSRPLLENKLK	424910	571900	262
Q86W56	291	Poly(ADP-ribose) glycohydrolase	PARG	0.999333	2.83E-05	143.64	S	KNDNKLTRQESCLGNSPPFEKESEPESPMDV	0	0	3974
Q8NDX5	616	Polyhomeotic-like protein 3	PHC3	0.999742	2.35E-05	143.63	S	DECVRMDRTPPPPTLSPAAITVGRGEDLTSE	2246400	1414100	4362
Q8NDX5	609	Polyhomeotic-like protein 3	PHC3	1	2.35E-05	143.63	T	EEMPEESDECVRMDRTPPPPTLSPAAITVGR	2246400	1414100	7892
Q15773	238	Myeloid leukemia factor 2	MLF2	0.997844	2.44E-05	143.58	S	RAEGPPRLAIQGPEDSPSRQSRRYDW_____	28184000	20962000	2984
Q9UMZ2	812	Synergin gamma	SYNRG	1	4.45E-05	143.58	S	AVAFRHTKEDSASVKSLDLPSIGGSSVGKED	2028700	1035900	6524
P15374	130	Ubiquitin carboxyl-terminal hydrolase isozyme L3	UCHL3	0.996226	5.65E-06	143.56	S	SGSTLKKFLEESVSMSPEERARYLENYDAIR	377340	404730	1073
O95835	464	Serine/threonine-protein kinase LATS1	LATS1	0.998303	4.91E-05	143.55	S	HEIPTWQPNIPVRSNSFNNPLGNRASHSANS	5253600	2030000	837
Q7Z2Z1	1334	Treslin	TICRR	1	2.46E-05	143.55	S	VSKKSPFRKSKIECPSPGELDQKEPQMSPSV	1186900	754790	3765
Q96EB6	26	NAD-dependent protein deacetylase sirtuin-1;SirtT1 75 kDa f	SIRT1	0.943645	2.46E-05	143.55	S	PGGSPSAAGADREAASSPAGEPLRKRPRRDG	655650	1038300	4885
Q9UKV3	710	Apoptotic chromatin condensation inducer in the nucleus	ACIN1	0.999994	0.000191199	143.55	S	ERTSTSSSSVQARRLSQPESAEKHVTQRLQP	2378000	2319200	6457
P46013	308	Antigen KI-67	MKI67	1	5.70E-07	143.48	S	RPKSGGSGHAVAEPASPEQELDQNKGKGRDV	4530600	3526400	1534
Q969V6	511	MKL/myocardin-like protein 1	MKL1	1	5.07E-05	143.42	S	ILVKEEGPRAGSCCLSPGGRAELEGRDKDQM	1636900	2353000	4750
Q96N67	182	Dedicator of cytokinesis protein 7	DOCK7	0.877551	2.79E-05	143.4	S	NSYQDDQDDLKRRSMSIDDTPRGSWACSIFD	0	0	5037
O15063	945	Uncharacterized protein KIAA0355	KIAA0355	0.459349	4.81E-13	143.36	S	SGPDLVAAVKQRRKHSSGEQDTSTLPSPPLL	0	0	273
O15063	946	Uncharacterized protein KIAA0355	KIAA0355	0.459349	4.81E-13	143.36	S	GPDLVAAVKQRRKHSSGEQDTSTLPSPPLLT	0	0	274
Q8IW35	770	Centrosomal protein of 97 kDa	CEP97	0.995104	4.81E-13	143.36	S	HGEWNKESSNNEQDNSLLEQYLTSVQQLEDA	0	2644200	4052
Q96B36	88	Proline-rich AKT1 substrate 1	AKT1S1	0.997973	7.33E-13	143.36	S	TAARPPAPPPAPQPPSPTPSPPRPTLAREDN	23690000	5460400	4796
Q96B36	92	Proline-rich AKT1 substrate 1	AKT1S1	0.99813	7.33E-13	143.36	S	PPAPPPAPQPPSPTPSPPRPTLAREDNEEDE	23690000	5460400	4797
Q13615	647	Myotubularin-related protein 3	MTMR3	0.998687	1.20E-06	143.33	S	CSDPSLNEKWQEHRRSLELSSLAGPGEDPLS	987530	570630	2605
Q14669	312	E3 ubiquitin-protein ligase TRIP12	TRIP12	0.999439	2.63E-05	143.31	S	AKLASLRKSTKKRSESPPAELPSLRRSTRQK	5674400	2618500	2754
Q9BRS8	56	La-related protein 6	LARP6	0.999994	5.54E-05	143.28	S	RGAGDPARYLSPGWGSASEEEPSRGHSGTTA	2312000	1601300	5331
Q16555	509	Dihydropyrimidinase-related protein 2	DPYSL2	0.998315	0.000223298	143.28	T	VPRGLYDGPVCEVSVTPKTVTPASSAKTSPA	974770	710970	7659
Q04637	318	Eukaryotic translation initiation factor 4 gamma 1	EIF4G1	0.85252	2.40E-06	143.17	T	ISRETGEPYRLSPEPTPLAEPILEVEVTLSK	0	0	7490
Q9UQ35	1320	Serine/arginine repetitive matrix protein 2	SRRM2	1	0.000123509	143.11	S	WGGPHFSPEHKELSNSPLRENSFGSPLEFRN	13078000	17521000	6617
Q9Y6A5	177	Transforming acidic coiled-coil-containing protein 3	TACC3	0.832194	5.10E-06	143.09	S	GSSENQMVSPGKVSGSPEQAVEENLSSYSLD	1174000	1000400	6991
Q9HA47	253	Uridine-cytidine kinase 1	UCK1	0.999439	5.88E-05	143.05	S	WHRGGSNGRSYKRTFSEPGDHPGMLTSGKRS	0	581930	5809
Q86TY3	769	Uncharacterized protein C14orf37	C14orf37	1	2.37E-05	143.01	S	REFNSMQDRVMLLADSSEDEF___________	0	656040	3910
Q86TY3	770	Uncharacterized protein C14orf37	C14orf37	1	2.37E-05	143.01	S	EFNSMQDRVMLLADSSEDEF____________	0	656040	3911
Q9Y248	182	DNA replication complex GINS protein PSF2	GINS2	0.978522	5.32E-05	142.97	S	MYKLRTNLQPLESTQSQDF_____________	0	3018700	6768
Q96L73	483	Histone-lysine N-methyltransferase, H3 lysine-36 and H4 lysi	NSD1	1	2.88E-05	142.95	S	HDLLLNGCLKSLAFDSEHSADEKEKPCAKSR	446870	351780	5014
Q96L73	486	Histone-lysine N-methyltransferase, H3 lysine-36 and H4 lysi	NSD1	1	2.88E-05	142.95	S	LLNGCLKSLAFDSEHSADEKEKPCAKSRARK	446870	351780	5015
P24928	1877	DNA-directed RNA polymerase II subunit RPB1	POLR2A	0.915936	0.000416108	142.95	T	PTSPKYSPTSPKYSPTSPTYSPTTPKYSPTS	0	0	7324
O00429	616	Dynamin-1-like protein	DNM1L	1	0.000157607	142.91	S	LLAEEKSKPIPIMPASPQKGHAVNLLDVPVP	1443500	1075700	119
Q9UHI6	714	Probable ATP-dependent RNA helicase DDX20	DDX20	1	5.47E-05	142.89	S	PPNGSDTPNPEKYQESPGIQMKTRLKEGASQ	6423300	3918700	6358
Q9Y3S1	1817	Serine/threonine-protein kinase WNK2	WNK2	0.999999	1.29E-05	142.88	S	LRYSAPPDVYLDEAPSSPDVKLAVRRAQTAS	1299400	549400	6869
Q9Y3S1	1818	Serine/threonine-protein kinase WNK2	WNK2	1	1.29E-05	142.88	S	RYSAPPDVYLDEAPSSPDVKLAVRRAQTASS	1299400	549400	6870
Q09666	212	Neuroblast differentiation-associated protein AHNAK	AHNAK	0.907221	2.94E-05	142.84	S	VETQSGKTVIRLPSGSGAASPTGSAVDIRAG	2066400	1261300	2279
Q00587	350	Cdc42 effector protein 1	CDC42EP1	1	5.94E-05	142.76	S	DARQERVEVLPQARASWESLDEEWRAPQAGS	716880	1606900	2025
Q12802	1929	A-kinase anchor protein 13	AKAP13	0.94799	5.94E-05	142.76	S	GNDENMSNTWKFLSHSTDSLNKISKVNESTE	2114400	1875800	2309
Q86TB9	179	Protein PAT1 homolog 1	PATL1	0.971766	3.02E-05	142.76	S	DDRDLSERALPRRSTSPIIGSPPVRAVPIGT	17477000	10401000	3896
Q9UQ35	2688	Serine/arginine repetitive matrix protein 2	SRRM2	1	0.0022253	142.76	S	RRSRSPRKPIDSLRDSRSLSYSPVERRRPSP	3085600	1321800	6647
Q02952	285	A-kinase anchor protein 12	AKAP12	0.590368	0.000147723	142.74	T	EEKQEKEPSKSAESPTSPVTSETGSTFKKFF	0	839120	7482
P46821	1256	Microtubule-associated protein 1B;MAP1B heavy chain;MAP1 li	MAP1B	0.995911	0.000215422	142.57	S	DSISAVSSEKVSPSKSPSLSPSPPSPLEKTP	140820	430760	1597
Q15154	869	Pericentriolar material 1 protein	PCM1	1	4.90E-05	142.57	S	QGLAETASPVAVSLRSDGSENLCTPQQSRTE	520450	0	2885
Q15154	872	Pericentriolar material 1 protein	PCM1	1	4.90E-05	142.57	S	AETASPVAVSLRSDGSENLCTPQQSRTEKTM	520450	0	2886
Q99638	328	Cell cycle checkpoint control protein RAD9A	RAD9A	0.999927	6.59E-05	142.57	S	TIGNEGSRVLPSISLSPGPQPPKSPGPHSEE	940960	699740	5242
Q9NPI6	525	mRNA-decapping enzyme 1A	DCP1A	0.999033	6.62E-05	142.57	S	TVTRSSDLERKASSPSPLTIGTPESQRKPSI	1595400	625310	5892
Q7Z417	692	Nuclear fragile X mental retardation-interacting protein 2	NUFIP2	1	3.84E-05	142.54	S	KQDPKRIITYNEAMDSPDQ____________	900070	1852800	3795
P23588	442	Eukaryotic translation initiation factor 4B	EIF4B	0.999998	0.000582672	142.4	S	TGTSTTSSRNARRRESEKSLENETLNKEEDC	0	0	1228
P23588	445	Eukaryotic translation initiation factor 4B	EIF4B	1	9.91E-05	142.4	S	STTSSRNARRRESEKSLENETLNKEEDCHSP	288500	489930	1229
P27448	469	MAP/microtubule affinity-regulating kinase 3	MARK3	1	6.20E-05	142.4	S	KSSGSAVGGKGIAPASPMLGNASNPNKADIP	5017100	2793000	1280
P98164	4618	Low-density lipoprotein receptor-related protein 2	LRP2	0.968708	0.000534498	142.4	S	NEQKESVAATPPPSPSLPAKPKPPSRRDPTP	0	909060	2009
P98164	4612	Low-density lipoprotein receptor-related protein 2	LRP2	0.999842	0.000534498	142.4	T	IYAQMENEQKESVAATPPPSPSLPAKPKPPS	1082800	909060	7469
Q13009	231	T-lymphoma invasion and metastasis-inducing protein 1	TIAM1	1	6.43E-05	142.39	S	TRASPRQLSTCQRANSLGDLYAQKNSGVTAN	1708400	1398300	2386
Q5TCX8	528	Mitogen-activated protein kinase kinase kinase MLK4	MLK4	1	6.43E-05	142.39	S	ISLPSDFQHKITVQASPNLDKRRSLNSSSSS	10603000	6898600	3298
Q86VQ1	345	Glucocorticoid-induced transcript 1 protein	GLCCI1	0.999952	3.29E-05	142.39	S	RRAPLPAHYRSSSTRSIDTQTPSVQERSSSC	911680	711920	3964
Q96CB9	206	5-methylcytosine rRNA methyltransferase NSUN4	NSUN4	0.999719	6.43E-05	142.39	S	LLQTGCCRNLAANDLSPSRIARLQKILHSYV	2048300	1349200	4827
Q16643	142	Drebrin	DBN1	0.9996	0.000140178	142.36	S	GAIGQRLSNGLARLSSPVLHRLRLREDENAE	26723000	13110000	3026
Q4VCS5	312	Angiomotin	AMOT	0.716527	1.27E-12	142.32	S	SLPLSARNSQPHSPTSSLTSGGSLPLLQSPP	14430000	0	3116
Q9Y4B5	1812	Microtubule cross-linking factor 1	MTCL1	1	0.000268572	142.32	S	ELGPGQETGTNSRGRSPSPIGVGSEMCREEG	627710	440440	6906
Q9Y4B5	1814	Microtubule cross-linking factor 1	MTCL1	1	0.000268572	142.32	S	GPGQETGTNSRGRSPSPIGVGSEMCREEGGE	627710	440440	6907
Q4KMP7	150	TBC1 domain family member 10B	TBC1D10B	0.979241	0.000284954	142.32	T	TEEARPSPAPGPGTPTGTPTRTPSRTAPGAL	1843000	1043600	7678
Q4VCS5	329	Angiomotin	AMOT	0.752775	1.27E-12	142.32	T	LTSGGSLPLLQSPPSTRLSPARHPLVPNQGD	14430000	0	7688
P46100	729	Transcriptional regulator ATRX	ATRX	0.999997	1.79E-05	142.23	S	NKLPKSKQSETVDQNSDSDEMLAILKEVSRM	1660800	1537300	1555
Q96N67	946	Dedicator of cytokinesis protein 7	DOCK7	0.973437	6.25E-06	142.23	S	VNTGGPKAAPWGSNPSPSAESTQAMDRSCNR	1637200	852700	5029
P25490	247	Transcriptional repressor protein YY1	YY1	0.99931	6.54E-13	142.21	S	IDHETVVEEQIIGENSPPDYSEYMTGKKLPP	2698600	3134100	1271
P78362;Q9	547;426;5	SRSF protein kinase 2;SRSF protein kinase 2 N-terminal;SRSF	SRPK2;SRPK	1	0.000148836	142.19	T	GNACWVHKHFTEDIQTRQYRSIEVLIGAGYS	0	0	7462
O00418	18	Eukaryotic elongation factor 2 kinase	EEF2K	1	6.81E-05	142.1	S	DEDLIFRLEGVDGGQSPRAGHDGDSDGDSDD	0	1195300	118
O14654	1107	Insulin receptor substrate 4	IRS4	0.991258	4.24E-05	142.1	S	FFAAARAAVSAFPTDSLERDLSPSSAPAVAS	8371000	0	166
Q9UQ35	990	Serine/arginine repetitive matrix protein 2	SRRM2	0.994077	2.91E-05	142.1	S	PDTKVKPETPPRQSHSGSISPYPKVKAQTPP	2324900	3436300	6674
Q9UQ35	994	Serine/arginine repetitive matrix protein 2	SRRM2	0.988249	2.91E-05	142.1	S	VKPETPPRQSHSGSISPYPKVKAQTPPGPSL	2324900	3423900	6676
Q15596	699	Nuclear receptor coactivator 2	NCOA2	0.850942	4.26E-05	142.08	S	KEKHKILHRLLQDSSSPVDLAKLTAEATGKD	893430	361910	2947
Q9GZR2	419	RNA exonuclease 4	REXO4	1	4.26E-05	142.08	S	MARDRRPLLTAPDHCSDDA____________	904840	545280	5554
Q92797	1259	Symplekin	SYMPK	1	1.66E-08	142.07	S	PQTLAPVGEDAMKTPSPAAEDAREPEAKGNS	10716000	7885300	4716
Q92797	1257	Symplekin	SYMPK	1	1.66E-08	142.07	T	RSPQTLAPVGEDAMKTPSPAAEDAREPEAKG	10716000	7885300	7945
P26368	2	Splicing factor U2AF 65 kDa subunit	U2AF2	1	6.32E-05	142	S	_______________MSDFDEFERQLNENKQE	6410000	9014100	1277
Q01518	310	Adenylyl cyclase-associated protein 1	CAP1	0.771845	0.000150242	141.91	S	SGPKPFSAPKPQTSPSPKRATKKEPAVLELE	0	2604100	2070
Q12888	1426	Tumor suppressor p53-binding protein 1	TP53BP1	1	1.28E-06	141.91	S	TRETAVPGPLGIEDISPNLSPDDKSFSRVVP	4920600	4390000	2339
Q12888	1430	Tumor suppressor p53-binding protein 1	TP53BP1	1	1.28E-06	141.91	S	AVPGPLGIEDISPNLSPDDKSFSRVVPRVPD	7421300	6341600	2340
Q8IWX8	822	Calcium homeostasis endoplasmic reticulum protein	CHERP	0.902224	1.89E-07	141.91	S	PGRRRRSRSRSPTPPSSAGLGSNSAPPIPDS	6123000	0	4079
Q96QD9	16	UAP56-interacting factor	FYTTD1	1	4.42E-05	141.91	S	MNRFGTRLVGATATSSPPPKARSNENLDKID	1933300	1182900	5080
Q9H175	280	Cysteine/serine-rich nuclear protein 2	CSRNP2	1	0.00538865	141.89	S	VRTHYLHTIMKLELESKRQVSRPAAPDEEPS	244150	237240	5590
Q9H175	267	Cysteine/serine-rich nuclear protein 2	CSRNP2	0.829583	0.00538865	141.89	T	GNMAGRIEFNPIRVRTHYLHTIMKLELESKR	244150	237240	8085
Q9HC35	895	Echinoderm microtubule-associated protein-like 4	EML4	0.611152	4.36E-17	141.87	S	TLTKAPVSSTESVIQSNTPTPPPSQPLNETA	0	6710500	5837
Q9HC35	897	Echinoderm microtubule-associated protein-like 4	EML4	0.846679	4.36E-17	141.87	T	TKAPVSSTESVIQSNTPTPPPSQPLNETAEE	14363000	8080100	8129
Q9HC35	899	Echinoderm microtubule-associated protein-like 4	EML4	0.998034	4.36E-17	141.87	T	APVSSTESVIQSNTPTPPPSQPLNETAEEES	15622000	8778900	8130
P35606	859	Coatomer subunit beta'	COPB2	0.994112	8.93E-09	141.86	S	PSRSTAQQELDGKPASPTPVIVASHTANKEE	19032000	8342900	1397
Q7Z6Z7	3816	E3 ubiquitin-protein ligase HUWE1	HUWE1	0.99751	6.00E-17	141.84	S	ASVRREESPMDVDQPSPSAQDTQSIASDGTP	6078000	4314000	3874
P51610	6	Host cell factor 1;HCF N-terminal chain 1;HCF N-terminal ch	HCFC1	0.999501	3.02E-05	141.82	S	__________MASAVSPANLPAVLLQPRWKR	674740	1357600	1788
Q9Y4E6	935	WD repeat-containing protein 7	WDR7	0.976744	7.77E-05	141.82	S	RPPRPSTPDLSKARGSPPTSSNIVQGQIKQV	1041300	507280	6909
Q16630	407	Cleavage and polyadenylation specificity factor subunit 6	CPSF6	0.999952	0.000284434	141.82	T	RGDYGPPGREMDTARTPLSEAEFEEIMNRNR	944680	575660	7660
P24928	1891	DNA-directed RNA polymerase II subunit RPB1	POLR2A	0.619922	1.03E-05	141.79	T	PTSPTYSPTTPKYSPTSPTYSPTSPVYTPTS	1393600	0	7322
Q8NDI1	426	EH domain-binding protein 1	EHBP1	0.888723	0.000435325	141.74	S	TGVLNENTVSAGKDLSTSPKPSPIPSPVLGR	0	0	4351
Q9UPU5	2077	Ubiquitin carboxyl-terminal hydrolase 24	USP24	0.993543	1.54E-08	141.74	S	EAEDLSLSAPSSPEISPQSSPRPHRPNNDRL	5644300	1826800	6583
Q9UPU5	2081	Ubiquitin carboxyl-terminal hydrolase 24	USP24	0.998074	1.54E-08	141.74	S	LSLSAPSSPEISPQSSPRPHRPNNDRLSILT	3387100	2899300	6585
Q9NTJ3	44	Structural maintenance of chromosomes protein 4	SMC4	0.837267	3.48E-05	141.74	T	DAEPEPPSGRTESPATAAETASEELDNRSLE	1403100	1205400	8164
Q96ST3	940	Paired amphipathic helix protein Sin3a	SIN3A	0.999447	0.000329237	141.73	S	EWEREVLGIKRDKSDSPAIQLRLKEPMDVDV	0	454310	5138
P49810	27	Presenilin-2;Presenilin-2 NTF subunit;Presenilin-2 CTF subu	PSEN2	0.934471	0.000153685	141.73	T	EVCDERTSLMSAESPTPRSCQEGRQGPEDGE	0	0	7426

Q9NXX6	30	Non-structural maintenance of chromosomes element 4 homolog	NSMCE4A	0.999995	0.000595296	141.71	S	RGRDPHRDRTRSRSRSRSPLSPRSRRGSARE	4493200	2067900	6083
Q9NXX6	32	Non-structural maintenance of chromosomes element 4 homolog	NSMCE4A	0.99999	0.000595296	141.71	S	RDPHRDRTRSRSRSRSPLSPRSRRGSARERR	4493200	2067900	6084
O60841	183	Eukaryotic translation initiation factor 5B	EIF5B	1	0.000792243	141.69	S	EDNSKKIKERSRINSSGESGDESDEFLQSRK	0	225320	530
Q9UQ35	1232	Serine/arginine repetitive matrix protein 2	SRRM2	0.742768	2.16E-06	141.64	S	GSSPETKEQNSALPTSSQDEELMEVVEKSEE	1353800	1414900	6625
P54727	162	UV excision repair protein RAD23 homolog B	RAD23B	0.976371	1.38E-06	141.64	T	KPAEKPAETPVATSPTATDSTSGDSSRSNLF	0	0	7449
O43318	389	Mitogen-activated protein kinase kinase kinase 7	MAP3K7	1	7.50E-05	141.58	S	SSVESLPPTSEGKRMSADMSEIEARIAATTA	1784000	1412800	360
O95453	628	Poly(A)-specific ribonuclease PARN	PARN	0.856052	4.72E-05	141.58	S	RMKKELSPAGSISKNSPATLFEVPDTW____	361590	443920	779
O95453	631	Poly(A)-specific ribonuclease PARN	PARN	0.877907	4.72E-05	141.58	T	KELSPAGSISKNSPATLFEVPDTW_______	361590	0	7240
P57682	250	Krueppel-like factor 3	KLF3	0.998823	0.000165306	141.37	S	PSVIVQPGKRPLPVESPDTQRKRRIHRCDYD	0	0	1898
O14733	58	Dual specificity mitogen-activated protein kinase kinase 7	MAP2K7	0.684627	7.04E-05	141.34	S	LQLPLANDGGSRSPSSESSPQHPTPPARPRH	1128300	0	202
Q8IZ21	116	Phosphatase and actin regulator 4	PHACTR4	0.494328	4.94E-05	141.34	S	AMLKNGHTTPIGNARSSSPVQVEEEPVRLAS	0	0	4181
Q8IZ21	117	Phosphatase and actin regulator 4	PHACTR4	0.494328	4.94E-05	141.34	S	MLKNGHTTPIGNARSSSPVQVEEEPVRLASL	0	0	4182
O43719	579	HIV Tat-specific factor 1	HTATSF1	1	0.00019135	141.3	S	EFEENGLEKDLDEEGSEKELHENVLDKELEE	4249100	4381800	406
Q9NQX3	188	Gephyrin;Molybdopterin adenylyltransferase;Molybdopterin mol	GPHN	1	1.84E-09	141.3	S	IVKVKEVHDELEDLPSPPPPLSPPPTTSPHK	15109000	8446700	5936
Q9NQX3	194	Gephyrin;Molybdopterin adenylyltransferase;Molybdopterin mol	GPHN	0.999886	1.84E-09	141.3	S	VHDELEDLPSPPPPLSPPPTTSPHKQTEDKG	17158000	9845600	5937
Q9NQX3	200	Gephyrin;Molybdopterin adenylyltransferase;Molybdopterin mol	GPHN	0.798341	1.84E-09	141.3	S	DLPSPPPPLSPPPTTSPHKQTEDKGVQCEEE	17158000	7351500	5938
P00558	203	Phosphoglycerate kinase 1	PGK1	1	0.000243598	141.22	S	FLMKKELNYFAKALESPERPFLAILGGAKVA	6352300	4986400	853
Q02880	1471	DNA topoisomerase 2-beta	TOP2B	0.80904	1.10E-05	141.1	S	KSEDDSAKFDSNEEDSASVFSPSFGLKQTDK	821400	497240	2101
Q9H814	350	Phosphorylated adapter RNA export protein	PHAX	0.587675	4.25E-05	141.06	S	KQAIKSLNFQEDDDTSRETFASDTNEALASL	0	1516600	5783
Q9UPT8	1104	Zinc finger CCCH domain-containing protein 4	ZC3H4	0.991081	1.40E-12	141	S	TASSRAAKPGPAEAPSPTASPSGDASPPATA	983590	5026300	6575
Q9UPT8	1114	Zinc finger CCCH domain-containing protein 4	ZC3H4	0.999049	1.40E-12	141	S	PAEAPSPTASPSGDASPPATAPYDPRVLAAG	12166000	11661000	6578
Q9UJV9	23	Probable ATP-dependent RNA helicase DDX41	DDX41	1	5.11E-05	140.96	S	RKRARTDEVPAGGSRSEAEDEDDEDYVPYVP	2458400	4025600	6411
Q99567	517	Nuclear pore complex protein Nup88	NUP88	1	8.39E-05	140.91	S	SPPLLCTREDVEVAESPLRVLAETPDSFEKH	0	0	5208
Q71RC2	722	La-related protein 4	LARP4	1	0.00036199	140.83	S	GVTRRNGKEQYVPPRSPK_____________	514410	306250	3698
P55081	52	Microfibrillar-associated protein 1	MFAP1	0.998104	3.89E-06	140.8	S	KRYVSGKRPDYAPMESSDEEDEEFQFIKKAK	3004400	1889900	1859
Q92576	1642	PHD finger protein 3	PHF3	1	0.000175531	140.78	S	GNVSCSENLVANTARSPQFINLKRDPRQAAG	2355200	1358900	4644
Q99700	936	Ataxin-2	ATXN2	0.805847	0.000189979	140.78	T	FNPRSFSQPKPSTTPTSPRPQAQPSPSMVGH	0	711830	8031
Q15811	986	Intersectin-1	ITSN1	0.89114	9.33E-05	140.77	S	GPIRKSTSMDSGSSESPASLKRVASPAAKPV	405940	243540	2988
Q8N5G2	306	Macoilin	TMEM57	0.5	8.48E-05	140.77	T	NHINSKRLNNDLVGSTENLLKEDSCTASSKN	870390	1125100	7878
P46013	2344	Antigen KI-67	MKI67	1	8.51E-05	140.75	S	DKPTTDEKTTKIACKSPQPDPVDTPASTKQR	0	0	1537
Q86VS8	238	Protein Hook homolog 3	HOOK3	0.999999	9.48E-05	140.68	S	LMERLNQSDSIEDPNSPAGRRHLQLQTQLEQ	476710	399430	3970
Q92538	1784	Golgi-specific brefeldin A-resistance guanine nucleotide exc	GBF1	0.996462	8.50E-05	140.68	S	EKPESPRAASSSSPGSPVASSPSRLSPTPDG	1369400	0	4623
P04049	296	RAF proto-oncogene serine/threonine-protein kinase	RAF1	0.968314	1.22E-12	140.67	S	IRSHSESASPSALSSSPNNLSPTGWSQPKTP	4062500	3753500	870
Q0JRZ9	496	F-BAR domain only protein 2	FCHO2	0.839196	7.01E-13	140.67	S	NEIPRPFSPPVTSNTSPPPAAPLARAESSSS	5371400	0	2285
Q8IYB3	769	Serine/arginine repetitive matrix protein 1	SRRM1	1	1.39E-08	140.67	S	SGSPEPAAKKPPAPPSPVQSQSPSTNWSPAV	251880000	157600000	4141
Q8IYB3	775	Serine/arginine repetitive matrix protein 1	SRRM1	0.999396	6.76E-08	140.67	S	AAKKPPAPPSPVQSQSPSTNWSPAVPVKKAK	98500000	50623000	4143
P13797	540	Plastin-3	PLS3	0.999981	0.000182102	140.49	S	VNWVNRTLSEAGKSTSIQSFKDKTISSSLAV	588950	238960	1050
Q14244	209	Ensconsin	MAP7	0.999999	8.89E-05	140.49	S	VISKRLSSSSATLLNSPDRARRLQLSPWESS	2261700	2268700	2729
Q9Y3S2	296	Zinc finger protein 330	ZNF330	0.603673	4.27E-05	140.46	S	EEEEDEGRKDSDTESSDLFTNLNLGRTYASG	0	0	6872
Q13426	327	DNA repair protein XRCC4	XRCC4	1	7.46E-05	140.45	S	EHISAENMSLETLRNSSPEDLFDEI______	1255000	1771900	2492
Q9Y2W1	682	Thyroid hormone receptor-associated protein 3	THRAP3	0.999293	7.71E-05	140.45	S	AKNKKSPEIHRRIDISPSTFRKHGLAHDEMK	45536000	31427000	6823
P46013	2105	Antigen KI-67	MKI67	1	9.11E-05	140.33	S	EESTTDDKTTKIACKSPPPESMDTPTSTRRR	684260	700630	1535
P49810	25	Presenilin-2;Presenilin-2 NTF subunit;Presenilin-2 CTF subur	PSEN2	0.989812	4.78E-05	140.33	S	EEEVCDERTSLMSAESPTPRSCQEGRQGPED	2154100	1943700	1724
Q07666	18	KH domain-containing, RNA-binding, signal transduction-assoc	KHDRBS1	0.809502	9.11E-05	140.33	S	RRDDPAARMSRSSGRSGSMDPSGAHPSVRQT	0	3834200	2229
Q8NC56	499	LEM domain-containing protein 2	LEMD2	0.995056	0.000215241	140.33	S	GEDMLVWRWTKPSSFSDSER___________	1572800	0	4324
Q9ULU4	406	Protein kinase C-binding protein 1	ZMYND8	0.999873	8.66E-05	140.33	S	IDKQEKVKLNFDMTASPKILMSKPVLSGGTG	1627200	1994600	6504
P49768	366	Presenilin-1;Presenilin-1 NTF subunit;Presenilin-1 CTF subur	PSEN1	0.865723	2.09E-05	140.32	S	HRSTPESRAAVQELSSSILAGEDPEERGVKL	1472100	1523200	1690
P49768	367	Presenilin-1;Presenilin-1 NTF subunit;Presenilin-1 CTF subur	PSEN1	0.819766	2.09E-05	140.32	S	RSTPESRAAVQELSSSILAGEDPEERGVKLG	724330	676010	1691
E9PAV3	2029	Nascent polypeptide-associated complex subunit alpha, muscle	NACA	0.9998	9.23E-18	140.27	S	SNIQENTQTPTVQEESEEEEVDETGVEVKDI	13579000	4659200	88
O15027	1943	Protein transport protein Sec16A	SEC16A	0.731311	5.61E-17	140.26	S	IANPLLAVPAPSPEHSSPSVRLLPSAPQTLP	995800	1058400	245
O15027	1944	Protein transport protein Sec16A	SEC16A	0.608328	5.61E-17	140.26	S	ANPLLAVPAPSPEHSSPSVRLLPSAPQTLPD	995800	1058400	246
O15027	1946	Protein transport protein Sec16A	SEC16A	0.576799	5.61E-17	140.26	S	PLLAVPAPSPEHSSPSVRLLPSAPQTLPDGP	995800	1058400	247
P29966	120	Myristoylated alanine-rich C-kinase substrate	MARCKS	0.782793	6.50E-17	140.19	T	KEAPAEGEAAEPGSPTAAEGEAASAASSTSS	2484300	2124600	7345
Q9UQ35	1451	Serine/arginine repetitive matrix protein 2	SRRM2	0.790034	0.000252365	140.12	S	SRRSRSGSSPGLRDGSGTPSRHSLSGSSPGM	1049500	305090	6608
Q9UQ35	1458	Serine/arginine repetitive matrix protein 2	SRRM2	0.999993	0.000155553	140.12	S	SSPGLRDGSGTPSRHSLSGSSPGMKDIPRTP	7188200	9872400	6610
Q9UQ35	1463	Serine/arginine repetitive matrix protein 2	SRRM2	0.998908	0.000113813	140.12	S	RDGSGTPSRHSLSGSSPGMKDIPRTPSRGRS	9455500	9368900	6613
P36507	394	Dual specificity mitogen-activated protein kinase kinase 2	MAP2K2	0.999231	0.00021006	140.09	T	FAGWLCKTLRLNQPGTPTRTAV_________	25537000	19501000	7368
O75152;A(	758;759	Zinc finger CCCH domain-containing protein 11A	ZC3H11A	0.98171	8.83E-05	139.95	S	SGPSSSQMSMKTRRLSSASTGKPPLSVEDDF	14410000	7870700	572
O75152;A(	761;762	Zinc finger CCCH domain-containing protein 11A	ZC3H11A	0.817804	8.83E-05	139.95	S	SSSQMSMKTRRLSSASTGKPPLSVEDDFEKL	13251000	7870700	574
Q08999	1112	Retinoblastoma-like protein 2	RBL2	0.998724	6.31E-05	139.86	S	TPTKKRGILLEDGSESPAKRICPENHSALLR	766360	603350	2252
Q8IY81	688	pre-rRNA processing protein FTSJ3	FTSJ3	1	5.32E-05	139.86	S	VIASSKKAKRDLIDNSFNRYTFNEDEGELPE	1250800	721230	4123
Q96TA1	691	Niban-like protein 1	FAM129B	0.726166	7.89E-06	139.81	S	APAGESPQPKAAPEASSPPASPLQHLLPGKA	10594000	7246900	5185
Q96TA1	696	Niban-like protein 1	FAM129B	1	7.89E-06	139.81	S	SPQPKAAPEASSPPASPLQHLLPGKAVDLGP	2131300	1803600	5187
O95785	996	Protein Wiz	WIZ	0.993839	5.17E-05	139.78	S	PSSPLLKKTPLALAGSPTPKNPEDKSPQLSL	1665900	1020000	820
O75167	25	Phosphatase and actin regulator 2	PHACTR2	0.999628	0.00011073	139.78	T	KASIANSDGPTAGSQTPPFKRKGKLSTIGKI	982620	601680	7198
Q08945	671	FACT complex subunit SSRP1	SSRP1	0.623374	8.24E-05	139.77	S	LSESFKSKEFVSSDESSSGENKSKKKRRRSE	0	0	2248
Q08945	672	FACT complex subunit SSRP1	SSRP1	0.623371	8.24E-05	139.77	S	SESFKSKEFVSSDESSSGENKSKKKRRRSED	0	0	2249
Q08945	673	FACT complex subunit SSRP1	SSRP1	0.753223	8.24E-05	139.77	S	ESFKSKEFVSSDESSSGENKSKKKRRRSEDS	0	0	2250
Q9UQ35	2583	Serine/arginine repetitive matrix protein 2	SRRM2	0.909439	0.000501636	139.76	T	LPVQPEVALKRVPSPTPAPKEAVREGRPPEP	3524300	0	8266
Q15365	189	Poly(rC)-binding protein 1	PCBP1	0.954203	2.20E-08	139.7	S	PQGRVMTIPYQPMPASSPVICAGGQDRCSDA	5900100	5995100	2905
P56962	288	Syntaxin-17	STX17	0.77212	0.000100686	139.66	S	KLIQRKKQKMMEKLTSSCPDLPSQTDKKCS_	2038100	1406300	1897
O75533	303	Splicing factor 3B subunit 1	SF3B1	1	0.000410516	139.66	T	ARKNRWDETPKTERDTPGHGSGWAETPRTDR	3786900	782780	7205
Q14694	576	Ubiquitin carboxyl-terminal hydrolase 10	USP10	1	2.54E-09	139.59	S	NHSVNEEEQEEQGEGSEDEWEQVGPRNKTSV	24677000	27516000	2801
Q13501	365	Sequestosome-1	SQSTM1	0.800694	2.18E-17	139.56	S	TGELQSLQMPESEGPSSLDPSQEGPTGLKEA	0	3756600	2550
Q13501	366	Sequestosome-1	SQSTM1	0.471394	2.18E-17	139.56	S	GELQSLQMPESEGPSSLDPSQEGPTGLKEAA	0	0	2551
Q01484	3823	Ankyrin-2	ANK2	1	0.000112644	139.49	S	EKLYLQTPTSSERGGSPIIQEPEEPSEHREE	669650	581650	2067
Q8IWI9	607	MAX gene-associated protein	MGA	1	0.000114133	139.49	S	KIATAAKVVNANQNASPNVPGKRGRPRKLKL	1761800	2887200	4059
Q13136	1174	Liprin-alpha-1	PPFIA1	0.652851	6.66E-05	139.48	S	TLPANFRVTSSMSSPSMQPKKMQMDGNVSGT	3133800	1716700	2422
Q01130	206	Serine/arginine-rich splicing factor 2	SRSF2	1	0.000319178	139.46	S	SPPPVSKRESKSRSRSKSPPKSPEEEGAVSS	13846000	8055000	2053
Q01130	208	Serine/arginine-rich splicing factor 2	SRSF2	1	0.000319178	139.46	S	PPVSKRESKSRSRSKSPPKSPEEEGAVSS__	13846000	8055000	2054
Q01130	212	Serine/arginine-rich splicing factor 2	SRSF2	1	0.000319178	139.46	S	KRESKSRSRSKSPPKSPEEEGAVSS______	1154900	1270200	2055
Q96Q42	1462	Alsin	ALS2	0.528991	2.09E-08	139.46	S	PTERKSFCTGKSDSRSESPEPGYVVTSSGLL	0	382290	5068
Q96Q42	1464	Alsin	ALS2	0.494939	2.09E-08	139.46	S	ERKSFCTGKSDSRSESPEPGYVVTSSGLLLP	0	0	5069
Q4VCS5	313	Angiomotin	AMOT	0.372047	2.13E-12	139.45	S	LPLSARNSQPHSPTSSLTSGGSLPLLQSPPS	0	0	3117
Q4VCS5	325	Angiomotin	AMOT	0.836267	2.13E-12	139.45	S	PTSSLTSGGSLPLLQSPPSTRLSPARHPLVP	0	12193000	3118
Q4VCS5	311	Angiomotin	AMOT	0.772128	2.13E-12	139.45	T	ISLPLSARNSQPHSPTSSLTSGGSLPLLQSP	1264700	726550	7686
Q4VCS5	315	Angiomotin	AMOT	0.372047	2.13E-12	139.45	T	LSARNSQPHSPTSSLTSGGSLPLLQSPPSTR	0	0	7687
P27824	554	Calnexin	CANX	1	0.00012895	139.39	S	DEEEEGEEKLEEKQKSDAEEDGGTVSQEEED	3220800	2423600	1300
O95071	1549	E3 ubiquitin-protein ligase UBR5	UBR5	0.999828	0.000234015	139.32	S	SQSSYIIRNPQQRRISQSQPVRGRDEEQDDI	3976300	2416900	726
Q8IYB3	616	Serine/arginine repetitive matrix protein 1	SRRM1	1	0.000443316	139.32	S	QRRYSPSPPPKRRTASPPPPPKRRASPSPPP	9185500	9301300	4162
Q8IYB3	614	Serine/arginine repetitive matrix protein 1	SRRM1	1	0.000443316	139.32	T	PIQRRYSPSPPPKRRTASPPPPPKRRASPSP	3962300	5373600	7865
Q9NYF8	658	Bcl-2-associated transcription factor 1	BCLAF1	0.998401	5.71E-05	139.31	S	STRQKSPEIHRRIDISPSTLRKHTRLAGEER	64691000	57298000	6106
Q9P206	342	Uncharacterized protein KIAA1522	KIAA1522	0.799727	9.93E-05	139.31	S	SPASVRSLGRFSSVSSPQPRSRHPSSSSDTW	2043300	1025300	6209
Q9UQ35	2690	Serine/arginine repetitive matrix protein 2	SRRM2	0.999984	4.75E-05	139.31	S	SRSPRKPIDSLRDSRSLSYSPVERRRPSPQP	5024900	4720300	6695
Q9UQ35	2694	Serine/arginine repetitive matrix protein 2	SRRM2	0.999994	4.75E-05	139.31	S	RKPIDSLRDSRSLSYSPVERRRPSPQPSPRD	30596000	17380000	6697
P24928	1881	DNA-directed RNA polymerase II subunit RPB1	POLR2A	0.422808	0.00194787	139.3	Y	KYSPTSPKYSPTSPTYSPTTPKYSPTSPTYS	0	0	8359
P78312	293	Protein FAM193A	FAM193A	0.999859	1.74E-08	139.28	S	PRLILTDSGSAPTFCSDDEDVAPLSAKFADI	4899700	4206800	1951
A2RU67	30	Uncharacterized protein KIAA1467	KIAA1467	0.999968	1.18E-12	139.25	S	KSPDLGEYDPLTQADSDESEDDLVLNLQKNG	4202600	2690400	22
A2RU67	33	Uncharacterized protein KIAA1467	KIAA1467	1	1.18E-12	139.25	S	DLGEYDPLTQADSDESEDDLVLNLQKNGGVK	4202600	2690400	23
Q9BVG4	197	Protein PBDC1	PBDC1	1	0.000482971	139.21	S	GEEENTKNGGEKGADSGEEKEEGINREDKTD	0	0	5400
P85037	422	Forkhead box protein K1	FOXK1	0.696857	0.000922101	139.21	T	TPFGPLSSRSAPASPTHPGLMSPRSGGLQTP	0	5634100	7465
Q15365	190	Poly(rC)-binding protein 1	PCBP1	0.947003	2.36E-08	139.2	S	QGRVMTIPYQPMPASSPVICAGGQDRCSDAA	1936700	1352900	2906
Q9H501	75	ESF1 homolog	ESF1	0.999986	0.00265651	139.2	S	ISHSTTEDLKRFYDLSDSDSNLSGEDSKALS	0	0	5708
Q9H501	77	ESF1 homolog	ESF1	1	0.00265651	139.2	S	HSTTEDLKRFYDLSDSDSNLSGEDSKALSQK	0	0	5709
Q9H501	79	ESF1 homolog	ESF1	0.999999	0.00265651	139.2	S	TTEDLKRFYDLSDSDSNLSGEDSKALSQKKI	0	0	5710
Q9H501	82	ESF1 homolog	ESF1	0.999847	0.00265651	139.2	S	DLKRFYDLSDSDSNLSGEDSKALSQKKIKKK	360880	0	5711
Q16539	2	Mitogen-activated protein kinase 14	MAPK14	0.999999	4.80E-05	139.15	S	_______________MSQERPTFYRQELNKTI	7830200	66103000	3014
Q8N5G2	332	Macoilin	TMEM57	0.776179	0.000106889	139.15	S	ASSKNYKNASGVVNSSPRSHSATNGSIPSSS	0	0	4285
Q13428	173	Treacle protein	TCOF1	0.640774	2.37E-12	139.12	T	RKSAEPSANTTLVSETEEEGSVPAFGAAAKP	1012700	833710	7536
Q9Y450	64	HBS1-like protein	HBS1L	0.492482	6.69E-05	139.08	S	SVEPVEEYDYEDLKESSNSVSNHQLSGFDQA	0	0	6888
Q9Y450	65	HBS1-like protein	HBS1L	0.492482	6.69E-05	139.08	S	VEPVEEYDYEDLKESSNSVSNHQLSGFDQAR	0	0	6889
O14617	634	AP-3 complex subunit delta-1	AP3D1	1	1.44E-12	139.04	S	EGLDLDAWINEPLSDSESEDERPRAVFHEEE	15571000	9831600	157
Q04323	199	UBX domain-containing protein 1	UBXN1	0.43724	3.22E-13	139	S	GSQPPPVAPEPGPVPSSPSQEPPTKREYDQC	0	0	2153
Q5VT52	484	Regulation of nuclear pre-mRNA domain-containing protein 2	RPRD2	0.903279	0.00032142	139	T	NTGVSPASRPSPGTPTSPSNLTSGLKTPAPA	5009400	1967500	7732
Q8N8A6	83	ATP-dependent RNA helicase DDX51	DDX51	1	0.000122062	138.98	S	RRPRRRRRVNDAEPGSPEAPQGKRRKADGED	1267000	2208000	4298
Q12888	831	Tumor suppressor p53-binding protein 1	TP53BP1	0.815338	1.32E-06	138.97	S	LKSGTAETEPVEQDSSQPSLPLVRADDPLRL	0	0	2366
Q8IZP0	183	Abl interactor 1	ABI1	0.999884	0.000542858	138.97	S	TGTLSRTNPPTQKPPSPPMSGRGTLGRNTPY	0	895380	4191
Q9UKV5	523	E3 ubiquitin-protein ligase AMFR	AMFR	0.964109	1.17E-08	138.82	S	DSIRPALNSPVERPSSDQEEGETSAQTERVP	1470900	811620	6460
O60749	104	Sorting nexin-2	SNX2	0.985015	2.48E-08	138.82	T	EPILSSEPSPAVTPVTPTTLIAPRIESKSMS	1028500	1412900	7185
Q14839	531	Chromodomain-helicase-DNA-binding protein 4	CHD4	1	2.80E-07	138.8	S	PTPVPRPPDADPNTPSPKPLEGRPERQFFVK	21786000	33553000	2821
Q14839	517	Chromodomain-helicase-DNA-binding protein 4	CHD4	0.837973	2.80E-07	138.8	T	VQKILIWKWGQPPSPTPVPRPPDADPNTPSP	780750	625230	7625
P42858	1874	Huntingtin	HTT	0.999997	6.95E-05	138.77	S	KRHSLSSTKLLSPQMSGEEEDSDLAAKLGMC	1262900	376190	1519
Q13242	211	Serine/arginine-rich splicing factor 9	SRSF9	1	0.000226841	138.77	S	RSGSRGRDSPYQSRGSPHYFSPFRPY_____	12630000	8854000	2435
Q13242	216	Serine/arginine-rich splicing factor 9	SRSF9	1	0.000226841	138.77	S	GRDSPYQSRGSPHYFSPFRPY__________	11362000	8804700	2436
Q9UKV3	410	Apoptotic chromatin condensation inducer in the nucleus	ACIN1	0.93766	3.88E-08	138.77	S	DRKKASLVALPEQTASEEETPPPLLTKEASS	1959100	2673900	6453
Q96JM3	436	Chromosome alignment-maintaining phosphoprotein 1	CHAMP1	1	6.10E-05	138.76	S	KPGPPLSPEIRSPAGSPELRKPSGSPDLWKL	0	0	4993
Q96N67	888	Dedicator of cytokinesis protein 7	DOCK7	1	0.000220605	138.76	S	SVHYATMARSAVRPASLNLNRSRSLSNSNPD	1746800	1444200	5031
P35221	634	Catenin alpha-1	CTNNA1	0.976902	5.43E-05	138.75	T	YDGIRDIRKAVLMIRTPEELDDSDFETEDFD	0	0	7358
Q8ND24	15	RING finger protein 214	RNF214	0.788839	8.17E-09	138.66	S	_MAASEVAGVVANAPSPPESSSLCASKSDEG	0	0	4335
Q9UQ35	1462	Serine/arginine repetitive matrix protein 2	SRRM2	0.991132	0.000113813	138.63	S	LRDGSGTPSRHSLSGSSPGMKDIPRTPSRGR	9455500	4106200	6612
Q7Z309	51	Protein FAM122B	FAM122B	0.658965	0.000331766	138.63	T	SQVFQPYTLRTRRNSTTIMSRHSLEEGLDMV	0	0	7800
Q8IY92	1329	Structure-specific endonuclease subunit SLX4	SLX4	0.986114	2.84E-12	138.59	S	QTPPPQTPSSCLTPVSPGTSDGRRQGHRSPS	4853400	3779300	4126
Q8IY92	1315	Structure-specific endonuclease subunit SLX4	SLX4	0.999156	2.84E-12	138.59	T	EGNEVAQKFSVIRPQTPPPQTPSSCLTPVSP	4853400	3779300	7858
Q08J23	751	tRNA (cytosine(34)-C(5))-methyltransferase	NSUN2	0.999999	3.40E-13	138.57	S	QRAGEPNSPDAEEANSPDVTAGCDPAGVHPP	82424000	64046000	2266
A0MZ66	506	Shootin-1	KIAA1598	1	7.52E-05	138.55	S	DSSSPTGILATSESKSMPVLGSVSSVTKTAL	579630	401140	9
Q9H0D6	448	5'-3' exoribonuclease 2	XRN2	0.999993	6.05E-05	138.55	S	TPSGILTPHALGSRNSPGSQVASNPRQAAYE	17394000	11231000	5575
P12268	416	Inosine-5'-monophosphate dehydrogenase 2	IMPDH2	1	0.000110007	138.45	S	FFSDGIRLKKYRGMGSLDAMDKHLSSQNRYF	3380400	1828400	1031
Q9C0C2	178	182 kDa tankyrase-1-binding protein	TNKS1BP1	1	0.000260423	138.42	S	EILAKMEQPRKEVLASPDRLWGSRLTFNHDG	421830	533810	5520
P46013	1131	Antigen KI-67	MKI67	1	0.000120122	138.31	S	EESMTDEKTTKIACKSPPPESVDTPTSTKQW	1054800	1159500	1536

A2RU67	62	Uncharacterized protein KIAA1467	KIAA1467	1	8.39E-10	138.28	S	VKNGKSPLGEAPEPDSDAEVAEAAKPHLSEV	1356200	1473400	24
O43684	211	Mitotic checkpoint protein BUB3	BUB3	1	0.000120474	138.28	S	LSSIEGRVAVEYLDPSPEVQKKKYAFKCHRL	2608900	1001700	405
P61244	142	Protein max	MAX	0.842082	6.97E-05	138.28	S	KGSTISAFDGGSDSSSESEPEEPQSRKKLRM	0	0	1908
P61244	144	Protein max	MAX	0.999924	6.97E-05	138.28	S	STISAFDGGSDSSSESEPEEPQSRKKLRMEA	1212100	255310	1909
Q14004	383	Cyclin-dependent kinase 13	CDK13	0.999728	0.000133059	138.28	S	PSYSRHSSYERGGDVSPSPYSSSSWRRSRSP	872410	422130	2635
Q9UDY2	986	Tight junction protein ZO-2	TJP2	1	0.000542986	138.28	S	AQMRRAASSDQLRDNSPPPAFKPEPPKAKTQ	9197900	5218800	6277
Q9UQ35	2382	Serine/arginine repetitive matrix protein 2	SRRM2	0.998662	0.000120474	138.28	S	TSARMAPALSGANLTSPRVPLSAYERVSGRT	4203900	1952800	6655
O15027	575	Protein transport protein Sec16A	SEC16A	0.890955	5.57E-06	138.25	T	GSFFKQIDSSPVGGETDETTVSQNYRGSVSQ	0	0	7142
P24928	1919	DNA-directed RNA polymerase II subunit RPB1	POLR2A	0.661417	0.000493249	138.25	T	PTSPKYSPTSPTYSPTSPKYSPTSPTYSPTS	0	4761800	7321
Q07157	329	Tight junction protein ZO-1	TJP1	1	6.29E-05	138.22	S	RSPDQRSEPSDHSRHSPQQPSNGSLRSRDEE	1744400	757640	2223
Q12830	1231	Nucleosome-remodeling factor subunit BPTF	BPTF	0.999765	0.000113049	138.2	S	GIGKTSTNSSKNLSESPVITKAKEGCQSDSM	1020500	742660	2312
Q13813	1217	Spectrin alpha chain, non-erythrocytic 1	SPTAN1	1	6.50E-05	138.2	S	VATFNSIKELNERWRSLQQLAEERSQLLGSA	5550200	4036200	2627
Q3KQU3	72	MAP7 domain-containing protein 1	MAP7D1	0.5	0.000132064	138.2	S	NATSSKQLPLEPESPSGQVGPRPAPPQEESP	2192000	0	3069
P49736	108	DNA replication licensing factor MCM2	MCM2	0.999855	1.26E-12	138.15	S	EGLALDDEDVEELTASQREAAERAMRQRDRE	5548500	3210800	1677
Q96BT3	397	Centromere protein T	CENPT	0.999484	0.000119841	138.05	S	PGASSGDEDASGRAASPESASSTPESLQARR	1321700	498810	4807
Q9HCD6	169	Protein TANC2	TANC2	0.568594	0.000119841	138.05	S	STNATAKDCSYGAVTSPTSTLESRDSGIIAT	0	0	5846
Q8IWS0	154	PHD finger protein 6	PHF6	0.5	1.74E-08	138.04	S	EADLEESFNEHELEPSSPKSKKKSRKGRPRK	0	6248400	4075
Q8IWS0	155	PHD finger protein 6	PHF6	0.981819	1.74E-08	138.04	S	ADLEESFNEHELEPSSPKSKKKSRKGRPRKT	9895800	8725500	4076
P16615	663	Sarcoplasmic/endoplasmic reticulum calcium ATPase 2	ATP2A2	1	0.000122661	137.95	S	TSKAFTGREFDELNPSAQRDACLNARCFARV	1040500	1175800	1092
P78362	497	SRSF protein kinase 2;SRSF protein kinase 2 N-terminal;SRSF	SRPK2	0.638151	6.48E-05	137.95	S	ESSPSHDRSRTVSASSTGDLPKAKTRAADLL	1138100	0	1966
Q8IYB3	605	Serine/arginine repetitive matrix protein 1	SRRM1	0.999952	0.000319174	137.95	S	SPSPRRYSPPIQRRYSPSPPPKRRTASPPPP	16533000	10436000	4166
Q8IYB3	607	Serine/arginine repetitive matrix protein 1	SRRM1	1	0.000319174	137.95	S	SPRRYSPPIQRRYSPSPPPKRRTASPPPPPK	16533000	10436000	4167
O43314	1006	Inositol hexakisphosphate and diphosphoinositol-pentakisphos	PPIP5K2	0.99998	8.08E-05	137.93	S	LHYTSGVGTGRRRRRSGEQITSSPVSPKSLA	2102700	1703800	359
Q6IN85	126	Serine/threonine-protein phosphatase 4 regulatory subunit 3/	SMEK1	0.506496	3.56E-08	137.89	S	QDLVDESEEERFDDMSSPGLELPSCELSRLE	2801600	4321100	3475
Q6P2E9	875	Enhancer of mRNA-decapping protein 4	EDC4	0.963518	8.31E-13	137.86	S	RPPYHLLQQRDSQDASAEQSDHDDEVASLAS	0	6307200	3514
Q16181	334	Septin-7	SEPT7	1	0.000117636	137.84	S	YNGVDNNKNKGQLTKSPLAQMEEERREHVAK	479070	715240	3001
Q9H4A3	167	Serine/threonine-protein kinase WNK1	WNK1	0.997983	0.000370353	137.79	S	APSTVPSSTSKDRPVSQPSLVGSKEEPPPAR	0	483580	5660
Q9UK61	971	Protein FAM208A	FAM208A	0.5	0.000138362	137.79	S	QEAFPNDPRVINRQRSSDYQFPSSPFTDTLK	0	0	6427
Q9UK61	972	Protein FAM208A	FAM208A	0.5	0.000138362	137.79	S	EAFPNDPRVINRQRSSDYQFPSSPFTDTLKG	0	0	6428
O75147	10	Obscurin-like protein 1	OBSL1	0.999972	0.000128454	137.73	S	______MKASSGDQGSPPCFLRFPRPVRVVS	1517800	935620	567
Q9BUR4	54	Telomerase Cajal body protein 1	WRAP53	1	5.69E-05	137.73	S	ELMPPPPERGDPPRLSPDPVAGSAVSQELRE	1098600	1025500	5386
Q9H2Y7	1026	Zinc finger protein 106	ZNF106	0.997453	0.000141763	137.73	S	GQSIRKKRRATGDGSSPELPSLERKNKRRKI	3499900	1099100	5625
Q12802	983	A-kinase anchor protein 13	AKAP13	0.998271	0.000139823	137.69	S	ISADCAKDKALQLSNSPGASSAFLKAETEHN	1048300	471950	2306
Q7Z6Z7	2391	E3 ubiquitin-protein ligase HUWE1	HUWE1	0.941894	8.54E-13	137.62	S	ESQEDVLMDEAPSNLSQASTLQANREDSMNI	2093300	915480	3884
Q9H4G0	546	Band 4.1-like protein 1	EPB41L1	0.99364	0.000126979	137.62	S	DWERERRLPSSPASPSPKGTPEKANERAGLR	847730	737250	5671
O75592	3505	E3 ubiquitin-protein ligase MYCBP2	MYCBP2	0.999999	0.000131394	137.52	S	ILPARVKAIPRRRVNSGDTEVGSSLLRHPSP	0	1750400	648
P29590	518	Protein PML	PML	1	0.000109224	137.52	S	RSSPEQPRPSTSKAVSPPHLDGPPSPRSPVI	10435000	3902900	1321
P29590	527	Protein PML	PML	1	0.000109224	137.52	S	STSKAVSPPHLDGPPSPRSPVIGSEVFLPNS	10435000	3902900	1322
Q9H4A3	2029	Serine/threonine-protein kinase WNK1	WNK1	0.998037	6.65E-05	137.52	S	PEAAFLSRDVDDGSGSPHSPHQLSSKSLPSQ	805390	811130	5663
Q9H4A3	2032	Serine/threonine-protein kinase WNK1	WNK1	0.999953	6.65E-05	137.52	S	AFLSRDVDDGSGSPHSPHQLSSKSLPSQNLS	805390	1955000	5664
P29558	41	RNA-binding motif, single-stranded-interacting protein 1	RBMS1	0.50325	3.70E-20	137.51	T	QSLVPAHPMAPPSPSTTSSNNNSSSSSNSGW	6533800	0	7342
O60508	22	Pre-mRNA-processing factor 17	CDC40	0.847865	1.64E-12	137.45	S	ALAASYGSGSGSESDSDSESSRCPLPAADSL	0	0	507
P05387	86	60S acidic ribosomal protein P2	RPLP2	1	4.42E-17	137.42	S	VAVSAAPGSAAPAAGSAPAAAEEKKDEKKEE	2966700	2648500	898
Q13425	222	Beta-2-syntrophin	SNTB2	0.950379	3.64E-13	137.42	S	PSLVSDLPWEGAAPQSPSFSGSEDSGSPKHQ	1401200	861720	2486
O60293	949	Zinc finger C3H1 domain-containing protein	ZFC3H1	0.989957	0.000123106	137.4	S	EQDRFGPNKMMRLDSSPVSSPRKHSAELIAM	1460900	646400	474
P39880	1237	Homeobox protein cut-like 1	CUX1	0.886505	6.03E-17	137.4	S	PCEPPSVGTEYSQGASPQPQHQLKKPRVVLA	0	1489100	1467
Q9H0E9	615	Bromodomain-containing protein 8	BRD8	0.974114	6.88E-05	137.4	S	ETQHKFEMSDSLKEESGTIFGSQIKDAPGED	784160	0	5578
Q9HCK8	2046	Chromodomain-helicase-DNA-binding protein 8	CHD8	0.999829	6.88E-05	137.4	S	VARSRPTPQDYEMRVSPSDTTPLVSRSVPPV	8758800	5426600	5871
P06493;P	14;14;14	Cyclin-dependent kinase 1;Cyclin-dependent kinase 2;Cyclin-c	CDK1;CDK2;	1	0.000172359	137.4	T	__MEDYTKIEKIGEGTYGVVYKGRHKTTGQV	41247000	40274000	7263
O15085	1295	Rho guanine nucleotide exchange factor 11	ARHGEF11	1	6.88E-05	137.39	S	MGLCSLEHLPPRTRNSGIWESPELDRNLAED	2335300	1441400	284
O15344	98	E3 ubiquitin-protein ligase Midline-1	MID1	0.499513	8.58E-05	137.39	S	IDRFQKASVSGPNSPSETRRERAFDANTMTS	0	0	312
O94979	799	Protein transport protein Sec31A	SEC31A	1	8.58E-05	137.39	S	DRLCRAQGEPVAGHESPKIPYEKQQLPKGRP	0	0	722
P24928	1843	DNA-directed RNA polymerase II subunit RPB1	POLR2A	0.648698	6.11E-05	137.39	S	SSPSYSPASPKYTPTSPSYSPSSPEYTPTSP	845160	243500	1253
P24928	1856	DNA-directed RNA polymerase II subunit RPB1	POLR2A	0.567099	6.11E-05	137.39	T	PTSPSYSPSSPEYTPTSPKYSPTSPKYSPTS	845160	243500	7325
Q9H9L4	149	KAT8 regulatory NSL complex subunit 2	KANSL2	1	1.59E-06	137.38	S	SSRSEASRILDEDSWSDGEQEPITVDQTWRG	2524600	1495900	5807
Q04724	284	Transducin-like enhancer protein 1	TLE1	0.568284	3.64E-05	137.32	S	RENGIDKNRLLKKDASSSPASTASSASSTSL	0	1950900	2168
Q5TCZ1	642	SH3 and PX domain-containing protein 2A	SH3PXD2A	0.771514	8.16E-05	137.32	S	PYAEDTLSARGSSGDSDSPGSSSLSLTRKNS	820340	474360	3300
Q92945	184	Far upstream element-binding protein 2	KHSRP	1	7.19E-05	137.32	S	KIQQDSGCKVQISPDSGGLPERSVSLTGAPE	0	2433100	4738
Q96G74	165	OTU domain-containing protein 5	OTUD5	1	5.38E-07	137.32	S	KRRRQAPGVGAVGGGSPEREEVGAGYNSEDE	15345000	8988800	4914
Q9H7N4	734	Splicing factor, arginine/serine-rich 19	SCAF1	0.980464	0.0001116	137.32	S	SPAPASSPKREVLYDSEGLSGEERGGKSSQK	1326300	659270	5766
Q7Z6Z7	3830	E3 ubiquitin-protein ligase HUWE1	HUWE1	0.997538	2.70E-16	137.3	T	PSPSAQDTQSIASDGTPQGEKEKEERPPELP	1091600	563730	7818
P24928	1913	DNA-directed RNA polymerase II subunit RPB1	POLR2A	0.993687	0.00105003	137.27	S	TSPVYTPTSPKYSPTSPTYSPTSPKYSPTSP	7973400	5256100	1243
Q9NTI5	1165	Sister chromatid cohesion protein PDS5 homolog B	PDS5B	0.653435	4.07E-05	137.27	S	SRMETVSNASSSSNPSSPGRIKGRLDSSEMD	2340900	0	6011
Q96L73	2471	Histone-lysine N-methyltransferase, H3 lysine-36 and H4 lysi	NSD1	1	0.000136492	137.17	S	MDLIDLTPRQKERAASPHQVTPQADEKMPVL	924350	424330	5010
P35658	657	Nuclear pore complex protein Nup214	NUP214	0.883437	0.000150648	137.16	S	SVLPSPSGRSAQGSSSPVPSMVQKSPRITPP	3401000	2539100	1416
Q13573	234	SNW domain-containing protein 1	SNW1	0.725885	0.000125643	137.13	S	PRGPPSPPAPVMHSPSRKMTVKEQQEWKIPP	1574300	622290	2583
Q99638	375	Cell cycle checkpoint control protein RAD9A	RAD9A	0.999647	0.000109798	137.13	S	KFRSLFFGSILAPVRSPQGPSPVLAEDSEGE	1609600	1367300	5239
P49454	1651	Centromere protein F	CENPF	0.772043	0.000133984	137.09	S	LELEVARLQLQGLDLSSRSLLGIDTEDAIQG	840930	536460	1665
O43426	1049	Synaptojanin-1	SYNJ1	0.56768	3.47E-08	137.04	S	SSSGLGTSPSSSPRTSPCQSPTISEGPVPSL	0	6066300	381
O43426	1053	Synaptojanin-1	SYNJ1	0.999737	3.47E-08	137.04	S	LGTSPSSSPRTSPCQSPTISEGPVPSLPIRP	0	6066300	382
Q13283	149	Ras GTPase-activating protein-binding protein 1	G3BP1	1	1.97E-12	137.04	S	QDEVFGGFVTEPQEESEEEVEEPEERQQTPE	47256000	51698000	2466
P29966	145	Myristoylated alanine-rich C-kinase substrate	MARCKS	0.934556	0.000153036	137.01	S	ASSTSSPKAEDGATPSPSNETPKKKKKRFSF	0	0	1326
Q13153	144	Serine/threonine-protein kinase PAK 1	PAK1	0.999733	0.00220913	136.99	S	EFYNSKKTSNSQKYMSFTDKSAEDYNSSNAL	1820200	980810	2426
O14686	4738	Histone-lysine N-methyltransferase 2D	KMT2D	1	0.000135765	136.96	S	PHLGSGRWEQEDRALSPVIPLIPRASIPVFP	981800	615380	188
Q6PCB5	6	Round spermatid basic protein 1-like protein	RSBN1L	1	1.42E-06	136.96	S	__________MAEPPSPVHCVAAAAPTATVS	1731500	1764600	3535
Q9UQ35	2368	Serine/arginine repetitive matrix protein 2	SRRM2	0.812841	0.000139494	136.96	S	AGSRTAAALAPASLTSARMAPALSGANLTSP	1311700	692860	6744
Q14157	605	Ubiquitin-associated protein 2-like	UBAP2L	0.994108	0.000113121	136.93	S	PLYEQRSTQTRRYPSSISSSPQKDLTQAKNG	4678800	0	2660
Q53EU6	68	Glycerol-3-phosphate acyltransferase 3	AGPAT9	0.999992	8.00E-05	136.93	S	IEKGTPKESILKNSASVGIIQRDESPMEKGL	450210	304080	3133
Q14168	145	MAGUK p55 subfamily member 2	MPP2	0.999766	5.08E-09	136.88	S	THDSVASKTYETPPPSPGLDPTFSNQPVPPD	1932500	1661000	2693
Q14168	141	MAGUK p55 subfamily member 2	MPP2	0.899637	5.08E-09	136.88	T	SLLETHDSVASKTYETPPPSPGLDPTFSNQP	1932500	0	7586
Q8WWM7	630	Ataxin-2-like protein	ATXN2L	0.716419	1.84E-17	136.86	S	SGGTEGPEQPPPPCPSQTGSPPVGLIKGEDK	0	1138800	4558
P07197	30	Neurofilament medium polypeptide	NEFM	0.999392	0.000130446	136.81	S	RRVTETRSSFSRVSGSPSSGFRSQSWSRGSP	2458100	1189600	932
Q8WYP5	1218	Protein ELYS	AHCTF1	0.994008	0.000524368	136.81	S	LRSTLRSTPLASPSPSPGRSPQRLKETRISF	985860	480530	4608
Q8WYP5	1222	Protein ELYS	AHCTF1	0.999946	0.000524368	136.81	S	LRSTPLASPSPSPGRSPQRLKETRISFVEED	985860	480530	4609
P11274	459	Breakpoint cluster region protein	BCR	0.99853	4.46E-06	136.77	S	EEQRRHQDGLPYIDDSPSSSPHLSSKGRGSR	3247200	2493700	1011
P11274	463	Breakpoint cluster region protein	BCR	0.741087	4.46E-06	136.77	S	RHQDGLPYIDDSPSSSPHLSSKGRGSRDALV	0	216240	1013
Q99567	35	Nuclear pore complex protein Nup88	NUP88	0.951176	4.53E-09	136.77	S	NHVVFLRLREGLKNQSPTEAEKPASSSLPSS	5608300	10645000	5209
Q9Y6A5	175	Transforming acidic coiled-coil-containing protein 3	TACC3	0.5	1.36E-05	136.77	S	SPGSSENQMVSPGKVSGSPEQAVEENLSSYS	0	0	6990
Q66K14	432	TBC1 domain family member 9B	TBC1D9B	0.999518	8.88E-17	136.74	S	TESSPAPQEGSEQPASPASPLSSRQSFCAQE	3580100	2972700	3400
Q00839	59	Heterogeneous nuclear ribonucleoprotein U	HNRNPU	1	2.28E-08	136.67	S	DEEAGGRPAMEPGNGSLDLGGDSAGRSGAGL	1608700	2238300	2037
Q8WUI4	487	Histone deacetylase 7	HDAC7	0.650206	8.45E-06	136.67	S	PLAQGGHRPLSRAQSSPAAPASLSAPEPASQ	2045600	0	4516
Q9UKV3	838	Apoptotic chromatin condensation inducer in the nucleus	ACIN1	0.988515	0.000155363	136.66	S	KISVVSATKGVPAGNSDTEGGQPGRKRRWGA	2436700	2170200	6454
Q9ULL5	651	Proline-rich protein 12	PRR12	1	1.93E-05	136.66	S	TEDEEFLIQHLLQAPSPPRTSGADGLVGEDG	1207800	552630	6491
Q9UEY8	64	Gamma-adducin	ADD3	1	0.000140406	136.6	S	FNMMEQRKRVTQILQSPAFREDLECLIQEQM	1073400	915820	6306
Q9UQ35	1842	Serine/arginine repetitive matrix protein 2	SRRM2	0.999876	0.00237435	136.53	S	RQESSRTSSRRRRGRSRTPPTSRKRSRSRTS	3580600	2880100	6725
Q9UK58	67	Cyclin-L1	CCNL1	0.740697	5.66E-05	136.53	T	LTIDHSLIPEERLSPTPSMQDGLDLPSETDL	519490	397010	8228
Q9UQ35	1847	Serine/arginine repetitive matrix protein 2	SRRM2	0.995061	0.00237435	136.53	T	RTSSRRRRGRSRTPPTSRKRSRSRTSPAPWK	3580600	2880100	8273
Q7Z401	1589	C-myc promoter-binding protein	DENND4A	0.998062	1.84E-07	136.5	S	TAMSKCPIFPMARSISTSGPLDKEDTGRQKL	0	929230	3790
P18583	2009	Protein SON	SON	1	0.000225632	136.49	S	TPSRRRRSRSVVRRRSFSISPVRLRRSRTPL	4865300	3128600	1138
Q9H6H4	152	Receptor expression-enhancing protein 4	REEP4	0.999635	0.000271429	136.49	S	ATKSQGALAGRLRSFSMQDLRSISDAPAPAY	2770500	1265000	5734
O14920	672	Inhibitor of nuclear factor kappa-B kinase subunit beta	IKBKB	0.996323	0.000147555	136.39	S	LLKIACSKVRGPVSGSPDSMNASRLSQPGQL	1674300	1204600	216
P00519	805	Tyrosine-protein kinase ABL1	ABL1	0.817672	0.00011791	136.39	S	NEEAADEVFKDIMESSPGSSPPNLTPKPLRR	0	664430	851
P00519	809	Tyrosine-protein kinase ABL1	ABL1	0.642293	0.00011791	136.39	S	ADEVFKDIMESSPGSSPPNLTPKPLRRQVTV	0	664430	852
Q8TCN5	195	Zinc finger protein 507	ZNF507	0.966879	9.50E-05	136.39	S	NDANIHTQSKAQQCVSPSSSLCRKTTERNET	1251500	778160	4461
Q9HCK8	2519	Chromodomain-helicase-DNA-binding protein 8	CHD8	0.998545	8.97E-05	136.36	S	HHHHHPGLRAPGYPSSPVTTASGTTLRLPPL	1461800	559890	5861
Q9Y4H2	915	Insulin receptor substrate 2	IRS2	0.999998	0.000159895	136.36	S	SLPSMHEYPLPPEPKSPGEYINIDFGEPGAR	0	442370	6927
O96013	148	Serine/threonine-protein kinase PAK 4	PAK4	1	0.000148466	136.33	S	GSRGRFAGHSEAGGGSGDRRRAGPEKRPKSS	2037800	363370	844
Q3KQU3	399	MAP7 domain-containing protein 1	MAP7D1	1	0.000313945	136.33	S	PAGERGERRKPNAGGSPAPVRRRPEASPVQK	1679100	895430	3062
Q9Y3T9	49	Nucleolar complex protein 2 homolog	NOC2L	1	0.00043083	136.33	S	NSPQAETREAREAARSPDKPGGSPSASRRKG	12052000	5024900	6875
Q9BXI6	22	TBC1 domain family member 10A	TBC1D10A	0.999607	0.000311714	136.33	T	ENGPRAPAAGESLSGTRESLAQGPDAATTDE	840140	683540	8063
Q05519	449	Serine/arginine-rich splicing factor 11	SRSF11	0.934941	9.41E-07	136.27	S	SEKEKKEEKKPIETGSPKTKECSVEKGTGDS	12802000	11087000	2188
Q14814	180	Myocyte-specific enhancer factor 2D	MEF2D	1	0.000144728	136.27	S	TPSLVTSSLTDPRLLSPQQPALQRNSVSPGL	797600	852440	2812
P98164	4616	Low-density lipoprotein receptor-related protein 2	LRP2	0.977245	0.0012559	136.26	S	MENEQKESVAATPPPSPSLPAKPKPPSRRDP	1082800	0	2008
Q9H4L7	132	SWI/SNF-related matrix-associated actin-dependent regulator	SMARCAD1	0.999758	3.24E-08	136.26	S	KDTVIIVSEPSEDEESQGLPTMARRNDDISE	3367800	4474600	5685
Q5H9R7	634	Serine/threonine-protein phosphatase 6 regulatory subunit 3	PPP6R3	1	0.00106927	136.24	S	EEDIWEEKHIAFTPESQRRSSSGSTDSEEST	1544100	974010	3165
Q5H9R7	631	Serine/threonine-protein phosphatase 6 regulatory subunit 3	PPP6R3	1	0.00106927	136.24	T	GSDEEDIWEEKHIAFTPESQRRSSSGSTDSE	1544100	974010	7699
Q8WWM7	634	Ataxin-2-like protein	ATXN2L	0.773181	1.97E-17	136.18	S	EGPEQPPPPCPSQTGSPPVGLIKGEDKDEGP	4122800	2763300	4559
Q04726	238	Transducin-like enhancer protein 3	TLE3	0.80099	3.64E-08	136.16	Y	EAKKRKAEEKDSLSRYDSDGDKSDDLVVDVS	5438600	0	8373
Q8N3F8	484	MICAL-like protein 1	MICALL1	1	8.10E-05	136.14	S	TSSPKTKKRPAPRAPSASPLALHASRLSHSE	3684100	2909800	4248
Q8N3F8	486	MICAL-like protein 1	MICALL1	1	8.10E-05	136.14	S	SPKTKKRPAPRAPSASPLALHASRLSHSEPP	3684100	2909800	4249
Q9UEY8	679	Gamma-adducin	ADD3	0.767039	9.74E-05	136.14	S	SPEKIEEVLSPEGSPSKSPSKKKKKFRTPSF	16407000	8493000	6301
Q8N163	681	Cell cycle and apoptosis regulator protein 2	CCAR2	0.999988	0.000777938	136.11	S	AKLEDSEVRSVASNQSEMEFSSLQDMPKELD	0	0	4215
Q32MZ4	521	Leucine-rich repeat flightless-interacting protein 1	LRRFIP1	0.997149	5.08E-17	136.1	S	TGLRDEKPIKTEVPGSPAGTEGNCQEATGPS	791370	600050	3056
P46379	1081	Large proline-rich protein BAG6	BAG6	0.999492	0.000140203	136.02	S	AVSRAAKAAGARPLTSPESLSRDLEAPEVQE	999510	930450	1567
Q5QJE6	117	Deoxynucleotidyltransferase terminal-interacting protein 2	DNTTIP2	0.987077	9.84E-05	136.02	S	TILRVTRRRQILIACSPVSSVRKKPKVTPTK	1545800	1229100	3207
Q9NZL9	282	Methionine adenosyltransferase 2 subunit beta	MAT2B	0.999985	7.87E-05	136.01	S	DAFNLPSSHLRPITDSPVLGAQRPRNAQLDC	1318900	623150	6159
Q9H2Y7	640	Zinc finger protein 106	ZNF106	0.977384	0.000321028	136.01	T	KEEDDRILKTSRELSTSPCNPIVRQKESELQ	1756300	0	8094
P07199	156	Major centromere autoantigen B	CENPB	0.999678	4.34E-08	135.98	S	ARARNAAPRTPAAPASPAAVPSEGSGGSTTG	1074400	669320	933
P78347	210	General transcription factor II-I	GTF2I	0.999966	9.92E-05	135.94	S	VGGRVMVTDADRSILSPGGSCGPIKVKTEPT	1335600	1647000	1963
Q71RC2	587	La-related protein 4	LARP4	0.329934	0.000147868	135.94	T	KDGLNQTTIPVSPPSTTKPSRASTASPCNNN	0	0	7786
Q8WXG6	1061	MAP kinase-activating death domain protein	MADD	0.971473	0.000323244	135.94	T	THYYSKEPDKRKRSPTESVNTPVGKDPGLAG	0	448560	7927
Q8IYB3	717	Serine/arginine repetitive matrix protein 1	SRRM1	0.586089	0.000754095	135.91	S	RGASSSPQRRQSPSPSTRPIRRVSRTPEPKK	0	19027000	4151
Q9UQ35	2141	Serine/arginine repetitive matrix protein 2	SRRM2	0.974359	7.95E-05	135.91	S	PLDRCRSPGMLEPLGSSRTPMSVLQQAGGSM	1287400	689210	6606
Q14671	709	Pumilio homolog 1	PUM1	0.999442	0.000327975	135.86	S	TAVANSNTGSGSRRDSLTGSSDLYKRTSSSL	0	988870	2755
Q9H0X4	21	Protein ITFG3	ITFG3	1	0.000619173	135.81	S	DLEAEIHPLKNEERKSQENLGNPSKNEDNVK	733760	552560	5586
Q9H6F5	47	Coiled-coil domain-containing protein 86	CCDC86	0.999347	4.00E-08	135.81	S	LVEFESNPEETREPGSPPSVQRAGLGSPERP	12863000	11127000	5726
Q9P0T7	137	Transmembrane protein 9	TMEM9	1	0.000100355	135.81	S	YTEQLHNEEENEDARSMAAAAASLGGPRANT	678370	526470	6187
Q5VUJ6	333	Leucine-rich repeat and calponin homology domain-containing	LRCH2	0.996009	0.000149842	135.79	S	LPSLSKRMPSQPLTDSMEDFYPNKNHGPDSG	765000	1081900	3349
Q6UN15	496	Pre-mRNA 3'-end-processing factor FIP1	FIP1L1	0.727146	9.18E-05	135.79	S	RTRERERERDHSPTPSVFNSDEERYRYREYA	0	13621000	3587

P78344	508	Eukaryotic translation initiation factor 4 gamma 2	EIF4G2	0.956175	0.00070631	135.72	T	QITMIPPSAQPPRTQTPPLGQTPQLGLKTNP	2090200	1579300	7459
Q9HAU0	937	Pleckstrin homology domain-containing family A member 5	PLEKHA5	0.960318	7.45E-06	135.69	S	KGLNVIGASDQSPLQSPSNLRDNPFRTTQTR	3915600	2619000	5818
Q96JM3	443	Chromosome alignment-maintaining phosphoprotein 1	CHAMP1	0.981438	0.000597374	135.6	S	PEIRSPAGSPELRKPSGSPDLWKLSPDQRKT	4018300	2569400	4974
Q5VT52	614	Regulation of nuclear pre-mRNA domain-containing protein 2	RPRD2	0.999994	0.000175528	135.57	S	SEVSSTSASKASIGQSPGLPSTTFKLPSNSL	1909300	1155100	3328
P61978	284	Heterogeneous nuclear ribonucleoprotein K	HNRNPK	1	0.000292433	135.55	S	GGRPMPPSRRDYDDMSPRRGPPPPPPGRGGR	1810800	4575600	1913
Q6PKG0	75	La-related protein 1	LARP1	1	1.09E-20	135.52	S	CAKPHKEGTGQQERESPRPLQLPGAEGPAIS	16780000	6182000	3556
P30622	200	CAP-Gly domain-containing linker protein 1	CLIP1	0.995972	0.000143824	135.49	S	ISNLTKTASESISNLSEAGSIKKGERELKIG	1359700	765830	1351
P30622	204	CAP-Gly domain-containing linker protein 1	CLIP1	1	0.000143824	135.49	S	TKTASESISNLSEAGSIKKGERELKIGDRVL	1359700	765830	1352
Q9UPP1	880	Histone lysine demethylase PHF8	PHF8	1	0.000147593	135.43	S	KSRPKKKKNSDAPWSPKARVTPTLPKQDRP	1154900	734020	6566
Q9H7S9	217	Zinc finger protein 703	ZNF703	0.461663	1.07E-12	135.37	S	FPPHGAPVSASSSSSSPGGRGGSPHHSDCK	0	0	5775
P49959	689	Double-strand break repair protein MRE11A	MRE11A	0.851626	1.93E-06	135.33	S	IMSQSVSKGVDFESSEDDDDDPFMNTSSLR	3170200	0	1744
Q9UMN6	570	Histone-lysine N-methyltransferase 2B	KMT2B	0.600875	1.35E-12	135.2	S	PKVEVSPVLRPPITTSPPVPQEPAPVPSPPR	0	0	6519
Q9UMN6	582	Histone-lysine N-methyltransferase 2B	KMT2B	1	1.35E-12	135.2	S	ITTSPPVPQEPAPVPSPPRAPTPPSTPVPLP	980360	721800	6520
O75044;P(427;426;(		SLIT-ROBO Rho GTPase-activating protein 2;SLIT-ROBO Rho GTPa	SRGAP2;SRGA	0.991016	0.000151707	135.1	S	CFQYSNSMESVKSTVSETFMSKPSIAKRRAN	1162600	312770	548
P51608	229	Methyl-CpG-binding protein 2	MECP2	0.921776	0.000151707	135.1	S	EKSPGKLLVKMPFQTSPGGKAEGGGATTSTQ	4557000	4139100	1786
Q13428	381	Treacle protein	TCOF1	0.999959	0.000807259	135.09	S	GKTSQVGAASAPAKESPRKGAAPAPPGKTGP	11567000	3008400	2503
Q86X27	329	Ras-specific guanine nucleotide-releasing factor RalGPS2	RALGPS2	1	0.00015915	135.09	S	KSVAAEGALLPQTPPSPRNLIPHGHRKCHSL	4704800	3214100	4005
P49321	503	Nuclear autoantigenic sperm protein	NASP	1	2.54E-08	135.08	S	DKTEEMPNDSVLENKSLQENEEEEIGNLELA	3944100	1545400	1651
Q7Z6Z7	3808	E3 ubiquitin-protein ligase HUWE1	HUWE1	0.999993	9.07E-17	135.08	S	TSESSQSEASVRREESPMDVDQPSPSAQDTQ	4642000	3911900	3873
Q16514	51	Transcription initiation factor TFIID subunit 12	TAF12	1	0.000160935	135.04	S	AVVKIPGTPGAGGRLSPENNQVLTKKKLQDL	18934000	14442000	3013
O94806;Q(	735;742	Serine/threonine-protein kinase D3;Serine/threonine-protein	PRKD3;PRKD	0.999995	0.000184042	135.02	S	DFGFARIIGEKSFRRSVVGTPAYLAPEVLRN;DFGI	542620	137100	696
Q92614	1640	Unconventional myosin-XVIIIa	MYO18A	0.999927	0.000152706	135.02	S	VLREKRELEGKLATLSDQVNRRDFESEKRLR	1152700	792930	4663
Q86VM9	6	Zinc finger CCCH domain-containing protein 18	ZC3H18	1	3.75E-05	134.98	S	__________MDVAESPERDPHSPEDEEPQQ	1151200	2880600	3952
Q8N519	178	Uncharacterized protein C12orf45	C12orf45	0.998801	0.000153215	134.98	S	LPNSEGGKGKIEVLDSPASKKKK________	2764400	3443800	4287
P62263	137	40S ribosomal protein S14	RPS14	0.794169	0.000108921	134.88	S	SGMKIGRIEDVTPIPSDSTRRKGGRRGRRL_	2552800	1304600	1920
Q8NE71	105	ATP-binding cassette sub-family F member 1	ABCF1	1	0.000180189	134.87	S	DDGEEKELMERLKKLSVPTSDEEDEVPAPKP	2597800	3487900	4366
Q7L4I2	27	Arginine/serine-rich coiled-coil protein 2	RSRC2	0.999985	2.75E-07	134.84	S	APEKTSPDRDKKKEQSEVSVSPRASKHHYSR	0	2084900	3729
Q5SW79	937	Centrosomal protein of 170 kDa	CEP170	0.885651	1.49E-08	134.83	T	SYNRDNSISPESDVDTASTISLVTGETERKS	1519400	1772800	7713
P19338	69	Nucleolin	NCL	0.847496	0.00165406	134.81	T	KKAATSAKKVVVSPTKKVAVATPAKKAAVT	754030	0	7306
Q9NQC3	7	Reticulon-4	RTN4	0.999999	2.07E-08	134.8	S	_________MEDLDQSPLVSSSDSPPRPQPA	6203100	4474800	5908
Q9NQC3	11	Reticulon-4	RTN4	0.559707	2.07E-08	134.8	S	_____MEDLDQSPLVSSSDSPPRPQPAFKYQ	661710	0	5909
Q9NV70	471	Exocyst complex component 1	EXOC1	0.979367	0.00121347	134.75	T	ETESLHGSSGKLTGSTSSLNKLSVQSSGNRR	0	0	8168
P78559	2235	Microtubule-associated protein 1A;MAP1A heavy chain;MAP1 li	MAP1A	0.990234	2.46E-12	134.66	S	VTKAPSLDSSLPQLPSPSSPGAPLLSNLPRP	6552500	5286700	1972
Q7Z434	165	Mitochondrial antiviral-signaling protein	MAVS	0.996261	7.40E-17	134.64	S	PESPGENSEQALQTLSPRAIPRNPDGGPLES	3421400	2518000	3806
Q9H0B6	611	Kinesin light chain 2	KLC2	0.68261	0.000167247	134.56	S	PGGTGLSDSRTLSSSSMDLSRRSSLVG____	2366900	0	5565
O94782	66	Ubiquitin carboxyl-terminal hydrolase 1	USP1	0.63332	1.66E-05	134.52	S	EYRASEIDQVVPAAQSSPINCEKRENLLPFV	0	0	684
O15541	80	RING finger protein 113A	RNF113A	0.88312	4.65E-08	134.52	Y	PMIQKTRDSGKQKAAYGDLSSEEEEENEPES	0	0	8346
O76094	621	Signal recognition particle subunit SRP72	SRP72	0.946399	0.000132401	134.49	S	TAGASSELDASKTVSSPPTSPRPGSAATVSA	1082000	1006100	680
O76094	625	Signal recognition particle subunit SRP72	SRP72	0.758534	0.000132401	134.49	S	SSELDASKTVSSPPTSPRPGSAATVSASTSN	0	0	681
Q9Y6D6	1569	Brefeldin A-inhibited guanine nucleotide-exchange protein 1	ARFGEF1	1	0.000168193	134.49	S	PSPVSEKPLDTISQKSVDIHDSIQPRSVDNR	0	1986600	7000
O95817	289	BAG family molecular chaperone regulator 3	BAG3	0.999983	0.000359168	134.4	S	SSREGSPARSSTPLHSPSPIRVHTVVDRPQQ	6859400	3863800	827
Q96ST2	398	Protein IWS1 homolog	IWS1	1	7.51E-05	134.4	S	EGEEEKVAKRKAAVLSDSEDEEKASAKKSRV	1534300	1066900	5125
Q96ST2	400	Protein IWS1 homolog	IWS1	1	7.51E-05	134.4	S	EEEKVAKRKAAVLSDSEDEEKASAKKSRVVS	1534300	1066900	5126
P51608	228	Methyl-CpG-binding protein 2	MECP2	0.744288	0.000654885	134.38	T	LEKSPGKLLVKMPFQTSPGGKAEGGGATTST	0	4139100	7439
Q14684	515	Ribosomal RNA processing protein 1 homolog B	RRP1B	0.850587	0.000691557	134.38	T	SGPSGSHPQGPRGSPTGGAQLLKRKRKLGVV	6861900	0	7614
Q6GYQ0	795	Ral GTPase-activating protein subunit alpha-1	RALGAPA1	0.757039	0.000194226	134.37	S	NEVFGALNEEQPLPRSSSTSDILEPFTVERA	0	0	3464
Q6GYQ0	796	Ral GTPase-activating protein subunit alpha-1	RALGAPA1	0.360453	0.000194226	134.37	S	EVFGALNEEQPLPRSSSTSDILEPFTVERAK	0	0	3465
Q6GYQ0	797	Ral GTPase-activating protein subunit alpha-1	RALGAPA1	0.305296	0.000194226	134.37	S	VFGALNEEQPLPRSSSTSDILEPFTVERAKV	0	0	3466
Q86X27	326	Ras-specific guanine nucleotide-releasing factor RalGPS2	RALGPS2	1	0.000306699	134.33	T	SGRKSVAAEGALLPQTPPSPRNLIPHGHRKC	3142600	2372500	7832
Q9NYV4	383	Cyclin-dependent kinase 12	CDK12	0.996892	0.000266213	134.3	S	SHSKKKRSSSRSRHSSISPVRLPLNSSLGAE	969870	864380	6125
Q9NYV4	385	Cyclin-dependent kinase 12	CDK12	0.999999	0.000266213	134.3	S	SKKKRSSSRSRHSSISPVRLPLNSSLGAELS	969870	864380	6126
Q9BYI3	453	Hyccin	FAM126A	1	1.85E-12	134.28	S	SSHGLAKTAATVFSKSFEQVSGVTVPHNPSS	985210	1161500	5470
Q9Y618	1017	Nuclear receptor corepressor 2	NCOR2	0.738747	4.96E-17	134.26	S	PQNLQPESDAPQQPGSSPRGKSRSPAPPADK	3124400	1264900	6978
Q08J23	743	tRNA (cytosine(34)-C(5))-methyltransferase	NSUN2	1	5.27E-13	134.17	S	PDNDVTEGQRAGEPNSPDAEEANSPDVTAGC	94919000	72286000	2265
O43491	58	Band 4.1-like protein 2	EPB41L2	0.779689	0.000198819	134.08	S	EEKGSQPPPAAESQSSLRRQKREKETSESRG	1852500	1065000	393
P48634	808	Protein PRRC2A	PRRC2A	0.997141	0.00262735	134.05	S	GDVFTATPAEPRPLTSPLRQAADEDDKGMRS	4109600	4329900	1628
Q8IYB3	450	Serine/arginine repetitive matrix protein 1	SRRM1	1	0.0016518	134.04	S	TSGKVTKHKGTEKRESPSPAPKPRKVELSES	3238000	4421500	4154
Q8IYB3	452	Serine/arginine repetitive matrix protein 1	SRRM1	1	0.0016518	134.04	S	GKVTKHKGTEKRESPSPAPKPRKVELSESEE	3238000	3377900	4155
A7MCY6	388	TANK-binding kinase 1-binding protein 1	TBKBP1	0.926588	0.000150947	134.02	S	PVPPCPSPQQRRSPASPSCPSPVPQRRSPVP	868120	730710	49
A7MCY6	393	TANK-binding kinase 1-binding protein 1	TBKBP1	0.999735	0.000150947	134.02	S	PSPQQRRSPASPSCPSPVPQRRSPVPPSCQS	0	896060	51
O00161	20	Synaptosomal-associated protein 23	SNAP23	0.998536	0.000116858	134.02	S	SSEEIQQRAHQITDESLESTRRILGLAIESQ	0	0	94
Q9BYG5	11	Partitioning defective 6 homolog beta	PARD6B	0.999966	0.000181585	134.02	S	_____MNRSHRHGAGSGCLGTMEVKSKFGAE	1619100	466730	5469
Q8WUA4	220	General transcription factor 3C polypeptide 2	GTF3C2	0.999332	0.0014747	133.99	S	ALPAPVSCPEGPKVSSPTKPKKIRQPAACPG	3627000	3751300	4507
Q8IYB3	777	Serine/arginine repetitive matrix protein 1	SRRM1	0.991381	1.39E-08	133.98	S	KKPPAPPSPVQSQSPSTNWSPAVPVKKAKSP	0	110290000	4144
Q8N4S0	131	Coiled-coil domain-containing protein 82	CCDC82	1	0.000174956	133.98	S	IKHRNIDLQDQEKHLSQEDNDLNKQTGQIIE	0	791710	4269
Q9NW Q4	86	G patch domain-containing protein 2-like	GPATCH2L	0.994336	0.000853333	133.98	S	EATKDCREVAPVTNFSDSDDTMVAKRHPALN	529330	1866400	6059
Q9NWQ4	88	G patch domain-containing protein 2-like	GPATCH2L	0.804786	0.000853333	133.98	S	TKDCREVAPVTNFSDSDDTMVAKRHPALNAI	1471600	1277500	6060
Q9NWQ4	91	G patch domain-containing protein 2-like	GPATCH2L	0.801274	0.000853333	133.98	T	CREVAPVTNFSDSDDTMVAKRHPALNAIVKS	1471600	1277500	8173
Q13523	852	Serine/threonine-protein kinase PRP4 homolog	PRPF4B	0.695046	2.16E-06	133.95	S	SASHVADNDITPYLVSRFYRAPEIIIGKSYD	5420600	0	2555
Q8WUB8	56	PHD finger protein 10	PHF10	0.826931	3.68E-08	133.93	S	QPSKRRMGSGDSSRSCETSSQDLGFSYYPA	0	808000	4512
P15056	363	Serine/threonine-protein kinase B-raf	BRAF	0.613869	2.17E-06	133.92	S	PADEDHRNQFGQRDRSSSAPNVHINTIEPVN	0	0	1065
P15056	364	Serine/threonine-protein kinase B-raf	BRAF	0.333331	2.17E-06	133.92	S	ADEDHRNQFGQRDRSSSAPNVHINTIEPVNI	0	0	1066
P15056	365	Serine/threonine-protein kinase B-raf	BRAF	0.333331	2.17E-06	133.92	S	DEDHRNQFGQRDRSSSAPNVHINTIEPVNID	0	0	1067
Q7Z5K2	459	Wings apart-like protein homolog	WAPAL	0.999996	0.00390551	133.92	S	GSSNYKIKYFGFDDLSESEDEDDDCQVERK	0	0	3845
Q7Z5K2	461	Wings apart-like protein homolog	WAPAL	1	0.00390551	133.92	S	SNYKIKYFGFDDLSESEDEDDDCQVERKTS	0	0	3846
P18583	1948	Protein SON	SON	1	0.000273505	133.91	S	TPSRRRRSRSVGRRRSFSISPSRRSRTPSRR	2372000	3863900	1135
P18583	1952	Protein SON	SON	0.999955	0.000273505	133.91	S	RRRSRSVGRRRSFSISPSRRSRTPSRRSRTP	3241800	4410100	1137
Q13188	385	Serine/threonine-protein kinase 3;Serine/threonine-protein k	STK3	0.99913	9.56E-05	133.91	S	EDEEEEDGTMKRNATSPQVQRPSFMDYFDKQ	0	692230	2432
Q8ND56	182	Protein LSM14 homolog A	LSM14A	0.5	9.56E-05	133.91	S	TQDTRSLKTQLSQGRSSPQLDPLRKSPTMEQ	40998000	15993000	4341
Q8ND56	183	Protein LSM14 homolog A	LSM14A	0.814319	9.56E-05	133.91	S	QDTRSLKTQLSQGRSSPQLDPLRKSPTMEQA	40998000	15993000	4342
Q96T58	2101	Msx2-interacting protein	SPEN	1	9.56E-05	133.91	S	AVDKSASLKNVDAAVSPRGAAAQAGERESGV	686010	757170	5176
Q9UGU0	574	Transcription factor 20	TCF20	1	0.00032902	133.91	S	SPAQGAQNEPPRLNASPAAREEATSPGAKDM	954590	834830	6316
Q9NYF3	232	Protein FAM53C	FAM53C	0.999996	0.000876182	133.9	S	SDAESLSPCPPQRRFSLSPSLGPQASRFLPS	829700	532730	6097
Q9NYF3	234	Protein FAM53C	FAM53C	0.985038	0.000876182	133.9	S	AESLSPCPPQRRFSLSPSLGPQASRFLPSAR	829700	532730	6098
Q8NE01	700	Metal transporter CNNM3	CNNM3	1	0.000176247	133.88	S	AAGSSHSRPGVPVEGSPGRNPGV________	6930600	4034300	4364
O43432	495	Eukaryotic translation initiation factor 4 gamma 3	EIF4G3	1	0.000754825	133.87	S	QTEEILDSQNLNSRRSPVPAQIAITVPKTWK	1498600	1177100	388
P46937	63	Transcriptional coactivator YAP1	YAP1	0.5	5.58E-05	133.66	T	PPAGHQIVHVRGDSETDLEALFNAVMNPKTA	1242800	0	7396
Q99956	172	Dual specificity protein phosphatase 9	DUSP9	0.9794	1.95E-17	133.56	S	VPVVGLGSLCLGSDCSDAESEADRDSMSCGL	3147900	2915400	5282
P40425	151	Pre-B-cell leukemia transcription factor 2	PBX2	0.971919	5.56E-13	133.48	S	SAAAAAAAASGGGVSPDNSIEHSDYRSKLA	1233500	1147600	1473
Q8IYB3	694	Serine/arginine repetitive matrix protein 1	SRRM1	0.9639	8.06E-05	133.48	S	NKRHSPSPRPRAPQTSSSPPPVRRGASSSPQ	4222600	3545900	4133
Q8IYB3	695	Serine/arginine repetitive matrix protein 1	SRRM1	0.739698	8.06E-05	133.48	S	KRHSPSPRPRAPQTSSSPPPVRRGASSSPQR	4222600	3545900	4134
Q96L91	1732	E1A-binding protein p400	EP400	0.999999	0.000121899	133.48	S	NALAVGEPGTASKPASPIGGPTQEEKTRLLK	0	748740	5018
O75179	2041	Ankyrin repeat domain-containing protein 17	ANKRD17	0.706619	2.15E-06	133.47	S	NATYPMPTAKEHYPVSSPSSPSPPAQPGGVS	6006000	3801200	581
Q8HWS3	111	DNA-binding protein RFX6	RFX6	0.999996	0.0110504	133.47	T	KTKAADQYLSQKKTITQIVKDKKKQTQLTLQ	0	0	7839
Q14315	2233	Filamin-C	FLNC	0.999934	9.91E-05	133.42	S	FPAVFGDFLGRERLGSFGSITRQQEGEASSQ	3457700	3352200	2734
Q5T200	1208	Zinc finger CCCH domain-containing protein 13	ZC3H13	0.944075	0.00011538	133.42	S	GSNRSNRSHTSGRLRSPSNDSAHRSGDDQSG	681040	472870	3261
Q5T200	1210	Zinc finger CCCH domain-containing protein 13	ZC3H13	0.5	0.00011538	133.42	S	NRSNRSHTSGRLRSPSNDSAHRSGDDQSGRK	681040	472870	3262
Q9Y2W1	210	Thyroid hormone receptor-associated protein 3	THRAP3	0.545526	0.000499148	133.42	T	DNQGDEAKEQTFSGGTSQDTKASESSKPWPD	0	240520	8287
Q9NZN5	1176	Rho guanine nucleotide exchange factor 12	ARHGEF12	1	0.000102687	133.25	S	KLKEEQHGISVTGLQSPDRDLGLESTLISSK	1309400	1011200	6163
Q96TA1	692	Niban-like protein 1	FAM129B	0.572121	1.52E-05	133.24	S	PAGESPQPKAAPEASSPPASPLQHLLPGKAV	0	0	5186
P42356	265	Phosphatidylinositol 4-kinase alpha	PI4KA	0.999927	0.000192975	133.23	S	TLKRKTSSVSSISQVSPERGMPPPSSPGGSA	1252600	475150	1503
O60271	203	C-Jun-amino-terminal kinase-interacting protein 4	SPAG9	1	1.01E-07	133.21	S	LESTAHSRIRKERPISLGIFPLPAGDGLLTP	3494200	2384500	455
Q9Y2W1	323	Thyroid hormone receptor-associated protein 3	THRAP3	0.515061	0.000199353	133.21	S	SLSPSKKSPVGKSPPTSGTYGSSQKEESAA	0	787110	6826
Q5T200	877	Zinc finger CCCH domain-containing protein 13	ZC3H13	0.999438	0.000185831	133.15	S	RLLSQVRPQESRSLSPSHLTEDRQGRWKEE	7177100	5322700	3270
Q9Y485	663	DmX-like protein 1	DMXL1	1	7.60E-09	133.15	T	VLPLLLTTSHHNALRTPDVNPEQPFDALNI	2180600	1370200	8300
Q9UQ35	1460	Serine/arginine repetitive matrix protein 2	SRRM2	0.962318	0.000155553	133.05	S	PGLRDGSGTPSRHSLSGSSPGMKDIPRTPSR	366300	3827000	6611
Q8IYB3	560	Serine/arginine repetitive matrix protein 1	SRRM1	1	0.000725813	132.97	S	RRRRSPSPPPTRRRRSPSPAPPPRRRRTPTP	10270000	5437800	4158
Q8IYB3	562	Serine/arginine repetitive matrix protein 1	SRRM1	1	0.000423945	132.97	S	RRSPSPPPTRRRRSPSPAPPPRRRRTPTPPP	14653000	6593600	4159
Q9H0E9	621	Bromodomain-containing protein 8	BRD8	0.842288	0.000100626	132.97	S	EMSDSLKEESGTIFGSQIKDAPGEDEEEDGV	0	842460	5579
P18583	1782	Protein SON	SON	0.674826	0.000725553	132.87	S	AASPVVSSMPERASESSSEEKDDYEIFVKVK	0	0	1129
P18583	1784	Protein SON	SON	0.866045	0.000725553	132.87	S	SPVVSSMPERASESSSEEKDDYEIFVKVKDT	2578100	0	1131
Q13425	95	Beta-2-syntrophin	SNTB2	1	0.000101374	132.86	S	GDSLPGSPSRGLGPPSPPAPPRGPAGEAGAS	3734700	2643900	2484
Q9NV70	472	Exocyst complex component 1	EXOC1	0.600769	0.000149241	132.86	S	TESLHGSSGKLTGSTSSLNKLSVQSSGNRRS	3183800	0	6029
Q5PRF9	172	Protein Smaug homolog 2	SAMD4B	1	3.62E-12	132.85	S	GFRSRPEPSYHSRQGSDEWGGPAELGPGEAG	2902300	2311100	3204
Q9NQS7	421	Inner centromere protein	INCENP	0.822183	0.000189387	132.81	S	TEIANSTPNPKPAASSPETPSAGQQEAKTDQ	0	401680	5924
P19634	703	Sodium/hydrogen exchanger 1	SLC9A1	1	0.000190538	132.79	S	AHKLDSPTMSRARIGSDPLAYEPKEDLPVIT	990250	588570	1175
Q8IYB3	740	Serine/arginine repetitive matrix protein 1	SRRM1	0.999996	0.000149983	132.79	S	SRTPEPKKIKKAASPSQSVRRVSSSRSVSG	8284700	8860000	4131
O75113	257	NEDD4-binding protein 1	N4BP1	0.481609	1.17E-16	132.77	S	TPVSELTKQMDTVLSSSPDVLFDPINGLTPD	0	0	557
O75113	258	NEDD4-binding protein 1	N4BP1	0.685852	1.17E-16	132.77	S	PVSELTKQMDTVLSSSPDVLFDPINGLTPDE	0	0	558
Q9P1Y6	1202	PHD and RING finger domain-containing protein 1	PHRF1	1	0.00020134	132.65	S	TRSHSPERKGAVREASPAPLAQGEPGREDLP	6200800	5133600	6200
P51956	479	Serine/threonine-protein kinase Nek3	NEK3	1	7.88E-09	132.65	T	ILDPERLEPGLDEEDTDFEEEDDNPDWVSEL	3876000	1972000	7443
Q9ULM3	463	YEATS domain-containing protein 2	YEATS2	0.499921	0.000159277	132.64	S	PGKSFQPITMSCKIVSGSPISTPSPSPLPRT	1172400	0	6495
Q9ULM3	471	YEATS domain-containing protein 2	YEATS2	0.555688	0.000159277	132.64	S	TMSCKIVSGSPISTPSPSPLPRTPTSTPVHV	1172400	0	6497
Q8TEK3	1001	Histone-lysine N-methyltransferase, H3 lysine-79 specific	DOT1L	0.999824	0.000179917	132.62	S	QHPLLLAQPRNSLPASPAHQLSSSPRLGGAA	3009500	1278700	4479
Q8TEK3	1009	Histone-lysine N-methyltransferase, H3 lysine-79 specific	DOT1L	0.985522	0.000179917	132.62	S	PRNSLPASPAHQLSSSPRLGGAAQGPLPEAS	3009500	1278700	4480
Q9NQG6	55	Mitochondrial dynamics protein MID51	MIEF1	1	0.000152315	132.56	S	GIATLAVKRMYDRAISAPTSPTRLSHSGKRS	2777900	2206400	5914
Q9NQG6	59	Mitochondrial dynamics protein MID51	MIEF1	0.905895	0.000152315	132.56	S	LAVKRMYDRAISAPTSPTRLSHSGKRSWEEP	0	2206400	5915
Q9UQ35	1984	Serine/arginine repetitive matrix protein 2	SRRM2	0.993592	0.00138183	132.56	S	RSRSRTSPITRRRSRSRTSPVTRRRSRSRTS	458960	2073700	6731
Q9UQ35	1987	Serine/arginine repetitive matrix protein 2	SRRM2	0.999272	0.00138183	132.56	S	SRTSPITRRRSRSRTSPVTRRRSRSRTSPVT	458960	2073700	6732
P50613	170	Cyclin-dependent kinase 7	CDK7	0.999999	0.000429054	132.56	T	DFGLAKSFGSPNRAYTHQVVTRWYRAPELLF	1542700	607250	7433
O75052	371	Carboxyl-terminal PDZ ligand of neuronal nitric oxide syntha	NOS1AP	0.998359	4.52E-05	132.52	S	VQELELKLSGQNAMGSQDSLLEITFRSGALP	633980	421680	552
O75052	374	Carboxyl-terminal PDZ ligand of neuronal nitric oxide syntha	NOS1AP	0.999853	4.52E-05	132.52	S	LELKLSGQNAMGSQDSLLEITFRSGALPVLC	633980	421680	553
Q9HCD5	9	Nuclear receptor coactivator 5	NCOA5	0.960013	8.86E-05	132.51	S	_______MNTAPSRPSPTRRDPYGFGDSRDS	0	0	5843
Q9UNX4	240	WD repeat-containing protein 3	WDR3	0.499309	3.19E-09	132.51	S	EIEDPEEPDPKKIKGSSPGIQDTLEAEDGAF	0	0	6551
Q9UNX4	241	WD repeat-containing protein 3	WDR3	0.499309	3.19E-09	132.51	S	IEDPEEPDPKKIKGSSPGIQDTLEAEDGAFE	0	0	6552
Q9UQ35	1008	Serine/arginine repetitive matrix protein 2	SRRM2	0.997707	0.0010174	132.51	S	ISPYPKVKAQTPPGPSLSGSKSPCPQEKSKD	802430	2398300	6602
Q5JTV8	147	Torsin-1A-interacting protein 1	TOR1AIP1	0.5	2.95E-05	132.51	T	QQHSEQPPLQPSPVMTRRGLRDSHSSEEDEA	0	2020600	7706
P18583	1783	Protein SON	SON	0.96346	0.000744565	132.5	S	ASPVVSSMPERASESSSEEKDDYEIFVKVKD	0	1278500	1130
Q32MZ4	733	Leucine-rich repeat flightless-interacting protein 1	LRRFIP1	0.566209	2.85E-08	132.48	S	PKSEDADRCTLPEHESPSQDISDACEAESTE	0	3628700	3053

P24723	675	Protein kinase C eta type	PRKCH	0.999979	0.000194714	132.47	S	EGHLPMINQDEFRNFSYVSPELQP_______	1158400	787500	1237
P36507	25	Dual specificity mitogen-activated protein kinase kinase 2	MAP2K2	0.643831	7.55E-17	132.42	T	PALTINPTIAEGPSPTSEGASEANLVDLQKK	450530	7030500	7367
Q96T58	1222	Msx2-interacting protein	SPEN	0.993473	7.58E-06	132.39	S	LVHEVGKPPQDVTDDSPPSKKKRMDHVDFDI	9565200	3682400	5180
Q9NZT2	521	Opioid growth factor receptor	OGFR	0.932771	6.51E-17	132.38	T	GRTGPKEGTPGSPSETPGPSPAGPAGDEPAE	2414900	2718200	8185
O43159	64	Ribosomal RNA-processing protein 8	RRP8	0.999996	5.12E-09	132.36	S	AASLSQHPPSLCISDSEEEEEERKKKCPKKA	16535000	11630000	340
Q5T6F0	15	DDB1- and CUL4-associated factor 12	DCAF12	0.999996	2.88E-08	132.33	S	_MARKVVSRKRKAPASPGAGSDAQGPQFGWD	1214900	1891900	3294
Q8N9M1	280	Uncharacterized protein C19orf47	C19orf47	0.982788	1.21E-16	132.33	S	RLGATPETDEDLAWDSDNDSSSSVLQYAGVL	2526600	2148800	4302
O14545	327	TRAF-type zinc finger domain-containing protein 1	TRAFD1	0.983369	0.000132568	132.32	S	CNPSRALPSLNTGSSSPRGVEEPDVIFQNFL	27006000	12909000	151
Q92618	118	Zinc finger protein 516	ZNF516	0.568808	0.000540509	132.32	T	GEMRASEGLDACASPTKSASACNRLLNGASQ	0	414880	7941
Q96S55	151	ATPase WRNIP1	WRNIP1	1	0.000988795	132.25	S	RKGSGKRPAAAAAAGSASPRSWDEAEAQEEE	1870700	763760	5109
P13051	63	Uracil-DNA glycosylase	UNG	0.55366	6.54E-07	132.24	S	AKKAPAGQEEPGTPPSSPLSAEQLDRIQRNK	1481600	803870	1046
P12694	337	2-oxoisovalerate dehydrogenase subunit alpha, mitochondrial	BCKDHA	0.798141	0.000102926	132.23	S	QPFLIEAMTYRIGHHSTSDDSSAYRSVDEVN	0	794520	1043
P53667	310	LIM domain kinase 1	LIMK1	0.972479	0.000207354	132.23	S	RSCSIDRSPGAGSLGSPASQRKDLGRSESLR	1022800	677080	1836
Q5VWN6	2009	Protein FAM208B	FAM208B	0.940939	0.000227818	132.23	S	DSQHSASSANVFPKESPTQISIGAFPSTKIS	720820	0	3360
P12694	338	2-oxoisovalerate dehydrogenase subunit alpha, mitochondrial	BCKDHA	0.762817	0.000102926	132.23	T	PFLIEAMTYRIGHHSTSDDSSAYRSVDEVNY	0	0	7281
P18583	1950	Protein SON	SON	1	0.00134482	132.17	S	SRRRRSRSVGRRRSFSISPSRRSRTPSRRSR	3241800	4410100	1136
P52594	370	Arf-GAP domain and FG repeat-containing protein 1	AGFG1	0.31689	2.91E-05	132.14	S	APVGSVVSVPSQSSASSDKYAALAELDSVFS	0	0	1813
P52594	371	Arf-GAP domain and FG repeat-containing protein 1	AGFG1	0.31689	2.91E-05	132.14	S	PVGSVVSVPSQSSASSDKYAALAELDSVFSS	0	0	1814
Q01082	2102	Spectrin beta chain, non-erythrocytic 1	SPTBN1	0.999744	0.000773975	132.14	S	VRRQQEEEERKRRPPSPEPSTKVSEEAESQQ	0	1016200	2048
Q9UQ35	1729	Serine/arginine repetitive matrix protein 2	SRRM2	0.740604	0.000106539	132.14	S	PETRSRTPPRHRRSPSVSSPEPAEKSRSSRR	0	0	6701
Q9H3P2	277	Negative elongation factor A	NELFA	1	0.0198697	132.14	T	LDMVGAGREAKRRRKTLDAEVVEKPAKEETV	0	0	8096
P62995;Q	264;260	Transformer-2 protein homolog beta;Transformer-2 protein hom	TRA2B;TRA2	1	0.000519975	132.06	S	GWRAAQDRDQIYRRRSPSPYYSRGGYRSRSR;GYD	24188000	9191100	1931
P62995;Q	266;262	Transformer-2 protein homolog beta;Transformer-2 protein hom	TRA2B;TRA2	0.999944	0.000519975	132.06	S	DRYEDYDYRYRRRSPSPYYSRYRSRSRSRSY;RAA	1789300	581070	1932
Q15057	521	Arf-GAP with coiled-coil, ANK repeat and PH domain-containi	ACAP2	0.95677	0.000107144	132.06	S	KYVERKFVDKYSISLSPPEQQKKFVSKSSEE	1346300	699790	2870
Q9Y2K5	349	R3H domain-containing protein 2	R3HDM2	0.933284	0.000107144	132.06	S	DGSVRSMRPPVTKASSFSGISILTRGDSIGS	476790	244910	6788
Q9BRD0	163	BUD13 homolog	BUD13	1	0.000189911	132.03	S	PDPSPRRARHDTPDPSPLRGARHDSDTSPPR	774970	793630	5320
Q16513	360	Serine/threonine-protein kinase N2	PKN2	0.946024	0.000232043	131.96	S	VPGRSKATSVALPGWSPSETRSSFMSRTSKS	1280900	812690	3012
Q9UNF1	162	Melanoma-associated antigen D2	MAGED2	0.909544	5.91E-17	131.95	S	QAPADEPEPESAAAQSQENQDTRPKVKAKKA	0	839230	6543
Q8IZ21	464	Phosphatase and actin regulator 4	PHACTR4	1	0.000192424	131.83	S	SDSFSEDSSTLGRTRSLPITIEMLKVPDDEE	2282900	1106700	4180
Q96MU7	308	YTH domain-containing protein 1	YTHDC1	1	0.000110506	131.83	S	GSDEKKKERKRARGISPIVFDRSGSSASESY	15252000	9851500	5023
Q13523	366	Serine/threonine-protein kinase PRP4 homolog	PRPF4B	1	0.000426078	131.79	S	KRRSLSPKPRDKSRRSRSPLLNDRRSKQSKS	17919000	15160000	2556
Q13523	368	Serine/threonine-protein kinase PRP4 homolog	PRPF4B	1	0.000426078	131.79	S	RSLSPKPRDKSRRSRSPLLNDRRSKQSKSPS	17919000	15160000	2557
Q96JH8	393	Ras-associating and dilute domain-containing protein	RADIL	0.999871	0.000109045	131.79	S	SCRLCGAALGARGAASPTQAALPRRQQLLLE	629450	471730	4964
Q9UBB9	59	Tuftelin-interacting protein 11	TFIP11	1	0.000491479	131.79	S	QTKEEATYGVWAERDSDDERPSFGGKRARDY	6209300	2802600	6257
Q04726	263	Transducin-like enhancer protein 3	TLE3	1	0.000178448	131.72	S	LVVDVSNEDPATPRVSPAHSPPENGLDKARS	5399000	5640100	2174
Q04726	267	Transducin-like enhancer protein 3	TLE3	1	0.000178448	131.72	S	VSNEDPATPRVSPAHSPPENGLDKARSLKKD	5399000	5640100	2175
Q96BD5	455	PHD finger protein 21A	PHF21A	0.99699	4.49E-12	131.59	S	FGALTPTSPQSSHPDSPENEKTETTFTFPAP	2489900	1351000	4806
Q9Y2X7	592	ARF GTPase-activating protein GIT1	GIT1	0.973751	3.03E-08	131.59	S	SQEGSRHTSKLSRHGSGADSDYENTQSGDPL	3213700	751490	6836
Q96BD5	446	PHD finger protein 21A	PHF21A	0.826459	4.49E-12	131.59	T	PPKYNAVLGFGALTPTSPQSSHPDSPENEKT	2489900	1351000	7970
Q14157	471	Ubiquitin-associated protein 2-like	UBAP2L	0.691114	1.76E-08	131.56	S	LPSKSTSAPQMSPGSSDNQSSSPQPAQQKLK	1042600	0	2671
Q15648	1156	Mediator of RNA polymerase II transcription subunit 1	MED1	0.980452	0.000382554	131.52	S	SQSKNSSQSGGKPGSSPITKHGLSSGSSSTK	0	450120	2965
O75494	133	Serine/arginine-rich splicing factor 10	SRSF10	1	0.00299012	131.5	S	RSRSRSYERRRSRSRSFDYNYRRSYSPRNSR	14597000	7673100	632
Q96F45	237	Zinc finger protein 503	ZNF503	0.990441	4.52E-12	131.5	S	FTPRTGSPSSSASACSPGGMLSSAGGAPEGK	3454200	3019600	4900
Q96F45	226	Zinc finger protein 503	ZNF503	0.724943	4.52E-12	131.5	T	GFRVPSATCQPFTPRTGSPSSSASACSPGGM	0	3019600	7980
P46821	1254	Microtubule-associated protein 1B;MAP1B heavy chain;MAP1 li	MAP1B	0.781183	0.000568734	131.48	S	IKDSISAVSSEKVSPSKSPSLSPSPPSPLEK	1598400	981660	1612
Q9UQ35	1231	Serine/arginine repetitive matrix protein 2	SRRM2	0.742613	9.16E-06	131.48	T	AGSSPETKEQNSALPTSSQDEELMEVVEKSE	395890	1414900	8256
Q9NWH9	551	SAFB-like transcription modulator	SLTM	0.965901	0.000900583	131.45	S	TSESIKKSEEKKRISSKSPGHMVILDQTKGD	2294300	939070	6057
A8CG34	325	Nuclear envelope pore membrane protein POM 121C	POM121C	0.774825	5.90E-09	131.33	S	LDGQENKRRRHDSSGSGHSAFEPLVASGVPA	0	2182000	56
A8CG34	328	Nuclear envelope pore membrane protein POM 121C	POM121C	0.846053	5.90E-09	131.33	S	QENKRRRHDSSGSGHSAFEPLVASGVPASFV	2816200	2182000	57
P37275	679	Zinc finger E-box-binding homeobox 1	ZEB1	0.999634	1.79E-08	131.33	S	SANANEPQDSTVNLQSPLKMTNSPVLPVGST	6030700	4360800	1440
P35659	15	Protein DEK	DEK	0.782409	3.53E-12	131.33	T	_MSASAPAAEGEGTPTQPASEKEPEMPGPRE	0	0	7366
P11274	462	Breakpoint cluster region protein	BCR	0.614974	6.87E-06	131.31	S	RRHQDGLPYIDDSPSSSPHLSSKGRGSRDAL	3247200	2493700	1012
Q15233	450	Non-POU domain-containing octamer-binding protein	NONO	1	4.09E-06	131.31	T	FGQAATMEGIGAIGGTPPAFNRAAPGAEFAP	914390	1133300	7633
Q9H7N4	238	Splicing factor, arginine/serine-rich 19	SCAF1	0.499547	8.96E-08	131.31	Y	PRFDIYDPFHPTDEAYSPPPAPEQKYDPFEP	0	0	8425
Q6ZRS2	2790	Helicase SRCAP	SRCAP	0.748624	8.95E-08	131.3	S	STLVPGVSETSASPGSPSVRSMSGPESSPPI	1042900	768550	3661
P51532	613	Transcription activator BRG1	SMARCA4	0.966708	6.50E-13	131.27	S	AIGPDGEPLDETSQMSDLPVKVIHVESGKIL	6647600	7609200	1782
P67809	176	Nuclease-sensitive element-binding protein 1	YBX1	0.987436	1.86E-12	131.26	S	NYQNSESGEKNEGSESAPEGQAQQRRPYRRR	2240400	1034900	1940
Q00613	323	Heat shock factor protein 1	HSF1	0.974956	5.47E-12	131.26	T	PRVEEASPGRPSSVDTLLSPTALIDSILRES	0	0	7471
Q13263	541	Transcription intermediary factor 1-beta	TRIM28	0.999906	1.02E-12	131.26	T	AAAAATGQPGTAPAGTPGAPPLAGMAIVKEE	2357200	3250500	7529
Q5VSY0	23	G kinase-anchoring protein 1	GKAP1	0.477587	8.97E-05	131.25	S	SVPTTASRFALLQVDSGSGSDSEPGKGKGRN	0	0	3322
P02545	404	Prelamin-A/C;Lamin-A/C	LMNA	0.368399	0.000238046	131.18	S	LSPSPTSQRSRGRASSHSSQTQGGGSVTKKR	0	0	859
Q86YS7	260	C2 domain-containing protein 5	C2CD5	0.998503	0.000238046	131.18	S	LDKLSSPAAFLPACNSPSKEMKEIPFNEDPN	4082400	3687500	4030
P24928	1915	DNA-directed RNA polymerase II subunit RPB1	POLR2A	0.832639	0.00109345	131.18	T	PVYTPTSPKYSPTSPTYSPTSPKYSPTSPTY	1145200	0	7320
P51610	2019	Host cell factor 1;HCF N-terminal chain 1;HCF N-terminal ch	HCFC1	0.836746	0.000474597	131.12	S	SKDSSGTKPANKRPMSSPEMKSAPKKSKADG	0	1493600	1790
P52948	670	Nuclear pore complex protein Nup98-Nup96;Nuclear pore compl	NUP98	1	0.000464466	131.12	T	VSHFYTNPIAKPIPQTPESAGNKHSNSNSVD	3671500	3913100	7445
Q9Y2D5	746	A-kinase anchor protein 2	AKAP2	0.680499	0.000820588	131.12	T	EREEELKRQRQVLQSTQSPRTKNAPSLPSRT	0	369340	8281
Q14980	1862	Nuclear mitotic apparatus protein 1	NUMA1	0.999049	9.10E-05	131.11	S	SLRATSSTQSLARLGSPDYGNSALLSLPGYR	173970	202800	2828
O94808	244	Glutamine--fructose-6-phosphate aminotransferase [isomerizi	GFPT2	0.998408	0.000143961	131.08	S	ENVKNICKTRMKRLDSSACLHAVGDKAVEFF	1510500	878220	698
P27824	583	Calnexin	CANX	1	0.000514515	131.08	S	EDRKPKAEEDEILNRSPRNRKPRRE______	15396000	9199800	1299
Q12769	1157	Nuclear pore complex protein Nup160	NUP160	0.999962	1.24E-08	131.06	S	IVQPVSGAVYDRPGASPKRNHDGECTAAPTN	2682300	902550	2293
P04075	37	Fructose-bisphosphate aldolase A	ALDOA	0.525102	0.00091998	130.95	T	RIVAPGKGILAADESTGSIAKRLQSIGTENT	1307100	0	7256
Q14202	51	Zinc finger MYM-type protein 3	ZMYM3	0.727912	1.84E-08	130.89	S	ESQTAPTRGWAPPGPSPSSGALDLLDTPAGL	1013000	843640	2711
Q5F1R6	370	DnaJ homolog subfamily C member 21	DNAJC21	1	1.17E-06	130.89	S	NFSRPQIDENPLDDNSEEEMEDAPKQKLSKK	661400	1331900	3159
O94864	108	STAGA complex 65 subunit gamma	SUPT7L	0.999963	5.67E-09	130.83	S	GVKTEESEPLPSCPGSPPLPDDLLPLDCKNP	3528600	1944100	708
P49327	976	Fatty acid synthase;[Acyl-carrier-protein] S-acetyltransfer	FASN	0.880004	2.90E-08	130.83	T	WDDPDPRLFDHPESPTPNPTEPLFLAQAEVY	0	54544	7412
Q96E09	35	Protein FAM122A	FAM122A	0.751549	1.86E-08	130.75	S	PAEGGGSGGGGGLRRSNSAPLIHGLSDTSPV	0	4653500	4871
Q96E09	37	Protein FAM122A	FAM122A	0.586086	1.86E-08	130.75	S	EGGGSGGGGGLRRSNSAPLIHGLSDTSPVFQ	1438300	558250	4872
Q99460	315	26S proteasome non-ATPase regulatory subunit 1	PSMD1	0.999999	0.00190463	130.73	S	EEKTSSAFVGKTPEASPEPKDQTLKMIKILS	1965300	1029800	5196
Q99460	311	26S proteasome non-ATPase regulatory subunit 1	PSMD1	0.999934	0.00190463	130.73	T	SMETEEKTSSAFVGKTPEASPEPKDQTLKMI	1965300	1029800	8021
Q8TDY2	647	RB1-inducible coiled-coil protein 1	RB1CC1	0.820942	0.000251963	130.69	S	ITDLLSEQKASVSQTSPQSASSPRMESTAGI	812860	571820	4471
Q9UQ35	2581	Serine/arginine repetitive matrix protein 2	SRRM2	0.999949	0.000688314	130.69	S	SSLPVQPEVALKRVPSPTPAPKEAVREGRPP	7541000	6956400	6679
O75179	2401	Ankyrin repeat domain-containing protein 17	ANKRD17	0.999998	0.000252394	130.66	S	NDSSAQSVSSGVRAPSPAPSSVPLGSEKPSN	1834100	1423800	580
Q14498	146	RNA-binding protein 39	RBM39	0.942073	0.000759013	130.64	T	FRKDKSPVREPIDNLTPEERDARTVFCMQLA	0	0	7596
O95677	361	Eyes absent homolog 4	EYA4	1	0.000148294	130.61	S	SSGSKSRGRGRKNNPSPPPDSDLERVFVWDL	1485000	2430900	793
Q13428	1111	Treacle protein	TCOF1	0.928734	3.30E-12	130.6	S	PSSGVDSAVGTLPATSPQSTSVQAKGTNKLR	9697600	8857500	2526
Q96T23	1364	Remodeling and spacing factor 1	RSF1	0.737403	2.93E-06	130.57	S	EDFENVGKVGSPLDYSLVDLPSTNGQSPGKA	0	2266800	5153
O14654	872	Insulin receptor substrate 4	IRS4	0.955948	0.000544271	130.47	S	KPSGEGSFSKPGDGGSPSKPSDHEPPKNKAK	0	4760800	174
O15027	569	Protein transport protein Sec16A	SEC16A	0.852825	6.73E-06	130.47	S	PEDEASGSFFKQIDSSPVGGETDETTVSQNY	1715200	705720	249
P49790	522	Nuclear pore complex protein Nup153	NUP153	0.894071	0.000255301	130.47	S	ALTNKVQMTSPSSTGSPMFKFSSPIVKSTEA	1682400	1493700	1699
Q8TEK3	1104	Histone-lysine N-methyltransferase, H3 lysine-79 specific	DOT1L	0.999862	0.00025537	130.47	S	RKRASAGTPSLSAGVSPKRRALPSVAGLFTQ	1683100	1019600	4477
Q8TEK3	834	Histone-lysine N-methyltransferase, H3 lysine-79 specific	DOT1L	1	0.000149627	130.47	S	VPGSMKLSPQDPRPLSPGALQLAGEKSSEKG	1970500	1109400	4481
P51532	1452	Transcription activator BRG1	SMARCA4	1	0.000209731	130.44	S	SKKQKKRGRPPAEKLSPNPPNLTKKMKKIVD	7623700	4765200	1783
Q9UQE7	1086	Structural maintenance of chromosomes protein 3	SMC3	0.834442	1.10E-05	130.38	S	GEGSGESERGSGSQSSVPSVDQFTGVGIRVS	965440	0	6760
Q15910	487	Histone-lysine N-methyltransferase EZH2	EZH2	1	0.000573897	130.27	T	KESSIIAPAPAEDVDTPPRKKKRKHRLWAAH	1701400	1278800	7652
Q8ND82	540	Zinc finger protein 280C	ZNF280C	0.994928	3.84E-12	130.27	T	TAPFGCAPGTSFLQVTPPTSQNTTARNPRKS	1693600	2635700	7889
P08559;P	295;293	Pyruvate dehydrogenase E1 component subunit alpha, somatic	PDHA1;PDHA	0.987588	0.000117699	130.15	S	ILMELQTYRYHGHSMSDPGVSYRTREEIQEV	0	2810600	956
P30414	1146	NK-tumor recognition protein;Putative peptidyl-prolyl cis-t	NKTR	0.988058	0.000225196	130.15	S	ICTPDRSSPAKVEETSPLGNARLDTPDINIV	411590	962570	1344
Q13136	1172	Liprin-alpha-1	PPFIA1	0.525114	0.000152519	130.15	S	AETLPANFRVTSSMSSPSMQPKKMQMDGNVS	0	0	2421
Q8IYB3	389	Serine/arginine repetitive matrix protein 1	SRRM1	1	0.000666979	130.15	S	PPKRTSSPPRKTRRLSPSASPPRRRHRPSPP	13962000	19549000	4146
Q8IYB3	391	Serine/arginine repetitive matrix protein 1	SRRM1	1	0.000666979	130.15	S	KRTSSPPRKTRRLSPSASPPRRRHRPSPPAT	26538000	22733000	4147
Q92522	31	Histone H1x	H1FX	0.999543	0.00035846	130.15	S	GMAKKVTKAGGSAALSPSKKRKNSKKKNQPG	10766000	11783000	4617
Q86WC4	333	Osteopetrosis-associated transmembrane protein 1	OSTM1	1	0.000153037	130.1	S	KRLKSSTSFANIQENSN______________	0	659280	3990
Q08AE8	464	Protein spire homolog 1	SPIRE1	1	4.70E-05	130.09	S	QRKKLLRAPTLAELDSSESEEETLHKSTSSS	944300	696150	2261
Q08AE8	465	Protein spire homolog 1	SPIRE1	0.999996	4.70E-05	130.09	S	RKKLLRAPTLAELDSSESEEETLHKSTSSSS	944300	696150	2262
Q08AE8	467	Protein spire homolog 1	SPIRE1	0.99986	4.70E-05	130.09	S	KLLRAPTLAELDSSESEEETLHKSTSSSSVS	944300	696150	2263
O00178	24	GTP-binding protein 1	GTPBP1	0.63602	4.81E-08	130.01	S	AMDSPVPASMFAPEPSSPGAARAAAAAARLH	969180	652730	102
Q5TKA1	309	Protein lin-9 homolog	LIN9	0.9979	3.35E-08	130.01	S	FFMTPPRLHYTPPLQSPIIDNDPLLGQSPWR	1026800	663420	3310
Q6QNY0	32	Biogenesis of lysosome-related organelles complex 1 subunit	BLOC1S3	0.76009	0.000233304	130.01	S	VVPGEATETDSERSASSSEEEELYLGPSGPT	1340100	482100	3574
Q6QNY0	33	Biogenesis of lysosome-related organelles complex 1 subunit	BLOC1S3	0.683502	0.000233304	130.01	S	VPGEATETDSERSASSSEEEELYLGPSGPTR	1340100	482100	3575
Q6QNY0	34	Biogenesis of lysosome-related organelles complex 1 subunit	BLOC1S3	0.975626	0.000233304	130.01	S	PGEATETDSERSASSSEEEELYLGPSGPTRG	1340100	482100	3576
P51114	411	Fragile X mental retardation syndrome-related protein 1	FXR1	0.708901	5.26E-08	130.01	T	TSGYGTNSELSNPSETESERKDELSDWSLAG	0	857150	7436
Q9NQG6	58	Mitochondrial dynamics protein MID51	MIEF1	0.876457	0.000876899	130.01	T	TLAVKRMYDRAISAPTSPTRLSHSGKRSWEE	2099200	1702900	8144
Q14155;Q	518;488	Rho guanine nucleotide exchange factor 7;Rho guanine nucleo	ARHGEF7;AR	1	0.000124334	129.89	S	LFPNVLLMLSASPRMSGFIYQGKLPTTGMTI;LFS	8167200	10184000	2658
Q9NZH5;O	165;165	Securin-2;Securin	PTTG2;PTTG	1	0.000229176	129.85	S	DEEGELEKLFQLGPPSPVKMPSPPWECNLLQ	3147400	2227600	841
Q9Y5B6	557	PAX3- and PAX7-binding protein 1	PAXBP1	0.999468	6.48E-09	129.84	S	AREQTGKMADHLEGLSSDDEETSTDITNFNL	5246300	1665500	6957
Q9Y5B6	558	PAX3- and PAX7-binding protein 1	PAXBP1	0.999403	6.48E-09	129.84	S	REQTGKMADHLEGLSSDDEETSTDITNFNLE	5246300	1665500	6958
Q86XP3	185	ATP-dependent RNA helicase DDX42	DDX42	0.92897	1.12E-16	129.82	S	AGVVQEEEEDNLEYDSDGNPIAPTKKIIDPL	0	2533700	4018
P29590	530	Protein PML	PML	0.999998	6.04E-09	129.8	S	KAVSPPHLDGPPSPRSPVIGSEVFLPNSNHV	3887900	2136800	1323
O14497	382	AT-rich interactive domain-containing protein 1A	ARID1A	0.99523	0.000156043	129.77	S	SGGGGQPLARTPQPSSPMDQMGKMRPQPYGG	1859800	1868200	147
Q13144	544	Translation initiation factor eIF-2B subunit epsilon	EIF2B5	1	0.000218117	129.76	S	SEQSMDSEEPDSRGGSPQMDDIKVFQNEVLG	4654300	4303900	2423
Q5T8P6	127	RNA-binding protein 26	RBM26	1	0.000230357	129.76	S	KEEEREKKFSRRLNHSPPQSSSRYRENRSRD	3929300	1392900	3296
Q4VCS5	328	Angiomotin	AMOT	0.487617	3.76E-12	129.66	S	SLTSGGSLPLLQSPPSTRLSPARHPLVPNQG	0	0	3119
Q6ZRV2	914	Protein FAM83H	FAM83H	1	0.000958029	129.54	S	EQKGSPTSAYPERRGSPVPPVPERRSSPVPP	793880	787650	3666
Q9H6R4	289	Nucleolar protein 6	NOL6	0.999928	0.000264567	129.43	S	VRSAWYRGQSPAGDGSPEPPTPRYNTWVLQD	521920	337260	5736
Q9BST9	220	Rhotekin	RTKN	0.961092	0.00328661	129.42	S	ALTGGPKRLATKLSSSLGRSSGRRVRASLDS	0	489280	5347
Q8TE77	16	Protein phosphatase Slingshot homolog 3	SSH3	0.425342	1.17E-05	129.36	S	MALVTVSRSPPGSGASTPVGPWDQAVQRRSR	0	0	4475
Q9HCE3	314	Zinc finger protein 532	ZNF532	0.999992	0.000126654	129.34	S	PLPKEVNDSPRAADKSPESQNLIDGTKKPSL	2445300	2473800	5856
Q8IVP5	13	FUN14 domain-containing protein 1	FUNDC1	0.964784	3.49E-08	129.32	S	___MATRNPPPQDYESDDDSYEVLDLTEYAR	0	0	4047
Q9P2M7	134	Cingulin	CGN	0.997594	7.38E-05	129.32	S	GAYWNGKLLRSHSQASLAGPGPVDPSNRSNS	2654300	3029500	6246
O00291	320	Huntingtin-interacting protein 1	HIP1	0.881607	1.65E-12	129.29	S	SEHISPVVVIPAEASSPDSEPVLEKDDLMDM	1191600	1099400	116
P33991	131	DNA replication licensing factor MCM4	MCM4	1	6.25E-09	129.23	S	PDLGSAQKGLQVDLQSDGAAAEDIVASEQSL	610290	378000	1366
Q13541	37	Eukaryotic translation initiation factor 4E-binding protein	EIF4EBP1	0.944315	2.07E-14	129.22	T	VLGDGVQLPPGDYSTTPGGTLFSTTPGGTRI	34317000	32432000	7553
Q13541	46	Eukaryotic translation initiation factor 4E-binding protein	EIF4EBP1	0.961267	2.07E-14	129.22	T	PGDYSTTPGGTLFSTTPGGTRIIYDRKFLME	34317000	19866000	7556
Q03164	3508	Histone-lysine N-methyltransferase 2A;MLL cleavage product	KMT2A	0.894109	1.96E-08	129.2	S	MGLEQNKALSSAVQASPTSPGGSPSSPSSGQ	4471300	3755400	2130
O43303	372	Centriolar coiled-coil protein of 110 kDa	CCP110	0.682821	0.000198331	129.17	S	LTGSYAKLPSPEPSMSPKMHRRRSRTSSACH	0	1177100	357
Q8N9B5	115	Junction-mediating and -regulatory protein	JMY	1	0.000238587	129.17	S	RSPGPRRSSAWAEGGSPRSTRSLLGDPRLRS	791110	485110	4301
Q68DK7	362	Male-specific lethal 1 homolog	MSL1	1	9.71E-05	129.05	S	LAPEFSKVKTKTPKHSPIKEEPCGSLSETVC	962940	0	3421
Q96D09	510	G-protein coupled receptor-associated sorting protein 2	GPRASP2	0.442441	0.000153442	129.05	S	TVEFKPGLFHGVGFRSTSPFGIPEEASEMLE	0	0	4837
Q96D09	512	G-protein coupled receptor-associated sorting protein 2	GPRASP2	0.333333	0.000153442	129.05	S	EFKPGLFHGVGFRSTSPFGIPEEASEMLEAK	0	0	4838

Q96D09	511	G-protein coupled receptor-associated sorting protein 2	GPRASP2	0.442441	0.000153442	129.05	T	VEFKPGLFHGVGFRSTSPFGIPEEASEMLEA	0	0	7974
Q03164	3510	Histone-lysine N-methyltransferase 2A;MLL cleavage product N	KMT2A	0.583274	3.05E-08	129.02	T	LEQNKALSSAVQASPTSPGGSPSSPSSGQRS	4063600	0	7483
Q96JM3	204	Chromosome alignment-maintaining phosphoprotein 1	CHAMP1	1	5.69E-05	128.98	S	KSVPVCESQKLAPVPSPEPQKPAPVSPESVK	62886000	37150000	4981
Q96JM3	214	Chromosome alignment-maintaining phosphoprotein 1	CHAMP1	0.999528	5.69E-05	128.98	S	LAPVPSPEPQKPAPVSPESVKATLSNPKPQK	5502700	3191900	4982
Q6EEV6;P(28;28;27		Small ubiquitin-related modifier 4;Small ubiquitin-related m	SUMO4;SUMO2	1	0.000134062	128.96	S	ENNNHINLKVAGQDGSVVQFKIKRQTPLSKL	519740	389170	1884
Q6IN85	771	Serine/threonine-protein phosphatase 4 regulatory subunit 3/	SMEK1	0.984435	1.39E-13	128.92	S	LTSQSSTTNLPGSPGSPGSPGSPGSPGSPVK	6293400	5723000	3478
Q6IN85	777	Serine/threonine-protein phosphatase 4 regulatory subunit 3/	SMEK1	0.997841	1.39E-13	128.92	S	TTNLPGSPGSPGSPGSPGSPGSVPKNTSQTA	16697000	8083400	3480
P40925	333	Malate dehydrogenase, cytoplasmic	MDH1	0.954384	0.000235091	128.91	S	KELTEEKESAFEFLSSA______________	112310	247080	1480
Q5VT52	485	Regulation of nuclear pre-mRNA domain-containing protein 2	RPRD2	0.795895	0.000249416	128.9	S	TGVSPASRPSPGTPTSPSNLTSGLKTPAPAT	0	1381300	3334
Q99459	303	Cell division cycle 5-like protein	CDC5L	1	7.79E-05	128.9	S	FTKKRSKLVLPAPQISDAELQEVVKVGQASE	843070	397070	5195
Q9NYA4	628	Myotubularin-related protein 4	MTMR4	0.333333	0.00122446	128.9	S	DLLSACDTSSPLTRTSSDPNLNNHCQEVRVG	0	0	6093
Q9NYA4	629	Myotubularin-related protein 4	MTMR4	0.902689	0.00122446	128.9	S	LLSACDTSSPLTRTSSDPNLNNHCQEVRVGL	0	0	6094
Q9NYA4	627	Myotubularin-related protein 4	MTMR4	0.333333	0.00122446	128.9	T	DDLLSACDTSSPLTRTSSDPNLNNHCQEVRV	0	0	8174
Q9C0J8	7	pre-mRNA 3' end processing protein WDR33	WDR33	1	6.34E-05	128.88	S	_________MATEIGSPPRFFHMPRFQHQAP	2846100	1643900	5548
O43491	87	Band 4.1-like protein 2	EPB41L2	0.966168	0.000452326	128.86	S	RGISRFIPPWLKKQKSYTLVVAKDGGDKKEP	0	1211900	395
O14523	660	C2 domain-containing protein 2-like	C2CD2L	0.997862	1.49E-05	128.85	S	KLIFRRRPRQKEAGLSQSHDDLSNATATPSV	3521300	3288600	150
Q92597	332	Protein NDRG1	NDRG1	0.767586	0.000233786	128.85	S	SASMTRLMRSRTASGSSVTSLDGTRSRSHTS	1663600	613990	4650
O75436	315	Vacuolar protein sorting-associated protein 26A	VPS26A	0.996353	0.000305949	128.82	S	EKLRKQRTNFHQRFESPESQASAEQPEM___	0	1893800	626
Q9P260	183	LisH domain and HEAT repeat-containing protein KIAA1468	KIAA1468	0.997556	0.00139278	128.82	T	TASGGGQLNRAGSISTLDSLDFARYSDDGNR	0	942490	8190
Q14152	882	Eukaryotic translation initiation factor 3 subunit A	EIF3A	0.990385	0.000454644	128.81	S	ERERRREEERRLGDSSLSRKDSRWGDRDSEG	0	447820	2652
P07814	880	Bifunctional glutamate/proline--tRNA ligase;Glutamate--tRNA	EPRS	0.736707	5.88E-05	128.74	S	KEKTGKEYIPGQPPLSQSSDSSPTRNSEPAG	10144000	0	935
Q15154	861	Pericentriolar material 1 protein	PCM1	0.764553	0.00031189	128.74	S	LMAEHQRRQGLAETASPVAVSLRSDGSENLC	591870	750280	2884
Q5H9R7	853	Serine/threonine-protein phosphatase 6 regulatory subunit 3	PPP6R3	0.944388	0.0013046	128.74	S	ETAEAKCAAPRPPSSSPEQRTGQPSAPGDTS	0	960210	3163
Q99569	337	Plakophilin-4	PKP4	0.984451	0.00031189	128.74	S	QTRVASPSQGQVGSSSPKRSGMTAVPQHLGP	2207300	0	5214
Q9H6T3	480	RNA polymerase II-associated protein 3	RPAP3	0.684016	0.00031189	128.74	S	LANVIAATGTTSKKNSSQDDLFPTSDTPRAK	795120	0	5740
Q96BY7	239	Autophagy-related protein 2 homolog B	ATG2B	0.5	0.000312856	128.73	S	SGVSLFWDEFSASAKSSPVCSTAPVETEPKL	971450	867990	4809
Q96BY7	240	Autophagy-related protein 2 homolog B	ATG2B	0.5	0.000312856	128.73	S	GVSLFWDEFSASAKSSPVCSTAPVETEPKLS	971450	867990	4810
Q9UQ35	952	Serine/arginine repetitive matrix protein 2	SRRM2	1	0.000205563	128.73	S	SPSRVTSRTTPRRSRSVPCSNVESRLLPRY	0	3887900	6742
Q9UQ35	954	Serine/arginine repetitive matrix protein 2	SRRM2	0.999982	0.000205563	128.73	S	SRVTSRTTPRRSRSVPCSNVESRLLPRYSH	10196000	9658000	6743
A6NKD9	246	Coiled-coil domain-containing protein 85C	CCDC85C	1	7.00E-05	128.68	S	GPHKAPDGKAGATRRSLDDLSAPPHHRSIPN	1745200	605590	39
Q14669	310	E3 ubiquitin-protein ligase TRIP12	TRIP12	0.69821	0.000140997	128.68	S	LQAKLASLRKSTKKRSESPPAELPSLRRSTR	0	0	2753
Q6NZY4	597	Zinc finger CCHC domain-containing protein 8	ZCCHC8	0.492082	3.54E-05	128.66	S	EVPEIFTKKSEAGHASSPDSEVTSLCQKEKA	0	0	3498
Q9P1Y5	350	Calmodulin-regulated spectrin-associated protein 3	CAMSAP3	0.588077	1.50E-05	128.66	S	PRGTEASPPQNNSGSSSPVFTFRHPLLSSGG	0	0	6194
Q9UQ35	508	Serine/arginine repetitive matrix protein 2	SRRM2	1	0.0130933	128.6	S	GRSRSRTPTKRGHSRSRSPQWRRSRSAQRWG	11563000	9700800	6709
Q9UQ35	510	Serine/arginine repetitive matrix protein 2	SRRM2	1	0.0130933	128.6	S	SRSRTPTKRGHSRSRSPQWRRSRSAQRWGRS	11563000	9700800	6710
Q13263	823	Transcription intermediary factor 1-beta	TRIM28	0.499986	5.11E-13	128.52	S	VLVEPPPMSLPGAGLSSQELSGGPGDGP___	0	12600000	2450
Q13263	824	Transcription intermediary factor 1-beta	TRIM28	0.986568	5.11E-13	128.52	S	LVEPPPMSLPGAGLSSQELSGGPGDGP____	3597200	12600000	2451
Q9H4G0	430	Band 4.1-like protein 1	EPB41L1	1	0.000169411	128.51	S	PFFERSSSKRYTMSRSLDGAEFSRPASVSEN	909550	420300	5672
Q9ULU4	432	Protein kinase C-binding protein 1	ZMYND8	0.988114	1.26E-05	128.51	S	SGGTGRRISLSDMPRSPMSTNSSVHTGSDVE	548610	483010	6505
Q9ULU4	444	Protein kinase C-binding protein 1	ZMYND8	0.983307	1.26E-05	128.51	S	MPRSPMSTNSSVHTGSDVEQDAEKKATSSHF	548610	483010	6506
P16989	369	Y-box-binding protein 3	YBX3	0.999237	6.08E-05	128.44	S	AGEAPTENPAPPTQQSSAE____________	0	633810	1098
P21333	1084	Filamin-A	FLNA	0.999715	0.000311268	128.44	S	KVKAFGPGLQGGSAGSPARFTIDTKGAGTGG	2260100	2224100	1195
Q8WVM7	20	Cohesin subunit SA-1	STAG1	0.961755	4.11E-13	128.4	S	ELPVLQDSTNETTAHSDAGSELEETEVKGKR	0	3395200	4541
Q8WVM7	24	Cohesin subunit SA-1	STAG1	0.961755	4.11E-13	128.4	S	LQDSTNETTAHSDAGSELEETEVKGKRKRGR	0	3395200	4542
Q15424;Q!234;233		Scaffold attachment factor B1;Scaffold attachment factor B2	SAFB;SAFB2	0.614274	9.05E-09	128.38	S	DILGETCKSEPVKEESSELEQPFAQDTSSVG	0	0	2920
P85037	245	Forkhead box protein K1	FOXK1	0.767059	6.85E-08	128.36	T	PEPDLRSMVSPVPSPTGTISVPNSCPASPRG	0	0	7467
Q8IYB3	420	Serine/arginine repetitive matrix protein 1	SRRM1	0.994564	0.00067044	128.35	S	ATPPPKTRHSPTPQQSNRTRKSRVSVSPGRT	933840	747210	4139
Q8IYB3	393	Serine/arginine repetitive matrix protein 1	SRRM1	1	0.000666979	128.35	S	TSSPPRKTRRLSPSASPPRRRHRPSPPATPP	28830000	23704000	4148
Q8NCD3	642	Holliday junction recognition protein	HJURP	0.999255	0.000178664	128.35	S	LNPDPHFQGFQKLPSSPLGCRKSLLGSTAIE	686340	720290	4326
Q92614	140	Unconventional myosin-XVIIIa	MYO18A	0.976787	0.0141214	128.35	S	GSLAKQNSQMIVKRFSFSQRSRDESASETST	0	0	4664
Q96PM5	257	RING finger and CHY zinc finger domain-containing protein 1	RCHY1	1	0.0141214	128.35	S	ICESYNTAQAGGRRISLDQQ___________	0	0	5054
Q9H6U6	869	Breast carcinoma-amplified sequence 3	BCAS3	0.991186	0.000254673	128.35	S	TEEGLRERLADAMAESPSRDVVGSGTELQRE	1001600	505590	5741
Q06587	232	E3 ubiquitin-protein ligase RING1	RING1	0.999992	7.07E-13	128.25	S	GGGTGGVGGGAGSEDSGDRGGTLGGGTLGPP	0	0	2205
Q06265	306	Exosome complex component RRP45	EXOSC9	0.901323	0.000257458	128.21	S	QRITAFKMEKAPIDTSDVEEKAEEIIAEAEP	0	1939400	2199
Q8TBN0	168	Guanine nucleotide exchange factor for Rab-3A	RAB3IL1	0.9795	0.000210302	128.21	S	EVTALKTLVITSTPASPNRELHPQLLSPTKA	0	0	4454
Q3YEC7	470	Rab-like protein 6	RABL6	0.989517	1.65E-11	128.17	S	PPLPAGPVPSQDITLSSEEEAEVAAPTKGPA	21411000	16236000	3083
Q3YEC7	471	Rab-like protein 6	RABL6	0.989517	1.65E-11	128.17	S	PLPAGPVPSQDITLSSEEEAEVAAPTKGPAP	21411000	16236000	3084
P56270	300	Myc-associated zinc finger protein	MAZ	0.863894	0.000176356	128.1	S	GKAFRDVYHLNRHKLSHSDEKPYQCPVCQQR	0	645810	1893
Q5JSH3	5	WD repeat-containing protein 44	WDR44	0.73115	3.14E-11	128.09	S	___________MASEDTEEFYDAPEDVHLG	3842400	2411000	3180
Q676U5	287	Autophagy-related protein 16-1	ATG16L1	0.993442	1.25E-05	128.04	S	AGGLLDSITNIFGRRSVSSFPVPQDNVDTHP	2875500	1107400	3415
Q5UIP0	1161	Telomere-associated protein RIF1	RIF1	0.709343	1.28E-08	128.04	T	EKSSLSNNECGSLDKTSPEMSNSNNDERKKA	1372200	618240	7728
Q96ET8;Q6;6		Golgi apparatus membrane protein TVP23 homolog C;Golgi appar	TVP23C;TVP2	0.981367	7.49E-11	128.01	S	__________MLQQDSNDDTEDVSLFDAEEE	5336300	4675400	4889
Q12888	1316	Tumor suppressor p53-binding protein 1	TP53BP1	0.464314	0.000731121	127.88	S	DISSFSSKASSLHRTSSGTSLSAMHSSGSSG	0	0	2369
Q12888	1315	Tumor suppressor p53-binding protein 1	TP53BP1	0.464314	0.000731121	127.88	T	GDISSFSSKASSLHRTSSGTSLSAMHSSGSS	0	0	7515
O76094	624	Signal recognition particle subunit SRP72	SRP72	0.769976	0.00344057	127.87	T	ASSELDASKTVSSPPTSPRPGSAATVSASTS	1082000	1006100	7226
Q8WWH5	11	Probable tRNA pseudouridine synthase 1	TRUB1	0.916496	0.000153937	127.83	S	_____MAASEAAVVSSPSLKTDTSPVLETAG	1188000	0	4552
Q9UQ35	2071	Serine/arginine repetitive matrix protein 2	SRRM2	1	0.00237115	127.79	S	SRTPRTARGKRSLTRSPPAIRRRSASGSSSD	16750000	12912000	6699
Q9UQ35	2069	Serine/arginine repetitive matrix protein 2	SRRM2	1	0.00237115	127.79	T	SRSRTPRTARGKRSLTRSPPAIRRRSASGSS	16750000	12912000	8268
Q9Y4E8	229	Ubiquitin carboxyl-terminal hydrolase 15	USP15	1	0.000162709	127.78	S	KNEDGTWPRGPSTPKSPGASNFSTLPKISPS	6434700	5114300	6910
Q8WUM0	44	Nuclear pore complex protein Nup133	NUP133	0.564336	4.20E-05	127.75	S	PRTASRKGLPLGSAVSSPVLFSPVGRRSSLS	640920	473940	4518
Q7Z309	58	Protein FAM122B	FAM122B	1	0.000164222	127.71	S	TLRTRRNSTTIMSRHSLEEGLDMVNRETAHE	0	0	3770
Q6UUV9	151	CREB-regulated transcription coactivator 1	CRTC1	0.492805	3.50E-11	127.67	S	MYLSPPADTSWRRTNSDSALHQSTMTPTQPE	0	0	3593
Q6UUV9	149	CREB-regulated transcription coactivator 1	CRTC1	0.492805	3.50E-11	127.67	T	GTMYLSPPADTSWRRTNSDSALHQSTMTPTQ	0	0	7772
Q92597	335	Protein NDRG1	NDRG1	0.673419	0.00291403	127.66	T	MTRLMRSRTASGSSVTSLDGTRSRSHTSEGT	0	0	7935
P10071	669	Transcriptional activator GLI3;Transcriptional repressor GLI	GLI3	0.868617	5.57E-06	127.58	T	RDSGSHSQSRSPGRPTQGALGEQQDLSNTTS	0	3441000	7270
Q92934	99	Bcl2-associated agonist of cell death	BAD	1	0.000270383	127.56	S	EGMGEEPSPFRGRSRSAPPNLWAAQRYGREL	6720600	2954900	4734
P27816	928	Microtubule-associated protein 4	MAP4	0.968473	0.000271151	127.52	S	PSSTTPRLSRLATNTSAPDLKNVRSKVGSTE	1544400	657690	1294
Q14684	513	Ribosomal RNA processing protein 1 homolog B	RRP1B	0.997674	0.000271151	127.52	S	SQSGPSGSHPQGPRGSPTGGAQLLKRKRKLG	0	5106300	2782
Q9Y5B0	740	RNA polymerase II subunit A C-terminal domain phosphatase	CTDP1	1	0.000278895	127.51	S	LFPLRDDHTKAQRENSPAAFPDREGVPPTAL	1053900	800690	6956
P26358	127	DNA (cytosine-5)-methyltransferase 1	DNMT1	1	0.00182825	127.49	S	QARSEARRVGMADANSPPKPLSKPRTPRRSK	2404200	1139300	1276
Q14126	680	Desmoglein-2	DSG2	1	0.000300994	127.49	S	EDKVVPSFLPVDQGGSLVGRNGVGGMAKEAT	685890	621110	2647
Q49A88	376	Coiled-coil domain-containing protein 14	CCDC14	0.908416	0.00178039	127.49	T	HGKETHLDSQTHRSPTQSQPAFLATNEEKCA	0	0	7674
P46100	675	Transcriptional regulator ATRX	ATRX	0.998798	7.46E-06	127.4	S	KTTPLRRPTETNPVTSNSDEECNETVKEKQK	1352300	1448100	1557
Q9NYI0	1011	PH and SEC7 domain-containing protein 3	PSD3	0.988885	0.00237719	127.37	S	LSNDESEAAGLKKSHSSPSLNPDTSPITAKV	542740	0	6119
Q5JSH3	158	WD repeat-containing protein 44	WDR44	0.371336	4.73E-06	127.37	T	SDETCEKPVDETTKLTQTSSTEQLNVLETET	0	0	7702
Q5JSH3	160	WD repeat-containing protein 44	WDR44	0.371336	4.73E-06	127.37	T	ETCEKPVDETTKLTQTSSTEQLNVLETETEV	0	0	7703
Q5JSH3	163	WD repeat-containing protein 44	WDR44	0.41911	4.73E-06	127.37	T	EKPVDETTKLTQTSSTEQLNVLETETEVLNK	0	0	7704
Q9NYI0	1019	PH and SEC7 domain-containing protein 3	PSD3	0.71268	0.00237719	127.37	T	AGLKKSHSSPSLNPDTSPITAKVKRNVSERK	542740	0	8177
Q13136	668	Liprin-alpha-1	PPFIA1	0.999846	0.000274394	127.36	S	TEQRAEEIESRVGSGSLDNLGRFRSMSSIPP	1510800	1313100	2420
O15027	568	Protein transport protein Sec16A	SEC16A	0.499999	1.61E-05	127.32	S	KPEDEASGSFFKQIDSSPVGGETDETTVSQN	0	705720	248
Q96T88	287	E3 ubiquitin-protein ligase UHRF1	UHRF1	1	0.000236063	127.32	S	IFVDEVFKIERPGEGSPMVDNPMRRKSGPSC	2925900	1207600	5184
Q16513	582	Serine/threonine-protein kinase N2	PKN2	0.834032	0.000403523	127.3	S	DFDLEPEPPPAPPRASSLGEIDESSELRVLD	2102700	0	3010
Q5T4S7	619	E3 ubiquitin-protein ligase UBR4	UBR4	0.952695	0.000152987	127.3	S	EKAAPPPPPPPPLESSPRVKSPSKQAPGEK	4072300	2234700	3284
Q5T4S7	620	E3 ubiquitin-protein ligase UBR4	UBR4	0.5	0.000152987	127.3	S	KAAPPPPPPPPLESSPRVKSPSKQAPGEKG	4072300	2234700	3285
Q92945	100	Far upstream element-binding protein 2	KHSRP	0.997534	5.14E-06	127.3	T	AAKIGGDAATTVNNSTPDFGFGGQKRQLEDG	145490	653550	7954
Q9C0E8	177	Protein lunapark	LNP	0.977521	2.53E-08	127.28	S	PGQEIRQRTAAQRNLSPTPASPNQGPPPQVP	4229000	597500	5543
Q9C0E8	182	Protein lunapark	LNP	0.999177	2.53E-08	127.28	S	RQRTAAQRNLSPTPASPNQGPPPQVPVSPGP	2363300	3492100	5544
Q9C0E8	194	Protein lunapark	LNP	1	2.53E-08	127.28	S	TPASPNQGPPPQVPVSPGPPKDSSAPGGPPE	2363300	1599500	5545
O75376	1472	Nuclear receptor corepressor 1	NCOR1	1	0.000411391	127.2	S	SVLRSTLHEAPKAQLSPGIYDDTSARRTPVS	922690	614440	592
Q6ZSR9	93	Uncharacterized protein FLJ45252		1	0.000194314	127.2	S	PGKLGGAVPFAPPEVSPEQAKTVQGGRKNQF	1025800	1260500	3671
Q96T58	736	Msx2-interacting protein	SPEN	1	0.00196532	127.2	S	PIERSQSPVHLRRPQSPGASPSQAERLPSDS	1785600	1549600	5177
P48378	28	DNA-binding protein RFX2	RFX2	1	0.000413254	127.18	S	SVALRPSAAAPPVPASPQRVLVQAASSNPKG	0	374940	1621
A3KN83	689	Protein strawberry notch homolog 1	SBNO1	0.92836	5.73E-08	127.17	S	RKKLYSLLGIDLTAPSNNSSPRDSPCKENKI	0	115430	27
A3KN83	692	Protein strawberry notch homolog 1	SBNO1	0.875347	5.73E-08	127.17	S	LYSLLGIDLTAPSNNSSPRDSPCKENKIKKR	794460	434810	28
A3KN83	693	Protein strawberry notch homolog 1	SBNO1	0.875347	5.73E-08	127.17	S	YSLLGIDLTAPSNNSSPRDSPCKENKIKKRK	2778000	434810	29
O43290	448	U4/U6.U5 tri-snRNP-associated protein 1	SART1	1	7.35E-06	127.11	S	GDFGSRLRGRGRRRVSEVEEEKEPVPQPLPS	6109600	3979400	350
Q96FS4	884	Signal-induced proliferation-associated protein 1	SIPA1	0.848201	1.07E-08	127.1	S	PSADSETPLTQDRPGSPSGSEDKGNPAPELR	12618000	6921400	4906
P46821	1262	Microtubule-associated protein 1B;MAP1B heavy chain;MAP1 lig	MAP1B	0.681276	0.000350425	127.07	S	SSEKVSPSKSPSLSPSPPSPLEKTPLGERSV	0	2538700	1600
O00257	434	E3 SUMO-protein ligase CBX4	CBX4	0.815572	0.000387612	127.05	S	GEREPCKKRLTARSISTPTCLGGSPAAERPA	1508400	484740	110
Q12770	851	Sterol regulatory element-binding protein cleavage-activatin	SCAP	1	0.000267924	127.02	S	RLSDGGKAGPEEPGDSPPLRHRPRGPPPPSL	688000	675950	2294
P04920	144	Anion exchange protein 2	SLC4A2	0.726996	8.53E-09	126.96	S	DEASEAEGARALTQPSPVSTPSSVQFFLQED	0	0	888
Q8IYB3	773	Serine/arginine repetitive matrix protein 1	SRRM1	0.999971	7.37E-08	126.96	S	EPAAKKPPAPPSPVQSQSPSTNWSPAVPVKK	11208000	9395700	4142
Q8IYB3	781	Serine/arginine repetitive matrix protein 1	SRRM1	0.999682	5.15E-08	126.96	S	APPSPVQSQSPSTNWSPAVPVKKAKSPTPSP	248290000	280290	4145
Q9H8M2	588	Bromodomain-containing protein 9	BRD9	0.99985	1.51E-10	126.93	S	GEQPDVTHDPYEFLQSPEPAASAKT______	3319700	1788600	5791
Q9NPI6	531	mRNA-decapping enzyme 1A	DCP1A	0.998453	0.00307242	126.91	T	DLERKASSPSPLTIGTPESQRKPSIILSKSQ	887430	625310	8137
Q9NWZ5	539	Uridine-cytidine kinase-like 1	UCKL1	1	2.69E-05	126.77	S	NFGDRYFGTDAVPDGSDEEEVAYTG______	1955300	1071200	6074
Q92560	395	Ubiquitin carboxyl-terminal hydrolase BAP1	BAP1	0.810065	1.78E-12	126.75	S	GVGRSRVPVRPPQQYSDDEDDYEDDEEDDVQ	0	1528100	4640
P21359	2543	Neurofibromin;Neurofibromin truncated	NF1	1	0.000304252	126.71	S	QPSQANTKKLLGTRKSFDHLISDTKAPKRQE	0	0	1197
Q6GYQ0	861	Ral GTPase-activating protein subunit alpha-1	RALGAPA1	0.993192	0.000288154	126.66	S	RLRSGNASTMTRRGSSPGSLEIPKDLPDILN	897310	585100	3460
P36915	51	Guanine nucleotide-binding protein-like 1	GNL1	0.97561	0.000332712	126.63	S	SRSGSRERREEQTDTSDGESVTHHIRRLNQQ	5961400	4280500	1432
P12270	2048	Nucleoprotein TPR	TPR	0.718805	2.85E-08	126.62	S	DSQNSGEGNTGAAESSFSQEVSREQQPSSAS	1079500	0	1037
Q6GYQ0	778	Ral GTPase-activating protein subunit alpha-1	RALGAPA1	0.333333	4.90E-08	126.61	T	LPRSTRVRHFSQSEETGNEVFGALNEEQPLP	0	0	7749
Q2M2I8	623	AP2-associated protein kinase 1	AAK1	0.762483	0.00226602	126.5	S	PPAVQGQKVGSLTPPSSPKTQRAGHRRILSD	9081200	0	3041
Q2M2I8	620	AP2-associated protein kinase 1	AAK1	0.999546	0.00226602	126.5	T	TTPPPAVQGQKVGSLTPPSSPKTQRAGHRRI	9081200	8921400	7664
Q9BW85	322	Coiled-coil domain-containing protein 94	CCDC94	0.958866	3.91E-11	126.45	S	LSQLGAYLDSDDSNSGN______________	9872300	8613700	5424
Q99741	45	Cell division control protein 6 homolog	CDC6	0.995722	0.000472811	126.42	S	SDAKLEPTNVQTVTCSPRVKALPLSPRKRLG	2336900	2616700	5272
Q14978	607	Nucleolar and coiled-body phosphoprotein 1	NOLC1	1	0.000712018	126.34	T	AKEAETPQAKKIKLQTPNTFPKRKKGEKRAS	14749000	7152300	7627
Q14978	610	Nucleolar and coiled-body phosphoprotein 1	NOLC1	1	0.000712018	126.34	T	AETPQAKKIKLQTPNTFPKRKKGEKRASSPF	14749000	7152300	7628
Q5T1M5	1100	FK506-binding protein 15	FKBP15	0.989292	9.56E-11	126.32	S	PDGPLQESSTRLSLTSDPEEGDPLALGPESP	3137500	2105900	3246
Q9BXW9	592	Fanconi anemia group D2 protein	FANCD2	0.999759	0.00031672	126.31	S	GAVTMAGIMAADRSESPSLTQERANLSDEQC	0	515180	5463
P55210	16	Caspase-7;Caspase-7 subunit p20;Caspase-7 subunit p11	CASP7	0.994098	1.75E-10	126.29	S	MADDQGCIEEQGVEDSANEDSVDAKPDRSSF	320850	1052300	1882
P78362	380	SRSF protein kinase 2;SRSF protein kinase 2 N-terminal;SRSF	SRPK2	0.993159	8.42E-06	126.29	S	DVDQELANIDPTWIESPKTNGHIENGPFSLE	942030	822890	1965
Q92908	533	Transcription factor GATA-6	GATA6	0.297734	1.92E-12	126.28	T	STSSNSDDCSKNTSPTTQPTASGAGAPVMTG	0	0	7950
Q92908	534	Transcription factor GATA-6	GATA6	0.297734	1.92E-12	126.28	T	TSSNSDDCSKNTSPTTQPTASGAGAPVMTGA	0	0	7951
Q8N302	7	Angiogenic factor with G patch and FHA domains 1	AGGF1	0.999975	2.72E-08	126.26	S	_________MASEAPSPPRSPPPPTSPEPEL	1963000	1502600	4245
Q8N302	11	Angiogenic factor with G patch and FHA domains 1	AGGF1	0.999991	2.72E-08	126.26	S	_____MASEAPSPPRSPPPPTSPEPELAQLR	1963000	1502600	4246
Q8NEZ4	1493	Histone-lysine N-methyltransferase 2C	KMT2C	1	0.000229909	126.22	S	QSSRPLSEEQLDGILSPELDKMVTDGAILGK	330720	235880	4385
Q9UK61	1103	Protein FAM208A	FAM208A	0.986871	0.00151207	126.16	S	IIIKASAKGGNLPPVSPNDSGAKIASNPLER	0	1294400	6424
Q5SW79	497	Centrosomal protein of 170 kDa	CEP170	0.677154	2.61E-10	126.1	S	QATSATSEKDNDDDQSDKGTYTIELENPNSE	0	3590500	3215

P20020	1155	Plasma membrane calcium-transporting ATPase 1	ATP2B1	1	7.43E-08	126.09	S	SIHNFMTHPEFRIEDSEPHIPLIDDTDAEDD	3391400	2700400	1177
P36578	295	60S ribosomal protein L4	RPL4	1	0.00138989	126.03	S	PMHKMINTDLSRILKSPEIQRALRAPRKKIH	3339800	1978500	1429
Q5T4S7	2718	E3 ubiquitin-protein ligase UBR4	UBR4	0.74839	0.000308378	126.03	S	RVLRPRNKRRHVTLPSSPRSNTPMGDKDDDD	6344100	3504000	3286
Q969J3	75	Loss of heterozygosity 12 chromosomal region 1 protein	LOH12CR1	0.655841	0.000315727	126.03	S	PTFQPLLKGLLSGQTSPTNAKLEKLDSQQVL	1444400	1186100	4746
Q96DR7	80	Rho guanine nucleotide exchange factor 26	ARHGEF26	1	0.000308378	126.03	S	PAQVPTASDSRTVHRSPLLLGAQRRAVANGG	1057800	496440	4864
Q96B36	246	Proline-rich AKT1 substrate 1	AKT1S1	0.998967	0.00241663	126.03	T	EDTQVFGDLPRPRLNTSDFQKLKRKY_____	8977800	3069300	7969
Q13427	356	Peptidyl-prolyl cis-trans isomerase G	PPIG	1	0.00463268	125.97	S	YRTPSRSRSRDRFRRSETPPHWRQEMQRAQR	14114000	7544500	2500
Q53LP3	213	Ankyrin repeat domain-containing protein SOWAHC	SOWAHC	0.978251	0.000301763	125.97	S	QRPARQNLRDLVMGSSPQLKRSVCPGGSSPG	1310500	0	3143
Q13427	358	Peptidyl-prolyl cis-trans isomerase G	PPIG	1	0.00463268	125.97	T	TPSRSRSRDRFRRSETPPHWRQEMQRAQRMR	15290000	8088600	7533
Q99613;B	178;178	Eukaryotic translation initiation factor 3 subunit C;Eukary	EIF3C;EIF3	1	1.72E-06	125.83	S	PEQSADEDAEKNEEDSEGSSDEDEDEDGVSA	0	0	70
B2RTY4	1299	Unconventional myosin-IXa	MYO9A	0.61211	0.000304744	125.82	S	IFSLELLKVRSLGGISPSEDRRWSTELVPEG	0	506890	62
P20340	2	Ras-related protein Rab-6A	RAB6A	0.5	0.00012979	125.82	S	______________MSTGGDFGNPLRKFKLV	936350	896600	1185
Q04727	208	Transducin-like enhancer protein 4	TLE4	0.982915	0.000304744	125.82	S	NDHQRDRDSIKSSSVSPSASFRGAEKHRNSA	5323900	2780300	2181
Q96JM3	382	Chromosome alignment-maintaining phosphoprotein 1	CHAMP1	1	0.000276242	125.82	S	SASWKSSSVSPSSWKSPPASPESWKSGPPEL	3019900	1883700	4994
Q96JM3	386	Chromosome alignment-maintaining phosphoprotein 1	CHAMP1	0.999502	0.000276242	125.82	S	KSSSVSPSSWKSPPASPESWKSGPPELRKTA	2068700	1883700	4995
P20340	3	Ras-related protein Rab-6A	RAB6A	0.5	0.00012979	125.82	T	_____________MSTGGDFGNPLRKFKLVF	936350	896600	7309
Q08170;Q	112;116;	Serine/arginine-rich splicing factor 4;Serine/arginine-rich	SRSF4;SRSF	0.875878	0.000324524	125.74	S	APPVRTENRLIVENLSSRVSWQDLKDFMRQA;GPP	0	0	2242
Q9BX63	1237	Fanconi anemia group J protein	BRIP1	0.99993	1.14E-09	125.74	S	ELGKTHEIEIKNFKPSPSKNKGMFPGFK___	1549400	811540	5443
O43237	405	Cytoplasmic dynein 1 light intermediate chain 2	DYNC1LI2	0.47113	0.000460183	125.73	S	GSPRTQGRGGPASVPSSSPGTSVKKPDPNIK	0	0	347
O43237	406	Cytoplasmic dynein 1 light intermediate chain 2	DYNC1LI2	0.622977	0.000460183	125.73	S	SPRTQGRGGPASVPSSSPGTSVKKPDPNIKN	0	1937000	348
Q8IVT5	406	Kinase suppressor of Ras 1	KSR1	0.983309	0.000527735	125.73	S	CRISFLPLTRLRRTESVPSDINNPVDRAAEP	1285700	528270	4048
Q96JM3	427	Chromosome alignment-maintaining phosphoprotein 1	CHAMP1	1	0.000236473	125.73	S	VPPVSPELRKPGPPLSPEIRSPAGSPELRKP	705990	674900	4972
Q9UQ35	2044	Serine/arginine repetitive matrix protein 2	SRRM2	1	0.00233888	125.72	S	RSRSRTPLLPRKRSRSRSPLAIRRRSRSRTP	108730000	75789000	6707
Q9UQ35	2046	Serine/arginine repetitive matrix protein 2	SRRM2	1	0.00233888	125.72	S	RSRTPLLPRKRSRSRSPLAIRRRSRSRTPRT	109310000	76038000	6708
Q96Q42	465	Alsin	ALS2	0.916041	0.000608218	125.71	S	REEQVKQESMQGKKSSSLVDIREEETEGGSR	0	351740	5070
Q03112	1039	MDS1 and EVI1 complex locus protein EVI1	MECOM	0.968029	0.00053211	125.67	S	TEIRNFIGNSNHGSQSPRNVEERMNGSHFKD	1005100	594030	2129
Q14839	1553	Chromodomain-helicase-DNA-binding protein 4	CHD4	0.999958	6.21E-08	125.59	T	PKTPTPSTPGDTQPNTPAPVPPAEDGIKIEE	4439800	3774300	7624
O75122	529	CLIP-associating protein 2	CLASP2	0.99989	0.000335409	125.54	S	ARSSRIPRPSVSQGCSREASRESSRDTSPVR	6348400	3937800	560
Q8NAF0	483	Zinc finger protein 579	ZNF579	0.984338	7.86E-08	125.51	S	ERAAALQALQAQAPTSPPPPPPPLKAEQEEE	3024400	2136700	4309
Q96T23	1345	Remodeling and spacing factor 1	RSF1	1	0.000545692	125.5	S	LPSEQESTKKPYRIESDEEEDFENVGKVGSP	785080	46237	5147
Q8NG27	277	E3 ubiquitin-protein ligase Praja-1	PJA1	1	0.000291831	125.5	T	NLASPNCVKPKIFFDTDDDDDMPHSTSRWRD	0	1263700	7896
Q08170	460	Serine/arginine-rich splicing factor 4	SRSF4	0.999998	0.00207375	125.47	S	RSNSKSKPNLPSESRSRSKSASKTRSRSKSR	0	2575100	2246
P01891	352	HLA class I histocompatibility antigen, A-68 alpha chain	HLA-A	0.98017	5.93E-08	125.45	S	SDRKGGSYSQAASSDSAQGSDVSLTACKV__	0	0	856
Q5VUA4	173	Zinc finger protein 318	ZNF318	1	9.89E-06	125.39	S	CVSTPERRRLSDRLGSPVDNLEDMDRDDLTD	966430	863460	3343
Q6P2E9	33	Enhancer of mRNA-decapping protein 4	EDC4	0.999926	0.00249946	125.39	S	DILKLDRPAGGPSAESPRPSSAYNGDLNGLL	424450	375610	3521
Q6ZN18	18	Zinc finger protein AEBP2	AEBP2	1	3.47E-10	125.39	S	AAITDMADLEELSRLSPLPPGSPGSAARGRA	3149900	4038800	3649
Q6ZN18	24	Zinc finger protein AEBP2	AEBP2	0.998181	3.47E-10	125.39	S	ADLEELSRLSPLPPGSPGSAARGRAEPPEEE	3149900	4038800	3650
Q8N108	122	Mesoderm induction early response protein 1	MIER1	0.801553	2.76E-12	125.39	S	SGCSGENKEENIKDSSGQEDETQSSNDDPSQ	3562100	0	4200
O43237	407	Cytoplasmic dynein 1 light intermediate chain 2	DYNC1LI2	0.454703	0.000480563	125.36	S	PRTQGRGGPASVPSSSPGTSVKKPDPNIKNN	0	0	349
P49450	17	Histone H3-like centromeric protein A	CENPA	0.99992	0.00166249	125.36	S	GPRRRSRKPEAPRRRSPSPTPTPGPSRRGPS	2621300	1707100	1659
P49450	19	Histone H3-like centromeric protein A	CENPA	0.971219	0.00166249	125.36	S	RRRSRKPEAPRRRSPSPTPTPGPSRRGPSLG	1055800	1048600	1660
Q8IX90	155	Spindle and kinetochore-associated protein 3	SKA3	1	4.34E-06	125.36	S	DPPVASSCISEKSPRSPQLSDFGLERYIVSQ	3983400	2648500	4097
A8CG34	323	Nuclear envelope pore membrane protein POM 121C	POM121C	0.546748	1.09E-08	125.35	S	IFLDGQENKRRRHDSSGSGHSAFEPLVASGV	2816200	2182000	55
P42166	158	Lamina-associated polypeptide 2, isoform alpha;Thymopoietin;	TMPO	0.746979	0.000400613	125.33	S	EKKLLKLREQGTESRSSTPLPTISSSAENTR	1723000	1429100	1493
P42166	159	Lamina-associated polypeptide 2, isoform alpha;Thymopoietin;	TMPO	0.747567	0.000400613	125.33	S	KKLLKLREQGTESRSSTPLPTISSSAENTRQ	1723000	1429100	1494
P42166	160	Lamina-associated polypeptide 2, isoform alpha;Thymopoietin;	TMPO	0.75946	0.000400613	125.33	T	KLLKLREQGTESRSSTPLPTISSSAENTRQN	1723000	1429100	7377
Q96EU6	73	Ribosomal RNA processing protein 36 homolog	RRP36	1	0.000348101	125.31	S	SQVGTKTYKQLVAGNSPKKQASRPPIQNACV	2160600	1471900	4890
Q9BST9	219	Rhotekin	RTKN	0.886871	0.00386886	125.31	S	GALTGGPKRLATKLSSSLGRSSGRRVRASLD	0	0	5346
Q5T4S7	2719	E3 ubiquitin-protein ligase UBR4	UBR4	0.499998	0.000353053	125.23	S	VLRPRNKRRHVTLPSSPRSNTPMGDKDDDDD	54456	3504000	3287
Q07002	74	Cyclin-dependent kinase 18	CDK18	1	1.23E-10	125.2	S	STFSPTDSGEEPGQLSPGVQFQRRQNQRRFS	4141600	1779700	2213
Q9H3Q1	292	Cdc42 effector protein 4	CDC42EP4	1	1.32E-10	125.2	S	PSHALEDEGWAAAAPSPGSARSMGSHTTRDS	20363000	14112000	5642
Q9H3Q1	295	Cdc42 effector protein 4	CDC42EP4	1	1.32E-10	125.2	S	ALEDEGWAAAAPSPGSARSMGSHTTRDSSSL	20363000	9678700	5643
Q13428	996	Treacle protein	TCOF1	0.623939	2.35E-08	125.03	S	ASTPRKARASESTARSSSSESEDEDVIPATQ	2426600	0	2521
Q13428	997	Treacle protein	TCOF1	0.655615	2.35E-08	125.03	S	STPRKARASESTARSSSSESEDEDVIPATQC	2426600	718800	2522
Q13428	998	Treacle protein	TCOF1	0.5127	2.35E-08	125.03	S	TPRKARASESTARSSSSESEDEDVIPATQCL	92982	718800	2523
Q13428	999	Treacle protein	TCOF1	0.687344	2.35E-08	125.03	S	PRKARASESTARSSSSESEDEDVIPATQCLT	1672300	0	2524
Q96JM3	652	Chromosome alignment-maintaining phosphoprotein 1	CHAMP1	0.911792	1.56E-05	125.03	S	IKTDLDAMDIKGQESSSDQEQVDVESIDFSK	2682200	2404400	4970
Q96JM3	653	Chromosome alignment-maintaining phosphoprotein 1	CHAMP1	0.969266	1.31E-05	125.03	S	KTDLDAMDIKGQESSSDQEQVDVESIDFSKE	2682200	2404400	4971
P51532	609	Transcription activator BRG1	SMARCA4	0.85109	9.27E-11	125	T	GQTPAIGPDGEPLDETSQMSDLPVKVIHVES	9884600	10023000	7438
Q96E09	267	Protein FAM122A	FAM122A	0.914344	2.97E-10	124.98	S	GSSCNSPAKVSTTTDSPVSPAQAASPFIPLD	1016400	1091600	4877
Q96E09	270	Protein FAM122A	FAM122A	0.978845	2.97E-10	124.98	S	CNSPAKVSTTTDSPVSPAQAASPFIPLDELS	9746800	5002500	4878
P29692	147	Elongation factor 1-delta	EEF1D	1	4.53E-12	124.85	T	VSPMRQVEPPAKKPATPAEDDEDDDIDLFGS	0	10643000	7343
Q8TEQ0	639	Sorting nexin-29	SNX29	0.919873	8.47E-08	124.82	S	RQELIDLRGPVPGDLSQTSEDQSLSDFEISN	923510	840580	4483
Q8TEQ0	642	Sorting nexin-29	SNX29	0.790294	8.47E-08	124.82	S	LIDLRGPVPGDLSQTSEDQSLSDFEISNRAL	923510	840580	4484
Q8TEQ0	641	Sorting nexin-29	SNX29	0.539956	8.47E-08	124.82	T	ELIDLRGPVPGDLSQTSEDQSLSDFEISNRA	0	840580	7909
O43164	308	E3 ubiquitin-protein ligase Praja-2	PJA2	0.516037	0.000237971	124.78	S	ICSEQNTNDREKNHGSSPEQVVRPKVRKLIS	0	0	341
Q8NF91	8223	Nesprin-1	SYNE1	1	0.000375922	124.78	S	KKLIRLPLPDDEHDLSDRELELEDSAALSDL	0	0	4388
Q13620	144	Cullin-4B	CUL4B	0.383171	3.71E-08	124.69	S	GFDAKMAEESSSSSSSSSPTAATSQQQQLKN	0	0	2614
Q13620	148	Cullin-4B	CUL4B	0.809012	3.71E-08	124.69	T	KMAEESSSSSSSSSPTAATSQQQQLKNKSIL	0	797810	7570
Q13586	575	Stromal interaction molecule 1	STIM1	1	0.000384178	124.67	S	IEGVHPGSLVEKLPDSPALAKKALLALNHGL	2618200	1477100	2586
Q53EL6	457	Programmed cell death protein 4	PDCD4	1	0.000384178	124.67	S	QLRDLCPSRGRKRFVSEGDGGRLKPESY___	5485900	3666200	3124
P28370	119	Probable global transcription activator SNF2L1	SMARCA1	0.999888	0.000322585	124.6	S	LFAHFIQPSAQKSPTSPLNMKLGRPRIKKDE	9259300	5886000	1309
P48634	759	Protein PRRC2A	PRRC2A	0.669499	0.000388534	124.51	S	YPPGVHPSGLVPRERSDSGGSSSEPFDRHAP	0	556100	1629
O94806	41	Serine/threonine-protein kinase D3	PRKD3	0.99891	0.000630886	124.5	S	ASPCSSPKTGLSARLSNGSFSAPSLTNSRGS	760990	654100	691
Q9H5H4	90	Zinc finger protein 768	ZNF768	1	0.000519459	124.5	S	QSPRFEPESPGFESRSPGLVPPSPEFAPRSP	5585200	4283700	5716
Q9H5H4	97	Zinc finger protein 768	ZNF768	1	0.000519459	124.5	S	ESPGFESRSPGLVPPSPEFAPRSPESDSQSP	5585200	4283700	5717
Q92908	531	Transcription factor GATA-6	GATA6	0.491533	3.08E-12	124.48	S	PTSTSSNSDDCSKNTSPTTQPTASGAGAPVM	0	0	4727
Q9BUA3	308	Uncharacterized protein C11orf84	C11orf84	0.999581	0.000389967	124.48	S	PKDREVAEGGLPRAESPSPAPPPGLRGTLDL	8070400	3911900	5375
Q92908	530	Transcription factor GATA-6	GATA6	0.491533	3.08E-12	124.48	T	TPTSTSSNSDDCSKNTSPTTQPTASGAGAPV	0	0	7949
Q9UHY1	433	Nuclear receptor-binding protein	NRBP1	0.50622	0.000987902	124.48	T	EEVTSPVVPPSVKTPTPEPAEVETRKVVLMQ	0	0	8221
P13807	641	Glycogen [starch] synthase, muscle	GYS1	1	0.000201732	124.47	S	NEADAAQGYRYPRPASVPPSPSLSRHSSPHQ	4574400	2328000	1054
P13807	645	Glycogen [starch] synthase, muscle	GYS1	0.994964	0.000201732	124.47	S	AAQGYRYPRPASVPPSPSLSRHSSPHQSEDE	5200600	2712700	1055
Q12986	50	Transcriptional repressor NF-X1	NFX1	0.866034	0.000242678	124.47	S	QRRLDSNRIGRRNYSSPPPCHLSRQVPYDEI	1636800	0	2385
O00264	54	Membrane-associated progesterone receptor component 1	PGRMC1	0.5	1.48E-05	124.46	S	IFLLYKIVRGDQPAASGDSDDDEPPPLPRLK	35154000	0	112
P43307	247	Translocon-associated protein subunit alpha	SSR1	0.484914	1.10E-08	124.45	S	RKRKRPIQKVEMGTSSQNDVDMSWIPQETLN	0	0	1524
Q9UQ35	2692	Serine/arginine repetitive matrix protein 2	SRRM2	0.999856	0.000330771	124.42	S	SPRKPIDSLRDSRSLSYSPVERRRPSPQPSP	24202000	14027000	6696
Q9NYV4	333	Cyclin-dependent kinase 12	CDK12	0.999991	5.52E-09	124.39	S	SYSGRSPSPYGRRRSSSPFLSKRSLSRSPLP	1432100	1333200	6134
Q9NYV4	334	Cyclin-dependent kinase 12	CDK12	0.999965	5.52E-09	124.39	S	YSGRSPSPYGRRRSSSPFLSKRSLSRSPLPS	2934900	2066400	6135
Q7L4I2	32	Arginine/serine-rich coiled-coil protein 2	RSRC2	0.631375	0.00037769	124.38	S	SPDRDKKKEQSEVSVSPRASKHHYSRSRSRS	892390	0	3730
Q9Y3B9	51	RRP15-like protein	RRP15	0.998637	2.33E-10	124.31	S	EDEATDTSDSEGSCGSEKDHFYSDDDAIEAD	3476000	2112700	6853
P42166	74	Lamina-associated polypeptide 2, isoform alpha;Thymopoietin;	TMPO	0.999934	2.89E-08	124.31	T	SKGPPDFSSDEEREPTPVLGSGAAAAGRSRA	16993000	11693000	7378
P46100	731	Transcriptional regulator ATRX	ATRX	0.999997	8.87E-05	124.26	S	LPKSKQSETVDQNSDSDEMLAILKEVSRMSH	1048300	1009800	1556
A3KN83	697	Protein strawberry notch homolog 1	SBNO1	0.999521	7.67E-08	124.25	S	GIDLTAPSNNSSPRDSPCKENKIKKRKGEEI	2778000	2593900	30
O95819	639	Mitogen-activated protein kinase kinase kinase kinase 4	MAP4K4	0.983608	0.000200021	124.25	S	PVRTTSRSPVLSRRDSPLQGSGQQNSQAGQR	1604400	0	833
Q92576	1722	PHD finger protein 3	PHF3	0.807955	2.04E-06	124.25	S	NSCVQQSDNLKVAQNSPSVENIQTSQAEQAK	0	0	4645
Q92576	1724	PHD finger protein 3	PHF3	0.941717	2.04E-06	124.25	S	CVQQSDNLKVAQNSPSVENIQTSQAEQAKPL	1936300	1508000	4646
Q96B36	90	Proline-rich AKT1 substrate 1	AKT1S1	0.673858	4.26E-10	124.25	T	ARPPAPPPAPQPPSPTPSPPRPTLAREDNEE	1171200	0	7967
Q14160	1566	Protein scribble homolog	SCRIB	1	5.56E-08	124.21	S	VEDLGPQTSTSPGRLSPDFAEELRSLEPSPS	9040700	4641100	2677
Q6VMQ6	477	Activating transcription factor 7-interacting protein 1	ATF7IP	0.987309	0.00038305	124.21	S	ETNPDLEEKMESSFGSPSKQESSESLPKEAF	1576400	0	3601
Q96I25	222	Splicing factor 45	RBM17	0.959489	1.50E-05	124.21	S	AAIPPPVYEEQDRPRSPTGPSNSFLANMGGT	15824000	6974000	4950
Q9UBC2	593	Epidermal growth factor receptor substrate 15-like 1	EPS15L1	1	0.000336548	124.21	S	DLANLSEGVSLAERGSFGAMDDPFKNKALLF	647390	529650	6259
O95359	2317	Transforming acidic coiled-coil-containing protein 2	TACC2	0.999016	4.73E-06	124.2	S	PKMKKTPEKLDNTPASPPRSPAEPNDIPIAK	11969000	7545000	748
O95359	2321	Transforming acidic coiled-coil-containing protein 2	TACC2	0.999958	4.73E-06	124.2	S	KTPEKLDNTPASPPRSPAEPNDIPIAKGTYT	11323000	7052700	749
Q15648	1051	Mediator of RNA polymerase II transcription subunit 1	MED1	0.932151	0.0026453	124.2	T	STGGSKSPGSAGRSQTPPGVATPPIPKITIQ	3640100	1869200	7646
Q9H7L9	236	Sin3 histone deacetylase corepressor complex component SDS3	SUDS3	0.606919	3.55E-06	124.19	S	RTLNKLKSPKRPASPSSPEHLPATPAESPAQ	0	49696000	5759
Q8N5Y2	400	Male-specific lethal 3 homolog	MSL3	0.943295	0.000338185	124.12	S	FLHLEKKTPVHSRSSSPIPLTPSKEGSAVFA	895390	404780	4290
Q96T58	1062	Msx2-interacting protein	SPEN	0.999957	0.000689167	124.08	S	RESKKIKLDRLNTVASPKDCQELASISVGSG	1069500	860650	5173
Q641Q2;Q	620;620	WASH complex subunit FAM21A;WASH complex subunit FAM21C	FAM21A;FAM	0.999982	2.44E-10	124.07	S	ASELSKKKASALLFSSDEEDQWNIPASQTHL	3594200	2879600	3385
Q92597	333	Protein NDRG1	NDRG1	0.99114	0.00241601	124.04	S	ASMTRLMRSRTASGSSVTSLDGTRSRSHTSE	0	3274100	4651
Q9BRS8	58	La-related protein 6	LARP6	0.999994	0.000459488	124.04	S	AGDPARYLSPGWGSASEEEPSRGHSGTTASG	1430200	950940	5332
P29536	555	Leiomodin-1	LMOD1	0.997164	0.000422457	123.98	S	PLAPPLIMENLKNSLSPATQRKMGDKVLPAQ	1875000	2507000	1319
P06744	107	Glucose-6-phosphate isomerase	GPI	0.979613	0.000391249	123.95	S	YTEGRAVLHVALRNRSNTPILVDGKDVMPEV	2819600	1330800	918
P49792;Q	2454;147	E3 SUMO-protein ligase RanBP2;RanBP2-like and GRIP domain-c	RANBP2;RGPI	1	0.000391184	123.95	S	AKTAQEKDSLITPHVSRSSTPRESPCGKIAV	7533900	6005100	1701
Q92685	13	Dol-P-Man:Man(5)GlcNAc(2)-PP-Dol alpha-1,3-mannosyltransfera	ALG3	0.604931	0.00025532	123.95	S	___MAAGLRKRGRSGSAAQAEGLCKQWLQRA	0	0	4690
Q96PU5	479	E3 ubiquitin-protein ligase NEDD4-like	NEDD4L	0.999971	4.96E-06	123.87	S	SPVRRAVKDTLSNPQSPQPSPYNSPKPQHKV	2148200	1268400	5057
Q96PU5	483	E3 ubiquitin-protein ligase NEDD4-like	NEDD4L	0.998231	4.96E-06	123.87	S	RAVKDTLSNPQSPQPSPYNSPKPQHKVTQSF	1196800	849070	5058
Q96PU5	485	E3 ubiquitin-protein ligase NEDD4-like	NEDD4L	0.84716	4.96E-06	123.87	Y	VKDTLSNPQSPQPSPYNSPKPQHKVTQSFLP	0	849070	8413
Q14152	884	Eukaryotic translation initiation factor 3 subunit A	EIF3A	0.82402	0.000699905	123.86	S	ERRREEERRLGDSSLSRKDSRWGDRDSEGTW	344580	0	2653
Q68EM7	575	Rho GTPase-activating protein 17	ARHGAP17	0.99989	3.86E-12	123.85	S	SSAGILEQGPSPGDGSPPKPKDPVSAAVPAP	29386000	23906000	3428
Q92974	696	Rho guanine nucleotide exchange factor 2	ARHGEF2	0.999294	3.71E-08	123.84	S	REPALPLEPDSGGNTSPGVTANGEARTFNGS	3726200	2872800	4739
Q5SW79	630	Centrosomal protein of 170 kDa	CEP170	0.980074	0.000683399	123.76	S	ETEISESGMTVRSTGSATSLASQGERRRRTL	761720	0	3226
Q9UHB6	686	LIM domain and actin-binding protein 1	LIMA1	0.998069	2.59E-10	123.74	S	SLEMENENLVENGADSDEDDNSFLKQQSPQE	1382300	0	6329
Q9H3S7	1123	Tyrosine-protein phosphatase non-receptor type 23	PTPN23	0.735401	2.28E-12	123.71	S	PCLRRGAAAADLLSSSPESQHGGTQSPGGGQ	6766700	4440900	5644
Q27J81	1188	Inverted formin-2	INF2	0.939817	5.39E-12	123.68	S	DEDEEDTAPESALDTSLDKSFSEDAVTDSSG	3248600	1091900	3035
O75976	1368	Carboxypeptidase D	CPD	0.999999	5.07E-06	123.68	T	TGSKKSLLSHEFQDETDTEEETLYSSKH___	5123200	3008500	7221
O75976	1370	Carboxypeptidase D	CPD	0.999681	5.07E-06	123.68	T	SKKSLLSHEFQDETDTEEETLYSSKH_____	5123200	3008500	7222
Q27J81	1187	Inverted formin-2	INF2	0.499963	5.39E-12	123.68	T	EDEDEEDTAPESALDTSLDKSFSEDAVTDSS	3248600	1091900	7663
Q27J81	589	Inverted formin-2	INF2	0.735412	1.19E-08	123.64	S	SNVAREHNSMWASLSSPDAEAVEPDFSSIER	0	822120	3034
Q96I24	296	Far upstream element-binding protein 3	FUBP3	1	0.00194412	123.63	S	NDAGVRIQFKPDDGISPERAAQVMGPPDRCQ	3159300	2280200	4949
Q9HDC5	465	Junctophilin-1	JPH1	0.999926	0.0258502	123.63	S	KESPHFYRKGTTPPRSPEASPKHSHSPASSP	0	0	5885
Q9UQ35	2236	Serine/arginine repetitive matrix protein 2	SRRM2	1	0.000695873	123.63	S	RTAPAANLASRIPAASAAAMNLASARTPAIP	570630	533810	6645
Q9UQ35	2335	Serine/arginine repetitive matrix protein 2	SRRM2	1	0.000693781	123.63	S	AANYPSSSRTPQAPASANLVGPRSAHATAPV	2515900	1580400	6747
Q96T23	604	Remodeling and spacing factor 1	RSF1	1	0.000401574	123.62	S	EKSKKTFLDKDAQRLSPIPEEVPKSTLESEK	5630200	3421400	5149
Q15942	308	Zyxin	ZYX	0.990863	3.78E-08	123.61	S	NQKLGHPEALSAGTGSPQPPSFTYAQQREKP	4379500	5468700	2996
Q96JM3	651	Chromosome alignment-maintaining phosphoprotein 1	CHAMP1	0.762867	1.31E-05	123.61	S	YIKTDLDAMDIKGQESSSDQEQVDVESIDFS	0	0	4969
P56270	302	Myc-associated zinc finger protein	MAZ	0.893808	0.000281525	123.59	S	AFRDVYHLNRHKLSHSDEKPYQCPVCQQRFK	1230900	0	1894
Q7Z6E9	984	E3 ubiquitin-protein ligase RBBP6	RBBP6	1	9.61E-05	123.59	T	NKTDSLFVLPSRDDATPVRDEPMDAESITFK	2890700	1818100	7813
Q96RG2	843	PAS domain-containing serine/threonine-protein kinase	PASK	0.995975	3.79E-08	123.58	S	VCLVSSEHYAASDRESPGHVPSTLDAGPEDT	1745700	660030	5089
Q66K74	759	Microtubule-associated protein 1S;MAP1S heavy chain;MAP1S li	MAP1S	0.999409	2.73E-05	123.54	S	EFEHRKAVPMAPAPASPGSSNDSSARSQERA	17467000	15976000	3403

P27816	624	Microtubule-associated protein 4	MAP4	0.970961	1.54E-12	123.51	S	SLQDVGQSAAPTFMISPETVTGTGKKCSLPA	2831200	1577800	1293
P53396	455	ATP-citrate synthase	ACLY	0.998988	0.000717267	123.51	S	NASGSTSTPAPSRTASFSESRADEVAPAKKA	10402000	6650300	1834
Q14C86	903	GTPase-activating protein and VPS9 domain-containing protei	GAPVD1	0.5	0.000448707	123.51	S	KQRHSYPERLVRSRSSDIVSSVRRPMSDPSW	8490500	0	2848
P82094	399	TATA element modulatory factor	TMF1	1	2.73E-10	123.44	S	LVIPTEEAEMEESGRSATPVNCEQPDILVSS	5561600	4031700	1986
P82094	401	TATA element modulatory factor	TMF1	1	2.73E-10	123.44	T	IPTEEAEMEESGRSATPVNCEQPDILVSSTP	5561600	4031700	7463
Q9H4Z3	30	Phosphorylated CTD-interacting factor 1	PCIF1	0.999948	3.92E-06	123.42	S	LLSHSPGTSNQSQPCSPKPIRLVQDLPEELV	7238800	4607200	5701
O14639	435	Actin-binding LIM protein 1	ABLIM1	0.999471	0.00555157	123.4	S	RQSLGESPRTLSPTPSAEGYQDVRDRMIHRS	1032700	439350	165
Q9Y6X4	637	Soluble lamin-associated protein of 75 kDa	FAM169A	1	0.00101635	123.4	S	LDQFTQSAEKAVDSSSEEIEVEVPVVDRRNL	1248100	1012300	7027
O14639	429	Actin-binding LIM protein 1	ABLIM1	0.509109	0.00555157	123.4	T	YDDKQERQSLGESPRTLSPTPSAEGYQDVRD	1032700	0	7125
Q7Z6Z7	2826	E3 ubiquitin-protein ligase HUWE1	HUWE1	0.999913	3.69E-08	123.36	S	PTRPSGEAETTQMELSPAPTITSLSPERAED	1506000	1689700	3879
Q7Z6Z7	2833	E3 ubiquitin-protein ligase HUWE1	HUWE1	0.79975	3.69E-08	123.36	S	AETTQMELSPAPTITSLSPERAEDSDALTAV	0	137450	3880
Q7Z6Z7	2835	E3 ubiquitin-protein ligase HUWE1	HUWE1	0.991464	3.69E-08	123.36	S	TTQMELSPAPTITSLSPERAEDSDALTAVSS	1506000	1689700	3881
Q96DR7	222	Rho guanine nucleotide exchange factor 26	ARHGEF26	0.999974	3.86E-08	123.36	S	QKSSSEQKLPLQRLPSQENELLENPSVVLST	2546300	1387500	4863
Q6P2E9	727	Enhancer of mRNA-decapping protein 4	EDC4	0.49957	4.79E-10	123.35	T	QEVEPLGLPQASPSRTRSPDVISSASTALSQ	0	0	7759
P17980	9	26S protease regulatory subunit 6A	PSMC3	0.998451	0.000190591	123.29	S	________MNLLPNIESPVTRQEKMATVWDEA	2040500	1450600	1124
P27708	1859	CAD protein;Glutamine-dependent carbamoyl-phosphate synthase	CAD	1	0.000271541	123.28	S	LPDGRFHLPPRIHRASDPGLPAEEPKEKSSR	11549000	16178000	1287
Q00587	353	Cdc42 effector protein 1	CDC42EP1	1	0.000295046	123.28	S	QERVEVLPQARASWESLDEEWRAPQAGSRTP	716880	889510	2026
Q9BRS8	409	La-related protein 6	LARP6	0.947445	0.000355323	123.26	S	RKSPLAEEGRLNCSTSPEIFRKCMDYSSDSS	2117500	824140	5328
Q13153	174	Serine/threonine-protein kinase PAK 1	PAK1	0.999994	2.80E-15	123.22	S	LNVKAVSETPAVPPVSEDEDDDDDDATPPPV	1074900	0	2424
Q07157	277	Tight junction protein ZO-1	TJP1	0.866926	1.04E-05	123.14	S	RDERATLLNVPDLSDSIHSANASERDDISEI	10560000	9537500	2217
Q07157	280	Tight junction protein ZO-1	TJP1	0.981877	1.04E-05	123.14	S	RATLLNVPDLSDSIHSANASERDDISEIQSL	10560000	9537500	2218
Q9NRA8	951	Eukaryotic translation initiation factor 4E transporter	EIF4ENIF1	0.999428	0.000471143	123.11	S	HMHSQLEHRPSQRSSSPVGLAKWFGSDVLQQ	2268300	220490	5956
Q01130	187	Serine/arginine-rich splicing factor 2	SRSF2	0.999999	0.00101169	123.1	S	KSSSVSRSRSRSRSRSRSRSPPPVSKRESKS	1663800	3219400	2058
Q9NRL2	1547	Bromodomain adjacent to zinc finger domain protein 1A	BAZ1A	0.876681	2.47E-05	123.08	T	KLGLHVTPSNVDQVSTPPAAKKSRI______	955020	958780	8155
O43379	1070	WD repeat-containing protein 62	WDR62	0.999672	0.000138001	123.01	S	EQEKFLRHHFETLTESPCRALGDVEASEAED	0	621120	368
Q8NEN9	967	PDZ domain-containing protein 8	PDZD8	0.999985	0.000742691	123.01	S	SKTRLSEPGTDLVEPSKHTPNTSDNEGSDT	795030	434610	4375
P49023	85	Paxillin	PXN	0.836482	0.00028676	123	S	QPSSSRFIHQQPQSSSPVYGSSAKTSSVSNP	662310	0	1643
Q9UQ35	2155	Serine/arginine repetitive matrix protein 2	SRRM2	1	1.58E-05	122.97	S	GSSRTPMSVLQQAGGSMMDGPGPRIPDHQRT	800870	1700800	6746
Q6VMQ6	673	Activating transcription factor 7-interacting protein 1	ATF7IP	1	0.000230094	122.96	S	EDLKKRHEHPPNPPVSPGKTVNDVNSNNNMS	1488300	1306600	3600
P49761	224	Dual specificity protein kinase CLK3	CLK3	1	0.000386408	122.93	S	YRERRDSDTYRCEERSPSFGEDYYGPSRSRH	6222800	2579700	1687
P49761	226	Dual specificity protein kinase CLK3	CLK3	1	0.000386408	122.93	S	ERRDSDTYRCEERSPSFGEDYYGPSRSRHRR	3567100	1736700	1688
O43159	62	Ribosomal RNA-processing protein 8	RRP8	0.999996	1.36E-08	122.87	S	LEAASLSQHPPSLCISDSEEEEEERKKKCPK	15239000	11114000	339
Q9BUJ2	718	Heterogeneous nuclear ribonucleoprotein U-like protein 1	HNRNPUL1	0.746983	4.58E-12	122.84	S	PPQPPPQQPPPPPSYSPARNPPGASTYNKNS	0	0	5380
Q15750	7	TGF-beta-activated kinase 1 and MAP3K7-binding protein 1	TAB1	0.998116	1.96E-10	122.76	S	__________MAAQRRSLLQSEQQPSWTDDLP	3024400	1700100	2982
Q9UQ35	1925	Serine/arginine repetitive matrix protein 2	SRRM2	0.999999	0.00665404	122.69	S	RSRSRASPVSRRRSRSRTPPVTRRRSRSRTP	44961000	27859000	6718
Q9BXB4	27	Oxysterol-binding protein-related protein 11	OSBPL11	0.99914	0.00761944	122.69	T	VSESEGKLEGQATAVTPNKNSSCGGGISSSS	0	1748100	8062
Q9UQ35	1927	Serine/arginine repetitive matrix protein 2	SRRM2	0.999998	0.00665404	122.69	T	RSRASPVSRRRSRSRTPPVTRRRSRSRTPTT	44961000	27859000	8271
Q8IU81	436	Interferon regulatory factor 2-binding protein 1	IRF2BP1	1	0.000367041	122.66	S	SPIAALKNVAEALGHSPKDPGGGGGPVRAGG	0	1806300	4037
Q9UGU5	497	HMG domain-containing protein 4	HMGXB4	1	0.000212741	122.66	S	SEGSMKVKASSVGVLSPQKKSPPTMLLPAS	0	1779000	6318
Q9Y2W1	264	Thyroid hormone receptor-associated protein 3	THRAP3	1	0.00409649	122.66	S	PALKSPLQSVVVRRRSPRPSPVPKPSPPLSS	2890000	1939800	6828
Q9Y2W1	268	Thyroid hormone receptor-associated protein 3	THRAP3	1	0.00409649	122.66	S	SPLQSVVVRRRSPRPSPVPKPSPPLSSTSQM	2890000	1939800	6829
Q15678	591	Tyrosine-protein phosphatase non-receptor type 14	PTPN14	0.667293	0.00023852	122.64	S	ATSTPDLASHRHKYVSGSSPDLVTRKVQLSV	0	1561200	2975
Q15678	594	Tyrosine-protein phosphatase non-receptor type 14	PTPN14	0.9913	0.00023852	122.64	S	TPDLASHRHKYVSGSSPDLVTRKVQLSVKTF	4325700	2195300	2977
Q6DN90	180	IQ motif and SEC7 domain-containing protein 1	IQSEC1	1	0.000432428	122.64	S	RMSRRIVLSNMRMQFSFEGPEKVHSSYFEGK	1318600	428260	3446
Q15366	187	Poly(rC)-binding protein 2	PCBP2	0.38959	0.000398984	122.63	S	QSPPKGVTIPYRPKPSSSPVIFAGGQDRYST	0	0	2907
Q9Y3E2	122	BolA-like protein 1	BOLA1	0.660268	0.00256976	122.63	T	QARTPAQWRENSQLDTSPPCLGGNKKTLGTP	0	0	8292
Q9NPG3	660	Ubinuclein-1	UBN1	1	3.14E-10	122.57	S	ASGGLANPPPVNLEDSLDEDLIRNPASSVEA	1124600	1749800	5889
P08238	445	Heat shock protein HSP 90-beta	HSP90AB1	0.972313	0.000435306	122.55	S	YEAFSKNLKLGIHEDSTNRRRLSELLRYHTS	6054200	4351100	950
Q9C0B5	398	Palmitoyltransferase ZDHHC5	ZDHHC5	0.999988	0.000369306	122.55	S	EPTSIAESSRHPSYRSEPSLEPESFRSPTFG	1169200	1025000	5513
Q9UQ35	1124	Serine/arginine repetitive matrix protein 2	SRRM2	0.999803	0.000435306	122.55	S	ASRSPIRQDRGEFSASPMLKSGMSPEQSRFQ	7646500	3356000	6627
O43166	1270	Signal-induced proliferation-associated 1-like protein 1	SIPA1L1	1	0.000369841	122.52	S	SPSGRLMRQDPVVHLSPNKQGHSDSHYSSHS	1803500	1129400	344
Q14004	395	Cyclin-dependent kinase 13	CDK13	1	0.00294201	122.52	S	GDVSPSPYSSSSWRRSRSPYSPVLRRSGKSR	2656600	1308800	2642
Q14004	397	Cyclin-dependent kinase 13	CDK13	0.999983	0.00294201	122.52	S	VSPSPYSSSSWRRSRSPYSPVLRRSGKSRSR	2656600	1308800	2643
P27816	282	Microtubule-associated protein 4	MAP4	0.920486	0.000669419	122.52	T	ATKTEVALAKDMESPTKLDVTLAKDMQPSME	1976500	1248500	7335
Q14004	399	Cyclin-dependent kinase 13	CDK13	0.877175	0.00294201	122.52	Y	PSPYSSSSWRRSRSPYSPVLRRSGKSRSRSP	2656600	1308800	8379
P49792	835	E3 SUMO-protein ligase RanBP2	RANBP2	0.686174	0.000371039	122.46	S	AIKKEMQELKLNSSNSASPHRWPTENYGPDS	1331100	0	1712
Q5VTR2	136	E3 ubiquitin-protein ligase BRE1A	RNF20	1	0.000486192	122.46	S	LTERKALVVPEPEPDSDSNQERKDDRERGEG	2640100	354540	3338
Q9P2D3	1737	HEAT repeat-containing protein 5B	HEATR5B	0.992655	1.45E-05	122.46	S	ILVRHMPHLSTKVSDSPSHIATKTRLSEESA	0	0	6236
Q9UIF9	1399	Bromodomain adjacent to zinc finger domain protein 2A	BAZ2A	0.676601	0.00121788	122.46	T	PKRRAGDPGEMPQSPTGLGQPKRRGRPPSKF	1644100	1340200	8222
P49792	1160	E3 SUMO-protein ligase RanBP2	RANBP2	0.999314	2.05E-10	122.44	S	PTLETANKNHETDGGSAHGDDDDDGPHFEPV	10814000	1735300	1714
Q6UUV7	162	CREB-regulated transcription coactivator 3	CRTC3	0.946836	0.000785548	122.44	S	KHPGFRLTSALNRTNSDSALHTSALSTKPQD	0	0	3592
Q7Z309	137	Protein FAM122B	FAM122B	0.99997	0.00040487	122.44	S	RGFGKMFVSSSGLPPSPVPSPRRFSRRSQSP	2338500	1407000	3773
Q7Z309	141	Protein FAM122B	FAM122B	1	0.00040487	122.44	S	KMFVSSSGLPPSPVPSPRRFSRRSQSPVKCI	2338500	1407000	3774
Q86X02	304	Cerebellar degeneration-related protein 2-like	CDR2L	0.878362	4.39E-06	122.44	S	QPGRGDDLGAQDGVSSPAASPGHVVRKSCSD	3571800	46236	3996
Q9BRD0	325	BUD13 homolog	BUD13	0.999983	0.000371758	122.42	S	LSFPKNSKYEYDPDISPPRKKQAKSHFGDKK	2012000	750280	5321
Q00613	314	Heat shock factor protein 1	HSF1	0.978116	5.64E-10	122.39	S	EEPPSPPQSPRVEEASPGRPSSVDTLLSPTA	1472000	820380	2034
Q9UPU5	2080	Ubiquitin carboxyl-terminal hydrolase 24	USP24	0.895973	1.10E-07	122.35	S	DLSLSAPSSPEISPQSSPRPHRPNNDRLSIL	0	2899300	6584
Q9BQG0	1310	Myb-binding protein 1A	MYBBP1A	0.999707	0.000441698	122.34	S	LARKKARLSLVIRSPSLLQSGAKKKAQVRKA	727810	196070	5313
Q9BXF6	307	Rab11 family-interacting protein 5	RAB11FIP5	0.999831	0.000441698	122.34	S	DSAQSPKLFTHKRTYSDEANQMRVAPPRALL	692910	528590	5449
Q9UPR0	584	Inactive phospholipase C-like protein 2	PLCL2	0.999999	1.12E-07	122.34	T	AKKLSSNCSGVEGDVTDEDEGAEMSQRMGKE	1141100	986040	8248
P42684	620	Abelson tyrosine-protein kinase 2	ABL2	0.998688	0.000352064	122.33	S	SLAPGFIRGAQASSGSPALPRKQRDKSPSSL	0	0	1510
Q5H9R7	852	Serine/threonine-protein phosphatase 6 regulatory subunit 3	PPP6R3	0.607175	0.00358978	122.33	S	PETAEAKCAAPRPPSSSPEQRTGQPSAPGDT	0	0	3162
Q8IWW6	240	Rho GTPase-activating protein 12	ARHGAP12	0.99853	7.26E-05	122.33	S	EQIRATTPPNQGRPDSPVYANLQELKISQSA	11235000	9844300	4077
Q8IWW6	230	Rho GTPase-activating protein 12	ARHGAP12	0.500021	7.26E-05	122.33	T	SGDELSSSSTEQIRATTPPNQGRPDSPVYAN	1409400	1332500	7845
Q8IWW6	231	Rho GTPase-activating protein 12	ARHGAP12	0.862892	7.26E-05	122.33	T	GDELSSSSTEQIRATTPPNQGRPDSPVYANL	11235000	9844300	7846
P22626	85	Heterogeneous nuclear ribonucleoproteins A2/B1	HNRNPA2B1	1	0.00429884	122.28	S	SSMAEVDAAMAARPHSIDGRVVEPKRAVARE	1848100	982220	1215
Q04637	1231	Eukaryotic translation initiation factor 4 gamma 1	EIF4G1	1	0.000374559	122.28	S	RGRDAVKREAALPPVSPLKAALSEEELEKKS	2768000	1838600	2155
O60885	1117	Bromodomain-containing protein 4	BRD4	1	0.00431212	122.25	S	VQPQPLVVVKEEKIHSPIIRSEPFSPSLRPE	1485800	828860	537
Q7Z7L8	406	Uncharacterized protein C11orf96	C11orf96	0.77379	0.000523293	122.18	S	KAKKSFLQSLECLRRSTQSLSLQREQLSSCK	0	0	3890
Q9BXP5	493	Serrate RNA effector molecule homolog	SRRT	1	0.000446931	122.18	S	ICWNLQNIRLRECELSPGVNRDLTRRVRNIN	917760	1050100	5457
Q16204	240	Coiled-coil domain-containing protein 6	CCDC6	1	0.000248185	122.16	S	EAEKRILQEKLDQPVSAPPSPRDISMEIDSP	5151400	4291700	3002
Q16204	244	Coiled-coil domain-containing protein 6	CCDC6	1	0.000248185	122.16	S	RILQEKLDQPVSAPPSPRDISMEIDSPENMM	6596800	5767500	3003
P49790	520	Nuclear pore complex protein Nup153	NUP153	0.782422	0.0062874	122.14	T	SQALTNKVQMTSPSSTGSPMFKFSSPIVKST	0	0	7420
Q7Z333	1017	Probable helicase senataxin	SETX	1	0.000546703	122.13	S	QNNVGDTSRGQVIIISDSDDDDDERILSLEK	1092800	897490	3776
Q7Z333	1019	Probable helicase senataxin	SETX	1	0.000546703	122.13	S	NVGDTSRGQVIIISDSDDDDDERILSLEKLT	1092800	897490	3777
Q13614	631	Myotubularin-related protein 2	MTMR2	0.589587	0.000809958	122.12	S	REISNRSTSSSERASSPAQCVTPVQTVV___	0	0	2604
Q6ZSR9	67	Uncharacterized protein FLJ45252		1	7.85E-05	122.12	S	GDMLDCSGSRPLLMESEEEDESCRPPPGKLG	2248000	1056700	3672
Q96S66	438	Chloride channel CLIC-like protein 1	CLCC1	0.993731	0.00135024	122.12	S	LRERDVDLRFQTGNKSPEVLRAFDVPDAEAR	0	353000	5115
Q8WXI9	134	Transcriptional repressor p66-beta	GATAD2B	0.825617	1.26E-05	122.1	S	LTPSPDIIVLSDNEASSPRSSSRMEERLKAA	0	3799300	4598
Q9P0K1	834	Disintegrin and metalloproteinase domain-containing protein	ADAM22	0.996121	0.000300051	122.1	S	KHISDICENGRPRSNSWQGNLGGNKKKIRGK	1525500	1583300	6180
P78559	1326	Microtubule-associated protein 1A;MAP1A heavy chain;MAP1 li	MAP1A	0.999546	0.000665606	121.99	S	SPDEHILTPDSSFSKSPESLPGPALEDIAIK	788250	501220	1979
Q6PKG0	766	La-related protein 1	LARP1	0.999241	0.000425509	121.95	S	ANKLFGAPEPSTIARSLPTTVPESPNYRNTR	40306000	17536000	3562
Q6PKG0	774	La-related protein 1	LARP1	0.999939	0.000425509	121.95	S	EPSTIARSLPTTVPESPNYRNTRTPRTPRTP	22323000	18412000	3563
Q9H2J7	701	Sodium-dependent neutral amino acid transporter B(0)AT2	SLC6A15	0.989349	0.000825881	121.95	S	PSPNFGKNIYRKQSGSPTLDTAPNGRYGIGY	1981400	988810	5618
Q6ZRV2	892	Protein FAM83H	FAM83H	0.991135	0.00124932	121.92	S	ERKGSPTPGFSTRRGSPTTGFIEQKGSPTSA	671250	620900	3664
Q15758	493	Neutral amino acid transporter B(0)	SLC1A5	0.5	0.000455729	121.9	S	GAGLLQNYVDRTESRSTEPELIQVKSELPLD	677190	376330	2983
Q86U44	43	N6-adenosine-methyltransferase 70 kDa subunit	METTL3	0.724563	0.000382108	121.9	S	QDSGHLDLRNPEAALSPTFRSDSPVPTAPTS	837130	299240	3917
Q15758	494	Neutral amino acid transporter B(0)	SLC1A5	0.5	0.000455729	121.9	T	AGLLQNYVDRTESRSTEPELIQVKSELPLDP	677190	376330	7649
Q96JM3	319	Chromosome alignment-maintaining phosphoprotein 1	CHAMP1	1	8.91E-05	121.84	S	APAVSPGSWKPGPPGSPRPWKSNPSASSGPW	27364000	12415000	4990
Q2NKX8	1188	DNA excision repair protein ERCC-6-like	ERCC6L	0.999993	3.49E-10	121.82	S	SLGAPEPLSGEQLVGSPQDKAAEATNDYETL	3111100	3613000	3048
Q6IN85	774	Serine/threonine-protein phosphatase 4 regulatory subunit 3/	SMEK1	0.997575	3.32E-12	121.75	S	QSSTTNLPGSPGSPGSPGSPGSPGSVPKNTS	11391000	1509100	3479
Q6IN85	780	Serine/threonine-protein phosphatase 4 regulatory subunit 3/	SMEK1	0.971961	3.32E-12	121.75	S	LPGSPGSPGSPGSPGSPGSVPKNTSQTAAIT	4472600	8480200	3481
Q15047	1066	Histone-lysine N-methyltransferase SETDB1	SETDB1	0.999994	0.00378369	121.72	S	MSVVTESSRNYGYNPSPVKPEGLRRPPSKTS	750490	414360	2863
Q13601	3	KRR1 small subunit processome component homolog	KRR1	0.999932	0.000381285	121.68	S	_______________MASPSLERPEKGAGKSEF	3348800	3906800	2600
Q5TDH0	105	Protein DDI1 homolog 2	DDI2	0.664403	0.00193881	121.68	S	NLPRIDFSSIAVPGTSSPRQRQPPGTQQSHS	0	2021000	3301
Q5TDH0	106	Protein DDI1 homolog 2	DDI2	0.979821	0.00137946	121.68	S	LPRIDFSSIAVPGTSSPRQRQPPGTQQSHSS	0	0	3302
Q6P6C2	361	RNA demethylase ALKBH5	ALKBH5	0.997044	0.000462626	121.68	S	ENRRSVLLPTHRRRGSFSSENYWRKSYESSE	1255600	1596000	3532
Q96EV2	741	RNA-binding protein 33	RBM33	0.984761	0.000462626	121.68	S	CSATPSAQVKPIVSASPPSRAVAGSRSSQGK	1345400	1211100	4892
Q5TDH0	104	Protein DDI1 homolog 2	DDI2	0.333333	0.00343593	121.68	T	PNLPRIDFSSIAVPGTSSPRQRQPPGTQQSH	0	0	7725
Q8WY36	159	HMG box transcription factor BBX	BBX	0.996044	0.000555153	121.61	S	NPGYKWCPTTNKPVKSPTPTVNPRKKLWAFP	43618	0	4601
P42677;Q78;78	40	S ribosomal protein S27;40S ribosomal protein S27-like	RPS27;RPS27	0.999999	0.000811957	121.6	S	LCQPTGGKARLTEGCSFRRKQH_________	13197000	3820200	1508
P02545	107	Prelamin-A/C;Lamin-A/C	LMNA	1	0.00440922	121.5	S	LDSVAKERARLQLELSKVREEFKELKARNTK	891300	614040	860
Q9Y519	402	Transmembrane protein 184B	TMEM184B	1	0.000389209	121.5	S	HSLSGARDNEKTLLLSSDDEF__________	1385400	1575800	6942
P27816	101	Microtubule-associated protein 4	MAP4	0.607624	7.84E-10	121.5	T	NGGHGVEGSDTTGSPTEFLEEKMAYQEYPNS	0	2365600	7340
P49327	974	Fatty acid synthase;[Acyl-carrier-protein] S-acetyltransfera	FASN	0.859123	4.45E-08	121.48	S	YQWDDPDPRLFDHPESPTPNPTEPLFLAQAE	3368100	1764500	1652
Q969V6	156	MKL/myocardin-like protein 1	MKL1	0.78768	7.61E-08	121.48	S	LEARVSEPLLSATSASPTQVVSQLPMGRDSR	2523100	3849300	4763
Q12888	523	Tumor suppressor p53-binding protein 1	TP53BP1	0.985685	0.000445895	121.45	S	LTGDSCKLMLSTSEYSQSPKMESLSSHRIDE	4294100	0	2347
P51532	610	Transcription activator BRG1	SMARCA4	0.85109	1.56E-10	121.44	S	QTPAIGPDGEPLDETSQMSDLPVKVIHVESG	9884600	10023000	1781
Q6P0Q8	148	Microtubule-associated serine/threonine-protein kinase 2	MAST2	0.744764	0.000695782	121.44	S	EASNLVRMRNQSLGQSAPSLTAGLKELSLPR	1117900	0	3503
P17275	259	Transcription factor jun-B	JUNB	1	0.00542058	121.42	S	QTVPEARSRDATPPVSPINMEDQERIKVERK	0	684100	1109
Q5SW79	356	Centrosomal protein of 170 kDa	CEP170	1	0.00150678	121.42	S	NPPQMLWERTEEDSKSIKSDVPVYLKRLKGN	6653700	2675700	3220
Q5SW79	359	Centrosomal protein of 170 kDa	CEP170	0.999999	0.00150678	121.42	S	QMLWERTEEDSKSIKSDVPVYLKRLKGNKHD	6653700	2675700	3221
Q8TB72	136	Pumilio homolog 2	PUM2	1	0.00142626	121.42	S	AETDGPEKGDQKGKASPFEEDQNRDLKQGDD	0	408090	4448
P17275	255	Transcription factor jun-B	JUNB	1	0.00542058	121.42	T	KEEPQTVPEARSRDATPPVSPINMEDQERIK	0	684100	7295
Q96A57	24	Transmembrane protein 230	TMEM230	0.900056	0.00087669	121.31	S	ATGIPSSKVKYSRLSSTDDGYIDLQFKKTPP	0	177220	4769
P07197	736	Neurofilament medium polypeptide	NEFM	1	0.000956177	121.26	S	KEEKPKDVPEKKKAESPVKEEAVAEVVTITK	901290	701460	928
Q3KQU3	125	MAP7 domain-containing protein 1	MAP7D1	0.965115	0.000270527	121.25	S	RRSSQPSPTAVPASDSPPTKQEVKKAGERHK	2109100	437280	3073
O14874	31	[3-methyl-2-oxobutanoate dehydrogenase [lipoamide]] kinase,	BCKDK	0.476983	0.0008825	121.24	S	PLRPLLGPALALARSTSATDTHHVEMARER	0	0	211
O14874	33	[3-methyl-2-oxobutanoate dehydrogenase [lipoamide]] kinase,	BCKDK	0.329786	0.0008825	121.24	S	RPLLGPALALARSTSATDTHHVEMARERSK	0	0	212
O14874	32	[3-methyl-2-oxobutanoate dehydrogenase [lipoamide]] kinase,	BCKDK	0.476983	0.0008825	121.24	T	LRPLLGPALALARSTSATDTHHVEMARERS	0	0	7133
O14681	326	Etoposide-induced protein 2.4 homolog	EI24	1	0.000396916	121.21	S	LQSALSSSTSAEKFPSPHPSPAKLKATAGH_	22613000	13464000	185
O14681	330	Etoposide-induced protein 2.4 homolog	EI24	1	0.000396916	121.21	S	LSSSTSAEKFPSPHPSPAKLKATAGH_____	21538000	12501000	186
P07900	453	Heat shock protein HSP 90-alpha	HSP90AA1	1	0.000477481	121.21	S	YEQFSKNIKLGIHEDSQNRKKLSELLRYYTS	7512100	4586500	942
Q9NWS9	137	Zinc finger protein 446	ZNF446	0.999546	4.87E-08	121.21	S	QEVLPAAQKTEEPLGSPHPSGTVESPGEGPQ	3611800	3005100	6068
Q9NWS9	146	Zinc finger protein 446	ZNF446	0.999623	4.87E-08	121.21	S	TEEPLGSPHPSGTVESPGEGPQDTRIEGSVQ	3611800	3005100	6069
Q9BXS9	752	Solute carrier family 26 member 6	SLC26A6	1	0.000329385	121.18	S	DAVTFALQHPRPVPDSPVSVTRL________	9561700	6014300	5459
Q9BXS9	755	Solute carrier family 26 member 6	SLC26A6	0.999198	0.000329385	121.18	S	TFALQHPRPVPDSPVSVTRL___________	5494000	3376600	5460
Q01804	1014	OTU domain-containing protein 4	OTUD4	0.883894	0.00219359	121.02	S	KTAADVVSPGANSVDSRVQRPKEESSEDENE	0	931990	2077
Q9UQ35	1453	Serine/arginine repetitive matrix protein 2	SRRM2	0.98817	0.00108535	121	T	RSRSGSSPGLRDGSGTPSRHSLSGSSPGMKD	4096300	5262700	8254

Q8WUZ0	114	B-cell CLL/lymphoma 7 protein family member C	BCL7C	0.958028	4.25E-12	120.98	S	QSFHSEGSLQKLGTEPSPGGTPQPSRPVSPAG	8545600	5506500	4523
P78312	1144	Protein FAM193A	FAM193A	1	0.000485298	120.96	S	SPGEHQQNSKLVLAESPQPKGKNKNKKKKKG	4169300	3038700	1952
P24928	1889	DNA-directed RNA polymerase II subunit RPB1	POLR2A	0.270079	0.000166095	120.9	S	YSPTSPTYSPTTPKYSPTSPTYSPTSPVYTP	0	0	1246
P24928	1892	DNA-directed RNA polymerase II subunit RPB1	POLR2A	0.375252	0.000166095	120.9	S	TSPTYSPTTPKYSPTSPTYSPTSPVYTPTSP	0	0	1247
Q69YN4	173	Protein virilizer homolog	KIAA1429	1	0.00409979	120.9	S	NPKHADGEKEDQFNGSPPRPQPRGRPTPPGP	0	1308600	3434
Q8NFQ8	118	Torsin-1A-interacting protein 2	TOR1AIP2	0.898093	0.000909629	120.9	S	LPSENLGKEPLDPDPSHSPSDKVGRADAHLG	517080	0	4403
Q9UQ35	2142	Serine/arginine repetitive matrix protein 2	SRRM2	0.94651	0.000329074	120.9	S	LDRCRSPGMLEPLGSSRTPMSVLQQAGGSMM	781210	0	6607
Q3KQU3	554	MAP7 domain-containing protein 1	MAP7D1	0.927795	3.17E-07	120.9	T	SAAPASPAPSAPSPTPAPPQKEQPPAETPT	24250000	1473000	7667
O60220	96	Mitochondrial import inner membrane translocase subunit Tim8	TIMM8A	0.992359	0.00357496	120.86	S	LEQTQKSKPVFSESLSD_______________	1276200	0	436
Q13620	141	Cullin-4B	CUL4B	0.266105	4.99E-08	120.85	S	EFVGFDAKMAEESSSSSSSSSPTAATSQQQQ	0	0	2611
Q13620	142	Cullin-4B	CUL4B	0.286488	4.99E-08	120.85	S	FVGFDAKMAEESSSSSSSSSPTAATSQQQQL	0	0	2612
P51116	601	Fragile X mental retardation syndrome-related protein 2	FXR2	0.993474	2.15E-05	120.8	S	NRSRRRRNRGNRTDGSISGDRQPVTVADYIS	9627100	2881500	1775
P51116	603	Fragile X mental retardation syndrome-related protein 2	FXR2	0.99997	2.15E-05	120.8	S	SRRRRNRGNRTDGSISGDRQPVTVADYISRA	9627100	2881500	1776
Q7Z5L9	406	Interferon regulatory factor 2-binding protein 2	IRF2BP2	0.746405	1.15E-07	120.74	S	MTPTSSFVSPPPPTASPHSNRTTPPEAAQNG	8089300	6584400	3848
Q3B726	299	DNA-directed RNA polymerase I subunit RPA43	TWISTNB	0.601211	8.02E-08	120.72	S	HQEVQDQDPVFQGSDSSGYQSDHKKKKKKRK	0	0	3058
O15164	811	Transcription intermediary factor 1-alpha	TRIM24	1	0.00060973	120.63	S	NSSNGKSEWLDPSQKSPLHVGETRKEDDPNE	4021800	3362400	297
Q96RG2	116	PAS domain-containing serine/threonine-protein kinase	PASK	0.630042	3.49E-05	120.63	S	PRGSVSCCSLLRGLSSGWSSPLLPAPVCNPN	0	139650	5090
O95104	656	Splicing factor, arginine/serine-rich 15	SCAF4	0.999994	7.33E-08	120.61	S	VAQNGGAETSHTEPVSPIPKPLPVPVPPIPV	3699800	2121100	730
Q9BUR4	26	Telomerase Cajal body protein 1	WRAP53	1	5.22E-12	120.61	S	PDCCPSDQDPAPAHPSPHASPMNKNADSELM	8016800	3035700	5390
Q9BUR4	30	Telomerase Cajal body protein 1	WRAP53	1	5.22E-12	120.61	S	PSDQDPAPAHPSPHASPMNKNADSELMPPPP	8016800	3035700	5391
Q96T17	290	MAP7 domain-containing protein 2	MAP7D2	0.91734	0.0025102	120.6	S	LRRCEFSGGIPKRPSSPVISKTATKAYPQSP	0	486670	5144
Q9NYV4	332	Cyclin-dependent kinase 12	CDK12	0.999994	0.000937525	120.6	S	GSYSGRSPSPYGRRRSSSPFLSKRSLSRSPL	0	313550	6133
Q5VUA4	2189	Zinc finger protein 318	ZNF318	0.999998	0.00057772	120.59	S	DFVTRTSGVQKDKLCSPLSEPGDPSKCSSLE	2300800	995930	3342
Q96EV2	744	RNA-binding protein 33	RBM33	0.923747	0.00049745	120.57	S	TPSAQVKPIVSASPPSRAVAGSRSSQGKTEV	0	0	4893
P46821	1396	Microtubule-associated protein 1B;MAP1B heavy chain;MAP1 lig	MAP1B	1	1.87E-08	120.54	S	DEPVPDSESPIEKVLSPLRSPPLIGSESAYE	16064000	5077000	1606
P46821	1400	Microtubule-associated protein 1B;MAP1B heavy chain;MAP1 lig	MAP1B	0.999998	1.87E-08	120.54	S	PDSESPIEKVLSPLRSPPLIGSESAYESFLS	16064000	5077000	1607
Q13613	652	Myotubularin-related protein 1	MTMR1	0.492504	0.000929242	120.53	S	REVATRAVSSSSERGSSPSHSATSVHTSV__	0	0	2601
Q13613	653	Myotubularin-related protein 1	MTMR1	0.696764	0.000929242	120.53	S	EVATRAVSSSSERGSSPSHSATSVHTSV___	0	0	2602
Q05655	647	Protein kinase C delta type;Protein kinase C delta type regu	PRKCD	0.920219	0.00391675	120.45	S	NFDQEFLNEKARLSYSDKNLIDSMDQSAFAG	0	0	2193
Q16513	958	Serine/threonine-protein kinase N2	PKN2	0.99996	1.41E-07	120.4	T	VSNFDDEFTSEAPILTPPREPRILSEEEQEM	0	550110	7657
Q96C36	304	Pyrroline-5-carboxylate reductase 2	PYCR2	0.989222	0.000757304	120.32	S	RVKLESPTVSTLTPSSPGKLLTRSLALGGKK	861810	572330	4820
Q9P1Y6	973	PHD and RING finger domain-containing protein 1	PHRF1	0.999703	4.81E-08	120.32	S	RTVTCVTVVEPEAPPSPDVLQAATHRVVELR	1350700	839420	6205
Q9H792	834	Pseudopodium-enriched atypical kinase 1	PEAK1	0.471837	1.85E-07	120.32	T	SLFTSQPSGEAEAPQTTDSPTTKVQKDPSIK	0	0	8110
Q5VSY0	25	G kinase-anchoring protein 1	GKAP1	0.585184	0.000287637	120.31	S	PTTASRFALLQVDSGSGSDSEPGKGKGRNTG	0	648830	3323
Q5VSY0	27	G kinase-anchoring protein 1	GKAP1	0.888965	0.000287637	120.31	S	TASRFALLQVDSGSGSDSEPGKGKGRNTGKS	1766800	648830	3324
Q5VSY0	29	G kinase-anchoring protein 1	GKAP1	0.842294	0.000287637	120.31	S	SRFALLQVDSGSGSDSEPGKGKGRNTGKSQT	936020	648830	3325
Q9HAW4	810	Claspin	CLSPN	0.999913	0.000876273	120.31	S	RGTSFFPTAGGFRSPSPGLFRASLVSSASKS	925670	530030	5827
Q9ULJ3	434	Zinc finger and BTB domain-containing protein 21	ZBTB21	0.929136	0.000947402	120.29	S	EGASPVTEVRIKTEPSSPLSDPSDIIRVTVG	0	0	6482
Q7Z4V5	454	Hepatoma-derived growth factor-related protein 2	HDGFRP2	1	0.000877931	120.27	S	KQQAKPVKVERTRKRSEGFSMDRKVEKKKEP	3078900	1957000	3832
Q9UPN9	1105	E3 ubiquitin-protein ligase TRIM33	TRIM33	0.987925	2.70E-10	120.27	S	PEFEQEEDDGEVTEDSDEDFIQPRRRKLKSD	8519100	5550200	6563
O15027	1907	Protein transport protein Sec16A	SEC16A	0.904448	4.24E-10	120.27	T	GVWHQDGALPQQCPGTPSSEMEQLDRPGLSQ	3300400	2158800	7139
P25054	780	Adenomatous polyposis coli protein	APC	1	8.27E-08	120.25	S	DAQHLSETFDNIDNLSPKASHRSKQRHKQSL	3953400	1506600	1258
Q92618	116	Zinc finger protein 516	ZNF516	0.764492	0.000596186	120.21	S	PLGEMRASEGLDACASPTKSASACNRLLNGA	0	0	4678
Q9NXV2	10	BTB/POZ domain-containing protein KCTD5	KCTD5	1	0.000147257	120.21	S	______MAENHCELLSPARGGIGAGLGGGLC	500730	442880	6082
Q96N67	180	Dedicator of cytokinesis protein 7	DOCK7	0.980342	0.000641505	120.06	S	DGNSYQDDQDDLKRRSMSIDDTPRGSWACSI	1724200	1696900	5036
Q9UQ35	2388	Serine/arginine repetitive matrix protein 2	SRRM2	0.999983	0.000888387	120.06	S	PALSGANLTSPRVPLSAYERVSGRTSPPLLD	753450	484040	6749
Q13459	1115	Unconventional myosin-IXb	MYO9B	0.948917	0.000514461	120.03	S	EPEVQPSDRSPLEHSSPEKEAPSPEKTLPPQ	825410	588980	2539
Q92934	118	Bcl2-associated agonist of cell death	BAD	1	0.000514461	120.03	S	NLWAAQRYGRELRRMSDEFVDSFKKGLPRPK	3096900	2021200	4733
Q96A00	26	Protein phosphatase 1 regulatory subunit 14A	PPP1R14A	1	0.000418924	120.03	S	LSKLQSPSRARGPGGSPGGLQKRHARVTVKY	4017900	3071100	4764
Q9HC35	150	Echinoderm microtubule-associated protein-like 4	EML4	0.823048	0.000645726	119.96	S	NDQSPQIRASPSPQPSSQPLQIHRQTPESKN	0	0	5840
Q9C0C2	672	182 kDa tankyrase-1-binding protein	TNKS1BP1	1	0.000611839	119.88	S	ETTQARTEAQDLCRASPEPPGPESSSRWLDD	3161600	3220800	5516
O15127	319	Secretory carrier-associated membrane protein 2	SCAMP2	0.803263	0.000612499	119.87	S	FSQGIFSSRTFHRAASSAAQGAFQGN______	812290	0	289
Q5JSH3	3	WD repeat-containing protein 44	WDR44	0.494931	2.47E-10	119.83	S	_____________MASESDTEEFYDAPEDVH	3842400	2411000	3179
Q8WWI1	867	LIM domain only protein 7	LMO7	0.919314	4.97E-08	119.79	S	KREDRVTTEIQLPSQSPVEEQSPASLSSLRS	1224200	0	4557
O00499	296	Myc box-dependent-interacting protein 1	BIN1	0.999998	0.000787098	119.77	S	VKAQPSDNAPAKGNKSPSPPDGSPAATPEIR	3798400	2573900	122
Q8TER5	266	Rho guanine nucleotide exchange factor 40	ARHGEF40	0.855955	0.000998309	119.77	S	PVRGSPTDAEGSPGLSRVRTVPTRKGAGGKG	0	0	4487
Q96T37	212	Putative RNA-binding protein 15	RBM15	0.970798	0.000616995	119.77	S	FGDVSVKISHLSGSGSGDERVAFVNFRRPED	0	0	5160
Q9Y608	332	Leucine-rich repeat flightless-interacting protein 2	LRRFIP2	0.400555	2.78E-05	119.76	S	TPLSGNSSRRGSGDTSSLIDPDTSLSELRDI	0	0	6973
Q9Y608	331	Leucine-rich repeat flightless-interacting protein 2	LRRFIP2	0.46303	2.78E-05	119.76	T	TTPLSGNSSRRGSGDTSSLIDPDTSLSELRD	0	0	8313
P18754	104	Regulator of chromosome condensation	RCC1	0.990494	0.000621058	119.69	S	YSFGCNDEGALGRDTSVEGSEMVPGKVELQE	1741500	1405500	1145
Q9H4L4	232	Sentrin-specific protease 3	SENP3	0.997059	4.49E-10	119.69	S	SGPPMEEDGLRWTPKSPLDPDSGLLSCTLPN	3494700	2105000	5677
Q9Y4U1	244	Methylmalonic aciduria and homocystinuria type C protein	MMACHC	0.498877	5.72E-10	119.69	S	QRLALLGLAQPSEKPSSPSPDLPFTTPAPKK	0	0	6938
Q9Y4U1	245	Methylmalonic aciduria and homocystinuria type C protein	MMACHC	0.701552	5.72E-10	119.69	S	RLALLGLAQPSEKPSSPSPDLPFTTPAPKKP	0	0	6939
Q6W2J9	1404	BCL-6 corepressor	BCOR	0.76128	0.00264104	119.68	S	DGKVTVRRFRKRPEPSSDYDLSPAKQEPKPF	425360	421610	3620
Q9NQX3	198	Gephyrin;Molybdopterin adenylyltransferase;Molybdopterin mol	GPHN	0.429865	2.30E-08	119.66	T	LEDLPSPPPPLSPPPTTSPHKQTEDKGVQCE	0	0	8149
Q9NQX3	199	Gephyrin;Molybdopterin adenylyltransferase;Molybdopterin mol	GPHN	0.429865	2.30E-08	119.66	T	EDLPSPPPPLSPPPTTSPHKQTEDKGVQCEE	0	0	8150
Q9Y6I3	470	Epsin-1	EPN1	0.996343	9.11E-05	119.66	T	PPPAATPTPTPPTRKTPESFLGPNAALVDLD	1512500	623770	8322
P02545	390	Prelamin-A/C;Lamin-A/C	LMNA	0.993031	0.00164367	119.62	S	AYRKLLEGEEERLRLSPSPTSQRSRGRASSH	16310000	12687000	861
P02545	395	Prelamin-A/C;Lamin-A/C	LMNA	0.509697	0.00164367	119.62	S	LEGEEERLRLSPSPTSQRSRGRASSHSSQTQ	0	11850000	863
Q86U42	150	Polyadenylate-binding protein 2	PABPN1	1	1.11E-05	119.62	S	KLKELQNEVEKQMNMSPPPGNAGPVIMSIEE	1240500	744650	3916
Q7Z417	631	Nuclear fragile X mental retardation-interacting protein 2	NUFIP2	0.459224	7.15E-06	119.62	T	SKDYEIESQNPLASPTNTLLGSAKEQRYQRG	0	0	7803
Q5T200	137	Zinc finger CCCH domain-containing protein 13	ZC3H13	1	0.00109882	119.56	S	REKEDIKITKERTPESEEENVEWETNRDDSD	669210	767230	3253
Q5T200	134	Zinc finger CCCH domain-containing protein 13	ZC3H13	1	0.00109882	119.56	T	GRHREKEDIKITKERTPESEEENVEWETNRD	669210	767230	7720
O15014	491	Zinc finger protein 609	ZNF609	1	0.000294675	119.54	S	PGTKVEPTVLDRNCPSPVLIDCPHPNCNKKY	3929400	2785200	233
Q6PKG0	624	La-related protein 1	LARP1	0.934838	0.00486022	119.51	T	FDEEMEQMDGRKNTFTAWSDEESDYEIDDRD	0	0	7765
O95817	381	BAG family molecular chaperone regulator 3	BAG3	0.51083	1.24E-07	119.48	S	VPPAPVPCPPPSPGPSAVPSSPKSVATEERA	5980900	0	830
Q92610	689	Zinc finger protein 592	ZNF592	0.276822	1.24E-07	119.48	S	APAPSSSPKHGLTSGSASPPPPALPLYPDPV	0	0	4657
Q92610	691	Zinc finger protein 592	ZNF592	0.281226	1.24E-07	119.48	S	APSSSPKHGLTSGSASPPPPALPLYPDPVRL	0	0	4658
Q9UJC3	235	Protein Hook homolog 1	HOOK1	0.997555	0.00273679	119.48	S	SENEMMNEKLDQLDGSFDDPNTVVAKKYFHA	0	441510	6394
Q86VM9	532	Zinc finger CCCH domain-containing protein 18	ZC3H18	1	0.000561735	119.45	S	DPWRRSKSPKKKLGVSVSPSRARRRRKTSAS	9120800	7656100	3950
Q05655	664	Protein kinase C delta type;Protein kinase C delta type regu	PRKCD	0.999993	3.66E-05	119.38	S	KNLIDSMDQSAFAGFSFVNPKFEHLLED___	675180	577450	2195
Q9H2J7	55	Sodium-dependent neutral amino acid transporter B(0)AT2	SLC6A15	0.999984	0.000164765	119.38	S	IVDGQEEKDTDVEEGSEVEDERPAWNSKLQY	4521500	4600900	5616
Q641Q2;Q[illegible]	619;619	WASH complex subunit FAM21A;WASH complex subunit FAM21C	FAM21A;FAM2	0.999735	4.99E-10	119.35	S	KASELSKKKASALLFSSDEEDQWNIPASQTH	5156200	2879600	3384
Q9BTC0	805	Death-inducer obliterator 1	DIDO1	0.999983	9.40E-08	119.34	S	KKTAPRQEAIPDLEDSPPVSDSEEQQESARA	4239500	6115500	5356
Q9BTC0	809	Death-inducer obliterator 1	DIDO1	0.867654	9.40E-08	119.34	S	PRQEAIPDLEDSPPVSDSEEQQESARAVPEK	0	6115500	5357
Q04637	1596	Eukaryotic translation initiation factor 4 gamma 1	EIF4G1	1	0.000684561	119.29	S	SVTAFFKWLREAEEESDHN______________	65561	39450	2156
O75928	499	E3 SUMO-protein ligase PIAS2	PIAS2	0.781938	0.00385042	119.27	S	PPAKRKCIFMSETQSSPTKGVLMYQPSSVRV	2881900	2123000	671
P12268	160	Inosine-5'-monophosphate dehydrogenase 2	IMPDH2	0.975322	0.000929933	119.22	S	TDTGRMGSRLVGIISSRDIDFLKEEEHDCFL	0	846210	1033
P28066	16	Proteasome subunit alpha type-5	PSMA5	0.999687	0.000540298	119.21	S	MFLTRSEYDRGVNTFSPEGRLFQVEYAIEAI	1176200	1460500	1302
Q2M2I8	624	AP2-associated protein kinase 1	AAK1	0.770968	0.00338485	119.21	S	PAVQGQKVGSLTPPSSPKTQRAGHRRILSDV	0	8921400	3042
Q6W2J9	422	BCL-6 corepressor	BCOR	0.784321	0.00178677	119.21	S	PGHARKTAVQDRKDGSSPPLLEKQTVTKDVT	1333600	0	3610
Q96JM3	445	Chromosome alignment-maintaining phosphoprotein 1	CHAMP1	0.918654	0.00221867	119.21	S	IRSPAGSPELRKPSGSPDLWKLSPDQRKTSP	4018300	32666	4975
P04920	147	Anion exchange protein 2	SLC4A2	0.437124	1.63E-08	119.18	S	SEAEGARALTQPSPVSTPSSVQFFLQEDDSA	0	0	889
Q9BTC0	1456	Death-inducer obliterator 1	DIDO1	1	1.62E-07	119.18	S	VDDLPNRMCADVRRNSVERPAEPVAGAATPS	3574900	1231600	5355
P04920	148	Anion exchange protein 2	SLC4A2	0.437124	1.63E-08	119.18	T	EAEGARALTQPSPVSTPSSVQFFLQEDDSAD	0	0	7259
P28066	56	Proteasome subunit alpha type-5	PSMA5	0.985358	0.000646158	119.17	S	QTSEGVCLAVEKRITSPLMEPSSIEKIVEID	1939400	1708200	1303
Q14160	1475	Protein scribble homolog	SCRIB	1	0.000436002	119.17	S	TAKAERRHQERLRVQSPEPPAPERALSPAEL	1872900	1739600	2690
Q969R5	689	Lethal(3)malignant brain tumor-like protein 2	L3MBTL2	0.596039	0.00104615	119.17	S	VKEEHLDVASPDKASSPELPVSVENIKQETD	2238900	1514300	4747
Q9GZT9	125	Egl nine homolog 1	EGLN1	1	0.00469102	119.04	S	GKVKAKPPADPAAAASPCRAAAGGQGSAVAA	4045500	2013100	5555
O60271	238	C-Jun-amino-terminal kinase-interacting protein 4	SPAG9	1	0.000939627	119.03	S	GGETPGSEQWKFQELSQPRSHTSLKVSNSPE	1116900	855630	457
Q13322	104	Growth factor receptor-bound protein 10	GRB10	1	0.000699236	119.03	S	QPRASGPPRSIQPQVSPRQRVQRSQPVHILA	1044700	0	2472
Q13523	277	Serine/threonine-protein kinase PRP4 homolog	PRPF4B	1	0.00141402	119.03	S	KVKIEDKSKSKDRKKSPIINESRSRDRGKKS	1050800	1267400	2554
Q8NDT2	267	Putative RNA-binding protein 15B	RBM15B	1	0.000438769	119.03	S	YLPLHGGYQYKQRSLSPVAAPPLREPRARHA	8667500	3813500	4358
Q9UQ35	778	Serine/arginine repetitive matrix protein 2	SRRM2	1	0.000453974	119.03	S	SSPRSKAKSRLSLRRSLSGSSPCPKQKSQTP	2516400	2775700	6692
Q9HAW0	385	Transcription factor IIIB 50 kDa subunit	BRF2	0.921047	1.65E-08	119.01	S	CPVPPVSTVTGDENISDSEIEQYLRTPQEVR	1625700	0	5821
P25116	418	Proteinase-activated receptor 1	F2R	0.998878	0.00376918	119	S	LMASKMDTCSSNLNNSIYKKLLT_________	746420	413310	1267
O95793	390	Double-stranded RNA-binding protein Staufen homolog 1	STAU1	0.993309	0.000622405	118.76	S	KKPGDGRKVTFFEPGSGDENGTSNKEDEFRM	1178900	807490	821
Q5BKY9	191	Protein FAM133B	FAM133B	0.920556	1.62E-07	118.76	S	GLSKKRKMYSEDKPLSSESLSESEYIEEVRA	4108400	3796400	3153
Q5BKY9	192	Protein FAM133B	FAM133B	0.920556	1.62E-07	118.76	S	LSKKRKMYSEDKPLSSESLSESEYIEEVRAK	1720200	793880	3154
Q7Z460	1196	CLIP-associating protein 1	CLASP1	1	0.000554463	118.76	S	SLRGVTEAIEKFSFRSQEDLNEPIKRDGKKE	569050	1265200	3812
Q9Y597	603	BTB/POZ domain-containing protein KCTD3	KCTD3	0.522464	0.00181073	118.76	T	TEEELLKLLDQCDLSTSRCATPNISPATSVV	1139000	0	8309
Q13428	1227	Treacle protein	TCOF1	0.750547	0.00108075	118.73	S	TPANSQASKATPKLDSSPSVSSTLAAKDDPD	0	0	2508
Q8IYB3	549	Serine/arginine repetitive matrix protein 1	SRRM1	1	0.00226398	118.73	S	RKRQKETSPRGRRRRSPSPPPTRRRRSPSPA	0	3081500	4160
Q8IYB3	551	Serine/arginine repetitive matrix protein 1	SRRM1	0.998824	0.00324361	118.73	S	RQKETSPRGRRRRSPSPPPTRRRRSPSPAPP	8246800	4691600	4161
Q9UQ35	2289	Serine/arginine repetitive matrix protein 2	SRRM2	0.990984	0.00272053	118.73	T	RTAVAPSAVNLADPRTPTAPAVNLAGARTPA	2853600	3992700	8276
O76021	358	Ribosomal L1 domain-containing protein 1	RSL1D1	0.5	0.000661259	118.71	T	HGKKKRGRGKAQVKATNESEDEIPQLVPIGK	11666000	5879700	7223
P23246	33	Splicing factor, proline- and glutamine-rich	SFPQ	1	0.000384399	118.61	S	RRGGGGRGGLHDFRSPPPGMGLNQNRGPMG	6319500	3760600	1220
Q15424;Q[illegible]	246;245	Scaffold attachment factor B1;Scaffold attachment factor B2	SAFB;SAFB2	0.746647	2.16E-08	118.59	S	KEESSELEQPFAQDTSSVGPDRKLAEEEDLF	7624300	359530	2922
Q8N3F8	578	MICAL-like protein 1	MICALL1	1	0.00109524	118.55	S	SGELVEPRVEQMPQASPGLAPRTRGSSGPQP	6571500	5359500	4257
Q9Y6A5	250	Transforming acidic coiled-coil-containing protein 3	TACC3	0.992567	1.87E-05	118.55	S	ECRHGGVCAPAAVATSPPGAIPKEACGGAPL	1148900	888010	6987
P15336	112	Cyclic AMP-dependent transcription factor ATF-2	ATF2	0.999889	0.00185144	118.52	S	KKASEDDIKKMPLDLSPLATPIIRSKIEEPS	367870	304810	1069
P61247	238	40S ribosomal protein S3a	RPS3A	0.757625	0.000677521	118.52	S	FELGKLMELHGEGSSSGKATGDETGAKVERA	1192000	803210	1911
Q8N1G0	1057	Zinc finger protein 687	ZNF687	1	0.000386562	118.52	S	EKHVQVRHGLQLGAQSPGRGTTLARGSSARA	2172900	2493100	4227
Q9NYZ3	461	G2 and S phase-expressed protein 1	GTSE1	0.969341	0.000677521	118.52	S	TPTNQFKIPKFSIGDSPDSSTPKLSRAQRPQ	1086800	0	6140
P46821	1298	Microtubule-associated protein 1B;MAP1B heavy chain;MAP1 lig	MAP1B	0.999645	3.47E-10	118.51	S	PNEIKVSAEAEVAPVSPEVTQEVVEEHCASP	7836900	7114800	1609
P46821	1312	Microtubule-associated protein 1B;MAP1B heavy chain;MAP1 lig	MAP1B	0.972647	3.47E-10	118.51	S	VSPEVTQEVVEEHCASPEDKTLEVVSPSQSV	7992900	5400000	1610
Q9Y618	1018	Nuclear receptor corepressor 2	NCOR2	0.724779	3.68E-12	118.47	S	QNLQPESDAPQQPGSSPRGKSRSPAPPADKE	3059900	1219300	6979
Q9UIS9	301	Methyl-CpG-binding domain protein 1	MBD1	0.4951	1.43E-07	118.46	T	AQPLPPPPPSQSPEPTEPHPRALAPSPPAEF	0	0	8224
Q9UKX7	330	Nuclear pore complex protein Nup50	NUP50	0.999995	1.69E-07	118.36	S	SPFPTKPLEGQAEGDSGECKGGDEEENDEPP	3080800	1673100	6461
P27348	232	14-3-3 protein theta	YWHAQ	0.945359	1.33E-07	118.32	S	IMQLLRDNLTLWTSDSAGEECDAAEGAEN__	7355600	0	1279
O15014	578	Zinc finger protein 609	ZNF609	0.9792	0.000326917	118.24	S	RSATPKVRLVEPHSPSPSSKFTKGLCKKKL	3416200	1235000	228
Q6PL24	14	Protein TMED8	TMED8	0.959412	0.000273286	118.24	S	__MSDLQAAEGPGSWSPTARPGSAGGVGDCQ	753410	910460	3572
O15020	2171	Spectrin beta chain, non-erythrocytic 2	SPTBN2	1	4.79E-06	118.23	S	QRLEHSSFPEGPGPGSGDEANGPRGERQTRT	0	1292000	237
Q8IXT5	638	RNA-binding protein 12B	RBM12B	0.997491	0.00402029	118.23	S	LEEDWRRPLEEDFRRSPTEDFRQLPEEDFRQ	172290	287560	4115
Q9H467	110	CUE domain-containing protein 2	CUEDC2	1	6.59E-10	118.22	S	NLQPQSSGVQGQVPISPEPLQRPEMLKEETR	4313000	3056500	5659
P28290	739	Sperm-specific antigen 2	SSFA2	0.990928	0.000692748	118.2	S	RYLFAKAGYPLRRSQSLPTTLLSPVRVVSSV	1065900	591110	1306
Q96B23	145	Uncharacterized protein C18orf25	C18orf25	0.986037	0.00312229	118.2	S	GVSGATVGRKSRSRSESETSTMAAKKNRQS	0	0	4795
Q13627	763	Dual specificity tyrosine-phosphorylation-regulated kinase 1	DYRK1A	0.621071	0.000392715	118.19	S	SPMTGVCVQQSPVASS_______________	0	1220600	2624
Q8IX21	21	Protein FAM178A	FAM178A	0.836505	0.00110707	118.16	S	MPARPGFPSSPAPGSSPPRCHLRPGSTAHAA	1147900	961720	4095

P35611	364	Alpha-adducin	ADD1	0.910959	0.00451374	118.16	T	YKAKSRSPGSPVGEGTGSPPKWQIGEQEFEA	0	0	7363
Q96AE4	630	Far upstream element-binding protein 1	FUBP1	0.857093	9.38E-08	118.15	S	WAEYYRQQAAYYAQTSPQGMPQHPPAPQGQ_	855350	198970	4773
Q06587	248	E3 ubiquitin-protein ligase RING1	RING1	0.999797	6.74E-12	118.09	S	GDRGGTLGGGTLGPPSPPGAPSPPEPGGEIE	7763200	6019100	2206
Q06587	254	E3 ubiquitin-protein ligase RING1	RING1	1	6.74E-12	118.09	S	LGGGTLGPPSPPGAPSPPEPGGEIELVFRPH	7763200	6835100	2207
Q9H7S9	252	Zinc finger protein 703	ZNF703	0.999978	0.000704735	118.08	S	VGGGELDKKDQEPKPSPEPAAVSRGGGGEPG	1519800	796590	5774
P15924	2606	Desmoplakin	DSP	0.307568	5.51E-08	118.05	S	SKISTISSVRNLTIRSSSFSDTLEESSPIAA	0	0	1078
P15924	2607	Desmoplakin	DSP	0.307568	5.51E-08	118.05	S	KISTISSVRNLTIRSSSFSDTLEESSPIAAI	0	0	1079
P15924	2608	Desmoplakin	DSP	0.307568	5.51E-08	118.05	S	ISTISSVRNLTIRSSSFSDTLEESSPIAAIF	0	0	1080
Q6W2J9	571	BCL-6 corepressor	BCOR	0.998936	0.000881951	118.05	S	NVSGSVSSAGRPASASPAPNANADGTKTSRS	0	651660	3618
Q96PU5	487	E3 ubiquitin-protein ligase NEDD4-like	NEDD4L	0.998262	1.02E-05	118.05	S	DTLSNPQSPQPSPYNSPKPQHKVTQSFLPPG	2148200	419320	5059
A7KAX9	856	Rho GTPase-activating protein 32	ARHGAP32	0.999999	7.21E-10	118.04	S	SQCQTPGSTASSEPVSPLQEKLSPFFTLDLS	1431800	908880	46
Q92576	1613	PHD finger protein 3	PHF3	0.905751	9.72E-06	118.02	S	QEDQENNLQDNQTSNSSPCRSNVGKGNIDGN	797610	621890	4642
Q9UNF1	190	Melanoma-associated antigen D2	MAGED2	0.801772	4.82E-06	118.02	S	KKARKVKHLDGEEDGSSDQSQASGTTGGRRV	0	0	6544
Q9Y4K4	335	Mitogen-activated protein kinase kinase kinase kinase 5	MAP4K5	0.998853	0.000759538	117.95	S	TIRSTNRNARAERTASEINFDKLQFEPPLRK	1349700	708100	6931
Q14839	1602	Chromodomain-helicase-DNA-binding protein 4	CHD4	1	4.85E-06	117.84	S	APETAIECTQAPAPASEDEKVVVEPPEGEEK	1587400	1608600	2818
Q9UK58	341	Cyclin-L1	CCNL1	0.580336	1.37E-07	117.83	S	PALSTLGGFSPASKPSSPREVKAEEKSPISI	14461000	8835000	6421
O60264	116	SWI/SNF-related matrix-associated actin-dependent regulator	SMARCA5	0.923269	0.00493244	117.81	S	LFAHFIQPAAQKTPTSPLKMKPGRPRIKKDE	5382200	2131300	452
Q08AD1	1019	Calmodulin-regulated spectrin-associated protein 2	CAMSAP2	0.999985	0.000464908	117.7	S	TPPRSVDSLPRLRRFSPSQVPIQTRSFVCFG	1169000	741220	2254
Q9C0C2	1138	182 kDa tankyrase-1-binding protein	TNKS1BP1	0.907254	0.000464908	117.7	S	QFGIIGNDRVSGAGFSPSSKMEGGHFVPPGK	861810	802210	5525
Q9Y385	266	Ubiquitin-conjugating enzyme E2 J1	UBE2J1	0.949267	0.00088349	117.65	S	SPRQRRAQQQSQRRLSTSPDVIQGHQPRDNH	0	0	6845
P43243	766	Matrin-3	MATR3	0.999992	1.24E-07	117.55	S	ADDPNKDTSENADGQSDENKDDYTIPDEYRI	6741500	7083800	1522
O95674	21	Phosphatidate cytidylyltransferase 2	CDS2	0.991364	0.000101821	117.53	S	QRVAHEPVAPPEDKESESEAKVDGETASDSE	3145500	2198300	792
Q13427	687	Peptidyl-prolyl cis-trans isomerase G	PPIG	0.999995	0.00497288	117.53	S	NSSNNSREKKADRDQSPFSKIKQSSQDNELK	3658900	5518200	2494
Q8IY67	14	Ribonucleoprotein PTB-binding 1	RAVER1	0.999997	0.000951673	117.53	S	__MAADVSVTHRPPLSPKSGAEVEAGDAAER	19366000	8946400	4119
O14924	850	Regulator of G-protein signaling 12	RGS12	0.923893	0.00115806	117.48	S	EVEGRALPDSQQVPSSPASKHSLGSDHSSVS	1589200	0	218
Q5T1M5	1097	FK506-binding protein 15	FKBP15	0.906114	3.82E-10	117.43	S	EVAPDGPLQESSTRLSLTSDPEEGDPLALGP	1684200	3027400	3245
Q5T1M5	1099	FK506-binding protein 15	FKBP15	0.666667	3.82E-10	117.43	T	APDGPLQESSTRLSLTSDPEEGDPLALGPES	0	2105900	7719
Q9UBF8	263	Phosphatidylinositol 4-kinase beta	PI4KB	0.397731	0.00274223	117.4	T	AHRKRELPSLSPAPDTGLSPSKRTHQRSKSD	0	0	8195
Q6PL24	63	Protein TMED8	TMED8	0.848025	7.09E-10	117.38	S	DTSLLASATDPEPCSSPHRPQMVSPVSKDAT	0	2079100	3569
O00567	467	Nucleolar protein 56	NOP56	0.705609	2.86E-09	117.35	S	KRLAALALASSENSSSTPEECEEMSEKPKKK	1311900	1626600	137
O00567	468	Nucleolar protein 56	NOP56	0.915349	2.86E-09	117.35	T	RLAALALASSENSSSTPEECEEMSEKPKKKK	1311900	1626600	7123
Q9H6F5	58	Coiled-coil domain-containing protein 86	CCDC86	1	0.00213412	117.27	S	REPGSPPSVQRAGLGSPERPPKTSPGSPRLQ	11606000	6830400	5725
Q5JTV8	220	Torsin-1A-interacting protein 1	TOR1AIP1	0.99868	9.06E-08	117.23	T	ATSVQQKVNFSEEGETEEDDQDSSHSSVTTV	0	0	7707
Q5TKA1	321	Protein lin-9 homolog	LIN9	1	1.06E-07	117.22	S	PLQSPIIDNDPLLGQSPWRSKISGSDTETLG	1026800	663420	3311
Q96E09	76	Protein FAM122A	FAM122A	1	0.000474854	117.2	S	NSTTFPSRHGLLLPASPVRMHSSRLHQIKQE	1155400	1108900	4867
Q96T58	2124	Msx2-interacting protein	SPEN	0.87445	0.00531727	117.2	S	AGERESGVVAVSPEKSESPQKEDGLSSQLKS	886510	0	5169
Q9HCK8	1420	Chromodomain-helicase-DNA-binding protein 8	CHD8	1	0.00010122	117.2	S	TRHFSTLKDDDLVEFSDLESEDDERPRSRRH	1303200	1815900	5862
Q9HCK8	1424	Chromodomain-helicase-DNA-binding protein 8	CHD8	1	0.00010122	117.2	S	STLKDDDLVEFSDLESEDDERPRSRRHDRHH	1303200	1815900	5863
Q05655	645	Protein kinase C delta type;Protein kinase C delta type regu	PRKCD	0.581168	0.00465782	117.14	S	YSNFDQEFLNEKARLSYSDKNLIDSMDQSAF	1793800	0	2192
Q9H2H9	52	Sodium-coupled neutral amino acid transporter 1	SLC38A1	0.885598	0.000804935	117.14	S	GQINSKFISDRESRRSLTNSHLEKKKCDEYI	272010	6083800	5614
Q9NVI1	407	Fanconi anemia group I protein	FANCI	0.889799	0.000605707	117.14	S	SYGPKKVLDGKTIETSPSLSRMPNQHACKLG	2130300	884140	6043
P61247	263	40S ribosomal protein S3a	RPS3A	1	0.000421499	117.07	S	AKVERADGYEPPVQESV______________	0	4010000	1910
Q15596	565	Nuclear receptor coactivator 2	NCOA2	1	0.000207199	117.07	S	SSLASPDLKMGNLQNSPVNMNPPPLSKMGSL	867380	1021800	2948
O14497	363	AT-rich interactive domain-containing protein 1A	ARID1A	0.998611	1.97E-05	117.02	S	AASGGAQQRSHHAPMSPGSSGGGGQPLARTP	3542900	2344200	146
Q0JRZ9	394	F-BAR domain only protein 2	FCHO2	1	0.00103906	117.02	S	VSIGNITLSPAISRHSPVQMNRNLSNEELTK	4147600	2319800	2283
Q92667	150	A-kinase anchor protein 1, mitochondrial	AKAP1	0.805966	0.000422704	117.02	S	LTGGEAKSIPLECPLSSPKGVLFSSKSAEVC	2155800	1743500	4686
Q96D71	162	RalBP1-associated Eps domain-containing protein 1	REPS1	0.993066	2.19E-09	117.02	S	DTVQPRTSADAQEPASPVVSPQQSPPTSPHT	5609400	2746500	4858
Q9H4L4	212	Sentrin-specific protease 3	SENP3	0.999991	0.000687484	117	S	LGLLGALMAEDGVRGSPPVPSGPPMEEDGLR	2310300	1513100	5674
P35612	532	Beta-adducin	ADD2	0.908989	0.000479178	116.98	S	SQLLASVIAEKSRSPSTESQLMSKGDEDTKD	454570	415920	1414
Q96JM3	284	Chromosome alignment-maintaining phosphoprotein 1	CHAMP1	0.870684	1.11E-05	116.98	S	KSARTTSPEPRKPSPSESPEPWKPFPAVSPE	0	0	4977
Q06124	562	Tyrosine-protein phosphatase non-receptor type 11	PTPN11	0.961275	7.92E-10	116.94	S	TNIKYSLADQTSGDQSPLPPCTPTPPCAEMR	3426500	2553800	2197
Q8TEK3	448	Histone-lysine N-methyltransferase, H3 lysine-79 specific	DOT1L	0.890263	1.86E-08	116.94	S	LDALHAQTVSQTAASSPQDAYRSPHSPFYQL	1892400	1616500	4478
Q9H2Y7	1328	Zinc finger protein 106	ZNF106	1	1.44E-07	116.94	S	RLSFASETPLEKEPHSPADQPEQQAESTLTS	4109900	1953500	5628
Q5XKK7	125	Protein FAM219B	FAM219B	0.998828	8.52E-10	116.92	S	TALSQSPDENLVSLDSDSDGELGSRYSSGYS	1835100	1001800	3377
Q5XKK7	127	Protein FAM219B	FAM219B	0.997735	8.52E-10	116.92	S	LSQSPDENLVSLDSDSDGELGSRYSSGYSSA	1835100	1001800	3378
P30566	9	Adenylosuccinate lyase	ADSL	0.999812	0.000321708	116.9	S	_______MAAGGDHGSPDSYRSPLASRYASP	1353900	892000	1346
Q96IY1	4	Kinetochore-associated protein NSL1 homolog	NSL1	1	0.000512742	116.87	S	____________MAGSPELVVLDPPWDKELA	1394300	1433900	4953
P41252	1049	Isoleucine--tRNA ligase, cytoplasmic	IARS	0.957615	0.000480155	116.84	S	FTTIKAPLKPYPVSPSDKVLIQEKTQLKGSE	483400	0	1489
Q9BXJ9	855	N-alpha-acetyltransferase 15, NatA auxiliary subunit	NAA15	0.836426	0.000351228	116.84	S	PPGYEEDMKITVNGDSSAEAEELANEI____	0	0	5451
O75376	1196	Nuclear receptor corepressor 1	NCOR1	0.791929	0.000618271	116.74	S	ALVKGSISRMPIEDSSPEKGREEAASKGHVI	0	0	603
Q96RK0	1373	Protein capicua homolog	CIC	0.97652	7.01E-08	116.74	S	RFAELPEFRPEEVLPSPTLQSLATSPRAILG	0	2682400	5091
Q96RK0	1381	Protein capicua homolog	CIC	0.764029	7.01E-08	116.74	T	RPEEVLPSPTLQSLATSPRAILGSYRKKRKN	0	0	8003
Q7Z5L9	409	Interferon regulatory factor 2-binding protein 2	IRF2BP2	0.621126	1.45E-07	116.73	S	TSSFVSPPPPTASPHSNRTTPPEAAQNGQSP	0	0	3849
Q6UUV9	167	CREB-regulated transcription coactivator 1	CRTC1	0.327087	2.51E-10	116.72	S	DSALHQSTMTPTQPESFSSGSQDVHQKRVLL	0	0	3595
Q96AY4	2104	Tetratricopeptide repeat protein 28	TTC28	0.991373	0.000402718	116.71	S	TAGGMRVSVSSKGSISTPNSPVKMTLIPSPN	0	0	4784
Q96AY4	2108	Tetratricopeptide repeat protein 28	TTC28	0.996575	0.000402718	116.71	S	MRVSVSSKGSISTPNSPVKMTLIPSPNSPFQ	0	0	4785
P12956	455	X-ray repair cross-complementing protein 6	XRCC6	1	0.00252382	116.71	T	ADDKRKMPFTEKIMATPEQVGKMKAIVEKLR	999700	814850	7282
Q5UIP0	1556	Telomere-associated protein RIF1	RIF1	0.476053	0.000423098	116.69	S	ASQGLLSSIENSESDSSEAKEEGSRKKRSGK	0	0	3313
O95817	284	BAG family molecular chaperone regulator 3	BAG3	0.620387	0.000359168	116.63	S	SVQGASSREGSPARSSTPLHSPSPIRVHTVV	1338200	410520	826
Q8WXG6	1059	MAP kinase-activating death domain protein	MADD	0.891989	0.00043207	116.63	S	AQTHYYSKEPDKRKRSPTESVNTPVGKDPGL	801880	0	4594
Q15678	593	Tyrosine-protein phosphatase non-receptor type 14	PTPN14	0.988495	0.00036036	116.58	S	STPDLASHRHKYVSGSSPDLVTRKVQLSVKT	3091500	0	2976
Q6MZP7	310	Protein lin-54 homolog	LIN54	1	0.00187389	116.58	S	STLNSTTQTPNKIAISPLKSPNKAVKSTVQT	7295100	3151000	3492
Q6MZP7	314	Protein lin-54 homolog	LIN54	1	0.00187389	116.58	S	STTQTPNKIAISPLKSPNKAVKSTVQTITVG	7295100	3151000	3493
Q99607	188	ETS-related transcription factor Elf-4	ELF4	0.939782	0.000774216	116.51	S	KSKKRIRKTKGNRSTSPVTDPSIPIRKKSKD	2702000	0	5230
O95104	154	Splicing factor, arginine/serine-rich 15	SCAF4	0.998029	4.65E-15	116.49	S	SNAAPVAENVTNNEGSPPPPVKVSSEPPTQA	1745100	1321000	729
Q92945	125	Far upstream element-binding protein 2	KHSRP	0.999846	2.00E-05	116.48	S	RQLEDGDQPESKKLASQGDSISSQLGPIHPP	0	1037400	4735
Q13541	83	Eukaryotic translation initiation factor 4E-binding protein	EIF4EBP1	0.927554	2.99E-08	116.45	S	TKTPPRDLPTIPGVTSPSSDEPPMEASQSHL	8897000	12094000	2571
Q6VMQ6	113	Activating transcription factor 7-interacting protein 1	ATF7IP	0.99986	5.09E-06	116.45	S	NVKNKQDDDLNCEPLSPHNITPEPVSKLPAE	1993200	0	3603
Q13541	82	Eukaryotic translation initiation factor 4E-binding protein	EIF4EBP1	0.495431	2.99E-08	116.45	T	VTKTPPRDLPTIPGVTSPSSDEPPMEASQSH	0	0	7551
P08651	333	Nuclear factor 1 C-type	NFIC	0.999711	0.000267088	116.43	S	EGGISSPVKKTEMDKSPFNSPSPQDSPRLSS	1929200	1220000	961
P08651	343	Nuclear factor 1 C-type	NFIC	0.999515	0.000267088	116.43	S	TEMDKSPFNSPSPQDSPRLSSFTQHHRPVIA	972620	394510	963
Q9UGV2	352	Protein NDRG3	NDRG3	0.526439	5.12E-06	116.27	S	GSGESPFSRSVTSNQSDGTQESCESPDVLDR	0	2406600	6320
Q9NZT2	539	Opioid growth factor receptor	OGFR	0.638513	3.74E-12	116.24	S	PSPAGPAGDEPAESPSETPGPRPAGPAGDEP	6420500	1363100	6171
Q9UQ35	2030	Serine/arginine repetitive matrix protein 2	SRRM2	1	0.00539784	116.19	S	RRRSRSRTPPAIRRRSRSRTPLLPRKRSRSR	7360800	4341500	6716
Q9UQ35	2032	Serine/arginine repetitive matrix protein 2	SRRM2	1	0.00539784	116.19	S	RSRSRTPPAIRRRSRSRTPLLPRKRSRSRSP	79079000	41101000	6717
Q9BXJ9	850	N-alpha-acetyltransferase 15, NatA auxiliary subunit	NAA15	0.929621	0.00339814	116.19	T	ALAFMPPGYEEDMKITVNGDSSAEAEELANE	922180	1435400	8064
Q9UQ35	2034	Serine/arginine repetitive matrix protein 2	SRRM2	1	0.00539784	116.19	T	RSRTPPAIRRRSRSRTPLLPRKRSRSRSPLA	79079000	41101000	8270
P25440	298	Bromodomain-containing protein 2	BRD2	0.999978	5.78E-10	116.08	S	ADTTTPTPTAILAPGSPASPPGSLEPKAARL	8554900	6621300	1269
P25440	301	Bromodomain-containing protein 2	BRD2	0.991878	5.78E-10	116.08	S	TTPTPTAILAPGSPASPPGSLEPKAARLPPM	7227800	5786500	1270
Q13541	45	Eukaryotic translation initiation factor 4E-binding protein	EIF4EBP1	0.601542	1.23E-11	116.08	T	PPGDYSTTPGGTLFSTTPGGTRIIYDRKFLM	0	0	7555
P13639	435	Elongation factor 2	EEF2	0.993054	0.00260891	116.05	T	LVSTGLKVRIMGPNYTPGKKEDLYLKPIQRT	3046000	2256300	7284
Q9UQ35	1264	Serine/arginine repetitive matrix protein 2	SRRM2	0.891714	0.000992079	116.04	S	AGQILSHLSSELKEMSTSNFESSPEVEERPA	828930	610380	6620
Q96S94	369	Cyclin-L2	CCNL2	1	0.000644155	115.91	S	NTKRRLEGAKKAKADSPVNGLPKGRESRSRS	1528100	1336800	5117
Q9NQS7	263	Inner centromere protein	INCENP	1	0.000873136	115.91	S	VGTGRSASKLRIAQVSPGPRDSPAFPDSPWR	3226400	3661500	5923
P28370	118	Probable global transcription activator SNF2L1	SMARCA1	0.81799	0.00679223	115.91	T	ELFAHFIQPSAQKSPTSPLNMKLGRPRIKKD	0	0	7341
Q8IX21	20	Protein FAM178A	FAM178A	0.973853	0.00128271	115.83	S	CMPARPGFPSSPAPGSSPPRCHLRPGSTAHA	0	0	4094
P46821	1067	Microtubule-associated protein 1B;MAP1B heavy chain;MAP1 lig	MAP1B	0.766936	0.0057954	115.82	T	AAEAGGAEEQYGFLTTPTKQLGAQSPGREPA	289220	323670	7387
P35611	366	Alpha-adducin	ADD1	0.91231	0.00114427	115.8	S	AKSRSPGSPVGEGTGSPPKWQIGEQEFEALM	1015300	342570	1402
Q86WB0	357	Nuclear-interacting partner of ALK	ZC3HC1	0.726635	9.81E-05	115.77	S	EKSPGPIVSRTRSWDSSSPVDRPEPEAASPT	599090	288480	3986
Q9NRA8	301	Eukaryotic translation initiation factor 4E transporter	EIF4ENIF1	1	5.20E-06	115.74	S	AADQEVPRDAVLPEQSPGDFDFNEFFNLDKV	514530	475870	5952
Q9UKV3	408	Apoptotic chromatin condensation inducer in the nucleus	ACIN1	0.547866	2.40E-07	115.74	T	TEDRKKASLVALPEQTASEEETPPPLLTKEA	0	4883900	8232
Q96AY2	87	Crossover junction endonuclease EME1	EME1	0.488651	0.000446964	115.73	S	ETVTQTQPVRLLSSESEDEEEFIPLAQRLTC	0	0	4779
Q9C0C2	695	182 kDa tankyrase-1-binding protein	TNKS1BP1	0.656502	0.000734134	115.73	S	SSSRWLDDLLASPPPSGGGARRGAGAELKDT	0	0	5528
Q9H4G0	685	Band 4.1-like protein 1	EPB41L1	0.5	0.00129355	115.68	S	DESGGIEDSPDRGACSTPDMPQFEPVKTETM	902520	684810	5670
Q9H4G0	686	Band 4.1-like protein 1	EPB41L1	0.5	0.00129355	115.68	T	ESGGIEDSPDRGACSTPDMPQFEPVKTETMT	902520	684810	8101
Q92890	247	Ubiquitin fusion degradation protein 1 homolog	UFD1L	1	0.00583733	115.66	S	GNRLDGKKKGVEPSPSPIKPGDIKRGIPNYE	2977300	1563700	4726
P49327	207	Fatty acid synthase;[Acyl-carrier-protein] S-acetyltransfera	FASN	0.999879	0.000506941	115.57	S	LKPNTSVQFLRLGMLSPEGTCKAFDTAGNGY	491250	458540	1653
P06744	109	Glucose-6-phosphate isomerase	GPI	0.999818	0.00522841	115.57	T	EGRAVLHVALRNRSNTPILVDGKDVMPEVNK	3092900	1321700	7264
Q3YEC7	640	Rab-like protein 6	RABL6	1	1.92E-07	115.56	S	SDLFGLGLEEAGPKESSEEGKEGKTPSKEKK	4512800	5145500	3085
Q3YEC7	641	Rab-like protein 6	RABL6	1	1.92E-07	115.56	S	DLFGLGLEEAGPKESSEEGKEGKTPSKEKKK	6164200	4044200	3086
Q12830	1251	Nucleosome-remodeling factor subunit BPTF	BPTF	1	6.42E-10	115.55	S	KAKEGCQSDSMRQEQSPNANNDQPEDLIQGC	2240800	2037300	2314
P46100	598	Transcriptional regulator ATRX	ATRX	0.973206	0.000462588	115.37	S	TKELYVKLTPVSLSNSPIKGADCQEVPQDKD	0	4232100	1554
P61326;Q9	106;108	Protein mago nashi homolog;Protein mago nashi homolog 2	MAGOH;MAGOI	1	0.000510927	115.37	S	VIGDEHISFTTSKIGSLIDVNQSKDPEGLRV	834610	559390	1912
Q68EM7	674	Rho GTPase-activating protein 17	ARHGAP17	0.840976	2.92E-08	115.36	S	TSQHPPSLSPKPPTRSPSPPTQHTGQPPGQP	23431000	10470000	3429
Q8NHM5	975	Lysine-specific demethylase 2B	KDM2B	1	7.92E-08	115.36	S	RTENSLANENQQPIKSEPESEGEEPKRPPGI	2078600	1447600	4415
Q8NHM5	979	Lysine-specific demethylase 2B	KDM2B	1	7.92E-08	115.36	S	SLANENQQPIKSEPESEGEEPKRPPGICERP	2078600	1447600	4416
P51610	413	Host cell factor 1;HCF N-terminal chain 1;HCF N-terminal cha	HCFC1	0.885786	1.40E-09	115.36	T	QKYDIPATAATATSPTPNPVPSVPANPPKSP	0	0	7441
Q68EM7	682	Rho GTPase-activating protein 17	ARHGAP17	0.562308	2.92E-08	115.36	T	SPKPPTRSPSPPTQHTGQPPGQPSAPSQLSA	0	0	7746
Q9P1Y5	368	Calmodulin-regulated spectrin-associated protein 3	CAMSAP3	1	0.000669404	115.34	S	VFTFRHPLLSSGGPQSPLRGSTGSLKSSPSM	1002100	510410	6197
O15027	1356	Protein transport protein Sec16A	SEC16A	1	0.00187706	115.24	S	EEVDRRSVHSEHSARSLHSAHSLASRRSSLS	3581800	1404800	250
O15027	1359	Protein transport protein Sec16A	SEC16A	0.999998	0.00187706	115.24	S	DRRSVHSEHSARSLHSAHSLASRRSSLSSHS	3581800	1404800	251
O15027	1362	Protein transport protein Sec16A	SEC16A	0.999957	0.00187706	115.24	S	SVHSEHSARSLHSAHSLASRRSSLSSHSHQS	1058700	974270	252
Q03252	37	Lamin-B2	LMNB2	0.99578	0.00362339	115.24	S	ATPLPGRAGGPATPLSPTRLSRLQEKEELRE	5322600	3021300	2150
Q5T200	241	Zinc finger CCCH domain-containing protein 13	ZC3H13	0.958256	0.000835432	115.24	S	KSSSASKKDRKTSAVSSPLLDQQRNSKTNQS	2116000	1212000	3276
Q6GYQ0	860	Ral GTPase-activating protein subunit alpha-1	RALGAPA1	0.998419	0.00222516	115.24	S	ERLRSGNASTMTRRGSSPGSLEIPKDLPDIL	3228900	1174700	3459
Q6GYQ0	864	Ral GTPase-activating protein subunit alpha-1	RALGAPA1	0.97712	0.00222516	115.24	S	SGNASTMTRRGSSPGSLEIPKDLPDILNKQN	2114600	1174700	3461
Q9P1Y5	362	Calmodulin-regulated spectrin-associated protein 3	CAMSAP3	0.878485	0.00135662	115.24	S	SGSSSPVFTFRHPLLSSGGPQSPLRGSTGSL	0	0	6196
Q03252	34	Lamin-B2	LMNB2	1	0.00362339	115.24	T	ATMATPLPGRAGGPATPLSPTRLSRLQEKEE	2787600	2815300	7489
Q9BQA1	5	Methylosome protein 50	WDR77	1	0.00447306	115.24	T	___________MRKETPPPLVPPAAREWNLP	2917300	1356800	8040
Q9H4L4	307	Sentrin-specific protease 3	SENP3	1	0.00112782	115.23	S	MAEEAERPGEKAGQHSPLREEHVTCVQSILD	2158600	1631000	5673
Q9UQ35	449	Serine/arginine repetitive matrix protein 2	SRRM2	1	0.000188264	115.2	S	ASSSPESPKPAPAPGSHREISSSPTSKNRSH	6212400	2191400	6639
Q9HD15	68	Steroid receptor RNA activator 1	SRA1	0.517037	2.03E-05	115.15	T	VAAPQDGSPRVPASETSPGPPPMGPPPPSSK	1800700	0	8135
Q9UBU7	381	Protein DBF4 homolog A	DBF4	1	0.000916613	115.14	S	LKKTEQKEKVELQHISQKDCQEDDTTVKEQN	782560	948360	6274
Q7Z569	117	BRCA1-associated protein	BRAP	0.969997	0.000468804	115.12	S	RSKDHSKECINAAPDSPSKQLPDQISFFSGN	2303500	2395200	3833
Q9H4L5	437	Oxysterol-binding protein-related protein 3	OSBPL3	0.999933	0.000516003	115.12	S	EENSRDENRALVHQLSNESRLSITDSLSEFF	6545000	1392500	5678
Q8TAQ2	283	SWI/SNF complex subunit SMARCC2	SMARCC2	0.998951	0.00175321	115.09	S	RRKKISAKTLTDEVNSPDSDRRDKKGGNYKK	0	1211300	4446
Q8WUM0	27	Nuclear pore complex protein Nup133	NUP133	1	0.00655465	115.09	S	GTGSRRGPLAGLGPGSTPRTASRKGLPLGSA	958090	671220	4521
Q9UEG4	52	Zinc finger protein 629	ZNF629	0.955987	4.22E-05	115.09	S	QESSGEEIIMGDPAQSPESKDSTEMSLERSS	0	505150	6285
Q8WUM0	28	Nuclear pore complex protein Nup133	NUP133	1	0.00655465	115.09	T	TGSRRGPLAGLGPGSTPRTASRKGLPLGSAV	958090	671220	7915

Q9NZT2	525	Opioid growth factor receptor	OGFR	0.865094	5.51E-12	115.06	S	PKEGTPGSPSETPGPSPAGPAGDEPAESPSE	996990	936650	6174
Q9NZT2	514	Opioid growth factor receptor	OGFR	0.449285	5.51E-12	115.06	T	GVEEDTEGRTGPKEGTPGSPSETPGPSPAGP	0	0	8184
Q9H6Z4	125	Ran-binding protein 3	RANBP3	0.377176	0.011346	115.04	S	REDGNYCPPVKRERTSSLTQFPPSQSEERSS	0	0	5745
Q9H6Z4	124	Ran-binding protein 3	RANBP3	0.377176	0.011346	115.04	T	DREDGNYCPPVKRERTSSLTQFPPSQSEERS	0	0	8107
Q9UHB7	674	AF4/FMR2 family member 4	AFF4	0.997181	0.000897327	115.04	T	LPPSSQTPKYPESNRTPVKPSSVEEEDSFFR	1709300	873510	8213
Q9Y3Z3	33	Deoxynucleoside triphosphate triphosphohydrolase SAMHD1	SAMHD1	0.999391	1.58E-07	115.02	S	SPRTPSNTPSAEADWSPGLELHPDYKTWGPE	3759300	2572000	6887
Q8N556	687	Actin filament-associated protein 1	AFAP1	0.411767	6.67E-10	115.01	S	RHRTLENSPISSCDTSDTEGPVPVNSAAVLK	0	0	4279
Q15311	9	RalA-binding protein 1	RALBP1	0.944755	0.000529403	115	T	________MTECFLPPTSSPSEHRRVEHGSGL	1432700	695020	7635
Q99504	268	Eyes absent homolog 3	EYA3	0.493915	3.56E-08	114.92	T	RLSSGDPSTSPSLSQTTPSKDTDDQSRKNMT	0	0	8024
P30622	147	CAP-Gly domain-containing linker protein 1	CLIP1	0.869557	7.54E-12	114.91	S	DEANGLQTTPASRATSPLCTSTASMVSSSPS	1874200	1920600	1347
Q00613	292	Heat shock factor protein 1	HSF1	0.857409	0.000930162	114.89	S	MASPGGSIDERPLSSSPLVRVKEEPPSPPQS	864010	629060	2033
Q13459	1114	Unconventional myosin-IXb	MYO9B	0.607851	0.000676304	114.89	S	SEPEVQPSDRSPLEHSSPEKEAPSPEKTLPP	0	588980	2538
Q14839	1537	Chromodomain-helicase-DNA-binding protein 4	CHD4	0.974837	0.00246733	114.89	S	AEVEENKKMSQPGSPSPKTPTPSTPGDTQPN	0	1770000	2817
Q99728	330	BRCA1-associated RING domain protein 1	BARD1	0.999937	0.00534584	114.89	S	PLENNGKRGHHNRLSSPISKRCRTSILSTSG	0	414460	5264
O43395	131	U4/U6 small nuclear ribonucleoprotein Prp3	PRPF3	0.795392	1.11E-07	114.88	S	FEEVEEEPEVIPGPPSESPGMLTKLQIKQMM	0	0	375
O60271	593	C-Jun-amino-terminal kinase-interacting protein 4	SPAG9	0.607953	0.000520886	114.87	S	YNAPTSHVTPSVKKRSSTLSQLPGDKSKAFD	1898600	0	464
O60271	594	C-Jun-amino-terminal kinase-interacting protein 4	SPAG9	0.459008	0.000520886	114.87	S	NAPTSHVTPSVKKRSSTLSQLPGDKSKAFDF	0	0	465
Q8N2M8	294	CLK4-associating serine/arginine rich protein	CLASRP	0.984277	0.00278051	114.87	S	LRGRKISPPSYARRDSPTYDPYKRSPSESSS	1811400	546030	4242
Q9Y2V2	41	Calcium-regulated heat stable protein 1	CARHSP1	0.999968	0.000520886	114.87	S	SRERSPSPLRGNVVPSPLPTRRTRTFSATVR	21874000	13121000	6812
P25098	670	Beta-adrenergic receptor kinase 1	ADRBK1	1	0.00114635	114.86	S	QQLVQRVPKMKNKPRSPVVELSKVPLVQRGS	848720	715740	1263
Q14202	1049	Zinc finger MYM-type protein 3	ZMYM3	0.996685	0.000931916	114.86	S	TMRKGQKRLVLSESCSRDSMSSQPSCTGLNY	1110600	576800	2714
Q99618	29	Cell division cycle-associated protein 3	CDCA3	0.985067	0.00114635	114.86	S	PPPHNKHLARVADPRSPSAGILRTPIQVESS	1262900	575660	5232
Q9UQ35	1444	Serine/arginine repetitive matrix protein 2	SRRM2	0.992575	0.00114635	114.86	S	DGLPRTPSRRSRSGSSPGLRDGSGTPSRHSL	834700	569260	6686
P04792	82	Heat shock protein beta-1	HSPB1	0.999544	0.000680396	114.76	S	AVAAPAYSRALSRQLSSGVSEIRHTADRWRV	12849000	5006400	887
P50851	10	Lipopolysaccharide-responsive and beige-like anchor protein	LRBA	0.999996	0.000238407	114.76	S	______MASEDNRVPSPPPTGDDGGGGGREE	3798900	2600000	1763
P78332	1025	RNA-binding protein 6	RBM6	0.999913	0.000937421	114.76	S	KGRGNDRREKLQSFDSPERKRIKYSRETDSD	3311000	2810000	1958
Q9NYV4	343	Cyclin-dependent kinase 12	CDK12	0.999984	0.002618	114.76	S	GRRRSSSPFLSKRSLSRSPLPSRKSMKSRSR	10836000	7201300	6137
Q9NYV4	345	Cyclin-dependent kinase 12	CDK12	0.998074	0.002618	114.76	S	RRSSSPFLSKRSLSRSPLPSRKSMKSRSRSP	10836000	7201300	6138
P27694	38	Replication protein A 70 kDa DNA-binding subunit;Replicatior	RPA1	0.911502	0.000682089	114.71	S	PILQVINIRPITTGNSPPRYRLLMSDGLNTL	199550	895560	1283
P55196	216	Afadin	MLLT4	0.997854	3.99E-05	114.71	S	EVYKDMPETSFTRTISNPEVVMKRRRQQKLE	2311500	644740	1872
Q14498	97	RNA-binding protein 39	RBM39	0.995409	0.00423859	114.71	S	SRSRSRDRRFRGRYRSPYSGPKFNSAIRGKI	1141400	598200	2741
Q8IWS0	145	PHD finger protein 6	PHF6	0.999921	1.20E-07	114.71	S	KHKKTAHNSEADLEESFNEHELEPSSPKSKK	2536800	2477000	4074
P01106;P(	62;62	Myc proto-oncogene protein;N-myc proto-oncogene protein	MYC;MYCN	0.995806	0.000861234	114.7	S	EDIWKKFELLPTPPLSPSRRSGLCSPSYVAV	1495600	1219200	854
Q9UN30	152	Sex comb on midleg-like protein 1	SCML1	0.824218	0.0162374	114.7	S	YSPTLPVSRRENNSPSNLPRPSFCMEEYQRA	0	204860	6526
O14686	468	Histone-lysine N-methyltransferase 2D	KMT2D	1	1.41E-05	114.64	S	PEESPTSPPPEASRLSPPPEELPASPLPEAL	7355300	5270200	196
O14686	477	Histone-lysine N-methyltransferase 2D	KMT2D	1	1.41E-05	114.64	S	PEASRLSPPPEELPASPLPEALHLSRPLEES	7355300	5270200	197
O15541	45	RING finger protein 113A	RNF113A	0.881819	3.77E-06	114.63	S	RRKRPACDPEPGESGSSSDEGCTVVRPEKKR	0	998140	330
O15541	46	RING finger protein 113A	RNF113A	0.91637	3.77E-06	114.63	S	RKRPACDPEPGESGSSSDEGCTVVRPEKKRV	6610900	0	331
O15541	47	RING finger protein 113A	RNF113A	0.957219	3.77E-06	114.63	S	KRPACDPEPGESGSSSDEGCTVVRPEKKRVT	6610900	0	332
Q9Y2J2	5	Band 4.1-like protein 3;Band 4.1-like protein 3, N-terminal]	EPB41L3	0.547031	3.50E-09	114.63	S	____________MTTESGSDSESKPDQEAEPQ	0	746060	6785
Q9Y2J2	3	Band 4.1-like protein 3;Band 4.1-like protein 3, N-terminal]	EPB41L3	0.50629	3.50E-09	114.63	T	______________MTTESGSDSESKPDQEAE	0	746060	8284
Q9UQ35	1012	Serine/arginine repetitive matrix protein 2	SRRM2	0.764082	0.00674726	114.58	S	PKVKAQTPPGPSLSGSKSPCPQEKSKDSLVQ	1192700	566600	6603
Q9UM63	341	Zinc finger protein PLAGL1	PLAGL1	1	1.32E-05	114.56	S	CNISLFEDLPLQEPQSPQKLNPGFDLAKGNA	2983600	1300900	6515
Q14676	448	Mediator of DNA damage checkpoint protein 1	MDC1	0.76109	3.19E-07	114.53	T	EDMPQRVVLLQRSQTTTERDSDTDVEEEELP	0	877590	7607
Q14676	449	Mediator of DNA damage checkpoint protein 1	MDC1	0.729536	3.19E-07	114.53	T	DMPQRVVLLQRSQTTTERDSDTDVEEEELPV	0	877590	7608
O43491	89	Band 4.1-like protein 2	EPB41L2	0.821733	0.00474363	114.51	T	ISRFIPPWLKKQKSYTLVVAKDGGDKKEPTQ	3792400	0	7172
Q9NP66	105	High mobility group protein 20A	HMG20A	0.999988	0.0011643	114.5	S	GRKRKKPLRDSNAPKSPLTGYVRFMNERREQ	2988500	1202400	5887
Q5VUA4	2035	Zinc finger protein 318	ZNF318	0.996769	0.00116901	114.4	S	MPVDFCTTRVSPAHRSPTVLCQKVCEENSVS	2632900	1515200	3348
Q6ZSZ5	1291	Rho guanine nucleotide exchange factor 18	ARHGEF18	0.999882	0.000957679	114.4	S	LMGKDESTSRNRRSLSPILPGRHSPAPPPDP	2539400	1085500	3674
Q96JN8	502	Neuralized-like protein 4	NEURL4	1	0.000957679	114.4	S	HSDRLRRNNAILRALSPEGALRRAAPAAQAE	1147400	272420	4999
Q9UKV5	522	E3 ubiquitin-protein ligase AMFR	AMFR	0.499982	8.48E-08	114.39	S	SDSIRPALNSPVERPSSDQEEGETSAQTERV	0	811620	6459
Q9BTC0	1260	Death-inducer obliterator 1	DIDO1	0.999852	1.55E-11	114.34	S	PLCSADAAVSTTPPGSPPPPPPLPEPPVLKV	6308300	4852000	5361
Q9BTC0	1256	Death-inducer obliterator 1	DIDO1	0.87926	1.55E-11	114.34	T	PSKYPLCSADAAVSTTPPGSPPPPPPLPEPP	6308300	0	8047
P49450	21	Histone H3-like centromeric protein A	CENPA	0.488085	0.00740117	114.31	T	RSRKPEAPRRRSPSPTPTPGPSRRGPSLGAS	0	0	7414
P49450	23	Histone H3-like centromeric protein A	CENPA	0.488085	0.00740117	114.31	T	RKPEAPRRRSPSPTPTPGPSRRGPSLGASSH	0	0	7415
P42677	11	40S ribosomal protein S27	RPS27	1	0.00336009	114.24	S	_____MPLAKDLLHPSPEEEKRKHKKKRLVQ	2215800	1346800	1507
Q5XPI4	675	E3 ubiquitin-protein ligase RNF123	RNF123	0.728993	0.000533294	114.24	S	AVGGPLPLPRPGWLSSPTLGRANRFLSTAAV	1575400	0	3379
Q6F5E8	1120	Leucine-rich repeat-containing protein 16C	RLTPR	0.809134	0.000489981	114.24	S	KKPRSTRGPRTDLETSPGAAPRTRKTTFGDL	0	461960	3456
Q9Y6I3	435	Epsin-1	EPN1	1	0.000533294	114.24	S	SAGELELLAGEVPARSPGAFDMSGVRGSLAE	2678100	1771200	7011
P08047	2	Transcription factor Sp1	SP1	1	0.000579988	114.21	S	_______________MSDQDHSMDEMTAVVKI	1358400	1459800	946
P08047	7	Transcription factor Sp1	SP1	0.99883	0.000579988	114.21	S	__________MSDQDHSMDEMTAVVKIEKGVG	1459800	1472200	947
Q9Y6D9	428	Mitotic spindle assembly checkpoint protein MAD1	MAD1L1	0.942596	1.33E-05	114.21	S	AILGSYDSELTPAEYSPQLTRRMREAEDMVQ	0	1008500	7001
Q9P2M7	159	Cingulin	CGN	0.866423	5.46E-06	114.19	S	SNRSNSMLELAPKVASPGSTIDTAPLSSVDS	539740	452870	6247
Q14814	251	Myocyte-specific enhancer factor 2D	MEF2D	0.999945	0.00110061	114.06	S	PVANGNSLNKVIPAKSPPPPTHSTQLGAPSR	1362600	387660	2814
P19532	556	Transcription factor E3	TFE3	0.740099	0.00667074	114.03	S	LSPLRAASDPLLSSVSPAVSKASSRRSSFSM	864320	0	1168
Q13541	41	Eukaryotic translation initiation factor 4E-binding protein	EIF4EBP1	0.902003	1.43E-11	114.01	T	GVQLPPGDYSTTPGGTLFSTTPGGTRIIYDR	2638100	0	7554
O43432	1412	Eukaryotic translation initiation factor 4 gamma 3	EIF4G3	0.430324	0.00593887	113.95	S	LLEQKLDFIESDSPCSSEALSKKELSAEELY	0	0	385
O60292	1544	Signal-induced proliferation-associated 1-like protein 3	SIPA1L3	0.986386	0.000539084	113.95	S	RDTGQSPQKGLQRTLSDESLCSGRREPSFAS	530920	0	467
Q9UKM9	135	RNA-binding protein Raly	RALY	1	0.00318268	113.95	S	RDDFYDRLFDYRGRLSPVPVPRAVPVKRPRV	10562000	4526500	6448
P62995	201	Transformer-2 protein homolog beta	TRA2B	0.996989	0.000751073	113.95	T	GRRIRVDFSITKRPHTPTPGIYMGRPTYGSS	2252200	1351900	7457
Q14694	208	Ubiquitin carboxyl-terminal hydrolase 10	USP10	0.804095	3.32E-10	113.93	T	DAEFMGDMPPSVTPRTCNSPQNSTDSVSDIV	16696000	7581000	7618
Q9BVV8	113	Uncharacterized membrane protein C19orf24	C19orf24	0.712214	1.43E-09	113.89	T	PQRRRYGLLANTEDPTEMASLDSDEETVFES	0	0	8055
Q9HAU0	933	Pleckstrin homology domain-containing family A member 5	PLEKHA5	0.99947	2.33E-05	113.88	S	REKKKGLNVIGASDQSPLQSPSNLRDNPFRT	3803300	3056300	5817
P31749;P:	308;309;:	RAC-alpha serine/threonine-protein kinase;RAC-beta serine/tl	AKT1;AKT2;/	0.996199	0.000110346	113.86	T	FGLCKEGIKDGATMKTFCGTPEYLAPEVLED;FGL(	663940	379350	7350
Q00610	67	Clathrin heavy chain 1	CLTC	0.998631	0.00589039	113.81	S	IIDMNDPSNPIRRPISADSAIMNPASKVIAL	0	330630	2031
O00629	60	Importin subunit alpha-3	KPNA4	0.999998	0.000243175	113.78	S	LLKRRNVPHEDICEDSDIDGDYRVQNTSLEA	1639300	1651100	139
Q14683	966	Structural maintenance of chromosomes protein 1A	SMC1A	0.954118	1.17E-07	113.78	S	SQEEGSSQGEDSVSGSQRISSIYAREALIEI	0	1686600	2780
Q6UB99	834	Ankyrin repeat domain-containing protein 11	ANKRD11	0.998125	0.00120443	113.69	S	NKNQFLENEDTKFSLSDDQRDRWFSDLSDSS	1198200	738180	3584
O15014	360	Zinc finger protein 609	ZNF609	0.591653	0.00391417	113.69	T	CTRHDWAPPRFCDSPTSDLEMRNGRGRGKRM	0	0	7137
Q96D71	428	RalBP1-associated Eps domain-containing protein 1	REPS1	0.873334	2.31E-10	113.68	S	ELNQSSEQWETFSERSSSSQTLTQFDSNIAP	3640400	0	4854
Q96D71	429	RalBP1-associated Eps domain-containing protein 1	REPS1	0.885175	2.31E-10	113.68	S	LNQSSEQWETFSERSSSSQTLTQFDSNIAPA	3640400	2088700	4855
Q96D71	430	RalBP1-associated Eps domain-containing protein 1	REPS1	0.510083	2.31E-10	113.68	S	NQSSEQWETFSERSSSSQTLTQFDSNIAPAD	0	2088700	4856
Q96D71	431	RalBP1-associated Eps domain-containing protein 1	REPS1	0.495479	2.31E-10	113.68	S	QSSEQWETFSERSSSSQTLTQFDSNIAPADP	0	0	4857
O60716	346	Catenin delta-1	CTNND1	1	0.000714822	113.67	S	SDQYYWAPLAQHERGSLASLDSLRKGGPPPP	7141800	5367300	514
Q13371	296	Phosducin-like protein	PDCL	0.795388	0.00144473	113.67	S	VLTSVRNSATCHSEDSDLEID__________	675250	0	2479
Q6ZRV2	870	Protein FAM83H	FAM83H	0.990376	0.000714822	113.67	S	EGSGAHQVLHNESKGSPTSAYPERKGSPTPG	1100100	1765000	3663
Q92615	664	La-related protein 4B	LARP4B	0.974552	0.000544522	113.67	S	SYAEICQRTSKEPPSSPLQPQKEQKPNTVGC	1425700	1084900	4669
Q9ULH0	1555	Kinase D-interacting substrate of 220 kDa	KIDINS220	1	0.000714822	113.67	S	DKDRKAEGKVERVPKSPEHSAEPIRTFIKAK	1976400	1538400	6470
Q07157	912	Tight junction protein ZO-1	TJP1	1	0.0055211	113.66	S	PYSPQAQPQPIHRIDSPGFKPASQQKAEASS	219880	163770	2224
Q9Y6G9	405	Cytoplasmic dynein 1 light intermediate chain 1	DYNC1LI1	1	0.0055211	113.66	S	AAGRPVDASPRVPGGSPRTPNRSVSSNVASV	0	839720	7009
O00178	8	GTP-binding protein 1	GTPBP1	0.513197	2.82E-07	113.65	S	_________MATERSRSAMDSPVPASMFAPEP	0	788610	100
Q9UJU6	269	Drebrin-like protein	DBNL	0.99052	0.00148199	113.65	S	AVHPREIFKQKERAMSTTSISSPQPGKLRSP	0	0	6406
Q8N556	686	Actin filament-associated protein 1	AFAP1	0.613447	1.15E-09	113.65	T	FRHRTLENSPISSCDTSDTEGPVPVNSAAVL	0	0	7876
Q8N556	689	Actin filament-associated protein 1	AFAP1	0.331863	1.15E-09	113.65	T	RTLENSPISSCDTSDTEGPVPVNSAAVLKKS	0	0	7877
Q9Y4R8	637	Telomere length regulation protein TEL2 homolog	TELO2	0.432143	1.12E-11	113.64	S	ELSRPGCLGRTPQPGSPSPNTPCLPEAAVSQ	0	0	6936
Q9Y4R8	639	Telomere length regulation protein TEL2 homolog	TELO2	0.432143	1.12E-11	113.64	S	SRPGCLGRTPQPGSPSPNTPCLPEAAVSQPG	0	0	6937
Q86WB0	358	Nuclear-interacting partner of ALK	ZC3HC1	0.464999	0.000122792	113.63	S	KSPGPIVSRTRSWDSSSPVDRPEPEAASPTT	0	0	3987
Q6PL24	62	Protein TMED8	TMED8	0.996388	9.15E-10	113.59	S	KDTSLLASATDPEPCSSPHRPQMVSPVSKDA	3942800	3489500	3568
Q6PL24	74	Protein TMED8	TMED8	0.876674	9.15E-10	113.59	S	EPCSSPHRPQMVSPVSKDATEDLRKATGPLE	1788200	1967400	3570
Q8NHG8	82	E3 ubiquitin-protein ligase ZNRF2	ZNRF2	1	0.00148736	113.59	S	AAAAAPAAPAAPRSRSLGGAVGSVASGARAA	856740	638550	4411
P33993	121	DNA replication licensing factor MCM7	MCM7	1	0.0005069	113.54	S	RLMMEQRSRDPGMVRSPQNQYPAELMRRFEL	886640	513030	1370
Q8TEW0	383	Partitioning defective 3 homolog	PARD3	0.999999	0.000547108	113.54	S	QLSQSEKNNYYSSRFSPDSQYIDNRSVNSAG	1485700	1031000	4493
Q96N67	1430	Dedicator of cytokinesis protein 7	DOCK7	0.853907	0.00149201	113.53	S	RGQLGTYTIASPPERSPSGSAFGSQENLRWR	3400800	3697600	5039
Q9BSM1	3	Polycomb group RING finger protein 1	PCGF1	1	0.000390963	113.53	S	______________MASPQGGQIAIAMRLRNQ	0	671210	5345
Q9UKE5	766	TRAF2 and NCK-interacting protein kinase	TNIK	0.694582	0.000918196	113.52	S	QAGSSERTRVRANSKSEGSPVLPHEPAKVKP	0	0	6436
Q8TER5	257	Rho guanine nucleotide exchange factor 40	ARHGEF40	0.592275	0.00608474	113.52	T	YVELLEVTLPVRGSPTDAEGSPGLSRVRTVP	0	599670	7910
P51116	598	Fragile X mental retardation syndrome-related protein 2	FXR2	0.941342	0.000388568	113.49	T	QRRNRSRRRRNRGNRTDGSISGDRQPVTVAD	2415500	1027900	7437
Q96AY4	28	Tetratricopeptide repeat protein 28	TTC28	0.838854	5.58E-06	113.47	S	QGPTPARSRRRREPESPPASAPIPLFGADTI	958950	642550	4782
Q66K14	411	TBC1 domain family member 9B	TBC1D9B	0.99884	8.32E-12	113.43	S	KTPSKQPGSIGSRKASVVDPSTESSPAPQEG	959660	491630	3399
Q6PID6	197	Tetratricopeptide repeat protein 33	TTC33	0.999984	0.000922535	113.43	S	QRIKKSEAPAEVTHFSPKSIPDYDFESDEIV	1875400	1134500	3545
Q9NUA8	190	Zinc finger and BTB domain-containing protein 40	ZBTB40	0.656989	2.21E-08	113.43	S	TEEAAHSVSQEMSVNSPTAQESQRNAETPAE	0	0	6019
Q9UKJ3	740	G patch domain-containing protein 8	GPATCH8	1	0.000718668	113.43	S	ADSERGPKPEPPGSGSPAPPRRRRRAQDDSQ	8378700	2995800	6443
Q7Z6Z7	3827	E3 ubiquitin-protein ligase HUWE1	HUWE1	0.853714	1.07E-11	113.34	S	VDQPSPSAQDTQSIASDGTPQGEKEKEERPP	0	1964900	3877
Q13547	393	Histone deacetylase 1	HDAC1	1	1.42E-11	113.29	S	GVQMQAIPEDAIPEESGDEDEDDPDKRISIC	9053600	5352600	2579
Q99959	151	Plakophilin-2	PKP2	0.99957	0.000459715	113.24	S	VEERSLRHPLRRLEISPDSSPERAHYTHSDY	0	584440	5286
Q99959	154	Plakophilin-2	PKP2	0.582696	0.000459715	113.24	S	RSLRHPLRRLEISPDSSPERAHYTHSDYQYS	0	584440	5287
Q3ZCW2	25	Galectin-related protein	LGALSL	0.94071	5.62E-06	113.23	S	AVVKLDDGHLNNSLSSPVQADVYFPRLIVPF	1067400	878700	3088
Q7Z4S6	1239	Kinesin-like protein KIF21A	KIF21A	0.999921	0.00102341	113.22	S	ISRQSSLSEKKIPEPSPVTRRKAYEKAEKSK	1985800	1616600	3821
Q12888	1317	Tumor suppressor p53-binding protein 1	TP53BP1	0.444989	0.000418926	113.14	S	ISSFSSKASSLHRTSSGTSLSAMHSSGSSGK	0	0	2370
Q12888	1320	Tumor suppressor p53-binding protein 1	TP53BP1	0.49876	0.000418926	113.14	S	FSSKASSLHRTSSGTSLSAMHSSGSSGKGAG	0	0	2371
Q12888	1322	Tumor suppressor p53-binding protein 1	TP53BP1	0.49876	0.000418926	113.14	S	SKASSLHRTSSGTSLSAMHSSGSSGKGAGPL	0	0	2372
Q9UQ35	1336	Serine/arginine repetitive matrix protein 2	SRRM2	0.844393	0.000503958	113.14	S	PLRENSFGSPLEFRNSGPLGTEMNTGFSSEV	976120	1473200	6662
Q15643	1891	Thyroid receptor-interacting protein 11	TRIP11	1	0.000965572	113.11	S	IPPPKLSVHDMKPLDSPGRRKRDTNAPESFK	0	904300	2961
Q9C0E8	179	Protein lunapark	LNP	0.64884	1.11E-07	113.08	T	QEIRQRTAAQRNLSPTPASPNQGPPPQVPVS	0	0	8079
O75494	131	Serine/arginine-rich splicing factor 10	SRSF10	1	0.00397134	113.07	S	YRRSRSRSYERRRSRSRSFDYNYRRSYSPRN	2580900	1243900	633
P35222	191	Catenin beta-1	CTNNB1	1	0.000733781	113.07	S	HQLSKKEASRHAIMRSPQMVSAIVRTMQNTN	2590300	1208300	1378
Q13595	86	Transformer-2 protein homolog alpha	TRA2A	0.999243	0.00905977	113.07	S	SRSHSHSHRRRSRSRSYTPEYRRRRSRSHSP	830510	380430	2597
Q13595	88	Transformer-2 protein homolog alpha	TRA2A	0.99998	0.00905977	113.07	T	SHSHSHRRRSRSRSYTPEYRRRRSRSHSPMS	1604100	593720	7566
O00178	12	GTP-binding protein 1	GTPBP1	0.879176	1.86E-07	113.05	S	_____MATERSRSAMDSPVPASMFAPEPSSPG	3575700	2910600	101
Q5W0B1	553	RING finger protein 219	RNF219	0.99263	2.21E-07	113.05	S	ISELDSMMSESDNSKSPCNNGFKSLDLDGLS	0	2240000	3375
Q96G74	64	OTU domain-containing protein 5	OTUD5	1	0.000251117	113.05	S	DRDRDSGVVGARPRASPPPQGPLPGPPGALH	8879600	3756000	4912
Q969J3	77	Loss of heterozygosity 12 chromosomal region 1 protein	LOH12CR1	0.965439	0.00385864	112.94	T	FQPLLKGLLSGQTSPTNAKLEKLDSQQVLQL	0	0	7956
Q6P4R8	351	Nuclear factor related to kappa-B-binding protein	NFRKB	0.978658	9.21E-12	112.86	S	PLSSTEGVAPLSQAPSPLAIPAIKEEPLEDL	4281900	2864100	3528
O94979	532	Protein transport protein Sec31A	SEC31A	1	2.44E-08	112.85	S	ALKDSDQVAQSDGEESPAAEEQLLGEHIKEE	5048000	10450000	724
Q8IYB3	653	Serine/arginine repetitive matrix protein 1	SRRM1	0.994612	0.00991265	112.84	S	SPPPKQRSSPVTKRRSPSLSSKHRKGSSPSR	2318400	3775200	4156
Q8IYB3	655	Serine/arginine repetitive matrix protein 1	SRRM1	0.777233	0.00991265	112.84	S	PPKQRSSPVTKRRSPSLSSKHRKGSSPSRST	0	0	4157
Q96T23	397	Remodeling and spacing factor 1	RSF1	1	0.000740823	112.84	S	IPLKKREIKLSDDFDSPVKGPLCKSVTPTKE	0	1745800	5148
Q13153	229	Serine/threonine-protein kinase PAK 1	PAK1	0.886682	8.96E-06	112.74	T	TRDVATSPISPTENNTTPPDALTRNTEKQKK	0	0	7527
O14654	1105	Insulin receptor substrate 4	IRS4	0.728179	0.00390316	112.71	T	QSFFAAARAAVSAFPTDSLERDLSPSSAPAV	0	3028600	7126

Q99700	667	Ataxin-2	ATXN2	0.4251	0.0034526	112.67	S	HNPPSEAATPPVARTSPSGGTWSSVVSGVPR	0	0	5261
Q9UBI6	49	Guanine nucleotide-binding protein G(I)/G(S)/G(O) subunit ga	GNG12	1	0.00117732	112.67	S	KASADLMSYCEEHARSDPLLIGIPTSENPFK	398700	367890	6266
Q99700	666	Ataxin-2	ATXN2	0.4251	0.0034526	112.67	T	SHNPPSEAATPPVARTSPSGGTWSSVVSGVP	0	0	8033
Q96ST2	248	Protein IWS1 homolog	IWS1	0.999986	0.000469483	112.64	S	QASDSENEELPKPRISDSESEDPPRHQASDS	976380	732860	5133
Q96ST2	250	Protein IWS1 homolog	IWS1	0.99985	0.000469483	112.64	S	SDSENEELPKPRISDSESEDPPRHQASDSEN	976380	732860	5134
P17544	322	Cyclic AMP-dependent transcription factor ATF-7	ATF7	0.883708	6.87E-12	112.6	S	LIQHPDAPSPAQPQVSPAQPTPSTGGRRRRT	666310	961700	1111
Q9GZR1	352	Sentrin-specific protease 6	SENP6	0.999929	1.50E-05	112.53	S	DDDDDNDRTNRRESISPQPADSACSSPAPST	1629400	734260	5552
Q9GZR1	362	Sentrin-specific protease 6	SENP6	0.908038	1.50E-05	112.53	S	RRESISPQPADSACSSPAPSTGKVEAALNEN	0	398730	5553
Q16629	192	Serine/arginine-rich splicing factor 7	SRSF7	1	0.00567328	112.5	S	RRSRSGSIKGSRYFQSPSRSRSRSRSISRPR	64729000	49208000	3018
Q16629	194	Serine/arginine-rich splicing factor 7	SRSF7	1	0.00567328	112.5	S	SRSGSIKGSRYFQSPSRSRSRSRSISRPRSS	61576000	49490000	3019
Q16629	196	Serine/arginine-rich splicing factor 7	SRSF7	1	0.00914381	112.5	S	SGSIKGSRYFQSPSRSRSRSRSISRPRSSRS	67514000	27448000	3020
Q8IY92	1469	Structure-specific endonuclease subunit SLX4	SLX4	1	0.000567657	112.5	S	PSRRMNEAADSRDCRSPGLLDTTPIRGSCTT	1257400	813500	4127
Q8IZ21	270	Phosphatase and actin regulator 4	PHACTR4	1	6.28E-10	112.47	S	AKQPPIPPPKPAHRNSNPVIAELSQAINSGT	2725500	1374400	4178
Q8IZ21	291	Phosphatase and actin regulator 4	PHACTR4	0.978332	6.28E-10	112.47	S	ELSQAINSGTLLSKPSPPLPPKRGIPSTSVP	3567800	1374400	4179
Q96PC5	1130	Melanoma inhibitory activity protein 2	MIA2	0.996949	0.000489914	112.44	S	VPNTAFGREHSPYGPSPLGWPSSETRAFLSP	1366800	1133400	5053
Q96PC5	1127	Melanoma inhibitory activity protein 2	MIA2	0.684111	0.000489914	112.44	Y	ALDVPNTAFGREHSPYGPSPLGWPSSETRAF	1366800	1133400	8412
Q13627	758	Dual specificity tyrosine-phosphorylation-regulated kinase 1	DYRK1A	0.970893	0.000519605	112.43	S	ADREESPMTGVCVQQSPVASS__________	0	610930	2623
Q8IYH5	113	ZZ-type zinc finger-containing protein 3	ZZZ3	0.999274	0.000753891	112.43	S	RQAIENCERRQTEPVSPVLKRIKRCLRSEAP	940890	1025800	4174
Q9UDY2	1067	Tight junction protein ZO-2	TJP2	0.771546	6.44E-10	112.42	S	PSTPIPPQEGEEVGESSEEQDNAPKSVLGKV	1849900	708180	6282
O95453	619	Poly(A)-specific ribonuclease PARN	PARN	0.998828	0.000754486	112.41	S	GRKKAKKLKRMKKELSPAGSISKNSPATLFE	3663800	2348600	775
Q53EL6	94	Programmed cell death protein 4	PDCD4	0.960236	0.000754486	112.41	S	DSGSDALRSGLTVPTSPKGRLLDRRSRSGKG	27318000	16568000	3125
P78559	2237	Microtubule-associated protein 1A;MAP1A heavy chain;MAP1 lig	MAP1A	0.827388	1.09E-09	112.35	S	KAPSLDSSLPQLPSPSSPGAPLLSNLPRPAS	2329700	0	1973
Q5VZ89	968	DENN domain-containing protein 4C	DENND4C	0.996445	1.92E-07	112.34	S	EVCDASAIVAKHSQPSPEPHSPTEPPAWGSS	11313000	5432400	3364
Q5VZ89	973	DENN domain-containing protein 4C	DENND4C	0.974665	1.92E-07	112.34	S	SAIVAKHSQPSPEPHSPTEPPAWGSSIVKVP	11313000	5432400	3365
Q13242	189	Serine/arginine-rich splicing factor 9	SRSF9	0.999907	3.93E-07	112.3	S	EGETSYIRVYPERSTSYGYSRSRSGSRGRDS	2149100	0	2437
Q8IY92	1452	Structure-specific endonuclease subunit SLX4	SLX4	0.772655	0.000571666	112.3	S	PIDHWNLERTGPLSTSSPSRRMNEAADSRDC	714350	343690	4128
P19525	33	Interferon-induced, double-stranded RNA-activated protein ki	EIF2AK2	1	0.000980224	112.24	S	RQKQGVVLKYQELPNSGPPHDRRFTFQVIID	0	0	1166
Q13523	576	Serine/threonine-protein kinase PRP4 homolog	PRPF4B	1	0.00441251	112.17	T	MSVPSEPSSPQSSTRTRSPSPDDILERVAAD	1331000	1286900	7549
O15027	1069	Protein transport protein Sec16A	SEC16A	1	0.000204803	112.16	S	LERAQQELVPPQQQASPPQLPKAMFSELSNP	12774000	6364200	240
Q9HAV4	826	Exportin-5	XPO5	1	2.30E-05	112.16	S	SAILGLPQPLLELNDSPVFKTVLERMQRFFS	263610	334500	5820
Q99856	119	AT-rich interactive domain-containing protein 3A	ARID3A	1	2.95E-05	112.14	S	RGREGPGEEHFEDMASDEDMKPKWEEEEMEE	8535600	2774100	5280
P30050	38	60S ribosomal protein L12	RPL12	1	0.00108682	112.13	S	GATSALAPKIGPLGLSPKKVGDDIAKATGDW	626120	519470	1340
Q9NYB9	227	Abl interactor 2	ABI2	0.999897	0.000579039	112.11	S	DYVPSPTRNMAPSQQSPVRTASVNQRNRTYS	13679000	8554500	6096
Q13813	1031	Spectrin alpha chain, non-erythrocytic 1	SPTAN1	0.934479	0.00315437	112.08	S	VPAAYVKKLDPAQSASRENLLEEQGSIALRQ	0	752440	2626
P24928	1847	DNA-directed RNA polymerase II subunit RPB1	POLR2A	0.623895	3.03E-05	112.02	S	YSPASPKYTPTSPSYSPSSPEYTPTSPKYSP	1469800	0	1255
Q7KZI7	486	Serine/threonine-protein kinase MARK2	MARK2	1	0.0049415	112.02	S	STNSVLSTSTNRSRNSPLLERASLGQASIQN	675420	998670	3714
P49585	329	Choline-phosphate cytidylyltransferase A	PCYT1A	0.999948	0.00424611	111.95	S	ISPKQSPSSSPTRERSPSPSFRWPFSGKTSP	2740600	4095300	1667
P49585	331	Choline-phosphate cytidylyltransferase A	PCYT1A	0.816634	0.00424611	111.95	S	PKQSPSSSPTRERSPSPSFRWPFSGKTSPPC	2740600	4095300	1668
Q5VUA4	136	Zinc finger protein 318	ZNF318	1	0.000591162	111.94	S	DGRGDHPGDSGSRRRSPGLCSDSLEKSLRIT	4543300	3636600	3347
Q6VMQ6	479	Activating transcription factor 7-interacting protein 1	ATF7IP	0.749238	0.000778055	111.94	S	NPDLEEKMESSFGSPSKQESSESLPKEAFLV	0	1342300	3602
Q96A49	269	Synapse-associated protein 1	SYAP1	0.764976	2.03E-10	111.93	S	KSQLKTQEDEEEISTSPGVSEFVSDAFDACN	0	118640	4768
Q8NI27	1222	THO complex subunit 2	THOC2	0.449556	9.14E-11	111.89	S	GPSSSSIGSASKSDESSTEETDKSRERSQCG	0	0	4427
Q6PK04	19	Coiled-coil domain-containing protein 137	CCDC137	1	0.00111214	111.86	S	AGRGAAVSRVQAGPGSPRRARGRQQVQPLGK	4934100	4782200	3555
Q13523	849	Serine/threonine-protein kinase PRP4 homolog	PRPF4B	0.97337	1.37E-05	111.82	Y	DFGSASHVADNDITPYLVSRFYRAPEIIIGK	0	2397100	8377
Q9H2Y7	1025	Zinc finger protein 106	ZNF106	0.992057	0.00135846	111.81	S	DGQSIRKKRRATGDGSSPELPSLERKNKRRK	1928000	1099100	5624
O60934	397	Nibrin	NBN	1	0.000792721	111.72	S	KEIKVSKMEQKFRMLSQDAPTVKESCKTSSN	344350	585790	542
Q9NYV4	341	Cyclin-dependent kinase 12	CDK12	0.999999	0.00773384	111.72	S	PYGRRRSSSPFLSKRSLSRSPLPSRKSMKSR	7236600	5331500	6136
Q9HCD5	11	Nuclear receptor coactivator 5	NCOA5	0.917664	0.000484942	111.72	T	_____MNTAPSRPSPTRRDPYGFGDSRDSRR	17908000	10071000	8131
P49810	19	Presenilin-2;Presenilin-2 NTF subunit;Presenilin-2 CTF subur	PSEN2	0.594861	0.0021722	111.66	S	FMASDSEEEVCDERTSLMSAESPTPRSCQEG	0	782230	1722
P49810	22	Presenilin-2;Presenilin-2 NTF subunit;Presenilin-2 CTF subur	PSEN2	0.999569	0.0021722	111.66	S	SDSEEEVCDERTSLMSAESPTPRSCQEGRQG	0	782230	1723
Q96AY4	32	Tetratricopeptide repeat protein 28	TTC28	0.860998	7.31E-06	111.65	S	PARSRRRREPESPPASAPIPLFGADTIGQRS	0	642550	4783
Q96QC0	542	Serine/threonine-protein phosphatase 1 regulatory subunit 1(	PPP1R10	0.944097	1.30E-10	111.64	S	SMDETPYVETLEPGGSGGSPDGAGGSKLPPV	6721900	5837100	5075
Q96QC0	545	Serine/threonine-protein phosphatase 1 regulatory subunit 1(	PPP1R10	0.499238	1.30E-10	111.64	S	ETPYVETLEPGGSGGSPDGAGGSKLPPVLAN	0	0	5076
O75533	326	Splicing factor 3B subunit 1	SF3B1	0.999976	0.00545169	111.64	T	AETPRTDRGGDSIGETPTPGASKRKSRWDET	0	0	7207
O75533	328	Splicing factor 3B subunit 1	SF3B1	0.999987	0.00545169	111.64	T	TPRTDRGGDSIGETPTPGASKRKSRWDETPA	0	0	7208
O15541	6	RING finger protein 113A	RNF113A	1	0.000585569	111.63	S	__________MAEQLSPGKAVDQVCTFLFKK	0	1023800	328
Q8IWQ3;Q8	489;563	Serine/threonine-protein kinase BRSK2;Serine/threonine-prote	BRSK2;BRSK1	1	0.0013749	111.63	S	PWRARLNSIKNSFLGSPRFHRRKLQVPTPEE	2710900	0	4064
Q9BRJ6	59	Uncharacterized protein C7orf50	C7orf50	1	2.22E-05	111.63	S	GEAVLRPGLDAEPELSPEEQRVLERKLKKER	1465600	1327500	5323
P12268	159	Inosine-5'-monophosphate dehydrogenase 2	IMPDH2	0.838087	0.00137893	111.61	S	ITDTGRMGSRLVGIISSRDIDFLKEEEHDCF	2061100	0	1032
Q6WKZ4	967	Rab11 family-interacting protein 1	RAB11FIP1	1	5.71E-07	111.57	S	MADLNLSLPSIPEVASDDERIDQVEDDGDQV	2273800	1033400	3641
P18887	447	DNA repair protein XRCC1	XRCC1	0.853618	4.30E-05	111.56	S	QPQTKTKPTQAAGPSSPQKPPTPEETKAASP	3563700	1711200	1158
Q16543	13	Hsp90 co-chaperone Cdc37;Hsp90 co-chaperone Cdc37, N-termina	CDC37	0.999792	2.11E-08	111.56	S	___MVDYSVWDHIEVSDDEDETHPNIDTASL	6997600	2964300	3015
Q5QP82	92	DDB1- and CUL4-associated factor 10	DCAF10	0.997338	2.43E-08	111.56	S	PESSTASAPGEPSPPSPPCRRPGPDCRAKSR	1604900	1162600	3208
P18887	457	DNA repair protein XRCC1	XRCC1	0.753562	0.000566034	111.56	T	AAGPSSPQKPPTPEETKAASPVLQEDIDIEG	523650	50928	7305
Q9NZ56	295	Formin-2	FMN2	0.999761	5.41E-08	111.53	S	PESLAAEPREPQQPPSPGGLPVSEAPSLPAA	1611600	1358000	6147
Q9UKE5	678	TRAF2 and NCK-interacting protein kinase	TNIK	0.993057	0.000669697	111.52	S	RQEEDIPPKVPQRTTSISPALARKNSPGNGS	420340	0	6438
Q9UKE5	680	TRAF2 and NCK-interacting protein kinase	TNIK	1	0.000669697	111.52	S	EEDIPPKVPQRTTSISPALARKNSPGNGSAL	2841900	1130400	6439
Q96S55	75	ATPase WRNIP1	WRNIP1	0.972959	0.00470498	111.5	S	RAKGPSPPGAKRRRLSESSALKQPATPTAAE	2895200	1409200	5108
Q9BYG3	223	MKI67 FHA domain-interacting nucleolar phosphoprotein	NIFK	0.949045	0.00330368	111.46	T	QVLRKKKKKVSGTLDTPEKTVDSQGPTPVCT	0	438070	8067
Q96K49	534	Transmembrane protein 87B	TMEM87B	0.999795	5.14E-08	111.4	S	IPSSFTDVALPVLVDSDEEIMTRSEMAEKMF	2656500	1382600	5003
P11274	122	Breakpoint cluster region protein	BCR	1	4.25E-08	111.33	S	PPPAEEPEARPDGEGSPGKARPGTARRPGAA	11986000	6509700	1014
Q9H792	1035	Pseudopodium-enriched atypical kinase 1	PEAK1	0.721285	0.000822998	111.27	S	AENALLQDSEKKRSHSSPSQIPKKILSHMTH	0	318410	5754
Q5SY16	88	Polynucleotide 5'-hydroxyl-kinase NOL9	NOL9	0.968991	9.52E-10	111.15	S	AAARRPNTATPSPIPSPTPASEPESEPELES	2063700	2813800	3230
Q5SY16	93	Polynucleotide 5'-hydroxyl-kinase NOL9	NOL9	0.990289	9.52E-10	111.15	S	PNTATPSPIPSPTPASEPESEPELESASSCH	2574600	1269100	3231
Q5SY16	97	Polynucleotide 5'-hydroxyl-kinase NOL9	NOL9	0.999887	9.52E-10	111.15	S	TPSPIPSPTPASEPESEPELESASSCHRPLL	4827000	2377400	3232
Q9NTI5	1255	Sister chromatid cohesion protein PDS5 homolog B	PDS5B	0.401746	0.00109819	111.12	T	EQKPKGSQRSRKRGHTASESDEQQWPEEKRL	0	0	8162
O43290	596	U4/U6.U5 tri-snRNP-associated protein 1	SART1	0.723485	4.12E-07	111.11	S	LMDFERDEERSANGGSESDGEENIGWSTVNL	0	745380	352
P04049	295	RAF proto-oncogene serine/threonine-protein kinase	RAF1	0.491727	8.87E-08	111.11	S	AIRSHSESASPSALSSSPNNLSPTGWSQPKT	0	0	869
Q9NZ63	253	Uncharacterized protein C9orf78	C9orf78	0.613436	0.00490659	111.11	T	RNKEEPKARPLRVGDTEKPEPERSPPNRKRP	655420	683920	8180
P50851	1118	Lipopolysaccharide-responsive and beige-like anchor protein	LRBA	0.820048	1.98E-08	111.09	S	EEEEDDDYVELKVEGSPTEEANLPTELQDNS	1338300	4080600	1767
Q9UPW6	593	DNA-binding protein SATB2	SATB2	0.789248	1.13E-05	111.09	S	PVQVLHRQQSQPAKESSPPREEAPPPPPPTE	2904400	2266700	6591
Q9UPW6	594	DNA-binding protein SATB2	SATB2	0.5	1.13E-05	111.09	S	VQVLHRQQSQPAKESSPPREEAPPPPPPTED	2904400	2266700	6592
Q12888	522	Tumor suppressor p53-binding protein 1	TP53BP1	0.698497	0.0211766	111.07	Y	SLTGDSCKLMLSTSEYSQSPKMESLSSHRID	0	0	8374
P29558	38	RNA-binding motif, single-stranded-interacting protein 1	RBMS1	0.793101	1.56E-12	111.05	S	QAKQSLVPAHPMAPPSPSTTSSNNNSSSSSN	0	0	1320
Q9ULU4	486	Protein kinase C-binding protein 1	ZMYND8	0.994174	5.39E-07	111.05	S	KSTASPASTKTGQAGSLSGSPKPFSPQLSAP	2470400	0	6507
Q9ULU4	490	Protein kinase C-binding protein 1	ZMYND8	0.920548	5.39E-07	111.05	S	SPASTKTGQAGSLSGSPKPFSPQLSAPITTK	2470400	0	6509
Q9Y6M1	161	Insulin-like growth factor 2 mRNA-binding protein 2	IGF2BP2	0.781409	0.000524915	111.01	S	ENYSFKISYIPDEEVSSPSPPQRAQRGDHSS	2717600	1571100	7013
Q92609	539	TBC1 domain family member 5	TBC1D5	0.735738	0.00180744	110.98	S	PVQLNKGLSSKNISSSPSVESLPGGREFTGS	701500	552250	4656
Q5XPI4	677	E3 ubiquitin-protein ligase RNF123	RNF123	0.586256	0.00268731	110.98	T	GGPLPLPRPGWLSSPTLGRANRFLSTAAVSL	0	1132700	7736
P49006	36	MARCKS-related protein	MARCKSL1	1	0.00149663	110.93	S	ASPAKANGQENGHVKSNGDLSPKGEGESPPV	1311900	1959500	1641
Q9UQ35	1890	Serine/arginine repetitive matrix protein 2	SRRM2	0.998924	0.00575866	110.93	S	RSRSRTPLISRRRSRSRTSPVSRRRSRSRTS	1791800	1554100	6729
Q9UQ35	1893	Serine/arginine repetitive matrix protein 2	SRRM2	0.996326	0.00575866	110.93	S	SRTPLISRRRSRSRTSPVSRRRSRSRTSVTR	1791800	1554100	6730
Q96QD9	15	UAP56-interacting factor	FYTTD1	1	0.00117451	110.92	S	_MNRFGTRLVGATATSSPPPKARSNENLDKI	1054100	1182900	5079
Q14181	147	DNA polymerase alpha subunit B	POLA2	0.99087	3.33E-05	110.89	S	LTKRSVSTRSPHQLLSPSSFSPSATPSQKYN	5535500	1421800	2695
Q96B23	69	Uncharacterized protein C18orf25	C18orf25	0.909459	0.000187568	110.89	S	SMPCLLMELRRDSSESQLASTESDKPTTGRV	751790	939140	4794
Q14012	363	Calcium/calmodulin-dependent protein kinase type 1	CAMK1	0.795673	0.000330125	110.84	S	GCCCRDCCVEPGTELSPTLPHQL________	301450	423810	2646
P42356	256	Phosphatidylinositol 4-kinase alpha	PI4KA	0.377495	0.00767939	110.77	S	NLLTVCQEGTLKRKTSSVSSISQVSPERGMP	0	0	1502
P46821	1793	Microtubule-associated protein 1B;MAP1B heavy chain;MAP1 lig	MAP1B	0.79162	0.000559435	110.77	S	LSPKSDISPLTPRESSPLYSPTFSDSTSAVK	0	5412700	1581
P42356	255	Phosphatidylinositol 4-kinase alpha	PI4KA	0.377495	0.00767939	110.77	T	SNLLTVCQEGTLKRKTSSVSSISQVSPERGM	0	0	7379
O60716	268	Catenin delta-1	CTNND1	0.976035	0.000865107	110.64	S	RQDVYGPQPQVRVGGSSVDLHRFHPEPYGLE	0	0	517
P49590	67	Probable histidine--tRNA ligase, mitochondrial	HARS2	1	0.000865107	110.64	S	KPNFIIKTPKGTRDLSPQHMVVREKILDLVI	2704400	1400300	1671
Q9UQB8	454	Brain-specific angiogenesis inhibitor 1-associated protein 2	BAIAP2	0.992266	0.000865107	110.64	S	GSDRLHMSLQQGKSSSTGNLLDKDDLAIPPP	0	0	6757
Q9Y2L1	730	Exosome complex exonuclease RRP44	DIS3	0.539936	1.01E-05	110.64	S	TDTAKSLAESLDQAESPTFPYLNTLLRILAT	281250	711550	6794
Q9ULM3	118	YEATS domain-containing protein 2	YEATS2	0.995548	0.00621643	110.63	S	DTMVFNHPAIKKFLESPSRSSSPANQRAETP	1034000	568430	6492
P35251	156	Replication factor C subunit 1	RFC1	0.997893	3.51E-06	110.6	S	MKKNEENTKTKNKPLSPIKLTPTSVLDYFGT	2062100	1360100	1388
Q6P6C2	69	RNA demethylase ALKBH5	ALKBH5	0.894716	0.000177496	110.6	S	SGAKRKYQEDSDPERSDYEEQQLQKEEEARK	0	4126000	3534
Q7Z2Z1	838	Treslin	TICRR	0.889189	0.00831738	110.6	S	LSVPFLSSARRSVSGSPESDELQELRTRSAK	542630	472980	3768
Q9BW71	98	HIRA-interacting protein 3	HIRIP3	0.954049	1.10E-05	110.6	S	PTPCSDPERKRFRFNSESESGSEASSPDYFG	2816900	904830	5412
Q9BW71	104	HIRA-interacting protein 3	HIRIP3	0.896196	1.10E-05	110.6	S	PERKRFRFNSESESGSEASSPDYFGPPAKNG	872990	348610	5415
P35251	161	Replication factor C subunit 1	RFC1	0.988077	3.51E-06	110.6	T	ENTKTKNKPLSPIKLTPTSVLDYFGTGSVQR	2062100	1360100	7360
P46060	428	Ran GTPase-activating protein 1	RANGAP1	0.909732	1.84E-09	110.57	S	KILDPNTGEPAPVLSSPPPADVSTFLAFPSP	0	17797000	1542
P46060	442	Ran GTPase-activating protein 1	RANGAP1	0.999961	1.84E-09	110.57	S	SSPPPADVSTFLAFPSPEKLLRLGPKSSVLI	14880000	18379000	1543
Q9H1B7	215	Interferon regulatory factor 2-binding protein-like	IRF2BPL	0.999468	0.00189186	110.57	S	PKPTPEEGPPELNRQSPNSSSAAASVASRRG	2826400	1891600	5599
Q9Y4F1	24	FERM, RhoGEF and pleckstrin domain-containing protein 1	FARP1	0.922812	0.00784693	110.57	T	TPGSRLGAPENSGISTLERGQKPPPTPSGKL	395140	420460	8302
Q12906	382	Interleukin enhancer-binding factor 3	ILF3	0.996012	0.00213035	110.55	S	ITPMKRPMEEDGEEKSPSKKKKKIQKKEEKA	0	1345100	2377
Q12923	908	Tyrosine-protein phosphatase non-receptor type 13	PTPN13	0.991262	0.00831932	110.55	S	LQAESVRGFNMGRAISTGSLASSTLNKLAVR	973460	497390	2378
Q8NFC6	3019	Biorientation of chromosomes in cell division protein 1-like	BOD1L1	0.917161	0.00156609	110.53	S	RSTTRSEAQRSKTQLSPSIKRKREVSPPGAR	1727200	1201100	4395
Q9UHB7	694	AF4/FMR2 family member 4	AFF4	1	0.00156609	110.53	S	SSVEEEDSFFRQRMFSPMEEKELLSPLSEPD	393070	362030	6344
P20700;Q(	391;405	Lamin-B1;Lamin-B2	LMNB1;LMNB2	0.999943	0.0033615	110.51	S	AYRKLLEGEEERLKLSPSPSSRVTVSRASSS;AYRI	46760000	24783000	1187
P20700;Q(	393;407	Lamin-B1;Lamin-B2	LMNB1;LMNB2	0.985894	0.0033615	110.51	S	RKLLEGEEERLKLSPSPSSRVTVSRASSSRS;RKLI	45301000	24952000	1188
Q86U90	37	YrdC domain-containing protein, mitochondrial	YRDC	1	0.00120586	110.51	S	EGPAGSRSGRLFRPPSPAPAAPGARLLRLPG	0	1851600	3921
Q9UMX0	13	Ubiquilin-1	UBQLN1	0.940974	7.57E-10	110.46	S	___MAESGESGGPPGSQDSAAGAEGAGAPAA	0	976490	6521
Q9UMX0	16	Ubiquilin-1	UBQLN1	0.940973	7.57E-10	110.46	S	MAESGESGGPPGSQDSAAGAEGAGAPAAAAS	1115000	976490	6522
Q07866	521	Kinesin light chain 1	KLC1	1	0.00314283	110.44	S	VAEVLNDPENMEKRRSRESLNVDVVKYESGP	4920900	2594000	2238
Q5JRA6	1739	Melanoma inhibitory activity protein 3	MIA3	0.391789	0.000287795	110.44	S	PSPSDPGSGTATMMNSSSRGSSPTRVLDEGK	0	0	3176
Q9UQR0	590	Sex comb on midleg-like protein 2	SCML2	0.999982	0.00115004	110.44	S	RSVPGTTSSPLVGDISPKSSPHEVKFQMQRK	1808600	1339300	6763
Q7Z6Z7	2832	E3 ubiquitin-protein ligase HUWE1	HUWE1	0.645591	1.77E-06	110.4	T	EAETTQMELSPAPTITSLSPERAEDSDALTA	1506000	0	7819
P49792;Q7	2250;127	E3 SUMO-protein ligase RanBP2;RanBP2-like and GRIP domain-co	RANBP2;RGPI	0.611829	9.41E-07	110.38	S	DDSVSSSSVHASPLASSPVRKNLFRFGESTT	5216700	0	1707
Q8N3F8	295	MICAL-like protein 1	MICALL1	1	0.000701467	110.38	S	IPTKPRVPGKLQELASPPAGRPTPAPRKASE	2262500	1209600	4250
P49790	102	Nuclear pore complex protein Nup153	NUP153	0.992461	1.73E-07	110.36	T	VYADEESSNITDGRITPEPAVSNTEEPSTTS	6365500	3895200	7419
O94806	27	Serine/threonine-protein kinase D3	PRKD3	0.966363	4.22E-05	110.33	S	KSVLPTAIPAVLPAASPCSSPKTGLSARLSN	1112400	561130	693
O94806	31	Serine/threonine-protein kinase D3	PRKD3	0.874676	4.22E-05	110.33	S	PTAIPAVLPAASPCSSPKTGLSARLSNGSFS	1112400	0	695
Q96AT1	146	Uncharacterized protein KIAA1143	KIAA1143	0.997995	0.00849788	110.22	S	VKKNSQKQIKNSSLLSFDNEDENE_______	797840	624760	4777
Q9H8V3	367	Protein ECT2	ECT2	0.946168	0.00199683	110.22	S	YLYEKANTPELKKSVSMLSLNTPNSNRKRRR	0	657560	5792
Q9Y2W1	406	Thyroid hormone receptor-associated protein 3	THRAP3	0.972715	0.0189486	110.22	S	DSFAPKTDSEKPFRGSQSPKRYKLRDDFEKK	0	660860	6831
Q9Y2W1	408	Thyroid hormone receptor-associated protein 3	THRAP3	0.999182	0.0189486	110.22	S	FAPKTDSEKPFRGSQSPKRYKLRDDFEKKMA	747900	660860	6832
Q9Y6D5	218	Brefeldin A-inhibited guanine nucleotide-exchange protein 2	ARFGEF2	0.988697	7.81E-06	110.21	S	QEARELEKPIQSKPQSPVIQAAAVSPKFVRL	583130	305040	6992
O43310	299	CBP80/20-dependent translation initiation factor	CTIF	0.999958	0.000753418	110.15	S	DTAGDTGHSSLEAPRSPDTLAPVASERLPPQ	1474000	1210100	358
Q9BY44	503	Eukaryotic translation initiation factor 2A;Eukaryotic trans	EIF2A	0.883091	0.000468838	110.12	S	QRKHEAKKAAKQEARSDKSPDLAPTPAPQST	6751800	0	5464

O14745	2	Na(+)/H(+) exchange regulatory cofactor NHE-RF1	SLC9A3R1	1	0.000324152	110.08	S	______________MSADAAAGAPLPRLCCL	0	0	205
Q07157	125	Tight junction protein ZO-1	TJP1	0.999926	1.02E-07	110.06	S	KKVQIPVSRPDPEPVSDNEEDSYDEEIHDPR	12413000	12018000	2226
Q07157	131	Tight junction protein ZO-1	TJP1	0.585593	1.02E-07	110.06	S	VSRPDPEPVSDNEEDSYDEEIHDPRSGRSGV	10450000	12018000	2227
Q9UJF2	16	Ras GTPase-activating protein nGAP	RASAL2	0.970085	1.97E-06	110.06	S	MQTPEVPAERSPRRRSISGTSTSEKPNSMDT	1539500	2534000	6398
O60318	527	Germinal-center associated nuclear protein	MCM3AP	0.975636	2.45E-06	110.01	S	KPFSLKEKKPGDGEVSPSTEDAPFQHSPLGK	2608000	2093000	477
Q14008	829	Cytoskeleton-associated protein 5	CKAP5	0.505505	2.15E-07	109.91	T	GQSPPAPTRGISKHSTSGTDEGEDGDEPDDG	0	0	7576
Q14008	832	Cytoskeleton-associated protein 5	CKAP5	0.493045	2.15E-07	109.91	T	PPAPTRGISKHSTSGTDEGEDGDEPDDGSND	0	0	7577
Q15648	1219	Mediator of RNA polymerase II transcription subunit 1	MED1	0.338111	0.00102456	109.84	S	KLASPMKPVPGTPPSSKAKSPISSGSGGSHM	0	0	2964
Q96HC4	136	PDZ and LIM domain protein 5	PDLIM5	0.597291	0.00130201	109.83	S	NNMAYNKAPRPFGSVSSPKVTSIPSPSSAFT	0	0	4944
Q96P16	158	Regulation of nuclear pre-mRNA domain-containing protein 1A	RPRD1A	0.83813	1.25E-06	109.83	S	IKVDENENCSSLGSPSEPPQTLDLVRALQDL	0	0	5045
P42566	796	Epidermal growth factor receptor substrate 15	EPS15	1	0.00169584	109.79	S	PCPLPPGKRSINKLDSPDPFKLNDPFQPFPG	2580200	1991900	1504
Q13614	630	Myotubularin-related protein 2	MTMR2	0.499999	0.0020544	109.79	S	QREISNRSTSSSERASSPAQCVTPVQTVV__	0	0	2603
Q9BXI6	409	TBC1 domain family member 10A	TBC1D10A	0.908506	0.000926458	109.72	S	LDAEPGPRPALQPSPSIRLPLDAPLPGSKAK	1278700	799240	5450
Q9UHX1	311	Poly(U)-binding-splicing factor PUF60	PUF60	0.999897	1.66E-08	109.69	T	YLRVGKAVTPPMPLLTPATPGGLPPAAAVAA	0	3395900	8218
Q9UHX1	314	Poly(U)-binding-splicing factor PUF60	PUF60	0.999979	1.66E-08	109.69	T	VGKAVTPPMPLLTPATPGGLPPAAAVAAAAA	0	3395900	8219
Q9ULM3	447	YEATS domain-containing protein 2	YEATS2	1	0.00208035	109.67	S	SSCKIVPQSQVPNPESPGKSFQPITMSCKIV	4139200	3838400	6494
O43379	1348	WD repeat-containing protein 62	WDR62	0.999971	1.33E-06	109.65	S	RLHGSAFRPSLPAPESPGLPAHPSNPQLPEA	3460800	3841600	369
P34932	538	Heat shock 70 kDa protein 4	HSPA4	1	2.03E-05	109.65	T	QVDQEEPHVEEQQQQTPAENKAESEEMETSQ	1277100	2043700	7353
Q86TV6	159	Tetratricopeptide repeat protein 7B	TTC7B	0.783564	0.0010533	109.6	S	AYATKGLCLEKLPISSSTSNLHVDREQDVIT	0	1298100	3906
Q8N2M8	501	CLK4-associating serine/arginine rich protein	CLASRP	0.991562	0.00110025	109.6	S	LRHHSSSRSRSSWSLSPSRSRSLTRSRSHSP	828710	150980	4243
Q8N2M8	503	CLK4-associating serine/arginine rich protein	CLASRP	0.999909	0.00110025	109.6	S	HHSSSRSRSSWSLSPSRSRSLTRSRSHSPSP	828710	150980	4244
Q7Z4Q2	17	HEAT repeat-containing protein 3	HEATR3	0.574021	6.08E-12	109.56	T	GKSRTKRFKRPQFSPTGDCQAEAAAAANGTG	0	1580100	7809
Q13573	14	SNW domain-containing protein 1	SNW1	0.987908	1.15E-05	109.54	S	__MALTSFLPAPTQLSQDQLEAEEKARSQRS	285950	599410	2580
Q96D71	540	RalBP1-associated Eps domain-containing protein 1	REPS1	0.940707	1.62E-06	109.54	S	QIGSNVTRQRSHSGTSPDNTAPPPPPPRPQP	2929000	550020	4846
Q96D71	556	RalBP1-associated Eps domain-containing protein 1	REPS1	0.582431	1.62E-06	109.54	S	PDNTAPPPPPPRPQPSHSRSSSLDMNRTFTV	2929000	550020	4847
Q96D71	539	RalBP1-associated Eps domain-containing protein 1	REPS1	0.765712	1.62E-06	109.54	T	EQIGSNVTRQRSHSGTSPDNTAPPPPPPRPQ	2235900	0	7976
Q9H063	68	Repressor of RNA polymerase III transcription MAF1 homolog	MAF1	0.47404	4.65E-07	109.46	S	PHVLEALSPPQTSGLSPSRLSKSQGGEEEGP	0	0	5557
Q9H063	70	Repressor of RNA polymerase III transcription MAF1 homolog	MAF1	0.47404	4.65E-07	109.46	S	VLEALSPPQTSGLSPSRLSKSQGGEEEGPLS	0	0	5558
P35568	1078	Insulin receptor substrate 1	IRS1	1	0.00659169	109.44	S	PQGPGGMSAFTRVNLSPNRNQSAKVIRADPQ	496980	0	1393
Q15149	1435	Plectin	PLEC	1	0.000767872	109.44	S	ELQLVTYKAQLEPVASPAKKPKVQSGSESVI	0	1382600	2874
Q96G61;Q8	154;154	Diphosphoinositol polyphosphate phosphohydrolase 3-beta;Dipl	NUDT11;NUD1	0.471429	9.72E-05	109.44	S	YLEKLKLGGSPTNGNSMAPSSPDSDP_____	0	0	4400
Q9UQ35	2684	Serine/arginine repetitive matrix protein 2	SRRM2	1	0.008969	109.44	S	PPGERRSRSPRKPIDSLRDSRSLSYSPVERR	0	1696200	6646
O15400	126	Syntaxin-7	STX7	0.604257	0.00820358	109.42	S	AAEREKEFVARVRASSRVSGSFPEDSSKERN	638210	558120	315
O15400	129	Syntaxin-7	STX7	0.898538	0.00820358	109.42	S	REKEFVARVRASSRVSGSFPEDSSKERNLVS	638210	558120	316
P24928	1845	DNA-directed RNA polymerase II subunit RPB1	POLR2A	0.405622	3.29E-05	109.42	S	PSYSPASPKYTPTSPSYSPSSPEYTPTSPKY	0	0	1254
P16949;Q9	46;80	Stathmin;Stathmin-2	STMN1;STMN2	1	0.00134334	109.39	S	SEAPRTLASPKKKDLSLEEIQKKLEAAEERR;SVPI	726490	829880	1095
Q8TB72	182	Pumilio homolog 2	PUM2	0.99999	0.000948738	109.39	S	ADCKDFNRTPGSRQASPTEVVERLGPNTNPS	480070	763310	4449
Q9Y520	2685	Protein PRRC2C	PRRC2C	0.524837	0.00738035	109.39	S	KPGTPPIAGRSTTPTSSPFRATSTSPNSQSS	0	0	6949
O60885	601	Bromodomain-containing protein 4	BRD4	0.97466	0.00978208	109.36	S	KEPAPMKSKPPPTYESEEEDKCKPMSYEEKR	717420	371500	539
O95831	268	Apoptosis-inducing factor 1, mitochondrial	AIFM1	0.99995	0.00663839	109.29	S	YEKCLIATGGTPRSLSAIDRAGAEVKSRTTL	1920000	1193400	834
P35269	389	General transcription factor IIF subunit 1	GTF2F1	0.989931	0.000185606	109.28	T	RKPSGGSSRGNSRPGTPSAEGGSTSSTLRAA	69061000	24921000	7361
P55199	309	RNA polymerase II elongation factor ELL	ELL	0.943067	2.07E-06	109.25	S	QPQSTGSLLGDPAASSPPGERGRSASPPQKR	2191000	2357800	1878
O15047	468	Histone-lysine N-methyltransferase SETD1A	SETD1A	0.999927	1.65E-06	109.23	S	REEVRTSPRPASPARSGSPAPETTNESVPFA	6572700	5542200	268
O15047	470	Histone-lysine N-methyltransferase SETD1A	SETD1A	0.999929	1.65E-06	109.23	S	EVRTSPRPASPARSGSPAPETTNESVPFAQH	6572700	5542200	269
Q8IX01	277	SURP and G-patch domain-containing protein 2	SUGP2	0.891653	1.54E-06	109.22	S	TPKTQGTNQIQKNTPSPDVTLGTNPGTEDIQ	1734100	0	4089
Q8IXQ4	141	GPALPP motifs-containing protein 1	GPALPP1	0.797947	2.09E-06	109.22	S	SDKGRDDPGQQETDSSEDEDIIGPMPAKGPV	1830200	78038	4110
Q8WUA4	132	General transcription factor 3C polypeptide 2	GTF3C2	0.983715	1.80E-07	109.19	S	PNPPSAPLVPGLLDQSNPLSTPMPKKRGRKS	533200	289340	4506
Q96QU8	208	Exportin-6	XPO6	0.615993	3.18E-09	109.17	S	TVWDKHSVTAATPPPSPTSGESGDLLSNLLQ	1359000	1064400	5084
Q96QU8	210	Exportin-6	XPO6	0.45794	3.18E-09	109.17	T	WDKHSVTAATPPPSPTSGESGDLLSNLLQSP	0	0	7999
Q2NKX8	1069	DNA excision repair protein ERCC-6-like	ERCC6L	1	0.00180628	109.16	S	SSVKQFDASTPKNDISPPGRFFSSQIPSSVN	1031300	742340	3047
Q9H7F4;Q8	333;333	Transmembrane protein 185B;Transmembrane protein 185A	TMEM185B;TM	0.999123	0.00136514	109.16	S	IAPIFGKKARVVITQSPGKYVPPPPKLNIDM	1063300	1017600	4391
Q9UKV3	655	Apoptotic chromatin condensation inducer in the nucleus	ACIN1	1	0.00574698	109.16	S	RSRSRSRSASSNSRKSLSPGVSRDSSTSYTE	1923400	2024700	6455
Q9UKV3	657	Apoptotic chromatin condensation inducer in the nucleus	ACIN1	0.999997	0.00574698	109.16	S	RSRSRSASSNSRKSLSPGVSRDSSTSYTETK	3660700	3155700	6456
Q8IY81	573	pre-rRNA processing protein FTSJ3	FTSJ3	0.99996	0.00349753	109.16	T	RRKGRQQQQKQQLPQTPPSCLKTEIMSPLYQ	995780	649360	7857
O60784	461	Target of Myb protein 1	TOM1	0.844765	0.000109101	109.13	S	FLEERAKAADRLPNLSSPSAEGPPGPPSGPA	3302400	2475600	521
P42166	168	Lamina-associated polypeptide 2, isoform alpha;Thymopoietin;	TMPO	0.890836	0.00179708	109.1	S	GTESRSSTPLPTISSSAENTRQNGSNDSDRY	0	804540	1497
Q8WUA7	132	TBC1 domain family member 22A	TBC1D22A	0.997155	0.00113741	109.1	S	GPGLQQKPRPEAEPPSPPSGDLRLVKSVSES	2542700	1766700	4508
O75152	108	Zinc finger CCCH domain-containing protein 11A	ZC3H11A	0.999966	0.00220419	109.07	S	LFLPPSKTVLPTVPESPEEEVKASQLSVQQN	2123600	3868300	575
Q7Z3C6	656	Autophagy-related protein 9A	ATG9A	0.980911	0.00222851	109.07	S	GSRHRAEVASALRSFSPLQPGQAPTGRAHST	1961000	883570	3779
Q13428	503	Treacle protein	TCOF1	1	0.00622292	109.04	S	ALAAMNAAQVKPLGKSPQVKPASTMGMGPLG	2087200	752820	2520
O95613	2352	Pericentrin	PCNT	0.565971	1.65E-06	108.99	S	DRSEKSDGSGFGARLSPGSGGPEAQTAGPVT	1791200	1041400	781
P39880	1216	Homeobox protein cut-like 1	CUX1	0.808386	6.39E-09	108.99	S	MKRMEKKAYMKRRHSSVSDSQPCEPPSVGTE	0	1020300	1463
P39880	1232	Homeobox protein cut-like 1	CUX1	0.874153	6.39E-09	108.99	Y	VSDSQPCEPPSVGTEYSQGASPQPQHQLKKP	769540	0	8363
Q03164	3527	Histone-lysine N-methyltransferase 2A;MLL cleavage product N	KMT2A	0.967348	0.000800302	108.98	S	PGGSPSSPSSGQRSASPSVPGPTKPKPKTKR	1071700	572160	2144
Q8TEQ6	778	Gem-associated protein 5	GEMIN5	0.999999	5.26E-05	108.97	S	EESMKENSGPVENGVSDQEGEEQAREPELPC	627670	549140	4485
Q9BQE9	126	B-cell CLL/lymphoma 7 protein family member B	BCL7B	0.855684	8.58E-07	108.96	T	SSPSPQQSESLSPAHTSDFRTDDSQPPTLGQ	0	1143800	8041
Q12767	221	Uncharacterized protein KIAA0195	KIAA0195	1	2.45E-06	108.92	S	DEHIVLEPGDLFPPFSPPPSPRGEVERGPQS	724160	663680	2291
Q12767	225	Uncharacterized protein KIAA0195	KIAA0195	1	2.45E-06	108.92	S	VLEPGDLFPPFSPPPSPRGEVERGPQSPQQH	724160	663680	2292
Q7Z6J9	230	tRNA-splicing endonuclease subunit Sen54	TSEN54	0.813152	2.55E-07	108.77	S	KALDNSLQPKSLAASSPPPCSQPSQCPEEKP	2779500	1732900	3862
Q7Z6J9	248	tRNA-splicing endonuclease subunit Sen54	TSEN54	0.5	2.55E-07	108.77	S	PCSQPSQCPEEKPQESSPMKGPGGPFQLLGS	2779500	1732900	3863
Q7Z6J9	249	tRNA-splicing endonuclease subunit Sen54	TSEN54	0.886153	2.55E-07	108.77	S	CSQPSQCPEEKPQESSPMKGPGGPFQLLGSL	2779500	1732900	3864
O15234	265	Protein CASC3	CASC3	1	0.00681043	108.74	S	PDDIKPRRIRKPRYGSPPQRDPNWNGERLNK	1300900	0	308
Q14676	966	Mediator of DNA damage checkpoint protein 1	MDC1	0.453131	1.75E-09	108.71	T	PEGGSQDQKGQASSPTPEPGVGAGDLPGPTS	0	0	7603
P51513	154	RNA-binding protein Nova-1	NOVA1	0.727053	0.000820679	108.7	S	QTTVNPDRIKQTLPSSPTTTKSSPSDPMTTS	1362800	791820	1778
Q16512	561	Serine/threonine-protein kinase N1	PKN1	0.461502	0.00151397	108.7	S	GDISVEKLNLGTDSDSSPQKSSRDPPSSPSS	0	0	3007
Q58EX7	64	Puratrophin-1	PLEKHG4	0.998657	2.28E-06	108.69	S	PRPPAGATQDEELQGSPLSRKFQLPPAADES	2306500	1398300	3149
Q15059	263	Bromodomain-containing protein 3	BRD3	0.988287	0.00456903	108.68	S	TTTPTTSAITASRSESPPPLSDPKQAKVVAR	1109900	929100	2871
Q8WUA4	165	General transcription factor 3C polypeptide 2	GTF3C2	0.620858	0.00370272	108.68	S	ELLLLKLSKDLDRPESQSPKRPPEDFETPSG	799820	515770	4504
P07195	44	L-lactate dehydrogenase B chain	LDHB	1	0.00941155	108.66	S	VGQVGMACAISILGKSLADELALVDVLEDKL	182210	219400	924
P38398	1266	Breast cancer type 1 susceptibility protein	BRCA1	0.999806	0.00231331	108.66	S	CLSKNTEENLLSLKNSLNDCSNQVILAKASQ	0	576630	1454
Q6ICG6	289	Uncharacterized protein KIAA0930	KIAA0930	0.92744	0.00830146	108.66	S	DSSPASPMHERVTSFSTPPTPERNNRPAFFS	1500300	725390	3473
Q8N201	307	Integrator complex subunit 1	INTS1	1	0.000910193	108.66	S	EEDSQTELLIAEEKLSPEQEGQLMPRYEELA	2356000	1615600	4238
Q6ICG6	293	Uncharacterized protein KIAA0930	KIAA0930	0.999077	0.00830146	108.66	T	ASPMHERVTSFSTPPTPERNNRPAFFSPSLK	1500300	1395000	7751
P46821	1302	Microtubule-associated protein 1B;MAP1B heavy chain;MAP1 lig	MAP1B	0.860994	1.99E-07	108.64	T	KVSAEAEVAPVSPEVTQEVVEEHCASPEDKT	0	0	7395
P38159;Q9	208;208	RNA-binding motif protein, X chromosome;RNA-binding motif pr	RBMX;RBMXL1	1	0.00816744	108.57	S	PPRREPLPSRRDVYLSPRDDGYSTKDSYSSR	23842000	12379000	1446
Q86WB0	56	Nuclear-interacting partner of ALK	ZC3HC1	0.813455	1.33E-06	108.5	S	APEEGGVDAKDTSATSQSVNGSPQAEQPSLE	0	1410300	3976
Q86WB0	58	Nuclear-interacting partner of ALK	ZC3HC1	0.981993	1.33E-06	108.5	S	EEGGVDAKDTSATSQSVNGSPQAEQPSLEST	0	1410300	3977
Q14168	138	MAGUK p55 subfamily member 2	MPP2	0.726914	9.02E-07	108.5	T	HFQSLLETHDSVASKTYETPPPSPGLDPTFS	0	1661000	7585
Q8N9B5	713	Junction-mediating and -regulatory protein	JMY	1	1.88E-06	108.49	S	STRLRLAHARRKGAASPVLQEDHCDSLPSVL	2392100	1110100	4300
Q9P2E9	1277	Ribosome-binding protein 1	RRBP1	0.9998	1.43E-07	108.49	S	KSHVEDGDIAGAPASSPEAPPAEQDPVQLKT	5851100	3558100	6238
Q7Z422	39	SUZ domain-containing protein 1	SZRD1	0.999914	3.30E-06	108.45	S	EKKLKITQKESRKSKSPPKVPIVIQDDSLPA	1648300	2910000	3802
Q9H2G9	15	Golgin-45	BLZF1	0.780403	0.00143309	108.43	S	_MTTKNLETKVTVTSSPIRGAGDGMETEEPP	0	510480	5613
Q13542	37	Eukaryotic translation initiation factor 4E-binding protein	EIF4EBP2	0.976507	6.68E-09	108.38	T	VAISDAAQLPHDYCTTPGGTLFSTTPGGTRI	120020000	88120000	7561
Q13542	46	Eukaryotic translation initiation factor 4E-binding protein	EIF4EBP2	0.919985	6.68E-09	108.38	T	PHDYCTTPGGTLFSTTPGGTRIIYDRKFLLD	12841000	11762000	7563
A0FGR8	753	Extended synaptotagmin-2	ESYT2	0.860372	0.000140207	108.37	T	SSLLASPGHISVKEPTPSIASDISLPIATQE	0	0	7106
P62942	9	Peptidyl-prolyl cis-trans isomerase FKBP1A	FKBP1A	0.997421	0.00156583	108.36	S	_______MGVQVETISPGDGRTFPKRGQTCV	0	788990	1927
Q9BSJ6	16	Protein FAM64A	FAM64A	1	0.000942257	108.36	S	MASRWQNMGTSVRRRSLQHQEQLEDSKELQP	207600	212240	5342
P27816	94	Microtubule-associated protein 4	MAP4	0.985991	3.41E-07	108.32	S	SKPTLLANGGHGVEGSDTTGSPTEFLEEKMA	2915400	1771800	1295
Q96DY7	639	Mdm2-binding protein	MTBP	0.998754	0.000945709	108.32	S	PIQKGEKTFVLTPELSPGKLQVLPFEKASVC	1079400	662150	4866
O60343	341	TBC1 domain family member 4	TBC1D4	0.999986	0.00117896	108.25	S	VHEGSQKSQPRRRHASAPSHVQPSDSEKNRT	3999500	1588000	488
Q4VCS5	200	Angiomotin	AMOT	0.999875	5.93E-05	108.25	S	TSGVKAHPPVTSAPLSPPQPNDLYKNPTSSS	2466400	1649700	3111
O60341	166	Lysine-specific histone demethylase 1A	KDM1A	0.999958	6.14E-09	108.19	S	LPPPPPQAPPEEENESEPEEPSGVEGAAFQS	9159600	4262800	485
Q7L2J0	254	7SK snRNA methylphosphate capping enzyme	MEPCE	0.991805	2.04E-06	108.17	S	LSLNTCTDEGHVVLASPLKTGRKRHRHRGQH	3545900	3098500	3724
Q5T4S7	2885	E3 ubiquitin-protein ligase UBR4	UBR4	0.499291	8.71E-09	108.05	S	DEGSTAATDGSTLRTSPADHGGSVGSESGGS	0	0	3288
Q5T4S7	2884	E3 ubiquitin-protein ligase UBR4	UBR4	0.499291	8.71E-09	108.05	T	DDEGSTAATDGSTLRTSPADHGGSVGSESGG	0	0	7723
P46087	786	Probable 28S rRNA (cytosine(4447)-C(5))-methyltransferase	NOP2	0.998516	0.000870463	107.99	S	QKQNDTPKGPQPPTVSPIRSSRPPPAKRKKS	4987900	2775200	1545
Q4KMP7	678	TBC1 domain family member 10B	TBC1D10B	1	0.00162112	107.99	S	PGLKSRGSRAAGGAPSPPPPVRRASAGPAPG	7780900	4649600	3096
Q96N67	929	Dedicator of cytokinesis protein 7	DOCK7	0.999426	0.000870463	107.99	S	EVRSIIGSKGLDRSNSWVNTGGPKAAPWGSN	0	1036800	5038
O75446	131	Histone deacetylase complex subunit SAP30	SAP30	0.999993	5.34E-09	107.94	S	KNLIQSVRNRRKRKGSDDDGGDSPVQDIDTP	3436400	2244400	628
O75446	138	Histone deacetylase complex subunit SAP30	SAP30	0.999868	5.34E-09	107.94	S	RNRRKRKGSDDDGGDSPVQDIDTPEVDLYQL	3436400	2244400	629
P55199	308	RNA polymerase II elongation factor ELL	ELL	0.5	2.93E-06	107.92	S	CQPQSTGSLLGDPAASSPPGERGRSASPPQK	0	0	1877
Q9H8Y8	222	Golgi reassembly-stacking protein 2	GORASP2	1	0.0049678	107.91	T	FEEGKKISLPGQMAGTPITPLKDGFTEVQLS	0	279440	8123
Q9H8Y8	225	Golgi reassembly-stacking protein 2	GORASP2	1	0.0049678	107.91	T	GKKISLPGQMAGTPITPLKDGFTEVQLSSVN	0	279440	8124
Q06124	558	Tyrosine-protein phosphatase non-receptor type 11	PTPN11	0.451711	3.54E-07	107.88	S	GHEYTNIKYSLADQTSGDQSPLPPCTPTPPC	0	0	2196
Q14694	215	Ubiquitin carboxyl-terminal hydrolase 10	USP10	0.428753	7.75E-08	107.87	S	MPPSVTPRTCNSPQNSTDSVSDIVPDSPFPG	0	0	2803
Q14694	216	Ubiquitin carboxyl-terminal hydrolase 10	USP10	0.428753	7.75E-08	107.87	T	PPSVTPRTCNSPQNSTDSVSDIVPDSPFPGA	0	0	7619
Q9NQX3	270	Gephyrin;Molybdopterin adenylyltransferase;Molybdopterin mol	GPHN	0.998205	0.00250026	107.74	S	VLPRDTASLSTTPSESPRAQATSRLSTASCP	806220	552370	5935
P08651	339	Nuclear factor 1 C-type	NFIC	0.986284	0.00061335	107.73	S	PVKKTEMDKSPFNSPSPQDSPRLSSFTQHHR	1929200	1220000	962
A0FGR8	699	Extended synaptotagmin-2	ESYT2	0.499834	1.76E-08	107.7	S	TSIKSHMSGSPGPGGSNTAPSTPVIGGSDKP	0	0	6
A0FGR8	701	Extended synaptotagmin-2	ESYT2	0.499834	1.76E-08	107.7	T	IKSHMSGSPGPGGSNTAPSTPVIGGSDKPGM	0	0	7107
A0FGR8	705	Extended synaptotagmin-2	ESYT2	0.953869	1.76E-08	107.7	T	MSGSPGPGGSNTAPSTPVIGGSDKPGMEEKA	1193200	582170	7108
Q13404	146	Ubiquitin-conjugating enzyme E2 variant 1	UBE2V1	0.999778	0.00101269	107.69	S	KENMKLPQPPEGQCYSN______________	0	0	2480
Q96PK6	206	RNA-binding protein 14	RBM14	1	0.00613605	107.66	T	FGNSTGGFDGQARQPTPPFFGRDRSPLRRSP	4088300	4484500	7996
Q13586	618	Stromal interaction molecule 1	STIM1	0.528046	0.0085794	107.65	S	APPGGSPHLDSSRSHSPSSPDPDTPSPVGDS	0	0	2587
Q7Z2Z1	599	Treslin	TICRR	1	0.00167428	107.65	S	VARLNVKAQKLHPDGSPDVAGEKGIQKIPSG	1057600	665430	3766
Q12948	272	Forkhead box protein C1	FOXC1	0.959145	1.53E-06	107.57	S	IESPDSSSSSLSSGSSPPGSLPSARPLSLDG	0	662400	2382
Q58WW2	657	DDB1- and CUL4-associated factor 6	DCAF6	0.995748	6.04E-05	107.57	S	EETSTRDSALQDTDDSDDDPVLIPGARYRAG	1509400	1464200	3152
Q58WW2	654	DDB1- and CUL4-associated factor 6	DCAF6	0.995748	6.04E-05	107.57	T	SAHEETSTRDSALQDTDDSDDDPVLIPGARY	1509400	1464200	7698
P15924	2610	Desmoplakin	DSP	0.263037	1.54E-06	107.56	S	TISSVRNLTIRSSSFSDTLEESSPIAAIFDT	0	0	1081
Q05209	435	Tyrosine-protein phosphatase non-receptor type 12	PTPN12	1	0.00718371	107.55	S	ESTIEQIDKKLERNLSFEIKKVPLQEGPKSF	643840	432350	2185
Q9UHY1	431	Nuclear receptor-binding protein	NRBP1	0.970906	0.00419709	107.53	T	QQEEVTSPVVPPSVKTPTPEPAEVETRKVVL	0	1563800	8220
Q9P0K7	327	Ankycorbin	RAI14	0.300536	8.44E-08	107.49	S	FKAEISSIRENKDRLSDSTTGADSLLDISSE	0	0	6181
Q9P0K7	329	Ankycorbin	RAI14	0.310904	8.44E-08	107.49	S	AEISSIRENKDRLSDSTTGADSLLDISSEAD	0	0	6182
Q9P0K7	330	Ankycorbin	RAI14	0.310904	8.44E-08	107.49	T	EISSIRENKDRLSDSTTGADSLLDISSEADQ	0	0	8187
Q9P0K7	331	Ankycorbin	RAI14	0.310904	8.44E-08	107.49	T	ISSIRENKDRLSDSTTGADSLLDISSEADQQ	0	0	8188
O94806;Q1	391;397	Serine/threonine-protein kinase D3;Serine/threonine-protein	PRKD3;PRKD1	0.962129	0.00255513	107.46	S	MQDPDPDHEDANRTISPSTSNNIPLMRVVQS;SDLI	1670700	802200	697
Q5T5U3	1605	Rho GTPase-activating protein 21	ARHGAP21	0.999999	0.00103615	107.46	S	TSSATYLTSLDSSRLSPEVQSVAESKGDEAD	425330	1129900	3289
Q9H788	315	SH2 domain-containing protein 4A	SH2D4A	0.972576	0.00103615	107.46	S	YPQKPLRNQGVVRTLSSSAQEDIIRWFKEEQ	363920	0	5748
P68363;Q9	48;48;48	Tubulin alpha-1B chain;Tubulin alpha-1C chain;Tubulin alpha-	TUBA1B;TUBA	0.845956	0.000135322	107.41	S	DGQMPSDKTIGGGDDSFNTFFSETGAGKHVP	2636300	1964700	1946

Q96C92	243	Serologically defined colon cancer antigen 3	SDCCAG3	0.999709	2.58E-06	107.41	S	PSWALSDTDSRVSPASPAGSPSADFAVHGES	6134800	6256000	4822
Q96C92	247	Serologically defined colon cancer antigen 3	SDCCAG3	0.883993	2.58E-06	107.41	S	LSDTDSRVSPASPAGSPSADFAVHGESLGDR	6134800	6256000	4823
P35580	1952	Myosin-10	MYH10	1	0.000362042	107.39	S	SSSRSGRRQLHLEGASLELSDDDTESKTSDV	1652900	842460	1395
P27348	229	14-3-3 protein theta	YWHAQ	0.552297	5.13E-06	107.39	T	STLIMQLLRDNLTLWTSDSAGEECDAAEGAE	0	4028200	7329
O14654	427	Insulin receptor substrate 4	IRS4	1	0.00108575	107.34	S	GRLHLPRGRRSRRAVSVPASFFRRLAPSPAR	1311400	789280	173
Q5SW79	1133	Centrosomal protein of 170 kDa	CEP170	0.602424	0.000253115	107.34	S	DADKASVASEVSTTSSTSKPPTGRRNISRID	2883500	0	3214
Q7Z309	50	Protein FAM122B	FAM122B	0.997987	0.00630342	107.34	S	LSQVFQPYTLRTRRNSTTIMSRHSLEEGLDM	1482600	886350	3775
Q9BWT3	648	Poly(A) polymerase gamma	PAPOLG	0.999652	0.000916534	107.34	S	GMSNITKTVTPKRSHSPSIDGTPKRLKDVEK	0	0	5432
Q9H6F5	113	Coiled-coil domain-containing protein 86	CCDC86	0.974758	0.00108575	107.34	S	LHLESPQRQPEYSPESPRCQPKPSEEAPKCS	0	1651200	5732
Q9P1Y6	991	PHD and RING finger domain-containing protein 1	PHRF1	1	0.00691852	107.34	S	VLQAATHRVVELRPPSRSRSTSSSRSRKKAK	3108000	1055300	6206
Q5SW79	1130	Centrosomal protein of 170 kDa	CEP170	0.417144	0.000253115	107.34	T	ELADADKASVASEVSTTSSTSKPPTGRRNIS	0	0	7709
Q5SW79	1134	Centrosomal protein of 170 kDa	CEP170	0.440732	0.000253115	107.34	T	ADKASVASEVSTTSSTSKPPTGRRNISRIDL	0	0	7711
P49006	178	MARCKS-related protein	MARCKSL1	0.642447	6.78E-12	107.31	T	AASEEEAGPQATEPSTPSGPESGPTPASAEQ	11825000	14468000	7407
O75533	129	Splicing factor 3B subunit 1	SF3B1	1	0.00213091	107.29	S	DREDEYKKHRRTMIISPERLDPFADGGKTPD	5441000	2256500	640
Q12857	300	Nuclear factor 1 A-type	NFIA	0.938522	1.62E-06	107.29	S	MDSPGEEPFYTGQGRSPGSGSQSSGWHEVEP	6099800	1855300	2318
Q9Y4R8	642	Telomere length regulation protein TEL2 homolog	TELO2	0.567571	1.03E-08	107.29	T	GCLGRTPQPGSPSPNTPCLPEAAVSQPGSAV	0	0	8305
Q9Y2D5	748	A-kinase anchor protein 2	AKAP2	0.627012	0.00109504	107.21	S	EEELKRQRQVLQSTQSPRTKNAPSLPSRTCY	915780	0	6777
Q8WWM7	632	Ataxin-2-like protein	ATXN2L	0.483755	2.06E-09	107.18	T	GTEGPEQPPPPCPSQTGSPPVGLIKGEDKDE	0	0	7923
Q9NQS7	311	Inner centromere protein	INCENP	0.608583	0.000902302	107.15	S	SRTDSQSVRHSPIAPSSPSPQVLAQKYSLVA	0	3285100	5920
O00257	432	E3 SUMO-protein ligase CBX4	CBX4	0.48228	0.00217746	107.13	S	THGEREPCKKRLTARSISTPTCLGGSPAAER	0	0	109
Q96GR2	56	Long-chain-fatty-acid--CoA ligase ACSBG1	ACSBG1	1	0.00218085	107.13	S	LKTSSLTDRQPLSKESLNHALELSVPEKVNN	1122700	1242400	4925
Q96GR2	64	Long-chain-fatty-acid--CoA ligase ACSBG1	ACSBG1	1	0.00218085	107.13	S	RQPLSKESLNHALELSVPEKVNNAQWDAPEE	1122700	1242400	4926
Q8IV50	21	LysM and putative peptidoglycan-binding domain-containing p	LYSMD2	1	0.00129397	107.11	S	PALSLREGGPRAPRPSAPSPPPRSRSGSESE	4095500	4007700	4039
Q8IV50	24	LysM and putative peptidoglycan-binding domain-containing p	LYSMD2	1	0.00129397	107.11	S	SLREGGPRAPRPSAPSPPPRSRSGSESEEAE	4095500	4007700	4040
Q9Y613	367	FH1/FH2 domain-containing protein 1	FHOD1	1	0.00110118	107.11	S	ERRKPSSEEGKRSRRSLEGGGCPARAPEPGP	0	0	6976
Q12888	1759	Tumor suppressor p53-binding protein 1	TP53BP1	0.935658	3.92E-05	107.09	S	KLASRSKLPDGPTGSSEEEEEFLEIPPFNKQ	1781100	1695800	2350
P52732	926	Kinesin-like protein KIF11	KIF11	0.989255	0.0038097	107.09	T	CFLEQDLKLDIPTGTTPQRKSYLYPSTLVRT	6478000	0	7444
Q12888	1756	Tumor suppressor p53-binding protein 1	TP53BP1	0.893206	8.17E-06	107.09	T	TSDKLASRSKLPDGPTGSSEEEEEFLEIPPF	1781100	1695800	7512
Q6PKG0	849	La-related protein 1	LARP1	0.566707	4.51E-07	107.07	S	WVMDSREHRPRTASISSSPSEGTPTVGSYGC	6026900	1088600	3564
Q6PKG0	851	La-related protein 1	LARP1	0.441954	4.51E-07	107.07	S	MDSREHRPRTASISSSPSEGTPTVGSYGCTP	0	0	3565
Q6PKG0	853	La-related protein 1	LARP1	0.441208	4.51E-07	107.07	S	SREHRPRTASISSSPSEGTPTVGSYGCTPQS	0	0	3566
Q9BTC0	811	Death-inducer obliterator 1	DIDO1	0.859764	2.75E-06	107.07	S	QEAIPDLEDSPPVSDSEEQQESARAVPEKST	4239500	0	5358
Q14160	688	Protein scribble homolog	SCRIB	1	9.59E-06	107.06	S	EVEEEEENRAEEEEASTEEEDKEGAVVSAPS	5461100	3601600	2673
Q14160	689	Protein scribble homolog	SCRIB	1	9.59E-06	107.06	T	VEEEEENRAEEEEASTEEEDKEGAVVSAPSV	5461100	3601600	7580
P51610	2020	Host cell factor 1;HCF N-terminal chain 1;HCF N-terminal cha	HCFC1	0.702178	0.00642575	107.03	S	KDSSGTKPANKRPMSSPEMKSAPKKSKADGQ	2381000	1493600	1791
Q9UER7	668	Death domain-associated protein 6	DAXX	0.999183	3.77E-06	107.02	S	RSVHEKNGKKICTLPSPPSPLASLAPVADSS	1418700	1176500	6287
Q9UER7	671	Death domain-associated protein 6	DAXX	0.999123	3.77E-06	107.02	S	HEKNGKKICTLPSPPSPLASLAPVADSSTRV	2298800	1828400	6288
Q96TA1	665	Niban-like protein 1	FAM129B	1	8.91E-09	106.98	S	VTEIRGLLAQGLRPESPPPAGPLLNGAPAGE	3695400	0	5188
Q07157	284	Tight junction protein ZO-1	TJP1	0.998014	4.88E-05	106.94	S	LNVPDLSDSIHSANASERDDISEIQSLASDH	2981200	9537500	2219
P49006	104	MARCKS-related protein	MARCKSL1	0.999997	0.00739556	106.88	S	KKKFSFKKPFKLSGLSFKRNRKEGGGDSSAS	11003000	2595900	1640
Q86YS7	295	C2 domain-containing protein 5	C2CD5	0.969625	0.00111682	106.88	S	SSGPSTPLKNQTYSFSPSKSYSRQSSSSDTD	4278800	3425100	4031
Q96T37	622	Putative RNA-binding protein 15	RBM15	1	0.00739556	106.88	S	AYEPLDSLDRRRDGWSLDRDRGDRDLPSSRD	2266300	1549400	5155
Q8IXK0	621	Polyhomeotic-like protein 2	PHC2	0.998931	3.60E-06	106.86	S	LPEKLPQQDHTTTTDSEMEEPYLQESKEEGA	1025600	718560	4101
O95292	206	Vesicle-associated membrane protein-associated protein B/C	VAPB	0.584248	0.00223681	106.82	S	KEEDGLRMRKTVQSNSPISALAPTGKEEGLS	623140	251880	745
P36915	55	Guanine nucleotide-binding protein-like 1	GNL1	0.702701	0.00174593	106.82	S	SRERREEQTDTSDGESVTHHIRRLNQQPSQG	0	471570	1433
Q92922	573	SWI/SNF complex subunit SMARCC1	SMARCC1	1	0.0107822	106.82	S	VLADTPSGLVPLHLRSPQVPAAQQMLNFPEK	559190	555590	4731
Q7Z5J4	892	Retinoic acid-induced protein 1	RAI1	1	0.00112318	106.79	S	LLGSPEQRPGMQDPLSPKAPLICTKEEVEEV	864600	393720	3840
Q86UE4	308	Protein LYRIC	MTDH	0.787711	0.000956109	106.79	S	LSSQISAGEEKWNSVSPASAGKRKTEPSAWS	108360	1124900	3927
P20700	20	Lamin-B1	LMNB1	0.998927	0.00451639	106.79	T	TPVPPRMGSRAGGPTTPLSPTRLSRLQEKEE	11597000	7945900	7310
Q9UQ35	2291	Serine/arginine repetitive matrix protein 2	SRRM2	0.961657	0.00736126	106.79	T	AVAPSAVNLADPRTPTAPAVNLAGARTPAAL	0	0	8277
Q13263	594	Transcription intermediary factor 1-beta	TRIM28	0.92794	4.81E-12	106.76	S	MALAEGPGAEGPRLASPSGSTSSGLEVVAPE	8475500	67613	2452
Q8NFA0	1422	Ubiquitin carboxyl-terminal hydrolase 32	USP32	0.565924	0.00500605	106.76	S	GRLRLPQIGSKNKLSSSKENLDASKENGAGQ	0	0	4390
Q9UQ35	2343	Serine/arginine repetitive matrix protein 2	SRRM2	0.999997	0.0026988	106.76	S	RTPQAPASANLVGPRSAHATAPVNIAGSRTA	3623400	1753900	6680
P01106;P(58;58		Myc proto-oncogene protein;N-myc proto-oncogene protein	MYC;MYCN	0.994981	0.0140786	106.76	T	PAPSEDIWKKFELLPTPPLSPSRRSGLCSPS	872830	755050	7253
Q66K74	731	Microtubule-associated protein 1S;MAP1S heavy chain;MAP1S li	MAP1S	0.999937	7.34E-05	106.73	S	GLSLPLRGPRARRSASPHDVDLCLVSPCEFE	2580900	1084600	3410
Q8IYB3	778	Serine/arginine repetitive matrix protein 1	SRRM1	0.821377	4.04E-06	106.72	T	KPPAPPSPVQSQSPSTNWSPAVPVKKAKSPT	3440400	7896300	7862
O43524	7	Forkhead box protein O3	FOXO3	1	7.96E-07	106.69	S	__________MAEAPASPAPLSPLEVELDPEF	0	0	398
O43524	12	Forkhead box protein O3	FOXO3	1	7.96E-07	106.69	S	_____MAEAPASPAPLSPLEVELDPEFEPQSR	588730	974660	399
Q96T23	622	Remodeling and spacing factor 1	RSF1	0.999956	4.25E-07	106.62	S	PEEVPKSTLESEKPGSPEAAETSPPSNIIDH	5869400	3444500	5150
P35612	596	Beta-adducin	ADD2	1	0.000139692	106.6	S	GEKETAPEEPGSPAKSAPASPVQSPAKEAET	695790	2342400	1410
P35612	600	Beta-adducin	ADD2	0.999757	0.000139692	106.6	S	TAPEEPGSPAKSAPASPVQSPAKEAETKSPL	695790	2342400	1411
Q9NQC3	12	Reticulon-4	RTN4	0.317173	2.87E-06	106.57	S	_____MEDLDQSPLVSSSDSPPRPQPAFKYQF	0	0	5910
P28749	1041	Retinoblastoma-like protein 1	RBL1	1	0.0018414	106.55	S	QRTKKRVIAIDSDAESPAKRVCQENDDVLLK	567440	1230600	1314
Q04759	695	Protein kinase C theta type	PRKCQ	1	0.000972803	106.55	S	RALINSMDQNMFRNFSFMNPGMERLIS_____	1444300	1093800	2182
Q9ULS5;O‹216;438		Transmembrane and coiled-coil domains protein 3;Transmembrar	TMCC3;TMCC2	1	0.000973288	106.54	S	NKSREFANLIRNKFGSADNIAHLKNSLEEFR	694840	334390	555
Q14669	991	E3 ubiquitin-protein ligase TRIP12	TRIP12	0.999999	0.000973288	106.54	S	GSPSLQHSRDDSLDLSPQGRLSDVLKRKRLP	1178100	0	2751
P62241	130	40S ribosomal protein S8	RPS8	1	0.0039683	106.54	T	SHYALPLGRKKGAKLTPEEEEILNKKRSKKI	88173	370560	7456
Q7Z589	1210	Protein EMSY	EMSY	0.848202	0.00229374	106.52	S	AGMANSTPQQQKCRESCSSPSTVGSSLTTRK	828570	0	3834
P49407	412	Beta-arrestin-1	ARRB1	0.999212	0.000709579	106.49	S	KGMKDDKEEEEDGTGSPQLNNR__________	0	0	1658
O00567	466	Nucleolar protein 56	NOP56	0.424763	1.75E-06	106.47	S	KKRLAALALASSENSSSTPEECEEMSEKPKK	0	0	136
A8CG34	322	Nuclear envelope pore membrane protein POM 121C	POM121C	0.526718	1.03E-06	106.43	S	QIFLDGQENKRRRHDSSGSGHSAFEPLVASG	0	2182000	54
P24928	1899	DNA-directed RNA polymerase II subunit RPB1	POLR2A	0.795145	0.00145943	106.42	S	TTPKYSPTSPTYSPTSPVYTPTSPKYSPTSP	862690	455940	1248
Q13595	204	Transformer-2 protein homolog alpha	TRA2A	0.913341	0.000905851	106.4	T	RIRVDYSITKRAHTPTPGIYMGRPTHSGGGG	3899300	0	7565
P46379	113	Large proline-rich protein BAG6	BAG6	0.998311	7.79E-09	106.38	S	ASSGTGSASATHGGGSPPGTRGPGASVHDRN	9757400	7644200	1564
Q9UQ35	357	Serine/arginine repetitive matrix protein 2	SRRM2	0.573064	2.70E-06	106.38	S	KKEKSATRPSPSPERSSTGPEPPAPTPLLAE	3971300	2421000	6665
Q9UQ35	358	Serine/arginine repetitive matrix protein 2	SRRM2	0.588495	9.29E-05	106.38	S	KEKSATRPSPSPERSSTGPEPPAPTPLLAER	0	0	6666
Q7Z434	152	Mitochondrial antiviral-signaling protein	MAVS	0.981297	5.62E-09	106.36	S	EPSYPMPVQETQAPESPGENSEQALQTLSPR	2139000	1906100	3804
Q9Y3E7	200	Charged multivesicular body protein 3	CHMP3	1	4.51E-09	106.36	S	TDALPEPEPPGAMAASEDEEEEEEALEAMQS	2901700	5496700	6856
Q9Y294	175	Histone chaperone ASF1A	ASF1A	0.594262	1.92E-06	106.35	S	LEDAESSNPNLQSLLSTDALPSASKGWSTSE	2176300	631060	6773
Q14669	1577	E3 ubiquitin-protein ligase TRIP12	TRIP12	0.76609	0.010387	106.31	S	MQRLLDTNPEINQSDSQDSRVAPRLDRKKRT	570340	226040	2752
Q9NYZ3	583	G2 and S phase-expressed protein 1	GTSE1	0.813492	0.0107325	106.31	S	DSRLVDVSPDRGSPPSRVPQALNFSPEESDS	0	6952000	6144
O43164	309	E3 ubiquitin-protein ligase Praja-2	PJA2	0.795603	0.00118978	106.3	S	CSEQNTNDREKNHGSSPEQVVRPKVRKLISS	1224100	810180	342
P52948	673	Nuclear pore complex protein Nup98-Nup96;Nuclear pore comple	NUP98	1	0.00367823	106.3	S	FYTNPIAKPIPQTPESAGNKHSNSNSVDDTI	464930	1199000	1829
Q92597	326	Protein NDRG1	NDRG1	0.491296	0.00432926	106.3	S	GMGYMPSASMTRLMRSRTASGSSVTSLDGTR	0	0	4648
Q9H6Z4	126	Ran-binding protein 3	RANBP3	0.35206	0.011346	106.3	S	EDGNYCPPVKRERTSSLTQFPPSQSEERSSG	0	0	5746
O14683	14	Tumor protein p53-inducible protein 11	TP53I11	0.999915	0.000583548	106.29	S	__MAAKQPPPLMKKHSQTDLVSRLKTRKILG	823500	0	187
Q9UQ35	2042	Serine/arginine repetitive matrix protein 2	SRRM2	1	0.00326316	106.29	S	RRRSRSRTPLLPRKRSRSRSPLAIRRRSRSR	6104900	3344400	6712
Q8NI27	1419	THO complex subunit 2	THOC2	0.452509	0.00138992	106.26	S	EREQKRRKIDTHPSPSHSSTVKDSLIELKES	0	0	4424
Q8NI27	1421	THO complex subunit 2	THOC2	0.452509	0.00138992	106.26	S	EQKRRKIDTHPSPSHSSTVKDSLIELKESSA	0	0	4425
Q6ZRS2	568	Helicase SRCAP	SRCAP	0.999985	4.30E-06	106.23	S	YLLARDEEQSEADAGSGPPTPGPTTLGPKKE	5978000	3564500	3660
Q6ZRS2	572	Helicase SRCAP	SRCAP	0.999117	4.30E-06	106.23	T	RDEEQSEADAGSGPPTPGPTTLGPKKEITDI	2822700	1281400	7783
P16949	63	Stathmin	STMN1	1	9.43E-05	106.18	S	EEIQKKLEAAEERRKSHEAEVLKQLAEKREH	1805600	30300	1097
Q13547	409	Histone deacetylase 1	HDAC1	0.5	0.00676021	106.16	S	GDEDEDDPDKRISICSSDKRIACEEEFSDSE	197290	0	2577
Q13547	410	Histone deacetylase 1	HDAC1	0.5	0.00676021	106.16	S	DEDEDDPDKRISICSSDKRIACEEEFSDSEE	197290	0	2578
Q68CP4	243	Heparan-alpha-glucosaminide N-acetyltransferase	HGSNAT	0.990406	0.00100185	106.14	S	ISSRETDRLINSELGSPSRTDPLDGDVQPAT	946550	609320	3418
Q9UKA4	422	A-kinase anchor protein 11	AKAP11	0.99657	0.000426619	106.11	S	ALPANVRKPTPRKPESPYGNLCDAPDSPRPV	2607200	1770400	6433
Q9UKA4	433	A-kinase anchor protein 11	AKAP11	1	0.000426619	106.11	S	RKPESPYGNLCDAPDSPRPVKASREDSGLFS	2607200	1770400	6434
Q9Y2D5	778	A-kinase anchor protein 2	AKAP2	0.91985	4.65E-07	106.11	S	YKTAPGKIEKVKPPPSPTTEGPSLQPDLAPE	0	1643100	6779
Q02952	505	A-kinase anchor protein 12	AKAP12	0.989684	6.47E-05	106.06	S	SKPPEGVVSEVEMLSSQERMKVQGSPLKKLF	1247300	1658200	2128
O75676	682	Ribosomal protein S6 kinase alpha-4	RPS6KA4	0.996337	0.000930017	106.02	S	GLRGSSWLQDGSARSSPPLRTPDVLESSGPA	2093700	2194000	653
O75676	687	Ribosomal protein S6 kinase alpha-4	RPS6KA4	0.999648	0.000930017	106.02	T	SWLQDGSARSSPPLRTPDVLESSGPAVRSGL	0	492020	7216
Q12888	301	Tumor suppressor p53-binding protein 1	TP53BP1	0.5	0.000418117	106	S	ESGLQIQKSPEPEVLSTQEDLFDQSNKTVSS	350750	381890	2368
Q9P258	43	Protein RCC2	RCC2	0.231246	1.03E-06	106	S	RGGPAGRKRERPERCSSSSGGGSSGDEDGLE	0	0	6215
Q12888	302	Tumor suppressor p53-binding protein 1	TP53BP1	0.5	0.000418117	106	T	SGLQIQKSPEPEVLSTQEDLFDQSNKTVSSD	350750	381890	7514
P10398	257	Serine/threonine-protein kinase A-Raf	ARAF	0.994305	0.00119556	105.95	S	DAAGSRGGSDGTPRGSPSPASVSSGRKSPHS	1805900	1279900	986
Q15022	539	Polycomb protein SUZ12	SUZ12	0.6237	0.00660261	105.92	S	PITHILVCRPKRTKASMSEFLESEDGEVEQQ	443060	735140	2856
P35611	645	Alpha-adducin	ADD1	0.995954	2.35E-05	105.91	S	DDSDAATFKPTLPDLSPDEPSEALGFPMLEK	647990	603610	1400
Q5T5Y3	1080	Calmodulin-regulated spectrin-associated protein 1	CAMSAP1	0.999961	0.000586946	105.91	S	QDHKVKAPVHFVEPLSPTGVAGHRKAPRLGQ	0	0	3292
P46087	181	Probable 28S rRNA (cytosine(4447)-C(5))-methyltransferase	NOP2	1	4.27E-07	105.82	S	ARKQKAREAAAGIQWSEEETEDEEEEKEVTP	4858800	5518200	1544
Q9Y4F5	986	Centrosomal protein of 170 kDa protein B	CEP170B	1	5.61E-05	105.82	S	PSPTTPQPLRAQKEMSPSPPAAQDPGGTALV	0	1163000	6913
Q9Y4F5	988	Centrosomal protein of 170 kDa protein B	CEP170B	1	5.61E-05	105.82	S	PTTPQPLRAQKEMSPSPPAAQDPGGTALVSA	0	1163000	6914
P46087	185	Probable 28S rRNA (cytosine(4447)-C(5))-methyltransferase	NOP2	0.999999	4.27E-07	105.82	T	KAREAAAGIQWSEEETEDEEEEKEVTPESGP	4858800	5518200	7383
O75151	459	Lysine-specific demethylase PHF2	PHF2	0.5	0.000105852	105.71	S	NASKAVRPEVNTVASSDEVCDGDREKEEPPS	0	920250	569
Q8N3X1	435	Formin-binding protein 4	FNBP4	0.99415	4.84E-07	105.71	S	ALEEGDGSVSGSSPRSDISQPASQDGMRRLM	3972000	1560000	4262
Q08AD1	1319	Calmodulin-regulated spectrin-associated protein 2	CAMSAP2	0.99496	0.0104066	105.66	S	SGKRTPRSESVEGFLSPSRCGSRNGEKDWEN	294240	505470	2257
Q7KZI7	456	Serine/threonine-protein kinase MARK2	MARK2	1	0.00103588	105.66	S	RESGRKASSTAKVPASPLPGLERKKTTPTPS	1047500	681800	3715
Q9NYL2	637	Mitogen-activated protein kinase kinase kinase MLT	ZAK	0.556182	0.00103588	105.66	S	IKYQQITPVNQSRSSSPTQYGLTKNFSSLHL	1151100	0	6120
O75909	328	Cyclin-K	CCNK	0.82459	0.00492396	105.65	S	QQQPAQQPKKPSPQPSSPRQVKRAVVVSPKE	803010	748750	668
Q3MII6	506	TBC1 domain family member 25	TBC1D25	1	0.00103639	105.65	S	ASQGPGGGGRLLRQASLDGLQQLRDNMGSRR	598880	381740	3075
Q15652	2053	Probable JmjC domain-containing histone demethylation protei	JMJD1C	0.5	0.0100556	105.63	S	EEREQDNSESPNGRTSPLVSQNNEQGSTLRD	1040600	580690	2972
Q86U90	60	YrdC domain-containing protein, mitochondrial	YRDC	1	0.00292765	105.63	S	ARLLRLPGSGAVQAASPERAGWTEALRAAVA	4237600	3112300	3922
Q15652	2052	Probable JmjC domain-containing histone demethylation protei	JMJD1C	0.5	0.0100556	105.63	T	KEEREQDNSESPNGRTSPLVSQNNEQGSTLR	1040600	580690	7647
Q9NY61	320	Protein AATF	AATF	0.999999	1.37E-06	105.56	S	YLVDGTKPNAGSEEISSEDDELVEEKKQQRR	29629000	15960000	6090
Q9NY61	321	Protein AATF	AATF	0.999999	1.37E-06	105.56	S	LVDGTKPNAGSEEISSEDDELVEEKKQQRRR	29629000	15960000	6091
P49454	1726	Centromere protein F	CENPF	0.735626	1.53E-07	105.52	S	ETGAVKPTGECSGEQSPDTNYEPPGEDKTQG	0	0	1664
Q9NRH2	609	SNF-related serine/threonine-protein kinase	SNRK	0.607338	2.57E-07	105.52	S	DKASPSENNAGGGSPSSGSGGNPTNTSGTTR	1800100	0	5965
Q9NYV4	423	Cyclin-dependent kinase 12	CDK12	1	0.00244031	105.52	S	AAAAAAKMDGKESKGSPVFLPRKENSSVEAK	3895100	2413000	6122
Q9UDY2	266	Tight junction protein ZO-2	TJP2	0.999977	0.00782276	105.52	S	RAYHRAYDPDYERAYSPEYRRGARHDARSRG	0	0	6276
Q70CQ2	3410	Ubiquitin carboxyl-terminal hydrolase 34	USP34	0.481375	2.47E-05	105.5	S	IIDPGTEQDLPSPENSSVKEYRMEVPSSFSE	0	0	3680
Q70CQ2	3411	Ubiquitin carboxyl-terminal hydrolase 34	USP34	0.481375	2.47E-05	105.5	S	IDPGTEQDLPSPENSSVKEYRMEVPSSFSED	0	0	3681
Q96JP5	82	E3 ubiquitin-protein ligase ZFP91	ZFP91	0.498224	0.00237482	105.5	S	AVSRRRKAEYPRRRRSSPSARPPDVPGQQPQ	0	0	5000
Q96JP5	83	E3 ubiquitin-protein ligase ZFP91	ZFP91	0.498224	0.00237482	105.5	S	VSRRRKAEYPRRRRSSPSARPPDVPGQQPQA	0	0	5001
Q4VC05	113	B-cell CLL/lymphoma 7 protein family member A	BCL7A	0.424974	2.20E-06	105.46	S	SIADASPIKQENSSNSSPAPEPNSAVPSDGT	0	0	3109
Q4VC05	114	B-cell CLL/lymphoma 7 protein family member A	BCL7A	0.58977	2.20E-06	105.46	S	IADASPIKQENSSNSSPAPEPNSAVPSDGTE	1340300	0	3110
Q15424	195	Scaffold attachment factor B1	SAFB	0.694788	1.30E-07	105.44	S	EHAIEDKETINNLDTSSSDFTILQEIEEPSL	11585000	199010	2924
Q15424	196	Scaffold attachment factor B1	SAFB	0.728802	1.30E-07	105.44	S	HAIEDKETINNLDTSSSDFTILQEIEEPSLE	11585000	199010	2925
Q15424	197	Scaffold attachment factor B1	SAFB	0.728694	1.30E-07	105.44	S	AIEDKETINNLDTSSSDFTILQEIEEPSLEP	11975000	582660	2926
Q659A1	605	Little elongation complex subunit 2	ICE2	0.970054	1.21E-07	105.44	S	GKTAVVGSNLSSRPASPNSSSGQASVGNQTN	2186400	1820500	3396
Q8IWS0	203	PHD finger protein 6	PHF6	0.499991	0.00774113	105.4	S	DEMESSSYRDRSPHRSSPSDTRPKCGFCHVG	0	970540	4072

Q8IWS0	204	PHD finger protein 6	PHF6	0.499991	0.00774113	105.4	S	EMESSSYRDRSPHRSSPSDTRPKCGFCHVGE	0	970540	4073
Q9NWQ4	447	G patch domain-containing protein 2-like	GPATCH2L	0.994954	0.00171754	105.4	S	MDAVEPTTPASAPKSPSSEWLVRTSAAEKA	626640	524600	6061
Q9UQ80	360	Proliferation-associated protein 2G4	PA2G4	0.746997	0.00171754	105.4	S	SEMEVQDAELKALLQSSASRKTQKKKKKKAS	591880	468310	6750
Q93100	701	Phosphorylase b kinase regulatory subunit beta	PHKB	0.652116	1.59E-05	105.35	S	EELEPPKHSKVKRQSSTPSAPELGQQPDVNI	1941100	601620	4745
Q93100	702	Phosphorylase b kinase regulatory subunit beta	PHKB	0.919779	1.59E-05	105.35	T	ELEPPKHSKVKRQSSTPSAPELGQQPDVNIS	3944500	601620	7955
Q8IY63	720	Angiomotin-like protein 1	AMOTL1	1	0.00173113	105.25	S	AATAAAERDTTIINHSRNGSYGESSLEAHIW	0	0	4118
P49792	2741	E3 SUMO-protein ligase RanBP2	RANBP2	0.990544	2.68E-07	105.24	S	ADTLKLPPTFFCGVCSDTDEDNGNGEDFQSE	2515100	2051000	1713
P46937	367	Transcriptional coactivator YAP1	YAP1	0.770012	1.15E-06	105.22	S	LPTLEQDGGTQNPVSSPGMSQELRTMTTNSS	0	945890	1617
Q96BD5	450	PHD finger protein 21A	PHF21A	0.343058	8.28E-07	105.22	S	NAVLGFGALTPTSPQSSHPDSPENEKTETTF	0	0	4805
Q96RK0	1382	Protein capicua homolog	CIC	0.7562	1.44E-06	105.22	S	PEEVLPSPTLQSLATSPRAILGSYRKKRKNS	4568600	0	5092
Q96RK0	1375	Protein capicua homolog	CIC	0.498915	1.44E-06	105.22	T	AELPEFRPEEVLPSPTLQSLATSPRAILGSY	0	0	8002
O75385	623	Serine/threonine-protein kinase ULK1	ULK1	0.5	0.00127515	105.2	S	LPDFLQRNPLPPILGSPTKAVPSFDFPKTPS	742840	898590	621
Q66K74	655	Microtubule-associated protein 1S;MAP1S heavy chain;MAP1S li	MAP1S	1	0.00658782	105.2	S	SPESHRSPAEGSERLSLSPLRGGEAGPDASP	1189200	1293300	3407
Q66K74	657	Microtubule-associated protein 1S;MAP1S heavy chain;MAP1S li	MAP1S	1	0.00658782	105.2	S	ESHRSPAEGSERLSLSPLRGGEAGPDASPTV	2179400	2159500	3408
O75385	625	Serine/threonine-protein kinase ULK1	ULK1	0.679945	0.00127515	105.2	T	DFLQRNPLPPILGSPTKAVPSFDFPKTPSSQ	742840	898590	7201
P02545	392	Prelamin-A/C;Lamin-A/C	LMNA	0.951705	0.00411345	105.19	S	RKLLEGEEERLRLSPSPTSQRSRGRASSHSS	16310000	717740	862
P40425	330	Pre-B-cell leukemia transcription factor 2	PBX2	0.99999	0.00127563	105.19	S	IYAVKTAVSVTQGGHSRTSSPTPPSSAGSGG	1485900	349250	1475
P49736	40	DNA replication licensing factor MCM2	MCM2	0.536123	0.00438994	105.19	S	TSSPGRSSRRTDALTSSPGRDLPPFEDESEG	0	431830	1682
P98175	723	RNA-binding protein 10	RBM10	0.993627	0.00481851	105.19	S	HTSMDLPKLASDDRPSPPRGLVAAYSGESDS	1874500	823980	2019
Q92786	511	Prospero homeobox protein 1	PROX1	0.999039	0.00574938	105.19	S	PLGAPSGSFSGKDRASPESLDLTRDTTSLRT	0	0	4702
Q9UJU6	275	Drebrin-like protein	DBNL	0.99009	0.00301662	105.19	S	IFKQKERAMSTTSISSPQPGKLRSPFLQKQL	0	3655900	6408
P11474	29	Steroid hormone receptor ERR1	ESRRA	0.432254	0.000459375	105.19	T	KAEPASPDSPKGSSETETEPPVALAPGPAPT	0	0	7279
Q2M2I8	606	AP2-associated protein kinase 1	AAK1	0.999039	0.00835277	105.19	T	PAIQAPVRQQPKVQTTPPPAVQGQKVGSLTP	1504400	1167900	7666
Q7Z2Z1	1354	Treslin	TICRR	0.905933	1.69E-06	105.16	S	DQKEPQMSPVAASLSCPVPSTPPELSQRAT	0	2300800	3760
Q7Z2Z1	1360	Treslin	TICRR	0.771532	1.69E-06	105.16	T	MSPVSAASLSCPVPSTPPELSQRATLDTVPP	0	2300800	7799
P02549	109	Spectrin alpha chain, erythrocytic 1	SPTA1	0.99998	0.00785148	105.14	S	HQSLEAEVQTKSRLMSELEKTREERFTMGHS	1750900	1453700	866
P11388	1354	DNA topoisomerase 2-alpha	TOP2A	1	0.00302528	105.14	S	FDEKTDEDFVPSDASPPKTKTSPKLSNKEL	275780	128850	1024
Q13442	178	28 kDa heat- and acid-stable phosphoprotein	PDAP1	0.997883	0.00250777	105.13	S	AKDDATLSGKRMQSLSLNK__________	1015800	273930	2534
Q13480	355	GRB2-associated-binding protein 1	GAB1	0.999959	0.00123384	105.13	S	IPPPRPPKPHPAHDRSPVETCSIPRTASDTD	1089100	0	2548
Q7L7X3	421	Serine/threonine-protein kinase TAO1	TAOK1	0.999006	0.00128163	105.13	S	YREEGDPRTRASDPQSPPQVSRHKSHYRNRE	2939900	1527500	3731
Q71DI3;Q|58;58;58		Histone H3.2;Histone H3.1t;Histone H3.3;Histone H3.1;Histone	HIST2H3A;HI	0.797973	0.0079545	105.1	S	RPGTVALREIRRYQKSTELLIRKLPFQRLVR	620320	276790	1948
P04637	6	Cellular tumor antigen p53	TP53	0.968815	3.07E-06	105.06	S	__________MEEPQSDPSVEPPLSQETFSD	6124500	14571000	883
P49321	390	Nuclear autoantigenic sperm protein	NASP	0.999688	5.53E-06	105.06	T	PKDGAVNGPSVVGDQTPIEPQTSIERLTETK	1432800	2038500	7409
O95359	2226	Transforming acidic coiled-coil-containing protein 2	TACC2	1	0.00175175	105.03	S	EGVVPPASGGGRVQNSPPVGRKTLPLTTAPE	1041100	602300	754
Q13595	84	Transformer-2 protein homolog alpha	TRA2A	0.999804	0.014305	105.03	S	TRSRSHSHSHRRRSRSRSYTPEYRRRRSRSH	0	0	2596
Q92499	481	ATP-dependent RNA helicase DDX1	DDX1	0.999479	0.000807259	105.02	S	TDDVHAKDNTRPGANSPEMWSEAIKILKGEY	0	3077400	4616
O00559	41	Receptor-binding cancer antigen expressed on SiSo cells	EBAG9	0.759724	0.000150869	105.01	T	ICRSGRGKLSGDQITLPTTVDYSSVPKQTD	0	0	7122
P36507	293	Dual specificity mitogen-activated protein kinase kinase 2	MAP2K2	0.5	0.00128424	105	S	IFGRPVVDGEEGEPHSISPRPRPPGRPVSGH	102220	4216100	1427
P38159	88	RNA-binding motif protein, X chromosome;RNA-binding motif pr	RBMX	0.920843	0.00851435	105	S	SLDGKAIKVEQATKPSFESGRRGPPPPPRSR	0	0	1448
Q13428	678	Treacle protein	TCOF1	0.617896	8.96E-05	105	S	TQAPRKAGTATSPAGSSPAVAGGTQRPAEDS	0	0	2505
Q96JH8	216	Ras-associating and dilute domain-containing protein	RADIL	0.438037	4.37E-07	104.97	S	GDARSSPPPRLRRTVSETSLSPVNALPAAAQ	0	0	4965
Q96JH8	214	Ras-associating and dilute domain-containing protein	RADIL	0.438037	4.37E-07	104.97	T	ALGDARSSPPPRLRRTVSETSLSPVNALPAA	0	0	7988
P39880	1218	Homeobox protein cut-like 1	CUX1	0.799634	8.18E-09	104.96	S	RMEKKAYMKRRHSSVSDSQPCEPPSVGTEYS	0	0	1464
P49454	1729	Centromere protein F	CENPF	0.945945	1.67E-07	104.96	T	AVKPTGECSGEQSPDTNYEPPGEDKTQGSSE	12548000	9621600	7416
Q6DN90	417	IQ motif and SEC7 domain-containing protein 1	IQSEC1	1	0.00124708	104.94	S	KRQSAYERSLGQQSPKHGPHSGAPKSLPR	235840	733790	3450
Q9Y580	136	RNA-binding protein 7	RBM7	0.988935	0.00124708	104.94	S	MDNMTSSAQIIQRSFSSPENFQRQAVMNSAL	2081900	612520	6953
Q9P2K8	667	Eukaryotic translation initiation factor 2-alpha kinase 4	EIF2AK4	1	0.0106247	104.9	T	YNAWIERHERPAGPGTPPPDSGPLAKDDRAA	640850	456520	8191
Q3YEC7	454	Rab-like protein 6	RABL6	1	1.26E-07	104.88	S	AGFQDDVLEDQPRGSPPLPAGPVPSQDITL	14478000	12446000	3081
Q14839	515	Chromodomain-helicase-DNA-binding protein 4	CHD4	0.984563	1.37E-06	104.86	S	GKVQKILIWKWGQPPSPTPVPRPPDADPNTP	35314000	21887000	2820
O60343	789	TBC1 domain family member 4	TBC1D4	0.856396	0.000113576	104.84	S	QRIFLRVASPMNKSPSAMQQQDGLDRNELLP	1479600	0	495
O60343	782	TBC1 domain family member 4	TBC1D4	0.999938	0.000113576	104.84	S	PRRISWRQRIFLRVASPMNKSPSAMQQQDGL	1479600	752520	501
Q99728	363	BRCA1-associated RING domain protein 1	BARD1	0.836374	9.11E-05	104.84	S	VKQTVPSENIPLPECSSPPSCKRKVGGTSGR	0	1254300	5265
Q99728	367	BRCA1-associated RING domain protein 1	BARD1	0.385199	9.11E-05	104.84	S	VPSENIPLPECSSPPSCKRKVGGTSGRKNSN	0	0	5267
O43639	90	Cytoplasmic protein NCK2	NCK2	0.887448	0.000117236	104.81	S	LGLGKTRRKTSARDASPTPSTDAEYPANGSG	803550	370480	404
P40222	514	Alpha-taxilin	TXLNA	0.5	0.00238746	104.81	S	SGPERRPEGPGAQAPSSPRVTEAPCYPGAPS	22447000	12524000	1468
Q6ZRS2	3148	Helicase SRCAP	SRCAP	1	0.00210745	104.81	S	TEKLPRKRAGAPVGGSPGLAKRGRLQPPSPL	1658600	1129200	3654
Q8IYB3	626	Serine/arginine repetitive matrix protein 1	SRRM1	1	0.00847009	104.81	S	KRRTASPPPPPKRRASPSPPPKRRVSHSPPP	0	623670	4152
Q8IYB3	628	Serine/arginine repetitive matrix protein 1	SRRM1	1	0.00847009	104.81	S	RTASPPPPPKRRASPSPPPKRRVSHSPPPKQ	0	623670	4153
Q7KZI7	619	Serine/threonine-protein kinase MARK2	MARK2	0.959399	6.04E-05	104.76	S	QVRDQQNLPYGVTPASPSGHSQGRRGASGSI	3918900	3340100	3711
Q7KZI7	621	Serine/threonine-protein kinase MARK2	MARK2	0.900057	6.29E-05	104.76	S	RDQQNLPYGVTPASPSGHSQGRRGASGSIFS	6539600	1815500	3712
O95149	350	Snurportin-1	SNUPN	0.86418	0.00311733	104.7	S	ELEHLSTPKLKGSSHSPDHPGCLMEN_____	0	0	731
Q8N3X1	18	Formin-binding protein 4	FNBP4	1	0.00508785	104.7	S	KKSRAVPGRRPILQLSPPGPRGSTPGRDPEP	0	0	4265
Q9NVD7	4	Alpha-parvin	PARVA	0.584931	0.000572714	104.7	S	____________MATSPQKSPSVPKSPTPKS	0	0	6034
Q9NVD7	8	Alpha-parvin	PARVA	0.999073	0.000572714	104.7	S	________MATSPQKSPSVPKSPTPKSPPSR	2335100	1756100	6035
O60716	349	Catenin delta-1	CTNND1	0.999972	0.00121585	104.59	S	YYWAPLAQHERGSLASLDSLRKGGPPPPNWR	8363100	5562100	515
Q32P44	198	Echinoderm microtubule-associated protein-like 3	EML3	0.938605	0.00133857	104.59	S	RSGSTESRGGKDPLSSPGGPGSRRSNYNLEG	502820	226360	3057
P20700	23	Lamin-B1	LMNB1	0.998927	0.0104487	104.55	S	PPRMGSRAGGPTTPLSPTRLSRLQEKEELRE	15675000	10633000	1186
O14686	624	Histone-lysine N-methyltransferase 2D	KMT2D	0.815748	6.77E-07	104.52	S	SPPPEESPLSPPPEASRLSPPPEDSPMSPPP	2153900	0	194
Q14839	529	Chromodomain-helicase-DNA-binding protein 4	CHD4	1	1.37E-06	104.49	T	PSPTPVPRPPDADPNTPSPKPLEGRPERQFF	21786000	3881000	7626
Q9Y6D6	400	Brefeldin A-inhibited guanine nucleotide-exchange protein 1	ARFGEF1	0.438367	2.75E-05	104.49	T	TPSLPDDRLSVSSNDTQESGNSSGPSPGAKF	0	0	8318
Q8N122	722	Regulatory-associated protein of mTOR	RPTOR	0.877286	7.10E-05	104.43	S	PVRDSPCTPRLRSVSSYGNIRAVATARSLNK	0	457360	4209
Q9NTI5	1383	Sister chromatid cohesion protein PDS5 homolog B	PDS5B	0.998302	0.00263162	104.42	S	QSTPQKGRGRPSKTPSPSQPKKNVRVGRSKQ	7629700	6988900	6013
Q96T37	700	Putative RNA-binding protein 15	RBM15	1	0.00577969	104.41	S	NSDNDRSSRLLLERPSPIRDRRGSLEKSQGD	1773400	517950	5163
P50851	1120	Lipopolysaccharide-responsive and beige-like anchor protein	LRBA	0.981189	2.20E-07	104.39	T	EEDDDYVELKVEGSPTEEANLPTELQDNSLS	3849100	0	7435
P51610	1507	Host cell factor 1;HCF N-terminal chain 1;HCF N-terminal cha	HCFC1	1	3.01E-07	104.38	S	PVPGPSVPPPEELQVSPGPRQQLPPRQLLQS	12282000	10698000	1789
Q8IXQ4	105	GPALPP motifs-containing protein 1	GPALPP1	1	0.00012154	104.38	S	FFGPALPPGFKKQDDSPPRPIIGPALPPGFI	1966100	1610600	4111
Q9P2E9	1276	Ribosome-binding protein 1	RRBP1	0.9998	3.01E-07	104.38	S	MKSHVEDGDIAGAPASSPEAPPAEQDPVQLK	1139400	934650	6237
Q9UKS6	354	Protein kinase C and casein kinase substrate in neurons prot	PACSIN3	0.996463	4.68E-07	104.38	S	PQSPGSPGTGQDEEWSDEESPRKAATGVRVR	7225400	3558200	6450
Q8NG08	967	DNA helicase B	HELB	0.999996	0.00320884	104.26	S	QSKLSSSGAPPADFPSPRKSSGDSGGPSTPS	1283600	589550	4407
Q9H2Y7	641	Zinc finger protein 106	ZNF106	0.973327	0.00137769	104.22	S	EEDDRILKTSRELSTSPCNPIVRQKESELQM	0	0	5626
Q86VR2	360	Protein FAM134C	FAM134C	1	0.00321938	104.21	S	DEDDTSIGMPSLMYRSPPGAEEPQAPPASRD	383020	345740	3966
Q9H792	568	Pseudopodium-enriched atypical kinase 1	PEAK1	0.836226	0.00267368	104.21	S	SGTGPNVPPRKNCHKSAPTSPTATNISSKTI	0	571890	5752
Q9H792	572	Pseudopodium-enriched atypical kinase 1	PEAK1	0.91229	0.00267368	104.21	S	PNVPPRKNCHKSAPTSPTATNISSKTIPVKS	0	571890	5753
Q08999	639	Retinoblastoma-like protein 2	RBL2	1	0.0112704	104.2	S	PPQNLERADEICIAGSPLTPRRVTEVRADTG	631170	644380	2251
Q7L9B9	173	Endonuclease/exonuclease/phosphatase family domain-containir	EEPD1	1	0.00823845	104.2	S	ALSIVDFRREHGPFRSVEDLVRMDGINAAFL	1469300	341990	3735
Q08999	642	Retinoblastoma-like protein 2	RBL2	1	0.0112704	104.2	T	NLERADEICIAGSPLTPRRVTEVRADTGGLG	631170	644380	7505
Q9Y4R8	632	Telomere length regulation protein TEL2 homolog	TELO2	0.803559	1.70E-08	104.14	T	TLAAQELSRPGCLGRTPQPGSPSPNTPCLPE	3697400	0	8304
Q6P1N0	455	Coiled-coil and C2 domain-containing protein 1A	CC2D1A	0.999988	0.00910142	104.09	S	EGPEDEEDEVPKKAQNSPVAPTAQPKAPPSRT	1152500	400040	3511
Q14676	760	Mediator of DNA damage checkpoint protein 1	MDC1	0.67202	2.84E-06	104.07	S	EPWEVLATQPFCLRESEDSETQPFDTHLEAY	0	0	2763
O60256	227	Phosphoribosyl pyrophosphate synthase-associated protein 2	PRPSAP2	1	0.00828267	104.06	S	GEAQDAESDLVDGRHSPPMVRSVAAIHPSLE	12797000	7390300	450
Q3KQU3	446	MAP7 domain-containing protein 1	MAP7D1	1	0.00269558	104.06	S	LARERSLKKRQSLPASPRARLSASTASELSP	0	1271700	3070
Q86W92	466	Liprin-beta-1	PPFIBP1	0.997106	0.00269716	104.05	S	QLCDKLLTSSLQKSSSLGNLKKETSDGEKET	1021700	520230	3975
Q8IZ73	68	RNA pseudouridylate synthase domain-containing protein 2	RPUSD2	1	0.00795793	103.97	S	HQQNGDAGGDAKVELSPGPPKPAGREVEPAP	253520	212240	4183
Q96T37	292	Putative RNA-binding protein 15	RBM15	1	0.00465997	103.97	S	VGGHRHPPGGGGGQRSLSPGGAALGYRDYRL	2259100	965920	5164
Q96F45	230	Zinc finger protein 503	ZNF503	0.731245	7.27E-07	103.94	S	PSATCQPFTPRTGSPSSSASACSPGGMLSSA	0	0	4899
Q8NI27	1417	THO complex subunit 2	THOC2	0.965088	0.00162935	103.88	S	EPEREQKRRKIDTHPSPSHSSTVKDSLIELK	3400500	1839600	4423
Q8IZ21	28	Phosphatase and actin regulator 4	PHACTR4	0.897132	7.41E-09	103.88	T	TEPGMVLDSVEAGDTTPPTKRKSKFSGFGKI	2083700	1591200	7868
Q9UBC2	797	Epidermal growth factor receptor substrate 15-like 1	EPS15L1	0.694445	1.01E-08	103.88	T	KKPAPPRPKPPSGKSTPVSQLGSADFPEAPD	2008400	0	8194
Q9BUA3	148	Uncharacterized protein C11orf84	C11orf84	0.994827	6.85E-07	103.84	S	SEGVALLQDVRAEQPSPPNSDSGQDAHPDPD	571890	737920	5374
P46783;Q|146;157		40S ribosomal protein S10;Putative 40S ribosomal protein S1(	RPS10;RPS1(	0.994331	0.00330553	103.77	S	AVPPGADKKAEAGAGSATEFQFRGGFGRGRG	1614900	1047500	1569
Q4LE39	675	AT-rich interactive domain-containing protein 4B	ARID4B	0.999895	0.00142302	103.77	S	CKLRRLSKPPFQTNPSPEMVSKLDLTDAKNS	0	3393800	3103
P78527	2675	DNA-dependent protein kinase catalytic subunit	PRKDC	0.418439	1.52E-07	103.75	S	TGSSTDPLVDHTSPSSDSLLFAHKRSERLQR	0	0	1970
Q12888	1758	Tumor suppressor p53-binding protein 1	TP53BP1	0.759247	8.17E-06	103.71	S	DKLASRSKLPDGPTGSSEEEEEFLEIPPFNK	3520700	0	2349
P46821	1252	Microtubule-associated protein 1B;MAP1B heavy chain;MAP1 lig	MAP1B	0.999273	0.000645459	103.67	S	LDIKDSISAVSSEKVSPSKSPSLSPSPPSPL	5047600	2717500	1611
Q9UQ35	2407	Serine/arginine repetitive matrix protein 2	SRRM2	0.985185	0.00552982	103.66	S	RVSGRTSPPLLDRARSRTPPSAPSQSRMTSE	23572000	8451500	6723
Q9UQ35	2412	Serine/arginine repetitive matrix protein 2	SRRM2	0.996773	0.00552982	103.66	S	TSPPLLDRARSRTPPSAPSQSRMTSERAPSP	7362500	3306500	6724
Q9UKE5	640	TRAF2 and NCK-interacting protein kinase	TNIK	0.996452	0.00334396	103.61	S	ALNVTSHRVEMPRQNSDPTSENPPLPTRIEK	503820	0	6435
Q9C0B5	621	Palmitoyltransferase ZDHHC5	ZDHHC5	0.999985	0.012703	103.6	S	PRFGKPDGLRGRGVGSPEPGPTAPYLGRSMS	1561800	534480	5511
Q9UFC0	212	Leucine-rich repeat and WD repeat-containing protein 1	LRWD1	1	0.00251148	103.6	S	EELVAASRTQVQKANSPEKPPEAGAAHKPRA	0	238950	6310
Q8TE77	87	Protein phosphatase Slingshot homolog 3	SSH3	0.993429	5.71E-06	103.59	S	EELHGDQTDFGQGSQSPQKQEEQRQHLHLMV	3747400	2255200	4473
Q8TF01	211	Arginine/serine-rich protein PNISR	PNISR	1	0.00278459	103.55	S	QNRRERPSSFRDRQRSPIALPVKQEPPQIDA	0	735390	4497
O60934	432	Nibrin	NBN	0.986324	0.00134587	103.46	S	VSNTLAKMRIPNYQLSPTKLPSINKSKDRAS	8701100	3386300	541
Q01826	38	DNA-binding protein SATB1	SATB1	1	0.00134587	103.46	S	PKGPPAKIARLEQNGSPLGRGRLGSTGAKMQ	625420	390910	2081
Q13625	698	Apoptosis-stimulating of p53 protein 2	TP53BP2	0.992041	0.00855872	103.46	S	SVQENHENERIPRPLSPTKLLPFLSNPYRNQ	1049700	0	2617
O94913	487	Pre-mRNA cleavage complex 2 protein Pcf11	PCF11	0.999762	0.00518677	103.43	S	GRSSTRKRSRSRSPKSRSPIIHSPKRRDRRS	2222400	812040	719
O94913	489	Pre-mRNA cleavage complex 2 protein Pcf11	PCF11	0.840751	0.00518677	103.43	S	SSTRKRSRSRSPKSRSPIIHSPKRRDRRSPK	2002200	1132200	720
Q15942	344	Zyxin	ZYX	1	0.00119384	103.43	S	QHPVPPPAQNQNVRSPGAPGPLTLKEVEEL	10669000	9422100	2997
Q5T5U3	1742	Rho GTPase-activating protein 21	ARHGAP21	0.97403	0.0013478	103.43	S	APSLTKVFDVMKKGKSTGSLLTPTRGESEKQ	0	528050	3290
Q9UQR1	783	Zinc finger protein 148	ZNF148	0.667026	0.00288952	103.43	S	EFPLVNVNDNRAGMTSSPDATTGQTFG____	0	63810	6764
Q96QR8	101	Transcriptional activator protein Pur-beta	PURB	0.822111	9.24E-09	103.42	S	SLGDFIEHYAQLGPSSPEQLAAGAEEGGGPR	6309100	3629000	5083
Q9HOW8	32	Protein SMG9	SMG9	0.998277	0.00289751	103.39	S	RRRWKEPGSGGPQNLSGPGGRERDYIAPWER	0	2067900	5585
Q99598	33	Translin-associated protein X	TSNAX	0.989628	0.0125146	103.34	S	FPHNQRREGKDVNSSSPVMLAFKSFQQELDA	1254200	765640	5228
Q96D71	389	RalBP1-associated Eps domain-containing protein 1	REPS1	0.374738	3.21E-07	103.33	Y	LEDSADVGDQPGEVGYSGSPAEAPPSKSPSM	0	0	8407
P10636	579	Microtubule-associated protein tau	MAPT	0.992613	0.0028255	103.31	S	PVPMPDLKNVKSKIGSTENLKHQPGGGKVQI	565350	853740	996
P40227	205	T-complex protein 1 subunit zeta	CCT6A	0.858077	0.00191299	103.31	S	MIEIMEMKHKSETDTSLIRGLVLDHGARHPD	0	0	1472
Q5T481	1047	RNA-binding protein 20	RBM20	0.59701	3.67E-06	103.31	S	VESSDVHPAPTVQQMSSPKPAEERARQPSPF	0	0	3281
Q9UQ35	983	Serine/arginine repetitive matrix protein 2	SRRM2	1	0.00242115	103.31	T	SHSGSSSPDTKVKPETPPRQSHSGSISPYPK	13768000	4897300	8279
P49815	1449	Tuberin	TSC2	0.990896	0.00405583	103.3	S	GEDSRGQPEGPLPSSSPRSPSGLRPRGYTIS	3282900	0	1725
P51610	598	Host cell factor 1;HCF N-terminal chain 1;HCF N-terminal cha	HCFC1	0.797874	0.00340103	103.3	S	TPGTTTLPATVKVASSPVMVSNPATRMLKTA	1710800	1826500	1793
Q8IYB3	718	Serine/arginine repetitive matrix protein 1	SRRM1	0.422655	0.0549622	103.3	T	GASSSPQRRQSPSPSTRPIRRVSRTPEPKKI	0	0	7863
O95671	239	N-acetylserotonin O-methyltransferase-like protein	ASMTL	0.979876	1.47E-06	103.28	S	VKHDSIPAADTFEDLSTLIRGLVLDNARQDAG	0	0	789
Q14004	315	Cyclin-dependent kinase 13	CDK13	1	0.00235696	103.26	S	YREDKTEPKAYRRRRSLSPLGGRDDSPVSHR	1020500	705980	2640
Q14004	317	Cyclin-dependent kinase 13	CDK13	1	0.00235696	103.26	S	EDKTEPKAYRRRRSLSPLGGRDDSPVSHRAS	1682700	928170	2641
Q7L7X3	965	Serine/threonine-protein kinase TAO1	TAOK1	1	0.00853453	103.26	S	PMQGVPRGSSMGVRNSPQALRRTASGGRTEQ	724590	959340	3732
Q6PKG0	143	La-related protein 1	LARP1	0.997808	1.19E-05	103.25	S	TKNALPPVTTVNGQSPPEHSAPAKVVRAAV	13189000	7049600	3560
O75674	323	TOM1-like protein 1	TOM1L1	0.999179	4.39E-06	103.24	S	SEPSAPSQDLLDLSPSPRMPRATLGELNTMN	5600000	4910800	650
Q9UK58	65	Cyclin-L1	CCNL1	0.842669	0.000105918	103.24	S	VSLTIDHSLIPEERLSPTPSMQDGLDLPSET	737610	0	6423
Q9H330	334	Transmembrane protein 245	TMEM245	0.616465	1.15E-06	103.24	T	TSPSPSSPSPTSPSPTLGRRRPEIGTFLRKK	3851200	3177400	8095
Q8TEV9	417	Smith-Magenis syndrome chromosomal region candidate gene 8 p	SMCR8	0.999517	0.00192222	103.21	S	SLEECPIPKVLISVGSYKSSVESVLIKMEQE	590050	527550	4492

Q9UJA3	574	DNA helicase MCM8	MCM8	0.499738	0.00673186	103.21	S	AANPVGGHYNKAKTVSENLKMGSALLSRFDL	0	0	6393
Q9UJA3	572	DNA helicase MCM8	MCM8	0.499738	0.00673186	103.21	T	IAAANPVGGHYNKAKTVSENLKMGSALLSRF	0	0	8225
Q9P2R6	142	Arginine-glutamic acid dipeptide repeats protein	RERE	0.5	3.49E-07	103.15	S	IQDFKLVHNSQACCRSPTPALCDPPACSLPV	3651300	1722500	6255
Q9P2R6	144	Arginine-glutamic acid dipeptide repeats protein	RERE	0.5	3.49E-07	103.15	T	DFKLVHNSQACCRSPTPALCDPPACSLPVAS	3651300	1722500	8193
P24864	103	G1/S-specific cyclin-E1	CCNE1	1	0.00149208	103.14	S	PNSTCKPRIIAPSRGSPLPVLSWANREEVWK	317100	266480	1239
Q5T200	875	Zinc finger CCCH domain-containing protein 13	ZC3H13	0.999888	0.00100978	103.14	S	KHRLLSQVVRPQESRSLSPSHLTEDRQGRWK	1952000	1799000	3269
Q6P1L5	273	Protein FAM117B	FAM117B	0.859298	0.00121474	103.14	S	EEYSEKKKGSHKRSASWGSTDQLKEIAKLRQ	2302900	759310	3505
Q8IYB3	429	Serine/arginine repetitive matrix protein 1	SRRM1	1	0.00742446	103.14	S	SPTPQQSNRTRKSRVSVSPGRTSGKVTKHKG	1539300	1752400	4169
Q8IYB3	431	Serine/arginine repetitive matrix protein 1	SRRM1	1	0.00742446	103.14	S	TPQQSNRTRKSRVSVSPGRTSGKVTKHKGTE	6285200	3732000	4170
Q9BQP7	343	Mitochondrial genome maintenance exonuclease 1	MGME1	0.999238	0.0263645	103.14	S	YTEKKKNQNIQKPEYSE______________	802220	718940	5317
Q8N4C6	1550	Ninein	NIN	0.998286	0.0121132	103.13	S	EEDSISNLKLGTLNGSQEEMWQKTETVKQEN	1157500	775000	4267
Q9HC52	311	Chromobox protein homolog 8	CBX8	0.999999	0.00343382	103.13	S	GQGALDPNGTRVRHGSGPPSSGGGLYRDMGA	0	651850	5842
Q96T58	1636	Msx2-interacting protein	SPEN	0.993383	1.31E-06	103.09	S	ESAPENKDSELKTPPSVGPPSVTVVTLESAP	3760700	3123700	5167
Q96T58	1633	Msx2-interacting protein	SPEN	0.961577	1.31E-06	103.09	T	KTPESAPENKDSELKTPPSVGPPSVTVVTLE	3760700	0	8015
Q14191	1133	Werner syndrome ATP-dependent helicase	WRN	1	0.00193441	103.08	S	ISSGSNISKKSIMVQSPEKAYSSSQPVISAQ	1317700	885280	2699
Q9Y2J2	760	Band 4.1-like protein 3;Band 4.1-like protein 3, N-terminal	EPB41L3	0.991774	0.0240916	103.08	S	TSTDTAVTNEWEKRLSTSPVRLAARQEDAPM	0	1881800	6783
Q9Y2J2	761	Band 4.1-like protein 3;Band 4.1-like protein 3, N-terminal	EPB41L3	0.880137	0.0240916	103.08	T	STDTAVTNEWEKRLSTSPVRLAARQEDAPMI	0	0	8283
Q6IN85	768	Serine/threonine-protein phosphatase 4 regulatory subunit 3/	SMEK1	0.990351	7.58E-09	103.07	S	KTNLTSQSSTTNLPGSPGSPGSPGSPGSPGS	4072800	2363700	3477
Q96GY3	135	Protein lin-37 homolog	LIN37	0.996654	1.88E-06	103.05	S	RAWMRNSPSVRERECSPSSPLPPLPEDEEGS	3735600	2383800	4930
Q96GY3	138	Protein lin-37 homolog	LIN37	0.846458	1.88E-06	103.05	S	MRNSPSVRERECSPSSPLPPLPEDEEGSEVT	3735600	0	4932
Q9NR19	267	Acetyl-coenzyme A synthetase, cytoplasmic	ACSS2	0.831322	0.00297122	103.01	S	HLGRAELGMGDSTSQSPPIKRSCPDVQISWN	1180100	0	5942
Q9BVV8	117	Uncharacterized membrane protein C19orf24	C19orf24	0.979096	8.13E-07	102.97	S	RYGLLANTEDPTEMASLDSDEETVFESRNLR	14022000	10194000	5409
P31323	69	cAMP-dependent protein kinase type II-beta regulatory subuni	PRKAR2B	0.877188	0.0121447	102.97	T	EGRTWGDLGAAAGGGTPSKGVNFAEEPMQSD	1231900	0	7349
Q96ER3	6	Protein SAAL1	SAAL1	1	0.00194646	102.95	S	__________MDRNPSPPPPGRDKEEEEEVA	13067000	9959600	4887
Q9NYZ3	464	G2 and S phase-expressed protein 1	GTSE1	0.469695	0.00238964	102.95	S	NQFKIPKFSIGDSPDSSTPKLSRAQRPQSCT	0	0	6141
Q8WY36	844	HMG box transcription factor BBX	BBX	0.999978	0.0108056	102.9	S	KTKITHLVRTADGRVSPAGGTLDDKPKEQLQ	1882500	1230500	4602
Q96EV2	765	RNA-binding protein 33	RBM33	1	0.00554893	102.9	S	SRSSQGKTEVKVKPASPVAQPKEEAKTETEF	4448900	2390300	4894
Q9H910	30	Hematological and neurological expressed 1-like protein	HN1L	0.997493	5.81E-06	102.9	S	RAMKPPGGESSNLFGSPEEATPSSRPNRMAS	3076100	1693900	5797
Q9BUA3	49	Uncharacterized protein C11orf84	C11orf84	0.999828	0.000188408	102.89	T	IPRPEPMLRVTQQEKTPPPRPSPLEAGSDGC	3240300	1948100	8051
Q09472	622	Histone acetyltransferase p300	EP300	0.999999	0.0146279	102.87	S	NLVAYARKVEGDMYESANNRAEYYHLLAEKI	0	1787100	2270
O75152;A(497;497		Zinc finger CCCH domain-containing protein 11A	ZC3H11A	0.606209	3.23E-05	102.79	S	ALRVQQSSESSTSSPSQHEATPGARRLLRIT	0	0	578
Q8NFH5	73	Nucleoporin NUP53	NUP35	1	0.000748931	102.79	S	SVGVMEMRSPLLAGGSPPQPVVPAHKDKSGA	4198900	1240900	4398
Q53F19	413	Uncharacterized protein C17orf85	C17orf85	0.578965	0.0193041	102.77	T	DSDEMDYDLELKMISTPSPKKSMKMTMYADE	0	1043200	7697
Q8NC56	496	LEM domain-containing protein 2	LEMD2	0.379868	0.00691671	102.73	S	RVAGEDMLVWRWTKPSSFSDSER________	0	0	4322
Q8NC56	497	LEM domain-containing protein 2	LEMD2	0.40282	0.00691671	102.73	S	VAGEDMLVWRWTKPSSFSDSER_________	0	0	4323
Q8TBN0	165	Guanine nucleotide exchange factor for Rab-3A	RAB3IL1	0.467432	0.00988213	102.73	T	LQAEVTALKTLVITSTPASPNRELHPQLLSP	0	0	7903
Q9Y3E2	123	BolA-like protein 1	BOLA1	0.782105	0.00417623	102.72	S	ARTPAQWRENSQLDTSPPCLGGNKKTLGTP_	857810	0	6855
Q15459	329	Splicing factor 3A subunit 1	SF3A1	1	0.00182845	102.69	S	YEKFGESEEVEMEVESDEEDDKQEKAEEPPS	439430	633310	2934
Q7Z2Z1	1430	Treslin	TICRR	0.999794	0.00128454	102.63	S	AAPTDSRDDQKGLSLSPQSPPERRGYPGPGL	1895300	1139400	3762
Q7Z2Z1	1433	Treslin	TICRR	0.999994	0.00128454	102.63	S	TDSRDDQKGLSLSPQSPPERRGYPGPGLRSD	1895300	1139400	3763
Q15942	267	Zyxin	ZYX	0.999991	0.00873361	102.62	S	PRGPPASSPAPAPKFSPVTPKFTPVASKFSP	2763800	2246900	2995
Q9NRL2	1546	Bromodomain adjacent to zinc finger domain protein 1A	BAZ1A	0.5	0.000112089	102.58	S	QKLGLHVTPSNVDQVSTPPAAKKSRI_____	955020	958780	5971
P29966	118	Myristoylated alanine-rich C-kinase substrate	MARCKS	0.920447	1.01E-08	102.57	S	VEKEAPAEGEAAEPGSPTAAEGEAASAASST	2011300	4262300	1328
Q96D71	562	RalBP1-associated Eps domain-containing protein 1	REPS1	0.654952	0.00296168	102.53	S	PPPPPRPQPSHSRSSSLDMNRTFTVTTGQQQ	1184600	1127300	4853
Q9NV92	205	NEDD4 family-interacting protein 2	NDFIP2	0.976175	0.0124047	102.53	S	EKAKAAAMAAAAAETSQRIQEEECPPRDDFS	375020	442690	6033
Q9Y597	604	BTB/POZ domain-containing protein KCTD3	KCTD3	0.653447	0.00125832	102.52	S	EEELLKLLDQCDLSTSRCATPNISPATSVVQ	0	503290	6954
O15027	2083	Protein transport protein Sec16A	SEC16A	0.911249	0.000671927	102.5	S	WDRADSGPTQPPLSLSPAPETKRPGQAAKKE	1132300	516480	238
Q7Z406	1969	Myosin-14	MYH14	0.999999	7.49E-09	102.5	S	TRTVRQVFRLEEGVASDEEAEEAQPGSGPSP	4125000	1827000	3792
Q7Z6E9	1221	E3 ubiquitin-protein ligase RBBP6	RBBP6	0.996028	0.00125938	102.5	S	AKKIKLNRETGKKIGSTENISNTKEPSEKLE	782830	0	3857
O95402	314	Mediator of RNA polymerase II transcription subunit 26	MED26	0.999393	5.11E-06	102.48	S	SPSPRPQALDATQVPSPLPLAQPSTPPVRRL	1589700	1672700	763
O95402	322	Mediator of RNA polymerase II transcription subunit 26	MED26	0.61918	5.11E-06	102.48	S	LDATQVPSPLPLAQPSTPPVRRLELLPSAES	1589700	1672700	764
Q9NRL2	270	Bromodomain adjacent to zinc finger domain protein 1A	BAZ1A	0.935209	4.75E-06	102.48	S	DFSYFFPDDPPTFIFSPANRRRGRPPKRIHI	606570	433240	5970
O95402	323	Mediator of RNA polymerase II transcription subunit 26	MED26	0.837454	5.11E-06	102.48	T	DATQVPSPLPLAQPSTPPVRRLELLPSAESP	0	1672700	7238
P42694	1766	Probable helicase with zinc finger domain	HELZ	0.493139	4.90E-06	102.45	S	RGPGRPLYQRRISSSSVQPCSEEVSTPQDSL	0	0	1515
Q86YS7	661	C2 domain-containing protein 5	C2CD5	0.602926	0.000824219	102.45	S	PEPRQRSRLLRSQSESSDEVTELDLSHGKKD	1352100	693320	4033
Q13111	206	Chromatin assembly factor 1 subunit A	CHAF1A	1	0.00141699	102.4	S	GGAGRRGDSQECSPRSCPELTSGPRMCPRKE	6058100	4816200	2402
P46013	584	Antigen KI-67	MKI67	0.954855	0.00358507	102.39	S	VEVKAQSLVISPPAPSPRKTPVASDQRRRSC	1022300	479450	1530
Q9Y2U5	163	Mitogen-activated protein kinase kinase kinase 2	MAP3K2	0.49976	0.000428865	102.39	S	RKKRLSIIGPTSRDRSSPPPGYIPDELHQVA	0	0	6802
Q9Y2U5	164	Mitogen-activated protein kinase kinase kinase 2	MAP3K2	0.49976	0.000428865	102.39	S	KKRLSIIGPTSRDRSSPPPGYIPDELHQVAR	0	0	6803
Q9UEY8	12	Gamma-adducin	ADD3	0.959111	9.66E-05	102.36	T	____MSSDASQGVITTPPPPSMPHKERYFDR	529920	278860	8206
P36507	23	Dual specificity mitogen-activated protein kinase kinase 2	MAP2K2	0.957788	7.70E-09	102.31	S	VLPALTINPTIAEGPSPTSEGASEANLVDLQ	450530	813930	1424
O15541	43	RING finger protein 113A	RNF113A	0.538517	8.78E-06	102.29	S	AGRRKRPACDPEPGESGSSSDEGCTVVRPEK	0	0	329
Q641Q2;Q(284;284		WASH complex subunit FAM21A;WASH complex subunit FAM21C	FAM21A;FAM2	0.999963	0.0218602	102.29	S	EEDIEDIEENTRPKRSRPTSFADELAARIKG	729730	811860	3394
Q641Q2;Q(287;287		WASH complex subunit FAM21A;WASH complex subunit FAM21C	FAM21A;FAM2	0.574717	0.0218602	102.29	T	IEDIEENTRPKRSRPTSFADELAARIKGDAM;IED	729730	811860	7738
P35219	5	Carbonic anhydrase-related protein	CA8	0.997922	0.0015425	102.28	S	____________MADLSFIEDTVAFPEKEEDE	239190	261170	1374
Q96D71	390	RalBP1-associated Eps domain-containing protein 1	REPS1	0.588966	1.78E-07	102.28	S	EDSADVGDQPGEVGYSGSPAEAPPSKSPSMP	0	0	4843
O95772	210	MLN64 N-terminal domain homolog	STARD3NL	0.994125	4.11E-07	102.27	S	RAALIPGGLSDGQFYSPPESEAGSEEAEEKQ	2668300	1578900	806
O95772	218	MLN64 N-terminal domain homolog	STARD3NL	0.944707	4.11E-07	102.27	S	LSDGQFYSPPESEAGSEEAEEKQDSEKPLLE	2668300	0	808
Q9BTC0	1254	Death-inducer obliterator 1	DIDO1	0.344519	1.43E-08	102.27	S	KKPSKYPLCSADAAVSTTPPGSPPPPPPLPE	0	0	5360
Q86U70	306	LIM domain-binding protein 1	LDB1	0.717674	0.000180541	102.27	T	QQPSKRRKRKMSGGSTMSSGGGNTNNSNSKK	1324100	757320	7822
Q9BTC0	1255	Death-inducer obliterator 1	DIDO1	0.638147	1.43E-08	102.27	T	KPSKYPLCSADAAVSTTPPGSPPPPPPLPEP	737710	0	8046
Q9NTI5	1361	Sister chromatid cohesion protein PDS5 homolog B	PDS5B	0.333333	0.000777518	102.24	S	HRVSRRAQQRAESPESSAIESTQSTPQKGRG	0	0	6004
Q9NTI5	1362	Sister chromatid cohesion protein PDS5 homolog B	PDS5B	0.333333	0.000777518	102.24	S	RVSRRAQQRAESPESSAIESTQSTPQKGRGR	0	0	6005
A1X283	291	SH3 and PX domain-containing protein 2B	SH3PXD2B	0.993924	7.62E-07	102.21	S	KKNSGEPLPPKPGPGSPSHPGALDLDGVSRQ	1904500	1268600	21
Q9UBL3	101	Set1/Ash2 histone methyltransferase complex subunit ASH2	ASH2L	0.999968	3.68E-05	102.19	S	EGAGDTSEVMDTQAGSVDEENGRQLGEVELQ	2153900	1807100	6268
O43795	891	Unconventional myosin-Ib	MYO1B	0.90687	0.0132677	102.15	S	RIVQKYFLEMKNKMPSLSPIDKNWPSRPYLF	0	204660	417
O43795	901	Unconventional myosin-Ib	MYO1B	1	0.0132677	102.15	S	KNKMPSLSPIDKNWPSRPYLFLDSTHKELKR	0	204660	419
Q07866	524	Kinesin light chain 1	KLC1	1	0.00494151	102.15	S	VLNDPENMEKRRSRESLNVDVVKYESGPDGG	2191200	1536100	2239
Q9UKJ3	738	G patch domain-containing protein 8	GPATCH8	1	0.00194114	102.15	S	APADSERGPKPEPPGSGSPAPPRRRRRAQDD	2504600	1233800	6442
P23443	447	Ribosomal protein S6 kinase beta-1	RPS6KB1	1	0.00890779	102.07	S	IRSPRRFIGSPRTPVSPVKFSPGDFWGRGAS	0	0	1221
Q8IVF5	1375	T-lymphoma invasion and metastasis-inducing protein 2	TIAM2	1	0.0288718	102.07	S	ILVYKENCKLKKKLPSNSRPAHNSTDLDPFK	15811000	0	4044
Q8IVF5	1377	T-lymphoma invasion and metastasis-inducing protein 2	TIAM2	1	0.0288718	102.07	S	VYKENCKLKKKLPSNSRPAHNSTDLDPFKFR	15811000	0	4045
Q9H0G5	275	Nuclear speckle splicing regulatory protein 1	NSRP1	1	0.00586836	102.07	T	IEETRVNCRREKVIETPENDFKHHRSQNHSR	1082700	1068500	8083
Q96T58	727	Msx2-interacting protein	SPEN	1	0.00304353	102.06	S	DRDRDHERRPIERSQSPVHLRRPQSPGASPS	10783000	7751500	5182
Q9H2G9	14	Golgin-45	BLZF1	0.653853	0.00203018	102.06	S	__MTTKNLETKVTVTSSPIRGAGDGMETEEP	560190	0	5612
Q9BUA3	248	Uncharacterized protein C11orf84	C11orf84	0.999882	5.19E-06	102.03	S	GRPMTKNLDPDPEPPSPDSPTETFAAPAEVR	2274600	873800	5376
Q6P4R8	226	Nuclear factor related to kappa-B-binding protein	NFRKB	0.995921	0.00203838	101.97	S	DEEDLSSWLPSSPARSPSPAVPLRVVPTLST	1859600	997960	3529
O00273	315	DNA fragmentation factor subunit alpha	DFFA	1	0.00130308	101.89	S	TQSLHSLRSISASKASPPGDLQNPKRARQDP	10347000	7070500	115
P49761	283	Dual specificity protein kinase CLK3	CLK3	1	0.0016287	101.75	S	SASSRSQQSSKRSSRSVEDDKEGHLVCRIGD	2214200	1074200	1689
Q9P275	742	Ubiquitin carboxyl-terminal hydrolase 36	USP36	1	0.00131264	101.75	S	PVVASTWPVHRARAVSPAPQSSSRLQPPFSP	0	418640	6225
O60890	654	Oligophrenin-1	OPHN1	0.965756	0.00659224	101.72	S	PIQRSGETDPGRKSPSRPILDGKLEPCPEVD	0	599460	540
P42858	1199	Huntingtin	HTT	0.999997	0.0025775	101.72	S	KGKEKEPGEQASVPLSPKKGSEASAASRQSD	1590500	1241400	1518
P62995	4	Transformer-2 protein homolog beta	TRA2B	0.510748	0.000560085	101.72	S	_____________MSDSGEQNYGERESRSASR	0	107900	1933
Q70EL1	1285	Inactive ubiquitin carboxyl-terminal hydrolase 54	USP54	0.818642	0.013582	101.72	S	CDSQLKHGAPRPGMKSSPHDSHTCVTYPERN	0	0	3686
Q70EL1	1290	Inactive ubiquitin carboxyl-terminal hydrolase 54	USP54	0.987214	0.013582	101.72	S	KHGAPRPGMKSSPHDSHTCVTYPERNHILLH	396640	306310	3688
Q9Y3M2	20	Protein chibby homolog 1	CBY1	0.81772	0.00164079	101.72	S	GNTFSPKKTPPRKSASLSNLHSLDRSTREVE	0	0	6862
Q13443	758	Disintegrin and metalloproteinase domain-containing protein	ADAM9	1	0.0160483	101.71	S	QANPSRQPGSVPRHVSPVTPPREVPIYANRF	491050	696870	2535
Q9ULU4	425	Protein kinase C-binding protein 1	ZMYND8	0.999939	0.00449375	101.71	S	LMSKPVLSGGTGRRISLSDMPRSPMSTNSSV	985650	601500	6503
Q9UN30	150	Sex comb on midleg-like protein 1	SCML1	0.999631	0.00146314	101.71	S	ESYSPTLPVSRRENNSPSNLPRPSFCMEEYQ	698870	1057400	6525
Q13443	761	Disintegrin and metalloproteinase domain-containing protein	ADAM9	1	0.0160483	101.71	T	PSRQPGSVPRHVSPVTPPREVPIYANRFAVP	491050	696870	7544
Q15424	604	Scaffold attachment factor B1	SAFB	1	0.00903437	101.66	S	SQDRKSASREKRSVVSFDKVKEPRKSRDSES	7600600	3036400	2929
Q9Y520	878	Protein PRRC2C	PRRC2C	0.999969	4.34E-06	101.66	S	IRESSEAQVQKFLSRSVEDVRPHHTDANNQS	3329300	207720	6951
Q92560	394	Ubiquitin carboxyl-terminal hydrolase BAP1	BAP1	0.35112	1.33E-08	101.66	Y	AGVGRSRVPVRPPQQYSDDEDDYEDDEEDDV	0	0	8400
Q05086	218	Ubiquitin-protein ligase E3A	UBE3A	0.805367	0.00259499	101.61	S	MEEDSEASSSRIGDSSQGDNNLQKLGPDDVS	0	387010	2183
Q12906	592	Interleukin enhancer-binding factor 3	ILF3	1	0.0105803	101.61	T	KAYAALAALEKLFPDTPLALDANKKKRAPVP	587120	874670	7519
Q9UBC2	229	Epidermal growth factor receptor substrate 15-like 1	EPS15L1	1	0.00260506	101.6	S	RKKTVFPGAVPVLPASPPPKDSLRSTPSHGS	1118200	1161200	6263
Q14676	763	Mediator of DNA damage checkpoint protein 1	MDC1	0.626478	3.68E-06	101.59	S	EVLATQPFCLRESEDSETQPFDTHLEAYGPC	1373500	1068600	2764
Q76I76	1217	Protein phosphatase Slingshot homolog 2	SSH2	0.509815	0.00132667	101.56	S	KMDPLPVACRLPHSSSSENIKSLSHSPGVVK	0	0	3702
Q7Z7B1	416	Phosphatidylinositol-glycan biosynthesis class W protein	PIGW	0.999232	0.00207764	101.56	S	LIKGALVPCSWKLIQSPVTNKKHSESLVPEA	613460	0	3887
O43432	230	Eukaryotic translation initiation factor 4 gamma 3	EIF4G3	1	0.00907283	101.54	S	DQKQEEKPKPDPVLKSPSPVLRLVLSGEKKE	2067700	1622300	386
Q8TD16	582	Protein bicaudal D homolog 2	BICD2	1	0.00907283	101.54	S	TSPGGRTSPEARGRRSPILLPKGLLAPEAGR	2782400	1525300	4462
P35612	699	Beta-adducin	ADD2	0.971352	3.79E-05	101.53	S	TESVTSGPMSPEGSPSKSPSKKKKKFRTPSF	5552800	2627700	1406
Q9BRS8	447	La-related protein 6	LARP6	0.999398	0.00122625	101.53	S	WVRRRRQAEMGTQEKSPGTSPLLSRKMQTAD	2380800	1150100	5329
Q9BRS8	451	La-related protein 6	LARP6	0.995575	0.00122625	101.53	S	RRQAEMGTQEKSPGTSPLLSRKMQTADGLPV	2380800	1150100	5330
Q14676	378	Mediator of DNA damage checkpoint protein 1	MDC1	0.999947	4.99E-06	101.53	T	APGLAHLQESQAGSDTDVEEGKAPQAVPLEK	0	3722400	7602
Q6RI45	1763	Bromodomain and WD repeat-containing protein 3	BRWD3	0.793975	1.76E-06	101.5	S	KTRNQGRRTVLYNDDSDNDNFVSTEDPLNLG	1519800	0	3579
Q9ULJ3	983	Zinc finger and BTB domain-containing protein 21	ZBTB21	0.930557	5.01E-06	101.49	S	AHKESEVCPVPTNSPSPPPLPPPPPLPKIQP	5685000	0	6477
Q9ULJ3	979	Zinc finger and BTB domain-containing protein 21	ZBTB21	0.840327	5.01E-06	101.49	T	SEESAHKESEVCPVPTNSPSPPPLPPPPPLP	5685000	2213100	8237
O15014	804	Zinc finger protein 609	ZNF609	0.973813	0.000817337	101.48	S	KAEADKIYSFTDNAPSPSIGGSSRLENTTPT	1765800	1397800	226
P62987;P(65;65;65		Ubiquitin-60S ribosomal protein L40;Ubiquitin;60S ribosomal	UBA52;RPS27	0.950501	0.00208658	101.46	S	LEDGRTLSDYNIQKESTLHLVLRLRGGIIEP	1239800	1793800	977
P62987;P(66;66;66		Ubiquitin-60S ribosomal protein L40;Ubiquitin;60S ribosomal	UBA52;RPS27	0.5	0.00208658	101.46	T	EDGRTLSDYNIQKESTLHLVLRLRGGIIEPS	0	1793800	7269
Q13595	98	Transformer-2 protein homolog alpha	TRA2A	1	0.00348364	101.45	S	RSRSYTPEYRRRRSRSHSPMSNRRRHTGSRA	291590	1835400	2590
Q13595	103	Transformer-2 protein homolog alpha	TRA2A	0.936776	0.0144438	101.45	S	TPEYRRRRSRSHSPMSNRRRHTGSRANPDPN	291590	476010	2592
Q13595	96	Transformer-2 protein homolog alpha	TRA2A	1	0.0144438	101.45	S	RSRSRSYTPEYRRRRSRSHSPMSNRRRHTGS	291590	476010	2595
Q68EM7	570	Rho GTPase-activating protein 17	ARHGAP17	0.993119	1.41E-08	101.45	S	GGTVPSSAGILEQGPSPGDGSPPKPKDPVSA	1863000	0	3427
Q8N1F8	389	Serine/threonine-protein kinase 11-interacting protein	STK11IP	0.632528	0.0029316	101.42	S	LHKVKSRVRVRRASISEPSDTDPEPRTLNPS	0	0	4217
Q14008	828	Cytoskeleton-associated protein 5	CKAP5	0.505505	6.46E-07	101.41	S	QGQSPPAPTRGISKHSTSGTDEGEDGDEPDD	0	0	2644
Q14008	830	Cytoskeleton-associated protein 5	CKAP5	0.505505	6.46E-07	101.41	S	QSPPAPTRGISKHSTSGTDEGEDGDEPDDGS	0	0	2645
O43719	616	HIV Tat-specific factor 1	HTATSF1	1	0.0241153	101.39	S	EFEDDGSEKVLDEEGSEREFDEDSDEKEEEE	2445900	1696100	415
Q86VQ1	223	Glucocorticoid-induced transcript 1 protein	GLCCI1	0.965379	0.00133853	101.39	S	WAEEGAEKRSHQRSASWGSADQLKEQIAKLR	3417800	1845500	3963
Q01082	2164	Spectrin beta chain, non-erythrocytic 1	SPTBN1	0.893887	0.00139388	101.38	S	EMVNGATEQRTSSKESSPIPSPTSDRKAKTA	0	0	2042
Q8NC51	394	Plasminogen activator inhibitor 1 RNA-binding protein	SERBP1	0.862657	0.00165918	101.36	S	GGRPNRGSRTDKSSASAPDVDDPEAFPALA_	1252800	490060	4321
Q8TEU7	1157	Rap guanine nucleotide exchange factor 6	RAPGEF6	0.999992	0.00148742	101.35	S	TKNLSEKRSAKSSEMSPVPMRSAGQTTKAHL	1158400	794760	4490
Q96MW1	139	Coiled-coil domain-containing protein 43	CCDC43	0.999408	9.40E-09	101.34	T	EKQRKAALLAQYADVTDEEDEADEKDDSGAT	5703900	2842900	7993
Q9Y294	176	Histone chaperone ASF1A	ASF1A	0.605829	5.09E-06	101.33	T	EDAESSNPNLQSLLSTDALPSASKGWSTSEN	0	0	8280
Q9C0C2	1141	182 kDa tankyrase-1-binding protein	TNKS1BP1	0.661481	0.00134331	101.32	S	IIGNDRVSGAGFSPSSKMEGGHFVPPGKTTA	0	0	5526
Q9UIU6	3	Homeobox protein SIX4	SIX4	0.25037	0.00140403	101.32	S	______________MSSSSPTGQIASAADIKQ	0	0	6388
Q9UIU6	4	Homeobox protein SIX4	SIX4	0.25037	0.00140403	101.32	S	_____________MSSSSPTGQIASAADIKQE	0	0	6389
Q9UIU6	5	Homeobox protein SIX4	SIX4	0.25037	0.00140403	101.32	S	____________MSSSSPTGQIASAADIKQEN	0	0	6390

P22059	240	Oxysterol-binding protein 1	OSBP	0.99948	0.00210202	101.3	S	NDLIAKHGTALQRSLSELESLKLPAESNEKI	589630	447120	1205
Q05519	447	Serine/arginine-rich splicing factor 11	SRSF11	0.962377	0.0112546	101.28	T	YDSEKEKKEEKKPIETEGSPKTKECSVEKGTG	12772000	0	7496
Q9Y6A5	249	Transforming acidic coiled-coil-containing protein 3	TACC3	0.5	0.00102388	101.23	T	EECRHGGVCAPAAVATSPPGAIPKEACGGAP	0	0	8317
Q9BSL1	98	Ubiquitin-associated domain-containing protein 1	UBAC1	1	0.00917661	101.21	S	IQDQDVLLLIKKRAPSPLPKMADVSAEEKKK	607700	0	5344
Q9H501	663	ESF1 homolog	ESF1	0.999999	9.02E-07	101.2	S	KQKALAEEASEEELPSDVDLNDPYFAEEVKQ	19031000	11125000	5707
Q8N108	131	Mesoderm induction early response protein 1	MIER1	0.517721	1.50E-08	101.04	S	ENIKDSSGQEDETQSSNDDPSQSVASQDAQE	1750600	0	4202
Q96CB8	127	Integrator complex subunit 12	INTS12	0.646801	0.00170711	101.04	S	VDIPKKPRLEKPETQSSPITVQSSKDLPMAD	4864700	0	4825
Q9UNZ2	272	NSFL1 cofactor p47	NSFL1C	0.981155	7.48E-06	101	S	GQKLGSTAPQVLSTSSPAQQAENEAKASSSI	1882100	1197900	6553
O60216	175	Double-strand-break repair protein rad21 homolog	RAD21	0.99833	8.67E-09	100.98	S	FGDFGMDDREIMREGSAFEDDDMLVSTTTSN	1737000	1449100	435
Q6PJG2	661	ELM2 and SANT domain-containing protein 1	ELMSAN1	1	0.0132567	100.97	S	ERSFELPPYTPPPILSPVREGSGLYFNAIIS	807350	416440	3548
Q9P1Y5	349	Calmodulin-regulated spectrin-associated protein 3	CAMSAP3	0.414146	8.12E-05	100.97	S	SPRGTEASPPQNNSGSSSPVFTFRHPLLSSG	0	0	6193
Q9P1Y5	351	Calmodulin-regulated spectrin-associated protein 3	CAMSAP3	0.832583	8.12E-05	100.97	S	RGTEASPPQNNSGSSSPVFTFRHPLLSSGGP	5065500	1422300	6195
Q6PJG2	655	ELM2 and SANT domain-containing protein 1	ELMSAN1	0.99579	0.0132567	100.97	T	LADHPSERSFELPPYTPPPILSPVREGSGLY	807350	416440	7763
Q58WW2	649	DDB1- and CUL4-associated factor 6	DCAF6	0.762095	0.000136188	100.95	S	EATGPSAHEETSTRDSALQDTDDSDDDPVLI	0	1399400	3151
Q5SY16	84	Polynucleotide 5'-hydroxyl-kinase NOL9	NOL9	0.997614	8.14E-09	100.95	S	VSRAAAARRPNTATPSPIPSPTPASEPESEP	4316200	2570800	3229
Q8NHV4	518	Protein NEDD1	NEDD1	0.454087	8.13E-05	100.95	S	LVTSGAESGNLNTSPSSNQTRNSEKFEKPEN	0	0	4421
Q9GZR1	350	Sentrin-specific protease 6	SENP6	0.999984	3.51E-05	100.94	S	SSDDDDDNDRTNRRESISPQPADSACSSPAP	763420	626580	5551
Q9NVD7	10	Alpha-parvin	PARVA	0.655448	0.0007437	100.93	S	______MATSPQKSPSVPKSPTPKSPPSRKK	2335100	0	6036
Q8N556	277	Actin filament-associated protein 1	AFAP1	0.724907	7.02E-06	100.9	S	PPSSPVHKAELEKKLSSERPSSDGEGVVENG	1386000	0	4275
Q9H792	837	Pseudopodium-enriched atypical kinase 1	PEAK1	0.505298	7.45E-06	100.9	S	TSQPSGEAEAPQTTDSPTTKVQKDPSIKPVT	0	1047500	5756
A1L170	223	Uncharacterized protein C1orf226	C1orf226	0.996507	0.0021411	100.88	S	QDESKLKMTECRRASSPSLIERNGFKLSLSP	2401000	2007400	11
Q9UHF7	216	Zinc finger transcription factor Trps1	TRPS1	1	3.77E-05	100.88	S	KTDLLVNDPDPAPLSPELQDFKCNICGYGY	358220	464620	6354
Q8NDV7	943	Trinucleotide repeat-containing gene 6A protein	TNRC6A	0.957441	0.00214663	100.82	S	SNQSLGWGDSSKPVSSPDWNKQQDIVGSWGI	546880	0	4361
Q9NRH2	607	SNF-related serine/threonine-protein kinase	SNRK	0.904566	4.39E-07	100.81	S	GVDKASPSENNAGGGSPSSGSGGNPTNTSGT	0	0	5964
P49454	1722	Centromere protein F	CENPF	0.992798	7.64E-07	100.79	S	EKITETGAVKPTGECSGEQSPDTNYEPPGED	4839100	5292500	1663
Q9H1A4	518	Anaphase-promoting complex subunit 1	ANAPC1	0.977841	0.000403322	100.77	S	VVRVGKVFIPGLPAPSLTMSNTMPRPSTPLD	956120	543900	5592
Q9H1A4	522	Anaphase-promoting complex subunit 1	ANAPC1	0.62511	0.000403322	100.77	S	GKVFIPGLPAPSLTMSNTMPRPSTPLDGVST	556510	319690	5593
O96013	187	Serine/threonine-protein kinase PAK 4	PAK4	0.962106	0.000138817	100.77	T	ESSRDKRPLSGPDVGTPQPAGLASGAKLAAG	0	0	7248
Q02952	96	A-kinase anchor protein 12	AKAP12	0.999999	3.69E-06	100.76	S	LNGQKGALNGQGALNSQEEEEVIVTEVGQRD	0	335950	2116
P35579	1943	Myosin-9	MYH9	1	0.0139459	100.73	S	FVVPRRMARKGAGDGSDEEVDGKADGAEAKP	0	447610	1394
Q96T37	127	Putative RNA-binding protein 15	RBM15	0.492754	0.0015318	100.69	S	YDTGGGSSSSRLHSYSSPSTKNSSGGGESRS	0	0	5161
Q96T37	128	Putative RNA-binding protein 15	RBM15	0.492754	0.0015318	100.69	S	DTGGGSSSSRLHSYSSPSTKNSSGGGESRSS	0	0	5162
Q3L8U1	2331	Chromodomain-helicase-DNA-binding protein 9	CHD9	0.926189	0.000754445	100.69	T	PTPPGAGVKEEHDQSTQMSKVKKHVREKEFT	779360	704700	7669
Q14671	212	Pumilio homolog 1	PUM1	0.79309	3.99E-06	100.68	S	SFHVNSEVNSVLSPRSESGGLGVSMVEYVLS	593980	0	2756
Q9Y4F5	655	Centrosomal protein of 170 kDa protein B	CEP170B	1	7.59E-06	100.67	S	GAAPQAEHQGLPVPGSPGGQKWVSRWASLAD	2896100	2470900	6916
Q9H4L7	33	SWI/SNF-related matrix-associated actin-dependent regulator	SMARCAD1	0.803683	2.95E-09	100.66	S	EEAPEATPQPSQPGPSSPISLSAEEENAEGE	9068500	2892100	5689
Q9H4L7	39	SWI/SNF-related matrix-associated actin-dependent regulator	SMARCAD1	0.920022	2.95E-09	100.66	S	TPQPSQPGPSSPISLSAEEENAEGEVSRANT	7745000	8530000	5692
Q9BW85	319	Coiled-coil domain-containing protein 94	CCDC94	0.973496	2.23E-07	100.65	S	ASSLSQLGAYLDSDDSNGSN____________	9872300	6730200	5423
P67809	174	Nuclease-sensitive element-binding protein 1	YBX1	0.942415	5.69E-07	100.62	S	QQNYQNSESGEKNEGSESAPEGQAQQRRPYR	0	0	1939
Q12888	1701	Tumor suppressor p53-binding protein 1	TP53BP1	0.998142	4.80E-07	100.62	S	SATVKPGAVGAGEFVSPCESGDNTGEPSALE	1292700	1530500	2355
Q12888	1705	Tumor suppressor p53-binding protein 1	TP53BP1	0.995043	4.80E-07	100.62	S	KPGAVGAGEFVSPCESGDNTGEPSALEEQRG	1292700	1530500	2356
Q68EM7	676	Rho GTPase-activating protein 17	ARHGAP17	0.7328	2.34E-06	100.44	S	QHPPSLSPKPPTRSPSPPTQHTGQPPGQPSA	1979400	0	3430
Q6ICG6	267	Uncharacterized protein KIAA0930	KIAA0930	0.78446	5.72E-06	100.44	S	GHAEMAVSRVSTGDTSPCGTEEDSSPASPMH	6263000	3422900	3472
Q13542	70	Eukaryotic translation initiation factor 4E-binding protein	EIF4EBP2	0.999998	7.11E-07	100.44	T	DRKFLLDRRNSPMAQTPPCHLPNIPGVTSPG	8213600	5583800	7557
Q68EM7	679	Rho GTPase-activating protein 17	ARHGAP17	0.768252	2.80E-06	100.44	T	PSLSPKPPTRSPSPPTQHTGQPPGQPSAPSQ	23431000	0	7745
O95785	1106	Protein Wiz	WIZ	1	0.00277958	100.4	S	RTQSRPGGPPNPPGPSPKALAKMMGGAGPGS	532620	0	817
P49792;Q	2276;130	E3 SUMO-protein ligase RanBP2;RanBP2-like and GRIP domain-co	RANBP2;RGPI	0.996784	0.00542028	100.4	S	GESTTGFNFSFKSALSPSKSPAKLNQSGTSV	3170100	3874000	1715
P49792;Q	2280;130	E3 SUMO-protein ligase RanBP2;RanBP2-like and GRIP domain-co	RANBP2;RGPI	0.997346	0.00542028	100.4	S	TGFNFSFKSALSPSKSPAKLNQSGTSVGTDE	3170100	3874000	1716
Q03164	523	Histone-lysine N-methyltransferase 2A;MLL cleavage product N	KMT2A	0.967094	7.78E-06	100.39	S	EVHPPLPISQSPENESNDRRSRRYSVSERSF	0	0	2146
Q8N108	488	Mesoderm induction early response protein 1	MIER1	0.993661	9.70E-06	100.39	S	ERPAKRRRVNSNGKESPGSSEFFQEAVSHGK	5985300	580500	4203
Q8N108	483	Mesoderm induction early response protein 1	MIER1	0.999772	9.70E-06	100.39	S	FDEKSERPAKRRRVNSNGKESPGSSEFFQEA	5937800	3782900	4207
Q9ULT8	631	E3 ubiquitin-protein ligase HECTD1	HECTD1	0.499853	4.89E-07	100.39	S	ARLGVISKVSTLAGPSSDDENEEESKPEKED	0	0	6501
Q99607	186	ETS-related transcription factor Elf-4	ELF4	0.398178	0.00278287	100.38	S	TGKSKKRIRKTKGNRSTSPVTDPSIPIRKKS	0	0	5229
Q99607	187	ETS-related transcription factor Elf-4	ELF4	0.398178	0.00278287	100.38	T	GKSKKRIRKTKGNRSTSPVTDPSIPIRKKSK	0	0	8027
P06748	10	Nucleophosmin	NPM1	0.999912	5.29E-06	100.28	S	______MEDSMDMDMSPLRPQNYLFGCELKA	9782400	2659500	922
Q9BRY0	125	Zinc transporter ZIP3	SLC39A3	0.921797	1.57E-08	100.24	S	KEKPSFIDLETFNAGSDVGSDSEYESPFMGG	5815300	0	5337
Q9BRY0	129	Zinc transporter ZIP3	SLC39A3	0.842557	1.57E-08	100.24	S	SFIDLETFNAGSDVGSDSEYESPFMGGARGH	5815300	3744400	5338
Q8TBN0	164	Guanine nucleotide exchange factor for Rab-3A	RAB3IL1	0.442644	0.00280629	100.22	S	MLQAEVTALKTLVITSTPASPNRELHPQLLS	0	0	4453
Q7Z589	1213	Protein EMSY	EMSY	0.418731	0.00351504	100.19	S	ANSTPQQQKCRESCSSPSTVGSSLTTRKIDP	0	0	3835
Q9ULJ3	435	Zinc finger and BTB domain-containing protein 21	ZBTB21	0.90656	0.00405835	100.18	S	GASPVTEVRIKTEPSSPLSDPSDIIRVTVGD	1045900	818240	6483
O60271	109	C-Jun-amino-terminal kinase-interacting protein 4	SPAG9	1	0.0018101	100.11	S	ALRKHAEEKFIEFEDSQEQEKKDLQTRVESL	1378100	768970	456
P28749	1037	Retinoblastoma-like protein 1	RBL1	1	0.00234645	100.11	S	RQGEQRTKKRVIAIDSDAESPAKRVCQENDD	0	677050	1313
Q01105	7	Protein SET	SET	1	0.00909719	100.09	S	_________MAPKRQSPLPPQKKKPRPPPAL	1998000	976530	2052
Q9H981	132	Actin-related protein 8	ACTR8	1	0.00221537	100.09	S	IWSKKMSNGTRRIPVSPEQARSYNKQMRPAI	868250	503500	5799
Q6VY07	46	Phosphofurin acidic cluster sorting protein 1	PACS1	1	1.14E-06	100.08	T	QPPPQQQQQQPPQQPTPPKLAQATSSSSSTS	1834400	1776300	7774
Q96T23	1371	Remodeling and spacing factor 1	RSF1	0.755374	6.28E-05	100.07	T	KVGSPLDYSLVDLPSTNGQSPGKAIENLIGK	0	714710	8013
Q14203	105	Dynactin subunit 1	DCTN1	0.51189	3.92E-06	100.04	S	RQSQIQVFEDGADTTSPETPDSSASKVLKRE	772350	893870	2721
Q96PV7	785	Protein FAM193B	FAM193B	1	0.00157533	100.04	S	GEGSRGSRPGPGWAGSPKTEKEKGSSWRNWP	1497700	811300	5063
Q9UQ35	1132	Serine/arginine repetitive matrix protein 2	SRRM2	0.999926	0.00221996	100.04	S	DRGEPSASPMLKSGMSPEQSRFQSDSSSYPT	0	848770	6684
Q9NQW6	323	Actin-binding protein anillin	ANLN	1	0.00955194	100.02	S	SCEGQNPELLPKTPISPLKTGVSKPIVKSTL	1502800	1204000	5934
P49792;Q	2450;147	E3 SUMO-protein ligase RanBP2;RanBP2-like and GRIP domain-co	RANBP2;RGPI	0.999999	0.0122626	100.02	T	IFDEAKTAQEKDSLITPHVSRSSTPRESPCG	4585800	4103300	7422
Q3YEC7	425	Rab-like protein 6	RABL6	0.829775	9.44E-09	99.987	S	ARDEKKVGAKAAQQDSDSDGEALGGNPMVAG	1130800	416540	3079
Q96HC4	137	PDZ and LIM domain protein 5	PDLIM5	0.957987	0.00222594	99.973	S	NMAYNKAPRPFGSVSSPKVTSIPSPSSAFTP	749100	456900	4945
Q96JM3	476	Chromosome alignment-maintaining phosphoprotein 1	CHAMP1	1	0.00340772	99.973	S	ASLDFPESQKSSRGGSPDLWKSSFFIEPQKP	4143700	3023000	4968
Q96HA1	348	Nuclear envelope pore membrane protein POM 121	POM121	0.89602	2.16E-06	99.957	S	LDGQENKRRRHDSSGSGHSAFEPLVANGVPA	994210	0	4939
Q96HA1	351	Nuclear envelope pore membrane protein POM 121	POM121	0.89602	2.16E-06	99.957	S	QENKRRRHDSSGSGHSAFEPLVANGVPASFV	994210	0	4940
Q96ST3	272	Paired amphipathic helix protein Sin3a	SIN3A	0.752411	1.20E-05	99.957	Y	QAHTPASQQTPPLPPYASPRSPPVQPHTPVT	0	894320	8415
Q9P0K7	341	Ankycorbin	RAI14	0.956375	1.84E-08	99.954	S	LSDSTTGADSLLDISSEADQQDLLSLLQAKV	966740	349120	6185
Q8ND24	501	RING finger protein 214	RNF214	0.857896	1.01E-06	99.93	S	FPILNPALSQPSQPSSPLPGSHGRNSPGLGS	1878200	1851800	4336
Q8TBB5	418	Kelch domain-containing protein 4	KLHDC4	1	0.0096708	99.93	S	TAPGSAGQPRSEDEDSLEEAGSPAPGPCPRS	0	0	4451
Q8WV99	172	AN1-type zinc finger protein 2B	ZFAND2B	0.820299	6.21E-06	99.923	T	STSTVPSPSQTMPSCTSPSRATTRSPSWTAP	0	0	7918
A6NDB9	544	Paralemmin-3	PALM3	1	0.014006	99.915	S	EKTQGTEGDLNLEQGSREGSESQAEEMNEAG	0	333550	35
Q86WB0	394	Nuclear-interacting partner of ALK	ZC3HC1	0.5	0.00295864	99.915	S	TRSMGTGDTPGLEVPSSPLRKAKRARLCSSS	993910	506800	3980
Q9UI36	493	Dachshund homolog 1	DACH1	1	0.0161246	99.915	S	RVETSVIKERVPDSPSPAPSLEEGRRPGSHP	0	530690	6374
Q9UI36	497	Dachshund homolog 1	DACH1	1	0.0161246	99.915	S	SVIKERVPDSPSPAPSLEEGRRPGSHPSSHR	0	530690	6375
Q92733	157	Proline-rich protein PRCC	PRCC	0.999999	0.000174801	99.891	S	KEPVKIAAPELHKGDSDSEEDEPTKKKTILQ	39112000	16547000	4692
Q6YBV0	35	Proton-coupled amino acid transporter 4	SLC36A4	0.82781	6.23E-06	99.881	T	MDVMRPLINEQNFDGTSDEEHEQELLPVQKH	142060	0	7779
O15063	723	Uncharacterized protein KIAA0355	KIAA0355	1	6.00E-06	99.873	S	HTPLTPQPGLAPQQQSPKQQQPQVQYYQHLL	3595400	2743200	272
Q14202	774	Zinc finger MYM-type protein 3	ZMYM3	0.847612	7.39E-07	99.859	S	NLDTQSGPESLLNSQSPESKPQTPSQTKVEN	1900100	999390	2706
Q99549	279	M-phase phosphoprotein 8	MPHOSPH8	0.577704	1.65E-08	99.859	S	SESSVLNDSPFPEDDSEGLHSDSREEKQNTK	407860	0	5204
Q5VUJ6	521	Leucine-rich repeat and calponin homology domain-containing	LRCH2	0.753572	8.13E-06	99.846	S	ECEKSVSADEVNSPLSPLTWQPLENQKDQID	0	696680	3351
Q9NRH2	518	SNF-related serine/threonine-protein kinase	SNRK	0.974633	0.00144791	99.844	S	ENLPPKLSRLKMNIASPGTVHKRYHRRKSQG	0	0	5969
Q9UDY2	398	Tight junction protein ZO-2	TJP2	0.805118	4.76E-07	99.836	S	RDSQQTLINIPSLNDSDSEIEDISEIESNRS	18751000	5669200	6279
Q7Z5K2	223	Wings apart-like protein homolog	WAPAL	0.866496	0.00141915	99.834	S	NDTWNSQFGKRPESPSEISPIKGSVRTGLFE	663880	0	3842
Q9BWF3	86	RNA-binding protein 4	RBM4	0.862721	0.00286551	99.834	S	KNKSKTSTKLHVGNISPTCTNKELRAKFEEY	828930	0	5428
Q8IZD4	275	mRNA-decapping enzyme 1B	DCP1B	0.990827	0.0034353	99.815	S	QQEKLPIRQGVVRSLSYEEPRRHSPPIEKQL	681930	0	4184
Q5VYV7	396	Protein SLX4IP	SLX4IP	0.986504	0.00159203	99.788	S	TGLATNTERLSTIQNSPTKKRKKYERGH___	1308400	538270	3363
Q8IWZ3	1679	Ankyrin repeat and KH domain-containing protein 1	ANKHD1	0.994946	0.00962393	99.788	S	VSLPLSSPNIKLNLTSPKRGQKREEGWKEVV	1862200	1049900	4083
Q8TEU7	1154	Rap guanine nucleotide exchange factor 6	RAPGEF6	0.767666	0.00159203	99.788	S	GTLTKNLSEKRSAKSSEMSPVPMRSAGQTTK	0	0	4489
P29966	135	Myristoylated alanine-rich C-kinase substrate	MARCKS	0.854886	1.41E-08	99.753	S	TAAEGEAASASSTSSPKAEDGATPSPSNET	4217900	3066100	1333
Q2TBE0	479	CWF19-like protein 2	CWF19L2	1	0.00224668	99.752	S	PKKEHLRDTKSTFAGSPERESIHILSVDEKN	1371600	1226200	3049
O75592	2787	E3 ubiquitin-protein ligase MYCBP2	MYCBP2	0.999826	0.00240301	99.669	S	LKSDAAKLRSDSHSRSLSPNHNTLQTLKSDG	4883500	2453100	646
O75592	2789	E3 ubiquitin-protein ligase MYCBP2	MYCBP2	0.999237	0.00240301	99.669	S	SDAAKLRSDSHSRSLSPNHNTLQTLKSDGRM	4376400	2224900	647
Q04727	206	Transducin-like enhancer protein 4	TLE4	0.567995	0.00121394	99.669	S	HDNDHQRDRDSIKSSSVSPSASFRGAEKHRN	0	892450	2180
P49736	5	DNA replication licensing factor MCM2	MCM2	0.558514	0.0130298	99.663	S	___________MAESSESFTMASSPAQRRRG	848080	0	1674
Q15185	113	Prostaglandin E synthase 3	PTGES3	0.999987	0.0152068	99.663	S	WLSVDFNNWKDWEDDSDEDMSNFDRFSEMMN	0	0	2892
Q9P1Y6	5	PHD and RING finger domain-containing protein 1	PHRF1	1	0.000791636	99.653	S	___________MDDDSLDELVARSPGPDGHP	263790	253170	6203
Q4ADV7	992	RAB6A-GEF complex partner protein 1	RIC1	0.929563	7.61E-07	99.614	T	HMIRFLKAIGSGESETPPSTPTAQEPSSSGG	1120300	705450	7675
Q9H1K0	217	Rabenosyn-5	RBSN	0.924009	0.00020262	99.603	S	LTSASKESLSTHTSPSQSPNSVHGSRRGSIS	9962000	3665000	5609
Q9H1K0	214	Rabenosyn-5	RBSN	0.737901	0.00020262	99.603	T	ANKLTSASKESLSTHTSPSQSPNSVHGSRRG	0	3665000	8089
Q9UQ35	1537	Serine/arginine repetitive matrix protein 2	SRRM2	1	0.000752392	99.6	S	SVDQKTVARTPLGQRSRSGSSQELDVKPSAS	0	998980	6706
P04049;P	621;582	RAF proto-oncogene serine/threonine-protein kinase;Serine/t	RAF1;ARAF	0.993584	0.00226392	99.568	S	IELLQHSLPKINRSASEPSLHRAAHTEDINA;IEL	3636900	2644100	868
Q08170	456	Serine/arginine-rich splicing factor 4	SRSF4	0.999786	0.00101713	99.568	S	RSRSRSNSKSKPNLPSESRSRSKSASKTRSR	6567700	950890	2244
Q92667	445	A-kinase anchor protein 1, mitochondrial	AKAP1	0.810219	0.00348493	99.53	S	PPPKTYVSCLKSLLSSPTKDSKPNISAHHIS	3858500	0	4688
P52701	830	DNA mismatch repair protein Msh6	MSH6	1	0.00227031	99.5	S	LPDLERLLSKIHNVGSPLKSQNHPDSRAIMY	2841100	1528700	1817
P27816	96	Microtubule-associated protein 4	MAP4	0.446477	1.51E-06	99.454	T	PTLLANGGHGVEGSDTTGSPTEFLEEKMAYQ	0	0	7338
P27816	97	Microtubule-associated protein 4	MAP4	0.7948	1.51E-06	99.454	T	TLLANGGHGVEGSDTTGSPTEFLEEKMAYQE	2915400	0	7339
Q14168	139	MAGUK p55 subfamily member 2	MPP2	0.574544	4.31E-06	99.441	Y	FQSLLETHDSVASKTYETPPPSPGLDPTFSN	0	0	8381
Q7L590	85	Protein MCM10 homolog	MCM10	0.999987	1.20E-06	99.423	T	EKENLATLFGDMEDLTDEEEVPASQSTENRV	6082500	4731100	7792
Q9UK58	342	Cyclin-L1	CCNL1	0.902539	2.62E-06	99.414	S	ALSTLGGFSPASKPSSPREVKAEEKSPISIN	2866200	0	6422
Q9UKV3	420	Apoptotic chromatin condensation inducer in the nucleus	ACIN1	0.614718	1.10E-05	99.413	T	PEQTASEEETPPPLLTKEASSPPPHPQLHSE	0	1847500	8234
Q6PKG0	546	La-related protein 1	LARP1	0.564336	0.000924913	99.409	S	KTEEVSNLKTLPKGLSASLPDLDSENWIEVK	513010	0	3558
Q9UQ35	1188	Serine/arginine repetitive matrix protein 2	SRRM2	1	0.000907371	99.407	S	QEDATASPPRQKDKFSPFPVQDRPESSLVFK	2322300	1943700	6614
P48634	609	Protein PRRC2A	PRRC2A	0.643519	0.00335365	99.392	T	PSKEGPEPPEEVPPPTTPPVPKVEPKGDGIG	1486800	879670	7402
O95772	214	MLN64 N-terminal domain homolog	STARD3NL	0.995673	5.31E-07	99.387	S	IPGGLSDGQFYSPPESEAGSEEAEEKQDSEK	0	1578900	807
P36915	32	Guanine nucleotide-binding protein-like 1	GNL1	1	0.00592928	99.371	S	QDKRERKRGLQDGLRSSSNSRSGSRERREEQ	0	0	1434
P36915	33	Guanine nucleotide-binding protein-like 1	GNL1	1	0.00592928	99.371	S	DKRERKRGLQDGLRSSSNSRSGSRERREEQT	0	0	1435
P36915	34	Guanine nucleotide-binding protein-like 1	GNL1	1	0.00592928	99.371	S	KRERKRGLQDGLRSSSNSRSGSRERREEQTD	0	0	1436
P36915	36	Guanine nucleotide-binding protein-like 1	GNL1	1	0.00592928	99.371	S	ERKRGLQDGLRSSSNSRSGSRERREEQTDTS	0	0	1437
Q9H4L7	212	SWI/SNF-related matrix-associated actin-dependent regulator	SMARCAD1	0.49589	9.94E-05	99.343	S	MFGDAGGGPRKRKLSSSSEPYEEDEFNDDQS	0	0	5695
Q8IZL8	1073	Proline-, glutamic acid- and leucine-rich protein 1	PELP1	0.828214	1.77E-11	99.29	S	PSAPPTLLEEETEDGSDKVQPPPETPAEEEM	841380	745880	4187
A1L170	249	Uncharacterized protein C1orf226	C1orf226	0.999763	4.23E-05	99.273	S	LSLSPISLAESWEDGSPPPQARTSSLDNEGP	2092300	0	12
Q12888	398	Tumor suppressor p53-binding protein 1	TP53BP1	0.99917	0.00018213	99.273	S	EQEGRQDKPMDTSVLSEEGGEPFQKKLQSGE	3157700	2836600	2357
Q9UQ35	351	Serine/arginine repetitive matrix protein 2	SRRM2	0.988999	2.70E-06	99.269	S	EDKDKDKKEKSATRPSPSPERSSTGPEPPAP	2616400	1925800	6663
Q9UQ35	353	Serine/arginine repetitive matrix protein 2	SRRM2	0.99931	2.70E-06	99.269	S	KDKDKKEKSATRPSPSPERSSTGPEPPAPTP	3965500	2656500	6664
Q9Y2J2	7	Band 4.1-like protein 3;Band 4.1-like protein 3, N-terminal	EPB41L3	0.389605	1.32E-06	99.269	S	_________MTTESGSDSESKPDQEAEPQEA	0	0	6786
Q9Y2J2	9	Band 4.1-like protein 3;Band 4.1-like protein 3, N-terminal	EPB41L3	0.398148	1.32E-06	99.269	S	_______MTTESGSDSESKPDQEAEPQEAAG	0	0	6787
Q9NV56	194	MRG/MORF4L-binding protein	MRGBP	0.481674	0.00425547	99.238	S	RSRVTDKVLTANSNPSSPSAAKRRRT_____	0	0	6026
Q9NV56	195	MRG/MORF4L-binding protein	MRGBP	0.481674	0.00425547	99.238	S	SRVTDKVLTANSNPSSPSAAKRRRT______	0	0	6027

Q96QU8	204	Exportin-6	XPO6	0.964508	1.99E-08	99.171	T	GILETVWDKHSVTAATPPPSPTSGESGDLLS	30070000	19668000	7998
Q9UPT8	808	Zinc finger CCCH domain-containing protein 4	ZC3H4	0.712435	1.07E-06	99.148	S	QDRENEEGDTGNWYSSDEDEGGSSVTSILKT	0	695010	6573
O75569	18	Interferon-inducible double-stranded RNA-dependent protein k	PRKRA	0.982966	0.00163574	99.136	S	QSRHRAEAPPLEREDSGTFSLGKMITAKPGK	5199300	2118000	643
O75694	992	Nuclear pore complex protein Nup155	NUP155	0.798243	0.00163574	99.136	S	DTLQELVNQSKAAPQSPSVPKKPGPPVLSSD	4822200	0	655
Q92541	58	RNA polymerase-associated protein RTF1 homolog	RTF1	0.993171	4.18E-05	99.097	S	KKRKGRVVIDSDTEDSGSDENLDQELLSLAK	486870	195800	4630
Q92541	60	RNA polymerase-associated protein RTF1 homolog	RTF1	0.999228	4.18E-05	99.097	S	RKGRVVIDSDTEDSGSDENLDQELLSLAKRK	1768800	551010	4631
Q92541	55	RNA polymerase-associated protein RTF1 homolog	RTF1	0.982531	4.18E-05	99.097	T	TMVKKRKGRVVIDSDTEDSGSDENLDQELLS	1768800	551010	7932
Q9UKS7	17	Zinc finger protein Helios	IKZF2	0.998291	0.00651157	99.092	S	ETEAIDGYITCDNELSPEREHSNMAIDLTSS	0	4158800	6452
Q9UKS7	3	Zinc finger protein Helios	IKZF2	0.999998	0.00651157	99.092	T	______________METEAIDGYITCDNELSP	6516700	14213000	8230
Q7Z4S6	853	Kinesin-like protein KIF21A	KIF21A	0.41329	6.57E-06	99.064	S	RPMSDKVAGKVTRKLSSSDAPAQDTGSSAAA	0	0	3822
O75376	1980	Nuclear receptor corepressor 1	NCOR1	0.643646	9.28E-06	99.059	S	RYETPSDAIEVISPASSPAPPQEKLQTYQPE	4843500	2524100	607
Q66PJ3	332	ADP-ribosylation factor-like protein 6-interacting protein 4	ARL6IP4	1	0.015418	99.055	S	QVEALPGPSLDQWHRSAGEEEDGPVLTDEQK	0	230840	3412
P49792	2900	E3 SUMO-protein ligase RanBP2	RANBP2	1	1.38E-08	99.048	S	VGTQSAGKVGEDEDGSDEEVVHNEDIHFEPI	4659900	2579100	1718
Q9HAU0	939	Pleckstrin homology domain-containing family A member 5	PLEKHA5	0.610379	0.000155805	99.044	S	LNVIGASDQSPLQSPSNLRDNPFRTTQTRRR	0	0	5819
A7MCY6	335	TANK-binding kinase 1-binding protein 1	TBKBP1	0.999377	0.0098674	99.013	S	RTLLQEQARSGGQRHSPLSQRHSPAPQCPSP	979640	0	48
Q9Y5U2	132	Protein TSSC4	TSSC4	1	0.00357517	99.013	S	SDNGGFKRPLAPSGRSPVEGLGRAHRSPASP	885330	805700	6969
Q9NR33	11	DNA polymerase epsilon subunit 4	POLE4	0.999039	0.00075844	99.013	T	_____MAAAAAAGSGTPREEEGPAGEAAASQ	988620	1004600	8151
P54259	101	Atrophin-1	ATN1	0.936541	0.000181127	99.011	S	APKKTKTEQELPRPQSPSDLDSLDGRSLNDD	1107100	764110	1847
P54259	107	Atrophin-1	ATN1	0.99944	0.000181127	99.011	S	TEQELPRPQSPSDLDSLDGRSLNDDGSSDPR	1107100	764110	1848
P20265	432	POU domain, class 3, transcription factor 2	POU3F2	0.999985	0.0121954	99.011	T	GTLPGAEDVYGGSRDTPPHHGVQTPVQ____	916470	337650	7308
Q99567	50	Nuclear pore complex protein Nup88	NUP88	0.873105	2.71E-06	98.973	S	SPTEAEKPASSSLPSSPPPQLLTRNVVFGLG	882870	0	5211
Q5T481	1048	RNA-binding protein 20	RBM20	0.743691	5.43E-06	98.968	S	ESSDVHPAPTVQQMSSPKPAEERARQPSPFV	2072000	1868900	3282
P30622	146	CAP-Gly domain-containing linker protein 1	CLIP1	0.708308	1.77E-08	98.967	T	EDEANGLQTTPASRATSPLCTSTASMVSSSP	0	0	7348
P19622	150	Homeobox protein engrailed-2	EN2	0.971463	5.12E-07	98.924	S	CAPGAGGPLPAAGSDSPGDGEGGSKTLSLHG	1190700	788320	1170
O95671	21	N-acetylserotonin O-methyltransferase-like protein	ASMTL	0.889814	0.0023267	98.898	S	VIGKLLHKRVVLASASPRRQEILSNAGLRFE	0	230820	791
Q07864	1940	DNA polymerase epsilon catalytic subunit A	POLE	1	0.00165163	98.898	S	IKGKVSSRIHCGLQDSQKAGGAEDEQENEDD	495350	399050	2237
Q86Y91	413	Kinesin-like protein KIF18B	KIF18B	1	0.000146444	98.882	S	VYEGGGQPPPQDLPGSPKSGPPPEHLPSSPL	1453400	1353800	4021
P46060	427	Ran GTPase-activating protein 1	RANGAP1	0.71888	1.51E-08	98.839	S	RKILDPNTGEPAPVLSSPPPADVSTFLAFPS	0	0	1541
P46821	1258	Microtubule-associated protein 1B;MAP1B heavy chain;MAP1 lig	MAP1B	0.664684	0.0154606	98.676	S	ISAVSSEKVSPSKSPSLSPSPPSPLEKTPLG	1060000	0	1598
Q9NWQ8	170	Phosphoprotein associated with glycosphingolipid-enriched mi	PAG1	0.378615	0.00153541	98.676	S	QGLGMEGPYEVLKDSSSQENMVEDCLYETVK	0	0	6063
P24928	1912	DNA-directed RNA polymerase II subunit RPB1	POLR2A	0.355822	0.0176891	98.676	T	PTSPVYTPTSPKYSPTSPTYSPTSPKYSPTS	0	0	7319
P49585	343	Choline-phosphate cytidylyltransferase A	PCYT1A	0.58338	0.0016326	98.654	S	RSPSPSFRWPFSGKTSPPCSPANLSRHKAAA	6009800	5654000	1669
P49585	347	Choline-phosphate cytidylyltransferase A	PCYT1A	0.999967	0.0016326	98.654	S	PSFRWPFSGKTSPPCSPANLSRHKAAAYDIS	6009800	5654000	1670
Q96JM3	507	Chromosome alignment-maintaining phosphoprotein 1	CHAMP1	0.992697	0.00806486	98.654	S	VFPETRKPGPSGPSESPKAASDIWKPVLSID	835320	733120	4973
Q9UBW7	838	Zinc finger MYM-type protein 2	ZMYM2	0.942554	0.00434313	98.654	S	CQTSRTKMTGSAPPPSPTPNKEMKNKAVLCK	0	547240	6275
Q5T200	207	Zinc finger CCCH domain-containing protein 13	ZC3H13	0.999651	0.00643394	98.592	S	IIKKEVSPEVVRSKLSPSPSLRKSSKSPKRK	45301000	0	3263
Q5T200	209	Zinc finger CCCH domain-containing protein 13	ZC3H13	0.972621	0.00643394	98.592	S	KKEVSPEVVRSKLSPSPSLRKSSKSPKRKSS	46773000	0	3264
Q9UI10	130	Translation initiation factor eIF-2B subunit delta	EIF2B4	0.989019	0.00435763	98.582	S	KQARKGEQGGPPPKASPSTAGETPSGVKRLP	0	801610	6372
Q92621	1939	Nuclear pore complex protein Nup205	NUP205	0.999896	0.0148689	98.573	S	LFASRTLFKSRRLQDSFASETNLDFRSGLAI	286520	273950	4679
Q92621	1942	Nuclear pore complex protein Nup205	NUP205	0.804172	0.0148689	98.573	S	SRTLFKSRRLQDSFASETNLDFRSGLAIVSQ	286520	273950	4680
Q9BQ52	212	Zinc phosphodiesterase ELAC protein 2	ELAC2	0.745864	0.000149416	98.562	S	WQSPERPLSRLSPERSSDSESNENEPHLPHG	3405500	1388400	5297
Q9BQ52	213	Zinc phosphodiesterase ELAC protein 2	ELAC2	0.765872	0.000149416	98.562	S	QSPERPLSRLSPERSSDSESNENEPHLPHGV	0	0	5298
Q9UGV2	355	Protein NDRG3	NDRG3	0.506676	6.95E-05	98.562	T	ESPFSRSVTSNQSDGTQESCESPDVLDRHQT	3239000	0	8210
O14497	772	AT-rich interactive domain-containing protein 1A	ARID1A	0.957471	0.000428473	98.552	S	PQMPQYSSPQPGSALSPRQPSGGQIHTGMGS	4400200	0	145
Q9UMN6	568	Histone-lysine N-methyltransferase 2B	KMT2B	0.448957	1.96E-06	98.538	T	KPPKVEVSPVLRPPITTSPPVPQEPAPVPSP	0	0	8241
P13807	727	Glycogen [starch] synthase, muscle	GYS1	0.939532	1.19E-06	98.533	S	DTATSSSLSTPSEPLSPTSSLGEERN_____	0	0	1051
P13807	730	Glycogen [starch] synthase, muscle	GYS1	0.35348	1.19E-06	98.533	S	TSSSLSTPSEPLSPTSSLGEERN________	0	0	1052
P13807	731	Glycogen [starch] synthase, muscle	GYS1	0.768583	1.19E-06	98.533	S	SSSLSTPSEPLSPTSSLGEERN_________	1989200	3033300	1053
P13807	729	Glycogen [starch] synthase, muscle	GYS1	0.540473	1.19E-06	98.533	T	ATSSSLSTPSEPLSPTSSLGEERN_______	1989200	0	7285
Q86VR2	320	Protein FAM134C	FAM134C	0.974524	6.68E-06	98.443	S	GQTPLTEGSEDLDGHSDPEESFARDLPDFPS	7502500	4288200	3965
P49736	4	DNA replication licensing factor MCM2	MCM2	0.961793	0.0136785	98.407	S	____________MAESSESFTMASSPAQRRR	278270	718960	1673
Q13009	1466	T-lymphoma invasion and metastasis-inducing protein 1	TIAM1	0.994526	0.0147813	98.407	S	ARNRFTIDSDAVSASSPEKESQQPPGGGDTD	1658800	896990	2387
P42224	727	Signal transducer and activator of transcription 1-alpha/bet	STAT1	0.999853	0.000151015	98.391	S	VHPSRLQTTDNLLPMSPEEFDEVSRIVGSVE	821540	1829600	1501
Q15776	12	Zinc finger protein with KRAB and SCAN domains 8	ZKSCAN8	0.984566	0.000234694	98.391	S	____MAEESRKPSAPSPPDQTPEEDLVIVKV	1504700	1241100	2986
Q7Z7L9	172	Zinc finger and SCAN domain-containing protein 2	ZSCAN2	0.84235	4.48E-05	98.391	S	RKIFSEMPEGESAQHSDGESDFERDAGIQRL	2163200	953040	3892
P42166	166	Lamina-associated polypeptide 2, isoform alpha;Thymopoietin;	TMPO	0.476948	0.00386554	98.368	S	EQGTESRSSTPLPTISSSAENTRQNGSNDSD	0	0	1495
P42166	167	Lamina-associated polypeptide 2, isoform alpha;Thymopoietin;	TMPO	0.476948	0.00386554	98.368	S	QGTESRSSTPLPTISSSAENTRQNGSNDSDR	0	0	1496
O75061	570	Putative tyrosine-protein phosphatase auxilin	DNAJC6	0.790405	0.00199729	98.337	S	STQSTPRRSATSTSASPTLRVGEGATFDPFG	644750	0	554
Q4KMP7	22	TBC1 domain family member 10B	TBC1D10B	1	0.00199729	98.337	S	PLVAPPRRHGAPAAPSPPPRGSRAGPVVVVA	936190	593880	3098
O75398	176	Deformed epidermal autoregulatory factor 1 homolog	DEAF1	0.979674	9.12E-06	98.299	S	TGLKGPAAPLTPGPQSPPTPLAPGQEKGGTK	2136200	1277800	622
Q96T23	628	Remodeling and spacing factor 1	RSF1	0.887081	3.10E-06	98.299	T	STLESEKPGSPEAAETSPPSNIIDHCEKLAS	5869400	185860	8012
Q9BWU0	143	Kanadaptin	SLC4A1AP	0.66803	0.00012398	98.292	S	FRSLQEEQSRPPTAVSSPGGPARAPPYQEPP	0	0	5436
Q9BWU0	144	Kanadaptin	SLC4A1AP	0.49962	0.00012398	98.292	S	RSLQEEQSRPPTAVSSPGGPARAPPYQEPPW	0	0	5437
Q9ULJ3	1005	Zinc finger and BTB domain-containing protein 21	ZBTB21	0.495987	4.50E-05	98.292	T	PPPLPKIQPLEPDSPTGLSENPTPATEKLFV	0	0	8238
Q9UN79	334	Transcription factor SOX-13	SOX13	0.597098	4.51E-05	98.267	S	VPRPPSHGGPTRDLQSSPPSLPLGFLGEGDA	561220	414690	6536
Q12888	1673	Tumor suppressor p53-binding protein 1	TP53BP1	0.898552	0.00771921	98.253	S	RASMGVLSGKRKLITSEEERSPAKRGRKSAT	907310	1416600	2345
Q8NFQ8	120	Torsin-1A-interacting protein 2	TOR1AIP2	0.822769	0.00442441	98.253	S	SENLGKEPLDPDPSHSPSDKVGRADAHLGSS	0	581410	4404
P31323	85	cAMP-dependent protein kinase type II-beta regulatory subuni	PRKAR2B	0.750337	1.92E-11	98.225	S	PSKGVNFAEEPMQSDSEDGEEEEAAPADAGA	2503600	0	1354
O00221	183	NF-kappa-B inhibitor epsilon	NFKBIE	0.980289	5.39E-07	98.21	S	LPESTSAPASGPSDGSPQPCTHPPGPVKEPQ	1920200	1168900	107
O60293	15	Zinc finger C3H1 domain-containing protein	ZFC3H1	0.97164	0.00166157	98.182	S	_MATADTPAPASSGLSPKEEGELEDGEISDD	0	0	469
Q8WWM7	684	Ataxin-2-like protein	ATXN2L	0.880974	0.00156564	98.182	S	TKPLLSVNKSTSTPTSPGPRTHSTPSIPVLT	3119400	2808200	4565
Q9P0K7	335	Ankycorbin	RAI14	0.635825	1.84E-08	98.151	S	RENKDRLSDSTTGADSLLDISSEADQQDLLS	592960	349120	6183
Q14141	418	Septin-6	SEPT6	0.499941	0.000153932	98.077	T	AELLQSQGSQAGGSQTLKRDKEKKNNPWLCT	0	1320700	7578
P38398	1217	Breast cancer type 1 susceptibility protein	BRCA1	0.99945	4.88E-06	98.073	S	YRRGAKKLESSEENLSSEDEELPCFQHLLFG	4014400	2801600	1452
P38398	1218	Breast cancer type 1 susceptibility protein	BRCA1	0.999841	4.88E-06	98.073	S	RRGAKKLESSEENLSSEDEELPCFQHLLFGK	4014400	2801600	1453
P36915	48	Guanine nucleotide-binding protein-like 1	GNL1	0.999975	0.0180613	98.062	T	SSNSRSGSRERREEQTDTSDGESVTHHIRRL	668090	647020	7371
Q9Y2V2	30	Calcium-regulated heat stable protein 1	CARHSP1	1	0.0144037	98.048	S	QASVGLLDTPRSRERSPSPLRGNVVPSPLPT	234940	4651300	6810
Q9Y2V2	32	Calcium-regulated heat stable protein 1	CARHSP1	1	0.0144037	98.048	S	SVGLLDTPRSRERSPSPLRGNVVPSPLPTRR	234940	4651300	6811
Q6NXT1	63	Ankyrin repeat domain-containing protein 54	ANKRD54	1	0.00170888	98.044	S	GGGAGLSGRASGGAQSPLRYLHVLWQQDAEP	0	633580	3494
Q52LA3	52	Protein lin-52 homolog	LIN52	0.464926	0.00240713	98.04	S	PGVAEFAASFKSPITSSPPKWMAEIERDDID	0	0	3121
Q52LA3	53	Protein lin-52 homolog	LIN52	0.464926	0.00240713	98.04	S	GVAEFAASFKSPITSSPPKWMAEIERDDIDM	0	0	3122
Q6KC79	305	Nipped-B-like protein	NIPBL	0.592477	0.000562263	98.035	S	GSRPPLILQSQSLPCSSPRDVPPDILLDSPE	2898500	0	3486
Q5JSH3	7	WD repeat-containing protein 44	WDR44	0.906353	4.77E-07	98.002	T	_________MASESDTEEFYDAPEDVHLGGG	3842400	2411000	7701
Q8WU79	219	Stromal membrane-associated protein 2	SMAP2	0.8916	0.00319625	98	S	SNTLEKDLDLLASVPSPSSSGSRKVVGSMPT	0	0	4501
Q9NSK0	611	Kinesin light chain 4	KLC4	0.685189	0.0031505	97.965	S	QPSAAPLQVSRGLSASTMDLSSSS_______	1116200	0	5999
O95402	7	Mediator of RNA polymerase II transcription subunit 26	MED26	0.999999	0.000673598	97.957	S	_________MTAAPASPQQIRDRLLQAIDPQ	0	597160	765
P11388	1392	DNA topoisomerase 2-alpha	TOP2A	0.543739	4.72E-06	97.904	S	SDLEADDVKGSVPLSSSPPATHFPDETEITN	661160	1066600	1017
P11388	1393	DNA topoisomerase 2-alpha	TOP2A	0.889939	4.72E-06	97.904	S	DLEADDVKGSVPLSSSPPATHFPDETEITNP	16317000	1066600	1018
Q14676	1033	Mediator of DNA damage checkpoint protein 1	MDC1	0.999979	2.45E-08	97.897	S	SRIRAAEKVSRGDQESPDACLPPTVPEAPAP	677120	1192600	2766
Q8N108	130	Mesoderm induction early response protein 1	MIER1	0.485112	1.93E-08	97.839	S	EENIKDSSGQEDETQSSNDDPSQSVASQDAQ	0	0	4201
Q12872	283	Splicing factor, suppressor of white-apricot homolog	SFSWAP	0.979994	1.18E-06	97.825	S	LAENKSDEKKKSGVSSDNEDDDDEEDGNYLH	0	4615900	2335
Q8WXE1	224	ATR-interacting protein	ATRIP	0.991404	0.00205244	97.813	S	SERANKLAAPSVSHVSPRKNPSVVIKPEACS	1727000	646010	4589
Q14676	1321	Mediator of DNA damage checkpoint protein 1	MDC1	0.794964	7.26E-06	97.813	T	TSRTTRSRTNMSSVKTPETVVPTAPELQIST	0	0	7611
Q9Y2Y0	157	ADP-ribosylation factor-like protein 2-binding protein	ARL2BP	0.999997	0.00205415	97.797	S	GLVVTSLCKSSSLPASQNNLRH_________	543510	545070	6839
Q9UKV3	1004	Apoptotic chromatin condensation inducer in the nucleus	ACIN1	1	0.00243164	97.779	S	VSITIDDPVRTAQVPSPPRGKISNIVHISNL	10441000	7996100	6458
Q9NQS7	197	Inner centromere protein	INCENP	0.834987	1.55E-06	97.776	S	HVTQLMSTEPLPRTLSPTPASATAPTSQGIP	3190100	2655900	5925
Q9NQS7	214	Inner centromere protein	INCENP	0.70358	1.55E-06	97.776	S	TPASATAPTSQGIPTSDEESTPKKSKARILE	0	1962200	5926
Q9NQS7	195	Inner centromere protein	INCENP	0.496267	1.55E-06	97.776	T	EQHVTQLMSTEPLPRTLSPTPASATAPTSQG	0	0	8145
Q9NQS7	199	Inner centromere protein	INCENP	0.333124	1.55E-06	97.776	T	TQLMSTEPLPRTLSPTPASATAPTSQGIPTS	0	0	8146
Q9NQS7	213	Inner centromere protein	INCENP	0.61243	1.55E-06	97.776	T	PTPASATAPTSQGIPTSDEESTPKKSKARIL	2486200	3937600	8147
Q13541	35	Eukaryotic translation initiation factor 4E-binding protein	EIF4EBP1	0.563511	2.80E-08	97.771	S	RVVLGDGVQLPPGDYSTTPGGTLFSTTPGGT	0	2492900	2572
Q9UN36	353	Protein NDRG2	NDRG2	0.692209	1.02E-05	97.713	S	SVDGNRSRSRTLSQSSESGTLSSGPPGHTME	3255600	0	6530
Q9UN36	348	Protein NDRG2	NDRG2	0.81946	1.02E-05	97.713	T	LTSAASVDGNRSRSRTLSQSSESGTLSSGPP	3255600	0	8243
O60333	1658	Kinesin-like protein KIF1B	KIF1B	0.5	7.04E-06	97.69	S	SNSLDQKTPEANSRASSPCPEFEQFQIVPAV	1755100	648880	480
O60333	1659	Kinesin-like protein KIF1B	KIF1B	0.5	7.04E-06	97.69	S	NSLDQKTPEANSRASSPCPEFEQFQIVPAVE	1755100	648880	481
P46821	1156	Microtubule-associated protein 1B;MAP1B heavy chain;MAP1 lig	MAP1B	0.975386	7.04E-06	97.689	S	DVMSDETNNEETESPSQEFVNITKYESSLYS	8330600	0	1579
Q9NZJ0	443	Denticleless protein homolog	DTL	0.289212	1.13E-08	97.639	S	STPAKAPRAKCNPSNSSPSSAACAPSCAGDL	0	0	6152
Q9Y2I7	307	1-phosphatidylinositol 3-phosphate 5-kinase	PIKFYVE	0.990875	0.00160474	97.631	S	VQEDAGKSPARNRSASITNLSLDRSGSPMVP	1175000	399600	6780
Q14160	1439	Protein scribble homolog	SCRIB	0.884944	8.72E-07	97.623	S	GEEEQEDEQPPWASPSPTSRQSPASPPPLGG	3089800	0	2679
Q9NZN5	341	Rho guanine nucleotide exchange factor 12	ARHGEF12	0.920913	0.00802828	97.621	S	KSETIQDTDTQSLVGSPSTRIAPHIIGAEDD	389870	221280	6164
Q8IX01	275	SURP and G-patch domain-containing protein 2	SUGP2	0.838499	1.11E-05	97.57	T	RITPKTQGTNQIQKNTPSPDVTLGTNPGTED	0	2392800	7849
Q9NPQ8	441	Synembryn-A	RIC8A	0.682066	1.22E-05	97.57	T	MAGGRPEGQYSEDEDTDTDEYKEAKASINPV	0	0	8140
Q9UEY8	11	Gamma-adducin	ADD3	0.497336	0.000134867	97.502	T	_____MSSDASQGVITTPPPPSMPHKERYFD	0	0	8205
Q9NQS7	218	Inner centromere protein	INCENP	0.570888	3.05E-06	97.5	S	ATAPTSQGIPTSDEESTPKKSKARILESITV	2123200	1975400	5927
Q9NQS7	219	Inner centromere protein	INCENP	0.5269	3.05E-06	97.5	T	TAPTSQGIPTSDEESTPKKSKARILESITVS	2123200	0	8148
Q9UEG4	18	Zinc finger protein 629	ZNF629	1	6.22E-06	97.49	S	PETALWGPDLQGPEQSPNDAHRGAESENEEE	3939800	2369700	6284
Q9ULJ3	981	Zinc finger and BTB domain-containing protein 21	ZBTB21	0.890843	6.85E-06	97.488	S	ESAHKESEVCPVPTNSPSPPPLPPPPPLPKI	5685000	2213100	6476
Q69YN4	133	Protein virilizer homolog	KIAA1429	0.583274	0.000269902	97.482	S	NCLTLAIYGSVDRVISHDRDSPPPPPPPPPP	0	0	3437
Q9P2N6	538	KAT8 regulatory NSL complex subunit 3	KANSL3	0.320077	1.40E-05	97.457	T	SPSGSEDLSSVSSSPTSSPKTKVTTVTSAQK	0	0	8192
Q8N556	668	Actin filament-associated protein 1	AFAP1	0.993403	0.00209004	97.456	S	LGLAIEPKSGTSSPQSPVFRHRTLENSPISS	4648200	1657400	4278
Q9H3N1	270	Thioredoxin-related transmembrane protein 1	TMX1	0.999993	0.00174856	97.452	S	EGTNKDFPQNAIRQRSLGPSLATDKS_____	3748800	1724000	5637
Q96E39	88	RNA binding motif protein, X-linked-like-1	RBMXL1	0.998792	0.00729045	97.45	S	SLDGKAIKVEQATKPSFERGRHGPPPPPRSR	875130	420400	4880
P46821	1949	Microtubule-associated protein 1B;MAP1B heavy chain;MAP1 lig	MAP1B	1	0.0187037	97.45	T	EDGDYSYEIIEKTTRTPEEGGYSYDISEKTT	825980	459080	7394
Q9UHB6	609	LIM domain and actin-binding protein 1	LIMA1	1	0.0103646	97.431	S	AASFQSTSVKSPKTVSPPIRKGWSMSEQSEE	940350	517390	6333
P11474	26	Steroid hormone receptor ERR1	ESRRA	0.316824	0.00420524	97.352	S	LYIKAEPASPDSPKGSSETETEPPVALAPGP	0	0	1026
P11474	27	Steroid hormone receptor ERR1	ESRRA	0.316824	0.00420524	97.352	S	YIKAEPASPDSPKGSSETETEPPVALAPGPA	0	0	1027
Q86X27	296	Ras-specific guanine nucleotide-releasing factor RalGPS2	RALGPS2	0.642174	0.000128246	97.352	S	SLKIEPGTSTPRSAASREDLVGPEVGASPQS	3876700	2288500	4004
Q9BYV8	121	Centrosomal protein of 41 kDa	CEP41	0.98192	7.20E-06	97.352	S	TARTNGKGNPGEQSPSPEQFINNAGAGDSSR	0	1520100	5472
Q13428	1001	Treacle protein	TCOF1	0.374655	9.75E-06	97.32	S	KARASESTARSSSSESEDEDVIPATQCLTPG	0	0	2525
Q13428	974	Treacle protein	TCOF1	0.818029	0.000458062	97.32	T	LIFVDPNRSPAGPAATPAQAQAASTPRKARA	0	1891100	7537
O60502	511	Protein O-GlcNAcase	MGEA5	0.974327	4.78E-06	97.3	S	LKPMDTDKESIAESKSPEMSMQEDCISDIAP	5030000	4964100	503
P14859	448	POU domain, class 2, transcription factor 1	POU2F1	0.96337	0.00466144	97.291	S	KEKRINPPSSGGTSSSPIKAIFPSPTSLVAT	10878000	5927700	1062
Q9H4A3	170	Serine/threonine-protein kinase WNK1	WNK1	0.508604	0.0147099	97.223	S	TVPSSTSKDRPVSQPSLVGSKEEPPPARSGS	0	0	5661
Q96PY6	1052	Serine/threonine-protein kinase Nek1	NEK1	0.997506	0.00248939	97.163	S	TGLFDANNPKMLRTCSLPDLSKLFRTLMDVP	1360300	1106800	5064
Q9BW71	102	HIRA-interacting protein 3	HIRIP3	0.567565	5.21E-05	97.08	S	SDPERKRFRFNSESESGSEASSPDYFGPPAK	2816900	0	5414
Q9C0H2	522	Protein tweety homolog 3	TTYH3	0.970429	0.0212142	97.073	S	AKYLATSQPRPDSSGSH______________	501140	177700	5547
Q7Z6Z7	2377	E3 ubiquitin-protein ligase HUWE1	HUWE1	0.830195	5.83E-07	97.059	S	SGNSTIIVSRSGEDESQEDVLMDEAPSNLSQ	1096900	0	3883
Q86UE4	568	Protein LYRIC	MTDH	0.997485	0.00250955	96.948	S	NSVPPSQTKSETSWESPKQIKKKKKARRET_	615930	412700	3924
O14683	16	Tumor protein p53-inducible protein 11	TP53I11	0.703968	0.00149821	96.82	T	MAAKQPPPLMKKHSQTDLVSRLKTRKILGVG	0	555460	7129

Q12778	329	Forkhead box protein O1	FOXO1	0.999923	1.26E-06	96.783	S	RPRTSSNASTISGRLSPIMTEQDDLGEGDVH	1254700	940930	2296
Q9UHF7	178	Zinc finger transcription factor Trps1	TRPS1	0.974248	2.53E-06	96.777	S	ETGQAQSGQANCQGLSPVSVASKNPQVPSDG	1709300	972140	6351
Q14004	1147	Cyclin-dependent kinase 13	CDK13	0.806456	1.59E-06	96.777	T	PAGILATGEKQTDPSTPQQESSKPLGGIQPS	3428200	0	7575
Q9NSV4	1093	Protein diaphanous homolog 3	DIAPH3	0.999922	0.00252756	96.756	S	RRKRTPMPKDVRQSLSPMSQRPVLKVCNHEN	1776600	843680	6000
Q13428	906	Treacle protein	TCOF1	1	0.00207955	96.734	S	GKTHQIRAALAPAKESPRKGAAPTPPGKTGP	2201000	1400700	2501
P22670	159	MHC class II regulatory factor RFX1	RFX1	0.841033	1.01E-05	96.718	S	RLLVQTSVQAKPGHVSPLQLTNIQVPQQALP	1134800	1084000	1216
P55884	154	Eukaryotic translation initiation factor 3 subunit B	EIF3B	0.937871	1.20E-08	96.707	S	EPRALENGDADEPSFSDPEDFVDDVSEEELL	24081000	33010000	1886
Q00013	243	55 kDa erythrocyte membrane protein	MPP1	0.950527	0.00588251	96.707	S	VASMAQSAPSEAPSCSPFGKKKKYKDKYLAK	633760	507620	2020
Q9Y3L3;Q(	544;552	SH3 domain-binding protein 1	SH3BP1	0.999993	0.0150646	96.707	S	ATKERTESEVPPRPASPKVTRSPPETAAPVE	880830	0	6861
P07741	30	Adenine phosphoribosyltransferase	APRT	1	0.0106041	96.668	S	SFPDFPTPGVVFRDISPVLKDPASFRAAIGL	0	454870	934
P58340	34	Myeloid leukemia factor 1	MLF1	0.991157	0.00398462	96.665	S	ILAHRENMRQMIRSFSEPFGRDLLSISDGRG	953180	449530	1900
Q15742	162	NGFI-A-binding protein 2	NAB2	1	0.000621817	96.665	S	SPGEKAGSARSFSPKSPLELGEKLSPLPGGP	595140	877500	2981
Q6PJI9	603	WD repeat-containing protein 59	WDR59	0.949407	0.00398462	96.665	S	KIRTEAPGNLRLYSGSPTRSEKEQVSISSFY	1058200	0	3551
Q96JM3	184	Chromosome alignment-maintaining phosphoprotein 1	CHAMP1	0.970266	0.000967197	96.665	S	TPLPSPEPSKPASVSSPEPPKSVPVCESQKL	0	192430	4989
Q9BUR4	114	Telomerase Cajal body protein 1	WRAP53	0.991562	3.71E-09	96.634	S	SENTSLPAEEANGSLSEEEANGPELGSGKAM	3461700	1298200	5389
Q9NVU0	503	DNA-directed RNA polymerase III subunit RPC5	POLR3E	1	1.53E-08	96.634	S	VPAVPPGVRIKEEPVSEEGEEDEEQEAEEEP	1279100	1375600	6049
Q9H6F5	21	Coiled-coil domain-containing protein 86	CCDC86	0.975027	0.0132087	96.561	S	RRSRRLGGLRPESPESLTSVSRTRRALVEFE	0	756340	5729
Q08J23	473	tRNA (cytosine(34)-C(5))-methyltransferase	NSUN2	0.981173	1.29E-06	96.505	S	ADLTEGKPTDPSKLESPSFTGTGDTEIAHAT	1711400	1628200	2269
Q8NDI1	335	EH domain-binding protein 1	EHBP1	0.906561	7.71E-07	96.504	S	LNPFDEPEAFVTIKDSPPQSTKRKNIRPVDM	1513500	1671300	4355
Q01581	495	Hydroxymethylglutaryl-CoA synthase, cytoplasmic	HMGCS1	0.980224	2.60E-07	96.466	S	GVGLVHSNIATEHIPSPAKKVPRLPATAAEP	0	0	2072
Q9NR55	31	Basic leucine zipper transcriptional factor ATF-like 3	BATF3	1	1.03E-05	96.455	S	VAAPGNQPQPQPQQQSPEDDDRKVRRREKNR	1482000	830000	5950
Q9Y6W5	308	Wiskott-Aldrich syndrome protein family member 2	WASF2	0.999999	5.03E-05	96.447	S	SVVSPSHPPPAPPLGSPPGPKPGFAPPPAPP	2278600	1744300	7023
Q9Y520	2673	Protein PRRC2C	PRRC2C	1	0.00186565	96.414	T	MELKAFGSGIDIKPGTPPIAGRSTTPTSSPF	950720	1654500	8306
Q13905	222	Rap guanine nucleotide exchange factor 1	RAPGEF1	0.896702	7.95E-06	96.382	S	IEKQGRPSPTSPVKPSSPASKPDGPAELPLT	0	2187400	2632
Q13905	216	Rap guanine nucleotide exchange factor 1	RAPGEF1	0.628975	7.95E-06	96.382	T	ELVRLTIEKQGRPSPTSPVKPSSPASKPDGP	0	2187400	7573
P46821	1782	Microtubule-associated protein 1B;MAP1B heavy chain;MAP1 li	MAP1B	0.999996	0.000334591	96.379	S	SEKVQSLEGEKLSPKSDISPLTPRESSPLYS	3078900	1742500	1591
P46821	1788	Microtubule-associated protein 1B;MAP1B heavy chain;MAP1 li	MAP1B	1	0.000334591	96.379	T	LEGEKLSPKSDISPLTPRESSPLYSPTFSDS	3078900	1742500	7391
Q13243	231	Serine/arginine-rich splicing factor 5	SRSF5	0.96998	0.00700668	96.371	S	RSRSRSRSRSKSRSVSRSPVPEKSQKRGSSS	0	1428700	2439
Q13243	233	Serine/arginine-rich splicing factor 5	SRSF5	0.999944	0.00700668	96.371	S	RSRSRSRSKSRSVSRSPVPEKSQKRGSSSRS	0	1428700	2440
Q9UQ35	1081	Serine/arginine repetitive matrix protein 2	SRRM2	0.332826	0.00220739	96.342	S	DHRSDTSSPEVRQSHSESPSLQSKSQTSPKG	0	0	6670
Q9UQ35	1083	Serine/arginine repetitive matrix protein 2	SRRM2	0.996748	0.00214626	96.342	S	RSDTSSPEVRQSHSESPSLQSKSQTSPKGGR	1533200	1426700	6671
Q9UQ35	1085	Serine/arginine repetitive matrix protein 2	SRRM2	0.332826	0.00220739	96.342	S	DTSSPEVRQSHSESPSLQSKSQTSPKGGRSR	0	0	6672
Q9Y2X7	596	ARF GTPase-activating protein GIT1	GIT1	0.709148	8.26E-06	96.341	S	SRHTSKLSRHGSGADSDYENTQSGDPLLGLE	1905800	751490	6837
Q9Y2X7	598	ARF GTPase-activating protein GIT1	GIT1	0.635991	8.26E-06	96.341	Y	HTSKLSRHGSGADSDYENTQSGDPLLGLEGK	1905800	751490	8436
P05023	57	Sodium/potassium-transporting ATPase subunit alpha-1	ATP1A1	0.986688	0.0216461	96.331	T	DDHKLSLDELHRKYGTDLSRGLTSARAAEIL	0	563660	7261
Q7Z6E9	246	E3 ubiquitin-protein ligase RBBP6	RBBP6	0.646288	3.55E-07	96.317	S	GKKEKPPFLPEEPSSSSEEDDPIPDELLCLI	944340	225110	3855
Q7Z6E9	247	E3 ubiquitin-protein ligase RBBP6	RBBP6	0.724407	3.55E-07	96.317	S	KKEKPPFLPEEPSSSSEEDDPIPDELLCLIC	2213000	602590	3856
Q9H0D6	471	5'-3' exoribonuclease 2	XRN2	0.617234	1.02E-06	96.317	S	NPRQAAYEMRMQNNSSPSISPNTSFTSDGSP	5822100	0	5570
Q9H0D6	475	5'-3' exoribonuclease 2	XRN2	0.916786	1.02E-06	96.317	S	AAYEMRMQNNSSPSISPNTSFTSDGSPSPLG	5822100	917750	5572
Q9NPI6	353	mRNA-decapping enzyme 1A	DCP1A	1	7.70E-06	96.312	S	NSTMMQAVKTTPRQRSPLLNQPVPELSHASL	1306400	688830	5896
Q96D71	392	RalBP1-associated Eps domain-containing protein 1	REPS1	0.61607	2.85E-07	96.287	S	SADVGDQPGEVGYSGSPAEAPPSKSPSMPSL	0	2458300	4844
Q14C86	1019	GTPase-activating protein and VPS9 domain-containing protei	GAPVD1	0.998574	0.00230346	96.23	S	DDLGPDRFSTLTDDPSPRLSAQAQVAEDILD	0	0	2845
Q7Z417	572	Nuclear fragile X mental retardation-interacting protein 2	NUFIP2	0.5	0.00694759	96.23	S	EHPVFPKAYELEKRTSPQVLGSILKSGTTSE	1629700	904820	3798
Q8IWQ3	418	Serine/threonine-protein kinase BRSK2	BRSK2	0.763846	0.00132635	96.23	S	MAQHGQRSRSISGASSGLSTSPLSSPRVTPH	575310	781190	4065
Q8IWQ3	421	Serine/threonine-protein kinase BRSK2	BRSK2	0.411242	0.00132635	96.23	S	HGQRSRSISGASSGLSTSPLSSPRVTPHPSP	0	0	4066
Q9UGU0	1335	Transcription factor 20	TCF20	0.762229	0.00561577	96.23	S	LPSPDSRNCPAVTLTSPAKTKILPPRKGRGL	0	0	6317
Q7Z417	571	Nuclear fragile X mental retardation-interacting protein 2	NUFIP2	0.630659	0.00694759	96.23	T	TEHPVFPKAYELEKRTSPQVLGSILKSGTTS	1629700	904820	7805
Q8IWQ3	422	Serine/threonine-protein kinase BRSK2	BRSK2	0.411242	0.00132635	96.23	T	GQRSRSISGASSGLSTSPLSSPRVTPHPSPR	0	0	7844
Q9UGU0	1334	Transcription factor 20	TCF20	0.492826	0.00561577	96.23	T	DLPSPDSRNCPAVTLTSPAKTKILPPRKGRG	0	0	8209
Q13112	458	Chromatin assembly factor 1 subunit B	CHAF1B	1	0.00183045	96.229	S	PTVIRDPPSITPAVKSPLPGPSEEKTLQPSS	636990	812430	2408
Q9BX63	990	Fanconi anemia group J protein	BRIP1	0.996766	0.00406739	96.19	S	EEAGKAEKIVISRSTSPTFNKQTKRVSWSSF	704060	452340	5444
Q13263	14	Transcription intermediary factor 1-beta	TRIM28	0.910341	2.50E-07	96.181	S	__MAASAAAASAAAASAASGSPGPGEGSAGG	1323700	1224200	2446
Q96F45	228	Zinc finger protein 503	ZNF503	0.421209	1.90E-06	96.181	S	RVPSATCQPFTPRTGSPSSSASACSPGGMLS	0	0	4898
O60551	38	Glycylpeptide N-tetradecanoyltransferase 2	NMT2	1	0.00183435	96.171	S	GIDGDNEEETEHAKGSPGGYLGAKKKKKKQK	2011000	1058300	509
Q96SN8	613	CDK5 regulatory subunit-associated protein 2	CDK5RAP2	1	0.00183435	96.171	S	VLSYQNLRKTLEEQISEIRRREEESFSLYSD	200270	239710	5124
Q641Q2;Q(	352;352	WASH complex subunit FAM21A;WASH complex subunit FAM21C	FAM21A;FAM	0.999485	0.00615769	96.143	S	EDNLFAPPKLTDEDFSPFGSGGGLFSGGKGL	0	490590	3390
Q9Y6W5	296	Wiskott-Aldrich syndrome protein family member 2	WASF2	0.94577	5.49E-05	96.143	S	QRGSGLAGPKRSSVVSPSHPPPAPPLGSPPG	1166100	889230	7022
O60784	359	Target of Myb protein 1	TOM1	0.996253	0.00232163	96.135	S	LGSSSVRAGLQSLEASGRLEDEFDMFALTRG	0	0	519
P54274	11	Telomeric repeat-binding factor 1	TERF1	0.999995	0.000949395	96.135	S	_____MAEDVSSAAPSPRGCADGRDADPTEE	0	1246100	1849
Q7L4I2	17	Arginine/serine-rich coiled-coil protein 2	RSRC2	0.97683	0.010047	96.135	S	AASDTERDGLAPEKTSPDRDKKKEQSEVSVS	768260	648810	3728
Q7Z6Z7	1368	E3 ubiquitin-protein ligase HUWE1	HUWE1	0.99987	0.00222916	96.135	S	LTHPPPIMGGVVRDLSMSEEDQMMRAIAMSL	617880	909130	3872
Q9H3P2	225	Negative elongation factor A	NELFA	0.982608	0.00485416	96.135	S	TTTPLKGIPKQAPFRSPTAPSVFSPTGNRTP	0	726200	5639
Q6AI08	336	HEAT repeat-containing protein 6	HEATR6	0.999998	6.43E-06	96.128	S	VKPKKIQQGEEEEKESSGEIEAAPVTGTGRV	4736400	4311200	3441
Q6AI08	337	HEAT repeat-containing protein 6	HEATR6	0.999995	6.43E-06	96.128	S	KPKKIQQGEEEEKESSGEIEAAPVTGTGRVN	4736400	4311200	3442
Q9NQG5	166	Regulation of nuclear pre-mRNA domain-containing protein 1B	RPRD1B	0.818156	8.06E-09	96.123	S	QQIQEEEDDDYPGSYSPQDPSAGPLLTEELI	12779000	0	5913
Q9Y6Q6	503	Tumor necrosis factor receptor superfamily member 11A	TNFRSF11A	0.5	0.0193807	96.113	S	EARDQPEDGADGRLPSSARAGAGSGSSPGGQ	1343900	649690	7019
Q9Y6Q6	504	Tumor necrosis factor receptor superfamily member 11A	TNFRSF11A	0.5	0.0193807	96.113	S	ARDQPEDGADGRLPSSARAGAGSGSSPGGQS	1343900	649690	7020
Q15678	579	Tyrosine-protein phosphatase non-receptor type 14	PTPN14	0.446967	0.00416633	96.096	T	FRPPPPYPRPRPATSTPDLASHRHKYVSGSS	0	0	7648
P49116	19	Nuclear receptor subfamily 2 group C member 2	NR2C2	0.999375	0.0159847	96.067	S	PSPRIQIISTDSAVASPQRIQIVTDQQTGQK	482720	246950	1650
Q99755;A	458;405	Phosphatidylinositol 4-phosphate 5-kinase type-1 alpha;Puta	PIP5K1A;PII	0.986051	0.0116827	96.067	S	RFMCNTVFKKIPLKPSPSKKFRSGSSFSRRA	859490	471290	5275
Q8WUM9	335	Sodium-dependent phosphate transporter 1	SLC20A1	1	0.0108035	96.034	S	KLGDLEEAPERERLPSVDLKEETSIDSTVNG	745610	578150	4522
Q9UHB6	362	LIM domain and actin-binding protein 1	LIMA1	0.999997	0.0108035	96.034	S	QVKSEVQQPVHPKPLSPDSRASSLSESSPPK	0	282310	6332
Q9UPU5	1143	Ubiquitin carboxyl-terminal hydrolase 24	USP24	0.777233	0.0108035	96.034	S	KTLLSESSSQSSKSPSLSSKQQHQPSASSIL	0	0	6587
Q9HCD6	1442	Protein TANC2	TANC2	0.999958	0.00118397	96.016	S	LQTEARPSQGLPVIQSPPSSPPHRDSAYISS	3894200	1800700	5850
Q9HCD6	1445	Protein TANC2	TANC2	0.645619	0.00118397	96.016	S	EARPSQGLPVIQSPPSSPPHRDSAYISSSPL	0	1800700	5851
Q14160	1581	Protein scribble homolog	SCRIB	0.494811	7.84E-06	96.012	S	SPDFAEELRSLEPSPSPGPQEEDGEVALVLL	0	0	2689
Q9UQ35	144	Serine/arginine repetitive matrix protein 2	SRRM2	0.499224	0.00893108	96.012	S	EKKNERLRAAFGISDSYVDGSSFDPQRRARE	0	0	6595
Q9NRY4	980	Rho GTPase-activating protein 35	ARHGAP35	0.87751	5.20E-06	95.996	S	EEVFNSPRAGSPLCNSNLQDSEEDIEPSYSL	5388000	0	5986
Q8IU60	284	m7GpppN-mRNA hydrolase	DCP2	1	0.00175077	95.988	S	SRTKFRHSQQLFPDGSPGDQWVKHRQPLQQK	0	1549900	4035
Q86TB9	184	Protein PAT1 homolog 1	PATL1	0.999999	0.0018665	95.981	S	SERALPRRSTSPIIGSPPVRAVPIGTPPKQM	9417900	5269800	3897
Q674X7	346	Kazrin	KAZN	0.990077	0.0166928	95.9	S	SSTPSDINSPRHRTHSLCNGDSPGPVQKNLH	368430	199520	3413
Q674X7	352	Kazrin	KAZN	1	0.0166928	95.9	S	INSPRHRTHSLCNGDSPGPVQKNLHNPIVQS	368430	199520	3414
P53355	696	Death-associated protein kinase 1	DAPK1	0.998611	0.00261951	95.775	S	TQNLQPRIKLKLFGHSGSGKTTLVESLKCGL	2640300	2762800	1831
P15056	446	Serine/threonine-protein kinase B-raf	BRAF	0.952472	0.000769857	95.767	S	SSEDRNRMKTLGRRDSSDDWEIPDGQITVGQ	0	0	1063
P15056	447	Serine/threonine-protein kinase B-raf	BRAF	0.83254	0.000769857	95.767	S	SEDRNRMKTLGRRDSSDDWEIPDGQITVGQR	1618400	716320	1064
P35612	693	Beta-adducin	ADD2	0.893606	0.000237253	95.767	S	DTSKDKTESVTSGPMSPEGSPSKSPSKKKKK	993730	1133300	1404
P35612	703	Beta-adducin	ADD2	0.965913	0.000237253	95.767	S	TSGPMSPEGSPSKSPSKKKKKFRTPSFLKKS	4527800	1974700	1408
Q8N3X1	431	Formin-binding protein 4	FNBP4	0.710902	1.23E-06	95.742	S	AELRALEEGDGSVSGSSPRSDISQPASQDGM	3972000	0	4260
Q9NUA8	192	Zinc finger and BTB domain-containing protein 40	ZBTB40	0.811782	4.23E-06	95.742	T	EAAHSVSQEMSVNSPTAQESQRNAETPAETP	1209000	383080	8166
Q12888	830	Tumor suppressor p53-binding protein 1	TP53BP1	0.563027	5.23E-05	95.71	S	DLKSGTAETEPVEQDSSQPSLPLVRADDPLR	0	0	2365
Q5SW79	1132	Centrosomal protein of 170 kDa	CEP170	0.653979	0.000658262	95.662	S	ADADKASVASEVSTTSSTSKPPTGRRNISRI	2883500	0	3213
Q86VM9	613	Zinc finger CCCH domain-containing protein 18	ZC3H18	0.974227	0.000658262	95.662	S	RSRSFSSSPSPSPTPSPHRPSIRTKGEPAPP	2341200	645670	3957
Q5SW79	1131	Centrosomal protein of 170 kDa	CEP170	0.332585	0.000658262	95.662	T	LADADKASVASEVSTTSSTSKPPTGRRNISR	0	0	7710
Q86VM9	611	Zinc finger CCCH domain-containing protein 18	ZC3H18	0.993987	0.000658262	95.662	T	RSRSRSFSSSPSPSPTPSPHRPSIRTKGEPA	3276700	652900	7827
Q7Z422	37	SUZ domain-containing protein 1	SZRD1	0.999961	2.86E-05	95.654	S	RLEKKLKITQKESRKSKSPPKVPIVIQDDSL	1648300	646770	3801
Q13085	80	Acetyl-CoA carboxylase 1;Biotin carboxylase	ACACA	0.989543	0.00187142	95.618	S	SSLQDGLALHIRSSMSGLHLVKQGRDRKKID	0	0	2397
Q9Y6X9	725	MORC family CW-type zinc finger protein 2	MORC2	0.987283	0.0134146	95.57	S	PSPKVIKTPVVKKTESPIKLSPATPSRKRSV	2630400	0	7032
Q99567	49	Nuclear pore complex protein Nup88	NUP88	0.969774	3.40E-06	95.551	S	QSPTEAEKPASSSLPSSPPPQLLTRNVVFGL	0	1075800	5210
Q13263	601	Transcription intermediary factor 1-beta	TRIM28	0.706677	1.47E-11	95.541	S	GAEGPRLASPSGSTSSGLEVVAPEGTSAPGG	24270000	10247000	2456
Q9NQ55	238	Suppressor of SWI4 1 homolog	PPAN	0.995322	1.88E-11	95.516	S	RLQDISELLATGAGLSESEAEPDGDHNITEL	8854300	11795000	5899
Q16637	4	Survival motor neuron protein	SMN1	1	0.000594789	95.5	S	____________MAMSSGGSGGGVPEQEDSV	2021200	1156200	3021
Q16637	5	Survival motor neuron protein	SMN1	1	0.0032221	95.5	S	___________MAMSSGGSGGGVPEQEDSVL	1144000	742230	3022
Q16637	8	Survival motor neuron protein	SMN1	1	0.000399858	95.5	S	________MAMSSGGSGGGVPEQEDSVLFRR	2040800	1205200	3023
Q13595	100	Transformer-2 protein homolog alpha	TRA2A	0.999986	0.00348364	95.483	S	RSYTPEYRRRRSRSHSPMSNRRRHTGSRANP	0	1359400	2591
Q9H4Z3	159	Phosphorylated CTD-interacting factor 1	PCIF1	0.935296	0.00264683	95.483	S	SPSIPGTPTLKMWGTSPEDKQQAALLRPTEV	534130	602290	5705
Q13542	65	Eukaryotic translation initiation factor 4E-binding protein	EIF4EBP2	1	1.04E-06	95.445	S	TRIIYDRKFLLDRRNSPMAQTPPCHLPNIPG	7925200	4684600	2574
Q9BTA9	523	WW domain-containing adapter protein with coiled-coil	WAC	0.521842	1.45E-06	95.444	S	EPVSPRSLQRSSSQRSPSPGPNHTSNSSNAS	8689400	0	5350
Q9BTA9	525	WW domain-containing adapter protein with coiled-coil	WAC	0.521842	1.45E-06	95.444	S	VSPRSLQRSSSQRSPSPGPNHTSNSSNASNA	8689400	0	5351
Q9BTA9	531	WW domain-containing adapter protein with coiled-coil	WAC	0.422512	1.45E-06	95.444	T	QRSSSQRSPSPGPNHTSNSSNASNATVVPQN	0	0	8045
Q9UHB6	487	LIM domain and actin-binding protein 1	LIMA1	0.82459	0.0205896	95.428	T	EEILERPAQLANARETPHSPGVEDAPIAKVG	0	5331700	8212
O43432	232	Eukaryotic translation initiation factor 4 gamma 3	EIF4G3	1	0.0135016	95.414	S	KQEEKPKPDPVLKSPSPVLRLVLSGEKKEQE	1229400	1020000	387
Q9BWH2	151	FUN14 domain-containing protein 2	FUNDC2	1	0.00265454	95.401	S	EKDMKKAKEQLKIRKSNQIPTEVRSKAEEVV	4866800	1688700	5430
Q9H444	184	Charged multivesicular body protein 4b	CHMP4B	0.952263	8.15E-06	95.361	S	EELEQEELDKNLLEISGPETVPLPNVPSIAL	776740	782750	5658
Q96JK2	794	DDB1- and CUL4-associated factor 5	DCAF5	1	0.00176576	95.358	S	KLNGKALSSRAEEPPSPPVPKASGSTLNSGS	1045100	0	4966
Q9H501	657	ESF1 homolog	ESF1	1	1.49E-06	95.302	S	KKRLKRKQKALAEEASEEELPSDVDLNDPYF	20638000	10418000	5706
Q92922	310	SWI/SNF complex subunit SMARCC1	SMARCC1	1	0.00161743	95.273	S	SFRQRISTKNEEPVRSPERRDRKASANARKR	0	0	4730
Q13586	519	Stromal interaction molecule 1	STIM1	0.848567	0.00221565	95.264	S	TEPQHGLGSQRDLTHSDSESSLHMSDRQRVA	1088500	702110	2584
Q8IW50	113	Protein FAM219A	FAM219A	0.617008	1.13E-06	95.255	T	SLDQSPDEKPLVALDTDSDDDFDMSRYSSSG	0	0	7840
P27707	74	Deoxycytidine kinase	DCK	0.999757	0.000508432	95.223	S	CNVQSTQDEFEELTMSQKNGGNVLQMMYEKP	348290	0	1286
Q02952	504	A-kinase anchor protein 12	AKAP12	0.817535	0.000147403	95.223	S	LSKPPEGVVSEVEMLSSQERMKVQGSPLKKL	1198900	143270	2127
Q13620	140	Cullin-4B	CUL4B	0.541113	5.86E-06	95.195	S	LEFVGFDAKMAEESSSSSSSSSPTAATSQQQ	1284700	0	2610
Q13620	143	Cullin-4B	CUL4B	0.258892	5.86E-06	95.195	S	VGFDAKMAEESSSSSSSSSPTAATSQQQQLK	0	0	2613
O43572	52	A-kinase anchor protein 10, mitochondrial	AKAP10	0.881568	0.00190054	95.183	S	KTSDVKSIKASISVHSPQKSTKNHALLEAAG	0	0	401
Q12802	1932	A-kinase anchor protein 13	AKAP13	0.968826	0.00147811	95.183	S	ENMSNTWKFLSHSTDSLNKISKVNESTESLT	2056100	44389	2310
Q13158	194	FAS-associated death domain protein	FADD	0.437605	0.00939647	95.183	S	EVQQARDLQNRSGAMSPMSWNSDASTSEAS_	0	0	2427
Q13158	197	FAS-associated death domain protein	FADD	0.437605	0.00939647	95.183	S	QARDLQNRSGAMSPMSWNSDASTSEAS____	0	0	2428
Q8WUB8	12	PHD finger protein 10	PHF10	1	1.50E-05	95.183	S	____MAAAAGPGAALSPRPCDSDPATPGAQS	1840400	942180	4509
Q92581	625	Sodium/hydrogen exchanger 6	SLC9A6	0.926636	0.00232937	95.183	S	EPATSSAPRRFMGNSSEDALDRELAFGDHEL	347580	226000	4647
Q9H5H4	83	Zinc finger protein 768	ZNF768	0.999947	0.00177813	95.183	S	QSPEFEPQSPRFEPESPGFESRSPGLVPPSP	6526600	5931500	5715
Q05209	571	Tyrosine-protein phosphatase non-receptor type 12	PTPN12	0.780618	1.48E-06	95.153	S	SDINYQTRKTVSLTPSPTTQVETPDLVDHDN	1821800	1229200	2186
Q9UID3	13	Vacuolar protein sorting-associated protein 51 homolog	VPS51	0.50465	8.90E-07	95.153	S	___MAAAAAAGPSPGSGPGDSPEGPEGEAPE	0	5851200	6377
Q05209	569	Tyrosine-protein phosphatase non-receptor type 12	PTPN12	0.954455	1.48E-06	95.153	T	NSSDINYQTRKTVSLTPSPTTQVETPDLVDH	1821800	1229200	7495
O00499	298	Myc box-dependent-interacting protein 1	BIN1	0.999991	0.00102336	95.094	S	AQPSDNAPAKGNKSPSPPDGSPAATPEIRVN	2671800	2385900	123
Q8TBZ3	432	WD repeat-containing protein 20	WDR20	0.499995	0.00513643	95.073	S	SVTTPGNSVPPPLPRSNSLPHSAVSNAGSKS	1075600	858330	4456
Q8TBZ3	434	WD repeat-containing protein 20	WDR20	0.947162	0.00312551	95.073	S	TTPGNSVPPPLPRSNSLPHSAVSNAGSKSSV	1075600	858330	4457
O15027	1325	Protein transport protein Sec16A	SEC16A	0.669021	0.000100706	94.958	T	RPEKRDNNWRYDPRFTGSFDDDPDPHRDPYG	1786600	1469500	7140
Q9NTJ3	1056	Structural maintenance of chromosomes protein 4	SMC4	0.989584	3.83E-06	94.955	S	KISLHPIEDNPIEEISVLSPEDLEAIKNPDS	851260	0	6017
Q9P2D1	2983	Chromodomain-helicase-DNA-binding protein 7	CHD7	0.999999	2.79E-08	94.929	S	SSLLEDEIAQGEELDSLDGGDEIENNENDE_	2105300	1178000	6235
Q5VT52	730	Regulation of nuclear pre-mRNA domain-containing protein 2	RPRD2	0.444062	5.39E-06	94.891	S	TSIDNIDGTPVRDERSGTPTQDEMMDKPTSS	0	0	3336

Q5VT52	732	Regulation of nuclear pre-mRNA domain-containing protein 2	RPRD2	0.444062	5.39E-06	94.891	T	IDNIDGTPVRDERSGTPTQDEMMDKPTSSSV	0	0	7733
Q9Y4U1	275	Methylmalonic aciduria and homocystinuria type C protein	MMACHC	0.999016	0.00277536	94.85	S	PGNPSRARSWLSPRVSPPASPGP________	1309900	782390	6940
P0DJ93	58	Small integral membrane protein 13	SMIM13	0.490979	5.72E-07	94.837	S	ELVGDTGSQEGDHEPSGSETEEDTSSSPHRI	0	0	979
P0DJ93	60	Small integral membrane protein 13	SMIM13	0.490979	5.72E-07	94.837	S	VGDTGSQEGDHEPSGSETEEDTSSSPHRIRS	0	0	980
Q5VWG9	229	Transcription initiation factor TFIID subunit 3	TAF3	0.989315	6.41E-08	94.837	S	EPLSSINTQKIPPMLSPVHVQDSTDLAPPSP	3502200	2517500	3357
Q5VWG9	236	Transcription initiation factor TFIID subunit 3	TAF3	0.380472	6.41E-08	94.837	S	TQKIPPMLSPVHVQDSTDLAPPSPEPPMLAP	0	0	3358
Q5VWG9	243	Transcription initiation factor TFIID subunit 3	TAF3	1	6.41E-08	94.837	S	LSPVHVQDSTDLAPPSPEPPMLAPVAKSQMP	3502200	2517500	3359
Q9H4Z3	19	Phosphorylated CTD-interacting factor 1	PCIF1	0.502849	6.62E-05	94.837	S	ENHGSPREEASLLSHSPGTSNQSQPCSPKPI	0	1923900	5699
Q9H4Z3	14	Phosphorylated CTD-interacting factor 1	PCIF1	0.93876	6.63E-05	94.834	S	__MANENHGSPREEASLLSHSPGTSNQSQPC	0	0	5698
Q12888	265	Tumor suppressor p53-binding protein 1	TP53BP1	1	0.00278589	94.82	S	KEQNPPPARSEDMPFSPKASVAAMEAKEQLS	1506100	1076200	2364
Q13523	292	Serine/threonine-protein kinase PRP4 homolog	PRPF4B	1	0.0170161	94.82	S	SPIINESRSRDRGKKSRSPVDLRGKSKDRRS	5889600	5791600	2558
Q13523	294	Serine/threonine-protein kinase PRP4 homolog	PRPF4B	1	0.0170161	94.82	S	IINESRSRDRGKKSRSPVDLRGKSKDRRSRS	5889600	5791600	2559
Q5VUA4	79	Zinc finger protein 318	ZNF318	1	0.0211266	94.82	S	RGRRASPSPPRGRRVSPSPPRARRGSPSPPR	0	686610	3345
Q5VUA4	81	Zinc finger protein 318	ZNF318	1	0.0211266	94.82	S	RRASPSPPRGRRVSPSPPRARRGSPSPPRGR	0	686610	3346
Q9NVE7	391	Pantothenate kinase 4	PANK4	0.494322	2.49E-08	94.802	S	QYSWGENYAGSSGLMSASPELGPAQRARSGT	0	0	6037
Q9NVE7	393	Pantothenate kinase 4	PANK4	0.943987	2.49E-08	94.802	S	SWGENYAGSSGLMSASPELGPAQRARSGTFD	0	2639400	6038
Q14135	149	Transcription cofactor vestigial-like protein 4	VGLL4	0.996929	0.00375062	94.781	S	ALTKNSLDASRPAGLSPTLTPGERQQNRPSV	615910	448630	2648
Q14739	97	Lamin-B receptor	LBR	0.861491	0.00190902	94.781	S	SRSRSPGRPPKSARRSASASHQADIKEARRE	0	0	2809
Q8WZ42	17195	Titin	TTN	0.820869	0.0270191	94.781	S	VIEKREANRKTWVHVSSEPKECTYTIPKLLE	0	0	4614
P49454	3054	Centromere protein F	CENPF	0.999998	0.00202191	94.767	S	TAGGSRSQKVKVAQRSPVDSGTILREPTTKS	2737100	2596100	1666
Q96FF9	21	Sororin	CDCA5	0.99997	0.0127571	94.692	S	TRSGGAAQRSGPRAPSPTKPLRRSQRKSGSE	1253200	0	4904
Q9BQG0	1290	Myb-binding protein 1A	MYBBP1A	0.999919	0.00449375	94.692	S	KQHQKALPKKGVLGKSPLSALARKKARLSLV	757590	339460	5312
P10412;P1	36;37;36	Histone H1.4;Histone H1.3;Histone H1.2	HIST1H1E;HI	0.999995	0.00253758	94.688	S	KKAKKAGATAGKRKASGPPVSELITKAVAAS;KKA	1255100	968620	987
Q8TAD8	35	Smad nuclear-interacting protein 1	SNIP1	1	0.000964857	94.688	S	DVVLPAGVVVKQERLSPEVAPPAHRRPDHSG	4691600	2172900	4430
Q2M2I8	605	AP2-associated protein kinase 1	AAK1	0.767267	0.0154785	94.688	T	EPAIQAPVRQQPKVQTTPPPAVQGQKVGSLT	0	0	7665
O75534	482	Cold shock domain-containing protein E1	CSDE1	0.775617	0.00380346	94.673	S	KLTIAFQAKDVEGSTSPQIGDKVEFSISDKQ	0	0	642
Q15154	1186	Pericentriolar material 1 protein	PCM1	0.948225	0.030491	94.673	S	SGKTEYMAFPKPFESSSSIGAEKPRNKKLPE	773710	0	2881
Q15154	1187	Pericentriolar material 1 protein	PCM1	0.984387	0.030491	94.673	S	GKTEYMAFPKPFESSSSIGAEKPRNKKLPEE	773710	0	2882
Q15154	1188	Pericentriolar material 1 protein	PCM1	0.906122	0.030491	94.673	S	KTEYMAFPKPFESSSSIGAEKPRNKKLPEEE	773710	0	2883
Q5BKY9	196	Protein FAM133B	FAM133B	0.87238	2.38E-05	94.671	S	RKMYSEDKPLSSESLSESEYIEEVRAKKKKS	4108400	3796400	3155
Q66K74	729	Microtubule-associated protein 1S;MAP1S heavy chain;MAP1S li	MAP1S	0.999937	0.000204272	94.671	S	EAGLSLPLRGPRARRSASPHDVDLCLVSPCE	904020	736130	3409
Q9UQ35	323	Serine/arginine repetitive matrix protein 2	SRRM2	0.958585	7.00E-06	94.671	S	APFSEPGTTSTQRPSSPETATKQPSSPYEDK	58931000	64490000	6631
O95785	1006	Protein Wiz	WIZ	0.999994	0.00701298	94.639	S	LALAGSPTPKNPEDKSPQLSLSPRPASPKAQ	968520	458970	814
O95785	1010	Protein Wiz	WIZ	0.863211	0.00782052	94.639	S	GSPTPKNPEDKSPQLSLSPRPASPKAQWPQS	774530	414290	815
Q9P107	919	GEM-interacting protein	GMIP	0.999979	0.0173663	94.639	S	PRGSLRGRGPSPAAASPEGSPLRRTPLPKHF	0	0	6189
Q9P107	923	GEM-interacting protein	GMIP	1	0.0173663	94.639	S	LRGRGPSPAAASPEGSPLRRTPLPKHFEITQ	0	0	6190
Q6P1L5	108	Protein FAM117B	FAM117B	0.397953	0.0157815	94.639	T	SPTRGGGNAAARTSPTVATQTGASATSTRGT	0	0	7755
Q92560	292	Ubiquitin carboxyl-terminal hydrolase BAP1	BAP1	1	0.00285698	94.616	S	QESQLPEESKSASNKSPLVLEANRAPAASEG	480840	601130	4639
Q9UHR5	113	SAP30-binding protein	SAP30BP	1	0.00453627	94.616	S	PQELVASFSERVRNMSPDEIKIPPEPPGRCS	594950	916940	6364
Q8TAD8	202	Smad nuclear-interacting protein 1	SNIP1	0.999382	0.00472809	94.61	S	REHRQRNDVGGGGSESQELVPRPGGNNKEKE	306120	726410	4431
P54725	123	UV excision repair protein RAD23 homolog A	RAD23A	0.968896	1.05E-06	94.57	S	TSGMSHPPPAAREDKSPSEESAPTTSPESVS	832460	560550	1851
O14654	1252	Insulin receptor substrate 4	IRS4	1	0.0118317	94.547	S	THVRMDFARRDNQFDSPKRGR__________	0	256680	179
Q9Y3X0	80	Coiled-coil domain-containing protein 9	CCDC9	0.994497	0.0117334	94.547	S	NVAVESEKNLGPSRRSPGTPRPPGASKGGRT	3631700	1393300	6884
P62995;Q1	268;264	Transformer-2 protein homolog beta;Transformer-2 protein hom	TRA2B;TRA2A	0.808386	0.00642323	94.538	Y	AQDRDQIYRRRSPSPYYSRGGYRSRSRSRSY;YEDY	0	0	8369
Q03164	1684	Histone-lysine N-methyltransferase 2A;MLL cleavage product N	KMT2A	0.5	0.0181181	94.532	S	PPDLNPETEESIPSRSSPEGPDPPVLTEVSK	790690	357210	2147
Q03164	1685	Histone-lysine N-methyltransferase 2A;MLL cleavage product N	KMT2A	0.5	0.0181181	94.532	S	PDLNPETEESIPSRSSPEGPDPPVLTEVSKQ	790690	357210	2148
O75145	1164	Liprin-alpha-3	PPFIA3	1	0.0168088	94.465	S	MLPPNFRSAAAGALGSPGLPLRKLQPEGQTS	461970	514830	566
Q13620	138	Cullin-4B	CUL4B	0.156931	1.74E-05	94.463	S	DTLEFVGFDAKMAEESSSSSSSSSPTAATSQ	0	0	2608
Q13620	139	Cullin-4B	CUL4B	0.156931	1.74E-05	94.463	S	TLEFVGFDAKMAEESSSSSSSSSPTAATSQQ	0	0	2609
Q9HCD6	1446	Protein TANC2	TANC2	0.856309	0.00133724	94.456	S	ARPSQGLPVIQSPPSSPPHRDSAYISSSPLG	3894200	0	5852
Q9GZN2	2	Homeobox protein TGIF2	TGIF2	1	0.00163045	94.454	S	_______________MSDSDLGEDEGLLSLAG	1062200	934820	5549
Q9GZN2	4	Homeobox protein TGIF2	TGIF2	1	0.00163045	94.454	S	_____________MSDSDLGEDEGLLSLAGKR	724820	579660	5550
O14828	76	Secretory carrier-associated membrane protein 3	SCAMP3	0.99822	0.0191036	94.409	S	LPPPSAPSLQPSRKLSPTEPKNYGSYSTQAS	1396400	782100	210
P53355	698	Death-associated protein kinase 1	DAPK1	0.684725	0.0029293	94.409	S	NLQPRIKLKLFGHSGSGKTTLVESLKCGLLR	0	2762800	1832
Q8IY21	545	Probable ATP-dependent RNA helicase DDX60	DDX60	0.622252	0.00266732	94.409	S	RSVQKYHVFQRFYGNSLETVSSKIIVTQTIK	7538400	18720000	4117
Q92530	153	Proteasome inhibitor PI31 subunit	PSMF1	0.968795	0.00465217	94.409	S	IITPIHEQWEKANVSSPHREFPPATAREVDP	2147600	3078500	4620
Q9Y365	284	PCTP-like protein	STARD10	0.999241	0.00482099	94.409	S	AESREERMGGAGGEGSDDDTSLT________	0	1669700	6842
Q9Y6X9	615	MORC family CW-type zinc finger protein 2	MORC2	1	0.0029293	94.409	S	RPSTEEPVRRPQRPRSPPLPAVIRNAPSRPP	9823000	6305700	7034
Q6W2J9	1139	BCL-6 corepressor	BCOR	0.899964	3.70E-05	94.403	S	MPHSPTLRVDRKRKVSGDSSHTETTAEEVPE	1624800	1007400	3612
Q8IYB3	555	Serine/arginine repetitive matrix protein 1	SRRM1	0.963646	0.00226398	94.402	T	TSPRGRRRRSPSPPPTRRRRSPSPAPPPRRR	0	0	7864
Q9UQ35	1550	Serine/arginine repetitive matrix protein 2	SRRM2	0.872559	0.00156867	94.359	S	QRSRSGSSQELDVKPSASPQERSESDSSPDS	4893100	770930	6690
O95429	280	BAG family molecular chaperone regulator 4	BAG4	0.768052	9.03E-10	94.352	S	PPGNLYMTESTSPWPSSGSPQSPPSPPVQQP	3882300	0	770
O95429	289	BAG family molecular chaperone regulator 4	BAG4	0.990019	9.03E-10	94.352	S	STSPWPSSGSPQSPPSPPVQQPKDSSYPYSQ	6094900	4711500	774
Q13427	256	Peptidyl-prolyl cis-trans isomerase G	PPIG	0.999999	2.59E-07	94.352	S	HKKEKKKRKKSKKSASSESEAENLEAQPQST	11377000	4728500	2497
Q13427	257	Peptidyl-prolyl cis-trans isomerase G	PPIG	0.999999	2.59E-07	94.352	S	KKEKKKRKKSKKSASSESEAENLEAQPQSTV	12024000	4917100	2498
Q13427	259	Peptidyl-prolyl cis-trans isomerase G	PPIG	0.999999	2.59E-07	94.352	S	EKKKRKKSKKSASSESEAENLEAQPQSTVRP	7955700	4846100	2499
Q5T0N5	501	Formin-binding protein 1-like	FNBP1L	0.999026	0.0157017	94.339	S	RHSSDINHLVTQGRESPEGSYTDDANQEVRG	432500	523080	3239
O14497	1754	AT-rich interactive domain-containing protein 1A	ARID1A	0.5	0.00601076	94.325	S	PGQRTLLDPGRFSKVSSPAPMEGGEEEEELL	1272600	0	148
O14497	1755	AT-rich interactive domain-containing protein 1A	ARID1A	0.5	0.00601076	94.325	S	GQRTLLDPGRFSKVSSPAPMEGGEEEEELLG	1272600	0	149
Q6VY07	528	Phosphofurin acidic cluster sorting protein 1	PACS1	0.326756	0.000830088	94.313	S	LKERQLSKPLSERTNSSDSERSPDLGHSTQI	0	0	3606
Q6VY07	529	Phosphofurin acidic cluster sorting protein 1	PACS1	0.326756	0.000830088	94.313	S	KERQLSKPLSERTNSSDSERSPDLGHSTQIP	0	0	3607
Q6VY07	534	Phosphofurin acidic cluster sorting protein 1	PACS1	0.943961	0.000830088	94.313	S	SKPLSERTNSSDSERSPDLGHSTQIPRKVVY	4053100	0	3609
Q96P48	229	Arf-GAP with Rho-GAP domain, ANK repeat and PH domain-contai	ARAP1	0.884144	2.99E-05	94.313	S	EIPPKPVRLFPEFDDSDYDEVPEEGPGAPAR	2333200	1310200	5047
Q6VY07	526	Phosphofurin acidic cluster sorting protein 1	PACS1	0.456065	0.000830088	94.313	T	TPLKERQLSKPLSERTNSSDSERSPDLGHST	0	0	7775
Q9BYW2	654	Histone-lysine N-methyltransferase SETD2	SETD2	0.99782	0.01336	94.309	S	EFITHDSHDSIKELDSLSKVKNDQLRSFCPI	1167800	0	5473
Q9P206	669	Uncharacterized protein KIAA1522	KIAA1522	0.999996	0.00023977	94.301	S	TLTPLQESPVISKDQSPPPSPPPSYHPPPPP	10758000	7949300	6207
Q9P206	673	Uncharacterized protein KIAA1522	KIAA1522	0.998063	0.00023977	94.301	S	LQESPVISKDQSPPPSPPPSYHPPPPPTKKP	10758000	7949300	6208
Q9UER7	737	Death domain-associated protein 6	DAXX	1	0.0102862	94.281	S	TSVATQCDPEEIIVLSDSD____________	0	0	6292
Q9UER7	739	Death domain-associated protein 6	DAXX	1	0.0102862	94.281	S	VATQCDPEEIIVLSDSD______________	0	0	6293
Q13542	45	Eukaryotic translation initiation factor 4E-binding protein	EIF4EBP2	0.470174	2.08E-07	94.275	T	LPHDYCTTPGGTLFSTTPGGTRIIYDRKFLL	0	0	7562
P18887	446	DNA repair protein XRCC1	XRCC1	0.49875	0.000470715	94.271	S	KQPQTKTKPTQAAGPSSPQKPPTPEETKAAS	0	0	1157
P18887	453	DNA repair protein XRCC1	XRCC1	0.997622	0.000470715	94.271	T	KPTQAAGPSSPQKPPTPEETKAASPVLQEDI	18999000	9375800	7304
P49327	1411	Fatty acid synthase;[Acyl-carrier-protein] S-acetyltransfera	FASN	0.999867	0.000915063	94.203	S	YGSTLFLCRRPTPQDSPIFLPVDDTSFRWVE	0	0	1654
Q14980	169	Nuclear mitotic apparatus protein 1	NUMA1	0.982937	2.76E-05	94.188	S	APVPSTCSSTFPEELSPPSHQAKREIRFLEL	1723000	1479200	2827
Q99613;B5	9;9	Eukaryotic translation initiation factor 3 subunit C;Eukaryo	EIF3C;EIF3C	0.78228	2.50E-06	94.185	S	_______MSRFFTTGSDSESESSLSGEELVT	13071000	436120	64
Q99613;B5	15;15	Eukaryotic translation initiation factor 3 subunit C;Eukaryo	EIF3C;EIF3C	0.693229	2.50E-06	94.185	S	_MSRFFTTGSDSESESSLSGEELVTKPVGGN	0	1944300	67
Q99613;B5	16;16	Eukaryotic translation initiation factor 3 subunit C;Eukaryo	EIF3C;EIF3C	0.702861	2.50E-06	94.185	S	MSRFFTTGSDSESESSLSGEELVTKPVGGNY	11782000	1944300	68
Q15629	365	Translocating chain-associated membrane protein 1	TRAM1	0.999514	0.00996175	94.176	S	TENGVNGTLTSNVADSPRNKKEKSS______	287830	198730	2950
Q4LE39	1029	AT-rich interactive domain-containing protein 4B	ARID4B	0.513679	1.70E-07	94.152	S	SGSNSVLNTPPTTPESPSSVTVTEGSRQQSS	829820	643920	3100
Q5T200	1455	Zinc finger CCCH domain-containing protein 13	ZC3H13	0.996667	8.73E-08	94.152	S	EGDDESKLDDAHSLGSGAGEGYEPISDDELD	3942400	2532600	3258
Q4LE39	1022	AT-rich interactive domain-containing protein 4B	ARID4B	0.994924	1.70E-07	94.152	T	RKAEFPSSGSNSVLNTPPTTPESPSSVTVTE	2596700	643920	7683
Q12802	1930	A-kinase anchor protein 13	AKAP13	0.678457	0.00215464	94.122	T	NDENMSNTWKFLSHSTDSLNKISKVNESTES	4024500	0	7509
O95671	19	N-acetylserotonin O-methyltransferase-like protein	ASMTL	0.734375	0.00303223	94.114	S	CPVIGKLLHKRVVLASASPRRQEILSNAGLR	0	0	790
Q9BVT8	98	Transmembrane and ubiquitin-like domain-containing protein 1	TMUB1	0.999945	3.88E-06	94.08	S	PEPSTGFTATPPAPDSPQEPLVLRLKFLNDS	595570	597100	5408
Q9BVT8	92	Transmembrane and ubiquitin-like domain-containing protein 1	TMUB1	0.987379	3.88E-06	94.08	T	RGQAAQPEPSTGFTATPPAPDSPQEPLVLRL	595570	597100	8054
O95487	329	Protein transport protein Sec24B	SEC24B	0.659684	2.54E-05	94.037	T	VSTVLSGSSGSSSTRTPPTANHPVEPVTSVT	113830	0	7241
Q13428	533	Treacle protein	TCOF1	0.987343	0.0116081	94.023	T	GKGAGPVPPGKVGPATPSAQVGKWEEDSESS	154110	476230	7542
O14617	829	AP-3 complex subunit delta-1	AP3D1	0.671556	0.00456534	94.007	S	EKLPIQKHRNTETSKSPEKDVPMVEKKSKKP	0	0	155
Q9HB90	16	Ras-related GTP-binding protein C	RRAGC	0.90647	0.000810015	94.006	Y	MSLQYGAEETPLAGSYGAADSFPKDFGYGVE	0	1331900	8426
Q02880	1400	DNA topoisomerase 2-beta	TOP2B	0.993896	3.61E-06	93.969	S	DDDDNNDLEELKVKASPITNDGEDEFVPSDG	4339900	8932300	2097
Q02880	1424	DNA topoisomerase 2-beta	TOP2B	0.987782	3.61E-06	93.969	S	EFVPSDGLDKDEYTFSPGKSKATPEKSLHDK	4339900	10381000	2099
Q9H4L5	303	Oxysterol-binding protein-related protein 3	OSBPL3	0.499995	0.00207279	93.909	S	TLQVPKPFSGPVRLHSSNPNLSTLDFGEEKN	0	2467400	5679
Q9NWH9	550	SAFB-like transcription modulator	SLTM	0.565759	0.00312754	93.909	S	QTSESIKKSEEKKRISSKSPGHMVILDQTKG	9788500	0	6056
Q7L2J0	330	7SK snRNA methylphosphate capping enzyme	MEPCE	0.981639	5.72E-09	93.807	S	PYELNTAINCRDEVVSPLPSALQGPSGSLSA	2261300	1944900	3723
P49792;Q7	2239;1264	E3 SUMO-protein ligase RanBP2;RanBP2-like and GRIP domain-co	RANBP2;RGPD	0.658655	2.55E-05	93.776	S	WDNYDLREDALDDSVSSSSVHASPLASSPVR	0	0	1703
O14617	636	AP-3 complex subunit delta-1	AP3D1	1	8.42E-06	93.767	S	LDLDAWINEPLSDSESEDERPRAVFHEEEQR	4064300	1905700	158
Q92793	983	CREB-binding protein	CREBBP	0.91099	1.08E-05	93.767	T	IDNRVPTPSSVASAETNSQQPGPDVPVLEMK	1375100	0	7944
O94854	77	Uncharacterized protein KIAA0754	KIAA0754	1	0.0023542	93.766	S	SKSIGIPEVQDFKNLSGDCQDFRFQQHSANP	530420	424080	707
Q92733	159	Proline-rich protein PRCC	PRCC	0.999992	0.00031285	93.753	S	PVKIAAPELHKGDSDSEEDEPTKKKTILQGS	37900000	16547000	4693
Q15398	798	Disks large-associated protein 5	DLGAP5	0.40994	3.00E-05	93.74	T	ETKISQSELFDNKSLTTECHLLDSPGLNCSN	0	0	7636
Q15398	799	Disks large-associated protein 5	DLGAP5	0.40994	3.00E-05	93.74	T	TKISQSELFDNKSLTTECHLLDSPGLNCSNP	0	0	7637
P38398	1336	Breast cancer type 1 susceptibility protein	BRCA1	0.999118	0.00426164	93.738	S	SKQMRHQSESQGVGLSDKELVSDDEERGTGL	0	0	1450
Q7Z3K3	1338	Pogo transposable element with ZNF domain	POGZ	0.804477	0.000252102	93.729	S	FLVASVLPGPDGNINSPTRNADMQEELIASL	1020700	746590	3781
Q9BVS4	337	Serine/threonine-protein kinase RIO2	RIOK2	0.834543	0.0178594	93.716	S	DDKNIETKEGSEFSFSDGEVAEKAEVYGSEN	414930	2291800	5406
Q0JRZ9	312	F-BAR domain only protein 2	FCHO2	0.999962	5.44E-08	93.667	S	KKEKDAESVECPDADSLNIPDVDEEGYSIKP	2567800	767870	2282
Q6DN90	924	IQ motif and SEC7 domain-containing protein 1	IQSEC1	0.530233	0.00506778	93.667	S	DSYASGEGLKRSALSSSLRDLSEAGKRGRRS	0	188240	3448
Q99569	281	Plakophilin-4	PKP4	0.999083	0.00180207	93.667	S	LPAARAASPYSQRPASPTAIRRIGSVTSRQT	0	473880	5212
B2RTY4	1301	Unconventional myosin-IXa	MYO9A	0.718919	0.00233079	93.649	S	SLELLKVRSLGGISPSEDRRWSTELVPEGLQ	534700	0	63
Q86X29	643	Lipolysis-stimulated lipoprotein receptor	LSR	1	0.00896806	93.649	S	SQASRERRLKKNLALSRESLVV_________	472500	514030	4007
Q86X29	646	Lipolysis-stimulated lipoprotein receptor	LSR	1	0.00896806	93.649	S	SRERRLKKNLALSRESLVV____________	472500	514030	4008
Q96AV8	410	Transcription factor E2F7	E2F7	0.99993	0.00302139	93.649	S	GQIQVCAKQKLARHGSFNTVQASERIQRKVN	299040	0	4778
Q6WCQ1	991	Myosin phosphatase Rho-interacting protein	MPRIP	0.657387	0.0144405	93.623	S	KSPDSATVSGYDIMKSKSNPDFLKKDRSCVT	0	0	3633
Q9UIG0	361	Tyrosine-protein kinase BAZ1B	BAZ1B	1	0.00234043	93.623	S	LSGSPLKVKNSKNSKSPEEHLEEMMKMMSPN	0	0	6386
Q8NHH9	24	Atlastin-2	ATL2	0.455941	1.36E-07	93.6	S	RGQQPHQGLWRRRRTSDPSAAVNHVSSTTSL	0	0	4412
Q8NHH9	23	Atlastin-2	ATL2	0.455941	1.36E-07	93.6	T	ARGQQPHQGLWRRRRTSDPSAAVNHVSSTTS	0	0	7900
Q14680	529	Maternal embryonic leucine zipper kinase	MELK	1	0.0119805	93.556	S	HMEETPKRKGAKVFGSLERGLDKVITVLTRS	2225800	1179400	2776
Q96G46	272	tRNA-dihydrouridine(47) synthase [NAD(P)(+)]-like	DUS3L	0.366468	4.66E-05	93.551	S	QENCGAQQVPAGPGTSTPPSSPVRTCGPLTD	0	0	4909
Q96G46	271	tRNA-dihydrouridine(47) synthase [NAD(P)(+)]-like	DUS3L	0.366468	4.66E-05	93.551	T	RQENCGAQQVPAGPGTSTPPSSPVRTCGPLT	0	0	7981
Q7Z2W4	346	Zinc finger CCCH-type antiviral protein 1	ZC3HAV1	0.730567	2.29E-07	93.529	S	LENGSQEDLLHGNPGSTYLASNSTSAPNWKS	9455700	0	3752
Q13263	50	Transcription intermediary factor 1-beta	TRIM28	0.815308	5.98E-09	93.494	S	APSAAASASASAAASSPAGGGAEALELLEHC	0	270790	2462
P33991	2	DNA replication licensing factor MCM4	MCM4	0.499991	0.00139348	93.429	S	_______________MSSPASTPSRRGSRRGR	0	15359000	1368
P33991	3	DNA replication licensing factor MCM4	MCM4	0.499991	0.00139348	93.429	S	______________MSSPASTPSRRGSRRGRA	0	15359000	1369
Q8TBP0	103	TBC1 domain family member 16	TBC1D16	0.991113	0.0191838	93.429	S	QRQDEEALRYITPESSPVRKAPRPRGRRTRS	0	606200	4455
Q8TBP0	99	TBC1 domain family member 16	TBC1D16	0.995501	0.0191838	93.429	T	NSRIQRQDEEALRYITPESSPVRKAPRPRGR	0	606200	7904
O15068	975	Guanine nucleotide exchange factor DBS	MCF2L	0.539668	0.000267463	93.427	S	LEQSQSLPLPAPTSTSPSRGNSRNIKKLEER	987910	0	276
Q9H165	328	B-cell lymphoma/leukemia 11A	BCL11A	0.719304	0.00592062	93.424	S	MDFSRRLRELAGNTSSPPLSPGRPSPMQRLL	829930	478240	5587
Q14684	458	Ribosomal RNA processing protein 1 homolog B	RRP1B	0.701182	3.97E-06	93.416	S	VAEPGAEATSSTGEESGSEHPPAVPMHNKRK	0	0	2790
Q8IWZ3	93	Ankyrin repeat and KH domain-containing protein 1	ANKHD1	0.623268	1.39E-07	93.411	S	KLAAAVLRTGGGGGASGSDEDEVSEVESFIL	1323200	1207300	4084
Q8IWZ3	95	Ankyrin repeat and KH domain-containing protein 1	ANKHD1	0.672233	1.39E-07	93.411	S	AAAVLRTGGGGGASGSDEDEVSEVESFILDQ	1323200	1207300	4085

Q8IWZ3	86	Ankyrin repeat and KH domain-containing protein 1	ANKHD1	0.657809	1.39E-07	93.411	T	GDAALDFKLAAAVLRTGGGGGASGSDEDEVS	1323200	1207300	7848
Q8WUB8	22	PHD finger protein 10	PHF10	0.497504	1.50E-05	93.388	T	PGAALSPRPCDSDPATPGAQSPKDDNEDNSN	0	0	7913
Q9NNW5	547	WD repeat-containing protein 6	WDR6	0.655688	9.75E-08	93.384	S	GKARAGAGAPVVGSGSSGGGNAFTGLGPVST	1102600	948320	5886
Q86VQ1	110	Glucocorticoid-induced transcript 1 protein	GLCCI1	0.926172	0.000267716	93.342	T	LPGPGAARGPSPSSPTPPAAAAPAEQAPRAK	3715600	1856000	7828
Q15742	171	NGFI-A-binding protein 2	NAB2	1	0.000621817	93.243	S	RSFSPKSPLELGEKLSPLPGGPGAGDPRIWP	595140	2175100	2980
Q96JH7	997	Deubiquitinating protein VCIP135	VCPIP1	0.751867	0.0123635	93.237	S	AVSQVRAEATTRSRESSPSHGLLKLGSGGVV	0	1049500	4961
Q96JH7	998	Deubiquitinating protein VCIP135	VCPIP1	0.80197	0.0123635	93.237	S	VSQVRAEATTRSRESSPSHGLLKLGSGGVVK	0	1049500	4962
Q9Y618	956	Nuclear receptor corepressor 2	NCOR2	1	0.0145009	93.228	S	RPSLLTPTGDPRANASPQKPLDLKQLKQRAA	0	928420	6977
Q9H3P7	43	Golgi resident protein GCP60	ACBD3	0.938798	4.47E-05	93.215	S	GAEGAPLLPPPLPPPSPPGSGRGPGASGEQP	1848100	939770	5640
Q8N9T8	628	Protein KRI1 homolog	KRI1	0.99999	6.72E-06	93.21	S	QRQLPALDGSLMGPESPPAQEEEAPVSPHKK	8245300	5565500	4306
P33240	524	Cleavage stimulation factor subunit 2	CSTF2	0.997307	8.45E-09	93.207	S	MQGASIQGGSQPGGFSPGQNQVTPQDHEKAA	1527600	805650	1363
Q8WVD3	139	E3 ubiquitin-protein ligase RNF138	RNF138	0.76647	2.45E-05	93.196	S	SQDSVGNSNRSETSTSDNTETYQENTSSSGH	1847200	0	4540
Q96E09	265	Protein FAM122A	FAM122A	0.308303	1.34E-05	93.187	T	SAGSSCNSPAKVSTTTDSPVSPAQAASPFIP	0	0	7979
P06400	794	Retinoblastoma-associated protein	RB1	0.874201	0.0122042	93.162	S	LSPIPHIPRSPYKFPSSPLRIPGGNIYISPL	749930	0	910
Q13501	332	Sequestosome-1	SQSTM1	0.999037	3.56E-07	93.147	S	ESEGRPEEQMESDNCSGGDDDWTHLSSKEVD	2117100	3000800	2552
Q15424	601	Scaffold attachment factor B1	SAFB	0.999994	0.0122151	93.143	S	ASKSQDRKSASREKRSVVSFDKVKEPRKSRD	437550	184020	2928
Q96ET8	223	Golgi apparatus membrane protein TVP23 homolog C	TVP23C	0.999005	0.0122151	93.143	S	ITDRALYWKPAPRLSSPPLRAAPGNCQQMAP	0	0	4888
Q9UHB7	1055	AF4/FMR2 family member 4	AFF4	1	0.00251914	93.143	S	QAPSPGLGSKAVGMPSPVSPKLSPGNSGNYS	3513800	2801600	6334
Q12789	1845	General transcription factor 3C polypeptide 1	GTF3C1	0.659053	4.97E-05	93.126	S	EDPQARPLEGSSSEDSPPEGQAPPSHSPRGT	1349100	0	2300
Q96JB3	479	Hypermethylated in cancer 2 protein	HIC2	0.488149	4.42E-07	93.126	T	HTEEELFIKEEGAYETGSGGAEEEAEDLSAP	0	0	7986
Q96N67	909	Dedicator of cytokinesis protein 7	DOCK7	0.59309	0.000303624	93.115	T	SRSLSNSNPDISGTPTSPDDEVRSIIGSKGL	0	2515800	7995
P46100	596	Transcriptional regulator ATRX	ATRX	0.910477	0.00327918	93.096	S	KVTKELYVKLTPVSLSNSPIKGADCQEVPQD	2704100	1640300	1553
Q9UQ35	1318	Serine/arginine repetitive matrix protein 2	SRRM2	1	0.00447849	93.096	S	SSWGGPHFSPEHKELSNSPLRENSFGSPLEF	1094300	2357100	6616
Q9UQ35	2453	Serine/arginine repetitive matrix protein 2	SRRM2	0.965199	0.00327918	93.096	S	SLLPPAQDQPRSPVPSAFSDQSRCLIAQTTP	1846700	0	6705
P29966	134	Myristoylated alanine-rich C-kinase substrate	MARCKS	0.477966	2.36E-07	93.001	S	PTAAEGEAASAASSTSSPKAEDGATPSPSNE	0	0	1332
Q8IWI9	2882	MAX gene-associated protein	MGA	1	0.00465084	92.943	S	DDDFSENEKQLAEPASEPDVLKIVIDSEIKD	87805	0	4058
Q5T5U3	1743	Rho GTPase-activating protein 21	ARHGAP21	0.497859	0.00262708	92.943	T	PSLTKVFDVMKKGKSTGSLLTPTRGESEKQE	0	0	7724
O60749	97	Sorting nexin-2	SNX2	0.932392	5.53E-05	92.932	S	SLDSPEREPILSSEPSPAVTPVTPTTLIAPR	628030	1107000	518
Q96JM3	603	Chromosome alignment-maintaining phosphoprotein 1	CHAMP1	1	0.0170413	92.925	S	DDQKCDILVQEELLASPKKLLEDTLFPSSKK	525030	263600	4967
Q13427	254	Peptidyl-prolyl cis-trans isomerase G	PPIG	0.999999	6.75E-07	92.833	S	RKHKKEKKRKKSKKSASSESEAENLEAQPQ	1105000	1523200	2496
Q9UQ35	864	Serine/arginine repetitive matrix protein 2	SRRM2	0.999999	0.00680937	92.805	S	PPRQGSITSPQANEQSVTPQRRSCFESSPDP	606940	388100	6669
Q6W2J9	1446	BCL-6 corepressor	BCOR	0.325988	1.73E-05	92.8	S	STQLPCSSSPQETTQSRPMPPEARRLIVNKN	0	0	3617
Q9P0V3	279	SH3 domain-binding protein 4	SH3BP4	1	0.00267725	92.792	S	SFFTGLKSPAPEQFQSREDFRTAWLNHRKLA	471220	257550	6188
Q9UQ35	322	Serine/arginine repetitive matrix protein 2	SRRM2	0.926825	2.22E-05	92.792	S	DAPFSEPGTTSTQRPSSPETATKQPSSPYED	5336900	8006800	6630
O95365	515	Zinc finger and BTB domain-containing protein 7A	ZBTB7A	0.70988	2.52E-05	92.792	T	KPRVRGGAPDPSPGATATPGAPAQPSSPDAR	0	0	7237
P07948	13	Tyrosine-protein kinase Lyn	LYN	0.991147	0.00265405	92.781	S	___MGCIKSKGKDSLSDDGVDLKTQPVRNTE	0	411310	945
Q5VVJ2	340	Histone H2A deubiquitinase MYSM1	MYSM1	1	0.00342569	92.781	S	KHDGRGIIVDARQLPSPEPCEIQKNLNDNEM	1604600	1086900	3356
Q86TI0	565	TBC1 domain family member 1	TBC1D1	0.89633	2.78E-06	92.695	S	KEALPISESSFKLLGSSEDLSSDSESHLPEE	3243100	1434500	3898
Q86TI0	585	TBC1 domain family member 1	TBC1D1	1	2.78E-06	92.695	S	SSDSESHLPEEPAPLSPQQAFRRRANTLSHF	5128000	1434500	3903
Q8TAP8	45	Protein phosphatase 1 regulatory subunit 35	PPP1R35	0.84846	0.000148701	92.679	S	VPQLRAPVPEPGLDLSLSPRPDSPQPRHGSP	2306100	1749600	4437
Q8TAP8	52	Protein phosphatase 1 regulatory subunit 35	PPP1R35	0.99917	0.000148701	92.679	S	VPEPGLDLSLSPRPDSPQPRHGSPGRRKGRA	4498300	3638600	4439
Q2M2Z5	317	Centrosomal protein kizuna	KIZ	1	0.000286209	92.671	S	EILTREHIEVEEKRASPPVSPIPVSEYCESE	4878800	1765700	3043
Q2M2Z5	321	Centrosomal protein kizuna	KIZ	0.999984	0.000286209	92.671	S	REHIEVEEKRASPPVSPIPVSEYCESENKWS	4878800	1765700	3044
P08240	297	Signal recognition particle receptor subunit alpha	SRPR	0.791762	3.37E-07	92.664	S	RGTGSGGQLQDLDCSSSDDEGAAQNSTKPSA	0	1606000	952
P08240	298	Signal recognition particle receptor subunit alpha	SRPR	0.943826	3.37E-07	92.664	S	GTGSGGQLQDLDCSSSDDEGAAQNSTKPSAT	1002800	1606000	953
P46821	1144	Microtubule-associated protein 1B;MAP1B heavy chain;MAP1 lig	MAP1B	0.957062	4.25E-05	92.613	S	EPTPMDMESTPRDVMSDETNNEETESPSQEF	1348400	0	1577
P23588	462	Eukaryotic translation initiation factor 4B	EIF4B	0.431995	0.00161733	92.575	S	ENETLNKEEDCHSPTSKPPKPDQPLKVMPAP	0	0	1227
Q9P2D1	2559	Chromodomain-helicase-DNA-binding protein 7	CHD7	1	0.00483103	92.575	S	AFEEDIETPPTRNIPSPGQLDPDTRIPVINL	3901600	3686200	6234
Q13428	673	Treacle protein	TCOF1	0.537129	0.000489872	92.551	T	PVRVGTQAPRKAGTATSPAGSSPAVAGGTQR	771900	0	7535
Q96JB3	481	Hypermethylated in cancer 2 protein	HIC2	0.488149	5.61E-07	92.545	S	EEELFIKEEGAYETGSGGAEEEAEDLSAPSA	0	0	4956
Q9UQ35	2397	Serine/arginine repetitive matrix protein 2	SRRM2	0.684676	0.0232377	92.538	T	SPRVPLSAYERVSGRTSPPLLDRARSRTPPS	24881000	0	8278
Q08AD1	611	Calmodulin-regulated spectrin-associated protein 2	CAMSAP2	0.975998	1.43E-07	92.501	S	NQSSPDNVTDTKGALSPITDNTEVDTGIHVP	594170	770530	2255
Q01484	3792	Ankyrin-2	ANK2	0.799273	0.000214228	92.492	S	PGTETSETQKAMIVPSSPSKTPEEVSTPAEE	1559700	0	2064
Q01484	3793	Ankyrin-2	ANK2	0.410524	0.000214228	92.492	S	GTETSETQKAMIVPSSPSKTPEEVSTPAEEE	0	0	2065
Q01484	3797	Ankyrin-2	ANK2	0.868951	0.000214228	92.492	T	SETQKAMIVPSSPSKTPEEVSTPAEEEKLYL	0	705100	7475
O00257	467	E3 SUMO-protein ligase CBX4	CBX4	1	5.82E-06	92.463	S	PPAAALPQPEVILLDSDLDEPIDLRCVKTRS	1930600	1202300	108
Q13905	223	Rap guanine nucleotide exchange factor 1	RAPGEF1	0.896702	6.63E-05	92.428	S	EKQGRPSPTSPVKPSSPASKPDGPAELPLTD	0	0	2633
Q8IZL8	481	Proline-, glutamic acid- and leucine-rich protein 1	PELP1	0.547886	0.0258684	92.427	S	ISPPADALKLRSPRGSPDGSLQTGKPSAPKK	6947100	4214300	4189
O75533	349	Splicing factor 3B subunit 1	SF3B1	0.541735	0.000320157	92.39	S	RKSRWDETPASQMGGSTPVLTPGKTPIGTPA	5147400	0	641
Q8WUB8	59	PHD finger protein 10	PHF10	0.438499	6.01E-05	92.336	T	KRRRMGSGDSSRSCETSSQDLGFSYYPAENL	0	0	7914
Q8WXF7	10	Atlastin-1	ATL1	0.999866	0.0127777	92.295	S	______MAKNRRDRNSWGGFSEKTYEWSSEE	0	0	4593
Q9NQC3	188	Reticulon-4	RTN4	0.941813	9.47E-05	92.29	T	PAAPKRRGSSGSVDETLFALPAASEPVIRSS	0	3603100	8143
Q13371	293	Phosducin-like protein	PDCL	0.796088	0.0064697	92.269	S	EVLVLTSVRNSATCHSEDSDLEID_______	0	0	2478
Q96T37	674	Putative RNA-binding protein 15	RBM15	0.969617	0.00359908	92.265	S	SDRPRKRHCAPSDRSPELSSSRDRYNSDND	65363000	57245000	5157
P33176	933	Kinesin-1 heavy chain	KIF5B	0.985847	0.00250179	92.264	S	AQIAKPIRPGQHPAASPTHPSAIRGGGAFVQ	4144500	2022100	1361
Q9NYF8	284	Bcl-2-associated transcription factor 1	BCLAF1	0.857334	0.00610473	92.264	Y	PERSGSGSVGNGSSRYSPSQNSPIHHIPSRR	372180	97495	8431
Q9NR19	30	Acetyl-coenzyme A synthetase, cytoplasmic	ACSS2	0.99839	0.0028582	92.247	S	RGQEEAGAGGRARSWSPPPEVSRSAHVPSLQ	3788700	1918700	5943
Q14966	1401	Zinc finger protein 638	ZNF638	0.949586	0.0058671	92.239	S	DVSSSKPSIKAVIVSSPKAKATVSKTENQKS	2397400	1599900	2823
Q92834	418	X-linked retinitis pigmentosa GTPase regulator	RPGR	0.999968	0.0058671	92.239	S	QRTLSARMRRRERERSPDSFSMRRTLPPIEG	561860	401750	4721
Q96PV4	153	Paraneoplastic antigen-like protein 5	PNMA5	1	0.0180824	92.239	S	VMPQVRSPPLEPPKESMWYRKLKVFSGTASP	0	0	5062
Q9UEY8	42	Gamma-adducin	ADD3	1	0.0127296	92.239	S	RINENDPEYIRERNMSPDLRQDFNMMEQRKR	1435700	824380	6304
Q8WY36	161	HMG box transcription factor BBX	BBX	0.618009	0.0150345	92.239	T	GYKWCPTTNKPVKSPTPTVNPRKKLWAFPSD	0	756310	7930
Q96PV4	156	Paraneoplastic antigen-like protein 5	PNMA5	1	0.0180824	92.239	Y	QVRSPPLEPPKESMWYRKLKVFSGTASPSPG	0	0	8414
Q7Z6Z7	3824	E3 ubiquitin-protein ligase HUWE1	HUWE1	0.453817	1.38E-06	92.225	S	PMDVDQPSPSAQDTQSIASDGTPQGEKEKEE	0	0	3876
Q7Z6Z7	3822	E3 ubiquitin-protein ligase HUWE1	HUWE1	0.652807	1.38E-06	92.225	T	ESPMDVDQPSPSAQDTQSIASDGTPQGEKEK	0	0	7817
Q9UQ88;P2	273;285	Cyclin-dependent kinase 11A;Cyclin-dependent kinase 11B	CDK11A;CDK	0.585324	0.000321264	92.211	S	MEERDLLSDLQDISDSERKTSSAESSSAESG	1609600	0	1193
Q9BRD0	159	BUD13 homolog	BUD13	1	0.0223808	92.19	T	RHDTPDPSPRRARHDTPDPSPLRGARHDSDT	774970	793630	8043
P49736	26	DNA replication licensing factor MCM2	MCM2	0.930456	0.00240129	92.151	S	ASSPAQRRRGNDPLTSSPGRSSRRTDALTSS	534030	982570	1680
P51812	715	Ribosomal protein S6 kinase alpha-3	RPS6KA3	1	0.00288869	92.151	S	KGAMAATYSALNRNQSPVLEPVGRSTLAQRR	2194700	1442300	1799
Q15555	223	Microtubule-associated protein RP/EB family member 2	MAPRE2	0.657486	0.0167623	92.151	S	SSPAAKPGSTPSRPSSAKRASSSGSASKSDK	0	186900	2946
Q8WYH8	118	Inhibitor of growth protein 5	ING5	0.999675	0.0207853	92.151	S	DLARFEADLKDKMEGSDFESSGGRGLKKGRG	627540	670440	4604
Q15424;Q	235;234	Scaffold attachment factor B1;Scaffold attachment factor B2	SAFB;SAFB2	0.492158	2.73E-05	92.147	S	ILGETCKSEPVKEESSELEQPFAQDTSSVGP	0	0	2921
Q96B49	13	Mitochondrial import receptor subunit TOM6 homolog	TOMM6	0.856687	8.61E-06	92.147	S	___MASSTVPVSAAGSANETPEIPDNVGDWL	0	684620	4802
P47712	437	Cytosolic phospholipase A2;Phospholipase A2;Lysophospholipas	PLA2G4A	0.994753	0.00157922	92.136	S	ENITTKHIVSNDSSDSDDESHEPKGTENEDA	2059000	1689700	1618
Q6P6C2	64	RNA demethylase ALKBH5	ALKBH5	0.994753	0.00105877	92.136	S	EPYPVSGAKRKYQEDSDPERSDYEEQQLQKE	3247600	4607800	3533
Q6P6C2	71	RNA demethylase ALKBH5	ALKBH5	0.727817	0.00131538	92.136	Y	AKRKYQEDSDPERSDYEEQQLQKEEEARKVK	2298600	0	8387
Q5SYE7	1386	NHS-like protein 1	NHSL1	0.999869	0.0002485	92.133	S	VLGRRDSDDDHSRNHSPSPPVTPTGAAPSLA	3002900	1377800	3233
Q5SYE7	1388	NHS-like protein 1	NHSL1	0.999732	0.0002485	92.133	S	GRRDSDDDHSRNHSPSPPVTPTGAAPSLASP	3002900	1377800	3234
Q15596	493	Nuclear receptor coactivator 2	NCOA2	0.999999	0.0029029	92.112	S	NPGQPTSMLSPRHRMSPGVAGSPRIPPSQFS	1631700	899340	2949
Q16760;Q	44;56	Diacylglycerol kinase delta;Diacylglycerol kinase eta	DGKD;DGKH	0.990136	0.0153244	92.112	S	PEAEPGSPQKLIRKVSTSGQIRQKTIIKEGM	563650	310160	3029
Q9BRU9	168	rRNA-processing protein UTP23 homolog	UTP23	0.999926	0.0189575	92.112	S	KTIAFVKAVESGQLVSVHEKESIKHLKEEQG	1688500	1279800	5335
Q9BRU9	174	rRNA-processing protein UTP23 homolog	UTP23	1	0.0189575	92.112	S	KAVESGQLVSVHEKESIKHLKEEQGLVKNTE	1688500	1279800	5336
Q9Y6A5	434	Transforming acidic coiled-coil-containing protein 3	TACC3	0.978329	0.00654461	92.112	S	GGDTKSGCSEAQPPESPETRLGQPAAEQLHA	0	0	6989
Q9ULJ3	343	Zinc finger and BTB domain-containing protein 21	ZBTB21	0.919715	0.0152193	92.096	S	SIDRSGPLVKSLLRRSLMSDQPVYSPSID	1799900	1423200	6479
Q8IZL8	1043	Proline-, glutamic acid- and leucine-rich protein 1	PELP1	1	1.32E-08	92.089	S	PEALPSQGEVEREGESPAAGPPPQELVEEEP	3432800	3398100	4186
O60508	14	Pre-mRNA-processing factor 17	CDC40	0.333922	1.22E-05	92.01	S	__MSAAIAALAASYGSGSGSESDSDSESSRC	0	0	505
O60508	24	Pre-mRNA-processing factor 17	CDC40	0.377893	1.22E-05	92.01	S	AASYGSGSGSESDSDSESSRCPLPAADSLMH	0	0	508
Q6F5E8	991	Leucine-rich repeat-containing protein 16C	RLTPR	0.790015	0.0049867	91.969	S	PGEDAEPQAGPSARGSPSPAAPGPPAGPLPR	2177900	0	3452
Q8NDT2	265	Putative RNA-binding protein 15B	RBM15B	1	0.00246012	91.961	S	LGYLPLHGGYQYKGRSLSPVAAPPLREPRAR	7220400	3085500	4357
Q9UQ35	1854	Serine/arginine repetitive matrix protein 2	SRRM2	0.975662	0.0145617	91.961	S	RGRSRTPPTSRKRSRSRTSPAPWKRSRSRAS	0	25294000	6726
Q9UQ35	1857	Serine/arginine repetitive matrix protein 2	SRRM2	0.996055	0.0145617	91.961	S	SRTPPTSRKRSRSRTSPAPWKRSRSRASPAT	34838000	25294000	6727
Q13905	214	Rap guanine nucleotide exchange factor 1	RAPGEF1	0.643203	7.69E-05	91.942	S	VKELVRLTIEKQGRPSPTSPVKPSSPASKPD	0	0	2630
O94885	1030	SAM and SH3 domain-containing protein 1	SASH1	0.568767	0.0122519	91.932	T	DAPCLPVKRGSPASPTSPSDCPPALAPRPLS	0	811550	7232
O14757	301	Serine/threonine-protein kinase Chk1	CHEK1	0.947658	0.000239141	91.926	S	SPSGFSKHIQSNLDFSPVNSASSEENVKYSS	1269600	1145300	209
P49792	3207	E3 SUMO-protein ligase RanBP2	RANBP2	0.987187	0.00605334	91.906	S	VKDGMDTVKKIESFGSPKGSVCRRITITECG	667040	401920	1709
Q7L1V2	59	Vacuolar fusion protein MON1 homolog B	MON1B	0.999963	0.000179028	91.889	S	EGLEETGSKDKDQPPSPSPPPQSEALSSTSR	1853500	3593700	3720
Q9NPQ8	502	Synembryn-A	RIC8A	1	0.00298422	91.867	S	FDKLSRNRVIQPMGMSPRGHLTSLQDAMCET	615280	398090	5897
Q86XP3	183	ATP-dependent RNA helicase DDX42	DDX42	0.752617	4.25E-07	91.819	Y	PTAGVVQEEEEDNLEYDSDGNPIAPTKKIID	28630000	627480	8393
Q8NEZ4	1307	Histone-lysine N-methyltransferase 2C	KMT2C	0.979453	0.0136547	91.789	S	RTGQGKTKRSVIRKDSSGSISEQLPCRDDGW	375390	235650	4383
Q8NEZ4	1310	Histone-lysine N-methyltransferase 2C	KMT2C	0.84981	0.0136547	91.789	S	QGKTKRSVIRKDSSGSISEQLPCRDDGWSEQ	375390	235650	4384
P62753	235	40S ribosomal protein S6	RPS6	1	0.00861512	91.712	S	AKEKRQEQIAKRRRLSSLRASTSKSESSQK_	893320	805980	1923
P62753	236	40S ribosomal protein S6	RPS6	0.99999	0.00861512	91.712	S	KEKRQEQIAKRRRLSSLRASTSKSESSQK__	893320	805980	1924
P62753	240	40S ribosomal protein S6	RPS6	0.777306	0.00861512	91.712	S	QEQIAKRRRLSSLRASTSKSESSQK______	893320	805980	1925
Q14324	905	Myosin-binding protein C, fast-type	MYBPC2	0.944638	0.02519	91.712	S	TSDFDTVFFVRQAARSDSGEYELSVQIENMK	6045000	12353000	2735
Q14324	907	Myosin-binding protein C, fast-type	MYBPC2	0.963704	0.02519	91.712	S	DFDTVFFVRQAARSDSGEYELSVQIENMKDT	6045000	12353000	2736
Q8TAP8	87	Protein phosphatase 1 regulatory subunit 35	PPP1R35	1	0.026647	91.712	S	AARQRRQVRFRLTPPSPVRSEPQPAVPQELE	945220	726130	4440
Q9UHB7	224	AF4/FMR2 family member 4	AFF4	0.85886	0.00305248	91.712	S	RSKSPRDPDANWDSPSRVPFSSGQHSTQSFP	369050	409440	6337
O14686	654	Histone-lysine N-methyltransferase 2D	KMT2D	1	0.00387531	91.701	S	PEESPMSPPPEVSRLSPLPVVSRLSPPPEES	2452900	1953400	195
Q86UU1	563	Pleckstrin homology-like domain family B member 1	PHLDB1	0.997537	0.025329	91.701	S	TGASPCQSPCVQRKLSSGDLRVPVTRERKNS	486960	0	3937
Q96AY4	47	Tetratricopeptide repeat protein 28	TTC28	1	0.00387531	91.701	S	SAPIPLFGADTIGQRSPDGPVLSKAEFVEKV	0	627740	4786
Q96JM3	432	Chromosome alignment-maintaining phosphoprotein 1	CHAMP1	0.996685	0.00387531	91.701	S	PELRKPGPPLSPEIRSPAGSPELRKPSGSPD	0	336480	4992
Q8N108	121	Mesoderm induction early response protein 1	MIER1	0.499563	3.56E-06	91.66	S	NSGCSGENKEENIKDSSGQEDETQSSNDDPS	0	0	4199
Q14CZ0	167	UPF0472 protein C16orf72	C16orf72	1	0.00619213	91.658	S	PPPRNSRAPPRLTVVSPNRATSTETSSSVET	1147100	687050	2850
P23588	459	Eukaryotic translation initiation factor 4B	EIF4B	0.897468	0.00179094	91.657	S	KSLENETLNKEEDCHSPTSKPPKPDQPLKVM	6175400	3861500	1226
Q659C4	361	La-related protein 1B	LARP1B	0.456775	0.0154838	91.657	S	KNSETSILQAMSRGLSTSLPDLDSEPWIEVK	0	0	3397
Q8TER5	262	Rho guanine nucleotide exchange factor 40	ARHGEF40	0.691892	0.00674112	91.657	S	EVTLPVRGSPTDAEGSPGLSRVRTVPTRKGA	698380	0	4486
Q659C4	362	La-related protein 1B	LARP1B	0.456775	0.0154838	91.657	T	NSETSILQAMSRGLSTSLPDLDSEPWIEVKK	0	0	7744
Q9NRA2	20	Sialin	SLC17A5	0.451903	0.00284579	91.657	T	VRDLARNDGEESTDRTPLLPGAPRAEAAPVC	0	0	8153
Q03164	2172	Histone-lysine N-methyltransferase 2A;MLL cleavage product N	KMT2A	0.65444	1.79E-05	91.643	T	PGCRPLPSAGSPTPTTHEIVTVGDPLLSSGL	0	0	7488
O15164	767	Transcription intermediary factor 1-alpha	TRIM24	0.475045	0.000158136	91.637	S	NANYPRSILTSLLLNSSQSSTSEETVLRSDA	0	0	293
O15164	768	Transcription intermediary factor 1-alpha	TRIM24	0.475038	0.000158136	91.637	S	ANYPRSILTSLLLNSSQSSTSEETVLRSDAP	0	0	294
O15164	770	Transcription intermediary factor 1-alpha	TRIM24	0.433551	0.000158136	91.637	S	YPRSILTSLLLNSSQSSTSEETVLRSDAPDS	0	0	295
O15164	772	Transcription intermediary factor 1-alpha	TRIM24	0.362215	0.000158136	91.637	T	RSILTSLLLNSSQSSTSEETVLRSDAPDSTG	0	0	7155
Q01167	239	Forkhead box protein K2	FOXK2	1	0.0113956	91.622	S	VGRVMPSDLNLMADNSQPENEKEASGGDSPK	0	497010	2062
Q5UIP0	1046	Telomere-associated protein RIF1	RIF1	0.5	0.0039664	91.62	S	SSLKVKGEILLEEEKSTDFVFIPPEGKDAKE	528980	300560	3316
Q7Z4V5	652	Hepatoma-derived growth factor-related protein 2	HDGFRP2	1	0.017877	91.62	S	LHDSVREGPDLDRPGSDRQERERARGDSEAL	1757400	2268300	3830
Q14676	1280	Mediator of DNA damage checkpoint protein 1	MDC1	1	0.0216045	91.62	T	TYQATRGRKNRSSVKTPEPVVPTAPELRPST	340470	296810	7610
Q5UIP0	1047	Telomere-associated protein RIF1	RIF1	0.5	0.0039664	91.62	T	SLKVKGEILLEEKSTDFVFIPPEGKDAKER	528980	300560	7729
P20810	237	Calpastatin	CAST	0.512444	1.65E-08	91.592	S	SSKPIGPDDAIDALSSDFTCGSPTAAGKKTE	0	0	1189

P25106	145	Atypical chemokine receptor 3	ACKR3	0.723951	0.00350592	91.584	S	GSIFFLTCMSVDRYLSITYFTNTPSSRKKMV	1133900	1236700	1264
P25106	154	Atypical chemokine receptor 3	ACKR3	0.654078	0.00350592	91.584	S	SVDRYLSITYFTNTPSSRKKMVRRVVCILVW	1409100	1633700	1265
P25106	155	Atypical chemokine receptor 3	ACKR3	0.654077	0.00350592	91.584	S	VDRYLSITYFTNTPSSRKKMVRRVVCILVWL	1409100	1633700	1266
P25106	147	Atypical chemokine receptor 3	ACKR3	0.627812	0.00350592	91.584	T	IFFLTCMSVDRYLSITYFTNTPSSRKKMVRR	357980	448130	7326
P25106	150	Atypical chemokine receptor 3	ACKR3	0.370652	0.00350592	91.584	T	LTCMSVDRYLSITYFTNTPSSRKKMVRRVVC	0	0	7327
P25106	152	Atypical chemokine receptor 3	ACKR3	0.786788	0.00350592	91.584	T	CMSVDRYLSITYFTNTPSSRKKMVRRVVCIL	1409100	1633700	7328
Q9Y3Z3	23	Deoxynucleoside triphosphate triphosphohydrolase SAMHD1	SAMHD1	0.573705	0.00020611	91.556	S	QPSKRPRCDDSPRTPSNTPSAEADWSPGLEL	857870	764170	6885
Q9Y3Z3	21	Deoxynucleoside triphosphate triphosphohydrolase SAMHD1	SAMHD1	0.825336	0.00020611	91.556	T	SEQPSKRPRCDDSPRTPSNTPSAEADWSPGL	10572000	764170	8298
Q9Y3Z3	25	Deoxynucleoside triphosphate triphosphohydrolase SAMHD1	SAMHD1	0.585477	0.00020611	91.556	T	SKRPRCDDSPRTPSNTPSAEADWSPGLELHP	857870	764170	8299
Q6PKG0	777	La-related protein 1	LARP1	0.499903	0.00533362	91.549	Y	TIARSLPTTVPESPNYRNTRTPRTPRTPQLK	0	0	8389
Q9Y6A5	317	Transforming acidic coiled-coil-containing protein 3	TACC3	0.867322	0.000362988	91.547	S	TAPTNHLVAGRAMTLSPQEEVAAGQMASSSR	796170	936060	6986
O94826	91	Mitochondrial import receptor subunit TOM70	TOMM70A	0.999566	5.71E-05	91.541	S	GLKRNSERKTPEGRASPAPGSGHPEGPGAHL	26049000	21025000	701
P67809	167	Nuclease-sensitive element-binding protein 1	YBX1	0.33257	9.11E-06	91.541	S	RGPPRNYQQNYQNSESGEKNEGSESAPEGQA	0	0	1938
P67809	162	Nuclease-sensitive element-binding protein 1	YBX1	0.33257	9.11E-06	91.541	Y	RYPRRRGPPRNYQQNYQNSESGEKNEGSESA	0	0	8370
O15068	973	Guanine nucleotide exchange factor DBS	MCF2L	0.2	0.000572283	91.518	S	RALEQSQSLPLPAPTSTSPSRGNSRNIKKLE	0	0	275
O15068	977	Guanine nucleotide exchange factor DBS	MCF2L	0.493773	0.000572283	91.518	S	QSQSLPLPAPTSTSPSRGNSRNIKKLEERKT	0	0	277
O15068	972	Guanine nucleotide exchange factor DBS	MCF2L	0.2	0.000572283	91.518	T	HRALEQSQSLPLPAPTSTSPSRGNSRNIKKL	0	0	7150
O15068	974	Guanine nucleotide exchange factor DBS	MCF2L	0.282974	0.000572283	91.518	T	ALEQSQSLPLPAPTSTSPSRGNSRNIKKLEE	0	0	7151
Q9NY61	316	Protein AATF	AATF	0.999858	5.40E-05	91.481	S	PDTRYLVDGTKPNAGSEEISSEDDELVEEKK	21942000	12938000	6089
Q9UHB7	703	AF4/FMR2 family member 4	AFF4	0.999999	0.00208849	91.475	S	FRQRMFSPMEEKELLSPLSEPDDRYPLIVKI	1608000	1017200	6338
Q9UHB7	706	AF4/FMR2 family member 4	AFF4	0.999965	0.00208849	91.475	S	RMFSPMEEKELLSPLSEPDDRYPLIVKIDLN	1608000	1017200	6339
Q9UNN5	582	FAS-associated factor 1	FAF1	0.99948	0.00395637	91.469	S	EENAEPVSKLRIRTPSGEFLERRFLASNKLQ	756980	663850	6548
Q9UQ35	1732	Serine/arginine repetitive matrix protein 2	SRRM2	0.59254	0.00405005	91.441	S	RSRTPPRHRRSPSVSSPEPAEKSRSSRRRRS	1465400	0	6703
O60343	759	TBC1 domain family member 4	TBC1D4	0.826125	0.00103362	91.401	S	EGRKRTSSTCSNESLSVGGTSVTPRRISWRQ	1403100	0	500
Q9BXK1	109	Krueppel-like factor 16	KLF16	0.64489	2.62E-05	91.378	S	APGGASPASSSSAASSPSSGRAPGAAPSAAA	1697100	0	5454
Q8TD19	868	Serine/threonine-protein kinase Nek9	NEK9	0.978415	0.00105952	91.355	S	VASEAPLEHKPQVEASSPRLNPAVTCAGKGT	0	4141300	4466
Q13595	18	Transformer-2 protein homolog alpha	TRA2A	0.521553	0.0281138	91.307	S	DVEENNFEGRESRSQSKSPTGTPARVKSESR	248740	765800	2593
Q13595	20	Transformer-2 protein homolog alpha	TRA2A	0.844716	0.0281138	91.307	S	EENNFEGRESRSQSKSPTGTPARVKSESRSG	248740	765800	2594
Q8TBE0	184	Bromo adjacent homology domain-containing 1 protein	BAHD1	1	0.00545234	91.307	S	KRPRLGDLGGGSRDLSPEPAPDEGPRRDGDP	0	809420	4452
Q96SB3	190	Neurabin-2	PPP1R9B	0.988424	0.00316995	91.307	S	GLQDRKLDVVVRFNGSTEALDKLDADAVSPT	1013000	629990	5120
Q9H4A4	7	Aminopeptidase B	RNPEP	0.998378	0.00232223	91.307	S	__________MASGEHSPGSGAARRPLHSAQA	0	3077700	5669
P98174	114	FYVE, RhoGEF and PH domain-containing protein 1	FGD1	0.712901	0.000837534	91.276	S	QPRPGLHGQNRILVKSLSLDPGQSLEPHPEG	320230	869060	2012
P06748	254	Nucleophosmin	NPM1	1	0.0132835	91.265	S	KGPSSVEDIKAKMQASIEKGGSLPKVEAKFI	1351600	1306800	923
P78559	2238	Microtubule-associated protein 1A;MAP1A heavy chain;MAP1 li	MAP1A	0.96458	1.84E-05	91.238	S	APSLDSSLPQLPSPSSPGAPLLSNLPRPASP	0	2977200	1974
Q14153	268	Protein FAM53B	FAM53B	1	0.00404969	91.202	S	TPELARRSSGLSRSRSQPCVLNDKKVGVKRR	0	465340	2655
Q92615	601	La-related protein 4B	LARP4B	1	0.00416126	91.202	S	VPASCAVSATYERSPSPAHLPDDPKVAEKQR	5622200	3097500	4677
Q14683	962	Structural maintenance of chromosomes protein 1A	SMC1A	0.474979	6.79E-05	91.166	S	MDDISQEEGSSQGEDSVSGSQRISSIYAREA	0	0	2779
P38398	1524	Breast cancer type 1 susceptibility protein	BRCA1	0.999846	0.00323094	91.123	S	YMHSCSGSLQNRNYPSQEELIKVVDVEEQQL	2057400	1496100	1455
Q14498	125	RNA-binding protein 39	RBM39	1	0.018322	91.123	S	GKIGLPHSIKLSRRRSRSKSPFRKDKSPVRE	1666400	1788700	2738
Q14498	127	RNA-binding protein 39	RBM39	1	0.018322	91.123	S	IGLPHSIKLSRRRSRSKSPFRKDKSPVREPI	1666400	1788700	2739
Q14498	129	RNA-binding protein 39	RBM39	1	0.018322	91.123	S	LPHSIKLSRRRSRSKSPFRKDKSPVREPIDN	1666400	1788700	2740
O95714	1942	E3 ubiquitin-protein ligase HERC2	HERC2	0.725699	1.30E-05	91.114	S	KLAELPAAAQPSAEDSDTEDDSEAEQTERNI	3009800	0	803
Q6WKZ4	528	Rab11 family-interacting protein 1	RAB11FIP1	0.709459	0.0147957	91.093	S	PEAEPESKSEPRPPISSPRAPQTRAVKPRLE	0	0	3640
Q9H2H9	56	Sodium-coupled neutral amino acid transporter 1	SLC38A1	0.999904	0.00339791	91.093	S	SKFISDRESRRSLTNSHLEKKKCDEYIPGTT	0	635950	5615
Q9NRR6	553	72 kDa inositol polyphosphate 5-phosphatase	INPP5E	0.499801	0.0101882	91.093	S	IGKDTYDSTSKQRTPSYTDRVLYRSRHKGDI	0	0	5981
Q9NRR6	555	72 kDa inositol polyphosphate 5-phosphatase	INPP5E	0.499801	0.0101882	91.093	T	KDTYDSTSKQRTPSYTDRVLYRSRHKGDICP	0	0	8160
Q9NRR6	560	72 kDa inositol polyphosphate 5-phosphatase	INPP5E	0.999958	0.0101882	91.093	Y	STSKQRTPSYTDRVLYRSRHKGDICPVSYSS	0	0	8428
P49792;Q[illegible]2293;131[illegible]		E3 SUMO-protein ligase RanBP2;RanBP2-like and GRIP domain-c	RANBP2;RGPI	0.863863	0.000253914	91.091	T	SKSPAKLNQSGTSVGTDEESDVTQEEERDGQ	419800	987930	7423
Q9BSJ8	1034	Extended synaptotagmin-1	ESYT1	0.999984	0.00408978	91.087	S	NRGTKRRTSQKKRTLSPEFNERFEWELPLDE	4150100	3189500	5343
O43934	204	UNC93-like protein MFSD11	MFSD11	0.838058	8.49E-07	91.086	S	LIRKPDSENVLGEDESSDDQDMEVNESAQNN	0	0	434
Q5VT52	479	Regulation of nuclear pre-mRNA domain-containing protein 2	RPRD2	0.99231	5.34E-05	91.086	S	TSVMKNTGVSPASRPSPGTPTSPSNLTSGLK	1978700	1308400	3333
Q5VT52	482	Regulation of nuclear pre-mRNA domain-containing protein 2	RPRD2	0.997527	0.000104672	91.086	T	MKNTGVSPASRPSPGTPTSPSNLTSGLKTPA	1294600	1308400	7731
Q13541	44	Eukaryotic translation initiation factor 4E-binding protein	EIF4EBP1	0.410047	7.94E-07	91.079	S	LPPGDYSTTPGGTLFSTTPGGTRIIYDRKFL	0	0	2573
O75694	994	Nuclear pore complex protein Nup155	NUP155	0.733559	0.00324903	91.069	S	LQELVNQSKAAPQSPSVPKKPGPPVLSSDPN	0	4991100	656
Q15052	52	Rho guanine nucleotide exchange factor 6	ARHGEF6	1	0.00656874	90.986	S	NGVVLCKLINRLMPGSVEKFCLDPQTEADCI	0	0	2868
O94806	30	Serine/threonine-protein kinase D3	PRKD3	0.581081	0.000461924	90.972	S	LPTAIPAVLPAASPCSSPKTGLSARLSNGSF	0	561130	694
Q9Y5S9	56	RNA-binding protein 8A	RBM8A	0.99988	0.00154961	90.968	S	GSEEGSRARMREDYDSVEQDGDEPGPQRSVE	0	0	6967
O43583	73	Density-regulated protein	DENR	0.999999	0.00660935	90.913	S	EKNFPNEFAKLTVENSPKQEAGISEGQGTAG	6796100	4885900	402
P10636	428	Microtubule-associated protein tau	MAPT	0.982355	1.99E-05	90.912	S	KHPTPGSSDPLIQPSSPAVCPEPPSSPKYVS	2660700	1514900	993
P11171	144	Protein 4.1	EPB41	0.49833	9.17E-05	90.907	S	KAPIAAPEPELKTDPSLDLHSLSSAETQPAQ	0	0	1009
P11171	151	Protein 4.1	EPB41	0.515705	9.17E-05	90.907	S	EPELKTDPSLDLHSLSSAETQPAQEELREDP	1081700	815500	1010
P39880	1233	Homeobox protein cut-like 1	CUX1	0.721156	4.51E-07	90.907	S	SDSQPCEPPSVGTEYSQGASPQPQHQLKKPR	3748600	1597800	1466
Q9UKV3	682	Apoptotic chromatin condensation inducer in the nucleus	ACIN1	0.995527	0.000261624	90.907	T	SYTETKDPSSGQEVATPPVPQLQVCEPKERT	1310900	1897100	8235
P35612	592	Beta-adducin	ADD2	0.999282	0.000139692	90.882	S	LELDGEKETAPEEPGSPAKSAPASPVQSPAK	0	1206000	1409
Q96ST2	17	Protein IWS1 homolog	IWS1	0.999639	0.0013349	90.882	T	DSEYYSGDQSDDGGATPVQDERDSGSDGEDD	0	0	8008
O43815	137	Striatin	STRN	0.656474	2.36E-08	90.858	S	GTELNQGDMKPPSYDSDEGNETEVQPQQNSQ	0	1174700	425
O00488	133	Zinc finger protein 593	ZNF593	0.400339	0.00355502	90.853	S	AVPTEVSTEVPEMDTST______________	0	0	121
P17252	226	Protein kinase C alpha type	PRKCA	0.965627	0.0180416	90.853	S	KTKTIRSTLNPQWNESFTFKLKPSDKDRRLS	567340	0	1108
O00488	134	Zinc finger protein 593	ZNF593	0.404456	0.00355502	90.853	T	VPTEVSTEVPEMDTST_______________	0	0	7121
P29374	863	AT-rich interactive domain-containing protein 4A	ARID4A	0.695702	0.00418069	90.827	S	QCVKEKKLKRKILGQSSPEKKIRIENGMEMT	1669300	1079900	1315
P29374	864	AT-rich interactive domain-containing protein 4A	ARID4A	0.5	0.00418069	90.827	S	CVKEKKLKRKILGQSSPEKKIRIENGMEMTN	1669300	0	1316
Q9UQ35	2398	Serine/arginine repetitive matrix protein 2	SRRM2	0.960717	0.00665754	90.827	S	PRVPLSAYERVSGRTSPPLLDRARSRTPPSA	0	18392000	6748
P50395	61	Rab GDP dissociation inhibitor beta	GDI2	0.999544	0.00339474	90.793	S	ITPLEDLYKRFKIPGSPPESMGRGRDWNVDL	2057700	1110100	1745
Q9UQ35	2426	Serine/arginine repetitive matrix protein 2	SRRM2	0.999962	0.0135614	90.777	S	PSAPSQSRMTSERAPSPSSRMGQAPSQSLLP	0	1785000	6601
P24928	1850	DNA-directed RNA polymerase II subunit RPB1	POLR2A	0.349237	0.00047526	90.765	S	ASPKYTPTSPSYSPSSPEYTPTSPKYSPTSP	0	0	1257
Q7KZI7	571	Serine/threonine-protein kinase MARK2	MARK2	0.994929	0.00878103	90.765	S	PRPSTAPQRVPVASPSAHNISSSGGAPDRTN	0	0	3716
O00178	44	GTP-binding protein 1	GTPBP1	0.959485	1.91E-05	90.762	S	ARAAAAAARLHGGFDSDCSEDGEALNGEPEL	4020900	2375800	98
Q8WVT3	182	Trafficking protein particle complex subunit 12	TRAPPC12	0.996702	0.00572084	90.759	S	APPASGDGFEPQMVKSPSFGGASEASARTPP	670750	396770	4544
P52701	43	DNA mismatch repair protein Msh6	MSH6	0.955937	4.86E-05	90.758	S	SREGGRAAAAPGASPSPGGDAAWSEAGPGPR	1115700	651800	1816
Q12756	1528	Kinesin-like protein KIF1A	KIF1A	0.993106	0.000212757	90.757	S	EGRYGATDLRTPQPCSRPASPEPELLPEADS	2505200	1357800	2289
Q12756	1532	Kinesin-like protein KIF1A	KIF1A	0.99992	0.000212757	90.757	S	GATDLRTPQPCSRPASPEPELLPEADSKKLP	2505200	1357800	2290
P38432	566	Coilin	COIL	0.866555	0.00598836	90.725	S	TVFWKELIDPRLIIESPSNTSSTEPA______	0	1501000	1457
P38432	571	Coilin	COIL	0.530752	0.00598836	90.725	S	ELIDPRLIIESPSNTSSTEPA__________	0	1501000	1458
Q5W0B1	551	RING finger protein 219	RNF219	0.776849	0.000112998	90.718	S	DKISELDSMMSESDNSKSPCNNGFKSLDLDG	2984600	0	3374
Q86VM9	13	Zinc finger CCCH domain-containing protein 18	ZC3H18	1	0.000307035	90.718	S	___MDVAESPERDPHSPEDEEQPQGLSDDDI	0	0	3947
Q86VM9	24	Zinc finger CCCH domain-containing protein 18	ZC3H18	1	0.000307035	90.718	S	RDPHSPEDEEQPQGLSDDDILRDSGSDQDLD	0	544640	3948
Q96GX9	87	Methylthioribulose-1-phosphate dehydratase	APIP	0.961953	0.00422197	90.709	S	DMFVCDINEKDISGPSPSKKLKKSQCTPLFM	1338500	1388600	4928
Q00537	137	Cyclin-dependent kinase 17	CDK17	1	0.017354	90.697	S	SPTGVCLRNRIHRRISMEDLNKRLSLPADIR	0	0	2023
Q8N6N3	158	UPF0690 protein C1orf52	C1orf52	0.991904	0.00721733	90.697	S	AKKARLLPEGEETLESDDEKDEHTSKKRKVE	215650	323000	4292
Q15424	188	Scaffold attachment factor B1	SAFB	0.902613	7.01E-06	90.694	T	ELPEQLQEHAIEDKETINNLDTSSSDFTILQ	0	0	7640
Q9UQ35	992	Serine/arginine repetitive matrix protein 2	SRRM2	0.778857	0.00443534	90.614	S	TKVKPETPPRQSHSGSISPYPKVKAQTPPGP	2226100	2101900	6675
P12277	4	Creatine kinase B-type	CKB	0.995946	0.00340419	90.601	S	____________MPFSNSHNALKLRFPAEDE	930040	494460	1041
Q9P206	545	Uncharacterized protein KIAA1522	KIAA1522	1	0.00346624	90.601	S	GTPTLPPKGLAGPPASPGKAQPPKPERVTSL	6067000	3555500	6211
Q9P260	453	LisH domain and HEAT repeat-containing protein KIAA1468	KIAA1468	0.96111	0.00425968	90.601	S	LSISDEADSTIPKENSPNSFPRREREGMPPS	1322400	559630	6220
O15446	490	DNA-directed RNA polymerase I subunit RPA34	CD3EAP	0.813093	2.00E-05	90.545	S	TVPQEEMPGPPLNSESGEEAPTGRDKKRKQQ	2938100	2296900	322
Q9P260	54	LisH domain and HEAT repeat-containing protein KIAA1468	KIAA1468	0.600509	1.58E-05	90.518	S	LRLGAGSGLDPGSAGSLSPQDPVALGSSARP	1225500	0	6222
Q9P260	56	LisH domain and HEAT repeat-containing protein KIAA1468	KIAA1468	0.989111	1.58E-05	90.518	S	LGAGSGLDPGSAGSLSPQDPVALGSSARRPGL	1225500	1393800	6223
Q9UQ35	317	Serine/arginine repetitive matrix protein 2	SRRM2	0.29465	3.98E-05	90.464	S	RRGEGDAPFSEPGTTSTQRPSSPETATKQPS	0	0	6629
Q9UQ35	318	Serine/arginine repetitive matrix protein 2	SRRM2	0.295165	3.98E-05	90.464	T	RGEGDAPFSEPGTTSTQRPSSPETATKQPSS	0	0	8259
Q6ZN18	210	Zinc finger protein AEBP2	AEBP2	1	0.00962627	90.442	S	GSSATSGGRRGSLEMSSDGEPLSRMDSEDSI	804740	518990	3652
O95801	243	Tetratricopeptide repeat protein 4	TTC4	0.857466	3.75E-07	90.397	S	ARNIRLSEAACEDEDSASEGLGELFLDGLST	1549400	1026500	822
O95071	1551	E3 ubiquitin-protein ligase UBR5	UBR5	0.723657	0.00691348	90.37	S	SSYIIRNPQQRRISQSQPVRGRDEEQDDIVS	0	1173600	727
Q7Z5J4	1374	Retinoic acid-induced protein 1	RAI1	1	0.0068851	90.316	S	PSLSDKDRGLKGAGGSPVGVEEGLVNVGTGQ	0	476900	3839
Q9UPU5	2602	Ubiquitin carboxyl-terminal hydrolase 24	USP24	0.573591	0.00333446	90.316	S	SSPANENGDRHLQQGSESPMMIGELRSDLDD	782470	760550	6581
Q13033	257	Striatin-3	STRN3	0.99997	2.55E-07	90.299	S	GDVLETFNFLENADDSDEDENDMIEGIPEG	16453000	5511100	2392
Q5SY16	90	Polynucleotide 5'-hydroxyl-kinase NOL9	NOL9	0.651501	9.14E-07	90.299	T	ARRPNTATPSPIPSPTPASEPESEPELESAS	1868500	900180	7716
P78559	1818	Microtubule-associated protein 1A;MAP1A heavy chain;MAP1 li	MAP1A	0.999641	0.00742566	90.259	S	PEMVGQRVPSAPGQESPIPDPKLMPHMKNEP	1999900	1591600	1983
P48730	347	Casein kinase I isoform delta	CSNK1D	0.935954	0.000127278	90.22	T	GRLRGTQEVAPPTPLTPTSHTANTSPRPVSG	0	1056200	7404
P48730	349	Casein kinase I isoform delta	CSNK1D	0.631854	0.000127278	90.22	T	LRGTQEVAPPTPLTPTSHTANTSPRPVSGME	0	1056200	7405
Q9NZJ0	444	Denticleless protein homolog	DTL	0.456206	3.89E-07	90.211	S	TPAKAPRAKCNPSNSSPSSAACAPSCAGDLP	0	0	6153
Q9NZJ0	446	Denticleless protein homolog	DTL	0.456206	3.89E-07	90.211	S	AKAPRAKCNPSNSSPSSAACAPSCAGDLPLP	0	0	6154
Q86VM9	595	Zinc finger CCCH domain-containing protein 18	ZC3H18	0.992526	0.0070372	90.149	S	SSYSSRSSRHSSFSGSRSRSRSFSSSPSPSP	584270	182870	3949
Q96Q42	466	Alsin	ALS2	0.556205	0.0070372	90.149	S	EEQVKQESMQGKKSSSLVDIREEETEGGSRR	771820	0	5071
Q9H4L7	34	SWI/SNF-related matrix-associated actin-dependent regulator	SMARCAD1	0.77347	1.47E-07	90.136	S	EAPEATPQPSQPGPSSPISLSAEEENAEGEV	1323500	6059000	5690
Q15642	502	Cdc42-interacting protein 4	TRIP10	0.263947	6.99E-05	90.127	S	ARPPDPPASAPPDSSSNSASQDTKESSEEPP	0	0	2958
Q15642	504	Cdc42-interacting protein 4	TRIP10	0.263947	6.99E-05	90.127	S	PPDPPASAPPDSSSNSASQDTKESSEEPPSE	0	0	2959
Q15642	506	Cdc42-interacting protein 4	TRIP10	0.263947	6.99E-05	90.127	S	DPPASAPPDSSSNSASQDTKESSEEPPSEES	0	0	2960
Q9NR30	171	Nucleolar RNA helicase 2	DDX21	0.965821	3.96E-07	90.127	S	HPEPDCNPSEAASEESNSEIEQEIPVEQKEG	3854200	1846600	5948
P04792	15	Heat shock protein beta-1	HSPB1	1	0.00705993	90.108	S	_MTERRVPFSLLRGPSWDPFRDWYPHSRLFD	564120	285480	886
O95714	1938	E3 ubiquitin-protein ligase HERC2	HERC2	0.578054	1.60E-05	90.097	S	KYDLKLAELPAAAQPSAEDSDTEDDSEAEQT	0	0	802
Q9H9L4	147	KAT8 regulatory NSL complex subunit 2	KANSL2	0.999999	0.000194635	90.062	S	PESSRSEASRILDEDSWSDGEQEPITVDQTW	719290	714290	5806
P49454	2900	Centromere protein F	CENPF	1	0.000520736	90.06	S	VAHLCSQQSKQDSRGSPLLGPVVPGPSPIPS	1729500	984080	1661
P49454	2911	Centromere protein F	CENPF	0.997916	0.000520736	90.06	S	DSRGSPLLGPVVPGPSPIPSVTEKRLSSGQN	1377200	693390	1662
Q96I25	224	Splicing factor 45	RBM17	0.543881	0.000890449	89.992	T	IPPPVYEEQDRPRSPTGPSNSFLANMGGTVA	0	988350	7984
P49841	9	Glycogen synthase kinase-3 beta	GSK3B	0.773018	0.000402118	89.946	S	__________MSGRPRTTSFAESCKPVQQPSAFG	2168900	0	1736
Q15398	806	Disks large-associated protein 5	DLGAP5	0.956225	6.09E-05	89.946	S	LFDNKSLTTECHLLDSPGLNCSNPFTQLERR	960400	1162900	2912
Q15398	812	Disks large-associated protein 5	DLGAP5	0.995459	6.09E-05	89.946	S	LTTECHLLDSPGLNCSNPFTQLERRHQEHAR	960400	1162900	2913
P56524	632	Histone deacetylase 4	HDAC4	0.983538	0.000246011	89.923	S	IPVSFGGHRPLSRAQSSPASATFPVSVQEPP	1399900	1261300	1895
Q6P0Q8	151	Microtubule-associated serine/threonine-protein kinase 2	MAST2	0.787167	0.00246755	89.911	S	NLVRMRNQSLGQSAPSLTAGLKELSLPRRGS	0	397870	3504
Q9H792	1250	Pseudopodium-enriched atypical kinase 1	PEAK1	0.990707	0.0037233	89.911	S	ISSLTTLSIKDRFSNSMESLSSRRGPSCRQG	379260	428040	5751
P05026	19	Sodium/potassium-transporting ATPase subunit beta-1	ATP1B1	1	0.029463	89.903	S	GKAKEEGSWKKFIWNSEKKEFLGRTGGSWFK	1490700	1945000	893
A1L390	76	Pleckstrin homology domain-containing family G member 3	PLEKHG3	0.99999	0.00450442	89.9	S	SNNNSSSWLNVKGPLSPFNSRAAAGPAHHKL	1012400	410450	17
P46379	978	Large proline-rich protein BAG6	BAG6	0.672533	0.00457173	89.866	T	ASPEPQRENASPAPGTTAEEAMSRGPPPAPE	0	2653400	7386
P22059	379	Oxysterol-binding protein 1	OSBP	0.56468	0.0128812	89.831	S	EIITMPENLGHKRTGSNISGASSDISLDEQY	482850	617770	1206
P22059	382	Oxysterol-binding protein 1	OSBP	0.688292	0.0128812	89.831	S	TMPENLGHKRTGSNISGASSDISLDEQYKHQ	482850	617770	1207
Q8N9T8	639	Protein KRI1 homolog	KRI1	1	1.80E-05	89.829	S	MGPESPPAQEEEAPVSPHKKPAPQKRRRAKK	1808600	1291600	4307
A7E2V4	1156	Zinc finger SWIM domain-containing protein 8	ZSWIM8	0.715931	8.07E-05	89.816	S	MASIDSSAPETTSDSSPTLSRRPLRGGWAPT	2671600	1572400	45
Q86UU1	518	Pleckstrin homology-like domain family B member 1	PHLDB1	0.933843	0.0137729	89.816	S	EDFSLTLGARGRTRSPSPTLGESLAPHKGS	1149200	660230	3938
Q9UQ35	854	Serine/arginine repetitive matrix protein 2	SRRM2	0.539775	0.0259184	89.816	S	SPHPKVKSGTPPRQGSITSPQANEQSVTPQR	0	0	6667

Q86UU1	522	Pleckstrin homology-like domain family B member 1	PHLDB1	0.962606	0.0128733	89.816	T	LTLGARGRRTRSPSPTLGESLAPHKGSFSGR	1149200	660230	7825
Q13057	183	Bifunctional coenzyme A synthase;Phosphopantetheine adenyly	COASY	0.999614	0.00366838	89.805	S	VPLPSTIRPASPVAGSPKQPVRGYYRGAVGG	875520	545600	2395
Q9H6S0	1221	Probable ATP-dependent RNA helicase YTHDC2	YTHDC2	0.942838	0.00376288	89.805	S	EFSDECTTAERVLMKSPSPALHPPQKYKDRG	1570400	857870	5738
Q9Y6A5	71	Transforming acidic coiled-coil-containing protein 3	TACC3	0.990321	0.00453786	89.805	S	TFQTPLRDPQTHRILSPSMASKLEAPFTQDD	841450	857530	6988
Q9Y3B9	45	RRP15-like protein	RRP15	0.660076	1.69E-05	89.803	S	AVASVLEDEATDTSDSEGSCGSEKDHFYSDD	1256300	584390	6851
Q14676	1702	Mediator of DNA damage checkpoint protein 1	MDC1	0.957993	1.58E-06	89.773	S	STVRAMPVPTTPEFQSPVTTDQPISPEPITQ	0	1334600	2760
Q14676	1711	Mediator of DNA damage checkpoint protein 1	MDC1	0.99579	1.58E-06	89.773	S	TTPEFQSPVTTDQPISPEPITQPSCIKRQRA	2006600	2029700	2761
Q14676	1696	Mediator of DNA damage checkpoint protein 1	MDC1	0.777994	1.58E-06	89.773	T	SKTLRSSTVRAMPVPTTPEFQSPVTTDQPIS	2006600	2029700	7598
Q14676	1697	Mediator of DNA damage checkpoint protein 1	MDC1	0.777999	1.58E-06	89.773	T	KTLRSSTVRAMPVPTTPEFQSPVTTDQPISP	1715600	1334600	7599
Q8IYB3	636	Serine/arginine repetitive matrix protein 1	SRRM1	1	0.0186841	89.752	S	PKRRASPSPPPKRRVSHSPPPKQRSSPVTKR	712950	1495300	4163
Q8IYB3	638	Serine/arginine repetitive matrix protein 1	SRRM1	1	0.0186841	89.752	S	RRASPSPPPKRRVSHSPPPKQRSSPVTKRRS	712950	1495300	4164
Q8NDF8	49	Non-canonical poly(A) RNA polymerase PAPD5	PAPD5	0.494842	2.33E-08	89.752	S	HQPGAWARRAGSSASSPPSASSSPHPSAAVP	0	0	4348
O43290	591	U4/U6.U5 tri-snRNP-associated protein 1	SART1	0.765774	5.85E-05	89.751	S	EEQEELMDFERDEERSANGGSESDGEENIGW	1363100	0	351
E9PAV3	2024	Nascent polypeptide-associated complex subunit alpha, muscle	NACA	0.794692	6.71E-07	89.751	T	QGEAVSNIQENTQTPTVQEESEEEEVDETGV	834540	14127000	7117
P38919	163	Eukaryotic initiation factor 4A-III;Eukaryotic initiation fa	EIF4A3	0.999999	0.00597664	89.747	T	EDIRKLDYGQHVVAGTPGRVFDMIRRRSLRT	1293900	668350	7374
Q13596	32	Sorting nexin-1	SNX1	0.99967	6.32E-09	89.742	S	PFPGLEPESEGAAGGSEPEAGDSDTEGEDIF	7793600	5557200	2598
Q13596	39	Sorting nexin-1	SNX1	0.996939	6.32E-09	89.742	S	ESEGAAGGSEPEAGDSDTEGEDIFTGAAVVS	2922700	5557200	2599
Q13596	41	Sorting nexin-1	SNX1	0.996944	6.32E-09	89.742	T	EGAAGGSEPEAGDSDTEGEDIFTGAAVVSKH	2922700	5557200	7567
Q96S59	483	Ran-binding protein 9	RANBP9	0.868188	0.000213184	89.721	S	PKSQDSYPVSPRPFSSPSMSPSHGMNIHNLA	6290600	11504000	5112
Q96S59	485	Ran-binding protein 9	RANBP9	0.624768	0.000213184	89.721	S	SQDSYPVSPRPFSSPSMSPSHGMNIHNLASG	6290600	257850	5113
P48634	1219	Protein PRRC2A	PRRC2A	1	0.00380258	89.698	S	PSKEPLKEKLIPGPLSPVARGGSNGGSNVGM	6180700	5155100	1626
Q68E01	995	Integrator complex subunit 3	INTS3	0.841355	0.014175	89.698	S	SSTKPPKSRRKAALSSPRSRKNATQPPNAEE	0	0	3424
Q07866	460	Kinesin light chain 1	KLC1	0.999396	0.00381559	89.663	S	SFGEYGGWYKACKVDSPTVTTTLKNLGALYR	3023300	1905900	2240
P49792;Q896;897;8		E3 SUMO-protein ligase RanBP2;RanBP2-like and GRIP domain-co	RANBP2;RGPI	0.862833	0.0187033	89.663	T	PAYNSQYLLRPAANVTPTKGPVYGMNRLPPQ	0	487860	7424
Q02040	537	A-kinase anchor protein 17A	AKAP17A	1	0.0157197	89.629	S	CKEVQSSCRVVPEDGSPEKRCPGGVLSCIPD	0	1170400	2090
Q9UQ35	2067	Serine/arginine repetitive matrix protein 2	SRRM2	1	0.0304255	89.629	S	RRSRSRTPRTARGKRSLTRSPPAIRRRSASG	4740500	5936900	6698
O75962	2455	Triple functional domain protein	TRIO	0.999623	0.00972862	89.604	S	SLGTLPLGKPRAGAASPLNSPLSSAVPSLGK	916790	0	672
O75962	2459	Triple functional domain protein	TRIO	0.628059	0.00972862	89.604	S	LPLGKPRAGAASPLNSPLSSAVPSLGKEPFP	916790	0	673
Q9UPN4	96	Centrosomal protein of 131 kDa	CEP131	0.937008	0.000405192	89.583	T	QVSQPRSGSPRPTEPTDFLMLFEGSPSGKKR	0	637010	8247
Q07157	294	Tight junction protein ZO-1	TJP1	0.8264	0.00377222	89.557	S	HSANASERDDISEIQSLASDHSGRSHDRPPR	0	595720	2220
Q7L2J0	152	7SK snRNA methylphosphate capping enzyme	MEPCE	1	0.0063341	89.507	S	QPHRPPGGGGGKRRNSCNVGGGGGGFKHPAF	1895400	1718600	3726
Q96AE4	629	Far upstream element-binding protein 1	FUBP1	0.557919	0.000133353	89.507	T	AWAEYYRQQAAYYAQTSPQGMPQHPPAPQGQ	0	4318100	7966
O00499	303	Myc box-dependent-interacting protein 1	BIN1	0.993854	0.0020874	89.502	S	NAPAKGNKSPSPPDGSPAATPEIRVNHEPEP	978750	726130	124
Q6YBV0	36	Proton-coupled amino acid transporter 4	SLC36A4	0.5	9.61E-05	89.483	S	DVMRPLINEQNFDGTSDEEHEQELLPVQKHY	0	0	3646
Q9BWU0	712	Kanadaptin	SLC4A1AP	0.999981	0.00196259	89.483	S	THFKETQTHENMSQLSEEEQNKDYQDCSKTT	0	811660	5433
Q5VSL9	339	Striatin-interacting protein 1	STRIP1	0.417243	0.00299396	89.468	S	SIKVIRNMRAASPPASASDLIEQQQKRGRRE	0	0	3320
Q5VSL9	341	Striatin-interacting protein 1	STRIP1	0.350419	0.00299396	89.468	S	KVIRNMRAASPPASASDLIEQQQKRGRREHK	0	0	3321
Q9H1E3	204	Nuclear ubiquitous casein and cyclin-dependent kinase substr	NUCKS1	1	0.00305187	89.468	S	VGRPTASKASKEKTPSPKEEDEEPESPPEKK	0	838520	5604
Q9H1E3	202	Nuclear ubiquitous casein and cyclin-dependent kinase substr	NUCKS1	1	0.00305187	89.468	T	GKVGRPTASKASKEKTPSPKEEDEEPESPPE	0	838520	8088
P51116	566	Fragile X mental retardation syndrome-related protein 2	FXR2	0.897211	8.55E-05	89.462	S	RRTDEDRTVMDGGLESDGPNMTENGLEDESR	1039600	984910	1777
Q86UU1	520	Pleckstrin homology-like domain family B member 1	PHLDB1	0.841339	0.0137729	89.46	S	FSLTLGARGRRTRSPSPTLGESLAPHKGSFS	0	0	3939
Q96QU8	211	Exportin-6	XPO6	0.450414	8.73E-07	89.434	S	DKHSVTAATPPPSPTSGESGDLLSNLLQSPS	0	0	5085
O75592	3931	E3 ubiquitin-protein ligase MYCBP2	MYCBP2	1	0.00324389	89.43	S	GDAEPTPEQEEKALLSSPEGEEKVYNATSDA	0	0	644
O75592	3932	E3 ubiquitin-protein ligase MYCBP2	MYCBP2	1	0.00324389	89.43	S	DAEPTPEQEEKALLSSPEGEEKVYNATSDAD	0	0	645
Q8N720	12	Zinc finger protein 655	ZNF655	1	0.00333123	89.43	S	____MEEIPAQEAAGSPRVQFQSLETQSECL	1027700	1216600	4295
Q96T17	289	MAP7 domain-containing protein 2	MAP7D2	0.646783	0.0191712	89.43	S	PLRRCEFSGGIPKRPSSPVISKTATKAYPQS	508620	486670	5143
P11388	1351	DNA topoisomerase 2-alpha	TOP2A	0.999998	0.00652945	89.356	S	FSDFDEKTDDEDFVPSDASPPKTKTSPKLSN	0	0	1023
Q6P2E9	565	Enhancer of mRNA-decapping protein 4	EDC4	0.999822	0.0004766	89.311	S	RPELGSEGLGSAAHGSQPDLRRIVELPAPAD	1223500	757920	3522
P78371	3	T-complex protein 1 subunit beta	CCT2	0.996963	0.00214949	89.301	S	_______________MASLSLAPVNIFKAGADE	253500	190100	1967
Q6UN15	259	Pre-mRNA 3'-end-processing factor FIP1	FIP1L1	0.994289	0.00395064	89.301	S	SEKETALPSTKAEFTSPPSLFKTGLPPSRNS	481850	343150	3585
Q14C86	757	GTPase-activating protein and VPS9 domain-containing protein	GAPVD1	0.494808	3.12E-05	89.297	S	SGLGSTSDDTDVREVSSRPSTPGLSVVSGIS	0	0	2839
Q14C86	758	GTPase-activating protein and VPS9 domain-containing protein	GAPVD1	0.531508	3.12E-05	89.297	S	GLGSTSDDTDVREVSSRPSTPGLSVVSGISA	0	799840	2840
Q14C86	761	GTPase-activating protein and VPS9 domain-containing protein	GAPVD1	0.494808	3.12E-05	89.297	S	STSDDTDVREVSSRPSTPGLSVVSGISATSE	0	0	2841
Q14C86	762	GTPase-activating protein and VPS9 domain-containing protein	GAPVD1	0.815707	3.12E-05	89.297	T	TSDDTDVREVSSRPSTPGLSVVSGISATSED	4284900	0	7629
Q13263	17	Transcription intermediary factor 1-beta	TRIM28	0.74633	4.53E-05	89.272	S	AASAAAASAAAASAASGSPGPGEGSAGGEKR	0	0	2447
Q06455	591	Protein CBFA2T1	RUNX1T1	0.444127	0.0142149	89.266	T	AGSPMDTPPAATPRSTTPGTPSTIETTPR__	0	0	7499
Q06455	592	Protein CBFA2T1	RUNX1T1	0.444117	0.0142149	89.266	T	GSPMDTPPAATPRSTTPGTPSTIETTPR___	0	0	7500
Q06455	601	Protein CBFA2T1	RUNX1T1	0.818842	0.0142149	89.266	T	ATPRSTTPGTPSTIETTPR____________	423380	0	7501
P46821	29	Microtubule-associated protein 1B;MAP1B heavy chain;MAP1 lig	MAP1B	0.354185	5.12E-06	89.197	S	SGSIANPAASTSPSLSHRFLDSKFYLLVVVG	0	0	1576
Q8IX07	487	Zinc finger protein ZFPM1	ZFPM1	1	1.05E-05	89.197	S	AAPILGPGEPGPQAPSRTPSPRSPAPARVKA	1827800	1033800	4092
Q8IX07	491	Zinc finger protein ZFPM1	ZFPM1	1	1.05E-05	89.197	S	LGPGEPGPQAPSRTPSPRSPAPARVKAELSS	1827800	1033800	4093
Q9Y3F4	335	Serine-threonine kinase receptor-associated protein	STRAP	0.99996	1.08E-05	89.197	S	IGFPETTEEELEEIASENSDCIFPSAPDVKA	9237900	9105500	6857
Q9Y3F4	338	Serine-threonine kinase receptor-associated protein	STRAP	0.999983	1.08E-05	89.197	S	PETTEEELEEIASENSDCIFPSAPDVKA___	13333000	11975000	6858
Q8IX07	489	Zinc finger protein ZFPM1	ZFPM1	1	1.05E-05	89.197	T	PILGPGEPGPQAPSRTPSPRSPAPARVKAEL	1827800	1033800	7850
P49815	1132	Tuberin	TSC2	0.999875	0.00475912	89.171	S	GQQVSRGARDRVRSMSGGHGLRVGALDVPAS	1354800	692040	1726
Q7Z739	385	YTH domain-containing family protein 3	YTHDF3	0.810254	2.19E-08	89.159	S	AGSENFGLGVVPVSASPSSVEVHPVLEKLKA	0	1060600	3886
O95835	613	Serine/threonine-protein kinase LATS1	LATS1	0.930779	0.00390892	89.08	S	ENVDSGDKEKKQITTSPITVRKNKKDEERRE	2042100	2193000	836
Q15648	1479	Mediator of RNA polymerase II transcription subunit 1	MED1	0.941535	0.000268699	89.079	S	ESESGSSIAEKSYQNSPSSDDGIRPLPEYST	5656000	3333700	2968
Q15648	1481	Mediator of RNA polymerase II transcription subunit 1	MED1	0.556937	0.000268699	89.079	S	ESGSSIAEKSYQNSPSSDDGIRPLPEYSTEK	5656000	3333700	2969
Q9BZL4	498	Protein phosphatase 1 regulatory subunit 12C	PPP1R12C	0.611249	8.36E-05	89.043	S	SVLSEVTKPPPCLENSSPPSRIPEPESPAKP	1650100	1207300	5493
P43686	28	26S protease regulatory subunit 6B	PSMC4	0.854776	0.000350243	89.039	S	DEIPALSVSRPQTGLSFLGPEPEDLEDLYSR	518340	0	1527
P22234	107	Multifunctional protein ADE2;Phosphoribosylaminoimidazole-su	PAICS	0.674636	0.00766419	89.029	S	EMIPIEWVCRRIATGSFLKRNPGVKEGYKFY	0	380170	1209
Q969V6	785	MKL/myocardin-like protein 1	MKL1	0.585267	5.16E-07	89.024	S	SLPGKEKPSPKTVCGSPLAAQPSPSAELPQA	814040	7130400	4755
Q969V6	807	MKL/myocardin-like protein 1	MKL1	0.892228	5.16E-07	89.024	S	SPSAELPQAAPPPPGSPSLPGRLEDFLESST	6603500	13494000	4757
Q7Z422	51	SUZ domain-containing protein 1	SZRD1	0.994663	0.000151275	89.011	S	KSKSPPKVPIVIQDDSLPAGPPPQIRILKRP	2054600	0	3803
Q6ZRS2	258	Helicase SRCAP	SRCAP	0.889354	0.0225099	88.992	S	YSDLLSQSLNQPLTSSKAGSSPCLGSSSAAS	390910	0	3662
Q96T58	2120	Msx2-interacting protein	SPEN	0.760464	0.000221755	88.986	S	AAAQAGERESGVVAVSPEKSESPQKEDGLSS	11183000	4612400	5168
Q96T58	2126	Msx2-interacting protein	SPEN	0.976914	0.000221755	88.986	S	ERESGVVAVSPEKSESPQKEDGLSSQLKSDP	11183000	4612400	5170
O43395	133	U4/U6 small nuclear ribonucleoprotein Prp3	PRPF3	0.788625	9.21E-05	88.983	S	EVEEEPEVIPGPPSESPGMLTKLQIKQMMEA	946000	1367000	376
Q9HCK8	1995	Chromodomain-helicase-DNA-binding protein 8	CHD8	0.5	0.016829	88.951	S	CSTPLLHQQYTSRTASPLPLRPDAPVEKSPE	989690	677760	5870
Q9H0S4	11	Probable ATP-dependent RNA helicase DDX47	DDX47	0.554267	0.000101334	88.951	T	_____MAAPEEHDSPTEASQPIVEEEETKTF	1324500	2608400	8084
Q9HCK8	1993	Chromodomain-helicase-DNA-binding protein 8	CHD8	0.5	0.016829	88.951	T	SRCSTPLLHQQYTSRTASPLPLRPDAPVEKS	989690	677760	8134
P04083	5	Annexin A1	ANXA1	1	0.00483712	88.948	S	_____________MAMVSEFLKQAWFIENEEQE	0	2305700	876
Q96RU2	712	Ubiquitin carboxyl-terminal hydrolase 28	USP28	0.193546	6.13E-06	88.917	S	EEQSCKIPQMESSTNSSSQDYSTSQEPSVAS	0	0	5102
Q9HAW0	387	Transcription factor IIIB 50 kDa subunit	BRF2	0.58746	4.19E-05	88.872	S	VPPVSTVTGDENISDSEIEQYLRTPQEVRDF	0	1345800	5822
Q96E09	45	Protein FAM122A	FAM122A	0.46592	7.26E-05	88.842	S	GGLRRSNSAPLIHGLSDTSPVFQAEAPSARR	0	0	4873
Q96T23	629	Remodeling and spacing factor 1	RSF1	0.717643	4.06E-05	88.829	S	TLESEKPGSPEAAETSPPSNIIDHCEKLASE	0	3444500	5151
Q8IU81	66	Interferon regulatory factor 2-binding protein 1	IRF2BP1	1	0.00778176	88.819	S	AARQLKRSHVLPEGRSPGPPALKHPATKDLA	2549000	1167400	4038
Q52LA3	51	Protein lin-52 homolog	LIN52	0.398173	0.0199058	88.819	T	LPGVAEFAASFKSPITSSPPKWMAEIERDDI	0	0	7690
Q5BKZ1	268	DBIRD complex subunit ZNF326	ZNF326	0.999919	0.0188073	88.803	S	IKKPKLAKPMEKISLSKSPTKTDPKNEEEEK	41251	335910	3157
Q5BKZ1	270	DBIRD complex subunit ZNF326	ZNF326	0.899521	0.0188073	88.803	S	KPKLAKPMEKISLSKSPTKTDPKNEEEEKRR	41251	335910	3158
O75694	3	Nuclear pore complex protein Nup155	NUP155	0.399818	0.000955991	88.79	S	_______________MPSSLLGAAMPASTSAAA	0	0	657
O75694	4	Nuclear pore complex protein Nup155	NUP155	0.399818	0.000955991	88.79	S	______________MPSSLLGAAMPASTSAAAL	0	0	658
O15127	320	Secretory carrier-associated membrane protein 2	SCAMP2	0.628434	0.0066901	88.78	S	SQGIFSSRTFHRAASSAAQGAFQGN______	0	0	290
Q8IX07	786	Zinc finger protein ZFPM1	ZFPM1	1	0.00813725	88.78	S	PGSGSGSGPGLAPARSPGPAADGPIDLSKKP	914850	674650	4091
Q9UQB8	261	Brain-specific angiogenesis inhibitor 1-associated protein 2	BAIAP2	0.579493	0.000122902	88.768	S	QVASNGATLPSALSASKSNLVISDPIPGAKP	1993500	0	6754
Q86UU1	516	Pleckstrin homology-like domain family B member 1	PHLDB1	0.599225	0.0137729	88.768	T	SPEDFSLTLGARGRRTRSPSPTLGESLAPHK	0	1227700	7826
Q14004	662	Cyclin-dependent kinase 13	CDK13	0.581519	8.72E-05	88.755	S	ADLPLPPELPGGDDLSKSPEEKKTATQLHSK	581000	422640	2634
Q9UPT8	1106	Zinc finger CCCH domain-containing protein 4	ZC3H4	0.838059	2.16E-05	88.709	T	SSRAAKPGPAEAPSPTASPSGDASPPATAPY	12151000	4101600	8249
Q8WUB8	327	PHD finger protein 10	PHF10	0.980708	2.75E-05	88.684	S	KRKNKGTSDSSSGNVSEGESPPDSQEDSFQG	1570300	1836900	4511
Q8WUA2	182	Peptidyl-prolyl cis-trans isomerase-like 4	PPIL4	0.603653	2.71E-05	88.684	T	DDPPDLLIPDRSPEPTREQLDSGRIGADEEI	0	0	7912
Q13330	386	Metastasis-associated protein MTA1	MTA1	0.999998	0.0185456	88.637	S	NVKAGVVNGTGAPGQSPGAGRACESCYTTQS	811600	0	2473
Q9HA82	342	Ceramide synthase 4	CERS4	0.987884	0.000147125	88.627	S	LYSFMKKGQMEKDIRSDVEESDSSEEAAAAQ	2912800	2338800	5810
Q9HA82	347	Ceramide synthase 4	CERS4	0.891304	0.000147125	88.627	S	KKGQMEKDIRSDVEESDSSEEAAAAQEPLQL	2912800	2338800	5811
Q9HA82	349	Ceramide synthase 4	CERS4	0.891304	0.000147125	88.627	S	GQMEKDIRSDVEESDSSEEAAAAQEPLQLKN	2912800	2338800	5812
Q9HA82	350	Ceramide synthase 4	CERS4	0.940716	0.000147125	88.627	S	QMEKDIRSDVEESDSSEEAAAAQEPLQLKNG	2912800	2338800	5813
Q3MHD2	16	Protein LSM12 homolog	LSM12	0.999986	0.00639607	88.596	S	MAAPPGEYFSVGSQVSCRTCQEQRLQGEVVA	3022300	1742000	3074
O14654	1231	Insulin receptor substrate 4	IRS4	0.99997	0.00153964	88.57	S	PPRSRRVPRPPEREDSDNDDDTHVRMDFARR	19766000	11166000	182
P49841	389	Glycogen synthase kinase-3 beta	GSK3B	0.76625	0.00321706	88.57	S	ATILIPPHARIQAAASTPTNATAASDANTGD	195250	194200	1735
Q13895	170	Bystin	BYSL	0.693141	0.000229084	88.57	S	LTEKQTEVETVMSEVSGFPMPQLDPRVLEVY	307520	199530	2629
Q9NVP2	198	Histone chaperone ASF1B	ASF1B	1	0.000527436	88.565	S	GLGLPGCIPGLLPENSMDCI___________	226330	279470	6046
P50851	1488	Lipopolysaccharide-responsive and beige-like anchor protein	LRBA	0.999993	0.0211101	88.552	S	KPMHSLIPLGKSAAKSPVDIVTGGISPVRDL	209960	315540	1766
Q8N6T3	189	ADP-ribosylation factor GTPase-activating protein 1	ARFGAP1	0.999982	0.0219986	88.552	T	SYQGAQGNRYVGFGNTPPPQKKEDDFLNNAM	675660	0	7881
P49023	320	Paxillin	PXN	0.249998	1.12E-05	88.544	S	GGQDEGGFMAQGKTGSSSPPGGPPKPGSQLD	0	0	1647
P49023	321	Paxillin	PXN	0.249998	1.12E-05	88.544	S	GQDEGGFMAQGKTGSSSPPGGPPKPGSQLDS	0	0	1648
P49023	322	Paxillin	PXN	0.521768	1.12E-05	88.544	S	QDEGGFMAQGKTGSSSPPGGPPKPGSQLDSM	6418100	0	1649
P49023	318	Paxillin	PXN	0.249998	1.12E-05	88.544	T	SPGGQDEGGFMAQGKTGSSSPPGGPPKPGSQ	0	0	7408
O15379	424	Histone deacetylase 3	HDAC3	0.999998	1.45E-05	88.518	S	APNEFYDGDHDNDKESDVEI___________	2557700	0	314
Q6FI81	182	Anamorsin	CIAPIN1	0.820073	0.0172653	88.507	S	EVGSSRQLKLSITKKSSPSVKPAVDPAAAKL	0	0	3457
Q6FI81	183	Anamorsin	CIAPIN1	0.931674	0.013458	88.507	S	VGSSRQLKLSITKKSSPSVKPAVDPAAAKLW	1592500	780610	3458
Q9C0B5	554	Palmitoyltransferase ZDHHC5	ZDHHC5	1	0.00796277	88.496	S	HIVASLQEREKLLRQSPPLPGREEEPGLGDS	2001500	1975600	5512
Q8N1G0	241	Zinc finger protein 687	ZNF687	0.609676	0.000231724	88.471	S	CSPHHPQVLAQQGSGSSPKATDIPASASPPP	0	0	4231
Q8N1G0	242	Zinc finger protein 687	ZNF687	0.701307	0.000231724	88.471	S	SPHHPQVLAQQGSGSSPKATDIPASASPPPV	4042500	0	4232
Q8TAD8	49	Smad nuclear-interacting protein 1	SNIP1	0.646731	0.00327734	88.471	S	LSPEVAPPAHRRPDHSGGSPSPPTSEPARSG	1896000	0	4432
Q13459	1405	Unconventional myosin-IXb	MYO9B	0.989848	0.000362542	88.413	S	KKKPGDASSLPDAGLSPGSQVDSKSTFKRLF	713680	675210	2537
Q9UHV7	530	Mediator of RNA polymerase II transcription subunit 13	MED13	0.977871	7.60E-07	88.413	S	DVAKTPQMHGTEMANSPQPPPLSPHPCDVVD	2732700	2500800	6368
Q9UHV7	537	Mediator of RNA polymerase II transcription subunit 13	MED13	0.99999	7.60E-07	88.413	S	MHGTEMANSPQPPPLSPHPCDVVDEGVTKTP	2732700	2500800	6369
Q13263	49	Transcription intermediary factor 1-beta	TRIM28	0.499279	2.47E-08	88.395	S	TAPSAAASASASAAASSPAGGGAEALELLEH	0	0	2461
Q99700	861	Ataxin-2	ATXN2	0.978959	4.40E-07	88.395	S	ENSSSNCTSGSSKPNSPSISPSILSNTEHKR	14832000	9601300	5250
Q99700	865	Ataxin-2	ATXN2	0.878079	4.40E-07	88.395	S	SNCTSGSSKPNSPSISPSILSNTEHKRGPEV	14832000	1449400	5252
Q8IXW5	414	Putative RNA polymerase II subunit B1 CTD phosphatase RPAP2	RPAP2	0.999185	0.000523669	88.385	S	EASLVKEELDEDDIISDPDSHFPAWRESQNS	0	0	4116
Q9UHB6	225	LIM domain and actin-binding protein 1	LIMA1	0.703297	3.29E-05	88.348	S	KILRAQSRSASGRKISENSYSLDDLEIGPGQ	0	824440	6331
Q96S59	487	Ran-binding protein 9	RANBP9	0.722001	0.000253179	88.329	S	DSYPVSPRPFSSPSMSPSHGMNIHNLASGKG	0	11504000	5114
P78356	326	Phosphatidylinositol 5-phosphate 4-kinase type-2 beta	PIP4K2B	0.796353	3.58E-07	88.293	S	GVGGNLLCSYGTPPDSPGNLLSFPRFFGPGE	0	1135200	1964
Q9NYF8	225	Bcl-2-associated transcription factor 1	BCLAF1	0.995945	2.54E-06	88.278	S	GDIWPGLSAYDNSPRSPHSPSPIATPPSQSS	2199800	958200	6108
Q9NYF8	230	Bcl-2-associated transcription factor 1	BCLAF1	0.974905	2.54E-06	88.278	S	GLSAYDNSPRSPHSPSPIATPPSQSSSCSDA	2199800	958200	6110
Q9NYF8	234	Bcl-2-associated transcription factor 1	BCLAF1	0.66965	2.54E-06	88.278	T	YDNSPRSPHSPSPIATPPSQSSSCSDAPMLS	2199800	0	8175
Q9NRR5	98	Ubiquilin-4	UBQLN4	0.371597	1.21E-09	88.247	S	KTPQKAQDPAAATASSPSTPDPASAPSTTPA	0	0	5977
Q9NRR5	100	Ubiquilin-4	UBQLN4	0.371597	1.21E-09	88.247	S	PQKAQDPAAATASSPSTPDPASAPSTTPASP	0	0	5978

Q9NRR5	109	Ubiquilin-4	UBQLN4	0.300628	1.21E-09	88.247	S	ATASSPSTPDPASAPSTTPASPATPAQPSTS	0	0	5979
Q9NRR5	110	Ubiquilin-4	UBQLN4	0.300628	1.21E-09	88.247	T	TASSPSTPDPASAPSTTPASPATPAQPSTSG	0	0	8157
Q9NRR5	111	Ubiquilin-4	UBQLN4	0.300628	1.21E-09	88.247	T	ASSPSTPDPASAPSTTPASPATPAQPSTSGS	0	0	8158
Q27J81	588	Inverted formin-2	INF2	0.430236	4.58E-05	88.244	S	PSNVAREHNSMWASLSSPDAEAVEPDFSSIE	0	0	3033
A1L390	1168	Pleckstrin homology domain-containing family G member 3	PLEKHG3	0.49987	1.59E-08	88.241	S	PSPTAGLEESSGQGPSSPVALLGQVQDFQQS	0	0	15
A1L390	1169	Pleckstrin homology domain-containing family G member 3	PLEKHG3	0.49987	1.59E-08	88.241	S	SPTAGLEESSGQGPSSPVALLGQVQDFQQSA	0	0	16
P55196	1201	Afadin	MLLT4	0.696173	9.24E-07	88.241	S	KSAYASGTTAKITSVSTGNLCTEEQTPPPRP	2565600	2110800	1866
A1L390	1156	Pleckstrin homology domain-containing family G member 3	PLEKHG3	0.526965	1.59E-08	88.241	T	NRRVIVMEKGPLPSPTAGLEESSGQGPSSPV	714600	845010	7109
Q13111	772	Chromatin assembly factor 1 subunit A	CHAF1A	0.938345	0.00435152	88.224	S	FQEHCRRGLLSNHTGSPRSPSTTYLHTPTPS	1937900	1494900	2400
Q9UPQ0	523	LIM and calponin homology domains-containing protein 1	LIMCH1	0.495986	0.00655139	88.224	S	ARASVLDTSMSAGSGSPSKTVTPKAVPMLTP	0	0	6569
Q13428	939	Treacle protein	TCOF1	0.780186	2.67E-08	88.191	S	AQAGKQDDSGSSSEESDSDGEAPAAVTSAQV	1747500	291070	2516
Q13428	941	Treacle protein	TCOF1	0.780186	2.67E-08	88.191	S	AGKQDDSGSSSEESDSDGEAPAAVTSAQVIK	0	291070	2517
Q7Z460	651	CLIP-associating protein 1	CLASP1	0.870501	0.00549283	88.19	T	SLGRIRTRRQSSGSATNVASTPDNRGRSRAK	0	2488700	7807
Q9H425	173	Uncharacterized protein C1orf198	C1orf198	0.57066	0.00436563	88.187	S	ASQGSQALKSSQGSRSSSLDALGPTRKEEEA	0	0	5655
Q9H425	174	Uncharacterized protein C1orf198	C1orf198	0.440008	0.00436563	88.187	S	SQGSQALKSSQGSRSSSLDALGPTRKEEEAS	0	0	5656
Q92769	394	Histone deacetylase 2	HDAC2	1	7.31E-07	88.186	S	GVQMQAIPEDAVHEDSGDEDGEDPDKRISIR	7595000	2401100	4700
A3KN83	214	Protein strawberry notch homolog 1	SBNO1	0.953588	0.0150403	88.177	S	EGARMWINDMKMRSFSPTMKVPVVKEDDEPE	699990	316530	31
Q9H7N4	724	Splicing factor, arginine/serine-rich 19	SCAF1	0.842389	0.00436921	88.177	S	RLDKSDPRGPSPAPASSPKREVLYDSEGLSG	2390100	0	5769
Q6ZRV2	924	Protein FAM83H	FAM83H	0.5	0.0042156	88.155	S	PERRGSPVPPVPERRSSPVPPVPERRGSLTL	827480	754320	3667
Q6ZRV2	925	Protein FAM83H	FAM83H	0.958492	0.0042156	88.155	S	ERRGSPVPPVPERRSSPVPPVPERRGSLTLT	827480	754320	3668
Q86WR7	179	Proline and serine-rich protein 2	PROSER2	1	0.0022524	88.155	S	LPSTPDPPRRELRAPSPPVEHPRLLRSVPTP	994790	617110	3993
Q12906	482	Interleukin enhancer-binding factor 3	ILF3	0.904759	3.38E-09	88.149	S	LPTGAEGRDSSKGEDSAEETEAKPAVVAPAP	0	2023200	2375
Q12906	486	Interleukin enhancer-binding factor 3	ILF3	0.654491	3.38E-09	88.149	T	AEGRDSSKGEDSAEETEAKPAVVAPAPVVEA	9519100	0	7517
O96017	378	Serine/threonine-protein kinase Chk2	CHEK2	0.706891	0.0208817	88.134	T	LIKITDFGHSKILGETSLMRTLCGTPTYLAP	0	1734200	7250
Q96T37	568	Putative RNA-binding protein 15	RBM15	1	0.01368	88.134	T	FGHRAPDPLRGARDRTPPLLYRDRDRDLYPD	927810	584780	8014
Q96N64	116	PWWP domain-containing protein 2A	PWWP2A	1	7.94E-07	88.108	S	ESAAAAPQGGPELPPSPASPPEQPPAPEERE	2042500	1282100	5026
Q96N64	119	PWWP domain-containing protein 2A	PWWP2A	1	7.94E-07	88.108	S	AAAPQGGPELPPSPASPPEQPPAPEEREEPP	2042500	1282100	5027
Q9UPU5	2604	Ubiquitin carboxyl-terminal hydrolase 24	USP24	0.751916	0.00384242	88.108	S	PANENGDRHLQQGSESPMMIGELRSDLDDVD	0	760550	6582
P49790	192	Nuclear pore complex protein Nup153	NUP153	0.999754	0.0190814	88.101	S	DDDNISTTSGFSSRASDKDITVSKNTSLPPL	0	0	1693
Q99590	798	Protein SCAF11	SCAF11	0.498444	0.0284648	88.101	S	KTKKPRTRRSRFHSPSTTWSPNKDTPQEKKR	0	0	5218
Q5T230	42	Undifferentiated embryonic cell transcription factor 1	UTF1	0.558238	0.000226817	88.071	S	RTPGDAPGTPPRRPASPSALGELGLPVSPGS	0	1253000	3278
Q5T230	44	Undifferentiated embryonic cell transcription factor 1	UTF1	0.819369	0.000226817	88.071	S	PGDAPGTPPRRPASPSALGELGLPVSPGSAQ	1635100	0	3279
Q5T230	54	Undifferentiated embryonic cell transcription factor 1	UTF1	0.982992	0.000226817	88.071	S	RPASPSALGELGLPVSPGSAQRTPWSARETE	1635100	1253000	3280
Q9Y4P8	412	WD repeat domain phosphoinositide-interacting protein 2	WIPI2	0.333333	0.00424833	88.056	S	TQTYGAAAGKGTYVPSSPTRLAYTDDLGAVG	0	0	6933
Q9Y4P8	413	WD repeat domain phosphoinositide-interacting protein 2	WIPI2	0.615601	0.00424833	88.056	S	QTYGAAAGKGTYVPSSPTRLAYTDDLGAVGG	0	2165300	6934
Q9Y4P8	415	WD repeat domain phosphoinositide-interacting protein 2	WIPI2	0.333333	0.00424833	88.056	T	YGAAAGKGTYVPSSPTRLAYTDDLGAVGGAC	0	0	8303
Q9BW85	316	Coiled-coil domain-containing protein 94	CCDC94	0.654912	7.52E-06	88.001	S	TPGASSLSQLGAYLDSDDSNGSN________	9872300	0	5422
P15336	265	Cyclic AMP-dependent transcription factor ATF-2	ATF2	0.482612	7.59E-05	88	S	RPVTMVPSVPGIPGPSSPQPVQSEAKMRLKA	0	0	1070
P15336	266	Cyclic AMP-dependent transcription factor ATF-2	ATF2	0.482612	7.59E-05	88	S	PVTMVPSVPGIPGPSSPQPVQSEAKMRLKAA	0	0	1071
P35611	600	Alpha-adducin	ADD1	1	0.000692811	88	S	GSEENLDEAREQKEKSPPDQPAVPHPPPSTP	3212000	1733600	1398
Q969V6	158	MKL/myocardin-like protein 1	MKL1	0.545318	0.000165662	88	T	ARVSEPLLSATSASPTQVVSQLPMGRDSREM	0	0	7964
Q7RTN6	46	STE20-related kinase adapter protein alpha	STRADA	0.622124	0.00852077	87.99	S	GEQPPGDTRRKTNDASSESIASFSKQEVMSS	522430	0	3744
Q70EL1	1286	Inactive ubiquitin carboxyl-terminal hydrolase 54	USP54	0.857549	0.024404	87.863	S	DSQLKHGAPRPGMKSSPHDSHTCVTYPERNH	396640	306310	3687
O94806	213	Serine/threonine-protein kinase D3	PRKD3	0.769933	0.00607293	87.824	S	FKIPNNCSGVRKRRLSNVSLPGPGLSVPRPL	0	0	692
P58658	397	Protein eva-1 homolog C	EVA1C	0.422662	0.0240219	87.824	S	SESDFPGELSGFCRTSYPIYSSIEAAELAER	0	0	1901
P58658	402	Protein eva-1 homolog C	EVA1C	0.349429	0.0240219	87.824	S	PGELSGFCRTSYPIYSSIEAAELAERIERRE	0	0	1902
P58658	396	Protein eva-1 homolog C	EVA1C	0.414354	0.0240219	87.824	T	PSESDFPGELSGFCRTSYPIYSSIEAAELAE	0	0	7453
P58658	398	Protein eva-1 homolog C	EVA1C	0.430422	0.0240219	87.824	Y	ESDFPGELSGFCRTSYPIYSSIEAAELAERI	0	0	8367
P58658	401	Protein eva-1 homolog C	EVA1C	0.358182	0.0240219	87.824	Y	FPGELSGFCRTSYPIYSSIEAAELAERIERR	0	0	8368
P85037	101	Forkhead box protein K1	FOXK1	1	0.00523587	87.806	S	AAPALVAAAAASVRQSPGPALARLEGREFEF	3946000	2253800	1996
Q96BY7	255	Autophagy-related protein 2 homolog B	ATG2B	0.988741	0.00834851	87.806	S	SPVCSTAPVETEPKLSPSWNPKIIYEPHPQL	1829100	817990	4808
Q8N5C6	149	S1 RNA-binding domain-containing protein 1	SRBD1	0.312721	7.78E-05	87.802	S	KVEEETSKASNLEGESNSSETPSTSTVWGGT	0	0	4280
Q8N5C6	151	S1 RNA-binding domain-containing protein 1	SRBD1	0.312721	7.78E-05	87.802	S	EEETSKASNLEGESNSSETPSTSTVWGGTCK	0	0	4281
Q8N5C6	152	S1 RNA-binding domain-containing protein 1	SRBD1	0.312721	7.78E-05	87.802	S	EETSKASNLEGESNSSETPSTSTVWGGTCKK	0	0	4282
Q53ET0	456	CREB-regulated transcription coactivator 2	CRTC2	0.613856	0.000140043	87.793	S	PADARRSQQQLPKQFSPTMSPTLSSITQGVP	0	0	3126
Q15139	235	Serine/threonine-protein kinase D1	PRKD1	0.989253	0.00524202	87.789	S	AELSTSAPDEPLLQKSPSESFIGREKRSNSQ	711690	497120	2873
Q9UHB7	814	AF4/FMR2 family member 4	AFF4	0.999981	0.00717559	87.789	S	ESSKQSAAKEKDLLPSPAGPVPSKDPKTEHG	834030	850300	6336
Q9Y6Y8	926	SEC23-interacting protein	SEC23IP	0.954878	0.00524202	87.789	S	EEEEKQVVEAEKVVESPDFSKDEDYLGKVGM	0	0	7035
O96017	516	Serine/threonine-protein kinase Chk2	CHEK2	0.333333	7.84E-05	87.739	S	EENESTALPQVLAQPSTSRKRPREGEAEGAE	0	0	846
O96017	518	Serine/threonine-protein kinase Chk2	CHEK2	0.333333	7.84E-05	87.739	S	NESTALPQVLAQPSTSRKRPREGEAEGAETT	0	0	847
Q9HCH0	567	Nck-associated protein 5-like	NCKAP5L	0.998982	1.05E-06	87.739	S	YENILDLSRSTFRGPSPEPPPSPLQVPTYPQ	0	772550	5857
Q9HCH0	573	Nck-associated protein 5-like	NCKAP5L	0.971981	1.05E-06	87.739	S	LSRSTFRGPSPEPPPSPLQVPTYPQLTLEVP	0	772550	5858
O96017	517	Serine/threonine-protein kinase Chk2	CHEK2	0.333333	7.84E-05	87.739	T	ENESTALPQVLAQPSTSRKRPREGEAEGAET	0	0	7249
Q13905	217	Rap guanine nucleotide exchange factor 1	RAPGEF1	0.673706	0.000169064	87.72	S	LVRLTIEKQGRPSPTSPVKPSSPASKPDGPA	2723600	0	2631
Q9NRA8	74	Eukaryotic translation initiation factor 4E transporter	EIF4ENIF1	0.999387	0.00453966	87.719	S	GVWDPEKWHASLYPASGRSSPVESLKKELDT	0	0	5957
Q92804	235	TATA-binding protein-associated factor 2N	TAF15	0.226841	6.09E-07	87.719	T	PRTDADSESDNSDNNTIFVQGLGEGVSTDQV	0	0	7947
Q5VWN6	685	Protein FAM208B	FAM208B	0.999999	0.00436216	87.713	S	EREILSPLFPRNGTKSPEAATPVGKVMPFRH	0	562410	3361
Q96E09	189	Protein FAM122A	FAM122A	0.999661	0.00526839	87.713	S	PIPSPTTRFTTRRSQSPINCIRPSVLGPLKR	4353700	3449400	4875
P16989	2	Y-box-binding protein 3	YBX3	0.949289	6.79E-07	87.712	S	______________MSEAGEATTTTTTTLPQ	402300	1839200	1100
P10721	943	Mast/stem cell growth factor receptor Kit	KIT	0.99997	0.00055972	87.676	S	ISESTNHIYSNLANCSPNRQKPVVDHSVRIN	3005900	3840400	1002
Q96T58	725	Msx2-interacting protein	SPEN	1	0.00700465	87.667	S	ESDRDRDHERRPIERSQSPVHLRRPQSPGAS	6280400	6260000	5181
P04114	3777	Apolipoprotein B-100;Apolipoprotein B-48	APOB	0.678205	0.0426255	87.647	S	KLDFREIQIYKKLRTSSFALNLPTLPEVKFP	0	0	877
P04114	3778	Apolipoprotein B-100;Apolipoprotein B-48	APOB	0.99997	0.0426255	87.647	S	LDFREIQIYKKLRTSSFALNLPTLPEVKFPE	0	0	878
P04114	3785	Apolipoprotein B-100;Apolipoprotein B-48	APOB	0.999994	0.0426255	87.647	T	IYKKLRTSSFALNLPTLPEVKFPEVDVLTKY	0	0	7257
Q13242	193	Serine/arginine-rich splicing factor 9	SRSF9	0.999692	0.00701709	87.641	S	SYIRVYPERSTSYGYSRSRSGSRGRDSPYQS	991800	0	2438
Q9UQ35	2409	Serine/arginine repetitive matrix protein 2	SRRM2	0.999922	0.0049873	87.641	T	SGRTSPPLLDRARSRTPPSAPSQSRMTSERA	0	6509300	8272
P48634	1004	Protein PRRC2A	PRRC2A	0.997544	0.00728382	87.568	S	GKLGGPKETPPNGNLSPAPRLRRDYSYERVG	984140	799030	1624
Q02543	123	60S ribosomal protein L18a	RPL18A	1	0.00848205	87.568	S	QCYRDMGARHRARAHSIQIMKVEEIAASKCR	518450	0	2096
Q99759	250	Mitogen-activated protein kinase kinase kinase 3	MAP3K3	1	0.00848205	87.568	S	ADSPSFRKSRMSRAQSFPDNRQEYSDRETQL	487590	323710	5276
Q8WXE1	239	ATR-interacting protein	ATRIP	1	0.00433378	87.519	S	SPRKNPSVVIKPEACSPQFGKTSFPTKESFS	877990	1367800	4591
P49815	1388	Tuberin	TSC2	0.499977	0.000568092	87.511	S	DLSFQPSQPLSKSSSSPELQTLQDILGDPGD	266960	183260	1731
Q9BWU0	82	Kanadaptin	SLC4A1AP	1	0.0141704	87.498	S	SQDLSGDFKKPALPVSPAARSKAPASSSSNP	0	558650	5434
A6NKD9	182	Coiled-coil domain-containing protein 85C	CCDC85C	0.319363	0.000145265	87.496	S	GGGGGGGAGSRSSIDSQASLSGPLSGGAPGA	0	0	42
Q6ICG6	271	Uncharacterized protein KIAA0930	KIAA0930	0.482198	0.000112375	87.496	T	MAVSRVSTGDTSPCGTEEDSSPASPMHERVT	0	0	7750
O15164	771	Transcription intermediary factor 1-alpha	TRIM24	0.54933	0.000277282	87.49	S	PRSILTSLLLNSSQSSTSEETVLRSDAPDST	0	393210	296
Q86V15	1758	Zinc finger protein castor homolog 1	CASZ1	0.996349	0.00014538	87.49	S	LAALGAPGPAPTAASSP______________	777210	847670	3944
A6H8Y1	2227	Transcription factor TFIIIB component B'' homolog	BDP1	1	0.0073288	87.476	S	TGPCTLGLDRGLGENSVEEPQIKDSKGDSVL	891410	0	34
Q7RTP6	1221	Protein-methionine sulfoxide oxidase MICAL3	MICAL3	1	0.015439	87.476	S	EKPKADAPSDLKAVHSPIRSQPVTLPEARTP	661950	368150	3745
O75044	989	SLIT-ROBO Rho GTPase-activating protein 2	SRGAP2	0.491535	0.000599947	87.388	T	LDTLEPLKTSPVVAPTSEPSSPLHTQLLKDP	0	0	7192
O75044	998	SLIT-ROBO Rho GTPase-activating protein 2	SRGAP2	0.496081	0.000599947	87.388	T	SPVVAPTSEPSSPLHTQLLKDPEPAFQRSAS	0	0	7193
Q9ULE6	406	Paladin	PALD1	0.999884	0.00511876	87.363	S	VVLENQKKLEGIRPESPAQGSGSRHSVWQRA	1314000	755460	6466
Q8NCF5	198	NFATC2-interacting protein	NFATC2IP	0.789834	0.0234337	87.356	S	DKEEKKKTEFLDLDNSPLSPPSPRTKSRTHT	318720	372410	4329
Q9Y6I3	454	Epsin-1	EPN1	0.987899	0.00014774	87.356	S	FDMSGVRGSLAEAVGSPPPAATPTPTPPTRK	1897600	833370	7010
P29966	131	Myristoylated alanine-rich C-kinase substrate	MARCKS	0.920984	8.77E-07	87.352	S	PGSPTAAEGEAASAASSTSSPKAEDGATPSP	749360	2106400	1330
P29966	132	Myristoylated alanine-rich C-kinase substrate	MARCKS	0.733687	8.77E-07	87.352	S	GSPTAAEGEAASAASSTSSPKAEDGATPSPS	0	2106400	1331
P10244	393	Myb-related protein B	MYBL2	0.767786	0.00011964	87.326	S	ISDLSRSSRGELIPISPSTEVGGSGIGTPPS	708850	0	984
P10244	405	Myb-related protein B	MYBL2	0.97144	0.00011964	87.326	T	IPISPSTEVGGSGIGTPPSVLKRQRKRRVAL	708850	694800	7272
Q53ET0	458	CREB-regulated transcription coactivator 2	CRTC2	0.477676	0.000148266	87.326	T	DARRSQQQLPKQFSPTMSPTLSSITQGVPLD	0	0	7692
Q96I25	227	Splicing factor 45	RBM17	0.333122	0.00116372	87.323	S	PVYEEQDRPRSPTGPSNSFLANMGGTVAHKI	0	0	4951
Q9UQ80	363	Proliferation-associated protein 2G4	PA2G4	0.563334	0.00540492	87.323	S	EVQDAELKALLQSSASRKTQKKKKKKASKTA	0	0	6751
Q02880	1336	DNA topoisomerase 2-beta	TOP2B	0.992992	0.000143155	87.319	S	SGKPSAKKVKKRNPWSDDESKSESDLEETEP	4498700	1674500	2102
Q02880	1340	DNA topoisomerase 2-beta	TOP2B	0.968441	0.000143155	87.319	S	SAKKVKKRNPWSDDESKSESDLEETEPVVIP	3366000	2462900	2103
Q9NTI5	1182	Sister chromatid cohesion protein PDS5 homolog B	PDS5B	0.887045	0.000148387	87.319	S	PGRIKGRLDSSEMDHSENEDYTMSSPLPGKK	3096900	0	6008
P52565	24	Rho GDP-dissociation inhibitor 1	ARHGDIA	0.99022	7.51E-07	87.28	S	EQLAQIAAENEEDEHSVNYKPPAQKSIQEIQ	159720	2095600	1812
Q12888	1462	Tumor suppressor p53-binding protein 1	TP53BP1	0.818698	1.04E-06	87.28	S	TRRTDVGAGALRRSDSPEIPFQAAAGPSDGL	9635200	7882100	2361
Q8IYB3	572	Serine/arginine repetitive matrix protein 1	SRRM1	1	0.00801477	87.216	T	RRRSPSPAPPPRRRRTPTPPPRRRTPSPPPR	7981400	5488900	7866
Q8IYB3	574	Serine/arginine repetitive matrix protein 1	SRRM1	1	0.00801477	87.216	T	RSPSPAPPPRRRRTPTPPPRRRTPSPPPRRR	7981400	5488900	7867
Q8N0Z3	232	Spindle and centriole-associated protein 1	SPICE1	0.467727	0.000236057	87.208	S	ISKLWTDIQQKIATQSQITPPGTPSSALSSG	0	0	4197
Q9C0C2	1024	182 kDa tankyrase-1-binding protein	TNKS1BP1	0.999952	0.000420682	87.208	S	LGEGSRDAGRPGERGSGGLFSPSTAHVPDGA	2067400	912280	5519
Q8N0Z3	230	Spindle and centriole-associated protein 1	SPICE1	0.467727	0.000236057	87.208	T	ELISKLWTDIQQKIATQSQITPPGTPSSALS	0	0	7871
Q9UPN4	531	Centrosomal protein of 131 kDa	CEP131	1	0.0258807	87.149	S	EPPEDGTLLSEAKLQSIMSFLDEMEKSGQDQ	246700	0	6559
Q9UPN4	534	Centrosomal protein of 131 kDa	CEP131	1	0.0258807	87.149	S	EDGTLLSEAKLQSIMSFLDEMEKSGQDQLDS	246700	0	6560
Q15007	305	Pre-mRNA-splicing regulator WTAP	WTAP	0.628226	0.00230135	87.138	S	SGFHREGNTTEDDFPSSPGNGNKSSNSSEER	0	0	2851
Q9ULH1	717	Arf-GAP with SH3 domain, ANK repeat and PH domain-containing	ASAP1	1	0.00803821	87.138	S	HVEYEWNLRQEEIDESDDDLDDKPSPIKKER	509530	332350	6474
Q8WWI1	865	LIM domain only protein 7	LMO7	0.548076	8.47E-05	87.102	S	FAKREDRVTTEIQLPSQSPVEEQSPASLSSL	0	1509700	4556
Q04656	1473	Copper-transporting ATPase 1	ATP7A	0.985381	0.000529892	87.098	S	NYSRASINSLLSDKRSLNSVVTSEPDKHSLL	1206900	78787	2160
Q04656	1486	Copper-transporting ATPase 1	ATP7A	0.990475	0.000529892	87.098	S	KRSLNSVVTSEPDKHSLLVGDFREDDDTAL_	1206900	78787	2161
Q9BTA9	532	WW domain-containing adapter protein with coiled-coil	WAC	0.363857	2.68E-05	87.098	S	RSSSQRSPSPGPNHTSNSSNASNATVVPQNS	0	0	5352
Q9BTA9	534	WW domain-containing adapter protein with coiled-coil	WAC	0.666764	2.68E-05	87.098	S	SSQRSPSPGPNHTSNSSNASNATVVPQNSSA	0	4679500	5353
P50851	1135	Lipopolysaccharide-responsive and beige-like anchor protein	LRBA	0.996762	7.85E-06	87.091	S	TEEANLPTELQDNSLSPAASEAGEKLDMFGN	3338900	2163500	1769
Q8NHG8	135	E3 ubiquitin-protein ligase ZNRF2	ZNRF2	1	0.027403	87.083	S	SPEDGGGGRDRPVGGSPGGPRLVIGSLPAHL	964520	452650	4410
O75381	3	Peroxisomal membrane protein PEX14	PEX14	0.495136	0.00010315	87.062	S	_____________MASSEQAEQPSQPSSTPG	0	0	611
O75381	4	Peroxisomal membrane protein PEX14	PEX14	0.495136	0.00010315	87.062	S	____________MASSEQAEQPSQPSSTPGS	0	0	612
Q9UJX5	777	Anaphase-promoting complex subunit 4	ANAPC4	0.5	3.20E-05	87.052	S	EEASNKPVKIKEEVLSESEAENQQAGAAALA	0	0	6413
Q9UJX5	779	Anaphase-promoting complex subunit 4	ANAPC4	0.5	3.20E-05	87.052	S	ASNKPVKIKEEVLSESEAENQQAGAAALAPE	0	0	6414
Q9H4L7	50	SWI/SNF-related matrix-associated actin-dependent regulator	SMARCAD1	0.998693	2.36E-07	87.025	S	PISLSAEEENAEGEVSRANTPDSDITEKTED	7201000	3050700	5693
Q14160	1559	Protein scribble homolog	SCRIB	0.383469	5.56E-08	86.946	S	PTPSPTPVEDLGPQTSTSPGRLSPDFAEELR	0	0	2675
Q14160	1558	Protein scribble homolog	SCRIB	0.383469	2.60E-05	86.946	T	APTPSPTPVEDLGPQTSTSPGRLSPDFAEEL	0	0	7582
Q07021	217	Complement component 1 Q subcomponent-binding protein, mitoc	C1QBP	0.455013	0.0253214	86.944	S	DIFSIREVSFQSTGESEWKDTNYTLNTDSLD	0	0	2215
Q8N122	877	Regulatory-associated protein of mTOR	RPTOR	0.999633	3.47E-05	86.918	S	ASPTNKGVHIHQAGGSPPASSTSSSSLTNDV	3099800	2673900	4208
P51956	355	Serine/threonine-protein kinase Nek3	NEK3	0.978348	0.00886629	86.882	S	REEKGNKSVHLRKASSPNLHRRQWEKNVPNT	1306600	829150	1803
P62888	10	60S ribosomal protein L30	RPL30	0.998935	0.00886629	86.882	S	______MVAAKKTKKSLESINSRLQLVMKSG	618840	285840	1926
Q5T200	64	Zinc finger CCCH domain-containing protein 13	ZC3H13	1	0.00886629	86.882	S	GNCLYGNTCRFVHGPSPRGKGYSSNYRRSPE	0	43034	3254
Q96G74	177	OTU domain-containing protein 5	OTUD5	0.862279	5.38E-07	86.869	S	GGGSPEREEVGAGYNSEDEYEAAAARIEAMD	2374500	1425900	4915
Q9UHR5	18	SAP30-binding protein	SAP30BP	0.96651	6.94E-07	86.869	S	GKKNVLSSLAVYAEDSEPESDGEAGIEAVGS	1137200	521520	6365
Q9UHR5	22	SAP30-binding protein	SAP30BP	0.998327	6.94E-07	86.869	S	VLSSLAVYAEDSEPESDGEAGIEAVGSAAEE	1137200	521520	6366
Q9BQ65	36	U6 snRNA phosphodiesterase	USB1	1	0.015786	86.866	S	GMRTRPGDGSHRRGQSPLPRQRFPVPDSVLN	0	444960	5302
Q99700	728	Ataxin-2	ATXN2	0.795775	5.68E-09	86.859	S	GIIPTEAVAMPIPAASPTPASPASNRAVTPS	2602000	1656900	5258
Q99613;B	18;18	Eukaryotic translation initiation factor 3 subunit C;Eukary	EIF3C;EIF3	0.89081	1.01E-05	86.785	S	RFFTTGSDSESESSLSGEELVTKPVGGNYGK	2533000	1457900	69

Q9NYV4	274	Cyclin-dependent kinase 12	CDK12	1	0.0267391	86.772	S	YDSYKKSPGSTSRRQSVSPPYKEPSAYQSST	3528000	2089800	6131
Q9NYV4	276	Cyclin-dependent kinase 12	CDK12	0.999735	0.0267391	86.772	S	SYKKSPGSTSRRQSVSPPYKEPSAYQSSTRS	3528000	2089800	6132
Q9BYW2	2080	Histone-lysine N-methyltransferase SETD2	SETD2	0.582471	0.00518758	86.738	S	PDKQTQNKEKRKRRSSLSPPSSAYERGTKRP	901820	741480	5477
Q9BYW2	2082	Histone-lysine N-methyltransferase SETD2	SETD2	0.987975	0.00518758	86.738	S	KQTQNKEKRKRRSSLSPPSSAYERGTKRPDD	901820	741480	5478
O96028	110	Histone-lysine N-methyltransferase NSD2	WHSC1	0.999981	0.0152904	86.738	T	DAKLRFESQEMKGIGTPPNTTPIKNGSPEIK	64750	4936900	7251
O96028	115	Histone-lysine N-methyltransferase NSD2	WHSC1	0.69101	0.0152904	86.738	T	FESQEMKGIGTPPNTTPIKNGSPEIKLKITK	0	0	7252
Q86YS7	817	C2 domain-containing protein 5	C2CD5	0.5	1.70E-05	86.727	S	LQTTKTPVEKSLQRASTDNEELLQFPLELCS	1561400	803380	4027
Q86YS7	818	C2 domain-containing protein 5	C2CD5	0.5	1.70E-05	86.727	T	QTTKTPVEKSLQRASTDNEELLQFPLELCSD	1561400	803380	7836
O95365	511	Zinc finger and BTB domain-containing protein 7A	ZBTB7A	0.548218	5.96E-05	86.707	S	RRGRKPRVRGGAPDPSPGATATPGAPAQPSS	1606100	0	755
P23246	381	Splicing factor, proline- and glutamine-rich	SFPQ	0.509245	9.09E-05	86.707	Y	FATHAAALSVRNLSPYVSNELLEEAFSQFGP	843580	374250	8357
Q9BTA9	535	WW domain-containing adapter protein with coiled-coil	WAC	0.598938	2.61E-05	86.701	S	SQRSPSPGPNHTSNSSNASNATVVPQNSSAR	0	0	5354
Q9HD20	899	Manganese-transporting ATPase 13A1	ATP13A1	0.999815	0.0049197	86.699	S	NAPERVVERRRRPRDSPTLSNSGIRATSRTA	3891500	0	5878
Q96DF8	292	Protein DGCR14	DGCR14	1	0.00563281	86.67	S	QGKVGPDGKELIPQESPRVGGFGFVATPSPA	1132600	0	4861
Q8N108	491	Mesoderm induction early response protein 1	MIER1	0.816663	0.000199915	86.641	S	AKRRRVNSNGKESPGSSEFFQEAVSHGKFEE	0	3782900	4204
Q9NRH2	610	SNF-related serine/threonine-protein kinase	SNRK	0.370694	1.71E-05	86.641	S	KASPSENNAGGGSPSSGSGGNPTNTSGTTRR	0	0	5966
Q9BYW2	321	Histone-lysine N-methyltransferase SETD2	SETD2	0.995759	0.0178836	86.625	S	GSKKKSSQSEGIFLGSESDEDSVRTSSSQRS	624790	515960	5479
Q9BYW2	323	Histone-lysine N-methyltransferase SETD2	SETD2	0.988629	0.0178836	86.625	S	KKKSSQSEGIFLGSESDEDSVRTSSSQRSHD	624790	515960	5480
Q9NRH2	390	SNF-related serine/threonine-protein kinase	SNRK	0.935304	3.58E-05	86.625	S	DDLTATPLSHATVPQSPARAADSVLNGHRSK	910220	540510	5968
Q8ND82	544	Zinc finger protein 280C	ZNF280C	0.374969	3.45E-05	86.617	S	GCAPGTSFLQVTPPTSQNTTARNPRKSNASR	0	0	4344
Q8ND82	543	Zinc finger protein 280C	ZNF280C	0.506087	3.45E-05	86.617	T	FGCAPGTSFLQVTPPTSQNTTARNPRKSNAS	0	1260300	7890
Q15648	1482	Mediator of RNA polymerase II transcription subunit 1	MED1	0.839463	0.000351177	86.604	S	SGSSIAEKSYQNSPSSDDGIRPLPEYSTEKH	0	3333700	2970
Q12888	1094	Tumor suppressor p53-binding protein 1	TP53BP1	0.999954	0.0015202	86.575	S	GDHTIRQSQQPMKPISPVKDPVSPASQKMVI	28574000	14151000	2358
Q12888	1101	Tumor suppressor p53-binding protein 1	TP53BP1	0.999974	0.0015202	86.575	S	SQQPMKPISPVKDPVSPASQKMVIQGPSSPQ	28574000	14151000	2359
P42694	629	Probable helicase with zinc finger domain	HELZ	0.978545	0.000445122	86.573	S	GVLFPDISMTPTIPWSPNRQWDEQLDPRLNA	528480	296060	1511
Q7KZI7	624	Serine/threonine-protein kinase MARK2	MARK2	0.979862	0.000478495	86.573	S	QNLPYGVTPASPSGHSQGRRGASGSIFSKFT	0	0	3713
Q8IWA0	796	WD repeat-containing protein 75	WDR75	0.854681	0.00123709	86.573	S	DSDEENDFTEKVQDTSNTGLGEDIIHQLSKS	1656200	0	4054
Q86VM9	609	Zinc finger CCCH domain-containing protein 18	ZC3H18	0.657369	0.00110214	86.557	S	GSRSRSRSFSSSPSPSPTPSPHRPSIRTKGE	1108200	278120	3956
Q86VM9	618	Zinc finger CCCH domain-containing protein 18	ZC3H18	0.933491	0.00110214	86.557	S	SSSPSPSPTPSPHRPSIRTKGEPAPPPGKAG	1108200	278120	3958
Q9BY67	434	Cell adhesion molecule 1	CADM1	0.992764	8.76E-05	86.557	S	DADTAIINAEGGQNNSEEKKEYFI_______	542230	0	5466
Q9BRT9	12	DNA replication complex GINS protein SLD5;DNA replication co	GINS4	0.999971	2.31E-05	86.551	S	____MTEEVDFLGQDSDGGSEEVVLTPAELI	29273000	6635000	5333
Q9BRT9	16	DNA replication complex GINS protein SLD5;DNA replication co	GINS4	0.999866	2.31E-05	86.551	S	MTEEVDFLGQDSDGGSEEVVLTPAELIERLE	26966000	8311400	5334
Q32MZ4	766	Leucine-rich repeat flightless-interacting protein 1	LRRFIP1	0.499998	0.00359976	86.548	S	EMSEHPSQTVRKALDSNSLENDDLSAPGREP	800830	1073200	3051
O75152;A(	494;494	Zinc finger CCCH domain-containing protein 11A	ZC3H11A	0.459814	0.000283353	86.539	S	LEKALRVQQSSESSTSSPSQHEATPGARRLL	0	0	576
O75152;A(	495;495	Zinc finger CCCH domain-containing protein 11A	ZC3H11A	0.459814	0.000283353	86.539	S	EKALRVQQSSESSTSSPSQHEATPGARRLLR	0	0	577
Q92538	1778	Golgi-specific brefeldin A-resistance guanine nucleotide exc	GBF1	0.484852	0.0072734	86.497	S	LGACDFEKPESPRAASSSSPGSPVASSPSRL	0	0	4621
Q8WWM7	683	Ataxin-2-like protein	ATXN2L	0.659886	0.0204177	86.463	T	PTKPLLSVNKSTSTPTSPGPRTHSTPSIPVL	0	0	7924
Q7Z7L9	168	Zinc finger and SCAN domain-containing protein 2	ZSCAN2	0.549491	0.000285648	86.453	S	ENESRKIFSEMPEGESAQHSDGESDFERDAG	0	0	3891
Q4LE39	1031	AT-rich interactive domain-containing protein 4B	ARID4B	0.450457	2.03E-06	86.449	S	SNSVLNTPPTTPESPSSVTVTEGSRQQSSVT	0	0	3101
Q4LE39	1032	AT-rich interactive domain-containing protein 4B	ARID4B	0.450457	2.03E-06	86.449	S	NSVLNTPPTTPESPSSVTVTEGSRQQSSVTV	0	0	3102
Q4LE39	1026	AT-rich interactive domain-containing protein 4B	ARID4B	0.642631	2.03E-06	86.449	T	FPSSGSNSVLNTPPTTPESPSSVTVTEGSRQ	1766900	0	7684
Q12857	303	Nuclear factor 1 A-type	NFIA	0.499243	9.19E-05	86.376	S	PGEEPFYTGQGRSPGSGSQSSGWHEVEPGMP	0	0	2319
Q9UQ35	315	Serine/arginine repetitive matrix protein 2	SRRM2	0.781011	0.000113569	86.357	T	SGRRGEGDAPFSEPGTTSTQRPSSPETATKQ	1114600	600480	8257
Q9UQ35	316	Serine/arginine repetitive matrix protein 2	SRRM2	0.782611	0.000113569	86.357	T	GRRGEGDAPFSEPGTTSTQRPSSPETATKQP	0	0	8258
P33991	326	DNA replication licensing factor MCM4	MCM4	1	0.00574664	86.344	S	HTTRVEMDRGRIAEPSVCGRCHTTHSMALIH	835170	94924	1367
Q9NYF3	122	Protein FAM53C	FAM53C	0.999858	0.00481634	86.344	S	PVPPAPPSKRHCRSLSVPVDLSRWQPVWRPA	899250	580250	6099
Q92804	228	TATA-binding protein-associated factor 2N	TAF15	0.324887	4.60E-07	86.304	S	GGHRDYGPRTDADSESDNSDNNTIFVQGLGE	0	0	4718
Q9NR09	577	Baculoviral IAP repeat-containing protein 6	BIRC6	0.861129	0.0079109	86.288	S	IPFPCLLAGGLLTYKSPATSPISSNSHRSLD	416030	0	5940
Q5JRA6	1740	Melanoma inhibitory activity protein 3	MIA3	0.439261	8.64E-05	86.222	S	SPSDPGSGTATMMNSSSRGSSPTRVLDEGKV	0	0	3177
P25054	2461	Adenomatous polyposis coli protein	APC	0.710159	0.0274273	86.214	S	EAPSPTLRRKLEESASFESLSPSSRPASPTR	572840	253030	1261
Q7Z6Z7	3660	E3 ubiquitin-protein ligase HUWE1	HUWE1	0.613398	0.0009956	86.213	T	LREYNLEQQRRAQCETLSPDGLPEEQPQTTK	0	0	7816
Q92598	557	Heat shock protein 105 kDa	HSPH1	0.958939	3.32E-08	86.208	S	PQVQTDAQQTSQSPPSPELTSEENKIPDADK	25825000	21974000	4655
Q9Y4G8	1116	Rap guanine nucleotide exchange factor 2	RAPGEF2	0.979673	0.000100759	86.191	S	KQAEDTISNASSQLSSPPTSPQSSPRKGYTL	1765900	650580	6920
Q9Y4G8	1120	Rap guanine nucleotide exchange factor 2	RAPGEF2	0.76995	0.000100759	86.191	S	DTISNASSQLSSPPTSPQSSPRKGYTLAPSG	0	650580	6921
Q8NAV1	209	Pre-mRNA-splicing factor 38A	PRPF38A	1	0.0162015	86.136	S	SEEEEEEDEKLERVPSPDHRRRSYRDLDKPR	0	866030	4311
P30622	158	CAP-Gly domain-containing linker protein 1	CLIP1	0.336652	2.62E-06	86.131	S	SRATSPLCTSTASMVSSSPSTPSNIPQKPSQ	0	0	1348
Q658Y4	355	Protein FAM91A1	FAM91A1	0.307506	5.20E-08	86.095	S	SPVQEASSATDTDTNSQEDPADTASVSSLSL	0	0	3395
Q658Y4	349	Protein FAM91A1	FAM91A1	0.221022	5.20E-08	86.095	T	DGGESRSPVQEASSATDTDTNSQEDPADTAS	0	0	7741
Q658Y4	351	Protein FAM91A1	FAM91A1	0.350281	5.20E-08	86.095	T	GESRSPVQEASSATDTDTNSQEDPADTASVS	0	0	7742
Q658Y4	353	Protein FAM91A1	FAM91A1	0.307506	5.20E-08	86.095	T	SRSPVQEASSATDTDTNSQEDPADTASVSSL	0	0	7743
O75530	34	Polycomb protein EED	EED	0.93164	6.25E-09	86.035	S	KKQKLSSDENSNPDLSGDENDDAVSIESGTN	0	3010500	637
O15027	1964	Protein transport protein Sec16A	SEC16A	0.999999	0.00128681	85.988	S	LLPSAPQTLPDGPLASPARVPMFPVPLPPGP	2396700	1749400	243
Q2KHR3	586	Glutamine and serine-rich protein 1	QSER1	1	0.0094979	85.976	S	VLQPQASLESSTQRLSDGEINAQESTYKVSK	970340	383790	3038
Q01082	2106	Spectrin beta chain, non-erythrocytic 1	SPTBN1	0.585656	0.0202836	85.973	S	QEEEERKRRPPSPEPSTKVSEEAESQQQWDT	962600	0	2049
Q92576	1133	PHD finger protein 3	PHF3	1	0.0051902	85.973	S	CKICIGRMAPPVDDLSPKKVKVVVGVARKHS	7014700	4126500	4641
Q15696	349	U2 small nuclear ribonucleoprotein auxiliary factor 35 kDa s	ZRSR2	0.999959	0.00938327	85.958	S	NPNNEFWEANRDIYLSPDRTGSSFGKNSERR	1394500	699910	2978
Q9NZM4	1413	Glioma tumor suppressor candidate region gene 1 protein	GLTSCR1	1	0.0049445	85.958	S	PGAPEGTPAGRARGGSPAPLPAKVDEATSGL	887050	676780	6162
O14686	1865	Histone-lysine N-methyltransferase 2D	KMT2D	0.999709	0.000167046	85.884	T	GVKASPVPSDPEKPGTPGEGMLSSDLDRIST	968560	988470	7131
P50613	164	Cyclin-dependent kinase 7	CDK7	1	0.0163573	85.862	S	GVLKLADFGLAKSFGSPNRAYTHQVVTRWYR	6698900	3539900	1758
Q9UPU5	1141	Ubiquitin carboxyl-terminal hydrolase 24	USP24	0.989065	0.0163573	85.862	S	RKKTLLSESSSQSSKSPSLSSKQQHQPSASS	0	2450800	6586
Q86X29	464	Lipolysis-stimulated lipoprotein receptor	LSR	1	0.00499273	85.813	S	GPALTPIRDEEWGGHSPRSPRGWDQEPAREQ	57890	0	4006
Q9ULC8	526	Probable palmitoyltransferase ZDHHC8	ZDHHC8	0.999789	0.00593426	85.807	S	PGATGDPPRPLPRSFSPVLGPRPREPSPVRY	0	0	6465
Q5VVJ2	218	Histone H2A deubiquitinase MYSM1	MYSM1	0.999155	7.80E-05	85.798	S	GRADPNLNAVKIEKLSDDEEVDITDEVDELS	947070	1431600	3355
Q14160	1579	Protein scribble homolog	SCRIB	0.534628	0.000136397	85.737	S	RLSPDFAEELRSLEPSPSPGPQEEDGEVALV	0	0	2688
Q9BTU6	44	Phosphatidylinositol 4-kinase type 2-alpha	PI4K2A	0.926941	0.00130841	85.734	S	PQVPGGAVRVAAAAGSGPSPPGSPGHDRERQ	0	1783700	5367
P06748	17	Nucleophosmin	NPM1	0.984301	0.00359178	85.734	Y	EDSMDMDMSPLRPQNYLFGCELKADKDYHFK	1041600	224630	8354
Q9ULU4	488	Protein kinase C-binding protein 1	ZMYND8	0.838725	0.000122593	85.728	S	TASPASTKTGQAGSLSGSPKPFSPQLSAPIT	0	0	6508
Q8NDF8	48	Non-canonical poly(A) RNA polymerase PAPD5	PAPD5	0.447731	4.08E-08	85.715	S	HHQPGAWARRAGSSASSPPSASSSPHPSAAV	0	0	4347
Q9UPT8	1108	Zinc finger CCCH domain-containing protein 4	ZC3H4	0.677833	8.18E-05	85.619	S	RAAKPGPAEAPSPTASPSGDASPPATAPYDP	0	4308600	6576
Q8NDI1	427	EH domain-binding protein 1	EHBP1	0.480991	0.00445776	85.619	T	GVLNENTVSAGKDLSTSPKPSPIPSPVLGRK	0	0	7891
Q96JC9	157	ELL-associated factor 1	EAF1	0.500105	0.0110415	85.619	T	PMPFRAPTKPPVGPKTSPLKDNPSPEPQLDD	5370900	2051200	7987
Q15633	152	RISC-loading complex subunit TARBP2	TARBP2	0.98581	1.01E-05	85.595	S	VVLTRSPPMELQPPVSPQQSECNPVGALQEL	6802600	6933300	2952
Q9H3U1	854	Protein unc-45 homolog A	UNC45A	0.999998	0.0109076	85.576	S	AGGLAMLTSMRPTLCSRIPQVTTHWLEILQA	0	0	5648
Q9H3U1	851	Protein unc-45 homolog A	UNC45A	0.97473	0.0109076	85.576	T	RAAAGGLAMLTSMRPTLCSRIPQVTTHWLEI	0	0	8098
Q9UP83	197	Conserved oligomeric Golgi complex subunit 5	COG5	1	0.00508469	85.536	S	ILNLSKRLQGQLQGGSREITKAAQSLNELDY	935230	0	6555
O60318	538	Germinal-center associated nuclear protein	MCM3AP	1	0.000279845	85.518	S	DGEVSPSTEDAPFQHSPLGKAAGRTGASSLL	10999000	6201500	479
Q9H4Z3	146	Phosphorylated CTD-interacting factor 1	PCIF1	0.404791	0.000100384	85.518	S	EIPVTPTGQSVPSSPSIPGTPTLKMWGTSPE	0	0	5704
Q6W2J9	1127	BCL-6 corepressor	BCOR	0.895581	0.00031086	85.51	S	VSEPPADQVASDMPHSPTLRVDRKRKVSGDS	1066800	1023600	3619
Q96KN1	288	Protein FAM84B	FAM84B	0.499998	0.000311508	85.496	T	LHPAEPEEGDSNVARTTPPPGRPPAPSSEEE	7136500	0	7989
Q96KN1	289	Protein FAM84B	FAM84B	0.499998	0.000311508	85.496	T	HPAEPEEGDSNVARTTPPPGRPPAPSSEEED	7136500	0	7990
Q14160	1448	Protein scribble homolog	SCRIB	0.999238	0.00896175	85.473	S	PPWASPSPTSRQSPASPPPLGGAPVRTAKA	1085700	747440	2685
Q13247	295	Serine/arginine-rich splicing factor 6	SRSF6	0.998799	0.00933729	85.469	S	SPKENGKGDIKSKSRSRSQSRSNSPLPVPPS	1392600	674790	2445
O15551	199	Claudin-3	CLDN3	0.786648	0.0107694	85.45	S	CPPREKKYTATKVVYSAPRSTGPGASLGTGY	0	85724	335
Q5VT06	1256	Centrosome-associated protein 350	CEP350	0.678284	0.00568634	85.45	S	VSVSSDKGRSQKTPTSPLSPSSQKSLQFDVA	0	430280	3326
Q5VT06	1259	Centrosome-associated protein 350	CEP350	0.990447	0.00568634	85.45	S	SSDKGRSQKTPTSPLSPSSQKSLQFDVAGTS	0	430280	3327
O15551	192	Claudin-3	CLDN3	0.999998	0.0107694	85.45	T	GALLCCSCPPREKKYTATKVVYSAPRSTGPG	0	85724	7161
O15551	194	Claudin-3	CLDN3	0.664081	0.0107694	85.45	T	LLCCSCPPREKKYTATKVVYSAPRSTGPGAS	0	85724	7162
O15551	191	Claudin-3	CLDN3	0.999998	0.0107694	85.45	Y	GGALLCCSCPPREKKYTATKVVYSAPRSTGP	0	85724	8347
O15085	1155	Rho guanine nucleotide exchange factor 11	ARHGEF11	0.999999	5.06E-07	85.399	S	QGKHQVLLEDPEQEGSAEEEELGVLPCPSTS	2826800	2179100	283
O14893	171	Gem-associated protein 2	GEMIN2	0.452736	1.53E-06	85.399	Y	DGAVGPATNESPGIDYVQIGFPPLLSIVSRM	0	0	8344
Q9H813	7	Transmembrane protein 206	TMEM206	0.331014	6.38E-05	85.376	S	__________MIRQERSTSYQELSEELVQVVE	0	0	5778
Q9H813	9	Transmembrane protein 206	TMEM206	0.255821	6.38E-05	85.376	S	________MIRQERSTSYQELSEELVQVVENS	0	0	5779
Q9H813	8	Transmembrane protein 206	TMEM206	0.255821	6.38E-05	85.376	T	_________MIRQERSTSYQELSEELVQVVEN	0	0	8119
Q4LE39	680	AT-rich interactive domain-containing protein 4B	ARID4B	0.614557	0.00492611	85.28	S	LSKPPFQTNPSPEMVSKLDLTDAKNSDTAHI	3503400	0	3104
P07900	726	Heat shock protein HSP 90-alpha	HSP90AA1	0.499997	8.12E-07	85.19	S	AVTEEMPPLEGDDDTSRMEEVD_________	977970	1017000	943
P07900	725	Heat shock protein HSP 90-alpha	HSP90AA1	0.499997	8.12E-07	85.19	T	AAVTEEMPPLEGDDDTSRMEEVD________	977970	1017000	7267
P13051	23	Uracil-DNA glycosylase	UNG	0.942092	9.64E-06	85.185	S	YSFFSPSPARKRHAPSPEPAVQGTGVAGVPE	6057500	2903100	1048
O95905	419	Protein SGT1	ECD	1	1.31E-06	85.184	S	AANLPPEDDDQWLDLSPDQLDQLLQEAVGKK	0	171720	838
P29966	133	Myristoylated alanine-rich C-kinase substrate	MARCKS	0.447148	1.04E-06	85.184	T	SPTAAEGEAASAASSTSSPKAEDGATPSPSN	0	0	7346
Q9H792	835	Pseudopodium-enriched atypical kinase 1	PEAK1	0.474369	0.000292149	85.177	T	LFTSQPSGEAEAPQTTDSPTTKVQKDPSIKP	0	0	8111
O75122	525	CLIP-associating protein 2	CLASP2	0.553009	0.0268122	85.176	S	RLSVARSSRIPRPSVSQGCSREASRESSRDT	5188300	2863500	559
Q16513	583	Serine/threonine-protein kinase N2	PKN2	0.574629	0.0098929	85.163	S	FDLEPEPPPAPPRASSLGEIDESSELRVLDI	0	1612600	3011
O14617	632	AP-3 complex subunit delta-1	AP3D1	1	3.85E-05	85.11	S	VPEGLDLDAWINEPLSDSESEDERPRAVFHE	14657000	2385100	156
Q8TD19	978	Serine/threonine-protein kinase Nek9	NEK9	0.998821	6.87E-05	85.097	S	DSDSWCLLGTDSCRPSL______________	2634300	1167300	4463
P55196	1107	Afadin	MLLT4	1	0.00050358	85.054	S	QGAIYHGLATLLNQPSPMMQRISDRRGSGKP	0	0	1868
Q13112	409	Chromatin assembly factor 1 subunit B	CHAF1B	0.783752	0.00579594	85.054	S	TPDTAKKTKSQTHRGSSPGPRPVEGTPASRT	0	0	2406
Q9HD15	69	Steroid receptor RNA activator 1	SRA1	0.439384	0.000790734	85.054	S	AAPQDGSPRVPASETSPGPPPMGPPPPSSKA	0	0	5877
Q2KHR3	1213	Glutamine and serine-rich protein 1	QSER1	0.92084	5.41E-05	85.013	S	TTPTPLVSETGGNSPSDKVDNELKNLEHLSS	3995500	2128000	3040
Q92538	1311	Golgi-specific brefeldin A-resistance guanine nucleotide exc	GBF1	0.920272	7.10E-05	85.013	S	AQSDSELPSYHQNDVSLDRGYTSDSEVYTDH	1609200	867680	4627
Q9BW85	220	Coiled-coil domain-containing protein 94	CCDC94	1	0.000483324	85.013	S	RRLLEDSDSEDEAAPSPLQPALRPNPTAILD	918460	952910	5427
O15541	52	RING finger protein 113A	RNF113A	0.999901	0.00345837	85.013	T	DPEPGESGSSSDEGCTVVRPEKKRVTHNPMI	0	0	7160
O75381	265	Peroxisomal membrane protein PEX14	PEX14	0.427752	1.06E-06	85	S	PSSPAAVNHHSSSDISPVSNESTSSSPGKEG	0	0	614
Q9UK58	338	Cyclin-L1	CCNL1	0.959479	0.000139806	84.993	S	DGTPALSTLGGFSPASKPSSPREVKAEEKSP	15899000	9369800	6420
P04637	18	Cellular tumor antigen p53	TP53	0.780173	0.000127486	84.946	T	EPQSDPSVEPPLSQETFSDLWKLLPENNVLS	0	0	7258
Q92733	261	Proline-rich protein PRCC	PRCC	0.721116	0.000232595	84.878	T	KAAAKSAALQVTKQITQEEDDSDEEVAPENF	32572000	0	7943
Q5THJ4	1765	Vacuolar protein sorting-associated protein 13D	VPS13D	0.742668	0.0307414	84.859	S	QKEVQDKDYPLTPPPSPTVDEPKILVGKSKF	0	494630	3307
Q03164	3036	Histone-lysine N-methyltransferase 2A;MLL cleavage product N	KMT2A	0.87276	0.000512237	84.829	S	DLTRNSSTPGLQVPVSPTVPIQNQKYVPNST	3951800	3700900	2141
Q9UKS7	11	Zinc finger protein Helios	IKZF2	0.734189	0.00683784	84.829	T	_____METEAIDGYITCDNELSPEREHSNMA	439730	0	8231
Q8IYB3	797	Serine/arginine repetitive matrix protein 1	SRRM1	0.983394	0.00532191	84.821	S	PAVPVKKAKSPTPSPSPPRNSDQEGGGKKKK	2229300	169950	4168
P29966	170	Myristoylated alanine-rich C-kinase substrate	MARCKS	1	0.0169515	84.817	S	KKRFSFKKSFKLSGFSFKKNKKEAGEGGEAE	1609400	701570	1339
Q96QC0	313	Serine/threonine-protein phosphatase 1 regulatory subunit 1(	PPP1R10	0.992793	0.0100219	84.817	S	APVPGIKIKKKKKVLSPTAAKPSPFEGKTST	4608200	1227800	5078
O95067	92	G2/mitotic-specific cyclin-B2	CCNB2	0.996802	0.0164557	84.734	S	SVKPVQMEKLAPKGPSPTPEDVSMKEENLCQ	2016700	2552200	725
O75376	2120	Nuclear receptor corepressor 1	NCOR1	1	0.00631495	84.718	S	SQAQSVHHQRPGSRVSPENLVDKSRGSRPGK	1693500	1311900	605
Q96C34	71	RUN domain-containing protein 1	RUNDC1	1	3.45E-05	84.68	S	FLEEATAEEPGAAPGSPPDSPGRTLRRLRAE	2302500	3510500	4818
Q96C34	75	RUN domain-containing protein 1	RUNDC1	1	3.45E-05	84.68	S	ATAEEPGAAPGSPPDSPGRTLRRLRAERRRL	2302500	3510500	4819
Q6ZRS2	562	Helicase SRCAP	SRCAP	0.999998	0.000162361	84.665	S	DDFGVEYLLARDEEQSEADAGSGPPTPGPTT	2346400	1533000	3659
Q8WYQ5	8	Microprocessor complex subunit DGCR8	DGCR8	0.951272	0.000170145	84.66	S	_________METDESPSPLPCGPAGEAVMESR	893140	856830	4613
Q6P4Q7	664	Metal transporter CNNM4	CNNM4	0.977884	0.00633564	84.658	S	SDRSPAHPTPLSRSASLSYPDRTDVSTAATL	825450	443490	3524
Q9Y3B9	43	RRP15-like protein	RRP15	0.433947	3.22E-05	84.646	S	TGAVASVLEDEATDTSDSEGSCGSEKDHFYS	0	0	6850
Q6DD87	46	Zinc finger protein 787	ZNF787	0.99931	0.0101069	84.62	S	IMDDDDVPSWPPTKLSPPQSAPPAGPPPRPR	2711800	2423500	3445

Q92558	313	Wiskott-Aldrich syndrome protein family member 1	WASF1	0.695033	0.000204048	84.581	T	SSATGLIENRPQSPATGRTPVFVSPTPPPPP	5867000	2794000	7933
Q7L9B9	225	Endonuclease/exonuclease/phosphatase family domain-containir	EEPD1	0.621837	2.27E-05	84.576	S	GLTFTAKPHPSPTSLSLQSEDLDLPPGGPTQ	672170	475910	3733
Q7L9B9	228	Endonuclease/exonuclease/phosphatase family domain-containir	EEPD1	0.946574	2.27E-05	84.576	S	FTAKPHPSPTSLSLQSEDLDLPPGGPTQIIS	672170	475910	3734
Q14694	365	Ubiquitin carboxyl-terminal hydrolase 10	USP10	0.985436	0.00875412	84.566	S	KPSSSSPVAYVETKYSPPAPSPLVSEKQVEV	2207700	1857500	2808
P50851	1133	Lipopolysaccharide-responsive and beige-like anchor protein	LRBA	0.992666	1.10E-05	84.515	S	SPTEEANLPTELQDNSLSPAASEAGEKLDMF	1490100	1093800	1768
O14924	849	Regulator of G-protein signaling 12	RGS12	0.490867	0.0101603	84.508	S	AEVEGRALPDSQQVPSSPASKHSLGSDHSSV	0	0	217
O43426	1057	Synaptojanin-1	SYNJ1	0.446558	0.000738892	84.499	S	PSSSPRTSPCQSPTISEGPVPSLPIRPSRAP	0	0	383
O43426	1048	Synaptojanin-1	SYNJ1	0.499877	0.000738892	84.499	T	SSSSGLGTSPSSSPRTSPCQSPTISEGPVPS	0	0	7171
Q9Y3R0	43	Glutamate receptor-interacting protein 1	GRIP1	1	0.0262734	84.479	S	SQTKPPDGALAVRRQSIPEEFKGSTVVELMK	0	0	6864
Q9UGU0	1518	Transcription factor 20	TCF20	0.755237	0.0221601	84.479	T	LAPEANPKAEEKENDTVTISPKQEGFPPKGY	204620	367240	8207
Q6VY07	531	Phosphofurin acidic cluster sorting protein 1	PACS1	0.72416	0.00177113	84.436	S	RQLSKPLSERTNSSDSERSPDLGHSTQIPRK	0	1583800	3608
Q9Y608	333	Leucine-rich repeat flightless-interacting protein 2	LRRFIP2	0.25	0.00112569	84.436	S	PLSGNSSRRGSGDTSSLIDPDTSLSELRDIY	0	0	6974
P33316	88	Deoxyuridine 5'-triphosphate nucleotidohydrolase, mitochond	DUT	1	0.0264436	84.403	S	VGAAGWKGELPKAGGSPAPGPETPAISPSKR	397180	280030	1364
Q9P246	523	Stromal interaction molecule 2	STIM2	0.981258	0.00578928	84.365	S	RSSSLCRSRRSIVPSSPQPQRAQLAPHAPHP	1982400	1143000	6213
Q15311	29	RalA-binding protein 1	RALBP1	0.753547	0.00579419	84.352	S	EHRRVEHGSGLTRTPSSEEISPTKFPGLYRT	0	495870	2903
Q8NEL9	731	Phospholipase DDHD1	DDHD1	0.928796	0.000120558	84.327	S	EGISTIPSPVTSPVLSRRHYGESITNIGKAS	14544000	11081000	4372
Q8WV99	173	AN1-type zinc finger protein 2B	ZFAND2B	0.385799	0.00011215	84.327	S	TSTVPSPSQTMPSCTSPSRATTRSPSWTAPP	0	0	4532
Q8WV99	175	AN1-type zinc finger protein 2B	ZFAND2B	0.385799	0.00011215	84.327	S	TVPSPSQTMPSCTSPSRATTRSPSWTAPPVI	0	0	4533
Q9NQ88	157	Fructose-2,6-bisphosphatase TIGAR	TIGAR	0.93406	0.000155698	84.324	S	LILKEADQKEQFSQGSPSNCLETSLAEIFPL	371520	436310	5902
Q02952	381	A-kinase anchor protein 12	AKAP12	0.999992	1.82E-06	84.318	S	HEPRLSAEYEKVELPSEEQVSGSQGPSEEKP	2134000	1616700	2123
P18031	50	Tyrosine-protein phosphatase non-receptor type 1	PTPN1	0.991398	0.00645716	84.31	S	AKLPKNKNRNRYRDVSPFDHSRIKLHQEDND	0	0	1127
Q02156	729	Protein kinase C epsilon type	PRKCE	0.999507	0.00580957	84.31	S	EAIVKQINQEEFKGFSYFGEDLMP_______	391310	218950	2091
P46100	594	Transcriptional regulator ATRX	ATRX	0.999464	0.00613275	84.297	S	TAKVTKELYVKLTPVSLSNSPIKGADCQEVP	2704100	1640300	1552
Q8WUA4	167	General transcription factor 3C polypeptide 2	GTF3C2	0.994868	0.0262065	84.297	S	LLLKLSKDLDRPESQSPKRPPEDFETPSGER	0	171270	4505
Q9UHB7	1058	AF4/FMR2 family member 4	AFF4	1	0.00483342	84.297	S	SPGLGSKAVGMPSPVSPKLSPGNSGNYSSGA	2489800	1934600	6335
Q99590	344	Protein SCAF11	SCAF11	0.639081	2.17E-05	84.291	S	AETASQSQRSPISDNSGCDAPGNSNPSLSVP	9039900	0	5223
Q8IXT5	575	RNA-binding protein 12B	RBM12B	1	0.0172778	84.244	S	HSSEDFRFPPEDFRHSPEDFRRPREEDFRRP	0	597840	4112
Q9NRA8	374	Eukaryotic translation initiation factor 4E transporter	EIF4ENIF1	0.94469	1.44E-06	84.214	S	HEELERLAGLEQAILSPGQNSGNYFAPIPLE	2209400	1226500	5954
Q8NFH5	66	Nucleoporin NUP53	NUP35	1	0.00374168	84.208	S	PRSISGPSVGVMEMRSPLLAGGSPPQPVVPA	1244800	391300	4397
O60231	160	Putative pre-mRNA-splicing factor ATP-dependent RNA helicase	DHX16	0.999756	0.0175048	84.188	S	KKKTGGSKQQTEKPESEDEWERTERERLQDL	0	1348900	440
Q14562	460	ATP-dependent RNA helicase DHX8	DHX8	0.999994	0.00649985	84.188	S	EPPFLRGHTKQSMDMSPIKIVKNPDGSLSQA	0	1547300	2744
P49792	2626	E3 SUMO-protein ligase RanBP2	RANBP2	0.499983	0.000245011	84.181	S	FKTPEKAKEKKKPEDSPSDDDVLIVYELTPT	0	0	1710
P49792	2628	E3 SUMO-protein ligase RanBP2	RANBP2	0.499983	0.000245011	84.181	S	TPEKAKEKKKPEDSPSDDDVLIVYELTPTAE	0	0	1711
Q9NRZ9	832	Lymphoid-specific helicase	HELLS	0.985081	0.010373	84.173	S	KEKMGIFKILENSEDSPECLF__________	953060	742010	5991
Q9NRZ9	833	Lymphoid-specific helicase	HELLS	0.981019	0.010373	84.173	S	EKMGIFKILENSEDSPECLF___________	953060	0	5992
Q6ZRV2	881	Protein FAM83H	FAM83H	0.999407	0.0240726	84.169	S	ESKGSPTSAYPERKGSPTPGFSTRRGSPTTG	0	732350	3665
Q12948	320	Forkhead box protein C1	FOXC1	0.741776	0.000203849	84.166	S	SQGFSVDNIMTSLRGSPQSAAAELSSGLLAS	623130	0	2380
Q8WXI9	486	Transcriptional repressor p66-beta	GATAD2B	0.957247	0.00101715	84.166	S	QEQEIEQRLQQQAALSPTTAPAVSSVSKQET	943380	944790	4600
Q96F24	113	Nuclear receptor-binding factor 2	NRBF2	0.988259	9.11E-05	84.159	S	LQTSHKPSAEDAEGQSPLSQKYSPSTEKCLP	1454300	568100	4896
Q9UHC6	1303	Contactin-associated protein-like 2	CNTNAP2	0.999998	4.93E-05	84.159	S	RHKGTYHTNEAKGAESAESADAAIMNNDPNF	796330	644580	6347
Q9UHC6	1306	Contactin-associated protein-like 2	CNTNAP2	0.999996	4.93E-05	84.159	S	GTYHTNEAKGAESAESADAAIMNNDPNFTET	796330	644580	6348
Q9Y2W1	232	Thyroid hormone receptor-associated protein 3	THRAP3	0.549736	0.000943936	84.125	S	SESSKPWPDATYGTGSASRASAVSELSPRER	1181500	764220	6816
O95782	655	AP-2 complex subunit alpha-1	AP2A1	0.88439	2.11E-06	84.107	S	INGGMEPTPSTVSTPSPSADLLGLRAAPPPA	1521000	1882600	810
P04637	15	Cellular tumor antigen p53	TP53	0.947274	0.000138018	84.107	S	_MEEPQSDPSVEPPLSQETFSDLWKLLPENN	4963900	16893000	885
P22314	21	Ubiquitin-like modifier-activating enzyme 1	UBA1	0.660473	0.000114324	84.107	S	LSKKRRVSGPDPKPGSNCSPAQSVLSEVPSV	3177100	2959000	1210
O95782	653	AP-2 complex subunit alpha-1	AP2A1	0.953834	2.11E-06	84.107	T	NDINGGMEPTPSTVSTPSPSADLLGLRAAPP	1521000	1882600	7245
Q9UL54	827	Serine/threonine-protein kinase TAO2	TAOK2	0.990489	0.0103796	84.046	S	KQQRILGEESGAPSPSQKHGSLVDEEVWGL	0	0	6464
Q86VQ1	107	Glucocorticoid-induced transcript 1 protein	GLCCI1	0.524773	0.00005835	84.035	S	LGSLPGPGAARGPSPSSPTPPAAAAPAEQAP	0	0	3961
Q92614	2020	Unconventional myosin-XVIIIa	MYO18A	0.982597	0.0018532	84.03	S	SSSPTSYWKSLAPDRSDDEHDPLDNTSRPRY	2160500	0	4665
Q53EL6	93	Programmed cell death protein 4	PDCD4	0.973278	0.0241001	83.998	T	SDSGSDALRSGLTVPTSPKGRLLDRRSRSGK	0	0	7691
Q9H1A4	524	Anaphase-promoting complex subunit 1	ANAPC1	0.891864	0.00156794	83.966	T	VFIPGLPAPSLTMSNTMPRPSTPLDGVSTPK	556510	319690	8086
Q9UIF9	499	Bromodomain adjacent to zinc finger domain protein 2A	BAZ2A	0.559256	0.00803685	83.894	T	VPLTASVTSPKASPVTSPAAAFPTASPANKD	230900	397960	8223
Q9H9J4	1219	Ubiquitin carboxyl-terminal hydrolase 42	USP42	0.983111	0.00297836	83.891	S	SKDKHRDRDSRHQQDSDLSAACSDADLHRHK	1238000	870650	5803
Q9H9J4	1226	Ubiquitin carboxyl-terminal hydrolase 42	USP42	0.999639	0.00297836	83.891	S	RDSRHQQDSDLSAACSDADLHRHKKKKKKKK	1238000	870650	5805
Q9UQ35	1443	Serine/arginine repetitive matrix protein 2	SRRM2	0.881789	0.010545	83.883	S	KDGLPRTPSRRSRSGSSPGLRDGSGTPSRHS	0	1138100	6685
Q8TEM1	1844	Nuclear pore membrane glycoprotein 210	NUP210	1	0.0243362	83.883	T	HTVCTPRDLAVPAALTPRASPGHSPHYFAAS	881070	377140	7908
Q68DK7	126	Male-specific lethal 1 homolog	MSL1	1	0.0055857	83.877	S	QAGIGGEPAAAGAGCSPRPKYQAVLPIQTGS	0	35252	3422
Q9Y657	199	Spindlin-1	SPIN1	0.920862	0.00909175	83.877	S	DYKEGDLRIMPDSNDSPPAEREPGEVVDSLV	1399800	1140300	6985
Q14153	165	Protein FAM53B	FAM53B	0.493059	0.00661152	83.869	S	GSVQRYSNGFSTMQRSSSFSLPSRANVLSSP	0	0	2656
Q14153	166	Protein FAM53B	FAM53B	0.493059	0.00661152	83.869	S	SVQRYSNGFSTMQRSSSFSLPSRANVLSSPC	0	0	2657
Q9UJU6	272	Drebrin-like protein	DBNL	0.428185	0.0105208	83.869	S	PREIFKQKERAMSTTSISSPQPGKLRSPFLQ	0	0	6407
Q9UK76	131	Hematological and neurological expressed 1 protein;Hematolog	HN1	0.997853	9.16E-09	83.854	S	LGQSEEKPVPAAPVPSPVAPAPVPSRRNPPG	11580000	11769000	6430
Q02952	1331	A-kinase anchor protein 12	AKAP12	0.999999	0.010618	83.753	S	KDDALELQSHAKSPPSPVEREMVVQVEREKT	0	449420	2122
Q9H6F5	164	Coiled-coil domain-containing protein 86	CCDC86	0.523554	7.13E-09	83.744	Y	APQHQLPPVPGSPEPYPGQQAPGPEPSQPLL	0	0	8424
Q15398	689	Disks large-associated protein 5	DLGAP5	0.5	0.000211461	83.733	S	EHVKKTLFLSIPESRSSIEDAQCPGLPDLIE	1091000	578080	2914
Q15398	690	Disks large-associated protein 5	DLGAP5	0.5	0.000211461	83.733	S	HVKKTLFLSIPESRSSIEDAQCPGLPDLIEE	1091000	578080	2915
P04049	301	RAF proto-oncogene serine/threonine-protein kinase	RAF1	0.477793	4.76E-05	83.727	S	ESASPSALSSSPNNLSPTGWSQPKTPVPAQR	0	0	871
Q13424	189	Alpha-1-syntrophin	SNTA1	0.997212	0.00946306	83.727	S	PYFKNSTGGTSVGWDSPPASPLQRQPSSPGP	504540	353650	2482
Q13424	193	Alpha-1-syntrophin	SNTA1	0.99997	0.00946306	83.727	S	NSTGGTSVGWDSPPASPLQRQPSSPGPTPRN	504540	353650	2483
P04049	303	RAF proto-oncogene serine/threonine-protein kinase	RAF1	0.477793	4.76E-05	83.727	T	ASPSALSSSPNNLSPTGWSQPKTPVPAQRER	0	0	7254
Q9UPN4	93	Centrosomal protein of 131 kDa	CEP131	0.333727	0.000789801	83.723	T	STTQVSQPRSGSPRPTEPTDFLMLFEGSPSG	0	0	8246
O15085	251	Rho guanine nucleotide exchange factor 11	ARHGEF11	0.993207	0.029522	83.692	S	SLMNRNSVLSDPGLDSPRTSPVIMARVAQHH	0	1562100	285
Q9Y385	251	Ubiquitin-conjugating enzyme E2 J1	UBE2J1	0.990351	0.0176172	83.647	S	SFHQPTQPVAKNTSMSPRQRRAQQQSQRRLS	0	262460	6844
P38159	91	RNA-binding motif protein, X chromosome;RNA-binding motif pr	RBMX	0.852783	0.00583588	83.617	S	GKAIKVEQATKPSFESGRRGPPPPPRSRGPP	2727100	1302400	1449
Q53F19	30	Uncharacterized protein C17orf85	C17orf85	0.666474	0.000361851	83.602	S	APAGPALGLPSPEAESGVDRGEPEPMEVEEG	0	5530000	3137
O60885	470	Bromodomain-containing protein 4	BRD4	0.837535	0.000366635	83.423	S	AKMPDEPEEPVVAVSSPAVPPPTKVVAPPSS	859400	862780	538
Q86X27	293	Ras-specific guanine nucleotide-releasing factor RalGPS2	RALGPS2	0.832905	0.000707582	83.395	S	YKLSLKIEPGTSTPRSAASREDLVGPEVGAS	0	0	4003
Q9BPW4	87	Apolipoprotein L4	APOL4	1	0.0231754	83.37	T	PREEADALYEALKNLTPYVAIEDKDMQQKEQ	514070	283780	8038
Q9BPW4	89	Apolipoprotein L4	APOL4	1	0.0231754	83.37	Y	EEADALYEALKNLTPYVAIEDKDMQQKEQQF	514070	283780	8417
Q12802	790	A-kinase anchor protein 13	AKAP13	0.754676	4.91E-05	83.362	S	PDQAVISDSTFSLANSPGSESVTKDDALSFV	901720	0	2307
P06400	608	Retinoblastoma-associated protein	RB1	0.850047	5.29E-07	83.318	S	NLPLQNNHTAADMYLSPVRSPKKKGSTTRVN	2670300	0	908
P06400	612	Retinoblastoma-associated protein	RB1	0.992244	5.29E-07	83.318	S	QNNHTAADMYLSPVRSPKKKGSTTRVNSTAN	2670300	1855700	909
Q02880	1413	DNA topoisomerase 2-beta	TOP2B	0.998152	5.43E-06	83.318	S	KASPITNDGEDEFVPSDGLDKDEYTFSPGKS	8191500	1758800	2098
Q9NVM9	623	Protein asunder homolog	ASUN	0.972953	0.000271638	83.318	S	ERGKEELAEAEIIKDSPDSPEPPNKKPLVEM	2009400	824120	6044
P49848	634	Transcription initiation factor TFIID subunit 6	TAF6	0.854459	2.36E-05	83.317	S	GGPTSHPSPVPPPASSPSPLSGSALCGGKQE	894780	756490	1739
Q6W2J9	1142	BCL-6 corepressor	BCOR	0.444695	0.000205784	83.251	S	SPTLRVDRKRKVSGDSSHTETTAEEVPEDPL	0	0	3613
Q6W2J9	1143	BCL-6 corepressor	BCOR	0.444695	0.000205784	83.251	S	PTLRVDRKRKVSGDSSHTETTAEEVPEDPLL	0	0	3614
O75533	308	Splicing factor 3B subunit 1	SF3B1	0.570713	0.00537298	83.243	S	WDETPKTERDTPGHGSGWAETPRTDRGGDSI	0	2662500	638
Q9UQ35	1691	Serine/arginine repetitive matrix protein 2	SRRM2	0.578396	0.0224909	83.243	S	PEPKTKSRTPPRRRSSRSSPELTRKARLSRR	289000	1714600	6738
Q9UQ35	1694	Serine/arginine repetitive matrix protein 2	SRRM2	0.947684	0.0224909	83.243	S	KTKSRTPPRRRSSRSSPELTRKARLSRRSRS	289000	1714600	6739
P30622	162	CAP-Gly domain-containing linker protein 1	CLIP1	0.702718	3.45E-06	83.236	S	SPLCTSTASMVSSSPSTPSNIPQKPSQPAAK	2651800	0	1350
Q53T59	139	HCLS1-binding protein 3	HS1BP3	0.999535	0.00585098	83.226	S	ELAGSPELLEFLGTRSPGAAGLTSRDSSVLD	0	727510	3146
Q96B23	67	Uncharacterized protein C18orf25	C18orf25	0.611084	0.00423165	83.221	S	ISSMPCLLMELRRDSSESQLASTESDKPTTG	0	0	4793
Q93100	700	Phosphorylase b kinase regulatory subunit beta	PHKB	0.551401	0.000372531	83.202	S	FEELEPPKHSKVKRQSSTPSAPELGQQPDVN	0	601620	4744
Q9H814	14	Phosphorylated adapter RNA export protein	PHAX	0.97313	1.13E-06	83.202	S	__MALEVGDMEDGQLSDSDSDMTVAPSDRPL	2986600	6260400	5780
P49792;Q	2241;126	E3 SUMO-protein ligase RanBP2;RanBP2-like and GRIP domain-co	RANBP2;RGPI	0.765622	0.000164089	83.167	S	NYDLREDALDDSVSSSSVHASPLASSPVRKN	1891100	0	1704
Q9H6Z4	108	Ran-binding protein 3	RANBP3	0.987021	0.000157655	83.167	S	AGRSAGGSSPEGGEDSDREDGNYCPPVKRER	5091400	2991800	5743
A0FGR8	691	Extended synaptotagmin-2	ESYT2	0.805904	1.56E-06	83.146	S	SVSKEGRKTSIKSHMSGSPGPGGSNTAPSTP	4633600	1528500	4
A0FGR8	693	Extended synaptotagmin-2	ESYT2	0.930989	1.56E-06	83.146	S	SKEGRKTSIKSHMSGSPGPGGSNTAPSTPVI	4633600	1528500	5
Q9BPY3	6	Protein FAM118B	FAM118B	0.355381	0.000108225	83.115	S	__________MASTGSQASDIDEIFGFFNDG	0	0	5295
Q9UN86	225	Ras GTPase-activating protein-binding protein 2	G3BP2	0.333333	0.0288643	83.106	S	KPQVEEKNLEELEEKSTTPPPAEPVSLPQEP	0	0	6540
Q02241	867	Kinesin-like protein KIF23	KIF23	0.989985	0.00431871	83.082	S	LAKCEKYMLTHQELASDGEIETKLIKGDIYK	0	0	2092
Q03164	2198	Histone-lysine N-methyltransferase 2A;MLL cleavage product N	KMT2A	0.448116	0.00589888	83.081	S	LSSGLRSIGSRRHSTSSLSPQRSKLRIMSPM	0	0	2139
Q03164	2199	Histone-lysine N-methyltransferase 2A;MLL cleavage product N	KMT2A	0.448116	0.00589888	83.081	S	SSGLRSIGSRRHSTSSLSPQRSKLRIMSPMR	0	0	2140
Q7L1V2	61	Vacuolar fusion protein MON1 homolog B	MON1B	0.999917	0.000469117	83.065	S	LEETGSKDKDQPPSPSPPPQSEALSSTSRLW	1853500	1115600	3721
Q92934	80	Bcl2-associated agonist of cell death	BAD	0.495981	0.000590804	83.063	T	GAVEIRSRHSSYPAGTEDDEGMGEEPSPFRG	0	0	7953
Q92793	977	CREB-binding protein	CREBBP	0.318162	5.03E-05	83.059	S	SQAAASIDNRVPTPSSVASAETNSQQPGPDV	0	0	4706
Q92793	980	CREB-binding protein	CREBBP	0.318162	5.03E-05	83.059	S	AASIDNRVPTPSSVASAETNSQQPGPDVPVL	0	0	4707
P25789	13	Proteasome subunit alpha type-4	PSMA4	0.999955	0.00689922	83.045	S	___MSRRYDSRTTIFSPEGRLYQVEYAMEAI	327060	236350	1273
Q9BTA9	511	WW domain-containing adapter protein with coiled-coil	WAC	1	0.00591094	83.045	S	QQPVTADKQQGHEPVSPRSLQRSSSQRSPSP	791660	0	5349
P54727	155	UV excision repair protein RAD23 homolog B	RAD23B	0.996554	0.000294792	82.948	T	ASAAKQEKPAEKPAETPVATSPTATDSTSGD	0	6596800	7447
Q92804	226	TATA-binding protein-associated factor 2N	TAF15	0.368997	9.95E-07	82.91	S	NFGGHRDYGPRTDADSESDNSDNNTIFVQGL	0	0	4717
Q92804	222	TATA-binding protein-associated factor 2N	TAF15	0.368997	9.95E-07	82.91	T	GGFKNFGGHRDYGPRTDADSESDNSDNNTIF	0	0	7946
Q63HN8	43	E3 ubiquitin-protein ligase RNF213	RNF213	0.999578	1.37E-06	82.904	S	AAPIADSENNNSTMASASEGEMECGQELKEE	1354300	1306400	3381
Q63HN8	45	E3 ubiquitin-protein ligase RNF213	RNF213	0.999813	1.37E-06	82.904	S	PIADSENNNSTMASASEGEMECGQELKEEGG	1354300	1306400	3382
Q9UPT8	1110	Zinc finger CCCH domain-containing protein 4	ZC3H4	0.776205	5.43E-05	82.885	S	AKPGPAEAPSPTASPSGDASPPATAPYDPRV	0	0	6577
Q9C0B0	389	RING finger protein unkempt homolog	UNK	0.747207	0.00166742	82.877	S	SSLGSPSNLCGSPPGSIRKPPNLEGIVFPGE	2190200	1387400	5510
Q96HC4	313	PDZ and LIM domain protein 5	PDLIM5	0.997204	0.00155212	82.865	S	SEADNTKKANNSQEPSPQLASSVASTRSMPE	1467400	1344100	4943
Q9ULV3	838	Cip1-interacting zinc finger protein	CIZ1	0.999694	0.0286357	82.85	S	GHFENLQKYKAAKNPSPTTRPVSRRCAINAR	2780700	1126100	6512
Q09666	93	Neuroblast differentiation-associated protein AHNAK	AHNAK	1	0.00728524	82.831	S	GHHTVGLKLHRKGDRSPEPGQTWTREVFSSC	0	2528800	2281
Q8N9N8	137	Probable RNA-binding protein EIF1AD	EIF1AD	0.632019	1.75E-06	82.773	S	PELPAEPQLSGEESSSEDDSDLFVNTNRRQY	0	0	4303
Q8N9N8	141	Probable RNA-binding protein EIF1AD	EIF1AD	0.840152	1.75E-06	82.773	S	AEPQLSGEESSSEDDSDLFVNTNRRQYHESE	2261300	0	4304
O75381	234	Peroxisomal membrane protein PEX14	PEX14	0.971056	0.0227141	82.75	S	LKGLLLNRRQFPPSPSAPKIPSWQIPVKSPS	0	491260	613
Q9NRG9	495	Aladin	AAAS	1	0.0181272	82.75	S	AHIPLYFVNAQFPRFSPVLGRAQEPPAGGGG	3670600	2700400	5963
O95295	14	SNARE-associated protein Snapin	SNAPIN	0.998929	0.00050283	82.709	T	__MAGAGSAAVSGAGTPVAGPTGRDLFAEGL	0	0	7235
Q9BUJ2	716	Heterogeneous nuclear ribonucleoprotein U-like protein 1	HNRNPUL1	0.694236	2.39E-06	82.665	S	PPPPQPPPQQPPPPPSYSPARNPPGASTYNK	11820000	0	5379
A0A3B3IU	36;36	RNMT-activating mini protein	FAM103A1	1	0.0144792	82.578	S	ENDKEYQEYLKRPPESPPIVEEWNSRAGGNQ	0	0	0
Q02880	829	DNA topoisomerase 2-beta	TOP2B	0.99778	0.00906493	82.578	T	RLHGGKDAASPRYIFTMLSTLARLLFPAVDD	74276	0	7478
Q02880	826	DNA topoisomerase 2-beta	TOP2B	0.89183	0.00906493	82.578	Y	FGTRLHGGKDAASPRYIFTMLSTLARLLFPA	74276	0	8372
Q96RT1	642	Protein LAP2	ERBB2IP	0.637095	0.000129615	82.56	S	VALSNNKKDDTKETDSLSDEVTHNSNQNNSN	0	531890	5101
Q8WVD3	138	E3 ubiquitin-protein ligase RNF138	RNF138	0.433486	0.000203524	82.56	T	ISQDSVGNSNRSETSTSDNTETYQENTSSSG	0	0	7919
P16383	430	GC-rich sequence DNA-binding factor 2	GCFC2	0.695936	5.58E-05	82.541	S	ARVLSGNCNHQEGTSSDDELPSAEMIDFQKS	614860	675370	1089
P16383	428	GC-rich sequence DNA-binding factor 2	GCFC2	0.540497	5.58E-05	82.541	T	RQARVLSGNCNHQEGTSSDDELPSAEMIDFQ	20615000	20071000	7290
O15014	777	Zinc finger protein 609	ZNF609	0.999989	0.0160363	82.515	S	ESSGDGMKMEGLLNGSSDPHQSRLASIKAEA	0	0	230
O15014	778	Zinc finger protein 609	ZNF609	0.99999	0.0160363	82.515	S	SSGDGMKMEGLLNGSSDPHQSRLASIKAEAD	0	0	231
Q13263	437	Transcription intermediary factor 1-beta	TRIM28	0.447023	3.39E-07	82.503	S	PMAPPRAPGPLSKQGSGSSQPMEVQEGYGFG	0	0	2457
Q13263	439	Transcription intermediary factor 1-beta	TRIM28	0.447023	3.39E-07	82.503	S	APPRAPGPLSKQGSGSSQPMEVQEGYGFGSG	0	0	2458
Q13263	440	Transcription intermediary factor 1-beta	TRIM28	0.333299	3.39E-07	82.503	S	PPRAPGPLSKQGSGSSQPMEVQEGYGFGSGD	0	0	2459
Q16594	149	Transcription initiation factor TFIID subunit 9	TAF9	0.982117	0.00709161	82.494	S	KKASTSAGRITVPRLSVGSVTSRPSPTPTLGT	637320	0	3017

Q14692	552	Ribosome biogenesis protein BMS1 homolog	BMS1	0.999985	0.00822264	82.483	S	ISGSKAAGEGSKAGLSPANCQSDRVNLEKSL	0	628040	2798
O75683	138	Surfeit locus protein 6	SURF6	1	0.00711874	82.417	S	EKIQEARGQGSAKELSPAALEKRRRRKQERD	1485400	1132600	654
Q03164	3515	Histone-lysine N-methyltransferase 2A;MLL cleavage product N	KMT2A	0.984401	0.000125468	82.406	S	ALSSAVQASPTSPGGSPSSPSSGQRSASPSV	4063600	3872600	2132
Q03164	3521	Histone-lysine N-methyltransferase 2A;MLL cleavage product N	KMT2A	0.459186	0.000125468	82.406	S	QASPTSPGGSPSSPSSGQRSASPSVPGPTKP	0	0	2135
Q9H5N1	193	Rab GTPase-binding effector protein 2	RABEP2	0.70626	0.00554992	82.367	S	QEIQRRPRHAPSLHGSTELLPLSRDPSPPLE	2202700	0	5720
Q9H5N1	200	Rab GTPase-binding effector protein 2	RABEP2	0.999999	0.00554992	82.367	S	RHAPSLHGSTELLPLSRDPSPPLEPLEELSG	2202700	1390500	5721
Q8N3F8	418	MICAL-like protein 1	MICALL1	0.73603	0.000441115	82.36	S	ENGGTEEVAQPSPTASLESKPYNPFEEEEED	0	0	4252
Q8N3F8	416	MICAL-like protein 1	MICALL1	0.361164	0.000620332	82.36	T	QVENGGTEEVAQPSPTASLESKPYNPFEEEE	0	0	7874
P49792;Q788;789;7		E3 SUMO-protein ligase RanBP2;RanBP2-like and GRIP domain-co	RANBP2;RGPI	0.675708	0.0183806	82.305	S	SEIKHSTPSPTRYSLSPSKSYKYSPKTPPRW	0	781300	1721
Q9H410	57	Kinetochore-associated protein DSN1 homolog	DSN1	0.673201	0.0114385	82.287	S	LEMNQGVSEERIHLGSSPKKGGNCDLSHQER	5042000	3571500	5651
P35612	11	Beta-adducin	ADD2	0.998836	0.000346618	82.265	S	______MSEETVPEAASPPPPQGQPYFDRFSE	0	0	1412
P54198	610	Protein HIRA	HIRA	1	0.0299677	82.259	S	EQNLVKELRPRDLLESSSDSDEKVPLAKASS	654050	875210	1841
P54198	611	Protein HIRA	HIRA	1	0.0299677	82.259	S	QNLVKELRPRDLLESSSDSDEKVPLAKASSL	654050	875210	1842
P54198	612	Protein HIRA	HIRA	1	0.0299677	82.259	S	NLVKELRPRDLLESSSDSDEKVPLAKASSLS	654050	875210	1843
P54198	614	Protein HIRA	HIRA	1	0.0299677	82.259	S	VKELRPRDLLESSSDSDEKVPLAKASSLSKR	654050	875210	1844
Q96M63	607	Coiled-coil domain-containing protein 114	CCDC114	0.986861	0.00570931	82.259	S	SASSGGHVTFRPVSASSYLGSTGYVGSSRGG	18562000	4368800	5019
Q96M63	608	Coiled-coil domain-containing protein 114	CCDC114	0.744011	0.00570931	82.259	S	ASSGGHVTFRPVSASSYLGSTGYVGSSRGGE	18562000	4368800	5020
Q9BX66	349	Sorbin and SH3 domain-containing protein 1	SORBS1	0.514645	0.00518984	82.259	T	RRVGEQDSAPTQEKPTSPGKAIEKRAKDDSR	0	795700	8060
Q96M63	615	Coiled-coil domain-containing protein 114	CCDC114	0.446367	0.0130639	82.259	Y	TFRPVSASSYLGSTGYVGSSRGGENTEGGVE	0	0	8411
P06400	249	Retinoblastoma-associated protein	RB1	0.999973	0.00659503	82.202	S	LLKEPYKTAVIPINGSPRTPRRGQNRSARIA	4447300	2210000	914
P36871	117	Phosphoglucomutase-1	PGM1	0.978547	0.0192743	82.189	S	IIRKIKAIGGIILTASHNPGGPNGDFGIKFN	743590	529760	1430
P85037	249	Forkhead box protein K1	FOXK1	0.990059	0.000445453	82.189	S	LRSMVSPVPSPTGTISVPNSCPASPRGAGSS	3693700	1696200	2004
Q14684	452	Ribosomal RNA processing protein 1 homolog B	RRP1B	0.79585	2.58E-05	82.177	S	KALKARVAEPGAEATSSTGEESGSEHPPAVP	3109800	0	2788
P11171	709	Protein 4.1	EPB41	0.999251	0.0193734	82.171	S	ESVPEPRPSEWDKRLSTHSPFRTLNINGQIP	0	0	1005
O15047	450	Histone-lysine N-methyltransferase SETD1A	SETD1A	1	8.58E-07	82.151	S	PEPPEPGGGGGGGGPSPEREEVRTSPRPASP	1112800	714350	267
Q7Z4V5	240	Hepatoma-derived growth factor-related protein 2	HDGFRP2	1	0.000466803	82.151	S	KKKAPSASDSDSKADSDGAKPEPVAMARSAS	17950000	6814300	3825
Q7Z4V5	232	Hepatoma-derived growth factor-related protein 2	HDGFRP2	1	0.000466803	82.151	S	RGPLGGRKKKKAPSASDSDSKADSDGAKPEP	0	0	3827
Q7Z4V5	236	Hepatoma-derived growth factor-related protein 2	HDGFRP2	1	0.000466803	82.151	S	GGRKKKKAPSASDSDSKADSDGAKPEPVAMA	0	5511300	3829
O75381	268	Peroxisomal membrane protein PEX14	PEX14	0.487966	1.35E-06	82.139	S	PAAVNHHSSSDISPVSNESTSSSPGKEGHSP	0	0	615
Q99590	780	Protein SCAF11	SCAF11	0.649039	0.00780629	82.102	T	EKVETVSQPSESPKDTIDKTKKPRTRRSRFH	0	0	8026
Q13111	65	Chromatin assembly factor 1 subunit A	CHAF1A	0.999998	0.000107618	82.086	S	ADDMSDDQGTSVQSKSPDLEASLDTLENNCH	999320	379660	2403
O75496	63	Geminin	GMNN	0.391996	0.000403773	82.034	S	LSKRKHRNDHLTSTTSSPGVIVPESSENKNL	0	0	634
Q86WB0	344	Nuclear-interacting partner of ALK	ZC3HC1	1	0.0115831	82.029	S	SQDATFSPGSEQAEKSPGPIVSRTRSWDSSS	3823100	3673100	3982
Q96PC5	1125	Melanoma inhibitory activity protein 2	MIA2	0.693956	0.00327101	82.007	S	PYALDVPNTAFGREHSPYGPSPLGWPSSETR	1366800	0	5052
Q9BX66	350	Sorbin and SH3 domain-containing protein 1	SORBS1	0.498296	0.00525657	81.992	S	RVGEQDSAPTQEKPTSPGKAIEKRAKDDSRR	0	0	5445
Q9NRA2	16	Sialin	SLC17A5	0.456586	0.0038833	81.992	S	MRSPVRDLARNDGEESTDRTPLLPGAPRAEA	0	0	5951
Q9NRA2	17	Sialin	SLC17A5	0.456586	0.0038833	81.992	T	RSPVRDLARNDGEESTDRTPLLPGAPRAEAA	0	0	8152
Q6UUV9	153	CREB-regulated transcription coactivator 1	CRTC1	0.318266	0.000107325	81.974	S	LSPPADTSWRRTNSDSALHQSTMTPTQPESF	0	0	3594
Q6UUV9	172	CREB-regulated transcription coactivator 1	CRTC1	0.746916	0.00287785	81.974	S	QSTMTPTQPESFSSGSQDVHQKRVLLLTVPG	0	0	3598
Q9HB09	245	Bcl-2-like protein 12	BCL2L12	0.470523	0.011615	81.972	S	SDPALRSKLVRLSSDSFARLVELFCSRDDSS	0	0	5833
Q5VUA4	709	Zinc finger protein 318	ZNF318	1	0.0185823	81.95	S	PHPPSPVDPYLLTKNSPPFLKSDHPVGHISG	963570	579350	3344
P22314	24	Ubiquitin-like modifier-activating enzyme 1	UBA1	0.421186	0.000135657	81.949	S	KRRVSGPDPKPGSNCSPAQSVLSEVPSVPTN	0	0	1211
Q14694	76	Ubiquitin carboxyl-terminal hydrolase 10	USP10	0.240065	0.000135657	81.949	S	VDEVIEPSDTLPRTPSYSISSTLNPQAPEFI	0	0	2806
Q14694	78	Ubiquitin carboxyl-terminal hydrolase 10	USP10	0.240065	0.000135657	81.949	S	EVIEPSDTLPRTPSYSISSTLNPQAPEFILG	0	0	2807
Q8IW35	762	Centrosomal protein of 97 kDa	CEP97	0.790293	4.03E-05	81.94	S	DLGDVSEEHGEWNKESSNNEQDNSLLEQYLT	1577200	832530	4050
Q9P0K7	340	Ankycorbin	RAI14	0.77874	1.37E-05	81.888	S	RLSDSTTGADSLLDISSEADQQDLLSLLQAK	639830	796430	6184
Q86U86	10	Protein polybromo-1	PBRM1	0.776415	0.000134485	81.884	S	______MGSKRRRATSPSSSVSGDFDDGHHS	0	0	3919
Q86U86	9	Protein polybromo-1	PBRM1	0.499407	0.000134485	81.884	T	_______MGSKRRRATSPSSSVSGDFDDGHH	0	0	7823
O94804	448	Serine/threonine-protein kinase 10	STK10	0.810273	0.00382203	81.854	S	QGGDLSPAANRSQKASQSRPNSSALETLGGE	0	0	686
Q99728	364	BRCA1-associated RING domain protein 1	BARD1	0.480771	0.000857941	81.807	S	KQTVPSENIPLPECSSPPSCKRKVGGTSGRK	0	0	5266
Q6UUV7	329	CREB-regulated transcription coactivator 3	CRTC3	0.913891	0.00674873	81.789	S	THLGIRSSSGLQSSRSNPSIQATLNKTVLSS	2924400	1753900	3591
Q8N3F8	414	MICAL-like protein 1	MICALL1	0.853118	0.000441115	81.788	S	SRQVENGGTEEVAQPSPTASLESKPYNPFEE	0	0	4251
Q8NDX6	44	Zinc finger protein 740	ZNF740	1	0.0117275	81.771	S	SQIASKQAENGERAGSPDVLRCSSQGHRKDS	890320	546290	4363
O15063	951	Uncharacterized protein KIAA0355	KIAA0355	0.224887	4.08E-05	81.763	T	AAVKQRRKHSSGEQDTSTLPSPPLLTTVEDV	0	0	7149
P49006	180	MARCKS-related protein	MARCKSL1	0.745645	4.04E-08	81.718	S	SEEEAGPQATEPSTPSGPESGPTPASAEQNE	245230	0	1636
Q9BZL4	509	Protein phosphatase 1 regulatory subunit 12C	PPP1R12C	0.999199	0.000244325	81.696	S	CLENSSPPSRIPEPESPAKPNVPTASTAPPA	2665900	1966000	5492
P11171	712	Protein 4.1	EPB41	0.99656	0.0193734	81.676	S	PEPRPSEWDKRLSTHSPFRTLNINGQIPTGE	625800	555600	1006
Q12888	394	Tumor suppressor p53-binding protein 1	TP53BP1	0.61804	0.00323286	81.643	T	SSPTEQEGRQDKPMDTSVLSEEGGEPFQKKL	0	1183300	7513
Q13029	742	PR domain zinc finger protein 2	PRDM2	0.747957	0.00958774	81.619	S	PVTSSRFKRRTSSPPSSPQHSPALRDFGKPS	530690	336330	2389
Q13029	743	PR domain zinc finger protein 2	PRDM2	0.89811	0.00958774	81.619	S	VTSSRFKRRTSSPPSSPQHSPALRDFGKPSD	530690	336330	2390
Q02880	1344	DNA topoisomerase 2-beta	TOP2B	0.956701	0.00024099	81.574	S	VKKRNPWSDDESKSESDLEETEPVVIPRDSL	6242200	1439200	2105
Q06413	240	Myocyte-specific enhancer factor 2C	MEF2C	1	0.00865014	81.565	S	LLVSPGNLNKNMQAKSPPPMNLGMNNRKPDL	906600	683650	2203
Q96E09	276	Protein FAM122A	FAM122A	0.996981	6.07E-05	81.548	S	VSTTTDSPVSPAQAASPFIPLDELSSK____	2180800	2141400	4879
O00303	258	Eukaryotic translation initiation factor 3 subunit F	EIF3F	1	0.0188243	81.525	S	DTERIGVDLIMKTCFSPNRVIGLSSDLQQVG	3182800	1569900	117
P98175	718	RNA-binding protein 10	RBM10	0.814408	0.0308616	81.525	S	LAERQHTSMDLPKLASDDRPSPPRGLVAAYS	0	0	2018
Q1ED39	387	Lysine-rich nucleolar protein 1	KNOP1	0.997299	0.011865	81.525	S	QKLKFLRLMGGFKNLSPSFSRPASTIARPNM	0	571660	3031
Q68DQ2	395	Very large A-kinase anchor protein	CRYBG3	1	0.00848135	81.525	S	CSALLTGSNHRKVPCSPDFQRVTTTENTIKE	909540	480470	3423
Q86UX6	18	Serine/threonine-protein kinase 32C	STK32C	0.989001	0.0115786	81.525	S	SGAERRGSSAAASPGSPPPGRARPAGSDAPS	1610300	0	3943
Q9UQR1	665	Zinc finger protein 148	ZNF148	0.999999	0.0118652	81.525	S	VYATMPINSFRSGMNSPLRTTPDKSHFGLIV	0	0	6767
O60307	348	Microtubule-associated serine/threonine-protein kinase 3	MAST3	0.777391	8.82E-05	81.48	S	VPLSHLEEEQPPAPESPESRALVGQSRRKPC	759740	0	475
Q9H4A3	2011	Serine/threonine-protein kinase WNK1	WNK1	0.827666	0.000178922	81.48	S	KEKPELSEPSHLNGPSSDPEAAFLSRDVDDG	10626000	0	5667
Q14C86	766	GTPase-activating protein and VPS9 domain-containing protei	GAPVD1	0.965288	7.07E-05	81.477	S	TDVREVSSRPSTPGLSVVSGISATSEDIPNK	25578000	0	2842
Q9H0D6	470	5'-3' exoribonuclease 2	XRN2	0.45838	6.04E-05	81.477	S	SNPRQAAYEMRMQNNSSPSISPNTSFTSDGS	0	0	5569
Q9H0D6	478	5'-3' exoribonuclease 2	XRN2	0.398957	6.04E-05	81.477	T	EMRMQNNSSPSISPNTSFTSDGSPSPLGGIK	0	0	8082
Q7Z739	383	YTH domain-containing family protein 3	YTHDF3	0.541598	5.01E-08	81.455	S	NGAGSENFGLGVVPVSASPSSVEVHPVLEKL	0	0	3885
P07814	883	Bifunctional glutamate/proline--tRNA ligase;Glutamate--tRNA	EPRS	0.363762	0.00242763	81.428	S	TGKEYIPGQPPLSQSSDSSPTRNSEPAGLET	0	0	937
P07814	888	Bifunctional glutamate/proline--tRNA ligase;Glutamate--tRNA	EPRS	0.692421	0.00242763	81.428	T	IPGQPPLSQSSDSSPTRNSEPAGLETPEAKV	10445000	0	7266
Q15047	504	Histone-lysine N-methyltransferase SETDB1	SETDB1	0.464168	0.000195913	81.379	S	KKSTSFRPGSVGSGHSSPTSPALSENVSGGK	0	0	2865
Q15047	505	Histone-lysine N-methyltransferase SETDB1	SETDB1	0.58895	0.000195913	81.379	S	KSTSFRPGSVGSGHSSPTSPALSENVSGGKP	0	0	2866
Q96RG2	850	PAS domain-containing serine/threonine-protein kinase	PASK	0.872798	0.000222636	81.329	T	HYAASDRESPGHVPSTLDAGPEDTCPSAEEP	0	0	8001
Q96F44	85	E3 ubiquitin-protein ligase TRIM11	TRIM11	1	0.00540207	81.327	S	PLAKMAEMARRLHPPSPVPQGVCPAHREPLA	0	0	4897
Q9HDC5	475	Junctophilin-1	JPH1	0.997214	0.00640086	81.327	S	TTPPRSPEASPKHSHSPASSPKPLKKQNPSS	3499300	1565700	5883
Q9HDC5	478	Junctophilin-1	JPH1	0.960462	0.00640086	81.327	S	PRSPEASPKHSHSPASSPKPLKKQNPSSGAR	3499300	1565700	5884
Q8NEY1	206	Neuron navigator 1	NAV1	0.557379	0.00112587	81.317	S	AAVSEDGKSDDELLSSKAKAQKSSGPVPSAK	391030	447840	4377
Q8WYQ5	377	Microprocessor complex subunit DGCR8	DGCR8	0.984689	0.00329467	81.317	S	EEREQSSDLTPSGDVSPVKPLSRSAELEFPL	10015000	5799100	4612
Q8WYQ5	371	Microprocessor complex subunit DGCR8	DGCR8	0.988072	0.00329467	81.317	T	KKMKDNEEREQSSDLTPSGDVSPVKPLSRSA	3696300	1515300	7931
Q14684	453	Ribosomal RNA processing protein 1 homolog B	RRP1B	0.536689	2.75E-05	81.312	S	ALKARVAEPGAEATSSTGEESGSEHPPAVPM	3109800	0	2789
Q7Z2Z1	1346	Treslin	TICRR	0.480242	9.59E-05	81.312	S	ECPSPGELDQKEPQMSPSVAASLSCPVPSTP	0	0	3757
Q7Z2Z1	1348	Treslin	TICRR	0.480252	9.59E-05	81.312	S	PSPGELDQKEPQMSPSVAASLSCPVPSTPPE	0	0	3758
Q7Z2Z1	1359	Treslin	TICRR	0.408914	9.59E-05	81.312	S	QMSPSVAASLSCPVPSTPPELSQRATLDTVP	0	0	3761
Q14684	454	Ribosomal RNA processing protein 1 homolog B	RRP1B	0.536689	2.75E-05	81.312	T	LKARVAEPGAEATSSTGEESGSEHPPAVPMH	3109800	0	7615
Q14676	453	Mediator of DNA damage checkpoint protein 1	MDC1	0.426369	0.000953727	81.226	S	RVVLLQRSQTTTERDSDTDVEEEELPVENRE	0	0	2771
Q14676	447	Mediator of DNA damage checkpoint protein 1	MDC1	0.430846	0.000953727	81.226	T	EEDMPQRVVLLQRSQTTTERDSDTDVEEEEL	0	0	7606
Q14676	455	Mediator of DNA damage checkpoint protein 1	MDC1	0.426369	0.000953727	81.226	T	VLLQRSQTTTERDSDTDVEEEELPVENREAV	0	0	7609
Q8WVT3	184	Trafficking protein particle complex subunit 12	TRAPPC12	0.951359	0.0104102	81.185	S	PASGDGFEPQMVKSPSFGGASEASARTPPQV	501280	589890	4545
Q9HB09	113	Bcl-2-like protein 12	BCL2L12	0.999985	0.000161391	81.144	S	LRVLAAFLRRGEAAGSPVPTPPRSPAQEEPT	1953500	1700500	5829
Q9HB09	132	Bcl-2-like protein 12	BCL2L12	0.847614	0.000161391	81.144	S	TPPRSPAQEEPTDFLSRLRRCLPCSLGRGAA	0	1700500	5831
Q9HB09	117	Bcl-2-like protein 12	BCL2L12	0.999597	0.000161391	81.144	T	AAFLRRGEAAGSPVPTPPRSPAQEEPTDFLS	1953500	1700500	8127
Q14160	1560	Protein scribble homolog	SCRIB	0.389053	5.56E-08	81.113	T	TPSPTPVEDLGPQTSTSPGRLSPDFAEELRS	0	0	7583
P31323	114	cAMP-dependent protein kinase type II-beta regulatory subuni	PRKAR2B	0.939972	0.00102407	81.017	S	GAFNAPVINRFTRRASVCAEAYNPDEEEDDA	0	0	1355
O43318	444	Mitogen-activated protein kinase kinase kinase 7	MAP3K7	0.935643	0.0050025	81.017	T	ISGNGQPRRRSIQDLTVTGTEPGQVSSRSSS	0	0	7166
P49792;Q;2237;126;		E3 SUMO-protein ligase RanBP2;RanBP2-like and GRIP domain-co	RANBP2;RGPI	0.444588	0.000216116	81.003	S	LEWDNYDLREDALDDSVSSSSVHASPLASSP	0	0	1702
Q14202	53	Zinc finger MYM-type protein 3	ZMYM3	0.471862	0.000145101	80.994	S	QTAPTRGWAPPGPSPSSGALDLLDTPAGLEK	0	0	2712
Q14202	54	Zinc finger MYM-type protein 3	ZMYM3	0.471862	0.000145101	80.994	S	TAPTRGWAPPGPSPSSGALDLLDTPAGLEKD	0	0	2713
Q9NR30	173	Nucleolar RNA helicase 2	DDX21	0.379018	1.10E-06	80.984	S	EPDCNPSEAASEESNSEIEQEIPVEQKEGAF	0	0	5949
Q8IZL8	1033	Proline-, glutamic acid- and leucine-rich protein 1	PELP1	0.999998	0.00066057	80.975	S	RGADTAPTLAPEALPSQGEVEREGESPAAGP	0	0	4188
Q96A49	268	Synapse-associated protein 1	SYAP1	0.421883	1.73E-06	80.943	T	IKSQLKTQEDEEEISTSPGVSEFVSDAFDAC	0	0	7965
P41212	203	Transcription factor ETV6	ETV6	1	0.0122058	80.916	S	TNHRPSPDPEQRPLRSPLDNMIRRLSPAERA	607880	291230	1486
Q8NAT2	709	Tudor domain-containing protein 5	TDRD5	0.825567	0.00351793	80.916	S	GYPSQQHYFNEDRKISPQSKESELRILDEIP	0	0	4310
Q9Y2W1	55	Thyroid hormone receptor-associated protein 3	THRAP3	0.999829	0.0122058	80.916	S	SRKRRLSSRSRSRSYSPAHNRERNHPRVYQN	2295600	479300	6830
Q96EB6	47	NAD-dependent protein deacetylase sirtuin-1;SirtT1 75 kDa fr	SIRT1	1	0.00895765	80.905	S	PLRKRPRRDGPGLERSPGEPGGAAPEREVPA	0	727510	4886
Q10570	756	Cleavage and polyadenylation specificity factor subunit 1	CPSF1	0.631573	3.82E-07	80.895	S	DDEEEMLYGDSGSLFSPSKEEARRSSQPPAD	147480	678120	2288
Q02952	612	A-kinase anchor protein 12	AKAP12	0.990497	0.00663237	80.871	S	TSDGEKKREGVTPWASFKKMVTPKKRVRRPS	704730	716760	2111
Q9NZ56	396	Formin-2	FMN2	0.99988	3.05E-05	80.85	S	EPEEEAQGPDAPAAASLPGSPAPSQRCFKPY	2196300	1266700	6148
Q9NZ56	400	Formin-2	FMN2	0.788373	3.05E-05	80.85	S	EAQGPDAPAAASLPGSPAPSQRCFKPYPLIT	3368400	2090100	6149
O94913	509	Pre-mRNA cleavage complex 2 protein Pcf11	PCF11	0.999739	0.00637716	80.834	S	SPKRRDRRSPKRRQRSMSPTSTPKAGKIRQS	0	976570	717
O94913	511	Pre-mRNA cleavage complex 2 protein Pcf11	PCF11	0.994049	0.00637716	80.834	S	KRRDRRSPKRRQRSMSPTSTPKAGKIRQSGA	0	976570	718
P49916	210	DNA ligase 3	LIG3	0.993741	0.00899071	80.834	S	KAVVQAKLTTTGQVTSPVKGASFVTSTNPRK	13622000	11408000	1741
Q96T58	2159	Msx2-interacting protein	SPEN	0.889915	6.32E-05	80.832	S	PDKEPEKEDVSASGPSPEATQLAKQMELEQA	0	0	5179
Q8IW35	763	Centrosomal protein of 97 kDa	CEP97	0.600495	4.69E-05	80.813	S	LGDVSEEHGEWNKESSNNEQDNSLLEQYLTS	0	0	4051
O75909	340	Cyclin-K	CCNK	1	0.0192624	80.755	S	PQPSSPRQVKRAVVVSPKEENKAAEPPPPKI	2383300	1336500	667
Q8IXK0	618	Polyhomeotic-like protein 2	PHC2	0.546551	0.000382613	80.752	T	QGFLPEKLPQQDHTTTTDSEMEEPYLQESKE	1025600	718560	7854
Q8NE71	140	ATP-binding cassette sub-family F member 1	ABCF1	1	0.0227667	80.746	S	KTKGGNVFAALIQDQSEEEEEEEKHPPKPAK	286260	288870	4365
Q8IZP0	225	Abl interactor 1	ABI1	0.994847	0.00770547	80.737	S	PNDYMTSPARLGSQHSPGRTASLNQRPRTHS	0	1039900	4190
Q92888	10	Rho guanine nucleotide exchange factor 1	ARHGEF1	0.999969	0.00770547	80.737	S	______MEDFARGAASPGPSRPGLVPVSIIG	0	450490	4725
Q13542	44	Eukaryotic translation initiation factor 4E-binding protein	EIF4EBP2	0.356884	1.89E-06	80.729	S	QLPHDYCTTPGGTLFSTTPGGTRIIYDRKFL	0	0	2576
Q92560	401	Ubiquitin carboxyl-terminal hydrolase BAP1	BAP1	0.816966	1.79E-06	80.729	Y	VPVRPPQQYSDDEDDYEDDEEDDVQNTNSAL	0	0	8401
Q07687	227	Homeobox protein DLX-2	DLX2	0.723949	6.72E-08	80.721	S	KSGEIPSEQHPGASASPPCASPPVSAPASWD	1484100	0	2233
Q07687	232	Homeobox protein DLX-2	DLX2	0.975736	6.72E-08	80.721	S	PSEQHPGASASPPCASPPVSAPASWDFGVPQ	1484100	1194400	2234
P46821	1427	Microtubule-associated protein 1B;MAP1B heavy chain;MAP1 lig	MAP1B	1	0.00771635	80.706	S	SFLSADDKASGRGAESPFEEKSGKQGSPDQV	120600	162340	1584
Q99594	10	Transcriptional enhancer factor TEF-5	TEAD3	0.565578	0.0182825	80.69	S	______MASNSWNASSSPGEAREDGPEGLDK	0	835500	5227
Q14683	956	Structural maintenance of chromosomes protein 1A	SMC1A	0.847694	0.000205421	80.683	S	PLSKGTMDDISQEEGSSQGEDSVSGSQRISS	0	0	2777
Q9BQQ3	243	Golgi reassembly-stacking protein 1	GORASP1	0.411573	1.51E-07	80.652	S	PPDALPPGPTPEDSPSLETGSRQSDYMEALL	0	0	5318
Q9BQQ3	216	Golgi reassembly-stacking protein 1	GORASP1	0.965184	1.51E-07	80.652	T	RIPTQPPSYHKKPPGTPPPSALPLGAPPPDA	1263700	396480	8042
O95801	10	Tetratricopeptide repeat protein 4	TTC4	0.788967	0.000779	80.585	S	______MEQPGQDPTSDDVMDSFLEKFQSQP	607440	0	823
O95801	9	Tetratricopeptide repeat protein 4	TTC4	0.499937	0.000779	80.585	T	_______MEQPGQDPTSDDVMDSFLEKFQSQ	607440	0	7246
Q9H3P2	363	Negative elongation factor A	NELFA	0.997604	0.00086371	80.541	S	SSREASRPPEEPSAPSPTLPAQFKQRAPMYN	2109800	2366600	5638
Q9HCN4	314	GPN-loop GTPase 1	GPN1	0.989982	0.00558447	80.534	S	MGSVALDAGTAKDSLSPVLHPSDLILTRGTL	0	0	5874
Q9UPP1	854	Histone lysine demethylase PHF8	PHF8	0.595286	0.0017524	80.508	S	DSLGACFKDAEYIYPSLESDDDDPALKSRPK	0	0	6564

Q86T24	241	Transcriptional regulator Kaiso	ZBTB33	0.663584	3.70E-08	80.465	T	ISDVAPSASNNSPPLTNITPTQKLPTPVNQA	763500	1450500	7821
Q13136	708	Liprin-alpha-1	PPFIA1	1	0.0194289	80.462	S	SPPGSGRSTPRRIPHSPAREVDRLGVMTLLP	1633700	1156400	2415
Q96QE3	817	ATPase family AAA domain-containing protein 5	ATAD5	0.676243	0.00658072	80.459	S	KAQKAADPVPSFDESSQDTSEKSQDCDVQCK	450740	236180	5081
Q9NRN7	258	L-aminoadipate-semialdehyde dehydrogenase-phosphopantetheiny	AASDHPPT	0.998326	0.00765043	80.459	S	VALRKPDGSRHQDVPSQDDSKPTQRQFTILN	0	0	5975
P54646	377	5'-AMP-activated protein kinase catalytic subunit alpha-2	PRKAA2	1	0.00677036	80.455	S	GLKPHPERMPPLIADSPKARCPLDALNTTKP	701800	486830	1850
Q01804	545	OTU domain-containing protein 4	OTUD4	0.572407	0.00780416	80.455	S	PSTLENITDDKYATVSSPSKSKKLECPSPAE	0	860940	2078
Q01804	546	OTU domain-containing protein 4	OTUD4	0.800925	0.00780416	80.455	S	STLENITDDKYATVSSPSKSKKLECPSPAEQ	1161200	0	2079
Q9Y4G8	1123	Rap guanine nucleotide exchange factor 2	RAPGEF2	0.593318	0.000144304	80.415	S	SNASSQLSSPPTSPQSSPRKGYTLAPSGTVD	736100	0	6922
Q9Y4G8	1124	Rap guanine nucleotide exchange factor 2	RAPGEF2	0.975565	0.000144304	80.415	S	NASSQLSSPPTSPQSSPRKGYTLAPSGTVDN	736100	0	6923
Q96D71	535	RalBP1-associated Eps domain-containing protein 1	REPS1	0.692722	0.000563168	80.406	S	TSDPEQIGSNVTRQRSHSGTSPDNTAPPPPP	1080200	0	4845
Q6WCQ1	362	Myosin phosphatase Rho-interacting protein	MPRIP	0.990539	0.000290679	80.382	S	FTNEAPPAPLPDASASPLSPHRRAKSLDRRS	1207400	86870	3627
Q6WCQ1	365	Myosin phosphatase Rho-interacting protein	MPRIP	0.908674	0.000290679	80.382	S	EAPPAPLPDASASPLSPHRRAKSLDRRSTEP	2334800	1067400	3628
Q9H0S4	9	Probable ATP-dependent RNA helicase DDX47	DDX47	0.569804	0.000235747	80.382	S	_______MAAPEEHDSPTEASQPIVEEEETK	0	2608400	5583
Q01543	241	Friend leukemia integration 1 transcription factor	FLI1	0.999999	0.00920665	80.371	S	RRGAWGNNMNSGLNKSPPLGGAQTISKNTEQ	1270500	820540	2071
Q12948	235	Forkhead box protein C1	FOXC1	0.954124	3.15E-05	80.359	S	PVRIQDIKTENGTCPSPPQPLSPAAALGSGS	0	1449400	2383
Q12948	241	Forkhead box protein C1	FOXC1	0.957137	3.15E-05	80.359	S	IKTENGTCPSPPQPLSPAAALGSGSAAAVPK	0	1449400	2384
Q969L4	58	U7 snRNA-associated Sm-like protein LSm10	LSM10	1	0.013873	80.318	T	IDNVDAFMNIRLAKVTYTDRWGHQVKLDDLF	0	0	7957
Q969L4	60	U7 snRNA-associated Sm-like protein LSm10	LSM10	1	0.013873	80.318	T	NVDAFMNIRLAKVTYTDRWGHQVKLDDLFVT	0	0	7958
Q969L4	59	U7 snRNA-associated Sm-like protein LSm10	LSM10	1	0.013873	80.318	Y	DNVDAFMNIRLAKVTYTDRWGHQVKLDDLFV	0	0	8406
Q96JM3	217	Chromosome alignment-maintaining phosphoprotein 1	CHAMP1	0.549017	0.000855345	80.316	S	VPSPEPQKPAPVSPESVKATLSNPKPQKQSH	0	0	4983
Q04727	205	Transducin-like enhancer protein 4	TLE4	0.477537	0.00606761	80.312	S	HHDNDHQRDRDSIKSSSVSPSASFRGAEKHR	0	0	2179
Q96JM3	405	Chromosome alignment-maintaining phosphoprotein 1	CHAMP1	0.997273	0.00729919	80.312	S	WKSGPPELRKTAPTLSPEHWKAVPPVSPELR	1355000	977040	4996
Q9Y283	614	Inversin	INVS	0.831809	0.000576206	80.236	S	EENKRKEAEQQKGRRSPDSCRPQALPCLPST	1788000	1242100	6770
Q9NYV4	303	Cyclin-dependent kinase 12	CDK12	0.993425	0.0195604	80.231	S	STRSPSPYSRRQRSVSPYSRRRSSSYERSGS	512310	177020	6139
O95155	88	Ubiquitin conjugation factor E4 B	UBE4B	0.645463	6.20E-05	80.171	S	AHRSQSSEGVSSLSSSPSNSLETQSQSLSRS	1646000	0	737
Q15424	194	Scaffold attachment factor B1	SAFB	0.701372	1.88E-05	80.132	T	QEHAIEDKETINNLDTSSSDFTILQEIEEPS	413830	199010	7641
Q8WVM7	17	Cohesin subunit SA-1	STAG1	0.604797	1.35E-06	80.132	T	ITSELPVLQDSTNETTAHSDAGSELEETEVK	0	0	7920
Q9H307	100	Pinin	PNN	0.999999	0.000474333	80.109	S	RLGGERRTRRESRQESDPEDDDVKKPALQSS	2309600	2728700	5633
Q8TC07	677	TBC1 domain family member 15	TBC1D15	0.499999	0.00608318	80.102	T	ALPTLSASGARNDSPTQIPVSSDVCRLTPA_	0	557670	7905
Q7Z4S6	854	Kinesin-like protein KIF21A	KIF21A	0.428546	0.000240296	80.031	S	PMSDKVAGKVTRKLSSSDAPAQDTGSSAAAV	0	0	3823
Q7Z4S6	855	Kinesin-like protein KIF21A	KIF21A	0.428546	0.000240296	80.031	S	MSDKVAGKVTRKLSSSDAPAQDTGSSAAAVE	0	0	3824
P15924	2101	Desmoplakin	DSP	0.999961	0.0178689	80.016	S	IYAAEKAITGFDDPFSGKTVSVSEAIKKNLI	0	95907	1075
P15924	2108	Desmoplakin	DSP	0.520435	0.0178689	80.016	S	ITGFDDPFSGKTVSVSEAIKKNLIDRETGMR	0	95907	1076
P15924	2094	Desmoplakin	DSP	1	0.0178689	80.016	T	DFDDRQQIYAAEKAITGFDDPFSGKTVSVSE	0	95907	7288
Q9NS56	101	E3 ubiquitin-protein ligase Topors	TOPORS	0.660405	0.0123914	80.014	S	TSKLQQTVPADASPDSKCPICLDRFDNVSYL	2506000	0	5995
Q13541	36	Eukaryotic translation initiation factor 4E-binding protein	EIF4EBP1	0.388688	2.61E-06	79.993	T	VVLGDGVQLPPGDYSTTPGGTLFSTTPGGTR	0	0	7552
Q12872	886	Splicing factor, suppressor of white-apricot homolog	SFSWAP	0.999525	0.00102263	79.97	S	PSKQAAPRPAAPAAHSAHSASVSPVESRGSS	1153800	227280	2327
Q12872	893	Splicing factor, suppressor of white-apricot homolog	SFSWAP	0.98613	0.00102263	79.97	S	RPAAPAAHSAHSASVSPVESRGSSQERSRGV	3504200	224000	2330
Q14C86	948	GTPase-activating protein and VPS9 domain-containing protei	GAPVD1	0.704411	0.000105137	79.942	S	AAIGATSLVAAPHSSSSSPSKDSSRGETEER	0	0	2835
Q14C86	952	GTPase-activating protein and VPS9 domain-containing protei	GAPVD1	0.636209	0.000105137	79.942	S	ATSLVAAPHSSSSSPSKDSSRGETEERKDSD	0	0	2838
Q9BYV8	119	Centrosomal protein of 41 kDa	CEP41	0.566505	0.000215633	79.935	S	ETTARTNGKGNPGEQSPSPEQFINNAGAGDS	1492200	0	5471
Q9Y320	286	Thioredoxin-related transmembrane protein 2	TMX2	0.70477	0.000262179	79.935	T	GDNIPEEQPVASTPTTVSDGENKKDK_____	0	1022800	8290
Q99700	849	Ataxin-2	ATXN2	0.330577	9.82E-07	79.917	S	DKIEPSAKDSFIENSSSNCTSGSSKPNSPSI	0	0	5245
Q99700	850	Ataxin-2	ATXN2	0.401581	9.82E-07	79.917	S	KIEPSAKDSFIENSSSNCTSGSSKPNSPSIS	0	0	5246
Q96CX2	176	BTB/POZ domain-containing protein KCTD12	KCTD12	0.788205	6.60E-08	79.911	S	YSEPEQQEGASAGAPSPTLELASRSPSGGAA	1812500	29805000	4831
A8MWK0	57	Putative fatty acid desaturase 2-like protein FADS2P1	FADS2P1	0.909686	0.00918187	79.886	S	KQEANGKCSTPRKSLSMYTWLEIQRHNHEAD	0	54566000	61
P48634	342	Protein PRRC2A	PRRC2A	1	0.0313648	79.886	S	AGAHEEVDYTEKLKFSDEEDGRDSDEEGAEG	0	0	1627
Q5UIP0	782	Telomere-associated protein RIF1	RIF1	0.999904	0.010414	79.886	S	IDFSPYNIKYQPKVKSPQRPSDWSKKKNEPL	1390400	0	3318
A8MWK0	60	Putative fatty acid desaturase 2-like protein FADS2P1	FADS2P1	0.877507	0.00918187	79.886	T	ANGKCSTPRKSLSMYTWLEIQRHNHEADQLV	0	54566000	7112
A8MWK0	59	Putative fatty acid desaturase 2-like protein FADS2P1	FADS2P1	0.890662	0.00918187	79.886	Y	EANGKCSTPRKSLSMYTWLEIQRHNHEADQL	0	54566000	8339
Q12872	742	Splicing factor, suppressor of white-apricot homolog	SFSWAP	0.585559	0.0127531	79.885	S	GRPLPTLEVKPPDRPSSKSKDPPREEEKEKK	12136000	5045000	2331
Q12872	743	Splicing factor, suppressor of white-apricot homolog	SFSWAP	0.882483	0.0127531	79.885	S	RPLPTLEVKPPDRPSSKSKDPPREEEKEKKK	0	5045000	2332
O43639	92	Cytoplasmic protein NCK2	NCK2	0.418803	0.00141567	79.88	T	LGKTRRKTSARDASPTPSTDAEYPANGSGAD	0	0	7173
Q9Y2W1	240	Thyroid hormone receptor-associated protein 3	THRAP3	0.999996	0.0057879	79.875	S	DATYGTGSASRASAVSELSPRERSPALKSPL	0	200410	6814
Q14166	15	Tubulin--tyrosine ligase-like protein 12	TTLL12	0.499809	9.82E-05	79.873	S	_MEAERGPERRPAERSSPGQTPEEGAQALAE	0	0	2691
Q14166	16	Tubulin--tyrosine ligase-like protein 12	TTLL12	0.499809	9.82E-05	79.873	S	MEAERGPERRPAERSSPGQTPEEGAQALAEF	0	0	2692
Q9UJX6	474	Anaphase-promoting complex subunit 2	ANAPC2	0.634096	1.18E-06	79.873	S	TDPASLETGQDSEDDSGEPEDWVPDPVDADP	3285200	2252200	6416
Q8TB61	427	Adenosine 3'-phospho 5'-phosphosulfate transporter 1	SLC35B2	1	0.00698084	79.82	S	RGRLKQRGKKAVPVESPVQKV__________	0	1817700	4447
Q13480	454	GRB2-associated-binding protein 1	GAB1	0.999997	9.23E-05	79.762	S	SSEELDENYVPMNPNSPPRQHSSSFTEPIQE	2056200	1405500	2547
Q13480	434	GRB2-associated-binding protein 1	GAB1	0.438056	9.23E-05	79.762	T	SLEGFHNHFKVKNVLTVGSVSSEELDENYVP	0	0	7547
Q99569	336	Plakophilin-4	PKP4	0.475592	0.0124252	79.744	S	AQTRVASPSQGQVGSSSPKRSGMTAVPQHLG	0	0	5213
Q9P2G1	737	Ankyrin repeat and IBR domain-containing protein 1	ANKIB1	1	0.00949849	79.744	S	QEFLASVARGVAPADSPEAPRRSFAGGTWDW	0	0	6242
Q9H4L7	37	SWI/SNF-related matrix-associated actin-dependent regulator	SMARCAD1	0.891449	4.44E-07	79.731	S	EATPQPSQPGPSSPISLSAEEENAEGEVSRA	6629200	2471000	5691
Q92538	1300	Golgi-specific brefeldin A-resistance guanine nucleotide exc	GBF1	0.580415	9.94E-05	79.69	S	ATARADAPDAGAQSDSELPSYHQNDVSLDRG	0	0	4626
P04049	259	RAF proto-oncogene serine/threonine-protein kinase	RAF1	0.650049	0.00182313	79.676	S	SPSSEGSLSQRQRSTSTPNVHMVSTTLPVDS	1653400	628160	872
Q92797	1170	Symplekin	SYMPK	0.384559	0.000886694	79.663	S	EEQKLKPGGVGAPSSSSPSPSPSARPGPPPS	0	0	4710
Q86U06	128	Probable RNA-binding protein 23	RBM23	0.999986	0.00808211	79.659	S	SRSRDHRREDRVHYRSPPLATGYRYGHSKSP	2237100	859340	3913
A7MCY6	365	TANK-binding kinase 1-binding protein 1	TBKBP1	1	0.0124692	79.652	S	PSPPARAAPPCPPCQSPVPQRRSPVPPCPSP	1284100	1106200	47
Q8TEJ3	797	SH3 domain-containing RING finger protein 3	SH3RF3	0.999997	0.0111615	79.652	S	MQGAMGMEPLHRKAGSLDLNFTSPSRQAPLS	434280	325480	4476
P46013	2223	Antigen KI-67	MKI67	0.999973	0.00431762	79.635	S	DKPTTHEKTTKIACRSPQPDPVGTPTIFKPQ	1780400	1741800	1538
Q12857	319	Nuclear factor 1 A-type	NFIA	0.928693	0.000170414	79.635	S	GSQSSGWHEVEPGMPSPTTLKKSEKSGFSSP	3404000	1855300	2320
Q96D71	558	RalBP1-associated Eps domain-containing protein 1	REPS1	0.499999	0.000591624	79.635	S	NTAPPPPPPRPQPSHSRSSSLDMNRTFTVTT	1022000	0	4848
Q9H981	412	Actin-related protein 8	ACTR8	0.999999	0.00097063	79.589	S	TFGIVGQKMTTLQHRSQGDPEDPHDEHYLLA	1665700	1147600	5800
Q9NRR5	114	Ubiquilin-4	UBQLN4	0.619974	2.75E-09	79.574	S	PSTPDPASAPSTTPASPATPAQPSTSGSASS	0	0	5980
Q9NRR5	101	Ubiquilin-4	UBQLN4	0.342112	2.75E-09	79.574	T	QKAQDPAAATASSPSTPDPASAPSTTPASPA	0	0	8156
Q15942	202	Zyxin	ZYX	0.483259	1.49E-05	79.553	S	TKPAAGGTAPLPPWKSPSSSQPLPQVPAPAQ	0	0	2998
Q15942	204	Zyxin	ZYX	0.483259	1.49E-05	79.553	S	PAAGGTAPLPPWKSPSSSQPLPQVPAPAQSQ	0	0	2999
Q15942	205	Zyxin	ZYX	0.301932	1.49E-05	79.553	S	AAGGTAPLPPWKSPSSSQPLPQVPAPAQSQT	0	0	3000
O15523;O(	592;594	ATP-dependent RNA helicase DDX3Y;ATP-dependent RNA helicase	DDX3Y;DDX3	1	0.0130048	79.489	S	HYKGGSRGRSKSNRFSGGFGARDYRQSSGSS	859770	520850	138
O60825	483	6-phosphofructo-2-kinase/fructose-2,6-bisphosphatase 2;6-pho	PFKFB2	0.998795	0.0199825	79.489	S	TPLSSSNTIRRPRNYSVGSRPLKPLSPLRAQ	0	510950	524
Q7Z460	647	CLIP-associating protein 1	CLASP1	0.967858	0.00787562	79.471	S	GSYASLGRIRTRRQSSGSATNVASTPDNRGR	3070500	110330	3810
Q8IVJ1	2	Solute carrier family 41 member 1	SLC41A1	0.994134	0.0107277	79.469	S	_______________MSSKPEPKDVHQLNGTG	0	0	4046
Q99081	388	Transcription factor 12	TCF12	0.656242	0.000719428	79.445	S	TSQWPRPGGQAPSSPSYENSLHSLQSRMEDR	0	0	5194
Q3YEC7	468	Rab-like protein 6	RABL6	0.824515	2.18E-05	79.426	T	GSPPLPAGPVPSQDITLSSEEEAEVAAPTKG	0	0	7673
Q96B36	97	Proline-rich AKT1 substrate 1	AKT1S1	0.533491	2.76E-05	79.426	T	PAPQPPSPTPSPPRPTLAREDNEEDEDEPTE	0	2659200	7968
Q8WUB8	301	PHD finger protein 10	PHF10	0.999967	7.35E-08	79.408	S	PPLDPELPALDSDGDSDDGEDGRGDEKRKNK	6564100	4022300	4514
Q05519	209	Serine/arginine-rich splicing factor 11	SRSF11	0.860076	0.00122284	79.393	S	VDPKLNHVAAGLVSPSLKSDTSSKEIEEAMK	1168500	1836000	2190
Q6UUV9	169	CREB-regulated transcription coactivator 1	CRTC1	0.340924	2.19E-05	79.382	S	ALHQSTMTPTQPESFSSGSQDVHQKRVLLLT	0	0	3596
Q6UUV9	170	CREB-regulated transcription coactivator 1	CRTC1	0.340924	2.19E-05	79.382	S	LHQSTMTPTQPESFSSGSQDVHQKRVLLLTV	0	0	3597
Q15424	200	Scaffold attachment factor B1	SAFB	0.517265	2.37E-05	79.382	T	DKETINNLDTSSSDFTILQEIEEPSLEPENE	0	0	7642
P10636	713	Microtubule-associated protein tau	MAPT	0.999279	0.00269529	79.373	S	NAKAKTDHGAEIVYKSPVVSGDTSPRHLSNV	4422500	2933200	998
O14893	166	Gem-associated protein 2	GEMIN2	0.66094	1.92E-06	79.365	S	EKLCADGAVGPATNESPGIDYVQIGFPPLLS	0	0	214
O14893	163	Gem-associated protein 2	GEMIN2	0.786286	1.92E-06	79.365	T	CLGEKLCADGAVGPATNESPGIDYVQIGFPP	0	0	7134
Q9BWQ6	3	Protein YIPF2	YIPF2	0.783369	2.73E-05	79.36	S	______________MASADELTFHEFEEATNL	0	0	5431
Q9BWQ6	8	Protein YIPF2	YIPF2	0.499296	2.73E-05	79.36	T	_________MASADELTFHEFEEATNLLADTP	0	0	8058
Q9UKG1	403	DCC-interacting protein 13-alpha	APPL1	0.499992	0.00342727	79.346	S	ARVNQSALEAVTPSPSFQQRHESLRPAAGQS	0	0	6441
P52948	932	Nuclear pore complex protein Nup98-Nup96;Nuclear pore comple	NUP98	1	0.00548186	79.317	S	PLQMALNGKPAPPPQSQSPEVEQLGRVVELD	0	0	1827
P55795;P:	107;107	Heterogeneous nuclear ribonucleoprotein H2;Heterogeneous nuc	HNRNPH2;HN	0.50317	0.00937769	79.317	T	EMDWVLKHTGPNSPDTANDGFVRLRGLPFGC	0	0	7351
Q96H79	256	Zinc finger CCCH-type antiviral protein 1-like	ZC3HAV1L	0.380816	0.000984794	79.311	S	YKHMKLHKMLENTDNSSPSTEHSQGLEKQGV	0	0	4935
P11388	1391	DNA topoisomerase 2-alpha	TOP2A	0.543739	0.00010988	79.237	S	VSDLEADDVKGSVPLSSSPPATHFPDETEIT	661160	1066600	1016
Q5JSZ5	1453	Protein PRRC2B	PRRC2B	0.814448	0.00616438	79.23	S	GGFGEKPVRPGGDTSPRYESQQNGTPLKVK	852180	566920	3193
O43572	47	A-kinase anchor protein 10, mitochondrial	AKAP10	0.833286	0.00718613	79.201	S	GKEQEKTSDVKSIKASISVHSPQKSTKNHAL	0	423780	400
Q96P47	443	Arf-GAP with GTPase, ANK repeat and PH domain-containing pro	AGAP3	0.689953	0.00975126	79.201	S	GKRLPRATPATAPGTSPRANGLSVERSNTQL	2980300	0	5046
Q9NSY1	947	BMP-2-inducible protein kinase	BMP2K	0.766193	0.000291834	79.163	S	FGSTPFQPFLTSTSKSESNEDLFGLVPFDEI	1637700	954050	6001
Q9NSY1	949	BMP-2-inducible protein kinase	BMP2K	0.5	0.000291834	79.163	S	STPFQPFLTSTSKSESNEDLFGLVPFDEITG	1637700	0	6002
P19634	693	Sodium/hydrogen exchanger 1	SLC9A1	0.979885	0.0131956	79.148	S	QKINNYLTVPAHKLDSPTMSRARIGSDPLAY	793570	449930	1176
Q01484	3795	Ankyrin-2	ANK2	0.5741	0.00251575	79.148	S	ETSETQKAMIVPSSPSKTPEEVSTPAEEEKL	1559700	1414200	2066
Q6PKG0	868	La-related protein 1	LARP1	0.961423	0.000147858	79.143	S	SEGTPTVGSYGCTPQSLPKFQHPSHELLKEN	4338700	508470	3567
Q6PKG0	845	La-related protein 1	LARP1	0.739629	0.000147858	79.143	T	SHVGWVMDSREHRPRTASISSSPSEGTPTVG	2560000	508470	7766
Q6PKG0	865	La-related protein 1	LARP1	0.94504	0.000147858	79.143	T	SSPSEGTPTVGSYGCTPQSLPKFQHPSHELL	4338700	2086100	7767
Q6NZY4	649	Zinc finger CCHC domain-containing protein 8	ZCCHC8	0.432118	0.0466691	79.07	S	DISNGGSQKLFPADTSPSTATKIHSPIPDMS	0	0	3497
Q96AY2	117	Crossover junction endonuclease EME1	EME1	0.719284	0.00981247	79.07	S	CKFLTHKQLSPEDSSSPVKSVLDHQNNEGAS	430410	292140	4780
Q6NZY4	648	Zinc finger CCHC domain-containing protein 8	ZCCHC8	0.432118	0.0466691	79.07	T	CDISNGGSQKLFPADTSPSTATKIHSPIPDM	0	0	7752
Q86VE0	177	Myb-related transcription factor, partner of profilin	MYPOP	0.725918	0.0077746	79.036	S	EDRRADTSAHSKAGSSSPEPWARPSCTPQEG	667880	0	3946
Q7Z434	163	Mitochondrial antiviral-signaling protein	MAVS	0.576378	4.65E-06	79.016	T	QAPESPGENSEQALQTLSPRAIPRNPDGGPL	0	0	7806
Q15021	1330	Condensin complex subunit 1	NCAPD2	0.453138	0.00187942	79.013	S	SAKKPSTGSRYQPLASTASDNDFVTPEPRRT	0	0	2854
Q9Y3Z3	27	Deoxynucleoside triphosphate triphosphohydrolase SAMHD1	SAMHD1	0.715309	0.000427542	78.991	S	RPRCDDSPRTPSNTPSAEADWSPGLELHPDY	0	6443100	6886
P41212	22	Transcription factor ETV6	ETV6	0.988213	0.00023931	78.975	S	QCSIKQERISYTPPESPVPSYASSTPLHVPV	1077400	732010	1485
Q9BZF1	807	Oxysterol-binding protein-related protein 8	OSBPL8	0.989947	0.000348608	78.975	S	KVAKGYSSPEPDIQDSSGSEAQSVKPSTRRK	11542000	8050300	5487
Q9BZF1	808	Oxysterol-binding protein-related protein 8	OSBPL8	0.98995	0.000348608	78.975	S	VAKGYSSPEPDIQDSSGSEAQSVKPSTRRKK	11542000	8050300	5488
Q9BZF1	810	Oxysterol-binding protein-related protein 8	OSBPL8	0.989948	0.000348608	78.975	S	KGYSSPEPDIQDSSGSEAQSVKPSTRRKKGI	15561000	9456400	5489
P41212	18	Transcription factor ETV6	ETV6	0.5057	0.00023931	78.975	T	ETPAQCSIKQERISYTPPESPVPSYASSTPL	0	0	7375
Q14694	547	Ubiquitin carboxyl-terminal hydrolase 10	USP10	0.9989	0.0133128	78.939	S	LNGLHEEMLNLKKLLSPSNEKLTISNGPKNH	0	1018800	2800
Q9NPI6	315	mRNA-decapping enzyme 1A	DCP1A	0.960018	1.81E-06	78.921	S	TQSNEKHAPTYTIPLSPVLSPTLPAEAPTAQ	3289500	2702100	5893
Q9NPI6	319	mRNA-decapping enzyme 1A	DCP1A	0.964353	1.81E-06	78.921	S	EKHAPTYTIPLSPVLSPTLPAEAPTAQVPPS	3289500	0	5894
Q9Y570	42	Protein phosphatase methylesterase 1	PPME1	0.932125	0.000165639	78.916	S	SGAKMRMGPGRKRDFSPVPWSQYFESMEDVE	1074100	433930	6952
Q15464	310	SH2 domain-containing adapter protein B	SHB	0.657833	6.74E-05	78.837	S	QLYDTPYEPEGQSVDSDSESTVSPRLRESKL	3182800	0	2936
O15231	446	Zinc finger protein 185	ZNF185	0.5	0.000744485	78.794	S	GQGDPAVPAQQPADPSTPERQSSPSGSEQLV	817820	751520	303
P46821	1785	Microtubule-associated protein 1B;MAP1B heavy chain;MAP1 li	MAP1B	0.999998	0.00207446	78.794	S	VQSLEGEKLSPKSDISPLTPRESSPLYSPTF	3163000	1482800	1592
Q99081	385	Transcription factor 12	TCF12	0.315518	0.000744485	78.794	S	LTGTSQWPRPGGQAPSSPSYENSLHSLQSRM	0	0	5192
Q99081	386	Transcription factor 12	TCF12	0.315518	0.000744485	78.794	S	TGTSQWPRPGGQAPSSPSYENSLHSLQSRME	0	0	5193
O15231	447	Zinc finger protein 185	ZNF185	0.5	0.000744485	78.794	T	QGDPAVPAQQPADPSTPERQSSPSGSEQLVR	817820	751520	7158
Q12789	1873	General transcription factor 3C polypeptide 1	GTF3C1	0.980144	0.00170123	78.794	T	RGTKRRASWASENGETDAEGTQMTPAKRPAL	2830400	0	7508
Q13017	1217	Rho GTPase-activating protein 5	ARHGAP5	0.562254	0.00591059	78.794	T	SPVETWKGGIDNPAITSDQELDDKKMKKKTH	1608100	1316300	7520
P78559	1797	Microtubule-associated protein 1A;MAP1A heavy chain;MAP1 li	MAP1A	0.999917	0.0306584	78.78	S	PEEEDKLTRSPFEIISPPASPPEMVGQRVPS	714640	471570	1980
P78559	1801	Microtubule-associated protein 1A;MAP1A heavy chain;MAP1 li	MAP1A	1	0.0306584	78.78	S	DKLTRSPFEIISPPASPPEMVGQRVPSAPGQ	714640	471570	1981
Q8WWM7	306	Ataxin-2-like protein	ATXN2L	0.789198	0.00839259	78.77	S	QRELRAAQLAREIESSPQYRLRIAMENDDGR	0	532530	4560
Q8WUB8	27	PHD finger protein 10	PHF10	0.951217	4.04E-05	78.758	S	SPRPCDSDPATPGAQSPKDDNEDNSNDGTQP	872630	619030	4510
Q9BVC5	184	Ashwin	C2orf49	0.610955	0.00126304	78.758	S	NDAKQNHDLTHRKSPSGPVKSPPLSPVGTTP	0	0	5397

A8CG34;A(939;546;(		Nuclear envelope pore membrane protein POM 121C;Putative nuc	POM121C;POM	0.377725	6.78E-05	78.736	S	TFNIPFGSSAKSPLPSYPGANPQPAFGAAEG	0	0	60
O15156	3	Zinc finger and BTB domain-containing protein 7B	ZBTB7B	0.999993	5.92E-05	78.717	S	______________MGSPEDDLIGIPFPDHSS	375180	463350	291
O43242	69	26S proteasome non-ATPase regulatory subunit 3	PSMD3	1	0.0193628	78.692	T	KTAAAAAEHSQRELDTVTLEDIKEHVKQLEK	1074900	593740	7164
O43242	71	26S proteasome non-ATPase regulatory subunit 3	PSMD3	1	0.0193628	78.692	T	AAAAAEHSQRELDTVTLEDIKEHVKQLEKAV	1074900	593740	7165
Q96CX2	178	BTB/POZ domain-containing protein KCTD12	KCTD12	0.716508	1.12E-07	78.642	T	EPEQQEGASAGAPSPTLELASRSPSGGAAGP	42301000	0	7972
Q9NRR5	97	Ubiquilin-4	UBQLN4	0.42083	2.91E-09	78.639	S	IKTPQKAQDPAAATASSPSTPDPASAPSTTP	0	0	5976
Q9Y2W1	698	Thyroid hormone receptor-associated protein 3	THRAP3	1	0.00634588	78.615	S	PSTFRKHGLAHDEMKSPREPGYKAEGKYKDD	0	0	6822
O75179	1709	Ankyrin repeat domain-containing protein 17	ANKRD17	0.999988	0.0205131	78.556	S	GPSPLSSPNGKLTVASPKRGQKREEGWKEVV	2182500	1145700	586
Q9H4L5	371	Oxysterol-binding protein-related protein 3	OSBPL3	0.419814	0.0299104	78.529	S	AEREKLKQLMEQDASSSPSAQVIGLKNALSS	0	0	5681
Q9H4L5	372	Oxysterol-binding protein-related protein 3	OSBPL3	0.419814	0.0299104	78.529	S	EREKLKQLMEQDASSSPSAQVIGLKNALSSA	0	0	5682
P02794	179	Ferritin heavy chain;Ferritin heavy chain, N-terminally proc	FTH1	0.996488	0.00741468	78.513	S	SGLAEYLFDKHTLGDSDNES____________	1384100	1296100	867
Q92738	714	USP6 N-terminal-like protein	USP6NL	0.424946	0.0267058	78.508	S	EVLPVDTGAGGYSGNSGSPKNGKLIIPPVDY	0	0	4695
Q92738	716	USP6 N-terminal-like protein	USP6NL	0.424946	0.0267058	78.508	S	LPVDTGAGGYSGNSGSPKNGKLIIPPVDYLP	0	0	4696
Q6F5E8	993	Leucine-rich repeat-containing protein 16C	RLTPR	0.621186	0.0106128	78.505	S	EDAEPQAGPSARGSPSPAAPGPPAGPLPRMD	0	1293700	3453
Q76FK4	1099	Nucleolar protein 8	NOL8	0.913335	0.0100754	78.505	S	SEEEDVTEETDHRNSSPGEASLLEKETTRFF	0	942870	3700
Q4VCS5	721	Angiomotin	AMOT	0.527278	0.00148755	78.501	T	RDTTVISHSPNTSYDTALEARIQKEEEEILM	0	3301200	7685
Q9NPI6	321	mRNA-decapping enzyme 1A	DCP1A	0.589658	2.51E-06	78.45	T	HAPTYTIPLSPVLSPTLPAEAPTAQVPPSLP	527450	600200	8138
Q9BW71	100	HIRA-interacting protein 3	HIRIP3	0.861264	0.0132276	78.418	S	PCSDPERKRFRFNSESESGSEASSPDYFGPP	0	904830	5413
Q8NF99	142	Zinc finger protein 397	ZNF397	1	0.000759639	78.401	S	DLEREFDDPGQQVPASPQGPAVPWKDLTCLR	1468700	1325000	4389
O15027	961	Protein transport protein Sec16A	SEC16A	0.470369	8.68E-07	78.343	S	KAGSALPGFANSPAGSTSVVLVPPAHGTLVP	0	0	239
O15027	962	Protein transport protein Sec16A	SEC16A	0.470369	8.68E-07	78.343	T	AGSALPGFANSPAGSTSVVLVPPAHGTLVPD	0	0	7138
P32926	33	Desmoglein-3	DSG3	0.997344	0.012451	78.342	Y	VILVHGELRIETKGQYDEEEMTMQQAKRRQK	3866600	6275500	8361
Q96HA7	719	Tonsoku-like protein	TONSL	1	0.01016	78.324	S	NSTRLPEASQAHVRVSPGQAAPAMARPRRSR	3122200	3470300	4941
Q9NVM9	626	Protein asunder homolog	ASUN	0.992175	0.000681955	78.324	S	KEELAEAEIIKDSPDSPEPPNKKPLVEMDET	0	0	6045
Q96TA1	641	Niban-like protein 1	FAM129B	0.984114	9.35E-07	78.311	S	VQDEEVGLPFEASPESPPPASPDGVTEIRGL	1116000	824780	5189
Q96TA1	646	Niban-like protein 1	FAM129B	0.993315	9.35E-07	78.311	S	VGLPFEASPESPPPASPDGVTEIRGLLAQGL	2179500	1537900	5190
Q8NEN9	521	PDZ domain-containing protein 8	PDZD8	0.897899	0.0080533	78.287	S	RAQNEFKDEAQSLSHSPKRVPTTLSIKPLGA	0	0	4374
Q86U42	90	Polyadenylate-binding protein 2	PABPN1	0.758451	9.61E-09	78.228	S	PRPRAPPGAPGPGPGSGAPGSQEEEEEPGLV	3104200	5040600	3914
Q9UGV2	333	Protein NDRG3	NDRG3	0.554999	0.00772167	78.228	S	SASMTRLARSRTHSTSSSLGSGESPFSRSVT	0	987530	6324
Q9UGV2	332	Protein NDRG3	NDRG3	0.554999	0.00772167	78.228	T	PSASMTRLARSRTHSTSSSLGSGESPFSRSV	0	987530	8211
Q05469	855	Hormone-sensitive lipase	LIPE	0.737661	0.0268093	78.225	S	QKMSEPIAEPMRRSVSEAALAQPQGPLGTDS	201630	0	2187
Q12846	117	Syntaxin-4	STX4	0.94336	0.00248864	78.166	S	KAIEPQKEEADENYNSVNTRMRKTQHGVLSQ	1483100	804160	2317
Q96I25	237	Splicing factor 45	RBM17	0.999916	0.00856679	78.166	T	SPTGPSNSFLANMGGTVAHKIMQKYGFREGQ	0	3424500	7985
Q86UQ4	3008	ATP-binding cassette sub-family A member 13	ABCA13	0.813592	0.0281469	78.149	S	SPNQLNCESLSKNLSSTLESFKSSLENATGQ	0	487280	3936
Q96E09	48	Protein FAM122A	FAM122A	0.911286	0.000186489	78.122	S	RRSNSAPLIHGLSDTSPVFQAEAPSARRNST	1438300	0	4874
P24928	1966	DNA-directed RNA polymerase II subunit RPB1	POLR2A	0.90641	8.80E-07	78.108	S	SPTYSLTSPAISPDDSDEEN____________	0	5908000	1241
Q04721	2078	Neurogenic locus notch homolog protein 2;Notch 2 extracellul	NOTCH2	0.598255	4.89E-05	78.098	S	DEYNVTPSPPGTVLTSALSPVICGPNRSFLS	0	925970	2166
Q8NG31	32	Protein CASC5	CASC5	1	0.020774	78.098	S	RPVRRRHSSILKPPRSPLQDLRGGNERVQES	1671700	965580	4408
Q04721	2077	Neurogenic locus notch homolog protein 2;Notch 2 extracellul	NOTCH2	0.806233	4.89E-05	78.098	T	LDEYNVTPSPPGTVLTSALSPVICGPNRSFL	0	925970	7492
O15085	1457	Rho guanine nucleotide exchange factor 11	ARHGEF11	0.878575	0.000738347	78.088	S	MELAHRELLKSLGGESSGGTTPVGSFHTEAA	1683500	974700	287
Q8WWM7	390	Ataxin-2-like protein	ATXN2L	0.871781	0.00535456	78.088	S	GLSSLPPRGPHHLDNSSPGPGSEARGINGGP	0	468140	4562
O15085	1462	Rho guanine nucleotide exchange factor 11	ARHGEF11	0.959612	0.000738347	78.088	T	RELLKSLGGESSGGTTPVGSFHTEAARWTDG	0	974700	7154
Q9NVR2	233	Integrator complex subunit 10	INTS10	0.55946	0.000526343	77.959	S	NQHQGAQDTSDLMSPSKRSSQKYIIEGLTEK	2277700	1083900	6048
O60315	731	Zinc finger E-box-binding homeobox 2	ZEB2	0.995577	4.82E-05	77.939	S	APNSNPPTKDSLLPRSPVKPMDSITSPSIAE	1310700	562790	476
Q7Z4V5	234	Hepatoma-derived growth factor-related protein 2	HDGFRP2	1	0.000758356	77.917	S	PLGGRKKKKAPSASDSDSKADSDGAKPEPVA	16804000	659700	3828
P43487	18	Ran-specific GTPase-activating protein	RANBP1	0.675606	8.29E-06	77.917	T	AAKDTHEDHDTSTENTDESNHDPQFEPIVSL	0	0	7382
Q5QJE6	434	Deoxynucleotidyltransferase terminal-interacting protein 2	DNTTIP2	0.498895	0.0429369	77.912	S	DFECDTKLYTSAPNTSQGKDNSVLLVLSSDE	0	0	3206
Q5QJE6	433	Deoxynucleotidyltransferase terminal-interacting protein 2	DNTTIP2	0.498895	0.0429369	77.912	T	VDFECDTKLYTSAPNTSQGKDNSVLLVLSSD	0	0	7708
Q9H773	169	dCTP pyrophosphatase 1	DCTPP1	0.440182	3.09E-05	77.907	S	AVGPADIPCDSTGQTST______________	0	0	5747
Q9H773	168	dCTP pyrophosphatase 1	DCTPP1	0.306067	3.09E-05	77.907	T	QAVGPADIPCDSTGQTST_____________	0	0	8108
Q9H773	170	dCTP pyrophosphatase 1	DCTPP1	0.440182	3.09E-05	77.907	T	VGPADIPCDSTGQTST_______________	0	0	8109
Q15648	1023	Mediator of RNA polymerase II transcription subunit 1	MED1	0.581132	0.00624523	77.899	S	KPPKRKKADTEGKSPSHSSSNRPFTPPTSTG	861790	0	2966
Q15648	1025	Mediator of RNA polymerase II transcription subunit 1	MED1	0.325849	0.00624523	77.899	S	PKRKKADTEGKSPSHSSSNRPFTPPTSTGGS	0	0	2967
Q8IY92	1044	Structure-specific endonuclease subunit SLX4	SLX4	1	0.0105595	77.894	S	PPHPCRFLLGPPQGGSPRGSHHTSGSSLSTP	774660	709340	4125
Q9H7Z6	37	Histone acetyltransferase KAT8	KAT8	0.926866	6.44E-08	77.882	S	EPGPGENAAAEGTAPSPGRVSPPTPARGEPE	21381000	16091000	5776
Q9H7Z6	42	Histone acetyltransferase KAT8	KAT8	0.999968	6.44E-08	77.882	S	ENAAAEGTAPSPGRVSPPTPARGEPEVTVEI	22579000	15962000	5777
Q9H7Z6	45	Histone acetyltransferase KAT8	KAT8	0.97698	6.44E-08	77.882	T	AAEGTAPSPGRVSPPTPARGEPEVTVEIGET	3137900	2918800	8118
Q7Z5L9	399	Interferon regulatory factor 2-binding protein 2	IRF2BP2	0.705544	0.000406252	77.873	S	TEGLKIPMTPTSSFVSPPPPTASPHSNRTTP	2339800	0	3847
Q7Z5L9	404	Interferon regulatory factor 2-binding protein 2	IRF2BP2	0.532994	0.000485793	77.873	T	IPMTPTSSFVSPPPPTASPHSNRTTPPEAAQ	492550	745530	7812
Q9UPT8	807	Zinc finger CCCH domain-containing protein 4	ZC3H4	0.463419	8.32E-05	77.838	S	KQDRENEEGDTGNWYSSDEDEGGSSVTSILK	0	0	6572
Q9Y3X0	376	Coiled-coil domain-containing protein 9	CCDC9	0.99466	0.000879245	77.838	S	YSDHDDRWETKEGAASPAPETPQPTSPETSP	1012800	1589600	6881
Q9UBP9	213	PTB domain-containing engulfment adapter protein 1	GULP1	0.549688	0.000289145	77.802	S	QVSAPPAGSMTPKSPSTDIFDMIPFSPISHQ	656110	429730	6269
Q9UBP9	223	PTB domain-containing engulfment adapter protein 1	GULP1	0.838775	0.000289145	77.802	S	TPKSPSTDIFDMIPFSPISHQSSMPTRNGTQ	656110	429730	6270
O60271	279	C-Jun-amino-terminal kinase-interacting protein 4	SPAG9	0.906011	3.94E-05	77.798	S	LSDVSQGGSKATTPASTANSDVATIPTDTPL	1377200	846710	453
O60271	283	C-Jun-amino-terminal kinase-interacting protein 4	SPAG9	0.980598	3.94E-05	77.798	S	SQGGSKATTPASTANSDVATIPTDTPLKEEN	1377200	846710	454
O60271	276	C-Jun-amino-terminal kinase-interacting protein 4	SPAG9	0.547941	3.94E-05	77.798	T	EDELSDVSQGGSKATTPASTANSDVATIPTD	1377200	846710	7176
Q99614	83	Tetratricopeptide repeat protein 1	TTC1	0.998924	0.0275305	77.754	S	ASFEEEPGADKVENKSNEDVNSSELDEEYLI	359220	255670	5231
Q96I99	3	Succinyl-CoA ligase [GDP-forming] subunit beta, mitochondri	SUCLG2	1	0.00367711	77.744	S	______________MASPVAAQAGKLLRALAL	1544200	0	4952
Q92615	524	La-related protein 4B	LARP4B	0.896227	1.17E-06	77.719	S	FTSSQTQSPTPPKPPSPSFELGLSSFPPLPG	0	5021700	4673
Q12872	889	Splicing factor, suppressor of white-apricot homolog	SFSWAP	0.978679	0.00109076	77.689	S	QAAPRPAAPAAHSAHSASVSPVESRGSSQER	3681500	185990	2328
Q12872	891	Splicing factor, suppressor of white-apricot homolog	SFSWAP	0.995408	0.00109076	77.689	S	APRPAAPAAHSAHSASVSPVESRGSSQERSR	2527700	227280	2329
A8CG34;A(935;542;(		Nuclear envelope pore membrane protein POM 121C;Putative nuc	POM121C;POM	0.658783	9.24E-05	77.675	S	TSTPTFNIPFGSSAKSPLPSYPGANPQPAFG	0	0	59
Q53F19	412	Uncharacterized protein C17orf85	C17orf85	0.500784	0.0142707	77.674	S	SDSDEMDYDLELKMISTPSPKKSMKMTMYAD	0	0	3138
Q86UU1	421	Pleckstrin homology-like domain family B member 1	PHLDB1	0.579769	0.0235126	77.674	S	FREPPGSERVLTTSPSRQLVGRTFSDGLATR	0	1103300	3940
Q9UHB7	836	AF4/FMR2 family member 4	AFF4	0.996789	0.00913476	77.674	S	SKDPKTEHGSRKRTISQSSSLKSSSNSNKET	0	602870	6346
Q6WCQ1	360	Myosin phosphatase Rho-interacting protein	MPRIP	0.767292	0.000387877	77.668	S	RDFTNEAPPAPLPDASASPLSPHRRAKSLDR	0	0	3626
Q53F19	415	Uncharacterized protein C17orf85	C17orf85	0.671092	0.0142847	77.662	S	DEMDYDLELKMISTPSPKKSMKMTMYADEVE	1385300	0	3139
Q9UQB8	259	Brain-specific angiogenesis inhibitor 1-associated protein 2	BAIAP2	0.480985	0.0120925	77.66	S	MQQVASNGATLPSALSASKSNLVISDPIPGA	0	0	6753
Q14202	291	Zinc finger MYM-type protein 3	ZMYM3	1	0.0165318	77.597	S	DEDFVPFRPRRSPRMSLRSSVSQRAGRSAVG	0	0	2715
Q14202	294	Zinc finger MYM-type protein 3	ZMYM3	1	0.0165318	77.597	S	FVPFRPRRSPRMSLRSSVSQRAGRSAVGTKM	0	0	2716
Q14202	295	Zinc finger MYM-type protein 3	ZMYM3	1	0.0165318	77.597	S	VPFRPRRSPRMSLRSSVSQRAGRSAVGTKMT	0	0	2717
Q14202	297	Zinc finger MYM-type protein 3	ZMYM3	1	0.0165318	77.597	S	FRPRRSPRMSLRSSVSQRAGRSAVGTKMTCA	0	0	2718
O95628	432	CCR4-NOT transcription complex subunit 4	CNOT4	0.803017	0.000144066	77.575	S	DLIEKELSVQDQPSLSPTSLQNSSSHTTTAK	1523700	1070000	782
P51610	411	Host cell factor 1;HCF N-terminal chain 1;HCF N-terminal cha	HCFC1	0.426772	0.000153885	77.575	S	QLQKYDIPATAATATSPTPNPVPSVPANPPK	0	0	1794
P51610	410	Host cell factor 1;HCF N-terminal chain 1;HCF N-terminal cha	HCFC1	0.426035	0.000153885	77.575	T	LQLQKYDIPATAATATSPTPNPVPSVPANPP	0	0	7440
Q5JSZ5	907	Protein PRRC2B	PRRC2B	0.994837	0.00271122	77.554	S	REGTAFNISSWDKNGSPNKQPSSEPEWTPEP	808960	0	3192
Q96ST3	274	Paired amphipathic helix protein Sin3a	SIN3A	0.545717	0.000296671	77.543	S	HTPASQQTPPLPPYASPRSPPVQPHTPVTIS	1482600	0	5140
O94811	160	Tubulin polymerization-promoting protein	TPPP	0.724107	0.009315	77.533	S	EGKAPIISGVTKAISSPTVSRLTDTTKFTGS	742170	334660	699
Q9BY89	128	Uncharacterized protein KIAA1671	KIAA1671	0.998658	0.0144403	77.533	S	LVGQEVGSGEGPRTSSPLFNKAVFLRPSSST	708140	267190	5467
Q9H792	281	Pseudopodium-enriched atypical kinase 1	PEAK1	0.999698	0.0144403	77.533	S	MRGQPRFANFRANTLSPVRFFVDKKWNTIPL	504020	270990	5749
Q9UJV9	618	Probable ATP-dependent RNA helicase DDX41	DDX41	0.5	0.00832203	77.533	S	TKQVSNIGRKDYLAHSSMDF____________	444480	360830	6409
Q9UJV9	619	Probable ATP-dependent RNA helicase DDX41	DDX41	0.5	0.00832203	77.533	S	KQVSNIGRKDYLAHSSMDF_____________	444480	360830	6410
Q9UBC2	253	Epidermal growth factor receptor substrate 15-like 1	EPS15L1	0.818196	0.00127415	77.505	S	STPSHGSVSSLNSTGSLSPKHSLKQTQPTVN	920540	331850	6260
O60343	106	TBC1 domain family member 4	TBC1D4	0.307542	4.67E-05	77.445	S	LRCVPAPGAGASGGTSPSATQPNPAVFIFEH	0	0	486
O60343	108	TBC1 domain family member 4	TBC1D4	0.307542	4.67E-05	77.445	S	CVPAPGAGASGGTSPSATQPNPAVFIFEHKA	0	0	487
Q9H0D6	473	5'-3' exoribonuclease 2	XRN2	0.725081	7.92E-05	77.445	S	RQAAYEMRMQNNSSPSISPNTSFTSDGSPSP	1808600	917750	5571
O60343	105	TBC1 domain family member 4	TBC1D4	0.307542	4.67E-05	77.445	T	FLRCVPAPGAGASGGTSPSATQPNPAVFIFE	0	0	7181
P49006	165	MARCKS-related protein	MARCKSL1	0.928739	1.41E-06	77.433	S	ESQEPQAKGAEASAASEEEAGPQATEPSTPS	9265200	5285800	1634
O95785	1127	Protein Wiz	WIZ	0.583297	0.00949755	77.372	S	KMMGGAGPGSSLEARSPSDLHISPLAKKLPP	839350	569080	818
O95785	1134	Protein Wiz	WIZ	0.999995	0.00949755	77.372	S	PGSSLEARSPSDLHISPLAKKLPPPPGSPLG	839350	569080	819
Q9H5N1	194	Rab GTPase-binding effector protein 2	RABEP2	0.539999	0.00963147	77.357	T	EIQRRPRHAPSLHGSTELLPLSRDPSPPLEP	0	1390500	8105
Q8IYL2	78	Probable tRNA (uracil-O(2)-)-methyltransferase	TRMT44	1	0.0134276	77.324	S	AGSEQKERGPGPGQGSPGGGPGPRSLSGPEQ	0	576600	4175
P18887	416	DNA repair protein XRCC1	XRCC1	0.514382	0.00970299	77.287	S	YLMAGPGSSSEEDEASHSGGSGDEAPKLPQK	0	0	1159
P18887	421	DNA repair protein XRCC1	XRCC1	0.883507	0.00970299	77.287	S	PGSSSEEDEASHSGGSGDEAPKLPQKQPQTK	0	0	1160
Q07960	51	Rho GTPase-activating protein 1	ARHGAP1	0.73384	0.0093613	77.279	S	DEMPDFPKSDDSKSSSPELVTHLKWDDPYYD	1749600	0	2241
Q14978	643	Nucleolar and coiled-body phosphoprotein 1	NOLC1	1	0.00964022	77.279	S	VREEEIEVDSRVADNSFDAKRGAAGDWGERA	0	485310	2826
O15027	1436	Protein transport protein Sec16A	SEC16A	0.779217	1.51E-05	77.276	S	YGYPADTVWPAMEQVSSRPTSPEKFSVPHVC	3852600	2422100	253
O15027	1437	Protein transport protein Sec16A	SEC16A	0.50059	1.51E-05	77.276	S	GYPADTVWPAMEQVSSRPTSPEKFSVPHVCA	3852600	0	254
O15027	1441	Protein transport protein Sec16A	SEC16A	0.499987	1.51E-05	77.276	S	DTVWPAMEQVSSRPTSPEKFSVPHVCARFGP	3852600	0	255
O15027	1440	Protein transport protein Sec16A	SEC16A	0.499987	1.51E-05	77.276	T	ADTVWPAMEQVSSRPTSPEKFSVPHVCARFG	3852600	0	7143
A7MCY6	372	TANK-binding kinase 1-binding protein 1	TBKBP1	1	0.00967977	77.24	S	APPCPPCQSPVPQRRSPVPPCPSPQQRRSPA	701410	0	52
A7MCY6	379	TANK-binding kinase 1-binding protein 1	TBKBP1	1	0.00967977	77.24	S	QSPVPQRRSPVPPCPSPQQRRSPASPSCPSP	701410	0	53
Q99638	326	Cell cycle checkpoint control protein RAD9A	RAD9A	0.747056	0.014564	77.24	S	ETTIGNEGSRVLPSISLSPGPQPPKSPGPHS	0	0	5241
Q9Y6K9	387	NF-kappa-B essential modulator	IKBKG	1	0.0134501	77.185	S	PAYLSSPLALPSQRRSPPEEPPDFCCPKCQY	758080	454830	7012
Q92615	516	La-related protein 4B	LARP4B	0.451869	2.84E-06	77.175	S	YRKKREEKFTSSQTQSPTPPKPPSPSFELGL	0	0	4672
Q92615	526	La-related protein 4B	LARP4B	0.717381	2.84E-06	77.175	S	SSQTQSPTPPKPPSPSFELGLSSFPPLPGAA	0	839180	4674
Q92615	518	La-related protein 4B	LARP4B	0.972698	2.84E-06	77.175	T	KKREEKFTSSQTQSPTPPKPPSPSFELGLSS	0	4073300	7940
Q96RU3	359	Formin-binding protein 1	FNBP1	0.987825	1.12E-05	77.149	S	PPASASPSAVPNGPQSPKQQKEPLSHRFNEF	0	690330	5103
P49674	363	Casein kinase I isoform epsilon	CSNK1E	0.798149	0.00890914	77.124	S	VASTPASRIQPAGNTSPRAISRVDRERKVSM	14238000	9793800	1672
Q7Z6Z7	2372	E3 ubiquitin-protein ligase HUWE1	HUWE1	0.877097	6.65E-05	77.065	S	ERDGGSGNSTIIVSRSGEDESQEDVLMDEAP	1096900	1597300	3882
O76080	52	AN1-type zinc finger protein 5	ZFAND5	0.653248	0.0010521	77.062	T	HLQRQQNSGRMSPMGTASGSNSPTSDSASVQ	0	2745200	7224
Q92859	1194	Neogenin	NEO1	0.999785	0.00229222	77.045	S	PDPNPIMTDTPIPRNSQDITPVDNSMDSNIH	208320	0	4723
Q8IWQ3	466	Serine/threonine-protein kinase BRSK2	BRSK2	0.46647	0.00063858	77.037	S	TPKESPAGTPNPTPPSSPSVGGVPWRARLNS	0	0	4060
Q8IWQ3	467	Serine/threonine-protein kinase BRSK2	BRSK2	0.466467	0.00063858	77.037	S	PKESPAGTPNPTPPSSPSVGGVPWRARLNSI	0	0	4061
Q8IWQ3	463	Serine/threonine-protein kinase BRSK2	BRSK2	0.987125	0.00063858	77.037	T	PVHTPKESPAGTPNPTPPSSPSVGGVPWRAR	1815300	1728300	7842
P37275	312	Zinc finger E-box-binding homeobox 1	ZEB1	0.311631	0.000841848	77.031	S	PVNGRPRTGLKTSQCSSPSLSASPGSPTRPQ	0	0	1441
P37275	313	Zinc finger E-box-binding homeobox 1	ZEB1	0.311631	0.000841848	77.031	S	VNGRPRTGLKTSQCSSPSLSASPGSPTRPQI	0	0	1442
P37275	315	Zinc finger E-box-binding homeobox 1	ZEB1	0.311547	0.000841848	77.031	S	GRPRTGLKTSQCSSPSLSASPGSPTRPQIRQ	0	0	1443
P37275	322	Zinc finger E-box-binding homeobox 1	ZEB1	0.988655	0.000841848	77.031	S	KTSQCSSPSLSASPGSPTRPQIRQKIENKPL	2937500	0	1444
O14639	655	Actin-binding LIM protein 1	ABLIM1	0.999375	0.0100685	76.943	S	SPINSASHIPSSKTASLPGYGRNGLHRPVST	1301000	702180	163
Q9NQ92	66	Coordinator of PRMT5 and differentiation stimulator	COPRS	0.999983	0.012162	76.927	S	AMDRLARGTQSIPNDSPARGEGTHSEEEGFA	0	2597000	5904
Q9UEW8	371	STE20/SPS1-related proline-alanine-rich protein kinase	STK39	1	0.00919088	76.927	S	DIAQRAKKVRRVPGSSGHLHKTEDGDWEWSD	1216300	373430	6298
P27708	1833	CAD protein;Glutamine-dependent carbamoyl-phosphate synthase	CAD	0.698018	0.000235977	76.889	T	PQLPPSAPATSEMTTTPERPRRGIPGLPDGR	0	0	7333
Q14204	4368	Cytoplasmic dynein 1 heavy chain 1	DYNC1H1	0.980535	0.00885954	76.868	S	DDLAYAETEKKTRTDSTSDGRPAWMRTLHTT	2899200	699530	2722
O43865	2	Putative adenosylhomocysteinase 2	AHCYL1	1	0.00600101	76.85	S	_______________MSMPDAMPLPGVGEELK	158450	416920	433
O96017	260	Serine/threonine-protein kinase Chk2	CHEK2	1	0.0230846	76.847	S	KVAIKIISKRKFAIGSAREADPALNVETEIE	932430	0	845
Q12948	268	Forkhead box protein C1	FOXC1	0.467695	0.000284215	76.818	S	AVPKIESPDSSSSSLSSGSSPPGSLPSARPL	0	0	2381
Q96D71	118	RalBP1-associated Eps domain-containing protein 1	REPS1	0.337672	0.000284264	76.817	S	ASKNEQESRHAASYSSDSENQGSYSGVIPPP	0	0	4840
Q92540	781	Protein SMG7	SMG7	0.994803	0.00417631	76.807	S	QQQQSPTKAVPALGKSPPHHSGFQQYQQADA	1230000	126270	4629

P27708	1831	CAD protein;Glutamine-dependent carbamoyl-phosphate synthase	CAD	0.358443	0.000243955	76.807	T	AVPQLPPSAPATSEMTTTPERPRRGIPGLPD	0	0	7331
P27708	1832	CAD protein;Glutamine-dependent carbamoyl-phosphate synthase	CAD	0.358443	0.000243955	76.807	T	VPQLPPSAPATSEMTTTPERPRRGIPGLPDG	0	0	7332
Q99700	733	Ataxin-2	ATXN2	0.846105	1.33E-06	76.779	S	EAVAMPIPAASPTPASPASNRAVTPSSEAKD	3240700	4654100	5259
P46821	1147	Microtubule-associated protein 1B;MAP1B heavy chain;MAP1 li	MAP1B	0.673091	0.000594385	76.777	T	PMDEMSTPRDVMSDETNNEETESPSQEFVNI	0	1108900	7389
Q96A47	157	Insulin gene enhancer protein ISL-2	ISL2	1	0.0102842	76.774	S	DHGLLLERAAAGSPRSPGPLPGARGLHLPDA	1380300	634390	4766
O75152;A(	759;760	Zinc finger CCCH domain-containing protein 11A	ZC3H11A	0.529914	0.00303994	76.768	S	GPSSSQMSMKTRRLSSASTGKPPLSVEDDFE	0	0	573
Q9NYF8	383	Bcl-2-associated transcription factor 1	BCLAF1	0.513349	0.00123874	76.768	Y	KGRAEGEWEDQEALDYFSDKESGKQKFNDSE	0	0	8429
Q6PIW4	156	Fidgetin-like protein 1	FIGNL1	0.35045	0.00136855	76.761	S	KEATVFDLPKFSVCGSSQESDSLPNSAHDRD	0	0	3546
Q6PIW4	157	Fidgetin-like protein 1	FIGNL1	0.35045	0.00136855	76.761	S	EATVFDLPKFSVCGSSQESDSLPNSAHDRDR	0	0	3547
Q9C0B5	693	Palmitoyltransferase ZDHHC5	ZDHHC5	0.774515	0.0230871	76.728	S	KSLGSASPGPGQPPLSSPTRGGVKKVSGVGG	1471300	0	5514
P46100	634	Transcriptional regulator ATRX	ATRX	0.999835	0.000112702	76.691	S	GLNPKLEKCGLGQENSDNEHLVENEVSLLLE	819930	355820	1550
O95487	332	Protein transport protein Sec24B	SEC24B	0.499262	0.000467108	76.668	T	VLSGSGSSSTRTPPTANHPVEPVTSVTQPS	0	0	7242
Q92667	151	A-kinase anchor protein 1, mitochondrial	AKAP1	0.829536	0.0127239	76.588	S	TGGEAKSIPLECPLSSPKGVLFSSKSAEVCK	0	0	4687
Q9UDY2	920	Tight junction protein ZO-2	TJP2	0.951838	1.51E-05	76.573	S	TAMGADYLSCDSRLISDFEDTDGEGGAYTDN	1398900	1088500	6281
Q9UPT8	159	Zinc finger CCCH domain-containing protein 4	ZC3H4	0.813013	0.000498797	76.573	S	DFSPSEKGHRKYREYSPPYAPSHQQYPPSHA	1003800	0	6574
Q9UDY2	933	Tight junction protein ZO-2	TJP2	0.816247	1.51E-05	76.573	T	LISDFEDTDGEGGAYTDNELDEPAEEPLVSS	0	1004900	8203
Q8TF74	266	WAS/WASL-interacting protein family member 2	WIPF2	0.387379	0.00050386	76.533	S	PPPPYRQPPGVPNGPSSPTNESAPELPQRHN	0	0	4499
Q8TF74	267	WAS/WASL-interacting protein family member 2	WIPF2	0.576563	0.00050386	76.533	S	PPPYRQPPGVPNGPSSPTNESAPELPQRHNS	0	5218600	4500
Q92613	566	Protein Jade-3	JADE3	0.940328	0.00050386	76.533	S	EDHRNSSTETDQQPHSPDSSSSVHSIRNMQV	1499000	1193900	4661
Q8TF74	269	WAS/WASL-interacting protein family member 2	WIPF2	0.317167	0.00050386	76.533	T	PYRQPPGVPNGPSSPTNESAPELPQRHNSLH	0	0	7911
Q15424;Q	344;343	Scaffold attachment factor B1;Scaffold attachment factor B2	SAFB;SAFB2	0.998391	2.09E-06	76.519	S	EASSEELAEAPTEAPSPEARDSKEDGRKFDF	7733500	13354000	2932
Q99700	730	Ataxin-2	ATXN2	0.780783	1.44E-06	76.519	T	IPTEAVAMPIPAASPTPASPASNRAVTPSSE	3240700	3400500	8032
Q02880	1342	DNA topoisomerase 2-beta	TOP2B	0.906323	0.000863771	76.492	S	KKVKKRNPWSDDESKSESDLEETEPVVIPRD	3366000	788400	2104
O43379	1248	WD repeat-containing protein 62	WDR62	0.483721	7.11E-06	76.478	S	EGPIVATLAQPLRRPSSVGELASLGQELQAI	0	0	370
O43379	1249	WD repeat-containing protein 62	WDR62	0.483721	7.11E-06	76.478	S	GPIVATLAQPLRRPSSVGELASLGQELQAIT	0	0	371
Q6NZI2	202	Polymerase I and transcript release factor	PTRF	0.999999	5.71E-06	76.478	S	GEGERPEEDAAALELSSDEAVEVEEVIEESR	804420	626750	3495
Q6NZI2	203	Polymerase I and transcript release factor	PTRF	0.999999	5.71E-06	76.478	S	EGERPEEDAAALELSSDEAVEVEEVIEESRA	804420	626750	3496
Q8WV99	170	AN1-type zinc finger protein 2B	ZFAND2B	0.464866	0.000319656	76.417	S	VASTSTVPSPSQTMPSCTSPSRATTRSPSWT	0	0	4531
Q14202	186	Zinc finger MYM-type protein 3	ZMYM3	0.997797	5.92E-06	76.329	S	GSPGQEEELPQGQPQSPNAPPSPSVGETLGD	3102400	2993300	2708
Q14202	192	Zinc finger MYM-type protein 3	ZMYM3	0.741926	5.92E-06	76.329	S	EELPQGQPQSPNAPPSPSVGETLGDGINSSQ	3102400	2993300	2709
Q14202	194	Zinc finger MYM-type protein 3	ZMYM3	0.618591	5.92E-06	76.329	S	LPQGQPQSPNAPPSPSVGETLGDGINSSQTK	1887900	3995000	2710
Q9UQ35	2118	Serine/arginine repetitive matrix protein 2	SRRM2	0.938299	0.00917244	76.329	S	RTPPVALNSSRMSCFSRPSMSPTPLDRCRSP	0	1254200	6657
Q9UQ35	2121	Serine/arginine repetitive matrix protein 2	SRRM2	0.997437	0.00917244	76.329	S	PVALNSSRMSCFSRPSMSPTPLDRCRSPGML	1603600	1254200	6658
Q02952	386	A-kinase anchor protein 12	AKAP12	0.461601	3.07E-05	76.294	S	SAEYEKVELPSEEQVSGSQGPSEEKPAPLAT	0	0	2124
Q02952	388	A-kinase anchor protein 12	AKAP12	0.461601	3.07E-05	76.294	S	EYEKVELPSEEQVSGSQGPSEEKPAPLATEV	0	0	2125
Q9UQ35	2347	Serine/arginine repetitive matrix protein 2	SRRM2	0.545907	0.0120081	76.262	T	APASANLVGPRSAHATAPVNIAGSRTAAALA	822370	355530	8267
Q14934	122	Nuclear factor of activated T-cells, cytoplasmic 4	NFATC4	0.670734	0.0117712	76.237	S	GGRVLECPSIRITSISPTPEPPAALEDNPDA	1019600	769970	2822
Q149N8	37	E3 ubiquitin-protein ligase SHPRH	SHPRH	0.966647	2.06E-05	76.237	S	HWNMHEDRRNEPIIISDDDEQPCPGSDTSSA	0	0	2830
Q4V328	690	GRIP1-associated protein 1	GRIPAP1	0.701271	0.000216307	76.237	S	ILREKESSAVPARSLSSSPQAQPPRPAELSD	1288900	985200	3105
Q4V328	692	GRIP1-associated protein 1	GRIPAP1	0.838625	0.000216307	76.237	S	REKESSAVPARSLSSSPQAQPPRPAELSDEE	1288900	985200	3106
Q9BVV8	124	Uncharacterized membrane protein C19orf24	C19orf24	0.972615	0.000251507	76.237	T	TEDPTEMASLDSDEETVFESRNLR_______	14022000	0	8056
Q86VM9	487	Zinc finger CCCH domain-containing protein 18	ZC3H18	0.999999	0.0242624	76.221	S	REKEREKEKGKPKPRSPQPPSRQAEPPKKEA	0	2093600	3959
P78344	395	Eukaryotic translation initiation factor 4 gamma 2	EIF4G2	0.999694	0.02436	76.17	S	GSGIGTGPGVIQDRFSPTMGRHRSNQLFNGH	14938000	18250000	1959
Q12872	279	Splicing factor, suppressor of white-apricot homolog	SFSWAP	0.333333	0.000177745	76.161	S	RYTVLAENKSDEKKKSGVSSDNEDDDDEEDG	0	0	2333
Q12872	282	Splicing factor, suppressor of white-apricot homolog	SFSWAP	0.333333	0.000177745	76.161	S	VLAENKSDEKKKSGVSSDNEDDDDEEDGNYL	0	0	2334
Q9H3T2	384	Semaphorin-6C	SEMA6C	0.862896	0.0108227	76.156	S	RPGSCAGVGGAALFSSSRDLPDDVLTFIKAH	1529300	1474800	5646
Q9H3T2	385	Semaphorin-6C	SEMA6C	0.987043	0.0108227	76.156	S	PGSCAGVGGAALFSSSRDLPDDVLTFIKAHP	566470	1446400	5647
Q9Y2D0	6	Carbonic anhydrase 5B, mitochondrial	CA5B	1	0.00686318	76.143	S	__________MVVMNSLRVILQASPGKLLWR	736530	430070	6774
O15085	1461	Rho guanine nucleotide exchange factor 11	ARHGEF11	0.760045	0.00107367	76.118	T	HRELLKSLGGESSGGTTPVGSFHTEAARWTD	0	974700	7153
O60678	27	Protein arginine N-methyltransferase 3	PRMT3	0.554492	1.76E-05	76.106	S	RGAVENEEDLPELSDSGDEAAWEDEDDADLP	3151600	0	512
Q8N4H0	100	Spermatogenesis associated 6-like protein	SPATA6L	0.673065	0.024559	76.064	S	ENTRDFLFPEPKLTPSHPRRCREVLMKTALG	1244500	804190	4268
P55199	442	RNA polymerase II elongation factor ELL	ELL	0.91742	0.00150371	76.051	S	LPLLTDCAQPSRPHGSPSRSKPKKKSKKHKD	0	0	1879
Q14686	1891	Nuclear receptor coactivator 6	NCOA6	0.331753	0.0115717	76.049	S	SKTPTPPAPTLLKMTSSPVGPGTASAGPSLP	0	0	2793
Q14686	1892	Nuclear receptor coactivator 6	NCOA6	0.552395	0.0115717	76.049	S	KTPTPPAPTLLKMTSSPVGPGTASAGPSLPG	0	0	2794
Q5T1M5	939	FK506-binding protein 15	FKBP15	1	0.000744107	76.049	S	VTLQLLNQQEQEKEESSSEEEEEKAEERPRR	0	0	3248
Q5T1M5	940	FK506-binding protein 15	FKBP15	1	0.000744107	76.049	S	TLQLLNQQEQEKEESSSEEEEEKAEERPRRP	0	0	3249
Q5T1M5	941	FK506-binding protein 15	FKBP15	1	0.000744107	76.049	S	LQLLNQQEQEKEESSSEEEEEKAEERPRRPS	0	0	3250
Q14686	1890	Nuclear receptor coactivator 6	NCOA6	0.331753	0.0115717	76.049	T	DSKTPTPPAPTLLKMTSSPVGPGTASAGPSL	0	0	7616
P55884	164	Eukaryotic translation initiation factor 3 subunit B	EIF3B	0.999984	1.99E-05	76.002	S	DEPSFSDPEDFVDDVSEEELLGDVLKDRPQE	25295000	16023000	1887
P56192	825	Methionine--tRNA ligase, cytoplasmic	MARS	0.5	0.0142534	75.968	S	IESLRQRFGGGQAKTSPKPAVVETVTTAKPQ	0	0	1892
P56192	824	Methionine--tRNA ligase, cytoplasmic	MARS	0.5	0.0142534	75.968	T	QIESLRQRFGGGQAKTSPKPAVVETVTTAKP	0	0	7452
P12270	2047	Nucleoprotein TPR	TPR	0.465401	0.000361437	75.945	S	ADSQNSGEGNTGAAESSFSQEVSREQQPSSA	0	0	1036
O95782	652	AP-2 complex subunit alpha-1	AP2A1	0.972486	7.94E-05	75.939	S	SNDINGGMEPTPSTVSTPSPSADLLGLRAAP	826040	946160	809
Q5VT52	758	Regulation of nuclear pre-mRNA domain-containing protein 2	RPRD2	0.998656	0.0156415	75.911	S	TSSSVDTMSLLSKIISPGSSTPSSTRSPPPG	1466700	790450	3330
P39880	1220	Homeobox protein cut-like 1	CUX1	0.371039	4.58E-05	75.897	S	EKKAYMKRRHSSVSDSQPCEPPSVGTEYSQG	0	0	1465
Q9UKJ3	1009	G patch domain-containing protein 8	GPATCH8	1	0.00887612	75.89	S	RSTRSPSQRSGSRKRSWGHSPEERHSGRRD	0	0	6445
Q9UKJ3	1014	G patch domain-containing protein 8	GPATCH8	1	0.00887612	75.89	S	PSQRSGSRKRSWGHSPEERHSGRRDFIRSK	0	0	6446
Q02880	1576	DNA topoisomerase 2-beta	TOP2B	0.33333	0.000578272	75.868	S	GRKTSKTTSKKPKKTSFDQSDVDIFPSDFP	0	0	2106
Q5T1M5	311	FK506-binding protein 15	FKBP15	0.808388	0.000113788	75.868	S	GSDGHSVSSRDSAAPSPIPGADNLSADPVVS	0	2334600	3242
Q5T1M5	326	FK506-binding protein 15	FKBP15	0.892299	0.000113788	75.868	S	SPIPGADNLSADPVVSPPTSIPFKSGEPALR	0	692810	3243
Q02880	1575	DNA topoisomerase 2-beta	TOP2B	0.33333	0.000578272	75.868	T	PGRKTSKTTSKKPKKTSFDQSDVDIFPSDF	0	0	7479
Q92786	199	Prospero homeobox protein 1	PROX1	0.992932	0.0107789	75.819	S	LRGNENEREMAPQSVSPRESYRENKRKQKLP	0	321120	4703
Q9UQB8	364	Brain-specific angiogenesis inhibitor 1-associated protein 2	BAIAP2	0.426756	0.0107789	75.819	S	PKNSYATTENKTLPRSSSMAAGLERNGRMRV	0	0	6755
Q9UQB8	365	Brain-specific angiogenesis inhibitor 1-associated protein 2	BAIAP2	0.426756	0.0107789	75.819	S	KNSYATTENKTLPRSSSMAAGLERNGRMRVK	0	0	6756
Q8NC44	138	Protein FAM134A	FAM134A	0.483775	0.000376651	75.773	S	RFLPDVSASSPEEPHSDSEGAGSGARPHLLS	0	0	4315
Q8NC44	140	Protein FAM134A	FAM134A	0.533519	0.000376651	75.773	S	LPDVSASSPEEPHSDSEGAGSGARPHLLSVP	1942200	0	4316
Q8NC44	145	Protein FAM134A	FAM134A	0.332909	0.000376651	75.773	S	ASSPEEPHSDSEGAGSGARPHLLSVPELCRY	0	0	4317
Q9P270	315	SLAIN motif-containing protein 2	SLAIN2	0.784429	6.61E-05	75.766	S	RSSGSSCNSTRRGTFSDQELDAQSLDDEDDN	1740000	650750	6224
Q9P2R6	1106	Arginine-glutamic acid dipeptide repeats protein	RERE	0.999642	1.06E-05	75.766	S	VQIKEEALDDAEEPESPPPPRSPSPEPTVV	1432600	0	6251
Q9P2R6	1113	Arginine-glutamic acid dipeptide repeats protein	RERE	0.990067	1.06E-05	75.766	S	LDDAEEPESPPPPRSPSPEPTVVDTPSHAS	1432600	0	6252
Q9P2R6	1115	Arginine-glutamic acid dipeptide repeats protein	RERE	0.969232	1.06E-05	75.766	S	DAEEPESPPPPRSPSPEPTVVDTPSHASQS	1432600	0	6253
Q9UQ35	1384	Serine/arginine repetitive matrix protein 2	SRRM2	0.383082	0.00774795	75.764	S	LDMKEQSTRSSGHSSSELSPDAVEKAGMSSN	0	0	6737
P06400	37	Retinoblastoma-associated protein	RB1	0.999997	5.03E-06	75.758	S	APPPPPPPEEDPEQDSGPEDLPLVRLEFEET	1586900	820610	913
Q8N1G0	214	Zinc finger protein 687	ZNF687	0.813189	8.71E-06	75.758	S	FELAQENGPGMQPPVSSPPLGALKQESCSPH	1553900	1593100	4225
Q8N1G0	191	Zinc finger protein 687	ZNF687	0.984605	8.71E-06	75.758	T	PLPPSAPSPTREGALTPPPFPSSFELAQENG	1085200	922130	7873
Q14517	4262	Protocadherin Fat 1;Protocadherin Fat 1, nuclear form	FAT1	0.795041	0.0114707	75.739	S	PQVPVRPISYTPSIPSDSRNNLDRNSFEGSA	0	591910	2742
Q14517	4257	Protocadherin Fat 1;Protocadherin Fat 1, nuclear form	FAT1	0.734139	0.0114707	75.739	T	YSDIPPQVPVRPISYTPSIPSDSRNNLDRNS	0	591910	7597
O75113	256	NEDD4-binding protein 1	N4BP1	0.427002	3.12E-05	75.718	S	GTPVSELTKQMDTVLSSSPDVLFDPINGLTP	0	0	556
P46821	25	Microtubule-associated protein 1B;MAP1B heavy chain;MAP1 li	MAP1B	0.364876	5.70E-05	75.71	S	EPEPSGSIANPAASTSPSLSHRFLDSKFYLL	0	0	1575
P46821	24	Microtubule-associated protein 1B;MAP1B heavy chain;MAP1 li	MAP1B	0.364876	5.70E-05	75.71	T	TEPEPSGSIANPAASTSPSLSHRFLDSKFYL	0	0	7388
Q13625	673	Apoptosis-stimulating of p53 protein 2	TP53BP2	0.998026	0.000887829	75.703	S	QHPENIYSNSQGKPGSPEPETEPVSSVQENH	2505600	2749300	2619
P07948	508	Tyrosine-protein kinase Lyn	LYN	0.840206	0.00013327	75.703	Y	LQSVLDDFYTATEGQYQQQP___________	0	0	8355
Q9BWW4	387	Single-stranded DNA-binding protein 3	SSBP3	0.975341	0.000280818	75.646	S	LHSFQNDNYSPSMTMSV______________	1009700	496090	5439
P61978	118	Heterogeneous nuclear ribonucleoprotein K	HNRNPK	0.471614	1.10E-05	75.625	T	KIIPTLEEGLQLPSPTATSQLPLESDAVECL	0	0	7454
P61978	120	Heterogeneous nuclear ribonucleoprotein K	HNRNPK	0.33072	1.10E-05	75.625	T	IPTLEEGLQLPSPTATSQLPLESDAVECLNY	0	0	7455
Q9UQ35	2115	Serine/arginine repetitive matrix protein 2	SRRM2	0.563817	0.00979386	75.611	S	SGSRTPPVALNSSRMSCFSRPSMSPTPLDRC	1603600	0	6656
P25054	2088	Adenomatous polyposis coli protein	APC	0.999985	0.00158217	75.608	S	GEDLTLDLKDIQRPDSEHGLSPDSENFDWKA	2650400	1714800	1259
P25054	2093	Adenomatous polyposis coli protein	APC	0.999465	0.00158217	75.608	S	LDLKDIQRPDSEHGLSPDSENFDWKAIQEGA	2650400	1714800	1260
Q9UNF1	191	Melanoma-associated antigen D2	MAGED2	0.498171	0.000903057	75.608	S	KARKVKHLDGEEDGSSDQSQASGTTGGRRVS	0	0	6545
Q53GS7	88	Nucleoporin GLE1	GLE1	0.81224	0.0308499	75.589	S	STSASALDQPSFVPKSPDASSAFSPASPATP	688970	206270	3140
Q96HA1	346	Nuclear envelope pore membrane protein POM 121	POM121	0.694573	0.00030633	75.588	S	IFLDGQENKRRRHDSSGSGHSAFEPLVANGV	0	0	4938
Q99700	857	Ataxin-2	ATXN2	0.661523	2.31E-05	75.588	S	DSFIENSSSNCTSGSSKPNSPSISPSILSNT	12985000	0	5249
Q99700	867	Ataxin-2	ATXN2	0.445388	2.31E-05	75.588	S	CTSGSSKPNSPSISPSILSNTEHKRGPEVTS	0	0	5253
Q86UE4	426	Protein LYRIC	MTDH	1	0.0105204	75.574	S	SLLKSQEPIPDDQKVSDDDKEKGEGALPTGK	0	2625200	3925
P41440	5	Folate transporter 1	SLC19A1	0.766164	0.00474562	75.566	S	___________MVPSSPAVEKQVPVEPGPDP	0	0	1491
Q9UPY3	1160	Endoribonuclease Dicer	DICER1	0.943878	0.0118271	75.566	S	NHDQMSVNCRTLLSESPGKLHVEVSADLTAI	0	892510	6593
Q96C92	249	Serologically defined colon cancer antigen 3	SDCCAG3	0.543889	0.000549896	75.533	S	DTDSRVSPASPAGSPSADFAVHGESLGDRHL	0	0	4824
Q15424;Q	245;244	Scaffold attachment factor B1;Scaffold attachment factor B2	SAFB;SAFB2	0.333333	0.0020161	75.533	T	VKEESSELEQPFAQDTSSVGPDRKLAEEEDL	0	0	7639
Q9NRY4	985	Rho GTPase-activating protein 35	ARHGAP35	0.961814	0.000428653	75.524	S	SPRAGSPLCNSNLQDSEEDIEPSYSLFREDT	5388000	2245500	5987
Q16643	335	Drebrin	DBN1	0.753125	0.000797364	75.524	T	SHLDSHRRMAPTPIPTRSPSDSSTASTPVAE	1392100	911230	7662
Q99613;B	11;11	Eukaryotic translation initiation factor 3 subunit C;Eukary	EIF3C;EIF3	0.686978	0.000129127	75.483	S	_____MSRFFTTGSDSESESSLSGEELVTKP	4676700	436120	65
Q99613;B	13;13	Eukaryotic translation initiation factor 3 subunit C;Eukary	EIF3C;EIF3	0.656557	0.000129127	75.483	S	___MSRFFTTGSDSESESSLSGEELVTKPVG	2533000	1242900	66
Q99613;B	24;24	Eukaryotic translation initiation factor 3 subunit C;Eukary	EIF3C;EIF3	0.77832	0.000129127	75.483	T	SDSESESSLSGEELVTKPVGGNYGKQPLLLS	3388700	0	7115
Q9H9P5	15	Putative E3 ubiquitin-protein ligase UNKL	UNKL	0.557561	0.0152044	75.463	S	_MPSVSKAAAAALSGSPPQTEKPTHYRYLKE	712810	0	5808
Q7Z6J0	735	E3 ubiquitin-protein ligase SH3RF1	SH3RF1	0.998998	8.10E-06	75.436	S	LKLLSGASTKRKPRVSPPASPTLEVELGSAE	1939400	0	3860
Q7Z6J0	739	E3 ubiquitin-protein ligase SH3RF1	SH3RF1	0.953743	8.10E-06	75.436	S	SGASTKRKPRVSPPASPTLEVELGSAELPLQ	1939400	0	3861
Q9BXK1	108	Krueppel-like factor 16	KLF16	0.538389	0.000297127	75.416	S	AAPGGASPASSSSAASSPSSGRAPGAAPSAA	0	489270	5453
Q9UJF2	35	Ras GTPase-activating protein nGAP	RASAL2	0.477418	0.000803834	75.416	S	TSTSEKPNSMDTANTSPFKVPGFFSKRLKGS	0	0	6400
Q6ZN17	202	Protein lin-28 homolog B	LIN28B	0.5	0.00756662	75.376	T	CTSTLPREVGGGHGCTSPPFPQEARAEISER	5671100	3877500	7782
P00441	99	Superoxide dismutase [Cu-Zn]	SOD1	0.875724	0.00053019	75.358	S	DLGNVTADKDGVADVSIEDSVISLSGDHCII	0	0	850
Q69YH5	400	Cell division cycle-associated protein 2	CDCA2	0.865525	0.000651925	75.358	S	LNMRKRKRVTFGEDLSPEVFDESLPANTPLR	534870	405410	3432
Q8NCY6	101	Myb/SANT-like DNA-binding domain-containing protein 4	MSANTD4	0.550333	0.000867142	75.358	S	KANIKLVGSGFPLPSSDLDDSLTEEIDEKIG	0	1396500	4333
Q8NCY6	106	Myb/SANT-like DNA-binding domain-containing protein 4	MSANTD4	0.90886	0.000887898	75.358	S	LVGSGFPLPSSDLDDSLTEEIDEKIGFRNDA	368960	0	4334
Q14202	960	Zinc finger MYM-type protein 3	ZMYM3	0.990618	0.00017331	75.326	S	LDKASSDLCDLVSNQSAEGLLEDCDLFGPAR	5302200	5042400	2703
Q9H2X6;Q	361;352	Homeodomain-interacting protein kinase 2;Homeodomain-intera	HIPK2;HIPK	0.818673	0.0233281	75.294	Y	DFGSASHVSKAVCSTYLQSRYYRAPEIILGL	0	780090	8394
Q92974	956	Rho guanine nucleotide exchange factor 2	ARHGEF2	1	0.0143103	75.229	S	SDPDTGSEEEGSSRLSPPHSPRDFTRMQDIP	0	5835300	4740
Q92974	960	Rho guanine nucleotide exchange factor 2	ARHGEF2	1	0.0143103	75.229	S	TGSEEEGSSRLSPPHSPRDFTRMQDIPEETE	0	5835300	4741
Q14684	350	Ribosomal RNA processing protein 1 homolog B	RRP1B	0.99658	3.10E-05	75.225	S	SEGSSISQLSFAEDISADEDDQILSQGKHKK	1175000	0	2781
P55196	1698	Afadin	MLLT4	0.872177	0.00174669	75.224	S	LCRPPLPRDYEPPSPSPAPGAPPPPPQRNAS	570400	275570	1864
Q99961	288	Endophilin-A2	SH3GL1	0.799015	0.0172344	75.213	S	GGFPCTTAPKIAASSSFRSSDKPIRTPSRSM	0	547180	5288
O14654	863	Insulin receptor substrate 4	IRS4	0.761111	0.000697414	75.184	S	NWDPKDAASKPSGEGSFSKPGDGGSPSKPSD	0	1319800	178
Q6L8Q7	220	2',5'-phosphodiesterase 12	PDE12	0.497704	3.79E-05	75.174	S	AAEPEVGVPSSLSPSSPSSSWTETDVEERVY	0	0	3490
Q9H1K0	215	Rabenosyn-5	RBSN	0.413697	0.00256149	75.143	S	NKLTSASKESLSTHTSPSQSPNSVHGSRRGS	0	0	5608
Q6DN90	925	IQ motif and SEC7 domain-containing protein 1	IQSEC1	0.495912	0.017359	75.109	S	SYASGEGLKRSALSSSLRDLSEAGKRGRRSS	0	0	3449
P04920	150	Anion exchange protein 2	SLC4A2	0.310965	0.000319093	75.106	S	EGARALTQPSPVSTPSSVQFFLQEDDSADRK	0	0	890
Q8NEZ4	4267	Histone-lysine N-methyltransferase 2C	KMT2C	0.967905	0.015186	75.102	S	AKNSENKESIPSLPQSPMRETPSKAFHQYSN	916790	1053200	4382
P49768	43	Presenilin-1;Presenilin-1 NTF subunit;Presenilin-1 CTF subu	PSEN1	0.999994	0.0152096	75.088	S	SQNDNRERQEHNDRRSLGHPEPLSNGRPQGN	1193800	0	1692
Q9Y4K3	438	TNF receptor-associated factor 6	TRAF6	0.999977	0.0152096	75.088	S	PWPFQGTIRLTILDQSEAPVRQNHEEIMDAK	1437400	1762000	6930
Q9Y4F5	829	Centrosomal protein of 170 kDa protein B	CEP170B	0.999992	0.00107076	75.064	S	AAPGDGEGLGQTAQPSPPARDGVYVSANGRM	0	0	6915

Q14558	215	Phosphoribosyl pyrophosphate synthase-associated protein 1	PRPSAP1	1	0.0264825	75.043	S	GEAQCTELDMDDGRHSPPMVKNATVHPGLEL	578950	311690	2743
Q9UPP5	802	Uncharacterized protein KIAA1107	KIAA1107	0.937177	0.0264825	75.043	S	SSSDTSTPEELKIYDSNLRIEVKMKKQSNND	0	0	6568
Q9UHY1	422	Nuclear receptor-binding protein	NRBP1	0.935116	0.00296225	74.993	S	FGLPRPQQPQQEEVTSPVVPPSVKTPTPEPA	1429200	1260200	6370
Q15796	8	Mothers against decapentaplegic homolog 2	SMAD2	0.999634	0.0187559	74.987	T	________MSSILPFTPPVVKRLLGWKKSAG	682860	244220	7650
Q9Y6N7	1055	Roundabout homolog 1	ROBO1	0.999963	0.0157577	74.944	S	DVDLSNKINEMKTFNSPNLKDGRFVNPSGQP	1433000	883250	7018
Q86Y13	1105	E3 ubiquitin-protein ligase DZIP3	DZIP3	0.706617	0.00024102	74.876	S	QSQGKSVSNVNCVSPSHSPSQPDAAQPPKPA	816280	450250	4019
Q86Y13	1107	E3 ubiquitin-protein ligase DZIP3	DZIP3	0.847318	0.00024102	74.876	S	QGKSVSNVNCVSPSHSPSQPDAAQPPKPAWR	816280	450250	4020
Q8WVV9	61	Heterogeneous nuclear ribonucleoprotein L-like	HNRNPLL	0.931754	0.0145158	74.853	S	TPRGGGDGGGGRSFSQPEAGGSHHKVSVSP	5474400	22374000	4546
Q8WVV9	68	Heterogeneous nuclear ribonucleoprotein L-like	HNRNPLL	0.999999	0.0145158	74.853	S	GGGGGRSFSQPEAGGSHHKVSVSPVVHVRGL	5474400	22374000	4547
Q8WYP5	1142	Protein ELYS	AHCTF1	0.999805	0.0176818	74.841	S	RPSQCSEFIQQSSMKSPLYLVSRSLPSSSQL	748840	505640	4607
Q6Y7W6	236	PERQ amino acid-rich with GYF domain-containing protein 2	GIGYF2	1	0.00776619	74.78	S	RLAGSRRDGERWRPHSPDGPRSAGWREHMER	1112700	0	3645
Q8N488	227	RING1 and YY1-binding protein	RYBP	0.999998	0.0132828	74.769	S	RSSTPKGDMSAVNDESF_______________	490820	666670	4266
Q53ET0	170	CREB-regulated transcription coactivator 2	CRTC2	0.31386	5.62E-05	74.766	S	EKGQLFRLPSALNRTSSDSALHTSVMNPSPQ	0	0	3128
Q53ET0	171	CREB-regulated transcription coactivator 2	CRTC2	0.31386	5.62E-05	74.766	S	KGQLFRLPSALNRTSSDSALHTSVMNPSPQD	0	0	3129
Q53ET0	169	CREB-regulated transcription coactivator 2	CRTC2	0.527339	5.62E-05	74.766	T	AEKGQLFRLPSALNRTSSDSALHTSVMNPSP	0	0	7693
Q9C0C2	504	182 kDa tankyrase-1-binding protein	TNKS1BP1	0.746894	0.000206082	74.763	S	VWRLDSPPPSPITEASEAAEAAEAGNLAVSS	1524000	0	5523
Q9C0C2	501	182 kDa tankyrase-1-binding protein	TNKS1BP1	0.746894	0.000206082	74.763	T	GLGVWRLDSPPPSPITEASEAAEAAEAGNLA	1524000	0	8076
O15085	255	Rho guanine nucleotide exchange factor 11	ARHGEF11	0.935214	0.00539565	74.732	S	RNSVLSDPGLDSPRTSPVIMARVAQHHRRQG	1462300	1047600	286
P11171	551	Protein 4.1	EPB41	0.733485	0.0017947	74.732	S	SKRASRSLDGAAAVDSADRSPRPTSAPAITQ	61671	2072600	1007
O15085	254	Rho guanine nucleotide exchange factor 11	ARHGEF11	0.617254	0.00539565	74.732	T	NRNSVLSDPGLDSPRTSPVIMARVAQHHRRQ	1462300	1047600	7152
Q9NY27	361	Serine/threonine-protein phosphatase 4 regulatory subunit 2	PPP4R2	0.499504	0.00104788	74.703	S	MEESDVSQAEKDLLHSEGSENEGPVSSSSSD	0	0	6085
Q9NY27	364	Serine/threonine-protein phosphatase 4 regulatory subunit 2	PPP4R2	0.524785	0.00104788	74.703	S	SDVSQAEKDLLHSEGSENEGPVSSSSSDCRE	0	0	6086
Q13615	619	Myotubularin-related protein 3	MTMR3	0.513612	0.00804077	74.703	T	PLSRLPKTRSYDNLTTACDNTVPLASRRCSD	2630800	1925100	7568
Q9HCD5	378	Nuclear receptor coactivator 5	NCOA5	0.513597	0.0132069	74.678	S	IINYLRERKERLMRSSTDSLPGPISRQPLGA	0	0	5844
Q9HCD5	381	Nuclear receptor coactivator 5	NCOA5	0.995972	0.0132069	74.678	S	YLRERKERLMRSSTDSLPGPISRQPLGATSG	0	385670	5845
P31260	93	Homeobox protein Hox-A10	HOXA10	0.987981	1.59E-05	74.666	S	CGLFPTLGGKRNEAASPGSGGGGGLGPGAH	1910900	1101200	1353
Q9NR30	164	Nucleolar RNA helicase 2	DDX21	0.296783	2.98E-05	74.65	S	KLKNGFPHPEPDCNPSEAASEESNSEIEQEI	0	0	5947
O94913	494	Pre-mRNA cleavage complex 2 protein Pcf11	PCF11	0.999999	0.0077444	74.611	S	RSRSRSPKSRSPIIHSPKRRDRRSPKRRQRS	2002200	1132200	721
Q04721	1845	Neurogenic locus notch homolog protein 2;Notch 2 extracellu	NOTCH2	0.706888	8.69E-06	74.605	S	TPLMLASLRGGSSDLSDEDEDAEDSSANIIT	393720	1560300	2164
Q02446	767	Transcription factor Sp4	SP4	0.528015	0.000481924	74.584	S	TEVLGSPRIVTVAAISQDSNPATPNVSTNME	0	0	2093
Q15424	26	Scaffold attachment factor B1	SAFB	0.349233	0.000174839	74.579	T	SGAAGAAALSSASSETGTRRLSDLRVIDLRA	0	0	7638
Q9HB09	242	Bcl-2-like protein 12	BCL2L12	0.967566	0.0180524	74.533	S	KLASDPALRSKLVRLSSDSFARLVELFCSRD	3968000	2689200	5832
Q9P2R6	1266	Arginine-glutamic acid dipeptide repeats protein	RERE	0.908445	0.0131453	74.533	S	PALRTLSEYARPHVMSPTNRNHPFYMPLNPT	604130	381430	6254
O15047	1152	Histone-lysine N-methyltransferase SETD1A	SETD1A	0.835878	0.0109834	74.519	S	PAGPPAPAPRPDERPSSPIPLLPPPKKRRKT	0	861440	266
P17600	551	Synapsin-1	SYN1	1	0.00134366	74.519	S	VAGGPGAPPAARPPASPSPQRQAGPPQATRQ	4849200	2335900	1117
P17600	553	Synapsin-1	SYN1	1	0.00134366	74.519	S	GGPGAPPAARPPASPSPQRQAGPPQATRQTS	4849200	2335900	1118
Q92667	429	A-kinase anchor protein 1, mitochondrial	AKAP1	1	7.76E-06	74.494	S	PPDAGLPLPGLPAEGSPPPKTYVSCLKSLLS	2687100	1434900	4689
P38159	352	RNA-binding motif protein, X chromosome;RNA-binding motif pr	RBMX	1	0.0181354	74.464	S	SGRDRVGRQERGLPPSMERGYPPPRDSYSSS	0	1474200	1447
Q5H9R7	851	Serine/threonine-protein phosphatase 6 regulatory subunit 3	PPP6R3	0.379338	0.0124815	74.46	S	CPETAEAKCAAPRPPSSSPEQRTGQPSAPGD	0	0	3161
Q8N556	342	Actin filament-associated protein 1	AFAP1	0.499526	0.000200953	74.392	S	IISLGKKKPSTDEQTSSAEEDVPTCGYLNVL	0	0	4273
Q8N556	343	Actin filament-associated protein 1	AFAP1	0.747294	0.000200953	74.392	S	ISLGKKKPSTDEQTSSAEEDVPTCGYLNVLS	1460700	926700	4274
P05387	79	60S acidic ribosomal protein P2	RPLP2	0.999172	3.20E-05	74.382	S	SVPAGGAVAVSAAPGSAAPAAGSAPAAAEEK	0	2102400	897
Q15776	193	Zinc finger protein with KRAB and SCAN domains 8	ZKSCAN8	0.853451	0.0128478	74.376	S	PVPQESQERAMSTSQSPTRSQKGSSGDQEMT	0	619670	2985
Q49AG3	387	Zinc finger BED domain-containing protein 5	ZBED5	0.993902	0.0277395	74.376	S	CLLYRHALAVKIMPTSLKNVLDQAVQIINYI	920010	972060	3089
Q01082	2337	Spectrin beta chain, non-erythrocytic 1	SPTBN1	0.584143	0.00330081	74.364	T	ASSRAQTLPTSVVTITSESSPGKREKDKEKD	0	0	7472
O14617	827	AP-3 complex subunit delta-1	AP3D1	0.492803	0.0167141	74.338	S	DSEKLPIQKHRNTETSKSPEKDVPMVEKKSK	0	0	154
P78527	3215	DNA-dependent protein kinase catalytic subunit	PRKDC	0.999999	0.00152814	74.324	S	LPEDNSMNVDQDGDPSDRMEVQEQEEDISSL	0	148900	1969
Q02952	392	A-kinase anchor protein 12	AKAP12	0.562208	6.21E-05	74.3	S	VELPSEEQVSGSQGPSEEKPAPLATEVFDEK	0	3759000	2126
Q15025	434	TNFAIP3-interacting protein 1	TNIP1	0.593159	0.000142341	74.287	S	LNKALEEALSIQTPPSPPTAFGSPEGAGAL	800000	723080	2858
Q15025	431	TNFAIP3-interacting protein 1	TNIP1	0.877595	0.000142341	74.287	T	IQRLNKALEEALSIQTPPSPPTAFGSPEGA	800000	723080	7631
Q9C0A6	852	SET domain-containing protein 5	SETD5	0.999995	0.0199304	74.214	S	RYLMEQNVTKLLRPLSPVTPPPPNSGSKSPQ	1969500	1116600	5506
Q9C0A6	855	SET domain-containing protein 5	SETD5	0.999966	0.0199304	74.214	T	MEQNVTKLLRPLSPVTPPPPNSGSKSPQLAT	1969500	1116600	8073
P78559	1069	Microtubule-associated protein 1A;MAP1A heavy chain;MAP1 li	MAP1A	0.999912	0.000223052	74.206	S	EEGTLEKEKVPPPRSPQAQEAPVNIDEGLT	3666600	3172500	1982
Q9H410	58	Kinetochore-associated protein DSN1 homolog	DSN1	0.776868	0.0184867	74.173	S	EMNQGVSEERIHLGSSPKKGGNCDLSHQERL	5042000	0	5652
O43426	1565	Synaptojanin-1	SYNJ1	0.999698	0.0131506	74.165	S	TSPPVDPFTTLASKASPTLDFTER________	1156400	456630	380
Q9HDC5	216	Junctophilin-1	JPH1	0.999928	0.0184959	74.165	S	AELAGKKKGGLFRRGSLLGSMKLRKSESKSS	307440	155510	5882
Q9NRR5	117	Ubiquilin-4	UBQLN4	0.456286	1.23E-06	74.132	T	PDPASAPSTTPASPATPAQPSTSGSASSDAG	0	0	8159
Q8IWX8	813	Calcium homeostasis endoplasmic reticulum protein	CHERP	0.998587	0.00108335	74.123	S	SRSRSKSYSPGRRRRSRSRSPTPPSSAGLGS	0	0	4082
Q9BTC8	428	Metastasis-associated protein MTA3	MTA3	0.979428	0.00255888	73.984	S	YGGLKMPTQSEEEKLSPSPTTEDPRVRSHVS	0	2405700	5362
Q9BTC8	430	Metastasis-associated protein MTA3	MTA3	0.895313	0.00255888	73.984	S	GLKMPTQSEEEKLSPSPTTEDPRVRSHVSRQ	754530	2770300	5363
Q9H3H1	443	tRNA dimethylallyltransferase, mitochondrial	TRIT1	0.988927	0.00205212	73.983	S	RLDSDAVNTIEESQSVSPDHNKEPKEKGSPGQ	1578300	922410	5635
O75381	271	Peroxisomal membrane protein PEX14	PEX14	0.200667	4.38E-05	73.982	S	VNHHSSSDISPVSNESTSSSPGKEGHSPEGS	0	0	616
O75381	274	Peroxisomal membrane protein PEX14	PEX14	0.200667	4.38E-05	73.982	S	HSSSDISPVSNESTSSSPGKEGHSPEGSTVT	0	0	617
O75381	275	Peroxisomal membrane protein PEX14	PEX14	0.590742	4.38E-05	73.982	S	SSSDISPVSNESTSSSPGKEGHSPEGSTVTY	0	0	618
O75381	272	Peroxisomal membrane protein PEX14	PEX14	0.200667	4.38E-05	73.982	T	NHHSSSDISPVSNESTSSSPGKEGHSPEGST	0	0	7200
Q96QU8	224	Exportin-6	XPO6	0.389376	0.000124246	73.916	S	PTSGESGDLLSNLLQSPSSAKLLNQPIPILD	0	0	5086
Q07002	132	Cyclin-dependent kinase 18	CDK18	0.996427	0.0135297	73.9	S	SPDLPKPLSRMSRRASLSDIGFGKLETYVKL	612330	637840	2212
Q9C073	29	Protein FAM117A	FAM117A	0.999996	0.0171995	73.887	S	WGPGRGGAGGLRRGCSPPAPAGSPRAGLQPL	690410	0	5502
P28347	11	Transcriptional enhancer factor TEF-1	TEAD1	0.590885	0.0207488	73.877	S	_____MEPSSWSGSESPAENMERMSDSADKP	638490	0	1307
P54725	125	UV excision repair protein RAD23 homolog A	RAD23A	0.826955	0.000133754	73.868	S	GMSHPPPAAREDKSPSEESAPTTSPESVSGS	0	0	1852
Q8WX93	1106	Palladin	PALLD	0.768681	0.00696369	73.841	S	MVQAVNQRGRSRSPSGHPHVRRPRSRSRDS	0	0	4584
Q96GX9	198	Methylthioribulose-1-phosphate dehydratase	APIP	0.99981	0.0211626	73.841	Y	EYPDSCAVLVRRHGVYVWGETWEKAKTMCEC	0	103330	8409
Q9UJF2	18	Ras GTPase-activating protein nGAP	RASAL2	0.492595	0.00107554	73.839	S	TPEVPAERSPRRRSISGTSTSEKPNSMDTAN	0	0	6399
Q9UEY8	683	Gamma-adducin	ADD3	0.688769	0.0135809	73.834	S	IEEVLSPEGSPSKSPSKKKKKFRTPSFLKKN	0	2293900	6303
Q8N302	17	Angiogenic factor with G patch and FHA domains 1	AGGF1	0.771937	0.000482232	73.831	S	ASEAPSPPRSPPPPTSPEPELAQLRRKVEKL	140440	0	4247
O60293	1298	Zinc finger C3H1 domain-containing protein	ZFC3H1	0.830758	0.00196016	73.809	S	KDKRKWKPKFWRKPISDNSFSSDEEQSTGPI	0	3929800	470
Q07157	275	Tight junction protein ZO-1	TJP1	0.76868	0.00156178	73.809	S	VQRDERATLLNVPDLSDSIHSANASERDDIS	0	0	2216
Q8WVC0	197	RNA polymerase-associated protein LEO1	LEO1	0.999635	0.00997864	73.802	S	DEKMQNTDDEERPQLSDDERQQLSEEEKANS	0	1379900	4538
Q9C0C4	760	Semaphorin-4C	SEMA4C	0.999984	0.000749332	73.797	S	IVPGHARCQPGGGPPSPPPGIPGQPLPSPTR	1150300	540980	5530
Q9C0C4	772	Semaphorin-4C	SEMA4C	0.525553	0.000749332	73.797	S	GPPSPPPGIPGQPLPSPTRLHLGGGRNSNAN	1150300	540980	5531
Q9C0C4	774	Semaphorin-4C	SEMA4C	0.500304	0.000749332	73.797	T	PSPPPGIPGQPLPSPTRLHLGGGRNSNANGY	1150300	0	8077
O94826	110	Mitochondrial import receptor subunit TOM70	TOMM70A	0.999999	0.000752226	73.771	S	GSGHPEGPGAHLDMNSLDRAQAAKNKGNKYF	7680000	3490700	702
Q96S59	482	Ran-binding protein 9	RANBP9	0.538886	0.00128885	73.76	S	SPKSQDSYPVSPRPFSSPSMSPSHGMNIHNL	0	0	5111
Q9NYF8	206	Bcl-2-associated transcription factor 1	BCLAF1	0.308209	0.00424475	73.76	S	FEHDPSESIDEFNKSSATSGDIWPGLSAYDN	0	0	6112
Q9Y5B9	986	FACT complex subunit SPT16	SUPT16H	0.980379	0.00209426	73.76	S	EESDYSKESLGSEEESGKDWDELEEEARKAD	0	0	6959
P62258	233	14-3-3 protein epsilon	YWHAE	0.747448	0.00678007	73.758	S	TLIMQLLRDNLTLWTSDMQGDGEEQNKEALQ	415530	328510	1919
Q04721	1841	Neurogenic locus notch homolog protein 2;Notch 2 extracellu	NOTCH2	0.597601	1.08E-05	73.758	S	PDGCTPLMLASLRGGSSDLSDEDEDAEDSSA	393720	515650	2162
Q04721	1842	Neurogenic locus notch homolog protein 2;Notch 2 extracellu	NOTCH2	0.612542	1.08E-05	73.758	S	DGCTPLMLASLRGGSSDLSDEDEDAEDSSAN	393720	515650	2163
Q92529	403	SHC-transforming protein 3	SHC3	0.871018	0.0160835	73.758	S	TFGEDWQQTPLRQGSSDIYSTPEGKLHVAPT	0	0	4618
Q92529	407	SHC-transforming protein 3	SHC3	0.522876	0.0160835	73.758	S	DWQQTPLRQGSSDIYSTPEGKLHVAPTGEAP	0	0	4619
Q9HB09	273	Bcl-2-like protein 12	BCL2L12	1	0.0202333	73.758	S	DSSRPSRACPGPPPPSPEPLARLALAMELSR	869380	598770	5828
Q9NUL3	455	Double-stranded RNA-binding protein Staufen homolog 2	STAU2	0.613863	0.000705068	73.722	S	SPKDMNQPSSSFFSISPTSNSSATIARELLM	498860	482320	6021
Q9UDY2	394	Tight junction protein ZO-2	TJP2	0.920927	0.000191159	73.676	S	LVVLRDSQQTLINIPSLNDSDSEIEDISEIE	569080	10034000	6278
Q9NYF8	219	Bcl-2-associated transcription factor 1	BCLAF1	0.767247	0.0152658	73.672	Y	KSSATSGDIWPGLSAYDNSPRSPHSPSPIAT	0	463400	8430
P40425	155	Pre-B-cell leukemia transcription factor 2	PBX2	0.444896	0.00011201	73.649	S	AAAAAASGGGVSPDNSIEHSDYRSKLAQIRH	0	0	1474
Q03164	2167	Histone-lysine N-methyltransferase 2A;MLL cleavage product N	KMT2A	0.239611	0.000247783	73.649	S	PTQRSPGCRPLPSAGSPTPTTHEIVTVGDPL	0	0	2143
Q9UJJ7	271	RNA pseudouridylate synthase domain-containing protein 1	RPUSD1	0.817788	0.000551135	73.649	S	ATPDPDPEDRGPRPGSPSALLPGPGRPPPPP	0	0	6401
Q03164	2169	Histone-lysine N-methyltransferase 2A;MLL cleavage product N	KMT2A	0.239611	0.000247783	73.649	T	QRSPGCRPLPSAGSPTPTTHEIVTVGDPLLS	0	0	7486
Q03164	2171	Histone-lysine N-methyltransferase 2A;MLL cleavage product N	KMT2A	0.239611	0.000247783	73.649	T	SPGCRPLPSAGSPTPTTHEIVTVGDPLLSSG	0	0	7487
A0FGR8	704	Extended synaptotagmin-2	ESYT2	0.538728	5.94E-05	73.616	S	HMSGSPGPGGSNTAPSTPVIGGSDKPGMEEK	0	0	7
Q969R5	67	Lethal(3)malignant brain tumor-like protein 2	L3MBTL2	0.580389	0.00495594	73.597	S	SEAENEDREAGELPTSPLHLLSPGTPRSLDG	984330	1408100	4748
Q969R5	73	Lethal(3)malignant brain tumor-like protein 2	L3MBTL2	0.809238	0.00495594	73.597	S	DREAGELPTSPLHLLSPGTPRSLDGSGSEPA	984330	1408100	4749
Q969R5	66	Lethal(3)malignant brain tumor-like protein 2	L3MBTL2	0.842934	0.00495594	73.597	T	SSEAENEDREAGELPTSPLHLLSPGTPRSLD	984330	1921300	7959
P13866	4	Sodium/glucose cotransporter 1	SLC5A1	0.674977	0.0299672	73.593	S	______________MDSSTWSPKTTAVTRPVET	1232300	756210	1059
Q9UN36	338	Protein NDRG2	NDRG2	0.939927	0.017807	73.593	S	TRLSRSRTASLTSAASVDGNRSRSRTLSQSS	940960	1438100	6528
Q96QC0	591	Serine/threonine-protein phosphatase 1 regulatory subunit 10	PPP1R10	0.942286	3.89E-05	73.548	S	GGGINVQEILTSIMGSPNSHPSEELLKQPDY	2603900	1279200	5074
Q14152	1336	Eukaryotic translation initiation factor 3 subunit A	EIF3A	1	0.0192972	73.499	S	VEERDPRPRVPPPALSRDRERDRDREREGEK	3378800	2218800	2654
Q99741	74	Cell division control protein 6 homolog	CDC6	0.999935	0.0157754	73.498	S	LGDDNLCNTPHLPPCSPPKQGKKENGPPHSH	1093100	545410	5273
P04637	9	Cellular tumor antigen p53	TP53	0.67622	0.000639221	73.459	S	_______MEEPQSDPSVEPPLSQETFSDLWK	13279000	10871000	884
Q8WX93	754	Palladin	PALLD	0.404424	2.33E-05	73.389	S	AAQNLGPASGHGTPASSPSSSSLPSPMSPTP	0	0	4575
Q8WX93	755	Palladin	PALLD	0.404424	2.33E-05	73.389	S	AQNLGPASGHGTPASSPSSSSLPSPMSPTPR	0	0	4576
Q8WX93	766	Palladin	PALLD	0.637766	2.33E-05	73.389	S	TPASSPSSSSLPSPMSPTPRQFGRAPVPPFA	1149700	0	4582
Q8WX93	768	Palladin	PALLD	0.468078	2.33E-05	73.389	T	ASSPSSSSLPSPMSPTPRQFGRAPVPPFAQP	0	0	7926
Q96GY3	182	Protein lin-37 homolog	LIN37	0.997821	0.000118691	73.341	S	TPPGPPGDACRSRIPSPLQPEMQGTPDDEPS	1406500	1124400	4933
Q9BQ52	208	Zinc phosphodiesterase ELAC protein 2	ELAC2	0.987571	0.000742817	73.299	S	KHQPWQSPERPLSRLSPERSSDSESNENEPH	3405500	1388400	5296
Q92598	554	Heat shock protein 105 kDa	HSPH1	0.534423	2.21E-05	73.276	S	GTQPQVQTDAQQTSQSPPSPELTSEENKIPD	0	1147900	4654
O95696	1052	Bromodomain-containing protein 1	BRD1	0.666521	0.0167762	73.273	S	DRAMNHLSRVHGEPTSDLSDID__________	0	32607	797
O95696	1055	Bromodomain-containing protein 1	BRD1	0.959175	0.0167762	73.273	S	MNHLSRVHGEPTSDLSDID_____________	0	32607	798
Q9Y2K7	718	Lysine-specific demethylase 2A	KDM2A	0.891205	0.000950858	73.269	S	RPLRSCDEPLTPPPHSPTSMLQLIHDPVSPR	1192200	0	6792
Q9Y2K7	731	Lysine-specific demethylase 2A	KDM2A	0.999608	0.000950858	73.269	S	PHSPTSMLQLIHDPVSPRGMVTRSSPGAGPS	1192200	0	6793
Q9Y2K7	713	Lysine-specific demethylase 2A	KDM2A	0.990775	0.000950858	73.269	T	QAKVLRPLRSCDEPLTPPPHSPTSMLQLIHD	1192200	0	8285
O14497	763	AT-rich interactive domain-containing protein 1A	ARID1A	0.333333	0.00670842	73.252	S	QDRGYMQRNPQMPQYSSPQPGSALSPRQPSG	0	0	142
O14497	764	AT-rich interactive domain-containing protein 1A	ARID1A	0.333333	0.00670842	73.252	S	DRGYMQRNPQMPQYSSPQPGSALSPRQPSGG	0	0	143
O14497	762	AT-rich interactive domain-containing protein 1A	ARID1A	0.333333	0.00670842	73.252	Y	AQDRGYMQRNPQMPQYSSPQPGSALSPRQPS	0	0	8340
Q8N556	336	Actin filament-associated protein 1	AFAP1	0.592932	0.000921516	73.247	S	GKKITKIISLGKKKPSTDEQTSSAEEDVPTC	0	0	4272
Q8N556	337	Actin filament-associated protein 1	AFAP1	0.592932	0.000921516	73.247	T	KKITKIISLGKKKPSTDEQTSSAEEDVPTCG	0	0	7875
O75179	1696	Ankyrin repeat domain-containing protein 17	ANKRD17	0.980787	0.025422	73.219	S	EGTSNSLSTCTKSGPSPLSSPNGKLTVASPK	603230	526580	589
O75179	1699	Ankyrin repeat domain-containing protein 17	ANKRD17	0.885398	0.025422	73.219	S	SNSLSTCTKSGPSPLSSPNGKLTVASPKRGQ	603230	526580	590
Q03164	1837	Histone-lysine N-methyltransferase 2A;MLL cleavage product N	KMT2A	0.983888	0.0013377	73.21	S	KIIPAPKKPGPEPDSPTPLHPPTPPILSTD	1385300	1279600	2138
Q9ULL5	582	Proline-rich protein 12	PRR12	0.997725	0.00164146	73.21	S	PLQSPPATGRPPGVGSPGAPGKYLSSVLASA	9629800	5073300	6490
Q03164	1845	Histone-lysine N-methyltransferase 2A;MLL cleavage product N	KMT2A	0.989555	0.0013377	73.21	T	KGPGEPDSPTPLHPPTPPILSTDRSREDSPE	1385300	1279600	7484
P06748	67	Nucleophosmin	NPM1	0.755829	0.00396863	73.208	Y	GAKDELHIVEAEAMNYEGSPIKVTLATLKMS	364120	0	8353
O14497	769	AT-rich interactive domain-containing protein 1A	ARID1A	0.703198	0.0159909	73.168	S	QRNPQMPQYSSPQPGSALSPRQPSGGQIHTG	0	1787500	144
Q13464	1102	Rho-associated protein kinase 1	ROCK1	0.533789	0.000372653	73.164	S	IEQLRAKLLDLSDSTSVASFPSADETDGNLP	0	0	2544
Q13464	1105	Rho-associated protein kinase 1	ROCK1	0.765849	0.000372653	73.164	S	LRAKLLDLSDSTSVASFPSADETDGNLPESR	0	831050	2545
O95935	109	T-box transcription factor TBX18	TBX18	0.999229	0.00065429	73.153	S	RGAAGGCEDGFQQGASPLASPGGSPKGSPAR	1392000	610370	839
O95935	117	T-box transcription factor TBX18	TBX18	0.887751	0.00065429	73.153	S	DGFQQGASPLASPGGSPKGSPARSLARPGTP	1392000	610370	840
Q8N556	283	Actin filament-associated protein 1	AFAP1	0.862083	0.000976757	73.153	S	HKAELEKKLSSERPSSDGEGVVENGITTCNG	0	614220	4277
Q96GY3	137	Protein lin-37 homolog	LIN37	0.723817	0.000572588	73.13	S	WMRNSPSVRERECSPSSPLPPLPEDEEGSEV	0	2383800	4931

Q9BZQ8	581	Protein Niban	FAM129A	0.400575	5.81E-05	73.13	S	HNLFEDNMALPSESVSSLTDLKPPTGSNQAS	0	0	5498
Q9BZQ8	582	Protein Niban	FAM129A	0.400575	5.81E-05	73.13	S	NLFEDNMALPSESVSSLTDLKPPTGSNQASP	0	0	5499
Q9BZQ8	592	Protein Niban	FAM129A	0.515621	5.81E-05	73.13	S	SESVSSLTDLKPPTGSNQASPARRASAILPG	780600	344060	5500
Q9BZQ8	596	Protein Niban	FAM129A	0.968282	5.81E-05	73.13	S	SSLTDLKPPTGSNQASPARRASAILPGVLGS	780600	344060	5501
Q9Y618	2413	Nuclear receptor corepressor 2	NCOR2	0.998551	0.000585579	73.13	S	GKAKVSGRPSSRKAKSPAPGLASGDRPPSVS	2586600	1007600	6983
Q9BZQ8	590	Protein Niban	FAM129A	0.515552	5.81E-05	73.13	T	LPSESVSSLTDLKPPTGSNQASPARRASAIL	780600	344060	8072
P34932	828	Heat shock 70 kDa protein 4	HSPA4	0.834142	3.25E-05	73.126	S	PGPQAAEQGTDTAVPSDSDKKLPEMDID___	1056400	1160400	1371
Q9UID3	10	Vacuolar protein sorting-associated protein 51 homolog	VPS51	0.50465	0.0087831	73.11	S	______MAAAAAAGPSPGSGPGDSPEGPEGE	0	0	6376
Q9Y3C6	147	Peptidyl-prolyl cis-trans isomerase-like 1	PPIL1	0.853103	0.0141985	73.11	T	VCQGIGMVNRVGMVETNSQDRPVDDVKIIKA	668620	5134000	8291
Q9UHB7	1070	AF4/FMR2 family member 4	AFF4	0.200356	0.000271749	73.109	S	SPVSPKLSPGNSGNYSSGASSASASGSSVTI	0	0	6341
Q9UHB7	1071	AF4/FMR2 family member 4	AFF4	0.200356	0.000271749	73.109	S	PVSPKLSPGNSGNYSSGASSASASGSSVTIP	0	0	6342
Q9C0C2	494	182 kDa tankyrase-1-binding protein	TNKS1BP1	0.97966	0.00028662	73.072	S	TFPTRPSGLGVWRLDSPPPSPITEASEAAEA	0	833240	5521
Q9Y520	887	Protein PRRC2C	PRRC2C	0.882371	0.000835387	72.99	T	QKFLSRSVEDVRPHHTDANNQSACFEAPDQK	5649300	3113100	8308
Q14004	1143	Cyclin-dependent kinase 13	CDK13	0.539546	0.000481366	72.949	T	KINLPAGILATGEKQTDPSTPQQESSKPLGG	0	0	7574
P55201	120	Peregrin	BRPF1	0.947328	0.00035961	72.935	S	GRVHRISIFDNLDVVSEDEEAPEEAPENGSN	1330200	1579800	1881
P19367	438	Hexokinase-1	HK1	1	0.0199852	72.928	S	YSRRFHKTLRRLVPDSDVRFLLSESGSGKGA	0	1984600	1162
Q96AT1	50	Uncharacterized protein KIAA1143	KIAA1143	1	0.000603921	72.927	S	TKRIQPQPPDEDGDHSDKEDEQPQVVVLKKG	26553000	12202000	4776
Q86W56	139	Poly(ADP-ribose) glycohydrolase	PARG	0.597996	0.0168095	72.898	T	EKLENVSQLSLDKSPTEKSTQYLNQHQTAAM	0	0	7831
O43399	19	Tumor protein D54	TPD52L2	0.414755	0.00734982	72.891	S	AGQDINLNSPNKGLLSDSMTDVPVDTGVAAR	0	0	377
Q92934	74	Bcl2-associated agonist of cell death	BAD	0.283371	0.00133943	72.883	S	SHGGAGAVEIRSRHSSYPAGTEDDEGMGEE	0	0	4732
Q9UBB4	430	Ataxin-10	ATXN10	0.963141	0.0216565	72.883	S	TQWVIYAIRNLTEDNSQNQDLIAKMEEQGLA	297280	446170	6256
P30622	160	CAP-Gly domain-containing linker protein 1	CLIP1	0.629056	5.55E-05	72.881	S	ATSPLCTSTASMVSSSPSTPSNIPQKPSQPA	0	1920600	1349
Q6P4R8	338	Nuclear factor related to kappa-B-binding protein	NFRKB	0.333182	5.93E-05	72.881	S	IKTIKSEAEDLAEPLSSTEGVAPLSQAPSPL	0	0	3525
Q6P4R8	339	Nuclear factor related to kappa-B-binding protein	NFRKB	0.333182	5.93E-05	72.881	S	KTIKSEAEDLAEPLSSTEGVAPLSQAPSPLA	0	0	3526
Q6P4R8	340	Nuclear factor related to kappa-B-binding protein	NFRKB	0.333182	5.93E-05	72.881	T	TIKSEAEDLAEPLSSTEGVAPLSQAPSPLAI	0	0	7760
Q9P1Y6	867	PHD and RING finger domain-containing protein 1	PHRF1	0.999383	0.020044	72.879	S	PGLLPSEITRTISINSPKAQTVQAVRCVTSY	783590	327660	6204
Q6Y7W6	25	PERQ amino acid-rich with GYF domain-containing protein 2	GIGYF2	0.426857	0.00576569	72.819	T	FGPEWLRALSSGGSITSPPLSPALPKYKLAD	0	0	7777
P36915	324	Guanine nucleotide-binding protein-like 1	GNL1	0.969485	7.09E-06	72.811	S	EKIARDVAGATWGNGSGEEEEEEDGPAVLVE	1631500	2014500	1431
Q8NHM5	443	Lysine-specific demethylase 2B	KDM2B	0.381975	0.000815477	72.805	S	EGEGRDRAPKPPTDGSTSPTSTPSEDQEALG	0	0	4413
Q8NHM5	447	Lysine-specific demethylase 2B	KDM2B	0.274782	0.000815477	72.805	T	RDRAPKPPTDGSTSPTSTPSEDQEALGKKPK	0	0	7902
Q13370	980	cGMP-inhibited 3',5'-cyclic phosphodiesterase B	PDE3B	0.733935	0.0307299	72.789	S	EEANLGLPISPFMDRSSPQLAKLQESFITHI	0	824340	2477
Q9BZF1	65	Oxysterol-binding protein-related protein 8	OSBPL8	0.695649	0.00396356	72.772	S	EAYPTPTKDLHQPSLSPASPHSQGFERGKED	2317600	887540	5485
Q9BZF1	68	Oxysterol-binding protein-related protein 8	OSBPL8	0.986615	0.00396356	72.772	S	PTPTKDLHQPSLSPASPHSQGFERGKEDISQ	2317600	887540	5486
Q8NCY6	100	Myb/SANT-like DNA-binding domain-containing protein 4	MSANTD4	0.652834	0.000867142	72.771	S	MKANIKLVGSGFPLPSSDLDDSLTEEIDEKI	0	1396500	4332
Q8NCY6	108	Myb/SANT-like DNA-binding domain-containing protein 4	MSANTD4	0.449437	0.000867142	72.771	T	GSGFPLPSSDLDDSLTEEIDEKIGFRNDANF	0	0	7887
Q8NI27	1223	THO complex subunit 2	THOC2	0.499015	3.09E-05	72.707	S	PSSSSIGSASKSDESSTEETDKSRERSQCGV	0	0	4428
O14908	232	PDZ domain-containing protein GIPC1	GIPC1	0.999995	0.0165444	72.681	S	AFDMISQRSAGGRPGSGPQLGTGRGTLRLRS	754680	114170	215
Q8N1G0	433	Zinc finger protein 687	ZNF687	0.592979	0.0212004	72.681	S	SVARKAVVLPGGTATSPKMIAKNVLGLVPQA	2746700	0	4222
Q9BZ23	169	Pantothenate kinase 2, mitochondrial	PANK2	0.551789	0.0170921	72.662	S	DPEGRRQEPLRRRASSASVPAVGASAEGTRR	2372400	1701900	5481
Q14160	1437	Protein scribble homolog	SCRIB	0.806352	0.000291961	72.654	S	TLGEEEQEDEQPPWASPSPTSRQSPASPPPL	0	737790	2678
P41162	245	ETS translocation variant 3	ETV3	0.999432	0.0190424	72.634	S	MLPLFARPGMYPDPHSPFAVSPIPGRGGVLN	247860	617980	1481
P41162	250	ETS translocation variant 3	ETV3	0.999998	0.0190424	72.634	S	ARPGMYPDPHSPFAVSPIPGRGGVLNVPISP	247860	617980	1482
Q9P1Y6	98	PHD and RING finger domain-containing protein 1	PHRF1	0.933302	0.0165989	72.585	S	LLEVAGTQGKLEAAGSFNSDDDAESCPICLN	569770	311290	6202
Q8NB49	447	Phospholipid-transporting ATPase IG	ATP11C	0.491228	0.00187971	72.585	T	HKYKGVTQEVDGLSQTDGTLTYFDKVDKNRE	0	0	7882
Q13542	82	Eukaryotic translation initiation factor 4E-binding protein	EIF4EBP2	0.482828	0.000180337	72.566	T	MAQTPPCHLPNIPGVTSPGTLIEDSKVEVNN	0	0	7558
O15027	589	Protein transport protein Sec16A	SEC16A	0.81751	0.000243239	72.539	S	ETDETTVSQNYRGSVSQPSTSPSPPKPTGIFQ	4375400	4705700	241
O15027	595	Protein transport protein Sec16A	SEC16A	0.990173	0.000243239	72.539	S	VSQNYRGSVSQPSTPSPPKPTGIFQTSANSS	7057900	4705700	242
O15027	593	Protein transport protein Sec16A	SEC16A	0.884292	0.000243239	72.539	T	TTVSQNYRGSVSQPSTPSPPKPTGIFQTSAN	7057900	4705700	7141
O43347	191	RNA-binding protein Musashi homolog 1	MSI1	0.997785	0.0154927	72.531	S	NKMVECKKAQPKEVMSPTGSARGRSRVMPYG	0	1144700	364
Q9H3T2	383	Semaphorin-6C	SEMA6C	0.825174	0.0143425	72.523	S	PRPGSCAGVGGAALFSSSRDLPDDVLTFIKA	1529300	1474800	5645
Q8IWQ3	367	Serine/threonine-protein kinase BRSK2	BRSK2	1	0.0205191	72.485	S	LPPRNEIDPPRKRVDSPMLNRHGKRRPERKS	46703	558890	4071
Q9H814	16	Phosphorylated adapter RNA export protein	PHAX	0.846753	5.45E-05	72.474	S	MALEVGDMEDGQLSDSDSDMTVAPSDRPLQL	0	0	5781
Q9H814	18	Phosphorylated adapter RNA export protein	PHAX	0.848727	5.45E-05	72.474	S	LEVGDMEDGQLSDSDSDMTVAPSDRPLQLPK	0	0	5782
Q86U06	149	Probable RNA-binding protein 23	RBM23	0.999999	0.0191482	72.446	S	GYRYGHSKSPHFREKSPVREPVDNLSPEERD	0	797940	3912
P11274	354	Breakpoint cluster region protein	BCR	0.998415	0.0158257	72.434	S	TSSEEDFSSGQSSRVSPSPTTYRMFRDKSRS	1252100	770500	1015
Q4KMP7	154	TBC1 domain family member 10B	TBC1D10B	0.458928	0.0171611	72.433	T	RPSPAPGPGTPTGTPTRTPSRTAPGALTAKP	0	0	7680
Q4KMP7	156	TBC1 domain family member 10B	TBC1D10B	0.514859	0.017056	72.433	T	SPAPGPGTPTGTPTRTPSRTAPGALTAKPPL	1843000	0	7681
P24928	1853	DNA-directed RNA polymerase II subunit RPB1	POLR2A	0.434664	0.00386234	72.421	Y	KYTPTSPSYSPSSPEYTPTSPKYSPTSPKYS	0	0	8360
Q6P2E9	768	Enhancer of mRNA-decapping protein 4	EDC4	0.485007	0.000303282	72.399	S	SRGFGSSAPEGLEPDSMASAASALHLLSPRP	0	0	3516
Q6P2E9	771	Enhancer of mRNA-decapping protein 4	EDC4	0.485007	0.000303282	72.399	S	FGSSAPEGLEPDSMASAASALHLLSPRPRPG	0	0	3517
Q8NHM5	444	Lysine-specific demethylase 2B	KDM2B	0.480668	0.0011659	72.399	T	GEGRDRAPKPPTDGSTSPTSTPSEDQEALGK	0	0	7901
P55198	382	Protein AF-17	MLLT6	0.948034	0.0018105	72.383	S	DKYSKPTAPAPSAPPSPSAPEPPKADLFEQK	1442100	0	1875
P49761	157	Dual specificity protein kinase CLK3	CLK3	0.999854	0.0170776	72.34	S	AAGAWETMHHCKRYRSPEPDPYLSYRWKRRR	859660	493160	1686
P62995	95	Transformer-2 protein homolog beta	TRA2B	0.999961	0.0115915	72.34	S	RSRSRSYSRDYRRRHSHSHSPMSTRRRHVGN	6664000	5529000	1928
P62995	97	Transformer-2 protein homolog beta	TRA2B	0.999685	0.0115915	72.34	S	RSRSYSRDYRRRHSHSHSPMSTRRRHVGNRA	6664000	5529000	1929
P62995	99	Transformer-2 protein homolog beta	TRA2B	0.999635	0.0115915	72.34	S	RSYSRDYRRRHSHSHSPMSTRRRHVGNRANP	6664000	5529000	1930
Q06730	8	Zinc finger protein 33A	ZNF33A	0.664963	0.0176478	72.321	S	________MNKVEQKSQESVSFKDVTVGFTQ	1522900	0	2210
Q06730	13	Zinc finger protein 33A	ZNF33A	0.99992	0.0176478	72.321	S	___MNKVEQKSQESVSFKDVTVGFTQEEWGH	1522900	0	2211
Q6WKZ4	199	Rab11 family-interacting protein 1	RAB11FIP1	0.55504	0.000794794	72.315	S	SGSDTASAIIPSTTPSVSDSDDESVVKDKKKK	0	0	3634
Q9ULU4	756	Protein kinase C-binding protein 1	ZMYND8	0.956408	0.0035753	72.302	S	DVVGKTPPSTTVGSHSPPETPVLTRSSAQTS	5595000	1554300	6511
Q9ULU4	746	Protein kinase C-binding protein 1	ZMYND8	0.99831	0.0035753	72.302	T	KEPKEPSPKQDVVGKTPPSTTVGSHSPPETP	2801500	1420300	8240
Q9P260	51	LisH domain and HEAT repeat-containing protein KIAA1468	KIAA1468	0.483977	0.00033111	72.292	S	RAVLRLGAGSGLDPGSAGSLSPQDPVALGSS	0	0	6221
P49736	7	DNA replication licensing factor MCM2	MCM2	0.633624	0.00845191	72.29	S	_________MAESSESFTMASSPAQRRRGND	0	509590	1675
Q8IZU2	333	WD repeat-containing protein 17	WDR17	0.911351	0.0207547	72.289	S	LHVLNSPPRKKFSVQSPTKNHYTSSTSEAVP	708140	0	4193
Q8N0Y2	232	Zinc finger protein 444	ZNF444	0.997346	0.00486356	72.289	S	RRKEHLRRHRDTHPGSPGSPGPALRPLPARE	491500	1756700	4194
Q8WYP5	1160	Protein ELYS	AHCTF1	0.999999	0.0207547	72.289	S	YLVSRSLPSSSQLKGSPQAISRASELHLLET	0	806060	4605
Q9UN79	385	Transcription factor SOX-13	SOX13	0.466495	0.0172287	72.243	S	GSPNTPFRKDLISLDSSPAKERLEDGCVHPL	0	0	6534
Q9UN79	386	Transcription factor SOX-13	SOX13	0.466495	0.0172287	72.243	S	SPNTPFRKDLISLDSSPAKERLEDGCVHPLE	0	0	6535
Q9NQ55	240	Suppressor of SWI4 1 homolog	PPAN	0.995322	1.47E-05	72.214	S	QDISELLATGAGLSESEAEPDGDHNITELPQ	6979500	6881300	5900
P49327	1407	Fatty acid synthase;[Acyl-carrier-protein] S-acetyltransfera	FASN	0.570992	0.00653794	72.184	T	KKSFYGSTLFLCRRPTPQDSPIFLPVDDTSF	0	978680	7413
P41212	16	Transcription factor ETV6	ETV6	0.355581	0.000750135	72.145	S	MSETPAQCSIKQERISYTPPESPVPSYASST	0	0	1484
Q09472	2309	Histone acetyltransferase p300	EP300	0.999209	0.0162577	72.096	S	LQGQQIPNSLSNQVRSPQPVPSPRPQSQPPH	1410100	963440	2271
Q15311	645	RalA-binding protein 1	RALBP1	1	0.0254252	72.096	S	AAKEQPKAGKEPAKPSPSRDRKETSI_____	0	0	2900
Q15311	647	RalA-binding protein 1	RALBP1	1	0.0254252	72.096	S	KEQPKAGKEPAKPSPSRDRKETSI_______	0	0	2901
Q86UP2	243	Kinectin	KTN1	0.5444	7.62E-05	72.073	S	IHATTYIPLMDNADSSPVVDKREVIDLLKPD	0	1265600	3934
Q99755	475	Phosphatidylinositol 4-phosphate 5-kinase type-1 alpha	PIP5K1A	0.939647	0.00252592	72.058	S	SKKFRSGSSFSRRAGSSGNSCITYQPSVSGE	0	740380	5274
Q12888	1460	Tumor suppressor p53-binding protein 1	TP53BP1	0.499986	8.22E-05	72.051	S	DSTRRTDVGAGALRRSDSPEIPFQAAAGPSD	2579500	7882100	2360
Q12888	1481	Tumor suppressor p53-binding protein 1	TP53BP1	0.66046	8.22E-05	72.051	S	PFQAAAGPSDGLDASSPGNSFVGLRVVAKWS	2579500	3992700	2363
Q8N163	484	Cell cycle and apoptosis regulator protein 2	CCAR2	0.802874	0.000100383	72.035	T	DALEQAADTSRRNAETPEATTQQETDTDLPE	0	0	7872
Q92558	310	Wiskott-Aldrich syndrome protein family member 1	WASF1	0.815412	0.00111038	71.969	S	TCISSATGLIENRPQSPATGRTPVFVSPTPP	13305000	8446800	4635
Q15047	501	Histone-lysine N-methyltransferase SETDB1	SETDB1	0.40937	0.000894824	71.946	S	QVAKKSTSFRPGSVGSGHSSPTSPALSENVS	0	0	2864
Q86U86	14	Protein polybromo-1	PBRM1	0.824846	0.000830488	71.946	S	__MGSKRRRATSPSSSVSGDFDDGHHSVSTP	5152700	4773800	3920
O60318	530	Germinal-center associated nuclear protein	MCM3AP	0.638116	0.00182329	71.946	T	SLKEKKPGDEVSPSTEDAPFQHSPLGKAAG	0	0	7180
Q9BZF1	63	Oxysterol-binding protein-related protein 8	OSBPL8	0.590847	0.00425942	71.929	S	GKEAYPTPTKDLHQPSLSPASPHSQGFERGK	0	887540	5484
Q9P2M7	129	Cingulin	CGN	0.385504	0.00063664	71.929	S	SDEEPGAYWNGKLLRSHSQASLAGPGPVDPS	0	0	6244
Q5VWN6	1025	Protein FAM208B	FAM208B	0.971354	0.000899262	71.905	S	VQTKTGTLQDLIQHGSPINNECHPSLERKDD	0	0	3362
O43379	1255	WD repeat-containing protein 62	WDR62	0.643567	5.39E-05	71.903	S	LAQPLRRPSSVGELASLGQELQAITTATTPS	0	827800	372
O43379	1267	WD repeat-containing protein 62	WDR62	0.700045	6.10E-05	71.903	T	ELASLGQELQAITTATTPSLDSEGQEPALRS	0	0	7168
Q6AI12	199	Ankyrin repeat domain-containing protein 40	ANKRD40	0.994999	0.00686016	71.894	T	LVQNGDVSAPSAILRTPESTKPGPVCQPPVS	3701700	3054400	7748
Q9NZJ0	557	Denticleless protein homolog	DTL	0.917562	0.0164148	71.888	S	EACSESRNRVKRRLDSSCLESVKQKCVKSCN	192590	143910	6155
O60341	69	Lysine-specific histone demethylase 1A	KDM1A	0.989237	7.45E-06	71.874	S	AVGERTPRKKEPPRASPPGGLAEPPGSAGPQ	2088100	0	483
A7MCY6	390	TANK-binding kinase 1-binding protein 1	TBKBP1	0.950885	0.0108227	71.872	S	PPCPSPQQRRSPASPSCPSPVPQRRSPVPPS	868120	329820	50
Q13625	569	Apoptosis-stimulating of p53 protein 2	TP53BP2	0.823395	0.00202984	71.842	S	LLSPSIPSVGQDQTLSPGSKQESPPAAVRP	826230	1228500	2621
Q9HCM7	141	Fibrosin-1-like protein	FBRSL1	1	0.00400815	71.842	S	PPAEPSENRRPLEAGSPGQDLEPACDGARKV	0	0	5873
Q99700	854	Ataxin-2	ATXN2	0.479286	5.13E-05	71.78	S	SAKDSFIENSSSNCTSGSSKPNSPSISPSIL	0	0	5247
Q99700	863	Ataxin-2	ATXN2	0.570988	5.13E-05	71.78	S	SSSNCTSGSSKPNSPSISPSILSNTEHKRGP	22235000	0	5251
P17600	510	Synapsin-1	SYN1	0.333325	0.000915156	71.756	S	GLGPPAGSPLPQRLPSPTSAPQQPASQAAPP	0	0	1115
P17600	513	Synapsin-1	SYN1	0.333325	0.000915156	71.756	S	PPAGSPLPQRLPSPTSAPQQPASQAAPPTQG	0	0	1116
P17600	512	Synapsin-1	SYN1	0.333325	0.000915156	71.756	T	GPPAGSPLPQRLPSPTSAPQQPASQAAPPTQ	0	0	7299
Q9Y618	149	Nuclear receptor corepressor 2	NCOR2	0.993352	0.00163761	71.735	S	DLTKDRSLTGKLEPVSPPSPPHTDPELELVP	5507900	3789300	6980
Q9Y618	152	Nuclear receptor corepressor 2	NCOR2	0.975814	0.00163761	71.735	S	KDRSLTGKLEPVSPPSPPHTDPELELVPPRL	5507900	0	6981
Q14C86	1096	GTPase-activating protein and VPS9 domain-containing protei	GAPVD1	0.999811	0.000154748	71.678	S	ESAHDSPRDEALQNISADDLPDSASQAAHPQ	2272800	2579200	2832
P49759	140	Dual specificity protein kinase CLK1	CLK1	0.999999	0.0172095	71.656	S	HRRSHGKSHRRKRTRSVEDDEEGHLICQSGD	0	0	1685
P49792;Q;2008;103		E3 SUMO-protein ligase RanBP2;RanBP2-like and GRIP domain-co	RANBP2;RGPI	0.757334	0.0034742	71.608	S	SGDFEKDDDAYKTEDSDDIHFEPVVQMPEKV	0	0	1700
P49792;Q;2005;103		E3 SUMO-protein ligase RanBP2;RanBP2-like and GRIP domain-co	RANBP2;RGPI	0.805959	0.0034742	71.608	T	ANTSGDFEKDDDAYKTEDSDDIHFEPVVQMP	0	0	7421
O75436	318	Vacuolar protein sorting-associated protein 26A	VPS26A	0.533613	0.0237349	71.607	S	RKQRTNFHQRFESPESQASAEQPEM______	2197900	0	627
Q8TAP8	47	Protein phosphatase 1 regulatory subunit 35	PPP1R35	0.500883	0.00192818	71.592	S	QLRAPVPEPGLDLSLSPRPDSPQPRHGSPGR	0	0	4438
Q92766	1320	Ras-responsive element-binding protein 1	RREB1	0.596219	0.00208039	71.592	S	VAGDAPVEQATAETASPVHREEHGRGESHEP	0	0	4697
Q8WXE1	518	ATR-interacting protein	ATRIP	0.997675	0.0183002	71.558	S	DSAAGEGNRSLVHRLSDGDMTSALRGVADDQ	586730	474540	4590
Q92729	792	Receptor-type tyrosine-protein phosphatase U	PTPRU	0.999931	0.0199663	71.555	T	YAYSYYPKPVNMTKATVNYRQEKTHMMSAVD	12443000	6666300	7942
Q92729	795	Receptor-type tyrosine-protein phosphatase U	PTPRU	0.999921	0.0199663	71.555	Y	SYYPKPVNMTKATVNYRQEKTHMMSAVDRSF	12443000	6666300	8402
Q99576	122	TSC22 domain family protein 3	TSC22D3	0.999876	0.000571904	71.54	S	EKFQSCLSPEEPAPESPQVPEAPGGSAV___	899830	907770	5216
P46821	1446	Microtubule-associated protein 1B;MAP1B heavy chain;MAP1 li	MAP1B	0.898604	0.00167209	71.535	S	EKSGKQGSPDQVSPVSEMTSTSLYQDKQEGK	6743700	0	1589
Q15413	419	Ryanodine receptor 3	RYR3	0.503303	0.0238577	71.531	S	ESQAARIIRNTTALFSQFVSGNNRTAAPITL	32893000	43861000	2916
Q9NTI5	1259	Sister chromatid cohesion protein PDS5 homolog B	PDS5B	0.333333	0.016779	71.531	S	KGSQRSRKRGHTASESDEQQWPEEKRLKEDI	0	0	6007
Q70CQ2	3358	Ubiquitin carboxyl-terminal hydrolase 34	USP34	0.968646	0.00612536	71.527	S	TKDDEGATPIKRRRVSSDEEHTVDSCISDMK	1489000	1258900	3682
Q70CQ2	3359	Ubiquitin carboxyl-terminal hydrolase 34	USP34	0.968646	0.00612536	71.527	S	KDDEGATPIKRRRVSSDEEHTVDSCISDMKT	1489000	1258900	3683
Q9H4Z3	143	Phosphorylated CTD-interacting factor 1	PCIF1	0.456604	0.000752508	71.527	S	PKIEIPVTPTGQSVPSSPSIPGTPTLKMWGT	0	0	5702
Q9H4Z3	144	Phosphorylated CTD-interacting factor 1	PCIF1	0.456604	0.000752508	71.527	S	KIEIPVTPTGQSVPSSPSIPGTPTLKMWGTS	0	0	5703
Q9H8Y8	436	Golgi reassembly-stacking protein 2	GORASP2	0.924915	0.00117167	71.527	S	APTTVEDRVGDSTPVSEKPVSAAVDANASES	0	2711300	5794
Q9H0E3	465	Histone deacetylase complex subunit SAP130	SAP130	1	0.018369	71.513	S	PAERSSLIPISGHRASPNPVAMETRSDNRPS	762770	592890	5577
Q9NVR2	231	Integrator complex subunit 10	INTS10	0.703373	0.00156004	71.506	S	IENQHQGAQDTSDLMSPSKRSSQKYIIEGLT	0	0	6047
Q8N556	282	Actin filament-associated protein 1	AFAP1	0.406796	0.00120306	71.492	S	VHKAELEKKLSSERPSSDGEGVVENGITTCN	0	0	4276
Q8NC44	281	Protein FAM134A	FAM134A	0.999969	0.000193124	71.47	S	KNAPPGGDEPLAETESESEAELAGFSPVVDV	3094900	2336500	4318
Q8NC44	283	Protein FAM134A	FAM134A	0.999972	0.000193124	71.47	S	APPGGDEPLAETESESEAELAGFSPVVDVKK	3094900	2336500	4319
Q9H6F5	160	Coiled-coil domain-containing protein 86	CCDC86	0.832074	8.89E-06	71.47	S	LTPGAPQHQLPPVPGSPEPYPGQQAPGPEPS	4997300	1343900	5727
Q8NC44	279	Protein FAM134A	FAM134A	0.999994	0.000193124	71.47	T	QGKNAPPGGDEPLAETESESEAELAGFSPVV	3094900	2336500	7885
O60749	101	Sorting nexin-2	SNX2	0.995132	0.00212115	71.434	T	PEREPILSSEPSPAVTPVTPTTLIAPRIESK	305180	477080	7184
O60749	106	Sorting nexin-2	SNX2	0.414913	0.00212115	71.434	T	ILSSEPSPAVTPVTPTTLIAPRIESKSMSAP	0	0	7186

O60749	107	Sorting nexin-2	SNX2	0.38399	0.00212115	71.434	T	LSSEPSPAVTPVTPTTLIAPRIESKSMSAPV	0	0	7187
O94842	174	TOX high mobility group box family member 4	TOX4	0.741105	0.00256508	71.428	S	GGTILPPAQSPEDRLSTTPSPTSSLHEDGVE	0	0	703
Q96RY5	307	Protein cramped-like	CRAMP1L	1	0.0117921	71.344	S	PMCRALKKLCDPDGLSDEEDQKPVRLPLKVP	0	0	5106
O95639	200	Cleavage and polyadenylation specificity factor subunit 4	CPSF4	0.45355	0.00664505	71.309	S	TQPPAKQSNNPPLQRSSSLIQLTSQNSSPNQ	0	0	784
O95639	201	Cleavage and polyadenylation specificity factor subunit 4	CPSF4	0.45355	0.00664505	71.309	S	QPPAKQSNNPPLQRSSSLIQLTSQNSSPNQQ	0	0	785
Q12888	1480	Tumor suppressor p53-binding protein 1	TP53BP1	0.491838	9.23E-05	71.295	S	IPFQAAAGPSDGLDASSPGNSFVGLRVVAKW	0	0	2362
Q5QJ74	41	Tubulin-specific chaperone cofactor E-like protein	TBCEL	0.99853	0.0224146	71.279	S	RGPGMGVHVPATPQGSPMKDRLNLPSVLVLN	0	659250	3205
Q13625	558	Apoptosis-stimulating of p53 protein 2	TP53BP2	0.828001	0.00230743	71.265	S	KPKPAGQQPRVLLSPSIPSVGQDQTLSPGSK	826230	1170300	2620
Q9Y618	1479	Nuclear receptor corepressor 2	NCOR2	0.999999	0.0220403	71.221	S	STTGSKKHDVRSLIGSPGRTFPPVHPLDVMA	1140200	472540	6982
Q9P1Y6	915	PHD and RING finger domain-containing protein 1	PHRF1	0.771545	0.000996431	71.202	S	SAMSKLRGAVAAEGASDTEREEPTESQGLAA	8640200	0	6201
Q99618	43	Cell division cycle-associated protein 3	CDCA3	0.803045	0.00170973	71.174	S	RSPSAGILRTPIQVESSPQPGLPAGEQLEGL	1355400	678120	5234
Q99618	44	Cell division cycle-associated protein 3	CDCA3	0.718969	0.00170973	71.174	S	SPSAGILRTPIQVESSPQPGLPAGEQLEGLK	1355400	678120	5235
Q99618	37	Cell division cycle-associated protein 3	CDCA3	0.666667	0.00170973	71.174	T	ARVADPRSPSAGILRTPIQVESSPQPGLPAG	1355400	678120	8028
Q9H251	2810	Cadherin-23	CDH23	0.999998	0.0137377	71.148	S	REAIFSFIVKASSNRSWTPPRGPSPTLDLVA	0	0	5610
Q9H251	2818	Cadherin-23	CDH23	0.983129	0.0137377	71.148	S	VKASSNRSWTPPRGPSPTLDLVADLTLQEVR	0	0	5611
Q9H251	2812	Cadherin-23	CDH23	0.999998	0.0137377	71.148	T	AIFSFIVKASSNRSWTPPRGPSPTLDLVADL	0	0	8090
Q9H251	2820	Cadherin-23	CDH23	0.623793	0.0137377	71.148	T	ASSNRSWTPPRGPSPTLDLVADLTLQEVRVV	0	0	8091
Q6ZRS2	274	Helicase SRCAP	SRCAP	0.775854	0.00161823	71.143	S	KAGSSPCLGSSSAASSPPPPASRLDDEDGDF	0	338630	3658
P61978	116	Heterogeneous nuclear ribonucleoprotein K	HNRNPK	0.471614	1.99E-05	71.098	S	LKKIIPTLEEGLQLPSPTATSQLPLESDAVE	0	0	1916
Q04721	2070	Neurogenic locus notch homolog protein 2;Notch 2 extracellu	NOTCH2	0.906096	0.000282425	71.091	S	HHDIVRLLDEYNVTPSPPGTVLTSALSPVIC	0	0	2165
Q04721	2081	Neurogenic locus notch homolog protein 2;Notch 2 extracellu	NOTCH2	0.869973	0.000282425	71.091	S	NVTPSPPGTVLTSALSPVICGPNRSFLSLKH	0	0	2167
Q92797	1173	Symplekin	SYMPK	0.929	0.00278698	71.073	S	KLKPGGVGAPSSSSPSPSPSARPGPPPSEEA	18969000	16200000	4712
Q00537	122	Cyclin-dependent kinase 17	CDK17	0.946727	0.000464672	71.071	S	GESDQASGTSSDEVQSPTGVCLRNRIHRRIS	1179900	1489600	2022
Q96RK0	142	Protein capicua homolog	CIC	0.974417	0.000381899	71.071	S	TEEEASGPPGEPRLDSETESDHDDAFLSIMS	813360	0	5093
Q96RK0	144	Protein capicua homolog	CIC	0.765741	0.000381899	71.071	T	EEASGPPGEPRLDSETESDHDDAFLSIMSPE	813360	0	8004
Q8N5U6	456	RING finger protein 10	RNF10	1	2.92E-05	71.068	S	SRPLALPLVEEEEAVSEPEPEGLPEACDDLE	1012900	1360900	4289
Q8N3X1	438	Formin-binding protein 4	FNBP4	0.37497	0.000452333	71.06	S	EGDGSVSGSSPRSDISQPASQDGMRRLMSKR	0	0	4263
P11388	1244	DNA topoisomerase 2-alpha	TOP2A	0.826845	0.00377723	71.06	T	AEAEKKNKKKIKNENTEGSPQEDGVELEGLK	1338100	1243300	7278
O14994	484	Synapsin-3	SYN3	0.986973	0.0176178	71.03	S	LSPQSGSPQQQRSPGSPQLSRASSGSSPNQA	916680	438710	223
P09496	105	Clathrin light chain A	CLTA	0.999968	0.0291484	71.03	S	PTDSYAAISQVDRLQSEPESIRKWREEQMER	14118000	251680	968
P15924	2209	Desmoplakin	DSP	0.999695	0.0222702	71.03	S	PHTGLLLLSVQKRSMSFQGIRQPVTVTELVD	748440	0	1077
Q13111	783	Chromatin assembly factor 1 subunit A	CHAF1A	0.907422	0.00319397	70.987	T	NHTGSPRSPSTTYLHTPTPSEDAAIPSKSRL	2964000	1251000	7523
Q15424	209	Scaffold attachment factor B1	SAFB	0.999999	0.000246213	70.983	S	TSSSDFTILQEIEEPSLEPENEKILDILGET	1032000	975730	2927
Q5VZ89	1125	DENN domain-containing protein 4C	DENND4C	0.493742	0.000997789	70.983	S	SRTCMSESTWNPEHRSSPVPEMLEESQELLE	0	0	3370
Q5VZ89	1126	DENN domain-containing protein 4C	DENND4C	0.493742	0.000997789	70.983	S	RTCMSESTWNPEHRSSPVPEMLEESQELLEP	0	0	3371
Q9UER7	176	Death domain-associated protein 6	DAXX	0.528073	1.53E-05	70.98	S	PTHLSLDPTNAENTASQSPRTRGSRRQTQRL	0	0	6289
Q5T200	318	Zinc finger CCCH domain-containing protein 13	ZC3H13	0.915462	0.0206275	70.944	S	DFERQREKRDKPRSTSPAGQHHSPISSRHHS	7102800	5087000	3272
Q5T200	325	Zinc finger CCCH domain-containing protein 13	ZC3H13	0.986854	0.0206275	70.944	S	KRDKPRSTSPAGQHHSPISSRHHSSSSSGSS	7102800	5087000	3273
Q96BY7	1579	Autophagy-related protein 2 homolog B	ATG2B	0.991244	0.0132423	70.942	S	DFGIVPPTSPAKSYISPHSSPSHTPTRHGRN	7891600	3509300	4812
Q96BY7	1582	Autophagy-related protein 2 homolog B	ATG2B	0.579853	0.0132423	70.942	S	IVPPTSPAKSYISPHSSPSHTPTRHGRNTVC	7891600	0	4813
Q96T60	122	Bifunctional polynucleotide phosphatase/kinase;Polynucleotid	PNKP	0.813299	0.00439767	70.942	T	EETRTPESQPDTPPGTPLVSQDEKRDAELPK	0	1055300	8018
P36507	26	Dual specificity mitogen-activated protein kinase kinase 2	MAP2K2	0.564597	4.31E-05	70.889	S	ALTINPTIAEGPSPTSEGASEANLVDLQKKL	0	1251400	1425
Q13136	763	Liprin-alpha-1	PPFIA1	0.851857	0.0110029	70.889	S	ALRLDRLHKGALHTVSHEDIRDIRNSTGSQD	1336600	0	2414
Q9UDY2	400	Tight junction protein ZO-2	TJP2	0.69526	0.000292754	70.799	S	SQQTLINIPSLNDSDSEIEDISEIESNRSFS	9040100	5669200	6280
Q03164	3038	Histone-lysine N-methyltransferase 2A;MLL cleavage product N	KMT2A	0.595691	0.0035285	70.767	T	TRNSSTPGLQVPVSPTVPIQNQKYVPNSTDS	0	0	7485
Q96A19	12	Coiled-coil domain-containing protein 102A	CCDC102A	0.998827	0.0180033	70.728	S	____MSHGPSPRLAESPQLSKGSLLTILGSP	284380	269570	4765
P49848	626	Transcription initiation factor TFIID subunit 6	TAF6	0.706189	0.000296098	70.704	S	LPPTGEGKGGPTSHPSPVPPASSPSPLSGS	1443000	1187900	1737
P49848	633	Transcription initiation factor TFIID subunit 6	TAF6	0.567791	0.000296098	70.704	S	KGGPTSHPSPVPPPASSPSPLSGSALCGGKQ	1443000	1187900	1738
Q8NEY1	1382	Neuron navigator 1	NAV1	0.900757	0.00121675	70.699	S	SGSTPGQVPGSSALSSPRRSLGLALTHSFGP	1609700	1228900	4378
P27707	15	Deoxycytidine kinase	DCK	0.394984	0.0246452	70.68	S	_MATPPKRSCPSFSASSEGTRIKKISIEGNI	0	0	1284
P27707	16	Deoxycytidine kinase	DCK	0.394984	0.0246452	70.68	S	MATPPKRSCPSFSASSEGTRIKKISIEGNIA	0	0	1285
P49736	53	DNA replication licensing factor MCM2	MCM2	0.993691	3.41E-05	70.666	S	LTSSPGRDLPPFEDESEGLLGTEGPLEEEED	4678400	3990300	1678
Q14676	1705	Mediator of DNA damage checkpoint protein 1	MDC1	0.523128	0.00017479	70.647	T	RAMPVPTTPEFQSPVTTDQPISPEPITQPSC	0	0	7600
Q14247	417	Src substrate cortactin	CTTN	0.499174	0.0131553	70.628	S	PPVSPAPQPTEERLPSSPVYEDAASFKAELS	0	0	2730
Q6WCQ1	294	Myosin phosphatase Rho-interacting protein	MPRIP	0.685652	0.0033028	70.615	S	AEEQQLPPPLSPPSPSTPNHRRSQVIEKFEA	2658100	684910	3625
Q6WCQ1	295	Myosin phosphatase Rho-interacting protein	MPRIP	0.685652	0.0033028	70.615	T	EEQQLPPPLSPPSPSTPNHRRSQVIEKFEAL	2658100	684910	7776
Q86T24	237	Transcriptional regulator Kaiso	ZBTB33	0.729182	8.02E-06	70.561	S	GPVAISDVAPSASNNSPPLTNITPTQKLPTP	763500	1450500	3894
O60293	1301	Zinc finger C3H1 domain-containing protein	ZFC3H1	0.679962	0.00270946	70.555	S	RKWKPKFWRKPISDNSFSSDEEQSTGPIKYA	13880000	0	471
Q9Y6A5	315	Transforming acidic coiled-coil-containing protein 3	TACC3	0.5	0.00454719	70.555	T	ESTAPTNHLVAGRAMTLSPQEEVAAGQMASS	0	0	8316
O15119	438	T-box transcription factor TBX3	TBX3	0.981925	0.0198719	70.552	S	RDSGRLDKASPDSRHSPATISSSTRGLGAEE	0	0	288
Q9H6F5	102	Coiled-coil domain-containing protein 86	CCDC86	1	0.0118711	70.54	S	PGAASPQRQQDLHLESPQRQPEYSPESPRCQ	0	1552900	5733
Q12888	103	Tumor suppressor p53-binding protein 1	TP53BP1	0.237367	0.000606306	70.533	S	KVADPVDSSNLDTCGSISQVIEQLPQPNRTS	0	0	2373
Q12888	105	Tumor suppressor p53-binding protein 1	TP53BP1	0.237367	0.000606306	70.533	S	ADPVDSSNLDTCGSISQVIEQLPQPNRTSSV	0	0	2374
Q12888	100	Tumor suppressor p53-binding protein 1	TP53BP1	0.237367	0.000606306	70.533	T	KENKVADPVDSSNLDTCGSISQVIEQLPQPN	0	0	7516
O94819	107	Kelch repeat and BTB domain-containing protein 11	KBTBD11	1	0.0267823	70.47	S	ASPEERACPEEPAAPSPEPRVWLEDPASPEE	562780	522570	700
Q03164	2098	Histone-lysine N-methyltransferase 2A;MLL cleavage product N	KMT2A	0.96961	0.0255854	70.47	S	INSTVEHDENRTIAHSPTSFTESSSKESQNT	0	0	2149
Q9UPN4	416	Centrosomal protein of 131 kDa	CEP131	0.423153	0.00702146	70.47	S	LPAAGPGDRCLPTSDSSPEPQQPPEDRTQDV	0	0	6557
Q9UPN4	417	Centrosomal protein of 131 kDa	CEP131	0.423153	0.00702146	70.47	S	PAAGPGDRCLPTSDSSPEPQQPPEDRTQDVL	0	0	6558
Q9HCK8	1978	Chromodomain-helicase-DNA-binding protein 8	CHD8	0.926138	0.00702979	70.452	S	NYMQNHQAGAPAPSLSRCSTPLLHQQYTSRT	0	623860	5864
Q53ET0	173	CREB-regulated transcription coactivator 2	CRTC2	0.167851	0.000104934	70.451	S	QLFRLPSALNRTSSDSALHTSVMNPSPQDTY	0	0	3130
Q53ET0	178	CREB-regulated transcription coactivator 2	CRTC2	0.166285	0.000104934	70.451	S	PSALNRTSSDSALHTSVMNPSPQDTYPGPTP	0	0	3131
Q53ET0	177	CREB-regulated transcription coactivator 2	CRTC2	0.167851	0.000104934	70.451	T	LPSALNRTSSDSALHTSVMNPSPQDTYPGPT	0	0	7694
Q53ET0	187	CREB-regulated transcription coactivator 2	CRTC2	0.542156	0.000104934	70.451	T	DSALHTSVMNPSPQDTYPGPTPPSILPSRRG	915970	1446000	7695
Q15154	960	Pericentriolar material 1 protein	PCM1	0.998369	0.0145955	70.438	S	NGKETKNRWKNNCPFSADENYRPLAKTRQQN	1595900	309960	2878
Q9BUW7	82	UPF0184 protein C9orf16	C9orf16	0.997837	0.0230566	70.438	S	RLEFQQQLGEAPSDASP______________	1468600	1565700	5393
P35251	312	Replication factor C subunit 1	RFC1	0.942577	0.0230309	70.399	S	QQHSKSSADKIGEVSSPKASSKLAIMKRKEE	0	463450	1384
Q9H7N4	725	Splicing factor, arginine/serine-rich 19	SCAF1	0.499858	0.020111	70.399	S	LDKSDPRGPSPAPASSPKREVLYDSEGLSGE	0	0	5770
Q9BWG6	183	Sodium channel modifier 1	SCNM1	0.576817	0.000658633	70.316	S	PQAEESATVSAPAPMSPTRRRALDHYLTLRS	641560	388630	5429
Q96D71	401	RalBP1-associated Eps domain-containing protein 1	REPS1	0.441487	0.000508437	70.295	S	EVGYSGSPAEAPPSKSPSMPSLNQTWPELNQ	0	0	4849
Q96D71	403	RalBP1-associated Eps domain-containing protein 1	REPS1	0.441487	0.000508437	70.295	S	GYSGSPAEAPPSKSPSMPSLNQTWPELNQSS	0	0	4850
Q8IY67	463	Ribonucleoprotein PTB-binding 1	RAVER1	1	0.00275434	70.295	T	GDREALGLGPPAAQLTPPPAPVGLRGSGLRG	1159800	1223200	7856
Q02880	1421	DNA topoisomerase 2-beta	TOP2B	0.793457	0.000346019	70.295	Y	GEDEFVPSDGLDKDEYTFSPGKSKATPEKSL	1721400	0	8371
Q66K74	741	Microtubule-associated protein 1S;MAP1S heavy chain;MAP1S li	MAP1S	0.999996	0.00319768	70.235	S	ARRSASPHDVDLCLVSPCEFEHRKAVPMAPA	0	859510	3411
Q92614	1942	Unconventional myosin-XVIIIa	MYO18A	0.999942	0.000186153	70.229	S	KRIGDLQAAIEDEMESDENEDLINSLQDMVT	1389300	1107300	4662
Q5SW79	627	Centrosomal protein of 170 kDa	CEP170	0.498139	0.0272662	70.197	S	LENETEISESGMTVRSTGSATSLASQGERRR	0	0	3225
O43795	893	Unconventional myosin-Ib	MYO1B	0.500011	0.0296638	70.167	S	VQKYFLEMKNKMPSLSPIDKNWPSRPYLFLD	0	204660	418
O43399	23	Tumor protein D54	TPD52L2	0.891004	0.00714863	70.121	T	INLNSPNKGLLSDSMTDVPVDTGVAARTPAV	459390	490420	7170
Q7Z2W4	347	Zinc finger CCCH-type antiviral protein 1	ZC3HAV1	0.312924	0.000100468	70.112	T	ENGSQEDLLHGNPGSTYLASNSTSAPNWKSL	0	0	7797
P35606	861	Coatomer subunit beta'	COPB2	0.92996	0.00116237	70.109	T	RSTAQQELDGKPASPTPVIVASHTANKEEKS	0	588880	7362
P46013	2466	Antigen KI-67	MKI67	0.999982	0.0233903	70.1	S	EESMTDDKITEVSCKSPQPESFKTSRSSKQR	0	516560	1539
O00159	408	Unconventional myosin-Ic	MYO1C	0.80696	0.0234768	70.028	S	LVGKINRSLASKDVESPSWRSTTVLGLLDIY	691020	372880	93
Q70E73	1010	Ras-associated and pleckstrin homology domains-containing pr	RAPH1	0.491791	0.0235918	70.028	S	PSVDSLVSKFTPPAESGSPSKETLPPPAAPP	0	0	3684
Q70E73	1012	Ras-associated and pleckstrin homology domains-containing pr	RAPH1	0.828021	0.0235918	70.028	S	VDSLVSKFTPPAESGSPSKETLPPPAAPPKP	0	0	3685
Q7Z6E9	244	E3 ubiquitin-protein ligase RBBP6	RBBP6	0.547834	0.000242991	70.027	S	AIGKKEKPPFLPEEPSSSSEEDDPIPDELLC	0	0	3854
Q969V6	792	MKL/myocardin-like protein 1	MKL1	0.542979	6.53E-05	70.024	S	PSPKTVCGSPLAAQPSPSAELPQAAPPPPGS	0	5799200	4756
Q969V6	809	MKL/myocardin-like protein 1	MKL1	0.599262	6.53E-05	70.024	S	SAELPQAAPPPPGSPSLPGRLEDFLESSTGL	814040	8725900	4758
P62491	190	Ras-related protein Rab-11A	RAB11A	0.934768	0.00140782	69.99	S	IVSQKQMSDRRENDMSPSNNVVPIHVPPTTE	2057400	0	1922
Q8WYQ5	373	Microprocessor complex subunit DGCR8	DGCR8	0.811467	0.00378806	69.979	S	MKDNEEREQSSDLTPSGDVSPVKPLSRSAEL	0	0	4611
P10244	395	Myb-related protein B	MYBL2	0.327223	0.00129387	69.947	S	DLSRSSRGELIPISPSTEVGGSGIGTPPSVL	0	0	985
P10244	396	Myb-related protein B	MYBL2	0.327227	0.00129387	69.947	T	LSRSSRGELIPISPSTEVGGSGIGTPPSVLK	0	0	7271
Q9Y2K6	132	Ubiquitin carboxyl-terminal hydrolase 20	USP20	0.990597	0.0247986	69.935	S	HPLKAVPIAVADEGESESEDDDLKPRGLTGM	934590	537110	6791
Q8NHM5	445	Lysine-specific demethylase 2B	KDM2B	0.614153	0.00114196	69.913	S	EGRDRAPKPPTDGSTSPTSTPSEDQEALGKK	4071700	0	4414
Q96L91	941	E1A-binding protein p400	EP400	0.873777	1.30E-05	69.873	S	ESSLDSGMSGRKRKASISLTDDEVDDEEETI	1596700	1151700	5016
Q96L91	943	E1A-binding protein p400	EP400	0.760667	1.30E-05	69.873	S	SLDSGMSGRKRKASISLTDDEVDDEEETIEE	1596700	1151700	5017
A7E2V4	1155	Zinc finger SWIM domain-containing protein 8	ZSWIM8	0.504925	0.00162121	69.857	S	GMASIDSSAPETTSDSSPTLSRRPLRGGWAP	1302200	0	44
A7E2V4	1152	Zinc finger SWIM domain-containing protein 8	ZSWIM8	0.476299	0.00162121	69.857	T	KHTGMASIDSSAPETTSDSSPTLSRRPLRGG	0	0	7111
Q8N1F8	761	Serine/threonine-protein kinase 11-interacting protein	STK11IP	1	0.0193418	69.846	S	CHPPGHGDHLDRAKNSPPQAPSTRDHGSWSL	1411100	985550	4216
Q68DK2	1762	Zinc finger FYVE domain-containing protein 26	ZFYVE26	0.80449	0.0219198	69.845	S	QEIVHQAADPETLPRSPSAEFSPAAPPGISS	573920	722890	3420
P85037	236	Forkhead box protein K1	FOXK1	0.521796	0.0016354	69.765	S	QISPLKIHIPEPDLRSMVSPVPSPTGTISVP	0	649690	2001
A3KN83	679	Protein strawberry notch homolog 1	SBNO1	0.999897	0.0184388	69.747	S	LIEKHFPAPDRKKLYSLLGIDLTAPSNNSSP	0	115430	26
A3KN83	678	Protein strawberry notch homolog 1	SBNO1	0.999926	0.0184388	69.747	Y	SLIEKHFPAPDRKKLYSLLGIDLTAPSNNSS	0	115430	8338
Q13428	671	Treacle protein	TCOF1	0.852507	0.00486334	69.737	T	VAPVRVGTQAPRKAGTATSPAGSSPAVAGGT	771900	0	7534
Q96C19	76	EF-hand domain-containing protein D2	EFHD2	0.583946	0.0132992	69.729	S	RRADLNQGIGEPQSPSRRVFNPYTEFKEFSR	6246900	0	4817
P54725	128	UV excision repair protein RAD23 homolog A	RAD23A	0.537815	0.000245732	69.726	S	HPPPAAREDKSPSEESAPTTSPESVSGSVPS	0	1557200	1853
P54725	133	UV excision repair protein RAD23 homolog A	RAD23A	0.799115	0.000245732	69.726	S	AREDKSPSEESAPTTSPESVSGSVPSSGSSG	832460	1557200	1854
Q7Z7L8	407	Uncharacterized protein C11orf96	C11orf96	0.497978	0.01929	69.721	T	AKKSFLQSLECLRRSTQSLSLQREQLSSCKL	0	0	7820
Q13136	786	Liprin-alpha-1	PPFIA1	0.321967	0.000912489	69.72	S	RNSTGSQDGPVSNPSSSNSSQDSLHKAPKKK	0	0	2416
Q53ET0	183	CREB-regulated transcription coactivator 2	CRTC2	0.527963	0.000127309	69.72	S	RTSSDSALHTSVMNPSPQDTYPGPTPPSILP	0	0	3132
P20700	575	Lamin-B1	LMNB1	1	0.000310183	69.704	T	AAGVVVEEELFHQQGTPRASNRSCAIM____	3131700	1882900	7311
Q9UEW8	370	STE20/SPS1-related proline-alanine-rich protein kinase	STK39	1	0.0103115	69.65	S	PDIAQRAKKVRRVPGSSGHLHKTEDGDWEWS	1216300	373430	6297
Q13112	410	Chromatin assembly factor 1 subunit B	CHAF1B	0.556542	0.015327	69.612	S	PDTAKKTKSQTHRGSSPGPRPVEGTPASRTQ	9476500	0	2407
Q92766	36	Ras-responsive element-binding protein 1	RREB1	0.990007	0.0100315	69.612	S	MSAVMSVGKVTENGGSPQGIKSPSKPPGPNR	1434700	640920	4698
Q92766	42	Ras-responsive element-binding protein 1	RREB1	0.631403	0.0100315	69.612	S	VGKVTENGGSPQGIKSPSKPPGPNRIGRRNQ	1434700	0	4699
Q969V6	447	MKL/myocardin-like protein 1	MKL1	0.711916	0.0229334	69.581	T	VVATVASSGVVKFGSTGSTPPVSPTPSERSL	0	1853900	7960
Q969V6	456	MKL/myocardin-like protein 1	MKL1	0.764549	0.0229334	69.581	T	VVKFGSTGSTPPVSPTPSERSLLSTGDENST	0	1853900	7962
Q9H4Z3	150	Phosphorylated CTD-interacting factor 1	PCIF1	0.943335	0.00134806	69.525	T	TPTGQSVPSSPSIPGTPTLKMWGTSPEDKQQ	2738100	2908700	8103
Q9NYZ3	705	G2 and S phase-expressed protein 1	GTSE1	0.952016	9.24E-05	69.521	S	VVGQLIDLSSPLIQLSPEAEKENVDSPLLKF	972850	1294200	6145
Q9UEY8	600	Gamma-adducin	ADD3	0.876974	3.93E-05	69.521	S	SDDASSVSQIQSQTQSPQNVPEKLEENHELF	0	1085500	6305
Q86T65	656	Disheveled-associated activator of morphogenesis 2	DAAM2	0.765693	0.026751	69.456	S	EKMFSAYQRHQKELGSTEDIYLASRKVKELS	792100	406980	3895
P25205	535	DNA replication licensing factor MCM3	MCM3	0.856985	2.32E-05	69.418	S	LGSAVDILATDDPNFSQEDQQDTQIYEKHDN	789700	973780	1268
Q9P258	51	Protein RCC2	RCC2	0.715104	0.0194878	69.398	S	RERPERCSSSSGGGSSGDEDGLELDGAPGGG	0	0	6217
P36507	30	Dual specificity mitogen-activated protein kinase kinase 2	MAP2K2	0.496571	7.03E-05	69.385	S	NPTIAEGPSPTSEGASEANLVDLQKKLEELE	0	0	1426
Q66K14	1238	TBC1 domain family member 9B	TBC1D9B	0.583801	0.0274177	69.345	S	IGLKIKDQKKVERQFSTASDHEQPGVSG___	1234700	622390	3402
Q96KC8	484	DnaJ homolog subfamily C member 1	DNAJC1	0.978826	0.00704817	69.331	S	KDFDIAEQNESSDEESLRKERARSAEEPWTQ	820000	617240	5006
P42694	1614	Probable helicase with zinc finger domain	HELZ	0.999727	0.000731256	69.292	S	PPQSRLLQYRQVQSRSPPAVPSPPSSTDHSS	8635500	2199300	1516
Q01970	926	1-phosphatidylinositol 4,5-bisphosphate phosphodiesterase be	PLCB3	0.73729	0.0030021	69.284	S	SPLDASPRRPPGPTTSPASTSLSSPGQRDDL	3894200	0	2088
Q01970	934	1-phosphatidylinositol 4,5-bisphosphate phosphodiesterase be	PLCB3	0.594079	0.0030021	69.284	S	RPPGPTTSPASTSLSSPGQRDDLIASILSEV	5632000	1188500	2089
Q96D71	560	RalBP1-associated Eps domain-containing protein 1	REPS1	0.443698	0.0243831	69.276	S	APPPPPPRPQPSHSRSSSLDMNRTFTVTTGQ	0	0	4851
Q96D71	561	RalBP1-associated Eps domain-containing protein 1	REPS1	0.443698	0.0243831	69.276	S	PPPPPPRPQPSHSRSSSLDMNRTFTVTTGQQ	0	0	4852

Q9H4A3	2012	Serine/threonine-protein kinase WNK1	WNK1	0.88025	0.00118739	69.272	S	EKPELSEPSHLNGPSSDPEAAFLSRDVDDGS	0	0	5668
Q7Z401	1015	C-myc promoter-binding protein	DENND4A	0.5	0.0302884	69.261	S	ETRNIQSRCFRKRHKSDNETNLQQQVVWGNR	0	399940	3789
Q5TDH0	118	Protein DDI1 homolog 2	DDI2	0.310597	0.000347501	69.248	S	GTSSPRQRQPPGTQQSHSSPGEITSSPQGLD	0	0	3303
Q5TDH0	120	Protein DDI1 homolog 2	DDI2	0.424841	0.000347501	69.248	S	SSPRQRQPPGTQQSHSSPGEITSSPQGLDNP	0	0	3304
Q5TDH0	121	Protein DDI1 homolog 2	DDI2	0.424833	0.000347501	69.248	S	SPRQRQPPGTQQSHSSPGEITSSPQGLDNPA	0	0	3305
Q5TDH0	128	Protein DDI1 homolog 2	DDI2	0.846448	0.000347501	69.248	S	PGTQQSHSSPGEITSSPQGLDNPALLRDMLL	2652500	2863600	3306
Q9UJF2	635	Ras GTPase-activating protein nGAP	RASAL2	0.463454	0.00345888	69.248	S	TPIQQQLRRFTEHNSSPNVSGSLSSGLQKIF	0	0	6396
Q9UJF2	641	Ras GTPase-activating protein nGAP	RASAL2	0.667645	0.00345888	69.248	S	LRRFTEHNSSPNVSGSLSSGLQKIFEDPTDS	588060	94148	6397
Q8IXM2	146	Chromatin complexes subunit BAP18	BAP18	0.932733	0.000579152	69.241	S	IKKQKADVTLSALNDSDANSDVVDIEGLGET	772000	533420	4103
Q8IXM2	150	Chromatin complexes subunit BAP18	BAP18	0.963005	0.000579152	69.241	S	KADVTLSALNDSDANSDVVDIEGLGETPPAK	772000	533420	4104
Q8IXM2	161	Chromatin complexes subunit BAP18	BAP18	0.999998	0.000579152	69.241	T	SDANSDVVDIEGLGETPPAKKLNFDQA____	772000	533420	7855
Q9HCD6	1530	Protein TANC2	TANC2	0.999782	0.018237	69.229	S	RFSPPPVGGQGKEYPSPPPSPLRRGPQYRAS	1209500	0	5847
Q9HCD6	1534	Protein TANC2	TANC2	0.999995	0.018237	69.229	S	PPVGGQGKEYPSPPPSPLRRGPQYRASPPAE	1209500	0	5848
Q8NGP4	154	Olfactory receptor 5M3	OR5M3	0.54151	0.0186646	69.152	S	CIRLITFPYIYGFLTSLAATLWTYGLYFCGK	960290	0	4409
Q8NGP4	153	Olfactory receptor 5M3	OR5M3	0.541411	0.0186646	69.152	T	VCIRLITFPYIYGFLTSLAATLWTYGLYFCG	960290	0	7897
Q8NGP4	147	Olfactory receptor 5M3	OR5M3	0.951997	0.0186646	69.152	Y	SKMSRVVCIRLITFPYIYGFLTSLAATLWTY	960290	0	8396
Q8NGP4	149	Olfactory receptor 5M3	OR5M3	0.948278	0.0186646	69.152	Y	MSRVVCIRLITFPYIYGFLTSLAATLWTYGL	960290	0	8397
Q5VUA4	1713	Zinc finger protein 318	ZNF318	0.719452	0.000143292	69.149	S	IWTSSSFQSDTSRDISPEKSELDLGEPGPPG	1494000	970570	3340
P19634	783	Sodium/hydrogen exchanger 1	SLC9A1	0.266747	0.00143773	69.122	S	ETSSPGTDDVFTPAPSDSPSSQRIQRCLSDP	0	0	1171
P19634	787	Sodium/hydrogen exchanger 1	SLC9A1	0.266747	0.00143773	69.122	S	PGTDDVFTPAPSDSPSSQRIQRCLSDPGPHP	0	0	1173
P19634	788	Sodium/hydrogen exchanger 1	SLC9A1	0.266747	0.00143773	69.122	S	GTDDVFTPAPSDSPSSQRIQRCLSDPGPHPE	0	0	1174
Q9UPT8	1118	Zinc finger CCCH domain-containing protein 4	ZC3H4	0.779784	0.00100759	69.071	T	PSPTASPSGDASPPATAPYDPRVLAAGGLGQ	0	0	8250
Q9ULJ3	345	Zinc finger and BTB domain-containing protein 21	ZBTB21	0.801888	0.00765042	69.069	S	DRSGPLVKSLLRRSLSMDSQVPVYSPSIDLK	0	0	6480
Q15464	312	SH2 domain-containing adapter protein B	SHB	0.473141	0.000583129	68.97	S	YDTPYEPEGQSVDSDSESTVSPRLRESKLPQ	0	0	2937
Q9P1Y5	341	Calmodulin-regulated spectrin-associated protein 3	CAMSAP3	0.959182	0.00249768	68.97	S	DLPDGHAASPRGTEASPPQNNSGSSSPVFTF	0	726110	6191
Q9P1Y5	347	Calmodulin-regulated spectrin-associated protein 3	CAMSAP3	0.671407	0.00249768	68.97	S	AASPRGTEASPPQNNSGSSSPVFTFRHPLLS	995700	1422300	6192
P50747	147	Biotin--protein ligase;Biotin--[methylmalonyl-CoA-carboxytra	HLCS	0.983362	0.000333121	68.948	S	PYDYSSSLESVADETSPEREGRRVNLTGKAP	1325900	684320	1760
Q9BQQ3	248	Golgi reassembly-stacking protein 1	GORASP1	0.496553	6.85E-05	68.948	S	PPGPTPEDSPSLETGSRQSDYMEALLQAPGS	0	0	5319
Q13111	780	Chromatin assembly factor 1 subunit A	CHAF1A	0.480082	0.00498117	68.916	Y	LLSNHTGSPRSPSTTYLHTPTPSEDAAIPSK	0	0	8375
Q9H8H2	737	Probable ATP-dependent RNA helicase DDX31	DDX31	1	0.0203187	68.915	S	YATYPRELKHIFHVRSLHLGHVAKSFGLRDA	3211100	0	5787
Q7Z589	238	Protein EMSY	EMSY	0.583295	0.0225365	68.846	S	AVVPVSKTITVPVSGSPKMSNIMQSIANSLP	714200	332550	3838
P16383	96	GC-rich sequence DNA-binding factor 2	GCFC2	0.905424	0.018545	68.845	S	APRADEGSESRTLDVSTDEEDKIHHSSESKD	0	0	1086
P16383	97	GC-rich sequence DNA-binding factor 2	GCFC2	0.905424	0.018545	68.845	T	PRADEGSESRTLDVSTDEEDKIHHSSESKDD	0	0	7289
O00161	6	Synaptosomal-associated protein 23	SNAP23	0.670781	0.0151744	68.786	S	__________MDNLSSEEIQQRAHQITDESL	223230	219220	96
Q9UQ35	892	Serine/arginine repetitive matrix protein 2	SRRM2	0.504388	0.0208621	68.786	S	PDPELKSRTPSRHSCSGSSPPRVKSSTPPRQ	0	0	6644
Q14676	793	Mediator of DNA damage checkpoint protein 1	MDC1	0.997125	0.00199813	68.771	S	CLSPPRAIPGDQHPESPVHTEPMGIQGRGRQ	10247000	0	2759
Q8N2M8	335	CLK4-associating serine/arginine rich protein	CLASRP	0.950478	8.36E-06	68.763	S	PGREEKITFITSFGGSDEEAAAAAAAAAASG	713700	0	4241
O95104	149	Splicing factor, arginine/serine-rich 15	SCAF4	0.823367	0.000121549	68.733	T	MAAGTSNAAPVAENVTNNEGSPPPPVKVSSE	2836300	0	7233
Q9HB09	121	Bcl-2-like protein 12	BCL2L12	0.986314	0.00166677	68.634	S	RRGEAAGSPVPTPPRSPAQEEPTDFLSRLRR	1953500	0	5830
O60934	343	Nibrin	NBN	0.940159	0.0217969	68.625	S	HPSTGLKTTTPGPSLSQGVSVDEKLMPSAPV	0	510770	543
Q99613;B	7;7	Eukaryotic translation initiation factor 3 subunit C;Eukaryo	EIF3C;EIF3	0.39762	0.00082743	68.589	T	_________MSRFFTTGSDSESESSLSGEEL	1288000	436120	7114
P21580	381	Tumor necrosis factor alpha-induced protein 3;A20p50;A20p37	TNFAIP3	0.995511	0.0230178	68.552	S	EGHAQNPMEPSVPQLSLMDVKCETPNCPFFM	0	0	1198
Q96CX2	171	BTB/POZ domain-containing protein KCTD12	KCTD12	0.981311	2.93E-05	68.516	S	LLPLGYSEPEQQEGASAGAPSPTLELASRSP	0	8015500	4830
Q96CX2	183	BTB/POZ domain-containing protein KCTD12	KCTD12	0.428039	2.93E-05	68.516	S	EGASAGAPSPTLELASRSPSGGAAGPLLTPS	0	0	4832
Q9H4Z3	152	Phosphorylated CTD-interacting factor 1	PCIF1	0.529334	0.00147766	68.516	T	TGQSVPSSPSIPGTPTLKMWGTSPEDKQQAA	0	0	8104
Q9NWV8	7	BRISC and BRCA1-A complex member 1	BABAM1	0.46156	0.0011022	68.49	S	_________MEVAEPSSPTEEEEEEEEHSAE	0	0	6070
Q9NWV8	8	BRISC and BRCA1-A complex member 1	BABAM1	0.659137	0.0011022	68.49	S	________MEVAEPSSPTEEEEEEEEHSAEP	2087500	0	6071
Q8NI27	1219	THO complex subunit 2	THOC2	0.254145	5.61E-05	68.489	S	PGGGPSSSSIGSASKSDESSTEETDKSRERS	0	0	4426
Q9UJX6	314	Anaphase-promoting complex subunit 2	ANAPC2	0.999993	0.0231344	68.463	S	WLGKVFLQDGPARPASPEAGNTLRRWRCHVQ	0	279450	6415
Q9UNF1	264	Melanoma-associated antigen D2	MAGED2	0.847058	0.0141265	68.463	S	KARRGKARRRAAKLQSSQEPEAPPPRDVALL	0	0	6546
Q5T200	1452	Zinc finger CCCH domain-containing protein 13	ZC3H13	0.995641	8.79E-05	68.412	S	ESLEGDDESKLDDAHSLGSGAGEGYEPISDD	1846700	981410	3257
Q7Z4V5	230	Hepatoma-derived growth factor-related protein 2	HDGFRP2	1	0.00553524	68.412	S	PRRGPLGGRKKKKAPSASDSDSKADSDGAKP	0	0	3826
Q07687	218	Homeobox protein DLX-2	DLX2	0.576643	2.98E-05	68.326	S	RRSKFKKMWKSGEIPSEQHPGASASPPCASP	0	1042500	2231
O15446	128	DNA-directed RNA polymerase I subunit RPA34	CD3EAP	0.703742	0.000130827	68.287	S	GTLRILEGPQQSLSGSPLQPIPASPPPQIPP	980030	0	320
Q01082	2323	Spectrin beta chain, non-erythrocytic 1	SPTBN1	0.717013	0.014485	68.262	S	SDKHEVSASTQSTPASSRAQTLPTSVVTITS	540140	542180	2047
Q14160	1442	Protein scribble homolog	SCRIB	0.309838	0.000488311	68.231	S	EQEDEQPPWASPSPTSRQSPASPPPLGGGAP	0	0	2680
Q14160	1441	Protein scribble homolog	SCRIB	0.309838	0.000488311	68.231	T	EEQEDEQPPWASPSPTSRQSPASPPPLGGGA	0	0	7584
Q96SN8	1887	CDK5 regulatory subunit-associated protein 2	CDK5RAP2	0.881529	0.0166019	68.171	S	GNLELRPGGAHPGTCSPSRPGS_________	814160	667290	5122
Q9UEG4	55	Zinc finger protein 629	ZNF629	0.81087	0.00442408	68.168	S	SGEEIIMGDPAQSPESKDSTEMSLERSSQDP	847390	355020	6286
Q7KZ85	1708	Transcription elongation factor SPT6	SUPT6H	0.281305	0.000467541	68.104	S	KQRLTPRPSPSPMIESTPMSIAGDATPLLDE	0	0	3707
Q7KZ85	1709	Transcription elongation factor SPT6	SUPT6H	0.281305	0.000467541	68.104	T	QRLTPRPSPSPMIESTPMSIAGDATPLLDEM	0	0	7789
Q7KZ85	1718	Transcription elongation factor SPT6	SUPT6H	0.998371	0.000467541	68.104	T	SPMIESTPMSIAGDATPLLDEMDR_______	0	626440	7790
P49736	59	DNA replication licensing factor MCM2	MCM2	0.99584	7.18E-05	68.101	T	RDLPPFEDESEGLLGTEGPLEEEEDGEELIG	0	0	7417
O43439	33	Protein CBFA2T2	CBFA2T2	1	0.0269571	67.997	S	QWKVGPEKRVPAMPGSPVEVKIQSRSSPPTM	602530	352830	389
Q8N8K9	291	Uncharacterized protein KIAA1958	KIAA1958	0.999897	0.025942	67.981	S	ISRPIVQKTARVSLASPNRGPPGTHGTNQQV	622730	0	4299
Q7Z4S6	862	Kinesin-like protein KIF21A	KIF21A	0.249063	0.00179763	67.979	T	KVTRKLSSSDAPAQDTGSSAAAVETDASRTG	0	0	7810
Q8WXI9	489	Transcriptional repressor p66-beta	GATAD2B	0.537493	0.00554285	67.979	T	EIEQRLQQQAALSPTTAPAVSSVSKQETIMR	0	0	7929
Q6P2E9	809	Enhancer of mRNA-decapping protein 4	EDC4	0.496414	0.000166253	67.951	S	GLDGGPGDGDRHNTPSLLEAALTQEASTPDS	0	0	3519
Q6P2E9	807	Enhancer of mRNA-decapping protein 4	EDC4	0.496044	0.000166253	67.951	T	QLGLDGGPGDGDRHNTPSLLEAALTQEASTP	0	0	7756
Q6P2E9	821	Enhancer of mRNA-decapping protein 4	EDC4	0.761757	0.000166253	67.951	T	NTPSLLEAALTQEASTPDSQVWPTAPDITRE	1106400	626120	7758
O75717	333	WD repeat and HMG-box DNA-binding protein 1	WDHD1	0.997112	0.000236407	67.911	S	RVEKDYNDLFDGDDMSNAGDFLNDNAVEIPS	4275500	0	659
Q9UQ35	988	Serine/arginine repetitive matrix protein 2	SRRM2	0.910354	0.0109276	67.902	S	SSPDTKVKPETPPRQSHSGSISPYPKVKAQT	2389500	3436300	6673
P55197	689	Protein AF-10	MLLT10	0.999506	0.0216407	67.879	S	LVGRGSSPRGSLSPRSPVSSLQIRYDQPGNS	4143200	2031400	1874
Q13427	753	Peptidyl-prolyl cis-trans isomerase G	PPIG	0.999777	0.0294578	67.846	S	KKFDHESSPGTDEDKSG______________	0	0	2495
Q9UK61	1107	Protein FAM208A	FAM208A	0.829734	0.0298582	67.846	S	ASAKGGNLPPVSPNDSGAKIASNPLERHVIP	0	0	6425
Q9C0C2	498	182 kDa tankyrase-1-binding protein	TNKS1BP1	0.529561	0.000536118	67.832	S	RPSGLGVWRLDSPPPSPITEASEAAEAAEAG	0	0	5522
Q13480	437	GRB2-associated-binding protein 1	GAB1	0.460613	0.000695974	67.829	S	GFHNHFKVKNVLTVGSVSSEELDENYVPMNP	0	0	2546
Q8WX93	757	Palladin	PALLD	0.214925	5.06E-05	67.809	S	NLGPASGHGTPASSPSSSSLPSPMSPTPRQF	0	0	4577
Q8WX93	758	Palladin	PALLD	0.221744	5.06E-05	67.809	S	LGPASGHGTPASSPSSSSLPSPMSPTPRQFG	0	0	4578
Q8WX93	759	Palladin	PALLD	0.345439	5.06E-05	67.809	S	GPASGHGTPASSPSSSSLPSPMSPTPRQFGR	0	0	4579
Q8WX93	760	Palladin	PALLD	0.261551	5.06E-05	67.809	S	PASGHGTPASSPSSSSLPSPMSPTPRQFGRA	0	0	4580
Q8WX93	763	Palladin	PALLD	0.422754	5.06E-05	67.809	S	GHGTPASSPSSSSLPSPMSPTPRQFGRAPVP	0	0	4581
Q99700	736	Ataxin-2	ATXN2	0.53954	1.34E-05	67.752	S	AMPIPAASPTPASPASNRAVTPSSEAKDSRL	0	0	5260
Q9UJM3	251	ERBB receptor feedback inhibitor 1	ERRFI1	0.980572	0.0242502	67.749	S	NPPPPQTHRRLRRSHSGPAGSFNKPAIRISN	586240	0	6403
P11388	1525	DNA topoisomerase 2-alpha	TOP2A	0.837956	0.0242872	67.726	S	AKSVRAKKPIKYLEESDEDDLF_________	2237500	3154100	1025
Q13304	296	Uracil nucleotide/cysteinyl leukotriene receptor	GPR17	0.791194	0.0223816	67.726	S	VNRSVYVLHYRSHGASCATQRILALANRITS	0	0	2469
Q9ULF5	573	Zinc transporter ZIP10	SLC39A10	0.685111	0.000836757	67.675	S	WLQLKPLAGTDDSVVSEDRLNETELTDLEGQ	1183000	0	6468
P78356	322	Phosphatidylinositol 5-phosphate 4-kinase type-2 beta	PIP4K2B	0.746936	0.000106143	67.658	T	CENDGVGGNLLCSYGTPPDSPGNLLSFPRFF	2489500	0	7461
Q14247	399	Src substrate cortactin	CTTN	0.625687	0.019402	67.648	T	QEEARRKLEEQARAKTQTPPVSPAPQPTEER	26885000	0	7594
Q15021	1331	Condensin complex subunit 1	NCAPD2	0.482372	0.00841777	67.645	T	AKKPSTGSRYQPLASTASDNDFVTPEPRRTT	0	0	7630
Q8N137	790	Centrobin	CNTROB	0.933525	0.00289158	67.579	S	EPPSSHSQGSGPSSGSPERGGDGLTFPRQLM	783450	335510	4212
Q9ULJ7	1163	Ankyrin repeat domain-containing protein 50	ANKRD50	0.752553	0.0017545	67.579	S	PSLRGLPNGPTHAFSSPSESPDSTVDRQKSS	1679800	548710	6484
Q9ULJ7	1165	Ankyrin repeat domain-containing protein 50	ANKRD50	0.566072	0.0017545	67.579	S	LRGLPNGPTHAFSSPSESPDSTVDRQKSSLS	1679800	548710	6485
P46821	1322	Microtubule-associated protein 1B;MAP1B heavy chain;MAP1 lig	MAP1B	0.962365	0.000641944	67.571	S	EEHCASPEDKTLEVVSPSQSVTGSAGHTPYY	1892500	2068300	1603
P46821	1334	Microtubule-associated protein 1B;MAP1B heavy chain;MAP1 lig	MAP1B	0.744439	0.000750501	67.571	T	EVVSPSQSVTGSAGHTPYYQSPTDEKSSHLP	0	1558000	7393
O14545	479	TRAF-type zinc finger domain-containing protein 1	TRAFD1	0.873782	0.0277487	67.519	S	QLSRSTSGPRPGCQPSSPCVPKLSNSDSQDI	0	0	153
Q68EM7	560	Rho GTPase-activating protein 17	ARHGAP17	0.38399	0.000138093	67.496	S	QSSRAESSSGGGTVPSSAGILEQGPSPGDGS	0	0	3425
Q68EM7	561	Rho GTPase-activating protein 17	ARHGAP17	0.383987	0.000138093	67.496	S	SSRAESSSGGGTVPSSAGILEQGPSPGDGSP	0	0	3426
Q7Z2Z1	1057	Treslin	TICRR	0.99865	0.0221333	67.494	S	RVHSFQQDKSDQRENSPVQSIRSPKSLLFGA	0	490380	3756
Q16204	367	Coiled-coil domain-containing protein 6	CCDC6	0.995391	0.00238769	67.463	S	SPIPYTPSPSSSRPISPGLSYASHTVGFTPP	1108500	716560	3004
Q6L8Q7	224	2',5'-phosphodiesterase 12	PDE12	0.315549	0.000133194	67.463	S	EVGVPSSLSPSSPSSSWTETDVEERVYTPSN	0	0	3491
Q16204	380	Coiled-coil domain-containing protein 6	CCDC6	0.741029	0.00238769	67.463	T	PISPGLSYASHTVGFTPPTSLTRAGMSYYNS	1108500	0	7655
Q6P2E9	816	Enhancer of mRNA-decapping protein 4	EDC4	0.436599	0.000174207	67.459	T	DGDRHNTPSLLEAALTQEASTPDSQVWPTAP	0	0	7757
Q96JH7	747	Deubiquitinating protein VCIP135	VCPIP1	0.712319	0.0267211	67.334	S	TEKLKQEQKGQPRTVSPSTIRDGPSSAPATP	430740	308230	4963
Q9UIG0	349	Tyrosine-protein kinase BAZ1B	BAZ1B	0.999188	0.0267211	67.334	S	PKLWCHVHLKKSLSGSPLKVKNSKNSKSPEE	667530	0	6385
Q9UMN6	1092	Histone-lysine N-methyltransferase 2B	KMT2B	0.689516	0.0023974	67.285	S	RRCVKQRPSYDIFEDSDDSEPGGPPAPRRRT	1584100	1287300	6517
Q9UMN6	1095	Histone-lysine N-methyltransferase 2B	KMT2B	0.898	0.0023974	67.285	S	VKQRPSYDIFEDSDDSEPGGPPAPRRRTPRE	1584100	1287300	6518
Q9UMN6	1086	Histone-lysine N-methyltransferase 2B	KMT2B	0.723168	0.00287207	67.285	Y	LQRKSARRCVKQRPSYDIFEDSDDSEPGGPP	1584100	0	8433
P35219	10	Carbonic anhydrase-related protein	CA8	0.660051	0.0121229	67.275	T	______MADLSFIEDTVAFPEKEEDEEEEEE	0	0	7355
Q9UPT8	908	Zinc finger CCCH domain-containing protein 4	ZC3H4	0.95595	0.00285885	67.251	S	ARALPTSKPEGSLHSSPVGPSSSKGSGPPPT	0	0	6571
O15534	27	Period circadian protein homolog 1	PER1	0.999956	0.00550181	67.214	S	GDPRPGESFCPGGVPSPGPPQHRPCPGPSLA	1560200	0	325
Q15910	362	Histone-lysine N-methyltransferase EZH2	EZH2	0.468886	0.0123048	67.153	S	PKRPGGRRRGRLPNNSSRPSTPTINVLESKD	0	0	2990
Q15910	363	Histone-lysine N-methyltransferase EZH2	EZH2	0.96771	0.0123048	67.153	S	KRPGGRRRGRLPNNSSRPSTPTINVLESKDT	0	0	2991
Q15910	366	Histone-lysine N-methyltransferase EZH2	EZH2	0.620743	0.0123048	67.153	S	GGRRRGRLPNNSSRPSTPTINVLESKDTDSD	3250700	0	2992
Q8IYB7	875	DIS3-like exonuclease 2	DIS3L2	0.999507	0.00153028	67.11	S	PGTQGHLGPEKEEEESDGEPEDSSTS_____	2036300	1373000	4173
Q9Y2U5	240	Mitogen-activated protein kinase kinase kinase 2	MAP3K2	0.712965	0.00603011	67.11	Y	DGESYPKSRMPRAQSYPDNHQEFSDYDNPIF	0	0	8435
A8CG34;Q	275;33;2	Nuclear envelope pore membrane protein POM 121C;POM121 and	POM121C;POM	0.679821	0.00114465	67.097	S	SRSAIPEQIISSTLSSPSSNAPDPCAKETVL	400410	481400	58
O95402	447	Mediator of RNA polymerase II transcription subunit 26	MED26	0.999993	0.0284509	67.095	S	RQIKPLTQKEPVRADSPVHMEQQSRTELDKQ	0	0	762
Q86UE4	298	Protein LYRIC	MTDH	0.998735	0.0252722	67.095	S	GGGWNEKSVKLSSQISAGEEKWNSVSPASAG	0	0	3923
P19484	109	Transcription factor EB	TFEB	1	0.00359281	67.033	S	EYLSETYGNKFAAHISPAQGSPKPPPAASPG	1575400	0	1163
P19484	114	Transcription factor EB	TFEB	1	0.00359281	67.033	S	TYGNKFAAHISPAQGSPKPPPAASPGVRAGH	1575400	0	1164
P19484	122	Transcription factor EB	TFEB	1	0.00359281	67.033	S	HISPAQGSPKPPPAASPGVRAGHVLSSSAGN	1575400	0	1165
Q9P206	379	Uncharacterized protein KIAA1522	KIAA1522	0.853356	0.000895121	66.98	S	DTIVSDGSTLSSKGGSEGQPESSTASNSVVP	0	1071000	6210
Q9H672	256	Ankyrin repeat and SOCS box protein 7	ASB7	0.564821	0.0227341	66.965	S	NTRNYEGQTPLAVSISISGSSRPCLDFLQEV	877100	0	5722
Q9H672	260	Ankyrin repeat and SOCS box protein 7	ASB7	0.708264	0.0227341	66.965	S	YEGQTPLAVSISISGSSRPCLDFLQEVTRQP	877100	0	5723
Q9H672	261	Ankyrin repeat and SOCS box protein 7	ASB7	0.669209	0.0227341	66.965	S	EGQTPLAVSISISGSSRPCLDFLQEVTRQPR	877100	0	5724
Q9H672	245	Ankyrin repeat and SOCS box protein 7	ASB7	0.927934	0.0227341	66.965	Y	ARMLYNYGADTNTRNYEGQTPLAVSISISGS	877100	0	8423
Q9Y484	50	WD repeat domain phosphoinositide-interacting protein 4	WDR45	1	0.0112645	66.939	S	EPLMEKGHLDHEQVGSMGLVEMLHRSNLLAL	0	1644500	6897
P50747	124	Biotin--protein ligase;Biotin--[methylmalonyl-CoA-carboxytra	HLCS	0.998816	0.000538313	66.925	S	CLELENSTIESVKFASAENIPDLPYDYSSSL	3317700	0	1759
P50747	146	Biotin--protein ligase;Biotin--[methylmalonyl-CoA-carboxytra	HLCS	0.626897	0.000579337	66.925	T	LPYDYSSSLESVADETSPEREGRRVNLTGKA	1534800	0	7434
Q14978	538	Nucleolar and coiled-body phosphoprotein 1	NOLC1	1	0.0282045	66.92	S	DDSSEEEEEKLKGKGSPRPQAPKANGTSALT	685210	0	2825
P27348	230	14-3-3 protein theta	YWHAQ	0.33294	0.021619	66.906	S	TLIMQLLRDNLTLWTSDSAGEECDAAEGAEN	0	0	1278
P46937	163	Transcriptional coactivator YAP1	YAP1	0.583822	0.0138084	66.893	S	VSGPAATPTAQHLRQSSFEIPDDVPLPAGWE	1023800	376220	1614
P35612	689	Beta-adducin	ADD2	0.750303	0.00600351	66.889	S	DTDVDTSKDKTESVTSGPMSPEGSPSKSPSK	0	1166200	1403
Q96E09	262	Protein FAM122A	FAM122A	0.364861	0.00101595	66.884	S	NSSSAGSSCNSPAKVSTTTDSPVSPAQAASP	0	0	4876
Q96E09	263	Protein FAM122A	FAM122A	0.364861	0.00101595	66.884	T	SSSAGSSCNSPAKVSTTTDSPVSPAQAASPF	0	0	7978
Q53EZ4	425	Centrosomal protein of 55 kDa	CEP55	0.639465	0.00507075	66.874	S	VTFQGETENREKVAASPKSPTAALNESLVEC	1405500	904810	3135
Q53EZ4	430	Centrosomal protein of 55 kDa	CEP55	0.719227	0.0113234	66.874	T	ETENREKVAASPKSPTAALNESLVECPKCNI	1405500	904810	7696
Q9UER7	178	Death domain-associated protein 6	DAXX	0.832508	2.43E-05	66.828	S	HLSLDPTNAENTASQSPRTRGSRRQIQRLEQ	4617700	0	6290

Q96PU5	475	E3 ubiquitin-protein ligase NEDD4-like	NEDD4L	0.925599	0.0048952	66.777	S	GAKDSPVRRAVKDTLSNPQSPQPSPYNSPKP	951390	419320	5056
Q8IWI9	2871	MAX gene-associated protein	MGA	0.998699	0.000404584	66.772	S	DSISPLLLHLEDDDFSENEKQLAEPASEPDV	1309100	585830	4056
Q8WUB8	297	PHD finger protein 10	PHF10	0.999963	4.51E-05	66.677	S	ALYEPPLDPELPALDSDGDSDDGEDGRGDEK	7472700	3615700	4513
O95714	3462	E3 ubiquitin-protein ligase HERC2	HERC2	1	0.0231935	66.663	S	NGEECMLAVDIEDRLSPNPWQEKREIVSSED	878010	341500	804
Q9H7N4	552	Splicing factor, arginine/serine-rich 19	SCAF1	0.850643	0.00175002	66.644	S	SGTQPAPPAPASPWDSKKHRSRDRKPGSHAS	1774200	0	5765
Q7Z589	236	Protein EMSY	EMSY	0.499959	0.0260127	66.621	S	PKAVVPVSKTITVPVSGSPKMSNIMQSIANS	714200	0	3837
O43379	1268	WD repeat-containing protein 62	WDR62	0.644806	0.000113511	66.58	T	LASLGQELQAITTATTPSLDSEGQEPALRSW	0	1392700	7169
O75376	2102	Nuclear receptor corepressor 1	NCOR1	0.98056	0.016724	66.568	S	ALVSTPVRTKTSNRYSPESQAQSVHHQRPGS	1212000	0	609
O75427	267	Leucine-rich repeat and calponin homology domain-containing	LRCH4	0.999997	0.0261087	66.56	S	LHIFKYLSTEAGQRGSALGDLAPSRPPSFSP	0	184110	625
O75376	174	Nuclear receptor corepressor 1	NCOR1	0.577235	0.00176356	66.536	S	PISGQPCGDDQNASPSKLSKEELIQSMDRVD	1607200	0	598
O75152;A(	762;763	Zinc finger CCCH domain-containing protein 11A	ZC3H11A	0.529824	0.0114891	66.501	T	SSQMSMKTRRLSSASTGKPPLSVEDDFEKLI	0	1370800	7197
Q8WW38	990	Zinc finger protein ZFPM2	ZFPM2	0.59698	0.00654245	66.448	S	AKGADQLSPYYGIKPSDYISGSLVIHNTDIE	1042600	915360	4550
Q8WW38	1007	Zinc finger protein ZFPM2	ZFPM2	0.999442	0.00654245	66.448	S	YISGSLVIHNTDIEQSRNAENESPKGQASSN	1042600	915360	4551
P18858	91	DNA ligase 1	LIG1	0.78096	3.14E-05	66.426	S	SPAKGQKPALDCSQVSPPRPATSPENNASLS	1507500	2546500	1148
P18858	104	DNA ligase 1	LIG1	0.578419	3.14E-05	66.426	S	QVSPPRPATSPENNASLSDTSPMDSSPSGIP	0	0	1150
Q6PGQ7	325	Protein aurora borealis	BORA	0.999946	0.0263701	66.393	S	GITNPCIRSPYIDGCSPIKNWSPMRLQMYSG	373320	561710	3539
Q8IVF2	3821	Protein AHNAK2	AHNAK2	0.915009	0.0246181	66.393	S	PKFKMPSFGVSAPGKSIEASVHVSAPKVEAD	2758400	3025600	4042
Q8IVF2	3829	Protein AHNAK2	AHNAK2	0.996916	0.0246181	66.393	S	GVSAPGKSIEASVHVSAPKVEADVSLPSMQG	2758400	3025600	4043
Q4VC05	110	B-cell CLL/lymphoma 7 protein family member A	BCL7A	0.249943	0.00120786	66.383	S	NQSSIADASPIKQENSSNSSPAPEPNSAVPS	0	0	3107
Q4VC05	111	B-cell CLL/lymphoma 7 protein family member A	BCL7A	0.249943	0.00120786	66.383	S	QSSIADASPIKQENSSNSSPAPEPNSAVPSD	0	0	3108
Q9UN36	350	Protein NDRG2	NDRG2	0.462491	0.00234254	66.36	S	SAASVDGNRSRSRTLSQSSESGTLSSGPPGH	0	0	6529
Q8N5I9	15	Uncharacterized protein C12orf45	C12orf45	0.712303	0.0243876	66.326	S	_MEVHGKPKASPSCSSPTRDSSGVPVSKELL	0	0	4286
Q96SB3	191	Neurabin-2	PPP1R9B	0.5	0.0243876	66.326	T	LQDRKLDVVVRFNGSTEALDKLDADAVSPTV	0	629990	8007
Q14676	1775	Mediator of DNA damage checkpoint protein 1	MDC1	0.804799	0.000574056	66.3	S	AVRAAESLTAIPEPASPQLLETPIHASQIQK	0	4008300	2758
P78312	393	Protein FAM193A	FAM193A	0.981006	0.000943712	66.294	S	VLGSRSPRTEESKADSPPPSYPTQQAEQAPN	0	0	1950
Q7Z2W4	335	Zinc finger CCCH-type antiviral protein 1	ZC3HAV1	0.89914	0.000147635	66.294	S	GTSQAGTSQRFLENGSQEDLLHGNPGSTYLA	0	8311900	3751
P27694	140	Replication protein A 70 kDa DNA-binding subunit;Replicatior	RPA1	0.672898	0.000271732	66.282	S	PQVAPPAPAASPAASSRPQPQNGSSGMGSTV	0	3013800	1282
Q8IVV2	1214	Lipoxygenase homology domain-containing protein 1	LOXHD1	0.588776	0.0236954	66.27	S	GAETLTFPCDRWLATSEDDKKTIRELVPYDI	0	525510	4049
Q96GY0	223	Zinc finger C2HC domain-containing protein 1A	ZC2HC1A	0.832696	0.0236999	66.267	S	VSSSSSSLGNKLQTLSPSHKGIAAPHAGANV	0	0	4929
Q99700	784	Ataxin-2	ATXN2	0.999979	0.0244731	66.267	S	NETSPSFSKAENKGISPVVSEHRKQIDDLKK	2037400	1409700	5255
Q96B01	14	RAD51-associated protein 1	RAD51AP1	0.502954	0.000893409	66.137	S	__MVRPVRHKKPVNYSQFDHSDSDDDFVSAT	1171100	0	4788
Q96B01	21	RAD51-associated protein 1	RAD51AP1	0.971612	0.000893409	66.137	S	RHKKPVNYSQFDHSDSDDDFVSATVPLNKKS	1171100	753040	4790
Q6WCQ1	289	Myosin phosphatase Rho-interacting protein	MPRIP	0.994188	0.00342553	66.12	S	KQDLKAEEQQLPPPLSPPSPSTPNHRRSQVI	0	1365500	3623
Q6WCQ1	292	Myosin phosphatase Rho-interacting protein	MPRIP	0.719884	0.00342553	66.12	S	LKAEEQQLPPPLSPPSPSTPNHRRSQVIEKF	0	1365500	3624
O95835	1111	Serine/threonine-protein kinase LATS1	LATS1	0.971531	0.000584043	66.075	S	PIEYEYINSQGSEQQSDEDDQNTGSEIKNRD	1272300	0	835
Q16513	535	Serine/threonine-protein kinase N2	PKN2	0.674193	0.000746309	66.075	S	LVRRAIPTVNHSGTFSPQAPVPTTVPVVDVR	890180	1026900	3009
P55196	1783	Afadin	MLLT4	0.428634	0.00587584	66.068	S	KEKRSKSQDADSPGSSGAPENLTFKERQRLF	0	0	1871
Q9BTK6	148	PAXIP1-associated glutamate-rich protein 1	PAGR1	0.951444	0.0254446	66.068	S	PRRPPTPEAQSEEERSDEEPEAKEEEEEKPH	1008100	742950	5366
Q9Y3T9	30	Nucleolar complex protein 2 homolog	NOC2L	0.735746	0.000184997	66.044	S	TVDEFLASGFDSESESESESENSPQAETREARE	1799100	1608400	6879
Q9Y3T9	15	Nucleolar complex protein 2 homolog	NOC2L	0.980411	0.000218545	66.044	T	_MAAAGSRKRRLAELTVDEFLASGFDSESES	1799100	0	8294
Q9Y450	67	HBS1-like protein	HBS1L	0.786051	0.00901888	66.022	S	PVEEYDYEDLKESSNSVSNHQLSGFDQARLY	5445900	0	6890
Q9Y450	69	HBS1-like protein	HBS1L	0.454093	0.00901888	66.022	S	EEYDYEDLKESSNSVSNHQLSGFDQARLYSC	0	0	6891
Q9BZF1	13	Oxysterol-binding protein-related protein 8	OSBPL8	0.49057	0.00621277	65.949	T	___MEGGLADGEPDRTSLLGDSKDVLGPSTV	0	0	8069
Q5JSZ5	556	Protein PRRC2B	PRRC2B	0.814448	0.0250823	65.842	S	KAGEARKQAEKEVPWSPSAEKASPQENGPAV	1225700	1049500	3190
Q92615	599	La-related protein 4B	LARP4B	1	0.025422	65.809	S	PSVPASCAVSATYERSPSPAHLPDDPKVAEK	955370	0	4676
O95684	152	FGFR1 oncogene partner	FGFR1OP	0.907381	0.00258818	65.784	S	QKEKGPTTGEGALDLSDVHSPPKSPEGKTSA	0	2059000	794
Q5SW79;Q(	1515;224	Centrosomal protein of 170 kDa;Cep170-like protein	CEP170;CEP:	0.932762	0.000113169	65.766	S	LEALNNMGFPSAMLPSPPKQKSSPVNNHHSP	618640	0	3219
Q8N556	264	Actin filament-associated protein 1	AFAP1	0.499759	0.00155549	65.765	S	SGCSGPVDSECPPPPSSPVHKAELEKKLSSE	0	0	4270
Q8N556	265	Actin filament-associated protein 1	AFAP1	0.499759	0.00155549	65.765	S	GCSGPVDSECPPPPSSPVHKAELEKKLSSER	0	0	4271
P46821	1330	Microtubule-associated protein 1B;MAP1B heavy chain;MAP1 lig	MAP1B	0.466236	0.000641944	65.753	S	DKTLEVVSPSQSVTGSAGHTPYYQSPTDEKS	0	0	1605
P46821	1336	Microtubule-associated protein 1B;MAP1B heavy chain;MAP1 lig	MAP1B	0.321008	0.000641944	65.753	Y	VSPSQSVTGSAGHTPYYQSPTDEKSSHLPTE	0	0	8364
P46821	1337	Microtubule-associated protein 1B;MAP1B heavy chain;MAP1 lig	MAP1B	0.322364	0.000641944	65.753	Y	SPSQSVTGSAGHTPYYQSPTDEKSSHLPTEV	0	0	8365
P55211	307	Caspase-9;Caspase-9 subunit p35;Caspase-9 subunit p10	CASP9	0.639206	0.000128805	65.744	S	KDHGFEVASTSPEDESPGSNPEPDATPFQEG	1807500	1639700	1883
Q969V6	781	MKL/myocardin-like protein 1	MKL1	0.599326	0.000150922	65.73	T	KEPPSLPGKEKPSPKTVCGSPLAAQPSPSAE	0	0	7963
Q9HCD6	1517	Protein TANC2	TANC2	1	0.0274606	65.695	S	PPHTSPAHQGGSYRFSPPPVGGQGKEYPSPP	871830	537270	5849
P78563	26	Double-stranded RNA-specific editase 1	ADARB1	1	0.0287767	65.627	S	SSSTDVKENRNLDNVSPKDGSTPGPGEGSQL	0	0	1984
Q14207	779	Protein NPAT	NPAT	0.472029	3.12E-05	65.624	S	NGENLPTIILSSPTKSPTKNAELVKCLSSEE	0	0	2727
Q14207	777	Protein NPAT	NPAT	0.371219	3.12E-05	65.624	T	SINGENLPTIILSSPTKSPTKNAELVKCLSS	0	0	7592
Q14207	781	Protein NPAT	NPAT	0.680418	5.58E-05	65.624	T	ENLPTIILSSPTKSPTKNAELVKCLSSEETV	0	1100600	7593
Q9UHB7	1062	AF4/FMR2 family member 4	AFF4	0.802284	0.000605964	65.581	S	GSKAVGMPSPVSPKLSPGNSGNYSSGASSAS	1711200	0	6340
Q06265	392	Exosome complex component RRP45	EXOSC9	0.999745	3.90E-05	65.577	S	VEVSDIGSQDAPIILSDSEEEEMIILEPDKN	1231900	586700	2200
Q06265	394	Exosome complex component RRP45	EXOSC9	0.999815	3.90E-05	65.577	S	VSDIGSQDAPIILSDSEEEEMIILEPDKNPK	1231900	586700	2201
O60832	497	H/ACA ribonucleoprotein complex subunit 4	DKC1	0.570674	0.0102092	65.511	T	GLESGAEPGDGDSDTTKKKKKKKKAKEVELV	420860	479960	7190
P13725	126	Oncostatin-M	OSM	1	0.0255715	65.5	S	ADLEQRLPKAQDLERSGLNIEDLEKLQMARP	0	0	1049
Q9UJU3	423	Zinc finger protein 112	ZNF112	0.999996	0.0279039	65.495	S	IHTEEKLYTDIEYGKSFICSSNLDIQHRVHM	0	9595500	6404
Q9UJU3	427	Zinc finger protein 112	ZNF112	0.989325	0.0279039	65.495	S	EKLYTDIEYGKSFICSSNLDIQHRVHMEENS	0	9595500	6405
P16150	355	Leukosialin	SPN	0.999998	0.0314416	65.467	S	PTLTTFFGRRKSRQGSLAMEELKSGSGPSLK	0	126790	1084
O15047	540	Histone-lysine N-methyltransferase SETD1A	SETD1A	0.893629	3.63E-05	65.465	T	DTGSEVPSGSGHGPCTPPPAPANFEDVAPTG	877190	1858000	7146
Q96E52	139	Metalloendopeptidase OMA1, mitochondrial	OMA1	1	0.0270539	65.425	S	PLSPASIRAIRNFHTSPRFQAAPVPLLLMIL	0	0	4881
Q12789	1840	General transcription factor 3C polypeptide 1	GTF3C1	0.756543	0.00205315	65.374	S	ADIQREDPQARPLEGSSSEDSPPEGQAPPSH	848800	925370	2299
Q13153	223	Serine/threonine-protein kinase PAK 1	PAK1	0.553616	0.00254223	65.374	S	PLPVTPTRDVATSPISPTENNTTPPDALTRN	1123800	904310	2425
Q9P246	522	Stromal interaction molecule 2	STIM2	0.564247	0.0280039	65.347	S	ARSSSLCRSRRSIVPSSPQPQRAQLAPHAPH	0	0	6212
Q99504	256	Eyes absent homolog 3	EYA3	0.527146	0.00126209	65.33	S	KPSVMAPAPAAQRLSSGDPSTSPSLSQTTPS	738000	681930	5197
Q99504	266	Eyes absent homolog 3	EYA3	0.770534	0.00126209	65.33	S	AQRLSSGDPSTSPSLSQTTPSKDTDDQSRKN	738000	681930	5198
Q14244	365	Ensconsin	MAP7	1	0.0215306	65.295	S	PPGSVKAAPAQVRPPSPGNIRPVKREVKVEP	1196500	893370	2728
P35659	231	Protein DEK	DEK	0.827097	0.0119296	65.287	S	KAKRTKCPEILSDESSSDEDEKKNKEESSDD	854160	322460	1419
P35659	232	Protein DEK	DEK	0.966039	0.0119296	65.287	S	AKRTKCPEILSDESSSDEDEKKNKEESSDDE	854160	322460	1420
Q674R7	364	Autophagy-related protein 9B	ATG9B	0.953083	0.0229329	65.241	Y	PRPLTELDIHHRILRYTNYQVALANKGLLPA	980210	950480	8385
Q674R7	367	Autophagy-related protein 9B	ATG9B	0.963703	0.0229329	65.241	Y	LTELDIHHRILRYTNYQVALANKGLLPARCP	980210	950480	8386
Q9H410	77	Kinetochore-associated protein DSN1 homolog	DSN1	0.499884	0.0150626	65.225	S	GGNCDLSHQERLQSKSLHLSPQEQSASYQDR	0	0	5653
Q9NYF8	228	Bcl-2-associated transcription factor 1	BCLAF1	0.990462	0.000153914	65.223	S	WPGLSAYDNSPRSPHSPSPIATPPSQSSSCS	0	958200	6109
O95155	86	Ubiquitin conjugation factor E4 B	UBE4B	0.693485	0.000855502	65.197	S	GVAHRSQSSEGVSSLSSSPSNSLETQSQSLS	1646000	0	736
Q9ULU4	754	Protein kinase C-binding protein 1	ZMYND8	0.685574	0.00611123	65.197	S	KQDVVGKTPPSTTVGSHSPPETPVLTRSSAQ	0	1420300	6510
Q9Y2L9	532	Leucine-rich repeat and calponin homology domain-containing	LRCH1	0.99822	0.00420641	65.197	S	QSNGSQYSPNEIRENSPAVSPTTNSTAPFGL	4378300	0	6796
Q9Y2L9	536	Leucine-rich repeat and calponin homology domain-containing	LRCH1	0.836281	0.00420641	65.197	S	SQYSPNEIRENSPAVSPTTNSTAPFGLKPRS	4378300	0	6797
Q9UQ35	2323	Serine/arginine repetitive matrix protein 2	SRRM2	0.356902	0.00160772	65.189	Y	ALSLTGSGTPPTAANYPSSSRTPQAPASANL	0	0	8434
Q9HCE1	814	Putative helicase MOV-10	MOV10	0.5	0.0293418	65.157	S	EAATVTSYLKLLLAPSSKKGKARLSPRSVGV	0	153710	5853
Q9HCE1	815	Putative helicase MOV-10	MOV10	0.5	0.0293418	65.157	S	AATVTSYLKLLLAPSSKKGKARLSPRSVGVI	0	153710	5854
Q14C86	946	GTPase-activating protein and VPS9 domain-containing protein	GAPVD1	0.693601	0.00110466	65.111	S	PAAAIGATSLVAAPHSSSSSPSKDSSRGETE	0	2757100	2833
Q14C86	947	GTPase-activating protein and VPS9 domain-containing protein	GAPVD1	0.433581	0.00110466	65.111	S	AAAIGATSLVAAPHSSSSSPSKDSSRGETEE	0	0	2834
P48169;Q	303;287	Gamma-aminobutyric acid receptor subunit alpha-4;Gamma-aminc	GABRA4;GABI	0.477958	0.00448581	65.097	S	RTVFGITTVLTMTTLSISARHSLPKVSYATA	0	0	1619
P48169;Q	305;289	Gamma-aminobutyric acid receptor subunit alpha-4;Gamma-aminc	GABRA4;GABI	0.770166	0.00448581	65.097	S	VFGITTVLTMTTLSISARHSLPKVSYATAMD	0	0	1620
P48169;Q	294;278	Gamma-aminobutyric acid receptor subunit alpha-4;Gamma-aminc	GABRA4;GABI	0.657209	0.00448581	65.097	T	WINKESVPARTVFGITTVLTMTTLSISARHS	0	0	7398
P48169;Q	295;279	Gamma-aminobutyric acid receptor subunit alpha-4;Gamma-aminc	GABRA4;GABI	0.663602	0.00448581	65.097	T	INKESVPARTVFGITTVLTMTTLSISARHSL	0	0	7399
P48169;Q	300;284	Gamma-aminobutyric acid receptor subunit alpha-4;Gamma-aminc	GABRA4;GABI	0.964193	0.00448581	65.097	T	VPARTVFGITTVLTMTTLSISARHSLPKVSY	1824500	1321100	7400
P48169;Q	301;285	Gamma-aminobutyric acid receptor subunit alpha-4;Gamma-aminc	GABRA4;GABI	0.898217	0.00448581	65.097	T	PARTVFGITTVLTMTTLSISARHSLPKVSYA	0	1321100	7401
Q13523	568	Serine/threonine-protein kinase PRP4 homolog	PRPF4B	0.472953	0.0027827	65.085	S	KYLAEDSNMSVPSEPSSPQSSTRTRSPSPDD	0	0	2566
Q5JRA6	1678	Melanoma inhibitory activity protein 3	MIA3	0.956436	4.70E-05	65.035	S	QNGSFGPSPVSGGECSPPLTVEPPVRPLSAT	0	1357000	3174
Q5JRA6	1691	Melanoma inhibitory activity protein 3	MIA3	0.87496	4.70E-05	65.035	S	ECSPPLTVEPPVRPLSATLNRRDMPRSEFGS	1391000	603260	3175
P51784	952	Ubiquitin carboxyl-terminal hydrolase 11	USP11	0.728965	0.000681335	64.954	S	LSPAGSSGAPASPACSSPPSSEFMDVN____	0	3301300	1796
P51784	953	Ubiquitin carboxyl-terminal hydrolase 11	USP11	0.600534	0.000681335	64.954	S	SPAGSSGAPASPACSSPPSSEFMDVN_____	4481000	0	1797
P51784	957	Ubiquitin carboxyl-terminal hydrolase 11	USP11	0.510602	0.000681335	64.954	S	SSGAPASPACSSPPSSEFMDVN_________	0	0	1798
P46821	23	Microtubule-associated protein 1B;MAP1B heavy chain;MAP1 lig	MAP1B	0.623508	0.000264046	64.845	S	ATEPEPSGSIANPAASTSPSLSHRFLDSKFY	0	0	1574
Q13523	518	Serine/threonine-protein kinase PRP4 homolog	PRPF4B	0.333333	0.000643632	64.733	S	KFKGSLSEGMKVEQESSSDDNLEDFDVEEED	0	0	2562
Q13523	519	Serine/threonine-protein kinase PRP4 homolog	PRPF4B	0.333333	0.000643632	64.733	S	FKGSLSEGMKVEQESSSDDNLEDFDVEEEDE	0	0	2563
Q13523	520	Serine/threonine-protein kinase PRP4 homolog	PRPF4B	0.333333	0.000643632	64.733	S	KGSLSEGMKVEQESSSDDNLEDFDVEEEDEE	0	0	2564
Q15555	9	Microtubule-associated protein RP/EB family member 2	MAPRE2	0.958678	0.000471551	64.733	S	_______MPGPTQTLSPNGENNNDIIQDNNG	0	1845900	2945
Q641Q2	5	WASH complex subunit FAM21A	FAM21A	0.499997	0.00233733	64.733	T	___________MMNRTTPDQELAPASEPVWE	564330	0	7739
Q641Q2	6	WASH complex subunit FAM21A	FAM21A	0.499997	0.00233733	64.733	T	__________MMNRTTPDQELAPASEPVWER	564330	0	7740
Q96SN8	1889	CDK5 regulatory subunit-associated protein 2	CDK5RAP2	0.348394	0.00588678	64.723	S	LELRPGGAHPGTCSPSRPGS___________	0	0	5123
Q9UQ35	1227	Serine/arginine repetitive matrix protein 2	SRRM2	0.996418	0.00347215	64.707	S	ERSGAGSSPETKEQNSALPTSSQDEELMEVV	546650	517960	6624
O43379	1048	WD repeat-containing protein 62	WDR62	0.730741	2.67E-05	64.654	S	TEDELSLPEGPSVPSSSLPQTPEQEKFLRHH	0	0	366
O43379	1053	WD repeat-containing protein 62	WDR62	0.999536	2.67E-05	64.654	T	SLPEGPSVPSSSLPQTPEQEKFLRHHFETLT	10743000	7352500	7167
Q8IY81	534	pre-rRNA processing protein FTSJ3	FTSJ3	0.998986	0.00106247	64.619	S	AVLQEEQANLWFSKGSFAGIEDDADEALEIS	299860	157340	4124
Q03164	2866	Histone-lysine N-methyltransferase 2A;MLL cleavage product N	KMT2A	0.825978	0.000140689	64.592	S	DFVLKNTPSMQALGESPESSSSELLNLGEGL	2851000	0	2142
Q9H0D6	485	5'-3' exoribonuclease 2	XRN2	0.693505	0.000623773	64.545	S	SSPSISPNTSFTSDGSPSPLGGIKRKAEDSD	0	917750	5574
Q8N5U6	746	RING finger protein 10	RNF10	0.869264	0.00313203	64.538	S	PKKDENSLVPPAPVDSDGESDNSDRVPVPSF	0	82928	4288
Q3T8J9	2107	GON-4-like protein	GON4L	0.8022	0.0308267	64.52	S	CPVHESPSGIDTSETSPKAPRGGLAKDSGTQ	0	0	3076
Q9H4A3	2027	Serine/threonine-protein kinase WNK1	WNK1	0.981715	0.0103479	64.52	S	SDPEAAFLSRDVDDGSGSPHSPHQLSSKSLP	2564100	5963600	5662
Q9H444	188	Charged multivesicular body protein 4b	CHMP4B	0.499998	0.00169187	64.488	T	QEELDKNLLEISGPETVPLPNVPSIALPSKP	0	782750	8100
Q8IXM2	35	Chromatin complexes subunit BAP18	BAP18	0.499838	0.00439752	64.454	S	TKLGELTMQLHPVADSSPAGAKWTETEIEML	0	0	4105
Q8IXM2	36	Chromatin complexes subunit BAP18	BAP18	0.499838	0.00439752	64.454	S	KLGELTMQLHPVADSSPAGAKWTETEIEMLR	0	0	4106
P29966	128	Myristoylated alanine-rich C-kinase substrate	MARCKS	0.523385	0.00015356	64.383	S	AAEPGSPTAAEGEAASAASSTSSPKAEDGAT	0	0	1329
Q9C086	97	INO80 complex subunit B	INO80B	0.999996	0.00183088	64.383	S	TKSVPTFTVIPEGPRSPSPLMVVDNEEEPME	1414300	760750	5504
Q9C086	99	INO80 complex subunit B	INO80B	0.999998	0.00183088	64.383	S	SVPTFTVIPEGPRSPSPLMVVDNEEEPMEGV	1414300	760750	5505
A6NJI9	2	Leucine-rich repeat-containing protein 72	LRRC72	1	0.0278038	64.374	S	______________MSWDPNPVPRTLRCWRL	156120	126250	38
Q9BQI0	133	Allograft inflammatory factor 1-like	AIF1L	0.5	0.0224792	64.374	S	AVLKLVMMFEGKANESSPKPVGPPPERDIAS	0	261650	5314
Q9BQI0	134	Allograft inflammatory factor 1-like	AIF1L	0.5	0.0224792	64.374	S	VLKLVMMFEGKANESSPKPVGPPPERDIASL	0	261650	5315
Q9Y2D5	630	A-kinase anchor protein 2	AKAP2	0.702487	2.10E-05	64.347	S	FGSEKPQSMFEPPQVSSPVQEKRDVLPKILP	597320	645510	6776
Q03164	518	Histone-lysine N-methyltransferase 2A;MLL cleavage product N	KMT2A	0.501049	0.00204311	64.318	S	RSDTPEVHPPLPISQSPENESNDRRSRRYSV	0	1811000	2145
Q9Y3T9	22	Nucleolar complex protein 2 homolog	NOC2L	0.41011	0.000184997	64.315	S	RKRRLAELTVDEFLASGFDSESESESESENSPQ	0	0	6876
Q9Y3T9	26	Nucleolar complex protein 2 homolog	NOC2L	0.621979	0.000184997	64.315	S	LAELTVDEFLASGFDSESESESESENSPQAETR	0	0	6877
Q9Y3T9	32	Nucleolar complex protein 2 homolog	NOC2L	0.5271	0.000184997	64.315	S	DEFLASGFDSESESESESENSPQAETREAREAA	0	0	6880
Q16576	95	Histone-binding protein RBBP7	RBBP7	0.994842	0.00646117	64.275	S	VARVHIPNDDAQFDASHCDSDKGEFGGFGSV	0	0	3016
Q9Y2S2	111	Lambda-crystallin homolog	CRYL1	0.99886	0.00222609	64.273	S	PEDLELKKKIFAQLDSIIDDRVILSSSTSCL	0	687800	6798
Q9Y2S2	122	Lambda-crystallin homolog	CRYL1	0.776982	0.00222609	64.273	S	AQLDSIIDDRVILSSSTSCLMPSKLFAGLVH	0	687800	6799
Q9Y2S2	124	Lambda-crystallin homolog	CRYL1	0.95548	0.00222609	64.273	S	LDSIIDDRVILSSSTSCLMPSKLFAGLVHVK	0	687800	6800
Q9Y2S2	129	Lambda-crystallin homolog	CRYL1	0.999258	0.00222609	64.273	S	DDRVILSSSTSCLMPSKLFAGLVHVKQCIVA	0	687800	6801
Q9Y2S2	123	Lambda-crystallin homolog	CRYL1	0.776982	0.00222609	64.273	T	QLDSIIDDRVILSSSTSCLMPSKLFAGLVHV	0	687800	8286

P16383	419	GC-rich sequence DNA-binding factor 2	GCFC2	0.685062	0.000963036	64.27	S	EIESRRTKRRQARVLSGNCNHQEGTSSDDEL	0	20071000	1087
P16383	429	GC-rich sequence DNA-binding factor 2	GCFC2	0.490272	0.000963036	64.27	S	QARVLSGNCNHQEGTSSDDELPSAEMIDFQK	0	0	1088
Q9Y618	156	Nuclear receptor corepressor 2	NCOR2	0.95087	0.00394264	64.27	T	LTGKLEPVSPPSPPHTDPELELVPPRLSKEE	0	3789300	8314
Q5T200	1366	Zinc finger CCCH domain-containing protein 13	ZC3H13	0.962866	0.0304167	64.265	S	KRRDLDRERERLISDSVERDRDRDRTFES	625820	249410	3260
Q9Y5X9	471	Endothelial lipase	LIPG	0.709304	0.0230387	64.26	S	TQRKLTFCTEDPENTSISPGRELWFRKCRDG	0	0	6970
Q9Y5X9	464	Endothelial lipase	LIPG	0.632307	0.0230387	64.26	T	IRVKSGETQRKLTFCTEDPENTSISPGRELW	0	0	8310
Q9Y5X9	470	Endothelial lipase	LIPG	0.709306	0.0230387	64.26	T	ETQRKLTFCTEDPENTSISPGRELWFRKCRD	0	0	8311
Q9BVC5	182	Ashwin	C2orf49	0.996526	0.00554463	64.256	S	NNNDAKQNHDLTHRKSPSGPVKSPPLSPVGT	4888000	3689000	5396
Q99638	270	Cell cycle checkpoint control protein RAD9A	RAD9A	0.465414	0.00072409	64.058	S	LLDGHFVLATLSDTDSHSQDLGSPERHQPVP	0	0	5236
Q99638	272	Cell cycle checkpoint control protein RAD9A	RAD9A	0.51759	0.00072409	64.058	S	DGHFVLATLSDTDSHSQDLGSPERHQPVPQL	0	764240	5237
Q99638	277	Cell cycle checkpoint control protein RAD9A	RAD9A	0.998575	0.00072409	64.058	S	LATLSDTDSHSQDLGSPERHQPVPQLQAHST	532220	764240	5238
O00512	278	B-cell CLL/lymphoma 9 protein	BCL9	1	0.0220399	64.04	S	PAPKPAAPPRPLDRESPGVENKLIPSVGSPA	1377900	0	127
Q9UHV7	330	Mediator of RNA polymerase II transcription subunit 13	MED13	0.530621	0.000882389	64.028	S	TRDPAMSSVTLTPPTSPEEVQTVTDPQSVQKW	0	3022900	6367
Q9UHV7	326	Mediator of RNA polymerase II transcription subunit 13	MED13	0.918639	0.000882389	64.028	T	VPASTRDPAMSSVTLTPPTSPEEVQTVTDPQS	0	3022900	8216
Q9UHV7	329	Mediator of RNA polymerase II transcription subunit 13	MED13	0.530721	0.000882389	64.028	T	STRDPAMSSVTLTPPTSPEEVQTVTDPQSVQK	0	3022900	8217
Q8IXJ6	366	NAD-dependent protein deacetylase sirtuin-2	SIRT2	0.36669	0.00328357	63.936	S	SIDAQSGAGVPNPSTSASPKKSPPPAKDEAR	0	0	4099
Q8IXJ6	368	NAD-dependent protein deacetylase sirtuin-2	SIRT2	0.36669	0.00328357	63.936	S	DAQSGAGVPNPSTSASPKKSPPPAKDEARTT	0	0	4100
Q8IXJ6	365	NAD-dependent protein deacetylase sirtuin-2	SIRT2	0.292548	0.00328357	63.936	T	ASIDAQSGAGVPNPSTSASPKKSPPPAKDEA	0	0	7851
Q14676	1664	Mediator of DNA damage checkpoint protein 1	MDC1	0.930235	0.000231178	63.932	T	TPKPVEPAASDLEPFTPTDQSVTPEAIAQGG	0	0	7604
Q14676	1671	Mediator of DNA damage checkpoint protein 1	MDC1	0.692484	0.000231178	63.932	T	AASDLEPFTPTDQSVTPEAIAQGGQSKTLRS	0	0	7605
O14879	478	Interferon-induced protein with tetratricopeptide repeats 3	IFIT3	0.799601	0.000119125	63.925	S	LEDGSEEMGQGAVSSSPRELLSNSEQLN___	0	360570	213
Q8WWM7	423	Ataxin-2-like protein	ATXN2L	0.394456	0.00267179	63.906	S	MSPKAQRPLRGAKTLSSPSNRPSGETSVPPP	0	0	4568
Q8WWM7	424	Ataxin-2-like protein	ATXN2L	0.627616	0.00267179	63.906	S	SPKAQRPLRGAKTLSSPSNRPSGETSVPPPP	0	6035600	4569
Q76L83	530	Putative Polycomb group protein ASXL2	ASXL2	0.99989	0.0046703	63.869	S	SESQESLVTSPSKPKSPGVEKPIVKPTAGAG	1437300	869710	3704
O60271	329	C-Jun-amino-terminal kinase-interacting protein 4	SPAG9	0.880919	0.000897495	63.803	S	SKHIEVQVAQETRNVSTGSAENEEKSEVQAI	0	1416400	458
O60271	332	C-Jun-amino-terminal kinase-interacting protein 4	SPAG9	0.76411	0.000897495	63.803	S	IEVQVAQETRNVSTGSAENEEKSEVQAIIES	0	1416400	459
O60271	339	C-Jun-amino-terminal kinase-interacting protein 4	SPAG9	0.918455	0.000897495	63.803	S	ETRNVSTGSAENEEKSEVQAIIESTPELDMD	0	1416400	460
O60271	330	C-Jun-amino-terminal kinase-interacting protein 4	SPAG9	0.764108	0.000897495	63.803	T	KHIEVQVAQETRNVSTGSAENEEKSEVQAIIE	0	1416400	7179
Q7Z2K6	5	Endoplasmic reticulum metallopeptidase 1	ERMP1	1	0.0279115	63.76	S	___________MEWGSESAAVRRHRVGVERR	0	1428000	3746
Q7Z2K6	7	Endoplasmic reticulum metallopeptidase 1	ERMP1	1	0.0279115	63.76	S	_________MEWGSESAAVRRHRVGVERREG	0	1428000	3747
O43379	1049	WD repeat-containing protein 62	WDR62	0.740008	2.85E-05	63.74	S	EDELSLPEGPSVPSSSLPQTPEQEKFLRHHF	0	0	367
Q13136	793	Liprin-alpha-1	PPFIA1	0.387069	0.00144305	63.726	S	DGPVSNPSSSNSSQDSLHKAPKKKGIKSSIG	0	0	2418
Q14C86	949	GTPase-activating protein and VPS9 domain-containing protei	GAPVD1	0.383088	0.00121015	63.726	S	AIGATSLVAAPHSSSSSPSKDSSRGETEERK	0	0	2836
Q14C86	950	GTPase-activating protein and VPS9 domain-containing protei	GAPVD1	0.383088	0.00121015	63.726	S	IGATSLVAAPHSSSSSPSKDSSRGETEERKD	0	0	2837
Q9Y2U8	185	Inner nuclear membrane protein Man1	LEMD3	0.499081	0.00664025	63.726	S	LKAPPAPLAASEVTNSNSAERRKPHSWWGAR	0	0	6806
Q96G74	175	OTU domain-containing protein 5	OTUD5	0.926455	0.000159924	63.706	Y	AVGGGSPEREEVGAGYNSEDEYEAAAARIEA	0	0	8408
Q5SYE7	1181	NHS-like protein 1	NHSL1	0.750855	0.000859758	63.701	S	RSPGAPSAGEAEARPSPSTTPLPDSSPSRKP	924930	255210	3235
Q5SYE7	1183	NHS-like protein 1	NHSL1	0.43731	0.000859758	63.701	S	PGAPSAGEAEARPSPSTTPLPDSSPSRKPPP	0	0	3236
Q5SYE7	1184	NHS-like protein 1	NHSL1	0.43731	0.000859758	63.701	T	GAPSAGEAEARPSPSTTPLPDSSPSRKPPPI	0	0	7717
Q9BXK1	228	Krueppel-like factor 16	KLF16	0.536862	0.000507765	63.63	S	HPDLLRRPGARSTSPSDSLPCSLAGSPAPSP	2278800	1219000	5455
Q9UGV2	358	Protein NDRG3	NDRG3	0.416263	0.00282544	63.606	S	FSRSVTSNQSDGTQESCESPDVLDRHQTMEV	0	0	6321
Q13330	449	Metastasis-associated protein MTA1	MTA1	0.989182	0.0307295	63.602	S	TRLDGERPGPNRSNMSPHGLPARSSGSPKFA	0	525530	2475
Q9NYF8	648	Bcl-2-associated transcription factor 1	BCLAF1	1	0.0197878	63.565	S	TSYQKATEEHSTRQKSPEIHRRIDISPSTLR	0	2859700	6107
Q92797	494	Symplekin	SYMPK	0.726409	0.000792562	63.563	S	EKVVKTESVLIKRRLSAQGQAISVVGSLSSM	1117200	0	4714
Q4KMP7	132	TBC1 domain family member 10B	TBC1D10B	1	0.0283907	63.534	S	VAGVETSRALAAGADSPKTEEARPSPAPGPG	989730	0	3097
Q86YS7	278	C2 domain-containing protein 5	C2CD5	0.884555	0.00371864	63.488	T	KEMKEIPFNEDPNPNTHSSGPSTPLKNQTYS	0	0	7837
Q86YS7	285	C2 domain-containing protein 5	C2CD5	0.931489	0.00371864	63.488	T	FNEDPNPNTHSSGPSTPLKNQTYSFSPSKSY	5810700	4331400	7838
Q14160	834	Protein scribble homolog	SCRIB	0.504711	0.00340686	63.488	Y	PENAVTITPLRPEDDYSPRERRGGGLRLPLL	0	0	8380
Q8IYB3	743	Serine/arginine repetitive matrix protein 1	SRRM1	0.771311	0.0284781	63.473	S	PEPKKIKKAASPSPQSVRRVSSSRSVSGSPE	0	1288100	4132
Q9Y5S9	42	RNA-binding protein 8A	RBM8A	0.97873	0.0272669	63.473	S	KLKEKAKKRKGRGFGSEEGSRARMREDYDSV	0	343020	6966
O95644	359	Nuclear factor of activated T-cells, cytoplasmic 1	NFATC1	0.998025	0.0306856	63.462	S	PSVALKVEPVGEDLGSPPPPADFAPEDYSSF	555630	499900	788
Q6PGQ7	270	Protein aurora borealis	BORA	0.619238	0.000132525	63.427	S	QASAKKYSLGSITSPSPISSPTFSPIEFQIG	823760	372450	3540
Q6PGQ7	274	Protein aurora borealis	BORA	0.623896	0.000132525	63.427	S	KKYSLGSITSPSPISSPTFSPIEFQIGETPL	823760	372450	3541
Q9UJF2	31	Ras GTPase-activating protein nGAP	RASAL2	0.731406	0.00804388	63.427	T	SISGTSTSEKPNSMDTANTSPFKVPGFFSKR	0	0	8226
P30414	468	NK-tumor recognition protein;Putative peptidyl-prolyl cis-t	NKTR	0.590844	0.0310331	63.408	S	QTKKRRILIPSDIESSKSSTRRMKSSCDRER	889660	1054900	1343
Q5TDH0	115	Protein DDI1 homolog 2	DDI2	0.657673	0.000810621	63.372	T	AVPGTSSPRQRQPPGTQQSHSSPGEITSSPQ	0	3255400	7726
O60271	732	C-Jun-amino-terminal kinase-interacting protein 4	SPAG9	0.87394	0.0230259	63.337	S	AGLDTEGSKQRSASQSSLDKLDQELKEQQKE	4706900	0	462
Q08211	87	ATP-dependent RNA helicase A	DHX9	0.89677	1.04E-05	63.304	S	INEIKSEEVPAFGVASPPPLTDTPDTTANAE	4548800	2677000	2247
Q9P253	3	Vacuolar protein sorting-associated protein 18 homolog	VPS18	0.642068	0.0284076	63.292	S	_______________MASILDEYENSLSRSAVL	0	0	6214
Q96GY3	202	Protein lin-37 homolog	LIN37	0.816953	0.000361794	63.291	S	EMQGTPDDEPSEPEPSPSTLIYRNMQRWKRI	0	0	4934
Q14004	1146	Cyclin-dependent kinase 13	CDK13	0.46728	0.000903528	63.272	S	LPAGILATGEKQTDPSTPQQESSKPLGGIQP	0	0	2639
Q9H5H4	17	Zinc finger protein 768	ZNF768	0.890502	0.00144817	63.225	S	EREALPWGLEPQDVQSSDEMRSPEGYLRGNM	843540	978150	5712
Q9H5H4	18	Zinc finger protein 768	ZNF768	0.660902	0.00144817	63.225	S	REALPWGLEPQDVQSSDEMRSPEGYLRGNMS	843540	978150	5713
Q9H5H4	23	Zinc finger protein 768	ZNF768	0.648422	0.00144817	63.225	S	WGLEPQDVQSSDEMRSPEGYLRGNMSENEEE	843540	978150	5714
Q99956	169	Dual specificity protein phosphatase 9	DUSP9	0.938528	7.31E-05	63.203	S	PGPVPVVGLGSLCLGSDCSDAESEADRDSMS	1315900	638000	5281
Q8IXT5	710	RNA-binding protein 12B	RBM12B	1	0.020549	63.185	S	PEDDFRRPPEEDFRHSPEEDFRQSPQEHFRR	1659000	773920	4113
Q8IXT5	718	RNA-binding protein 12B	RBM12B	1	0.020549	63.185	S	PEEDFRHSPEEDFRQSPQEHFRRPPQEHFRR	1659000	773920	4114
Q96H79	263	Zinc finger CCCH-type antiviral protein 1-like	ZC3HAV1L	0.75018	0.00471969	63.145	S	KMLENTDNSSPSTEHSQGLEKQGVHAAGAAE	8009800	0	4937
P21675	526	Transcription initiation factor TFIID subunit 1	TAF1	0.596437	0.000123257	63.128	S	LEIPDEKEEATSNSPSKESKKESSLKKSRIL	2060200	1292000	1200
Q9BRY0	120	Zinc transporter ZIP3	SLC39A3	0.689372	0.000581765	63.116	T	ILTFRKEKPSFIDLETFNAGSDVGSDSEYES	0	3744400	8044
Q96QR8	100	Transcriptional activator protein Pur-beta	PURB	0.499464	0.000228181	63.085	S	DSLGDFIEHYAQLGPSSPEQLAAGAEEGGGP	0	0	5082
O14782	173	Kinesin-like protein KIF3C	KIF3C	0.97687	0.0226405	63.076	T	SKEPGKRLELKENPETGVYIKDLSSFVTKNV	2026200	0	7132
Q9C0C2	429	182 kDa tankyrase-1-binding protein	TNKS1BP1	0.999674	0.0257432	63.062	S	GDAHLRPTSLVQRRFSEGVLQSPSQDQEKLG	0	349630	5517
Q96L73	2334	Histone-lysine N-methyltransferase, H3 lysine-36 and H4 lysi	NSD1	0.687257	0.00591725	63.042	S	PPAVAGPRPQLSDKPSPVTSPSSSPSVRSQP	1462200	0	5011
Q96L73	2342	Histone-lysine N-methyltransferase, H3 lysine-36 and H4 lysi	NSD1	0.335594	0.00591725	63.042	S	PQLSDKPSPVTSPSSSPSVRSQPLERPLGTA	0	0	5012
Q96L73	2344	Histone-lysine N-methyltransferase, H3 lysine-36 and H4 lysi	NSD1	0.335626	0.00591725	63.042	S	LSDKPSPVTSPSSSPSVRSQPLERPLGTADP	0	0	5013
Q14202	957	Zinc finger MYM-type protein 3	ZMYM3	0.95575	0.000773203	63.029	S	AEELDKASSDLCDLVSNQSAEGLLEDCDLFG	2449000	2719600	2702
P50570	762	Dynamin-2	DNM2	0.307371	4.34E-05	63.008	S	PVPPPVDDTWLQSASSHSPTPQRRPVSSIHP	0	0	1756
P50570	764	Dynamin-2	DNM2	0.413166	4.34E-05	63.008	S	PPPVDDTWLQSASSHSPTPQRRPVSSIHPPG	0	0	1757
P50570	755	Dynamin-2	DNM2	0.269099	4.34E-05	63.008	T	STSTVSTPVPPPVDDTWLQSASSHSPTPQRR	0	0	7431
P18669;Q8N0Y7 118;118		Phosphoglycerate mutase 1;Probable phosphoglycerate mutase 4	PGAM1;PGAM4	0.963123	0.00381732	62.999	S	TAAKHGEAQVKIWRRSYDVPPPPMEPDHPFY	7044000	4891300	1144
P55198	384	Protein AF-17	MLLT6	0.632657	0.00373413	62.966	S	YSKPTAPASAPPSPSAPEPPKADLFEQKVV	0	753570	1876
Q9H7L9	244	Sin3 histone deacetylase corepressor complex component SDS3	SUDS3	0.974711	0.00353505	62.966	T	PKRPASPSSPEHLPATPAESPAQRFEARIED	0	1164100	8112
Q8TF01	393	Arginine/serine-rich protein PNISR	PNISR	0.82768	0.000197875	62.848	S	ALASLTGLGGLGGYGSGDSEDERSDRGSESS	3565700	1462700	4495
Q8TF01	401	Arginine/serine-rich protein PNISR	PNISR	0.984709	0.000197875	62.848	S	GGLGGYGSGDSEDERSDRGSESSDTDDEELR	3565700	1462700	4496
Q13428	932	Treacle protein	TCOF1	0.295267	4.20E-05	62.833	S	GKTGPSAAQAGKQDDSGSSSEESDSDGEAPA	0	0	2512
Q13428	934	Treacle protein	TCOF1	0.295267	4.20E-05	62.833	S	TGPSAAQAGKQDDSGSSSEESDSDGEAPAAV	0	0	2513
Q13428	935	Treacle protein	TCOF1	0.447159	4.20E-05	62.833	S	GPSAAQAGKQDDSGSSSEESDSDGEAPAAVT	0	0	2514
Q13428	936	Treacle protein	TCOF1	0.295267	4.20E-05	62.833	S	PSAAQAGKQDDSGSSSEESDSDGEAPAAVTS	0	0	2515
Q8WXH0	6361	Nesprin-2	SYNE2	0.977206	0.00194884	62.765	S	RLTSCTPGLEDEKEASENETDMEDPREIQTD	3013900	1210300	4595
Q9Y320	288	Thioredoxin-related transmembrane protein 2	TMX2	0.854683	0.00237899	62.735	S	NIPEEQPVASTPTTVSDGENKKDK_______	0	0	6841
O95714	1944	E3 ubiquitin-protein ligase HERC2	HERC2	0.431694	0.000733146	62.717	T	AELPAAAQPSAEDSDTEDDSEAEQTERNIHP	0	0	7243
Q92551	377	Inositol hexakisphosphate kinase 1	IP6K1	0.681349	0.000138922	62.713	S	DMVLPEVASSCGPSTSPSNTSPEAGPSSQPK	510020	881340	4633
Q92551	379	Inositol hexakisphosphate kinase 1	IP6K1	0.466965	0.000138922	62.713	S	VLPEVASSCGPSTSPSNTSPEAGPSSQPKVD	0	0	4634
Q8N7P1	193	Inactive phospholipase D5	PLD5	0.864153	0.0296824	62.633	S	SQNIEIKLVSDVTADSKVLEALKLKGAEVTY	854580	0	4296
Q02446	770	Transcription factor Sp4	SP4	0.508825	0.00154369	62.589	S	LGSPRIVTVAAISQDSNPATPNVSTNMEEF_	0	0	2094
Q9BZF1	814	Oxysterol-binding protein-related protein 8	OSBPL8	0.844413	0.001925	62.589	S	SPEPDIQDSSGSEAQSVKPSTRRKKGIELGD	4018800	9456400	5490
Q9Y6R0	270	Numb-like protein	NUMBL	0.641368	0.000252986	62.582	S	GPAQPGHVSPTPATTSPGEKGEAGTPVAAGT	1016000	577170	7021
Q9Y6R0	268	Numb-like protein	NUMBL	0.641368	0.000252986	62.582	T	APGPAQPGHVSPTPATTSPGEKGEAGTPVAA	1016000	577170	8323
Q9Y6R0	269	Numb-like protein	NUMBL	0.641368	0.000252986	62.582	T	PGPAQPGHVSPTPATTSPGEKGEAGTPVAAG	1016000	577170	8324
Q15633	142	RISC-loading complex subunit TARBP2	TARBP2	0.999851	0.000378909	62.557	S	AAAAATPVPSVVLTRSPPMELQPPVSPQQSE	5634800	6265800	2951
P13994	367	Coiled-coil domain-containing protein 130	CCDC130	0.711468	0.00498258	62.556	S	QEGSRQDKPLSPAGSSQEAADTPDTRHPCSL	771500	702890	1060
Q14676	372	Mediator of DNA damage checkpoint protein 1	MDC1	0.97641	0.000128005	62.547	S	GTRGPGAPGLAHLQESQAGSDTDVEEGKAPQ	3646900	0	2767
Q86XP3	754	ATP-dependent RNA helicase DDX42	DDX42	0.928534	1.87E-05	62.51	S	SVPTNSAQQGHNSPDSPVTSAAKGIPGFGNT	2525900	907460	4013
Q14207	774	Protein NPAT	NPAT	0.398239	3.12E-05	62.455	S	AVSSINGENLPTIILSSPTKSPTKNAELVKC	0	0	2725
Q14207	775	Protein NPAT	NPAT	0.398239	3.12E-05	62.455	S	VSSINGENLPTIILSSPTKSPTKNAELVKCL	0	0	2726
P11388	1297	DNA topoisomerase 2-alpha	TOP2A	0.502542	0.0300053	62.408	S	FKPIKKGKKRNPWSDSESDRSSDESNFDVPP	623860	312670	1021
A1L390	576	Pleckstrin homology domain-containing family G member 3	PLEKHG3	0.999334	7.71E-05	62.383	S	VDFVAAESTEDLKALSSEEEEEMGGAAQEPE	5631700	4729300	13
A1L390	577	Pleckstrin homology domain-containing family G member 3	PLEKHG3	0.999334	7.71E-05	62.383	S	DFVAAESTEDLKALSSEEEEEMGGAAQEPES	5631700	4729300	14
P40222	18	Alpha-taxilin	TXLNA	0.499957	5.73E-05	62.283	S	NQDKKNGAAKQSNPKSSPGQPEAGPEGAQER	1143500	399990	1470
P40222	19	Alpha-taxilin	TXLNA	0.499957	5.73E-05	62.283	S	QDKKNGAAKQSNPKSSPGQPEAGPEGAQERP	1143500	399990	1471
Q9H0D6	479	5'-3' exoribonuclease 2	XRN2	0.587984	0.000810174	62.254	S	MRMQNNSSPSISPNTSFTSDGSPSPLGGIKR	0	0	5573
Q99700	888	Ataxin-2	ATXN2	0.475977	0.0166811	62.203	S	EHKRGPEVTSQGVQTSSPACKQEKDDKEEKK	0	0	5256
Q3KQU3	471	MAP7 domain-containing protein 1	MAP7D1	0.333332	0.0303028	62.2	S	ASELSPKSKARPSSPSTSWHRPASPCPSPGP	0	0	3066
Q3KQU3	473	MAP7 domain-containing protein 1	MAP7D1	0.333332	0.0303028	62.2	S	ELSPKSKARPSSPSTSWHRPASPCPSPGPGH	0	0	3067
Q69YH5	437	Cell division cycle-associated protein 2	CDCA2	0.76168	0.000965153	62.193	S	CKKDFSGLSSLLLEQSPVPEPLPQPDFDDKG	448110	375010	3431
Q96KQ7	171	Histone-lysine N-methyltransferase EHMT2	EHMT2	0.439496	0.000871449	62.193	T	GAQGAAAAGSEPPPATTSPEGQPKVHRARKT	0	0	7991
Q96KQ7	172	Histone-lysine N-methyltransferase EHMT2	EHMT2	0.439496	0.000871449	62.193	T	AQGAAAAGSEPPPATTSPEGQPKVHRARKTM	0	0	7992
Q9Y3T9	28	Nucleolar complex protein 2 homolog	NOC2L	0.677292	0.000214826	62.069	S	ELTVDEFLASGFDSESESESENSPQAETREA	0	1608400	6878
Q9BW85	313	Coiled-coil domain-containing protein 94	CCDC94	0.507338	0.00691395	62.032	Y	TPLTPGASSLSQLGAYLDSDDSNGSN_____	0	0	8420
Q4ADV7	995	RAB6A-GEF complex partner protein 1	RIC1	0.528883	0.000812518	62.028	S	RFLKAIGSGESETPPSTPTAQEPSSSGGFEF	1120300	542080	3090
Q4ADV7	996	RAB6A-GEF complex partner protein 1	RIC1	0.528883	0.000812518	62.028	T	FLKAIGSGESETPPSTPTAQEPSSSGGFEFF	1120300	542080	7676
Q9BVS4	442	Serine/threonine-protein kinase RIO2	RIOK2	0.973665	0.000975728	62.005	S	NRQDGQRVQGGVPAGSDEYEDECPHLIALSS	0	0	5407
Q15047	508	Histone-lysine N-methyltransferase SETDB1	SETDB1	0.706748	0.00210628	61.985	S	SFRPGSVGSGHSSPTSPALSENVSGGKPGIN	1213500	925150	2867
Q8IXK0	616	Polyhomeotic-like protein 2	PHC2	0.281348	0.00339264	61.963	T	YAQGFLPEKLPQQDHTTTTDSEMEEPYLQES	0	0	7852
Q8IXK0	617	Polyhomeotic-like protein 2	PHC2	0.281349	0.00339264	61.963	T	AQGFLPEKLPQQDHTTTTDSEMEEPYLQESK	0	0	7853
Q13309	64	S-phase kinase-associated protein 2	SKP2	0.994512	0.00196221	61.959	S	ENIPQELLSNLGHPESPPRKRLKSKGSDKDF	0	5615400	2471
Q96RS0	154	Trimethylguanosine synthase	TGS1	0.745879	0.00234034	61.959	S	QESWRKEYEEDDILASDDPSSIEQYENTRTY	921800	753160	5098
P38398	1212	Breast cancer type 1 susceptibility protein	BRCA1	0.681386	0.00172599	61.89	S	HLAQGYRRGAKKLESSEENLSSEDEELPCFQ	0	2801600	1451
Q14207	1203	Protein NPAT	NPAT	0.627172	0.00835217	61.862	S	IGQQNGGLRSEKSIASLQEMTKKQGTSSNNK	0	4158800	2723
Q14207	1215	Protein NPAT	NPAT	0.993182	0.00835217	61.862	S	SIASLQEMTKKQGTSSNNKNVLSVGTAVKDL	0	4158800	2724
Q14207	1208	Protein NPAT	NPAT	0.996168	0.00835217	61.862	T	GGLRSEKSIASLQEMTKKQGTSSNNKNVLSV	0	4158800	7590
Q14207	1213	Protein NPAT	NPAT	0.752973	0.00835217	61.862	T	EKSIASLQEMTKKQGTSSNNKNVLSVGTAVK	0	4158800	7591
Q9BYG4	295	Partitioning defective 6 homolog gamma	PARD6G	0.996399	1.86E-05	61.84	S	PAPRVLQNFHPDEAESDEDNDVVIEGTLEPA	1262700	1397700	5468
Q8NFP9	1714	Neurobeachin	NBEA	0.995848	0.00531147	61.811	S	AMSELLSTLSSEVKKSQESLTENPSETLKPA	1095300	0	4401
Q8NFP9	1717	Neurobeachin	NBEA	0.883184	0.00531147	61.811	S	ELLSTLSSEVKKSQESLTENPSETLKPATSI	1095300	0	4402
Q9H8M2	566	Bromodomain-containing protein 9	BRD9	0.5	0.0153408	61.78	S	SSLSNASERDQHHLGSPSRLSVGEQPDVTHD	0	0	5789
Q9H8M2	568	Bromodomain-containing protein 9	BRD9	0.5	0.0153408	61.78	S	LSNASERDQHHLGSPSRLSVGEQPDVTHDPY	0	0	5790
O14686	1151	Histone-lysine N-methyltransferase 2D	KMT2D	0.999757	0.00198955	61.703	S	EPGQTPGSLASELKGSPVLLDPEELAPVTPM	834910	586150	190

P98174	116	FYVE, RhoGEF and PH domain-containing protein 1	FGD1	0.872593	0.00730079	61.703	S	RPGLHQGNRILVKSLSLDPGQSLEPHPEGPQ	1197000	0	2013
O60293	655	Zinc finger C3H1 domain-containing protein	ZFC3H1	0.998123	0.0012674	61.685	S	RAFKPEETSSNSDPPSPPVLNNSHPVPRSNL	2200200	1702500	468
O43815	381	Striatin	STRN	0.319507	0.00401066	61.612	S	ELPSLQPSVGSPSRPSSSRLPEHEINRADEV	0	0	421
O43815	382	Striatin	STRN	0.319507	0.00401066	61.612	S	LPSLQPSVGSPSRPSSSRLPEHEINRADEVE	0	0	422
O43815	383	Striatin	STRN	0.319507	0.00401066	61.612	S	PSLQPSVGSPSRPSSSRLPEHEINRADEVEA	0	0	423
O75676	678	Ribosomal protein S6 kinase alpha-4	RPS6KA4	0.992853	0.0013033	61.602	S	LKLEGLRGSSWLQDGSARSSPPLRTPDVLES	2093700	1702000	651
O75676	681	Ribosomal protein S6 kinase alpha-4	RPS6KA4	0.996337	0.0013033	61.602	S	EGLRGSSWLQDGSARSSPPLRTPDVLESSGP	2093700	1702000	652
O14654	764	Insulin receptor substrate 4	IRS4	0.309707	0.000490776	61.602	T	RVSPPPAPSPPKAPDTNKEDDSKDNDSESDY	0	0	7128
Q8TC71	507	Mitochondria-eating protein	SPATA18	1	0.027958	61.593	S	AFWNSVRSVSRCRSRSLSPICPRSQIGLNTM	0	393670	4459
Q8TC71	509	Mitochondria-eating protein	SPATA18	1	0.027958	61.593	S	WNSVRSVSRCRSRSLSPICPRSQIGLNTMSR	0	393670	4460
P16333	166	Cytoplasmic protein NCK1	NCK1	0.577843	0.000139019	61.538	S	QVGWFPSNYVTEEGDSPLGDHVGSLSEKLAA	1278100	1089600	1085
Q8IWX8	823	Calcium homeostasis endoplasmic reticulum protein	CHERP	0.912301	0.00163141	61.537	S	GRRRRSRSRSPTPPSSAGLGSNSAPPIPDSR	2600300	0	4080
Q96B01	19	RAD51-associated protein 1	RAD51AP1	0.945143	0.00120514	61.437	S	PVRHKKPVNYSQFDHSDSDDDFVSATVPLNK	1171100	753040	4789
Q9NRZ9	503	Lymphoid-specific helicase	HELLS	0.739152	0.0314225	61.419	S	IANMFGSSEKETIELSPTGRPKRRTRKSINY	458420	375720	5990
Q8IXM2	141	Chromatin complexes subunit BAP18	BAP18	0.64277	0.00105261	61.415	S	AGGPPIKKQKADVTLSALNDSDANSDVVDIE	772000	0	4102
Q5SY16	80	Polynucleotide 5'-hydroxyl-kinase NOL9	NOL9	0.5454	0.000493375	61.394	T	GARQVSRAAAARRPNTATPSPIPSPTPASEP	0	657120	7714
P51956	319	Serine/threonine-protein kinase Nek3	NEK3	1	0.0296064	61.375	S	NEASTVQEEEQDRKGSHTDLESINENLVESA	780870	523180	1804
P51956	325	Serine/threonine-protein kinase Nek3	NEK3	1	0.0296064	61.375	S	QEEEQDRKGSHTDLESINENLVESALRRVNR	780870	523180	1805
P51956	333	Serine/threonine-protein kinase Nek3	NEK3	1	0.0296064	61.375	S	GSHTDLESINENLVESALRRVNREEKGNKSV	780870	523180	1806
Q5TDH0	126	Protein DDI1 homolog 2	DDI2	0.377799	0.000843869	61.37	T	QPPGTQQSHSSPGEITSSPQGLDNPALLRDM	0	0	7727
P49006	184	MARCKS-related protein	MARCKSL1	0.602682	3.61E-05	61.353	S	AGPQATEPSTPSGPESGPTPASAEQNE____	0	0	1637
P11388	1397	DNA topoisomerase 2-alpha	TOP2A	0.347608	0.00203279	61.345	T	DDVKGSVPLSSSPPATHFPDETEITNPVPKK	0	0	7277
Q86X10	417	Ral GTPase-activating protein subunit beta	RALGAPB	0.617209	0.00343378	61.304	S	STAHASKVQHQTSSTSPLSSPNQTSSEPRPL	870240	608020	3999
Q86X10	421	Ral GTPase-activating protein subunit beta	RALGAPB	0.966024	0.00343378	61.304	S	ASKVQHQTSSTSPLSSPNQTSSEPRPLPAPR	870240	608020	4000
Q13263	626	Transcription intermediary factor 1-beta	TRIM28	0.464318	5.70E-05	61.283	T	TSAPGGGPGTLDDSATICRVCQKPGDLVMCN	0	0	7531
Q9UN36	357	Protein NDRG2	NDRG2	0.513711	0.00266078	61.276	T	NRSRSRTLSQSSESGTLSSGPPGHTMEVSC_	0	1505300	8244
Q5JR12	68	Protein phosphatase 1J	PPM1J	0.76141	0.0188019	61.265	S	GSATPAKAVEARASFSRPTFLQLSPGGLRRA	545660	360760	3170
Q5JR12	76	Protein phosphatase 1J	PPM1J	0.926113	0.0188019	61.265	S	VEARASFSRPTFLQLSPGGLRRADDHAGRAV	545660	360760	3171
Q9UQ35	431	Serine/arginine repetitive matrix protein 2	SRRM2	0.986576	0.00520414	61.238	S	SSSESSPPSPQPTKVSRHASSSPESPKPAPA	4410100	1763800	6653
Q8NFU1	135	Bestrophin-2	BEST2	0.999888	0.0299584	61.235	S	RGRLYRRTLMRYAGLSAVLILRSVSTAVFKR	243590	0	4405
Q8NFU1	144	Bestrophin-2	BEST2	0.837802	0.0299584	61.235	S	MRYAGLSAVLILRSVSTAVFKRFPTIDHVVE	243590	0	4406
Q7Z5L9	392	Interferon regulatory factor 2-binding protein 2	IRF2BP2	0.477136	0.00318697	61.186	T	QPWLSTSTEGLKIPMTPTSSFVSPPPPTASP	0	0	7811
Q15911	1590	Zinc finger homeobox protein 3	ZFHX3	0.334841	0.00409881	61.181	S	RALQESATGQPEPTSSPDNKPFKCNTCNVAY	0	0	2993
Q6PJG2	700	ELM2 and SANT domain-containing protein 1	ELMSAN1	0.914395	0.00241958	61.177	S	PITPKSAHRTLLRTNSAEVTPPVLSVMGEAT	844640	549950	3549
Q6PJG2	704	ELM2 and SANT domain-containing protein 1	ELMSAN1	0.891868	0.00241958	61.177	T	KSAHRTLLRTNSAEVTPPVLSVMGEATPVSI	844640	549950	7764
Q9UIF9	500	Bromodomain adjacent to zinc finger domain protein 2A	BAZ2A	0.333334	0.00803685	61.165	S	PLTASVTSPKASPVTSPAAAFPTASPANKDV	0	0	6381
P46821	1016	Microtubule-associated protein 1B;MAP1B heavy chain;MAP1 li	MAP1B	1	0.00664214	61.16	S	EEDMDEAIEKGEAEQSEEEADEEDKAEDARE	852350	0	1585
Q13535	428	Serine/threonine-protein kinase ATR	ATR	0.98948	0.000589518	61.141	S	CQTQQENLSSNSDGISPKRRRLSSSLNPSKR	1152100	548790	2570
O15075	312	Serine/threonine-protein kinase DCLK1	DCLK1	0.406734	0.00401624	61.139	S	KSPGPSRRSKSPASTSSVNGTPGSQLSTPRS	0	0	281
O15075	313	Serine/threonine-protein kinase DCLK1	DCLK1	0.324229	0.00401624	61.139	S	SPGPSRRSKSPASTSSVNGTPGSQLSTPRSG	0	0	282
Q8ND24	506	RING finger protein 214	RNF214	0.427194	0.00102476	61.136	S	PALSQPSQPSSPLPGSHGRNSPGLGSLVSPH	0	0	4337
P49023	84	Paxillin	PXN	0.494325	0.0112166	61.127	S	WQPSSSRFIHQQPQSSSPVYGSSAKTSSVSN	0	0	1642
Q15545	200	Transcription initiation factor TFIID subunit 7	TAF7	0.935485	0.0112166	61.127	S	AEDETKEAENQGLDISSPGMSGHRQGHDSLE	1992200	0	2942
Q5VZ89	999	DENN domain-containing protein 4C	DENND4C	0.410489	0.000359105	61.112	S	IVKVPSGIFDVNSRKSSTGSISNVLFSTQDP	0	0	3366
Q5VZ89	1000	DENN domain-containing protein 4C	DENND4C	0.410489	0.000359105	61.112	S	VKVPSGIFDVNSRKSSTGSISNVLFSTQDPV	0	0	3367
Q5VZ89	1003	DENN domain-containing protein 4C	DENND4C	0.408706	0.000359105	61.112	S	PSGIFDVNSRKSSTGSISNVLFSTQDPVEDA	0	0	3368
Q5VZ89	1005	DENN domain-containing protein 4C	DENND4C	0.28023	0.000359105	61.112	S	GIFDVNSRKSSTGSISNVLFSTQDPVEDAVF	0	0	3369
Q5VZ89	1001	DENN domain-containing protein 4C	DENND4C	0.410489	0.000359105	61.112	T	KVPSGIFDVNSRKSSTGSISNVLFSTQDPVE	0	0	7734
O14654	874	Insulin receptor substrate 4	IRS4	0.861245	0.00706267	61.099	S	SGEGSFSKPGDGGSPSKPSDHEPPKNKAKRP	0	0	175
O14654	859	Insulin receptor substrate 4	IRS4	0.59101	0.00706267	61.099	S	EVSYNWDPKDAASKPSGEGSFSKPGDGGSPS	0	0	177
P21675	522	Transcription initiation factor TFIID subunit 1	TAF1	0.295644	0.000140415	61.03	S	ENLILEIPDEKEEATSNSPSKESKKESSLKK	0	0	1199
Q9Y2U5	297	Mitogen-activated protein kinase kinase kinase 2	MAP3K2	0.912921	0.000637936	61.022	S	KTFPRARRTQGTSLRSPVSFSPTDHSLSTSS	1797500	994020	6804
Q9Y2U5	302	Mitogen-activated protein kinase kinase kinase 2	MAP3K2	0.542644	0.000637936	61.022	S	ARRTQGTSLRSPVSFSPTDHSLSTSSGSSIF	1797500	0	6805
P13861	105	cAMP-dependent protein kinase type II-alpha regulatory subu	PRKAR2A	0.522786	0.0105882	61.015	Y	VPSRFNRRVSVCAETYNPDEEEEDTDPRVIH	821230	569860	8356
P39880	1215	Homeobox protein cut-like 1	CUX1	0.436563	0.000228048	60.951	S	DMKRMEKKAYMKRRHSSVSDSQPCEPPSVGT	0	0	1462
Q8IWA0	798	WD repeat-containing protein 75	WDR75	0.839245	0.0112902	60.938	T	DEENDFTEKVQDTSNTGLGEDIIHQLSKSEE	0	1335500	7841
Q5JVS0	108	Intracellular hyaluronan-binding protein 4	HABP4	0.999968	0.00347145	60.826	S	KERKSLPAPVAQRPDSPGGGLQAPGQKRTPR	0	5968000	3203
Q14160	1549	Protein scribble homolog	SCRIB	0.77603	0.0001258	60.762	T	ERLAEAPSPAPTPSPTPVEDLGPQTSTSPGR	0	0	7581
O60318	529	Germinal-center associated nuclear protein	MCM3AP	0.442503	0.00263683	60.761	S	FSLKEKKPGDGEVSPSTEDAPFQHSPLGKAA	0	0	478
Q13136	787	Liprin-alpha-1	PPFIA1	0.321967	0.00204592	60.761	S	NSTGSQDGPVSNPSSSNSSQDSLHKAPKKKG	0	0	2417
Q9Y3S1	1843	Serine/threonine-protein kinase WNK2	WNK2	0.999955	0.00195749	60.761	S	AQTASSIEVGVGEPVSSDSGDEGPRARPPVQ	732610	0	6865
Q9Y3S1	1844	Serine/threonine-protein kinase WNK2	WNK2	0.999955	0.00195749	60.761	S	QTASSIEVGVGEPVSSDSGDEGPRARPPVQK	732610	0	6866
Q9Y3S1	1846	Serine/threonine-protein kinase WNK2	WNK2	0.999971	0.00195749	60.761	S	ASSIEVGVGEPVSSDSGDEGPRARPPVQKQA	732610	0	6867
P55884	152	Eukaryotic translation initiation factor 3 subunit B	EIF3B	0.54388	0.000315679	60.728	S	EAEPRALENGDADEPSFSDPEDFVDDVSEEE	25295000	0	1885
P49792	2805	E3 SUMO-protein ligase RanBP2	RANBP2	0.751875	0.00611767	60.635	S	FCKSEEPDSITKSISSPSVSSETMDKPVDLS	1338400	840730	1717
P23468	890	Receptor-type tyrosine-protein phosphatase delta	PTPRD	0.346607	0.00868417	60.564	S	EKEDHFTATDIHKGASYVFRLSARNKVGFGE	0	0	1222
P23468	896	Receptor-type tyrosine-protein phosphatase delta	PTPRD	0.346607	0.00868417	60.564	S	TATDIHKGASYVFRLSARNKVGFGEEMVKEI	0	0	1223
Q9H2Y7	1372	Zinc finger protein 106	ZNF106	0.499667	0.00625759	60.564	T	KKKSLRAAHVPENSDTEQDVLTVKPVRKVKA	0	0	8093
Q14202	263	Zinc finger MYM-type protein 3	ZMYM3	0.95157	0.000118405	60.562	S	PPKPEVVDSTESIPVSDEDSDAMVDDPNDED	644950	461810	2700
Q6P1L5	410	Protein FAM117B	FAM117B	0.453105	0.000524594	60.527	S	QTPGGADRGSNNSSRSQSVSPTSFLTISNEG	0	0	3506
Q6P1L5	412	Protein FAM117B	FAM117B	0.453105	0.000524594	60.527	S	PGGADRGSNNSSRSQSVSPTSFLTISNEGSE	0	0	3507
Q6P1L5	414	Protein FAM117B	FAM117B	0.323961	0.000524594	60.527	S	GADRGSNNSSRSQSVSPTSFLTISNEGSEES	0	0	3508
Q6P1L5	417	Protein FAM117B	FAM117B	0.386706	0.000524594	60.527	S	RGSNNSSRSQSVSPTSFLTISNEGSEESPCS	0	0	3509
Q9ULL5	1925	Proline-rich protein 12	PRR12	0.998182	0.000403293	60.527	S	LHTFPQLQVEQSGEGSPEEGAVRLRPAGEPY	1807700	1008400	6486
Q6P1L5	416	Protein FAM117B	FAM117B	0.323961	0.000524594	60.527	T	DRGSNNSSRSQSVSPTSFLTISNEGSEESPC	0	0	7753
Q86UQ4	3007	ATP-binding cassette sub-family A member 13	ABCA13	0.665649	0.0281469	60.436	S	SSPNQLNCESLSKNLSSTLESFKSSLENATG	0	487280	3935
P10636	717	Microtubule-associated protein tau	MAPT	0.98965	0.0108445	60.419	S	KTDHGAEIVYKSPVVSGDTSPRHLSNVSSTG	4422500	2933200	999
Q86VZ4	360	Low-density lipoprotein receptor-related protein 11	LRP11	0.650118	0.0174798	60.419	S	QNLGLDRKMVTHTAASPALPRTTGPSEDAGG	2355700	0	3971
Q86VZ4	370	Low-density lipoprotein receptor-related protein 11	LRP11	0.947431	0.0174798	60.419	S	THTAASPALPRTTGPSEDAGGDSLVEKSQKA	2355700	0	3972
Q86VZ4	377	Low-density lipoprotein receptor-related protein 11	LRP11	0.996405	0.0174798	60.419	S	ALPRTTGPSEDAGGDSLVEKSQKATAPNKPP	2355700	0	3973
Q86VZ4	366	Low-density lipoprotein receptor-related protein 11	LRP11	0.673942	0.0174798	60.419	T	RKMVTHTAASPALPRTTGPSEDAGGDSLVEK	2355700	0	7829
Q86VZ4	367	Low-density lipoprotein receptor-related protein 11	LRP11	0.673942	0.0174798	60.419	T	KMVTHTAASPALPRTTGPSEDAGGDSLVEKS	2355700	0	7830
Q5T200	1465	Zinc finger CCCH domain-containing protein 13	ZC3H13	0.998913	0.000935604	60.363	S	AHSLGSGAGEGYEPISDDELDEILAGDAEKR	3448300	1429400	3259
Q5T200	1461	Zinc finger CCCH domain-containing protein 13	ZC3H13	0.508658	0.000935604	60.363	Y	KLDDAHSLGSGAGEGYEPISDDELDEILAGD	3448300	1429400	8384
Q5VT52	476	Regulation of nuclear pre-mRNA domain-containing protein 2	RPRD2	0.363937	0.00868849	60.33	S	SSLTSVMKNTGVSPASRPSPGTPTSPSNLTS	0	0	3332
Q04637	1077	Eukaryotic translation initiation factor 4 gamma 1	EIF4G1	0.772634	0.01106	60.307	S	RPIDTSRLTKITKPGSIDSNNQLFAPGGRLS	2128600	626440	2158
Q7Z3K3	419	Pogo transposable element with ZNF domain	POGZ	0.627197	0.01106	60.307	S	YQKKGKSLDSEPSVPSAAKPPSPEKTAPVAS	0	1021900	3782
Q9Y2W1	297	Thyroid hormone receptor-associated protein 3	THRAP3	0.390223	0.00015653	60.26	T	QMGSTLPSGAGYQSGTHQGQFDHGSGSLSPS	0	0	8288
Q96T21	309	Selenocysteine insertion sequence-binding protein 2	SECISBP2	0.97494	0.0103082	60.218	S	ELSWTPMGYVVRQTLSTELSAAPKNVTSMIN	0	0	5145
Q96T21	321	Selenocysteine insertion sequence-binding protein 2	SECISBP2	0.724277	0.0103082	60.218	S	QTLSTELSAAPKNVTSMINLKTIASSADPKN	0	0	5146
Q96T21	307	Selenocysteine insertion sequence-binding protein 2	SECISBP2	0.885118	0.0103082	60.218	T	TRELSWTPMGYVVRQTLSTELSAAPKNVTSM	0	0	8010
Q96T21	310	Selenocysteine insertion sequence-binding protein 2	SECISBP2	0.929546	0.0103082	60.218	T	LSWTPMGYVVRQTLSTELSAAPKNVTSMINL	0	0	8011
Q9C0F3	10	Zinc finger protein 436	ZNF436	0.999149	0.0155307	60.171	S	______MAATLLMAGSQAPVTFEDMAMYLTR	0	0	5546
Q9C0F3	4	Zinc finger protein 436	ZNF436	0.998756	0.0155307	60.171	T	____________MAATLLMAGSQAPVTFEDM	0	0	8080
Q9C0F3	15	Zinc finger protein 436	ZNF436	0.977139	0.0155307	60.171	T	_MAATLLMAGSQAPVTFEDMAMYLTREEWRP	0	0	8081
O95759	592	TBC1 domain family member 8	TBC1D8	0.978099	0.0102355	60.161	S	LTAYAHRNPKIGYCQSMNILTSVLLLYTKEE	0	0	805
O95759	604	TBC1 domain family member 8	TBC1D8	0.559635	0.0102355	60.161	T	YCQSMNILTSVLLLYTKEEEAFWLLVAVCER	0	0	7244
O95759	589	TBC1 domain family member 8	TBC1D8	0.997082	0.0102355	60.161	Y	RRVLTAYAHRNPKIGYCQSMNILTSVLLLYT	0	0	8350
Q9NYF8	389	Bcl-2-associated transcription factor 1	BCLAF1	0.876298	0.0264592	60.104	S	EWEDQEALDYFSDKESGKQKFNDSEGDDTEE	0	0	6102
O75530	29	Polycomb protein EED	EED	0.449958	0.000100735	60.078	S	DMPAAKKQKLSSDENSNPDLSGDENDDAVSI	0	0	636
Q5SW79;Q	1522;231	Centrosomal protein of 170 kDa;Cep170-like protein	CEP170;CEP	0.800711	0.00512682	59.991	S	GFPSAMLPSPPKQKSSPVNNHHSPGQTPTLG	0	9852700	3223
Q5SW79;Q	1529;238	Centrosomal protein of 170 kDa;Cep170-like protein	CEP170;CEP	0.70013	0.00512682	59.991	S	PSPPKQKSSPVNNHHSPGQTPTLGQPEARAL	0	9852700	3224
Q7Z434	157	Mitochondrial antiviral-signaling protein	MAVS	0.911557	0.000983115	59.991	S	MPVQETQAPESPGENSEQALQTLSPRAIPRN	1396300	1267900	3805
Q9NQG6	94	Mitochondrial dynamics protein MID51	MIEF1	0.989623	0.00518873	59.912	S	SPRLLNRDMKTGLSRSLQTLPTDSSTFDTDT	1932000	1917100	5916
Q9Y5J3	40	Hairy/enhancer-of-split related with YRPW motif protein 1	HEY1	0.876038	0.0025673	59.912	S	SADENGNLSSALGSMSPTTSSQILARKRRRG	0	738590	6960
Q96T58	2496	Msx2-interacting protein	SPEN	0.631554	0.0190271	59.862	T	SPPVASGGIPHQSPPTKVTEWITRQEEPRAQ	0	4185300	8016
Q6ZS86	2	Putative glycerol kinase 5	GK5	0.999925	0.0165518	59.84	S	_______________MSGLLTDPEQRAQEPRY	1181000	784930	3670
Q8NEZ4	2935	Histone-lysine N-methyltransferase 2C	KMT2C	0.642193	0.0216697	59.838	S	SLLANEKSDNSDIRPSGSPPPPTLPASPSNH	1520800	736330	4386
Q8NEZ4	2946	Histone-lysine N-methyltransferase 2C	KMT2C	0.488021	0.0216697	59.838	S	DIRPSGSPPPPTLPASPSNHVSSLPPFIAPP	0	0	4387
Q8NEZ4	2942	Histone-lysine N-methyltransferase 2C	KMT2C	0.392404	0.0216697	59.838	T	SDNSDIRPSGSPPPPTLPASPSNHVSSLPPF	0	0	7895
P49006	177	MARCKS-related protein	MARCKSL1	0.389327	0.000392018	59.789	S	SAASEEEAGPQATEPSTPSGPESGPTPASAE	0	0	1635
Q9NZM3	884	Intersectin-2	ITSN2	0.939898	0.00657725	59.789	S	VNTSWQKKSAFTRTVSPGSVSPIHGQGQVVE	4378900	3643500	6160
Q9NZM3	889	Intersectin-2	ITSN2	0.959086	0.00657725	59.789	S	QKKSAFTRTVSPGSVSPIHGQGQVVENLKAQ	4378900	3643500	6161
Q9BPY3	3	Protein FAM118B	FAM118B	0.29806	0.00319568	59.674	S	______________MASTGSQASDIDEIFGFF	0	0	5294
Q9BPY3	4	Protein FAM118B	FAM118B	0.29806	0.00319568	59.674	T	_____________MASTGSQASDIDEIFGFFN	0	0	8039
Q15633	156	RISC-loading complex subunit TARBP2	TARBP2	0.582856	0.00234425	59.661	S	RSPPMELQPPVSPQQSECNPVGALQELVVQK	0	0	2953
Q3KQU3	479	MAP7 domain-containing protein 1	MAP7D1	0.996221	0.00658774	59.65	S	KARPSSPSTSWHRPASPCPSPGPGHTLPPKP	3037700	2003800	3064
Q3KQU3	496	MAP7 domain-containing protein 1	MAP7D1	0.989357	0.00658774	59.65	S	CPSPGPGHTLPPKPPSRGTTASPKGRVRRK	3037700	2003800	3065
P20020	1165	Plasma membrane calcium-transporting ATPase 1	ATP2B1	0.988396	0.00652633	59.618	T	FRIEDSEPHIPLIDDTDAEDDAPTKRNSSPP	411580	440940	7307
Q6VMQ6	118	Activating transcription factor 7-interacting protein 1	ATF7IP	0.518029	0.00844813	59.585	T	QDDDLNCEPLSPHNITPEPVSKLPAEPVSGD	0	1874900	7773
Q9ULU4	442	Protein kinase C-binding protein 1	ZMYND8	0.491858	0.0093324	59.585	T	SDMPRSPMSTNSSVHTGSDVEQDAEKKATSS	0	0	8239
P10636	726	Microtubule-associated protein tau	MAPT	0.794622	0.00119623	59.567	S	YKSPVVSGDTSPRHLSNVSSTGSIDMVDSPQ	523400	105340	989
P10636	729	Microtubule-associated protein tau	MAPT	0.531906	0.00119623	59.567	S	PVVSGDTSPRHLSNVSSTGSIDMVDSPQLAT	523400	105340	990
P10636	730	Microtubule-associated protein tau	MAPT	0.531905	0.00119623	59.567	S	VVSGDTSPRHLSNVSSTGSIDMVDSPQLATL	523400	105340	991
P10636	733	Microtubule-associated protein tau	MAPT	0.531905	0.00119623	59.567	S	GDTSPRHLSNVSSTGSIDMVDSPQLATLADE	523400	105340	992
P10636	731	Microtubule-associated protein tau	MAPT	0.531905	0.00119623	59.567	T	VSGDTSPRHLSNVSSTGSIDMVDSPQLATLA	523400	105340	7275
Q8NI08	179	Nuclear receptor coactivator 7	NCOA7	0.727478	0.00369365	59.55	S	SSTLRLSSSSPGATVSPSSSDAEYDKLPDAD	0	1258000	4422
Q8NI08	187	Nuclear receptor coactivator 7	NCOA7	0.926185	0.00369365	59.55	Y	SSPGATVSPSSSDAEYDKLPDADLARKALKP	0	0	8399
O15432	74	Probable low affinity copper uptake protein 2	SLC31A2	0.636273	0.000624249	59.536	S	PTSISQQTIAETDGDSAGSDSFPVGRTHHRW	1526800	0	317
O15432	77	Probable low affinity copper uptake protein 2	SLC31A2	0.79874	0.000624249	59.536	S	ISQQTIAETDGDSAGSDSFPVGRTHHRWYLC	1526800	0	318
Q5VUA4	1717	Zinc finger protein 318	ZNF318	0.560702	0.00169453	59.536	S	SSFQSDTSRDISPEKSELDLGEPGPPGVEPP	1494000	0	3341
Q9BUR4	112	Telomerase Cajal body protein 1	WRAP53	0.962547	0.00904423	59.434	S	ELSENTSLPAEEANGSLSEEEANGPELGSGK	0	0	5388
Q8N1F8	459	Serine/threonine-protein kinase 11-interacting protein	STK11IP	0.319385	0.000161828	59.401	S	LPATPTTSAPSAPPASSQGPDTAPRPSPPQE	0	0	4218
Q8N1F8	460	Serine/threonine-protein kinase 11-interacting protein	STK11IP	0.319385	0.000161828	59.401	S	PATPTTSAPSAPPASSQGPDTAPRPSPPQEE	0	0	4219
Q9UBT2	289	SUMO-activating enzyme subunit 2	UBA2	0.455801	0.00114057	59.372	S	RKRKPPVPLDWAEVQSQGEETNASDQQNEPQ	0	0	6272
Q9UBT2	294	SUMO-activating enzyme subunit 2	UBA2	0.455801	0.00114057	59.372	T	PVPLDWAEVQSQGEETNASDQQNEPQLGLKD	0	0	8201
Q7Z460	649	CLIP-associating protein 1	CLASP1	0.413172	0.0277926	59.345	S	YASLGRIRTRRQSSGSATNVASTPDNRGRSR	0	0	3811
P48634	610	Protein PRRC2A	PRRC2A	0.5	0.0160979	59.345	T	SKEGPEPPEEVPPPTTPPVPKVEPKGDGIGP	2349500	805110	7403
Q96GM8	428	Target of EGR1 protein 1	TOE1	0.681473	0.00574625	59.342	S	EIADRATSEVPGSQASPNPVPGDGLHRAGFD	534250	1164600	4919

Q6ZN08	521	Putative zinc finger protein 66	ZNF66	0.977617	0.02062	59.265	S	DKPYKCEECGKDFKYSSTLTRHKKIHTGGKP	2268500	0	3647
Q6ZN08	523	Putative zinc finger protein 66	ZNF66	0.833231	0.02062	59.265	T	PYKCEECGKDFKYSSTLTRHKKIHTGGKPHK	2268500	0	7780
Q6ZN08	525	Putative zinc finger protein 66	ZNF66	0.690347	0.02062	59.265	T	KCEECGKDFKYSSTLTRHKKIHTGGKPHKCN	2268500	0	7781
Q6ZN08	520	Putative zinc finger protein 66	ZNF66	0.999943	0.02062	59.265	Y	ADKPYKCEECGKDFKYSSTLTRHKKIHTGGK	2268500	0	8391
Q6RI45	1756	Bromodomain and WD repeat-containing protein 3	BRWD3	0.515698	0.00622994	59.237	T	FSRLPRIKTRNQGRRTVLYNDDSDNDNFVST	0	0	7770
Q71F56	817	Mediator of RNA polymerase II transcription subunit 13-like	MED13L	0.98755	0.000342227	59.23	S	DLAPSLHDLDNIFDNSDDDELGAVSPALRSS	652920	0	3692
Q71F56	826	Mediator of RNA polymerase II transcription subunit 13-like	MED13L	0.999556	0.000342227	59.23	S	DNIFDNSDDDELGAVSPALRSSKMPAVGTED	652920	0	3693
Q13685	210	Angio-associated migratory cell protein	AAMP	1	0.0241035	59.227	T	DGNTWMWKVPNGDCKTFQGPNCPATCGRVLP	1094100	0	7571
Q13685	219	Angio-associated migratory cell protein	AAMP	1	0.0241035	59.227	T	PNGDCKTFQGPNCPATCGRVLPDGKRAVVGY	1094100	0	7572
Q9UDY2	925	Tight junction protein ZO-2	TJP2	0.858092	0.00130006	59.226	T	DYLSCDSRLISDFEDTDGEGGAYTDNELDEP	1398900	0	8202
P46821	1817	Microtubule-associated protein 1B;MAP1B heavy chain;MAP1 lig	MAP1B	0.26602	0.00513329	59.189	S	DSTSAVKEKTATCHSSSSPPIDAASAEPYGF	0	0	1602
Q9Y462	459	Zinc finger protein 711	ZNF711	1	0.0255903	59.185	S	KTHEFTEYTRRYREASPLSSNKLILRDKEPK	0	200710	6892
Q9Y462	462	Zinc finger protein 711	ZNF711	1	0.0255903	59.185	S	EFTEYTRRYREASPLSSNKLILRDKEPKMHK	0	0	6893
Q9Y462	463	Zinc finger protein 711	ZNF711	1	0.0255903	59.185	S	FTEYTRRYREASPLSSNKLILRDKEPKMHKC	0	0	6894
Q9Y462	455	Zinc finger protein 711	ZNF711	1	0.0255903	59.185	Y	NKVDKTHEFTEYTRRYREASPLSSNKLILRD	0	0	8437
Q13620	84	Cullin-4B	CUL4B	0.652772	0.0142255	59.182	S	SSNEREDFDSTSSSSSTPPLQPRDSASPSTS	726520	0	2607
O75030	491	Microphthalmia-associated transcription factor	MITF	0.848376	0.00292061	59.017	S	KMGSKLEDILMDDTLSPVGVTDPLLSSVSPG	0	1058100	546
O75030	504	Microphthalmia-associated transcription factor	MITF	0.666778	0.00292061	59.017	S	TLSPVGVTDPLLSSVSPGASKTSSRRSSMSM	0	1058100	547
Q9BV44	207	THUMP domain-containing protein 3	THUMPD3	0.712955	0.00793967	59.011	S	AIKEDVSTLIGDDLASCKDETDESSKEETEP	0	0	5394
Q9BV44	215	THUMP domain-containing protein 3	THUMPD3	0.704472	0.00793967	59.011	S	LIGDDLASCKDETDESSKEETEPQVLKFRVT	0	0	5395
Q8IZL8	745	Proline-, glutamic acid- and leucine-rich protein 1	PELP1	0.70525	0.0051571	58.998	T	HRAGSNEDPILAPSGTPPPTIPPDETFGGRV	3980300	0	7870
Q9HCE1	969	Putative helicase MOV-10	MOV10	0.995854	0.0145529	58.98	S	DLQQGQNLLQGLSKLSPSTSGPHSHDYLPQE	0	0	5855
P06400	606	Retinoblastoma-associated protein	RB1	0.55185	0.000959497	58.98	Y	PLNLPLQNNHTAADMYLSPVRSPKKKGSTTR	0	1855700	8351
Q86T24	234	Transcriptional regulator Kaiso	ZBTB33	0.515922	0.000237725	58.95	S	SNPGPVAISDVAPSASNNSPPLTNITPTQKL	11825000	0	3893
P46821	1326	Microtubule-associated protein 1B;MAP1B heavy chain;MAP1 lig	MAP1B	0.43764	0.00363413	58.92	S	ASPEDKTLEVVSPSQSVTGSAGHTPYYQSPT	0	0	1604
Q9BWW4	381	Single-stranded DNA-binding protein 3	SSBP3	0.689706	0.00444188	58.92	S	ELGGNFLHSFQNDNYSPSMTMSV________	5974400	0	5438
P46821	1328	Microtubule-associated protein 1B;MAP1B heavy chain;MAP1 lig	MAP1B	0.437755	0.00363413	58.92	T	PEDKTLEVVSPSQSVTGSAGHTPYYQSPTDE	0	0	7392
O60271	280	C-Jun-amino-terminal kinase-interacting protein 4	SPAG9	0.679766	0.00788656	58.919	T	SDVSQGGSKATTPASTANSDVATIPTDTPLK	1377200	0	7177
O94842	180	TOX high mobility group box family member 4	TOX4	0.483931	0.0192816	58.914	T	PAQSPEDRLSTTPSPTSSLHEDGVEDFRRQL	0	0	7231
Q7Z4S6	1271	Kinesin-like protein KIF21A	KIF21A	0.670434	0.00797209	58.913	S	KEQKHSDSGTSEASLSPPSSPPSRPRNELNV	0	0	3818
Q7Z4S6	1275	Kinesin-like protein KIF21A	KIF21A	0.834045	0.00797209	58.913	S	HSDSGTSEASLSPPSSPPSRPRNELNVFNRL	0	0	3820
P50851	1767	Lipopolysaccharide-responsive and beige-like anchor protein	LRBA	0.841938	0.000377145	58.867	S	VSSVDSAQASDMGGESPGSRSSNAKLPSVPT	1778500	0	1764
Q7Z3K6	122	Mesoderm induction early response protein 3	MIER3	0.568797	0.00227444	58.867	S	EIAKDLLSGDDEETQSSADDLTPSVTSHETS	841210	523360	3785
Q7Z3K6	120	Mesoderm induction early response protein 3	MIER3	0.864859	0.00227444	58.867	T	KEEIAKDLLSGDDEETQSSADDLTPSVTSHE	841210	523360	7802
P43487	21	Ran-specific GTPase-activating protein	RANBP1	0.40756	0.00105789	58.861	S	DTHEDHDTSTENTDESNHDPQFEPIVSLPEQ	0	0	1525
Q8N1G0	215	Zinc finger protein 687	ZNF687	0.727838	0.000848401	58.861	S	ELAQENGPGMQPPVSSPPLGALKQESCSPHH	1553900	0	4226
Q8NHM5	1031	Lysine-specific demethylase 2B	KDM2B	0.499158	0.0309728	58.824	S	LRSPPRVISRPPPSVSPPKCIQMERHVIRPP	0	0	4417
Q86XP3	744	ATP-dependent RNA helicase DDX42	DDX42	0.364007	0.000271756	58.823	S	SGWTSAGSLNSVPTNSAQQGHNSPDSPVTSA	0	0	4011
Q86XP3	751	ATP-dependent RNA helicase DDX42	DDX42	0.801518	0.000271756	58.823	S	SLNSVPTNSAQQGHNSPDSPVTSAAKGIPGF	1661600	0	4012
Q13586	523	Stromal interaction molecule 1	STIM1	0.656341	0.0231332	58.817	S	HGLGSQRDLTHSDSESSLHMSDRQRVAPKPP	0	702110	2585
Q9Y388	146	RNA-binding motif protein, X-linked 2	RBMX2	0.884573	0.0233599	58.814	S	LQEKGCGARTPSPSLSESSEDEKPTKKHKKD	878800	308180	6847
Q15424	21	Scaffold attachment factor B1	SAFB	0.231084	0.00307506	58.802	S	SGLGDSGAAGAAALSSASSETGTRRLSDLRV	0	0	2917
O60291	524	E3 ubiquitin-protein ligase MGRN1	MGRN1	0.877959	0.0149695	58.723	S	QGTRAASIENVLQDSSPEHCGRGPPADIYLP	2754800	1185300	466
Q9Y2K5;Q[illegible]	142;141	R3H domain-containing protein 2;R3H domain-containing protei	R3HDM2;R3HI	0.499591	0.00582126	58.722	S	DKNKEKIPRKMLSRDSSQEYTDSTGIDLHEF	0	0	6789
Q9Y2K5;Q[illegible]	143;142	R3H domain-containing protein 2;R3H domain-containing protei	R3HDM2;R3HI	0.499591	0.00582126	58.722	S	KNKEKIPRKMLSRDSSQEYTDSTGIDLHEFL	0	0	6790
Q9H814	21	Phosphorylated adapter RNA export protein	PHAX	0.857625	0.00220238	58.722	T	GDMEDGQLSDSDSDMTVAPSDRPLQLPKVLG	2986600	0	8120
Q96GM8	425	Target of EGR1 protein 1	TOE1	0.810237	0.0067253	58.693	S	ARPEIADRATSEVPGSQASPNPVPGDGLHRA	0	0	4918
Q02880	1403	DNA topoisomerase 2-beta	TOP2B	0.742028	0.00533766	58.666	T	DNNDLEELKVKASPITNDGEDEFVPSDGLDK	0	0	7476
Q9BXK1	230	Krueppel-like factor 16	KLF16	0.218171	0.0032715	58.498	S	DLLRRPGARSTSPSDSLPCSLAGSPAPSPAP	0	0	5456
P55884	85	Eukaryotic translation initiation factor 3 subunit B	EIF3B	0.642854	0.00017819	58.482	S	PAAEAEAASGPSESPSPPAAEELPGSHAEPP	0	0	1891
Q9ULJ3	348	Zinc finger and BTB domain-containing protein 21	ZBTB21	0.432072	0.0153651	58.479	S	GPLVKSLLRRSLSMDSQVPVYSPSIDLKSSQ	0	0	6481
O15014	252	Zinc finger protein 609	ZNF609	0.936129	0.001231	58.47	S	NECRLLKKVKSEKMESPVSTPAVLPIHLLVP	779420	508600	232
Q8NEZ4	4300	Histone-lysine N-methyltransferase 2C	KMT2C	1	0.00498871	58.418	S	STLDVHCLPQLPEKASPPASPPIAFPPAFEA	2432300	1994500	4380
Q8NEZ4	4304	Histone-lysine N-methyltransferase 2C	KMT2C	1	0.00498871	58.418	S	VHCLPQLPEKASPPASPPIAFPPAFEAAQVE	2432300	1994500	4381
Q04721	2074	Neurogenic locus notch homolog protein 2;Notch 2 extracellul	NOTCH2	0.668006	0.00486651	58.418	T	VRLLDEYNVTPSPPGTVLTSALSPVICGPNR	0	0	7491
Q9UDY2	932	Tight junction protein ZO-2	TJP2	0.54809	0.0025499	58.398	Y	RLISDFEDTDGEGGAYTDNELDEPAEEPLVS	0	0	8432
P42166	156	Lamina-associated polypeptide 2, isoform alpha;Thymopoietin;	TMPO	0.599952	0.0113432	58.366	S	LYEKKLLKLREQGTESRSSTPLPTISSSAEN	0	457240	1492
Q7KZ85	7	Transcription elongation factor SPT6	SUPT6H	0.441426	0.00520755	58.366	S	_________MSDFVESEAEESEEEYNDEGEV	0	0	3708
Q7KZ85	12	Transcription elongation factor SPT6	SUPT6H	0.441426	0.00520755	58.366	S	____MSDFVESEAEESEEEYNDEGEVVPRVT	0	0	3709
P42166	154	Lamina-associated polypeptide 2, isoform alpha;Thymopoietin;	TMPO	0.599967	0.0113432	58.366	T	RKLYEKKLLKLREQGTESRSSTPLPTISSSA	0	457240	7376
O76094	618	Signal recognition particle subunit SRP72	SRP72	0.580448	0.00801982	58.361	T	QGATAGASSELDASKTVSSPPTSPRPGSAAT	12401000	0	7225
O76094	633	Signal recognition particle subunit SRP72	SRP72	0.397444	0.00801982	58.361	T	TVSSPPTSPRPGSAATVSASTSNIIPPRHQK	0	0	7227
Q7Z460	246	CLIP-associating protein 1	CLASP1	0.861961	0.00925715	58.339	S	NMIQSANDKNFDDEDSVDGNRPSSASSTSSK	0	476320	3807
Q05519	212	Serine/arginine-rich splicing factor 11	SRSF11	0.372469	0.0120238	58.29	S	KLNHVAAGLVSPSLKSDTSSKEIEEAMKRVR	0	0	2191
Q5JR12	25	Protein phosphatase 1J	PPM1J	0.992116	0.0096421	58.29	S	AHLVSSGGAPPPRPKSPDLPNAASAPPAAAP	688740	340430	3172
Q6V9R5	2	Zinc finger protein 562	ZNF562	0.967324	0.0290267	58.274	S	______________MSAFDMSHGFFPREPIC	577280	552880	3599
Q5T1M5	307	FK506-binding protein 15	FKBP15	0.926743	0.00352523	58.26	S	ARDSGSDGHSVSSRDSAAPSPIPGADNLSAD	1703100	0	3241
Q96T58	1629	Msx2-interacting protein	SPEN	0.956615	0.004396	58.26	S	ENHPKTPESAPENKDSELKTPPSVGPPSVTV	0	3123700	5166
Q99613;B[illegible]	6;6	Eukaryotic translation initiation factor 3 subunit C;Eukaryo	EIF3C;EIF3(	0.710335	0.00522564	58.21	T	__________MSRFFTTGSDSESESSLSGEE	0	1242900	7113
Q7Z2K8	436	G protein-regulated inducer of neurite outgrowth 1	GPRIN1	0.999991	0.011946	58.168	S	LGKMDPMCSGKPELLSPGQAERVSVGKAGTV	10976000	5374900	3748
Q7Z2K8	444	G protein-regulated inducer of neurite outgrowth 1	GPRIN1	0.999995	0.011946	58.168	S	SGKPELLSPGQAERVSVGKAGTVSPGKEDPV	10976000	5374900	3749
P10588	40	Nuclear receptor subfamily 2 group F member 6	NR2F6	0.899577	0.000977317	58.137	S	PRAAEDDSASPPGAASDAEPGDEERPGLQVD	1179600	858030	988
Q9ULL5	570	Proline-rich protein 12	PRR12	0.9971	0.0094567	58.123	S	GGGEASPSHIIRPLQSPPATGRPPGVGSPGA	6335600	3644900	6489
Q5SY16	82	Polynucleotide 5'-hydroxyl-kinase NOL9	NOL9	0.553516	0.00603203	58.122	T	RQVSRAAAARRPNTATPSPIPSPTPASEPES	726810	0	7715
Q9UK58	329	Cyclin-L1	CCNL1	0.474451	0.0122497	58.015	S	KLKAKGLNPDGTPALSTLGGFSPASKPSSPR	0	0	6418
Q8NCF5	84	NFATC2-interacting protein	NFATC2IP	1	0.0102003	57.945	S	DEVEVEPPEPPGPVASRDNSNSDSEGEDRRP	2190100	728520	4328
P50851	1770	Lipopolysaccharide-responsive and beige-like anchor protein	LRBA	0.493098	0.000529651	57.925	S	VDSAQASDMGGESPGSRSSNAKLPSVPTVDS	0	0	1765
P22314	28	Ubiquitin-like modifier-activating enzyme 1	UBA1	0.742253	0.00630874	57.902	S	SGPDPKPGSNCSPAQSVLSEVPSVPTNGMAK	0	0	1212
Q13428	1179	Treacle protein	TCOF1	0.48022	0.000300186	57.832	T	QGAKSAHTLGPTPSRTETLVEETAAESSEDD	0	0	7539
Q13428	1181	Treacle protein	TCOF1	0.48022	0.000300186	57.832	T	AKSAHTLGPTPSRTETLVEETAAESSEDDVV	0	0	7540
Q13428	1186	Treacle protein	TCOF1	0.376639	0.000300186	57.832	T	TLGPTPSRTETLVEETAAESSEDDVVAPSQS	0	0	7541
O75533	273	Splicing factor 3B subunit 1	SF3B1	0.998575	0.00425494	57.768	T	PSHTPAGAATPGRGDTPGHATPGHGGATSSA	0	0	7206
Q53ET0	465	CREB-regulated transcription coactivator 2	CRTC2	0.212411	0.00734535	57.688	S	QLPKQFSPTMSPTLSSITQGVPLDTSKLSTD	0	0	3127
Q13542	25	Eukaryotic translation initiation factor 4E-binding protein	EIF4EBP2	0.749854	0.00360935	57.673	S	QPSQSRAIPTRTVAISDAAQLPHDYCTTPGG	0	11762000	2575
Q14654	208	ATP-sensitive inward rectifier potassium channel 11	KCNJ11	0.999831	0.0271712	57.567	S	HGRLCFMLRVGDLRKSMIISATIHMQVVRKT	0	59986	2749
Q14654	212	ATP-sensitive inward rectifier potassium channel 11	KCNJ11	0.999643	0.0271712	57.567	S	CFMLRVGDLRKSMIISATIHMQVVRKTTSPE	0	59986	2750
Q96CX2	151	BTB/POZ domain-containing protein KCTD12	KCTD12	0.999014	0.000921728	57.517	S	GPGPPPSRRGVHKEGSLGDELLPLGYSEPEQ	2341400	0	4829
Q15154	794	Pericentriolar material 1 protein	PCM1	0.486183	0.0117419	57.499	S	SVGNCPTKKYMPAVTSTPTVNQHETSTSKSV	0	0	2890
Q15154	806	Pericentriolar material 1 protein	PCM1	0.307351	0.0117419	57.499	S	AVTSTPTVNQHETSTSKSVFEPEDSSIVDNE	0	0	2891
Q96PC5	1144	Melanoma inhibitory activity protein 2	MIA2	0.997298	0.00862061	57.496	S	PSPLGWPSSETRAFLSPPTLLEGPLRLSPLL	1162700	422260	5048
Q96PC5	1156	Melanoma inhibitory activity protein 2	MIA2	0.966001	0.00862061	57.496	S	AFLSPPTLLEGPLRLSPLLPGGGGRGSRGPG	1162700	422260	5049
Q14202	172	Zinc finger MYM-type protein 3	ZMYM3	0.999916	0.00058651	57.488	S	IEEEETTSIATARRGSPGGQEEELPQGQPQSP	1214600	829200	2707
P56537	239	Eukaryotic translation initiation factor 6	EIF6	0.479079	0.0109041	57.482	S	LNEAQPSTIATSMRDSLIDSLT_________	0	0	1896
O95429	281	BAG family molecular chaperone regulator 4	BAG4	0.403577	0.00056719	57.443	S	PGNLYMTESTSPWPSSGSPQSPPSPPVQQPK	0	0	771
Q8WV07	4	Oral cancer-overexpressed protein 1	ORAOV1	1	0.0203779	57.434	S	____________MAGSQDIFDAIVMADERFH	0	231780	4526
O15446	488	DNA-directed RNA polymerase I subunit RPA34	CD3EAP	0.496332	0.00605582	57.432	S	PETVPQEEMPGPPLNSESGEEAPTGRDKKRK	0	0	321
Q5T5Y3	575	Calmodulin-regulated spectrin-associated protein 1	CAMSAP1	0.947285	0.00773198	57.427	S	PRASPRALGLTANARSPQGQLDTSESKPDSF	559380	472060	3293
Q07820	162	Induced myeloid leukemia cell differentiation protein Mcl-1	MCL1	0.660073	0.000465558	57.417	S	VGESGNNTSTDGSLPSTPPPAEEEEDELYRQ	0	1206200	2236
Q9Y5Q9	51	General transcription factor 3C polypeptide 3	GTF3C3	0.87652	0.00946307	57.39	S	LQEKGKLSAEENPDDSEVPSSSGINSTKSQD	7170100	4308900	6963
Q8WX93	1104	Palladin	PALLD	0.985863	0.0202815	57.347	S	RLMVQAVNQRGRSPRSPSGHPHVRRPRSRSR	0	486420	4583
Q6TGC4	441	Protein-arginine deiminase type-6	PADI6	0.99997	0.0109055	57.339	S	KVQGKEYPLGRVLIGSSFYPSAEGRAMSKTL	0	0	3581
Q6TGC4	442	Protein-arginine deiminase type-6	PADI6	0.999962	0.0109055	57.339	S	VQGKEYPLGRVLIGSSFYPSAEGRAMSKTLR	0	0	3582
Q6TGC4	453	Protein-arginine deiminase type-6	PADI6	0.776694	0.0109055	57.339	S	LIGSSFYPSAEGRAMSKTLRDFLYAQQVQAP	0	0	3583
Q6TGC4	444	Protein-arginine deiminase type-6	PADI6	0.999365	0.0109055	57.339	Y	GKEYPLGRVLIGSSFYPSAEGRAMSKTLRDF	0	0	8390
Q8WXE0	403	Caskin-2	CASKIN2	0.791606	0.00920326	57.293	S	PRVGLSPDSPAGDRNSVGSEGSVGSIRSAGS	2554500	804380	4586
Q9NVI1	1114	Fanconi anemia group I protein	FANCI	0.739364	0.0113316	57.293	T	VDWLITKLKGQVSQETLSEEASSQATLPNQP	0	3358100	8169
Q15036	331	Sorting nexin-17	SNX17	0.666245	0.0211745	57.288	S	TRMRCWRVTSSVPLPSGSTSSPGRGRGEVRL	711960	270250	2859
Q6W2J9	1437	BCL-6 corepressor	BCOR	0.325323	0.00690393	57.278	S	LQQLLPASQSTQLPCSSSPQETTQSRPMPPE	0	0	3615
Q7Z4S6	1269	Kinesin-like protein KIF21A	KIF21A	0.287954	0.0110456	57.278	S	KAKEQKHSDSGTSEASLSPPSSPPSRPRNEL	0	0	3817
Q7Z4S6	1274	Kinesin-like protein KIF21A	KIF21A	0.498643	0.0110456	57.278	S	KHSDSGTSEASLSPPSSPPSRPRNELNVFNR	0	0	3819
O60244	1128	Mediator of RNA polymerase II transcription subunit 14	MED14	0.926789	0.00768481	57.222	S	PQVSGPSPAARMPGMSPANPSLHSPVPDASH	3488100	1409400	442
O60244	1133	Mediator of RNA polymerase II transcription subunit 14	MED14	0.538861	0.00768481	57.222	S	PSPAARMPGMSPANPSLHSPVPDASHSPRAG	3488100	1409400	443
O60244	1136	Mediator of RNA polymerase II transcription subunit 14	MED14	0.538695	0.00768481	57.222	S	AARMPGMSPANPSLHSPVPDASHSPRAGTSS	3488100	1409400	444
O60244	1142	Mediator of RNA polymerase II transcription subunit 14	MED14	0.627266	0.00768481	57.222	S	MSPANPSLHSPVPDASHSPRAGTSSQTMPTN	3488100	1409400	445
Q9H3P7	47	Golgi resident protein GCP60	ACBD3	0.531641	0.000681279	57.205	S	APLLPPPLPPPSPPGSGRGPGASGEQPEPGE	178780	0	5641
Q9BQF6	443	Sentrin-specific protease 7	SENP7	0.999881	0.00593042	57.19	S	NEGNQSLISAEPIVVSSDEEGPVEHKSSEIL	1813700	0	5306
Q9BQF6	444	Sentrin-specific protease 7	SENP7	0.999949	0.00593042	57.19	S	EGNQSLISAEPIVVSSDEEGPVEHKSSEILK	1813700	0	5307
P17252	651	Protein kinase C alpha type	PRKCA	0.45624	0.000706017	57.184	S	VLTPPDQLVIANIDQSDFEGFSYVNPQFVHP	0	0	1106
P17252	657	Protein kinase C alpha type	PRKCA	0.456109	0.000706017	57.184	S	QLVIANIDQSDFEGFSYVNPQFVHPILQSAV	0	0	1107
P17252	638	Protein kinase C alpha type	PRKCA	0.999111	0.000706017	57.184	T	AENFDKFFTRGQPVLTPPDQLVIANIDQSDF	11135000	0	7294
Q14160	1298	Protein scribble homolog	SCRIB	0.907438	0.00616966	57.152	S	VPSGAAGGKMAESPCSPSGQQPPSPPSPDEL	1060300	0	2681
Q00613	319	Heat shock factor protein 1	HSF1	0.328328	0.00823783	57.145	S	PPQSPRVEEASPGRPSSVDTLLSPTALIDSI	0	0	2035
Q00613	320	Heat shock factor protein 1	HSF1	0.328328	0.00823783	57.145	S	PQSPRVEEASPGRPSSVDTLLSPTALIDSIL	0	0	2036
Q9UGU0	974	Transcription factor 20	TCF20	0.407154	0.0142303	57.142	S	ESYRGNASPGAATHDSLSDYGPQDSRPTPMR	0	0	6315
Q9UGU0	971	Transcription factor 20	TCF20	0.407154	0.0142303	57.142	T	IKHESYRGNASPGAATHDSLSDYGPQDSRPT	0	0	8208
O76080	54	AN1-type zinc finger protein 5	ZFAND5	0.725015	0.0133234	57.058	S	QRQQNSGRMSPMGTASGSNSPTSDSASVQRA	0	0	677
P22830	81	Ferrochelatase, mitochondrial	FECH	0.99998	0.01302	57.045	T	KPKTGILMLNMGGPETLGDVHDFLLRLFLDR	5734500	7166200	7314
P48169;Q[illegible]	289;273	Gamma-aminobutyric acid receptor subunit alpha-4;Gamma-amino	GABRA4;GABI	0.860098	0.0220182	57.009	T	SQVSFWINKESVPARTVFGITTVLTMTTLSI	1824500	0	7397
P23497	50	Nuclear autoantigen Sp-100	SP100	0.999997	0.0169713	56.959	Y	QRMFTEDQGVDDRLLYDIVFKHFKRNKVEIS	4008400	0	8358
P15927	29	Replication protein A 32 kDa subunit	RPA2	0.876428	0.000813666	56.957	S	YGGAGGYTQSPGGFGSPAPSQAEKKSRARAQ	1470500	0	1083
Q96L50	142	Leucine-rich repeat protein 1	LRR1	0.996697	0.025679	56.956	S	KTSEFENFKTKMVITSKKDYPLSKNFPYSLE	0	745310	5009
Q96L50	146	Leucine-rich repeat protein 1	LRR1	0.998132	0.025679	56.956	Y	FENFKTKMVITSKKDYPLSKNFPYSLEHLQT	0	745310	8410
P46937	366	Transcriptional coactivator YAP1	YAP1	0.540924	0.00740075	56.947	S	QLPTLEQDGGTQNPVSSPGMSQELRTMTTNS	1286800	0	1616
Q6PI73	169	Leukocyte immunoglobulin-like receptor subfamily A member 6	LILRA6	0.792273	0.0184861	56.766	S	VLMKEGEHQLPRTLDSQQLHSGGFQALFPVG	0	0	3542
Q6PI73	174	Leukocyte immunoglobulin-like receptor subfamily A member 6	LILRA6	0.792273	0.0184861	56.766	S	GEHQLPRTLDSQQLHSGGFQALFPVGPVTPS	0	0	3543
Q6PI73	189	Leukocyte immunoglobulin-like receptor subfamily A member 6	LILRA6	0.999998	0.0184861	56.766	S	SGGFQALFPVGPVTPSHRWRFTCYYYYTNTP	0	0	3544
Q8WXE0	396	Caskin-2	CASKIN2	0.610839	0.0168996	56.766	S	PLTYSQLPRVGLSPDSPAGDRNSVGSEGSVG	1382700	0	4585
Q8WXE0	406	Caskin-2	CASKIN2	0.88081	0.0168996	56.766	S	GLSPDSPAGDRNSVGSEGSVGSIRSAGSGQS	1382700	0	4587
Q8WXE0	409	Caskin-2	CASKIN2	0.976246	0.0168996	56.766	S	PDSPAGDRNSVGSEGSVGSIRSAGSGQSSEG	1382700	0	4588

Q6PI73	187	Leukocyte immunoglobulin-like receptor subfamily A member 6	LILRA6	0.999998	0.0184861	56.766	T	LHSGGFQALFPVGPVTPSHRWRFTCYYYYTN	0	0	7762
Q8N1G0	197	Zinc finger protein 687	ZNF687	0.455631	0.00120326	56.728	S	PSPTREGALTPPPFPSSFELAQENGPGMQPP	0	0	4223
Q8N1G0	198	Zinc finger protein 687	ZNF687	0.455631	0.00120326	56.728	S	SPTREGALTPPPFPSSFELAQENGPGMQPPV	0	0	4224
Q9UQ35	1455	Serine/arginine repetitive matrix protein 2	SRRM2	0.91628	0.0307551	56.72	S	RSGSSPGLRDGSGTPSRHSLSGSSPGMKDIP	5145800	305090	6609
Q9HD15	66	Steroid receptor RNA activator 1	SRA1	0.366724	0.0109147	56.719	S	KRVAAPQDGSPRVPASETSPGPPPMGPPPPS	0	0	5876
Q9Y666	30	Solute carrier family 12 member 7	SLC12A7	0.998489	0.0151446	56.719	T	DGGGDETAERTEAPGTPEGPEPERPSPGDGN	2123200	1543400	8315
P49356	3	Protein farnesyltransferase subunit beta	FNTB	0.475914	0.00318941	56.68	S	______________MASPSSFTYYCPPSSSPV	0	0	1655
P49356	5	Protein farnesyltransferase subunit beta	FNTB	0.475914	0.00318941	56.68	S	____________MASPSSFTYYCPPSSSPVWS	0	0	1656
Q9H6Z4	211	Ran-binding protein 3	RANBP3	0.756111	0.001559	56.68	S	GVSLPADCTGAVPAASPDTAAWRSPSEAADE	0	1636400	5742
Q12830	1677	Nucleosome-remodeling factor subunit BPTF	BPTF	0.464495	0.0098289	56.588	S	IETSEVKKVTSSPITSEEESNLSNDFIDENG	0	0	2316
Q12830	1676	Nucleosome-remodeling factor subunit BPTF	BPTF	0.464495	0.0098289	56.588	T	EIETSEVKKVTSSPITSEEESNLSNDFIDEN	0	0	7510
Q86XP3	742	ATP-dependent RNA helicase DDX42	DDX42	0.728227	0.00071807	56.547	T	GASGWTSAGSLNSVPTNSAQQGHNSPDSPVT	0	0	7833
P48730	356	Casein kinase I isoform delta	CSNK1D	0.46199	0.0131254	56.529	S	APPTPLTPTSHTANTSPRPVSGMERERKVSM	0	0	1633
P48730	355	Casein kinase I isoform delta	CSNK1D	0.46199	0.0131254	56.529	T	VAPPTPLTPTSHTANTSPRPVSGMERERKVS	0	0	7406
Q5JRA6	1682	Melanoma inhibitory activity protein 3	MIA3	0.926818	0.00335327	56.483	T	FGPSPVSGGECSPPLTVEPPVRPLSATLNRR	1391000	0	7700
Q9Y3B9	48	RRP15-like protein	RRP15	0.626594	0.0064162	56.461	S	SVLEDEATDTSDSEGSCGSEKDHFYSDDDAI	2424800	0	6852
P51784	948	Ubiquitin carboxyl-terminal hydrolase 11	USP11	0.786428	0.00690804	56.453	S	ARRLLSPAGSSGAPASPACSSPPSSEFMDVN	4481000	3301300	1795
Q9NRA8	587	Eukaryotic translation initiation factor 4E transporter	EIF4ENIF1	0.805959	0.00921881	56.416	S	QTRAASADYLRPRIPSPIGFTPGPQQLLGDP	743740	0	5953
Q07687	225	Homeobox protein DLX-2	DLX2	0.588679	0.00129608	56.383	S	MWKSGEIPSEQHPGASASPPCASPPVSAPAS	0	0	2232
P35611	613	Alpha-adducin	ADD1	0.608359	0.0159788	56.332	S	EKSPPDQPAVPHPPPSTPIKLEEDLVPEPTT	1125100	909020	1399
Q6ZRS2	262	Helicase SRCAP	SRCAP	0.473928	0.011055	56.332	S	LSQSLNQPLTSSKAGSSPCLGSSSAASSPPP	0	0	3655
Q6ZRS2	263	Helicase SRCAP	SRCAP	0.473385	0.011055	56.332	S	SQSLNQPLTSSKAGSSPCLGSSSAASSPPPP	0	0	3656
Q6ZRS2	273	Helicase SRCAP	SRCAP	0.750286	0.011055	56.332	S	SKAGSSPCLGSSSAASSPPPPASRLDDEDGD	661430	0	3657
Q9NPI6	180	mRNA-decapping enzyme 1A	DCP1A	0.697776	0.001365	56.294	S	KDEYERNQMGDSNISSPGLQPSTQLSNLGST	429380	0	5895
Q8N6N3	155	UPF0690 protein C1orf52	C1orf52	0.961757	0.0202009	56.215	T	PQNAKKARLLPEGEETLESDDEKDEHTSKKR	6244100	0	7879
Q96C57	179	Uncharacterized protein C12orf43	C12orf43	0.35896	0.0098058	56.017	S	AAVSASDILQESAIHSPGTVEKEAKKKRKLK	0	0	4821
Q96C57	182	Uncharacterized protein C12orf43	C12orf43	0.35896	0.0098058	56.017	T	SASDILQESAIHSPGTVEKEAKKKRKLKKKA	0	0	7971
Q96D09	542	G-protein coupled receptor-associated sorting protein 2	GPRASP2	0.602851	0.00287201	55.998	S	KPKNLELSPEGEEQESLLQPDQPSPEFTFQY	2224900	0	4836
Q15464	307	SH2 domain-containing adapter protein B	SHB	0.350204	0.00868317	55.994	S	KGIQLYDTPYEPEGQSVDSDSESTVSPRLRE	0	0	2935
Q15464	317	SH2 domain-containing adapter protein B	SHB	0.711376	0.00868317	55.994	S	EPEGQSVDSDSESTVSPRLRESKLPQDDDRP	832250	715000	2938
Q92820	59	Gamma-glutamyl hydrolase	GGH	0.699983	0.0124965	55.994	S	RNKVMKNYGRYYIAASYVKYLESAGARVVPV	0	0	4719
Q92820	66	Gamma-glutamyl hydrolase	GGH	0.987024	0.0124965	55.994	S	YGRYYIAASYVKYLESAGARVVPVRLDLTEK	0	0	4720
Q92820	51	Gamma-glutamyl hydrolase	GGH	0.815461	0.0124965	55.994	Y	IGILMQKCRNKVMKNYGRYYIAASYVKYLES	0	0	8403
Q92820	54	Gamma-glutamyl hydrolase	GGH	0.587789	0.0124965	55.994	Y	LMQKCRNKVMKNYGRYYIAASYVKYLESAGA	0	0	8404
Q92820	55	Gamma-glutamyl hydrolase	GGH	0.589134	0.0124965	55.994	Y	MQKCRNKVMKNYGRYYIAASYVKYLESAGAR	0	0	8405
P50552	322	Vasodilator-stimulated phosphoprotein	VASP	0.272252	0.00916227	55.988	S	RRPWEKNSTTLPRMKSSSSVTTSETQPCTPS	0	0	1751
P50552	323	Vasodilator-stimulated phosphoprotein	VASP	0.272252	0.00916227	55.988	S	RPWEKNSTTLPRMKSSSSVTTSETQPCTPSS	0	0	1752
P50552	324	Vasodilator-stimulated phosphoprotein	VASP	0.272252	0.00916227	55.988	S	PWEKNSTTLPRMKSSSSVTTSETQPCTPSSS	0	0	1753
P50552	325	Vasodilator-stimulated phosphoprotein	VASP	0.241823	0.00916227	55.988	S	WEKNSTTLPRMKSSSSVTTSETQPCTPSSSD	0	0	1754
Q9C0A6	865	SET domain-containing protein 5	SETD5	0.999134	0.0194057	55.988	S	PLSPVTPPPPNSGSKSPQLATPGSSHPGEEE	1597300	1173600	5507
Q96AC1	159	Fermitin family homolog 2	FERMT2	0.999942	0.00292773	55.895	S	PRDPTKKKKKKLDDQSEDEALELEGPLITPG	1757900	679570	4772
Q6P0Q8	1503	Microtubule-associated serine/threonine-protein kinase 2	MAST2	0.925523	0.00126633	55.846	S	RRESLQKQEAIREVDSSEDDTEEGPENSQGA	729680	603320	3501
Q6P0Q8	1504	Microtubule-associated serine/threonine-protein kinase 2	MAST2	0.925523	0.00126633	55.846	S	RESLQKQEAIREVDSSEDDTEEGPENSQGAQ	729680	603320	3502
Q9H3S1	185	Semaphorin-4A	SEMA4A	0.984306	0.0131731	55.844	T	MEGKGQSPFDPAHKHTAVLVDGMLYSGTMNN	0	1698000	8097
Q709C8	872	Vacuolar protein sorting-associated protein 13C	VPS13C	0.859163	0.00384454	55.783	S	TKGLLGTSLLLDTVESESDDEYFDAEDGEPQ	852010	348830	3677
Q709C8	874	Vacuolar protein sorting-associated protein 13C	VPS13C	0.720199	0.00384454	55.783	S	GLLGTSLLLDTVESESDDEYFDAEDGEPQTC	852010	348830	3678
Q13263	596	Transcription intermediary factor 1-beta	TRIM28	0.525559	0.00117053	55.779	S	LAEGPGAEGPRLASPSGSTSSGLEVVAPEGT	12879000	0	2453
Q13263	598	Transcription intermediary factor 1-beta	TRIM28	0.525559	0.00150835	55.779	S	EGPGAEGPRLASPSGSTSSGLEVVAPEGTSA	12879000	0	2454
Q96ST3	277	Paired amphipathic helix protein Sin3a	SIN3A	0.978882	0.00119533	55.767	S	ASQQTPPLPPYASPRSPPVQPHTPVTISLGT	1720300	429880	5141
Q9H7Z6	34	Histone acetyltransferase KAT8	KAT8	0.821719	0.000168492	55.767	T	GEGEPGPGENAAAEGTAPSPGRVSPPTPARG	3137900	0	8117
Q96GA3	331	Protein LTV1 homolog	LTV1	0.986039	0.00354526	55.765	S	EPLEDQDLPMNELDESEEEEMITVVLEEAKE	0	552480	4916
Q96S82	230	Ubiquitin-like protein 7	UBL7	0.997986	0.0101808	55.763	S	SSYRDMPGGFLFEGLSDDEDDFHPNTRSTPS	915280	519390	5116
Q9NW07	553	Zinc finger protein 358	ZNF358	0.49658	0.0132575	55.734	S	SPTRGTVSPALPTGESPEWVQEQGALLGPDG	0	0	6051
Q9NW07	550	Zinc finger protein 358	ZNF358	0.673548	0.0132575	55.734	T	DPCSPTRGTVSPALPTGESPEWVQEQGALLG	0	0	8172
P46937	164	Transcriptional coactivator YAP1	YAP1	0.5	0.0138084	55.718	S	SGPAATPTAQHLRQSSFEIPDDVPLPAGWEM	1023800	376220	1615
Q9H4L7	96	SWI/SNF-related matrix-associated actin-dependent regulator	SMARCAD1	0.783146	0.00993626	55.718	S	SYFKNQRGIQYIDLSSDSEDVVSPNCSNTVQ	2905000	1973500	5687
Q9H4L7	98	SWI/SNF-related matrix-associated actin-dependent regulator	SMARCAD1	0.919022	0.00993626	55.718	S	FKNQRGIQYIDLSSDSEDVVSPNCSNTVQEK	2905000	1973500	5688
Q70Z35	379	Phosphatidylinositol 3,4,5-trisphosphate-dependent Rac excha	PREX2	1	0.0292226	55.688	S	KGLKLGMEQDTWVMISEQGEKLYKMMCRQGN	697760	918070	3689
P05412	243	Transcription factor AP-1	JUN	0.783364	0.00936885	55.634	S	EPQTVPEMPGETPPLSPIDMESQERIKAERK	0	5006300	902
Q969V6	114	MKL/myocardin-like protein 1	MKL1	0.49588	0.00149923	55.629	S	IIVGQVNYPKVADSSSFDEDSSDALSPEQPA	0	0	4759
Q969V6	119	MKL/myocardin-like protein 1	MKL1	0.432004	0.00149923	55.629	S	VNYPKVADSSSFDEDSSDALSPEQPASHESQ	0	0	4760
Q969V6	120	MKL/myocardin-like protein 1	MKL1	0.432004	0.00149923	55.629	S	NYPKVADSSSFDEDSSDALSPEQPASHESQG	0	0	4761
Q8WWM7	602	Ataxin-2-like protein	ATXN2L	0.331496	0.000840956	55.608	S	LTSEPMGSPVSSKTESVSDKEDKPPLAPSGG	0	0	4566
Q8WWM7	604	Ataxin-2-like protein	ATXN2L	0.331496	0.000840956	55.608	S	SEPMGSPVSSKTESVSDKEDKPPLAPSGGTE	0	0	4567
Q8WWM7	600	Ataxin-2-like protein	ATXN2L	0.331496	0.000840956	55.608	T	GLLTSEPMGSPVSSKTESVSDKEDKPPLAPS	0	0	7925
Q86X10	377	Ral GTPase-activating protein subunit beta	RALGAPB	0.387914	0.0239782	55.536	S	PTPVNRLSMPQSAAVSTTPPHNRRHRAVTVN	0	0	3998
Q5T1M5	330	FK506-binding protein 15	FKBP15	0.404308	0.00693023	55.526	S	GADNLSADPVVSPPTSIPFKSGEPALRTKSN	0	0	3244
Q86YS7	281	C2 domain-containing protein 5	C2CD5	0.299829	0.0168327	55.453	S	KEIPFNEDPNPNTHSSGPSTPLKNQTYSFSP	0	0	4028
Q86YS7	284	C2 domain-containing protein 5	C2CD5	0.299829	0.0168327	55.453	S	PFNEDPNPNTHSSGPSTPLKNQTYSFSPSKS	0	0	4029
P12318;P:	315;321	Low affinity immunoglobulin gamma Fc region receptor II-a;Lo	FCGR2A;FCGI	0.99999	0.0270078	55.414	S	DKNIYLTLPPNDHVNSNN_____________	1966900	577080	1042
P24928	1962	DNA-directed RNA polymerase II subunit RPB1	POLR2A	0.627357	0.00258297	55.413	S	YSPTSPTYSLTSPAISPDDSDEEN_______	7528100	0	1240
Q9NRA8	213	Eukaryotic translation initiation factor 4E transporter	EIF4ENIF1	0.463239	0.00446831	55.406	S	FGDSKRVFGERRRNDSYTEEEPEWFSAGPTS	0	0	5955
P55884	78	Eukaryotic translation initiation factor 3 subunit B	EIF3B	0.305281	0.00038592	55.324	S	ESEVRTEPAAEAEAASGPSESPSPPAAEELP	0	0	1888
P55884	83	Eukaryotic translation initiation factor 3 subunit B	EIF3B	0.51224	0.00038592	55.324	S	TEPAAEAEAASGPSESPSPPAAEELPGSHAE	21491000	0	1890
P11142	637	Heat shock cognate 71 kDa protein	HSPA8	0.560338	0.00129505	55.314	S	PGGFPGGGAPPSGGASSGPTIEEVD______	0	0	1004
O75528	275	Transcriptional adapter 3	TADA3	0.569036	0.0103244	55.208	S	LTQRLLQALVEENIISPMEDSPIPDMSGKES	279410	546140	635
Q01831	346	DNA repair protein complementing XP-C cells	XPC	0.604411	0.0137177	55.206	S	GKKPSKERLTADPGGSSETSSQVLENHTKPK	0	0	2082
Q01831	347	DNA repair protein complementing XP-C cells	XPC	0.604391	0.0137177	55.206	S	KKPSKERLTADPGGSSETSSQVLENHTKPKT	0	0	2083
Q9UQ35	1198	Serine/arginine repetitive matrix protein 2	SRRM2	0.577503	0.0312946	55.186	S	QKDKFSPFPVQDRPESSLVFKDTLRTPPRER	0	0	6615
Q02880	1422	DNA topoisomerase 2-beta	TOP2B	0.420645	0.0119859	55.061	T	EDEFVPSDGLDKDEYTFSPGKSKATPEKSLH	0	0	7477
Q00577	127	Transcriptional activator protein Pur-alpha	PURA	0.999767	0.00695649	55.049	S	DYLGDFIEHYAQLGPSQPPDLAQAQDEPRRA	0	0	2024
Q14202	772	Zinc finger MYM-type protein 3	ZMYM3	0.468813	0.0019688	55.033	S	QPNLDTQSGPESLLNSQSPESKPQTPSQTKV	0	0	2705
O95785	1146	Protein Wiz	WIZ	0.999807	0.0122448	55.031	S	LHISPLAKKLPPPPGSPLGHSPTASPPPTAR	3956300	880040	811
Q9Y485	917	DmX-like protein 1	DMXL1	0.416836	0.02099	55.011	S	TKLSEAVWQPEEHYSSSPEKILSPFSQKYQA	0	0	6898
Q9Y485	918	DmX-like protein 1	DMXL1	0.466697	0.02099	55.011	S	KLSEAVWQPEEHYSSSPEKILSPFSQKYQAC	0	0	6899
Q15545	201	Transcription initiation factor TFIID subunit 7	TAF7	0.61401	0.021083	54.953	S	EDETKEAENQGLDISSPGMSGHRQGHDSLEH	0	1071400	2943
Q9C0C9	439	E2/E3 hybrid ubiquitin-protein ligase UBE2O	UBE2O	0.808751	0.000514809	54.926	S	KSEAESASPEETPDGSASPVEMQDEGAEEPH	1055000	325820	5538
Q13263	600	Transcription intermediary factor 1-beta	TRIM28	0.562795	0.00114765	54.904	S	PGAEGPRLASPSGSTSSGLEVVAPEGTSAPG	12879000	0	2455
Q96RU3	497	Formin-binding protein 1	FNBP1	0.455649	0.0214831	54.857	S	VEGRLPARSEQARRQSGLYDSQNPPTVNNCA	0	0	5104
Q3YEC7	464	Rab-like protein 6	RABL6	0.820693	0.00652468	54.745	S	DQPRGSPPLPAGPVPSQDITLSSEEEAEVAA	1141800	0	3082
Q86UE4	214	Protein LYRIC	MTDH	0.20406	0.00255403	54.703	S	REKRQQRKRDKVLTDSGSLDSTIPGIENTIT	0	0	3926
Q86UE4	212	Protein LYRIC	MTDH	0.20406	0.00255403	54.703	T	SHREKRQQRKRDKVLTDSGSLDSTIPGIENT	0	0	7824
Q86UP2	77	Kinectin	KTN1	0.544804	0.0253687	54.668	S	NKKKEIQNGNLHESDSESVPRDFKLSDALAV	271550	4180300	3933
Q9UJF2	634	Ras GTPase-activating protein nGAP	RASAL2	0.436081	0.0166861	54.66	S	PTPIQQQLRRFTEHNSSPNVSGSLSSGLQKI	0	0	6395
Q3SYG4	134	Protein PTHB1	BBS9	0.942077	0.0203846	54.658	T	NQCQMKLMYEHNLQRTACNMTYGSFGGVKGR	0	3256400	7671
Q9NVI1	1120	Fanconi anemia group I protein	FANCI	0.329636	0.0112915	54.559	S	KLKGQVSQETLSEEASSQATLPNQPVEKAII	0	0	6041
Q9NVI1	1121	Fanconi anemia group I protein	FANCI	0.329636	0.0112915	54.559	S	LKGQVSQETLSEEASSQATLPNQPVEKAIIM	0	0	6042
Q9NVI1	1124	Fanconi anemia group I protein	FANCI	0.329636	0.0112915	54.559	T	QVSQETLSEEASSQATLPNQPVEKAIIMQLG	0	0	8170
Q86WQ0	12	Nuclear receptor 2C2-associated protein	NR2C2AP	0.939247	0.0272262	54.539	S	____MTHSLVCPETVSRVSSVLNRNTRQFGK	0	2540900	3991
Q9H7N4	997	Splicing factor, arginine/serine-rich 19	SCAF1	0.354448	0.00272978	54.514	S	APPPPALTPDSQTVDSSCKTPEVSFLPEEAT	0	0	5762
Q9H7N4	998	Splicing factor, arginine/serine-rich 19	SCAF1	0.354448	0.00272978	54.514	S	PPPPALTPDSQTVDSSCKTPEVSFLPEEATE	0	0	5763
Q9H7N4	989	Splicing factor, arginine/serine-rich 19	SCAF1	0.904949	0.00272978	54.514	T	PSTPPPKAAPPPPALTPDSQTVDSSCKTPEV	0	3911100	8113
P21675	521	Transcription initiation factor TFIID subunit 1	TAF1	0.291826	0.00402479	54.466	T	DENLILEIPDEKEEATSNSPSKESKKESSLK	0	0	7313
P51003	526	Poly(A) polymerase alpha	PAPOLA	0.339338	0.00820115	54.419	S	EGVKLTALNDSSLDLSMDSDNSMSVPSPTSA	0	0	1770
P51003	529	Poly(A) polymerase alpha	PAPOLA	0.339376	0.00820115	54.419	S	KLTALNDSSLDLSMDSDNSMSVPSPTSATKT	0	0	1771
P51003	537	Poly(A) polymerase alpha	PAPOLA	0.528046	0.00820115	54.419	S	SLDLSMDSDNSMSVPSPTSATKTSPLNSSGS	1491500	0	1773
O43815	143	Striatin	STRN	0.379949	0.00217474	54.388	T	GDMKPPSYDSDEGNETEVQPQQNSQLMWKQG	0	0	7174
A0JNW5	1402	UHRF1-binding protein 1-like	UHRF1BP1L	0.699777	0.00646371	54.311	S	YDLKKQRSVTQATQTSPGVPWPSQSANFPEF	862450	590180	8
Q9NRA8	215	Eukaryotic translation initiation factor 4E transporter	EIF4ENIF1	0.343409	0.00530075	54.311	T	DSKRVFGERRRNDSYTEEEPEWFSAGPTSQS	0	0	8154
Q9BTC0	1244	Death-inducer obliterator 1	DIDO1	0.387009	0.00683425	54.3	Y	PKVATVPQSEKKPSKYPLCSADAAVSTTPPG	0	0	8419
O95251	124	Histone acetyltransferase KAT7	KAT7	0.629699	0.0024016	54.273	S	DFSDRETKNTADHDESPPRTPTGNAPSSESD	2389200	1769000	743
O95251	128	Histone acetyltransferase KAT7	KAT7	0.638052	0.0024016	54.273	T	RETKNTADHDESPPRTPTGNAPSSESDIDIS	2389200	1769000	7234
Q6ZXV5	437	Transmembrane and TPR repeat-containing protein 3	TMTC3	0.756723	0.0278425	54.253	S	FHRNWDWESEYTLFMSALKVNKNNAKLWNNV	0	3752600	3676
Q7Z401	1511	C-myc promoter-binding protein	DENND4A	0.562679	0.0186106	54.253	S	RDLRRPGRYFLKSSPSTENMHFPSSISSQTR	1798600	408880	3791
Q96JA1	1046	Leucine-rich repeats and immunoglobulin-like domains protein	LRIG1	0.755206	0.01346	54.225	S	PDSTELQPASSLTSGSPERAEAQYLLVSNGH	0	0	4955
P15336	272	Cyclic AMP-dependent transcription factor ATF-2	ATF2	0.403496	0.0117936	54.223	S	SVPGIPGPSSPQPVQSEAKMRLKAALTQQHP	0	0	1072
Q9BZ29	1338	Dedicator of cytokinesis protein 9	DOCK9	0.850633	0.0117936	54.223	S	WNKASTSELMDFFTISEVCLHQFQYMGKRYI	996670	0	5483
Q8TDH9	2	Biogenesis of lysosome-related organelles complex 1 subunit	BLOC1S5	0.699313	0.0305158	54.167	S	_______________MSGGGTETPVGCEAAPG	0	1533300	4468
Q8TDH9	20	Biogenesis of lysosome-related organelles complex 1 subunit	BLOC1S5	0.999701	0.0305158	54.167	S	GTETPVGCEAAPGGGSKKRDSLGTAGSAHLI	0	1533300	4469
Q8N1F8	470	Serine/threonine-protein kinase 11-interacting protein	STK11IP	0.768132	0.000638853	54.115	S	APPASSQGPDTAPRPSPPQEEARGPQESPQK	0	0	4220
Q7Z3K6	123	Mesoderm induction early response protein 3	MIER3	0.83961	0.00677704	54.096	S	IAKDLLSGDDEETQSSADDLTPSVTSHETSD	841210	523360	3786
O60784	154	Target of Myb protein 1	TOM1	0.996738	0.0215358	54.073	T	TIYEDLRRKGLEFPMTDLDMLSPIHTPQRTV	952380	0	7188
O60784	164	Target of Myb protein 1	TOM1	0.964578	0.0215358	54.073	T	LEFPMTDLDMLSPIHTPQRTVFNSETQSGQD	952380	902610	7189
Q15911	1588	Zinc finger homeobox protein 3	ZFHX3	0.734353	0.0236709	54.058	T	LKRALQESATGQPEPTSSPDNKPFKCNTCNV	577810	0	7654
Q99590	338	Protein SCAF11	SCAF11	0.845012	0.00802445	54.049	S	STRNTRAETASQSQRSPISDNSGCDAPGNSN	0	5104500	5222
Q99700	856	Ataxin-2	ATXN2	0.343042	0.00530877	54.049	S	KDSFIENSSSNCTSGSSKPNSPSISPSILSN	0	0	5248
O60784	160	Target of Myb protein 1	TOM1	0.923288	0.0240456	54.004	S	RRKGLEFPMTDLDMLSPIHTPQRTVFNSETQ	0	902610	520
Q9UBC2	800	Epidermal growth factor receptor substrate 15-like 1	EPS15L1	0.363608	0.0062468	53.968	S	APPRPKPPSGKSTPVSQLGSADFPEAPDPFQ	0	0	6262
P15924	2617	Desmoplakin	DSP	0.280376	0.0121793	53.964	S	LTIRSSSFSDTLEESSPIAAIFDTENLEKIS	0	0	1082
Q8WWM7	426	Ataxin-2-like protein	ATXN2L	0.258103	0.0163746	53.958	S	KAQRPLRGAKTLSSPSNRPSGETSVPPPPAV	0	0	4570
Q3ZCN5	1318	Otogelin-like protein	OTOGL	0.455543	0.0203898	53.768	S	HQGLWIPGYSAFELYSKKGFFIIFTDSSVKA	0	0	3087
Q7Z6Z7	3919	E3 ubiquitin-protein ligase HUWE1	HUWE1	0.999896	0.0278926	53.758	S	RESQLAHIKDEPPPLSPAPLTPATPSSLDPF	988000	854790	3869
Q6IBW4	240	Condensin-2 complex subunit H2	NCAPH2	0.838525	0.00078917	53.744	S	MEVSVCRSPVPALGFSQEPGPSPEGPMPLGG	3991800	672760	3470
O14654	700	Insulin receptor substrate 4	IRS4	0.907318	0.00105348	53.728	Y	GPHRARAFDEDEDDPYVPMRPGVATPLVSSS	2523200	1338800	8341
Q96D71	125	RalBP1-associated Eps domain-containing protein 1	REPS1	0.595477	0.0126034	53.679	S	SRHAASYSSDSENQGSYSGVIPPPPGRGQVK	0	822030	4842
Q13627	748	Dual specificity tyrosine-phosphorylation-regulated kinase 1	DYRK1A	0.928953	0.0166233	53.625	S	TEGHLTMRQGADREESPMTGVCVQQSPVASS	0	610930	2622
P43487	7	Ran-specific GTPase-activating protein	RANBP1	0.543876	0.0154388	53.615	T	_________MAAAKDTHEDHDTSTENTDESN	7468500	0	7381
Q92615	511	La-related protein 4B	LARP4B	0.337383	0.00300155	53.569	S	KNSFGYRKKREEKFTSSQTQSPTPPKPPSPS	0	0	4670
Q92615	512	La-related protein 4B	LARP4B	0.337383	0.00300155	53.569	S	NSFGYRKKREEKFTSSQTQSPTPPKPPSPSF	0	0	4671

Q92615	510	La-related protein 4B	LARP4B	0.337383	0.00300155	53.569	T	RKNSFGYRKKREEKFTSSQTQSPTPPKPPSP	0	0	7938
Q92615	514	La-related protein 4B	LARP4B	0.337351	0.00300155	53.569	T	FGYRKKREEKFTSSQTQSPTPPKPPSPSFEL	0	0	7939
O75376	913	Nuclear receptor corepressor 1	NCOR1	0.999968	0.0204284	53.553	S	MFPMDSKPSLLNPTGSILVSSPLKPNPLDLP	1649000	956180	599
O75376	917	Nuclear receptor corepressor 1	NCOR1	0.999968	0.0204284	53.553	S	DSKPSLLNPTGSILVSSPLKPNPLDLPQLQH	1649000	956180	600
O75376	918	Nuclear receptor corepressor 1	NCOR1	0.999968	0.0204284	53.553	S	SKPSLLNPTGSILVSSPLKPNPLDLPQLQHR	1649000	956180	601
Q8WV35	37	Leucine-rich repeat-containing protein 29	LRRC29	0.999346	0.0205223	53.553	S	AQALGCMHGAPSQLASLSLAHCSSLKSRPEL	1628700	0	4527
Q8WV35	39	Leucine-rich repeat-containing protein 29	LRRC29	0.99928	0.0205223	53.553	S	ALGCMHGAPSQLASLSLAHCSSLKSRPELEH	1628700	0	4528
Q8WV35	44	Leucine-rich repeat-containing protein 29	LRRC29	0.999879	0.0205223	53.553	S	HGAPSQLASLSLAHCSSLKSRPELEHQASGT	1628700	0	4529
Q8WV35	45	Leucine-rich repeat-containing protein 29	LRRC29	0.999875	0.0205223	53.553	S	GAPSQLASLSLAHCSSLKSRPELEHQASGTK	1628700	0	4530
Q9H4A3	2002	Serine/threonine-protein kinase WNK1	WNK1	0.270108	0.0159173	53.553	S	AVLPAVIPKKEKPELSEPSHLNGPSSDPEAA	0	0	5665
Q9H4A3	2005	Serine/threonine-protein kinase WNK1	WNK1	0.270108	0.0159173	53.553	S	PAVIPKKEKPELSEPSHLNGPSSDPEAAFLS	0	0	5666
Q9NQ88	165	Fructose-2,6-bisphosphatase TIGAR	TIGAR	0.443808	0.0205819	53.553	S	KEQFSQGSPSNCLETSLAEIFPLGKNHSSKV	0	0	5903
O75376	911	Nuclear receptor corepressor 1	NCOR1	0.999974	0.0204284	53.553	T	QRMFPMDSKPSLLNPTGSILVSSPLKPNPLD	1649000	956180	7199
Q9NQ88	164	Fructose-2,6-bisphosphatase TIGAR	TIGAR	0.443808	0.0205819	53.553	T	QKEQFSQGSPSNCLETSLAEIFPLGKNHSSK	0	0	8142
O60271	275	C-Jun-amino-terminal kinase-interacting protein 4	SPAG9	0.505273	0.0219122	53.475	T	QEDELSDVSQGGSKATTPASTANSDVATIPT	0	846710	7175
Q86VM9	604	Zinc finger CCCH domain-containing protein 18	ZC3H18	0.332601	0.0218113	53.444	S	HSSFSGSRSRSRSFSSSPSPSPTPSPHRPSI	0	0	3954
O95785	1155	Protein Wiz	WIZ	0.77791	0.0158686	53.361	S	LPPPPGSPLGHSPTASPPPTARKMFPGLAAP	0	0	813
P28290	1055	Sperm-specific antigen 2	SSFA2	0.682504	0.0102431	53.361	S	SPFRSSALMGMCGSRSADNLSCPSPLNVMEP	0	374300	1304
O14654	713	Insulin receptor substrate 4	IRS4	0.196704	0.00170933	53.32	S	DPYVPMRPGVATPLVSSSDYMPMAPQNVSAS	0	0	167
O14654	714	Insulin receptor substrate 4	IRS4	0.196704	0.00170933	53.32	S	PYVPMRPGVATPLVSSSDYMPMAPQNVSASK	0	0	168
O14654	715	Insulin receptor substrate 4	IRS4	0.196704	0.00170933	53.32	S	YVPMRPGVATPLVSSSDYMPMAPQNVSASKK	0	0	169
O14654	709	Insulin receptor substrate 4	IRS4	0.196704	0.00170933	53.32	T	EDEDDPYVPMRPGVATPLVSSSDYMPMAPQN	0	0	7127
Q9UK76	115	Hematological and neurological expressed 1 protein;Hematolog	HN1	0.811568	0.00108503	53.264	S	GEGDIHENVDTDLPGSLGQSEEKPVPAAPVP	431920	848830	6429
Q04637	1080	Eukaryotic translation initiation factor 4 gamma 1	EIF4G1	0.770069	0.0258233	53.255	S	DTSRLTKITKPGSIDSNNQLFAPGGRLSWGK	0	0	2159
Q07820	163	Induced myeloid leukemia cell differentiation protein Mcl-1	MCL1	0.660505	0.0009655	53.236	T	GESGNNTSTDGSLPSTPPPAEEEEDELYRQS	0	0	7503
P42694	1620	Probable helicase with zinc finger domain	HELZ	0.822569	0.0140208	53.184	S	LQYRQVQSRSPPAVPSPPSSTDHSSHFSNFN	4101200	0	1517
O43379	1047	WD repeat-containing protein 62	WDR62	0.333334	0.00142583	53.094	S	PTEDELSLPEGPSVPSSSLPQTPEQEKFLRH	0	0	365
Q8IZL8	743	Proline-, glutamic acid- and leucine-rich protein 1	PELP1	0.527421	0.0105791	53.089	S	ENHRAGSNEDPILAPSGTPPPTIPPDETFGG	5164900	0	4185
O00139	78	Kinesin-like protein KIF2A	KIF2A	0.918168	0.00425255	53.064	T	NPDLVPDEEIEPSPETPPPPASSAKVNKIVK	5293600	2056700	7119
Q96JM3	344	Chromosome alignment-maintaining phosphoprotein 1	CHAMP1	0.65302	0.0191437	52.97	S	ASSGPWKPAKPAPSVSPGPWKPIPSVSPGPW	905240	0	4985
Q96JM3	355	Chromosome alignment-maintaining phosphoprotein 1	CHAMP1	0.634136	0.0191437	52.97	S	APSVSPGPWKPIPSVSPGPWKPTPSVSSASW	905240	0	4986
Q9BYW2	685	Histone-lysine N-methyltransferase SETD2	SETD2	0.537163	0.0211525	52.898	T	ELNINGSPGAESDLATFCTSKTDAVLMTSDD	0	0	8068
P52948	625	Nuclear pore complex protein Nup98-Nup96;Nuclear pore comple	NUP98	0.411865	0.0282188	52.887	S	LFSPVNRDSENLASPSEYPENGERFSFLSKP	0	0	1825
O95785	1151	Protein Wiz	WIZ	0.891976	0.021886	52.824	S	LAKKLPPPPGSPLGHSPTASPPPTARKMFPG	1678900	50226	812
Q08211	92	ATP-dependent RNA helicase A	DHX9	0.513128	0.000831199	52.812	T	SEEVPAFGVASPPPLTDTPDTTANAEGDLPT	1567500	0	7504
Q7LBC6	730	Lysine-specific demethylase 3B	KDM3B	0.257576	0.00268501	52.777	S	PSLSAMGNGRSSSPTSSLTQPIEMPTLSSSP	0	0	3739
Q7LBC6	731	Lysine-specific demethylase 3B	KDM3B	0.257576	0.00268501	52.777	S	SLSAMGNGRSSSPTSSLTQPIEMPTLSSSPT	0	0	3740
Q7LBC6	729	Lysine-specific demethylase 3B	KDM3B	0.454577	0.00268501	52.777	T	GPSLSAMGNGRSSSPTSSLTQPIEMPTLSSS	0	0	7793
Q7LBC6	751	Lysine-specific demethylase 3B	KDM3B	0.499726	0.00268501	52.777	T	IEMPTLSSSPTEERPTVGPGQQDNPLLKTFS	0	0	7796
P07199	150	Major centromere autoantigen B	CENPB	0.499667	0.0023964	52.773	T	CSGVARARARNAAPRTPAAPASPAAVPSEGS	0	0	7265
Q9UER7	169	Death domain-associated protein 6	DAXX	0.562515	0.00253664	52.722	T	EPSGNNPPTHLSLDPTNAENTASQSPRTRGS	0	3346400	8204
O00139	75	Kinesin-like protein KIF2A	KIF2A	0.935358	0.00425255	52.669	S	FSLNPDLVPDEEIEPSPETPPPPASSAKVNK	2397400	2056700	90
Q9NQW6	95	Actin-binding protein anillin	ANLN	0.504292	0.00503776	52.669	S	SNLENKQPVESTSAKSCSPSPVSPQVQPQAA	1720500	0	5930
Q9NQW6	97	Actin-binding protein anillin	ANLN	0.504292	0.00503776	52.669	S	LENKQPVESTSAKSCSPSPVSPQVQPQAADT	1720500	0	5931
Q9NQW6	102	Actin-binding protein anillin	ANLN	0.63193	0.00503776	52.669	S	PVESTSAKSCSPSPVSPQVQPQAADTISDSV	0	1388000	5933
Q9UBS0	11	Ribosomal protein S6 kinase beta-2	RPS6KB2	0.786799	0.00322468	52.642	T	______MAAVFDLDLETEEGSEGEGEPELSPA	0	621790	8200
O75385	477	Serine/threonine-protein kinase ULK1	ULK1	1	0.0308295	52.599	S	IRRSGSTSPLGFARASPSPPAHAEHGGVLAR	2687500	1400900	619
O75385	479	Serine/threonine-protein kinase ULK1	ULK1	1	0.0308295	52.599	S	RSGSTSPLGFARASPSPPAHAEHGGVLARKM	2687500	1400900	620
P33240	518	Cleavage stimulation factor subunit 2	CSTF2	0.42197	0.00269195	52.575	S	VMQGTGMQGASIQGGSQPGGFSPGQNQVTPQ	0	0	1362
Q9BT92	469	Trichoplein keratin filament-binding protein	TCHP	0.987804	0.0252162	52.509	S	QEEEEEEEARRVEQLSDALLQQEAETMAEQG	0	0	5348
Q9BT92	485	Trichoplein keratin filament-binding protein	TCHP	0.999903	0.0252162	52.509	Y	DALLQQEAETMAEQGYRPKPYGHPKIAWN__	0	0	8418
P46100	92	Transcriptional regulator ATRX	ATRX	0.495218	0.00382948	52.465	S	SRSKRKPSIVTKYVESDDEKPLDDETVNEDA	0	0	1559
Q9ULH0	1590	Kinase D-interacting substrate of 220 kDa	KIDINS220	0.472267	0.0173744	52.432	S	DALLDKKDSSDSGVRSSESSPNHSLHNEVAD	0	0	6471
Q9ULH0	1591	Kinase D-interacting substrate of 220 kDa	KIDINS220	0.512126	0.0173744	52.432	S	ALLDKKDSSDSGVRSSESSPNHSLHNEVADD	1061300	765280	6472
Q9ULH0	1607	Kinase D-interacting substrate of 220 kDa	KIDINS220	0.357449	0.0173744	52.432	S	ESSPNHSLHNEVADDSQLEKANLIELEDDSH	0	0	6473
Q5JU85	528	IQ motif and SEC7 domain-containing protein 2	IQSEC2	0.944371	0.00981164	52.377	S	FSDLPLYLDDTVPQQSPERLPSTEPPPQGRP	432280	668810	3202
Q9BQG0	1186	Myb-binding protein 1A	MYBBP1A	0.713951	0.0221755	52.366	S	KKGFLPETKKRKKRKSEDGTPAEDGTPAATG	0	0	5310
Q15386	427	Ubiquitin-protein ligase E3C	UBE3C	0.949332	0.0256304	52.359	S	VWRDSASEEVFTTMASVCHTLMVQHRMMVPK	0	7575700	2910
Q96JM3	181	Chromosome alignment-maintaining phosphoprotein 1	CHAMP1	0.295099	0.000967197	52.342	S	ELQTPLPSPEPSKPASVSSPEPPKSVPVCES	0	0	4987
Q96JM3	183	Chromosome alignment-maintaining phosphoprotein 1	CHAMP1	0.295099	0.000967197	52.342	S	QTPLPSPEPSKPASVSSPEPPKSVPVCESQK	0	0	4988
Q96JM3	173	Chromosome alignment-maintaining phosphoprotein 1	CHAMP1	0.897229	0.000967197	52.342	S	KPGSVVSPELQTPLPSPEPSKPASVSSPEPP	1332900	921800	4991
Q9H7N4	1001	Splicing factor, arginine/serine-rich 19	SCAF1	0.732486	0.00362355	52.326	T	PALTPDSQTVDSSCKTPEVSFLPEEATEEAG	3141700	0	8115
Q7Z2Z1	1352	Treslin	TICRR	0.243158	0.020787	52.321	S	ELDQKEPQMSPSVAASLSCPVPSTPPELSQR	0	0	3759
Q9BYW2	676	Histone-lysine N-methyltransferase SETD2	SETD2	0.791919	0.0143365	52.321	S	DQLRSFCPIELNINGSPGAESDLATFCTSKT	529130	707940	5476
O60244	986	Mediator of RNA polymerase II transcription subunit 14	MED14	0.931234	0.00308088	52.237	S	QDARRRSVNEDDNPPSPIGGDMMDSLISQLQ	3680500	3759000	447
P04049	260	RAF proto-oncogene serine/threonine-protein kinase	RAF1	0.402479	0.0220057	52.185	T	PSSEGSLSQRQRSTSTPNVHMVSTTLPVDSR	0	0	7255
Q6W2J9	1439	BCL-6 corepressor	BCOR	0.398059	0.0145749	52.162	S	QLLPASQSTQLPCSSSPQETTQSRPMPPEAR	0	0	3616
P85037	288	Forkhead box protein K1	FOXK1	0.352643	0.00269739	52.106	S	TSDLQLAAEFAAKAASEQQADTSGGDSPKDE	0	0	1988
P85037	295	Forkhead box protein K1	FOXK1	0.709324	0.00269739	52.106	S	AEFAAKAASEQQADTSGGDSPKDESKPPFSY	418010	182070	1989
P85037	294	Forkhead box protein K1	FOXK1	0.322384	0.00269739	52.106	T	AAEFAAKAASEQQADTSGGDSPKDESKPPFS	0	0	7464
P78312	976	Protein FAM193A	FAM193A	0.496676	0.0143662	52.054	T	SVPNSGNIHNGSLEQTEEPETSSHSPSRHMN	0	0	7458
Q14694	218	Ubiquitin carboxyl-terminal hydrolase 10	USP10	0.418601	0.0124648	51.963	S	SVTPRTCNSPQNSTDSVSDIVPDSPFPGALG	0	0	2804
Q14694	220	Ubiquitin carboxyl-terminal hydrolase 10	USP10	0.488549	0.0124648	51.963	S	TPRTCNSPQNSTDSVSDIVPDSPFPGALGSD	0	0	2805
Q8NCF5	385	NFATC2-interacting protein	NFATC2IP	0.999935	0.0231373	51.956	S	PLKTLMSHYEEAMGLSGRKLSFFFDGTKLSG	0	0	4327
Q8NCF5	373	NFATC2-interacting protein	NFATC2IP	0.481799	0.0231373	51.956	T	HQTLEVSLSRDSPLKTLMSHYEEAMGLSGRK	0	0	7886
Q8NCF5	378	NFATC2-interacting protein	NFATC2IP	0.998857	0.0231373	51.956	Y	VSLSRDSPLKTLMSHYEEAMGLSGRKLSFFF	0	0	8395
P78347	8	General transcription factor II-I	GTF2I	0.520098	0.0178435	51.888	T	________MAQVAMSTLPVEDEESSESRMVV	0	182410	7460
P85037	299	Forkhead box protein K1	FOXK1	0.770993	0.00210471	51.868	S	AKAASEQQADTSGGDSPKDESKPPFSYAQLI	418010	182070	1990
P85037	304	Forkhead box protein K1	FOXK1	0.583417	0.00210471	51.868	S	EQQADTSGGDSPKDESKPPFSYAQLIVQAIS	0	0	1991
Q8IXJ6	364	NAD-dependent protein deacetylase sirtuin-2	SIRT2	0.292548	0.016052	51.777	S	HASIDAQSGAGVPNPSTSASPKKSPPPAKDE	0	0	4098
P54259	746	Atrophin-1	ATN1	0.984024	0.027952	51.645	S	AEEYETPESPVPPARSPSPPPKVVDVPSHAS	1380300	534970	1845
P54259	748	Atrophin-1	ATN1	0.99477	0.027952	51.645	S	EYETPESPVPPARSPSPPPKVVDVPSHASQS	1380300	534970	1846
Q8IYL3	189	UPF0688 protein C1orf174	C1orf174	0.988495	0.00298556	51.617	S	KPPLQMDNSVFLDDDSNQPMPVSRFFGNVEL	4341100	2075300	4177
Q5VV52	39	Zinc finger protein 691	ZNF691	1	0.0293067	51.591	S	PLSSEGSEMGSEKEQSPEPHLPEEGEGGKPW	0	0	3354
O75717	323	WD repeat and HMG-box DNA-binding protein 1	WDHD1	0.721719	0.0180807	51.568	Y	GKTSSSKVSSRVEKDYNDLFDGDDMSNAGDF	0	2520000	8349
O43815	378	Striatin	STRN	0.23516	0.0222764	51.566	S	DVDELPSLQPSVGSPSRPSSSRLPEHEINRA	0	0	420
Q6W2J9	1290	BCL-6 corepressor	BCOR	0.763939	0.0197258	51.566	S	LKPSDNEQGLPVFSGSPPMKSLSSTSAGGKK	0	693000	3622
Q6WKZ4	206	Rab11 family-interacting protein 1	RAB11FIP1	0.741512	0.0143219	51.554	S	AIIPSTTPSVDSDDESVVKDKKKKSKIKTLL	0	0	3636
Q15154	1765	Pericentriolar material 1 protein	PCM1	0.989245	0.0194582	51.475	S	QTQTSEVYDGPKNVRSDISDQEEDEESEGCP	1250700	1659400	2879
Q15154	1768	Pericentriolar material 1 protein	PCM1	0.991094	0.0194582	51.475	S	TSEVYDGPKNVRSDISDQEEDEESEGCPVSI	1250700	1659400	2880
Q9UHB7	1075	AF4/FMR2 family member 4	AFF4	0.438959	0.0125734	51.473	S	KLSPGNSGNYSSGASSASASGSSVTIPQKIH	0	0	6343
Q9Y4B4	1195	Helicase ARIP4	RAD54L2	0.354054	0.00534051	51.469	S	QQYADVAAARESRQSSPSTNAALPGPPAQLM	0	0	6904
Q9Y4B4	1197	Helicase ARIP4	RAD54L2	0.354054	0.00534051	51.469	S	YADVAAARESRQSSPSTNAALPGPPAQLMDS	0	0	6905
Q9UN72;Q(	303;303	Protocadherin alpha-7;Protocadherin alpha-9	PCDHA7;PCDI	0.982641	0.0279453	51.348	T	DIKSKFHMDPLSGAITVIGHMDFEESRAHKI	7657500	3239200	8245
Q8NOY2	235	Zinc finger protein 444	ZNF444	0.999113	0.0262137	51.265	S	EHLRRHRDTHPGSPGSPGPALRPLPAREKPH	0	1351500	4195
Q8TF74	235	WAS/WASL-interacting protein family member 2	WIPF2	1	0.02999	51.265	S	GREGPPAPPPVKPPPSPVNIRTGPSGQSLAP	0	0	4498
Q99700	848	Ataxin-2	ATXN2	0.330577	0.00552506	51.236	S	KDKIEPSAKDSFIENSSSNCTSGSSKPNSPS	0	0	5244
Q16204	386	Coiled-coil domain-containing protein 6	CCDC6	0.352274	0.0244339	51.234	T	SYASHTVGFTPPTSLTRAGMSYYNSPGLHVQ	0	0	7656
Q86VM9	603	Zinc finger CCCH domain-containing protein 18	ZC3H18	0.786817	0.0263581	51.193	S	RHSSFSGSRSRSRSFSSSPSPSPTPSPHRPS	2341200	645670	3953
O14686	3199	Histone-lysine N-methyltransferase 2D	KMT2D	0.850521	0.0134818	51.19	S	SFGATGGPPAHLLTPSPLSGPGGSSLLEKFE	300150	422450	189
O14686	3188	Histone-lysine N-methyltransferase 2D	KMT2D	0.734727	0.0134818	51.19	T	FSSSGHTAEKASFGATGGPPAHLLTPSPLSG	300150	422450	7130
Q9H9J4	1222	Ubiquitin carboxyl-terminal hydrolase 42	USP42	0.59851	0.029334	51.127	S	KHRDRDSRHQQDSDLSAACSDADLHRHKKKK	0	0	5804
P15923	379	Transcription factor E2-alpha	TCF3	0.775311	0.0197099	51.089	S	AGTSQWPRAGAPGALSPSYDGGLHGLQSKIE	0	0	1074
P54727	159	UV excision repair protein RAD23 homolog B	RAD23B	0.408832	0.0214336	50.986	T	KQEKPAEKPAETPVATSPTATDSTSGDSSRS	0	0	7448
Q5SW79;Q(	1521;230	Centrosomal protein of 170 kDa;Cep170-like protein	CEP170;CEP:	0.800711	0.0199054	50.87	S	MGFPSAMLPSPPKQKSSPVNNHHSPGQTPTL	0	9852700	3222
Q9Y3L3	598	SH3 domain-binding protein 1	SH3BP1	0.780841	0.0139885	50.87	S	SKSSPPAPPLPPGSGSPGTPQALPRRLVGSS	692820	0	6860
Q8WYQ5	367	Microprocessor complex subunit DGCR8	DGCR8	0.537128	0.0270143	50.869	S	CLHYKKMKDNEEREQSSDLTPSGDVSPVKPL	0	2110500	4610
P23246	379	Splicing factor, proline- and glutamine-rich	SFPQ	0.811881	0.0174512	50.827	S	VRFATHAAALSVRNLSPYVSNELLEEAFSQF	0	0	1219
Q92610	1262	Zinc finger protein 592	ZNF592	0.724262	0.00895658	50.802	T	NDHSQPQASQDQDSHTLSPQV__________	0	884970	7937
P67809	36	Nuclease-sensitive element-binding protein 1	YBX1	0.718156	0.0166425	50.783	S	SAADTKPGTTGSGAGSGGPGGLTSAAPAGGD	770390	475220	1941
P17544	255	Cyclic AMP-dependent transcription factor ATF-7	ATF7	0.363104	0.00463805	50.767	S	PNIPGIPGPPVNSSGISIPSPGHIPSEAKMR	0	0	1112
P17544	257	Cyclic AMP-dependent transcription factor ATF-7	ATF7	0.363104	0.00463805	50.767	S	IPGIPGPPVNSSGISIPSPGHIPSEAKMRLK	0	0	1113
Q8WUZ0	111	B-cell CLL/lymphoma 7 protein family member C	BCL7C	0.654746	0.0122752	50.757	T	NSNQSFHSEGSLQKGTEPSPGGTPQPSRPVS	0	0	7916
P85037	309	Forkhead box protein K1	FOXK1	0.371989	0.00311265	50.706	S	TSGGDSPKDESKPPFSYAQLIVQAISSAQDR	0	0	1992
Q9HCM4	436	Band 4.1-like protein 5	EPB41L5	0.571039	0.00688209	50.705	S	ISSAPVPVEIENLPQSPGTDQHDRKCIPLNI	1093200	983710	5872
O15047	534	Histone-lysine N-methyltransferase SETD1A	SETD1A	0.688666	0.00163953	50.645	S	MVLGARDTGSEVPSGSGHGPCTPPPAPANFE	0	0	264
O15047	565	Histone-lysine N-methyltransferase SETD1A	SETD1A	0.463495	0.00163953	50.645	S	DVAPTGSGEPGATRESPKANGQNQASPCSSG	0	0	265
O15047	562	Histone-lysine N-methyltransferase SETD1A	SETD1A	0.463495	0.00163953	50.645	T	NFEDVAPTGSGEPGATRESPKANGQNQASPC	0	0	7147
Q7LBC6	725	Lysine-specific demethylase 3B	KDM3B	0.209786	0.00271912	50.627	S	LTSGGPSLSAMGNGRSSSPTSSLTQPIEMPT	0	0	3736
Q7LBC6	726	Lysine-specific demethylase 3B	KDM3B	0.209786	0.00271912	50.627	S	TSGGPSLSAMGNGRSSSPTSSLTQPIEMPTL	0	0	3737
Q7LBC6	727	Lysine-specific demethylase 3B	KDM3B	0.209786	0.00271912	50.627	S	SGGPSLSAMGNGRSSSPTSSLTQPIEMPTLS	0	0	3738
Q7LBC6	744	Lysine-specific demethylase 3B	KDM3B	0.397105	0.00271912	50.627	S	TSSLTQPIEMPTLSSSPTEERPTVGPGQQDN	0	0	3743
Q7LBC6	733	Lysine-specific demethylase 3B	KDM3B	0.133418	0.00271912	50.627	T	SAMGNGRSSSPTSSLTQPIEMPTLSSSPTEE	0	0	7794
Q7LBC6	746	Lysine-specific demethylase 3B	KDM3B	0.397105	0.00271912	50.627	T	SLTQPIEMPTLSSSPTEERPTVGPGQQDNPL	0	0	7795
O43815	134	Striatin	STRN	0.28784	0.00330765	50.583	S	LKYGTELNQGDMKPPSYDSDEGNETEVQPQQ	0	0	424
O75022	530	Leukocyte immunoglobulin-like receptor subfamily B member 3	LILRB3	0.974449	0.019857	50.498	S	AAVKDTQSEDRVELDSQSPHDEDPQAVTYAP	0	11355000	544
O75022	532	Leukocyte immunoglobulin-like receptor subfamily B member 3	LILRB3	0.95561	0.019857	50.498	S	VKDTQSEDRVELDSQSPHDEDPQAVTYAPVK	0	11355000	545
Q07666	58	KH domain-containing, RNA-binding, signal transduction-assoc	KHDRBS1	0.497718	0.00143452	50.479	S	RSRGGGGSRGGARASPATQPPPLLPPSATG	0	0	2228
Q07666	61	KH domain-containing, RNA-binding, signal transduction-assoc	KHDRBS1	0.497718	0.00143452	50.479	T	GGGGSRGGARASPATQPPPLLPPSATGPDA	0	0	7502
Q9BS31	384	Zinc finger protein 649	ZNF649	0.977653	0.0286219	50.387	S	TGKTPFVCPECGQPCSQKSGLIRHQKIHSGE	1955800	1730800	5339
O14628	340	Zinc finger protein 195	ZNF195	0.782873	0.0285342	50.358	S	EHYRCEEFGKVFNQCSHLTEHEHGTEEKPCK	0	1533300	159
Q12872	604	Splicing factor, suppressor of white-apricot homolog	SFSWAP	0.5919	0.00263676	50.35	S	DLLPLEKNRVKLDDDSDDDEESKEGQESSSS	738190	0	2321
Q92598	552	Heat shock protein 105 kDa	HSPH1	0.416424	0.00238963	50.341	S	EAGTQPQVQTDAQQTSQSPPSPELTSEENKI	0	0	4653
Q14789	3010	Golgin subfamily B member 1	GOLGB1	0.863036	0.0178949	50.323	S	SSQTQPLKVQYQRQASPETSASPDGSQNLVY	361460	0	2810
Q14160	1348	Protein scribble homolog	SCRIB	0.627419	0.00879057	50.224	S	PEDAPAQPPTPGPAASPEQLSFRERQKYFEL	1775900	880060	2674
O95359	2567	Transforming acidic coiled-coil-containing protein 2	TACC2	0.190474	0.0177879	50.202	S	DTSQESPVKSSPVRMSESPTPCSGSSFEETE	0	0	750
O95359	2569	Transforming acidic coiled-coil-containing protein 2	TACC2	0.190474	0.0177879	50.202	S	SQESPVKSSPVRMSESPTPCSGSSFEETEAL	0	0	751
O95359	2576	Transforming acidic coiled-coil-containing protein 2	TACC2	0.190474	0.0177879	50.202	S	SSPVRMSESPTPCSGSSFEETEALVNTAAKN	0	0	752
O95359	2577	Transforming acidic coiled-coil-containing protein 2	TACC2	0.190474	0.0177879	50.202	S	SPVRMSESPTPCSGSSFEETEALVNTAAKNQ	0	0	753

O95359	2571	Transforming acidic coiled-coil-containing protein 2	TACC2	0.190474	0.0177879	50.202	T	ESPVKSSPVRMSESPTPCSGSSFEETEALVN	0	0	7236
Q96HW7	600	Integrator complex subunit 4	INTS4	0.449937	0.0162414	50.189	S	PALRLPGRKLVSSAVSPSIIPQEDPSQQFLQ	0	0	4947
Q96HW7	602	Integrator complex subunit 4	INTS4	0.449937	0.0162414	50.189	S	LRLPGRKLVSSAVSPSIIPQEDPSQQFLQQS	0	0	4948
Q03164	142	Histone-lysine N-methyltransferase 2A;MLL cleavage product	KMT2A	0.884623	0.0152862	50.14	S	RRFRAVFGESGGGGGSGEDEQFLGFGSDEEV	0	0	2136
Q03164	153	Histone-lysine N-methyltransferase 2A;MLL cleavage product	KMT2A	0.999862	0.0152862	50.14	S	GGGGSGEDEQFLGFGSDEEVRVRSPTRSPSV	0	0	2137
Q92610	1260	Zinc finger protein 592	ZNF592	0.42184	0.0061592	49.967	S	GHNDHSQPQASQDQDSHTLSPQV________	0	0	4659
Q92610	1264	Zinc finger protein 592	ZNF592	0.42184	0.0061592	49.967	S	HSQPQASQDQDSHTLSPQV____________	0	0	4660
P46821	1376	Microtubule-associated protein 1B;MAP1B heavy chain;MAP1 li	MAP1B	0.538686	0.0277385	49.871	S	VSFEFSDAKDENERASVSPMDEPVPDSESPI	0	633170	1570
Q9HCK8	2200	Chromodomain-helicase-DNA-binding protein 8	CHD8	0.447477	0.00545031	49.744	S	MVTGGILGPGNHLLDSPSLTPGEYGDSPVPT	0	0	5869
Q9HCK8	2208	Chromodomain-helicase-DNA-binding protein 8	CHD8	0.463141	0.00545031	49.744	Y	PGNHLLDSPSLTPGEYGDSPVPTPRSSSAAS	0	0	8427
Q9H0E3	300	Histone deacetylase complex subunit SAP130	SAP130	0.99186	0.0235428	49.708	S	IPPAVVATVSATRAQSPVITTTAAHATDSAL	0	477080	5576
Q9H792	779	Pseudopodium-enriched atypical kinase 1	PEAK1	0.959885	0.0170175	49.638	S	STVLSQIVASIQPPQSPPETPQSGPKACSVE	1326700	609990	5750
Q9NQZ2	37	Something about silencing protein 10	UTP3	0.773745	0.00289802	49.626	S	PTLTDENGDDLGLPPSPGDTSYYQDQVDDFH	795890	512570	5939
Q03468;P(9;9		DNA excision repair protein ERCC-6	ERCC6	0.706657	0.00887649	49.608	S	_______MPNEGIPHSSQTQEQDCLQSQPVS	0	0	2152
Q5JTC6	286	APC membrane recruitment protein 1	AMER1	0.939765	0.026258	49.595	S	QPKPAPEASSLEEPHSPETGEKVVAGEVNPP	1231000	845060	3194
Q13263	599	Transcription intermediary factor 1-beta	TRIM28	0.503232	0.00233605	49.57	T	GPGAEGPRLASPSGSTSSGLEVVAPEGTSAP	12879000	0	7530
Q8WYB5	1502	Histone acetyltransferase KAT6B	KAT6B	1	0.0190275	49.559	S	SEPKELAGDPEAVPESDEEPPPGEQAQKQDQ	262470	316640	4603
Q9NZT2	378	Opioid growth factor receptor	OGFR	1	0.030378	49.537	S	GDEAGGHGEDRPEPLSPKESKKRKLELSRRE	1876200	560700	6177
Q86U42	95	Polyadenylate-binding protein 2	PABPN1	0.782765	0.000920665	49.518	S	PPGAPGPGPGSGAPGSQEEEEEPGLVEGDPG	0	0	3915
Q13542	86	Eukaryotic translation initiation factor 4E-binding protein	EIF4EBP2	0.287294	0.0144368	49.486	T	PPCHLPNIPGVTSPGTLIEDSKVEVNNLNNL	0	0	7559
Q8WUI4	493	Histone deacetylase 7	HDAC7	0.694729	0.022441	49.34	S	HRPLSRAQSSPAAPASLSAPEPASQARVLSS	4247700	2016200	4517
P17544	259	Cyclic AMP-dependent transcription factor ATF-7	ATF7	0.264657	0.00533155	49.199	S	GIPGPPVNSSGSISPSGHPIPSEAKMRLKAT	0	0	1114
Q07687	236	Homeobox protein DLX-2	DLX2	0.658994	0.00553778	49.187	S	HPGASASPPCASPPVSAPASWDFGVPQRMAG	0	1042500	2235
Q8N1G1	287	RNA exonuclease 1 homolog	REXO1	0.974	0.0214308	49.141	S	PKKLCDPFGSCDARFSDSEDEAATVPGNEPT	0	1213600	4234
Q8N1G1	289	RNA exonuclease 1 homolog	REXO1	0.978569	0.0214308	49.141	S	KLCDPFGSCDARFSDSEDEAATVPGNEPTTA	0	1213600	4235
Q13439	122	Golgin subfamily A member 4	GOLGA4	0.905926	0.00792997	49.095	S	LDSSTASFDPPSDMDSEAEDLVGNSDSLNKE	0	726770	2530
Q9H4Z3	26	Phosphorylated CTD-interacting factor 1	PCIF1	0.538703	0.0242487	49.09	S	EEASLLSHSPGTSNQSQPCSPKPIRLVQDLP	807720	604770	5700
Q9H4Z3	22	Phosphorylated CTD-interacting factor 1	PCIF1	0.209284	0.0242487	49.09	T	GSPREEASLLSHSPGTSNQSQPCSPKPIRLV	0	0	8102
Q86VM9	607	Zinc finger CCCH domain-containing protein 18	ZC3H18	0.357134	0.0306902	49.049	S	FSGSRSRSRSFSSSPSPSPTPSPHRPSIRTK	0	0	3955
Q9UKX7	291	Nuclear pore complex protein Nup50	NUP50	0.389037	0.0211428	48.958	T	KVDSSVLGSLSSVPLTGFSFSPGNSSLFGKD	0	0	8236
Q7L014	294	Probable ATP-dependent RNA helicase DDX46	DDX46	0.680826	0.00838296	48.947	Y	KKKGELMENDQDAMEYSSEEEEVDLQTALTG	1158500	0	8392
Q92614	83	Unconventional myosin-XVIIIa	MYO18A	0.7003	0.0311767	48.729	S	IKVASGSDLHLTDIDSDSNRGSVILDSGHLS	1524600	1491800	4666
Q9P2C4	588	Transmembrane protein 181	TMEM181	0.491565	0.00685271	48.694	S	AFSMLNDSDDDVIYGSDYEEMPLQNGQAIRA	0	0	6231
Q96FH0	104	Protein MEF2BNB	MEF2BNB	0.776334	0.0234777	48.677	S	GLLKQAISIRDHMNASAQGHSPEEPPPPSSA	0	0	4905
Q13501	355	Sequestosome-1	SQSTM1	0.659995	0.00598165	48.672	S	HLSSKEVDPSTGELQSLQMPESEGPSSLDPS	0	587430	2549
Q06730	130	Zinc finger protein 33A	ZNF33A	0.861264	0.0219972	48.656	S	KEQGDVIGIPFNVDVSSFPSRKMFCQCDSCG	1126500	961070	2208
Q06730	134	Zinc finger protein 33A	ZNF33A	0.99411	0.0219972	48.656	S	DVIGIPFNVDVSSFPSRKMFCQCDSCGMSFN	1126500	961070	2209
Q9NRS6	179	Sorting nexin-15	SNX15	0.519802	0.00373891	48.65	S	RRGLEELEVPVDPPPSSPAQEALDLLFNCES	0	1218300	5982
Q9NRS6	201	Sorting nexin-15	SNX15	0.908565	0.00373891	48.65	S	LDLLFNCESTEEASGSPARGPLTEAELALFD	2339700	1218300	5984
Q9UK58	330	Cyclin-L1	CCNL1	0.295472	0.0247579	48.641	T	LKAKGLNPDGTPALSTLGGFSPASKPSSPRE	0	0	8227
Q14694	211	Ubiquitin carboxyl-terminal hydrolase 10	USP10	0.462055	0.0168578	48.626	S	FMGDMPPSVTPRTCNSPQNSTDSVSDIVPDS	0	0	2802
Q9BQ61	39	Uncharacterized protein C19orf43	C19orf43	0.59978	0.00133253	48.614	S	GGSRWAESGSGTSPESGDEEVSGAGSSPVSG	1548000	0	5299
Q9C0C9	441	E2/E3 hybrid ubiquitin-protein ligase UBE2O	UBE2O	0.7142	0.00114526	48.551	S	EAESASPEETPDGSASPVEMQDEGAEEPHEA	4233600	1276600	5539
Q12872	610	Splicing factor, suppressor of white-apricot homolog	SFSWAP	0.38246	0.00301919	48.467	S	KNRVKLDDDSDDDEESKEGQESSSSAANTNP	0	0	2322
Q13112	538	Chromatin assembly factor 1 subunit B	CHAF1B	0.945242	0.00280216	48.467	S	STEEIQSETPGDAQGSPPELKRPRLDENKGG	5419700	2329100	2409
Q9BQA9	173	Uncharacterized protein C17orf62	C17orf62	0.625667	0.0083679	48.422	S	TSFLELHCLESPTELSQSSDSEAGDPASQS_	511780	309820	5303
Q13542	36	Eukaryotic translation initiation factor 4E-binding protein	EIF4EBP2	0.467531	0.0106391	48.41	T	TVAISDAAQLPHDYCTTPGGTLFSTTPGGTR	0	0	7560
Q9H7N4	992	Splicing factor, arginine/serine-rich 19	SCAF1	0.330004	0.00480859	48.392	S	PPPKAAPPPPALTPDSQTVDSSCKTPEVSFL	0	0	5761
Q7LBC6	742	Lysine-specific demethylase 3B	KDM3B	0.242915	0.00470806	48.388	S	SPTSSLTQPIEMPTLSSSPTEERPTVGPGQQ	0	0	3741
O95714	2928	E3 ubiquitin-protein ligase HERC2	HERC2	1	0.0211199	48.337	S	IRAEEEDLAAVPFLASDNEEEEDEKGNSGSL	0	0	799
Q96D71	120	RalBP1-associated Eps domain-containing protein 1	REPS1	0.27579	0.0207064	48.244	S	KNEQESRHAASYSSDSENQGSYSGVIPPPPG	0	0	4841
Q9C0C9	431	E2/E3 hybrid ubiquitin-protein ligase UBE2O	UBE2O	0.434483	0.00114526	48.221	S	DKKGESKTKSEAESASPEETPDGSASPVEMQ	0	0	5537
Q86XP3	96	ATP-dependent RNA helicase DDX42	DDX42	0.839775	0.00711507	48.213	S	EDSSNVDLPYIPAENSPTRQQFHSKPVDSDS	870410	0	4015
Q86XP3	98	ATP-dependent RNA helicase DDX42	DDX42	0.498939	0.00711507	48.213	T	SSNVDLPYIPAENSPTRQQFHSKPVDSDSDD	0	0	7835
P41440	4	Folate transporter 1	SLC19A1	0.797215	0.031363	48.188	S	____________MVPSSPAVEKQVPVEPGPD	0	292490	1490
P07196	63	Neurofilament light polypeptide	NEFL	0.391784	0.0143744	48.106	S	SSSLSVRRSYSSSSGSLMPSLENLDLSQVAA	0	0	927
P15170	36	Eukaryotic peptide chain release factor GTP-binding subunit	GSPT1	0.897234	0.00401656	48.106	S	WEHKEEISEAEPGGGSLGDGRPPEESAHEMM	0	0	1068
Q9BXT6	448	Putative helicase Mov10l1	MOV10L1	0.441571	0.0273859	48.097	S	FSDFLIGRYLEVNVISGEESLIAAREPFSWK	0	0	5461
Q6PJT7	527	Zinc finger CCCH domain-containing protein 14	ZC3H14	0.958396	0.00433196	47.989	S	KPASPKFIVTLDGVPSPPGYMSDQEEDMCFE	0	1103300	3553
Q6PJT7	531	Zinc finger CCCH domain-containing protein 14	ZC3H14	0.747134	0.00433196	47.989	Y	PKFIVTLDGVPSPPGYMSDQEEDMCFEGMKP	0	0	8388
Q13111	123	Chromatin assembly factor 1 subunit A	CHAF1A	0.691146	0.0122686	47.919	S	TSIGQSTVIIDLTEDSNEQPDSLVDHNKLNS	581100	0	2401
Q13309	57	S-phase kinase-associated protein 2	SKP2	0.673837	0.0216231	47.808	S	EKEEPDSENIPQELLSNLGHPESPPRKRLKS	0	0	2470
Q9UPT8	907	Zinc finger CCCH domain-containing protein 4	ZC3H4	0.268782	0.0295016	47.808	S	LARALPTSKPEGSLHSSPVGPSSSKGSGPPP	0	0	6570
Q5I0X4	6	Uncharacterized protein C6orf226	C6orf226	0.334483	0.00596361	47.77	S	__________MERPRSPQCSAPASASASVTL	0	0	3168
Q5I0X4	10	Uncharacterized protein C6orf226	C6orf226	0.334483	0.00596361	47.77	S	______MERPRSPQCSAPASASASVTLAQLL	0	0	3169
Q13464	1098	Rho-associated protein kinase 1	ROCK1	0.419878	0.0211543	47.745	S	KESDIEQLRAKLLDLSDSTSVASFPSADETD	0	0	2542
Q13464	1100	Rho-associated protein kinase 1	ROCK1	0.372444	0.0211543	47.745	S	SDIEQLRAKLLDLSDSTSVASFPSADETDGN	0	0	2543
Q13464	1101	Rho-associated protein kinase 1	ROCK1	0.374345	0.0211543	47.745	T	DIEQLRAKLLDLSDSTSVASFPSADETDGNL	0	0	7546
O15432	79	Probable low affinity copper uptake protein 2	SLC31A2	0.517985	0.00462699	47.723	S	QQTIAETDGDSAGSDSFPVGRTHHRWYLCHF	982340	0	319
Q92598	561	Heat shock protein 105 kDa	HSPH1	0.655155	0.00391366	47.674	T	TDAQQTSQSPPSPELTSEENKIPDADKANEK	0	1147900	7936
Q8NB50	7	Zinc finger protein 62 homolog	ZFP62	0.495532	0.0193097	47.554	S	_________MSHLKTSTEDEEPTEEYENVGN	0	0	4313
Q8NB50	6	Zinc finger protein 62 homolog	ZFP62	0.711189	0.0193097	47.554	T	__________MSHLKTSTEDEEPTEEYENVG	0	1042900	7883
Q8NB50	8	Zinc finger protein 62 homolog	ZFP62	0.495185	0.0193097	47.554	T	________MSHLKTSTEDEEPTEEYENVGNA	0	0	7884
Q99956	375	Dual specificity protein phosphatase 9	DUSP9	0.233976	0.00736538	47.537	S	ASAASNPPSFFTTPTSDGAFELAPT______	0	0	5283
Q99956	371	Dual specificity protein phosphatase 9	DUSP9	0.233976	0.00736538	47.537	T	SGGQASAASNPPSFFTTPTSDGAFELAPT__	0	0	8035
Q99956	372	Dual specificity protein phosphatase 9	DUSP9	0.233976	0.00736538	47.537	T	GGQASAASNPPSFFTTPTSDGAFELAPT___	0	0	8036
Q99956	374	Dual specificity protein phosphatase 9	DUSP9	0.233976	0.00736538	47.537	T	QASAASNPPSFFTTPTSDGAFELAPT_____	0	0	8037
Q9NPI6	197	mRNA-decapping enzyme 1A	DCP1A	0.307225	0.00342406	47.534	T	GLQPSTQLSNLGSTETLEEMPSGSQDKSAPS	0	0	8139
P34932	830	Heat shock 70 kDa protein 4	HSPA4	0.537662	0.0061295	47.395	S	PQAAEQGTDTAVPSDSDKKLPEMDID_____	0	0	1372
Q9UKX7	296	Nuclear pore complex protein Nup50	NUP50	0.483349	0.0151348	47.395	S	VLGSLSSVPLTGFSFSPGNSSLFGKDTTQSK	0	0	6463
Q8N1G0	239	Zinc finger protein 687	ZNF687	0.385968	0.0237593	47.392	S	ESCSPHHPQVLAQQGSGSSPKATDIPASASP	0	0	4230
Q7Z2Z1	1413	Treslin	TICRR	0.84567	0.0177425	47.286	S	CQPDASATPGVGTADSPAAPTDSRDDQKGLS	1583500	1027700	3767
Q7L014	295	Probable ATP-dependent RNA helicase DDX46	DDX46	0.658633	0.00838296	47.191	S	KKGELMENDQDAMEYSSEEEEVDLQTALTGY	1158500	0	3718
Q7L014	296	Probable ATP-dependent RNA helicase DDX46	DDX46	0.658633	0.00838296	47.191	S	KGELMENDQDAMEYSSEEEEVDLQTALTGYQ	1158500	0	3719
Q8NGP9	246	Olfactory receptor 5AR1	OR5AR1	0.843	0.0226836	47.155	T	AEGRLKAFSTCGSHLTGITLFYGTVMFMYLR	2745900	1379100	7898
Q8NGP9	254	Olfactory receptor 5AR1	OR5AR1	0.840425	0.0226836	47.155	T	STCGSHLTGITLFYGTVMFMYLRPTSSYSLD	2745900	1379100	7899
Q8NGP9	259	Olfactory receptor 5AR1	OR5AR1	0.774847	0.0226836	47.155	Y	HLTGITLFYGTVMFMYLRPTSSYSLDQDKWA	2745900	1379100	8398
P05387	74	60S acidic ribosomal protein P2	RPLP2	0.503612	0.00771932	47.104	S	IGKLASVPAGGAVAVSAAPGSAAPAAGSAPA	2203300	0	896
O14654	582	Insulin receptor substrate 4	IRS4	0.999831	0.0229589	47.089	S	GHGSGGGQGPGDGHGSGGGKNSGGGKGSGSG	1104500	598170	176
Q9NQ55	228	Suppressor of SWI4 1 homolog	PPAN	0.542402	0.00335805	47.006	S	LLQEKFPNMSRLQDISELLATGAGLSESEAE	0	0	5898
Q8WW12	108	PEST proteolytic signal-containing nuclear protein	PCNP	0.460821	0.0358742	46.982	S	SKPKETVPTLAPKTLSVAAAFNEDEDSEPEE	0	0	4548
E9PAV3	2037	Nascent polypeptide-associated complex subunit alpha, muscle	NACA	0.519674	0.0223281	46.968	T	TPTVQEESEEEEVDETGVEVKDIELVMSQAN	3022100	1176800	7118
Q99996	3118	A-kinase anchor protein 9	AKAP9	0.999219	0.01708	46.956	S	GRKITLKREQESEKPSQELLEYNIQQKQSQM	590130	640540	5289
Q99996	3141	A-kinase anchor protein 9	AKAP9	0.499722	0.01708	46.956	S	IQQKQSQMLEMQVELSSMKDRATELQEQLSS	0	0	5290
Q99996	3142	A-kinase anchor protein 9	AKAP9	0.499722	0.01708	46.956	S	QQKQSQMLEMQVELSSMKDRATELQEQLSSE	0	0	5291
Q99996	3124	A-kinase anchor protein 9	AKAP9	0.980823	0.01708	46.956	Y	KREQESEKPSQELLEYNIQQKQSQMLEMQVE	590130	640540	8416
Q8N1G0	227	Zinc finger protein 687	ZNF687	0.742395	0.0265226	46.786	S	PVSSPPLGALKQESCSPHHPQVLAQQGSGSS	0	0	4229
Q14676	1444	Mediator of DNA damage checkpoint protein 1	MDC1	0.445159	0.0149221	46.678	T	TSQATRGRTNRSSVKTPETVVPTAPELQPST	0	0	7612
Q14676	1447	Mediator of DNA damage checkpoint protein 1	MDC1	0.445159	0.0149221	46.678	T	ATRGRTNRSSVKTPETVVPTAPELQPSTSTD	0	0	7613
Q99700	853	Ataxin-2	ATXN2	0.323996	0.0108979	46.464	T	PSAKDSFIENSSSNCTSGSSKPNSPSISPSI	0	0	8029
Q9NRY5	2	Protein FAM114A2	FAM114A2	0.95626	0.0196172	46.43	S	_______________MSDKDDIETPLLTEAAP	525190	495670	5989
P05412	249	Transcription factor AP-1	JUN	0.591385	0.0232093	46.403	S	EMPGETPPLSPIDMESQERIKAERKRMRNRI	0	0	903
Q9UN36	359	Protein NDRG2	NDRG2	0.212062	0.0237568	46.368	S	SRSRTLSQSSESGTLSSGPPGHTMEVSC___	0	0	6531
Q9UN36	360	Protein NDRG2	NDRG2	0.212062	0.0237568	46.368	S	RSRTLSQSSESGTLSSGPPGHTMEVSC____	0	0	6532
Q9P0K7	350	Ankycorbin	RAI14	0.415985	0.0198403	46.361	S	SLLDISSEADQQDLLSLLQAKVASLTLHNKE	0	0	6186
Q8N1G0	225	Zinc finger protein 687	ZNF687	0.553873	0.0272247	46.353	S	QPPVSSPPLGALKQESCSPHHPQVLAQQGSG	12559000	6232000	4228
Q96RE7	145	Nucleus accumbens-associated protein 1	NACC1	0.890724	0.00108401	46.247	S	SQGLHAEEAPSSEPQSPVAQTSGWPACSTPL	1488900	1092600	5087
Q12906	506	Interleukin enhancer-binding factor 3	ILF3	0.483452	0.00135	46.186	S	AVVAPAPVVEAVSTPSAAFPSDATAEQGPIL	0	0	2376
Q96RL1	397	BRCA1-A complex subunit RAP80	UIMC1	0.190859	0.0025125	46.174	S	KSLKEKLLLEEEPTTSHGQSSQGIVEETSEE	0	0	5094
Q96RL1	401	BRCA1-A complex subunit RAP80	UIMC1	0.190859	0.0025125	46.174	S	EKLLLEEEPTTSHGQSSQGIVEETSEEGNSV	0	0	5095
Q96RL1	402	BRCA1-A complex subunit RAP80	UIMC1	0.190859	0.0025125	46.174	S	KLLLEEEPTTSHGQSSQGIVEETSEEGNSVP	0	0	5096
Q96RL1	395	BRCA1-A complex subunit RAP80	UIMC1	0.190859	0.0025125	46.174	T	SIKSLKEKLLLEEEPTTSHGQSSQGIVEETS	0	0	8005
Q96RL1	396	BRCA1-A complex subunit RAP80	UIMC1	0.190859	0.0025125	46.174	T	IKSLKEKLLLEEEPTTSHGQSSQGIVEETSE	0	0	8006
O76094	620	Signal recognition particle subunit SRP72	SRP72	0.355232	0.0239022	46.132	S	ATAGASSELDASKTVSSPPTSPRPGSAATVS	0	0	679
Q969V6	124	MKL/myocardin-like protein 1	MKL1	0.601756	0.00406935	46.082	S	VADSSSFDEDSSDALSPEQPASHESQGSVPS	3212400	0	4762
P50570	761	Dynamin-2	DNM2	0.260537	0.00245422	46.034	S	TPVPPPVDDTWLQSASSHSPTPQRRPVSSIH	0	0	1755
Q9BQI3	258	Eukaryotic translation initiation factor 2-alpha kinase 1	EIF2AK1	0.86825	0.0312532	45.862	S	RADRAAIELPSLEVLSDQEEDREQCGVKNDE	0	0	5316
Q12872	616	Splicing factor, suppressor of white-apricot homolog	SFSWAP	0.161559	0.00363858	45.856	S	DDDSDDDEESKEGQESSSSAANTNPAVAPPC	0	0	2323
Q12872	617	Splicing factor, suppressor of white-apricot homolog	SFSWAP	0.161559	0.00363858	45.856	S	DDSDDDEESKEGQESSSSAANTNPAVAPPCV	0	0	2324
Q12872	618	Splicing factor, suppressor of white-apricot homolog	SFSWAP	0.161559	0.00363858	45.856	S	DSDDDEESKEGQESSSSAANTNPAVAPPCVV	0	0	2325
Q12872	619	Splicing factor, suppressor of white-apricot homolog	SFSWAP	0.161559	0.00363858	45.856	S	SDDDEESKEGQESSSSAANTNPAVAPPCVVV	0	0	2326
Q12872	623	Splicing factor, suppressor of white-apricot homolog	SFSWAP	0.161559	0.00363858	45.856	T	EESKEGQESSSSAANTNPAVAPPCVVVEEKK	0	0	7511
Q8IWZ3	101	Ankyrin repeat and KH domain-containing protein 1	ANKHD1	0.463135	0.00733002	45.836	S	TGGGGGASGSDEDEVSEVESFILDQEDLDNP	0	0	4086
P55884	81	Eukaryotic translation initiation factor 3 subunit B	EIF3B	0.263372	0.00101491	45.761	S	VRTEPAAEAEAASGPSESPSPPAAEELPGSH	0	0	1889
P98155	829	Very low-density lipoprotein receptor	VLDLR	0.956269	0.0311885	45.696	S	GGYLMWRNWQHKNMKSMNFDNPVYLKTTEED	1175200	770550	2006
P98155	846	Very low-density lipoprotein receptor	VLDLR	0.998272	0.0311885	45.696	S	NFDNPVYLKTTEEDLSIDIGRHSASVGHTYP	1175200	770550	2007
O43290	474	U4/U6.U5 tri-snRNP-associated protein 1	SART1	1	0.00740213	45.685	S	QPLPSDDTRVENMDISDEEEGGAPPPGSPQV	1691800	1951100	353
O43290	486	U4/U6.U5 tri-snRNP-associated protein 1	SART1	1	0.00740213	45.685	S	MDISDEEEGGAPPPGSPQVLEEDEAELELQK	1691800	1951100	354
Q9H0B6	175	Kinesin light chain 2	KLC2	0.993063	0.0041763	45.685	S	PKDTLDDLFPNEDEQSPAPSPGGGDVSGQHG	719210	939960	5560
Q9H0B6	179	Kinesin light chain 2	KLC2	0.79757	0.0041763	45.685	S	LDDLFPNEDEQSPAPSPGGGDVSGQHGGYEI	719210	939960	5561
Q9H4L7	95	SWI/SNF-related matrix-associated actin-dependent regulator	SMARCAD1	0.654666	0.0261157	45.679	S	ISYFKNQRGIQYIDLSSDSEDVVSPNCSNTV	4541600	0	5686
Q9H7N4	994	Splicing factor, arginine/serine-rich 19	SCAF1	0.270582	0.00361397	45.652	T	PKAAPPPPALTPDSQTVDSSCKTPEVSFLPE	0	0	8114
P50747	18	Biotin--protein ligase;Biotin--[methylmalonyl-CoA-carboxytra	HLCS	0.666667	0.0270205	45.546	S	DRLHMDNGLVPQKIVSVHLQDSTLKEVKDQV	0	2690600	1761
P50747	24	Biotin--protein ligase;Biotin--[methylmalonyl-CoA-carboxytra	HLCS	0.666667	0.0270205	45.546	S	NGLVPQKIVSVHLQDSTLKEVKDQVSNKQAQ	0	2690600	1762
Q96ST3	284	Paired amphipathic helix protein Sin3a	SIN3A	0.744085	0.00500141	45.527	T	LPPYASPRSPPVQPHTPVTISLGTAPSLQNN	177100	0	8009
O75533	257	Splicing factor 3B subunit 1	SF3B1	0.455954	0.00425494	45.349	T	SETPGATPGSKIWDPTPSHTPAGAATPGRGD	0	0	7209
Q86TI0	566	TBC1 domain family member 1	TBC1D1	0.494823	0.0139899	45.328	S	EALPISESSFKLLGSSEDLSSDSESHLPEEP	0	0	3899
Q86TI0	571	TBC1 domain family member 1	TBC1D1	0.363632	0.0139899	45.328	S	SESSFKLLGSSEDLSSDSESHLPEEPAPLSP	0	0	3901
Q86TI0	573	TBC1 domain family member 1	TBC1D1	0.363632	0.0139899	45.328	S	SSFKLLGSSEDLSSDSESHLPEEPAPLSPQQ	0	0	3902

Q8IYA2;A	1115;130	Putative coiled-coil domain-containing protein 144C;Coiled-c	CCDC144CP;	0.99851	0.0311631	45.243	S	LSNELSRTNEMIAEVSTQLTVEKEQTRSRSL	1241000	1143300	25
P18858	98	DNA ligase 1	LIG1	0.365144	0.0115192	45.003	S	PALDCSQVSPPRPATSPENNASLSDTSPMDS	0	0	1149
P18858	97	DNA ligase 1	LIG1	0.365144	0.0115192	45.003	T	KPALDCSQVSPPRPATSPENNASLSDTSPMD	0	0	7303
Q92615	532	La-related protein 4B	LARP4B	0.506623	0.00597095	44.916	S	PTPPKPPSPSFELGLSSFPPLPGAAGNLKTE	0	0	4675
O95429	283	BAG family molecular chaperone regulator 4	BAG4	0.31054	0.00246114	44.915	S	NLYMTESTSPWPSSGSPQSPPSPPVQQPKDS	0	0	772
O95429	286	BAG family molecular chaperone regulator 4	BAG4	0.557876	0.00246114	44.915	S	MTESTSPWPSSGSPQSPPSPPVQQPKDSSYP	0	4104200	773
Q9BQ61	50	Uncharacterized protein C19orf43	C19orf43	0.426342	0.00172927	44.869	S	TSPESGDEEVSGAGSSPVSGGVNLFANDGSF	0	0	5300
Q9BQ61	53	Uncharacterized protein C19orf43	C19orf43	0.426342	0.00172927	44.869	S	ESGDEEVSGAGSSPVSGGVNLFANDGSFLEL	0	0	5301
Q9NRH2	612	SNF-related serine/threonine-protein kinase	SNRK	0.465799	0.0180013	44.712	S	SPSENNAGGGSPSSGSGGNPTNTSGTTRRCA	0	0	5967
Q5T1M5	329	FK506-binding protein 15	FKBP15	0.418223	0.0194247	44.648	T	PGADNLSADPVVSPPTSIPFKSGEPALRTKS	0	0	7718
Q9HAF1	122	Chromatin modification-related protein MEAF6	MEAF6	0.284508	0.002458	44.641	S	QDQLIEKREPGSGTESDTSPDFHNQENEPSQ	0	0	5814
Q9HAF1	125	Chromatin modification-related protein MEAF6	MEAF6	0.284508	0.002458	44.641	S	LIEKREPGSGTESDTSPDFHNQENEPSQEDP	0	0	5815
Q9HAF1	124	Chromatin modification-related protein MEAF6	MEAF6	0.284508	0.002458	44.641	T	QLIEKREPGSGTESDTSPDFHNQENEPSQED	0	0	8126
Q15477	270	Helicase SKI2W	SKIV2L	0.796147	0.00825173	44.616	S	SSLEDLVLKEASTAVSTPEAPEPPSQEQWAI	0	0	2940
Q15477	271	Helicase SKI2W	SKIV2L	0.796147	0.00825173	44.616	T	SLEDLVLKEASTAVSTPEAPEPPSQEQWAIP	0	0	7643
Q12802	793	A-kinase anchor protein 13	AKAP13	0.416425	0.0241083	44.596	S	AVISDSTFSLANSPGSESVTKDDALSFVPSQ	0	0	2308
Q4VXU2	51	Polyadenylate-binding protein 1-like	PABPC1L	0.786224	0.0187858	44.555	S	PILSIRVCRDVATRRSLGYAYINFQQPADAE	0	2305800	3120
Q4VXU2	71	Polyadenylate-binding protein 1-like	PABPC1L	0.992133	0.0187858	44.555	T	YINFQQPADAERALDTMNFEMLKGQPIRIMW	0	2305800	7689
Q9UJV9	21	Probable ATP-dependent RNA helicase DDX41	DDX41	0.945721	0.0264539	44.474	S	PERKRARTDEVPAGGSRSEAEDEDDEDYVPY	0	2021300	6412
Q9HD20	634	Manganese-transporting ATPase 13A1	ATP13A1	0.991709	0.0307223	44.457	S	KIHQRFHFASALKRMSVLASYEKLGSTDLCY	5501900	0	5879
Q9HD20	638	Manganese-transporting ATPase 13A1	ATP13A1	0.990867	0.0307223	44.457	S	RFHFASALKRMSVLASYEKLGSTDLCYIAAV	5501900	0	5880
Q9HD20	644	Manganese-transporting ATPase 13A1	ATP13A1	0.470397	0.0307223	44.457	S	ALKRMSVLASYEKLGSTDLCYIAAVKGAPET	0	0	5881
Q9C0B2	887	Cilia- and flagella-associated protein 74	CFAP74	1	0.0245867	44.382	T	RHSLPEDAGRYFDKETRVLEAPMTIWVADQN	0	624530	8074
Q9C0B2	895	Cilia- and flagella-associated protein 74	CFAP74	1	0.0245867	44.382	T	GRYFDKETRVLEAPMTIWVADQNKPVGFTVH	0	624530	8075
Q9C0B2	882	Cilia- and flagella-associated protein 74	CFAP74	1	0.0245867	44.382	Y	LKFLPRHSLPEDAGRYFDKETRVLEAPMTIW	0	624530	8421
P01891	349	HLA class I histocompatibility antigen, A-68 alpha chain	HLA-A	0.400724	0.0288144	44.312	S	RKSSDRKGGSYSQAASSDSAQGSDVSLTACK	0	0	855
O75427	19	Leucine-rich repeat and calponin homology domain-containing	LRCH4	0.403185	0.0131652	44.308	T	AVAAPLAAGGEEAAATTSVPGSPGLPGRRSA	0	0	7203
O75427	20	Leucine-rich repeat and calponin homology domain-containing	LRCH4	0.403185	0.0131652	44.308	T	VAAPLAAGGEEAAATTSVPGSPGLPGRRSAE	0	0	7204
Q9NQ55	251	Suppressor of SWI4 1 homolog	PPAN	0.639435	0.00489337	44.289	T	GLSESEAEPDGDHNITELPQAVAGRGNMRAQ	4153700	0	8141
Q9UN72;Q	313;313	Protocadherin alpha-7;Protocadherin alpha-9	PCDHA7;PCD	0.983873	0.0285138	44.238	S	LSGAITVIGHMDFEESRAHKIPVEAVDKGFP	0	1148800	6533
Q9NWS9	180	Zinc finger protein 446	ZNF446	0.501195	0.00418443	44.215	S	SVKEEPNVDGQEVAPSSPPLAAQSPEGNHGH	4169800	0	6065
Q9NWS9	181	Zinc finger protein 446	ZNF446	0.501195	0.00418443	44.215	S	VKEEPNVDGQEVAPSSPPLAAQSPEGNHGHQ	4169800	0	6066
Q9NWS9	188	Zinc finger protein 446	ZNF446	0.918785	0.00418443	44.215	S	DGQEVAPSSPPLAAQSPEGNHGHQEPASTSF	4169800	2988600	6067
P16989	34	Y-box-binding protein 3	YBX3	0.642677	0.000372465	44.201	S	PTEAAAAAPQDPAPKSPVGSGAPQAAAPAPA	0	20102000	1101
P51003	532	Poly(A) polymerase alpha	PAPOLA	0.543672	0.0199773	44.167	S	ALNDSSLDLSMDSDNSMSVPSPTSATKTSPL	0	0	1772
O75717	348	WD repeat and HMG-box DNA-binding protein 1	WDHD1	0.49984	0.0156795	44.164	S	SNAGDFLNDNAVEIPSFSKGIINDDEDDEDL	0	0	660
O75717	350	WD repeat and HMG-box DNA-binding protein 1	WDHD1	0.49984	0.0156795	44.164	S	AGDFLNDNAVEIPSFSKGIINDDEDDEDLMM	0	0	661
Q6P4R8	347	Nuclear factor related to kappa-B-binding protein	NFRKB	0.955882	0.0119753	44.164	S	DLAEPLSSTEGVAPLSQAPSPLAIPAIKEEP	247180	333820	3527
Q9P2F8	383	Signal-induced proliferation-associated 1-like protein 2	SIPA1L2	0.497973	0.0185896	44.161	S	QMPTGQTGNCESPLGSKEDLNSKENLDADEG	0	0	6240
O75122	461	CLIP-associating protein 2	CLASP2	0.863164	0.0173028	44.05	S	RSRTKMVSQSQPGSRSGSPGRVLTTTALSTV	3947300	1673900	561
O75122	463	CLIP-associating protein 2	CLASP2	0.863166	0.0173028	44.05	S	RTKMVSQSQPGSRSGSPGRVLTTTALSTVSS	3947300	1673900	562
O75122	474	CLIP-associating protein 2	CLASP2	0.240576	0.0173028	44.05	S	SRSGSPGRVLTTTALSTVSSGVQRVLVNSAS	0	0	563
O75122	470	CLIP-associating protein 2	CLASP2	0.240576	0.0173028	44.05	T	SQPGSRSGSPGRVLTTTALSTVSSGVQRVLV	0	0	7194
O75122	471	CLIP-associating protein 2	CLASP2	0.240576	0.0173028	44.05	T	QPGSRSGSPGRVLTTTALSTVSSGVQRVLVN	0	0	7195
O75122	475	CLIP-associating protein 2	CLASP2	0.240576	0.0173028	44.05	T	RSGSPGRVLTTTALSTVSSGVQRVLVNSASA	0	0	7196
O15040	751	Tectonin beta-propeller repeat-containing protein 2	TECPR2	0.211139	0.00249852	44.023	S	HKPWLEQPPRDQTLTSSDEEDIYAHGLPSSS	0	0	256
O15040	752	Tectonin beta-propeller repeat-containing protein 2	TECPR2	0.211139	0.00249852	44.023	S	KPWLEQPPRDQTLTSSDEEDIYAHGLPSSSS	0	0	257
O15040	764	Tectonin beta-propeller repeat-containing protein 2	TECPR2	0.151739	0.00249852	44.023	S	LTSSDEEDIYAHGLPSSSSETSVTELGPSCS	0	0	258
O15040	765	Tectonin beta-propeller repeat-containing protein 2	TECPR2	0.193026	0.00249852	44.023	S	TSSDEEDIYAHGLPSSSSETSVTELGPSCSQ	0	0	259
O15040	766	Tectonin beta-propeller repeat-containing protein 2	TECPR2	0.193026	0.00249852	44.023	S	SSDEEDIYAHGLPSSSSETSVTELGPSCSQQ	0	0	260
O15040	767	Tectonin beta-propeller repeat-containing protein 2	TECPR2	0.193026	0.00249852	44.023	S	SDEEDIYAHGLPSSSSETSVTELGPSCSQQD	0	0	261
O15040	748	Tectonin beta-propeller repeat-containing protein 2	TECPR2	0.239943	0.00249852	44.023	T	ASTHKPWLEQPPRDQTLTSSDEEDIYAHGLP	0	0	7144
O15040	750	Tectonin beta-propeller repeat-containing protein 2	TECPR2	0.211139	0.00249852	44.023	T	THKPWLEQPPRDQTLTSSDEEDIYAHGLPSS	0	0	7145
Q86XP3	758	ATP-dependent RNA helicase DDX42	DDX42	0.364608	0.00240888	43.995	S	NSAQQGHNSPDSPVTSAAKGIPGFGNTGNIS	0	0	4014
Q86XP3	757	ATP-dependent RNA helicase DDX42	DDX42	0.364608	0.00240888	43.995	T	TNSAQQGHNSPDSPVTSAAKGIPGFGNTGNI	0	0	7834
Q6IBW4	232	Condensin-2 complex subunit H2	NCAPH2	0.993406	0.00230235	43.951	S	TGRTEEQPMEVSVCRSPVPALGFSQEPGPSP	1673500	672760	3469
Q9BUR4	103	Telomerase Cajal body protein 1	WRAP53	0.481946	0.00904423	43.831	S	ELSPRIEEQELSENTSLPAEEANGSLSEEEA	0	0	5387
Q9BUR4	102	Telomerase Cajal body protein 1	WRAP53	0.481946	0.00904423	43.831	T	SELSPRIEEQELSENTSLPAEEANGSLSEEE	0	0	8052
Q08AE8	169	Protein spire homolog 1	SPIRE1	0.515992	0.0037485	43.804	S	EQLIDHMANTVEADGSNDEGYEAAEEGLGDE	562590	520620	2264
P16989	38	Y-box-binding protein 3	YBX3	0.498825	0.000372465	43.695	S	AAAAPQDPAPKSPVGSGAPQAAAPAPAAHVA	0	0	1102
Q8N0Y2	162	Zinc finger protein 444	ZNF444	0.536649	0.0197967	43.612	S	PGDSQAVRPYKQEPSSPPLAPGLPAFLAAPG	781780	0	4196
Q6PJT7	533	Zinc finger CCCH domain-containing protein 14	ZC3H14	0.535297	0.00512606	43.579	S	FIVTLDGVPSPPGYMSDQEEDMCFEGMKPVN	0	1103300	3554
Q8IYL3	182	UPF0688 protein C1orf174	C1orf174	0.582024	0.00676751	43.579	S	LQTEDVQKPPLQMDNSVFLDDDSNQPMPVSR	1599800	0	4176
Q8WUZ0	122	B-cell CLL/lymphoma 7 protein family member C	BCL7C	0.581595	0.0227018	43.554	S	LQKGTEPSPGGTPQPSRPVSPAGPPEGVPEE	3697300	0	4524
Q8WUZ0	118	B-cell CLL/lymphoma 7 protein family member C	BCL7C	0.589552	0.0227018	43.554	T	SEGSLQKGTEPSPGGTPQPSRPVSPAGPPEG	3697300	0	7917
Q9BZQ4	49	Nicotinamide/nicotinic acid mononucleotide adenylyltransfera	NMNAT2	0.868083	0.0304758	43.523	S	YLHKTGRFIVIGGIVSPVHDSYGKQGLVSSR	30670000	26064000	5494
Q9BZQ4	54	Nicotinamide/nicotinic acid mononucleotide adenylyltransfera	NMNAT2	0.999704	0.0304758	43.523	S	GRFIVIGGIVSPVHDSYGKQGLVSSRHRLIM	30670000	26064000	5495
Q9BZQ4	62	Nicotinamide/nicotinic acid mononucleotide adenylyltransfera	NMNAT2	0.999974	0.0304758	43.523	S	IVSPVHDSYGKQGLVSSRHRLIMCQLAVQNS	30670000	26064000	5496
Q9BZQ4	63	Nicotinamide/nicotinic acid mononucleotide adenylyltransfera	NMNAT2	0.999974	0.0304758	43.523	S	VSPVHDSYGKQGLVSSRHRLIMCQLAVQNSD	30670000	26064000	5497
Q9NWZ3	151	Interleukin-1 receptor-associated kinase 4	IRAK4	0.347777	0.0284787	43.497	S	TPVQNLEQSYMPPDSSSPENKSLEVSDTRFH	0	0	6072
Q9NWZ3	152	Interleukin-1 receptor-associated kinase 4	IRAK4	0.347777	0.0284787	43.497	S	PVQNLEQSYMPPDSSSPENKSLEVSDTRFHS	0	0	6073
P28290	1060	Sperm-specific antigen 2	SSFA2	0.525649	0.0232971	43.436	S	SALMGMCGSRSADNLSCPSPLNVMEPVTELM	568170	0	1305
Q96DF8	305	Protein DGCR14	DGCR14	0.992728	0.0249733	43.356	S	QESPRVGGFGFVATPSPAPGVNESPMMTWGE	377190	235310	4862
Q15303	1043	Receptor tyrosine-protein kinase erbB-4;ERBB4 intracellular	ERBB4	0.499058	0.0228443	43.156	S	FNIPPPIYTSRARIDSNRSEIGHSPPPAYTP	0	0	2897
Q15303	1046	Receptor tyrosine-protein kinase erbB-4;ERBB4 intracellular	ERBB4	0.499058	0.0228443	43.156	S	PPPIYTSRARIDSNRSEIGHSPPPAYTPMSG	0	0	2898
Q15303	1060	Receptor tyrosine-protein kinase erbB-4;ERBB4 intracellular	ERBB4	0.797756	0.0228443	43.156	S	RSEIGHSPPPAYTPMSGNQFVYRDGGFAAEQ	391650	0	2899
Q15303	1057	Receptor tyrosine-protein kinase erbB-4;ERBB4 intracellular	ERBB4	0.978808	0.0228443	43.156	T	DSNRSEIGHSPPPAYTPMSGNQFVYRDGGFA	391650	0	7634
Q15303	1056	Receptor tyrosine-protein kinase erbB-4;ERBB4 intracellular	ERBB4	0.977398	0.0228443	43.156	Y	IDSNRSEIGHSPPPAYTPMSGNQFVYRDGGF	391650	0	8382
Q8IZ69	595	tRNA (uracil-5-)-methyltransferase homolog A	TRMT2A	0.782086	0.0090202	43.155	T	CEMLILFERVEHPNGTGVLGPHSPPAQPTPG	1372500	701280	7869
P16989	79	Y-box-binding protein 3	YBX3	0.963029	0.00112681	43.118	S	ATGTAAAASLATAAGSEDAEKKVLATKVLGT	0	0	1104
O95155	83	Ubiquitin conjugation factor E4 B	UBE4B	0.309415	0.0293441	43.099	S	GASGVAHRSQSSEGVSSLSSSPSNSLETQSQ	0	0	734
O95155	84	Ubiquitin conjugation factor E4 B	UBE4B	0.314883	0.0293441	43.099	S	ASGVAHRSQSSEGVSSLSSSPSNSLETQSQS	0	0	735
O95155	90	Ubiquitin conjugation factor E4 B	UBE4B	0.344474	0.0293441	43.099	S	RSQSSEGVSSLSSSPSNSLETQSQSLSRSQS	0	0	738
O95155	92	Ubiquitin conjugation factor E4 B	UBE4B	0.336344	0.0293441	43.099	S	QSSEGVSSLSSSPSNSLETQSQSLSRSQSMD	0	0	739
Q15424;Q	331;330	Scaffold attachment factor B1;Scaffold attachment factor B2	SAFB;SAFB2	0.394011	0.00487134	43.083	S	PAVEQSSAASELAEASSEELAEAPTEAPSPE	0	0	2930
Q15424;Q	332;331	Scaffold attachment factor B1;Scaffold attachment factor B2	SAFB;SAFB2	0.394011	0.00487134	43.083	S	AVEQSSAASELAEASSEELAEAPTEAPSPEA	0	0	2931
Q9NRS6	180	Sorting nexin-15	SNX15	0.492377	0.00849381	43.048	S	RGLEELEVPVDPPPSSPAQEALDLLFNCEST	0	0	5983
Q01484	846	Ankyrin-2	ANK2	0.799871	0.00929384	42.843	S	HKLNVPETMTEVLDVSDEEGDDTMTGDGGEY	0	0	2068
O60341	80	Lysine-specific histone demethylase 1A	KDM1A	0.480435	0.00549241	42.756	S	PPRASPPGGLAEPPGSAGPQAGPTVVPGSAT	0	0	484
P07196	56	Neurofilament light polypeptide	NEFL	0.314212	0.0225611	42.669	S	SSYSAPVSSSLSVRRSYSSSSGSLMPSLENL	0	0	925
P07196	58	Neurofilament light polypeptide	NEFL	0.314212	0.0225611	42.669	S	YSAPVSSSLSVRRSYSSSSGSLMPSLENLDL	0	0	926
Q68CZ2	690	Tensin-3	TNS3	0.541477	0.00780097	42.652	S	VNGAGPELSTGPSPGSPTLDIDQSIEQLNRL	853770	485460	3419
O95453	570	Poly(A)-specific ribonuclease PARN	PARN	0.736597	0.00666103	42.65	S	NNSFTAPSTVGKRNLSPSQEEAGLEDGVSGE	0	4816200	776
O95453	587	Poly(A)-specific ribonuclease PARN	PARN	0.376962	0.00666103	42.65	S	SQEEAGLEDGVSGEISDTELEQTDSCAEPLS	0	0	777
O95453	589	Poly(A)-specific ribonuclease PARN	PARN	0.376962	0.00666103	42.65	T	EEAGLEDGVSGEISDTELEQTDSCAEPLSEG	0	0	7239
Q9UBK2	289	Peroxisome proliferator-activated receptor gamma coactivato	PPARGC1A	0.935741	0.0124017	42.59	S	PFENKTIERTLSVELSGTAGLTPPTTPPHKA	602420	950340	6267
Q9UBK2	291	Peroxisome proliferator-activated receptor gamma coactivato	PPARGC1A	0.932977	0.0124017	42.59	T	ENKTIERTLSVELSGTAGLTPPTTPPHKANQ	602420	950340	8196
Q9UBK2	295	Peroxisome proliferator-activated receptor gamma coactivato	PPARGC1A	0.975779	0.0124017	42.59	T	IERTLSVELSGTAGLTPPTTPPHKANQDNPF	602420	950340	8197
Q9UBK2	298	Peroxisome proliferator-activated receptor gamma coactivato	PPARGC1A	0.976002	0.0124017	42.59	T	TLSVELSGTAGLTPPTTPPHKANQDNPFRAS	602420	950340	8198
Q9UBK2	299	Peroxisome proliferator-activated receptor gamma coactivato	PPARGC1A	0.99171	0.0124017	42.59	T	LSVELSGTAGLTPPTTPPHKANQDNPFRASP	602420	950340	8199
Q14684	460	Ribosomal RNA processing protein 1 homolog B	RRP1B	0.776576	0.0300468	42.571	S	EPGAEATSSTGEESGSEHPPAVPMHNKRKRP	0	2397800	2791
Q6P2E9	820	Enhancer of mRNA-decapping protein 4	EDC4	0.317695	0.017819	42.461	S	HNTPSLLEAALTQEASTPDSQVWPTAPDITR	0	0	3520
P24386	7	Rab proteins geranylgeranyltransferase component A 1	CHM	0.979	0.0280115	42.454	S	_________MADTLPSEFDVIVIGTGLPESI	2819700	0	1231
P24386	28	Rab proteins geranylgeranyltransferase component A 1	CHM	0.689099	0.0280115	42.454	S	VIGTGLPESIIAAACSRSGRRVLHVDSRSYY	2819700	0	1232
Q9NVI1	1111	Fanconi anemia group I protein	FANCI	0.766548	0.0309363	42.454	S	LEEVDWLITKLKGQVSQETLSEEASSQATLP	0	0	6040
P16989	72	Y-box-binding protein 3	YBX3	0.600185	0.00129874	42.451	S	GGDAAPAATGTAAAASLATAAGSEDAEKKVL	34969000	0	1103
P50570	766	Dynamin-2	DNM2	0.413166	0.00895531	42.301	T	PVDDTWLQSASSHSPTPQRRPVSSIHPPGRP	0	0	7432
P78347	830	General transcription factor II-I	GTF2I	0.431951	0.0183364	42.295	S	IKESTSSKSPPRKINSSPNVNTTASGVEDLN	0	0	1961
P78347	831	General transcription factor II-I	GTF2I	0.431951	0.0183364	42.295	S	KESTSSKSPPRKINSSPNVNTTASGVEDLNI	0	0	1962
Q13439	118	Golgin subfamily A member 4	GOLGA4	0.590163	0.0162897	42.295	S	NRLDLDSSTASFDPPSDMDSEAEDLVGNSDS	0	0	2529
P33240	531	Cleavage stimulation factor subunit 2	CSTF2	0.419248	0.0103916	42.241	T	GGSQPGGFSPGQNQVTPQDHEKAALIMQVLQ	0	0	7352
P18858	106	DNA ligase 1	LIG1	0.208963	0.0283694	42.024	S	SPPRPATSPENNASLSDTSPMDSSPSGIPKR	0	0	1151
P18858	109	DNA ligase 1	LIG1	0.208963	0.0283694	42.024	S	RPATSPENNASLSDTSPMDSSPSGIPKRRTA	0	0	1152
Q14202	198	Zinc finger MYM-type protein 3	ZMYM3	0.419443	0.00664528	42.015	T	QPQSPNAPPSPSVGETLGDGINSSQTKPGGS	0	0	7589
O15234	381	Protein CASC3	CASC3	0.892921	0.00728775	41.695	S	ADAPVLGSPEKEEAASEPPAAAPDAAPPPPD	9912500	0	306
P27694	135	Replication protein A 70 kDa DNA-binding subunit;Replicatio	RPA1	0.529776	0.023464	41.455	S	EGLGQPQVAPPAPAASPAASSRPQPQNGSSG	3462200	0	1281
O60244	977	Mediator of RNA polymerase II transcription subunit 14	MED14	0.884369	0.023766	41.434	S	FLNMFVDSNQDARRRSVNEDDNPPSPIGGDM	1054000	0	446
O60244	995	Mediator of RNA polymerase II transcription subunit 14	MED14	0.367642	0.023766	41.434	S	EDDNPPSPIGGDMMDSLISQLQPPPQQQPFP	0	0	448
O60244	998	Mediator of RNA polymerase II transcription subunit 14	MED14	0.367642	0.023766	41.434	S	NPPSPIGGDMMDSLISQLQPPPQQQPFPKQP	0	0	449
P08238	718	Heat shock protein HSP 90-beta	HSP90AB1	1	0.0148477	41.434	S	AVPDEIPPLEGDEDASRMEEVD_________	269130	0	951
Q9P2C4	575	Transmembrane protein 181	TMEM181	0.487054	0.0168495	41.432	S	KNALYESQLKDNPAFSMLNDSDDDVIYGSDY	0	0	6229
Q9P2C4	580	Transmembrane protein 181	TMEM181	0.487054	0.0168495	41.432	S	ESQLKDNPAFSMLNDSDDDVIYGSDYEEMPL	0	0	6230
Q5T5X7	489	BEN domain-containing protein 3	BEND3	0.389601	0.0128842	41.388	S	QRINDELEGLGLDAGSEGDPPRDDCYDSSSL	0	0	3291
Q02952	723	A-kinase anchor protein 12	AKAP12	0.380538	0.0240096	41.253	T	GGDHQKADEAGKDKETGTDGILAGSQEHDPG	0	0	7480
Q02952	725	A-kinase anchor protein 12	AKAP12	0.380538	0.0240096	41.253	T	DHQKADEAGKDKETGTDGILAGSQEHDPGQG	0	0	7481
P36915	342	Guanine nucleotide-binding protein-like 1	GNL1	0.56021	0.00829643	41.232	T	EEEEEDGPAVLVEQQTDSAMEPTGPTQERYK	2190200	0	7370
Q9Y4B4	1194	Helicase ARIP4	RAD54L2	0.249182	0.017731	41.133	S	LQQYADVAAARESRQSSPSTNAALPGPPAQL	0	0	6903
Q9Y4B4	1198	Helicase ARIP4	RAD54L2	0.249182	0.017731	41.133	T	ADVAAARESRQSSPSTNAALPGPPAQLMDSS	0	0	8301
Q86TI0	570	TBC1 domain family member 1	TBC1D1	0.478756	0.0234942	41.097	S	ISESSFKLLGSSEDLSSDSESHLPEEPAPLS	0	0	3900
Q96RE7	151	Nucleus accumbens-associated protein 1	NACC1	0.262934	0.00716282	41.097	S	EEAPSSEPQSPVAQTSGWPACSTPLPLVSRV	0	0	5088
Q96RE7	150	Nucleus accumbens-associated protein 1	NACC1	0.262934	0.00716282	41.097	T	AEEAPSSEPQSPVAQTSGWPACSTPLPLVSR	0	0	8000
Q7Z3K6	114	Mesoderm induction early response protein 3	MIER3	0.746124	0.0203981	40.941	S	PDMTLDKEEIAKDLLSGDDEETQSSADDLTP	752980	465660	3784
Q9H6Z4	214	Ran-binding protein 3	RANBP3	0.538886	0.0227265	40.872	T	LPADCTGAVPAASPDTAAWRSPSEAADEVCA	0	0	8106
Q14202	768	Zinc finger MYM-type protein 3	ZMYM3	0.310148	0.015912	40.8	S	SQQNQPNLDTQSGPESLLNSQSPESKPQTPS	0	0	2704
Q9NQW6	99	Actin-binding protein anillin	ANLN	0.366671	0.0187153	40.799	S	NKQPVESTSAKSCSPSPVSPQVQPQAADTIS	1720500	0	5932
Q7Z7F0	44	UPF0469 protein KIAA0907	KIAA0907	0.36903	0.00357561	40.737	S	LPPAAPGGEVTSSGGSPGGTTAAPSGALDAA	0	0	3888
Q9H0E9	579	Bromodomain-containing protein 8	BRD8	0.663584	0.0304002	40.718	S	GKGDETPLTNVKTEASPESMLSPSHGSNPIE	1390300	1117200	5580
Q9H0E9	590	Bromodomain-containing protein 8	BRD8	0.429055	0.0304002	40.718	S	KTEASPESMLSPSHGSNPIEDPLEAETQHKF	0	0	5581
Q14202	267	Zinc finger MYM-type protein 3	ZMYM3	0.943479	0.0131947	40.697	S	EVVDSTESIPVSDEDSDAMVDDPNDEDFVPF	644950	461810	2701

P49023	243	Paxillin	PXN	0.734813	0.0114591	40.513	S	VPSPVPAITVNQGEMSSPQRVTSTQQQTRIS	1081500	500640	1644
Q7LBC6	743	Lysine-specific demethylase 3B	KDM3B	0.233577	0.0130522	40.166	S	PTSSLTQPIEMPTLSSSPTEERPTVGPGQQD	0	0	3742
Q9NZR2	4093	Low-density lipoprotein receptor-related protein 1B	LRP1B	0.659106	0.0311509	40.142	S	VDYFSERIYWADFELSIIGSVLYDGSNSVVS	0	0	6167
Q9NZR2	4097	Low-density lipoprotein receptor-related protein 1B	LRP1B	0.684787	0.0311509	40.142	S	SERIYWADFELSIIGSVLYDGSNSVVSVSSK	0	0	6168
Q9NZR2	4105	Low-density lipoprotein receptor-related protein 1B	LRP1B	0.433462	0.0311509	40.142	S	FELSIIGSVLYDGSNSVVSVSSKQGLLHPHR	0	0	6169
